# Supplementary material for: Genome-wide CRISPR screens identify PKMYT1 as a therapeutic target in pancreatic ductal adenocarcinoma
Source: EMBO Mol Med. 2024 Apr 3;16(5):5. doi: 10.1038/s44321-024-00060-y (PMC11099189; doi:10.1038/s44321-024-00060-y)
Supplement: Supplementary file 2 — Appendix [file 44321_2024_60_MOESM2_ESM.pdf]

Appendix Information:

Genome-wide CRISPR screens identify PKMYT1 as a therapeutic target in pancreatic ductal adenocarcinoma

Authors: Simin Wang[1], Yangjie Xiong[1], Yuxiang Luo[1], Yanying Shen[2], Fengrui Zhang[3], Haoqi Lan[1], Yuzhi Pang[1], Xiaofang Wang[1], Xiaoqi Li[4], Xufen Zheng[1], Xiaojing Lu[1], Xiaoxiao Liu[1], Yumei Cheng[1], Tanwen Wu[1], Yue Dong[1], Yuan Lu[3], Jiujie Cui[5], Xiaona Jia[1], Sheng Yang[6], Liwei Wang[5, †, *], Yuexiang Wang[1, †, *]

Author Affiliations:

[1]CAS Key Laboratory of Tissue Microenvironment and Tumor, Shanghai Institute of Nutrition and Health, Chinese Academy of Sciences , University of Chinese Academy of Sciences, 200031, Shanghai, China.

[2]Department of Pathology, Ren Ji Hospital, School of Medicine, Shanghai Jiao Tong University, 200127, Shanghai, China.

[3]Department of Gynecology, Obstetrics and Gynecology Hospital, Fudan University, 200011, Shanghai, China.

[4]Department of Gastrointestinal Surgery, Ren Ji Hospital, School of Medicine, Shanghai Jiao Tong University, 200127, Shanghai, China.

[5]Department of Oncology, Ren Ji Hospital, School of Medicine, Shanghai Jiao Tong University, 200127, Shanghai, China.

[6]Departments of Oncology, Fujian Medical University Union Hospital, 350001, Fuzhou, Fujian, China.

†These authors jointly supervised this work.

*Corresponding authors

This file includes:

Appendix Figure S1 2

Appendix Figure S2 3

Appendix Figure S3 5

Appendix Figure S4 6

Appendix Figure S5 7

Appendix Figure S1

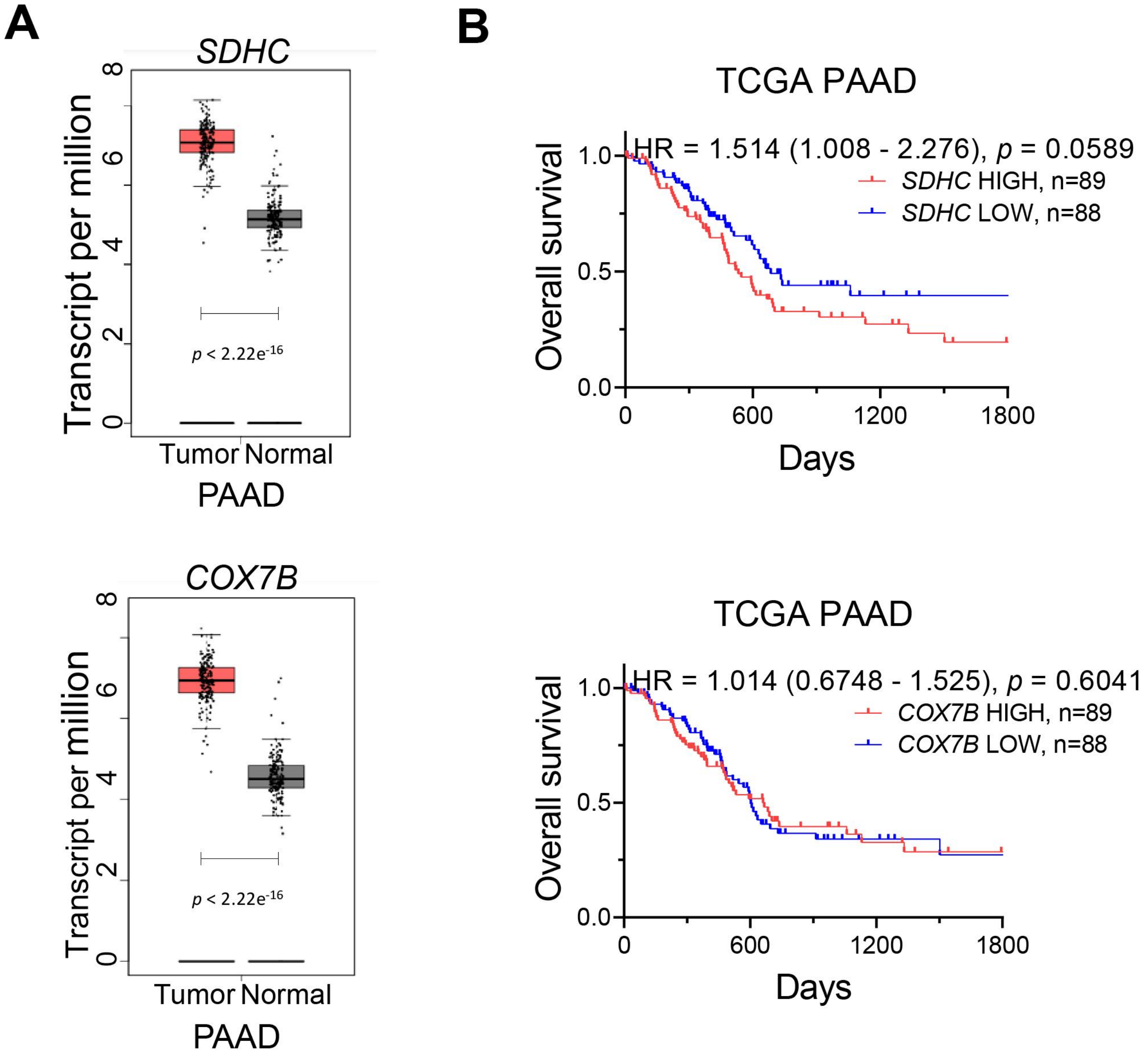

Appendix Figure S1: The expression and prognostic significance of the candidate genes identified by screening in PDAC.

A. Expression of *SDHC* and *COX7B* in human pancreatic cancer tissues (n = 179) compared with noncancerous tissues (n = 171) from the TCGA-PAAD cohort. The low bound, centerline, and upper bound of boxplot represent the first quartile, the median, and the third quartile of data, respectively; the upper and lower whiskers extend to the largest and smallest value within 1.5 times of the interquartile range. Unpaired *t* test; pearson correlation coefficient was used.

B. Kaplan–Meier survival curves for overall survival based on *SDHC* and *COX7B* expression using the TCGA pancreatic adenocarcinoma dataset (n = 177).

Appendix Figure S2

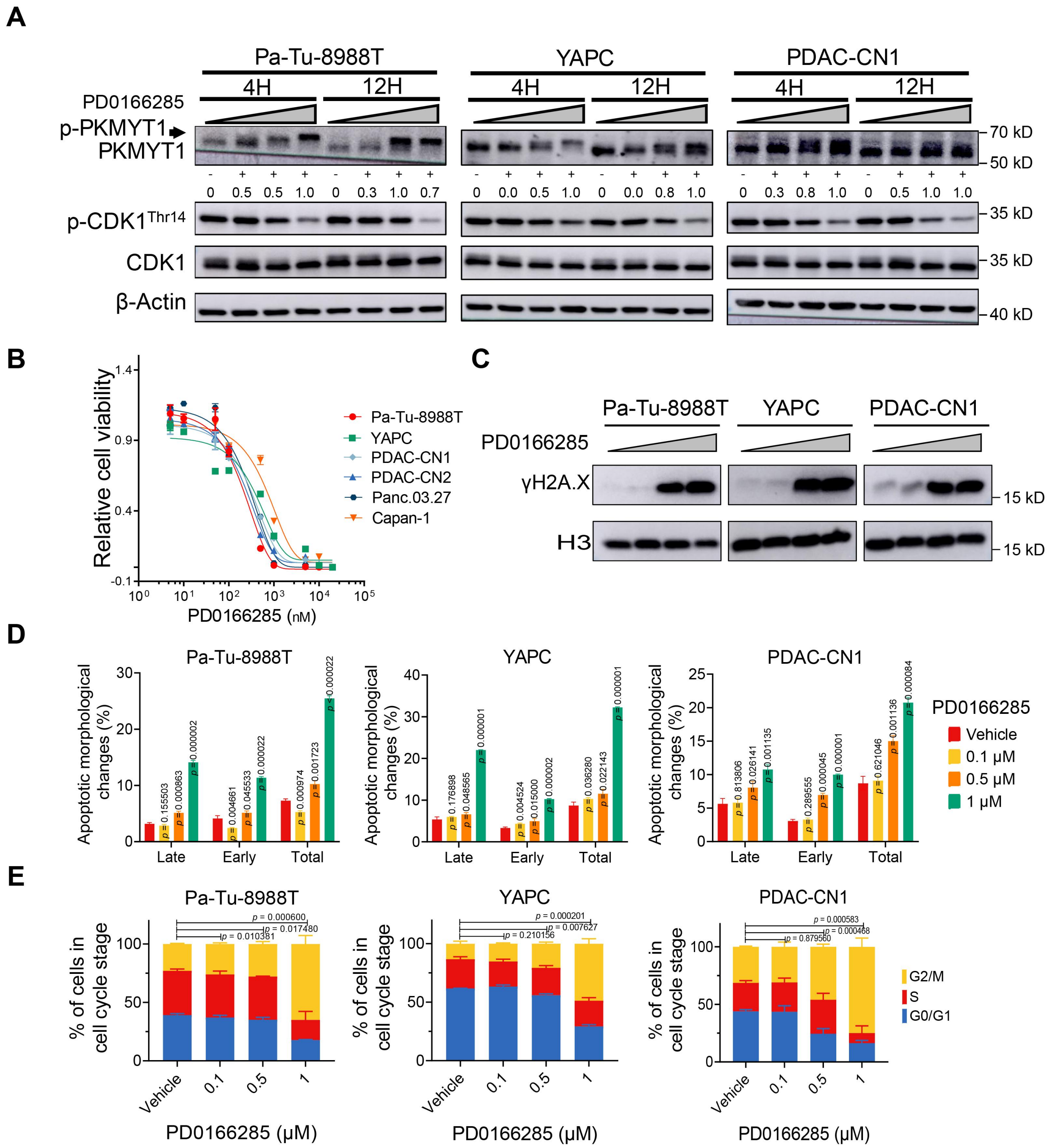

Appendix Figure S2: Other PKMYT1 inhibitor, such as the pyridopyrimidine derivative PD0166285, reduces cell viability in multiple PDAC cell models and mimics PKMYT1 knockout in regard to apoptosis and cell cycle progression.

A. PD016628 treatment increases the hyperphosphorylation of PKMYT1 and inhibits phosphorylation of CDK1 Thr14. The mobility of PKMYT1 was made qualitative and relative quantitative assessments.

B. PD0166285 is an effective inhibitor in PDAC cell lines and primary cultured cell. The error bars indicate the

mean ± s.e.m. of 3 replicates.

C. PD016628 treatment increases γH2A.X accumulation.

D. PD0166285 treatment increases apoptosis, as determined by flow cytometry. The error bars indicate the mean ± s.d. of 3 replicates. Unpaired *t* test.

E. PD0166285 treatment causes cell cycle arrest, as determined by flow cytometry. The error bars indicate the mean ± s.d. of 3 replicates. Unpaired *t* test.

Concentrations of compound used (A, C) are listed in “Methods”. Appendix Fig. S2D, E represent data from two biological replicates.

Appendix Figure S3

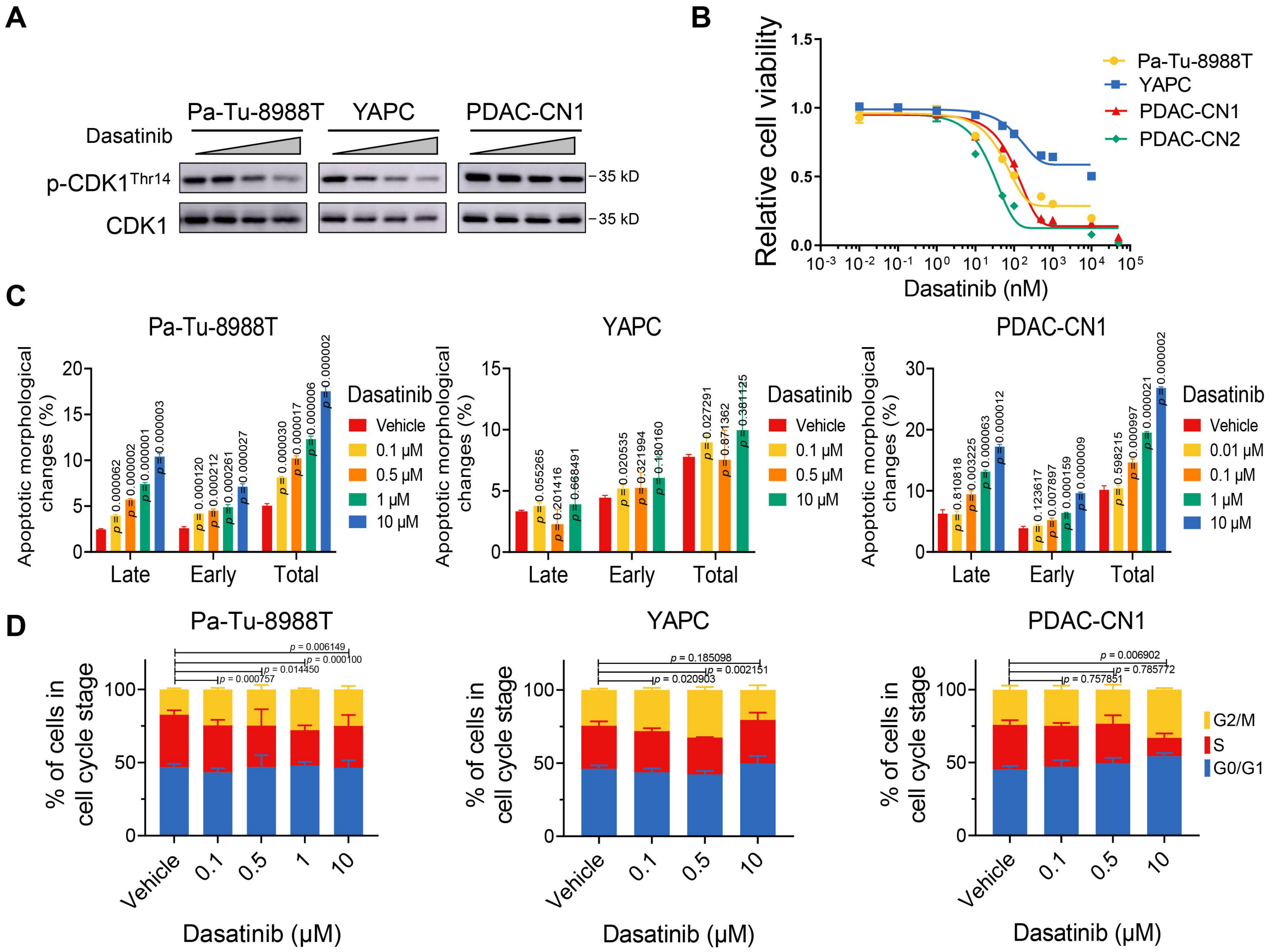

Appendix Figure S3: Other PKMYT1 inhibitor, such as dasatinib, reduces cell viability in multiple PDAC cell models and mimics PKMYT1 knockout in regard to apoptosis and cell cycle progression.

A. Dasatinib treatment decreases phosphorylation of CDK1 Thr14. Concentrations of compound used are listed in "Methods".

B. Dasatinib is an effective inhibitor in PDAC cells. The error bars indicate the mean ± s.e.m. of 3 replicates.

C. Dasatinib treatment increases apoptosis, as determined by flow cytometry. The error bars indicate the mean ± s.d. of 3 replicates. Unpaired *t* test.

D. Dasatinib treatment causes cell cycle arrest, as determined by flow cytometry. The error bars indicate the mean ± s.d. of 3 replicates. Unpaired *t* test.

Appendix Fig. S3C, D represent data from two biological replicates.

Appendix Figure S4

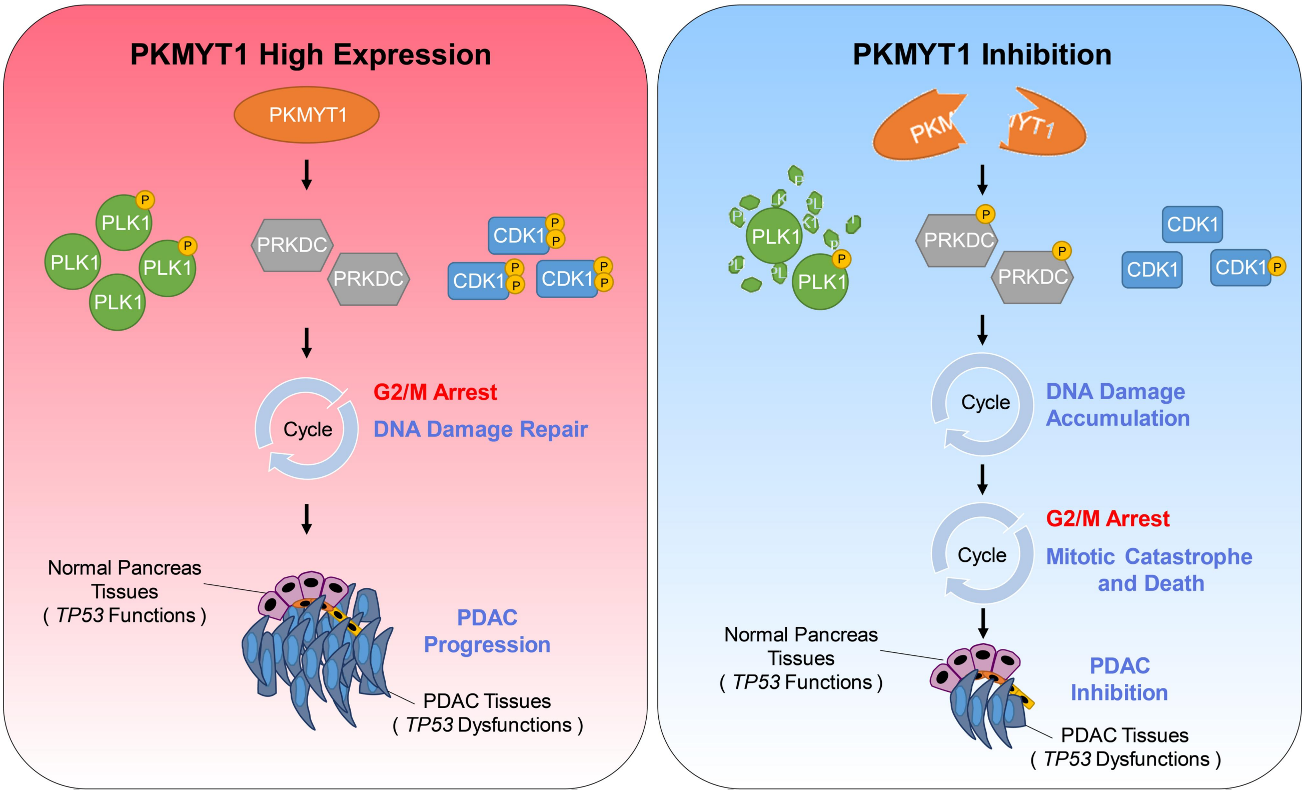

Appendix Figure S4: A model depicting how the PKMYT1 pathways control cell proliferation and progression in PDAC. Left: The PDAC cells with high PKMYT1 expression. Right: The PDAC cells with PKMYT1 inhibition.

Appendix Figure S5

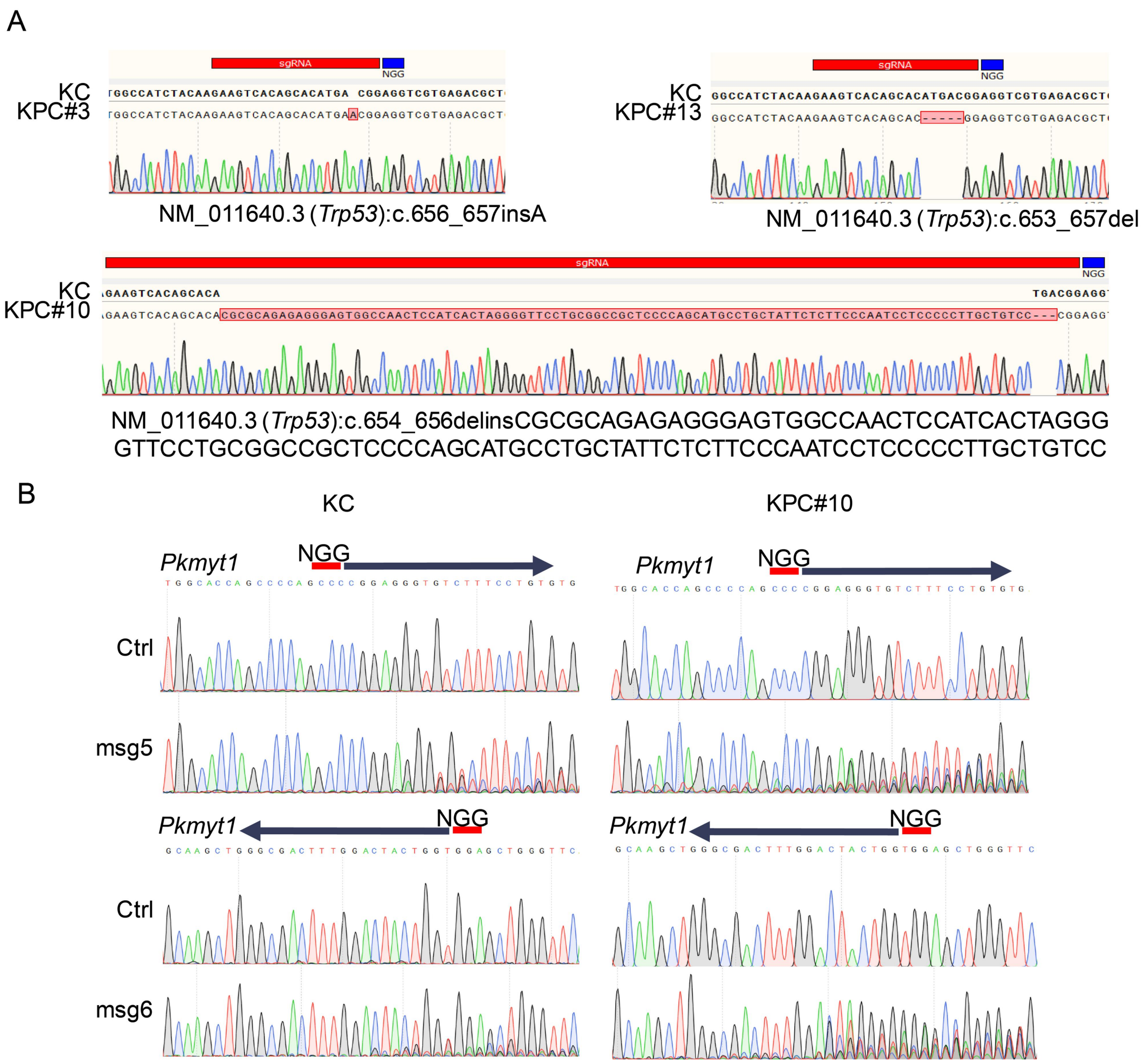

Appendix Figure S5: Sanger sequencing confirms *Trp53* and *Pkmyt1* mutation status.

A. Sanger sequencing confirms *Trp53* inactivation in KPC cells.

B. Sanger sequencing validates indel mutations induced by *Pkmyt1* sgRNA in KC and KPC cells.
