# Supplementary figures and images for: Genome-wide CRISPR screens identify PKMYT1 as a therapeutic target in pancreatic ductal adenocarcinoma (part 1 of 4)
Source: EMBO Mol Med. 2024 Apr 3;16(5):5. doi: 10.1038/s44321-024-00060-y (PMC11099189; doi:10.1038/s44321-024-00060-y)

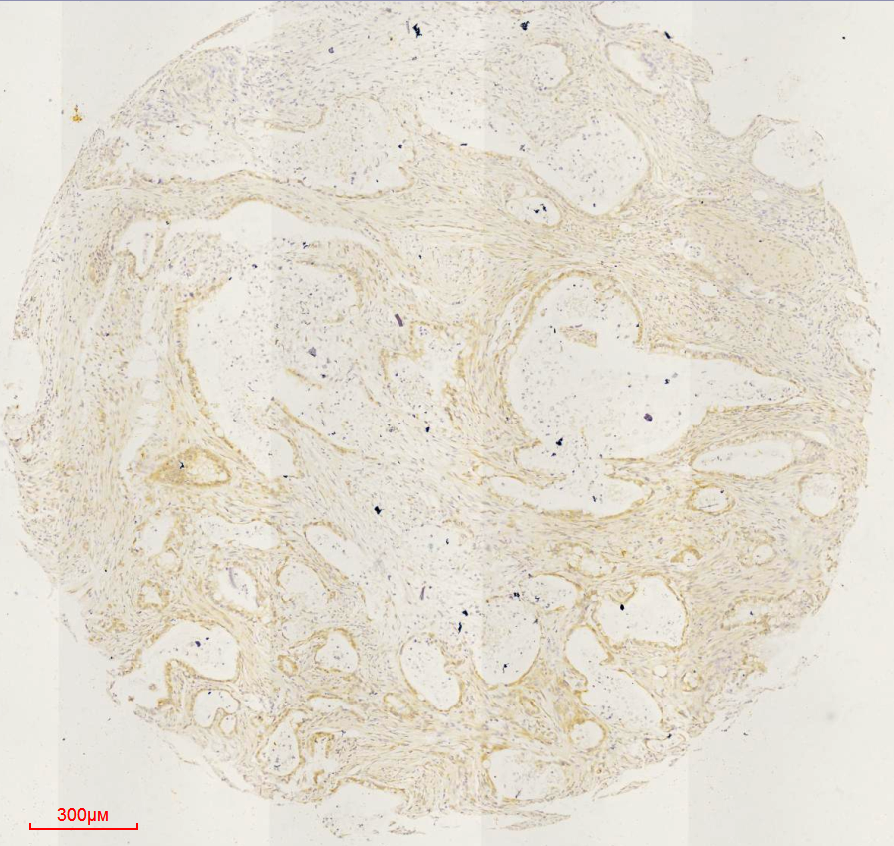

Supplement: Supplementary file 7 — Source data Fig. 2 [file 44321_2024_60_MOESM7_ESM.zip › Figure 2/2A/PKMYT1-High 1/300um .png]

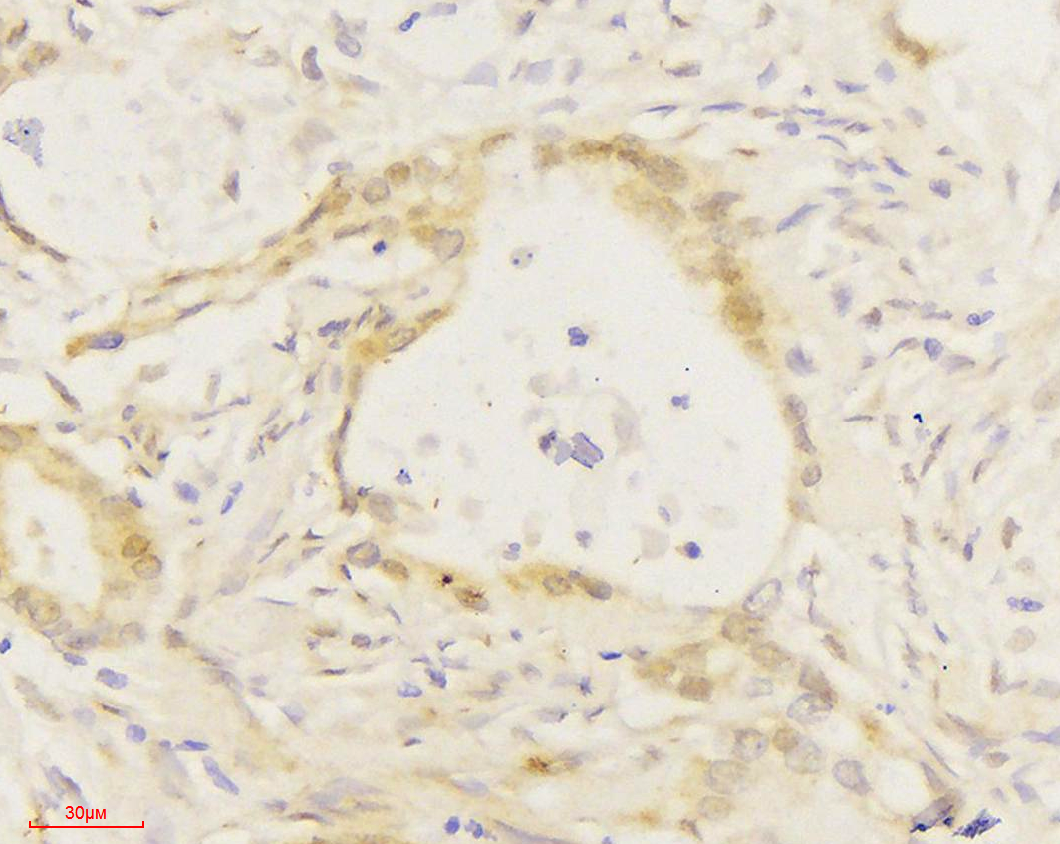

Supplement: Supplementary file 7 — Source data Fig. 2 [file 44321_2024_60_MOESM7_ESM.zip › Figure 2/2A/PKMYT1-High 1/30um .png]

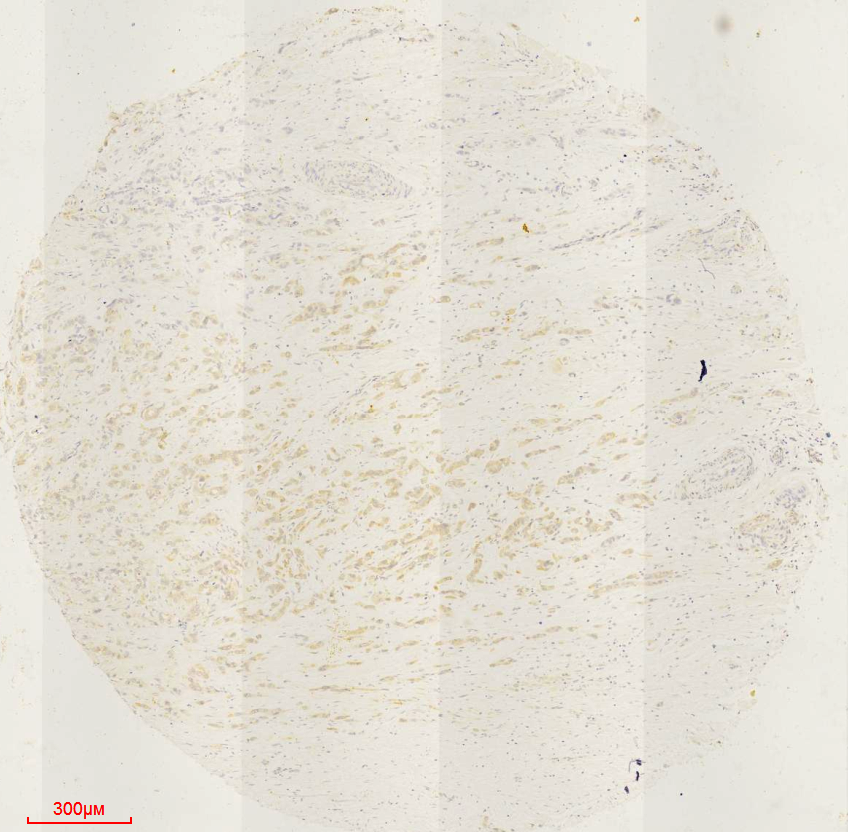

Supplement: Supplementary file 7 — Source data Fig. 2 [file 44321_2024_60_MOESM7_ESM.zip › Figure 2/2A/PKMYT1-High 2/300um .png]

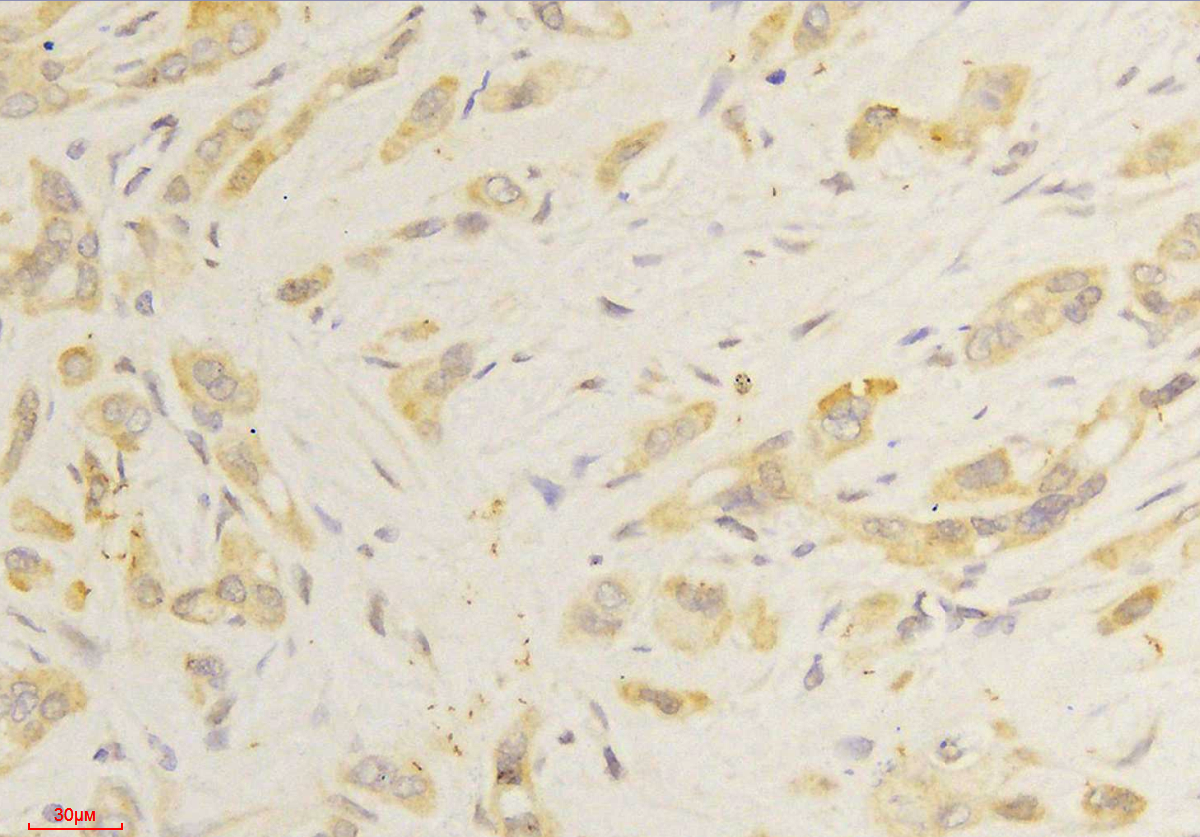

Supplement: Supplementary file 7 — Source data Fig. 2 [file 44321_2024_60_MOESM7_ESM.zip › Figure 2/2A/PKMYT1-High 2/30um .PNG]

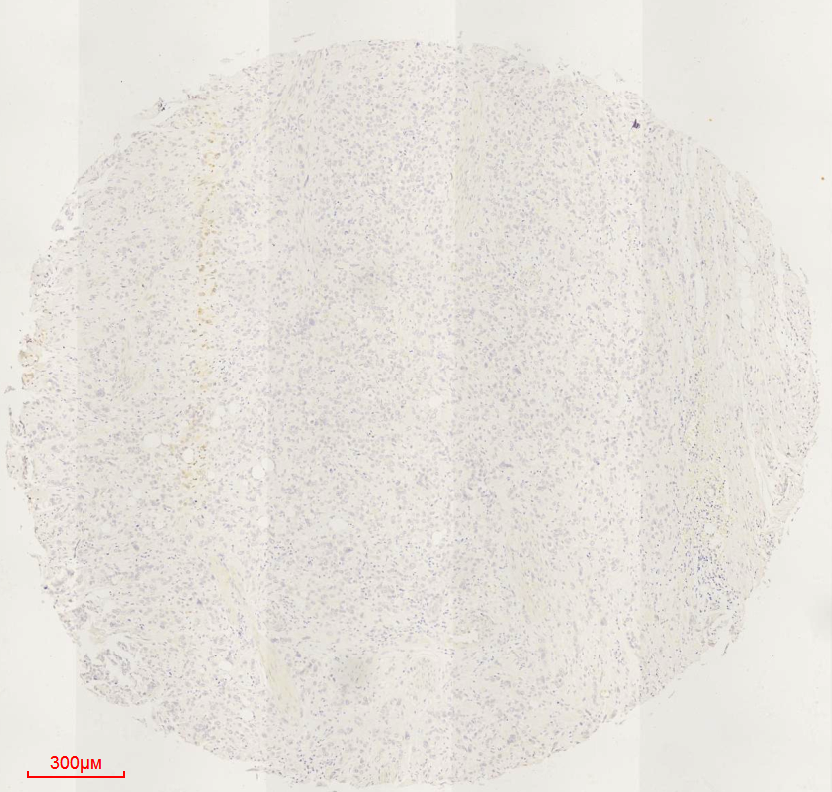

Supplement: Supplementary file 7 — Source data Fig. 2 [file 44321_2024_60_MOESM7_ESM.zip › Figure 2/2A/PKMYT1-Low 1/300um .PNG]

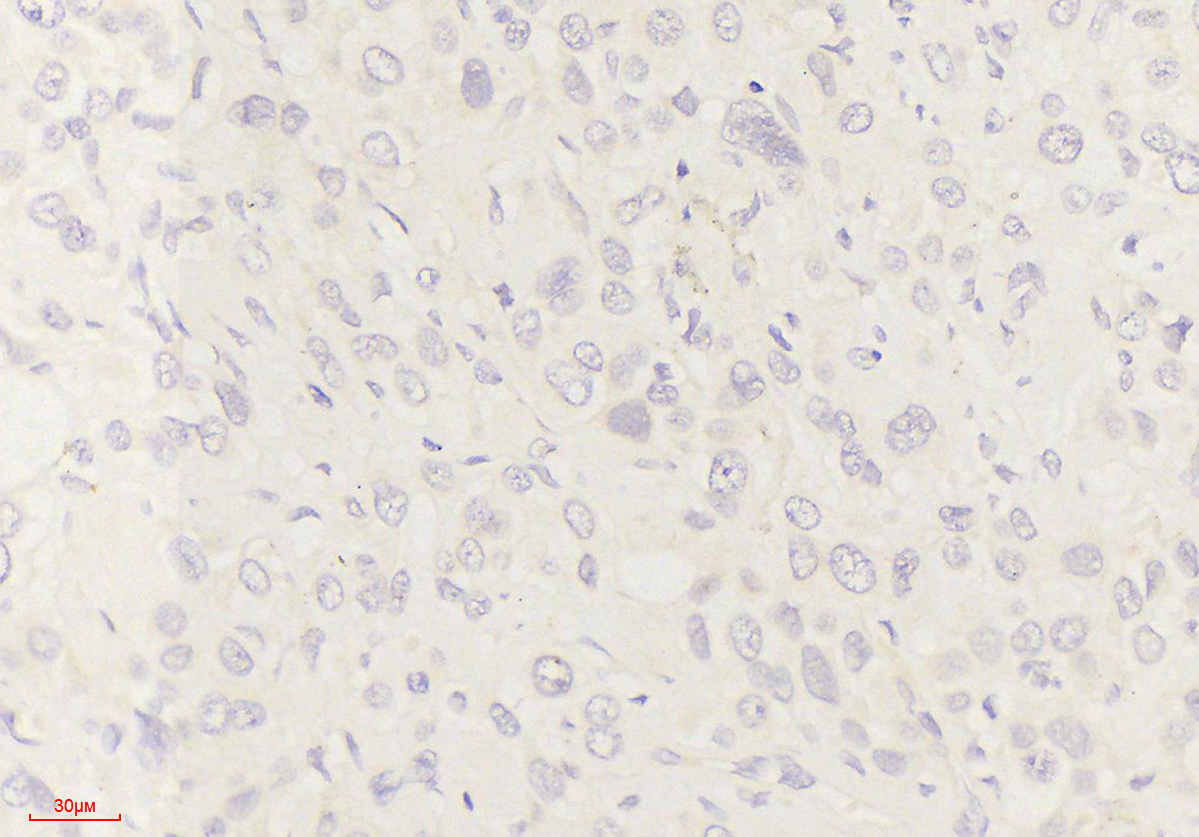

Supplement: Supplementary file 7 — Source data Fig. 2 [file 44321_2024_60_MOESM7_ESM.zip › Figure 2/2A/PKMYT1-Low 1/30um .PNG]

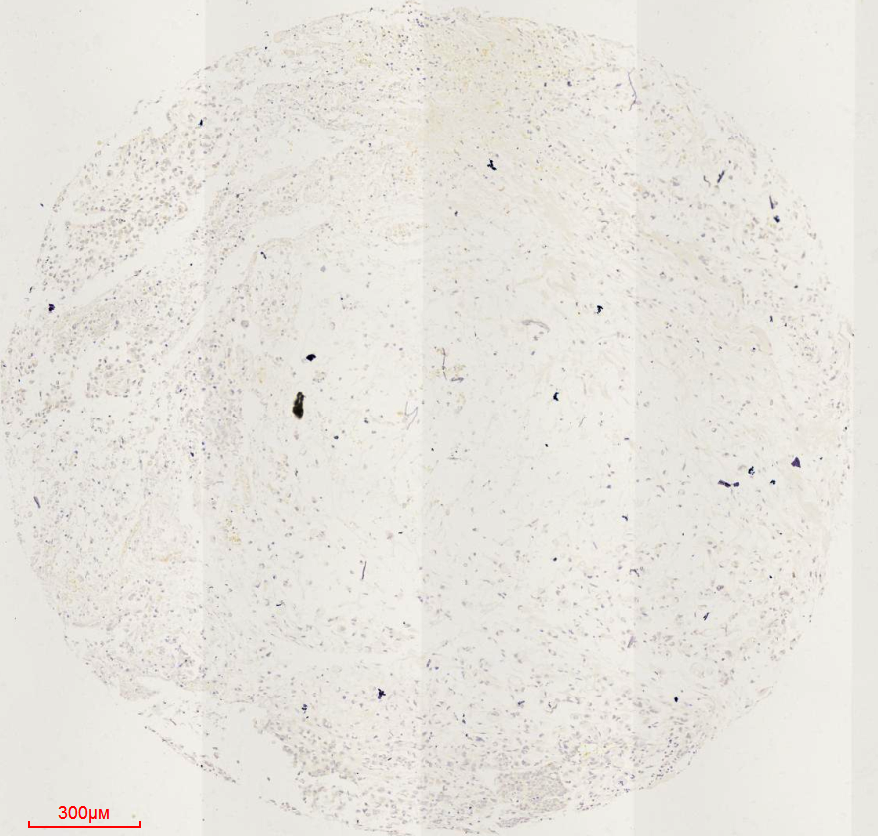

Supplement: Supplementary file 7 — Source data Fig. 2 [file 44321_2024_60_MOESM7_ESM.zip › Figure 2/2A/PKMYT1-Low 2/300um .png]

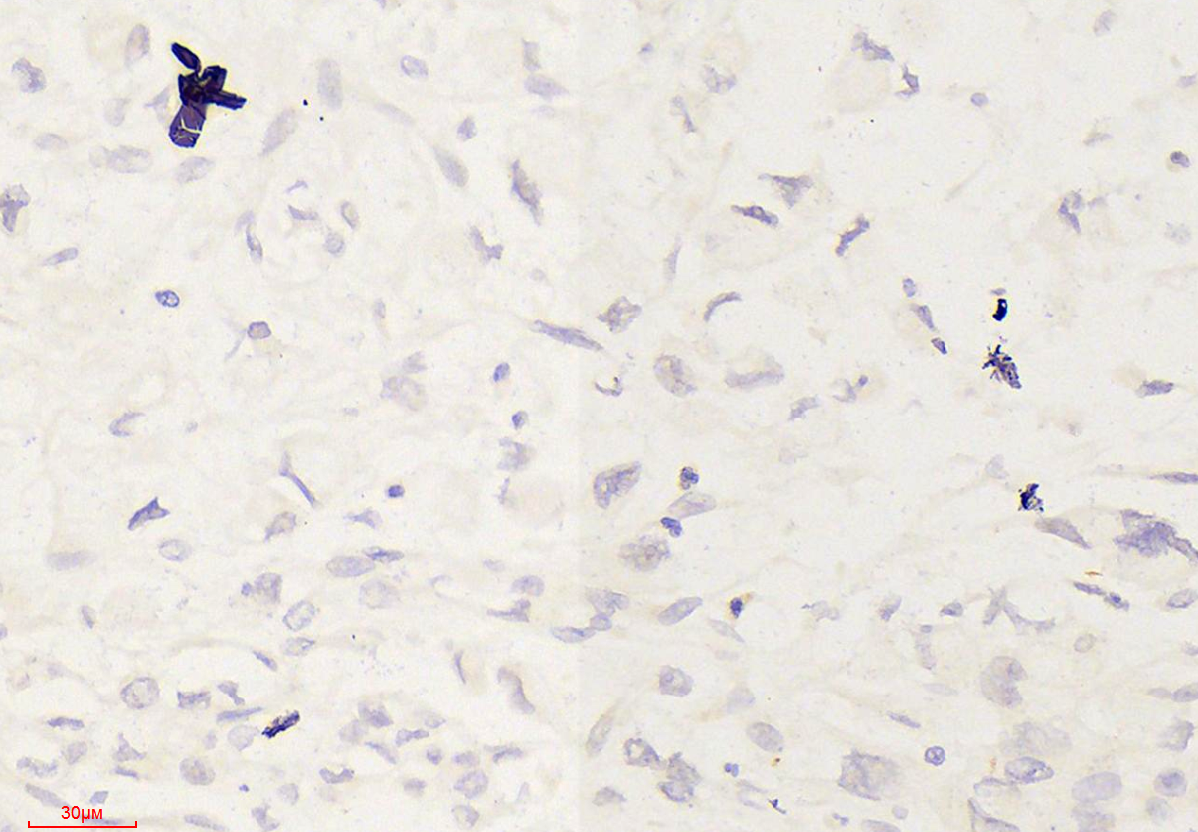

Supplement: Supplementary file 7 — Source data Fig. 2 [file 44321_2024_60_MOESM7_ESM.zip › Figure 2/2A/PKMYT1-Low 2/30um .png]

## Slide 1
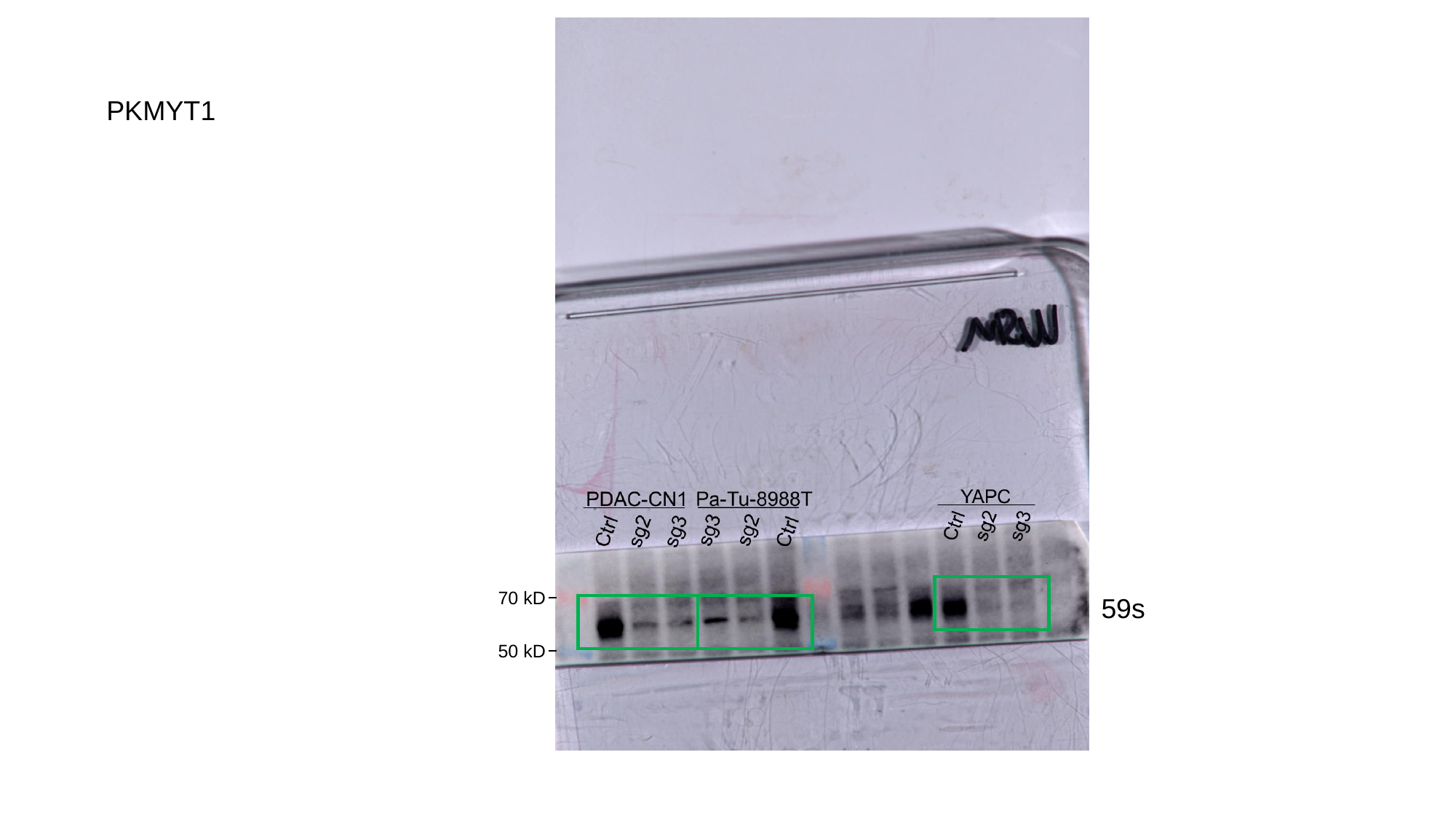

PKMYT1
70 kD
59s
50 kD

## Slide 2
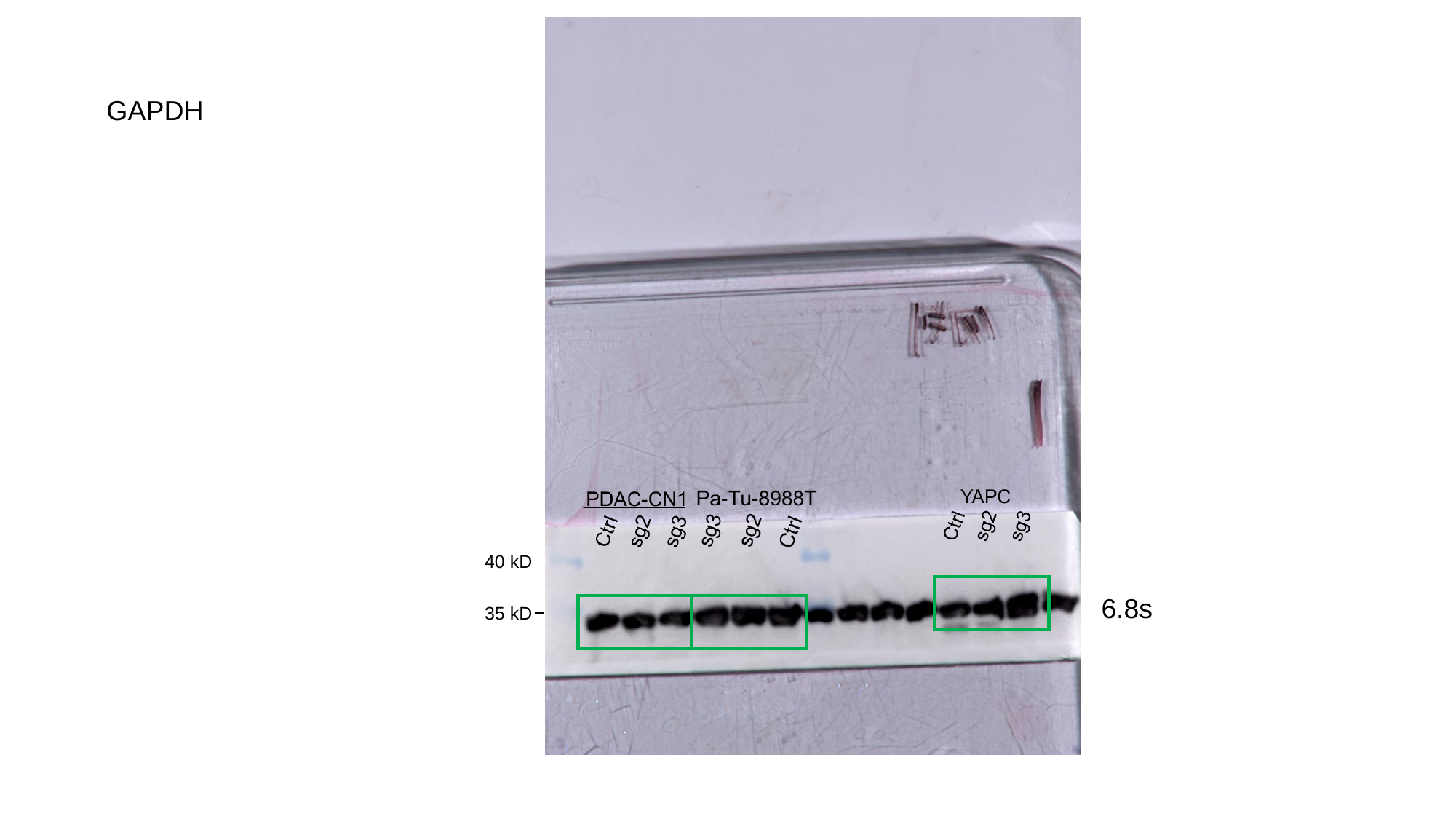

GAPDH
40 kD
6.8s
35 kD

Supplement: Supplementary file 8 — Source data Fig. 3 [file 44321_2024_60_MOESM8_ESM.zip › Source data-Figure 3 (44321_2024_60_MOESM8_ESM)_updated/Figure 3/3A/3A.pptx]

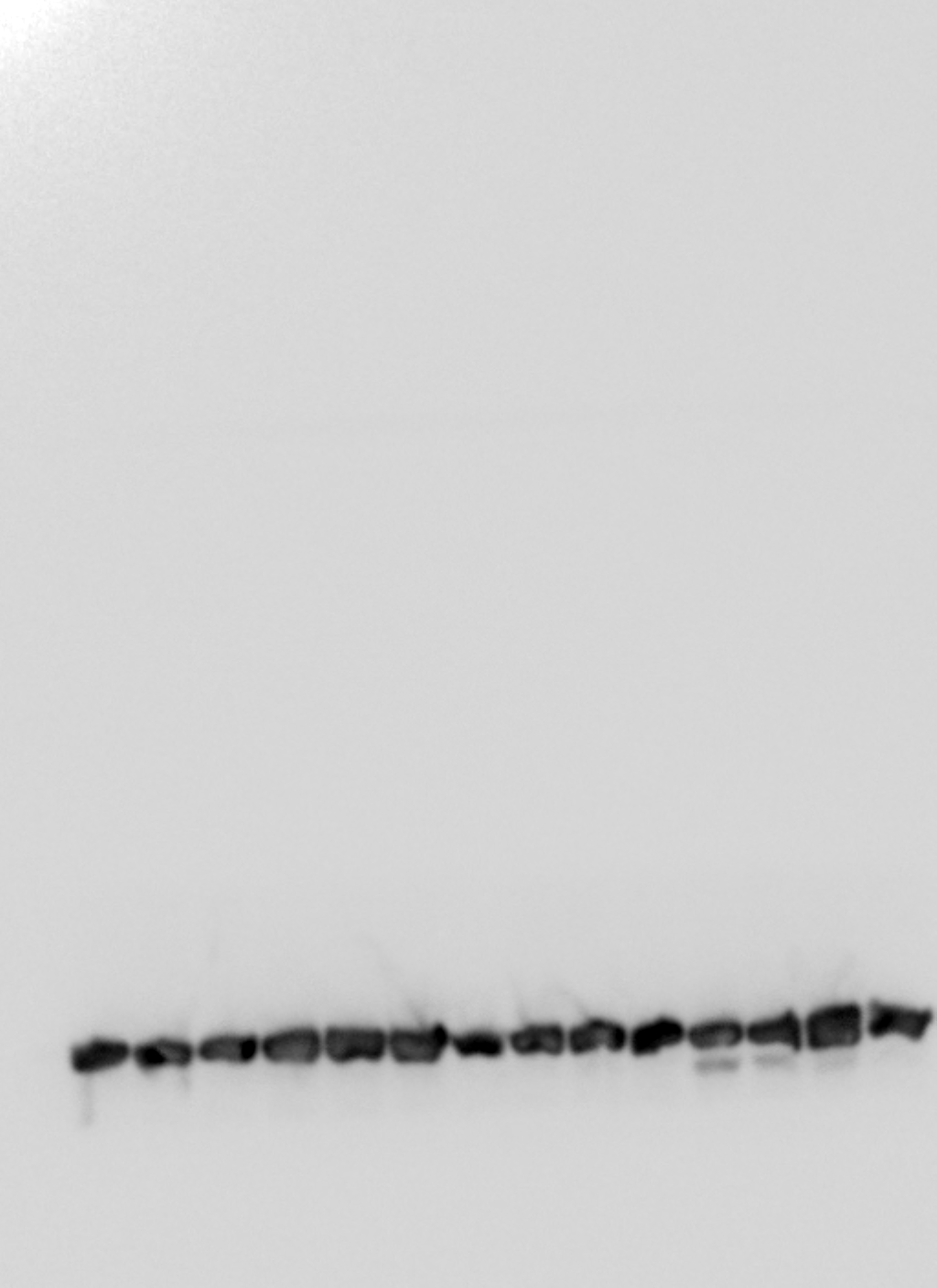

Supplement: Supplementary file 8 — Source data Fig. 3 [file 44321_2024_60_MOESM8_ESM.zip › Source data-Figure 3 (44321_2024_60_MOESM8_ESM)_updated/Figure 3/3A/Western GAPDH/wsm 7 GAPDH 6.8 _Ch.tif]

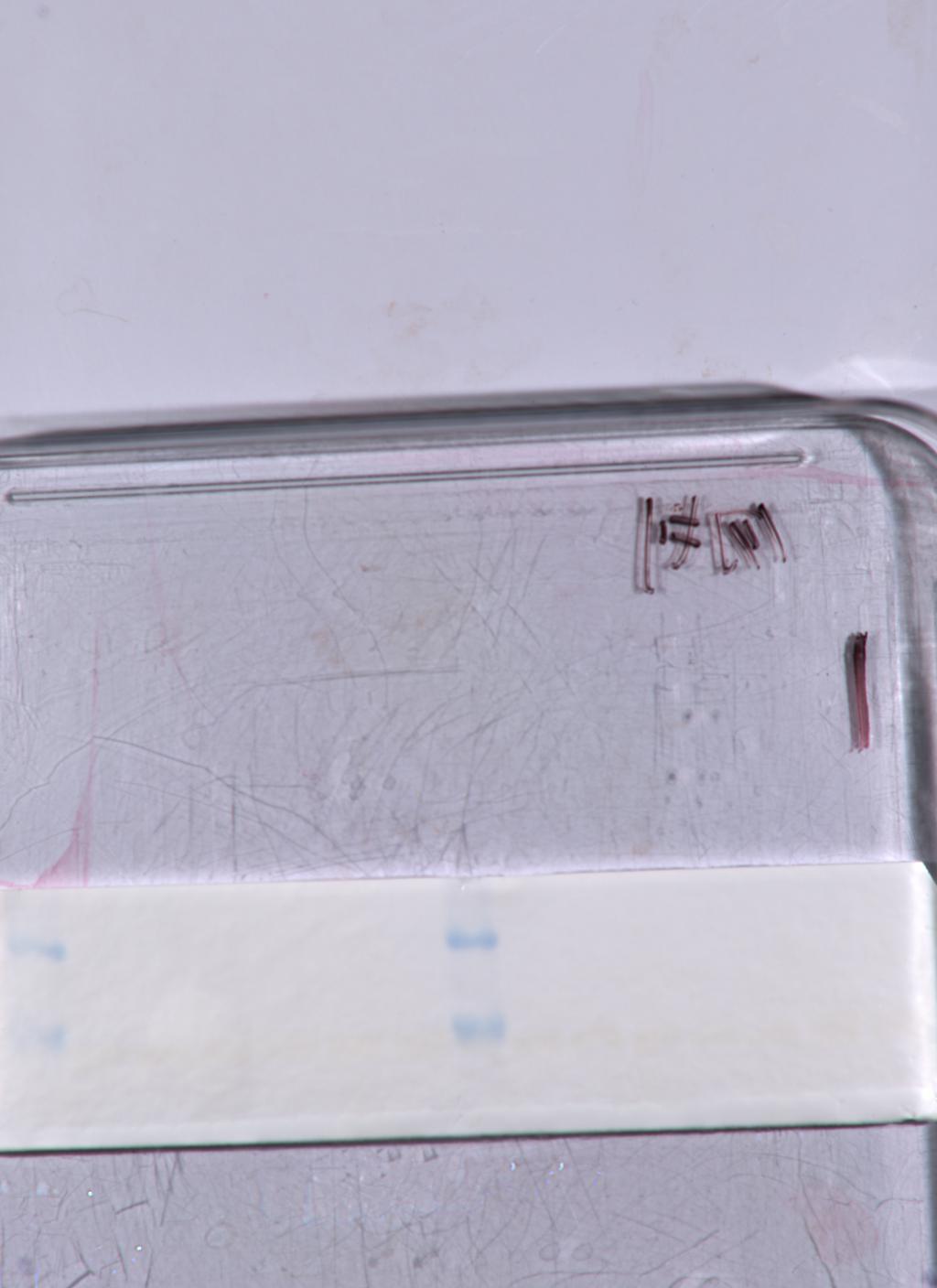

Supplement: Supplementary file 8 — Source data Fig. 3 [file 44321_2024_60_MOESM8_ESM.zip › Source data-Figure 3 (44321_2024_60_MOESM8_ESM)_updated/Figure 3/3A/Western GAPDH/wsm 7 GAPDH 6.8 _Ch-Marker.jpg]

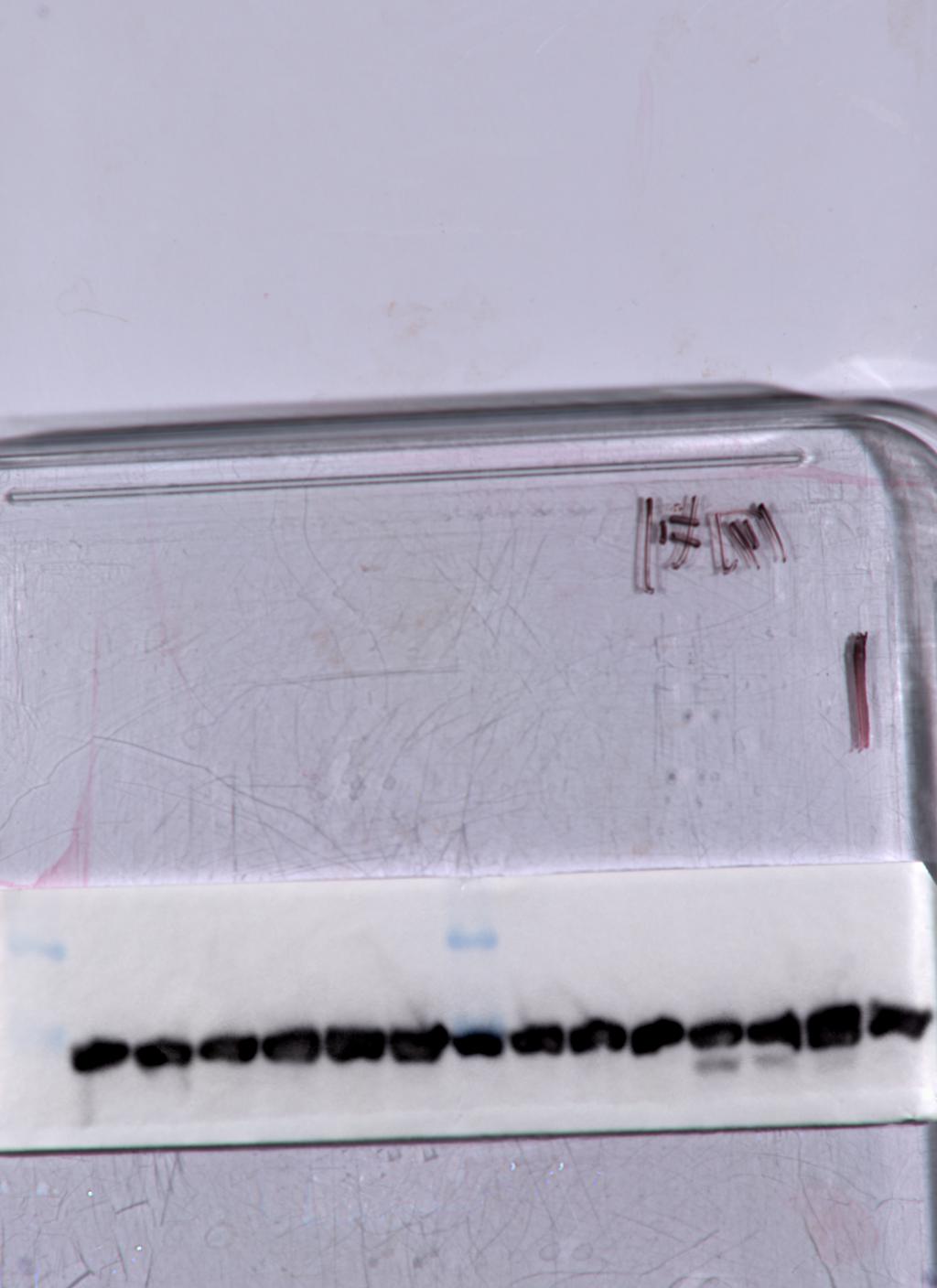

Supplement: Supplementary file 8 — Source data Fig. 3 [file 44321_2024_60_MOESM8_ESM.zip › Source data-Figure 3 (44321_2024_60_MOESM8_ESM)_updated/Figure 3/3A/Western GAPDH/wsm 7 GAPDH 6.8 2_Ch+Marker.jpg]

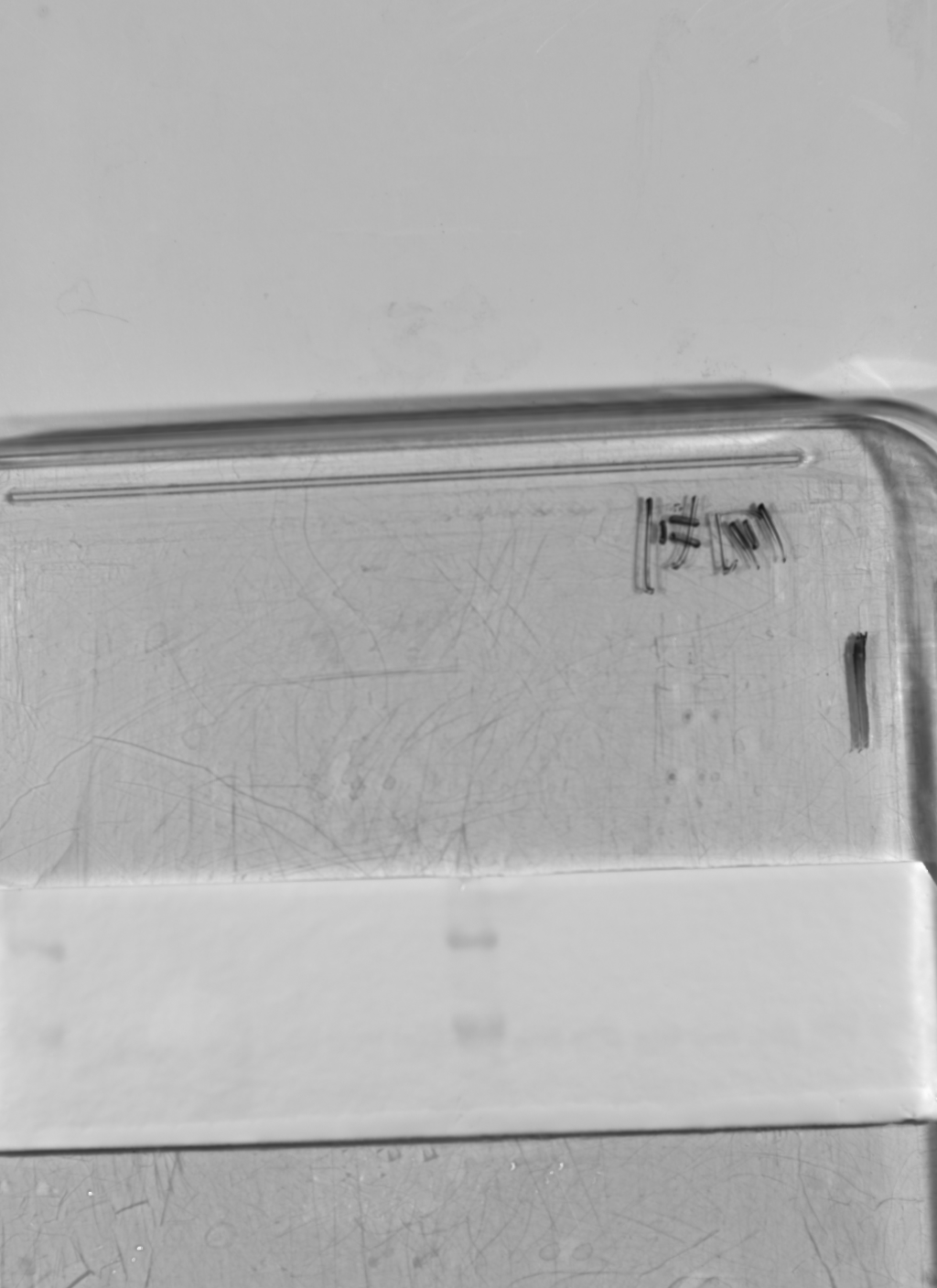

Supplement: Supplementary file 8 — Source data Fig. 3 [file 44321_2024_60_MOESM8_ESM.zip › Source data-Figure 3 (44321_2024_60_MOESM8_ESM)_updated/Figure 3/3A/Western GAPDH/wsm 7 GAPDH 6.8_Ch-Marker.tif]

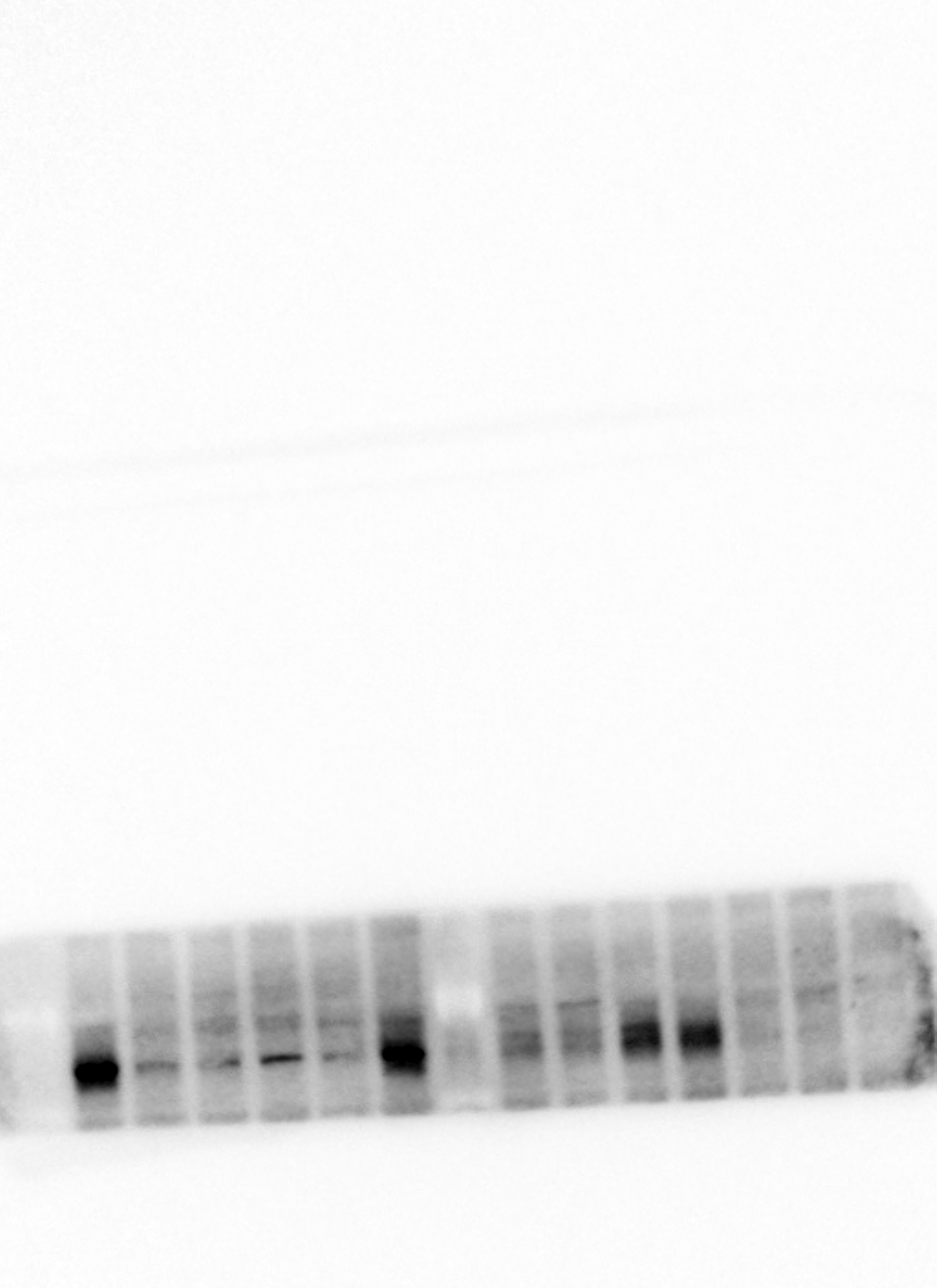

Supplement: Supplementary file 8 — Source data Fig. 3 [file 44321_2024_60_MOESM8_ESM.zip › Source data-Figure 3 (44321_2024_60_MOESM8_ESM)_updated/Figure 3/3A/Western PKMYT1/wsm 7 7th PKM 59_Ch.tif]

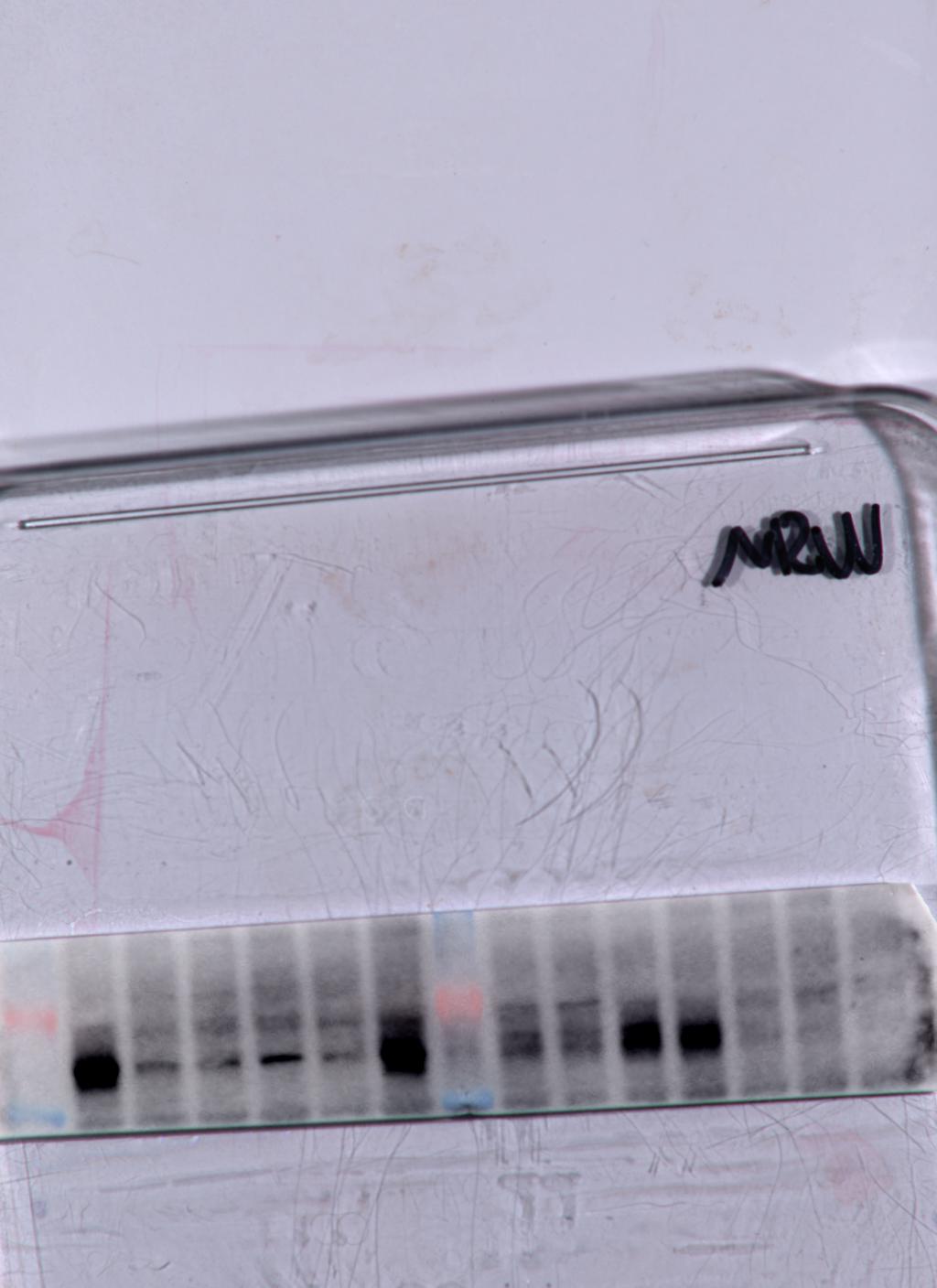

Supplement: Supplementary file 8 — Source data Fig. 3 [file 44321_2024_60_MOESM8_ESM.zip › Source data-Figure 3 (44321_2024_60_MOESM8_ESM)_updated/Figure 3/3A/Western PKMYT1/wsm 7 7th PKM 59_Ch+Marker.jpg]

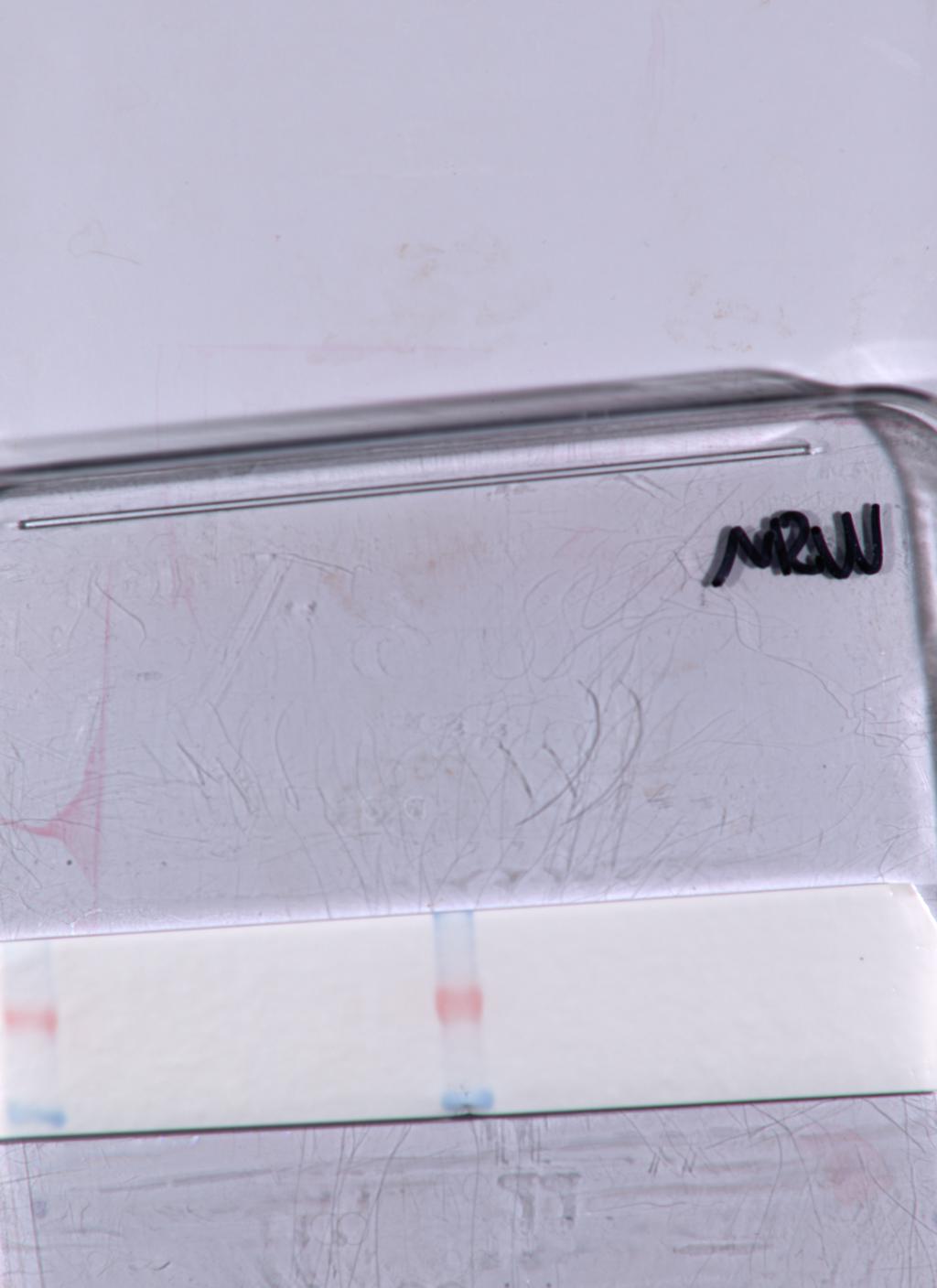

Supplement: Supplementary file 8 — Source data Fig. 3 [file 44321_2024_60_MOESM8_ESM.zip › Source data-Figure 3 (44321_2024_60_MOESM8_ESM)_updated/Figure 3/3A/Western PKMYT1/wsm 7 7th PKM 59_Ch-Marker.jpg]

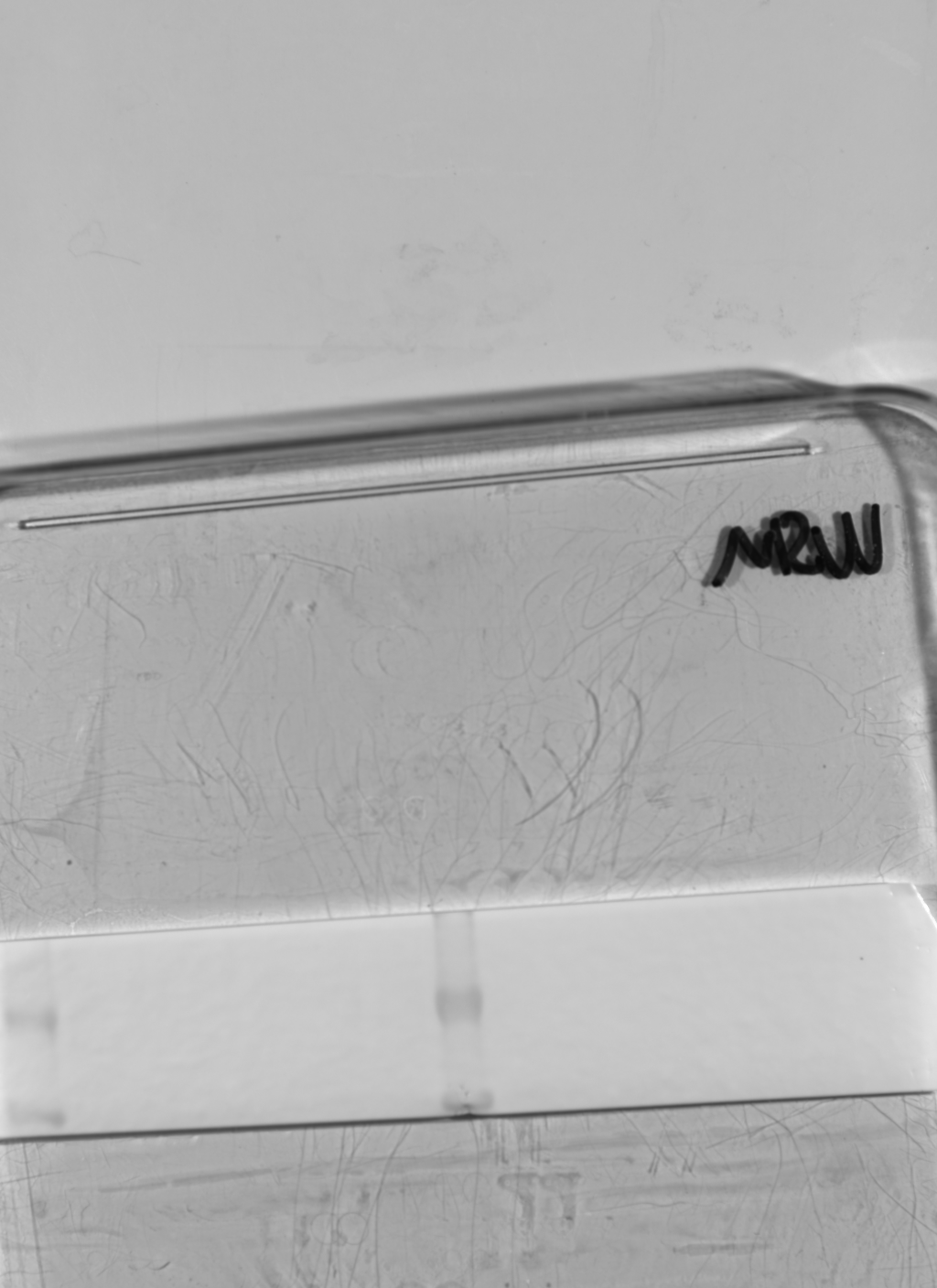

Supplement: Supplementary file 8 — Source data Fig. 3 [file 44321_2024_60_MOESM8_ESM.zip › Source data-Figure 3 (44321_2024_60_MOESM8_ESM)_updated/Figure 3/3A/Western PKMYT1/wsm 7 7th PKM 59_Ch-Marker.tif]

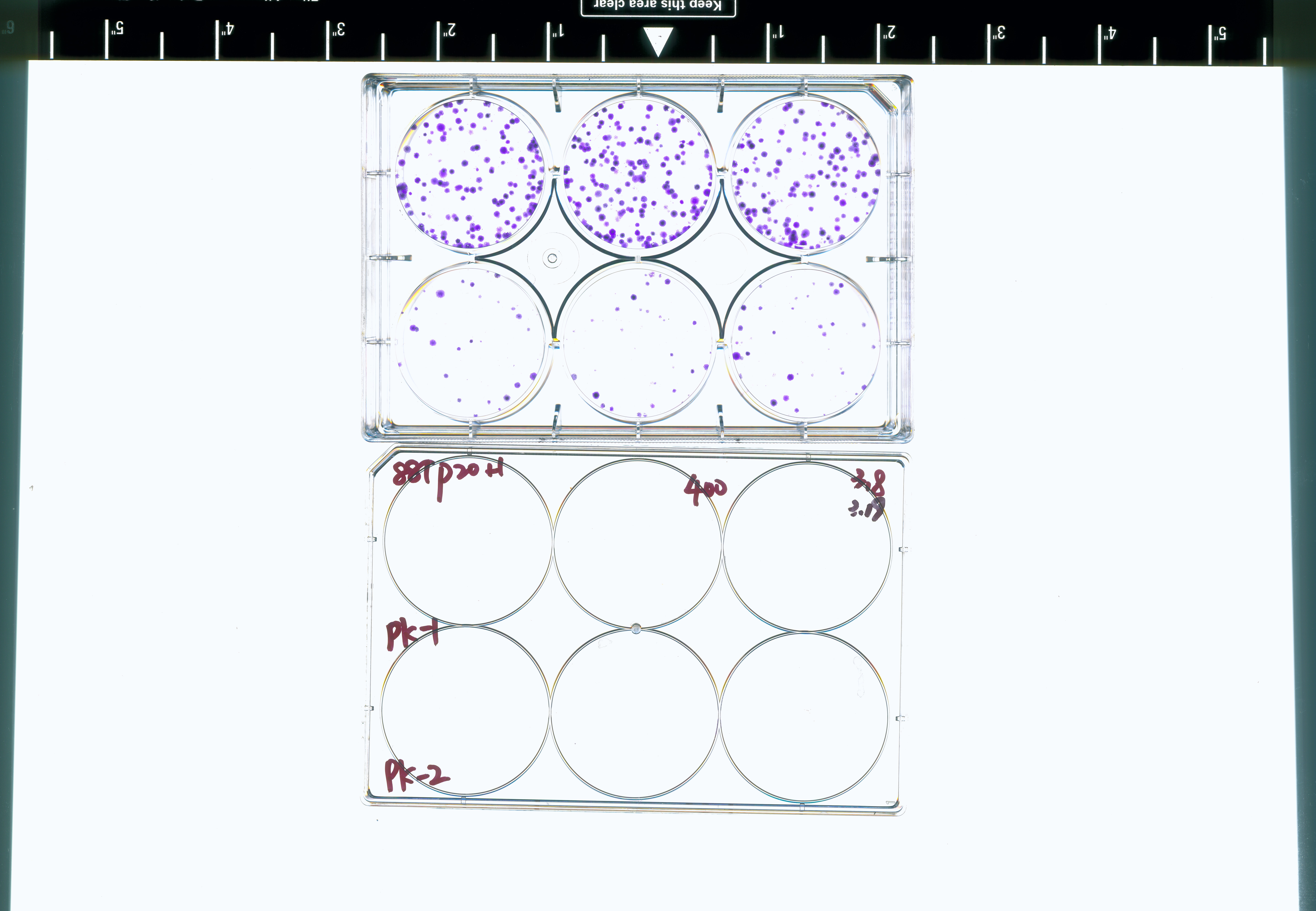

Supplement: Supplementary file 8 — Source data Fig. 3 [file 44321_2024_60_MOESM8_ESM.zip › Source data-Figure 3 (44321_2024_60_MOESM8_ESM)_updated/Figure 3/3C/88T/21.03.20 400 88T PK-1_PK-2 .jpg]

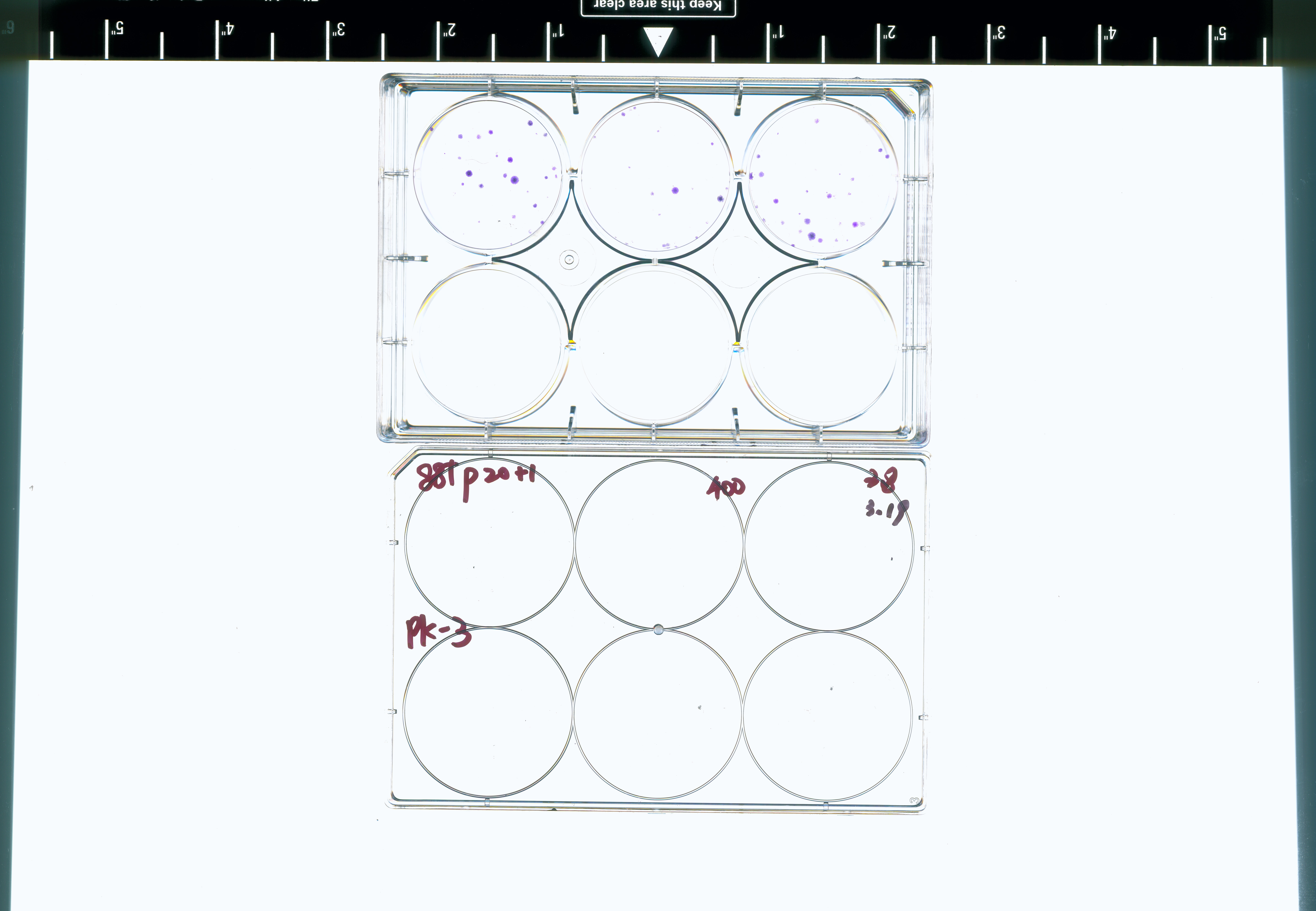

Supplement: Supplementary file 8 — Source data Fig. 3 [file 44321_2024_60_MOESM8_ESM.zip › Source data-Figure 3 (44321_2024_60_MOESM8_ESM)_updated/Figure 3/3C/88T/21.03.20 400 88T PK-3.jpg]

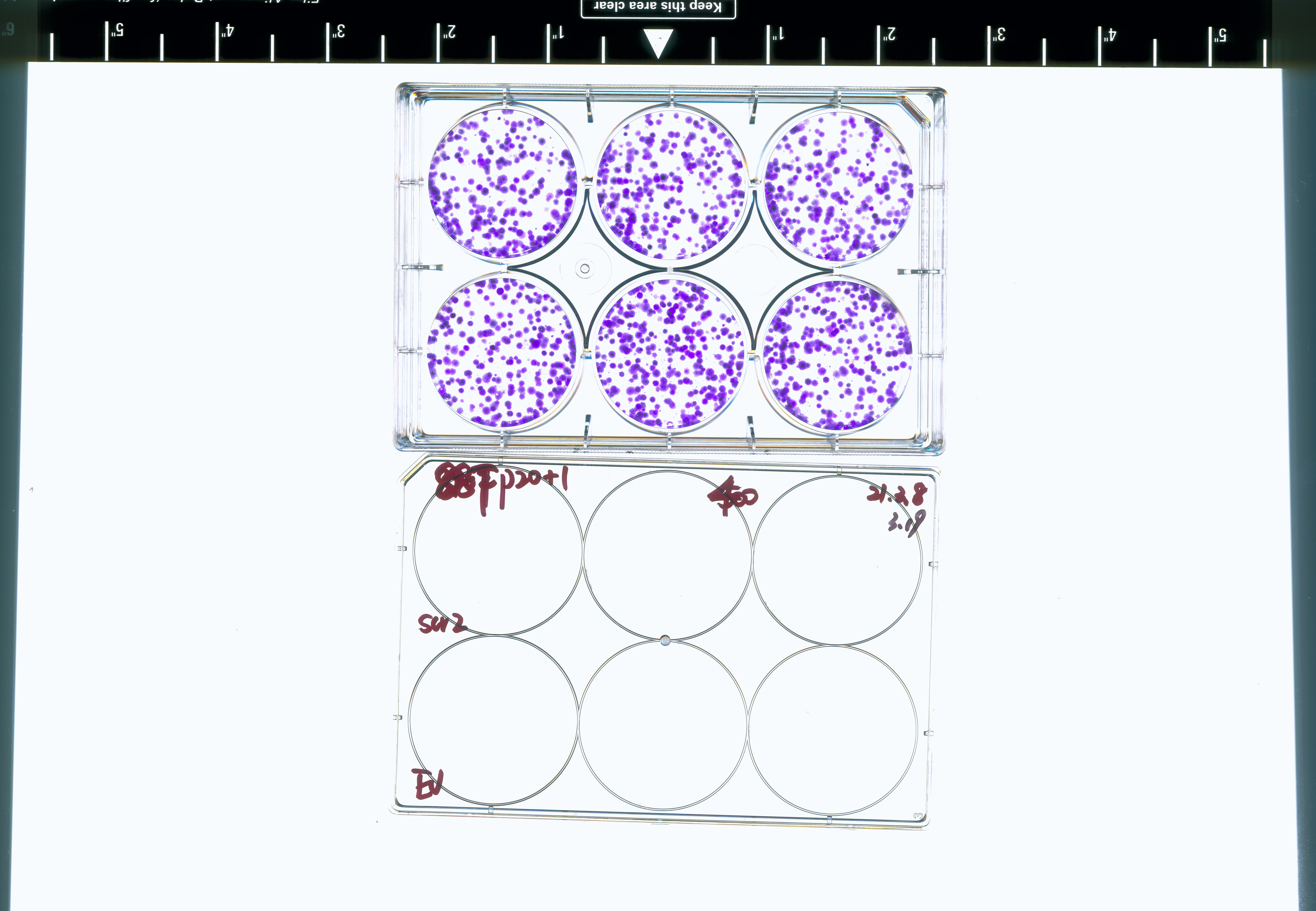

Supplement: Supplementary file 8 — Source data Fig. 3 [file 44321_2024_60_MOESM8_ESM.zip › Source data-Figure 3 (44321_2024_60_MOESM8_ESM)_updated/Figure 3/3C/88T/21.03.20 400 88T SCR EV.jpg]

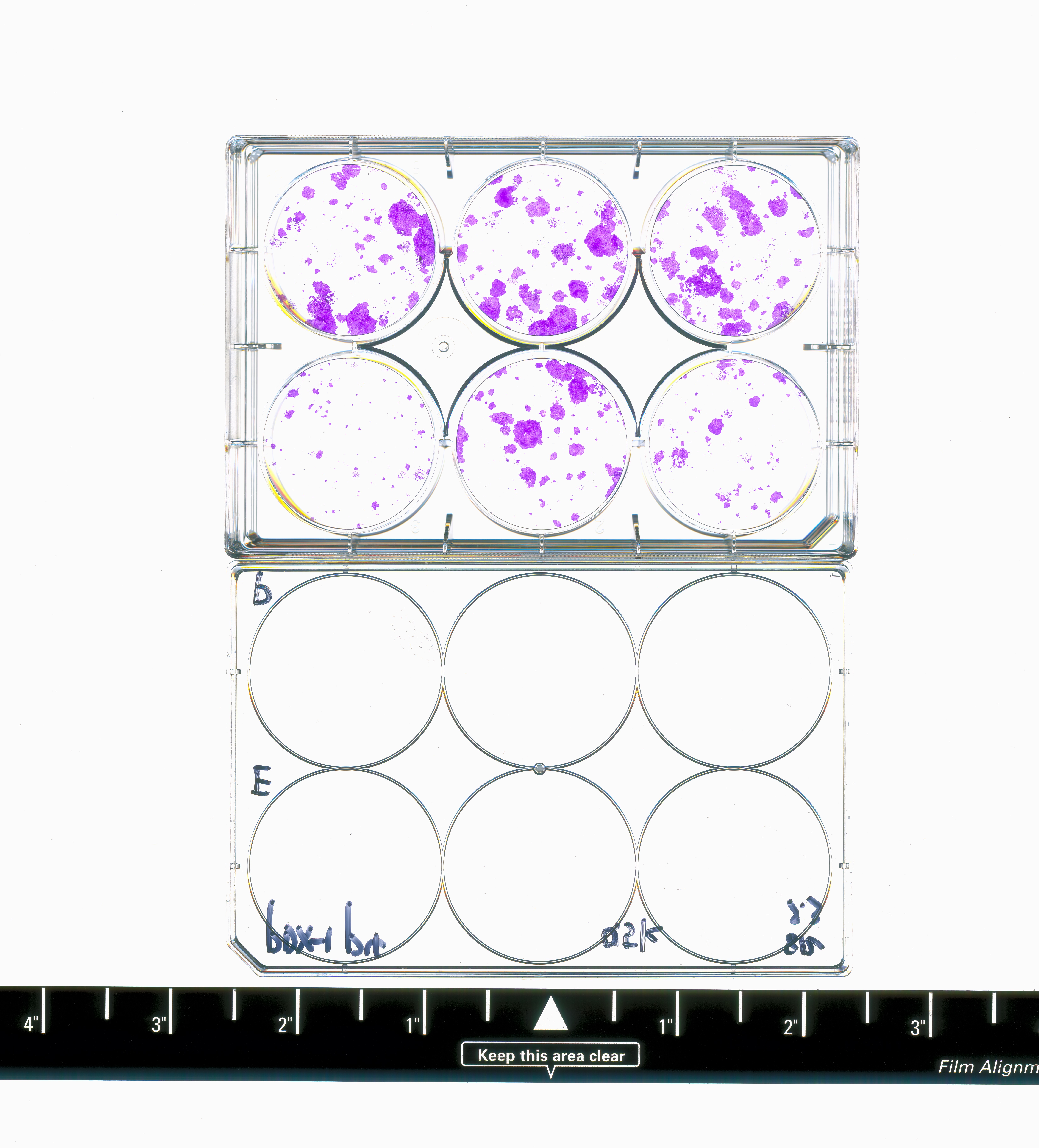

Supplement: Supplementary file 8 — Source data Fig. 3 [file 44321_2024_60_MOESM8_ESM.zip › Source data-Figure 3 (44321_2024_60_MOESM8_ESM)_updated/Figure 3/3C/CN1/21.09.06 PD1 EV P.jpg]

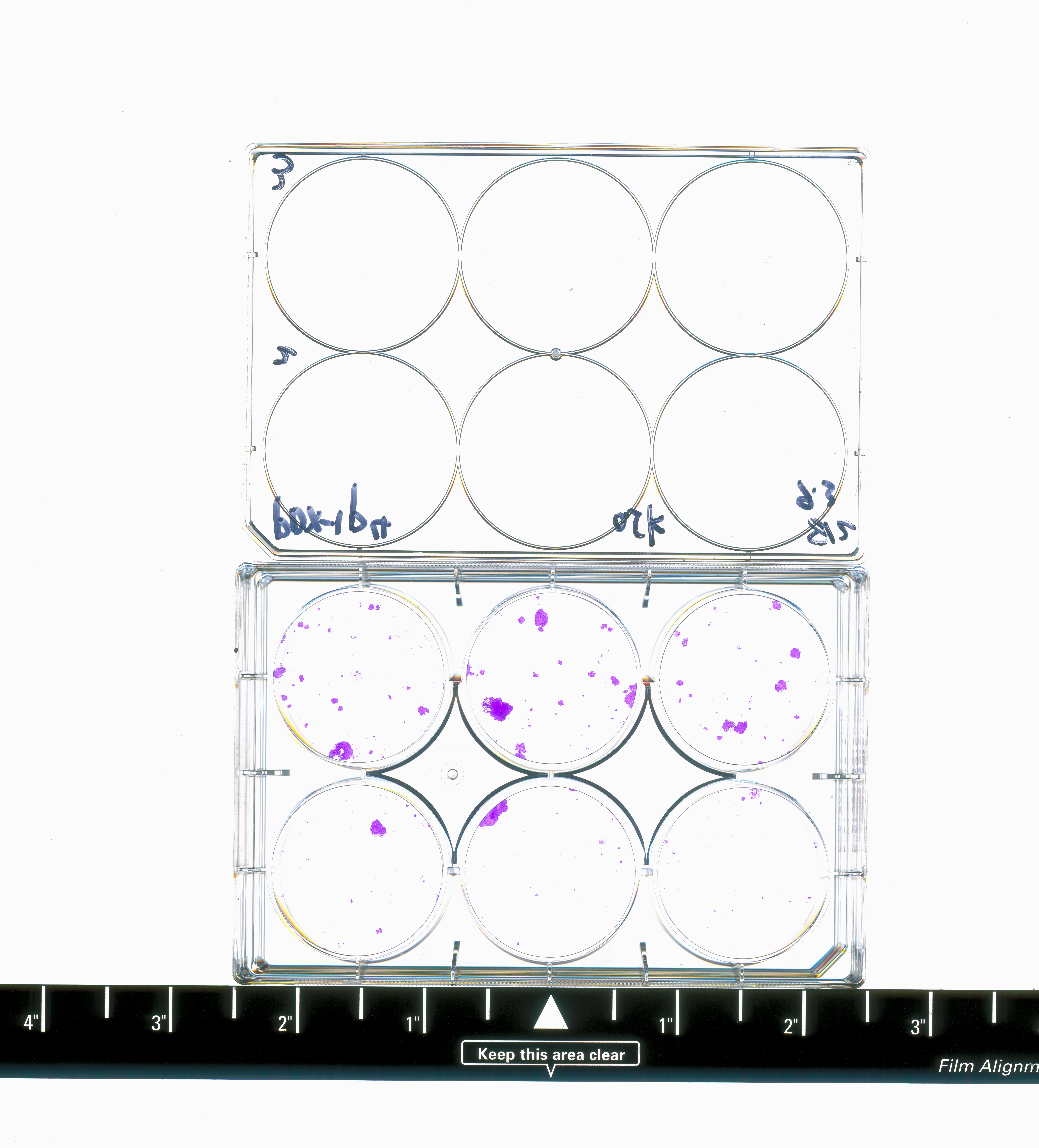

Supplement: Supplementary file 8 — Source data Fig. 3 [file 44321_2024_60_MOESM8_ESM.zip › Source data-Figure 3 (44321_2024_60_MOESM8_ESM)_updated/Figure 3/3C/CN1/21.09.06 PD1 sg2-sg3.jpg]

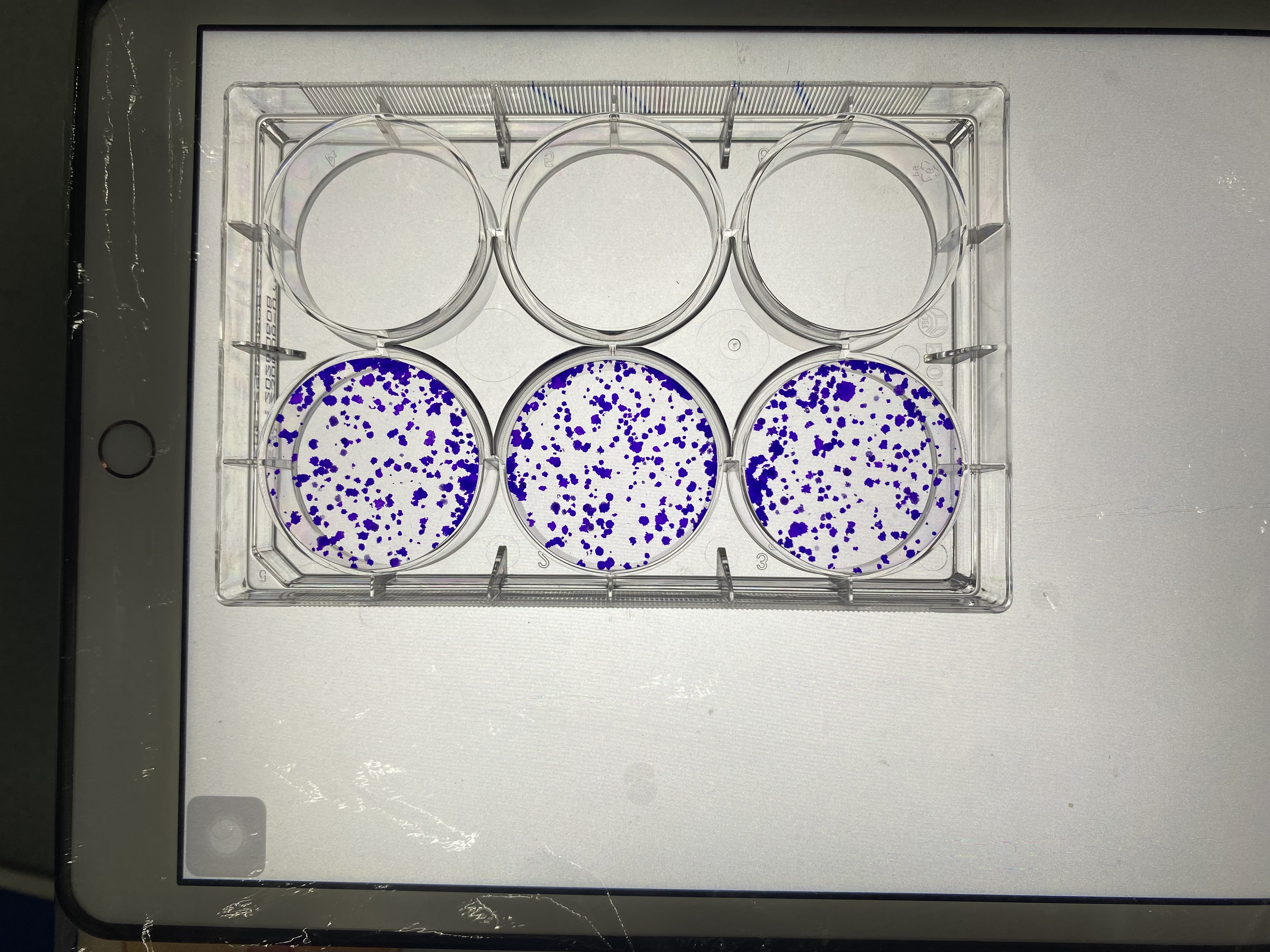

Supplement: Supplementary file 8 — Source data Fig. 3 [file 44321_2024_60_MOESM8_ESM.zip › Source data-Figure 3 (44321_2024_60_MOESM8_ESM)_updated/Figure 3/3C/YAPC/EV.jpg]

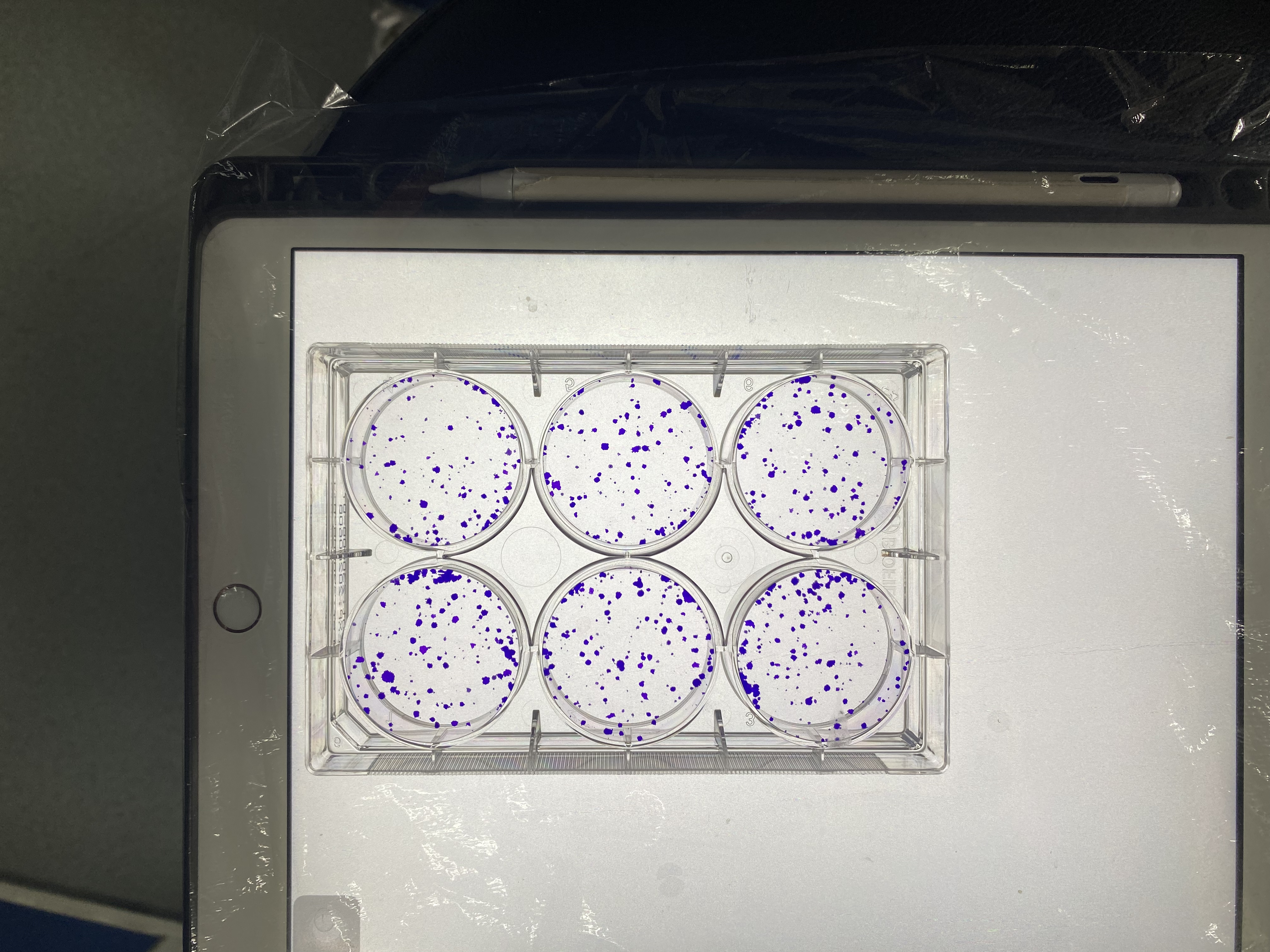

Supplement: Supplementary file 8 — Source data Fig. 3 [file 44321_2024_60_MOESM8_ESM.zip › Source data-Figure 3 (44321_2024_60_MOESM8_ESM)_updated/Figure 3/3C/YAPC/sg.jpg]

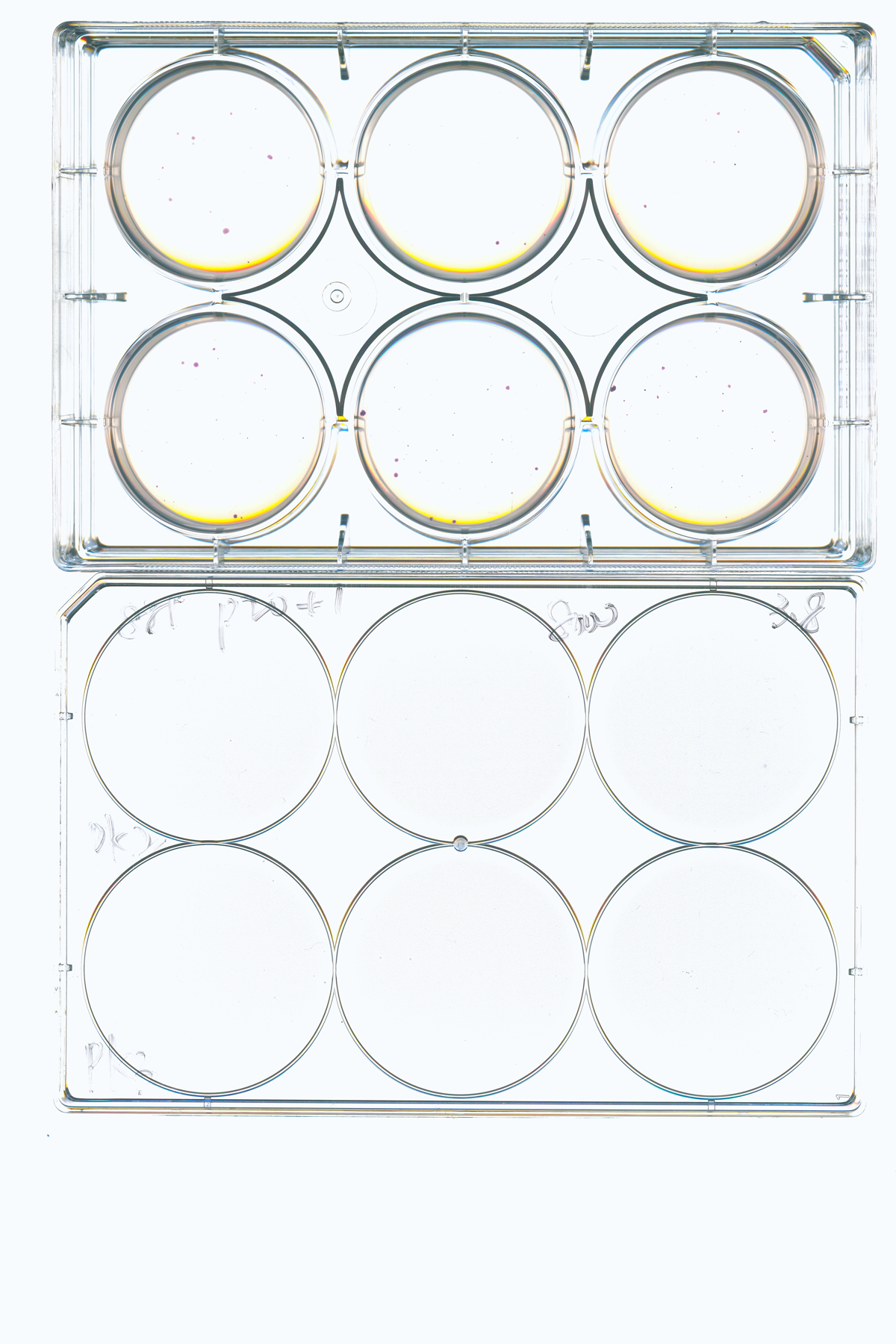

Supplement: Supplementary file 8 — Source data Fig. 3 [file 44321_2024_60_MOESM8_ESM.zip › Source data-Figure 3 (44321_2024_60_MOESM8_ESM)_updated/Figure 3/3D/88T/88T 8000 PK-2_PK-3.tif]

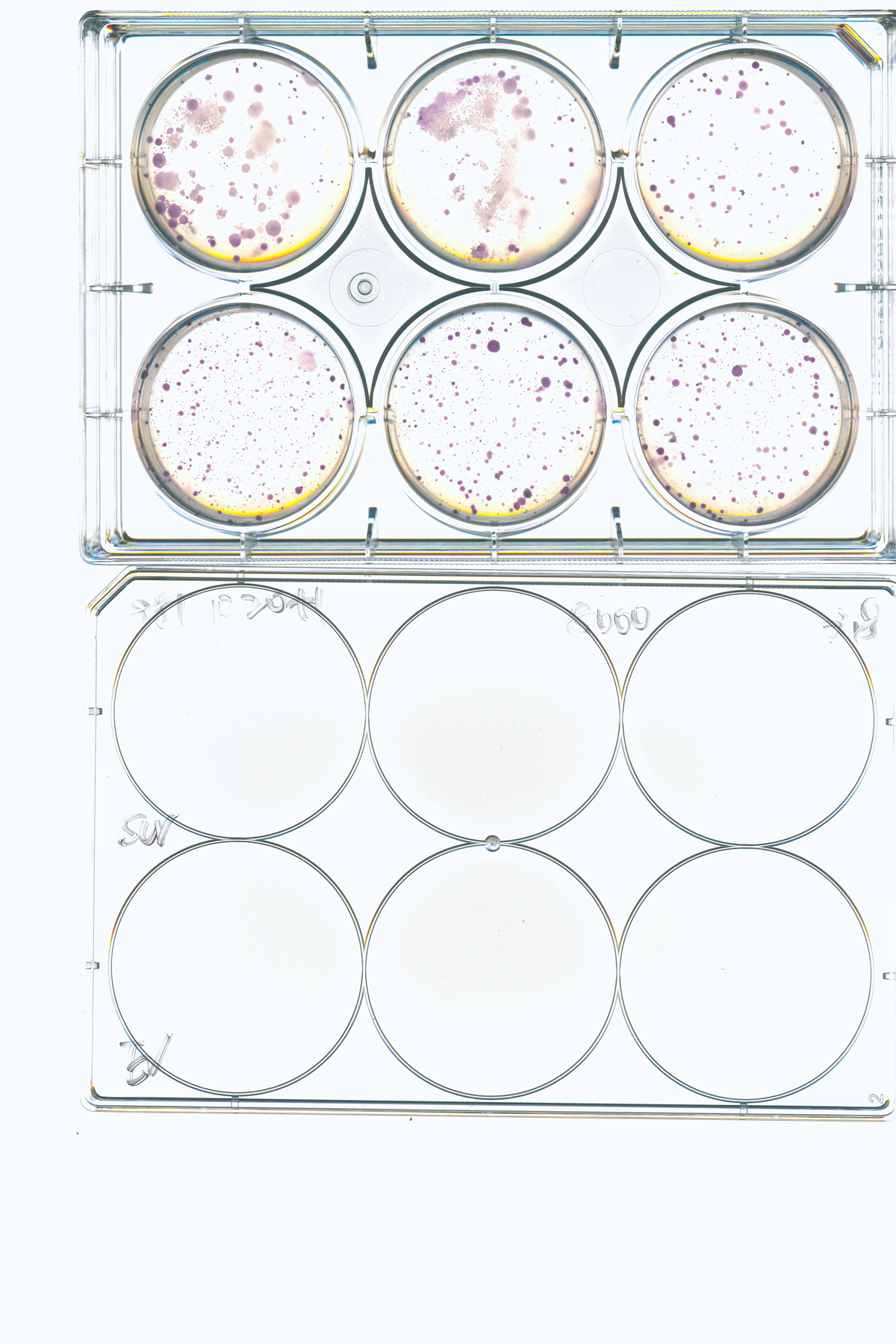

Supplement: Supplementary file 8 — Source data Fig. 3 [file 44321_2024_60_MOESM8_ESM.zip › Source data-Figure 3 (44321_2024_60_MOESM8_ESM)_updated/Figure 3/3D/88T/88T 8000 SC_EV.tif]

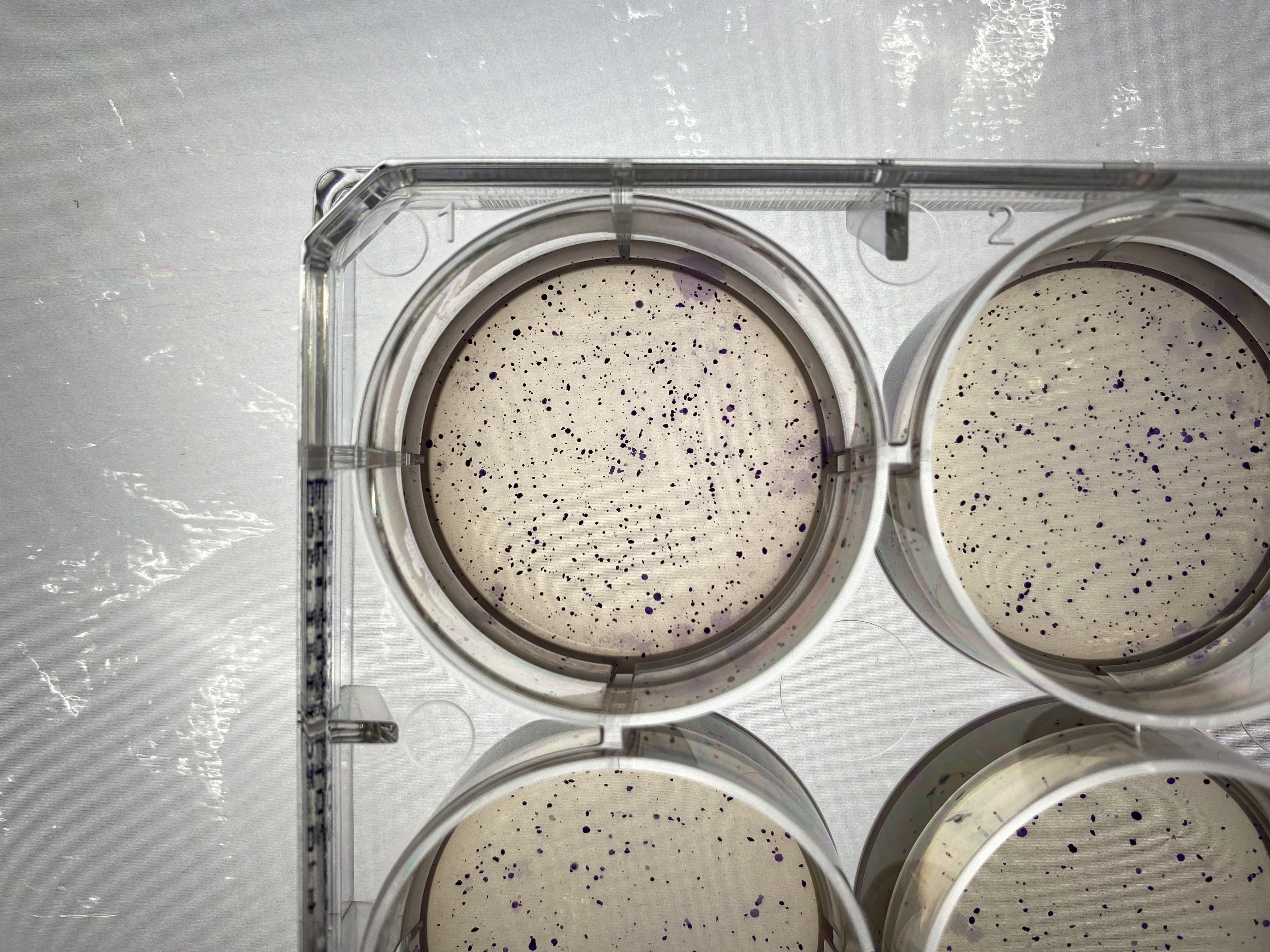

Supplement: Supplementary file 8 — Source data Fig. 3 [file 44321_2024_60_MOESM8_ESM.zip › Source data-Figure 3 (44321_2024_60_MOESM8_ESM)_updated/Figure 3/3D/YAPC/25K EV-1.jpg]

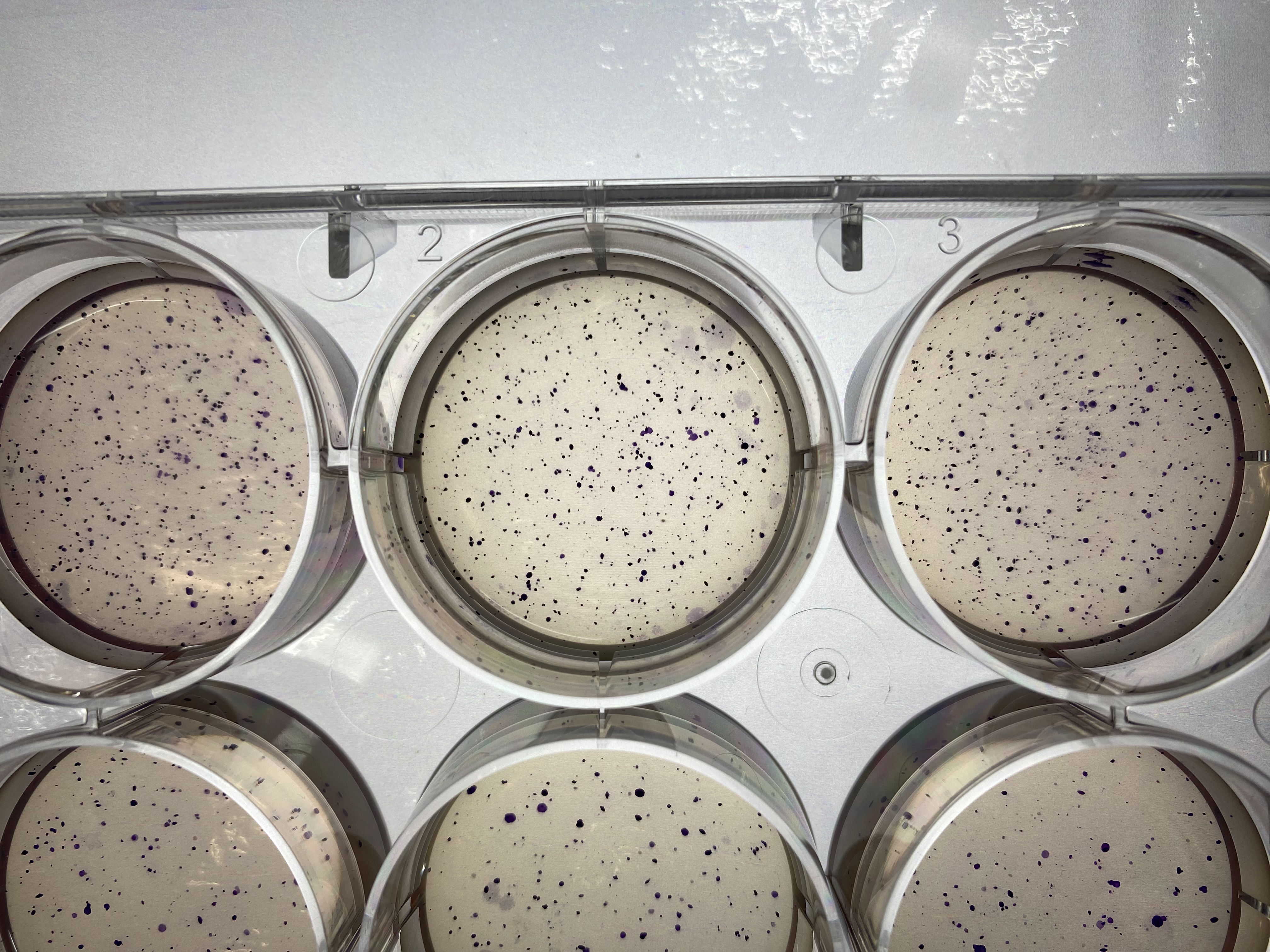

Supplement: Supplementary file 8 — Source data Fig. 3 [file 44321_2024_60_MOESM8_ESM.zip › Source data-Figure 3 (44321_2024_60_MOESM8_ESM)_updated/Figure 3/3D/YAPC/25K EV-2.jpg]

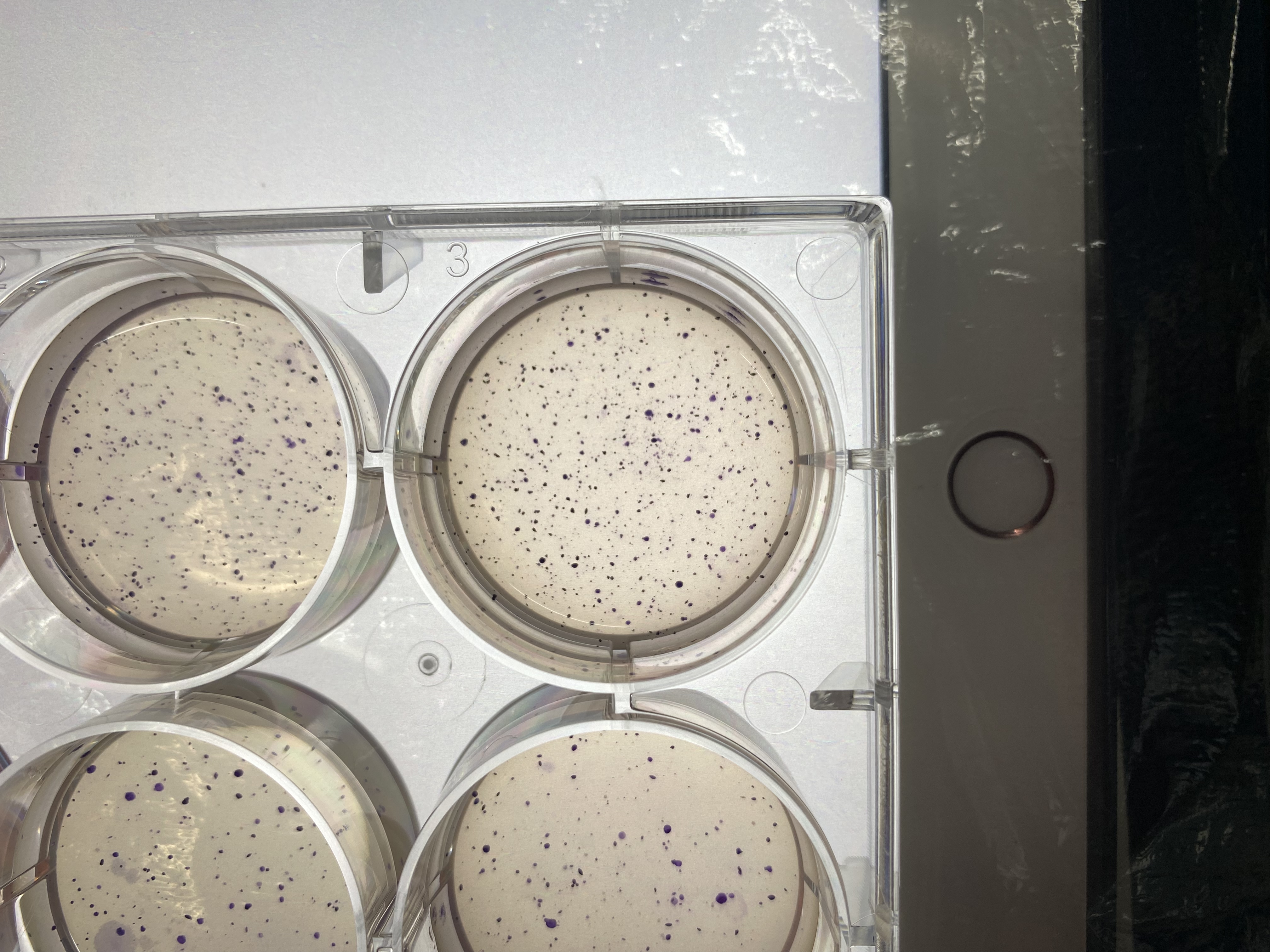

Supplement: Supplementary file 8 — Source data Fig. 3 [file 44321_2024_60_MOESM8_ESM.zip › Source data-Figure 3 (44321_2024_60_MOESM8_ESM)_updated/Figure 3/3D/YAPC/25K EV-3.jpg]

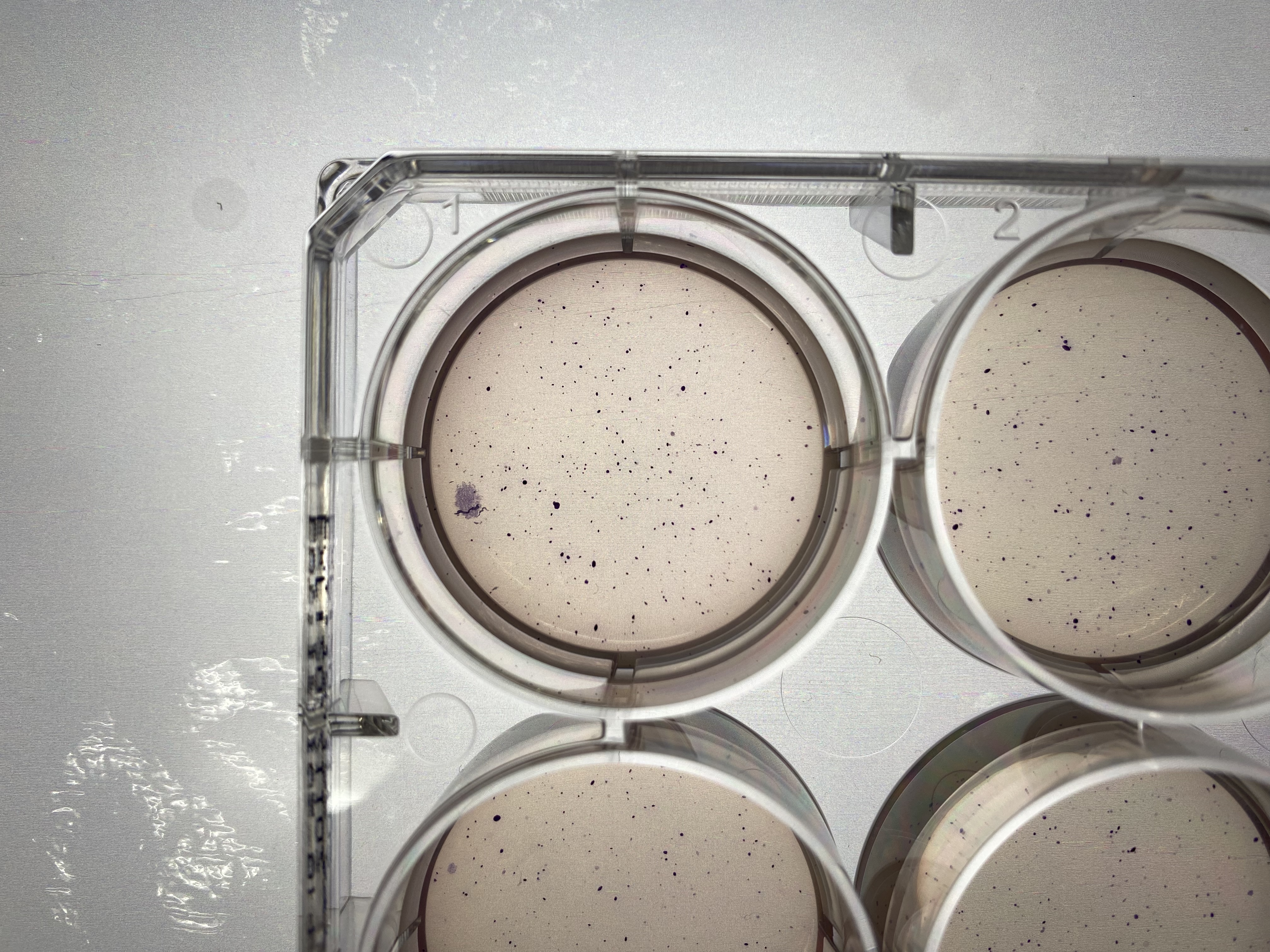

Supplement: Supplementary file 8 — Source data Fig. 3 [file 44321_2024_60_MOESM8_ESM.zip › Source data-Figure 3 (44321_2024_60_MOESM8_ESM)_updated/Figure 3/3D/YAPC/25K sg2-1.jpg]

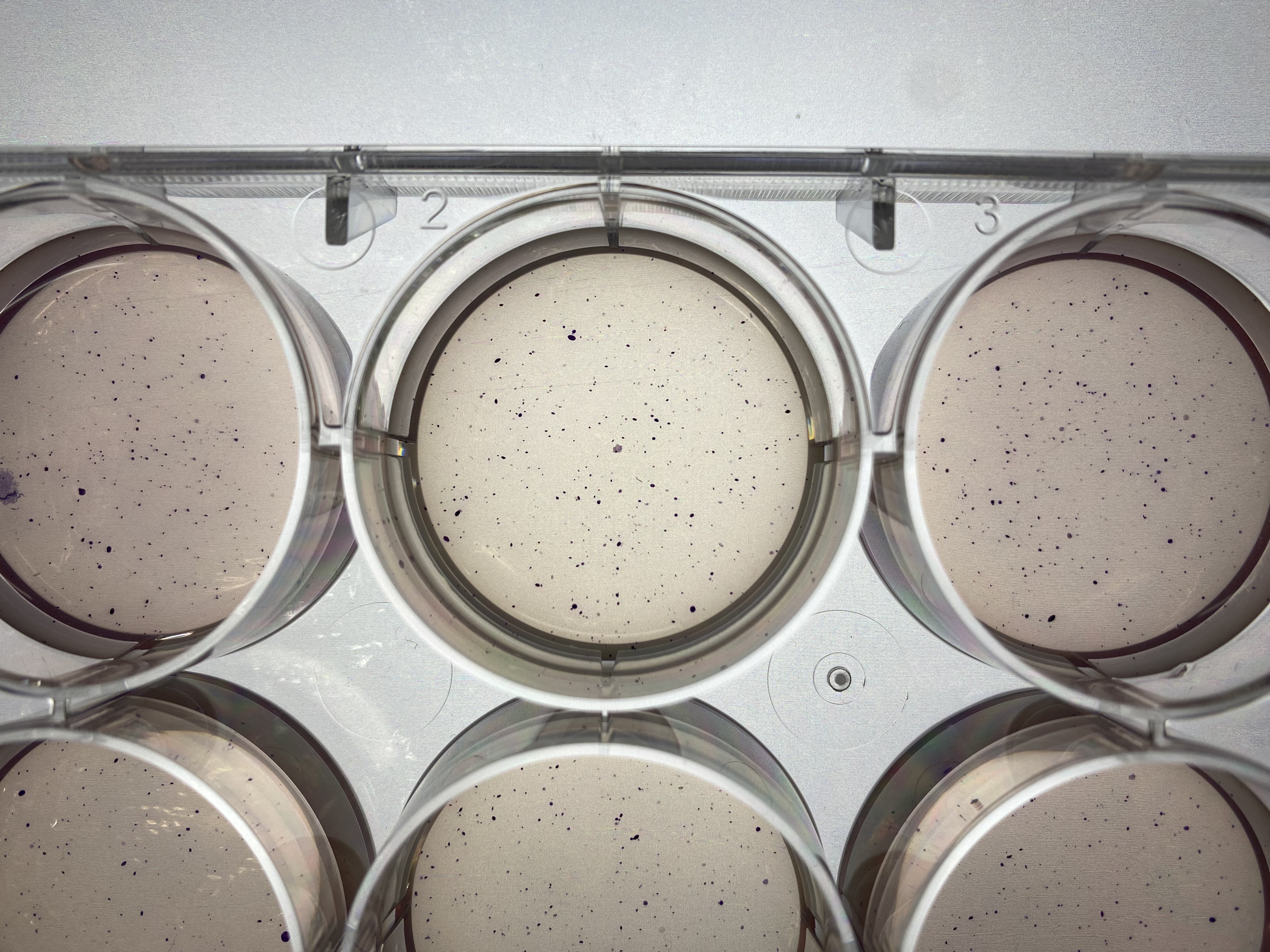

Supplement: Supplementary file 8 — Source data Fig. 3 [file 44321_2024_60_MOESM8_ESM.zip › Source data-Figure 3 (44321_2024_60_MOESM8_ESM)_updated/Figure 3/3D/YAPC/25K sg2-2.jpg]

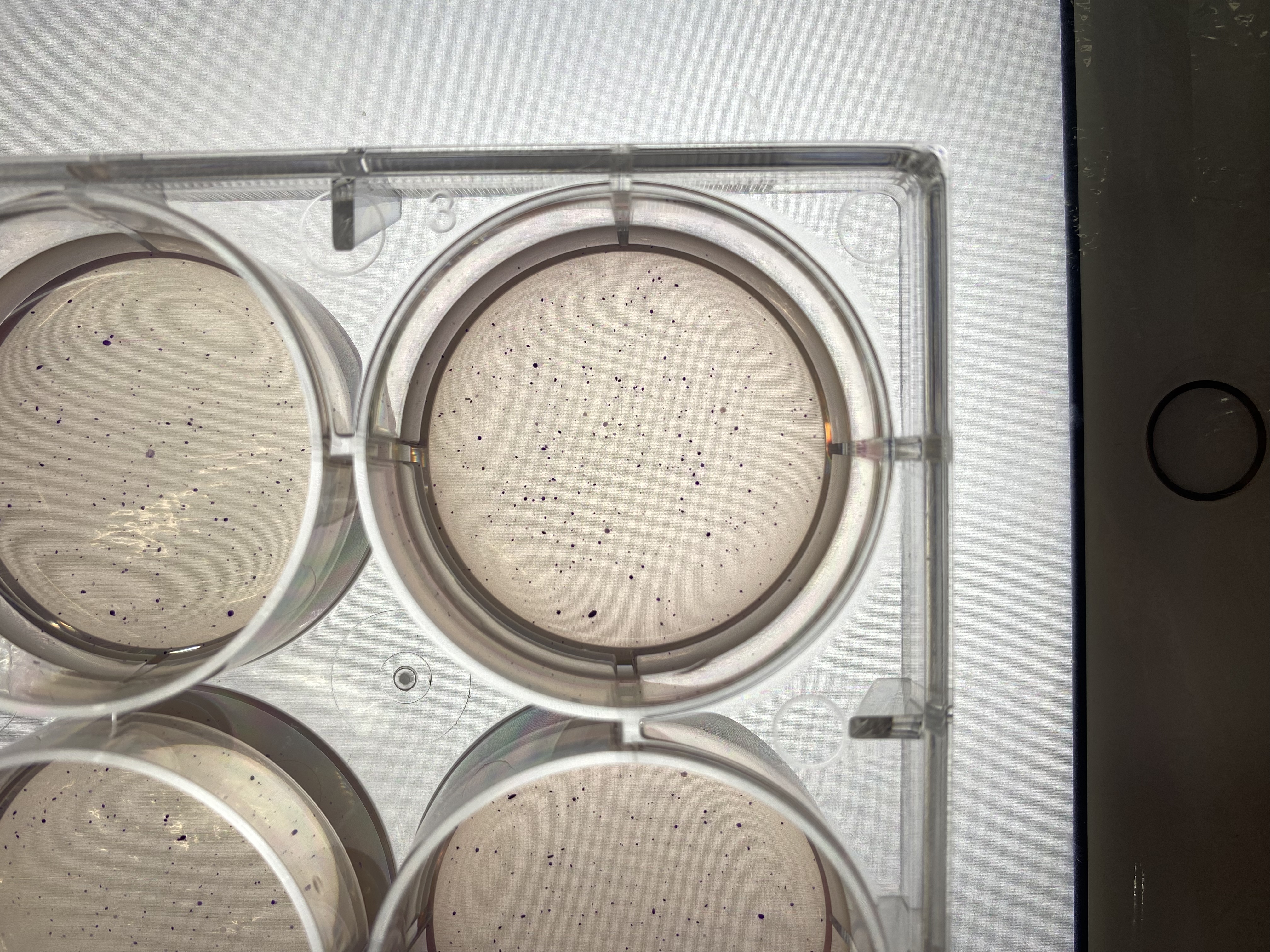

Supplement: Supplementary file 8 — Source data Fig. 3 [file 44321_2024_60_MOESM8_ESM.zip › Source data-Figure 3 (44321_2024_60_MOESM8_ESM)_updated/Figure 3/3D/YAPC/25K sg2-3.jpg]

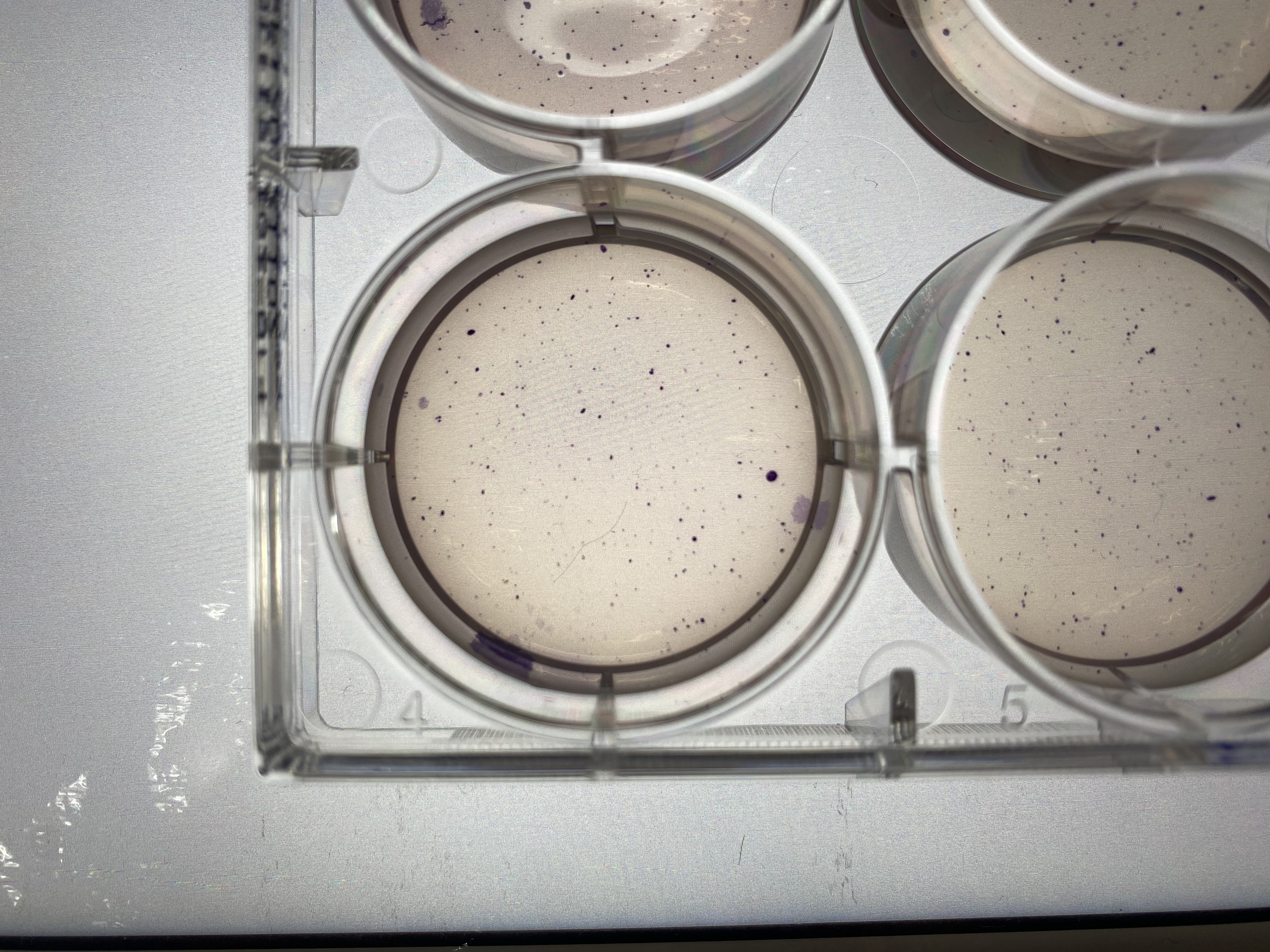

Supplement: Supplementary file 8 — Source data Fig. 3 [file 44321_2024_60_MOESM8_ESM.zip › Source data-Figure 3 (44321_2024_60_MOESM8_ESM)_updated/Figure 3/3D/YAPC/25K sg3-1.jpg]

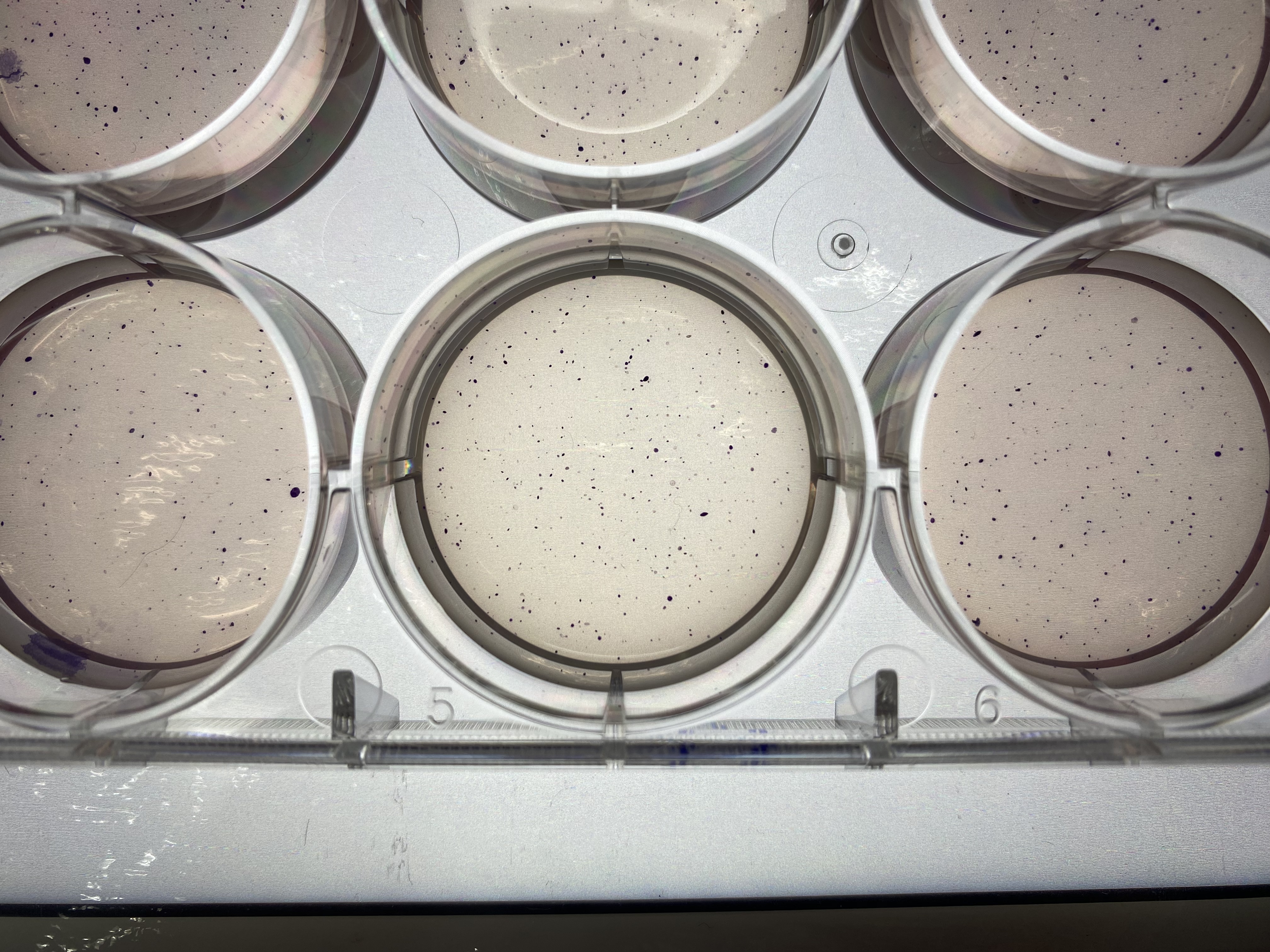

Supplement: Supplementary file 8 — Source data Fig. 3 [file 44321_2024_60_MOESM8_ESM.zip › Source data-Figure 3 (44321_2024_60_MOESM8_ESM)_updated/Figure 3/3D/YAPC/25K sg3-2.jpg]

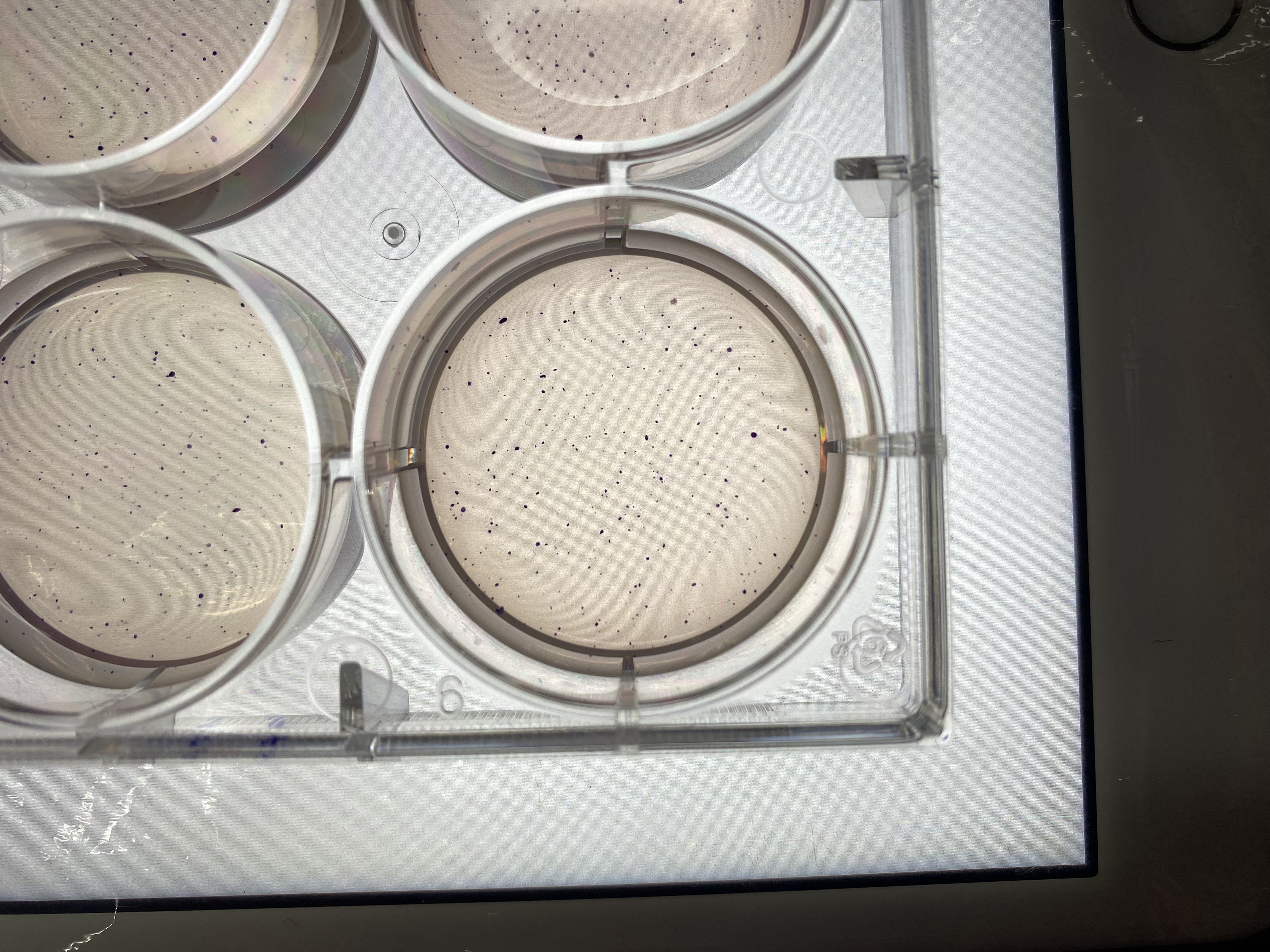

Supplement: Supplementary file 8 — Source data Fig. 3 [file 44321_2024_60_MOESM8_ESM.zip › Source data-Figure 3 (44321_2024_60_MOESM8_ESM)_updated/Figure 3/3D/YAPC/25K sg3-3.jpg]

## Slide 1
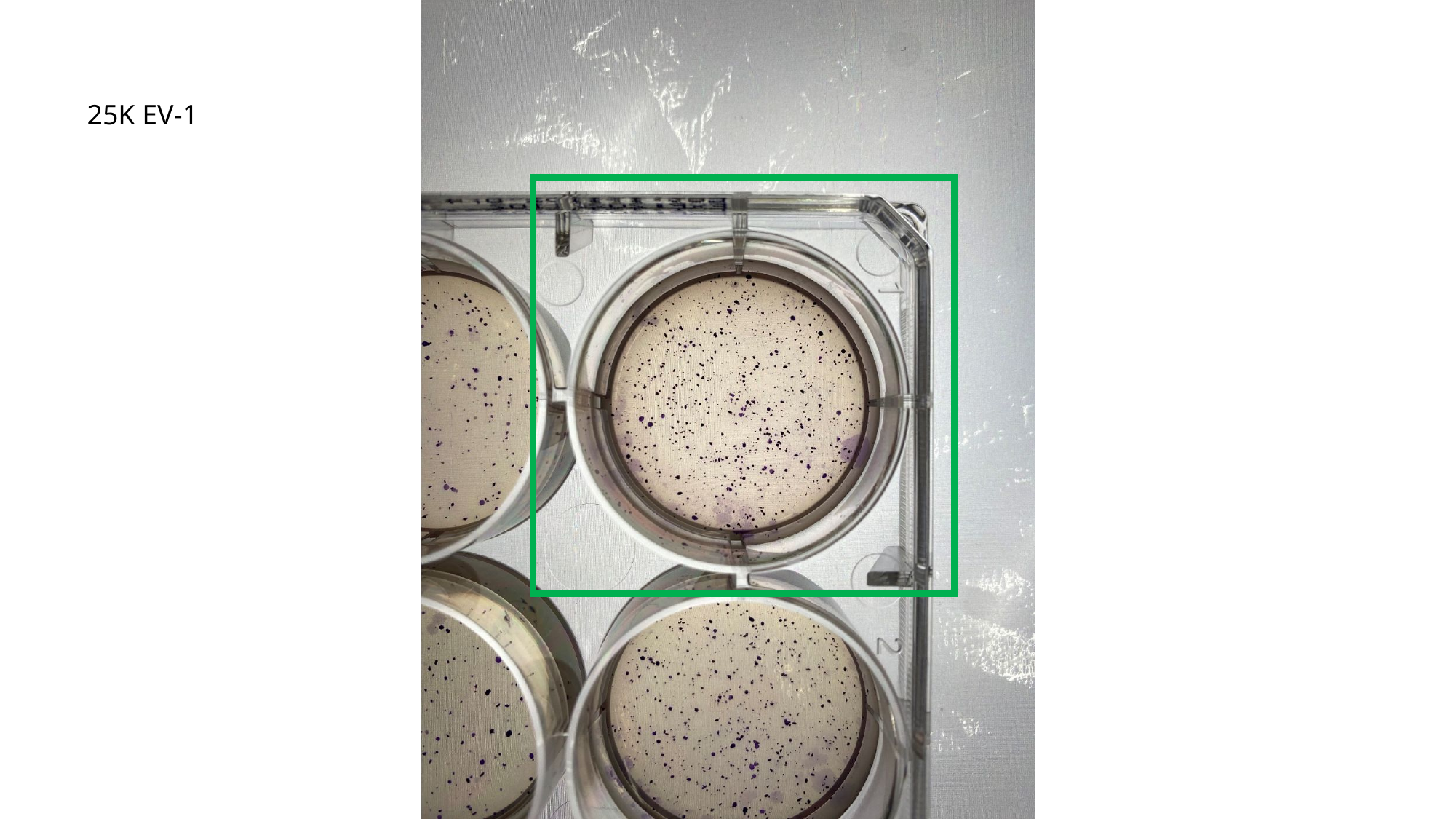

25K EV-1

## Slide 2
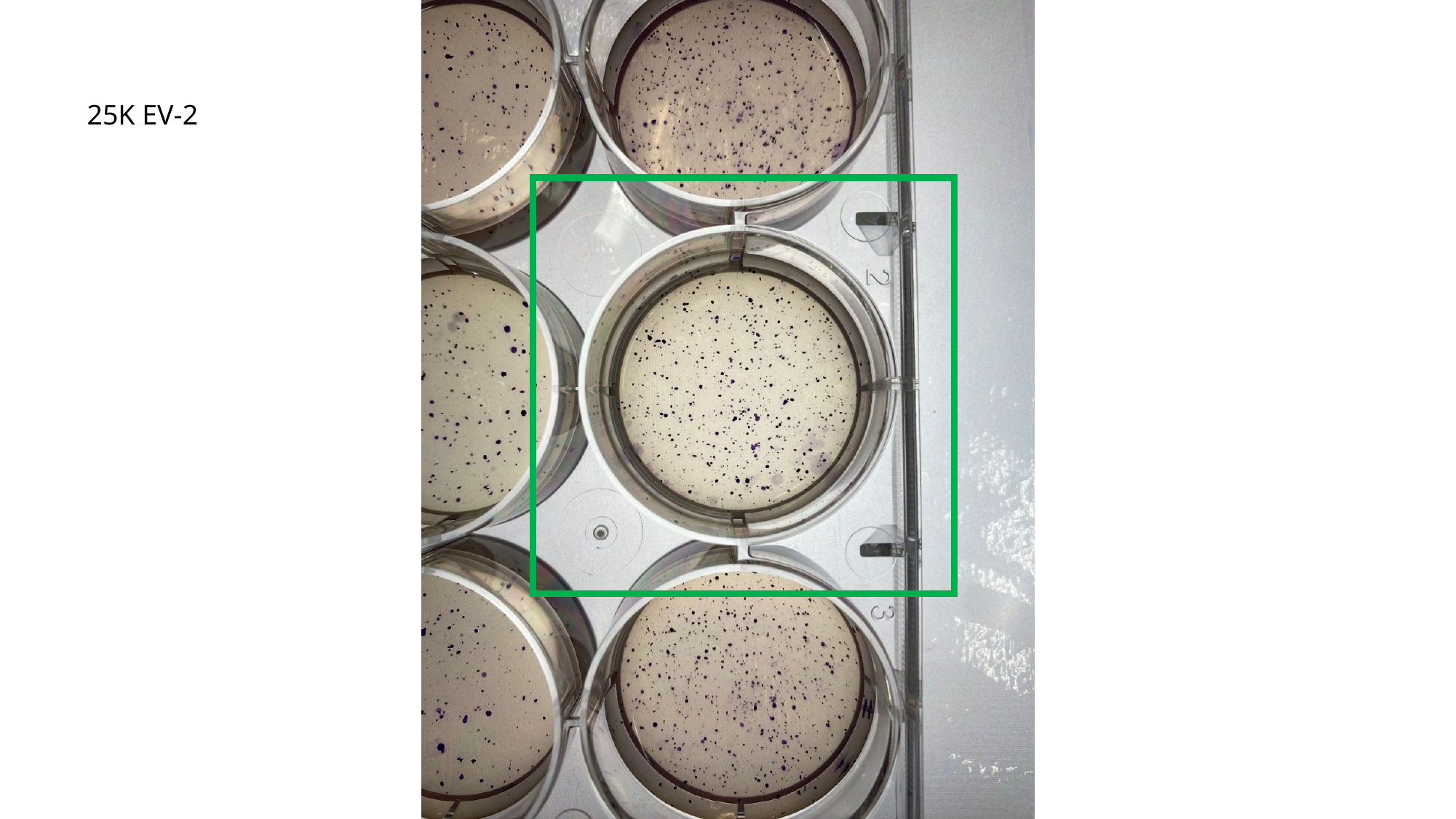

25K EV-2

## Slide 3
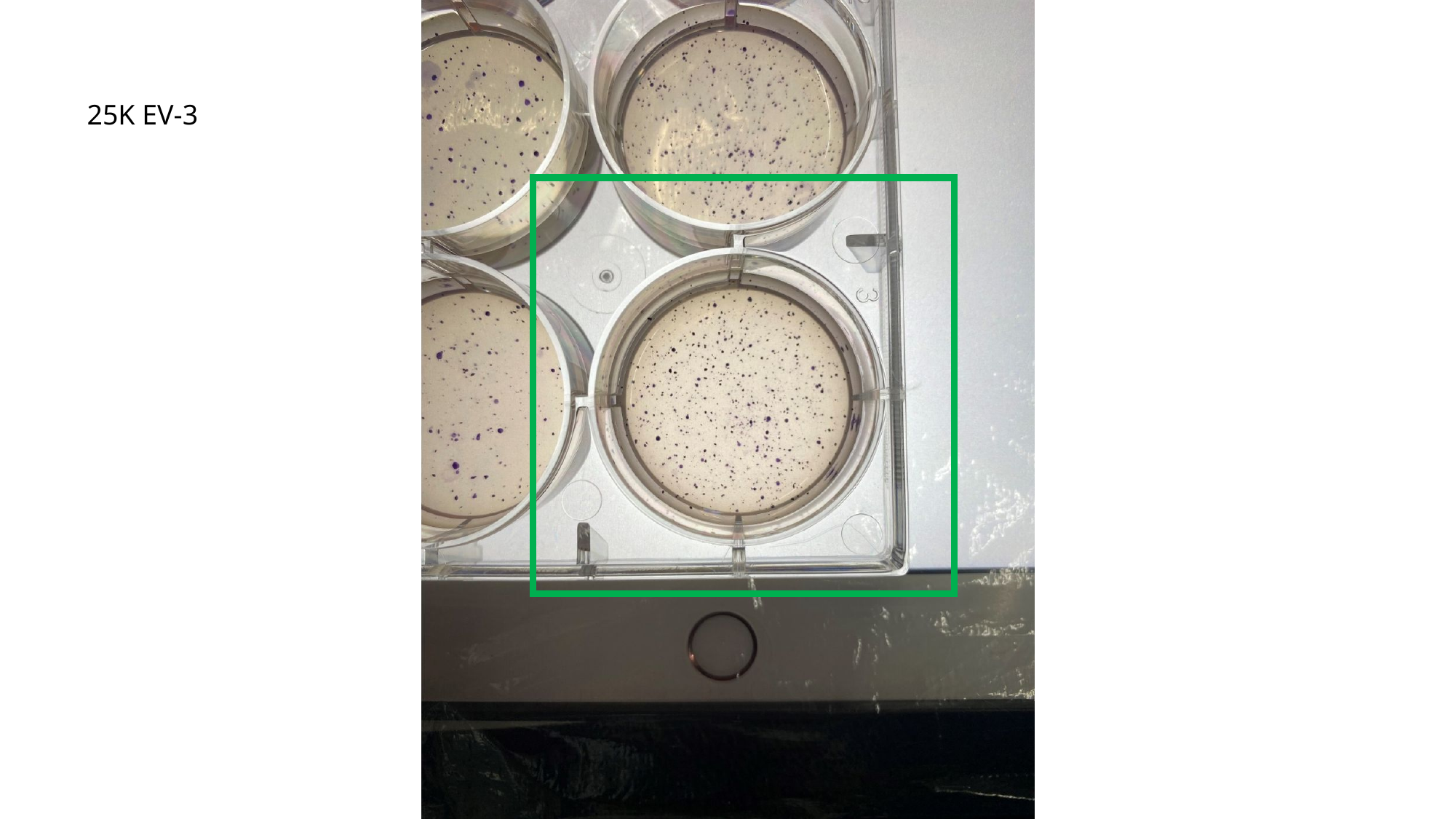

25K EV-3

## Slide 4
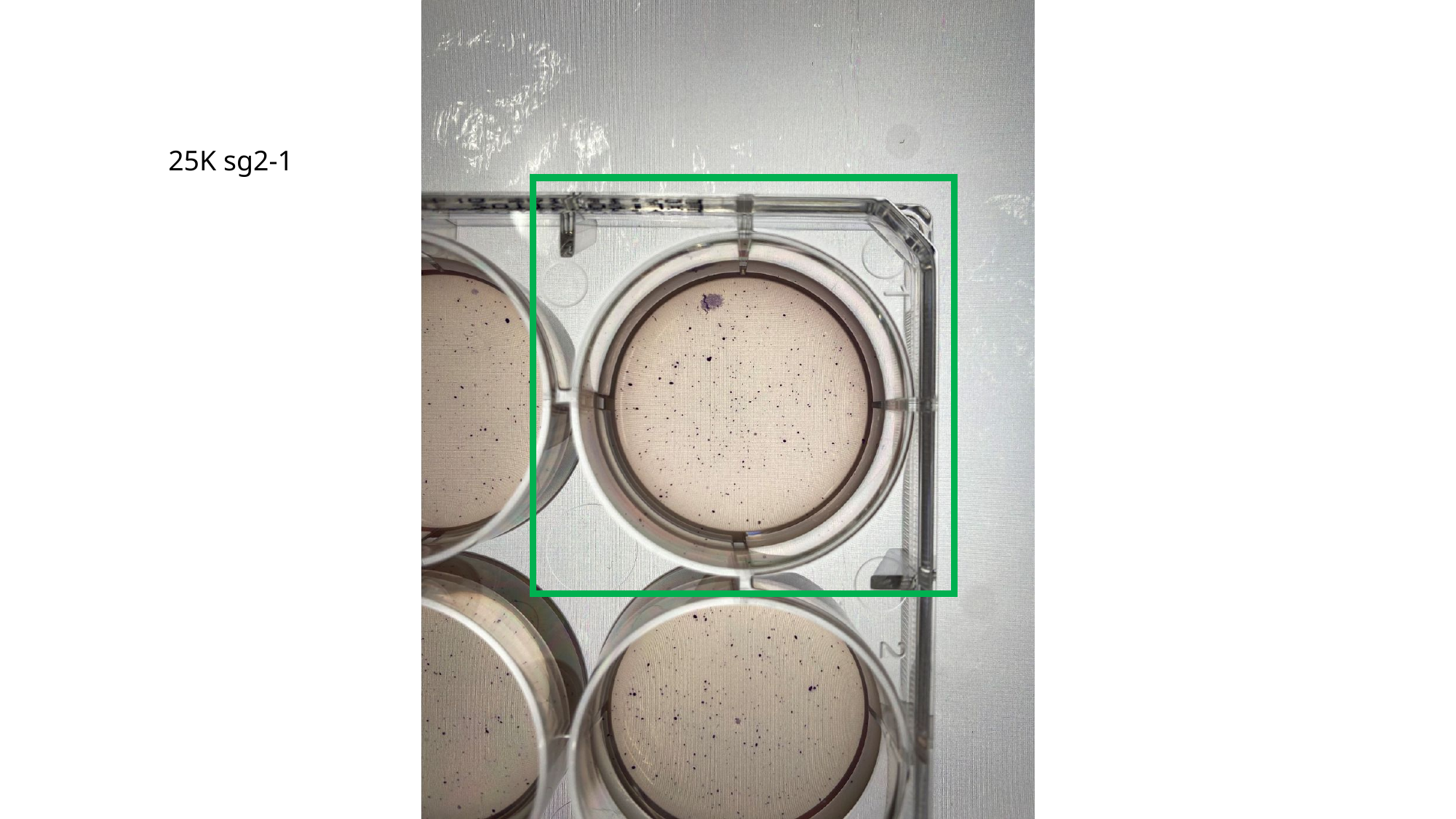

25K sg2-1

## Slide 5
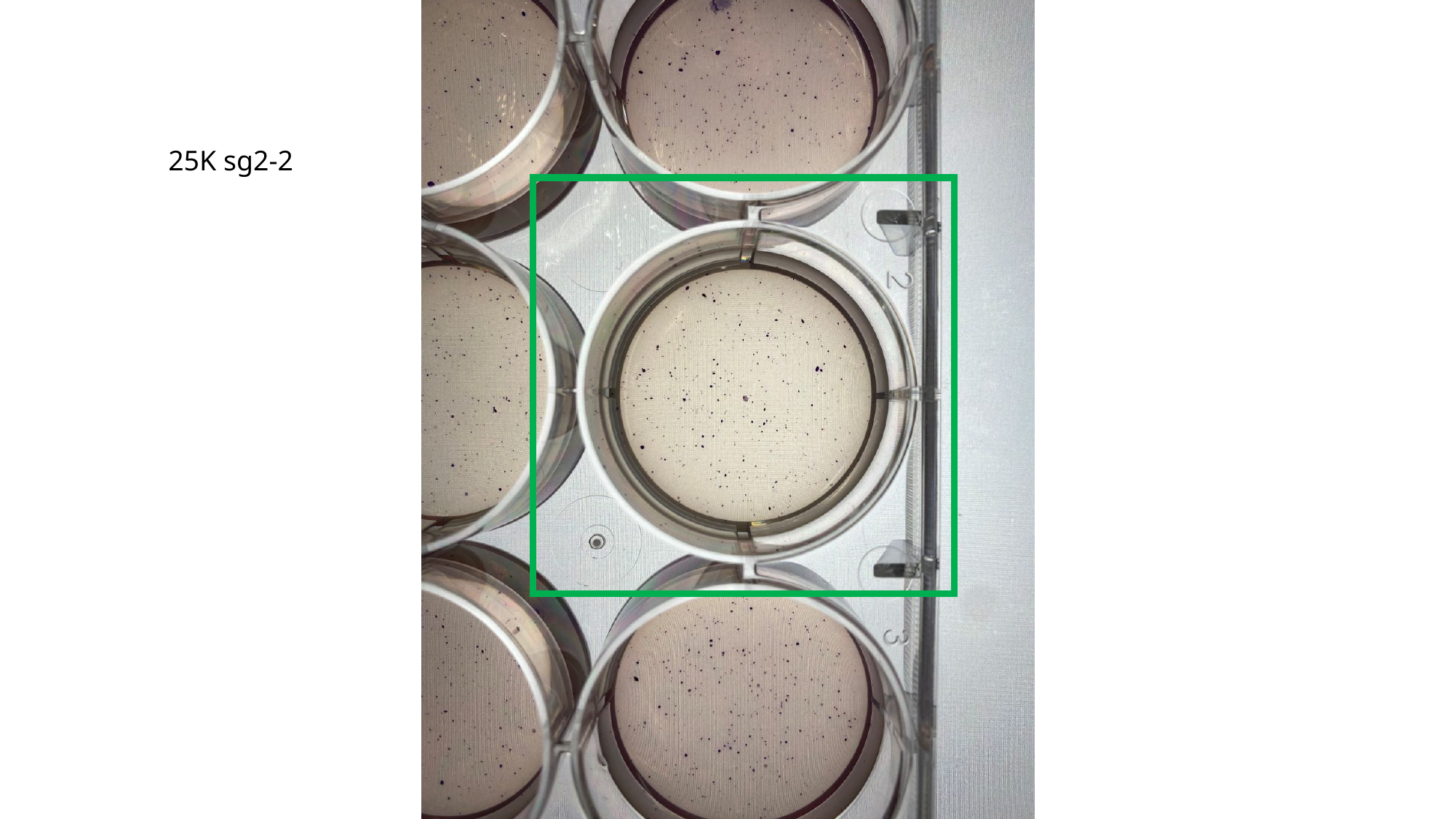

25K sg2-2

## Slide 6
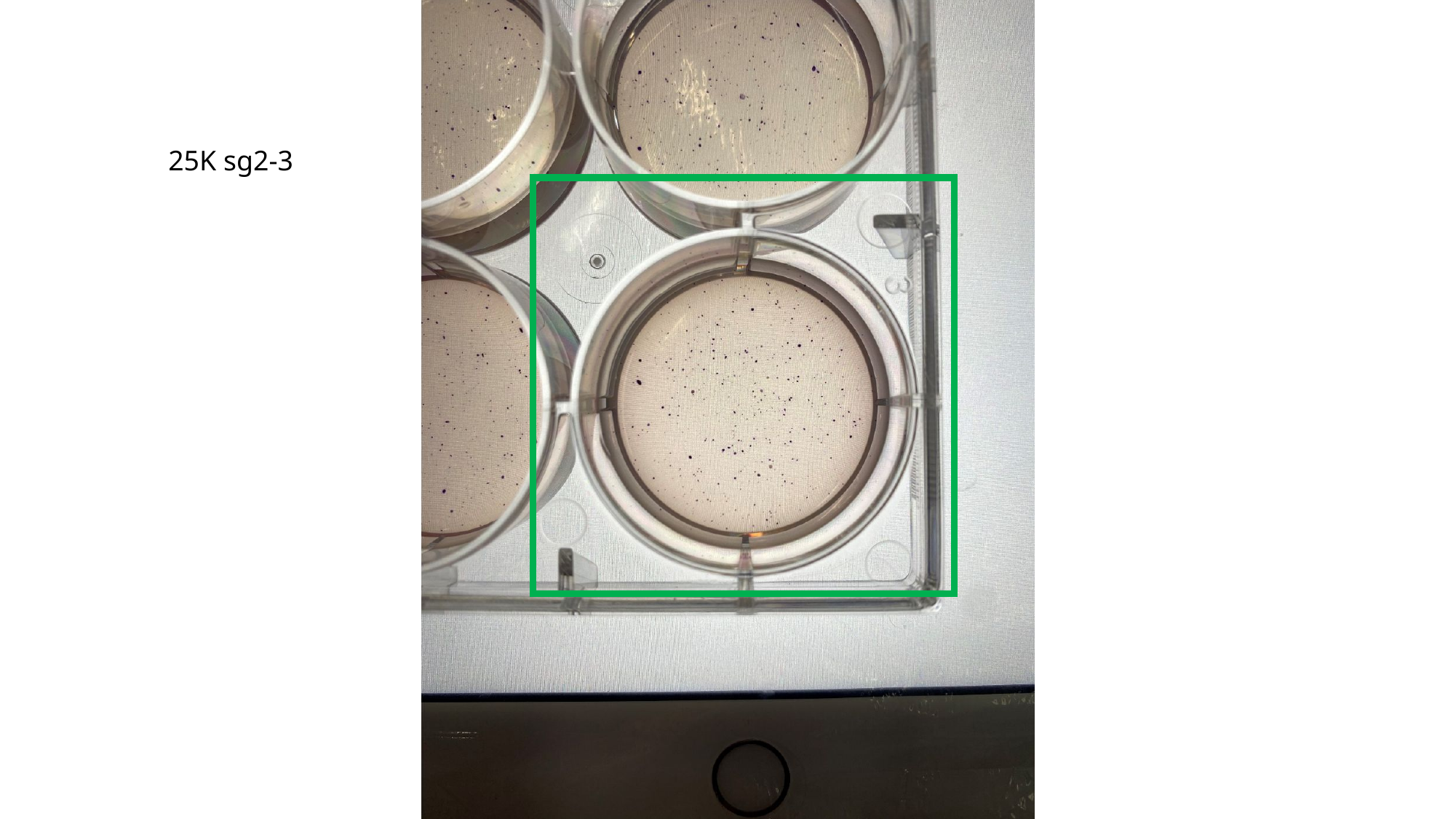

25K sg2-3

## Slide 7
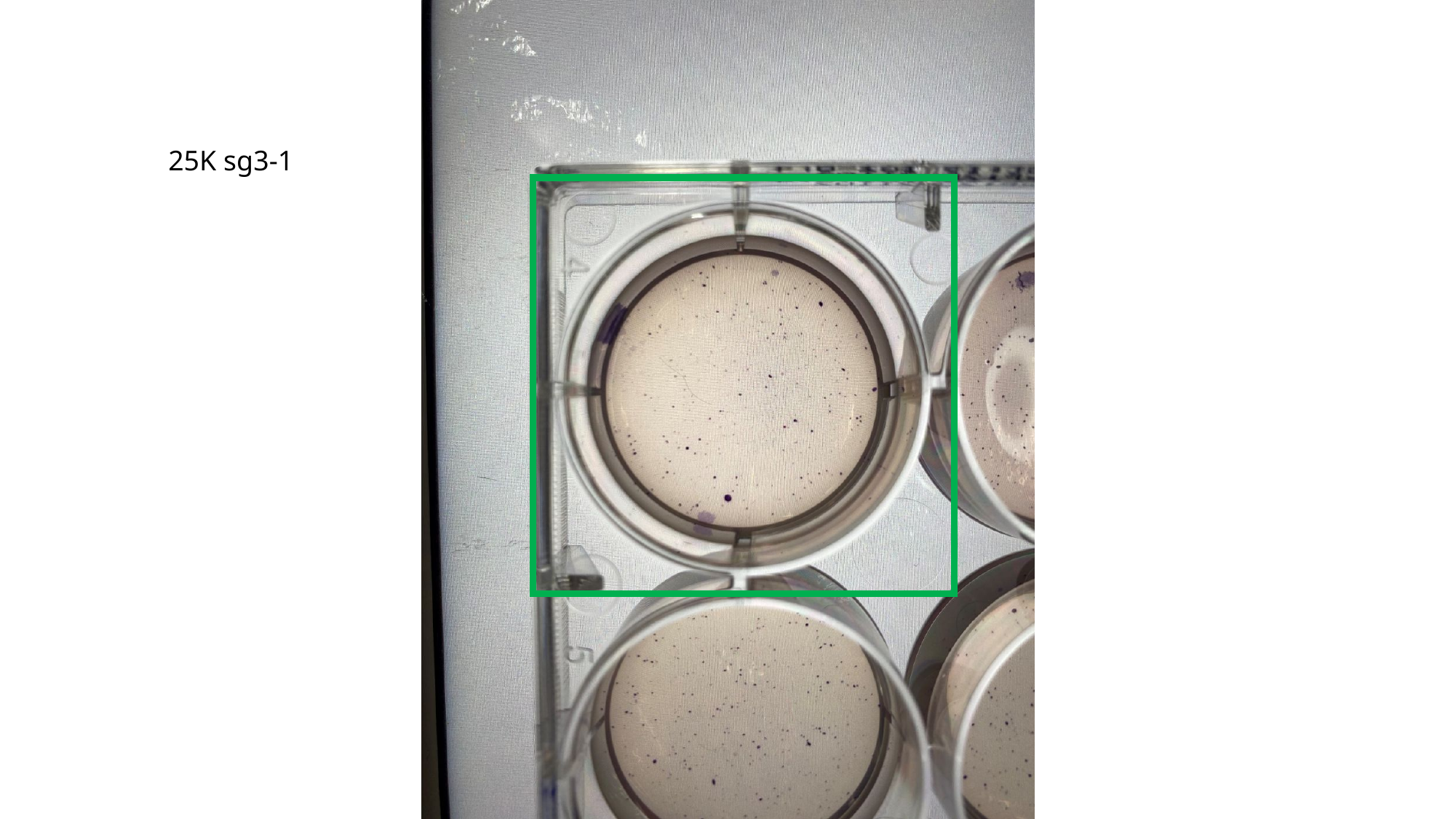

25K sg3-1

## Slide 8
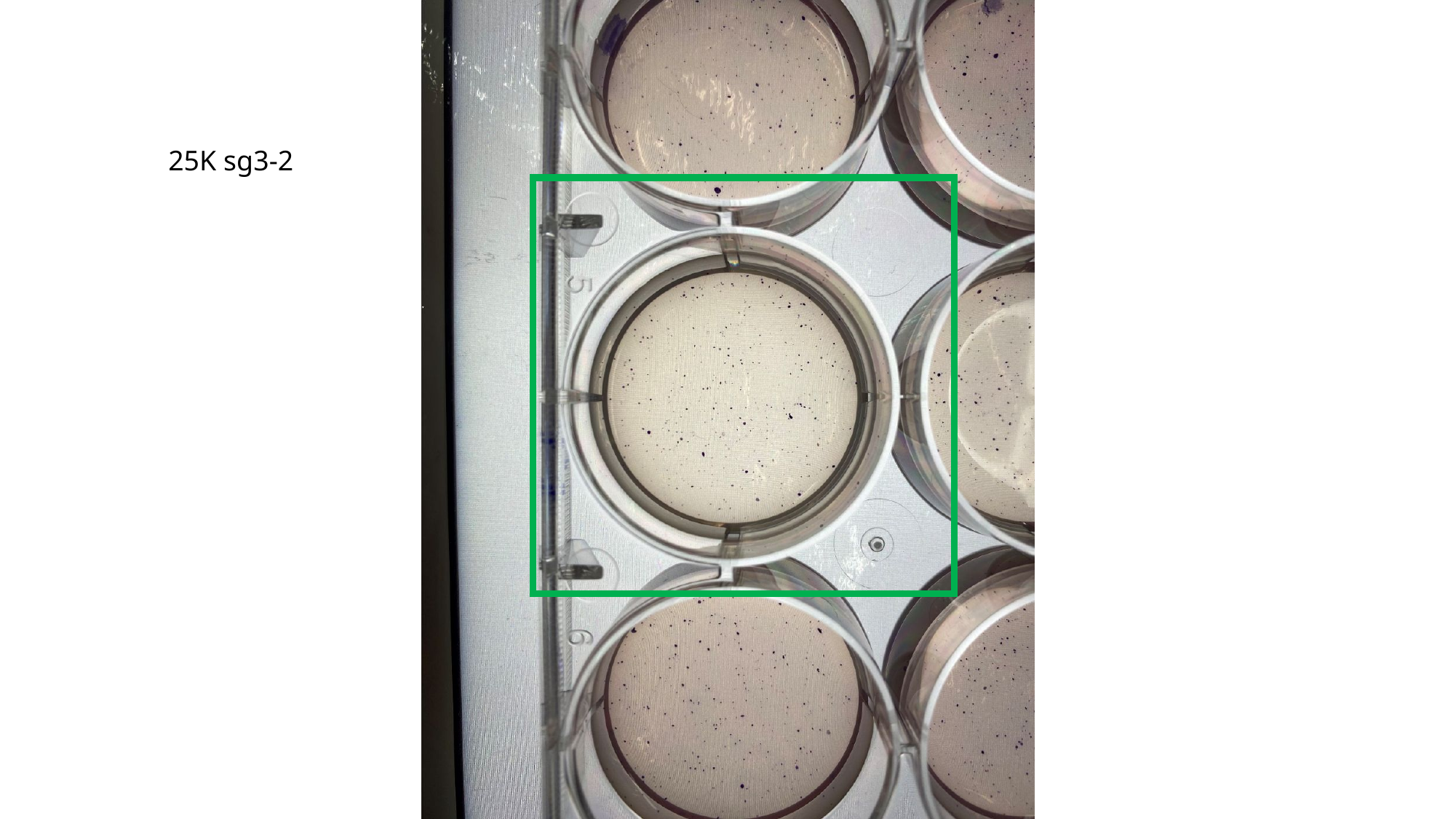

25K sg3-2

## Slide 9
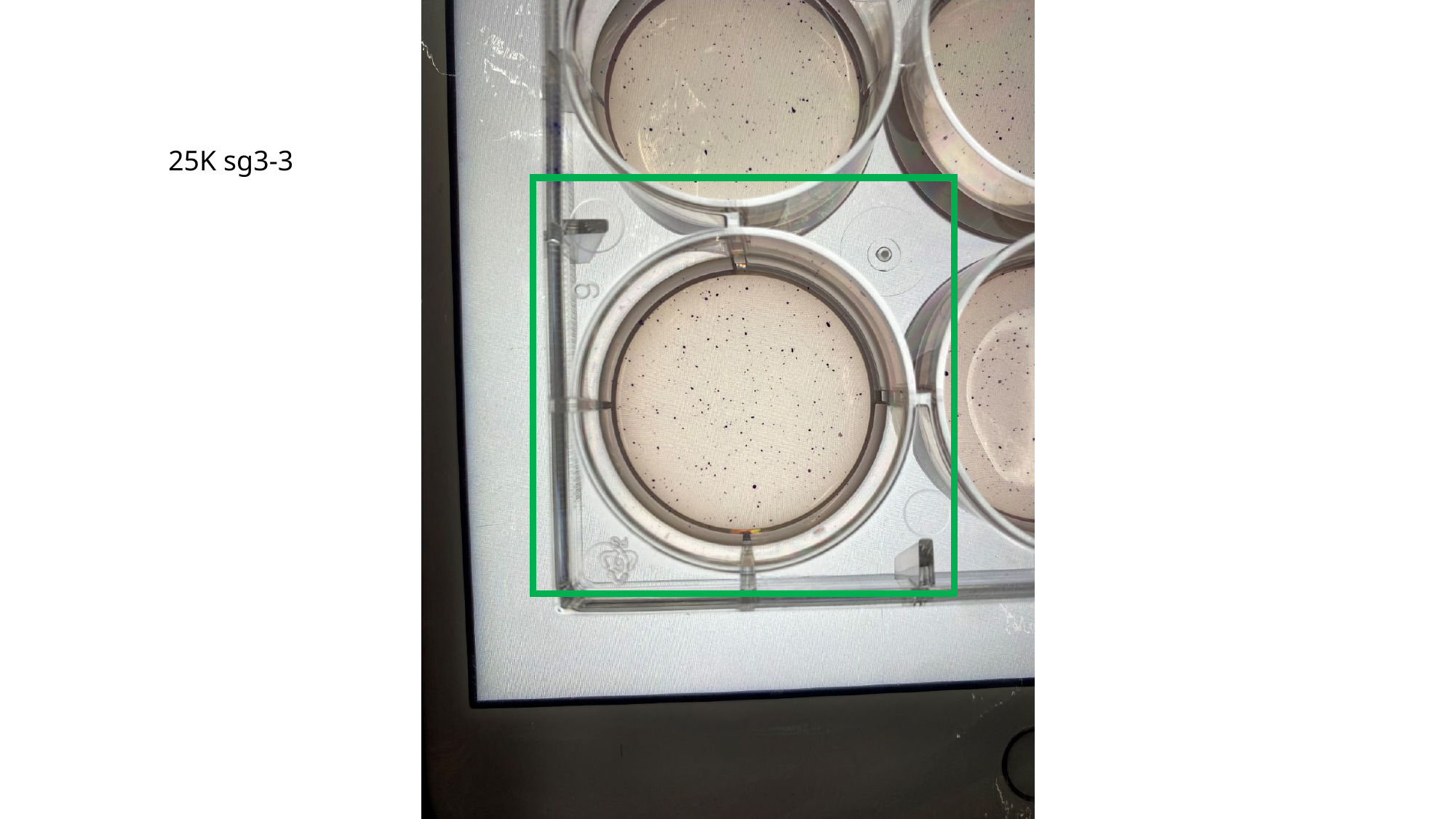

25K sg3-3

Supplement: Supplementary file 8 — Source data Fig. 3 [file 44321_2024_60_MOESM8_ESM.zip › Source data-Figure 3 (44321_2024_60_MOESM8_ESM)_updated/Figure 3/3D/YAPC/3D YAPC.pptx]

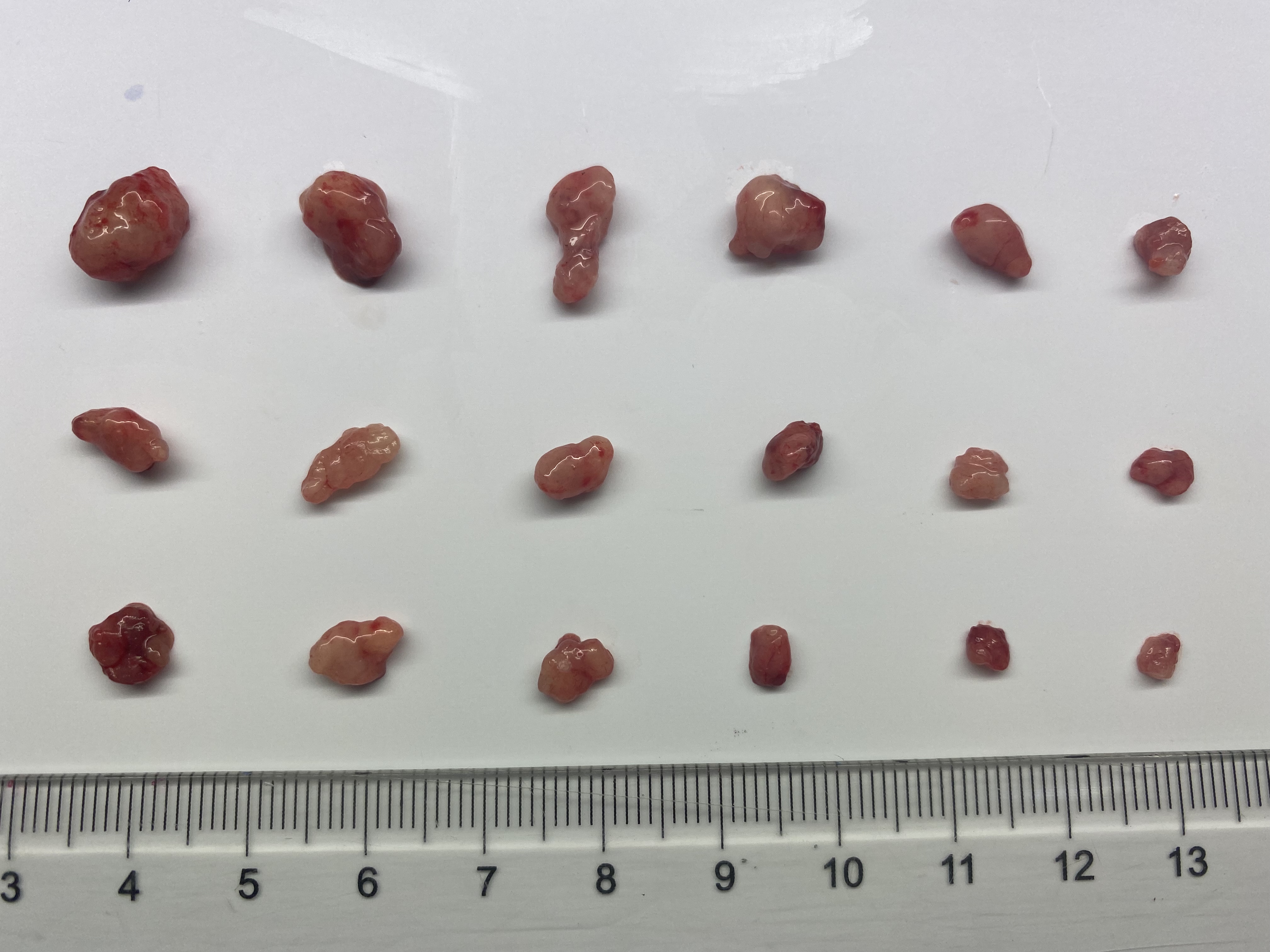

Supplement: Supplementary file 8 — Source data Fig. 3 [file 44321_2024_60_MOESM8_ESM.zip › Source data-Figure 3 (44321_2024_60_MOESM8_ESM)_updated/Figure 3/3F/88T.jpg]

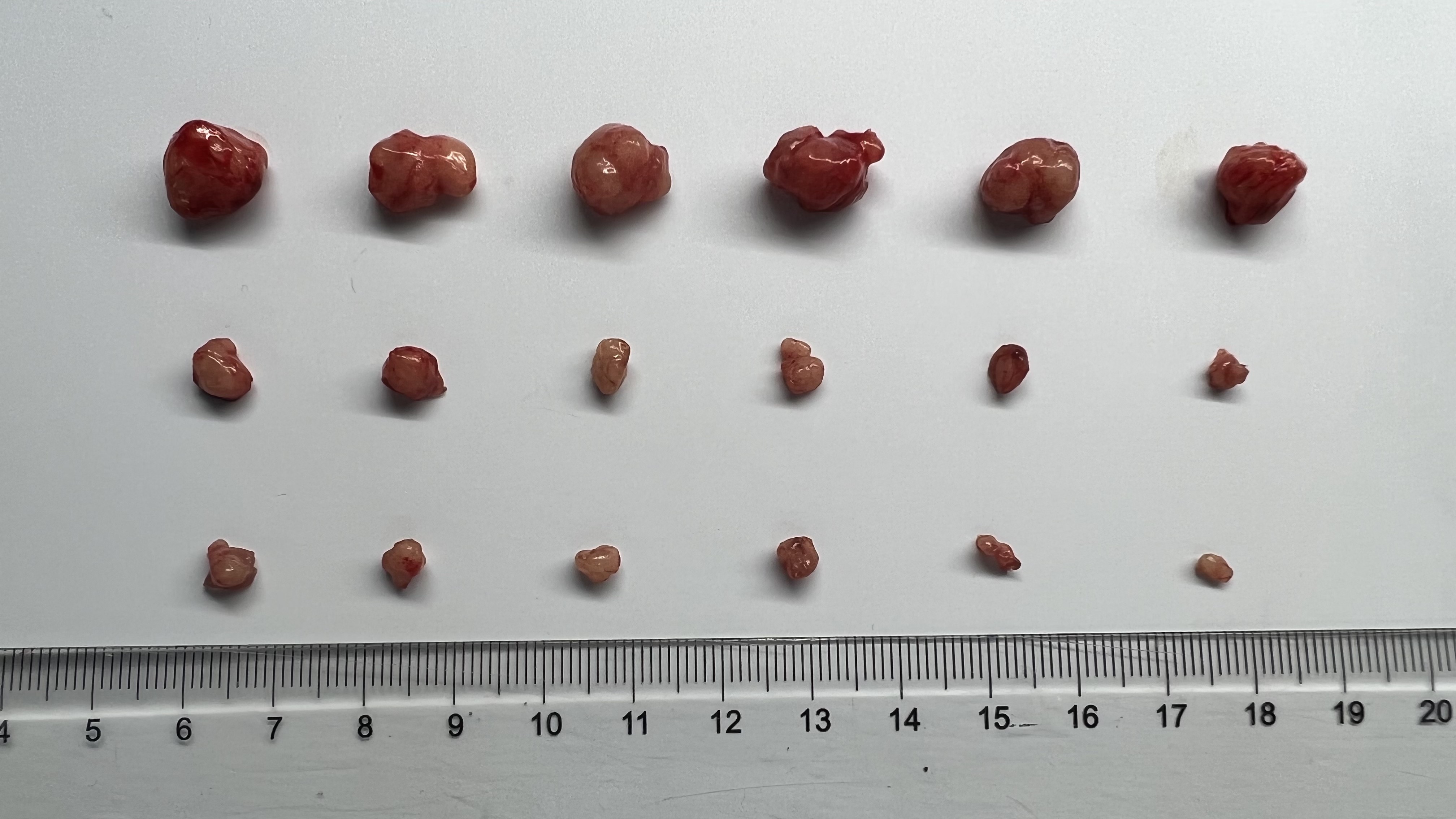

Supplement: Supplementary file 8 — Source data Fig. 3 [file 44321_2024_60_MOESM8_ESM.zip › Source data-Figure 3 (44321_2024_60_MOESM8_ESM)_updated/Figure 3/3F/CN1.jpg]

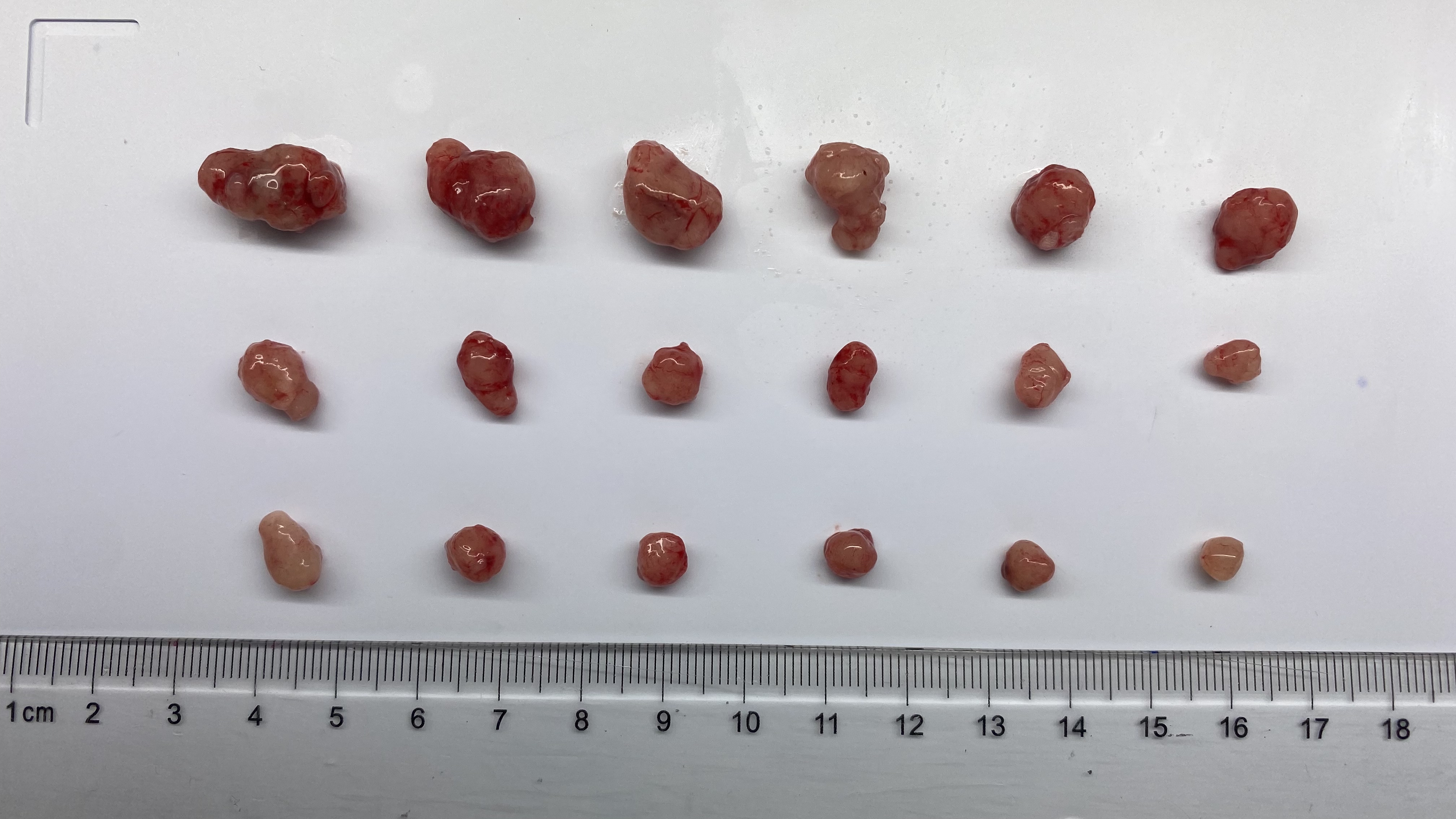

Supplement: Supplementary file 8 — Source data Fig. 3 [file 44321_2024_60_MOESM8_ESM.zip › Source data-Figure 3 (44321_2024_60_MOESM8_ESM)_updated/Figure 3/3F/YAPC.jpg]

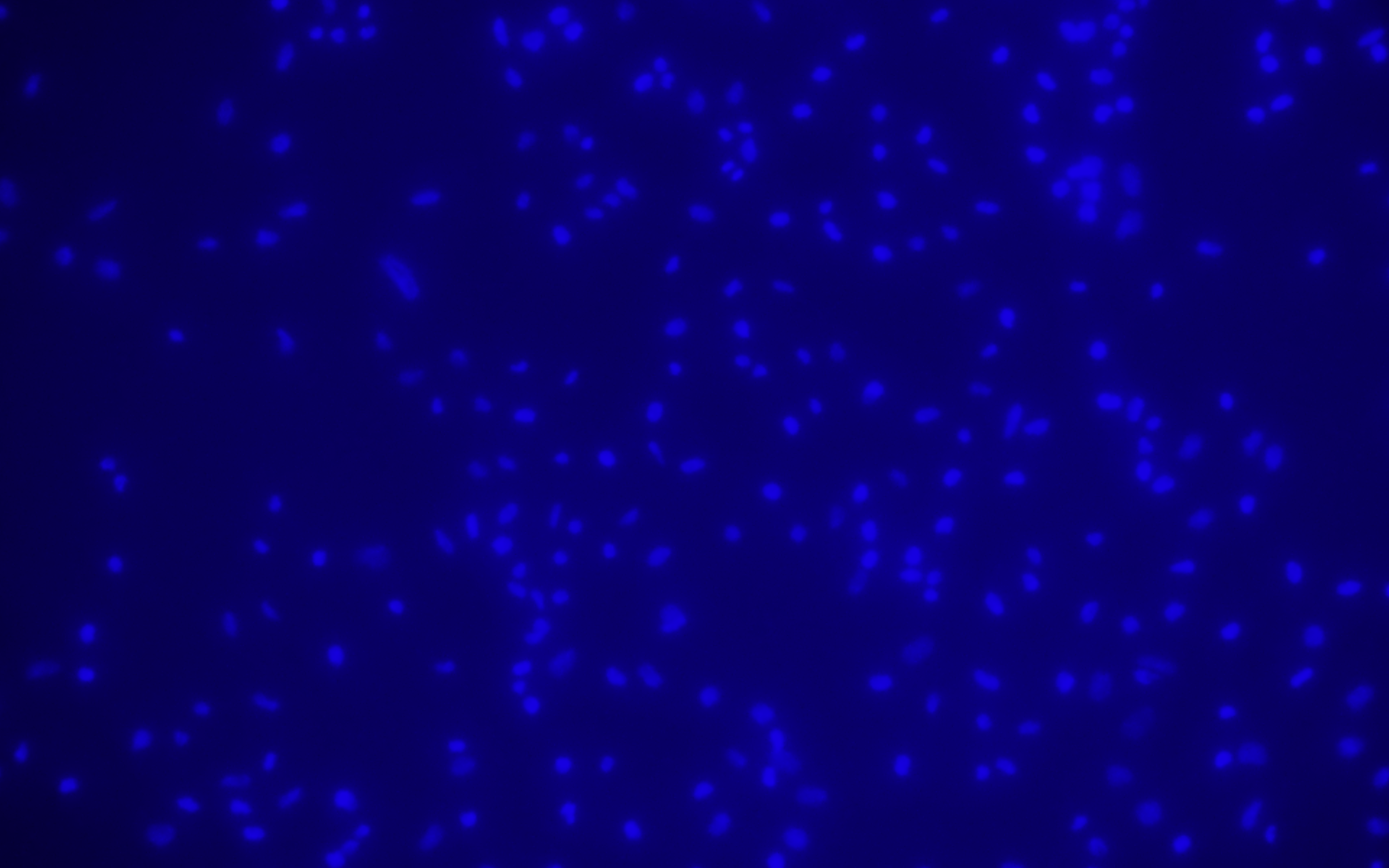

Supplement: Supplementary file 8 — Source data Fig. 3 [file 44321_2024_60_MOESM8_ESM.zip › Source data-Figure 3 (44321_2024_60_MOESM8_ESM)_updated/Figure 3/3I/88T/EV DAPI.tif]

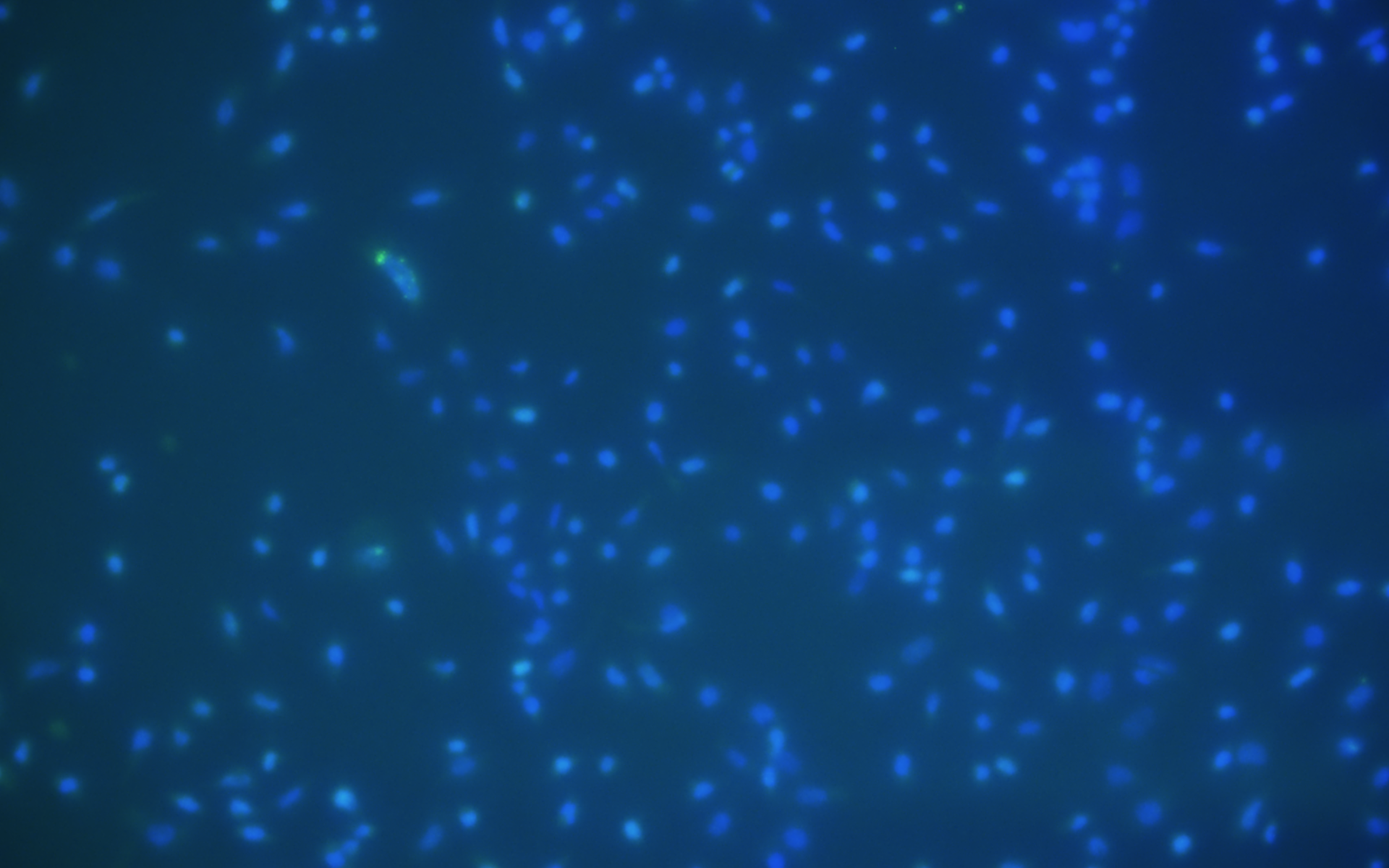

Supplement: Supplementary file 8 — Source data Fig. 3 [file 44321_2024_60_MOESM8_ESM.zip › Source data-Figure 3 (44321_2024_60_MOESM8_ESM)_updated/Figure 3/3I/88T/EV Merge.tif]

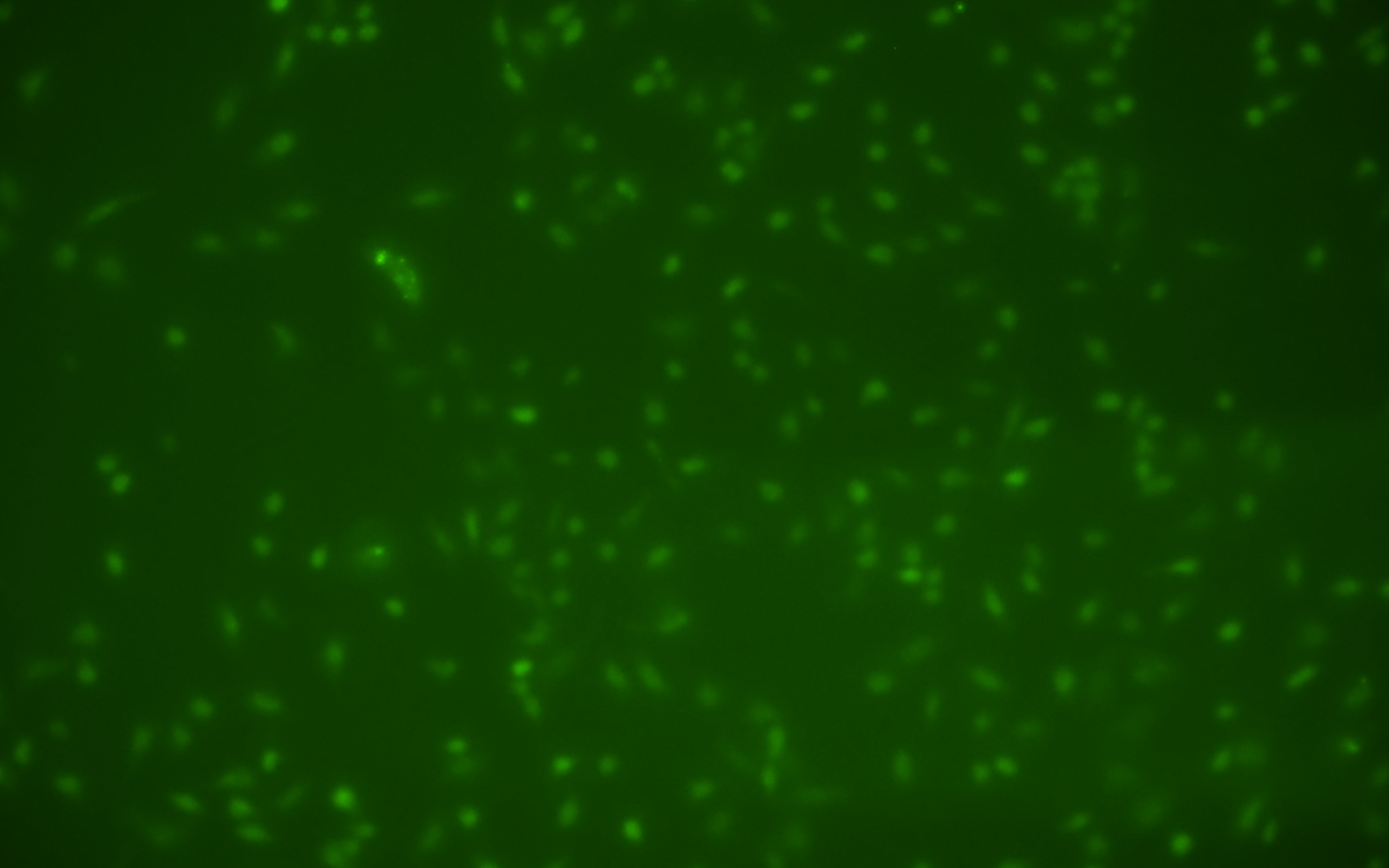

Supplement: Supplementary file 8 — Source data Fig. 3 [file 44321_2024_60_MOESM8_ESM.zip › Source data-Figure 3 (44321_2024_60_MOESM8_ESM)_updated/Figure 3/3I/88T/EV a├H2A.X.tif]

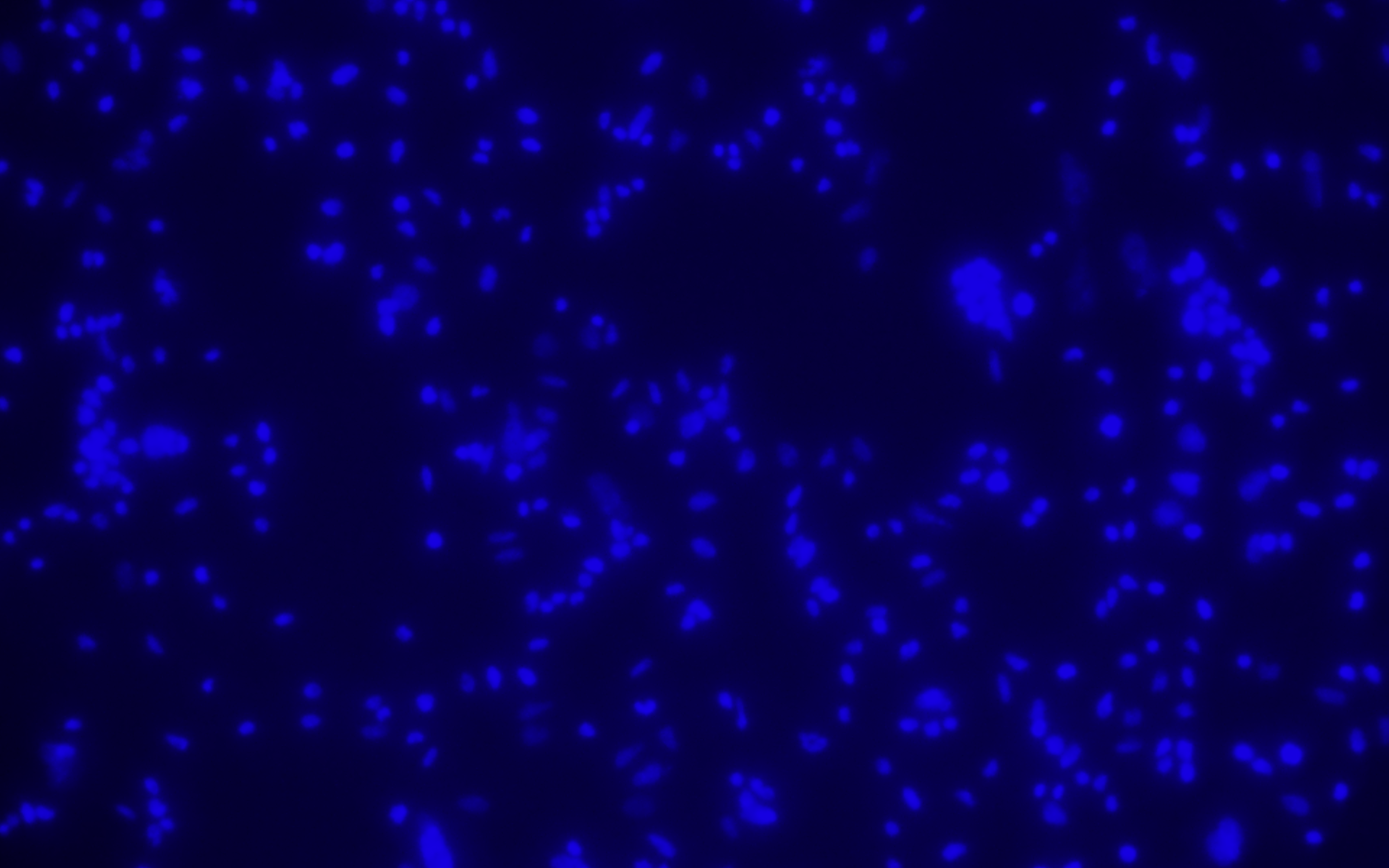

Supplement: Supplementary file 8 — Source data Fig. 3 [file 44321_2024_60_MOESM8_ESM.zip › Source data-Figure 3 (44321_2024_60_MOESM8_ESM)_updated/Figure 3/3I/88T/sg2 DAPI.tif]

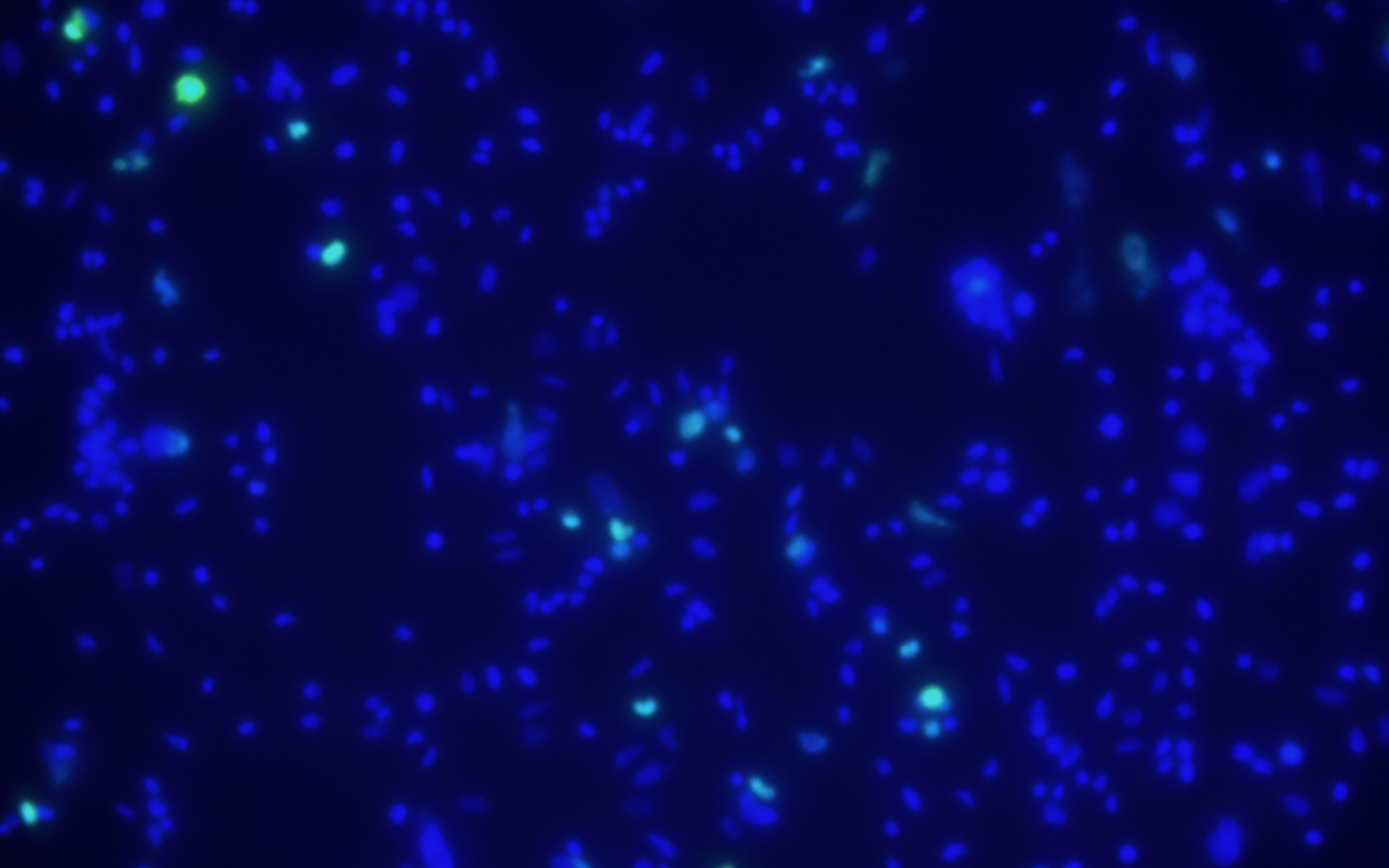

Supplement: Supplementary file 8 — Source data Fig. 3 [file 44321_2024_60_MOESM8_ESM.zip › Source data-Figure 3 (44321_2024_60_MOESM8_ESM)_updated/Figure 3/3I/88T/sg2 Merge.tif]

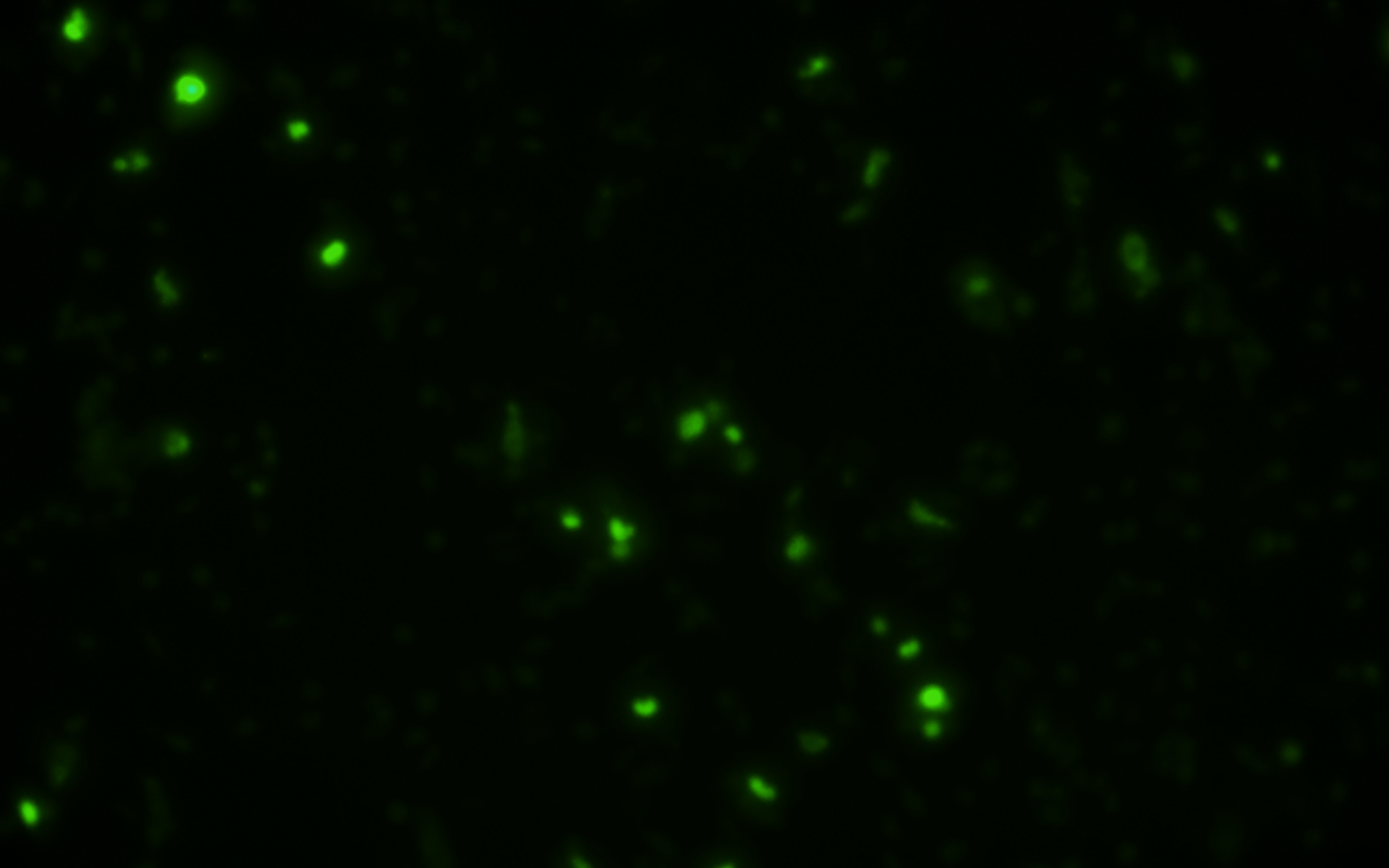

Supplement: Supplementary file 8 — Source data Fig. 3 [file 44321_2024_60_MOESM8_ESM.zip › Source data-Figure 3 (44321_2024_60_MOESM8_ESM)_updated/Figure 3/3I/88T/sg2 a├H2A.X.tif]

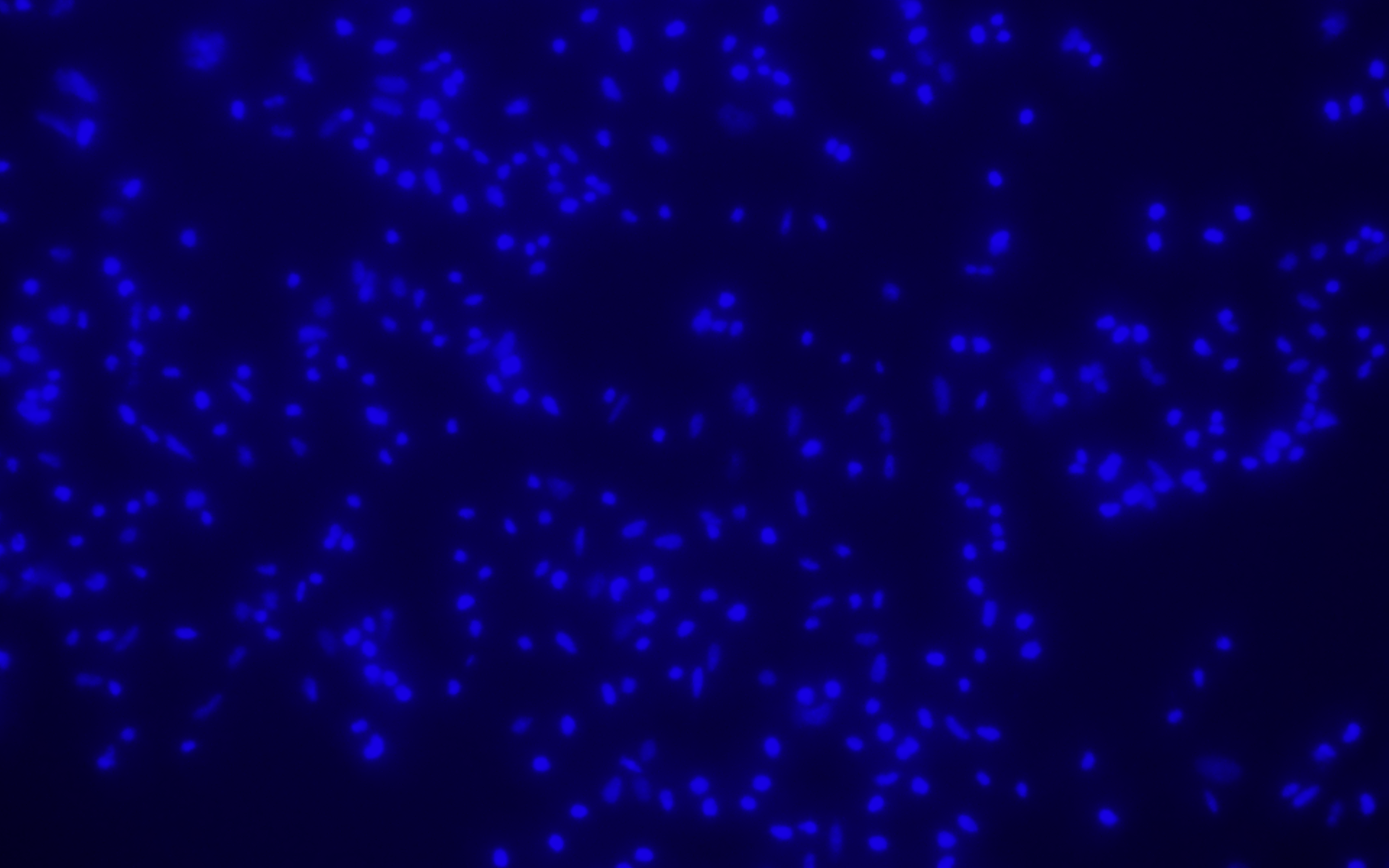

Supplement: Supplementary file 8 — Source data Fig. 3 [file 44321_2024_60_MOESM8_ESM.zip › Source data-Figure 3 (44321_2024_60_MOESM8_ESM)_updated/Figure 3/3I/88T/sg3 DAPI.tif]

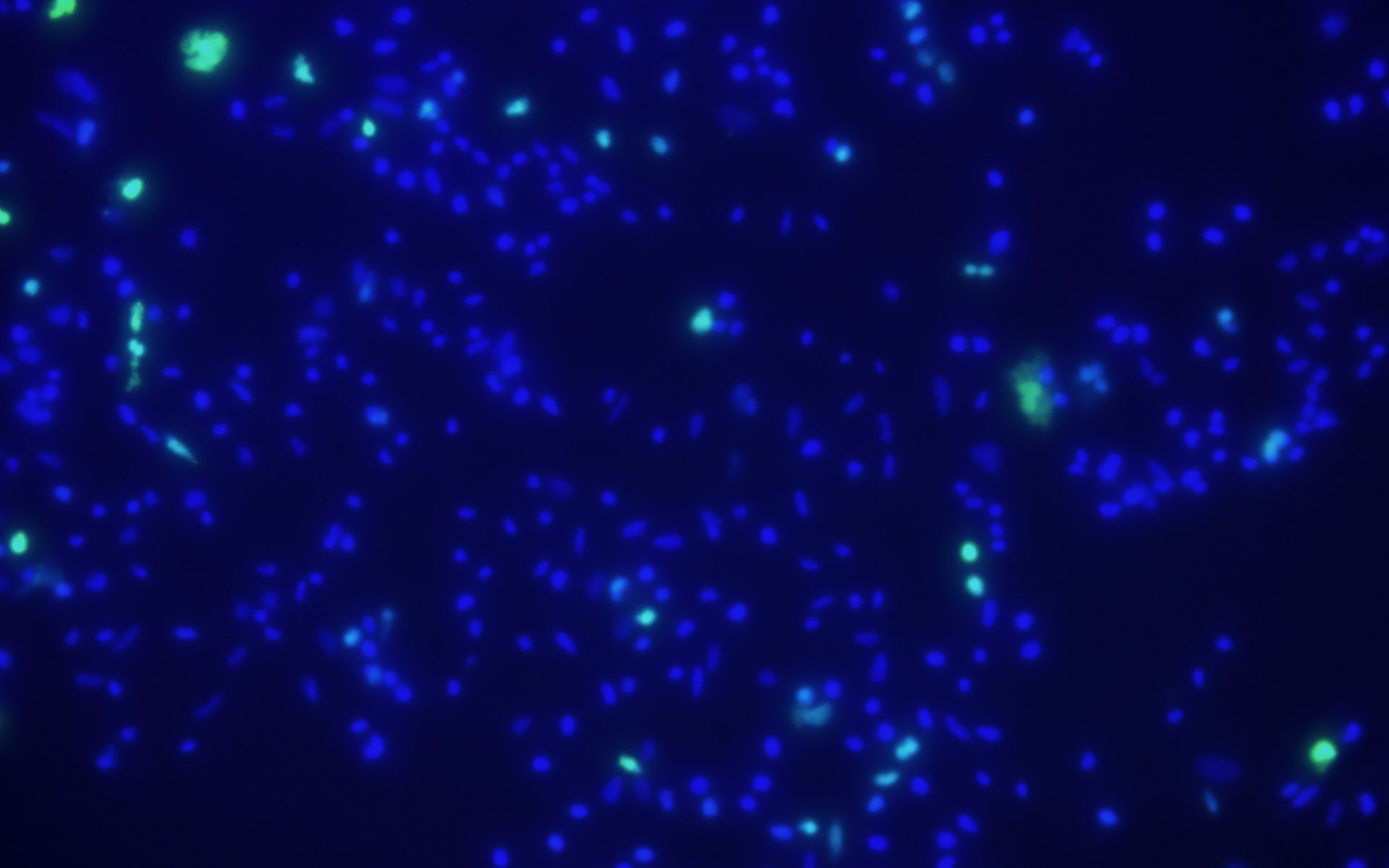

Supplement: Supplementary file 8 — Source data Fig. 3 [file 44321_2024_60_MOESM8_ESM.zip › Source data-Figure 3 (44321_2024_60_MOESM8_ESM)_updated/Figure 3/3I/88T/sg3 Merge.tif]

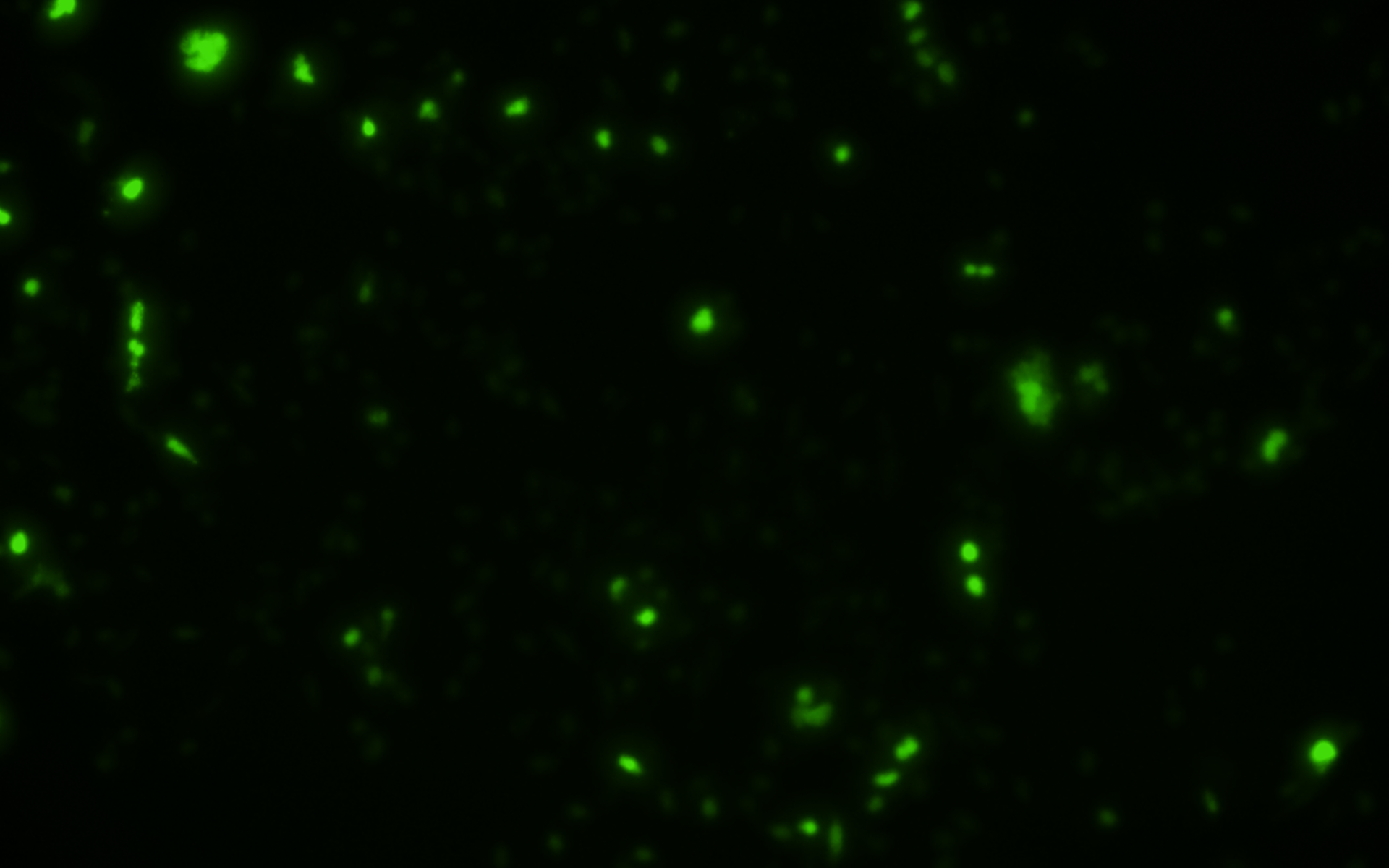

Supplement: Supplementary file 8 — Source data Fig. 3 [file 44321_2024_60_MOESM8_ESM.zip › Source data-Figure 3 (44321_2024_60_MOESM8_ESM)_updated/Figure 3/3I/88T/sg3 a├H2A.X.tif]

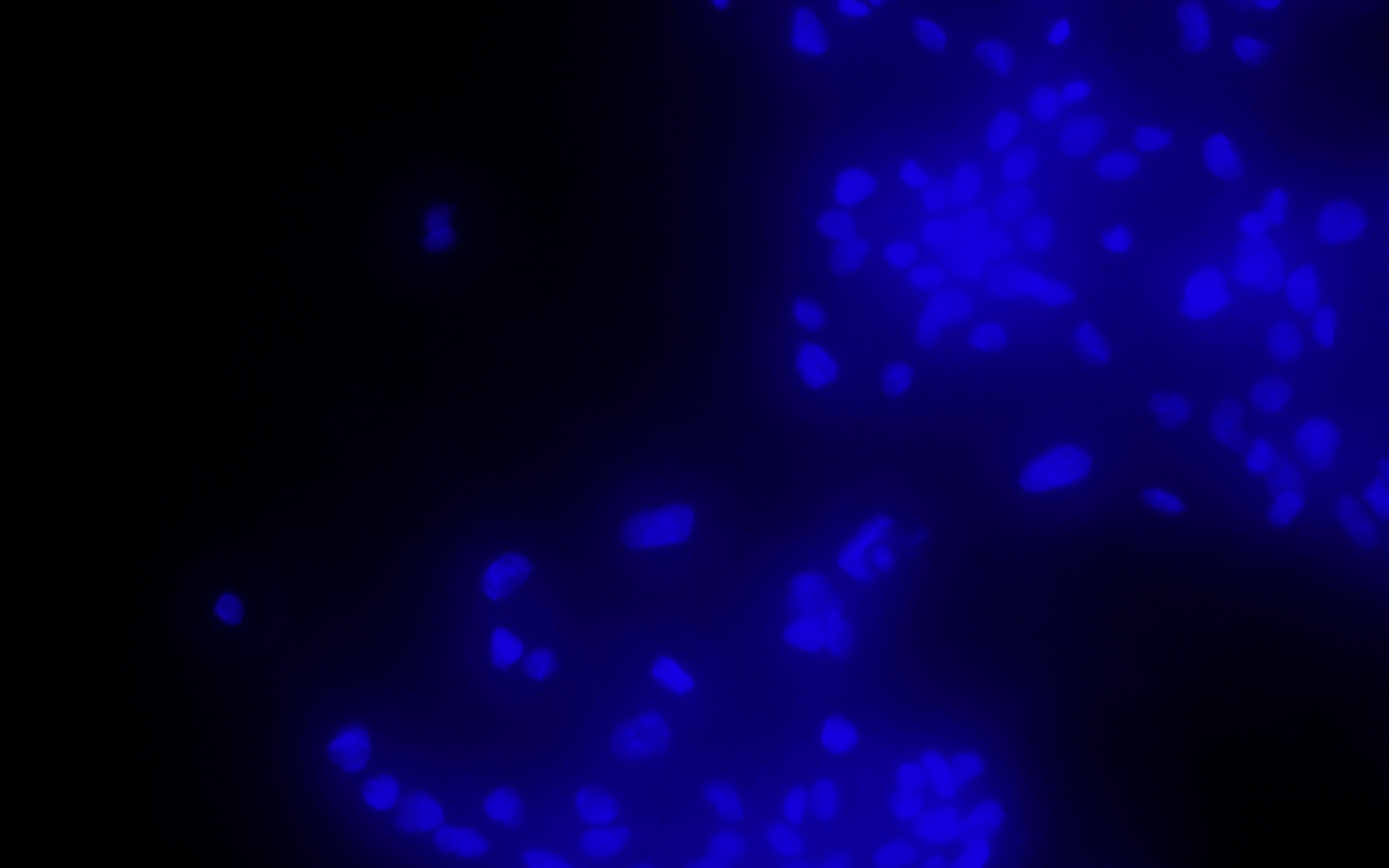

Supplement: Supplementary file 8 — Source data Fig. 3 [file 44321_2024_60_MOESM8_ESM.zip › Source data-Figure 3 (44321_2024_60_MOESM8_ESM)_updated/Figure 3/3I/YAPC/EV DAPI.tif]

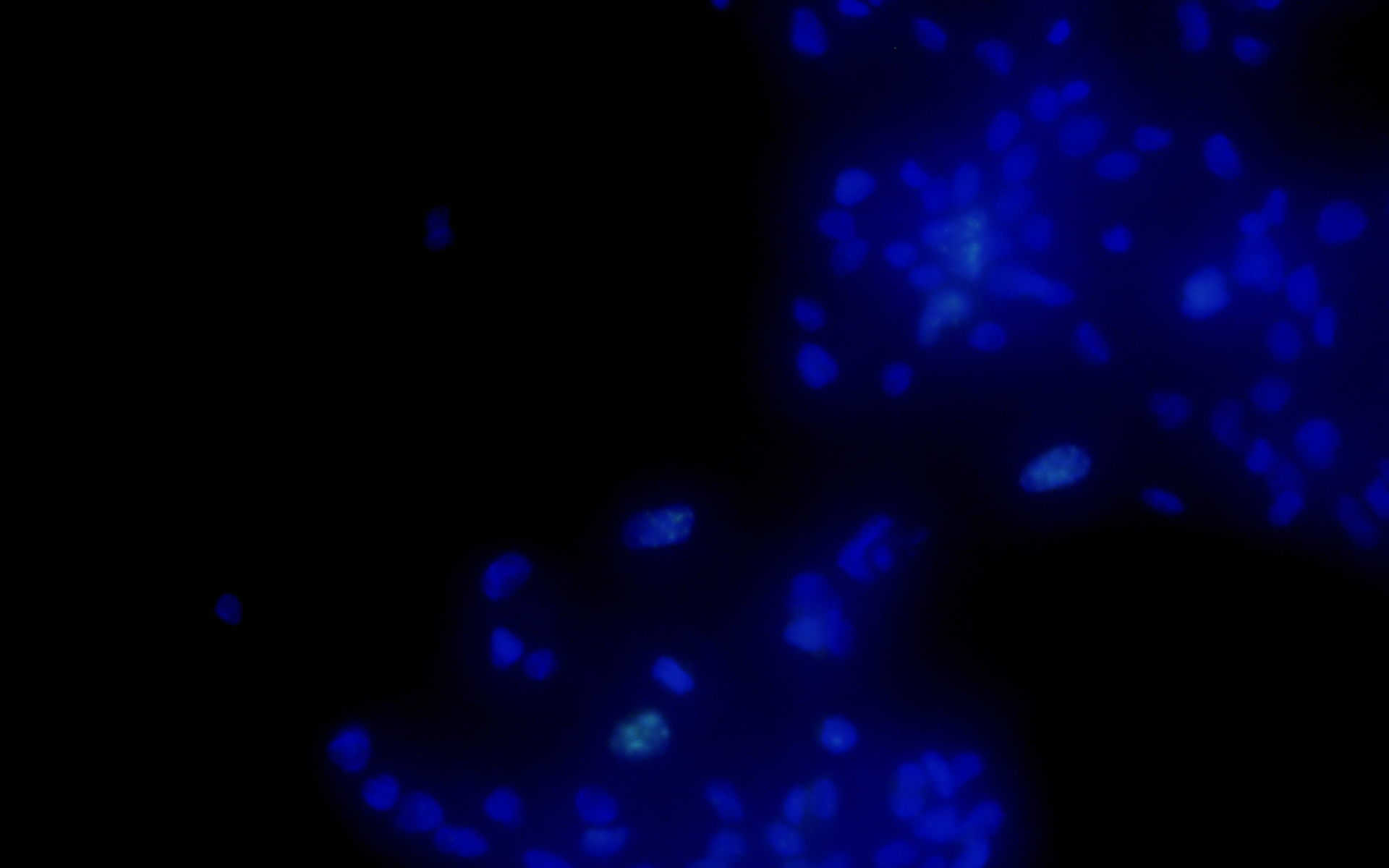

Supplement: Supplementary file 8 — Source data Fig. 3 [file 44321_2024_60_MOESM8_ESM.zip › Source data-Figure 3 (44321_2024_60_MOESM8_ESM)_updated/Figure 3/3I/YAPC/EV Merge.tif]

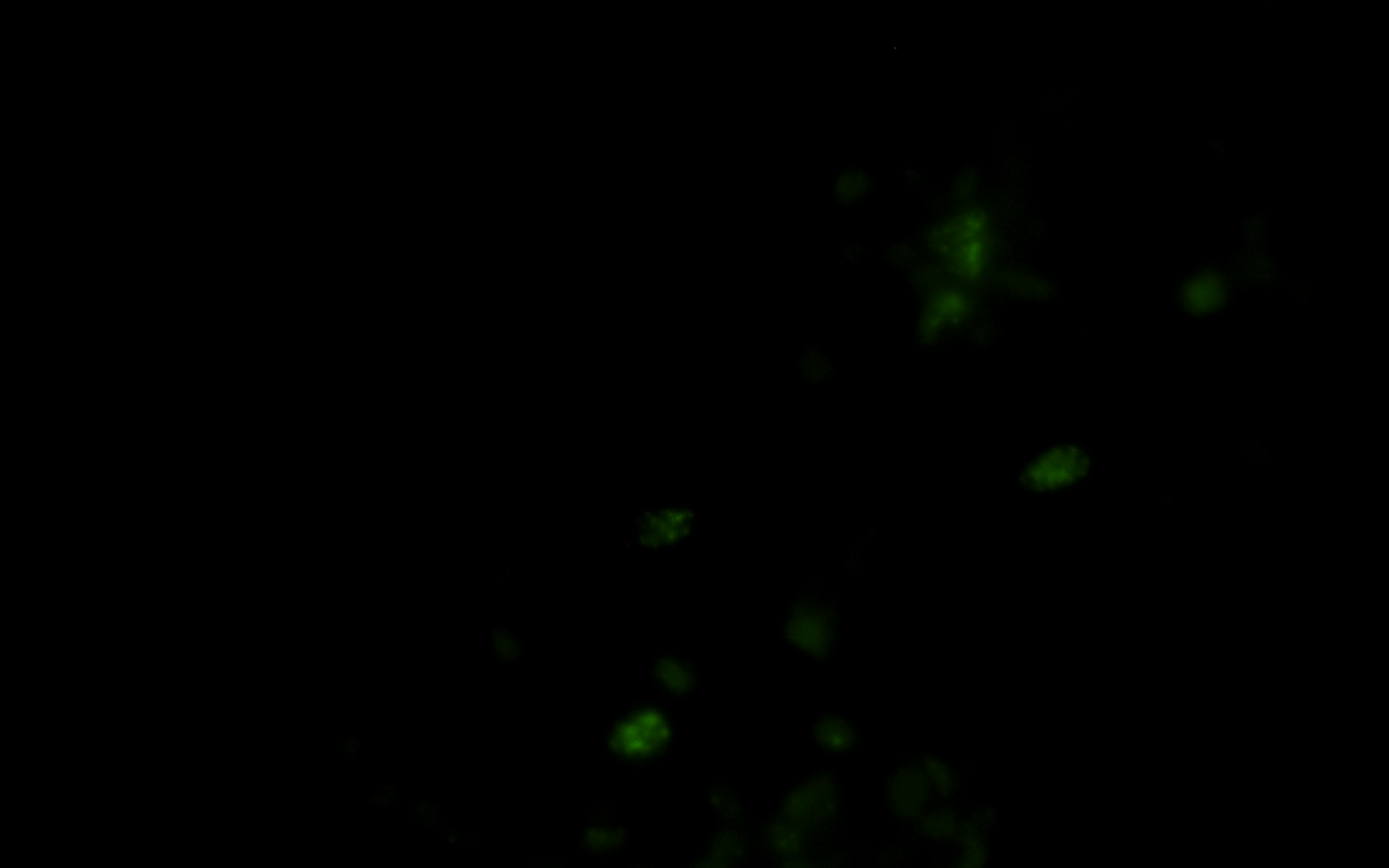

Supplement: Supplementary file 8 — Source data Fig. 3 [file 44321_2024_60_MOESM8_ESM.zip › Source data-Figure 3 (44321_2024_60_MOESM8_ESM)_updated/Figure 3/3I/YAPC/EV a├H2A.X.tif]

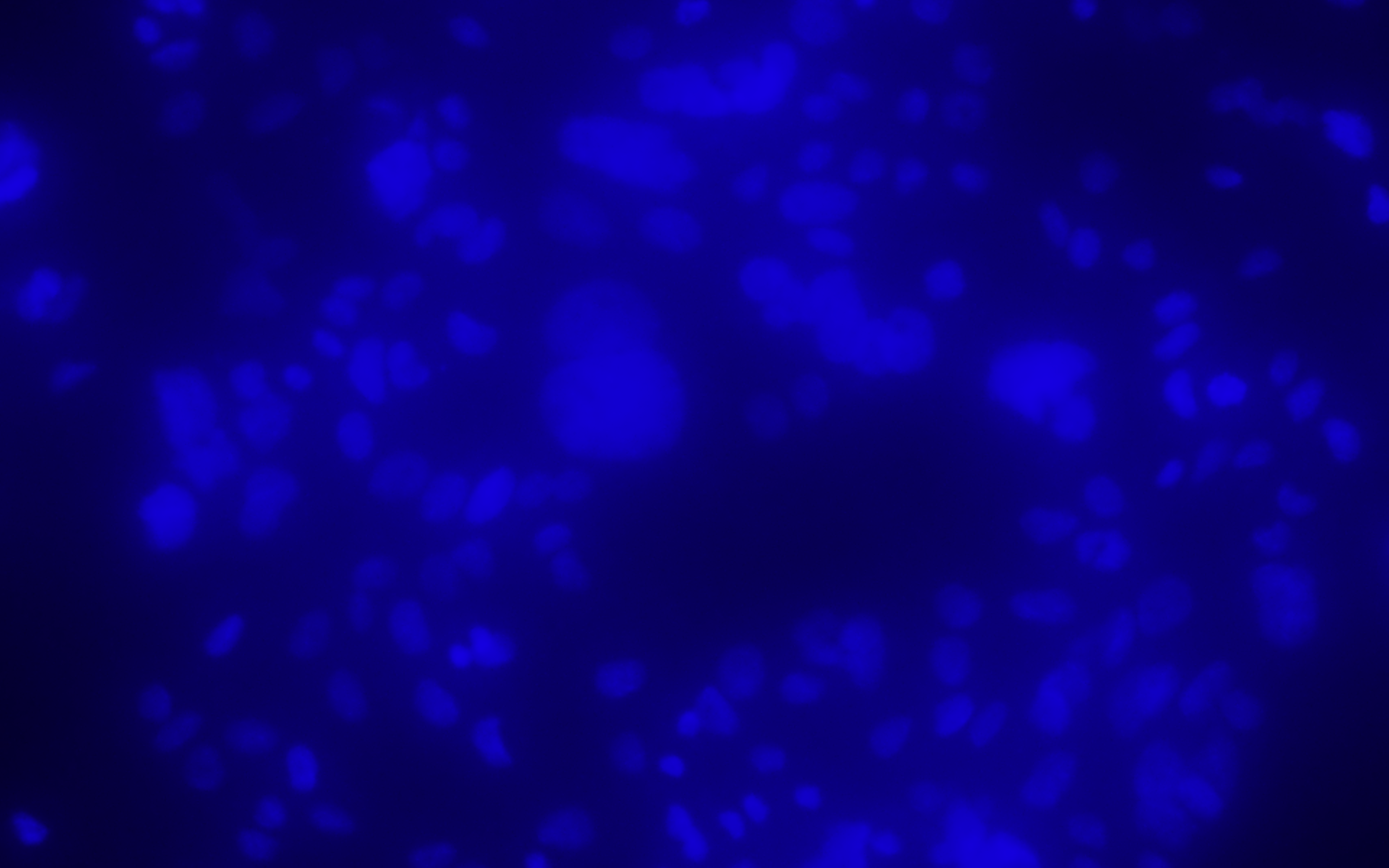

Supplement: Supplementary file 8 — Source data Fig. 3 [file 44321_2024_60_MOESM8_ESM.zip › Source data-Figure 3 (44321_2024_60_MOESM8_ESM)_updated/Figure 3/3I/YAPC/sg2 DAPI.tif]

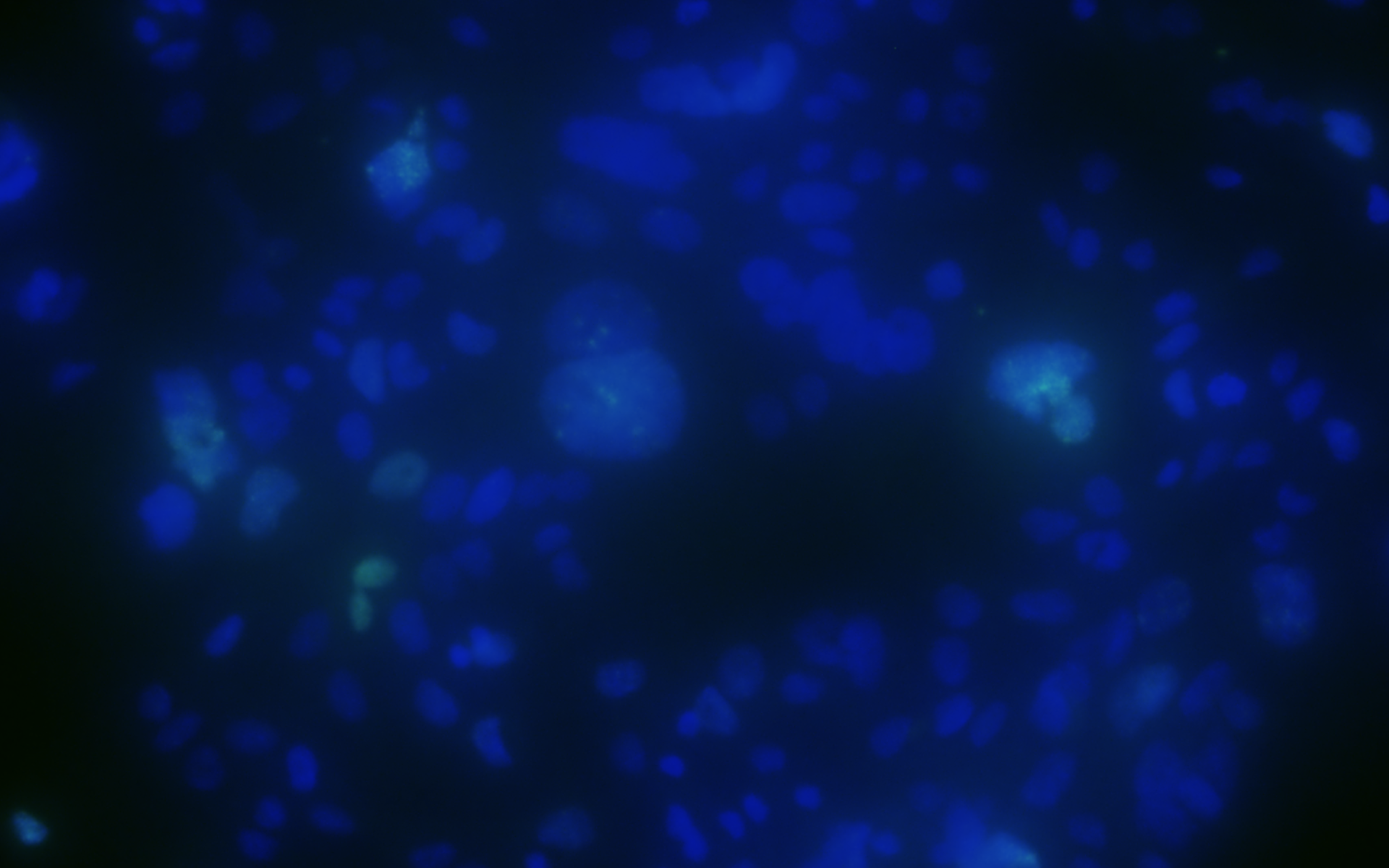

Supplement: Supplementary file 8 — Source data Fig. 3 [file 44321_2024_60_MOESM8_ESM.zip › Source data-Figure 3 (44321_2024_60_MOESM8_ESM)_updated/Figure 3/3I/YAPC/sg2 Merge.tif]

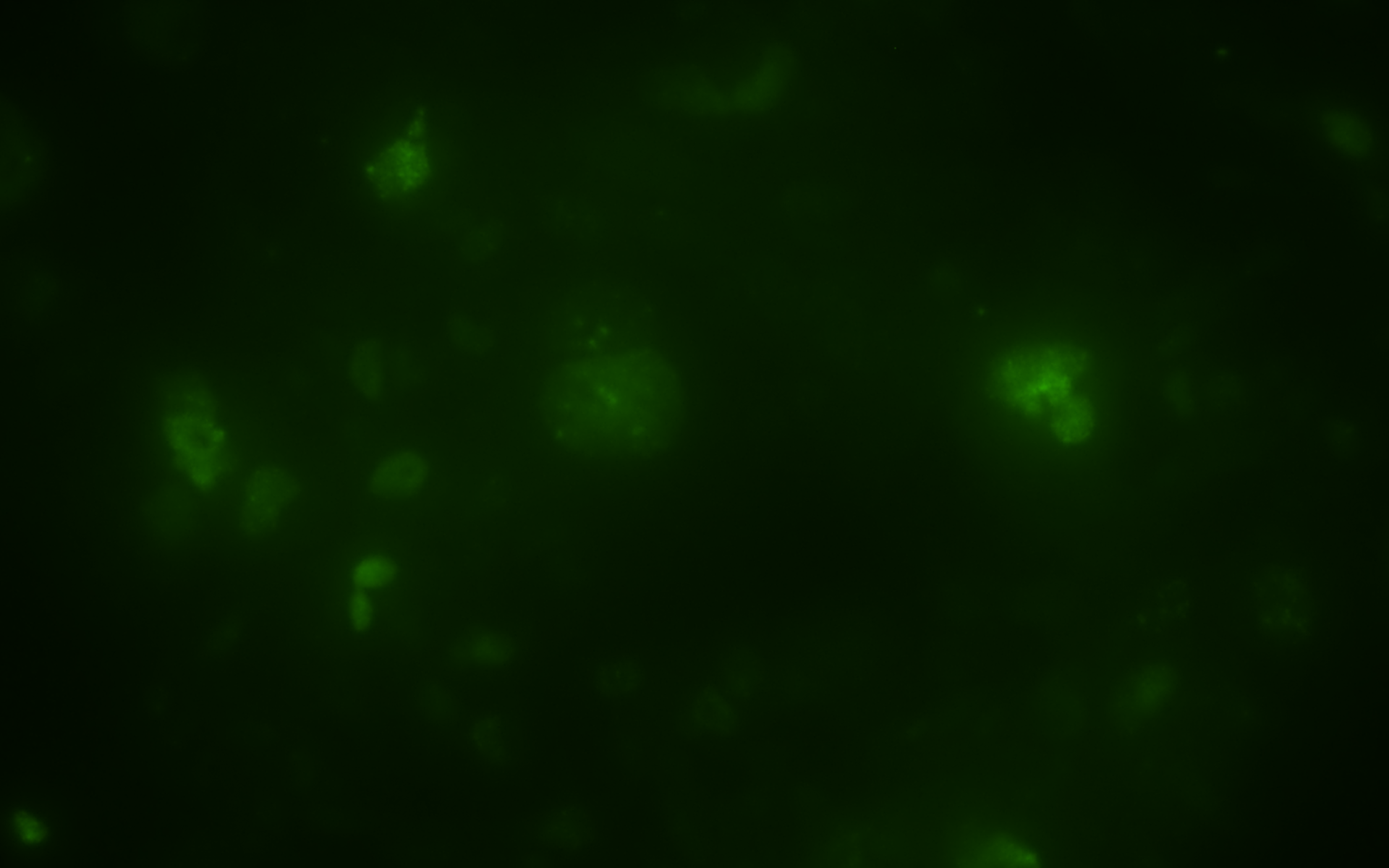

Supplement: Supplementary file 8 — Source data Fig. 3 [file 44321_2024_60_MOESM8_ESM.zip › Source data-Figure 3 (44321_2024_60_MOESM8_ESM)_updated/Figure 3/3I/YAPC/sg2 a├H2A.X.tif]

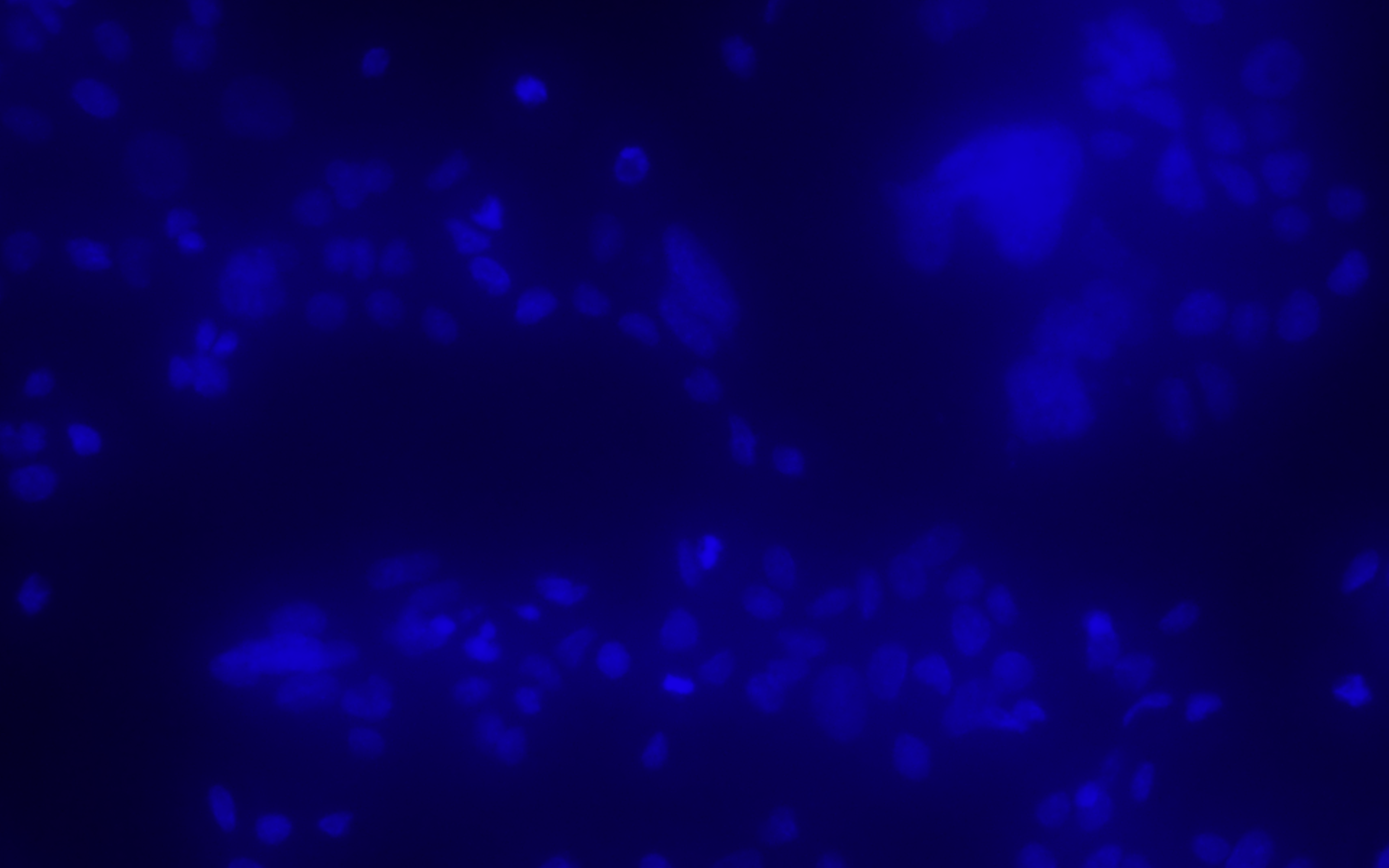

Supplement: Supplementary file 8 — Source data Fig. 3 [file 44321_2024_60_MOESM8_ESM.zip › Source data-Figure 3 (44321_2024_60_MOESM8_ESM)_updated/Figure 3/3I/YAPC/sg3 DAPI.tif]

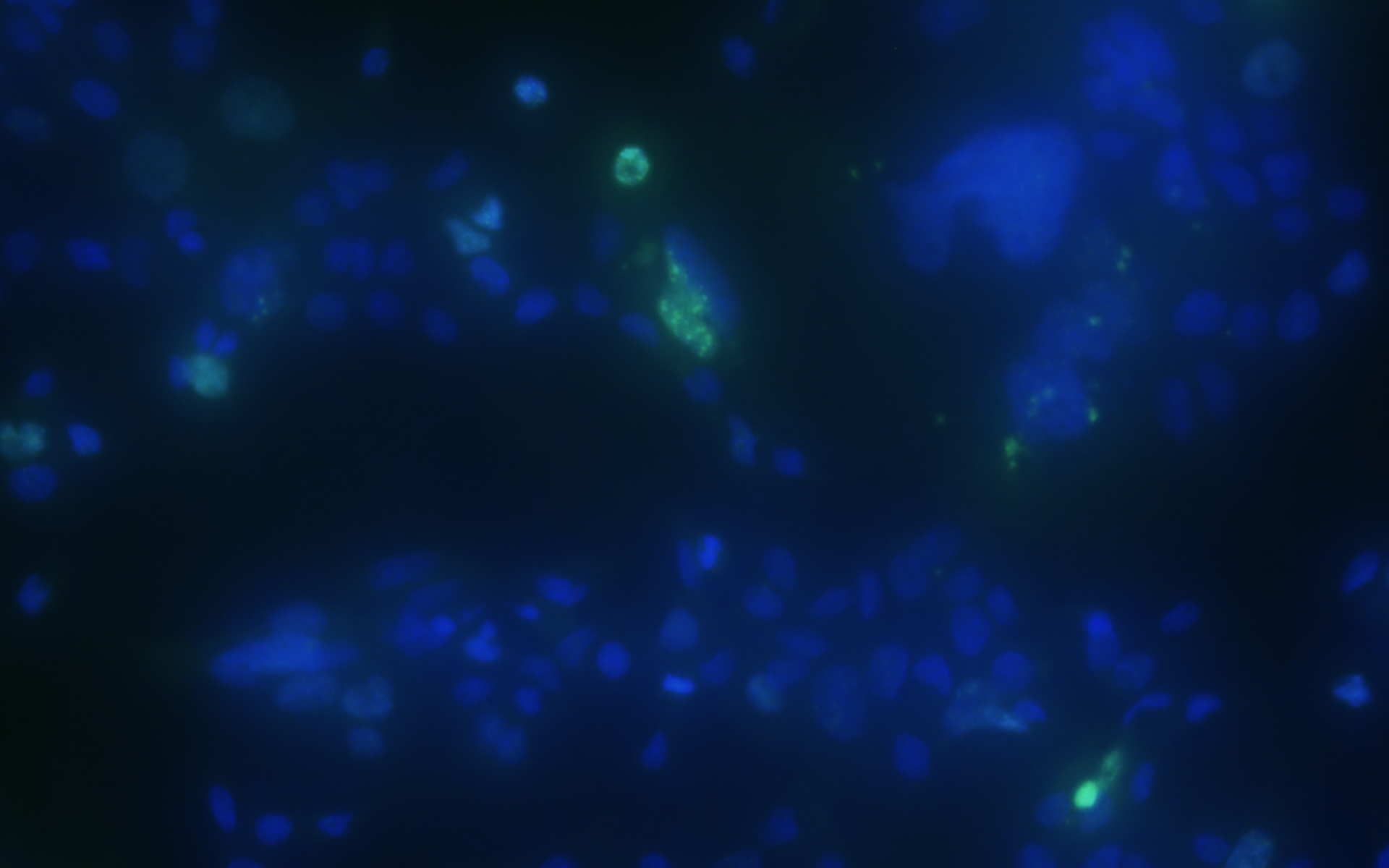

Supplement: Supplementary file 8 — Source data Fig. 3 [file 44321_2024_60_MOESM8_ESM.zip › Source data-Figure 3 (44321_2024_60_MOESM8_ESM)_updated/Figure 3/3I/YAPC/sg3 Merge.tif]

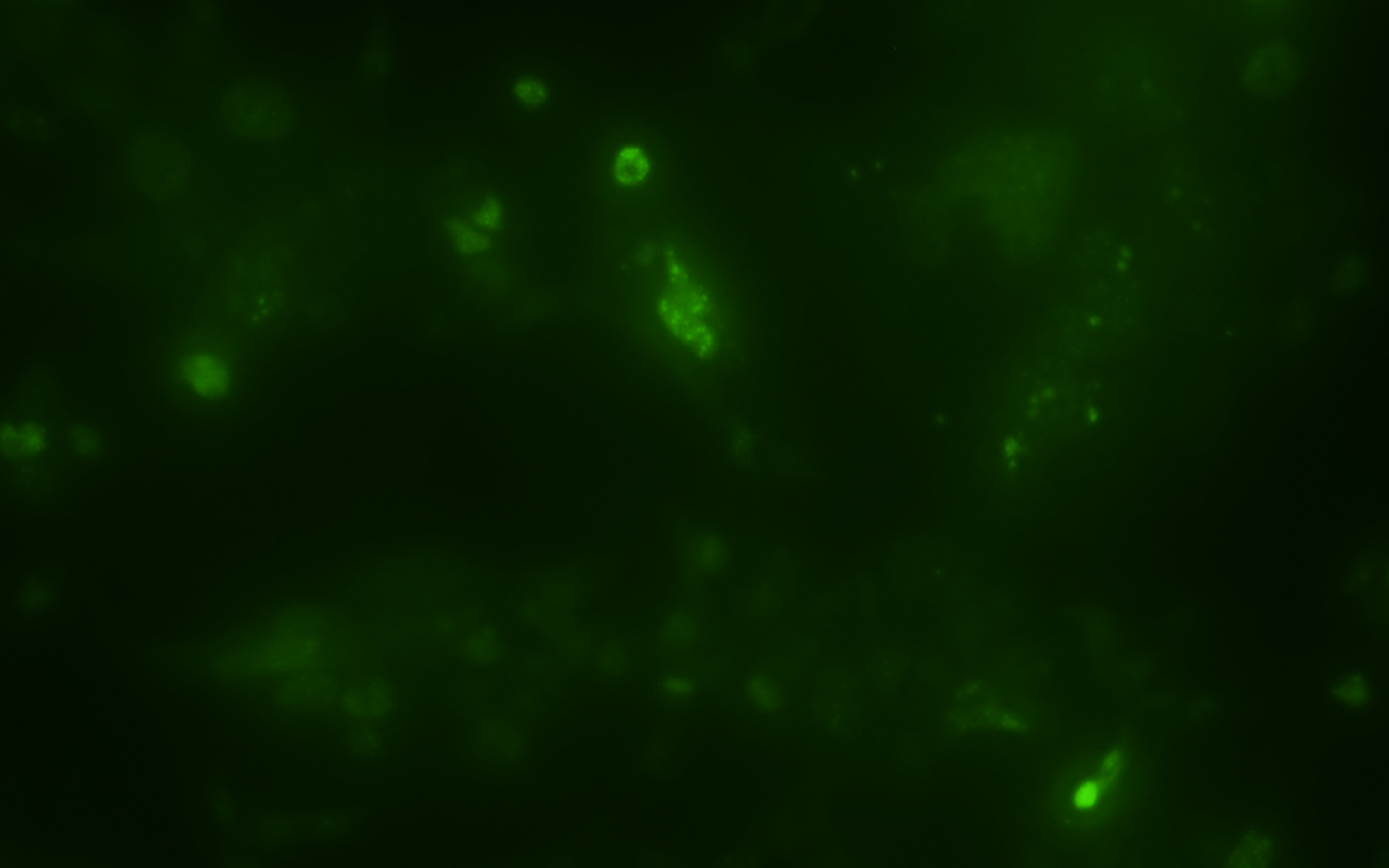

Supplement: Supplementary file 8 — Source data Fig. 3 [file 44321_2024_60_MOESM8_ESM.zip › Source data-Figure 3 (44321_2024_60_MOESM8_ESM)_updated/Figure 3/3I/YAPC/sg3 a├H2A.X.tif]

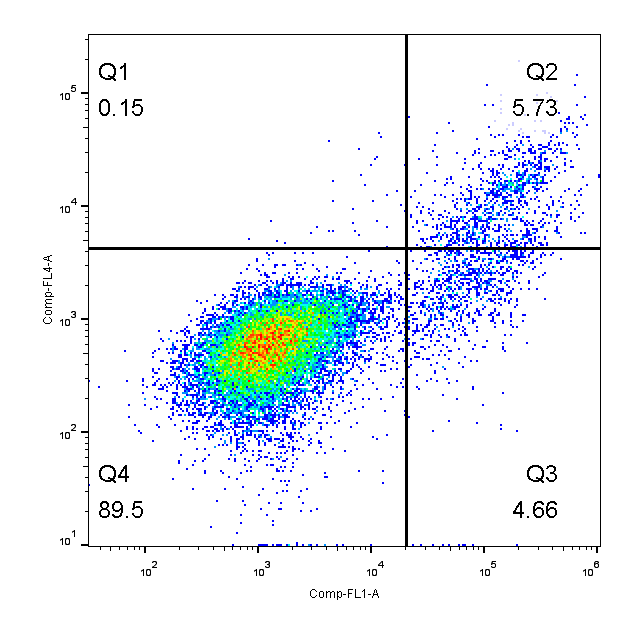

Supplement: Supplementary file 8 — Source data Fig. 3 [file 44321_2024_60_MOESM8_ESM.zip › Source data-Figure 3 (44321_2024_60_MOESM8_ESM)_updated/Figure 3/3J/YAPC/005yapc 05Ctrl 002.LMD_apopotosis.png]

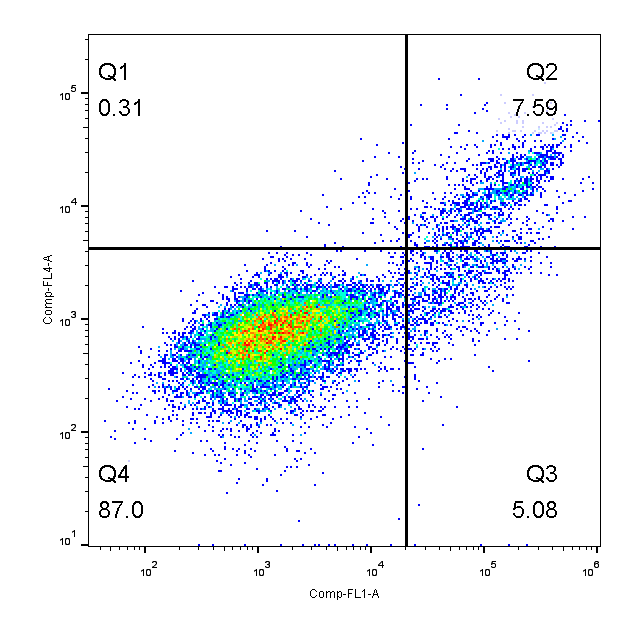

Supplement: Supplementary file 8 — Source data Fig. 3 [file 44321_2024_60_MOESM8_ESM.zip › Source data-Figure 3 (44321_2024_60_MOESM8_ESM)_updated/Figure 3/3J/YAPC/005yapc 05sg2 003.LMD_apopotosis.png]

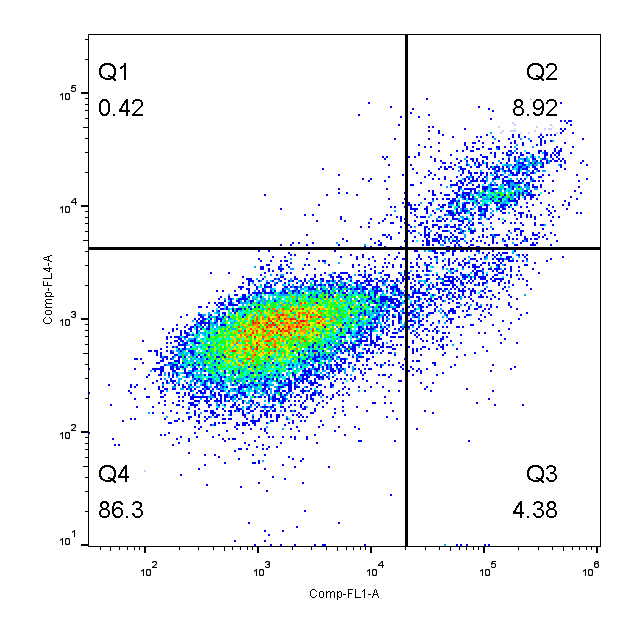

Supplement: Supplementary file 8 — Source data Fig. 3 [file 44321_2024_60_MOESM8_ESM.zip › Source data-Figure 3 (44321_2024_60_MOESM8_ESM)_updated/Figure 3/3J/YAPC/005yapc 05sg3 004.LMD_apopotosis.png]

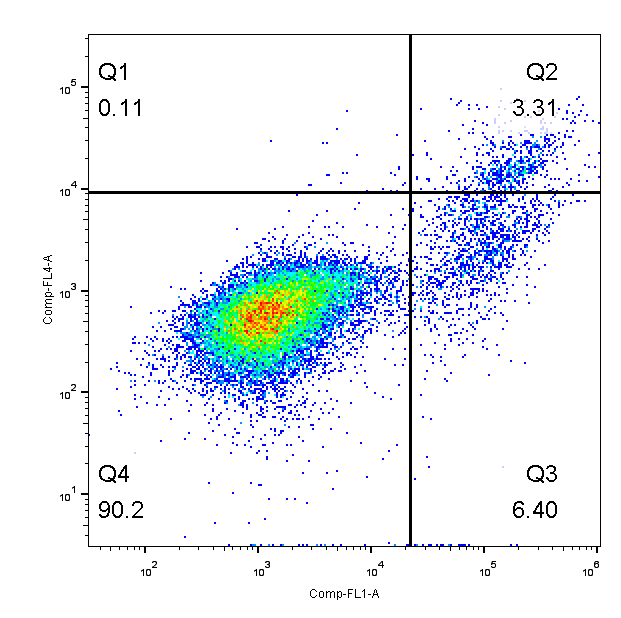

Supplement: Supplementary file 8 — Source data Fig. 3 [file 44321_2024_60_MOESM8_ESM.zip › Source data-Figure 3 (44321_2024_60_MOESM8_ESM)_updated/Figure 3/3J/YAPC/03YAPC 03Ctrl 002.LMD_C apo.png]

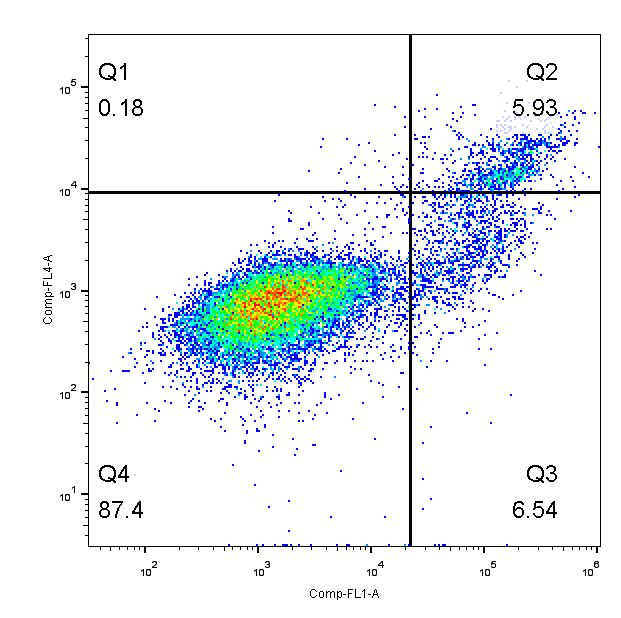

Supplement: Supplementary file 8 — Source data Fig. 3 [file 44321_2024_60_MOESM8_ESM.zip › Source data-Figure 3 (44321_2024_60_MOESM8_ESM)_updated/Figure 3/3J/YAPC/03YAPC 03sg2 003.LMD_C apo.png]

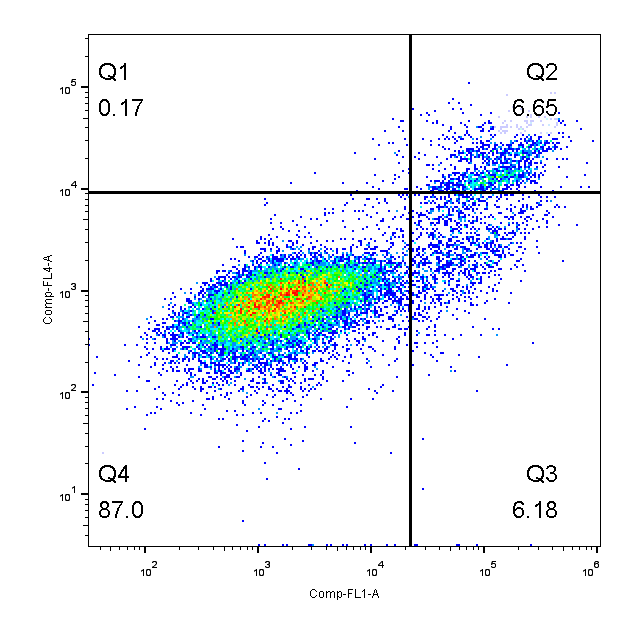

Supplement: Supplementary file 8 — Source data Fig. 3 [file 44321_2024_60_MOESM8_ESM.zip › Source data-Figure 3 (44321_2024_60_MOESM8_ESM)_updated/Figure 3/3J/YAPC/03YAPC 03sg3 004.LMD_C apo.png]

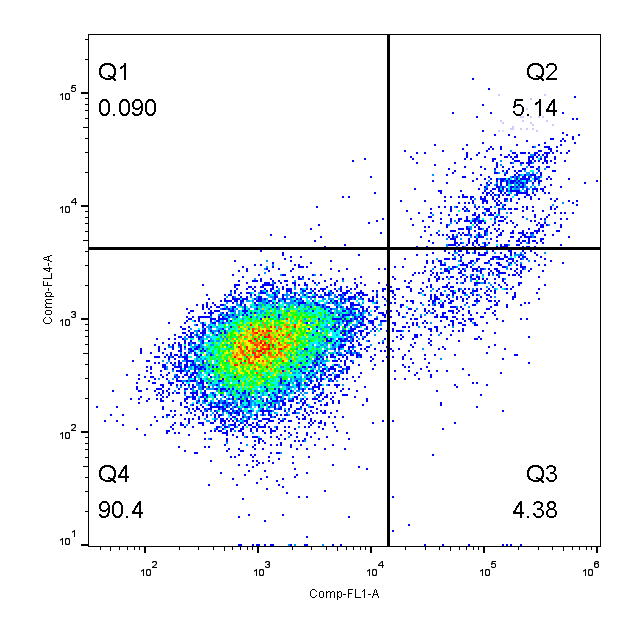

Supplement: Supplementary file 8 — Source data Fig. 3 [file 44321_2024_60_MOESM8_ESM.zip › Source data-Figure 3 (44321_2024_60_MOESM8_ESM)_updated/Figure 3/3J/YAPC/04YAPC 04Ctrl 002.LMD_apo.png]

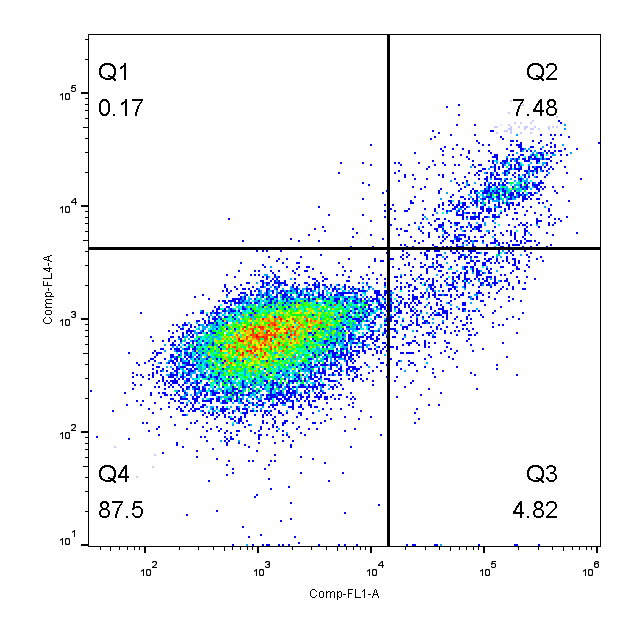

Supplement: Supplementary file 8 — Source data Fig. 3 [file 44321_2024_60_MOESM8_ESM.zip › Source data-Figure 3 (44321_2024_60_MOESM8_ESM)_updated/Figure 3/3J/YAPC/04YAPC 04sg2 003.LMD_apo.png]

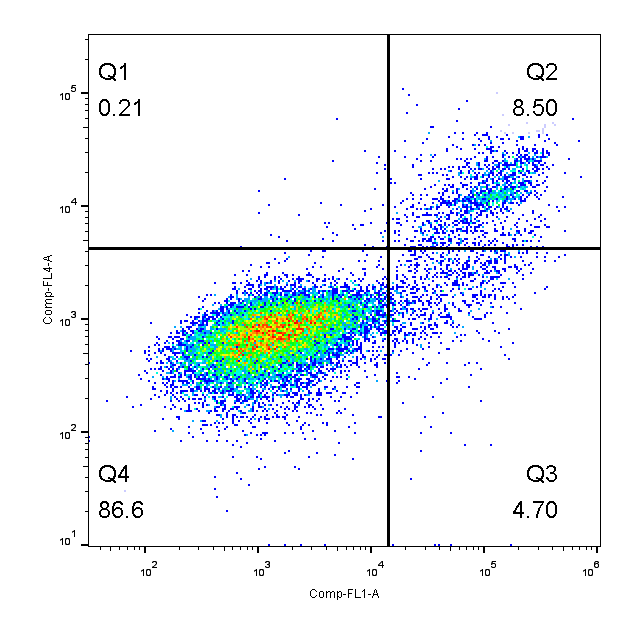

Supplement: Supplementary file 8 — Source data Fig. 3 [file 44321_2024_60_MOESM8_ESM.zip › Source data-Figure 3 (44321_2024_60_MOESM8_ESM)_updated/Figure 3/3J/YAPC/04YAPC 04sg3 004.LMD_apo.png]

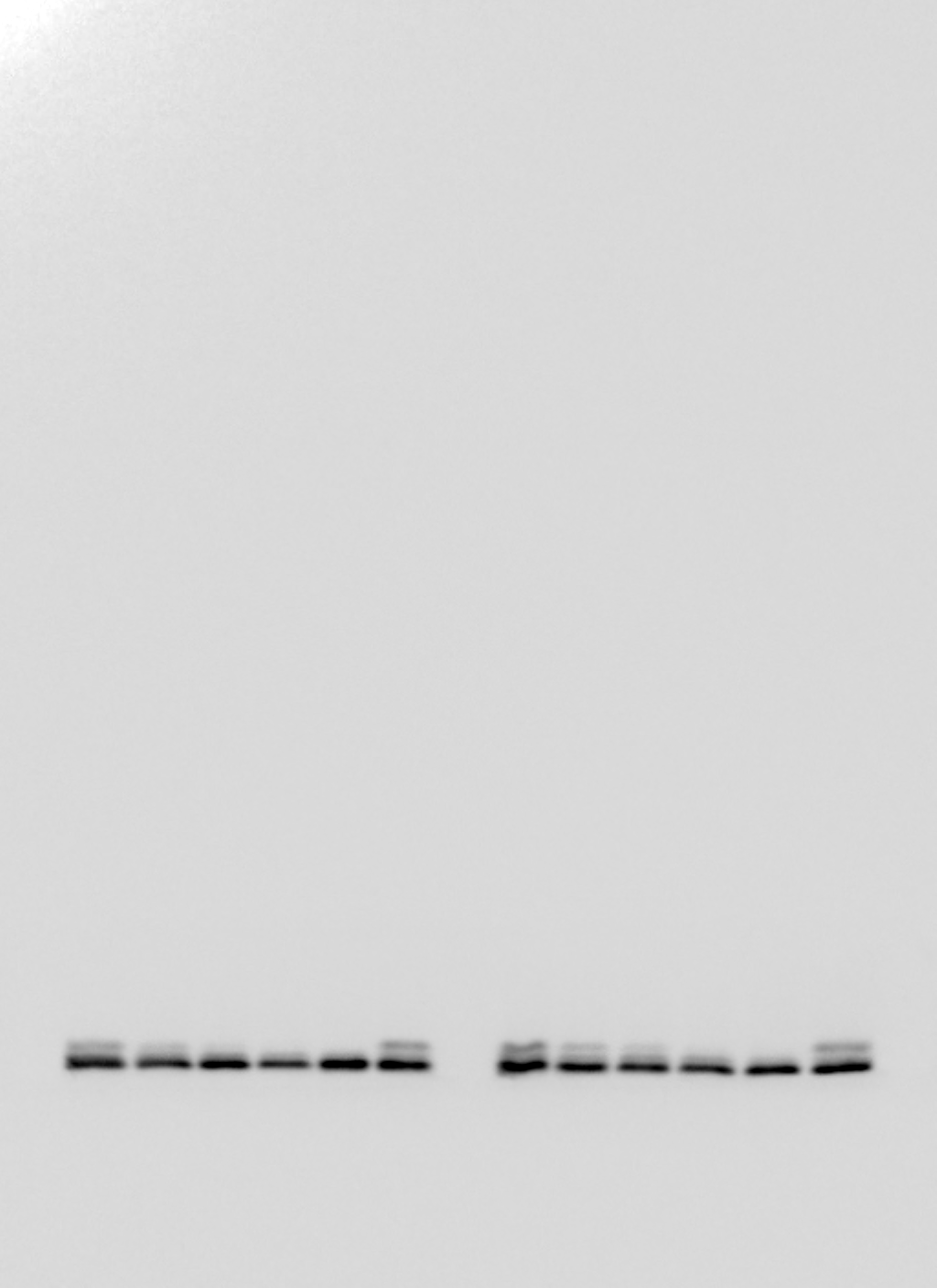

Supplement: Supplementary file 9 — Source data Fig. 4 [file 44321_2024_60_MOESM9_ESM.zip › Figure 4/4B/88T/Western CDK1/1 1st CDK1 7 _Ch.tif]

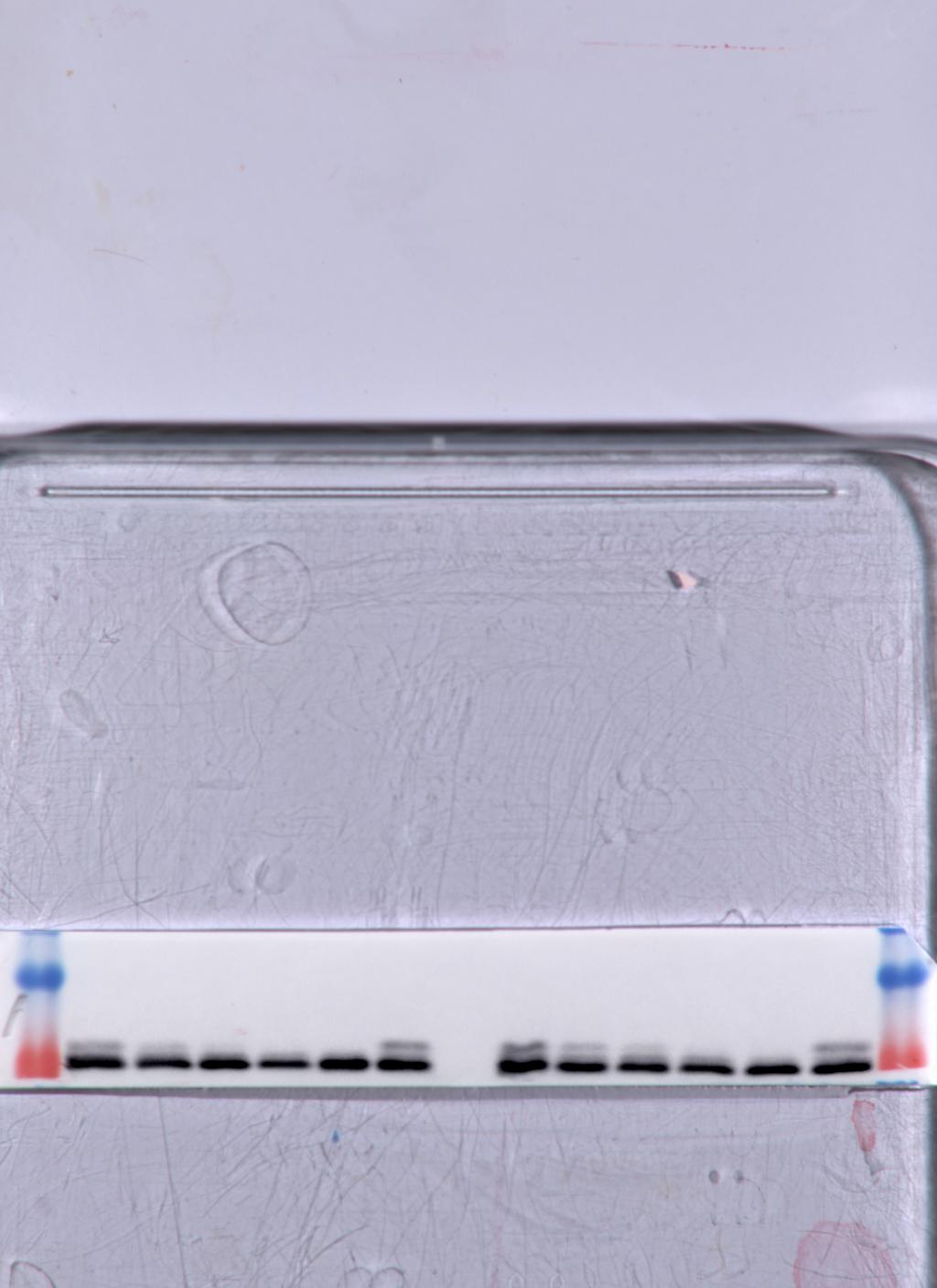

Supplement: Supplementary file 9 — Source data Fig. 4 [file 44321_2024_60_MOESM9_ESM.zip › Figure 4/4B/88T/Western CDK1/1 1st CDK1 7 _Ch+Marker.jpg]

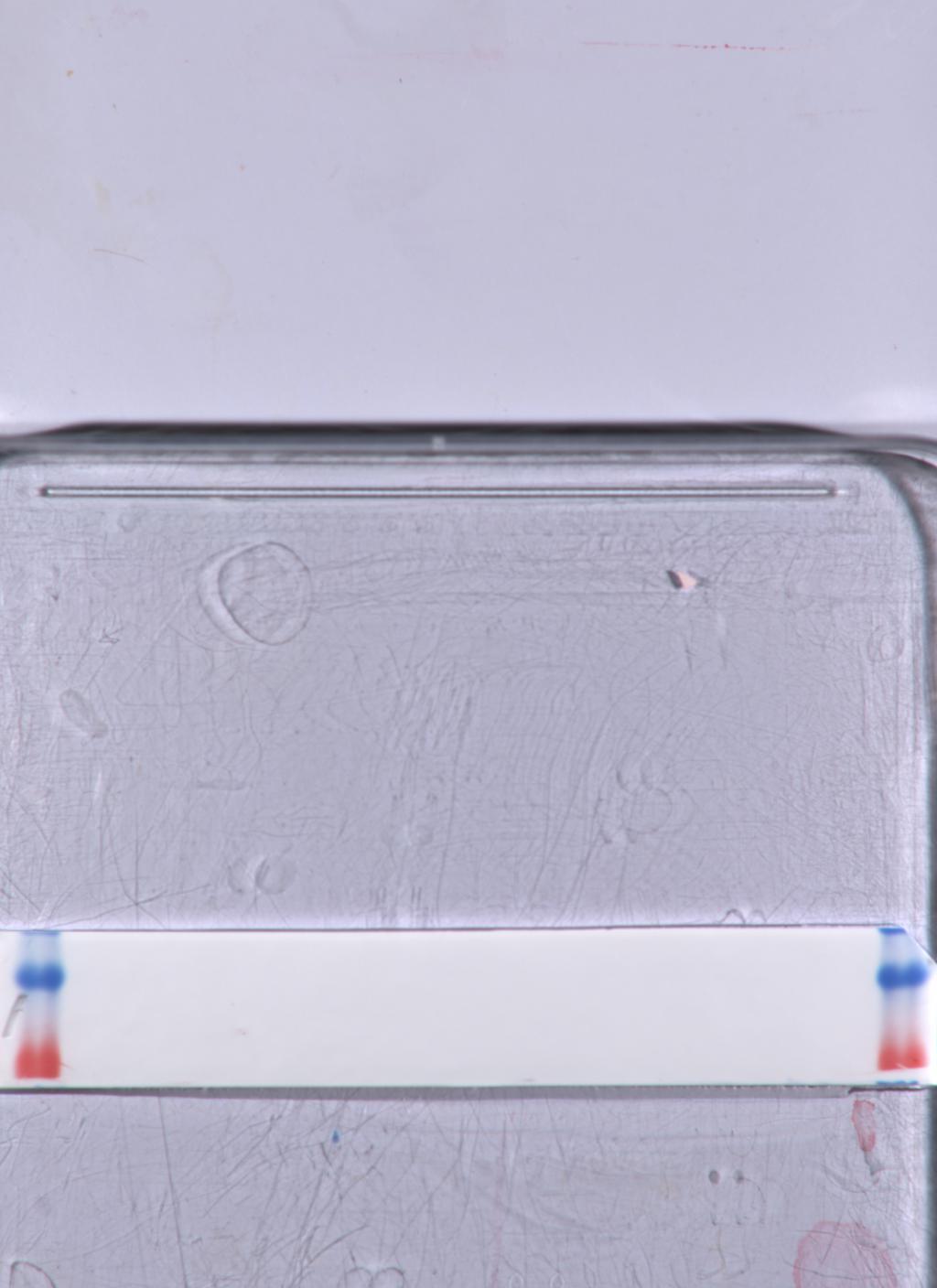

Supplement: Supplementary file 9 — Source data Fig. 4 [file 44321_2024_60_MOESM9_ESM.zip › Figure 4/4B/88T/Western CDK1/1 1st CDK1 7 _Ch-Marker.jpg]

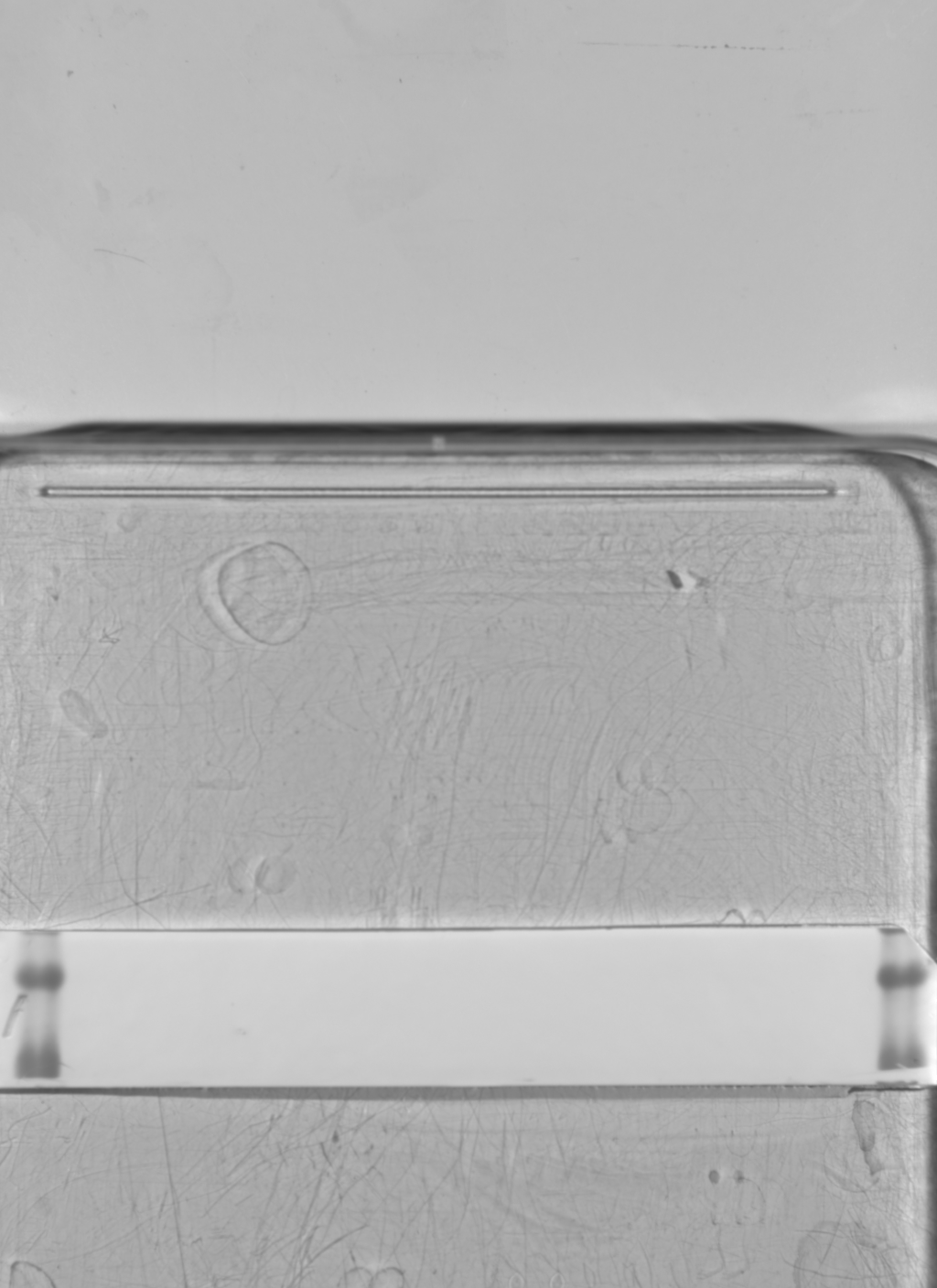

Supplement: Supplementary file 9 — Source data Fig. 4 [file 44321_2024_60_MOESM9_ESM.zip › Figure 4/4B/88T/Western CDK1/1 1st CDK1 7 _Ch-Marker.tif]

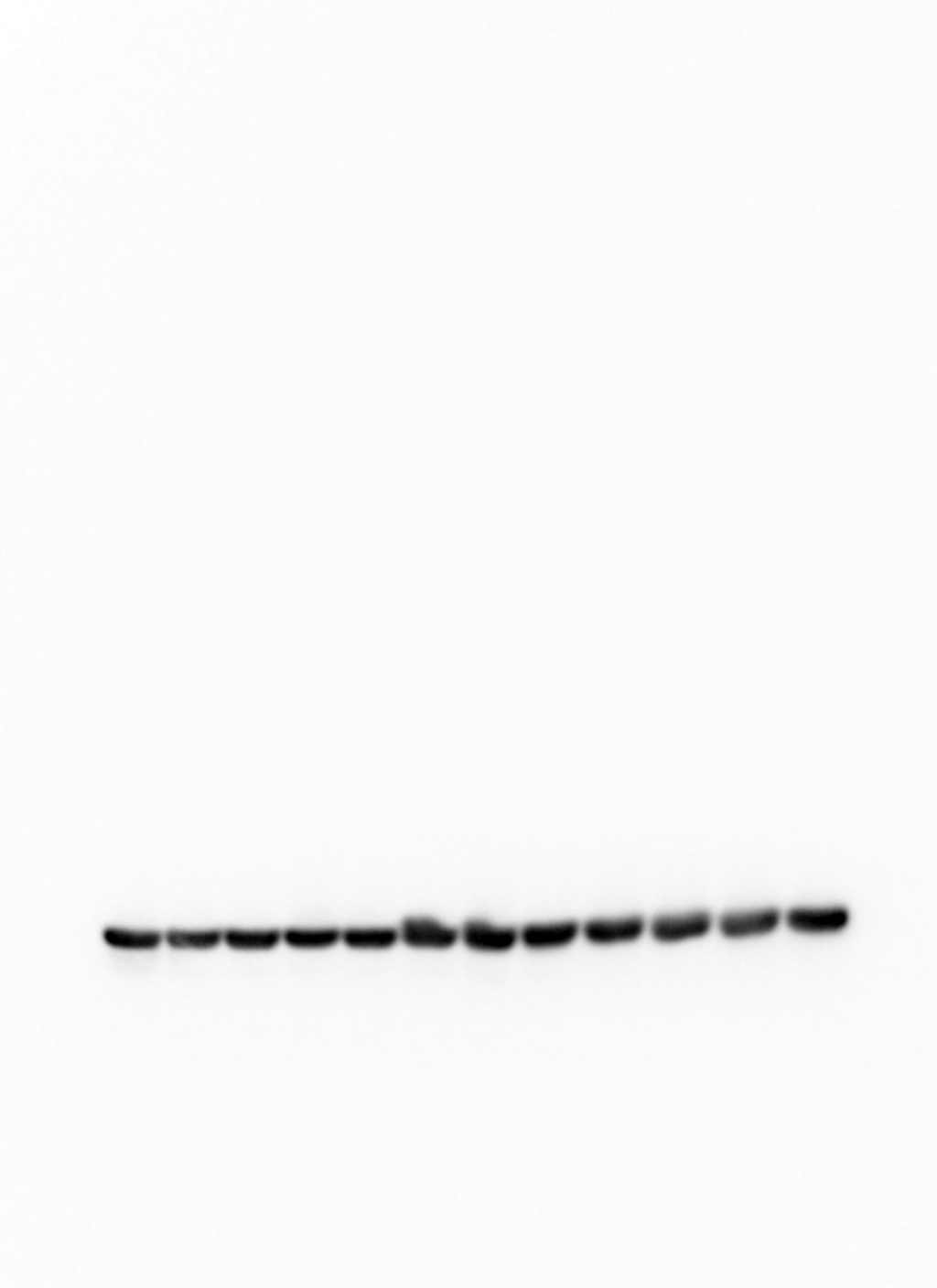

Supplement: Supplementary file 9 — Source data Fig. 4 [file 44321_2024_60_MOESM9_ESM.zip › Figure 4/4B/88T/Western GAPDH/4 2nd GAP 0.5 _Ch.tif]

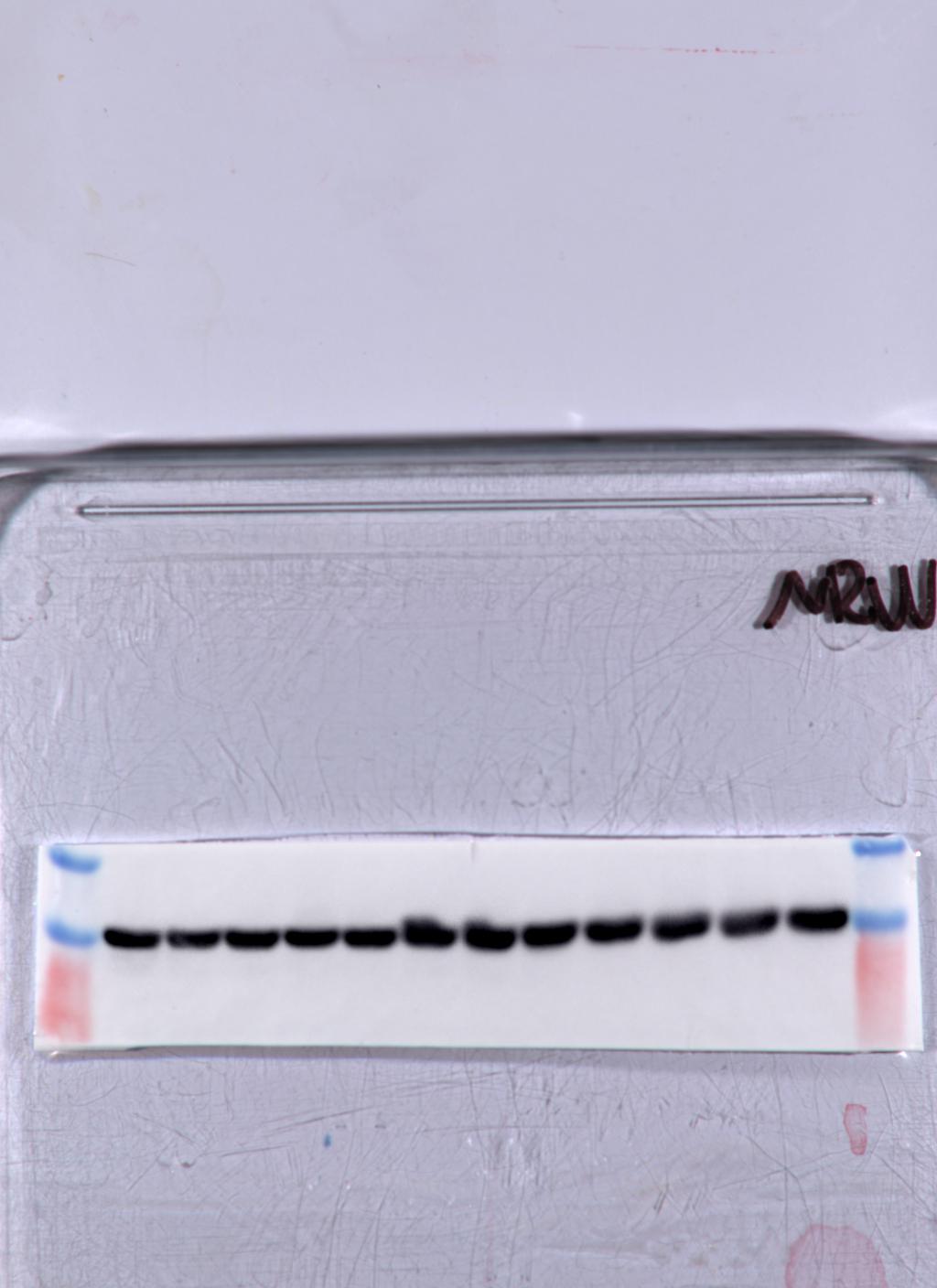

Supplement: Supplementary file 9 — Source data Fig. 4 [file 44321_2024_60_MOESM9_ESM.zip › Figure 4/4B/88T/Western GAPDH/4 2nd GAP 0.5 _Ch+Marker.jpg]

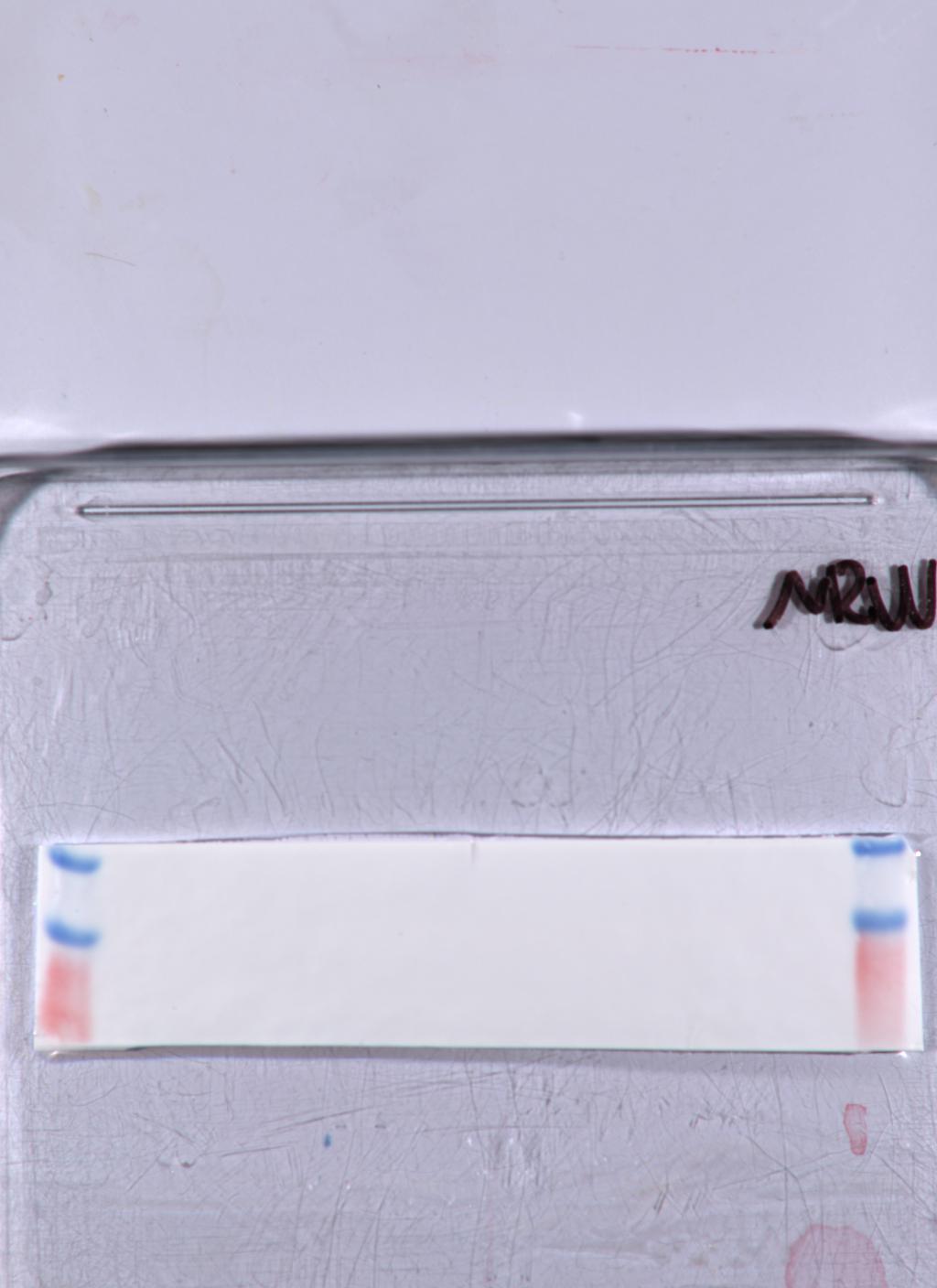

Supplement: Supplementary file 9 — Source data Fig. 4 [file 44321_2024_60_MOESM9_ESM.zip › Figure 4/4B/88T/Western GAPDH/4 2nd GAP 0.5 _Ch-Marker.jpg]

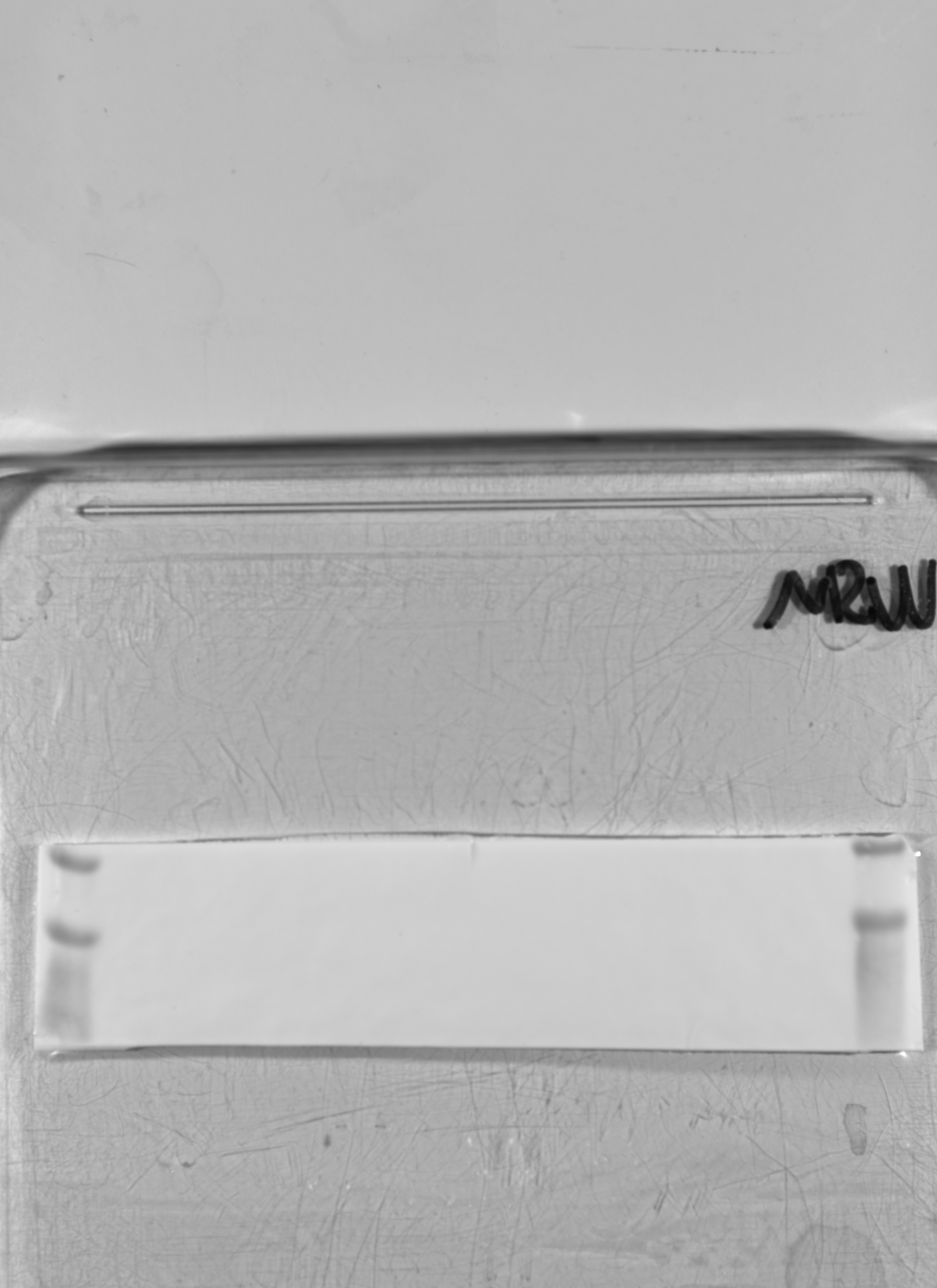

Supplement: Supplementary file 9 — Source data Fig. 4 [file 44321_2024_60_MOESM9_ESM.zip › Figure 4/4B/88T/Western GAPDH/4 2nd GAP 0.5 _Ch-Marker.tif]

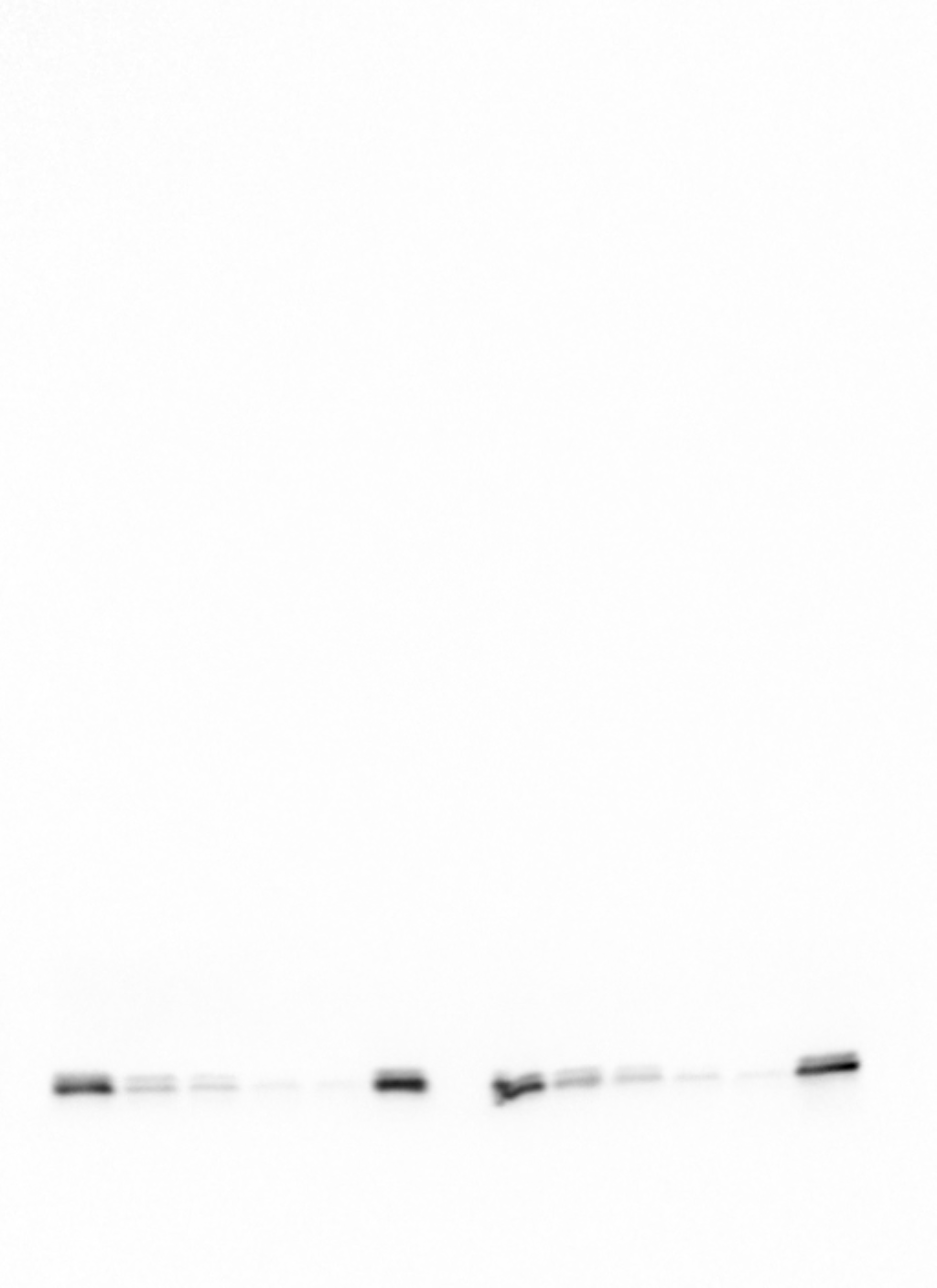

Supplement: Supplementary file 9 — Source data Fig. 4 [file 44321_2024_60_MOESM9_ESM.zip › Figure 4/4B/88T/Western phoCDK1/1 1st phoCDK1 10.4 _Ch.tif]

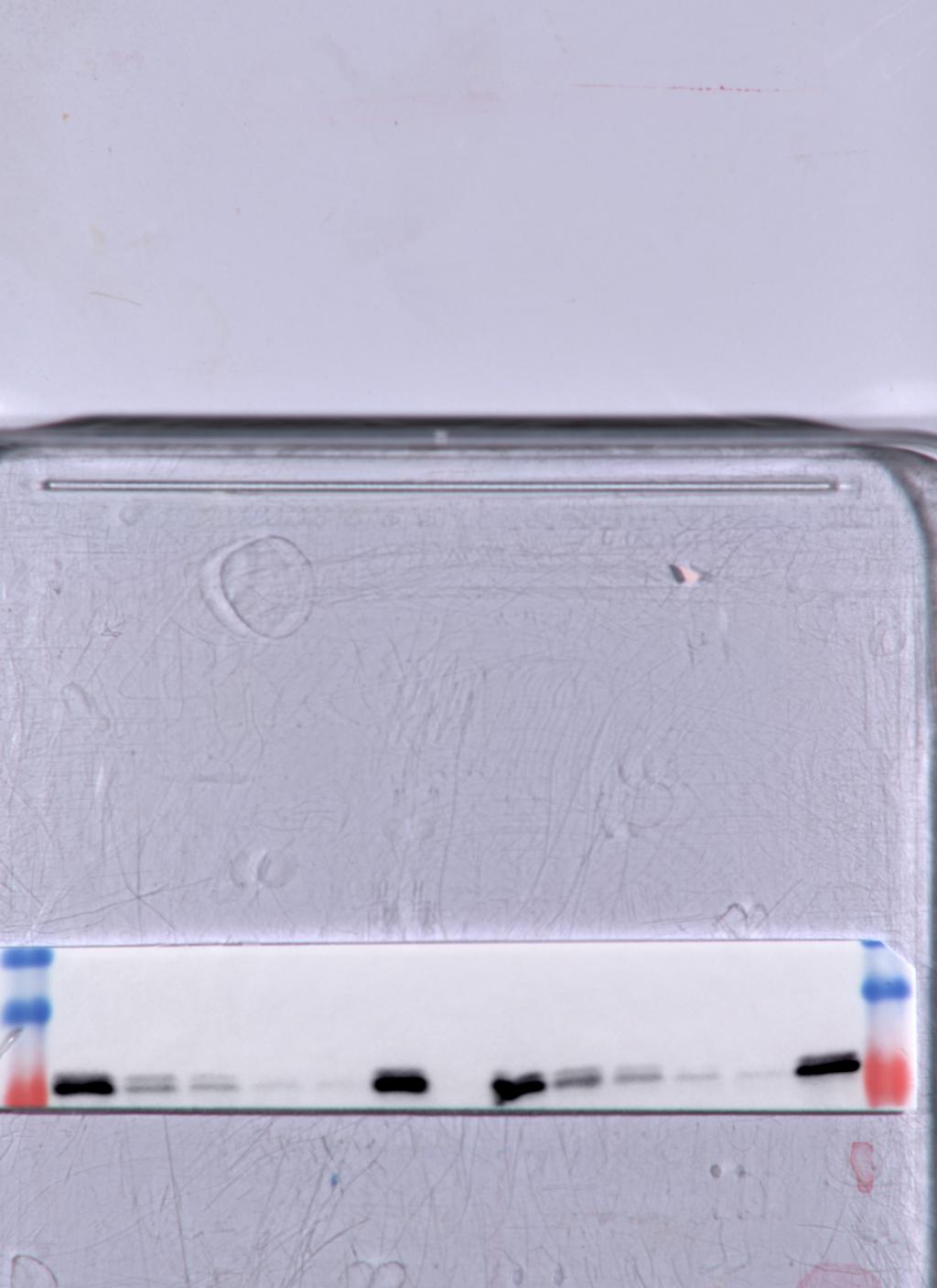

Supplement: Supplementary file 9 — Source data Fig. 4 [file 44321_2024_60_MOESM9_ESM.zip › Figure 4/4B/88T/Western phoCDK1/1 1st phoCDK1 10.4 _Ch+Marker.jpg]

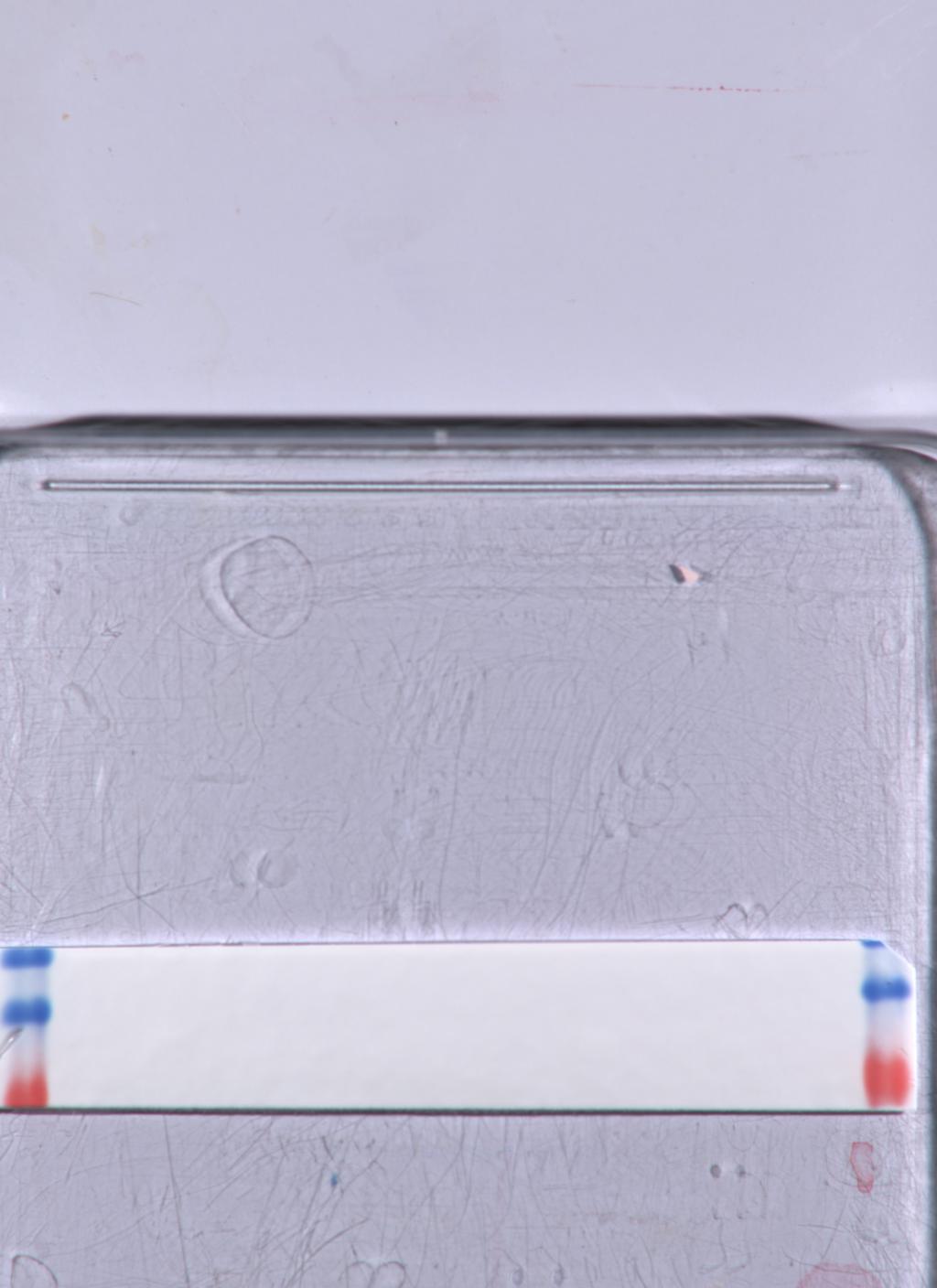

Supplement: Supplementary file 9 — Source data Fig. 4 [file 44321_2024_60_MOESM9_ESM.zip › Figure 4/4B/88T/Western phoCDK1/1 1st phoCDK1 10.4 _Ch-Marker.jpg]

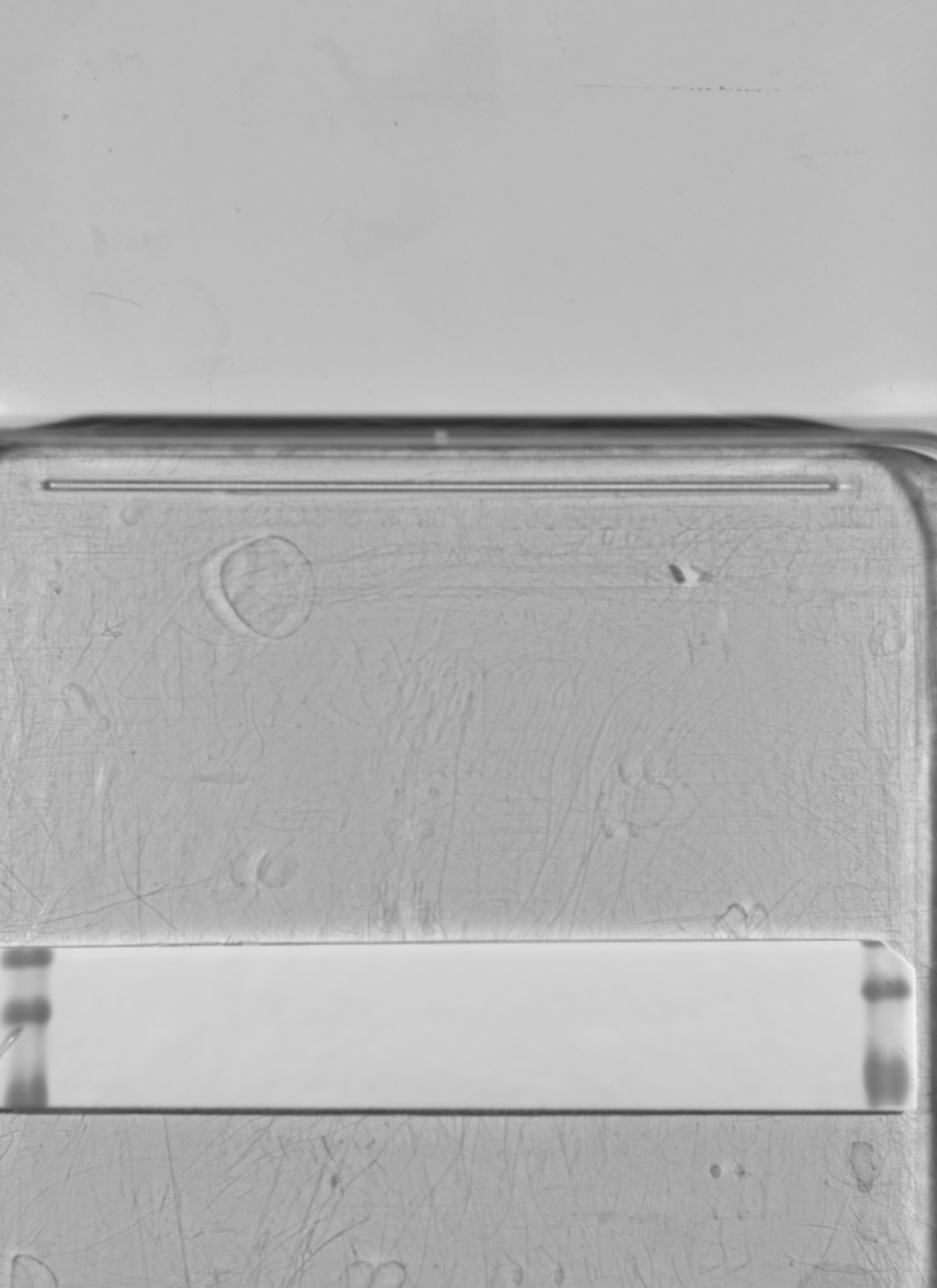

Supplement: Supplementary file 9 — Source data Fig. 4 [file 44321_2024_60_MOESM9_ESM.zip › Figure 4/4B/88T/Western phoCDK1/1 1st phoCDK1 10.4 _Ch-Marker.tif]

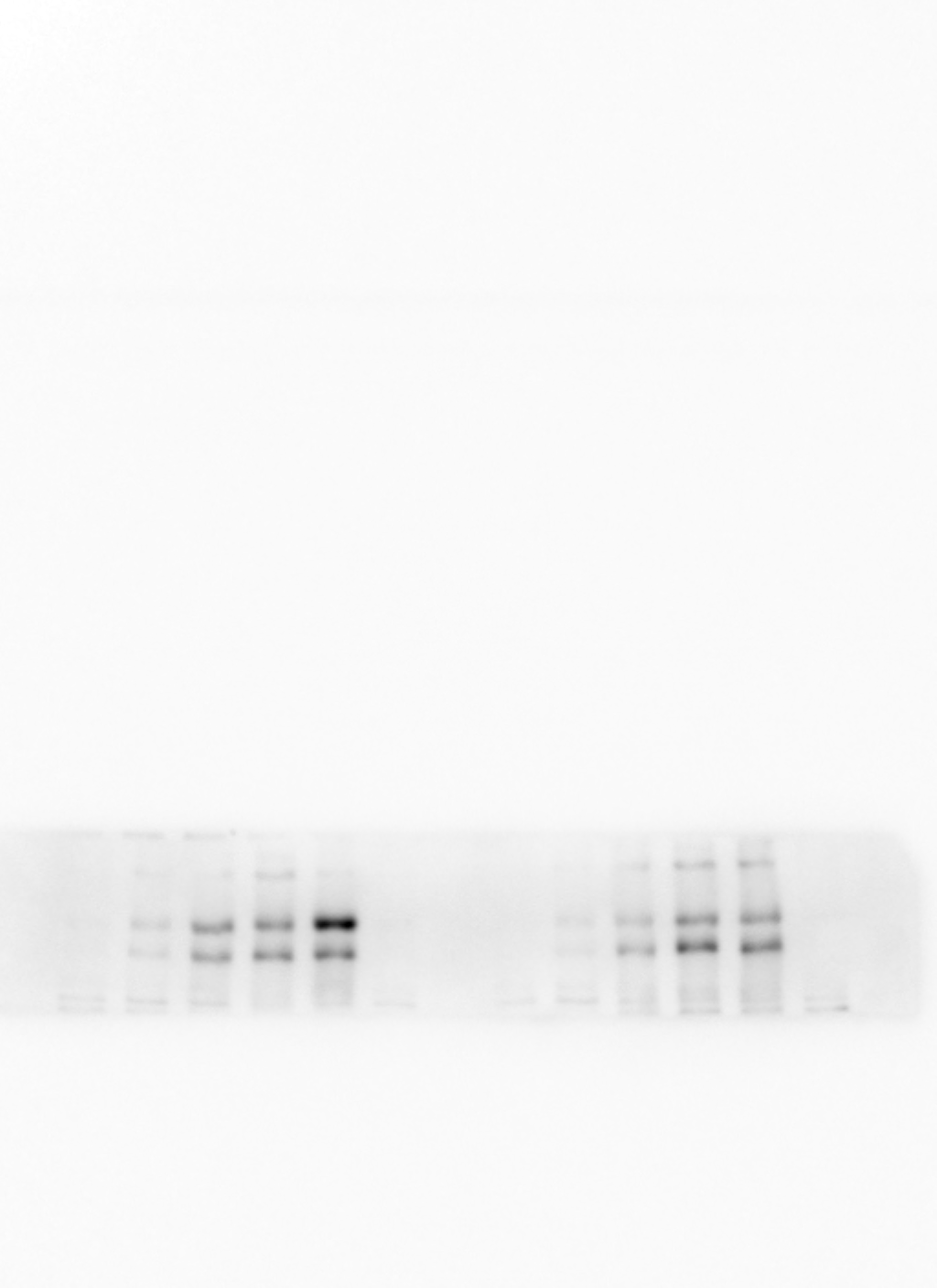

Supplement: Supplementary file 9 — Source data Fig. 4 [file 44321_2024_60_MOESM9_ESM.zip › Figure 4/4B/88T/Western phoPRKDC/1 1st phoPRK 1.1 _Ch.tif]

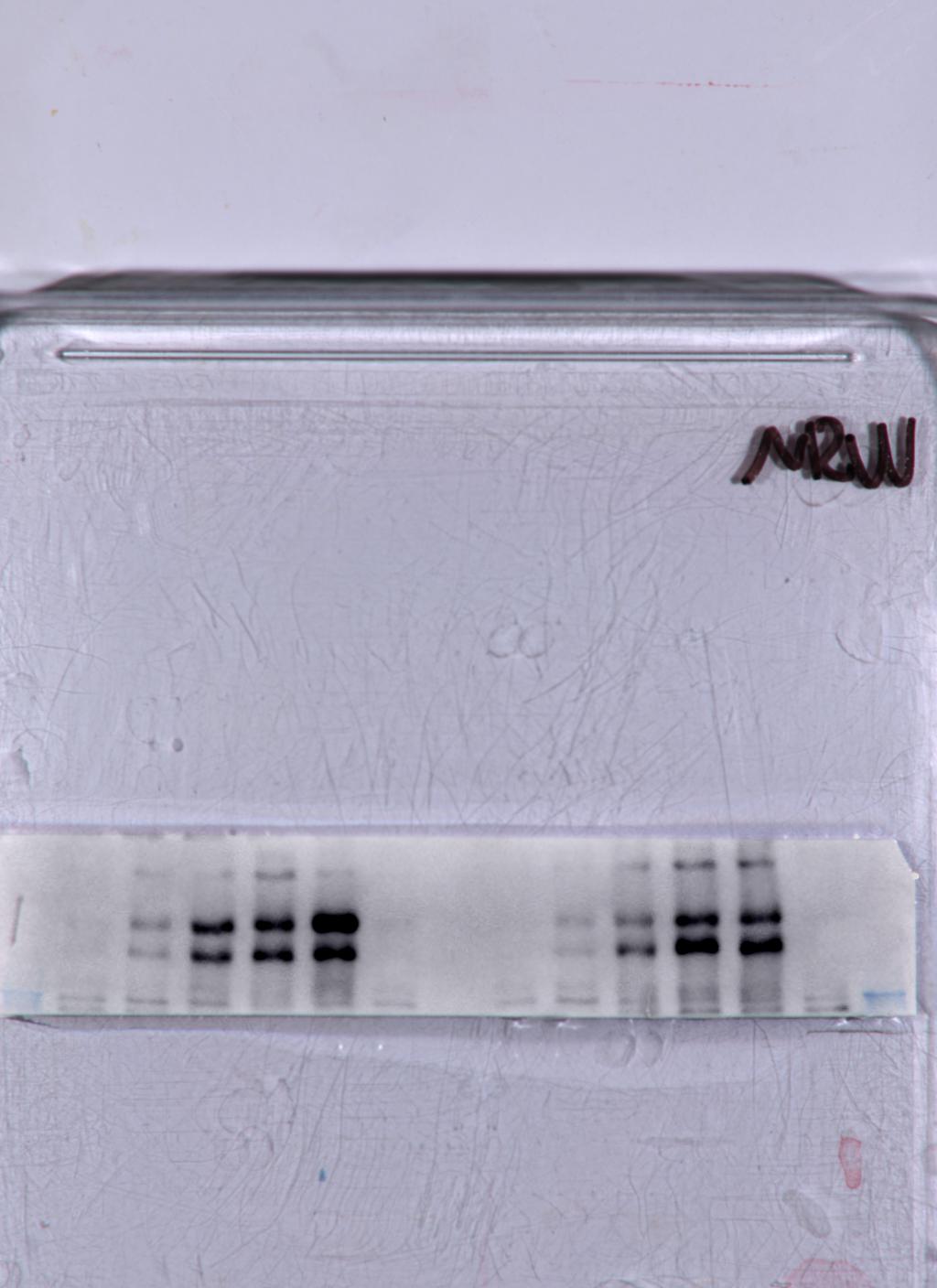

Supplement: Supplementary file 9 — Source data Fig. 4 [file 44321_2024_60_MOESM9_ESM.zip › Figure 4/4B/88T/Western phoPRKDC/1 1st phoPRK 1.1 _Ch+Marker.jpg]

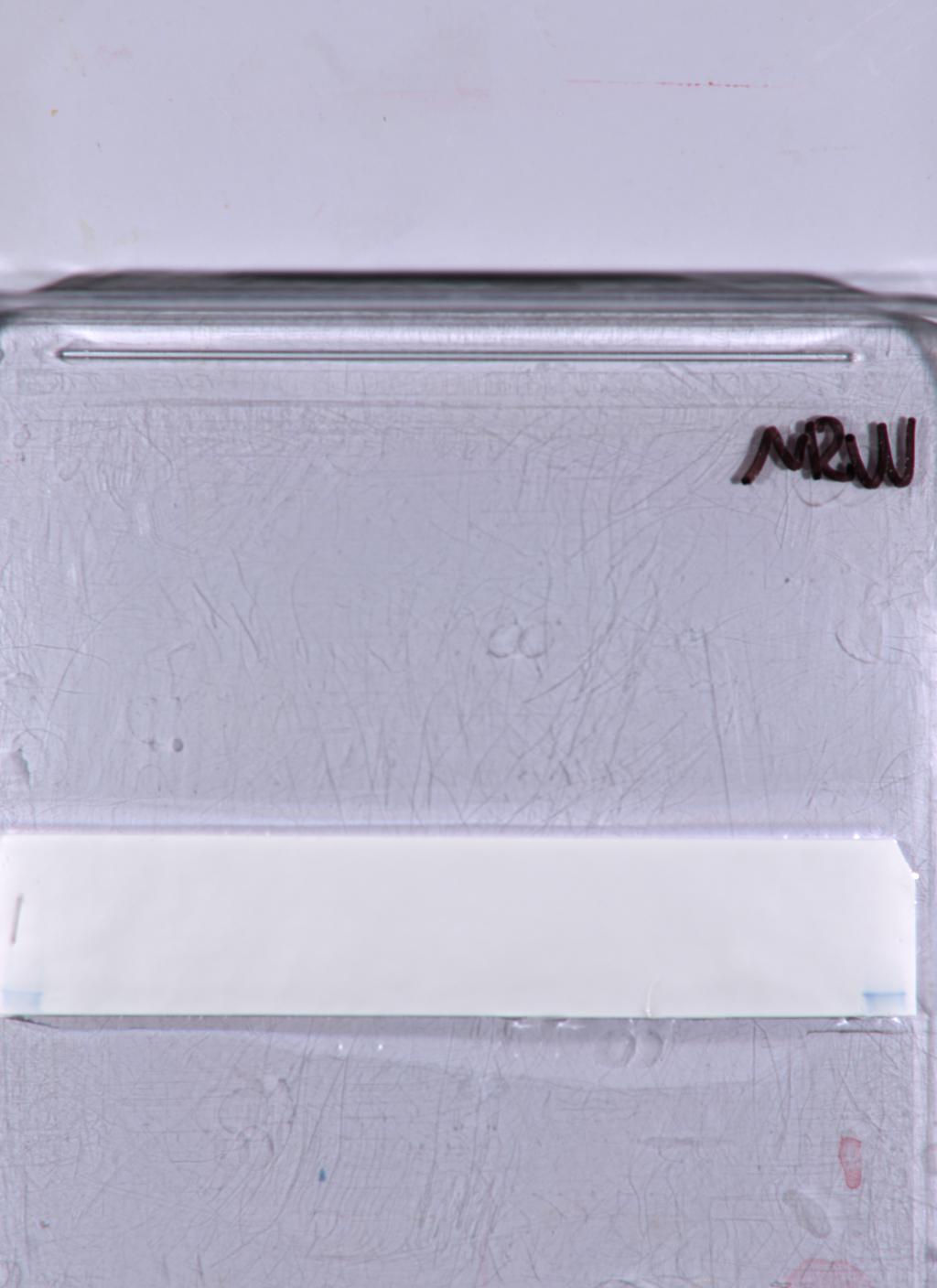

Supplement: Supplementary file 9 — Source data Fig. 4 [file 44321_2024_60_MOESM9_ESM.zip › Figure 4/4B/88T/Western phoPRKDC/1 1st phoPRK 1.1 _Ch-Marker.jpg]

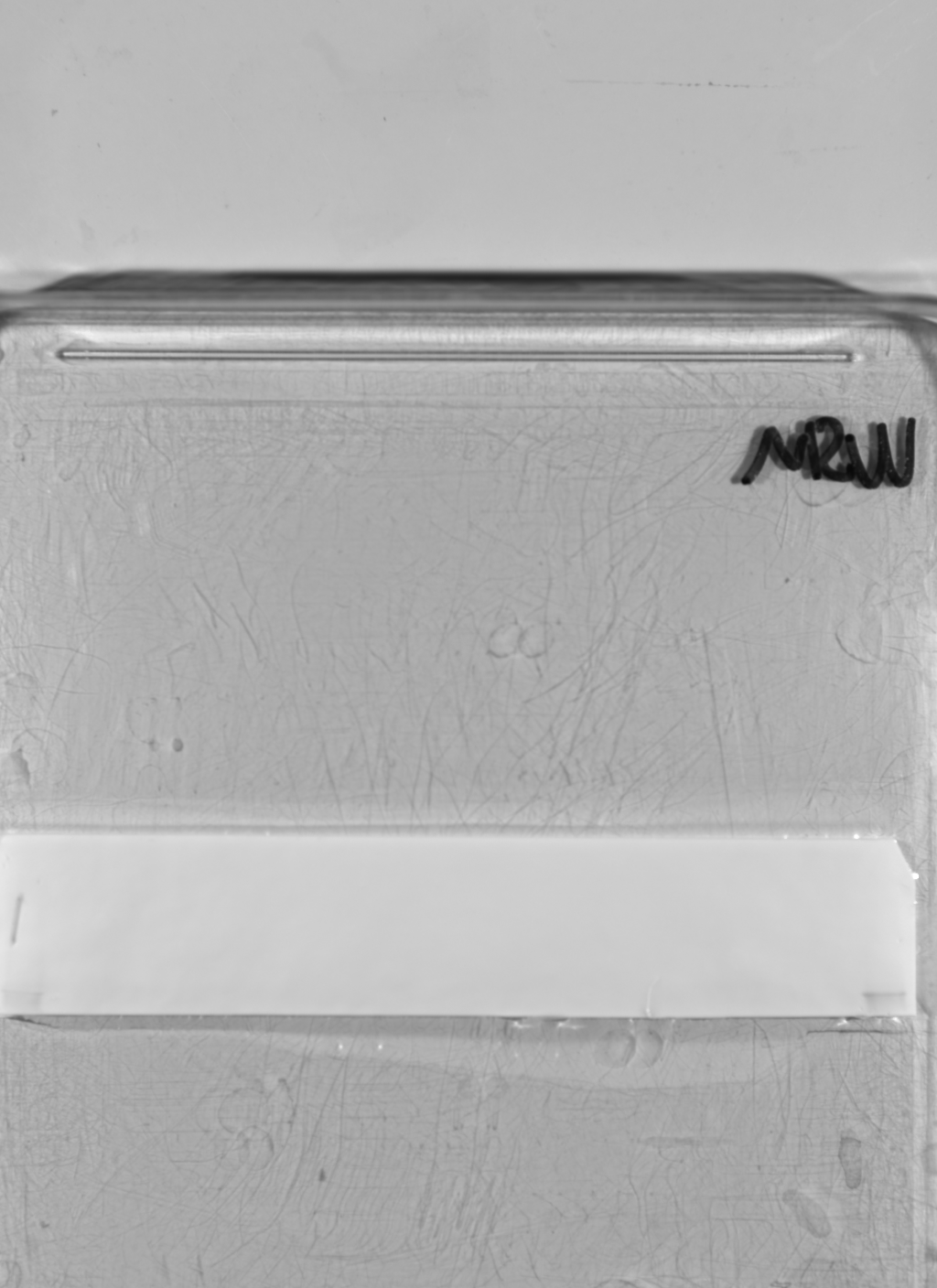

Supplement: Supplementary file 9 — Source data Fig. 4 [file 44321_2024_60_MOESM9_ESM.zip › Figure 4/4B/88T/Western phoPRKDC/1 1st phoPRK 1.1 _Ch-Marker.tif]

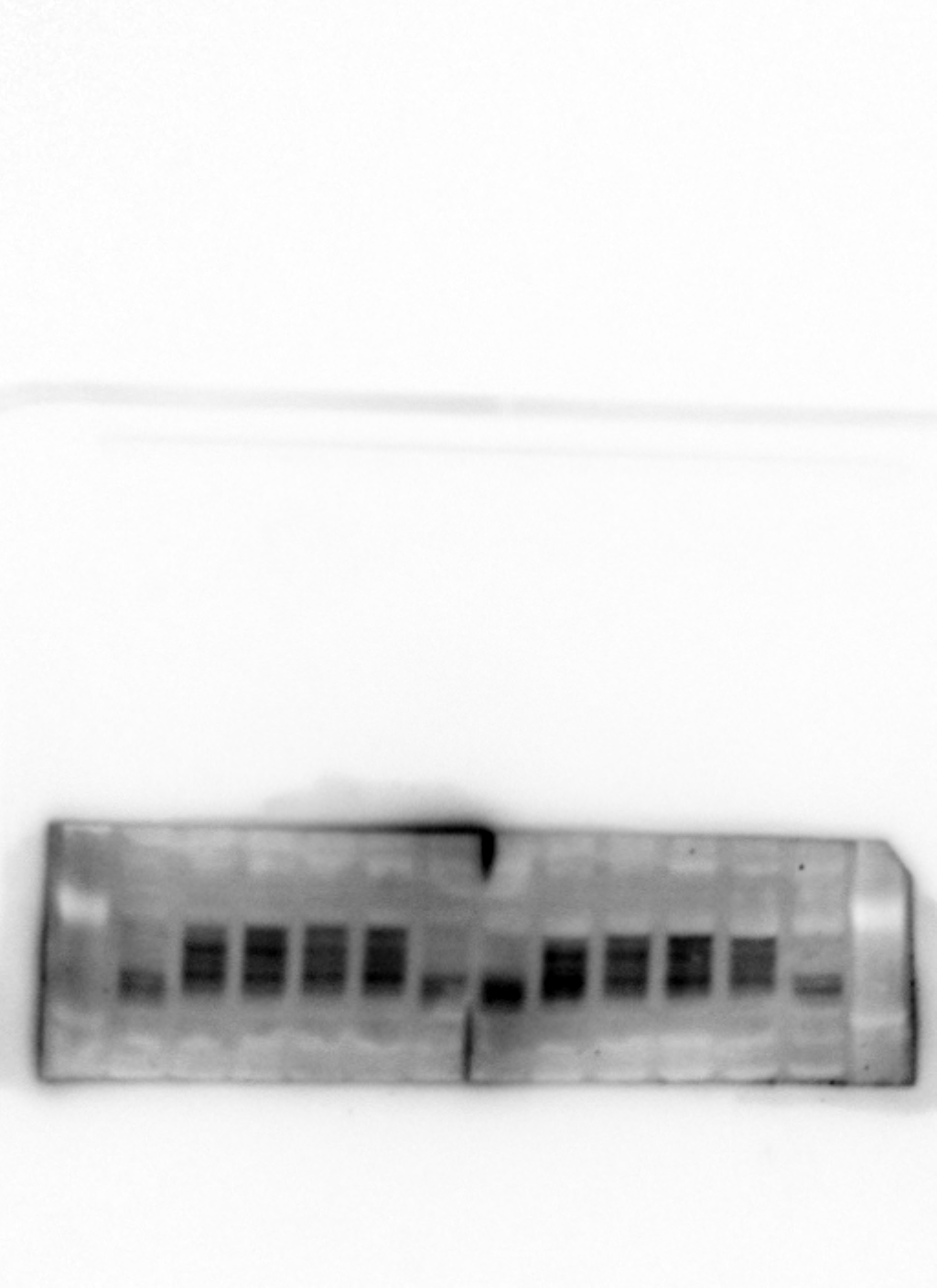

Supplement: Supplementary file 9 — Source data Fig. 4 [file 44321_2024_60_MOESM9_ESM.zip › Figure 4/4B/88T/Western PKMYT1/4 1st PK 20.3 _Ch.tif]

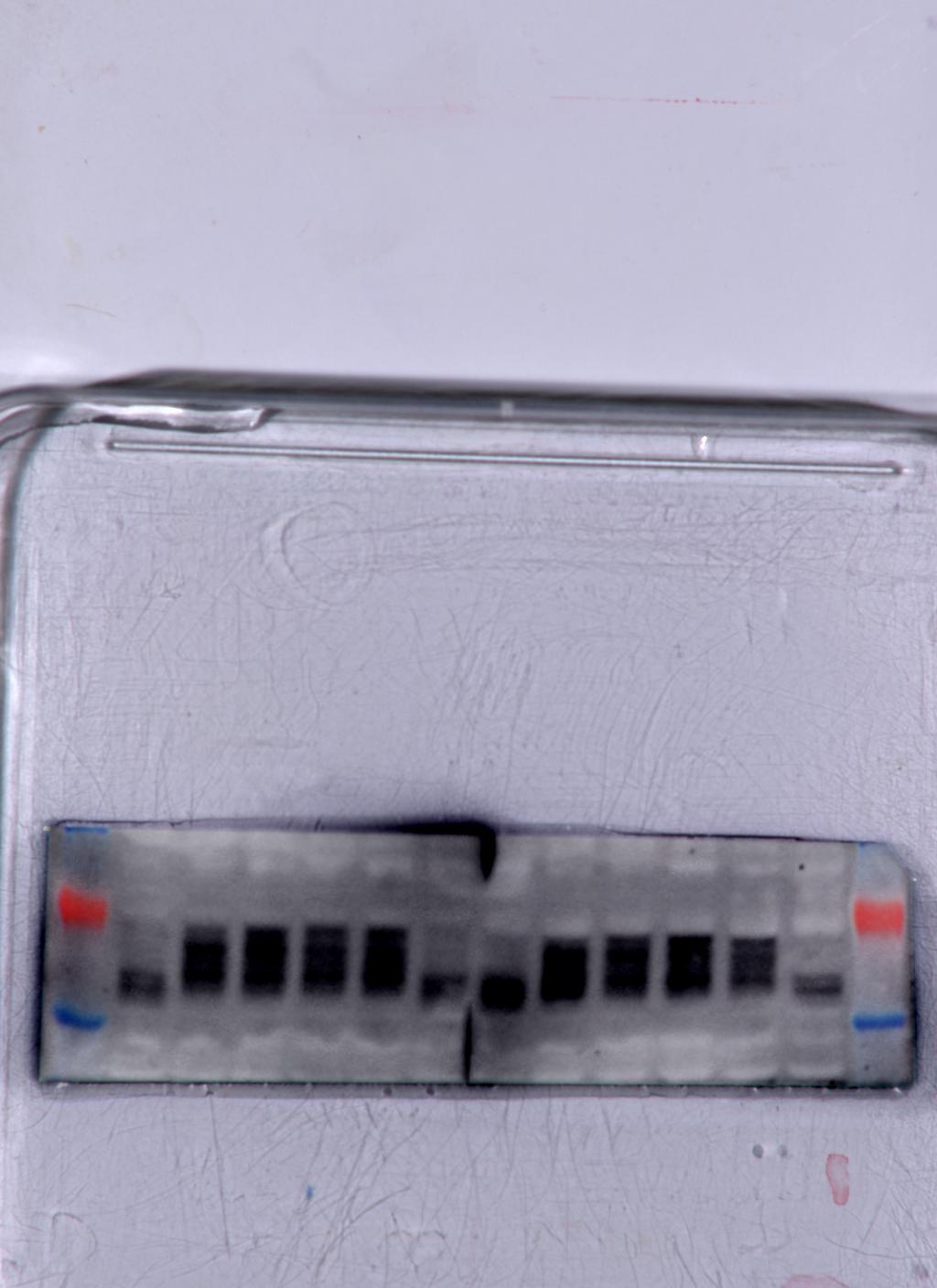

Supplement: Supplementary file 9 — Source data Fig. 4 [file 44321_2024_60_MOESM9_ESM.zip › Figure 4/4B/88T/Western PKMYT1/4 1st PK 20.3 _Ch+Marker.jpg]

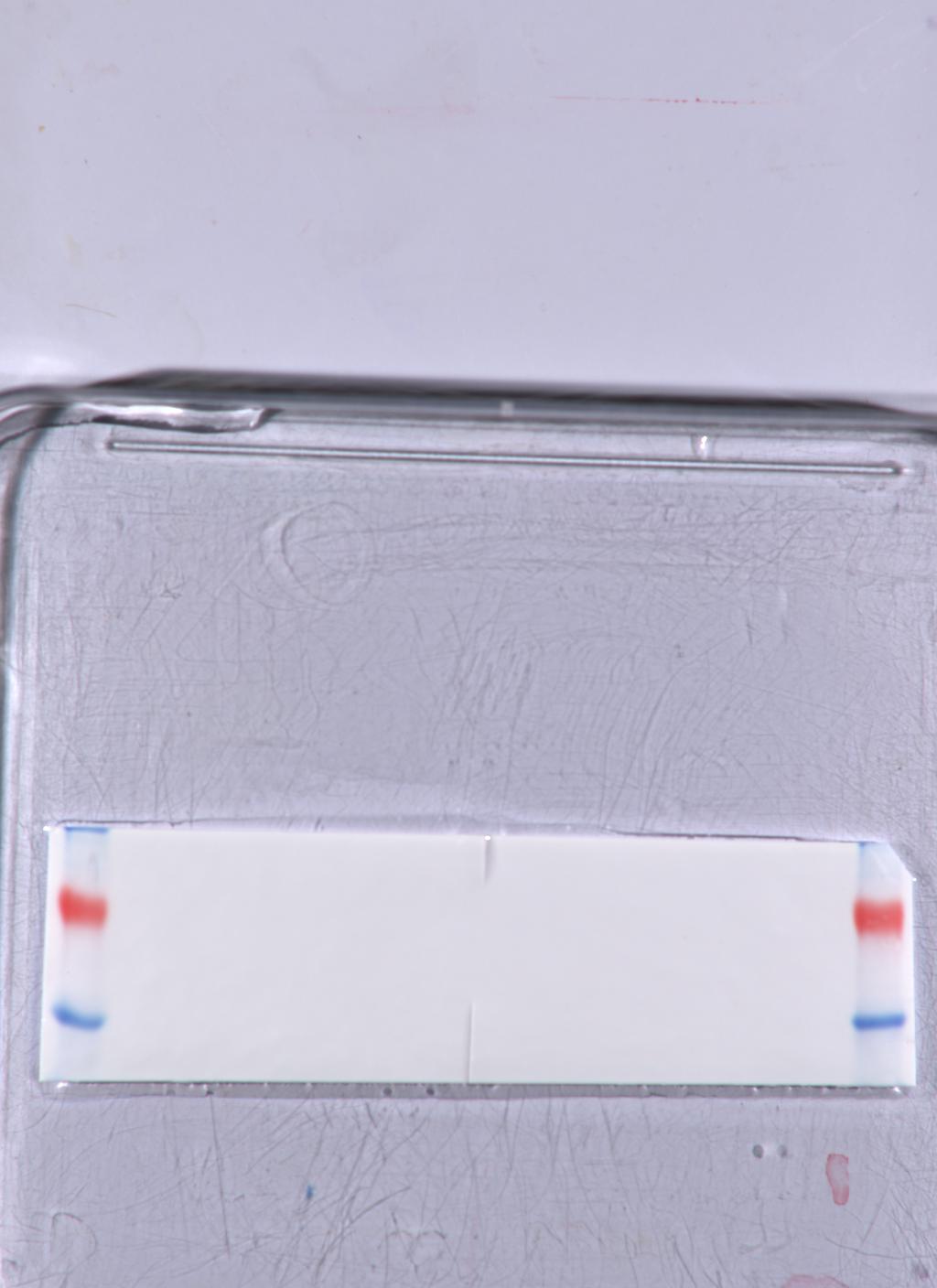

Supplement: Supplementary file 9 — Source data Fig. 4 [file 44321_2024_60_MOESM9_ESM.zip › Figure 4/4B/88T/Western PKMYT1/4 1st PK 20.3 _Ch-Marker.jpg]

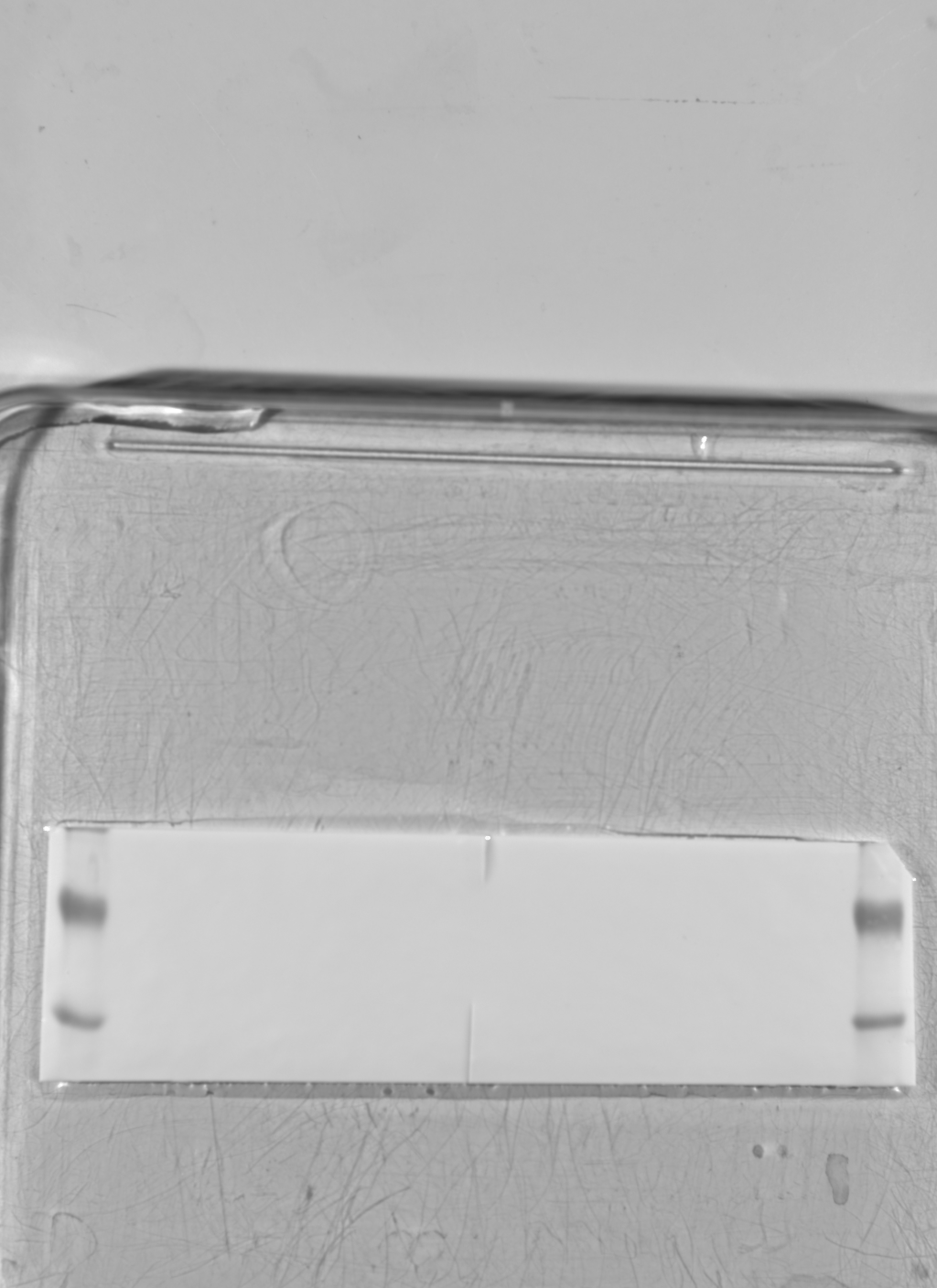

Supplement: Supplementary file 9 — Source data Fig. 4 [file 44321_2024_60_MOESM9_ESM.zip › Figure 4/4B/88T/Western PKMYT1/4 1st PK 20.3 _Ch-Marker.tif]

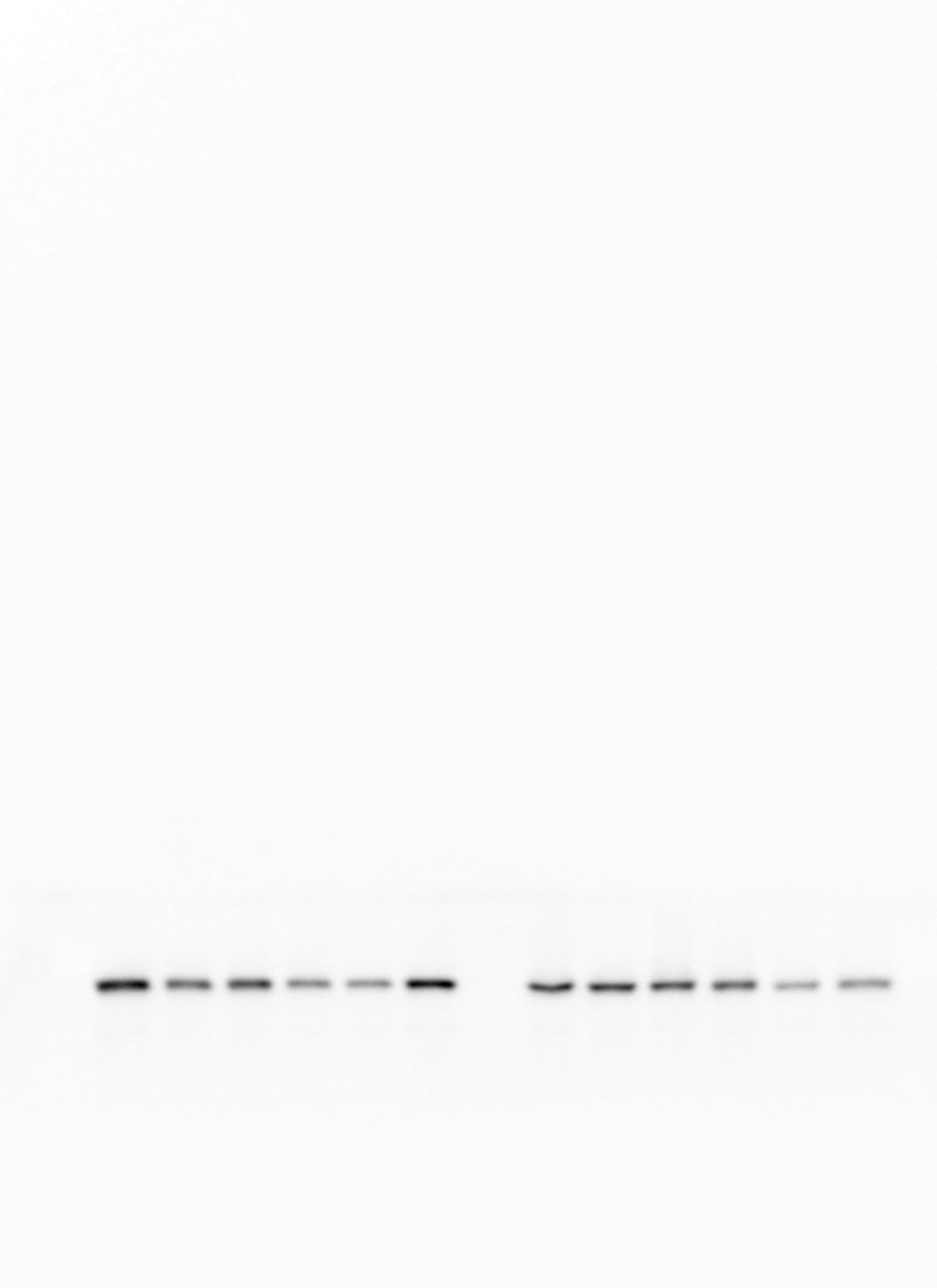

Supplement: Supplementary file 9 — Source data Fig. 4 [file 44321_2024_60_MOESM9_ESM.zip › Figure 4/4B/88T/Western PLK1/1 2nd PLK 0.9 _Ch.tif]

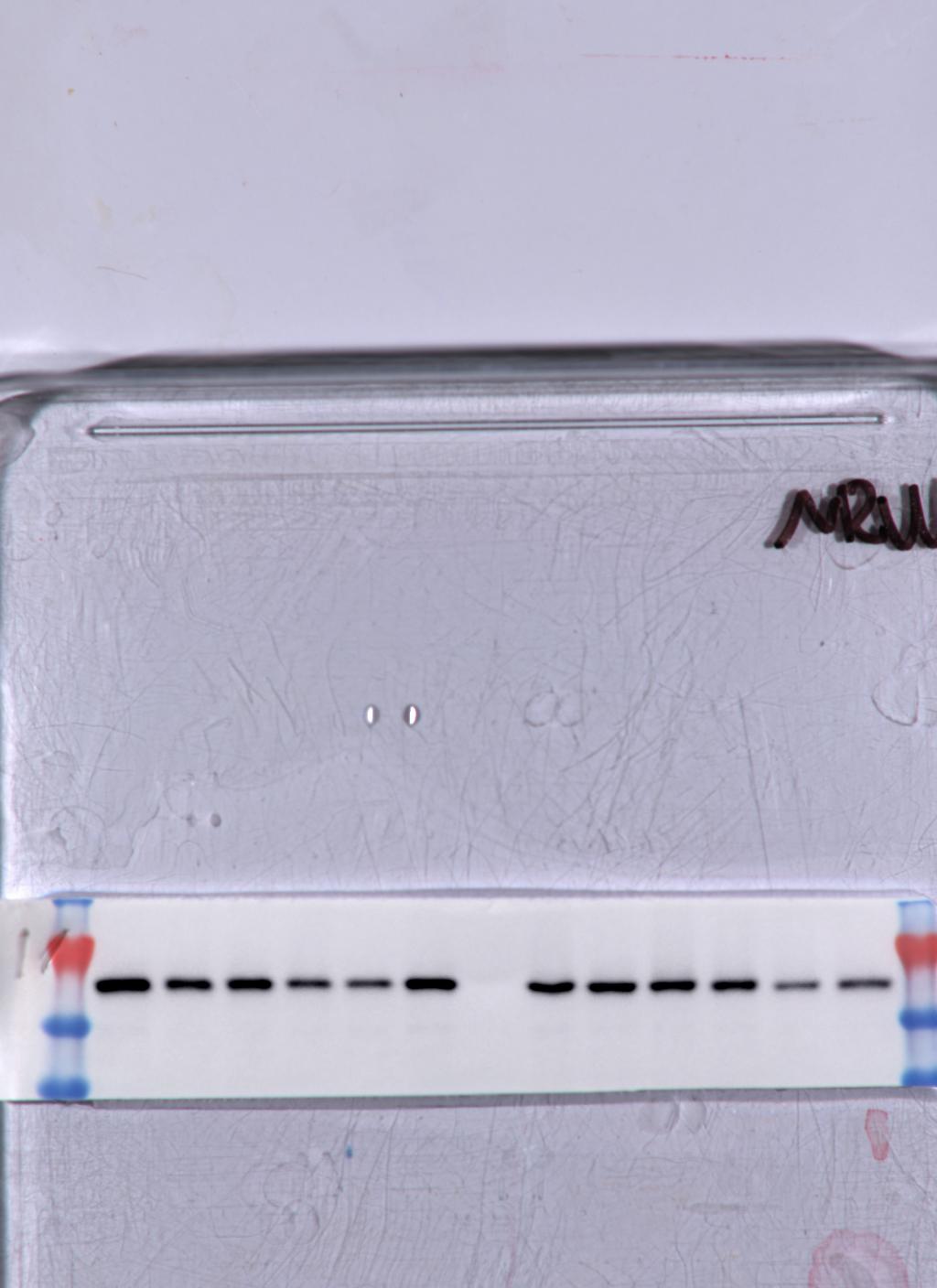

Supplement: Supplementary file 9 — Source data Fig. 4 [file 44321_2024_60_MOESM9_ESM.zip › Figure 4/4B/88T/Western PLK1/1 2nd PLK 0.9 _Ch+Marker.jpg]

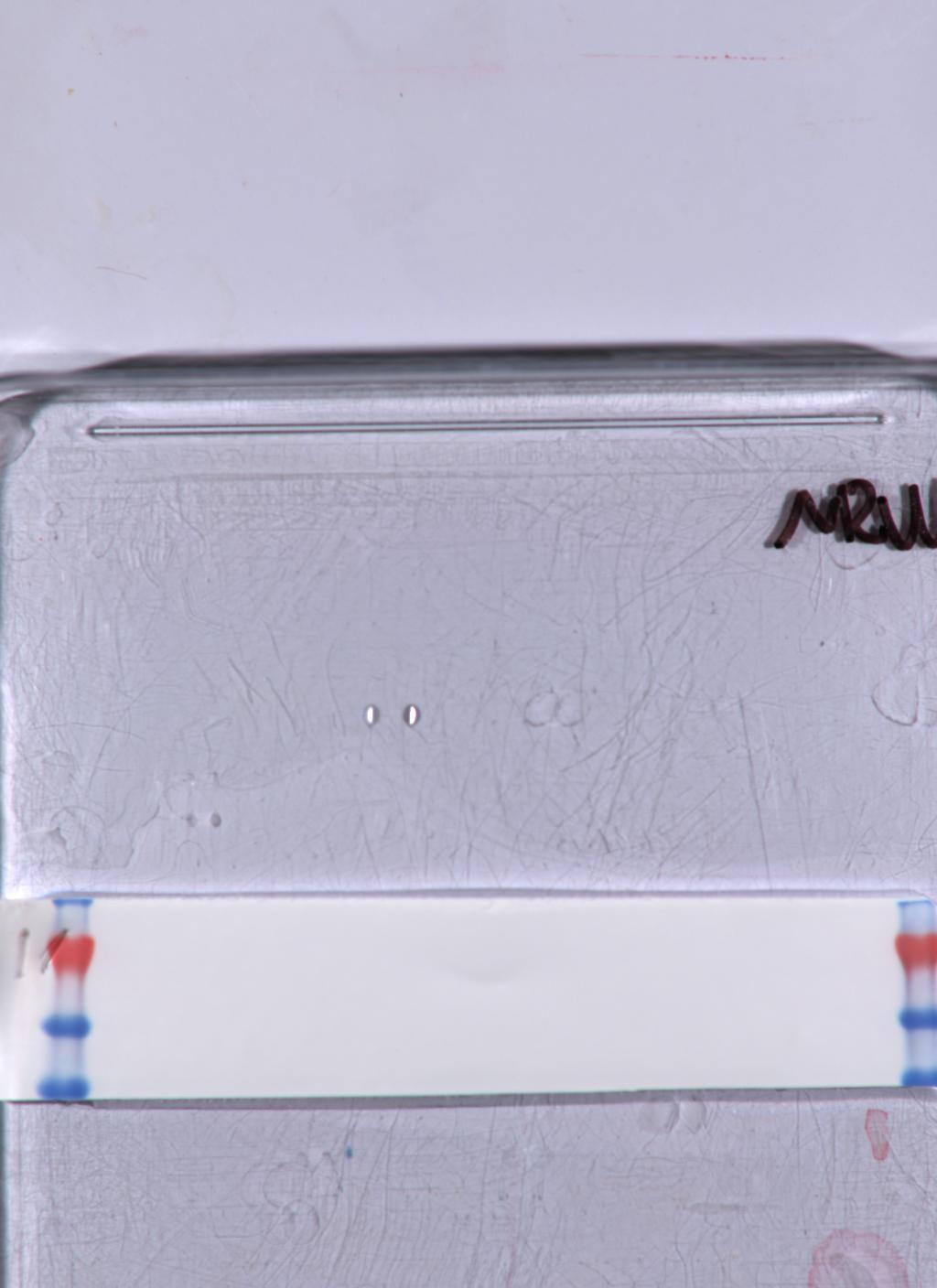

Supplement: Supplementary file 9 — Source data Fig. 4 [file 44321_2024_60_MOESM9_ESM.zip › Figure 4/4B/88T/Western PLK1/1 2nd PLK 0.9 _Ch-Marker.jpg]

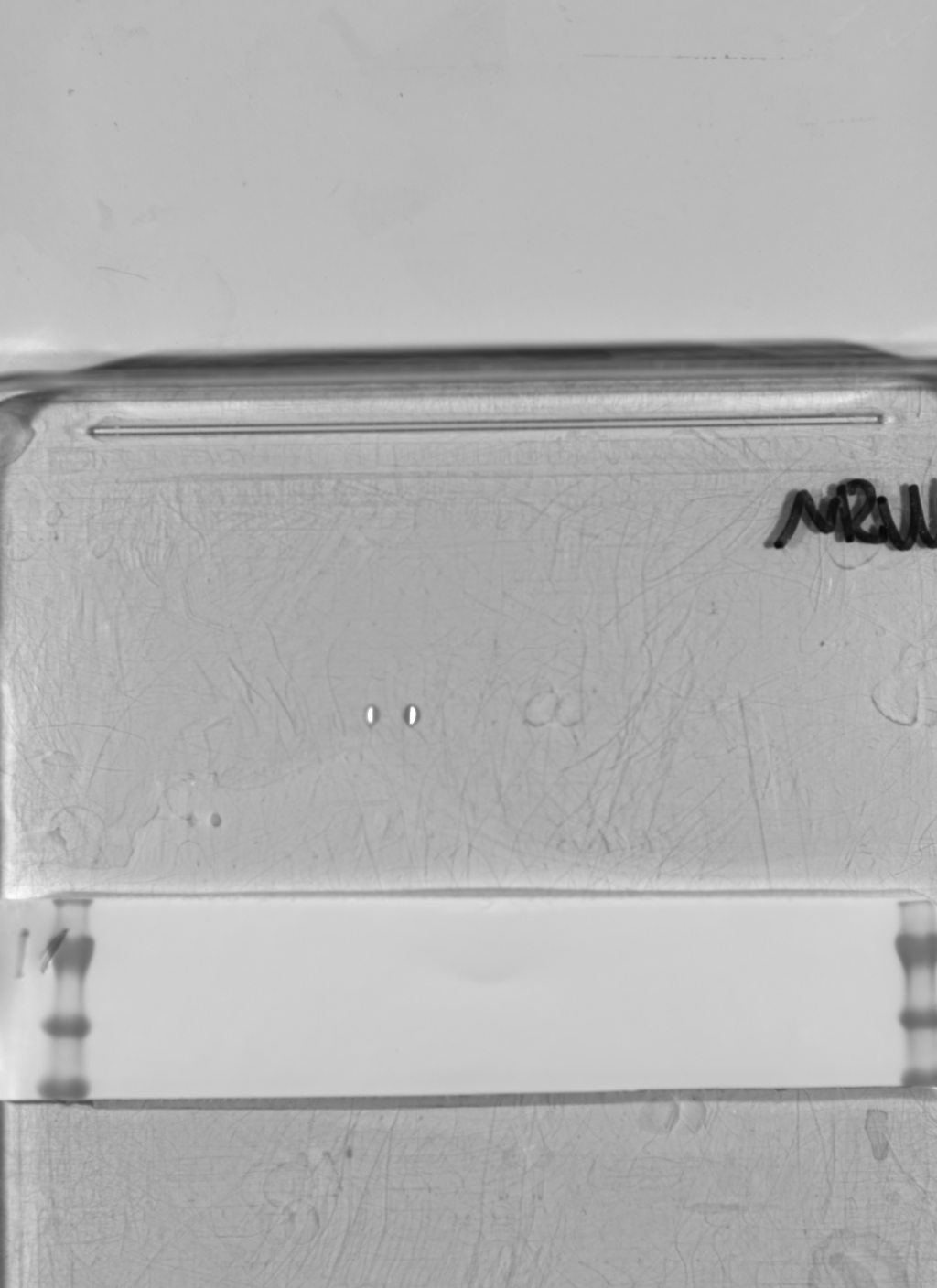

Supplement: Supplementary file 9 — Source data Fig. 4 [file 44321_2024_60_MOESM9_ESM.zip › Figure 4/4B/88T/Western PLK1/1 2nd PLK 0.9 _Ch-Marker.tif]

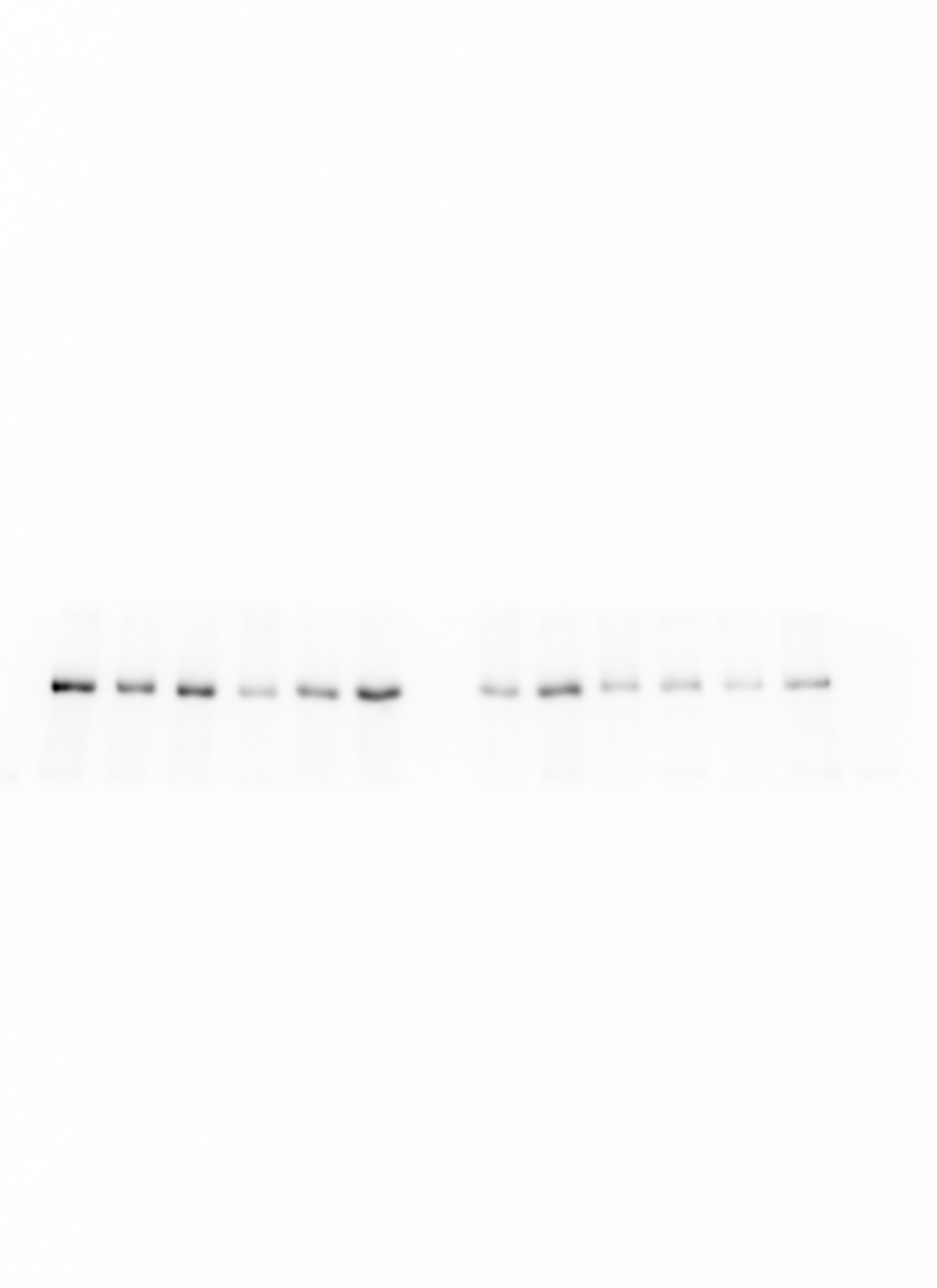

Supplement: Supplementary file 9 — Source data Fig. 4 [file 44321_2024_60_MOESM9_ESM.zip › Figure 4/4B/88T/Western PRKDC/1 1st PRK 0.1 _Ch.tif]

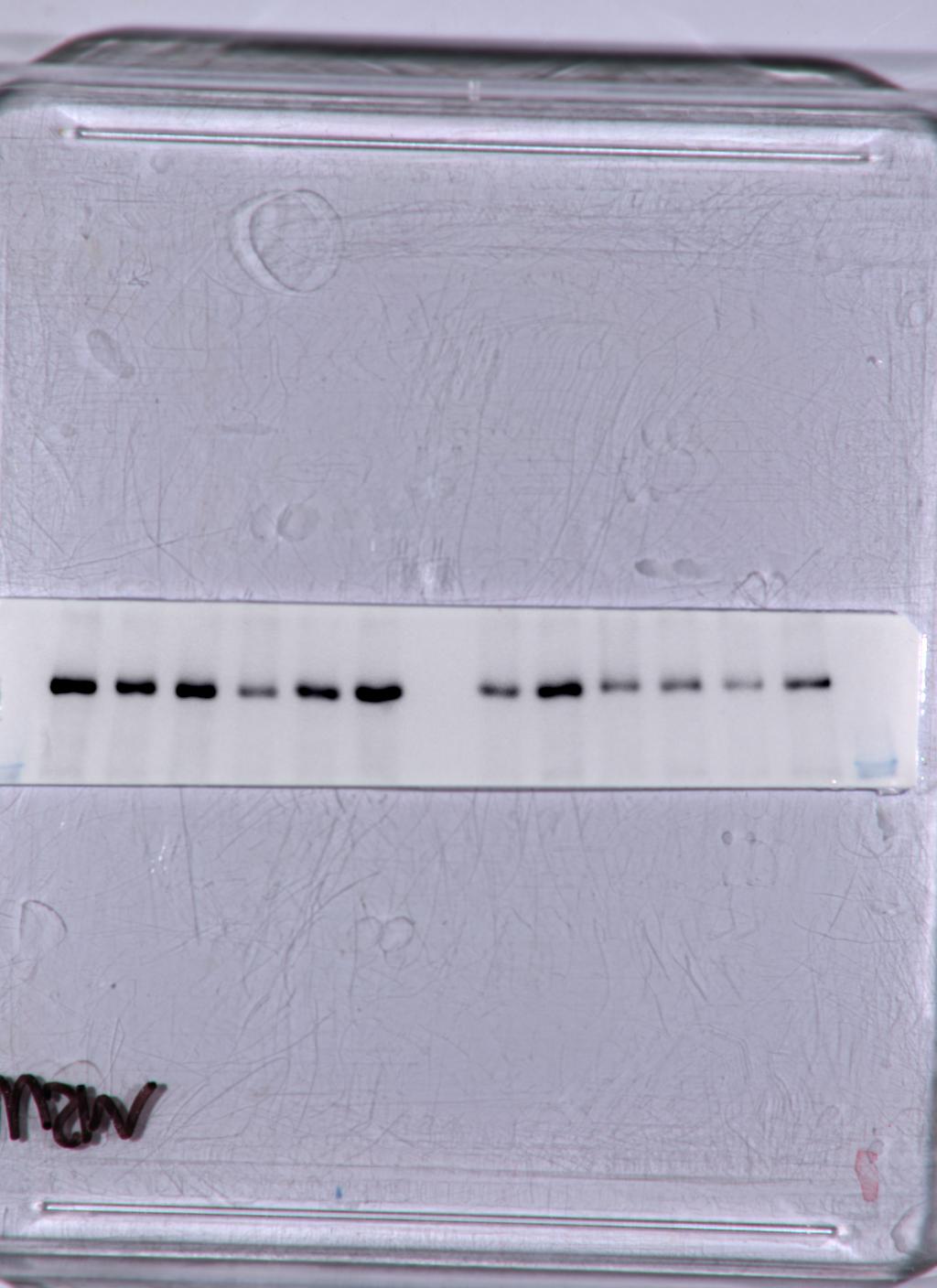

Supplement: Supplementary file 9 — Source data Fig. 4 [file 44321_2024_60_MOESM9_ESM.zip › Figure 4/4B/88T/Western PRKDC/1 1st PRK 0.1 _Ch+Marker.jpg]

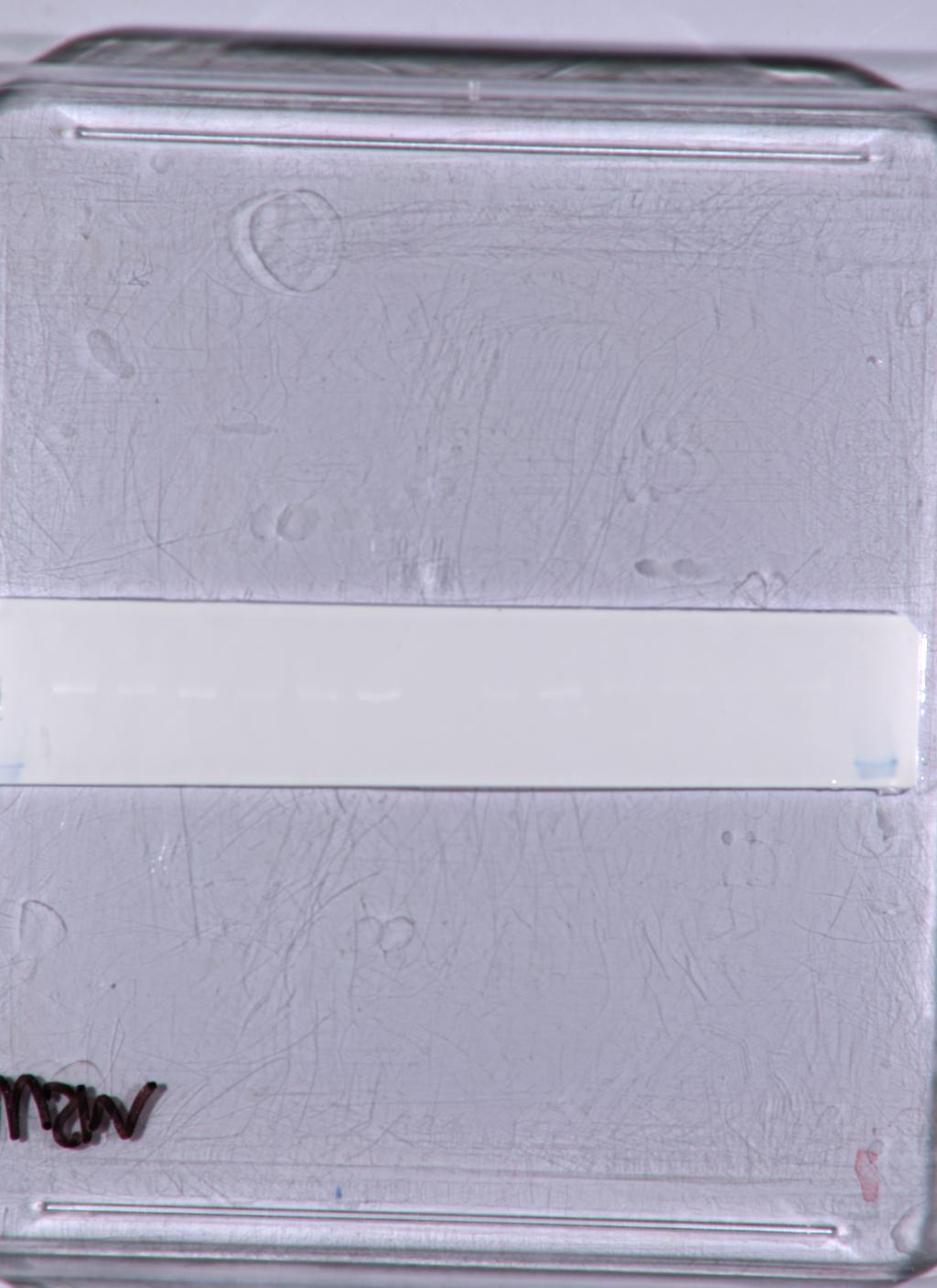

Supplement: Supplementary file 9 — Source data Fig. 4 [file 44321_2024_60_MOESM9_ESM.zip › Figure 4/4B/88T/Western PRKDC/1 1st PRK 0.1 _Ch-Marker.jpg]

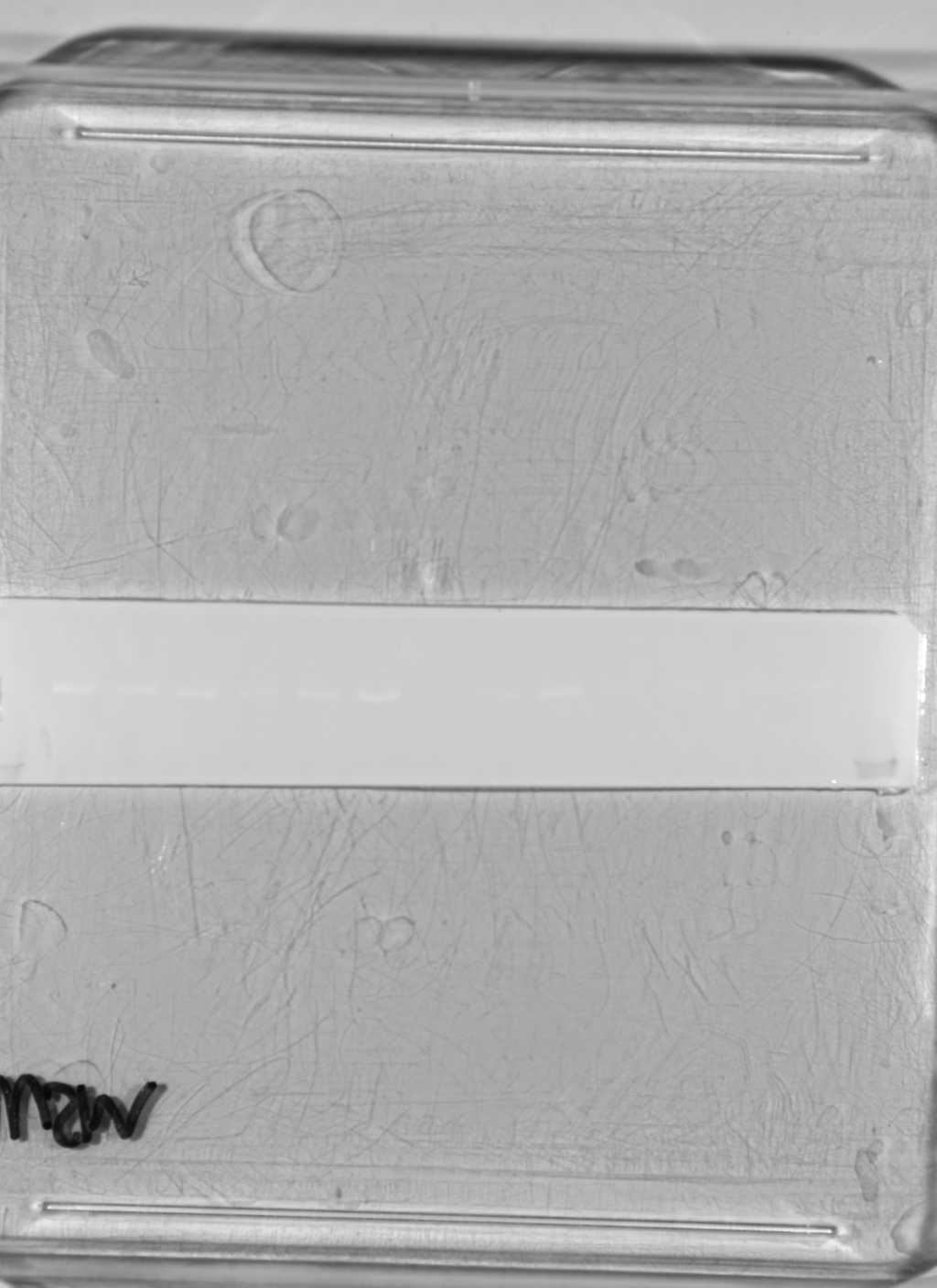

Supplement: Supplementary file 9 — Source data Fig. 4 [file 44321_2024_60_MOESM9_ESM.zip › Figure 4/4B/88T/Western PRKDC/1 1st PRK 0.1 _Ch-Marker.tif]

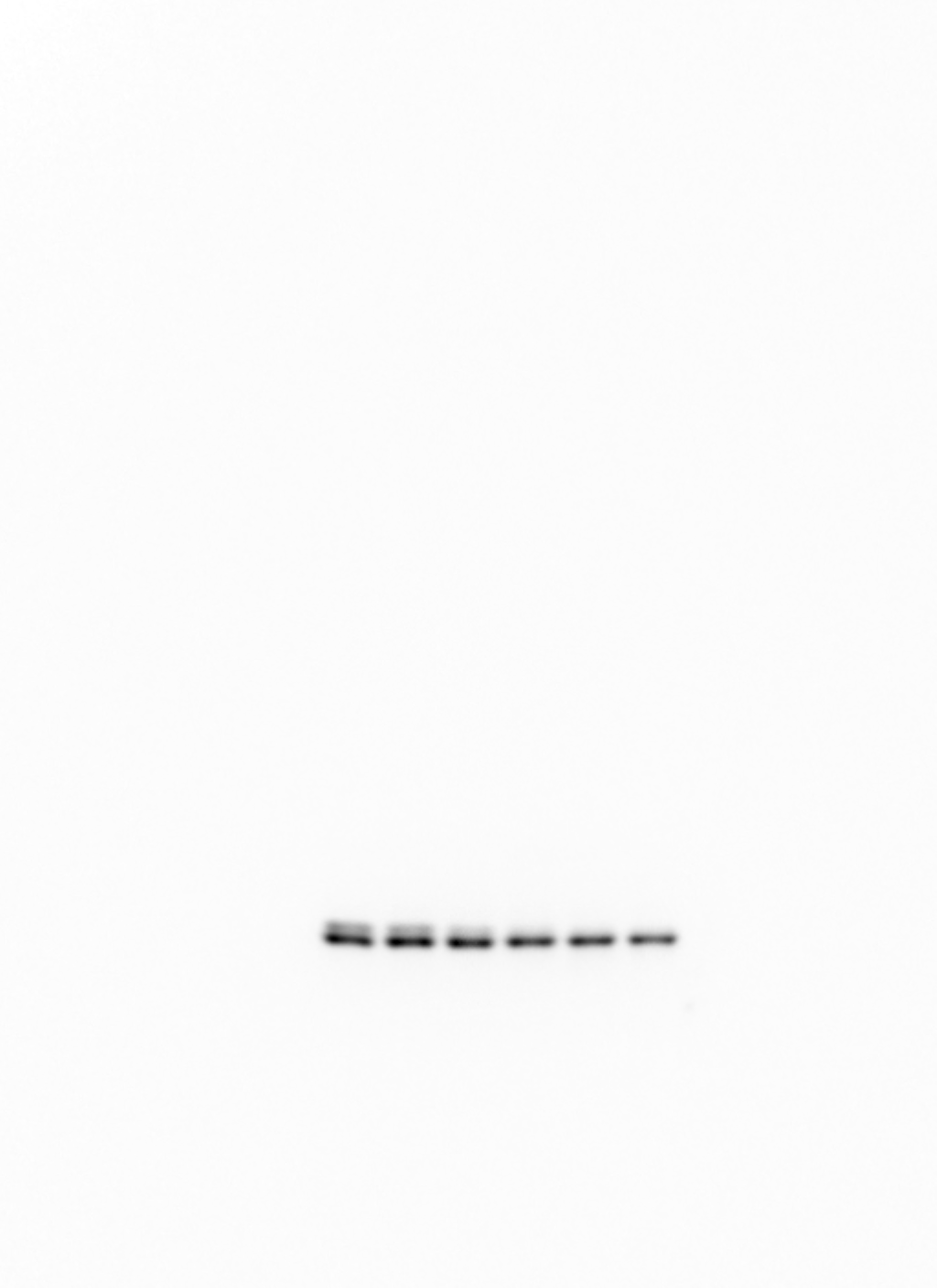

Supplement: Supplementary file 9 — Source data Fig. 4 [file 44321_2024_60_MOESM9_ESM.zip › Figure 4/4B/CN1/Western CDK 0.7S/1 5th CDK 0.7S _Ch.tif]

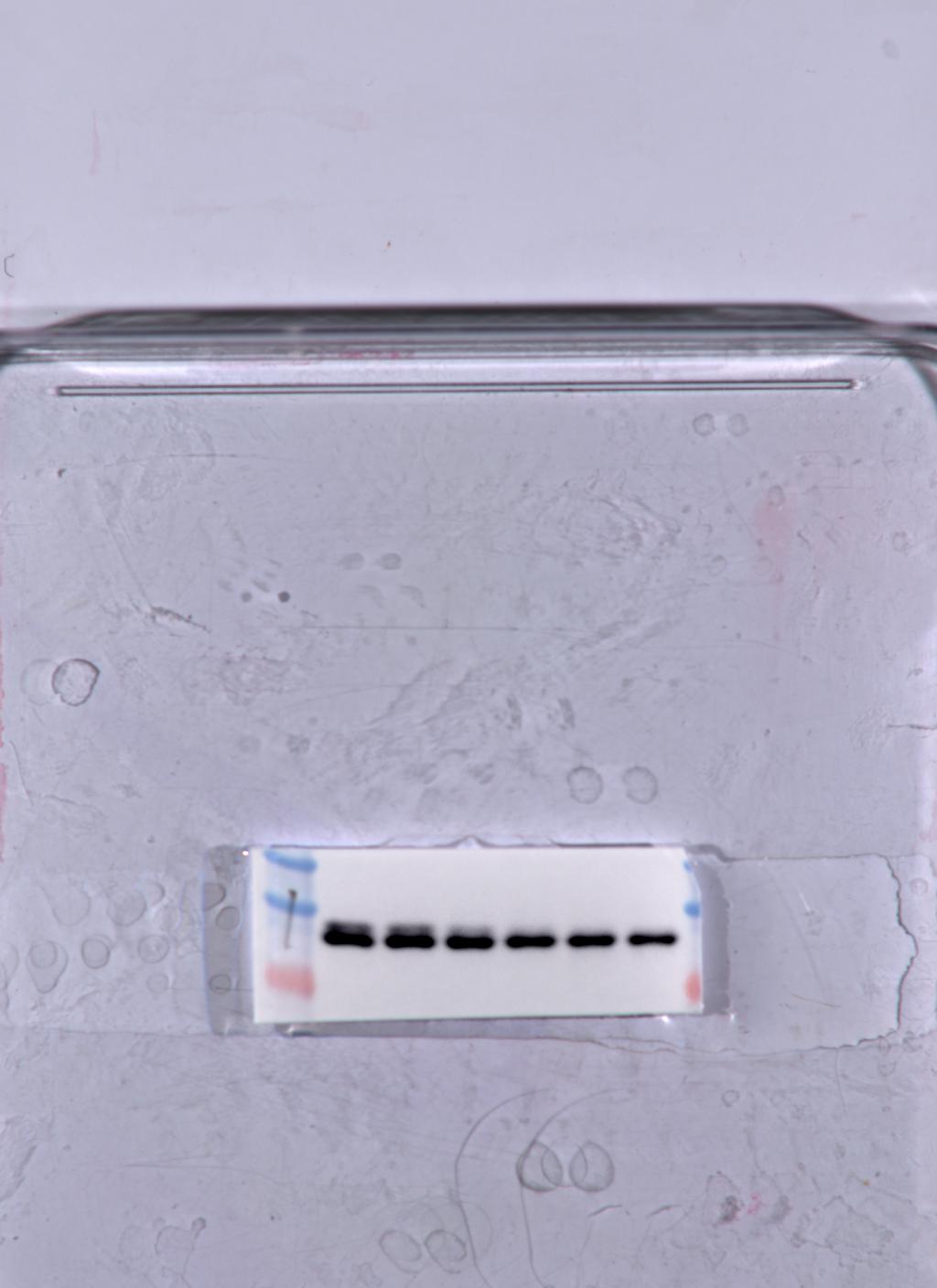

Supplement: Supplementary file 9 — Source data Fig. 4 [file 44321_2024_60_MOESM9_ESM.zip › Figure 4/4B/CN1/Western CDK 0.7S/1 5th CDK 0.7S _Ch+Marker.jpg]

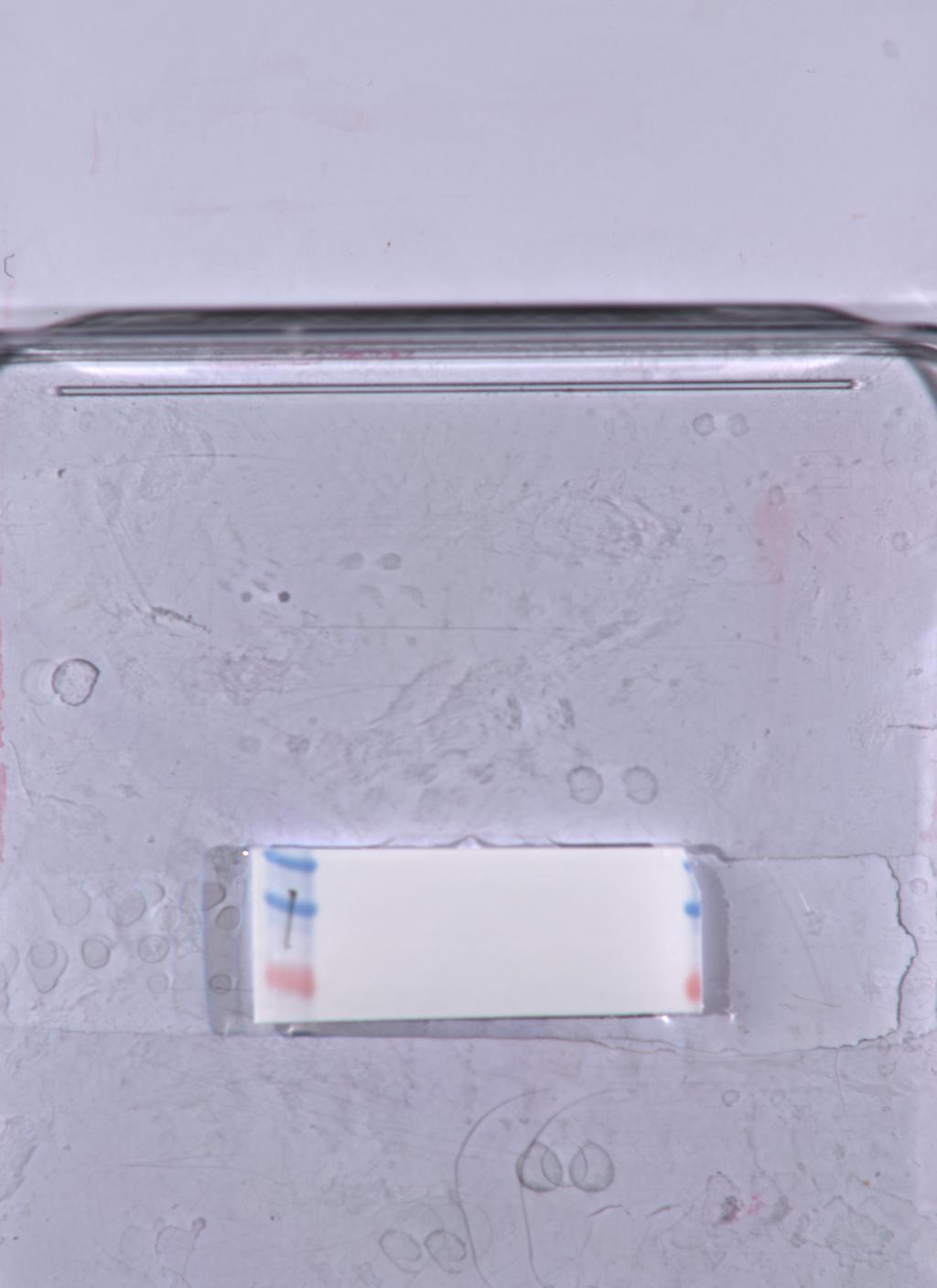

Supplement: Supplementary file 9 — Source data Fig. 4 [file 44321_2024_60_MOESM9_ESM.zip › Figure 4/4B/CN1/Western CDK 0.7S/1 5th CDK 0.7S _Ch-Marker.jpg]

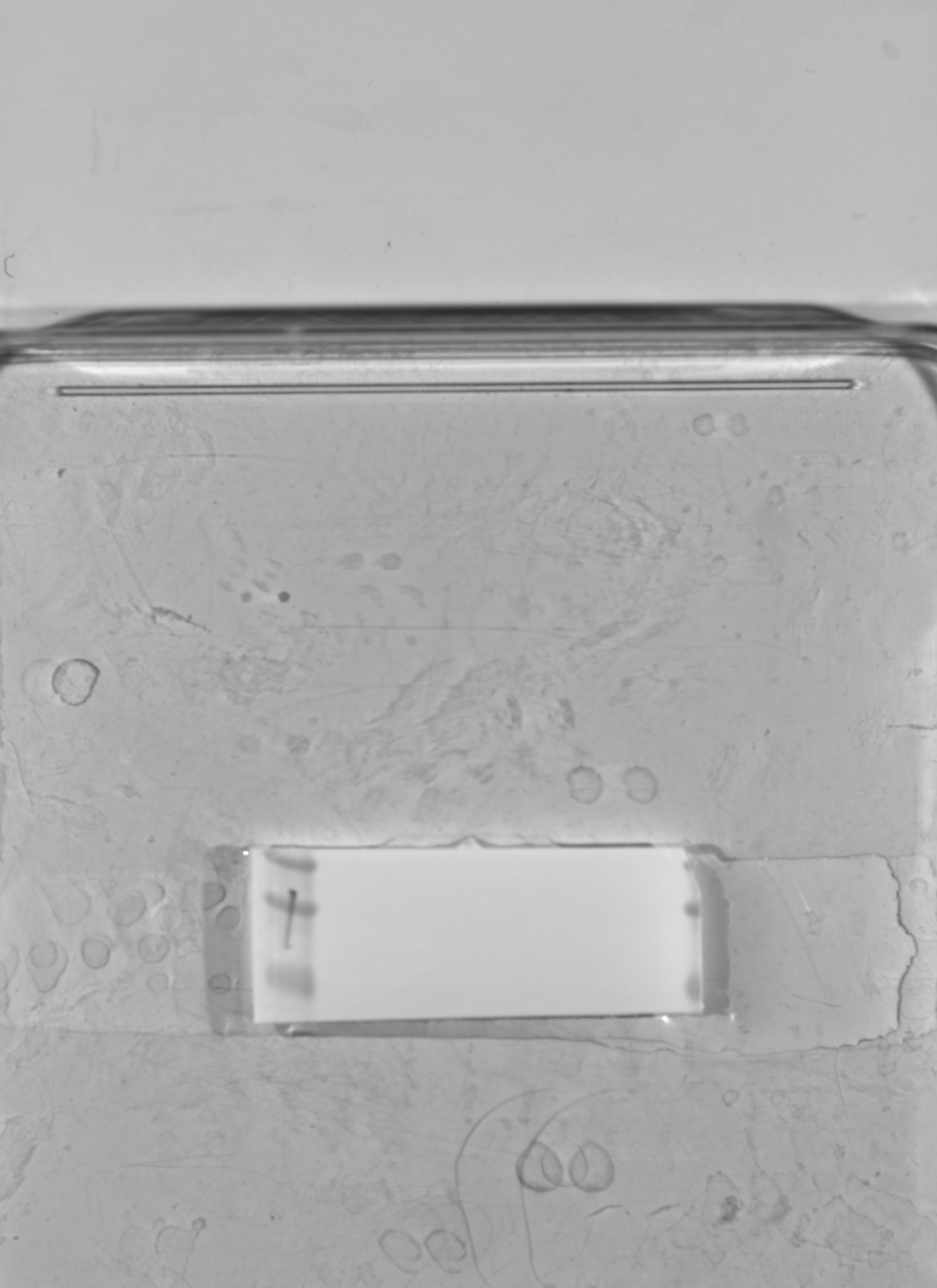

Supplement: Supplementary file 9 — Source data Fig. 4 [file 44321_2024_60_MOESM9_ESM.zip › Figure 4/4B/CN1/Western CDK 0.7S/1 5th CDK 0.7S _Ch-Marker.tif]

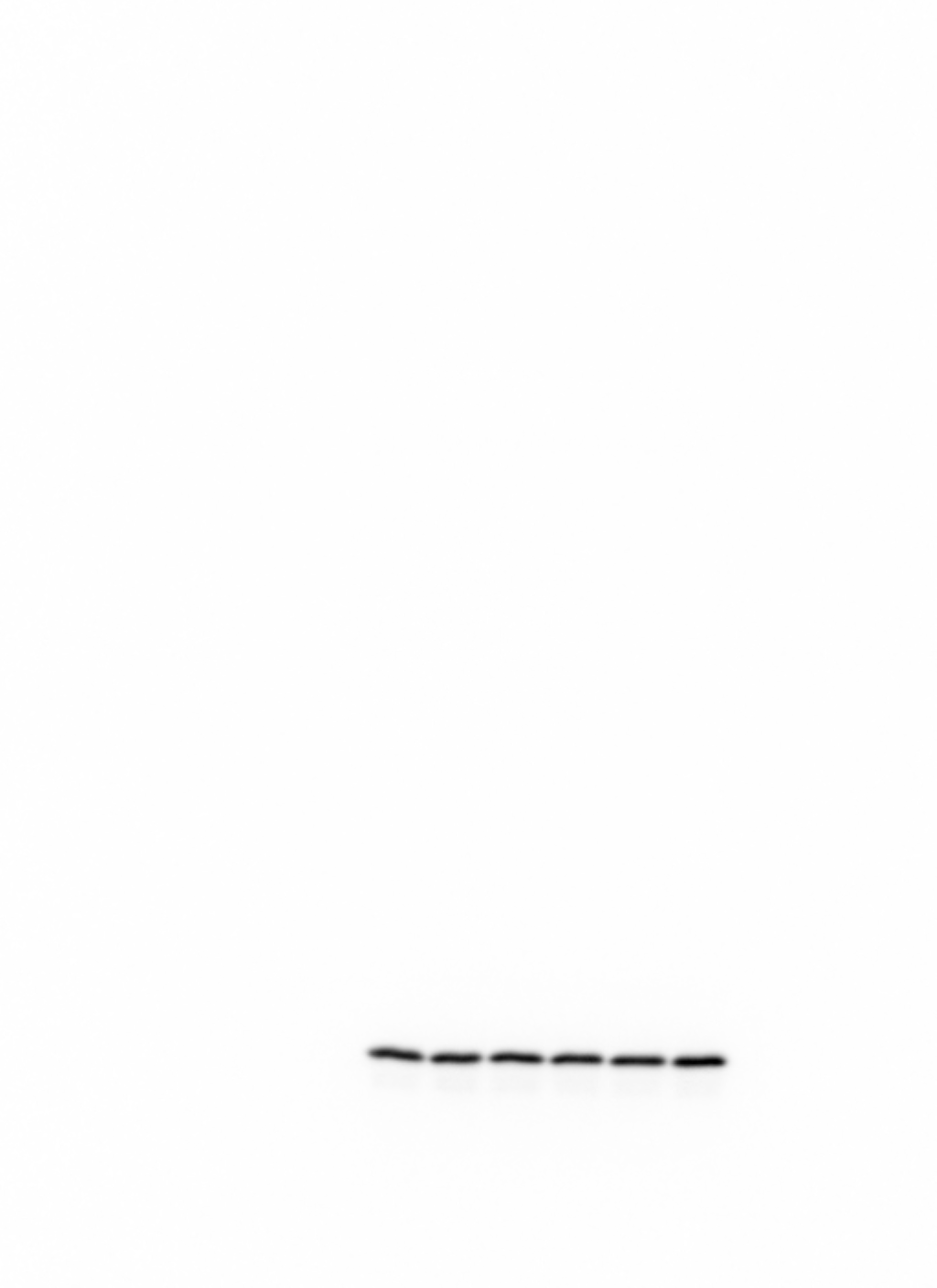

Supplement: Supplementary file 9 — Source data Fig. 4 [file 44321_2024_60_MOESM9_ESM.zip › Figure 4/4B/CN1/Western GAPDH 0.1S/WSM 4 0.1S _Ch.tif]

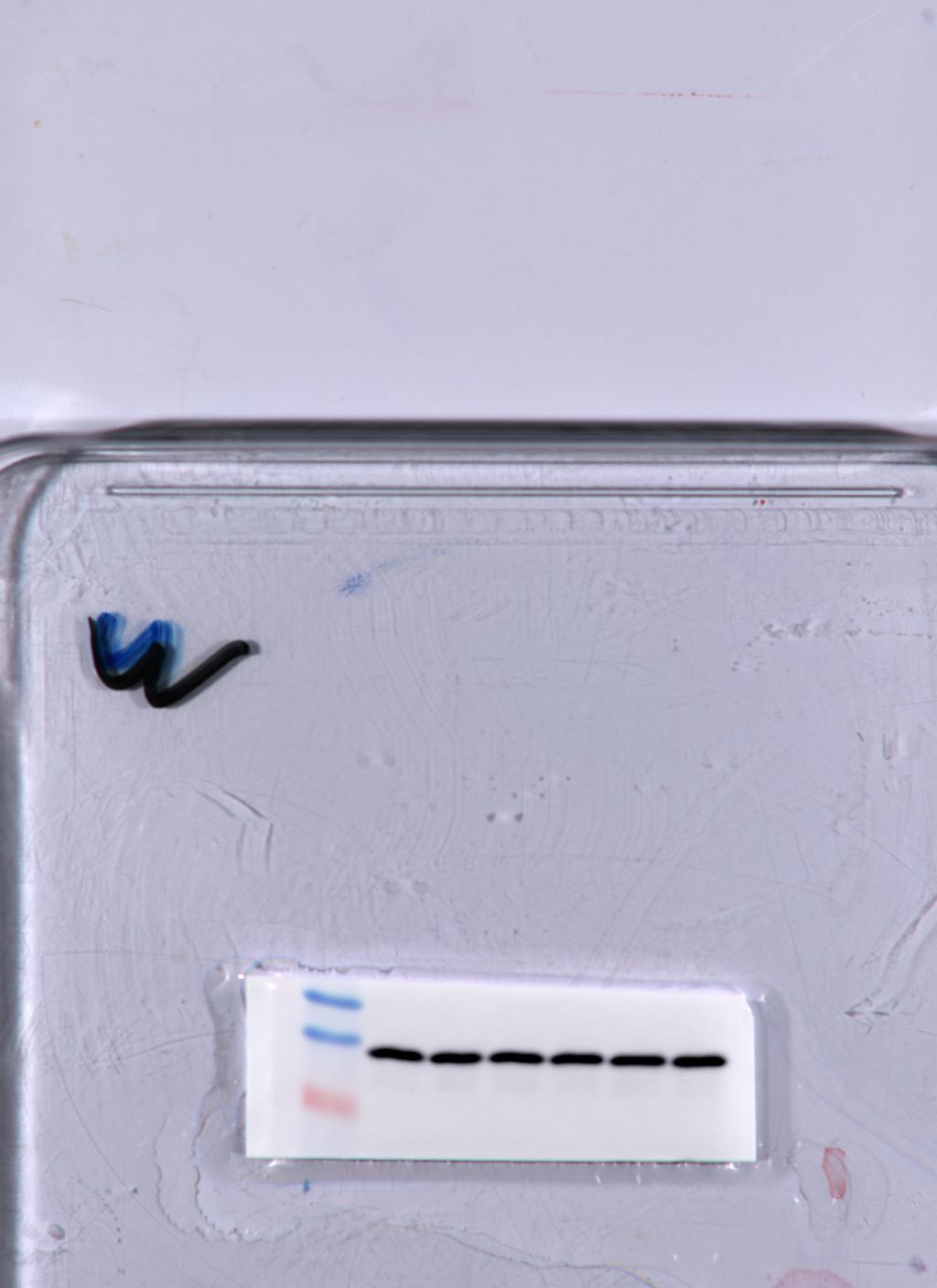

Supplement: Supplementary file 9 — Source data Fig. 4 [file 44321_2024_60_MOESM9_ESM.zip › Figure 4/4B/CN1/Western GAPDH 0.1S/WSM 4 0.1S _Ch+Marker.jpg]
