# Supplementary material for: Genome-wide CRISPR screens identify PKMYT1 as a therapeutic target in pancreatic ductal adenocarcinoma
Source: EMBO Mol Med. 2024 Apr 3;16(5):5. doi: 10.1038/s44321-024-00060-y (PMC11099189; doi:10.1038/s44321-024-00060-y)
Supplement: Supplementary file 9 — Source data Fig. 4 [file 44321_2024_60_MOESM9_ESM.zip › Figure 4/4B/88T/4B 88T.pptx]

## Slide 1
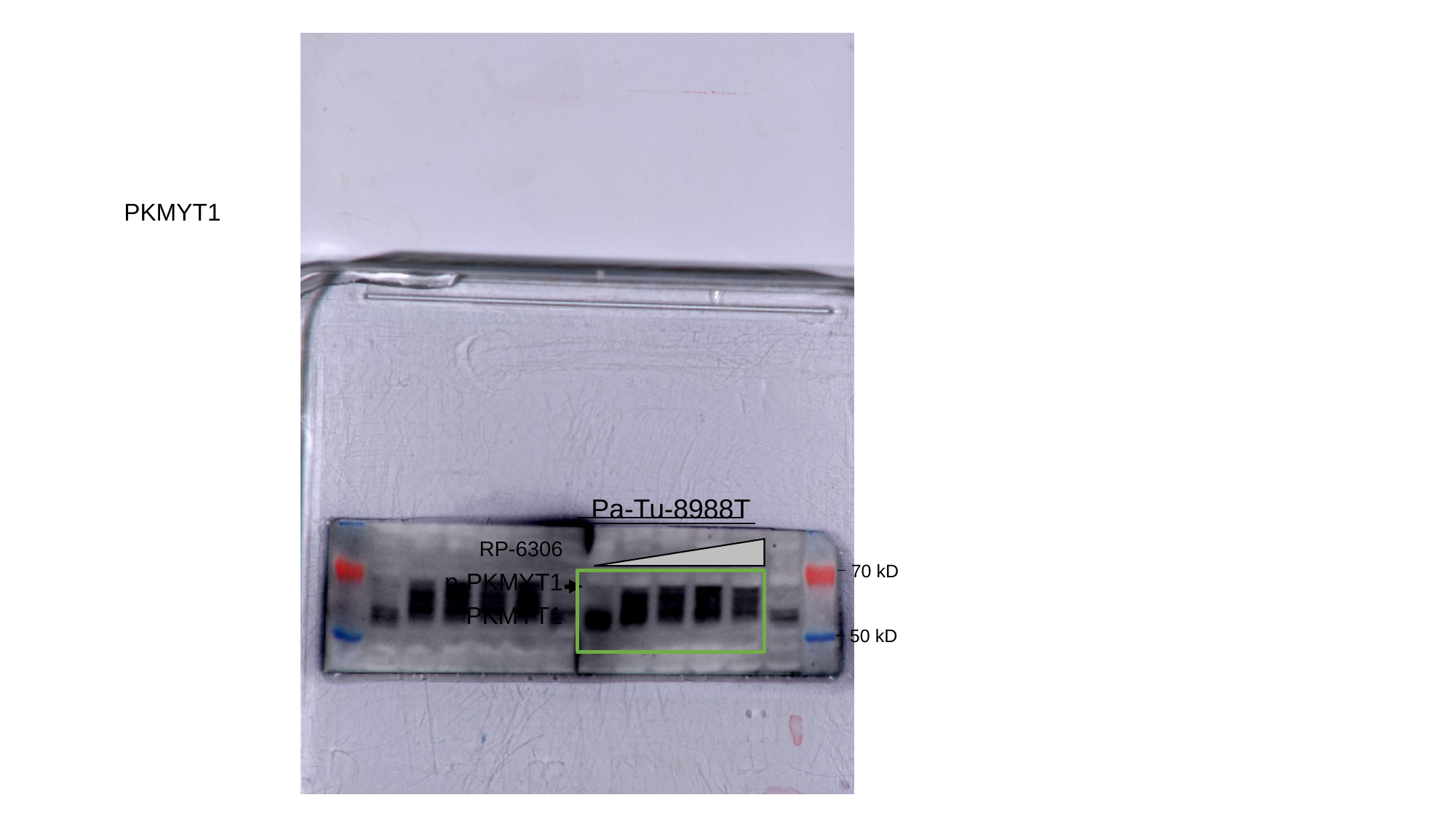

PKMYT1
Pa-Tu-8988T
RP-6306
70 kD
p-PKMYT1
PKMYT1
50 kD

## Slide 2
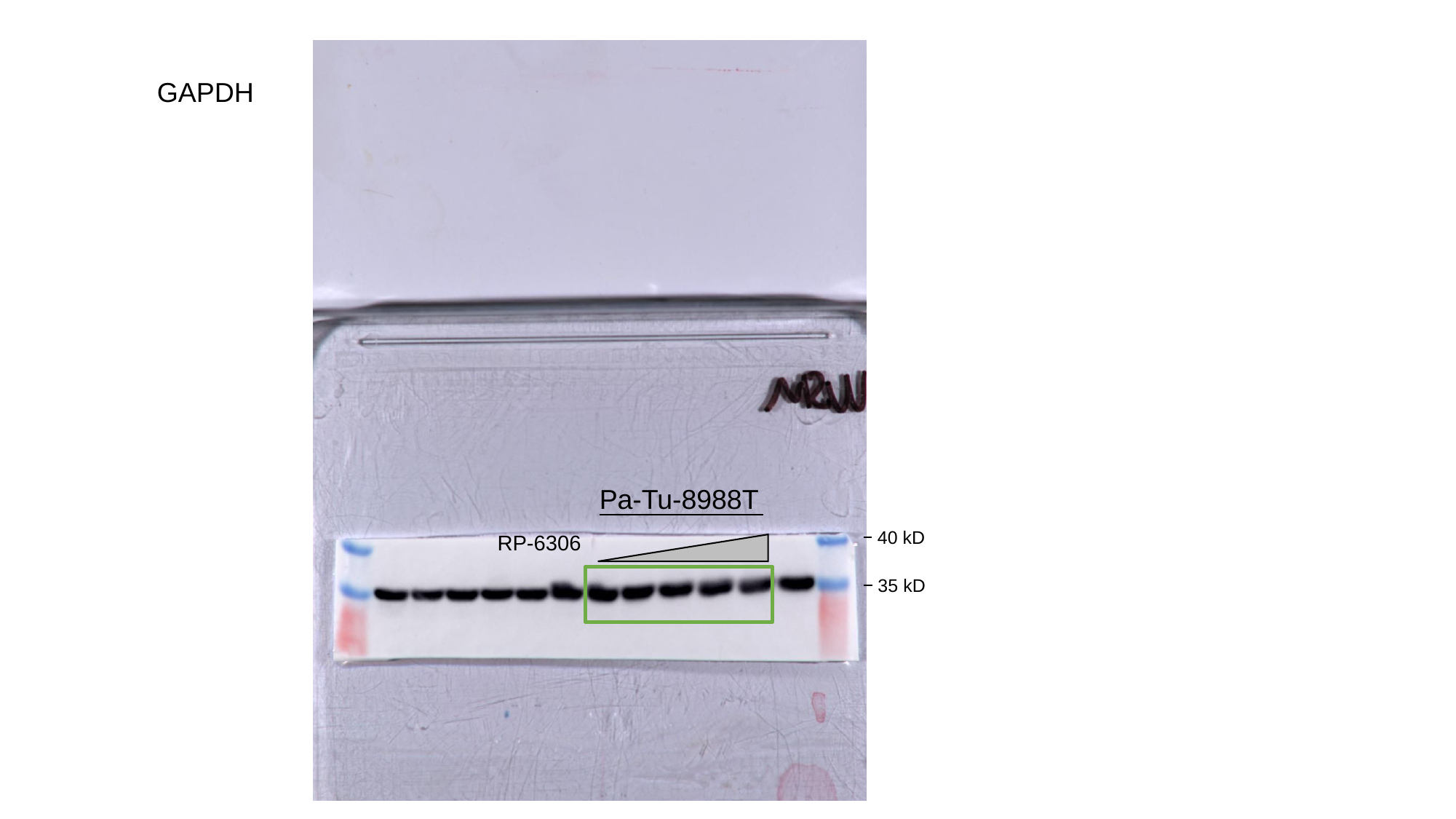

GAPDH
Pa-Tu-8988T
40 kD
RP-6306
35 kD

## Slide 3
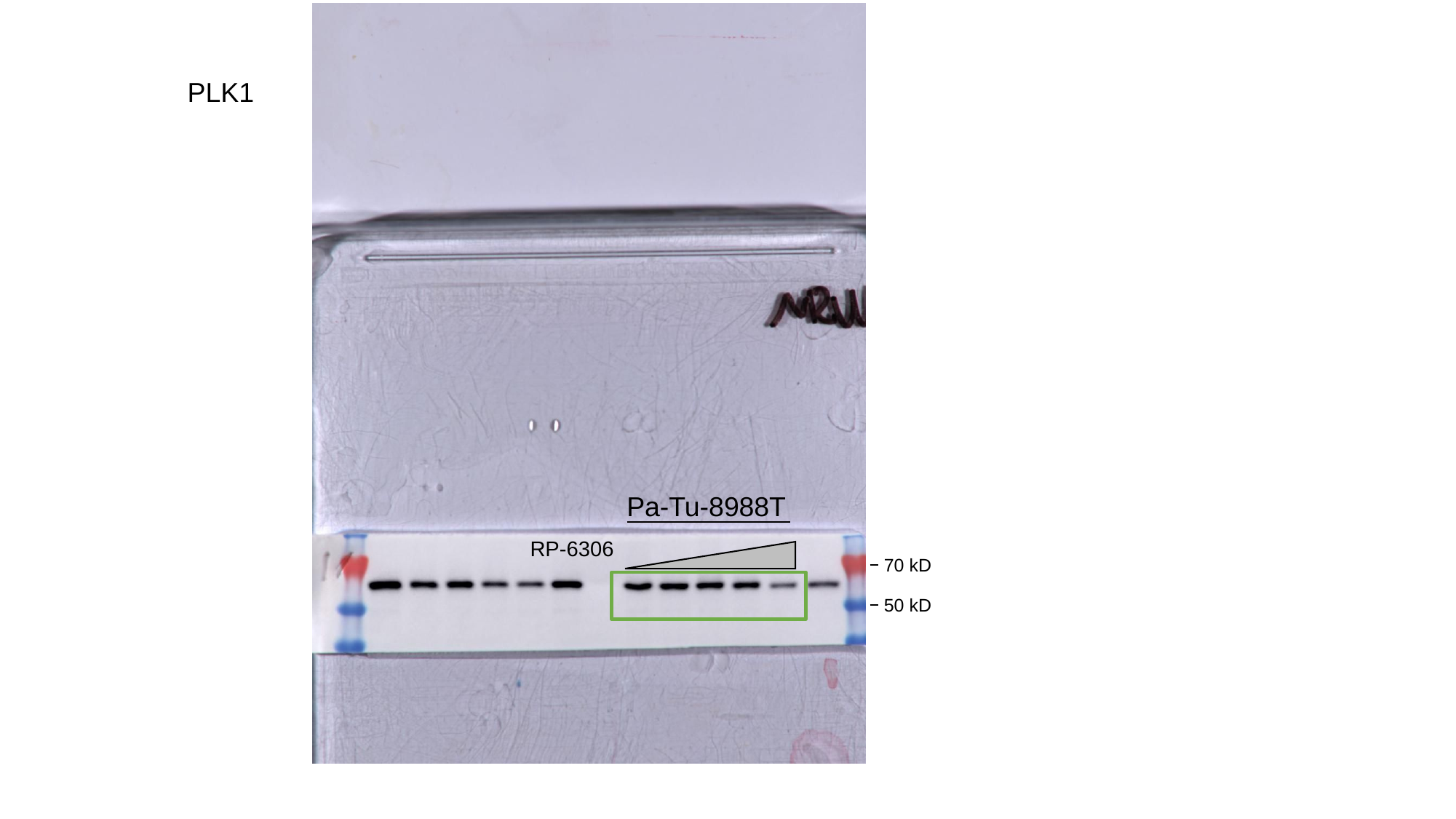

PLK1
Pa-Tu-8988T
RP-6306
70 kD
50 kD

## Slide 4
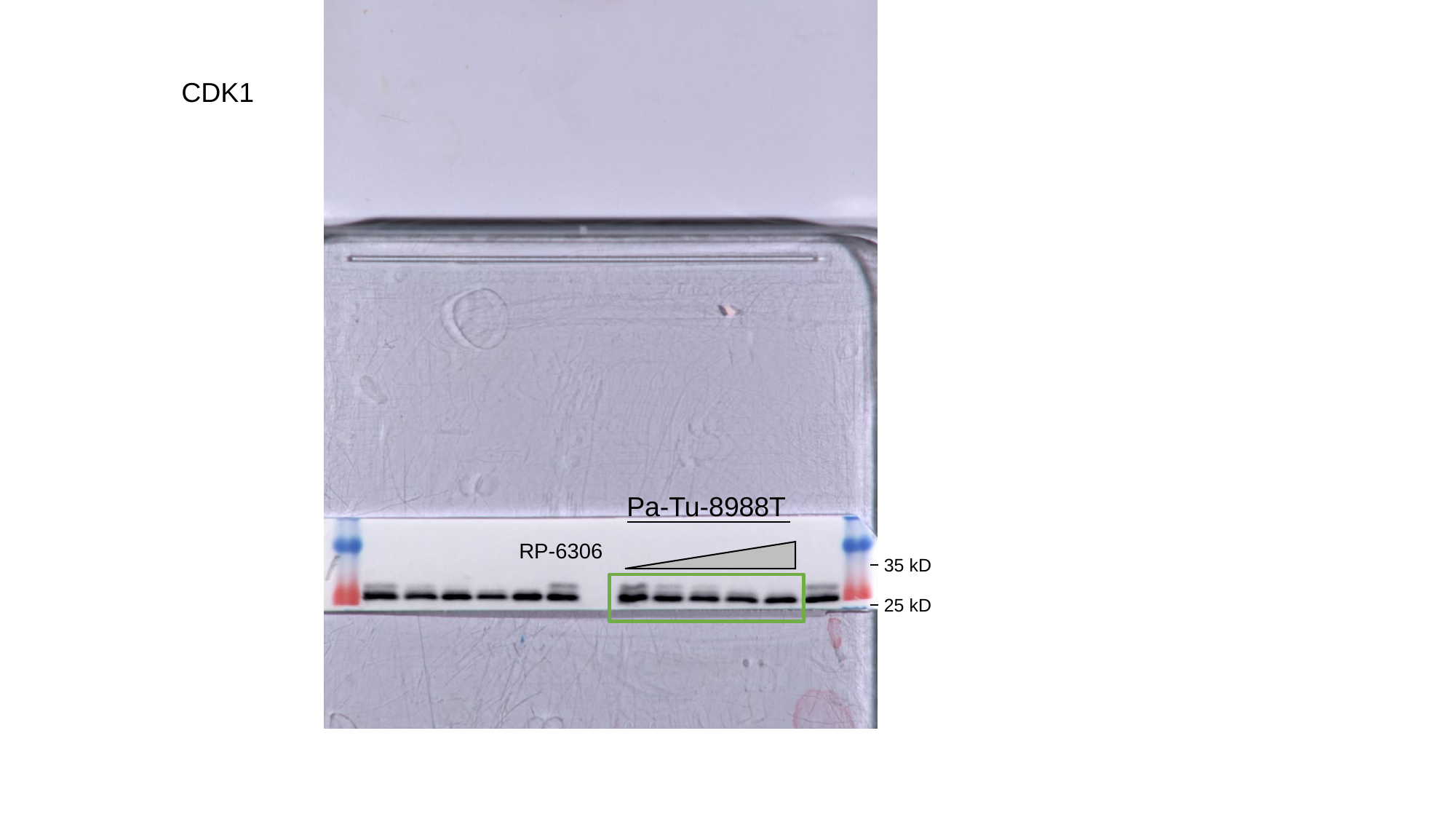

CDK1
Pa-Tu-8988T
RP-6306
35 kD
25 kD

## Slide 5
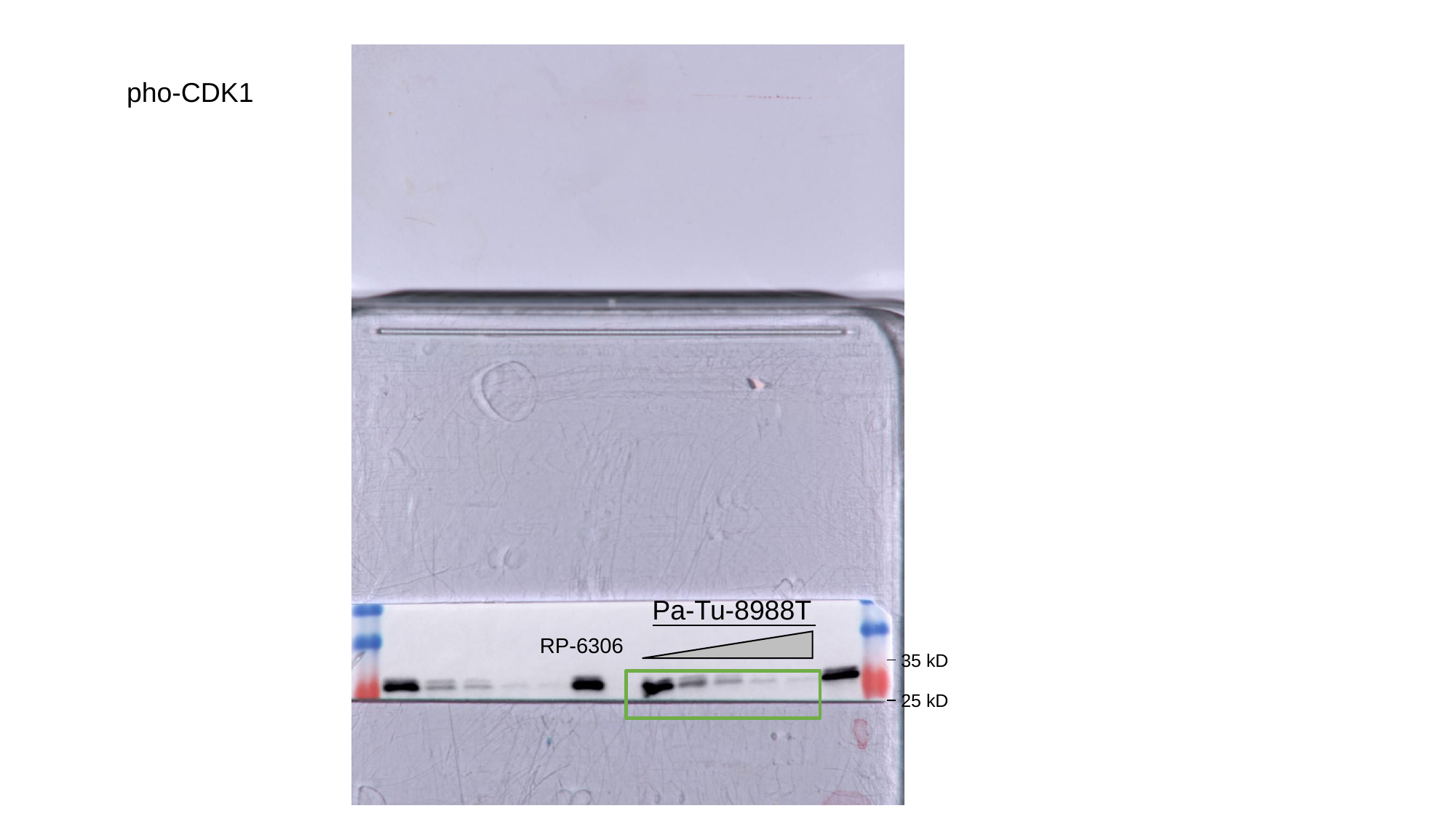

pho-CDK1
Pa-Tu-8988T
RP-6306
35 kD
25 kD

## Slide 6
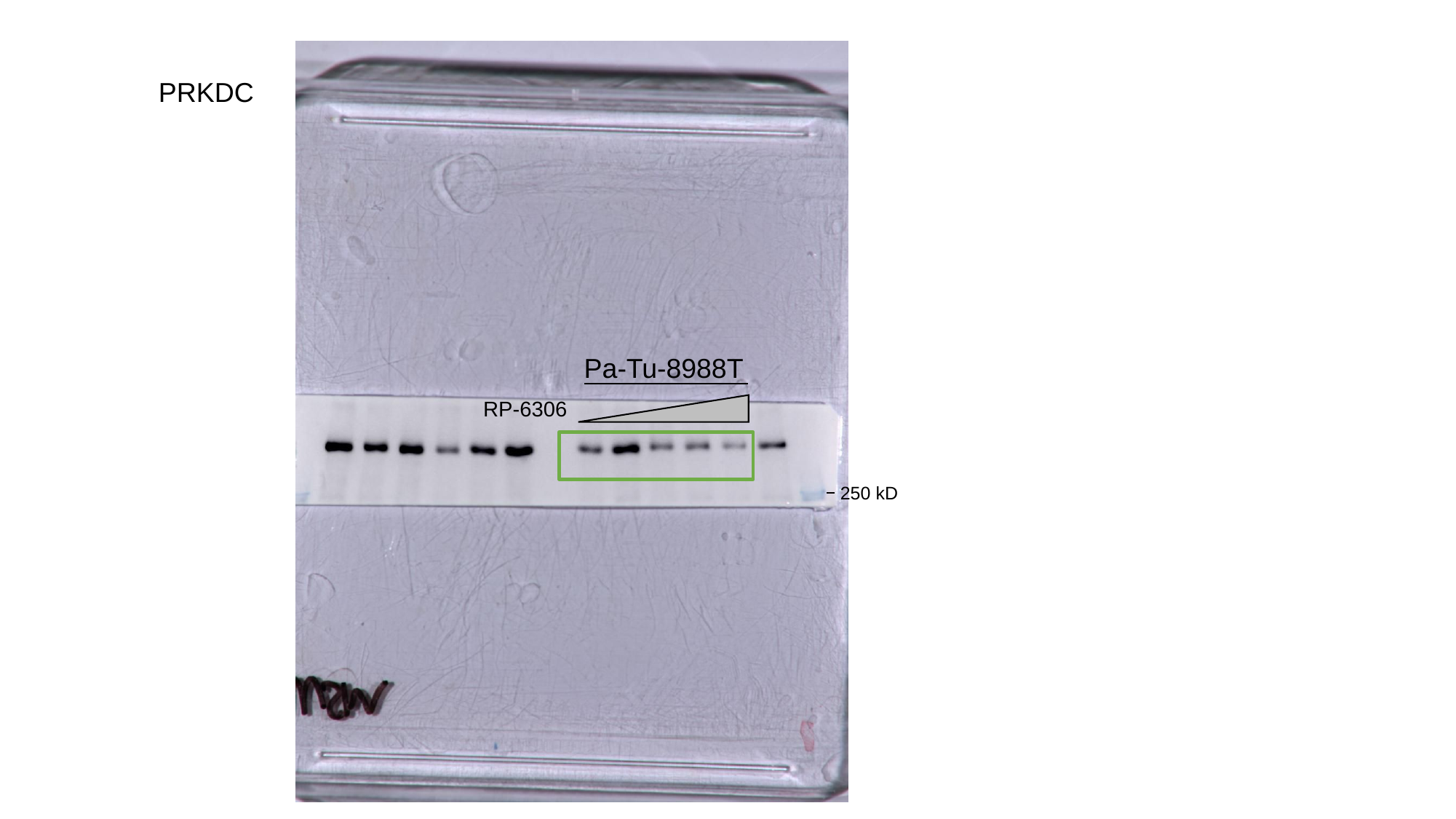

PRKDC
Pa-Tu-8988T
RP-6306
250 kD

## Slide 7
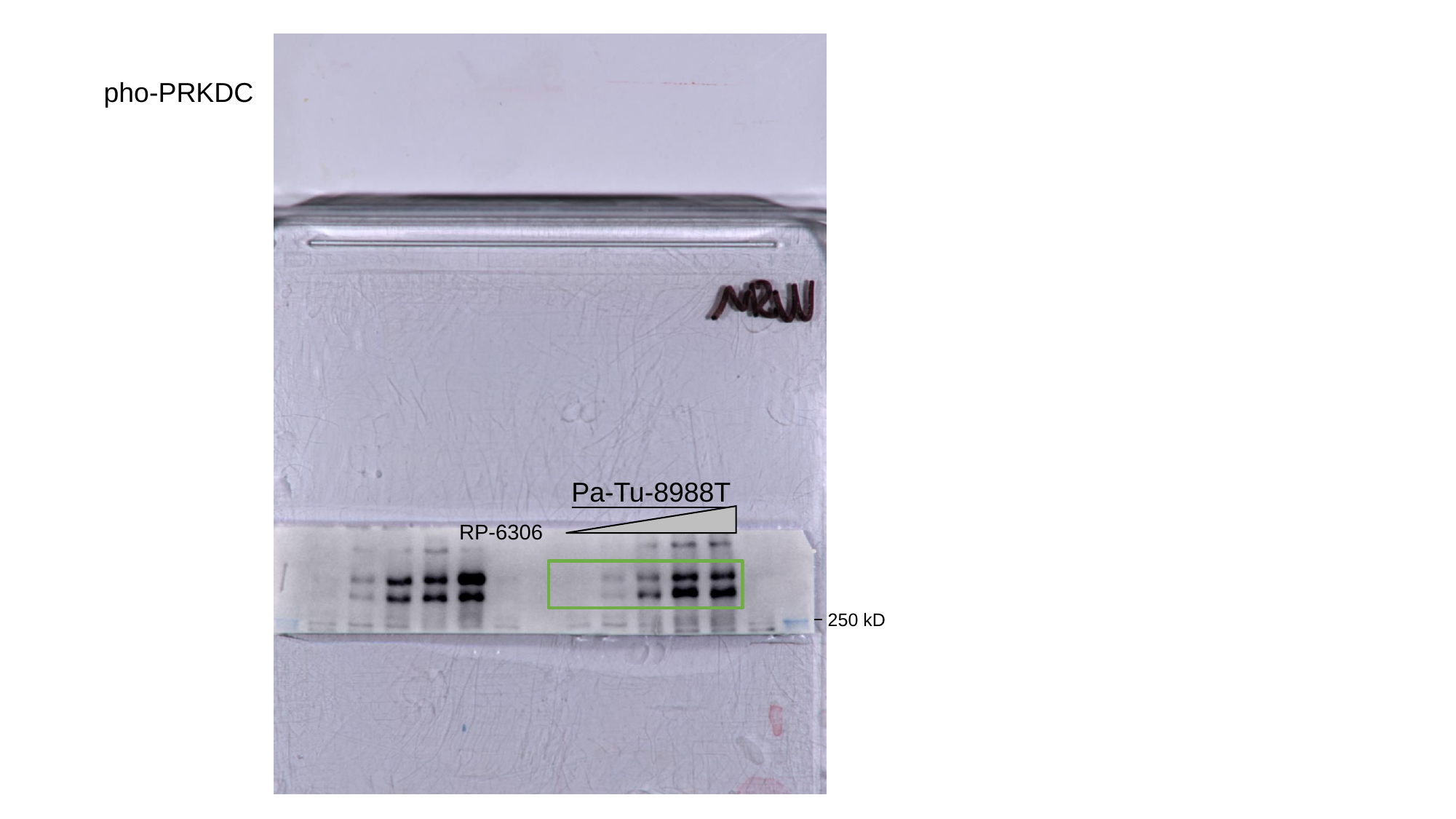

pho-PRKDC
Pa-Tu-8988T
RP-6306
250 kD
