# Supplementary figures and images for: Genome-wide CRISPR screens identify PKMYT1 as a therapeutic target in pancreatic ductal adenocarcinoma (part 2 of 4)
Source: EMBO Mol Med. 2024 Apr 3;16(5):5. doi: 10.1038/s44321-024-00060-y (PMC11099189; doi:10.1038/s44321-024-00060-y)

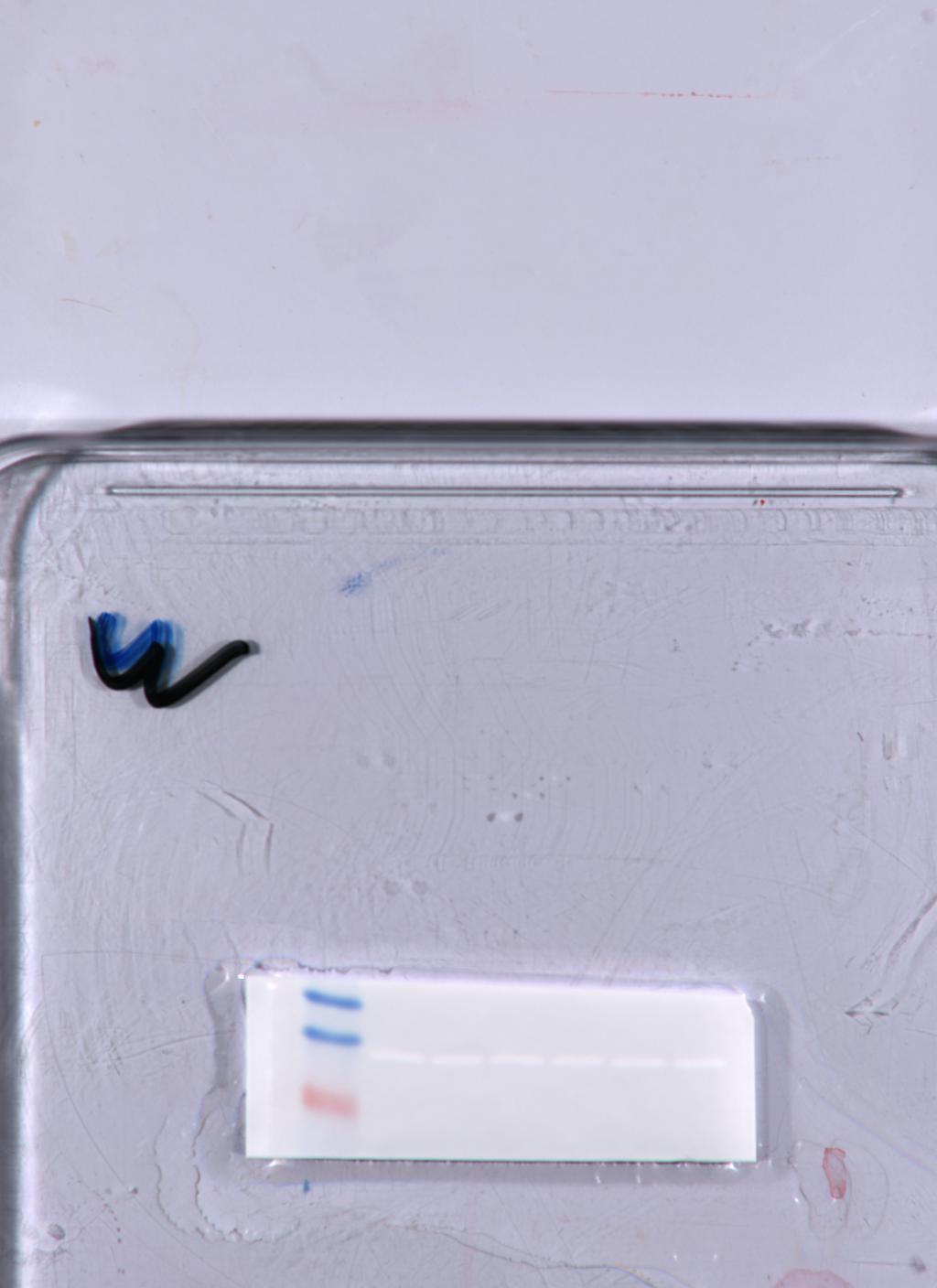

Supplement: Supplementary file 9 — Source data Fig. 4 [file 44321_2024_60_MOESM9_ESM.zip › Figure 4/4B/CN1/Western GAPDH 0.1S/WSM 4 0.1S _Ch-Marker.jpg]

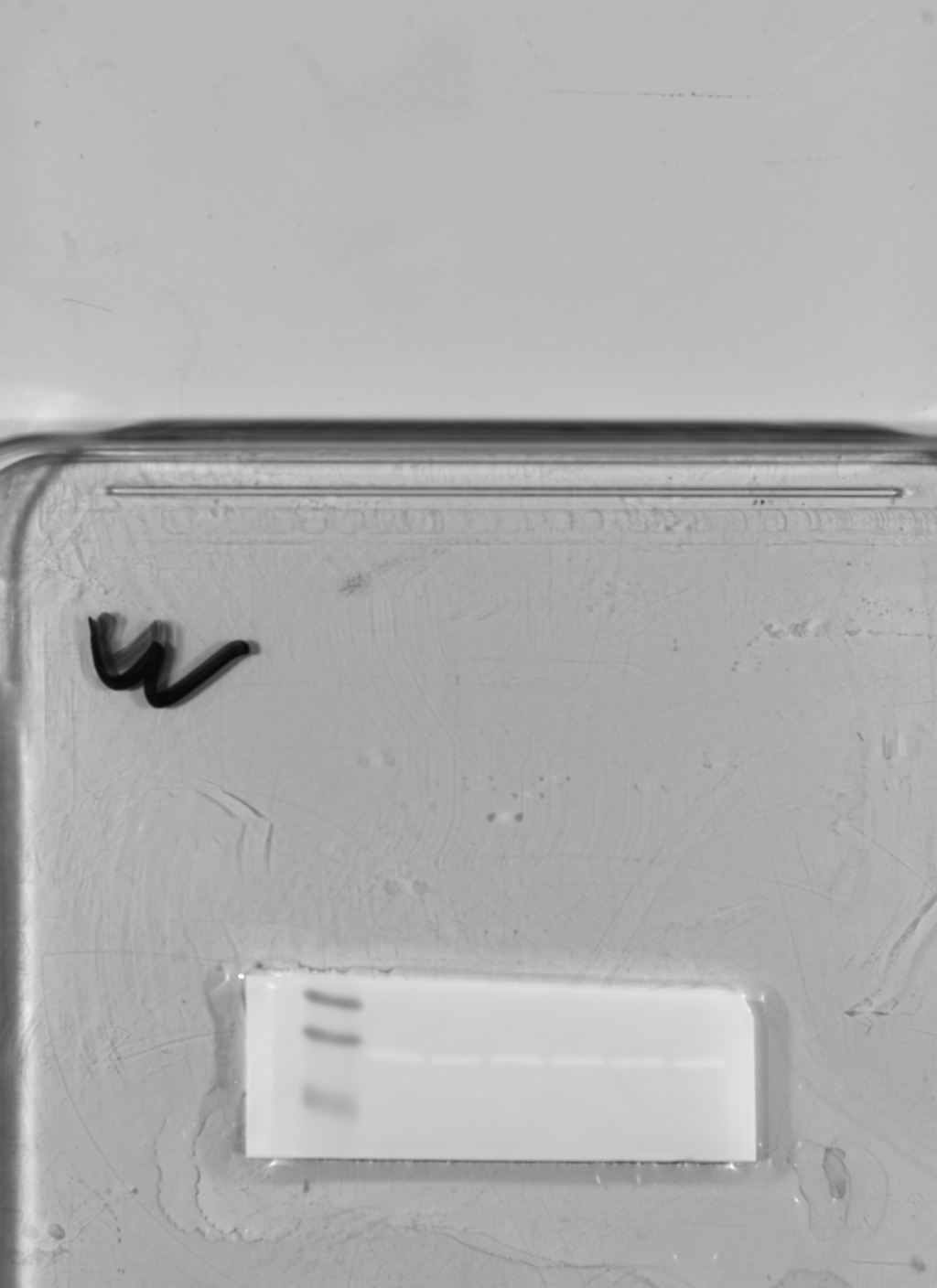

Supplement: Supplementary file 9 — Source data Fig. 4 [file 44321_2024_60_MOESM9_ESM.zip › Figure 4/4B/CN1/Western GAPDH 0.1S/WSM 4 0.1S _Ch-Marker.tif]

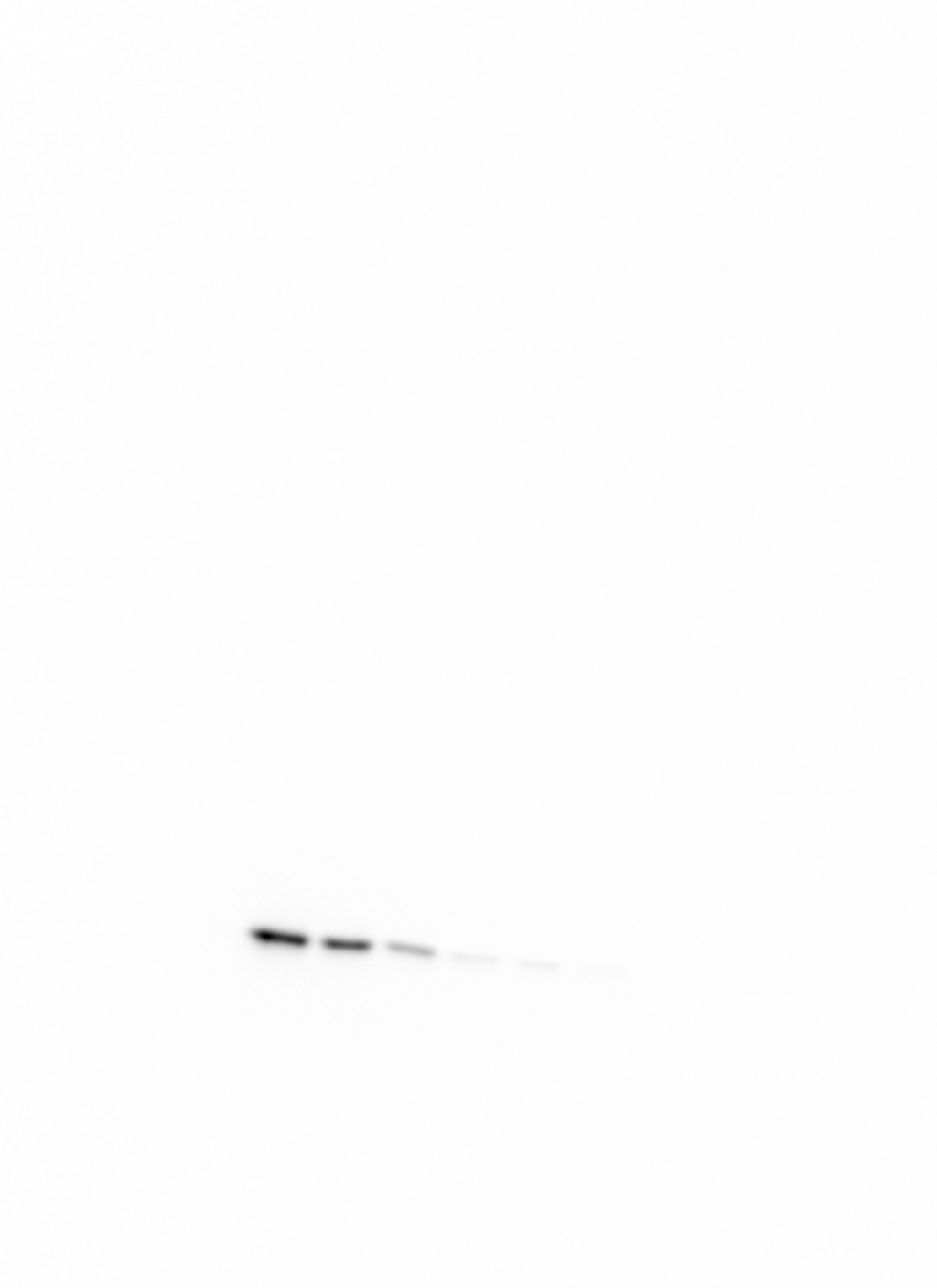

Supplement: Supplementary file 9 — Source data Fig. 4 [file 44321_2024_60_MOESM9_ESM.zip › Figure 4/4B/CN1/Western phoCDK 0.2/1 2nd phoCDK 0.2S _Ch.tif]

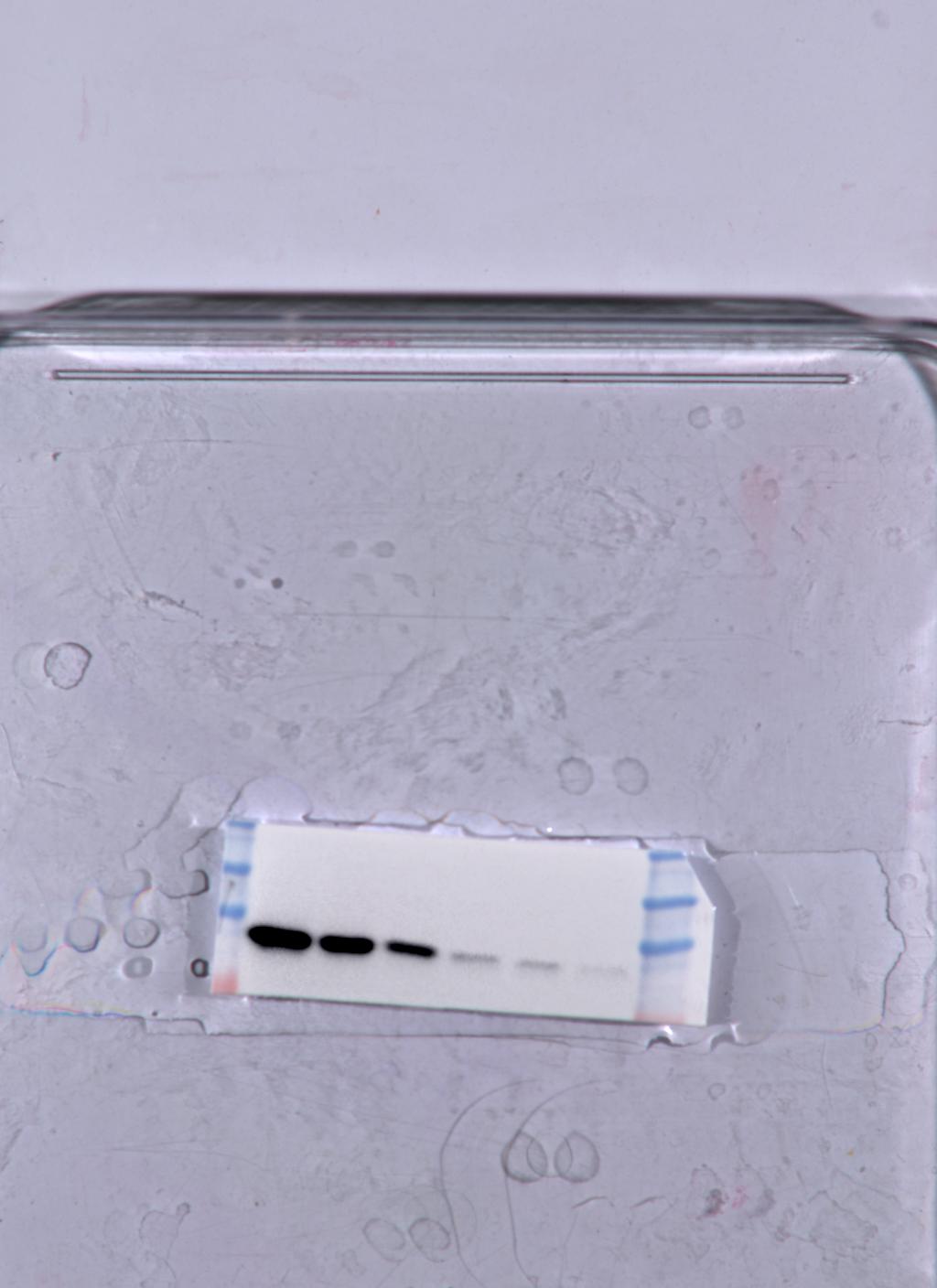

Supplement: Supplementary file 9 — Source data Fig. 4 [file 44321_2024_60_MOESM9_ESM.zip › Figure 4/4B/CN1/Western phoCDK 0.2/1 2nd phoCDK 0.2S _Ch+Marker.jpg]

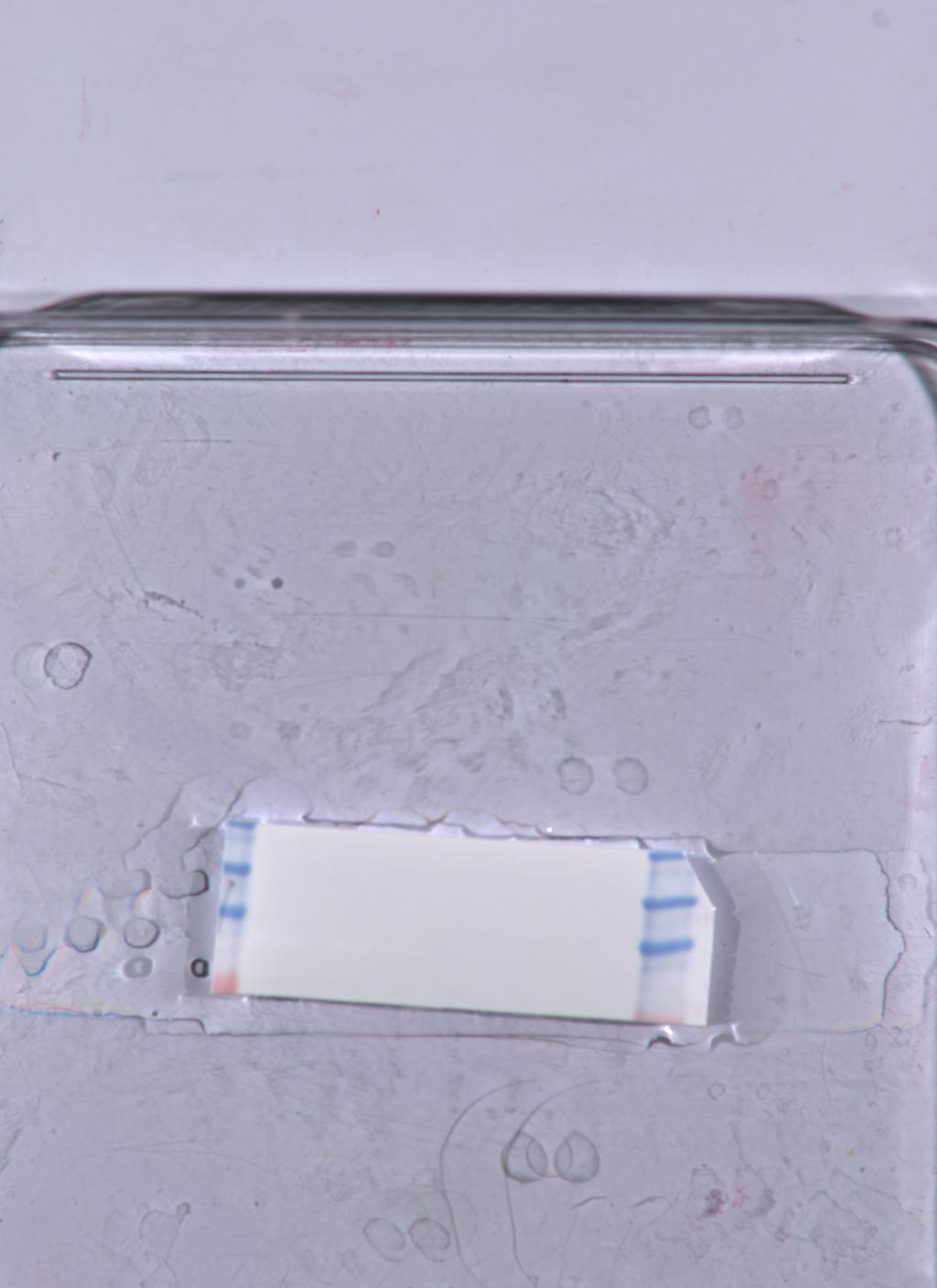

Supplement: Supplementary file 9 — Source data Fig. 4 [file 44321_2024_60_MOESM9_ESM.zip › Figure 4/4B/CN1/Western phoCDK 0.2/1 2nd phoCDK 0.2S _Ch-Marker.jpg]

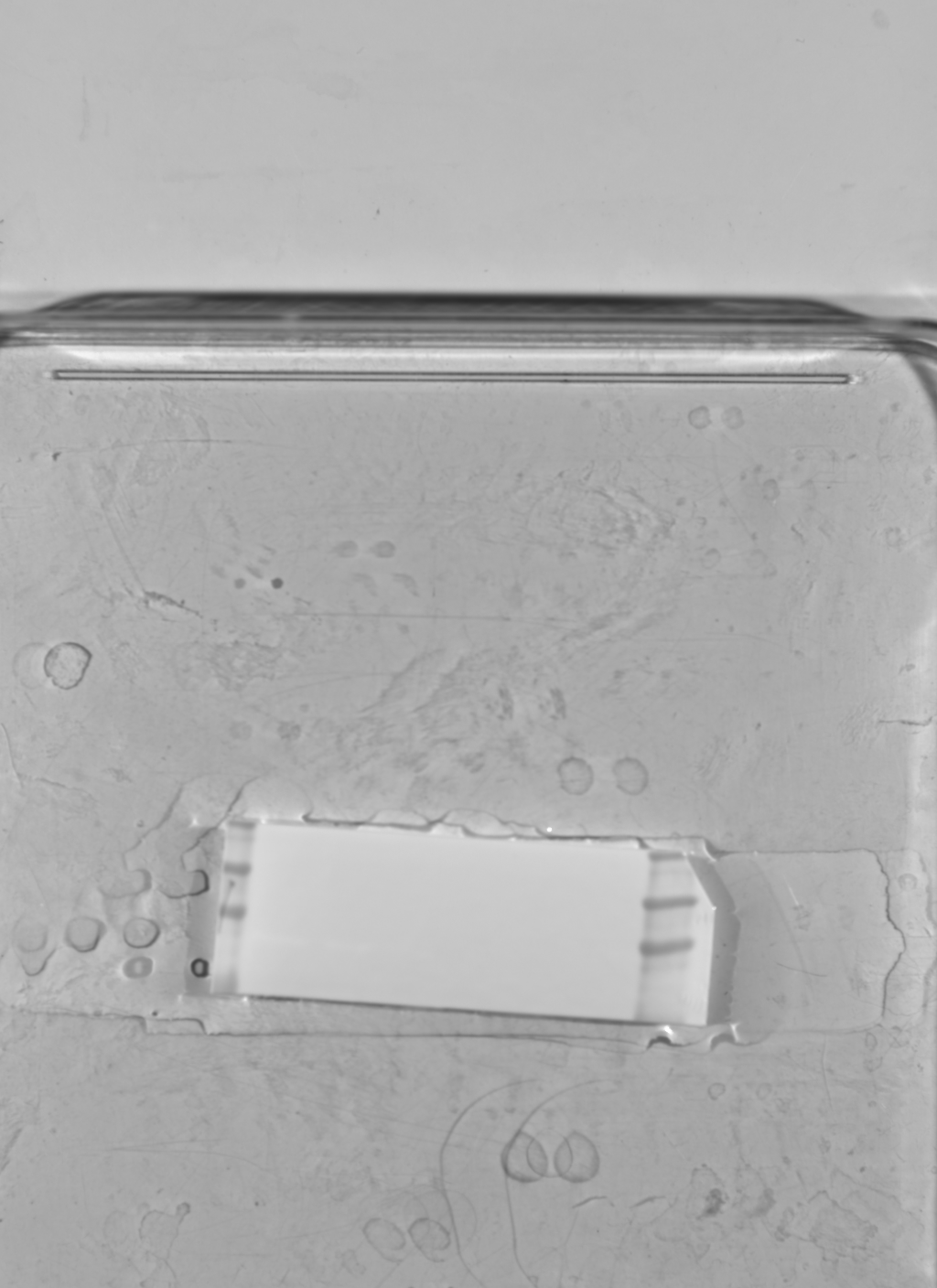

Supplement: Supplementary file 9 — Source data Fig. 4 [file 44321_2024_60_MOESM9_ESM.zip › Figure 4/4B/CN1/Western phoCDK 0.2/1 2nd phoCDK 0.2S _Ch-Marker.tif]

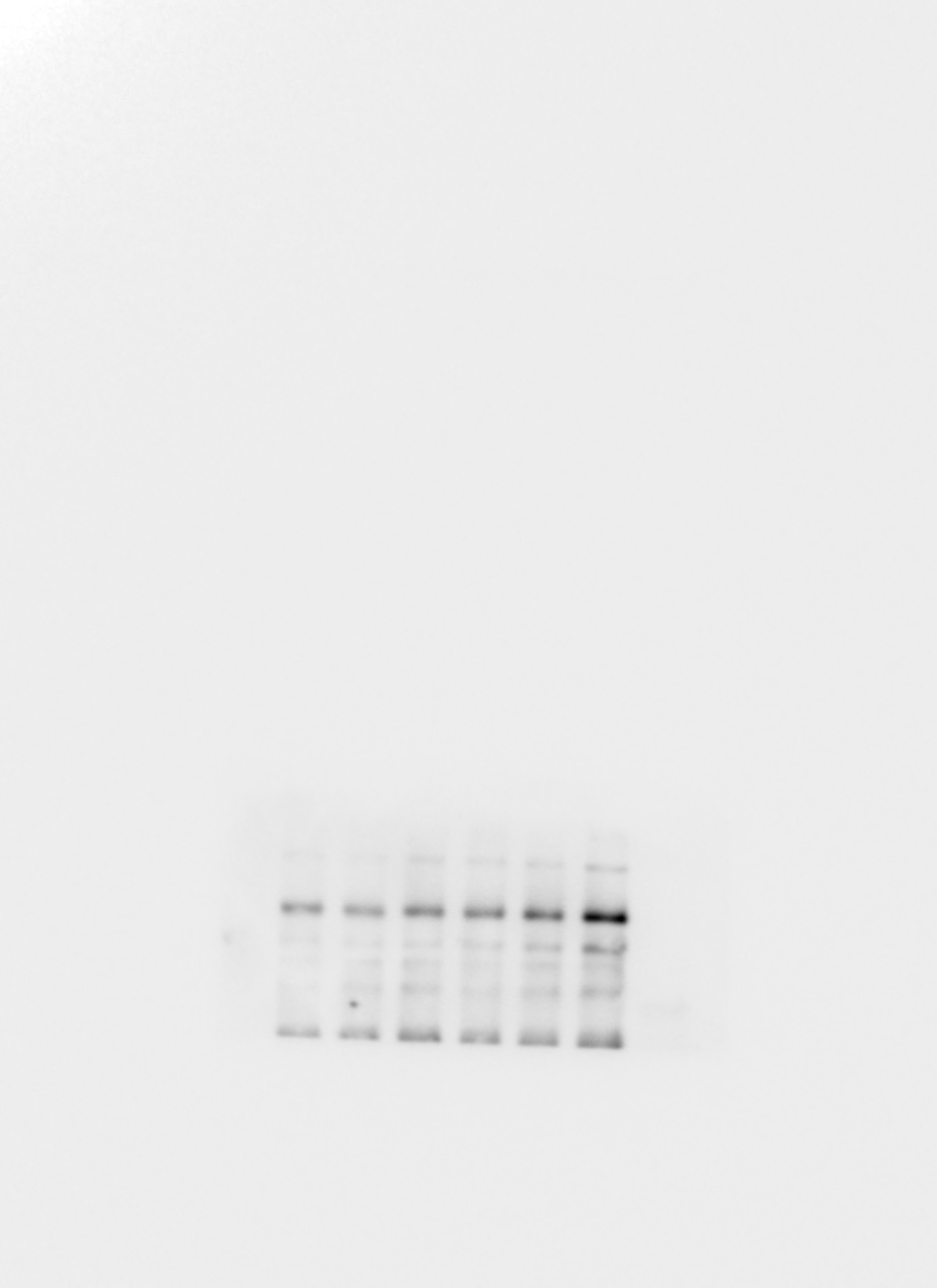

Supplement: Supplementary file 9 — Source data Fig. 4 [file 44321_2024_60_MOESM9_ESM.zip › Figure 4/4B/CN1/Western phoPRKDC 4.6S/WSM 2 4.6S _Ch.tif]

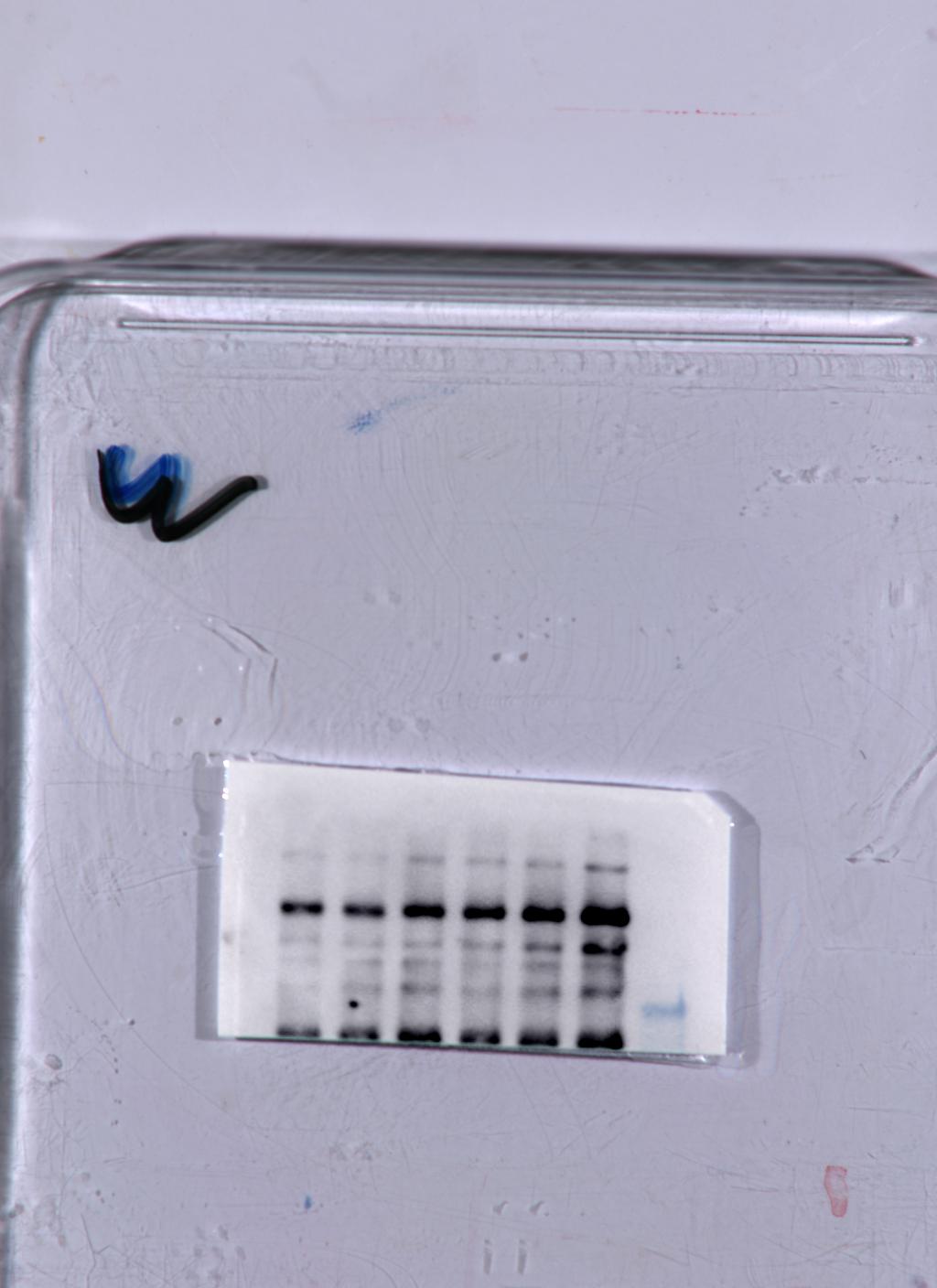

Supplement: Supplementary file 9 — Source data Fig. 4 [file 44321_2024_60_MOESM9_ESM.zip › Figure 4/4B/CN1/Western phoPRKDC 4.6S/WSM 2 4.6S _Ch+Marker.jpg]

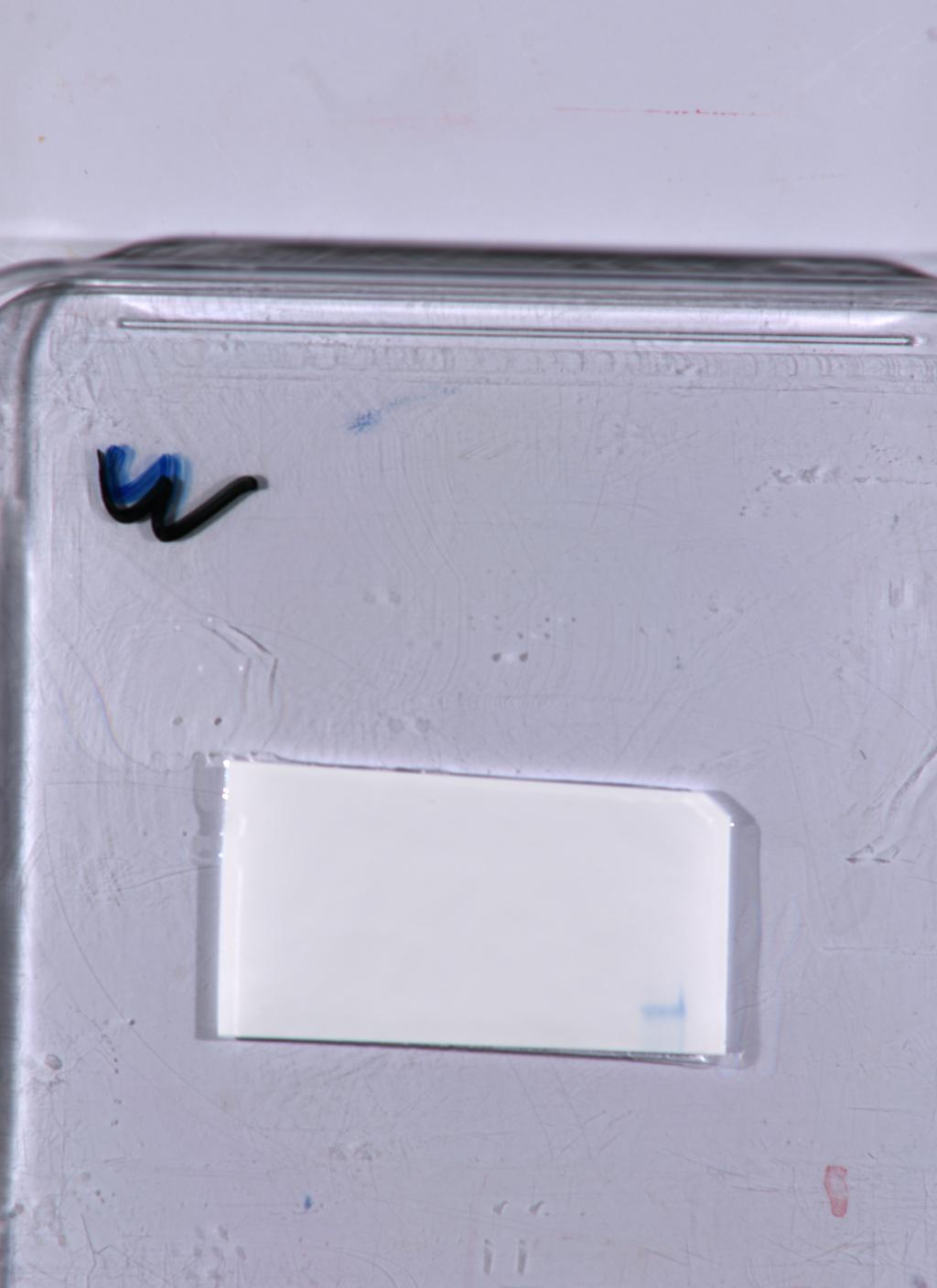

Supplement: Supplementary file 9 — Source data Fig. 4 [file 44321_2024_60_MOESM9_ESM.zip › Figure 4/4B/CN1/Western phoPRKDC 4.6S/WSM 2 4.6S _Ch-Marker.jpg]

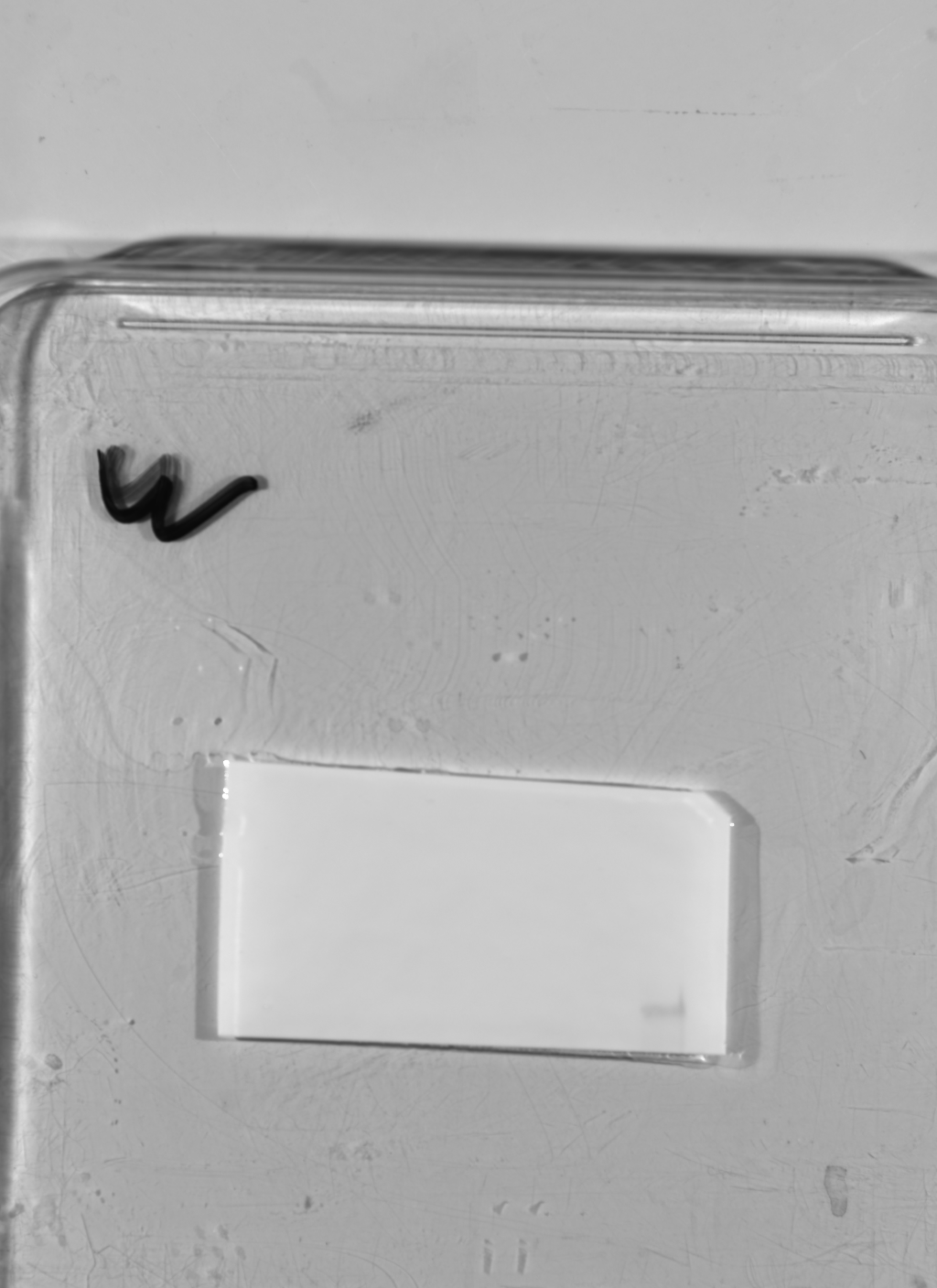

Supplement: Supplementary file 9 — Source data Fig. 4 [file 44321_2024_60_MOESM9_ESM.zip › Figure 4/4B/CN1/Western phoPRKDC 4.6S/WSM 2 4.6S _Ch-Marker.tif]

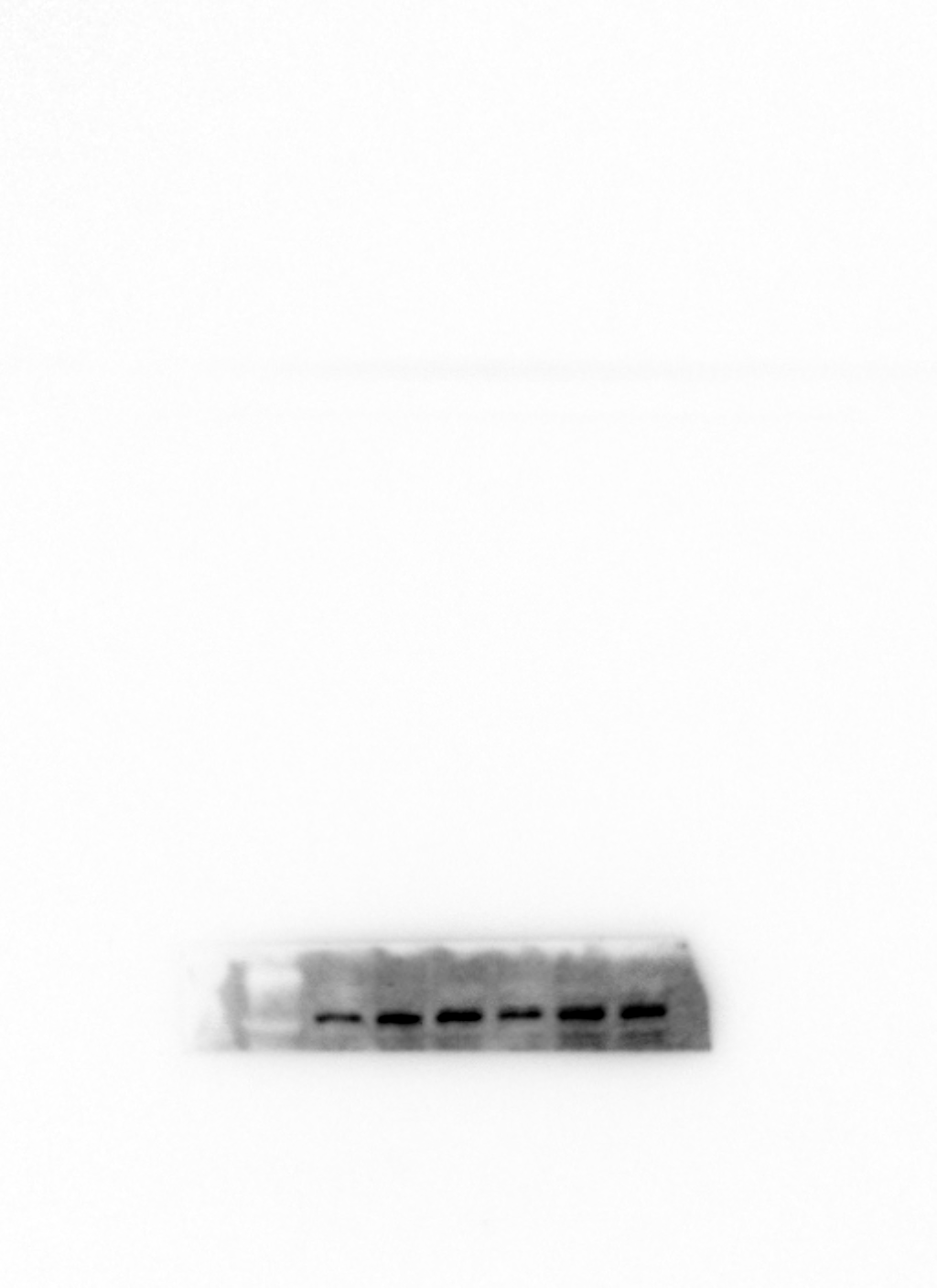

Supplement: Supplementary file 9 — Source data Fig. 4 [file 44321_2024_60_MOESM9_ESM.zip › Figure 4/4B/CN1/Western PKMYT1 28.5S/WSM 5 28.5S _Ch.tif]

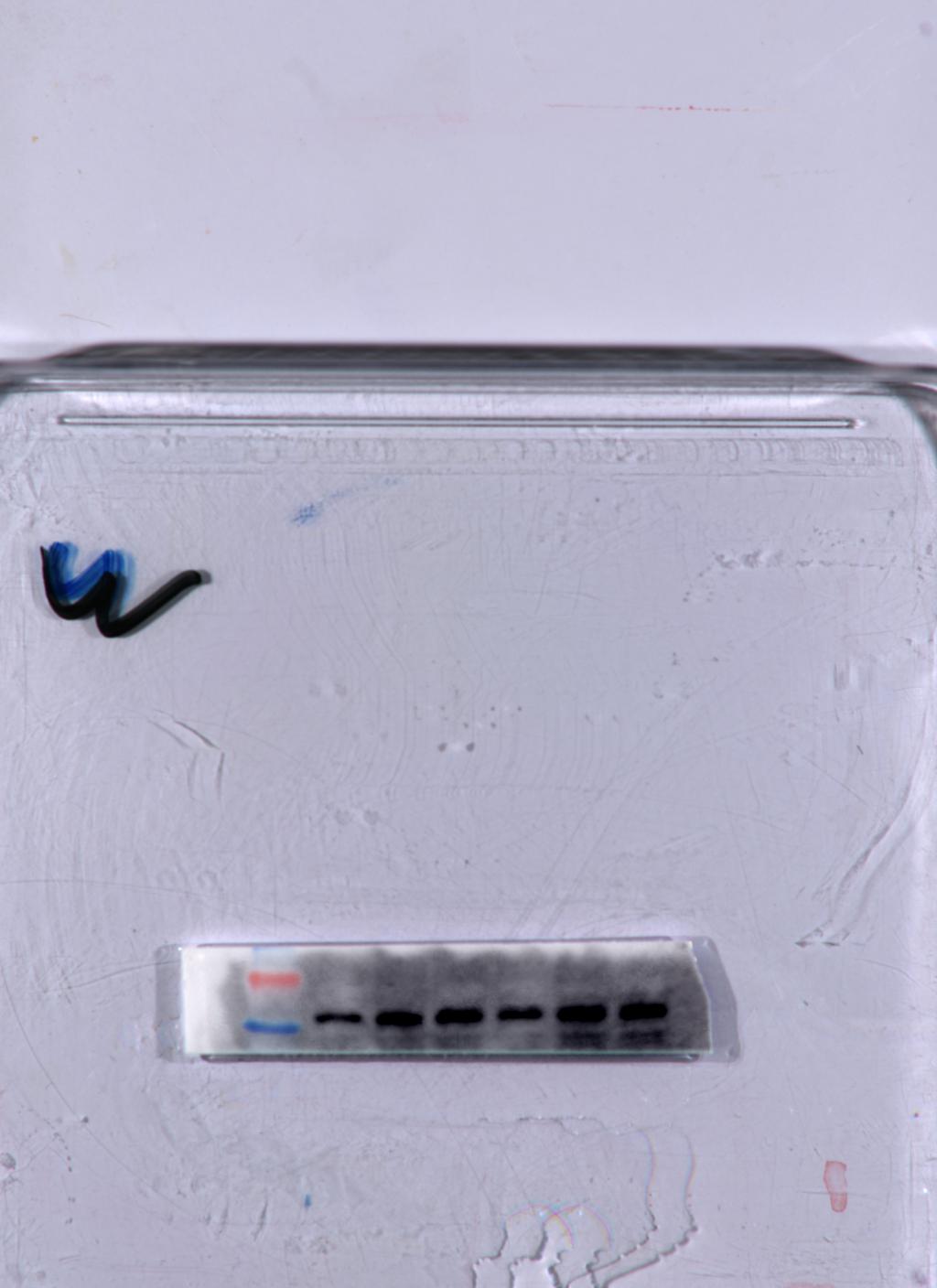

Supplement: Supplementary file 9 — Source data Fig. 4 [file 44321_2024_60_MOESM9_ESM.zip › Figure 4/4B/CN1/Western PKMYT1 28.5S/WSM 5 28.5S _Ch+Marker.jpg]

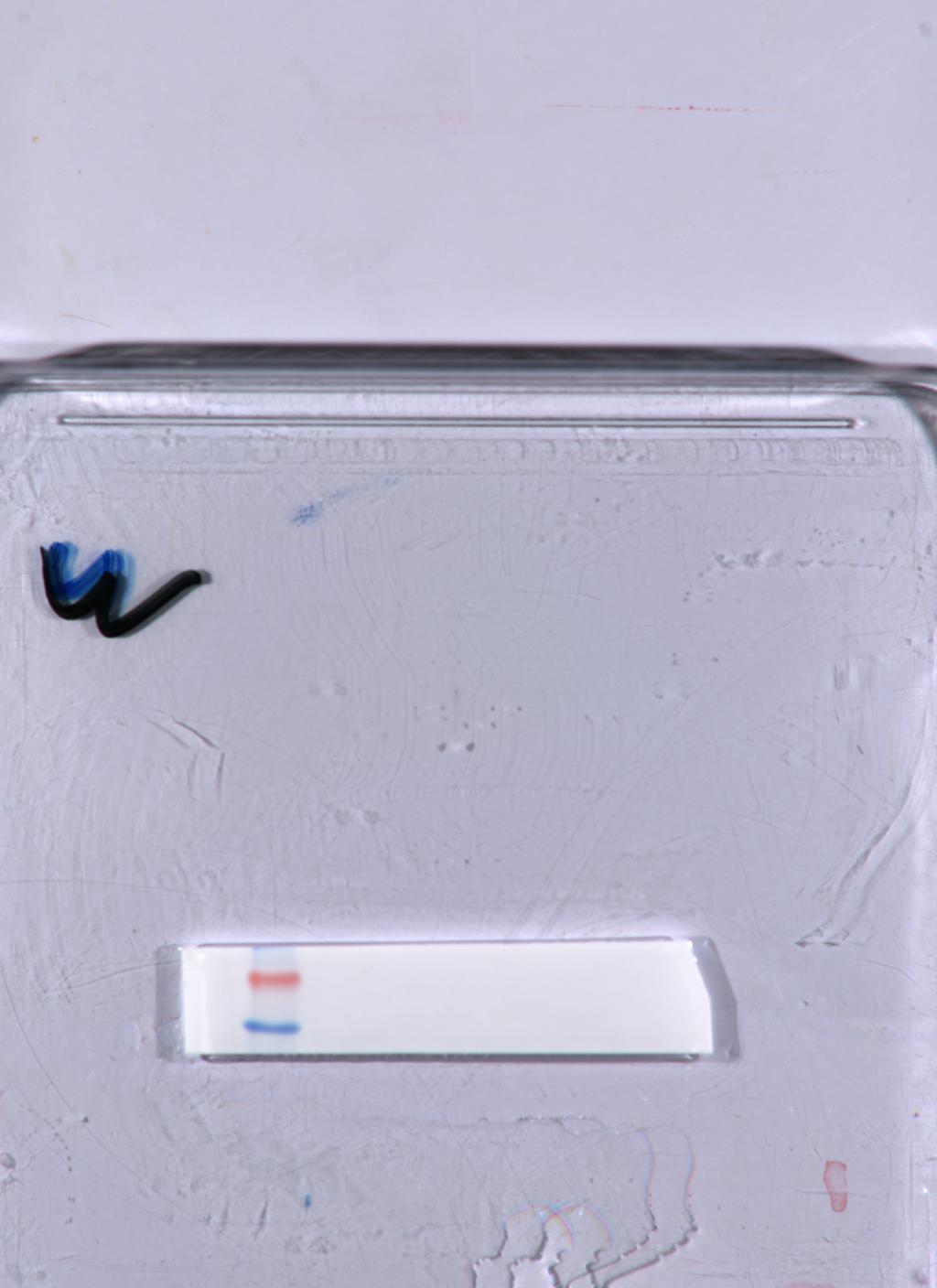

Supplement: Supplementary file 9 — Source data Fig. 4 [file 44321_2024_60_MOESM9_ESM.zip › Figure 4/4B/CN1/Western PKMYT1 28.5S/WSM 5 28.5S _Ch-Marker.jpg]

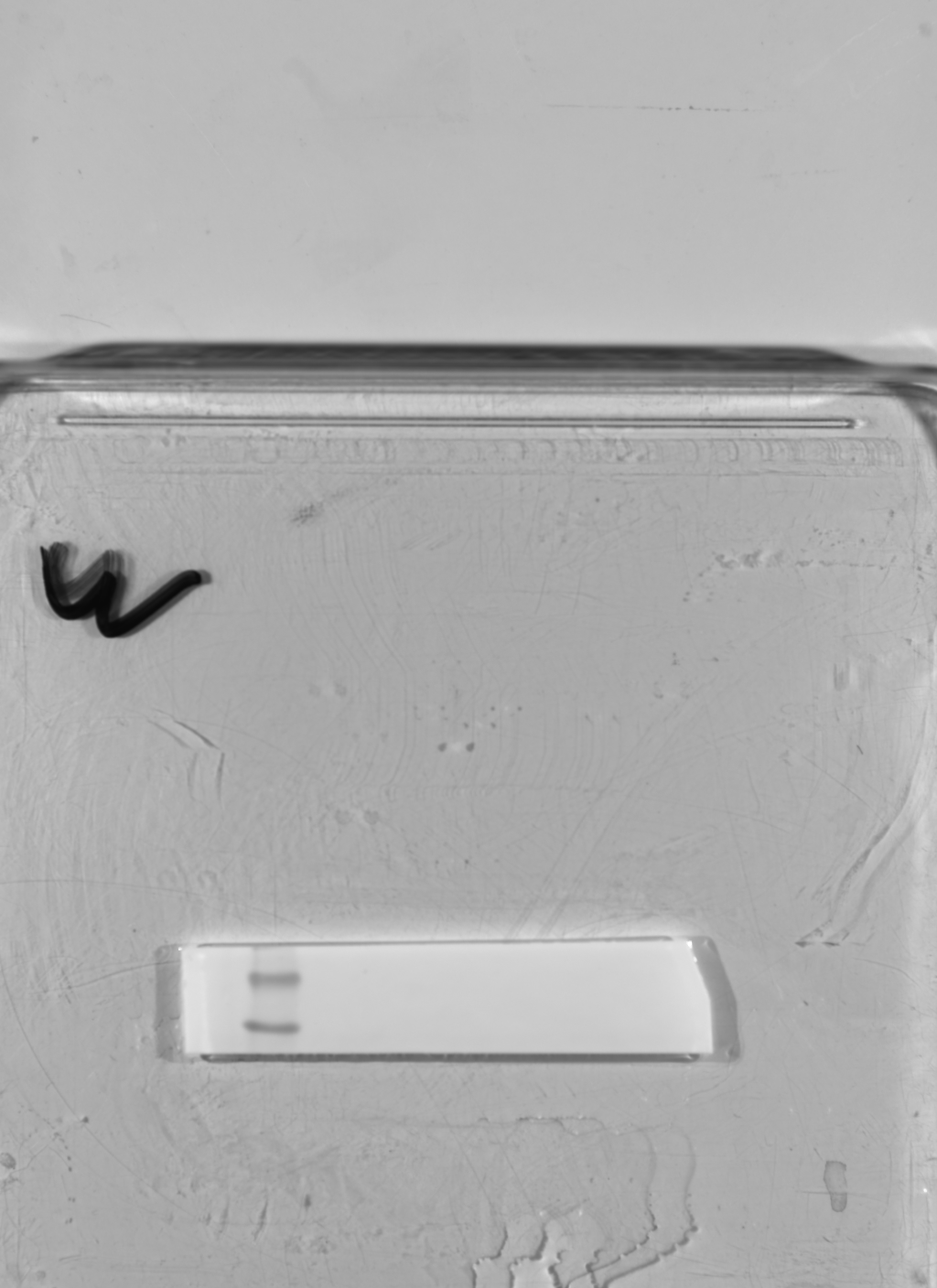

Supplement: Supplementary file 9 — Source data Fig. 4 [file 44321_2024_60_MOESM9_ESM.zip › Figure 4/4B/CN1/Western PKMYT1 28.5S/WSM 5 28.5S _Ch-Marker.tif]

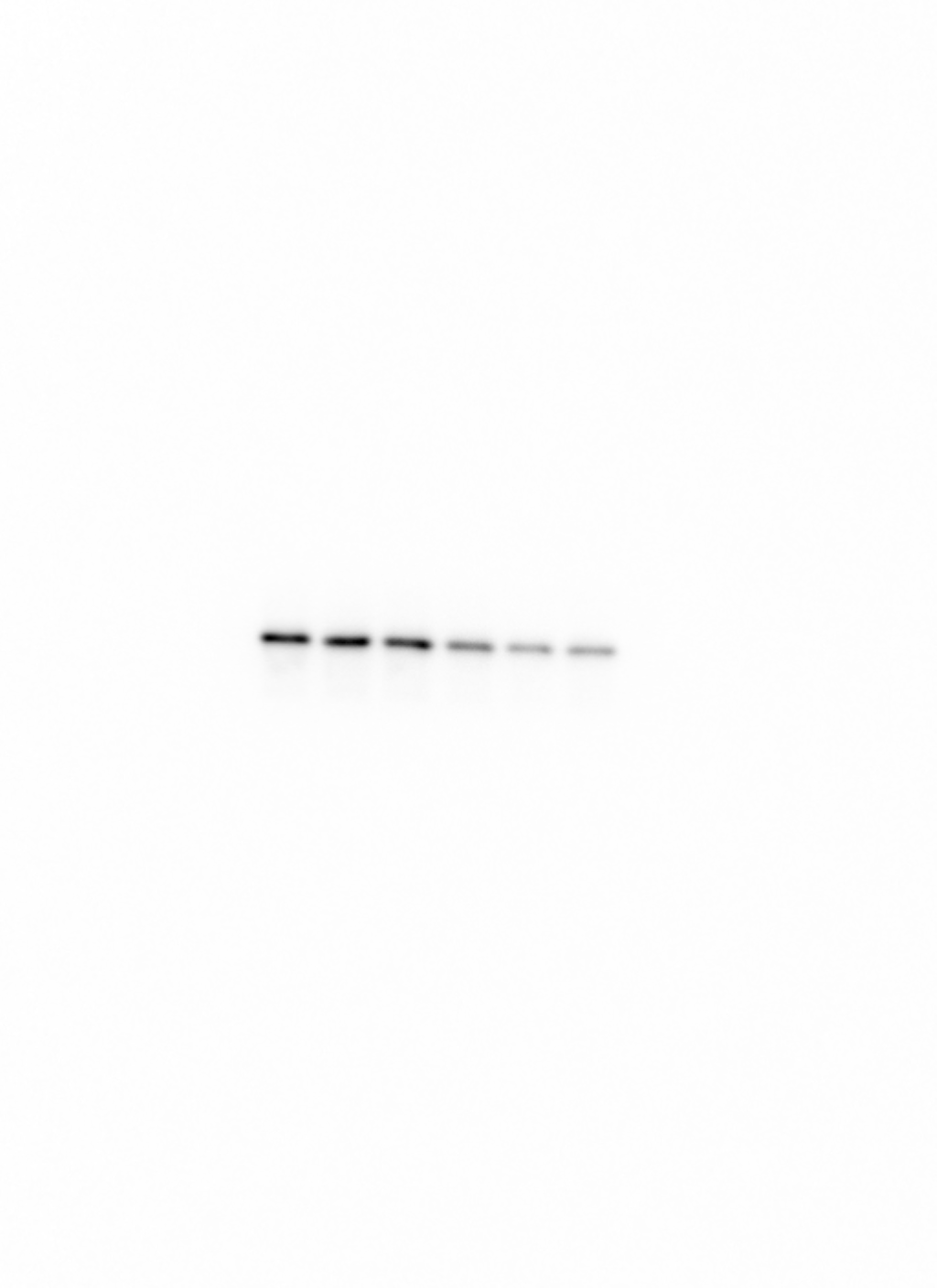

Supplement: Supplementary file 9 — Source data Fig. 4 [file 44321_2024_60_MOESM9_ESM.zip › Figure 4/4B/CN1/Western PLK1 0.2S/WSM 1 0.2S _Ch.tif]

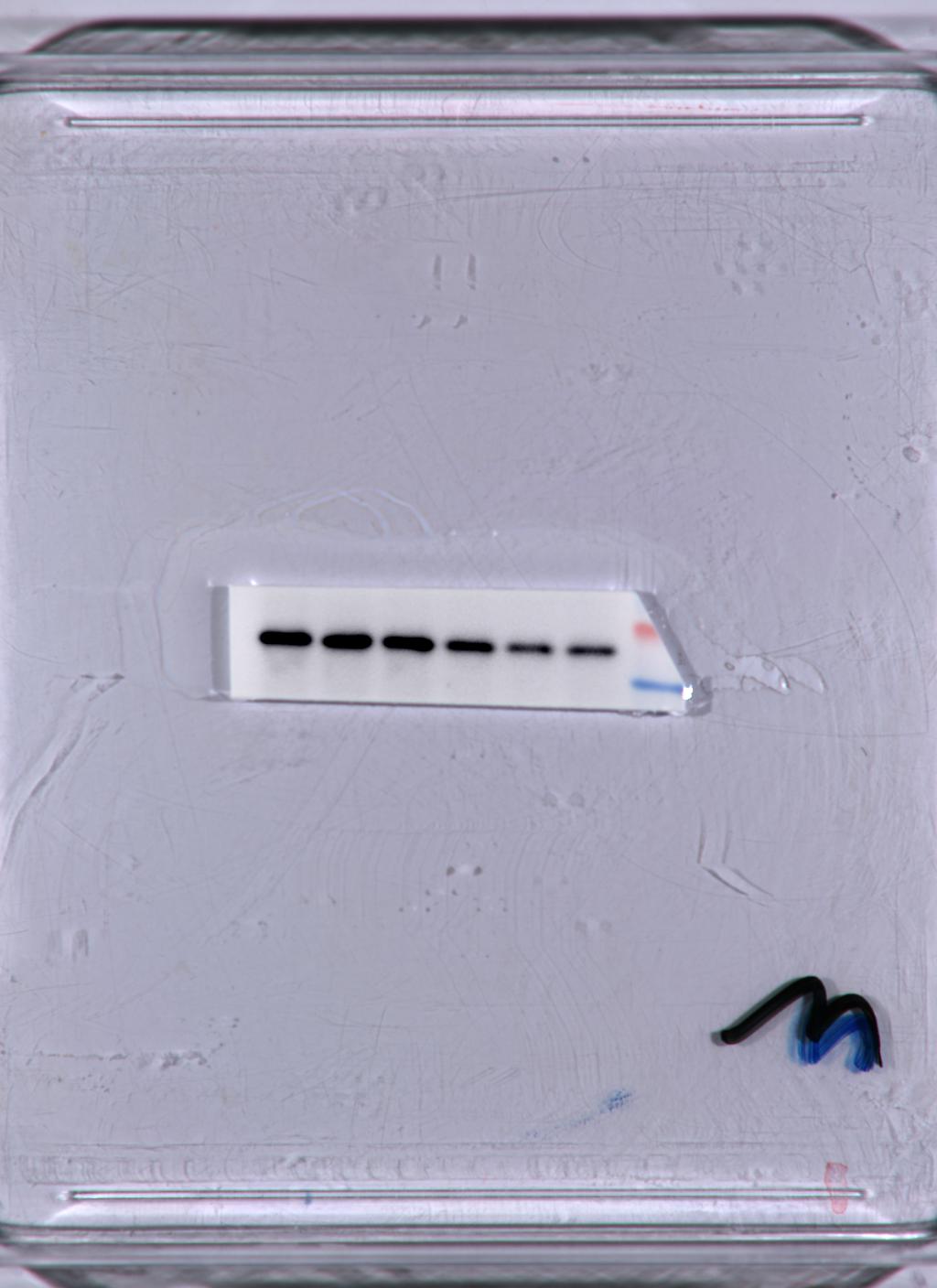

Supplement: Supplementary file 9 — Source data Fig. 4 [file 44321_2024_60_MOESM9_ESM.zip › Figure 4/4B/CN1/Western PLK1 0.2S/WSM 1 0.2S _Ch+Marker.jpg]

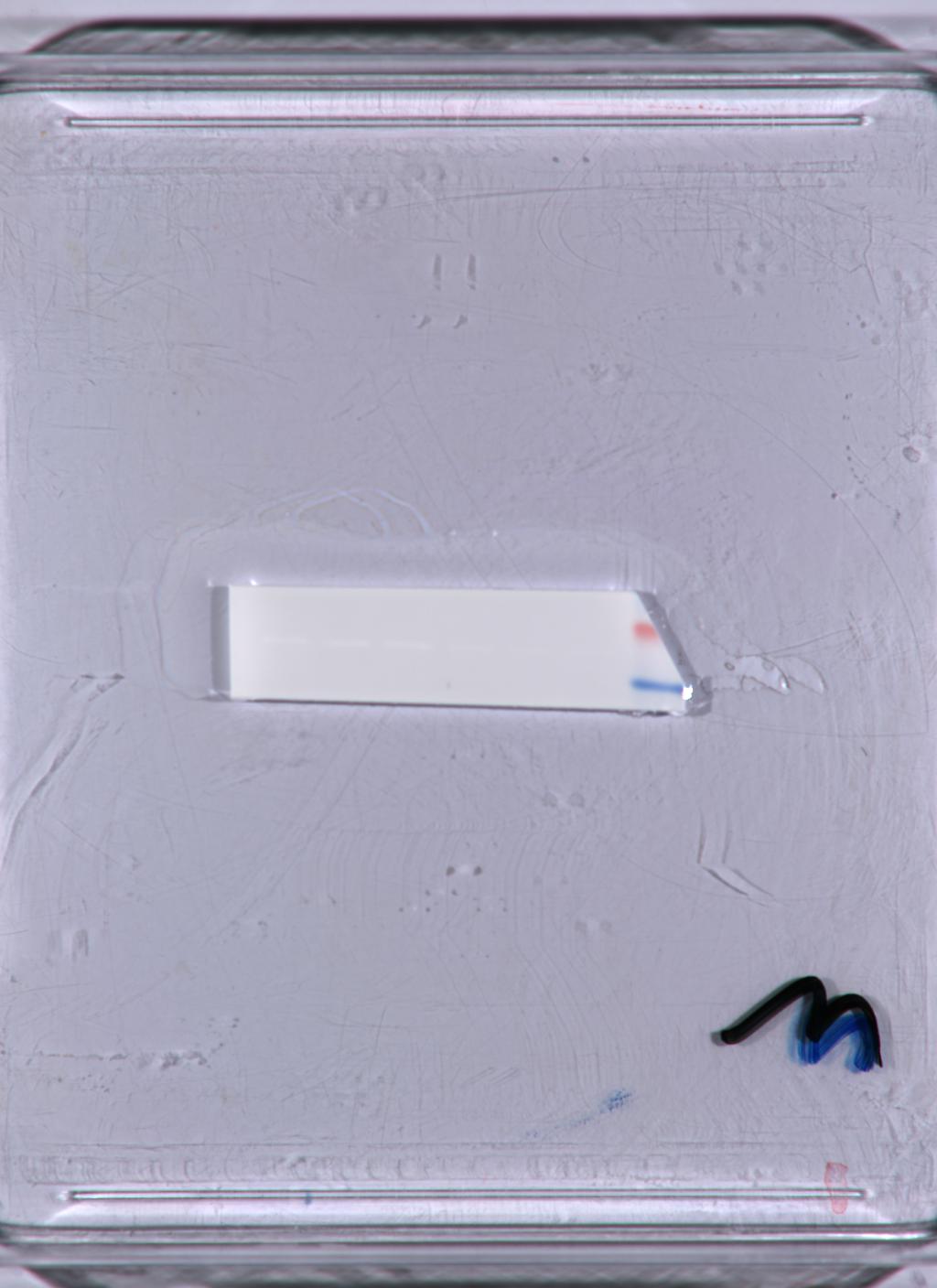

Supplement: Supplementary file 9 — Source data Fig. 4 [file 44321_2024_60_MOESM9_ESM.zip › Figure 4/4B/CN1/Western PLK1 0.2S/WSM 1 0.2S _Ch-Marker.jpg]

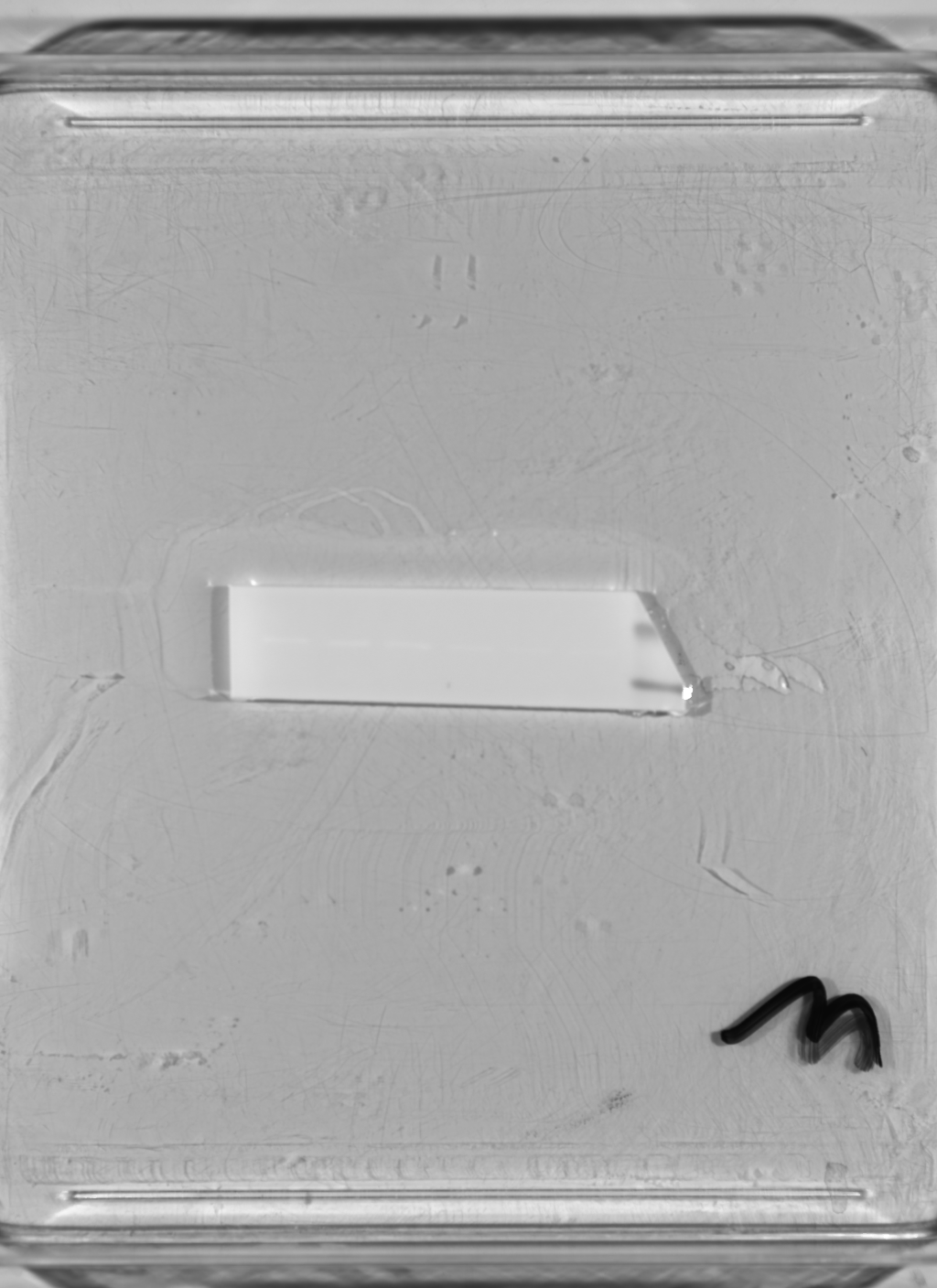

Supplement: Supplementary file 9 — Source data Fig. 4 [file 44321_2024_60_MOESM9_ESM.zip › Figure 4/4B/CN1/Western PLK1 0.2S/WSM 1 0.2S _Ch-Marker.tif]

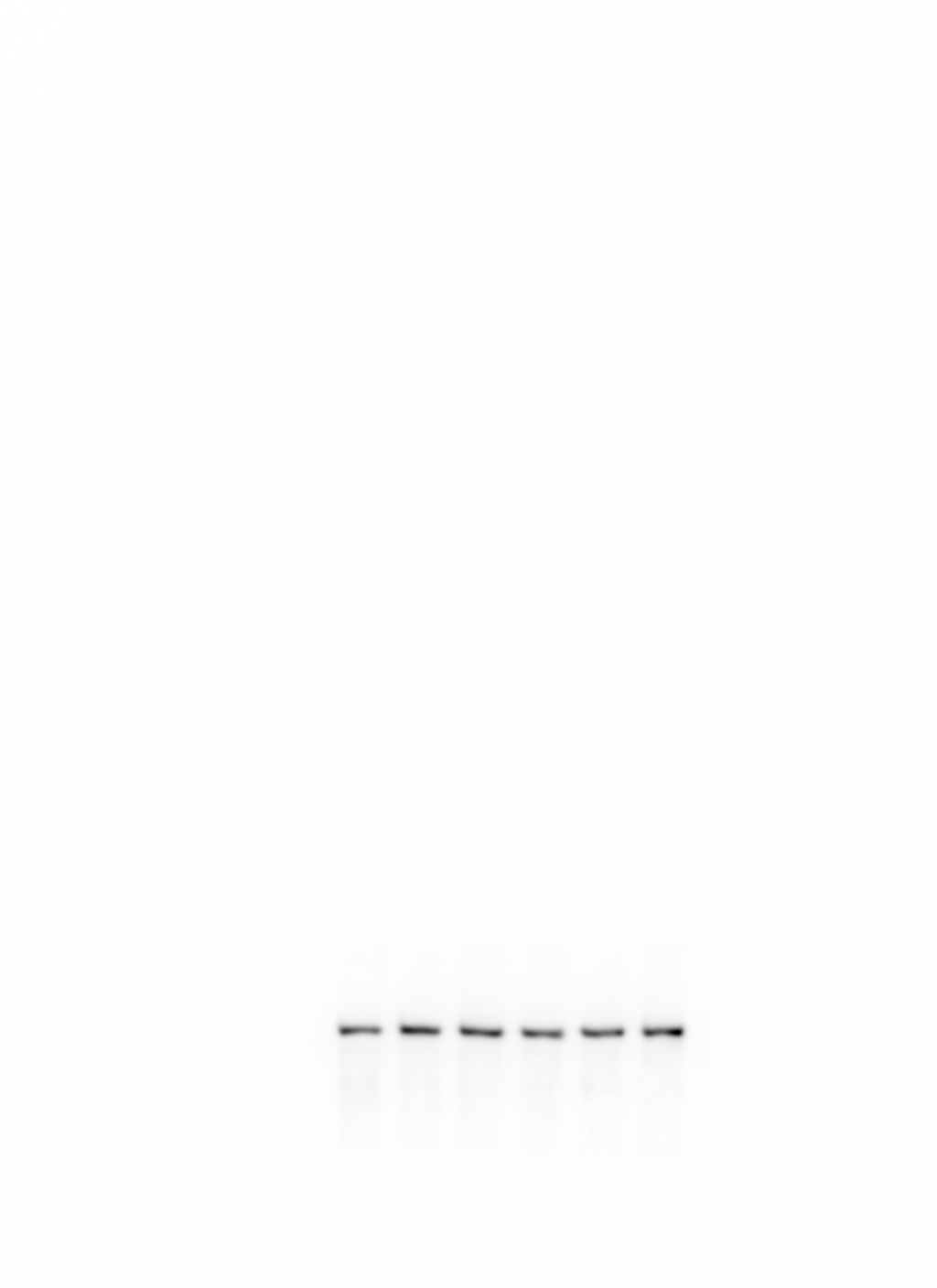

Supplement: Supplementary file 9 — Source data Fig. 4 [file 44321_2024_60_MOESM9_ESM.zip › Figure 4/4B/CN1/Western PRKDC 0.2S/WSM 3 0.2S _Ch.tif]

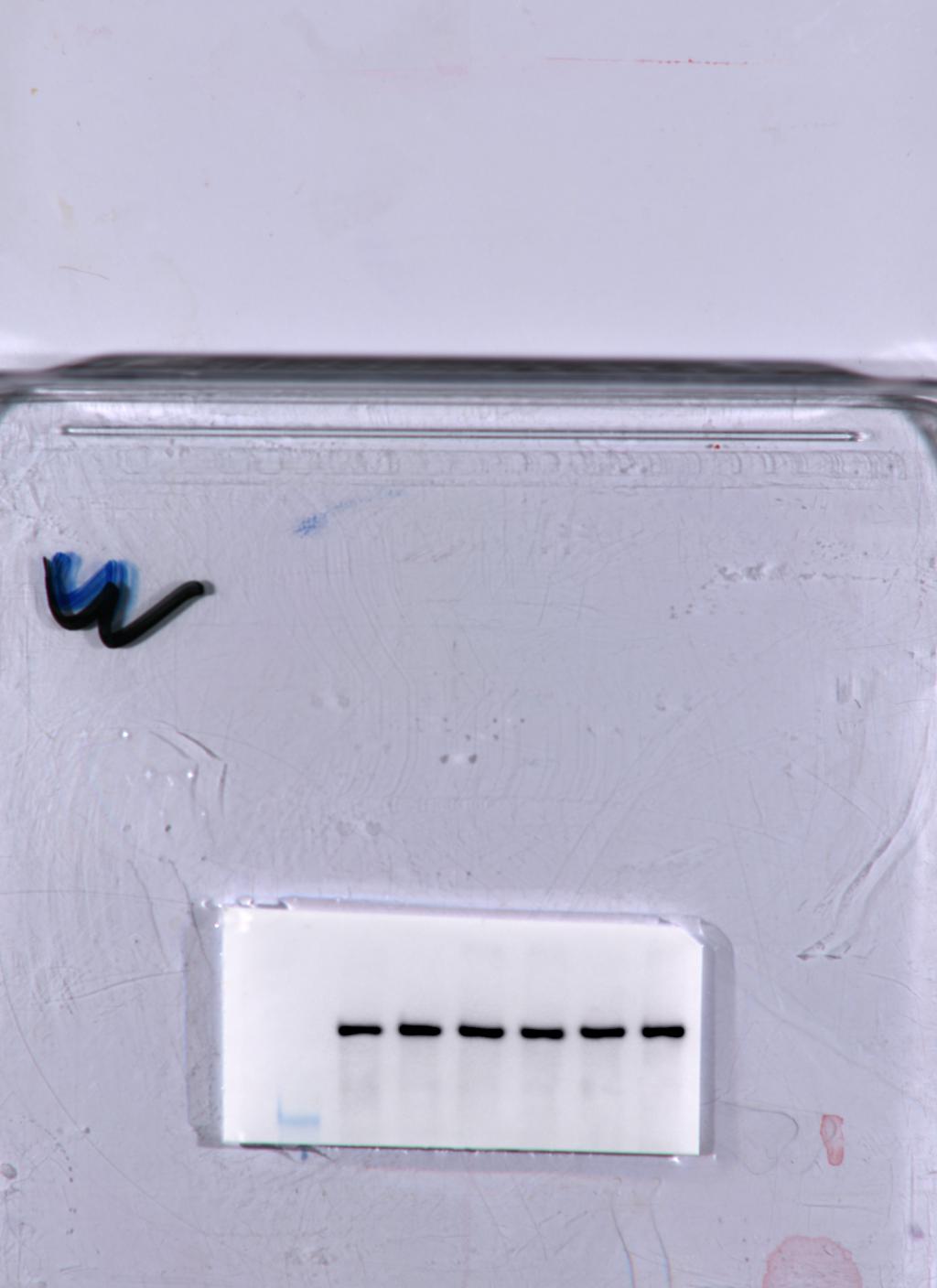

Supplement: Supplementary file 9 — Source data Fig. 4 [file 44321_2024_60_MOESM9_ESM.zip › Figure 4/4B/CN1/Western PRKDC 0.2S/WSM 3 0.2S _Ch+Marker.jpg]

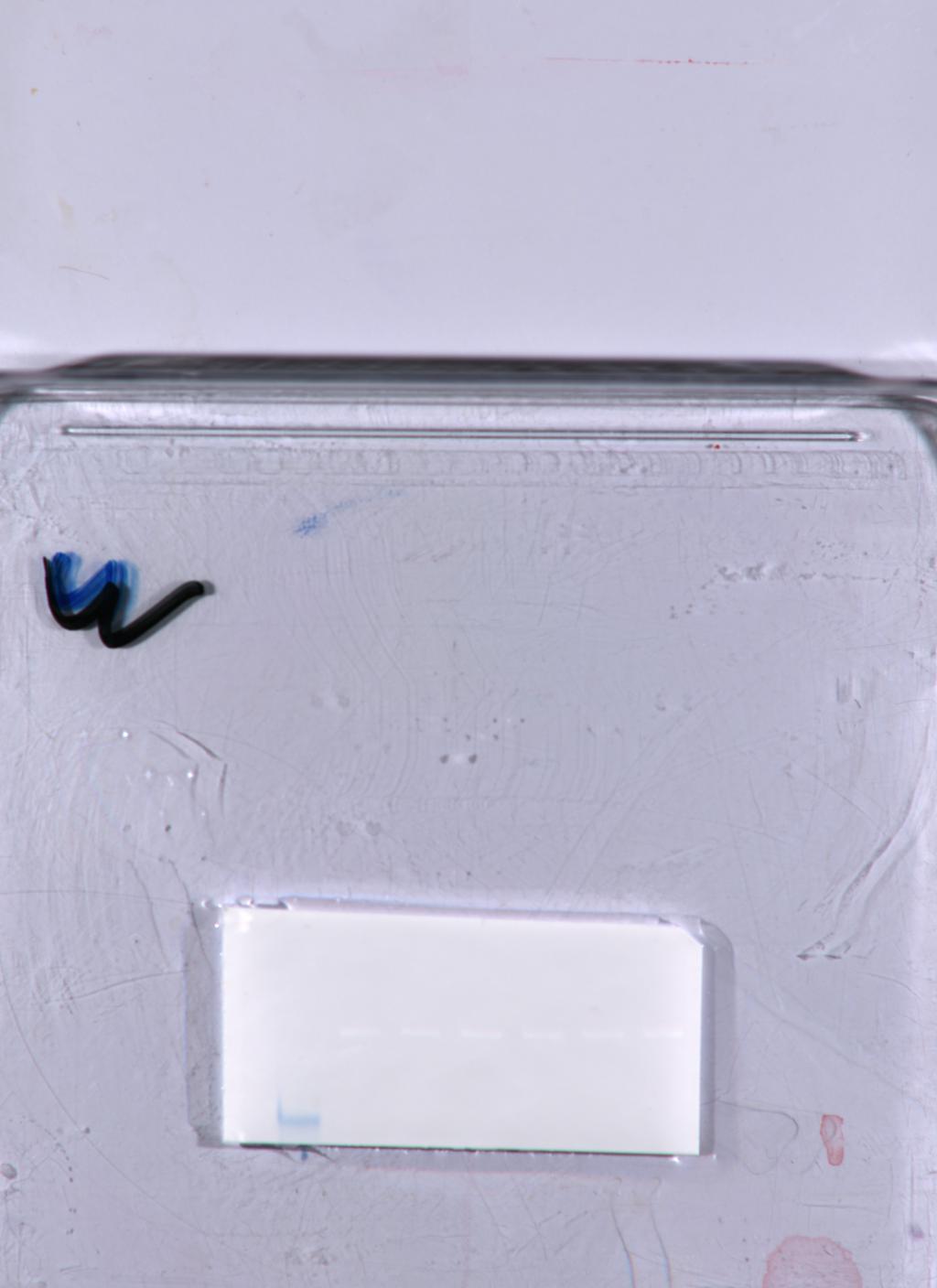

Supplement: Supplementary file 9 — Source data Fig. 4 [file 44321_2024_60_MOESM9_ESM.zip › Figure 4/4B/CN1/Western PRKDC 0.2S/WSM 3 0.2S _Ch-Marker.jpg]

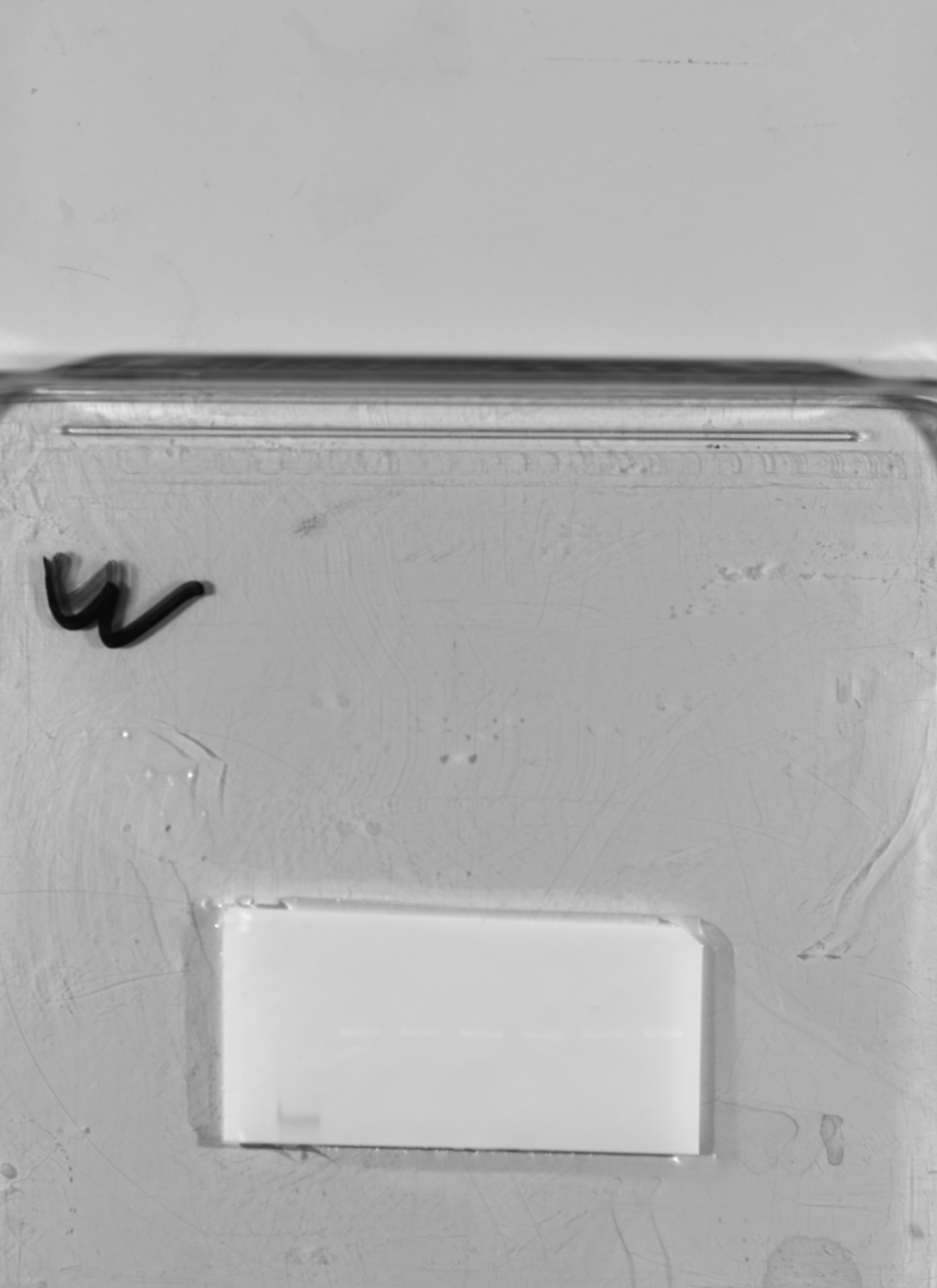

Supplement: Supplementary file 9 — Source data Fig. 4 [file 44321_2024_60_MOESM9_ESM.zip › Figure 4/4B/CN1/Western PRKDC 0.2S/WSM 3 0.2S _Ch-Marker.tif]

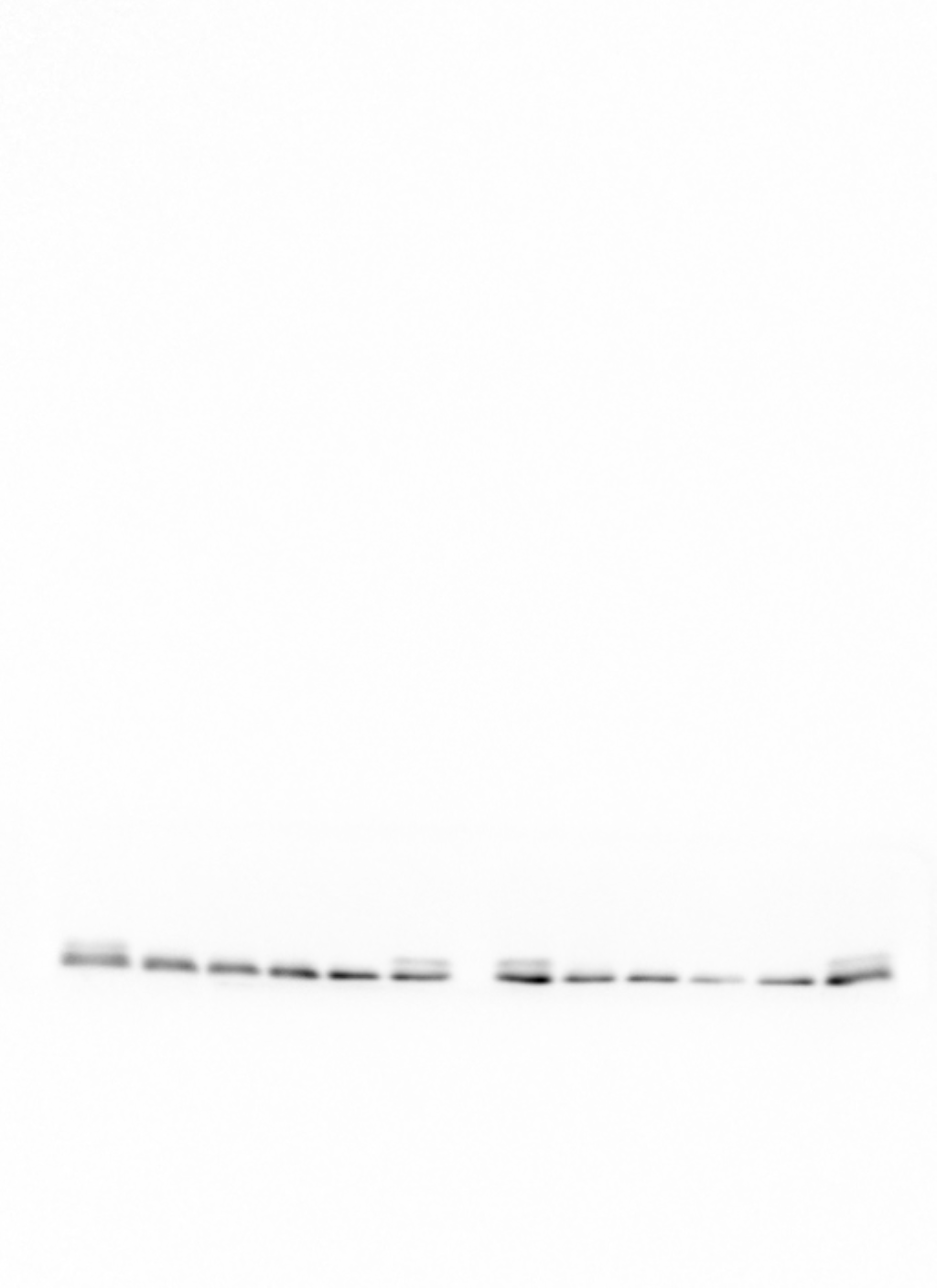

Supplement: Supplementary file 9 — Source data Fig. 4 [file 44321_2024_60_MOESM9_ESM.zip › Figure 4/4B/YAPC/Western CDK1/2 1st CDK1 12.5 _Ch.tif]

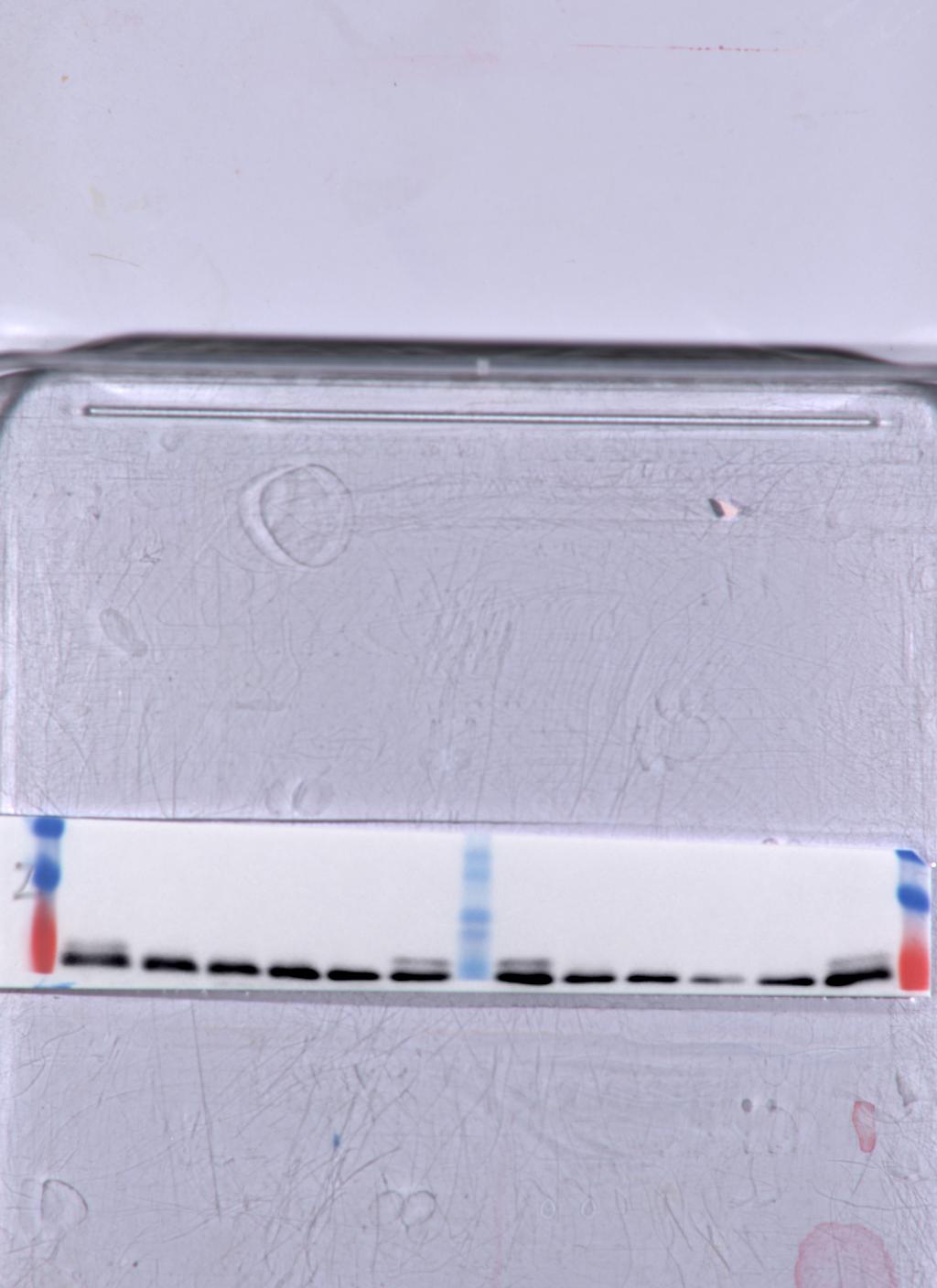

Supplement: Supplementary file 9 — Source data Fig. 4 [file 44321_2024_60_MOESM9_ESM.zip › Figure 4/4B/YAPC/Western CDK1/2 1st CDK1 12.5 _Ch+Marker.jpg]

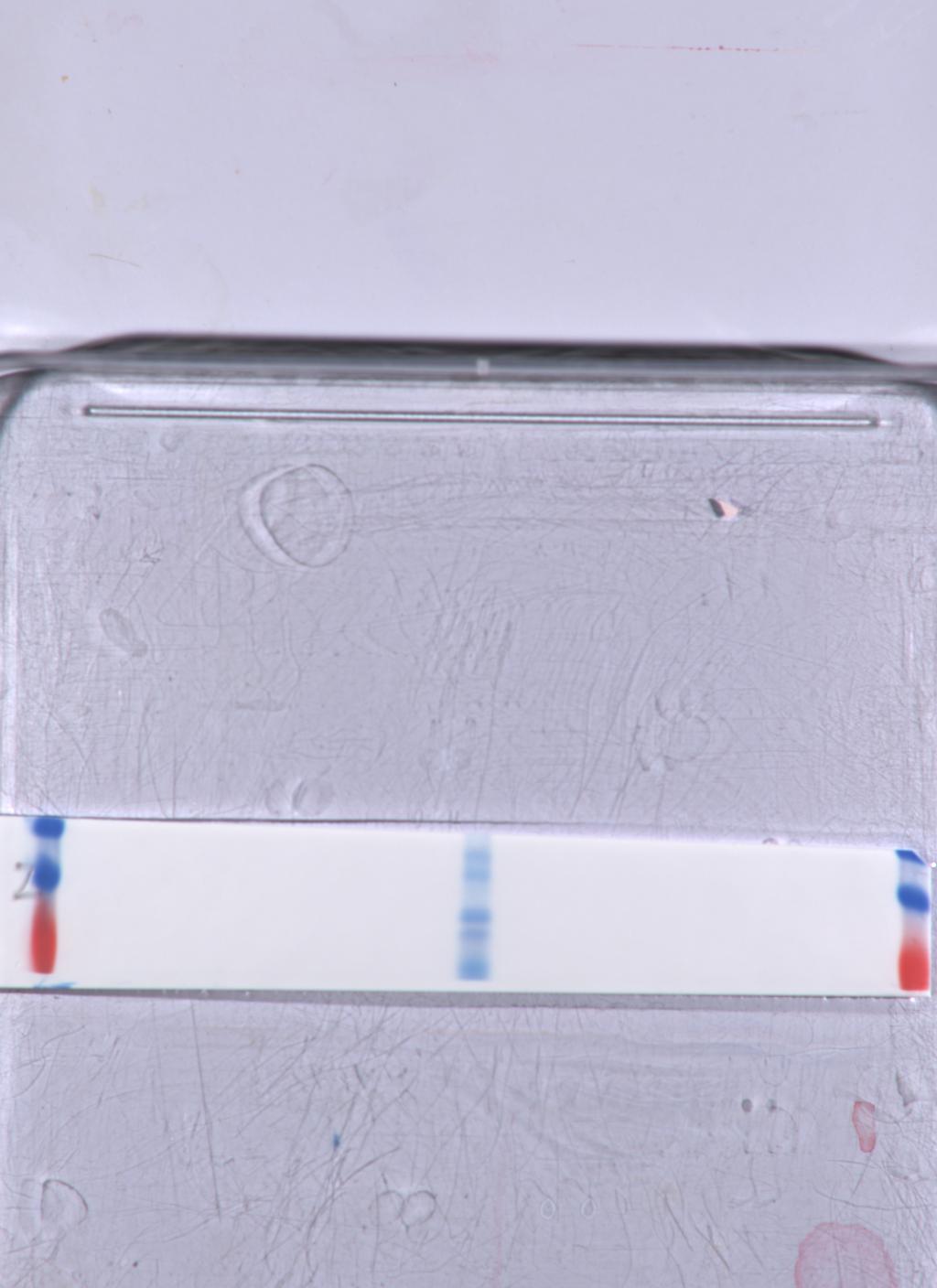

Supplement: Supplementary file 9 — Source data Fig. 4 [file 44321_2024_60_MOESM9_ESM.zip › Figure 4/4B/YAPC/Western CDK1/2 1st CDK1 12.5 _Ch-Marker.jpg]

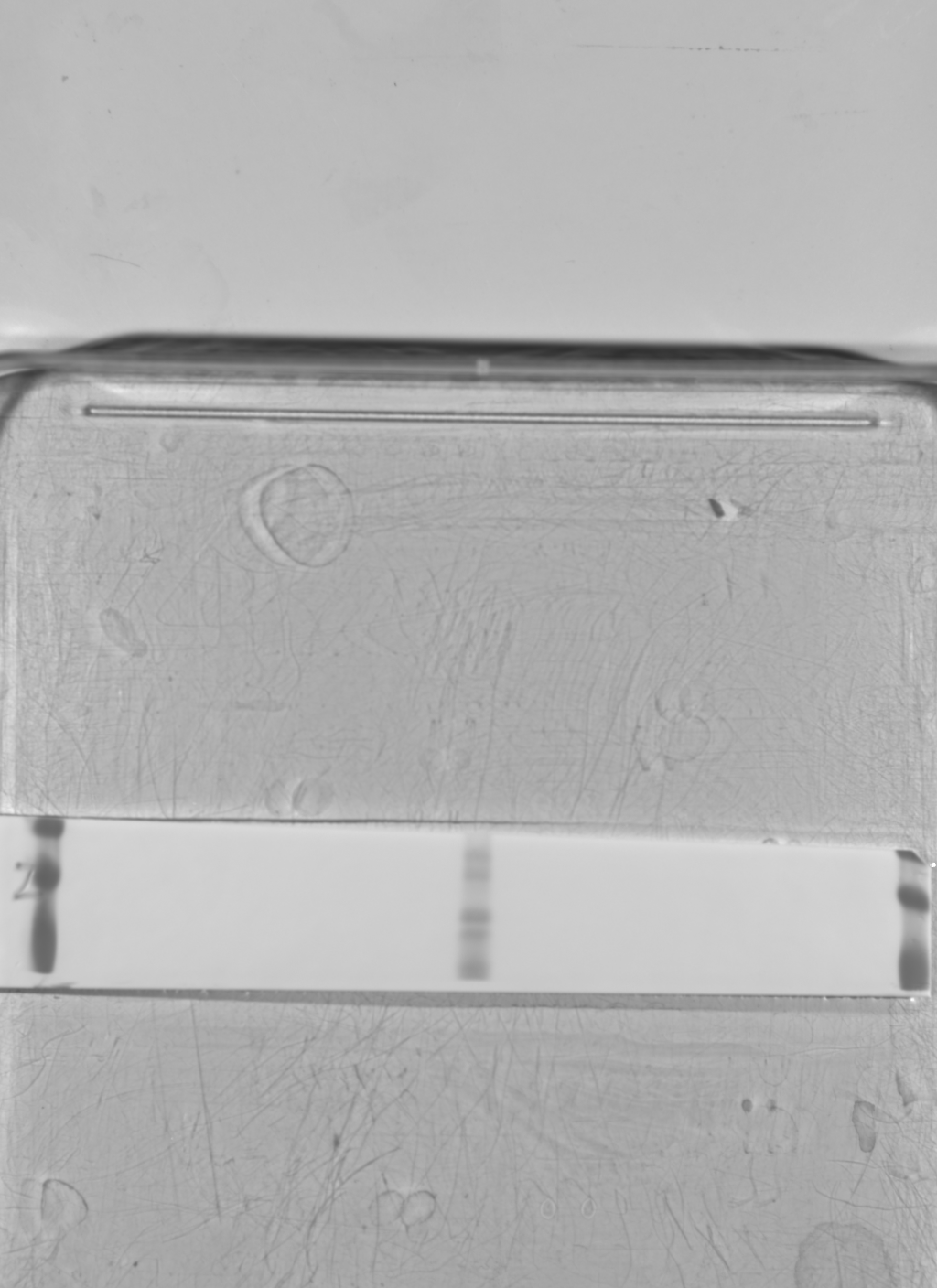

Supplement: Supplementary file 9 — Source data Fig. 4 [file 44321_2024_60_MOESM9_ESM.zip › Figure 4/4B/YAPC/Western CDK1/2 1st CDK1 12.5 _Ch-Marker.tif]

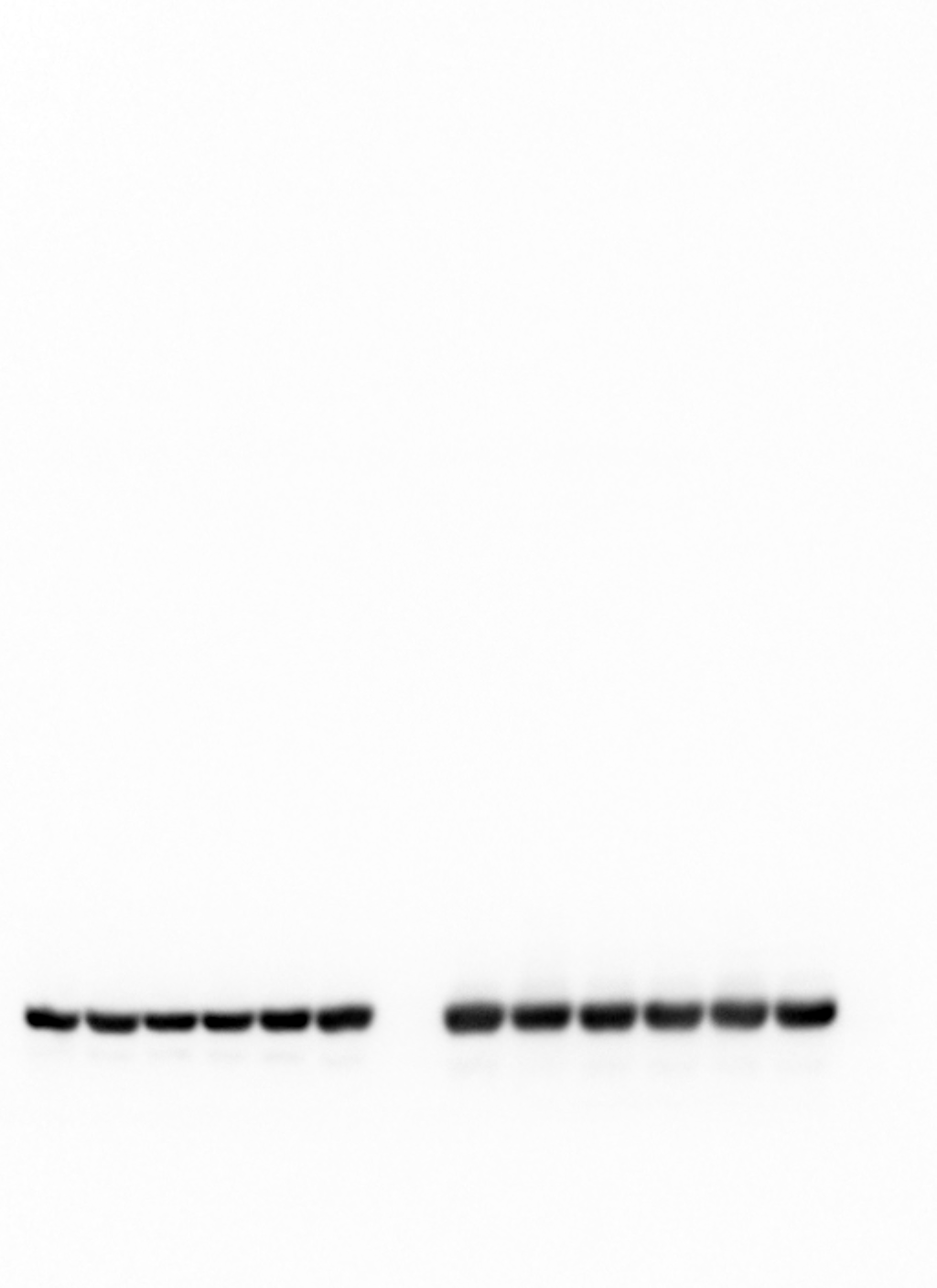

Supplement: Supplementary file 9 — Source data Fig. 4 [file 44321_2024_60_MOESM9_ESM.zip › Figure 4/4B/YAPC/Western GAPDH/2 2nd GAP 0.3 _Ch.tif]

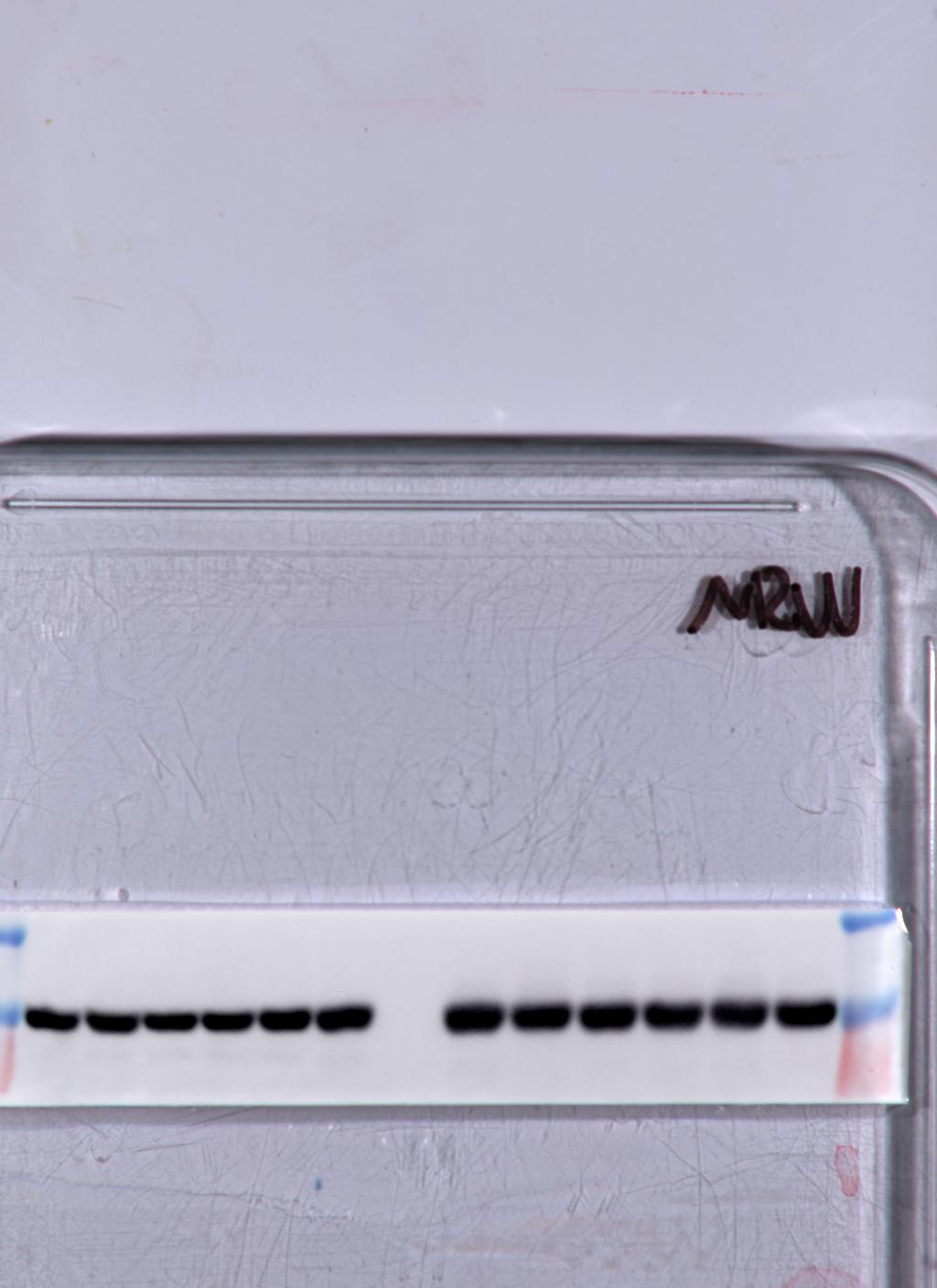

Supplement: Supplementary file 9 — Source data Fig. 4 [file 44321_2024_60_MOESM9_ESM.zip › Figure 4/4B/YAPC/Western GAPDH/2 2nd GAP 0.3 _Ch+Marker.jpg]

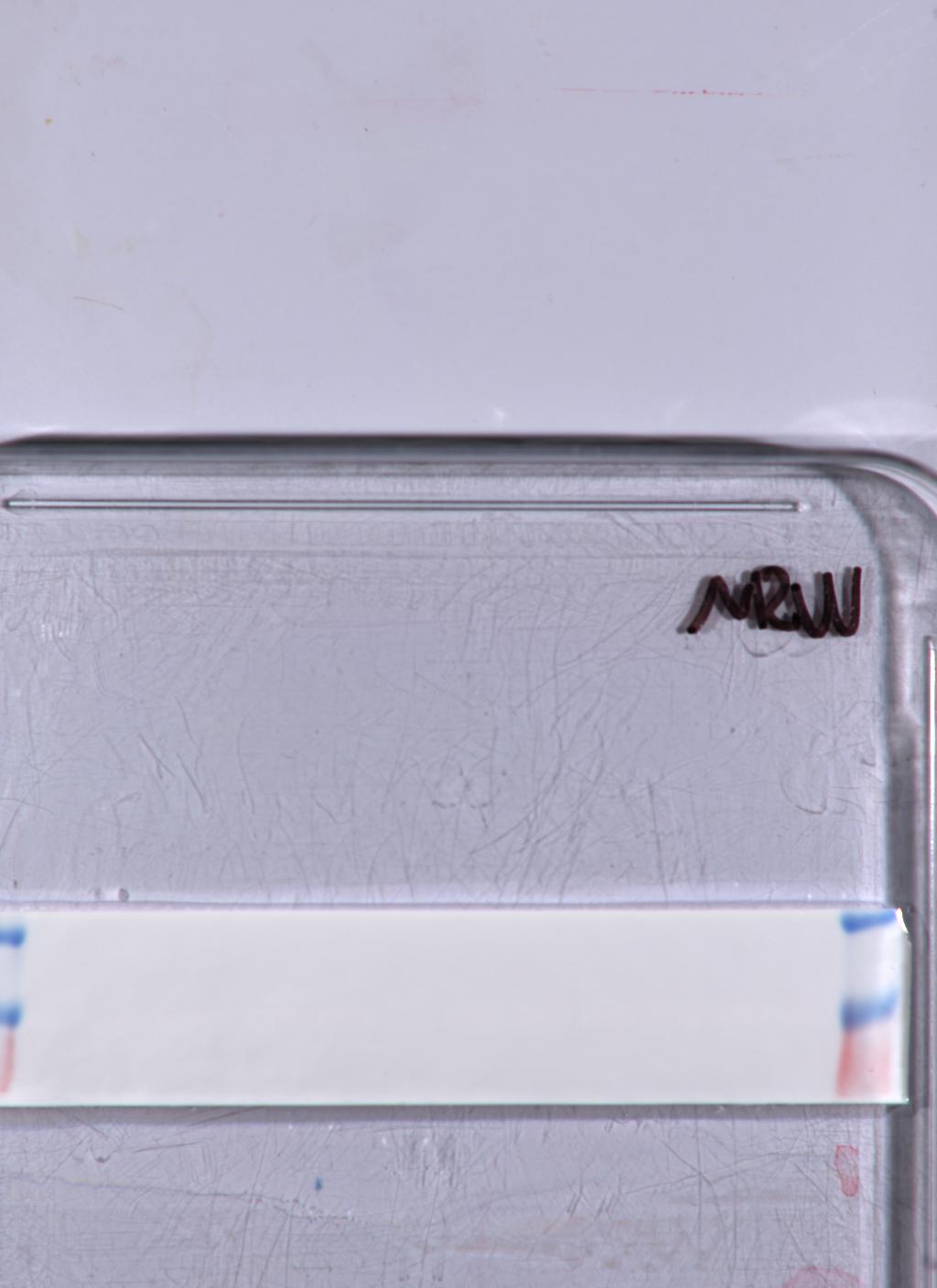

Supplement: Supplementary file 9 — Source data Fig. 4 [file 44321_2024_60_MOESM9_ESM.zip › Figure 4/4B/YAPC/Western GAPDH/2 2nd GAP 0.3 _Ch-Marker.jpg]

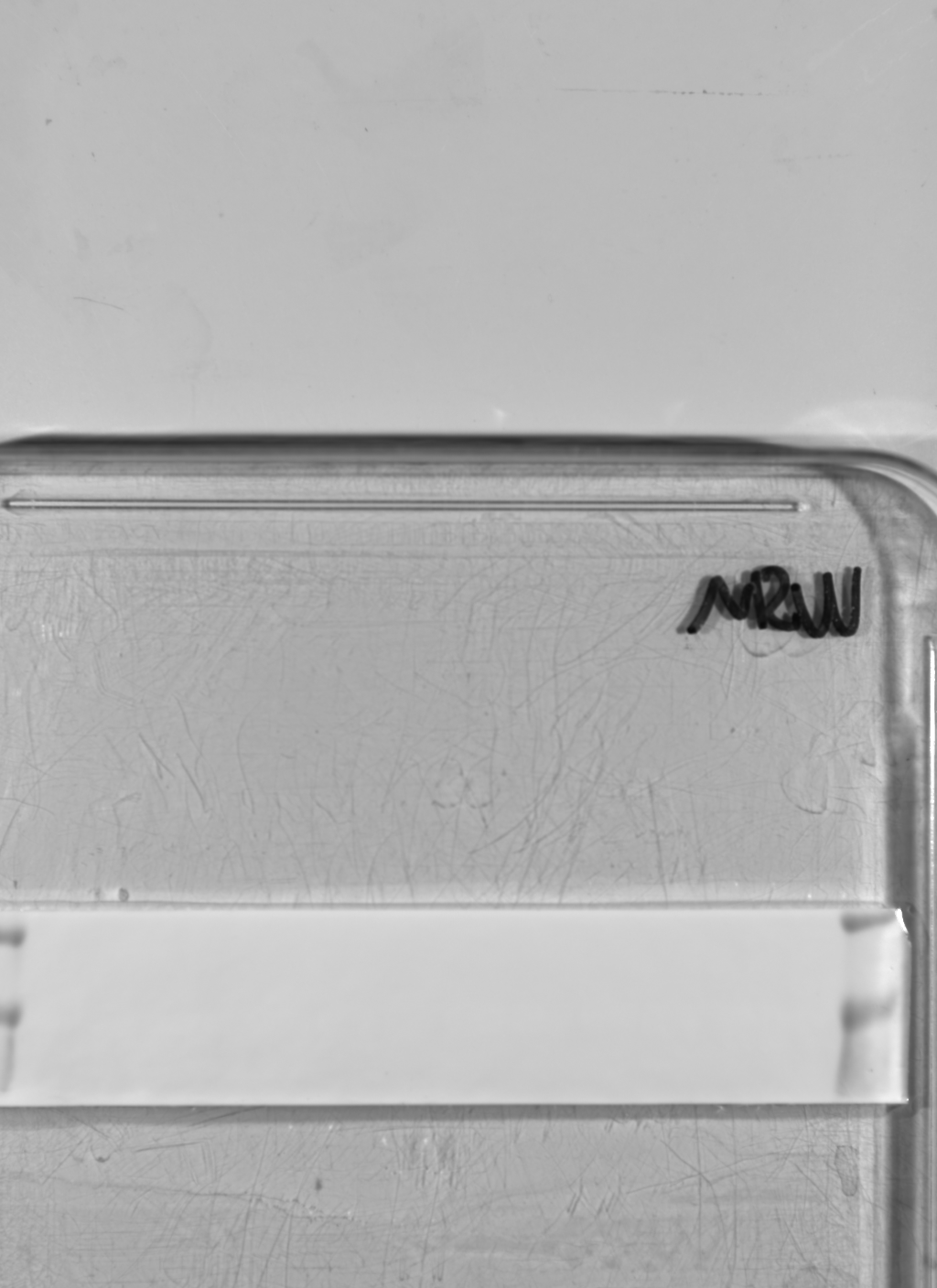

Supplement: Supplementary file 9 — Source data Fig. 4 [file 44321_2024_60_MOESM9_ESM.zip › Figure 4/4B/YAPC/Western GAPDH/2 2nd GAP 0.3 _Ch-Marker.tif]

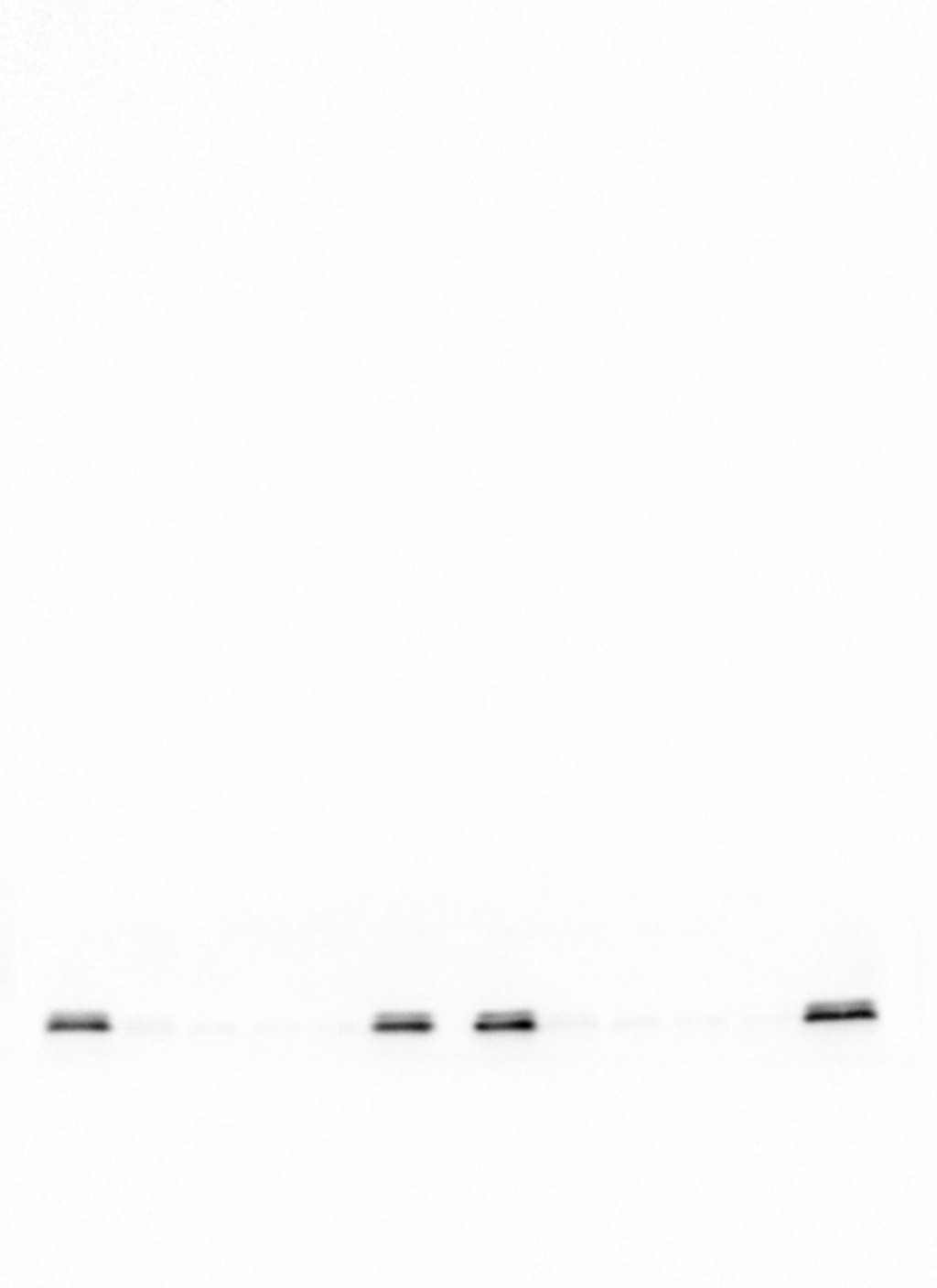

Supplement: Supplementary file 9 — Source data Fig. 4 [file 44321_2024_60_MOESM9_ESM.zip › Figure 4/4B/YAPC/Western phoCDK1/2 1st phoCDK1 18.4 _Ch.tif]

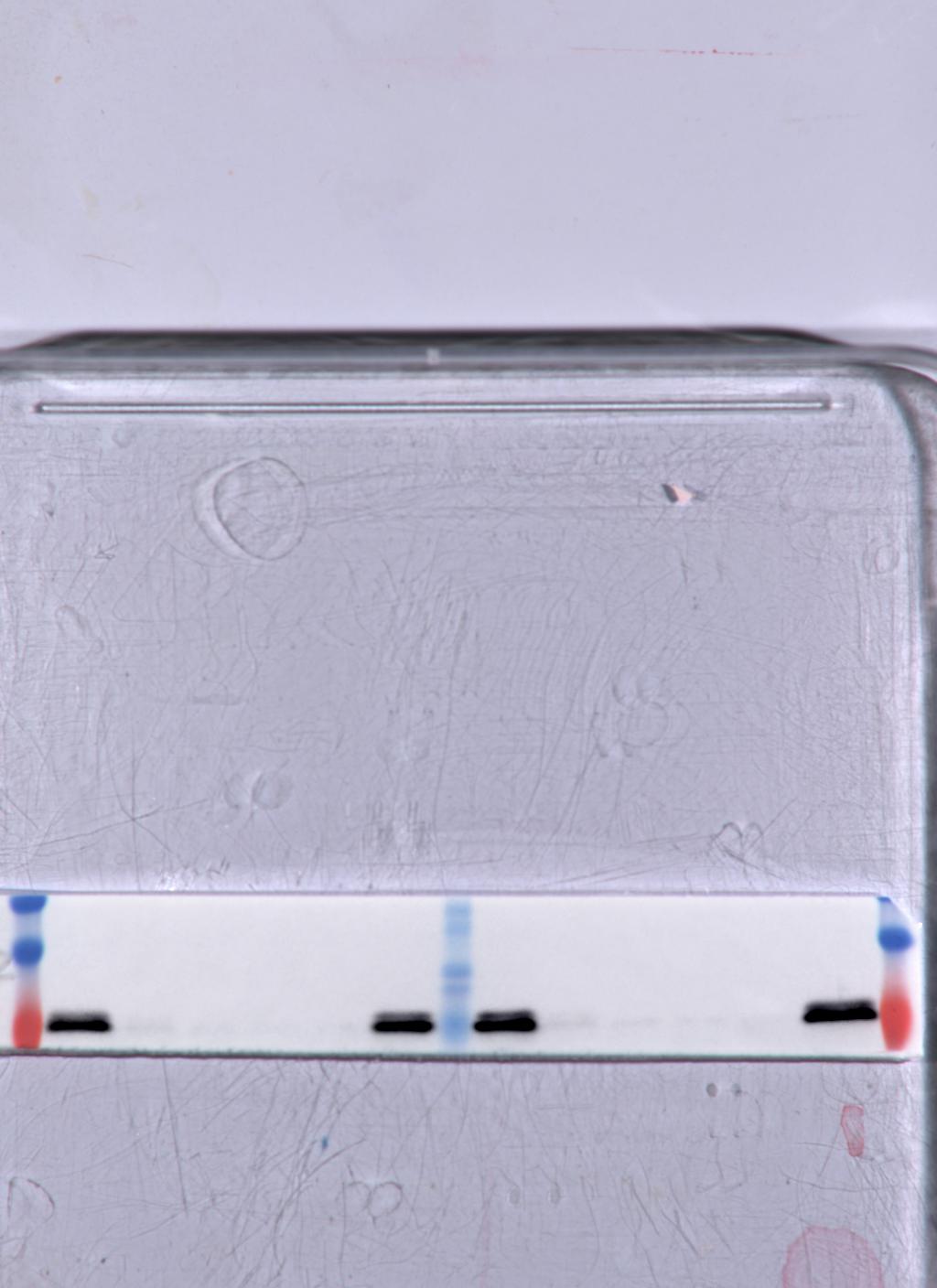

Supplement: Supplementary file 9 — Source data Fig. 4 [file 44321_2024_60_MOESM9_ESM.zip › Figure 4/4B/YAPC/Western phoCDK1/2 1st phoCDK1 18.4 _Ch+Marker.jpg]

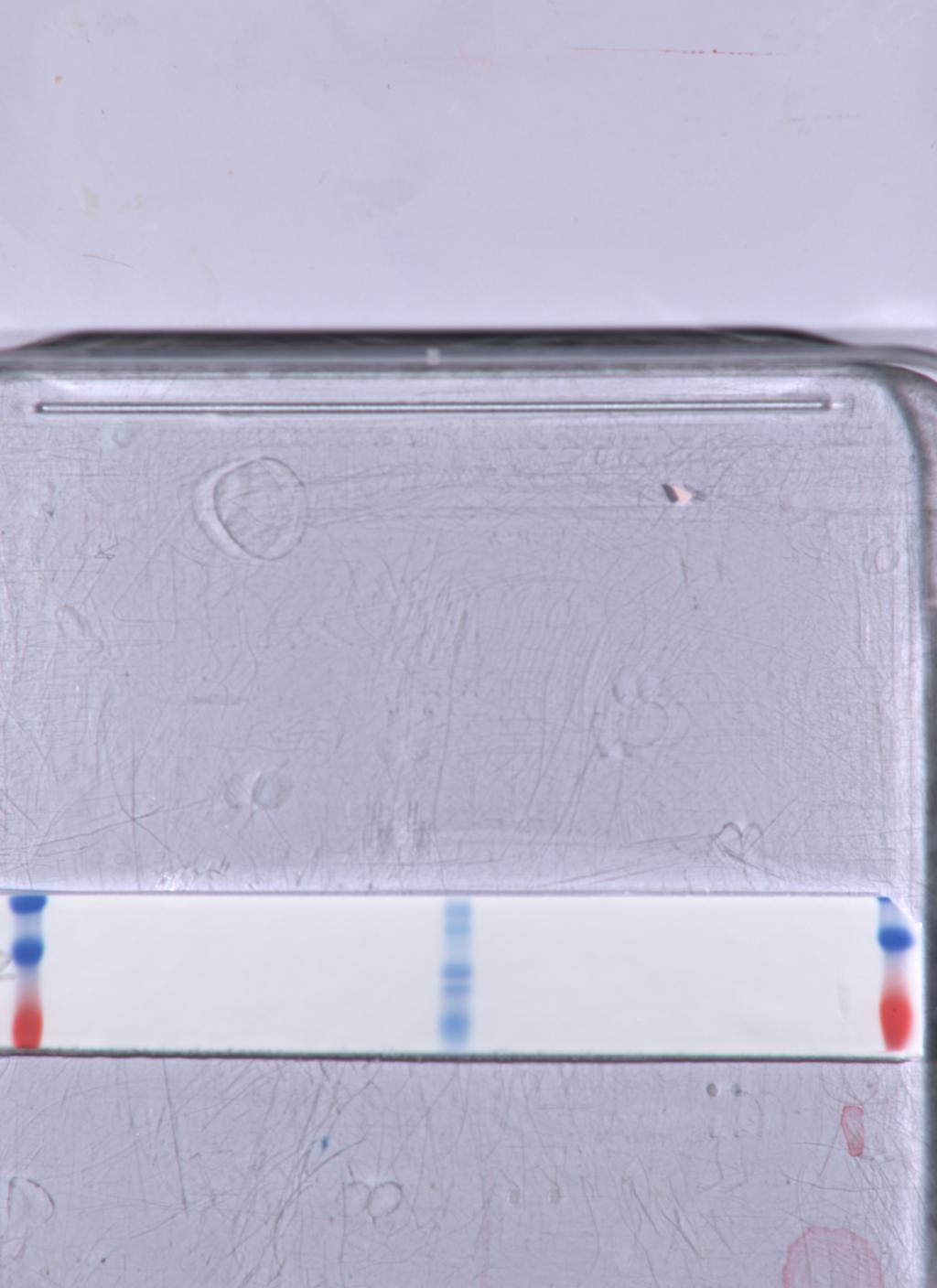

Supplement: Supplementary file 9 — Source data Fig. 4 [file 44321_2024_60_MOESM9_ESM.zip › Figure 4/4B/YAPC/Western phoCDK1/2 1st phoCDK1 18.4 _Ch-Marker.jpg]

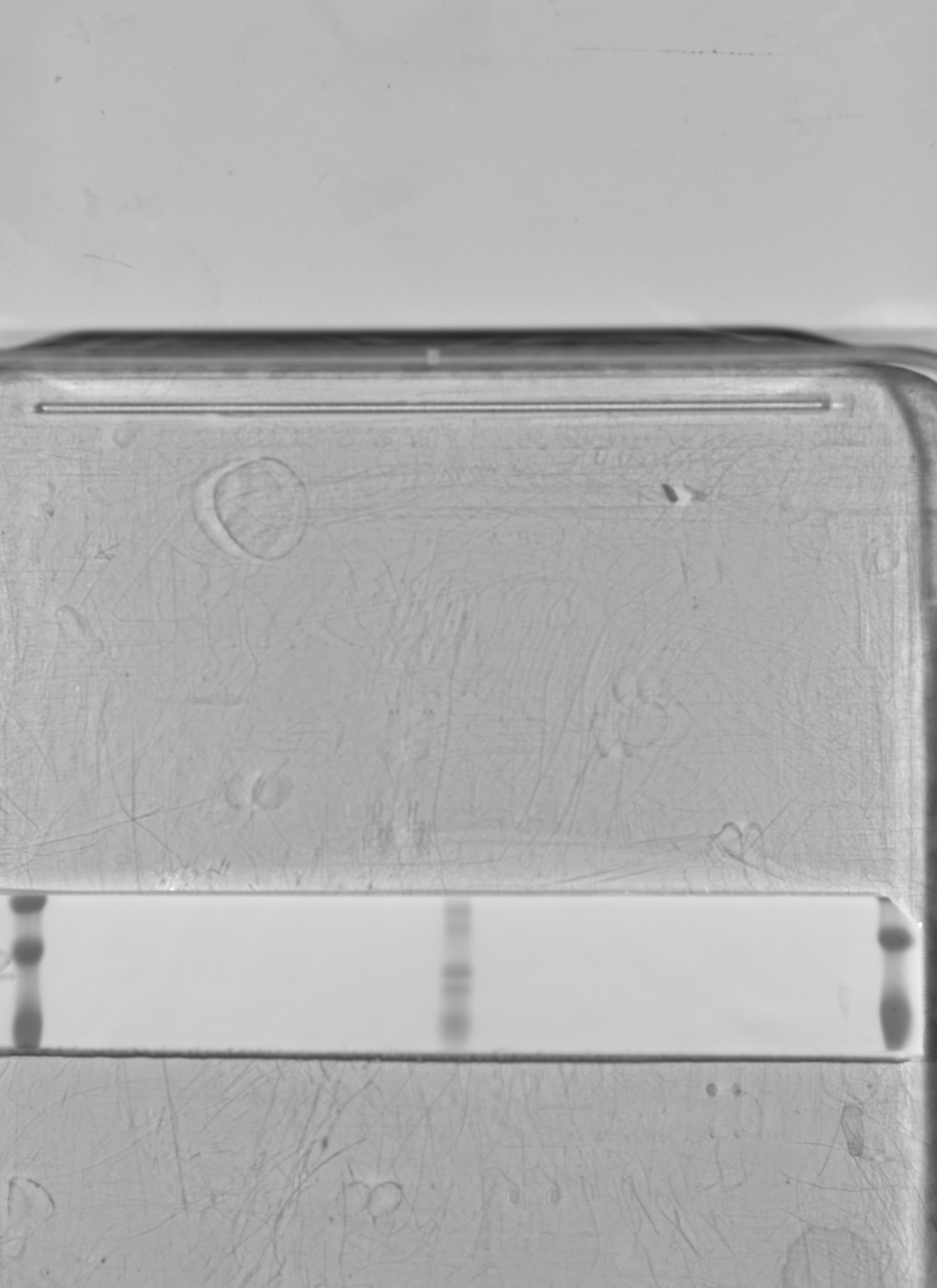

Supplement: Supplementary file 9 — Source data Fig. 4 [file 44321_2024_60_MOESM9_ESM.zip › Figure 4/4B/YAPC/Western phoCDK1/2 1st phoCDK1 18.4 _Ch-Marker.tif]

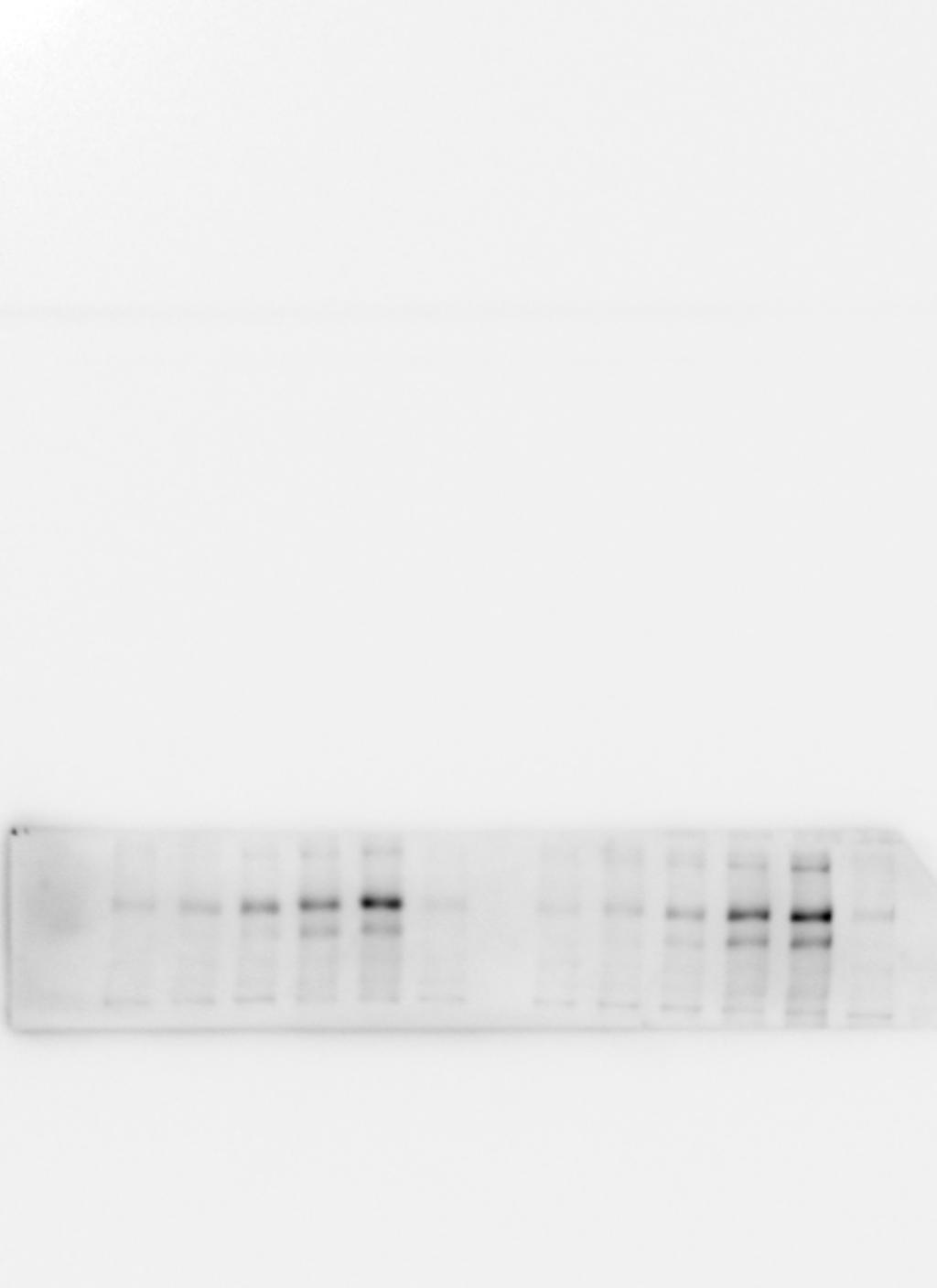

Supplement: Supplementary file 9 — Source data Fig. 4 [file 44321_2024_60_MOESM9_ESM.zip › Figure 4/4B/YAPC/Western phoPRKDC/2 1st phoPRK 3.6 _Ch.tif]

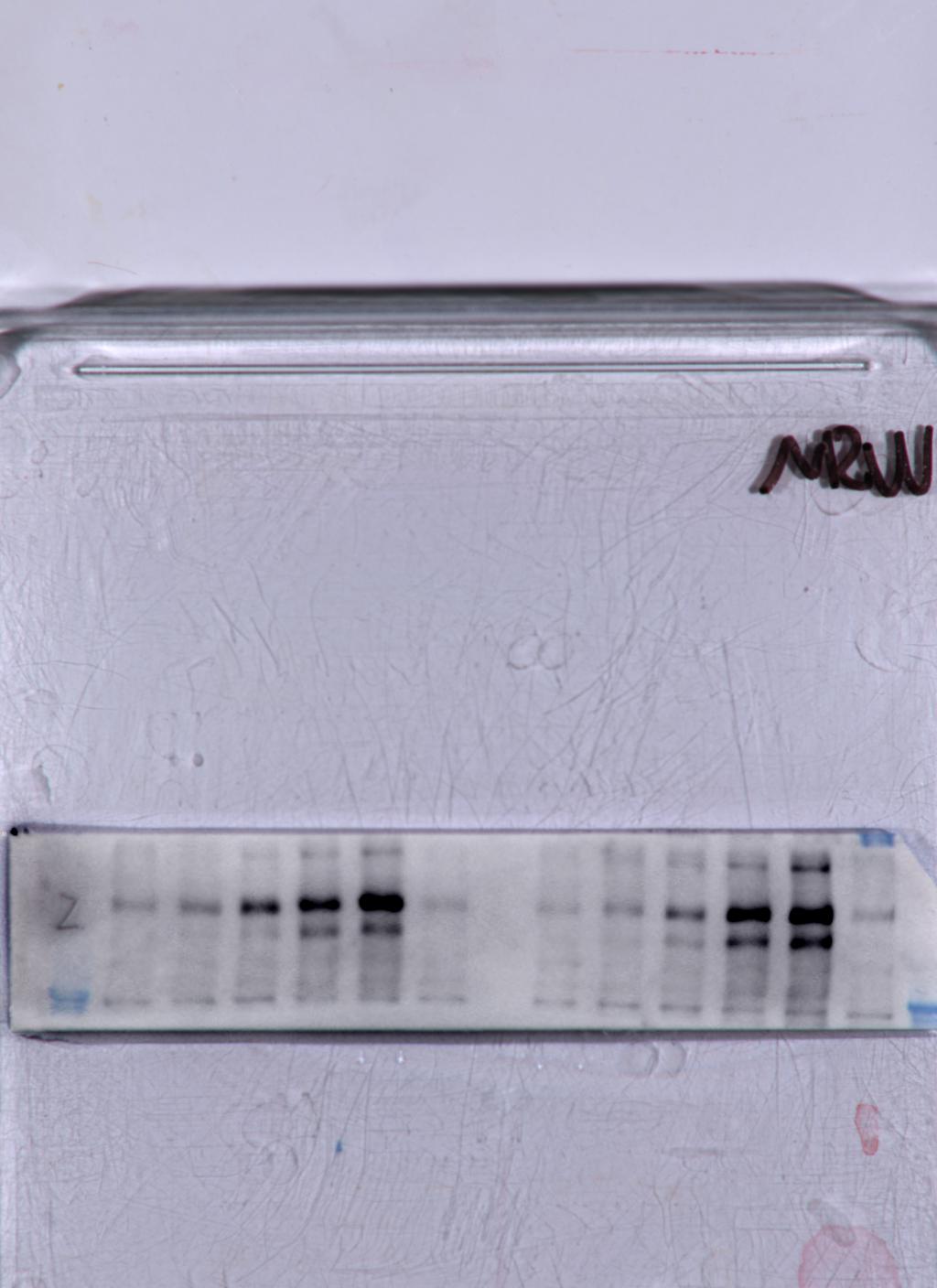

Supplement: Supplementary file 9 — Source data Fig. 4 [file 44321_2024_60_MOESM9_ESM.zip › Figure 4/4B/YAPC/Western phoPRKDC/2 1st phoPRK 3.6 _Ch+Marker.jpg]

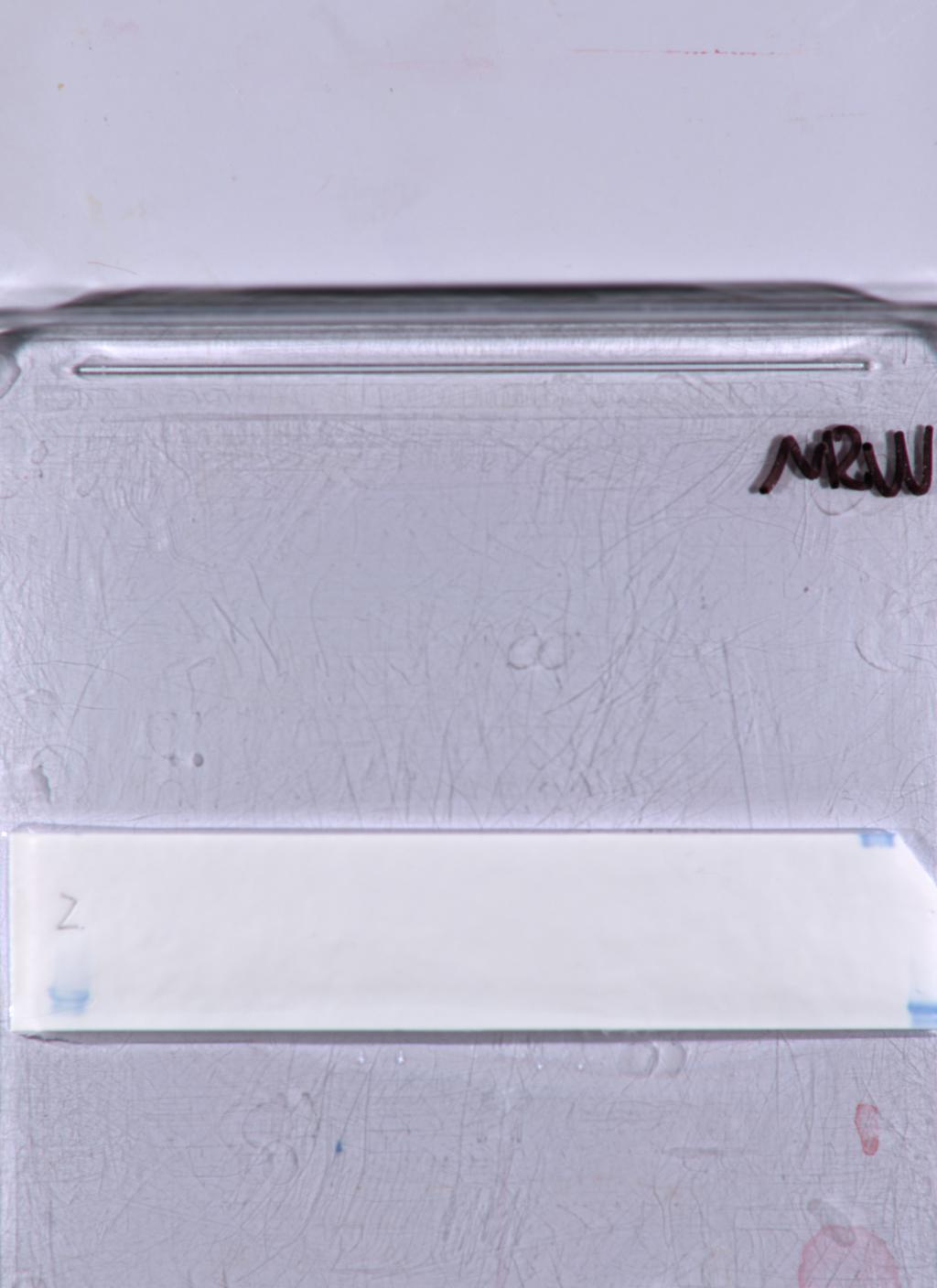

Supplement: Supplementary file 9 — Source data Fig. 4 [file 44321_2024_60_MOESM9_ESM.zip › Figure 4/4B/YAPC/Western phoPRKDC/2 1st phoPRK 3.6 _Ch-Marker.jpg]

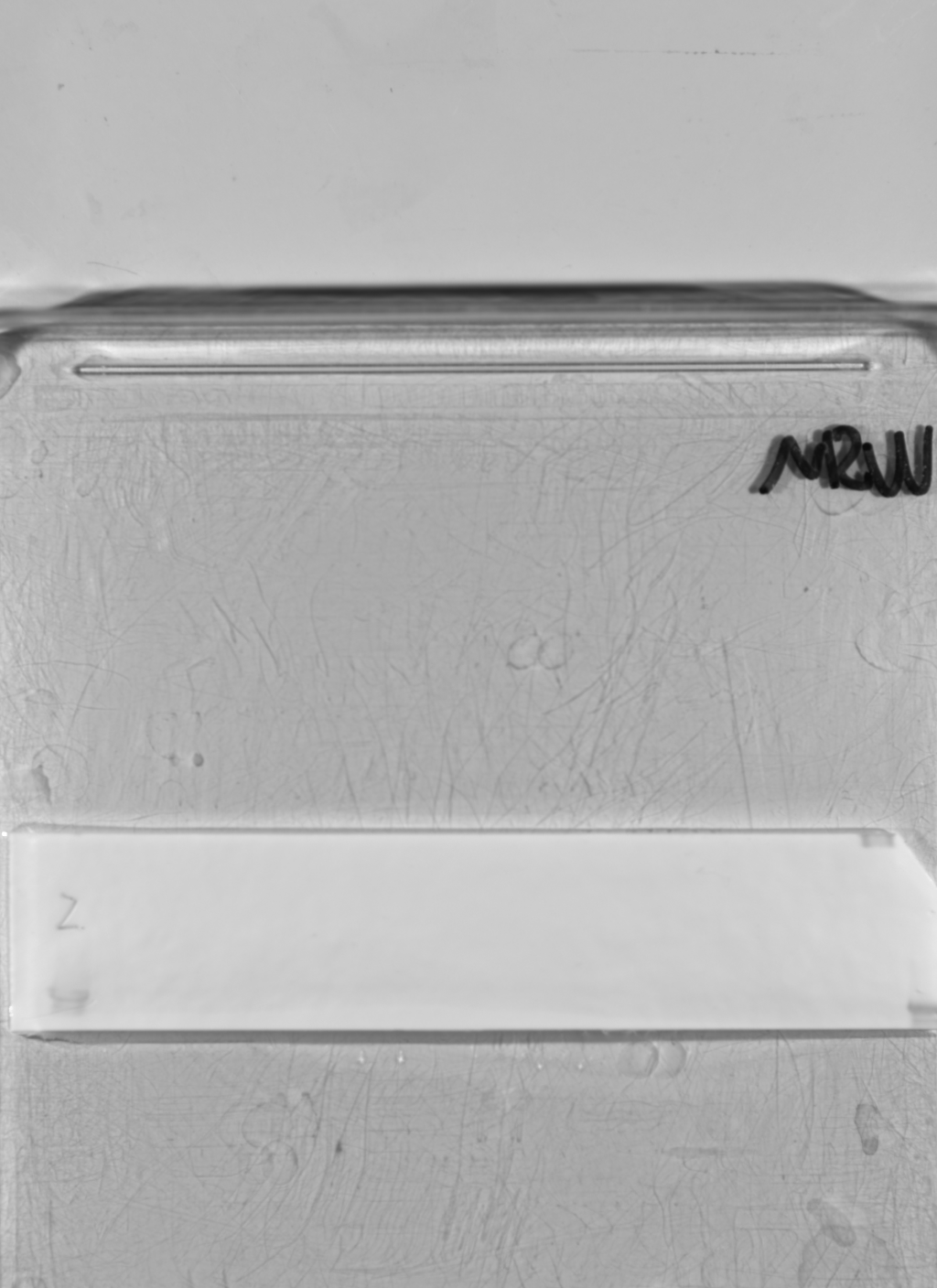

Supplement: Supplementary file 9 — Source data Fig. 4 [file 44321_2024_60_MOESM9_ESM.zip › Figure 4/4B/YAPC/Western phoPRKDC/2 1st phoPRK 3.6 _Ch-Marker.tif]

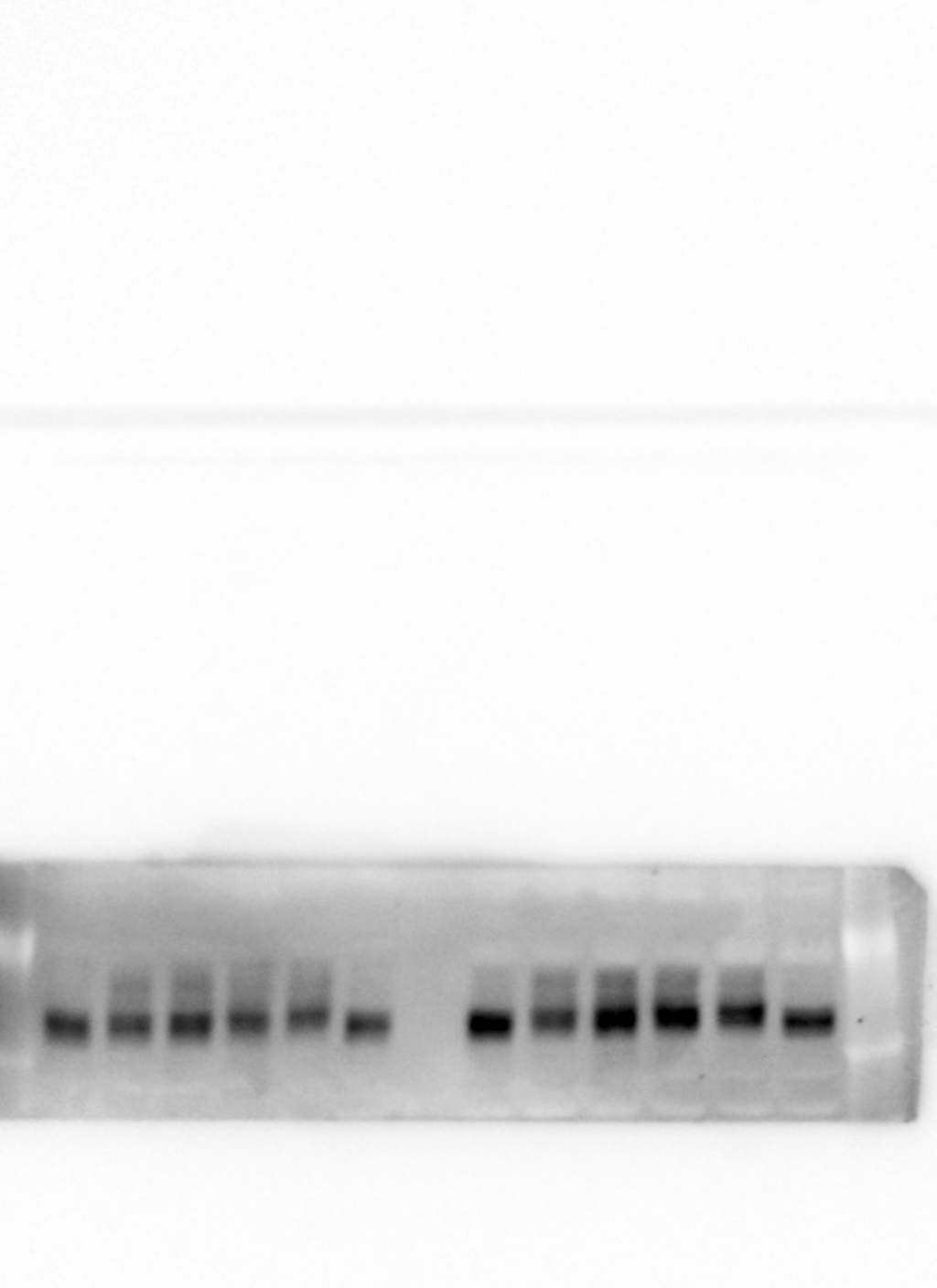

Supplement: Supplementary file 9 — Source data Fig. 4 [file 44321_2024_60_MOESM9_ESM.zip › Figure 4/4B/YAPC/Western PKMYT1/2 1st PK 8.2 _Ch.tif]

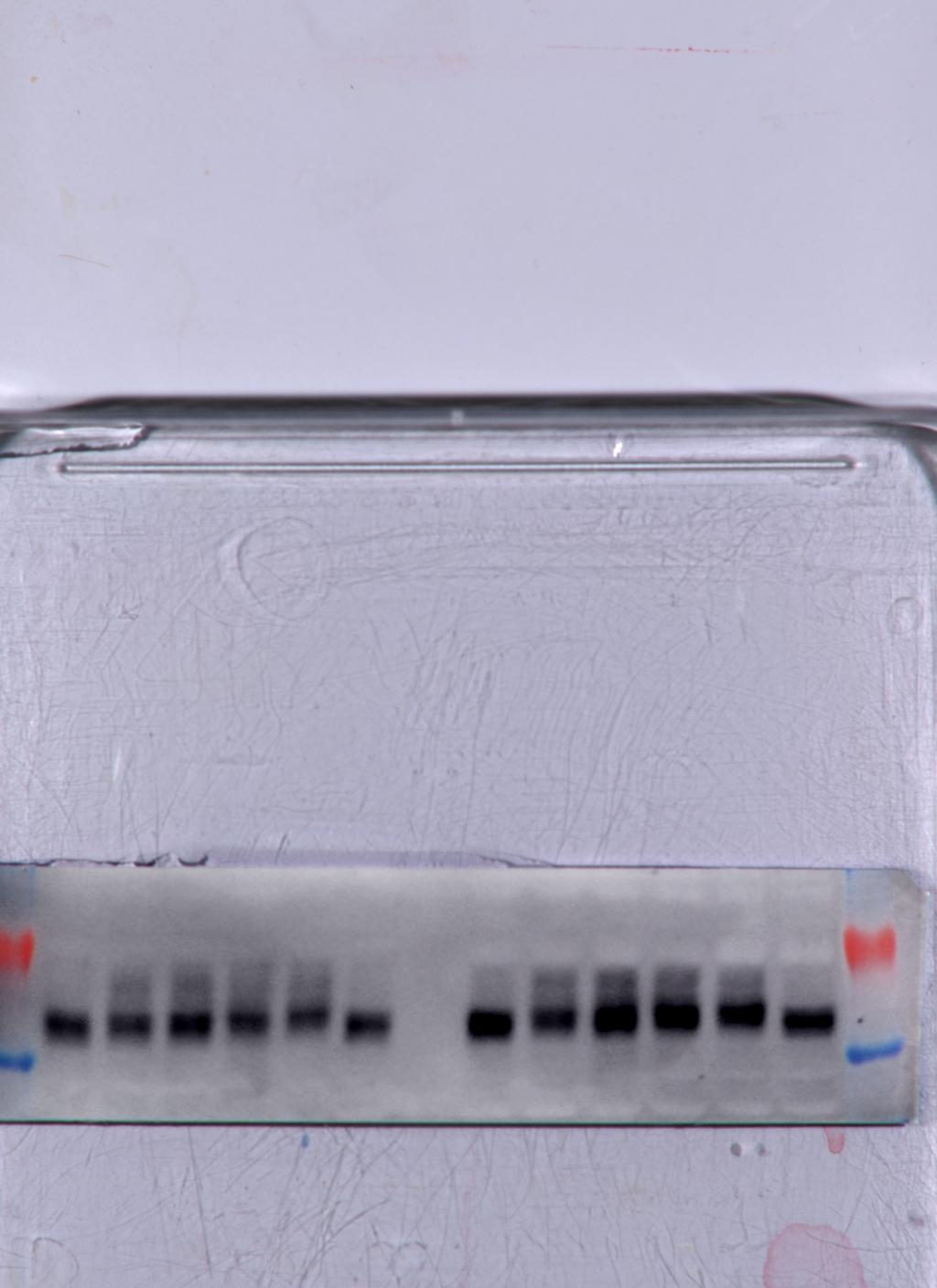

Supplement: Supplementary file 9 — Source data Fig. 4 [file 44321_2024_60_MOESM9_ESM.zip › Figure 4/4B/YAPC/Western PKMYT1/2 1st PK 8.2 _Ch+Marker.jpg]

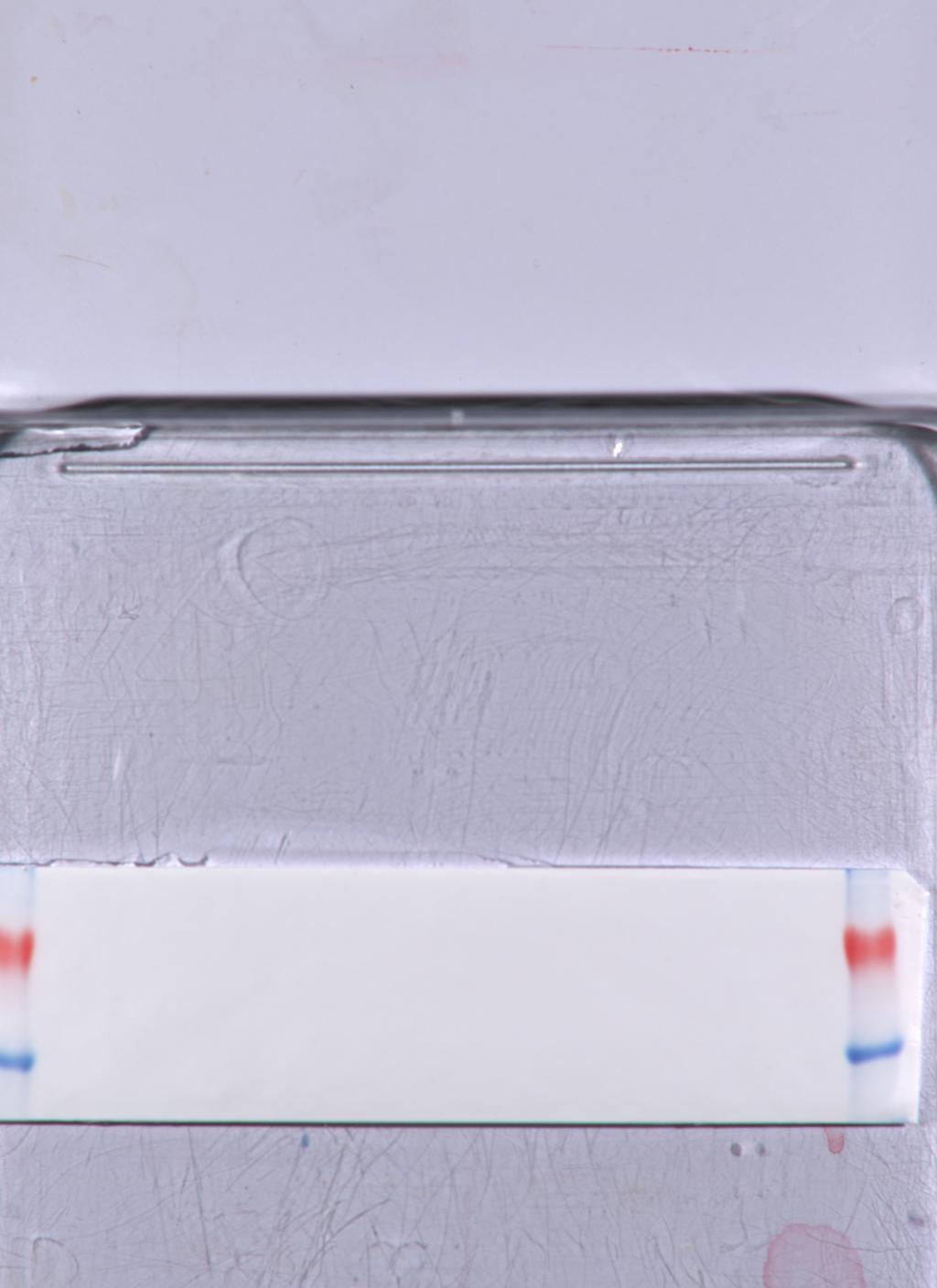

Supplement: Supplementary file 9 — Source data Fig. 4 [file 44321_2024_60_MOESM9_ESM.zip › Figure 4/4B/YAPC/Western PKMYT1/2 1st PK 8.2 _Ch-Marker.jpg]

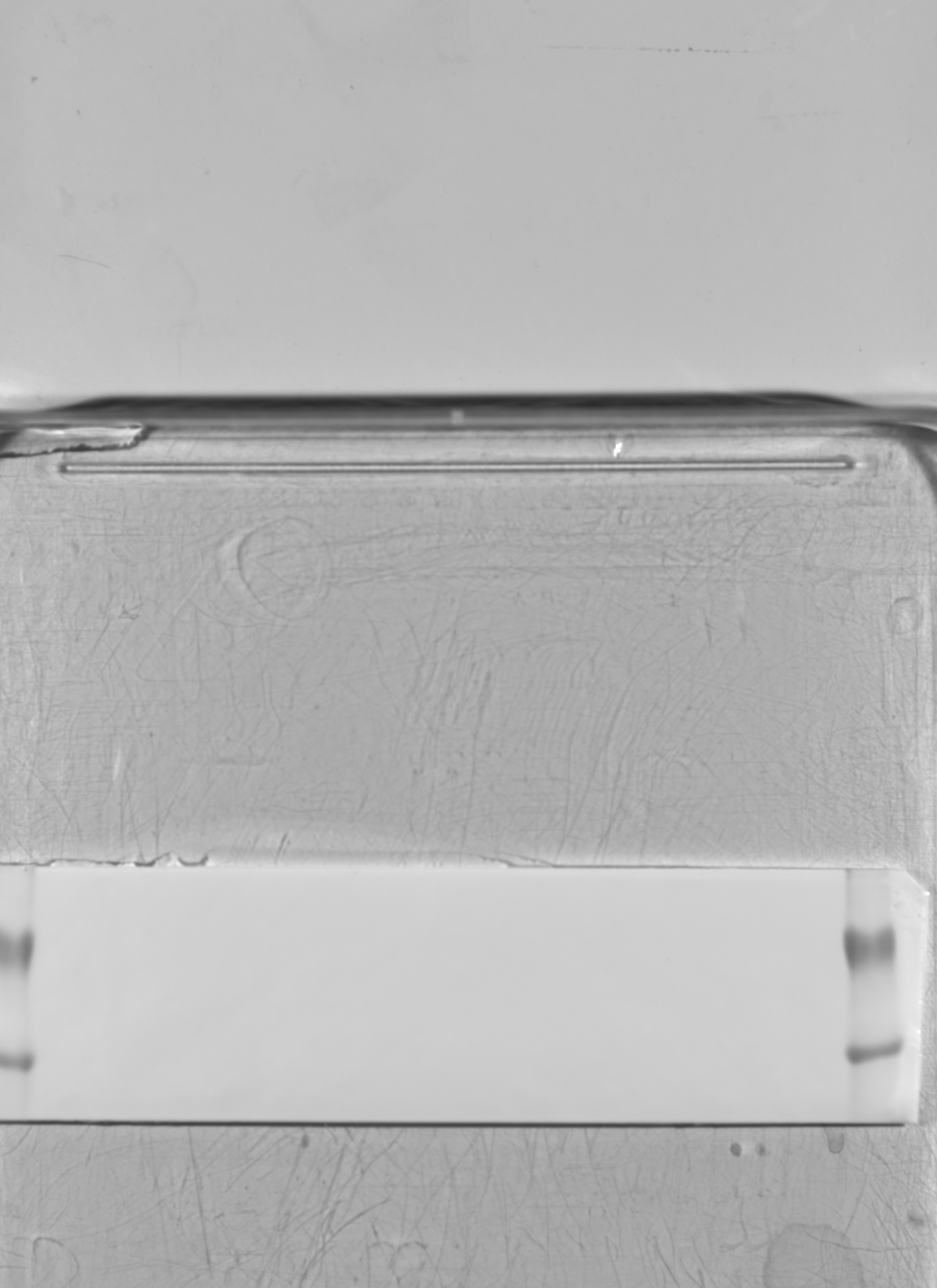

Supplement: Supplementary file 9 — Source data Fig. 4 [file 44321_2024_60_MOESM9_ESM.zip › Figure 4/4B/YAPC/Western PKMYT1/2 1st PK 8.2 _Ch-Marker.tif]

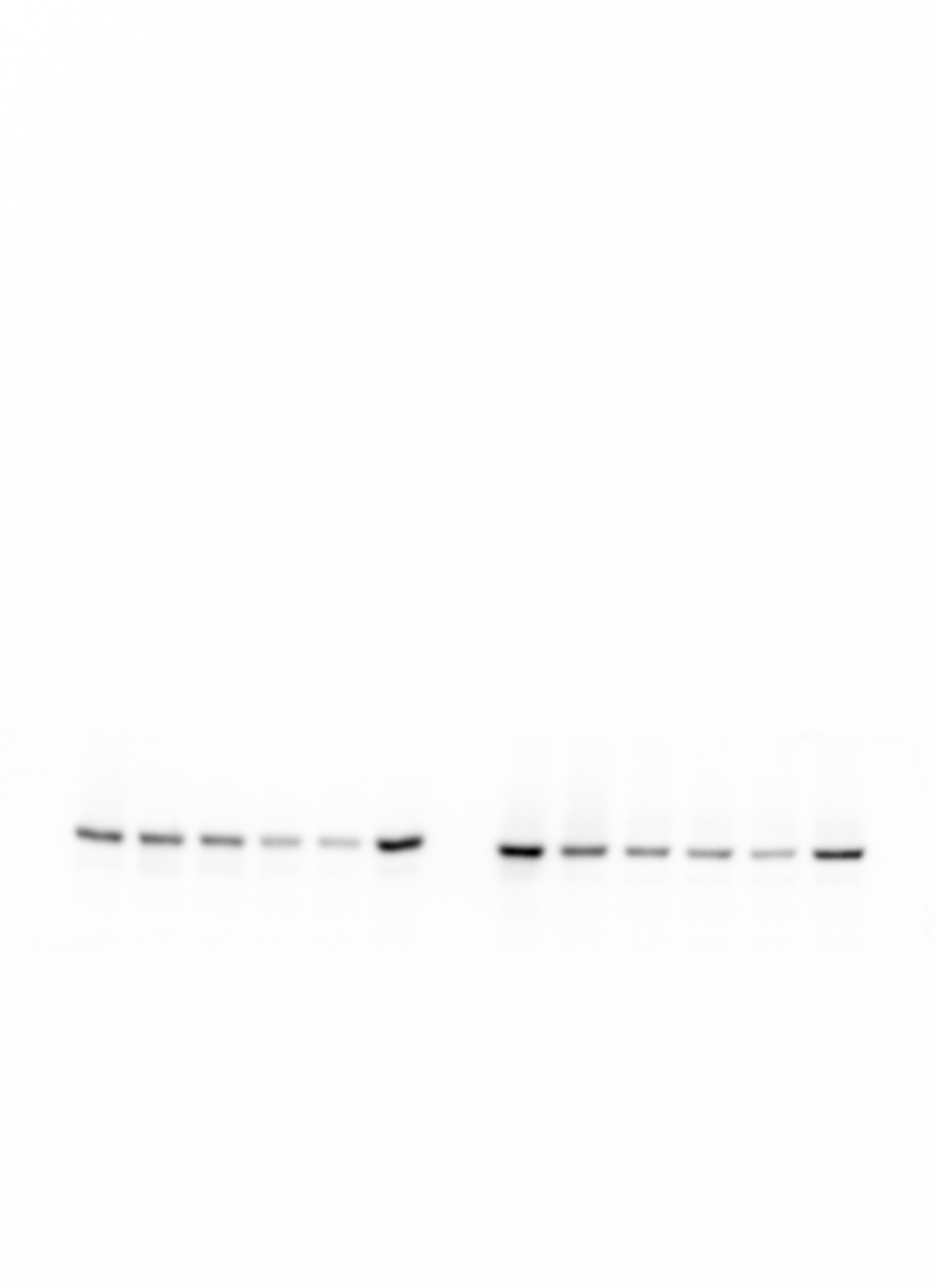

Supplement: Supplementary file 9 — Source data Fig. 4 [file 44321_2024_60_MOESM9_ESM.zip › Figure 4/4B/YAPC/Western PLK1/1 1st PLK 0.2 _Ch.tif]

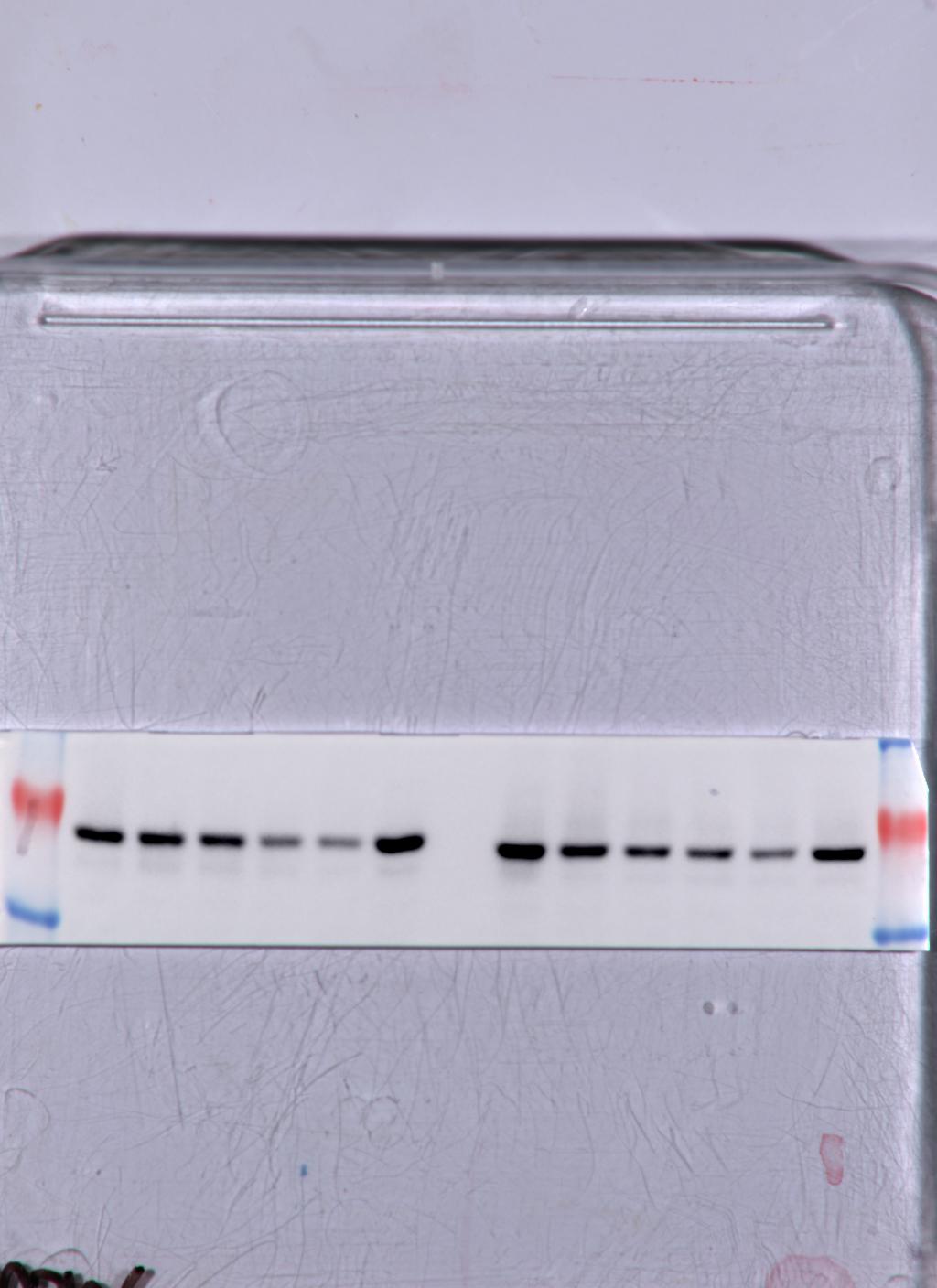

Supplement: Supplementary file 9 — Source data Fig. 4 [file 44321_2024_60_MOESM9_ESM.zip › Figure 4/4B/YAPC/Western PLK1/1 1st PLK 0.2 _Ch+Marker.jpg]

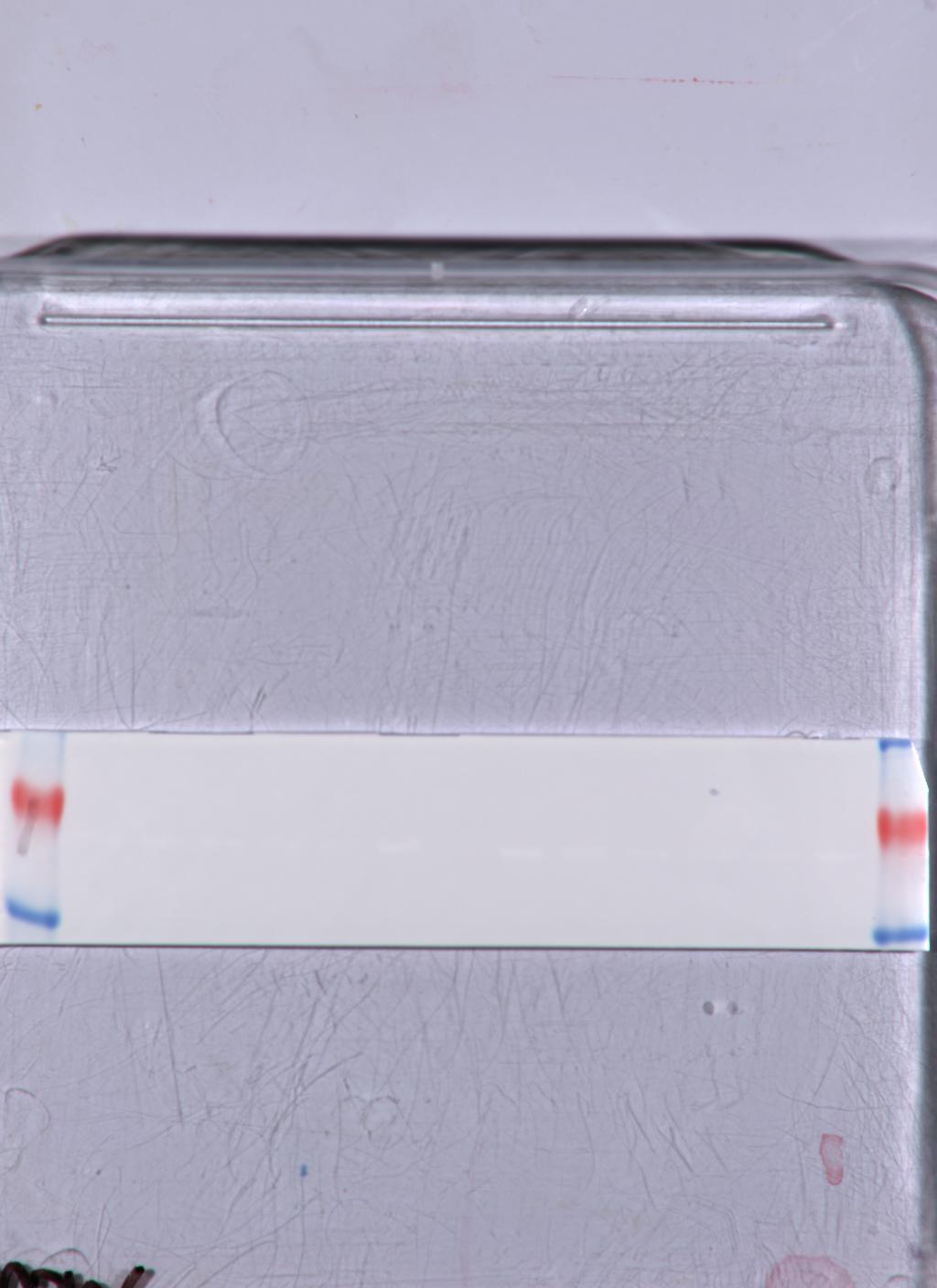

Supplement: Supplementary file 9 — Source data Fig. 4 [file 44321_2024_60_MOESM9_ESM.zip › Figure 4/4B/YAPC/Western PLK1/1 1st PLK 0.2 _Ch-Marker.jpg]

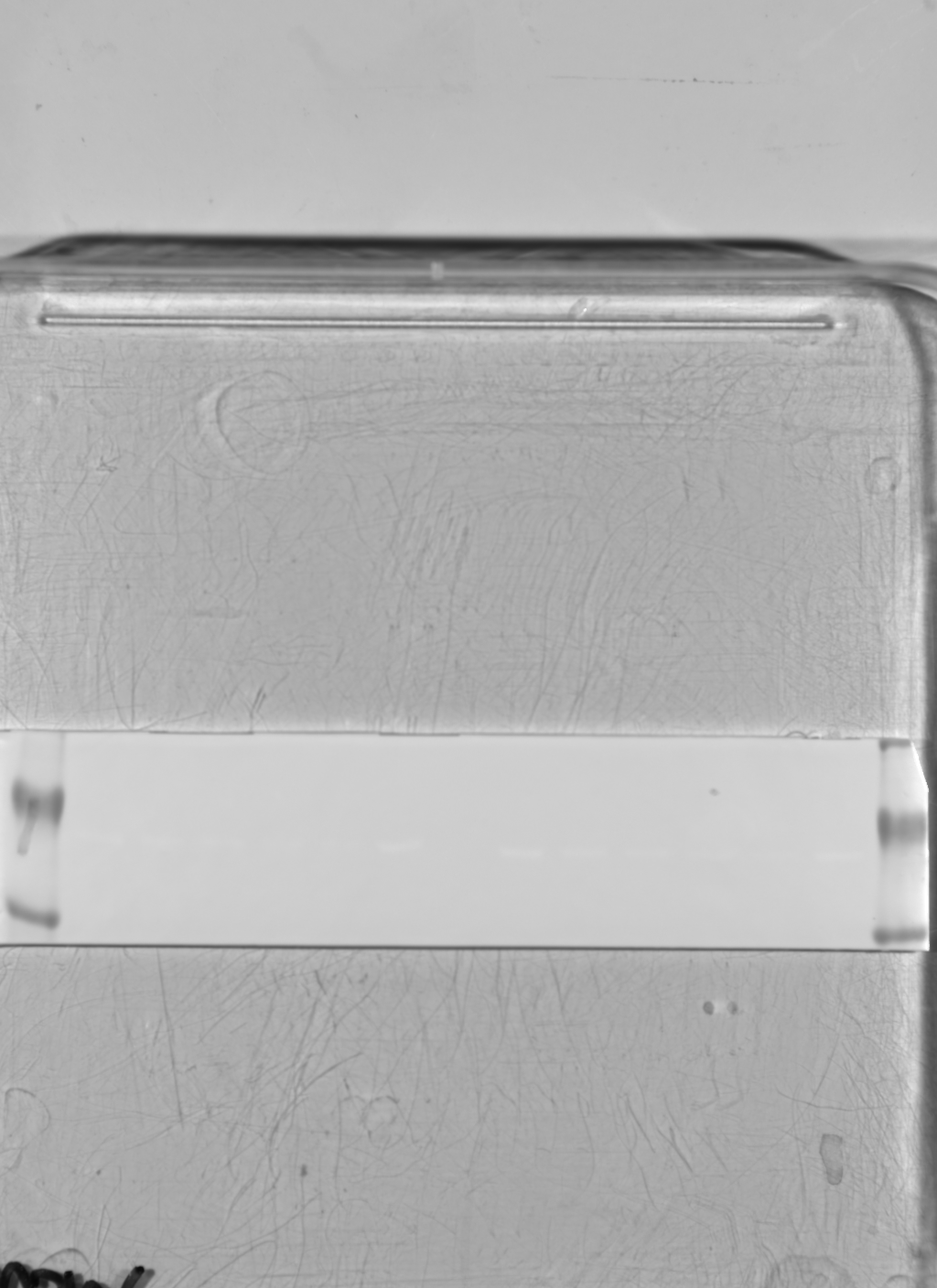

Supplement: Supplementary file 9 — Source data Fig. 4 [file 44321_2024_60_MOESM9_ESM.zip › Figure 4/4B/YAPC/Western PLK1/1 1st PLK 0.2 _Ch-Marker.tif]

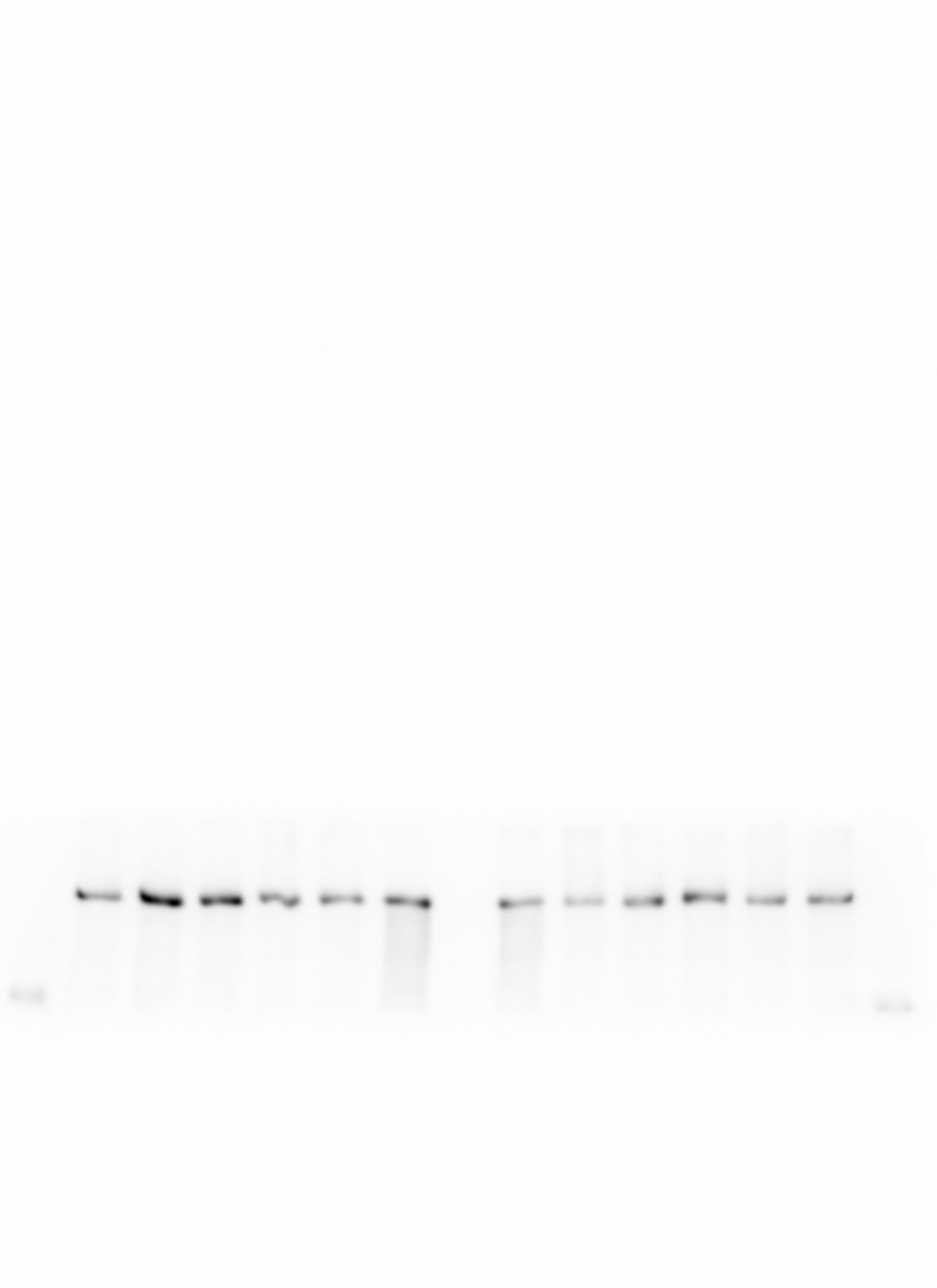

Supplement: Supplementary file 9 — Source data Fig. 4 [file 44321_2024_60_MOESM9_ESM.zip › Figure 4/4B/YAPC/Western PRKDC/2 1st PRK 0.2 _Ch.tif]

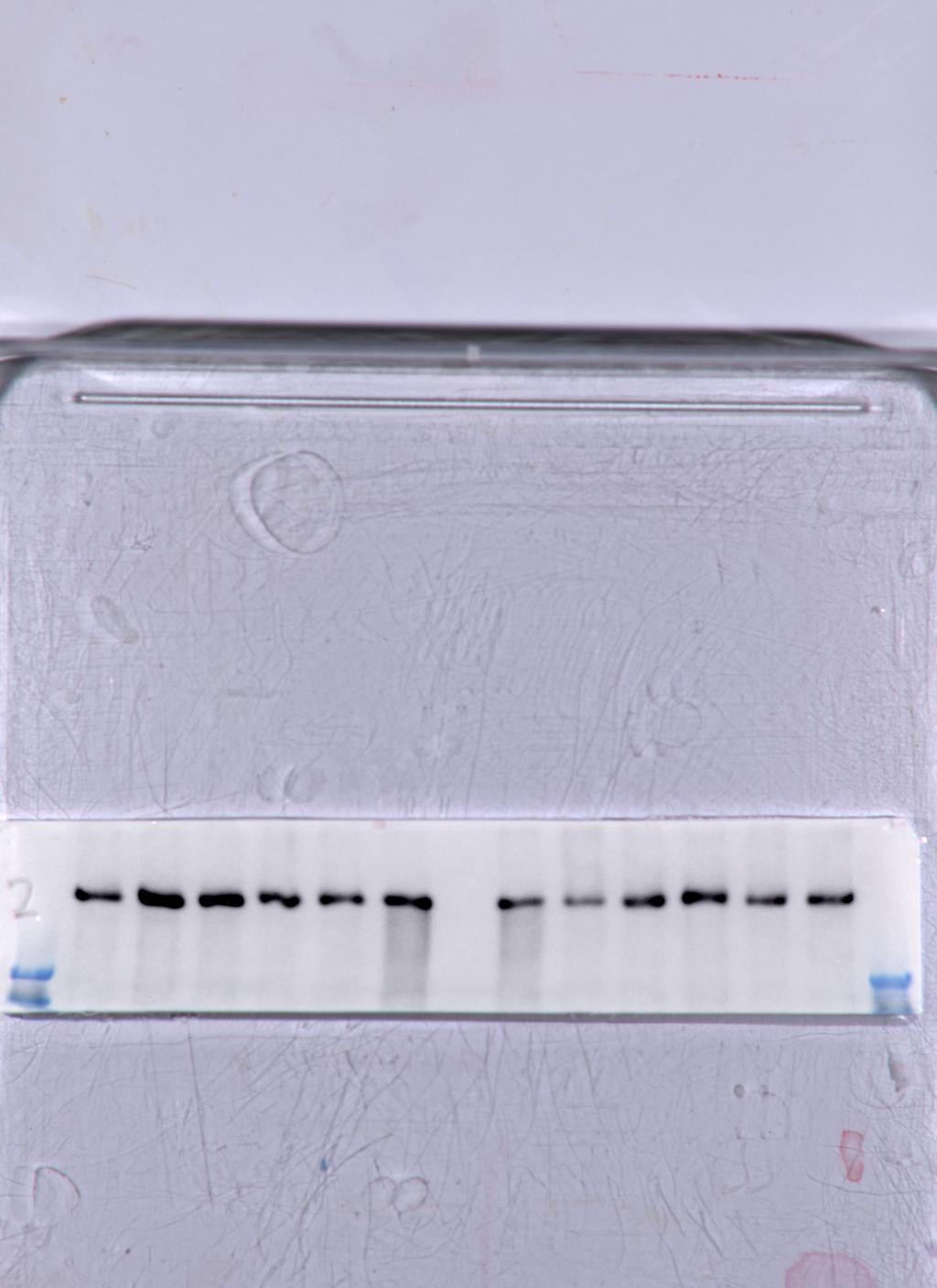

Supplement: Supplementary file 9 — Source data Fig. 4 [file 44321_2024_60_MOESM9_ESM.zip › Figure 4/4B/YAPC/Western PRKDC/2 1st PRK 0.2 _Ch+Marker.jpg]

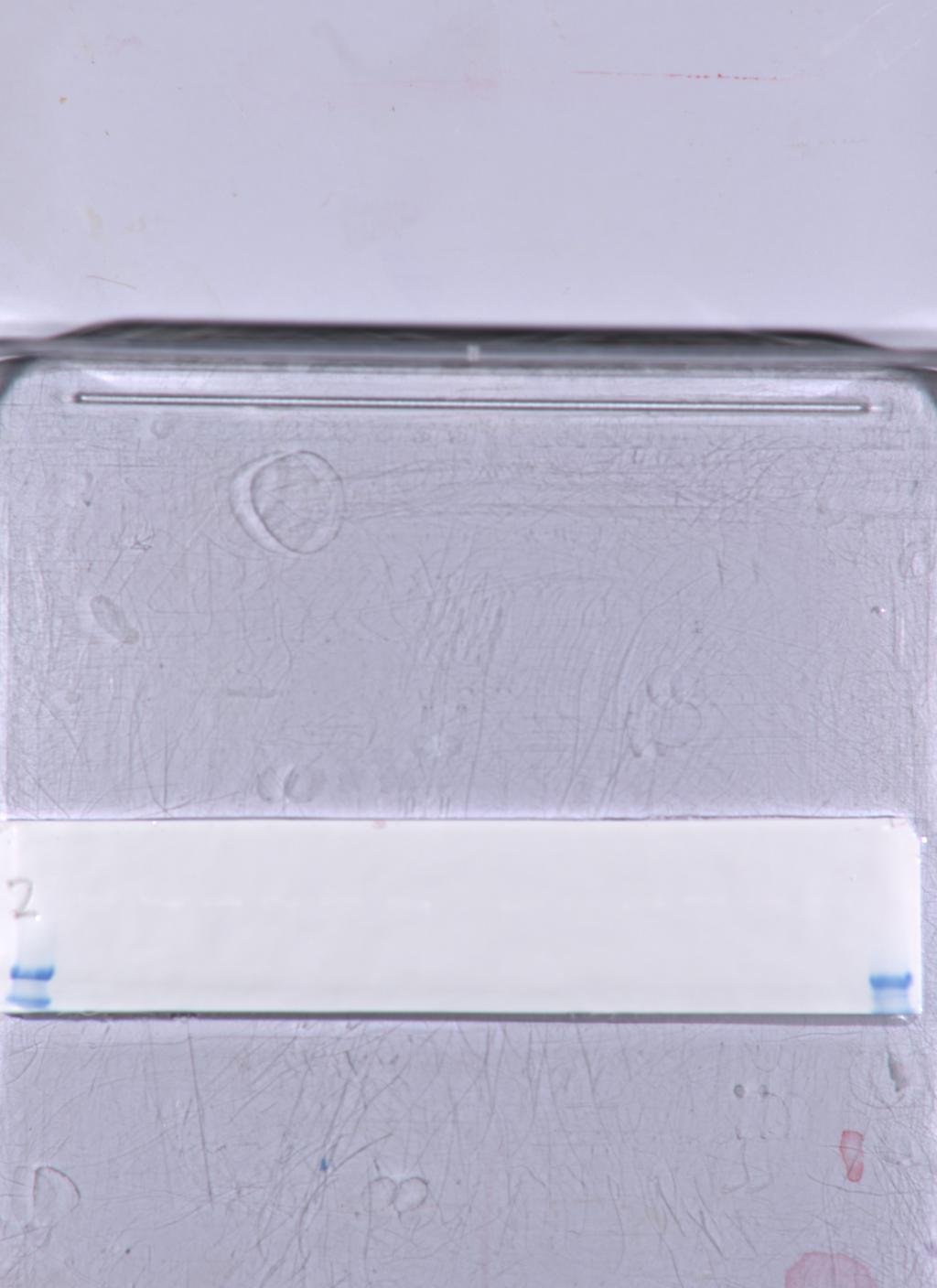

Supplement: Supplementary file 9 — Source data Fig. 4 [file 44321_2024_60_MOESM9_ESM.zip › Figure 4/4B/YAPC/Western PRKDC/2 1st PRK 0.2 _Ch-Marker.jpg]

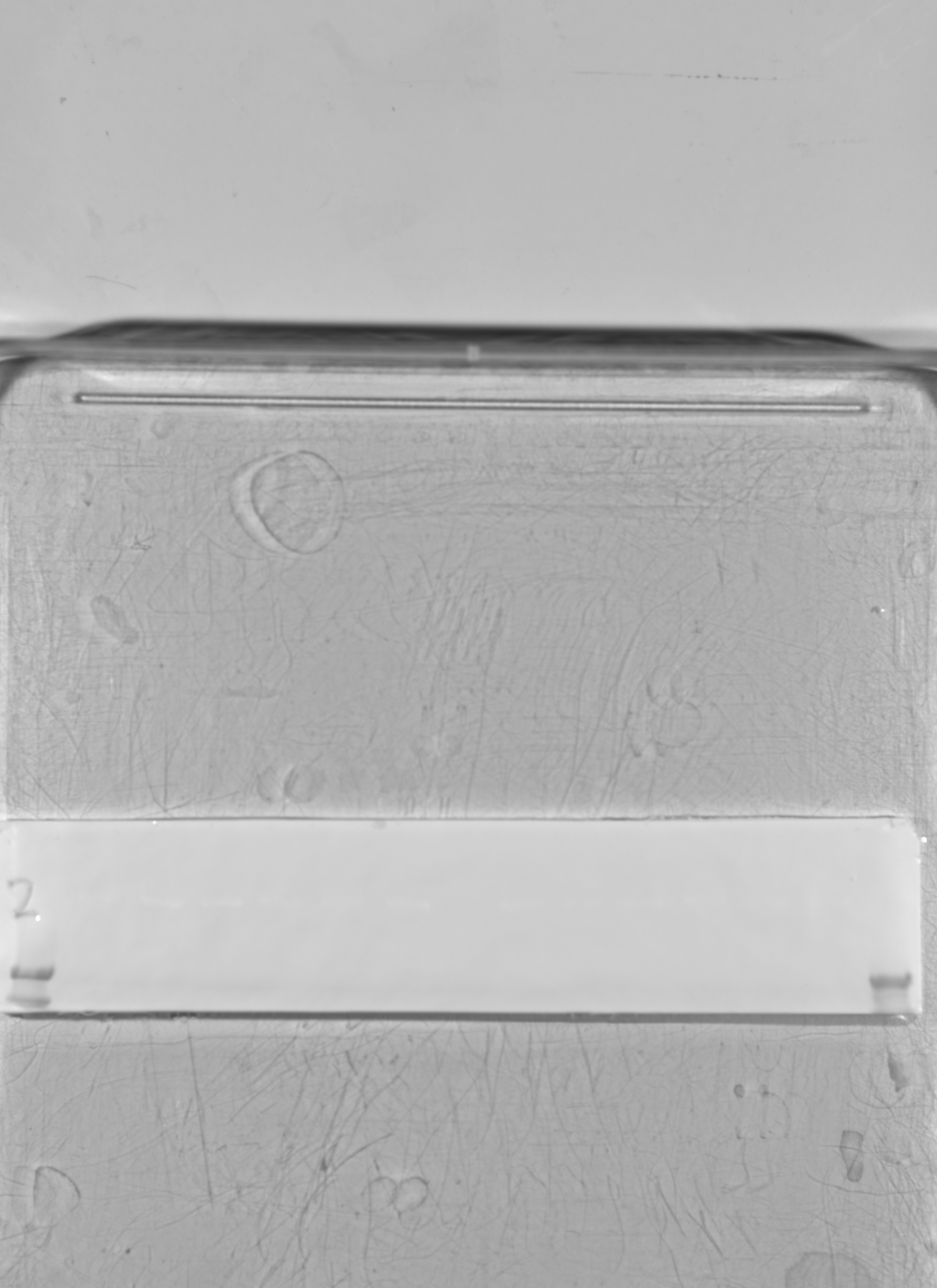

Supplement: Supplementary file 9 — Source data Fig. 4 [file 44321_2024_60_MOESM9_ESM.zip › Figure 4/4B/YAPC/Western PRKDC/2 1st PRK 0.2 _Ch-Marker.tif]

## Slide 1
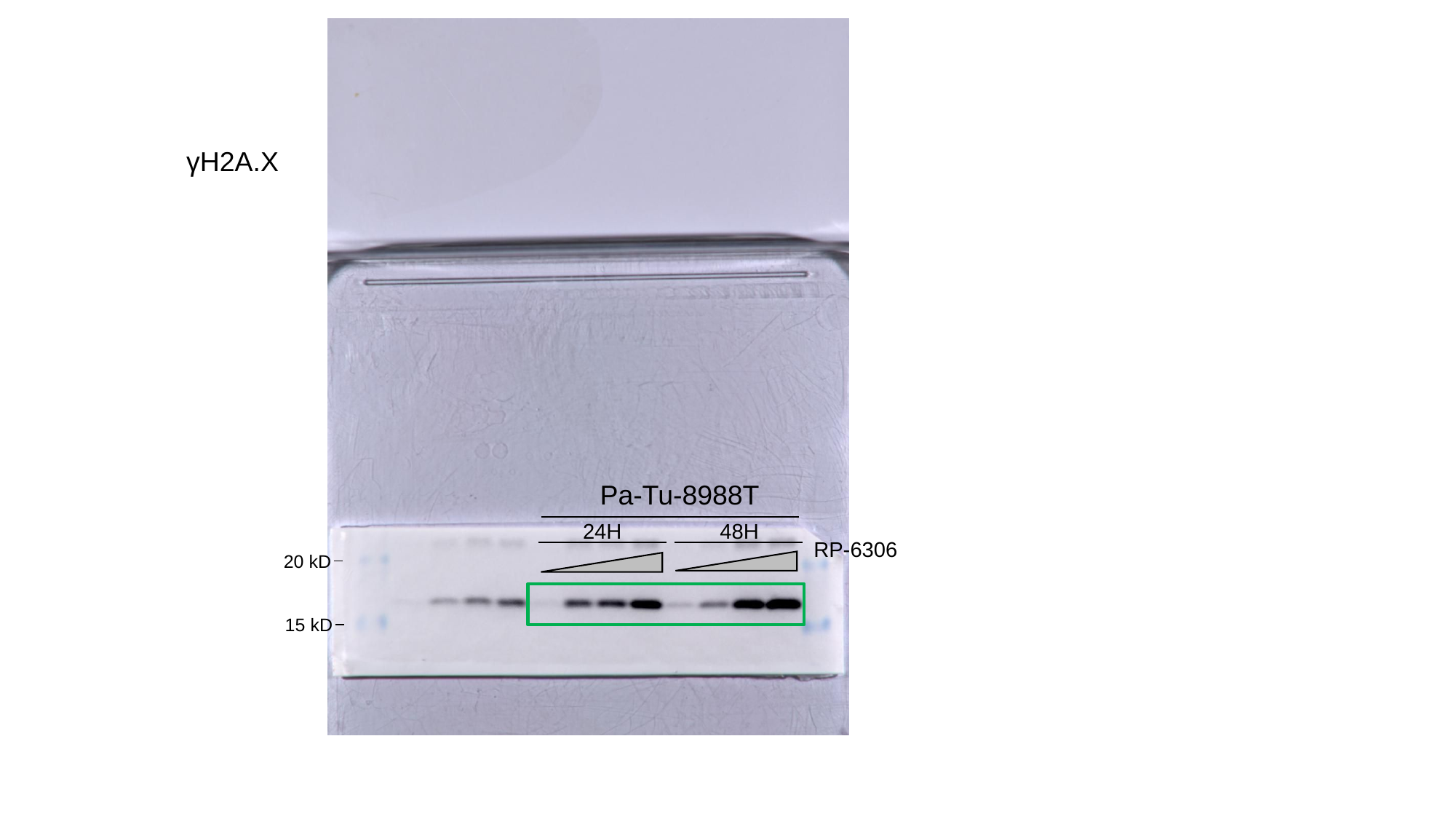

γH2A.X
Pa-Tu-8988T
24H
48H
RP-6306
20 kD
15 kD

## Slide 2
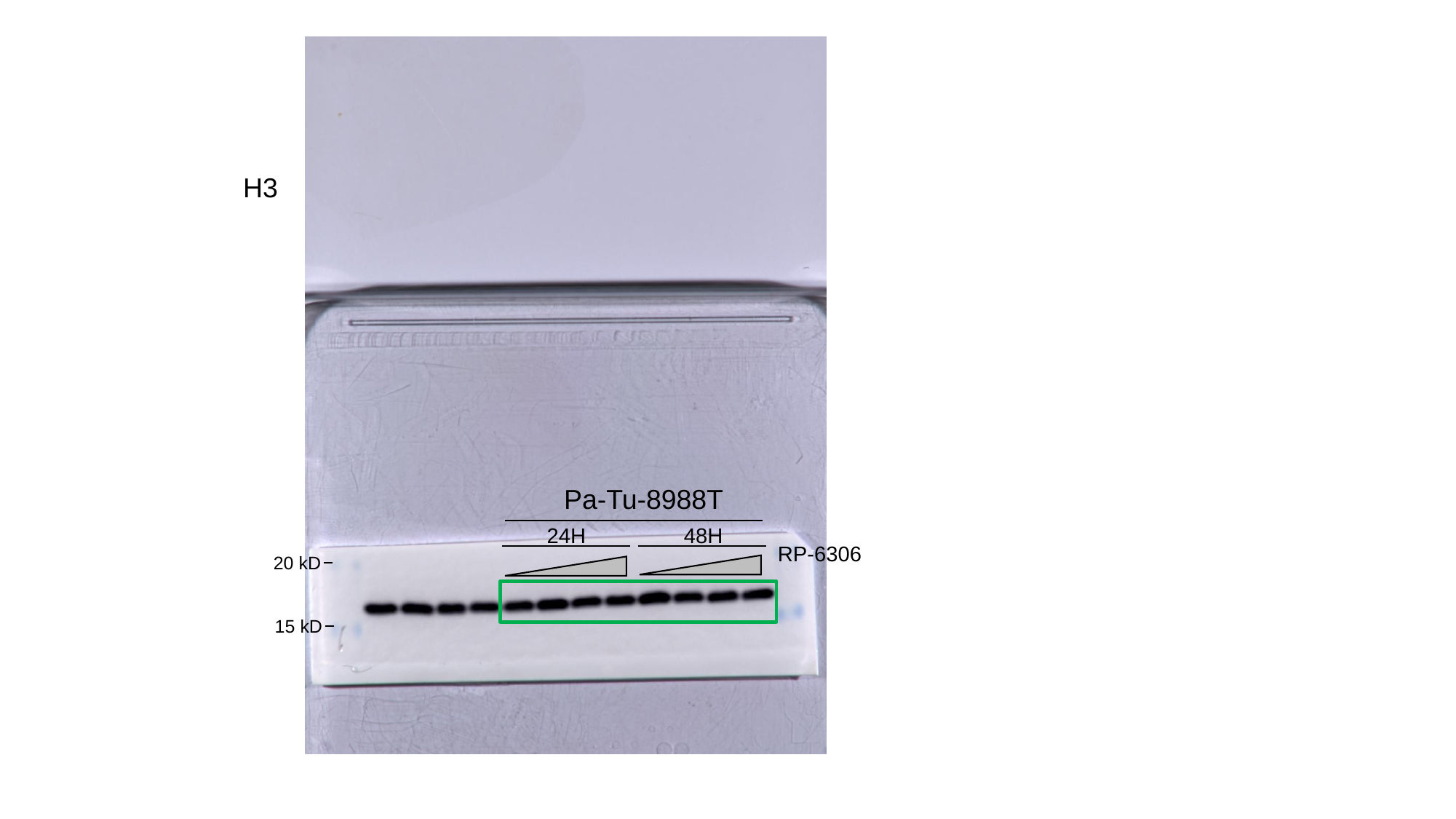

H3
Pa-Tu-8988T
24H
48H
RP-6306
20 kD
15 kD

## Slide 3
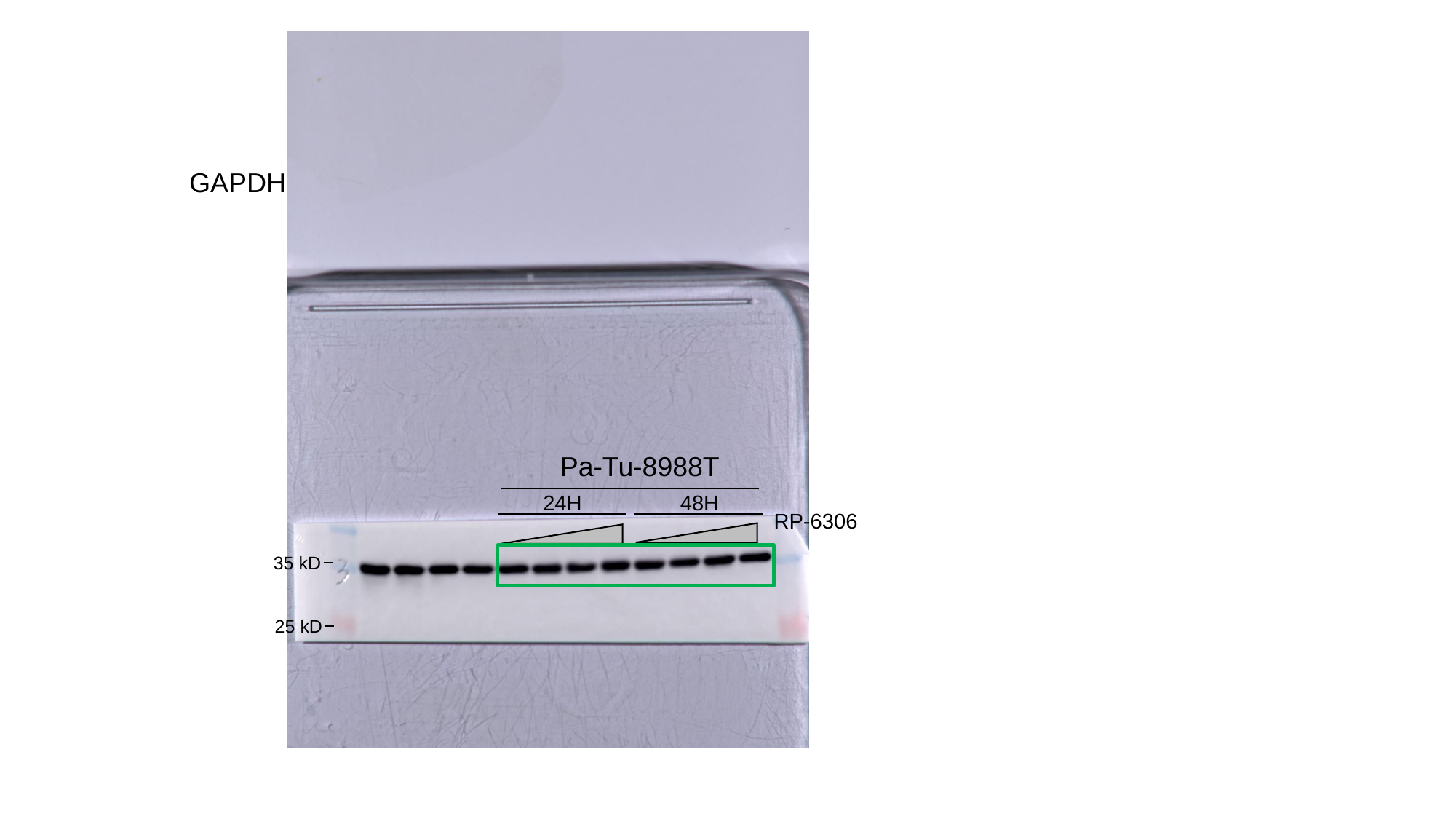

GAPDH
Pa-Tu-8988T
24H
48H
RP-6306
35 kD
25 kD

Supplement: Supplementary file 9 — Source data Fig. 4 [file 44321_2024_60_MOESM9_ESM.zip › Figure 4/4C/88T/4C 88T.pptx]

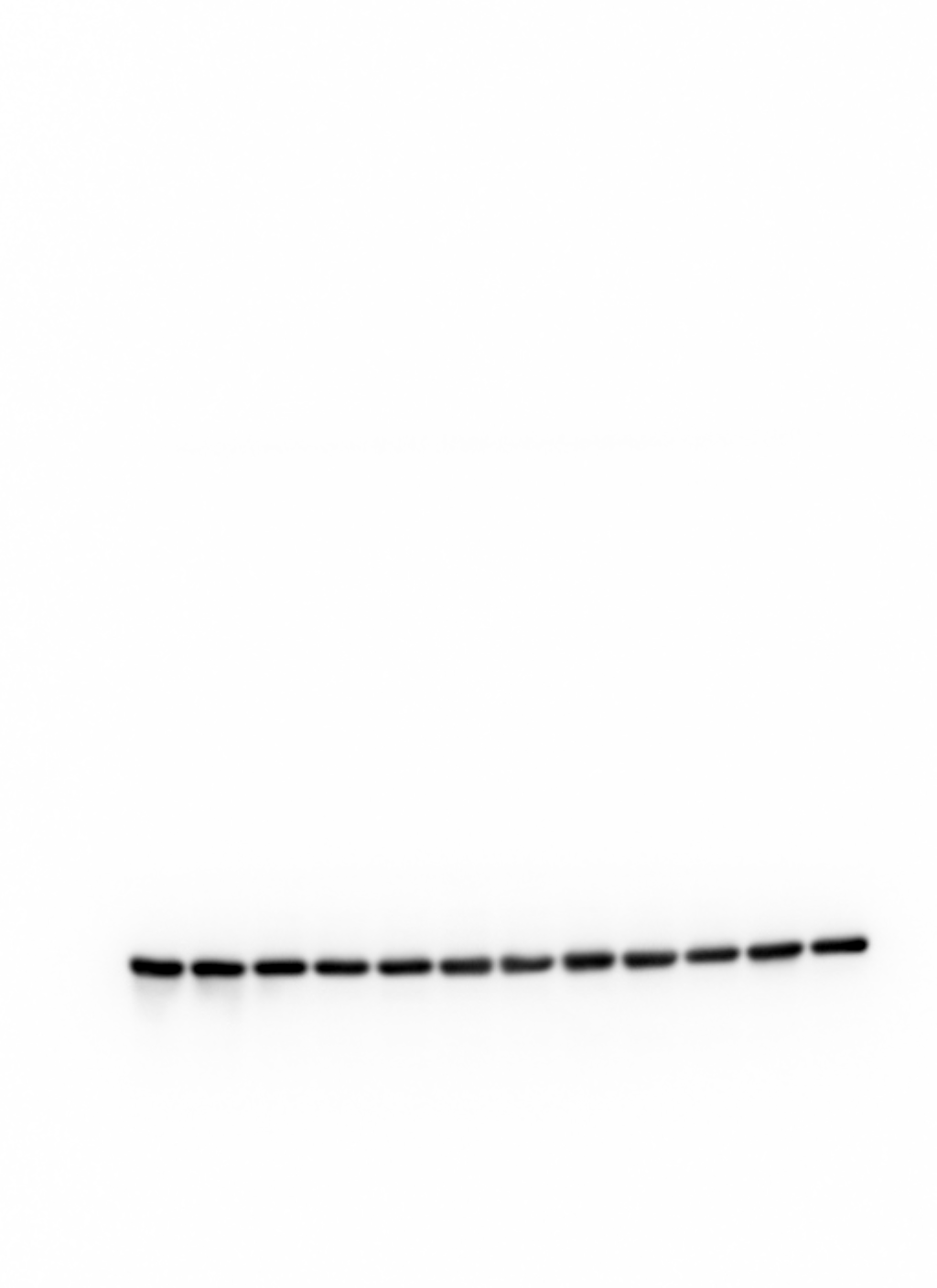

Supplement: Supplementary file 9 — Source data Fig. 4 [file 44321_2024_60_MOESM9_ESM.zip › Figure 4/4C/88T/Western GAPDH 0.1/3 GAP 0.1 _Ch.tif]

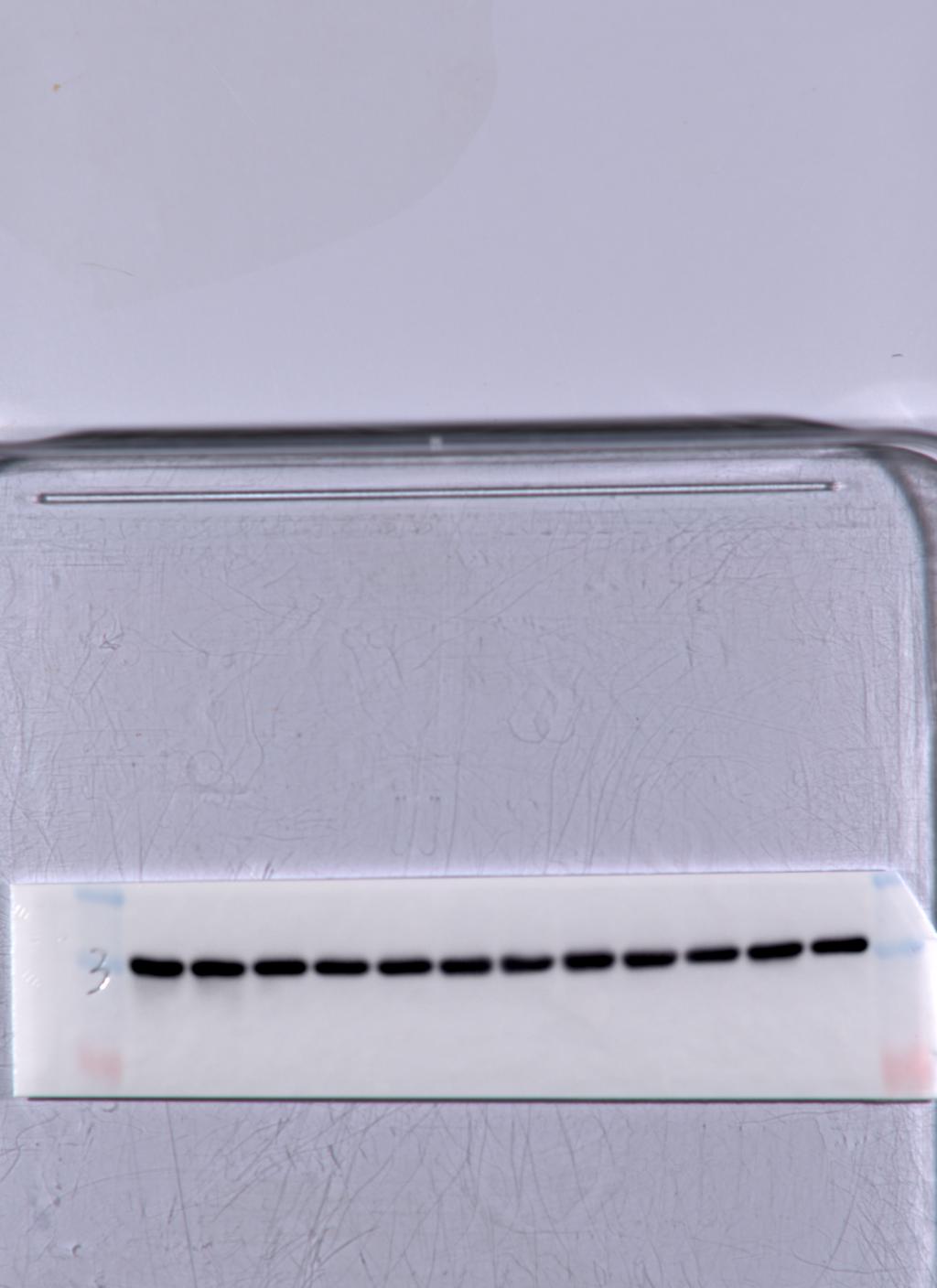

Supplement: Supplementary file 9 — Source data Fig. 4 [file 44321_2024_60_MOESM9_ESM.zip › Figure 4/4C/88T/Western GAPDH 0.1/3 GAP 0.1 _Ch+Marker.jpg]

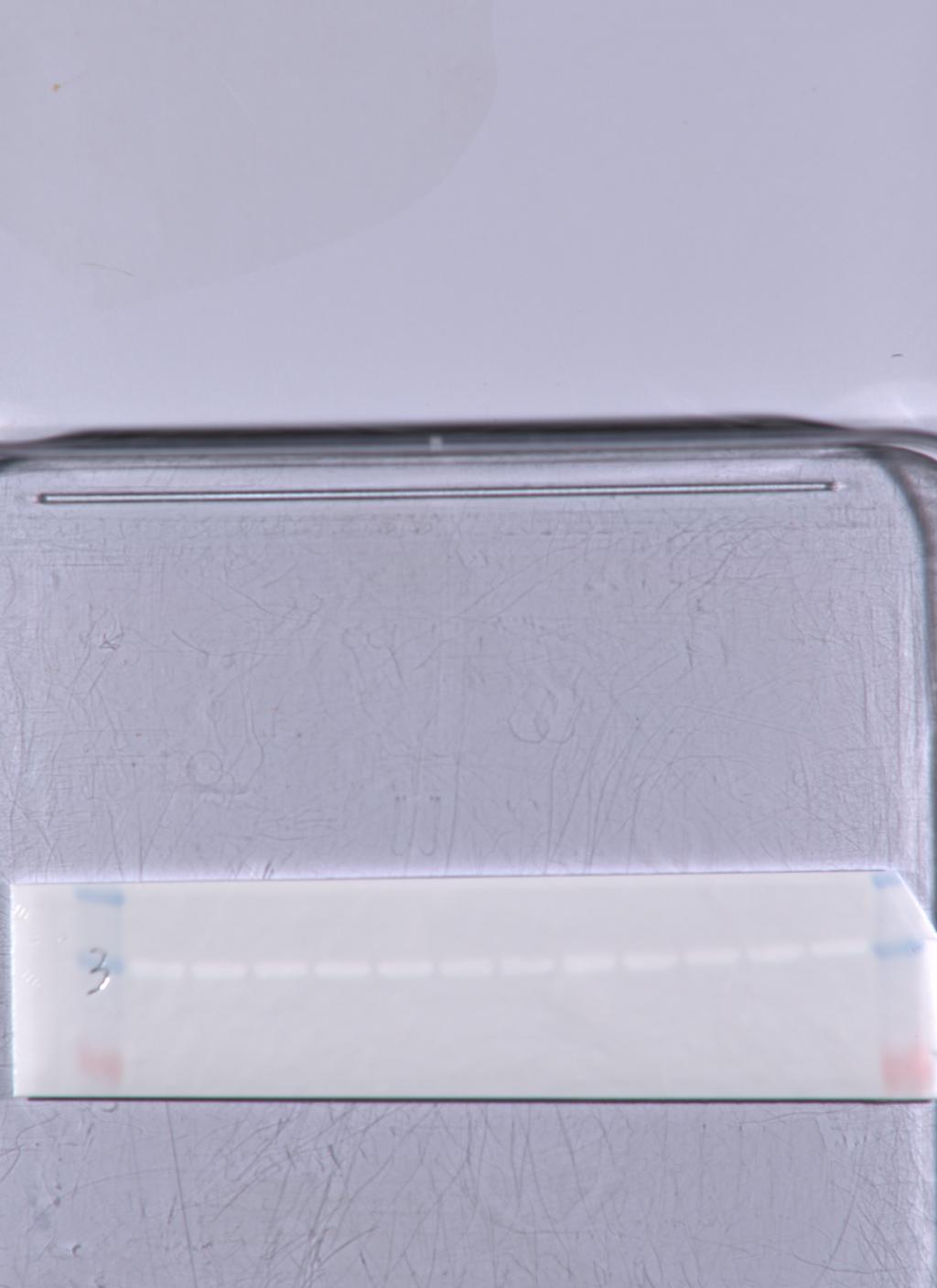

Supplement: Supplementary file 9 — Source data Fig. 4 [file 44321_2024_60_MOESM9_ESM.zip › Figure 4/4C/88T/Western GAPDH 0.1/3 GAP 0.1 _Ch-Marker.jpg]

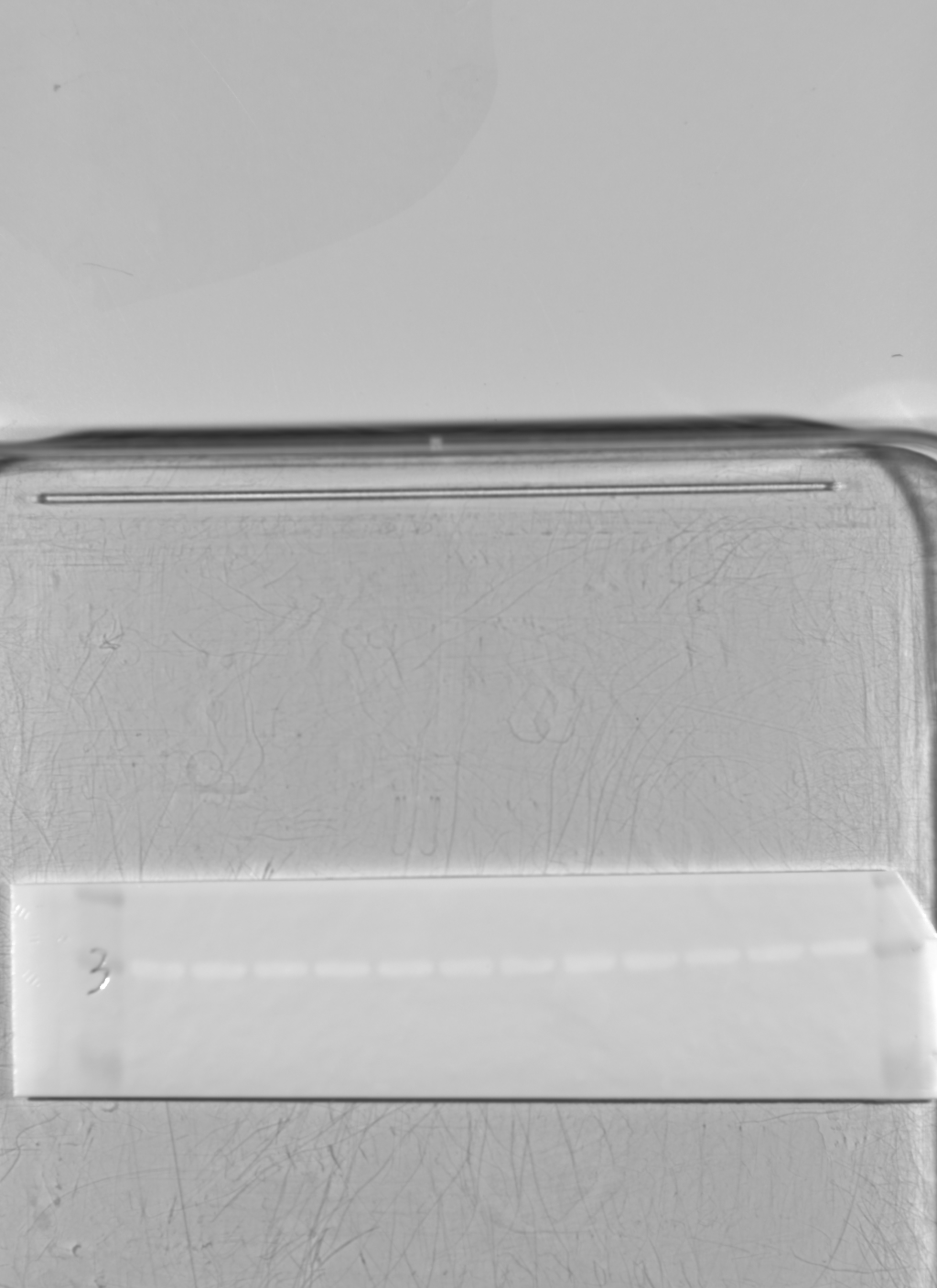

Supplement: Supplementary file 9 — Source data Fig. 4 [file 44321_2024_60_MOESM9_ESM.zip › Figure 4/4C/88T/Western GAPDH 0.1/3 GAP 0.1 _Ch-Marker.tif]

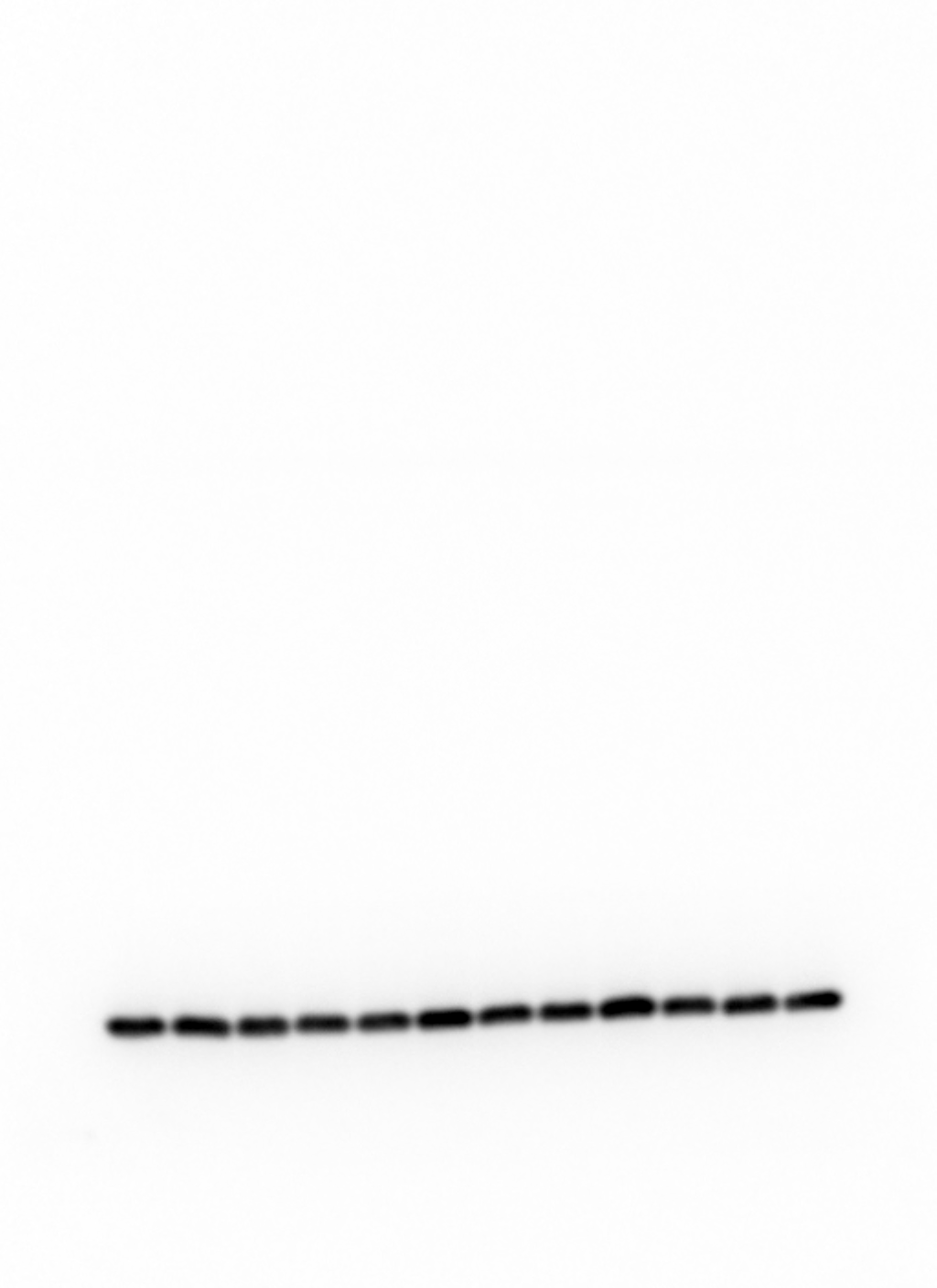

Supplement: Supplementary file 9 — Source data Fig. 4 [file 44321_2024_60_MOESM9_ESM.zip › Figure 4/4C/88T/Western H3 0.1/1 1st H3 0.1 _Ch.tif]

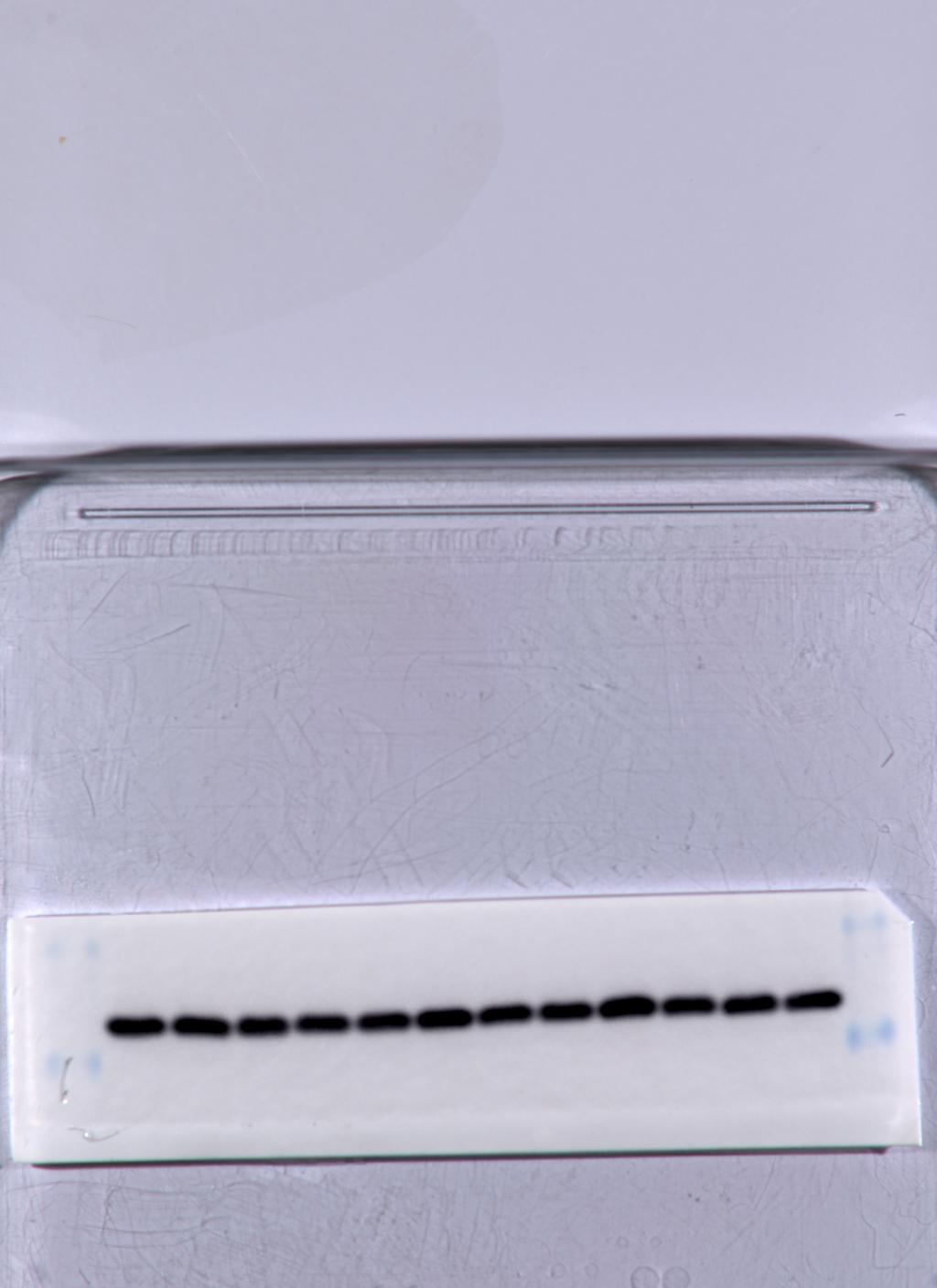

Supplement: Supplementary file 9 — Source data Fig. 4 [file 44321_2024_60_MOESM9_ESM.zip › Figure 4/4C/88T/Western H3 0.1/1 1st H3 0.1 _Ch+Marker.jpg]

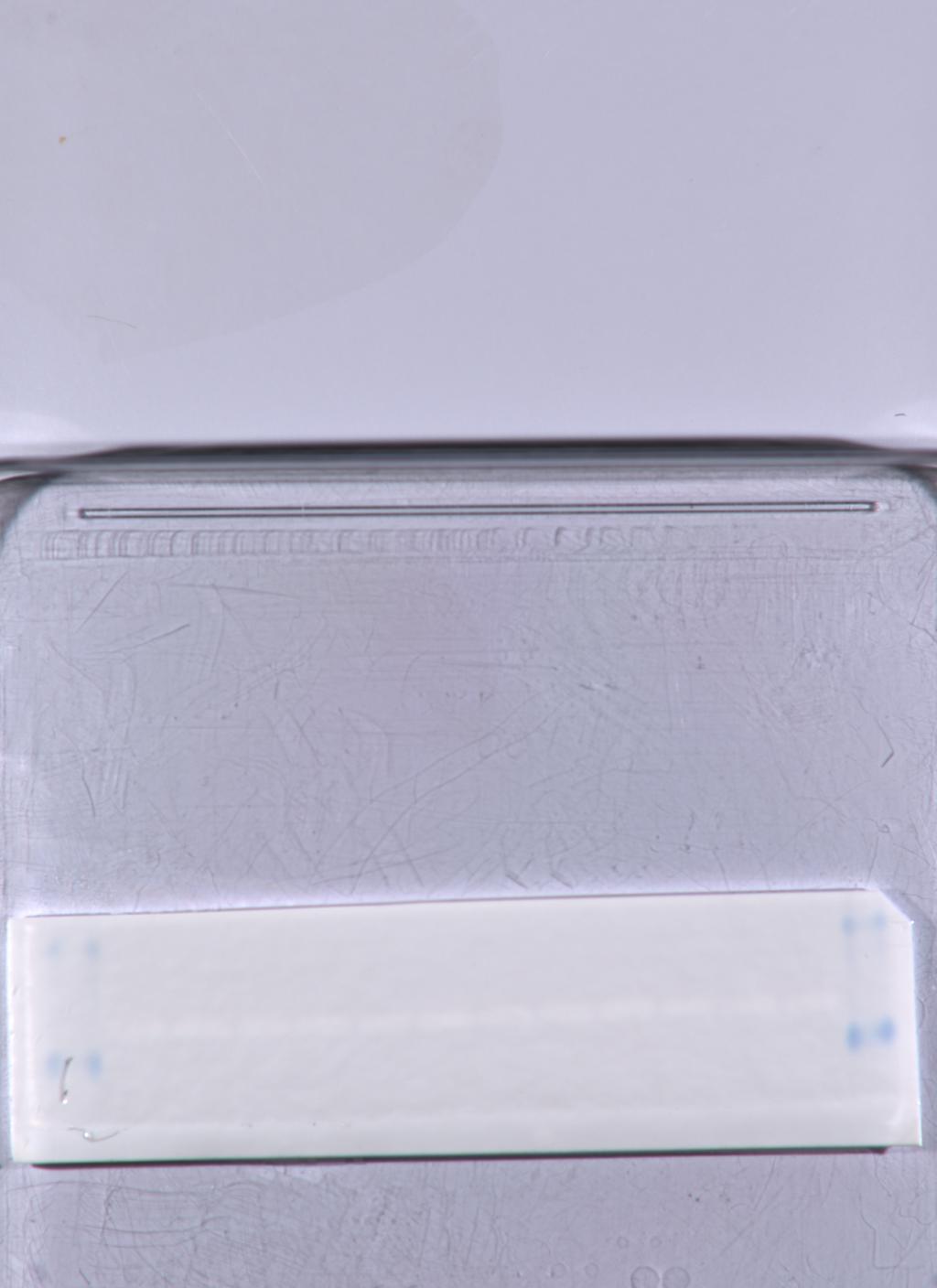

Supplement: Supplementary file 9 — Source data Fig. 4 [file 44321_2024_60_MOESM9_ESM.zip › Figure 4/4C/88T/Western H3 0.1/1 1st H3 0.1 _Ch-Marker.jpg]

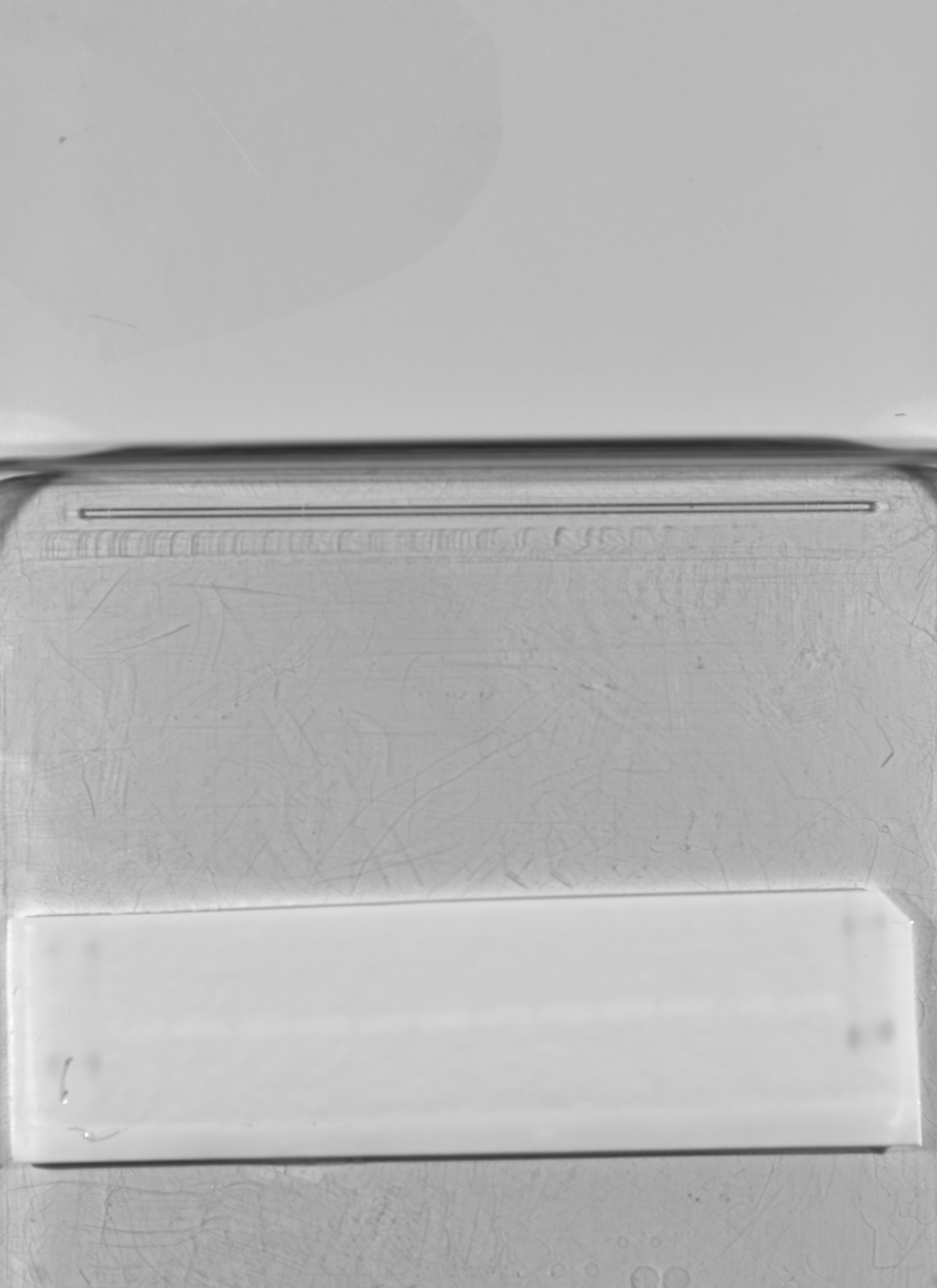

Supplement: Supplementary file 9 — Source data Fig. 4 [file 44321_2024_60_MOESM9_ESM.zip › Figure 4/4C/88T/Western H3 0.1/1 1st H3 0.1 _Ch-Marker.tif]

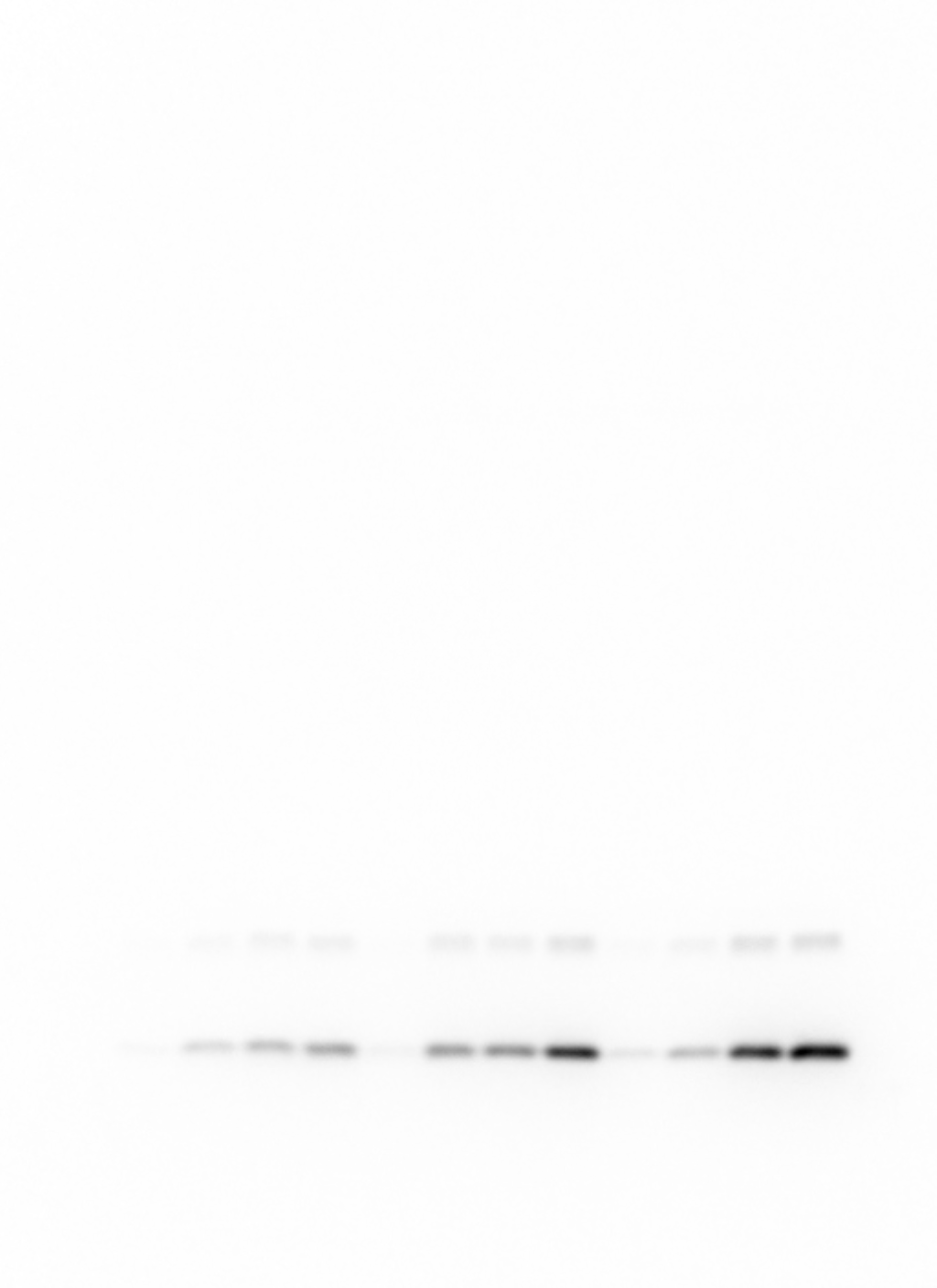

Supplement: Supplementary file 9 — Source data Fig. 4 [file 44321_2024_60_MOESM9_ESM.zip › Figure 4/4C/88T/Western rH2A 0.2/2 1st rH2A 0.2 _Ch.tif]

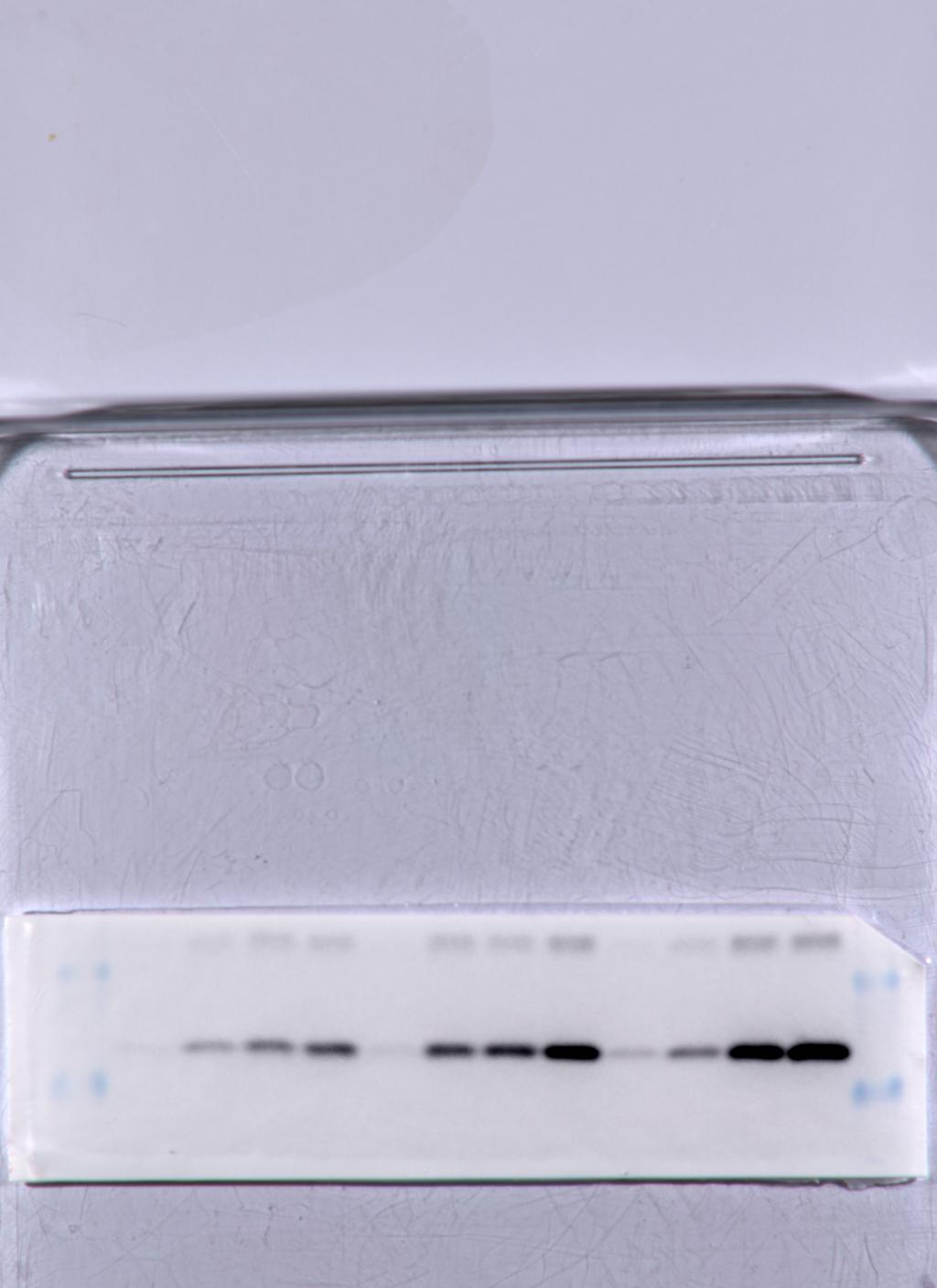

Supplement: Supplementary file 9 — Source data Fig. 4 [file 44321_2024_60_MOESM9_ESM.zip › Figure 4/4C/88T/Western rH2A 0.2/2 1st rH2A 0.2 _Ch+Marker.jpg]

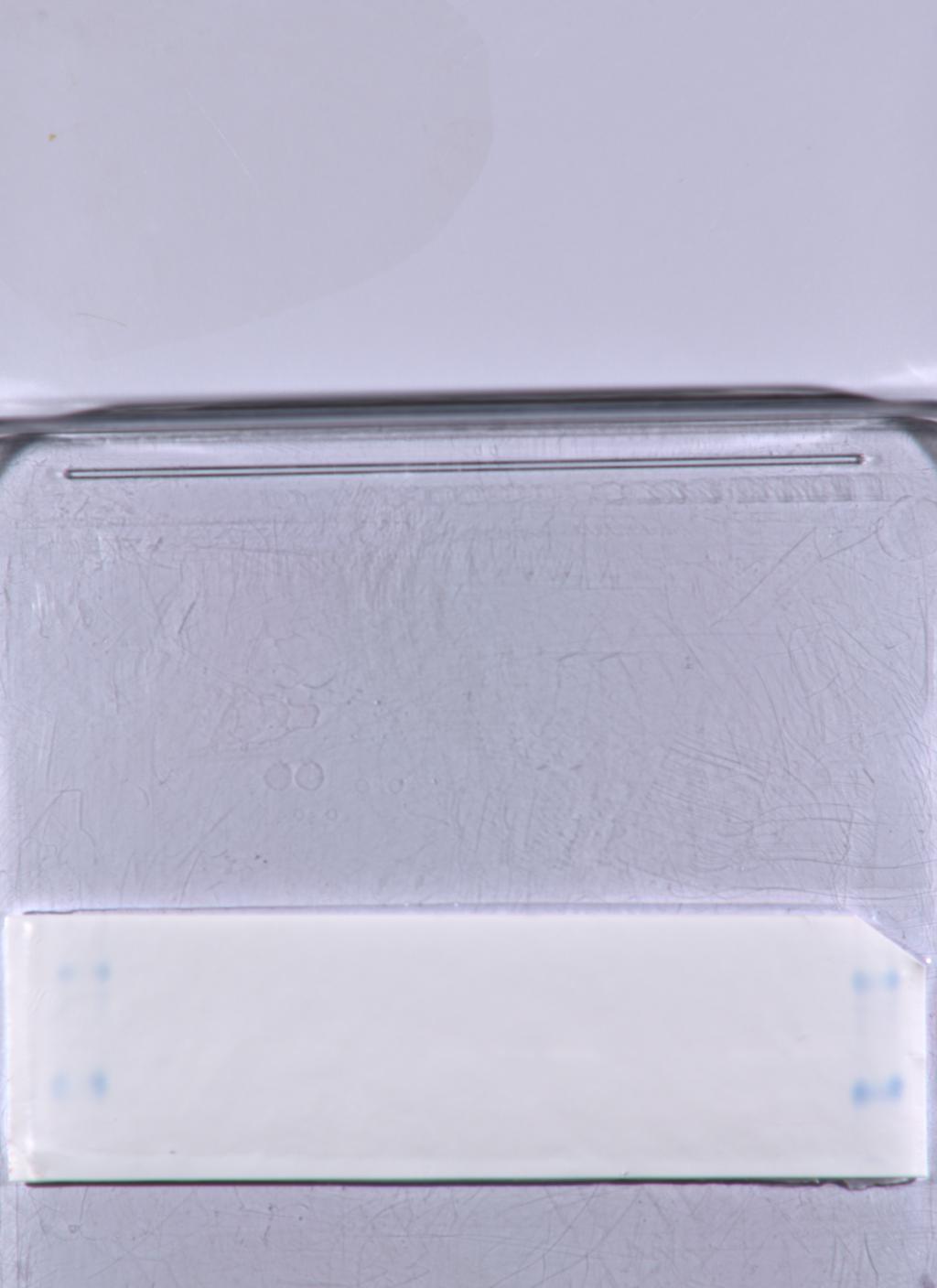

Supplement: Supplementary file 9 — Source data Fig. 4 [file 44321_2024_60_MOESM9_ESM.zip › Figure 4/4C/88T/Western rH2A 0.2/2 1st rH2A 0.2 _Ch-Marker.jpg]

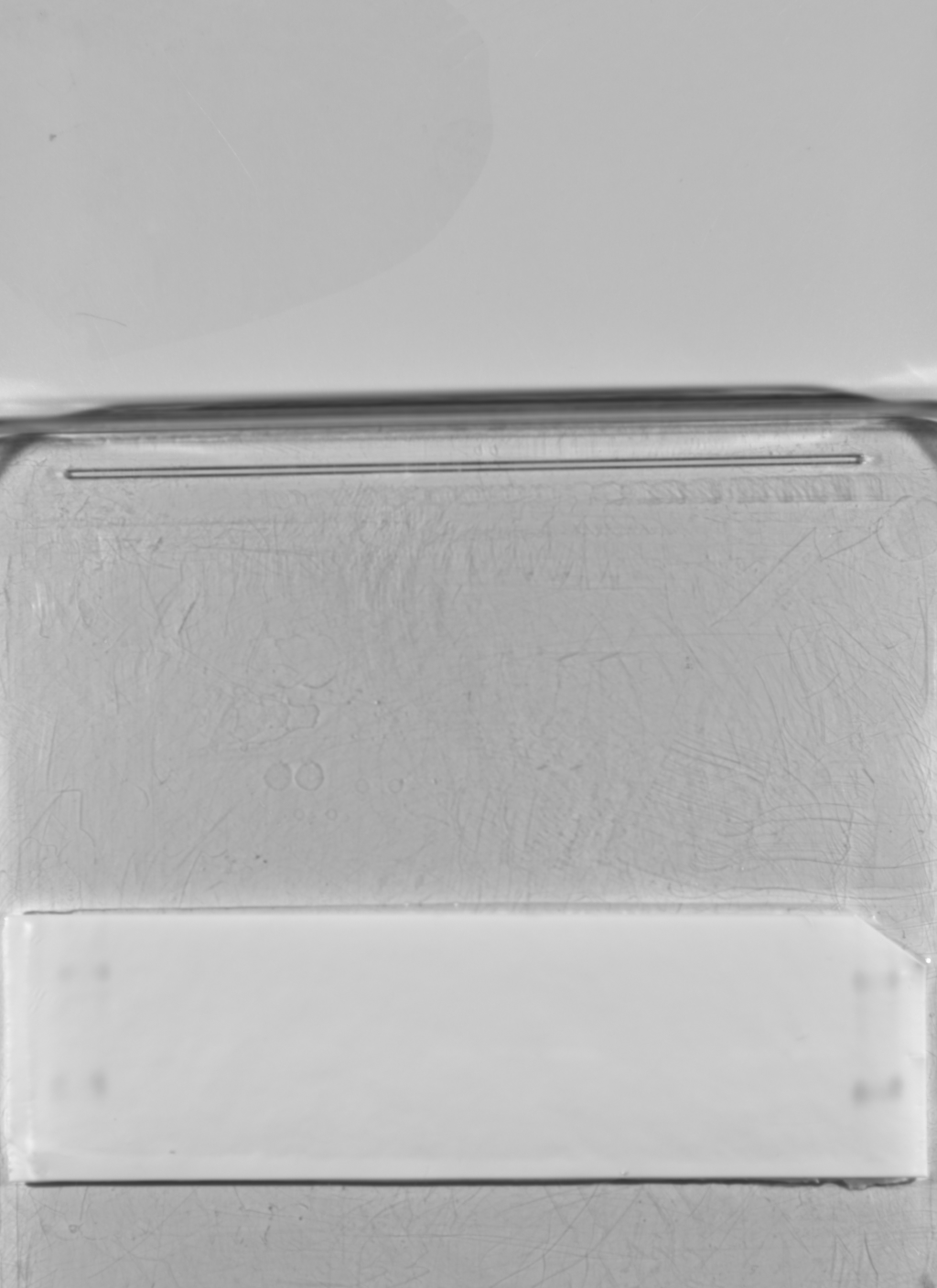

Supplement: Supplementary file 9 — Source data Fig. 4 [file 44321_2024_60_MOESM9_ESM.zip › Figure 4/4C/88T/Western rH2A 0.2/2 1st rH2A 0.2 _Ch-Marker.tif]

## Slide 1
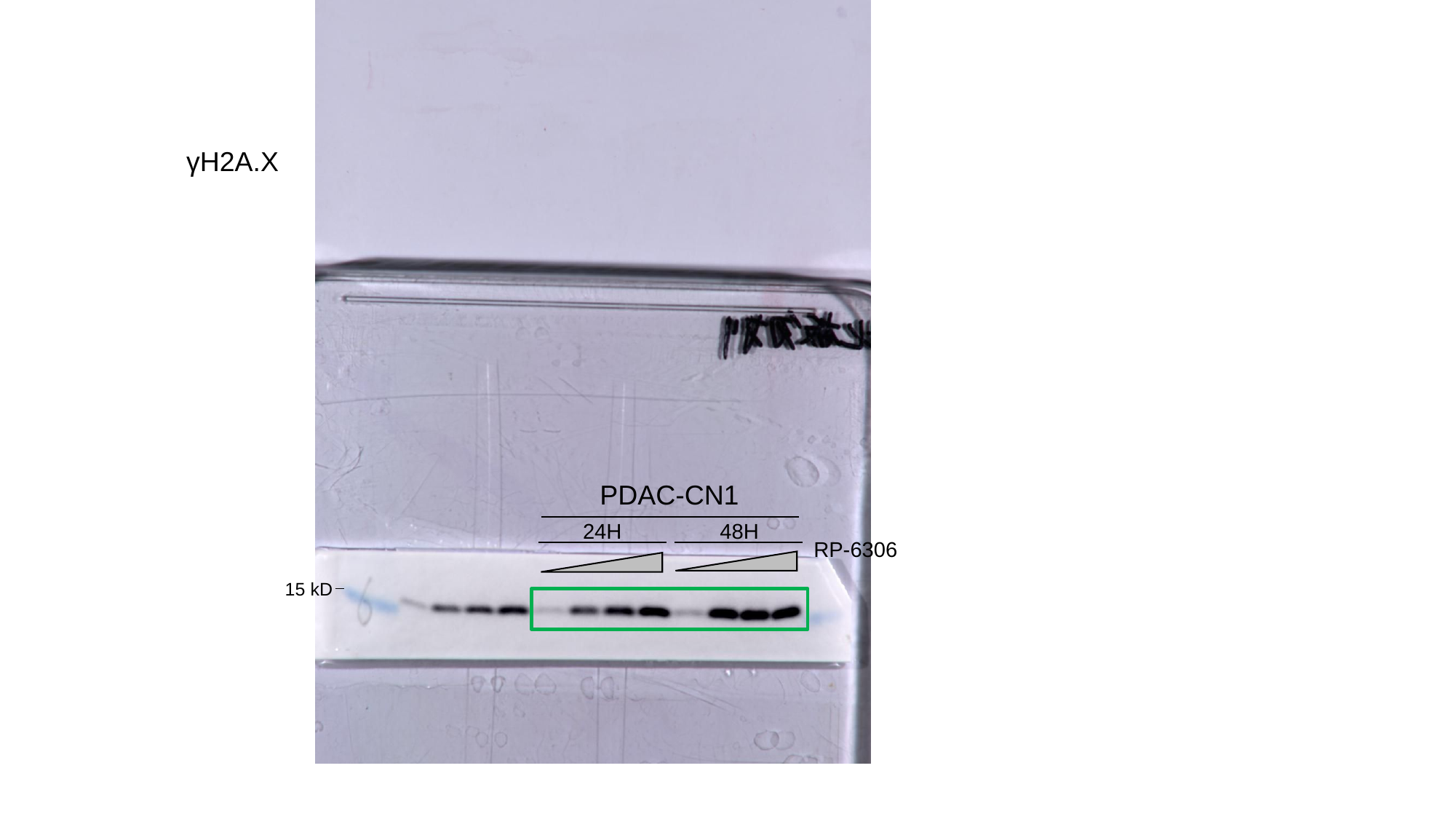

γH2A.X
PDAC-CN1
24H
48H
RP-6306
15 kD

## Slide 2
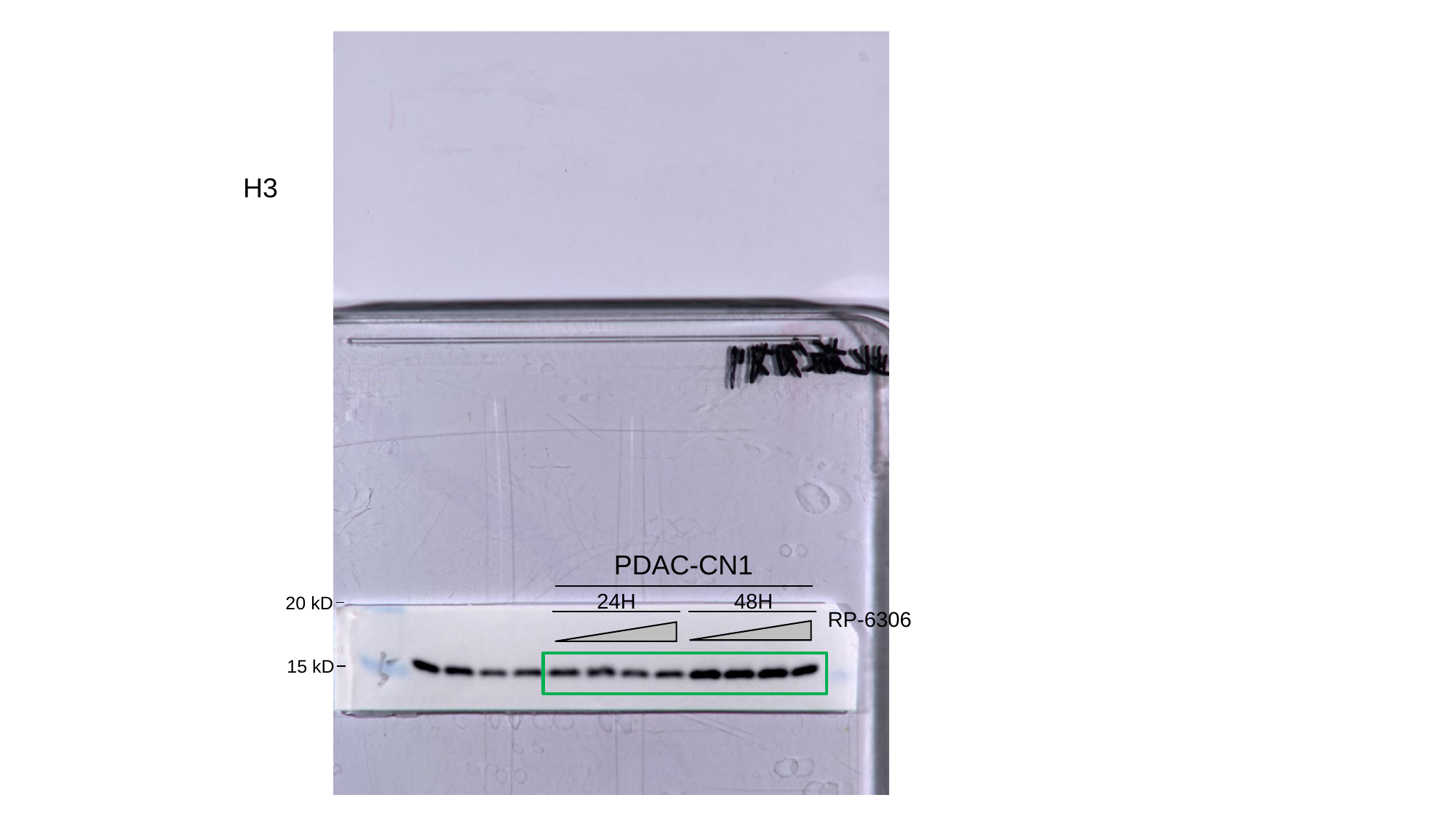

H3
PDAC-CN1
24H
48H
20 kD
RP-6306
15 kD

## Slide 3
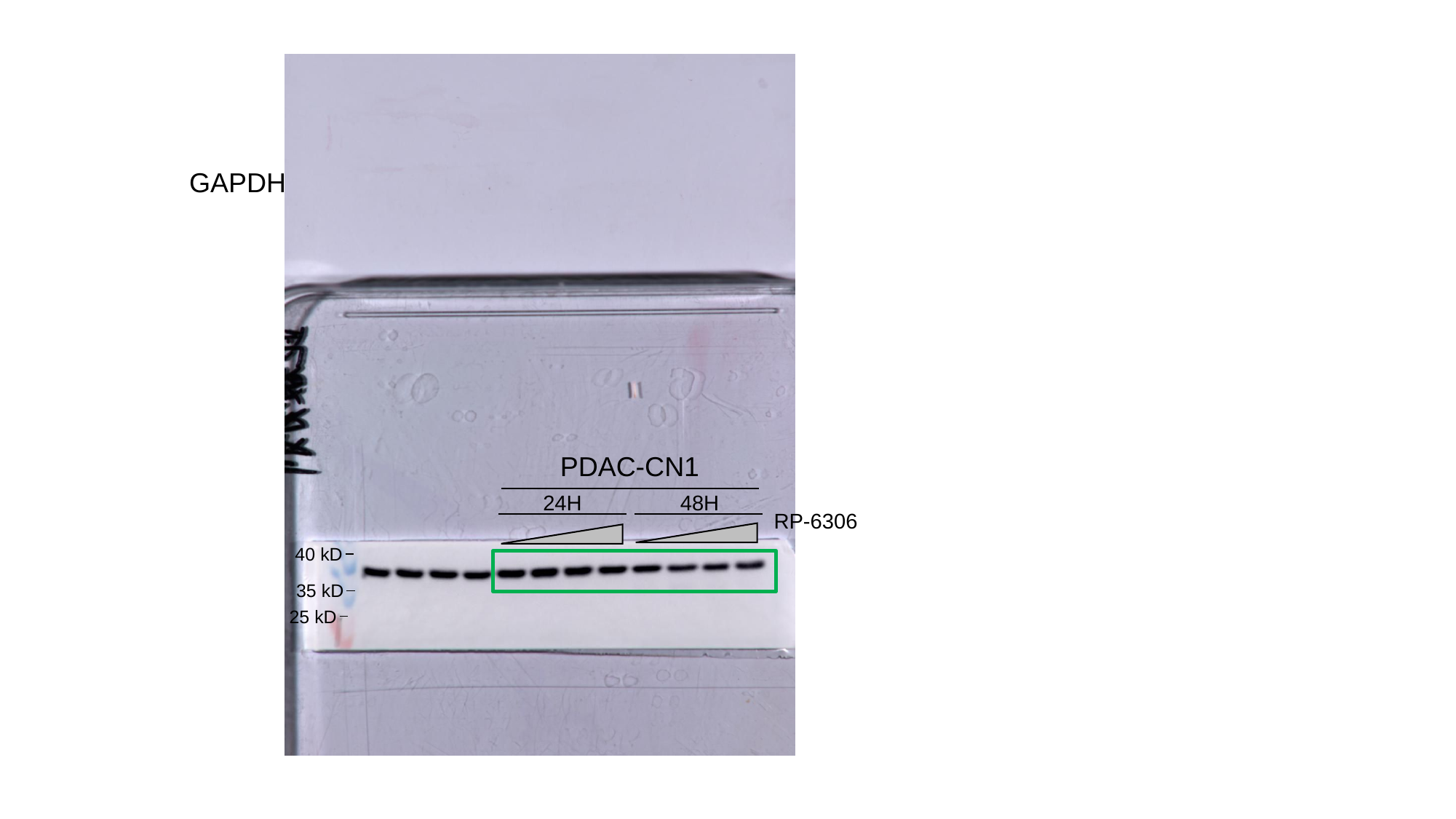

GAPDH
PDAC-CN1
24H
48H
RP-6306
40 kD
35 kD
25 kD

Supplement: Supplementary file 9 — Source data Fig. 4 [file 44321_2024_60_MOESM9_ESM.zip › Figure 4/4C/CN1/4C CN1.pptx]

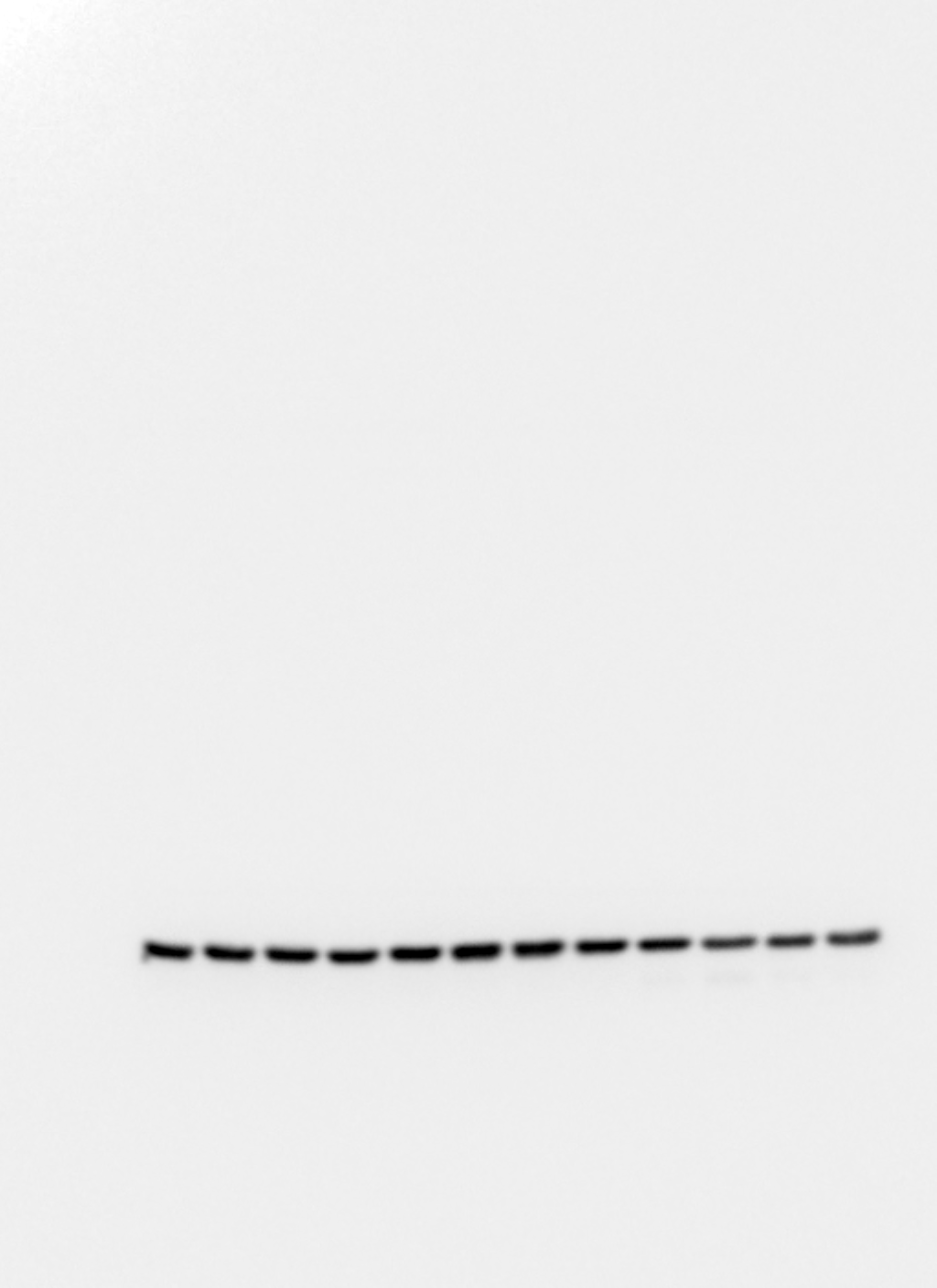

Supplement: Supplementary file 9 — Source data Fig. 4 [file 44321_2024_60_MOESM9_ESM.zip › Figure 4/4C/CN1/Western GAPDH 3.1/1 GAP 3.1 _Ch.tif]

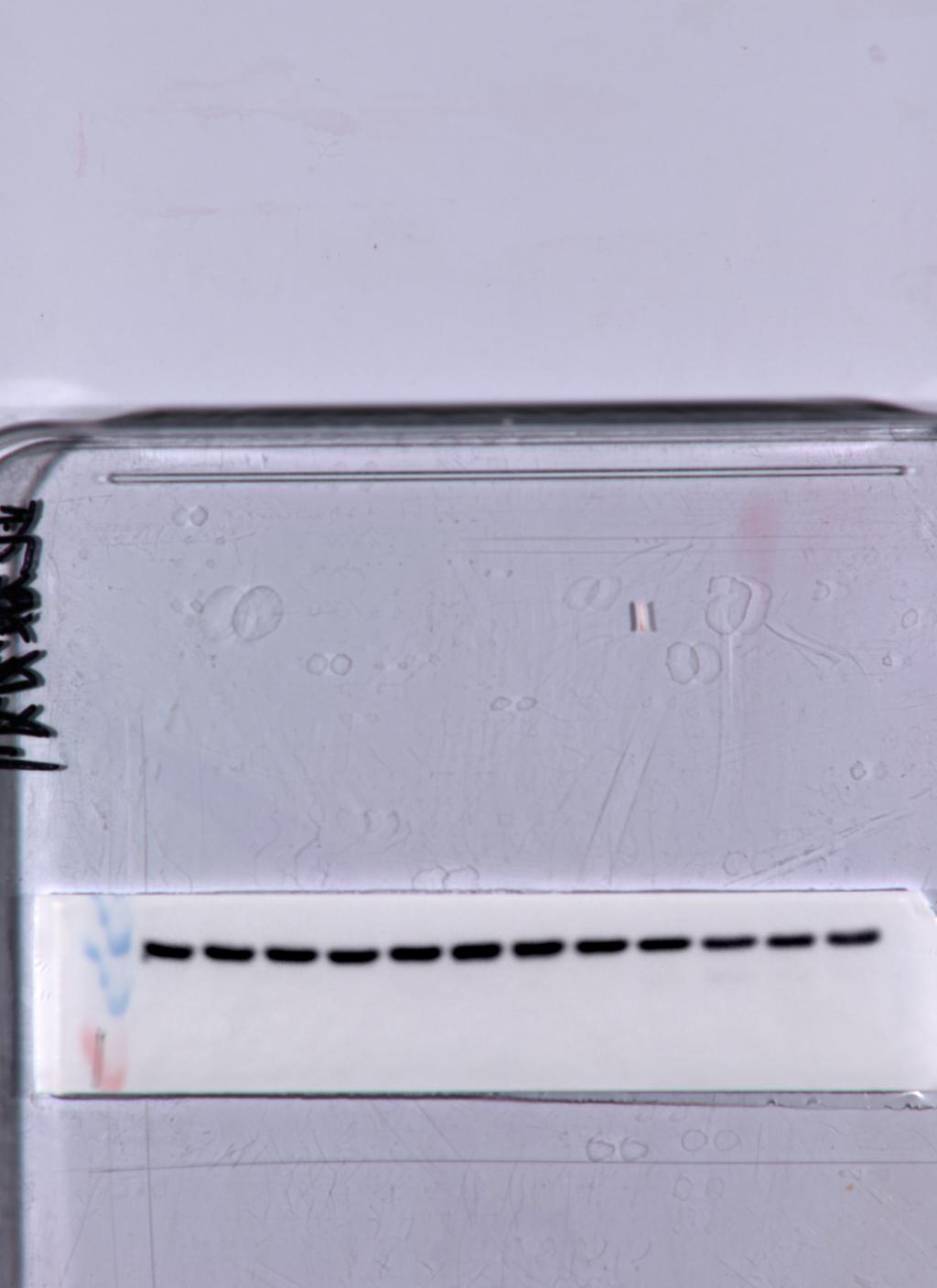

Supplement: Supplementary file 9 — Source data Fig. 4 [file 44321_2024_60_MOESM9_ESM.zip › Figure 4/4C/CN1/Western GAPDH 3.1/1 GAP 3.1 _Ch+Marker.jpg]

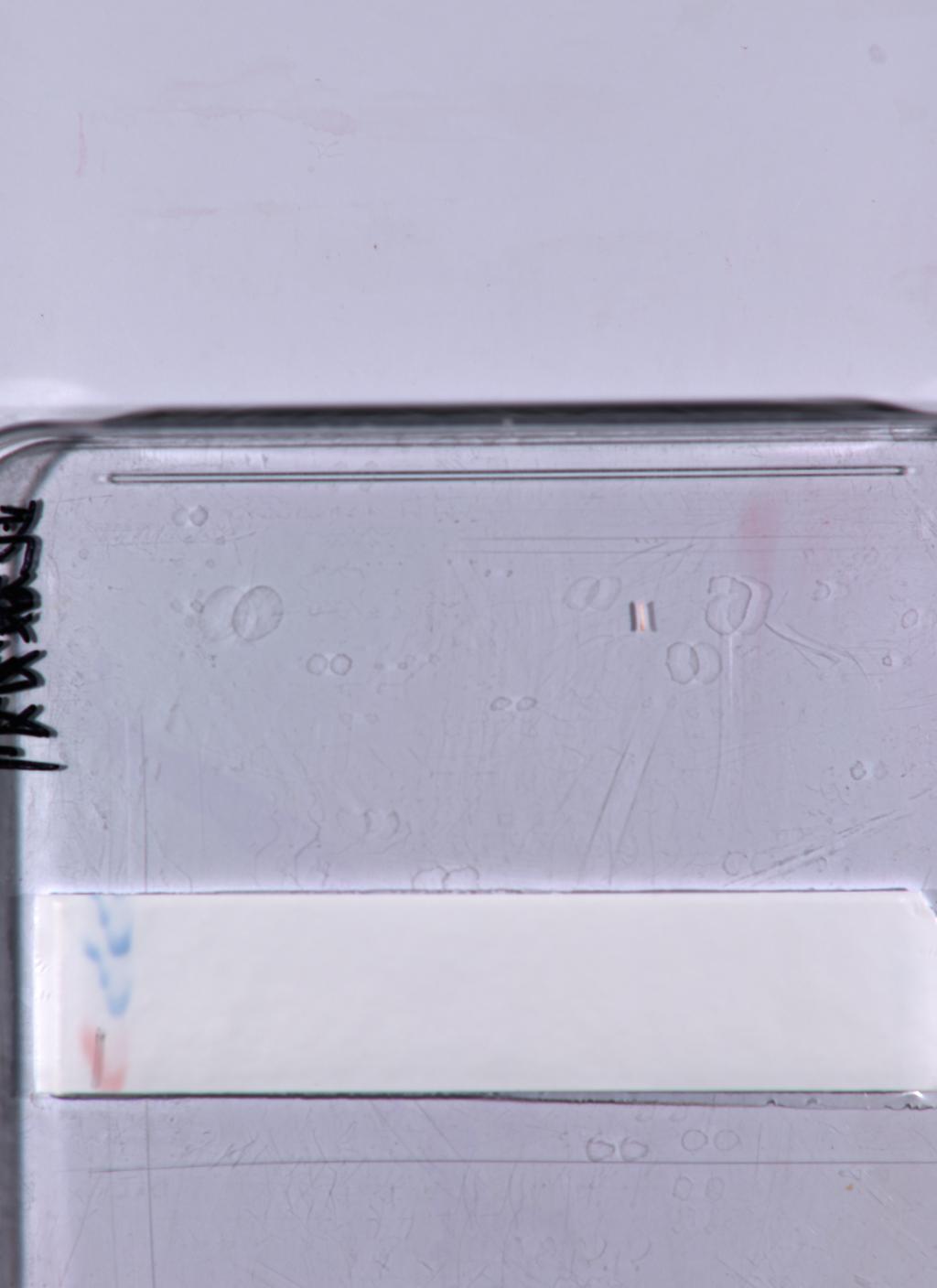

Supplement: Supplementary file 9 — Source data Fig. 4 [file 44321_2024_60_MOESM9_ESM.zip › Figure 4/4C/CN1/Western GAPDH 3.1/1 GAP 3.1 _Ch-Marker.jpg]

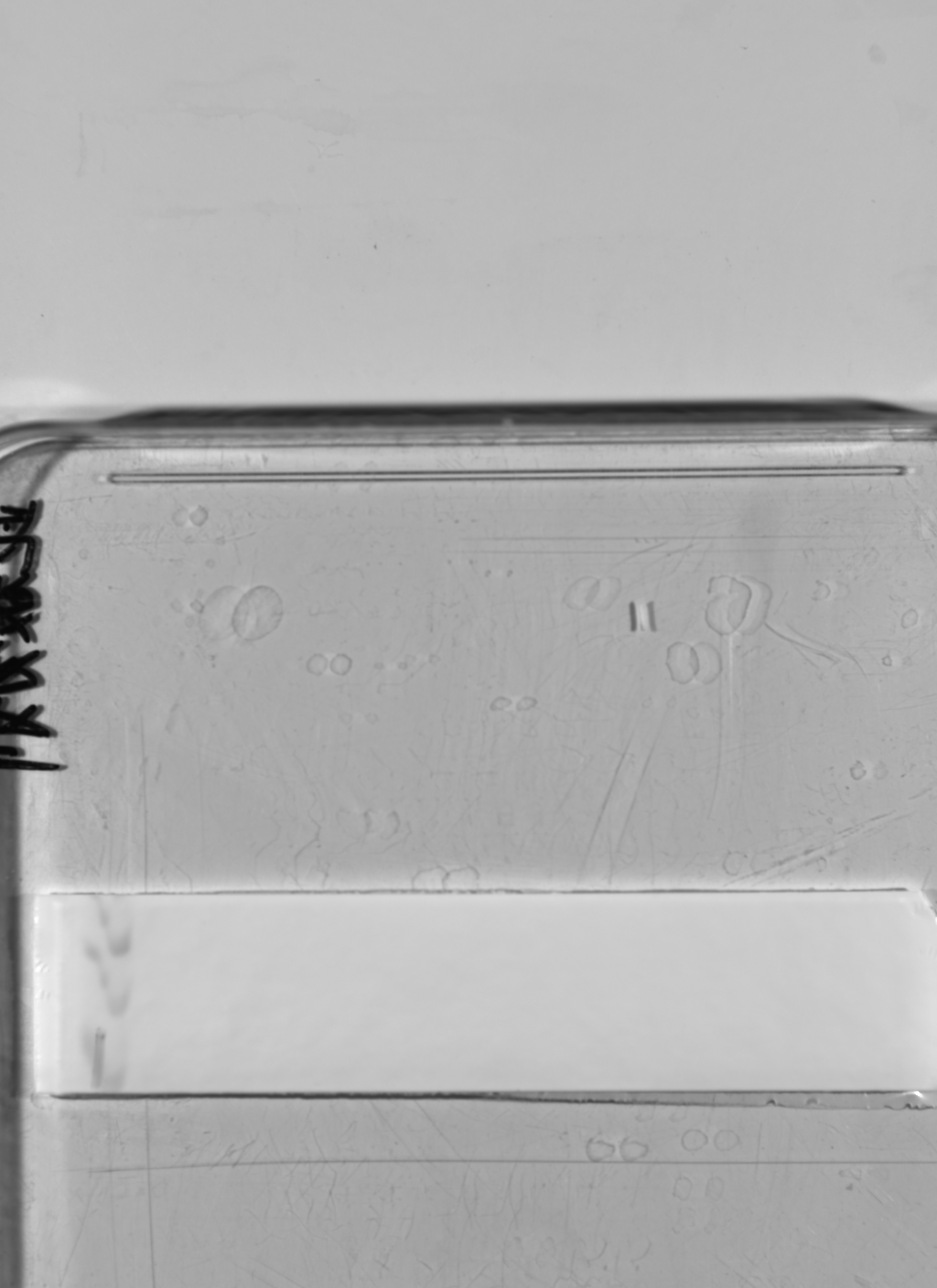

Supplement: Supplementary file 9 — Source data Fig. 4 [file 44321_2024_60_MOESM9_ESM.zip › Figure 4/4C/CN1/Western GAPDH 3.1/1 GAP 3.1 _Ch-Marker.tif]

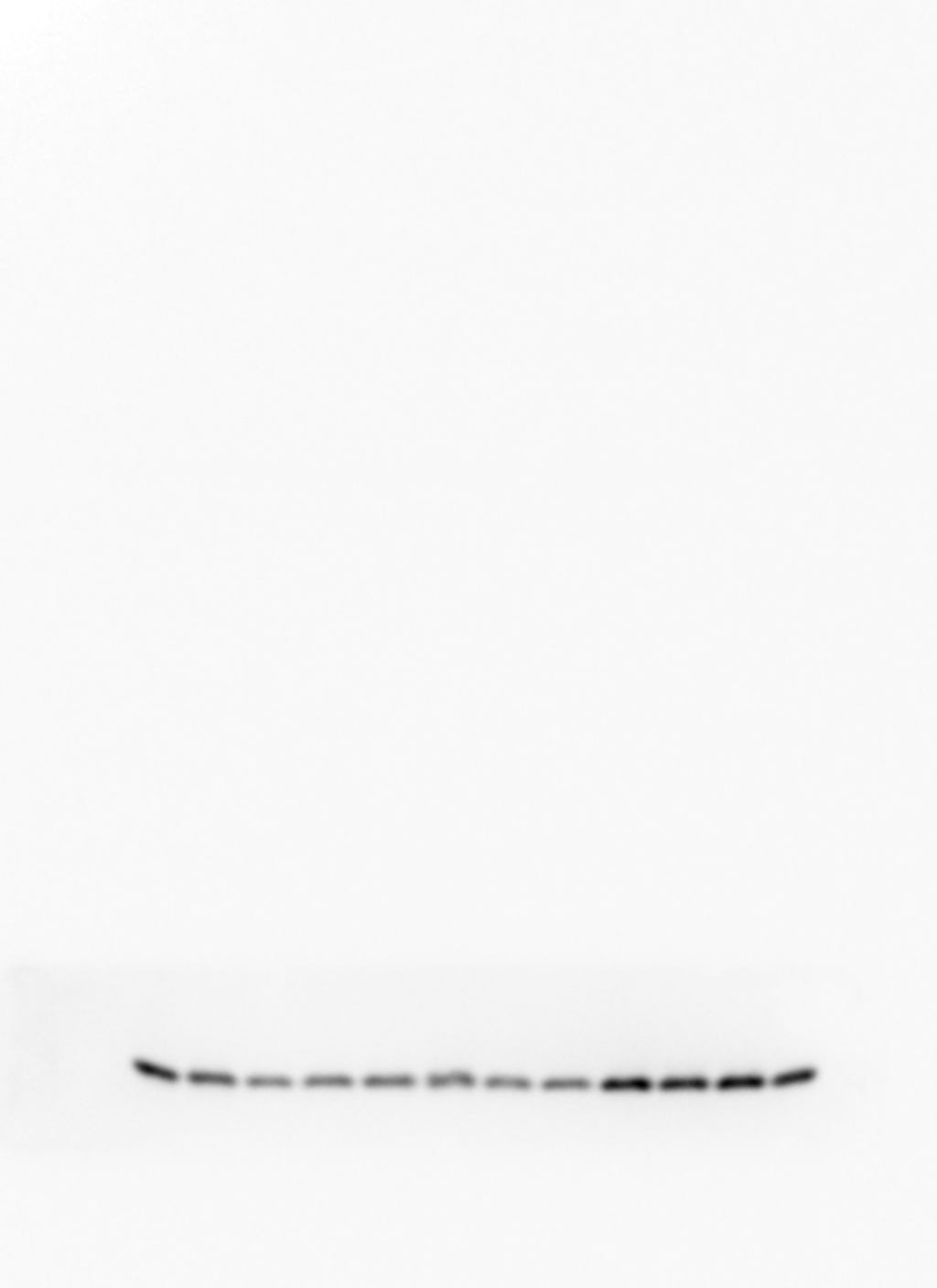

Supplement: Supplementary file 9 — Source data Fig. 4 [file 44321_2024_60_MOESM9_ESM.zip › Figure 4/4C/CN1/Western H3 1.6/5 6th H3 1.6 _Ch.tif]

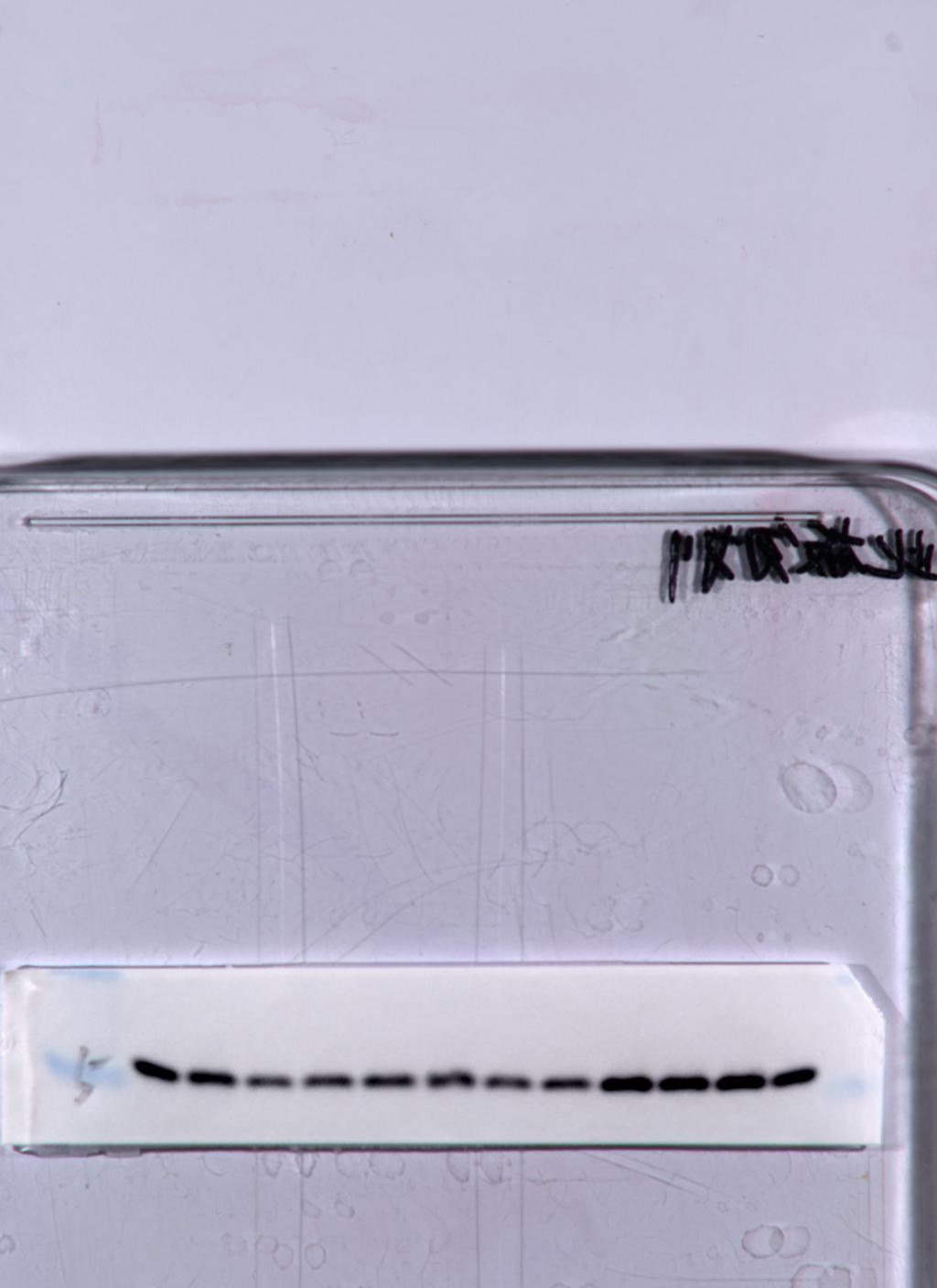

Supplement: Supplementary file 9 — Source data Fig. 4 [file 44321_2024_60_MOESM9_ESM.zip › Figure 4/4C/CN1/Western H3 1.6/5 6th H3 1.6 _Ch+Marker.jpg]

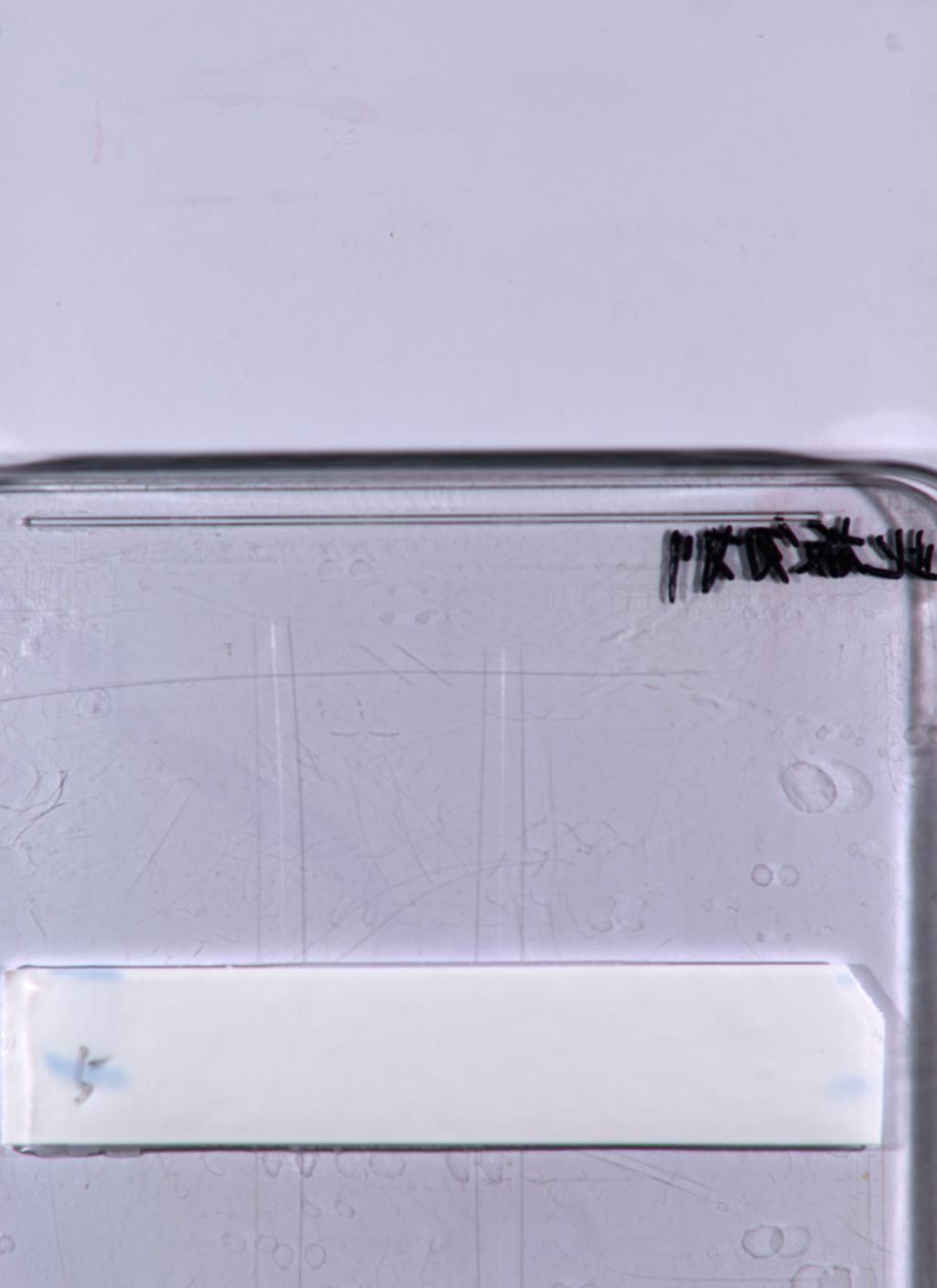

Supplement: Supplementary file 9 — Source data Fig. 4 [file 44321_2024_60_MOESM9_ESM.zip › Figure 4/4C/CN1/Western H3 1.6/5 6th H3 1.6 _Ch-Marker.jpg]

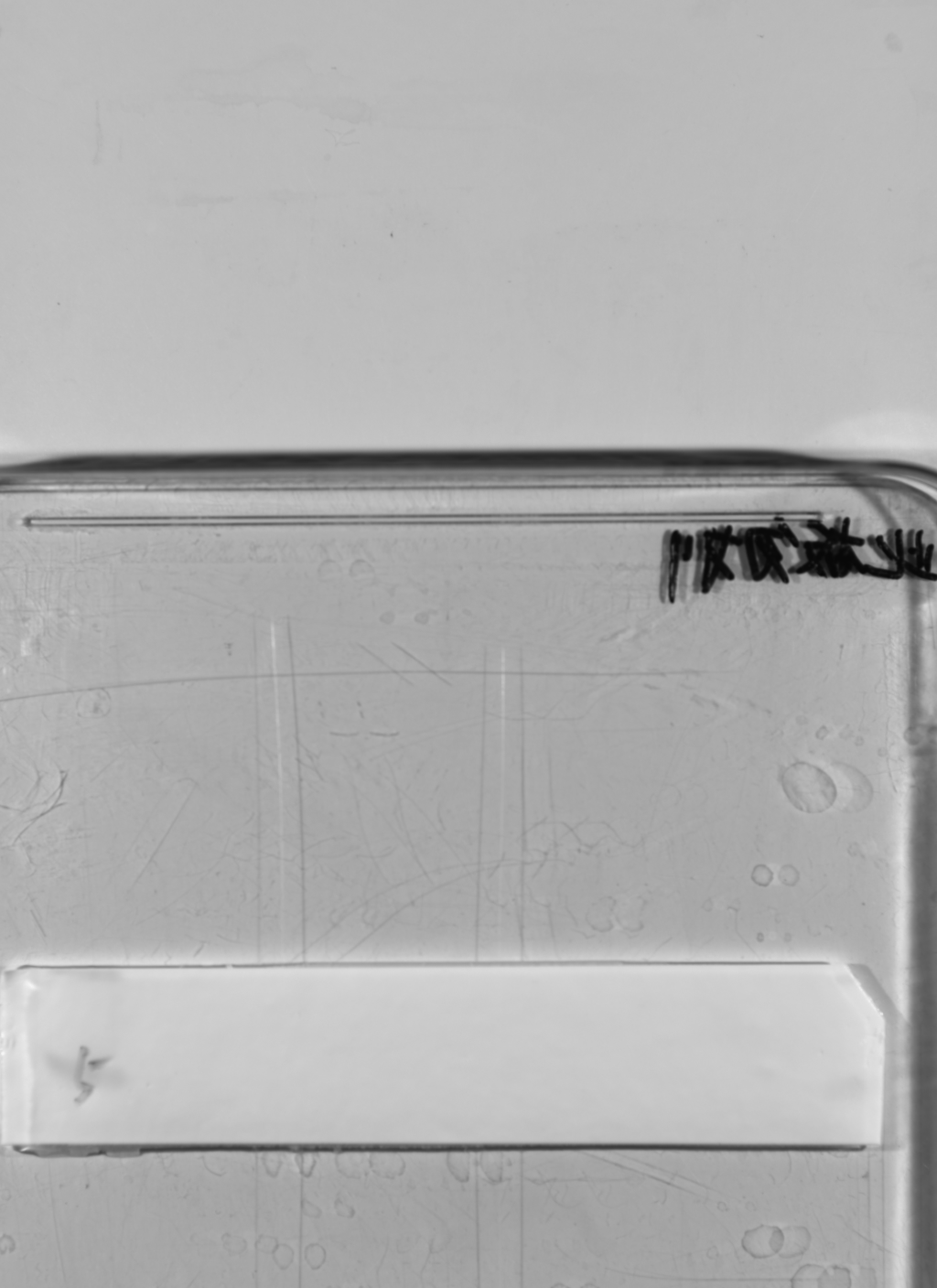

Supplement: Supplementary file 9 — Source data Fig. 4 [file 44321_2024_60_MOESM9_ESM.zip › Figure 4/4C/CN1/Western H3 1.6/5 6th H3 1.6 _Ch-Marker.tif]

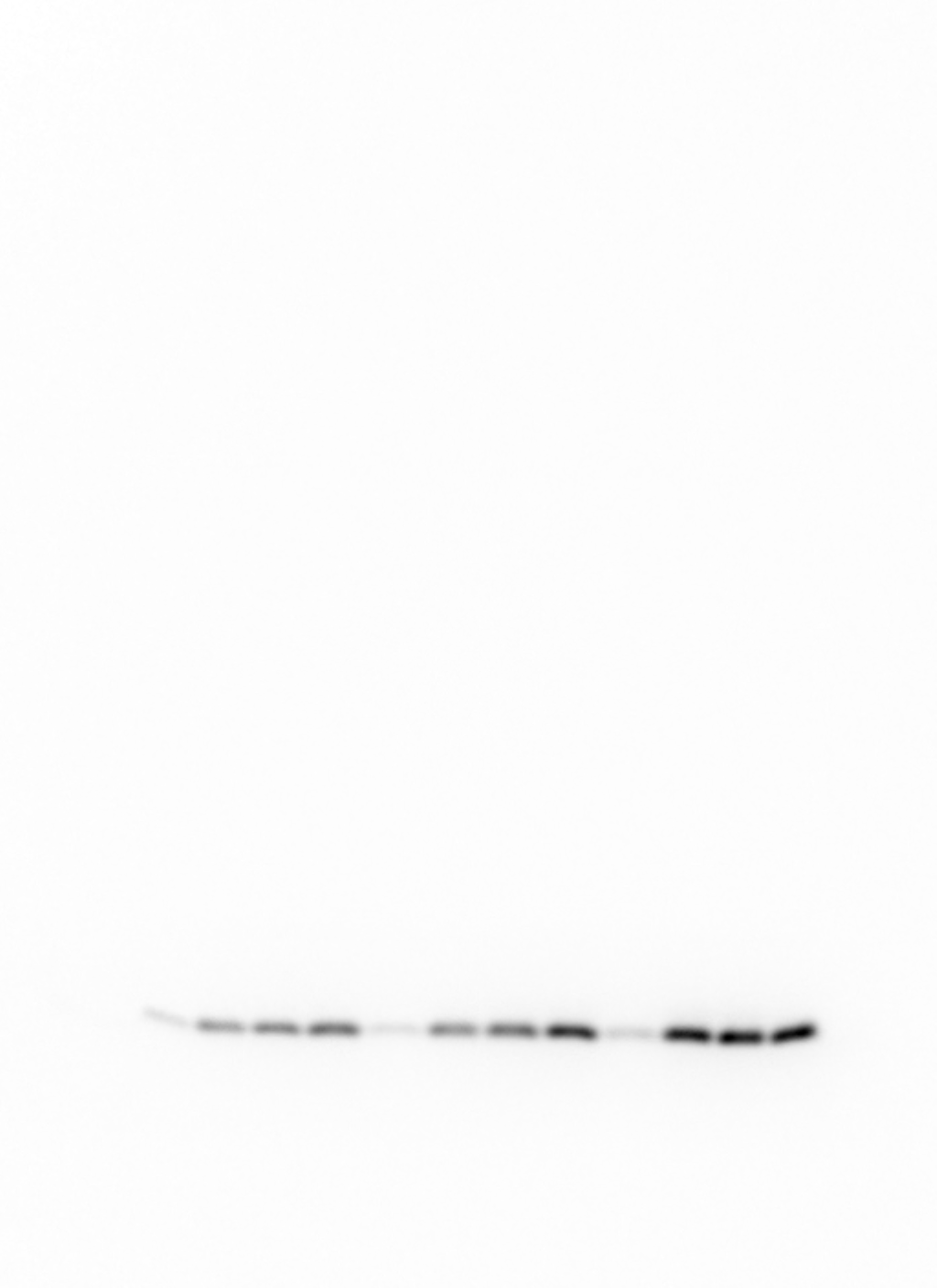

Supplement: Supplementary file 9 — Source data Fig. 4 [file 44321_2024_60_MOESM9_ESM.zip › Figure 4/4C/CN1/Western rH2A 0.4/6 6th rH2A 0.4 _Ch.tif]

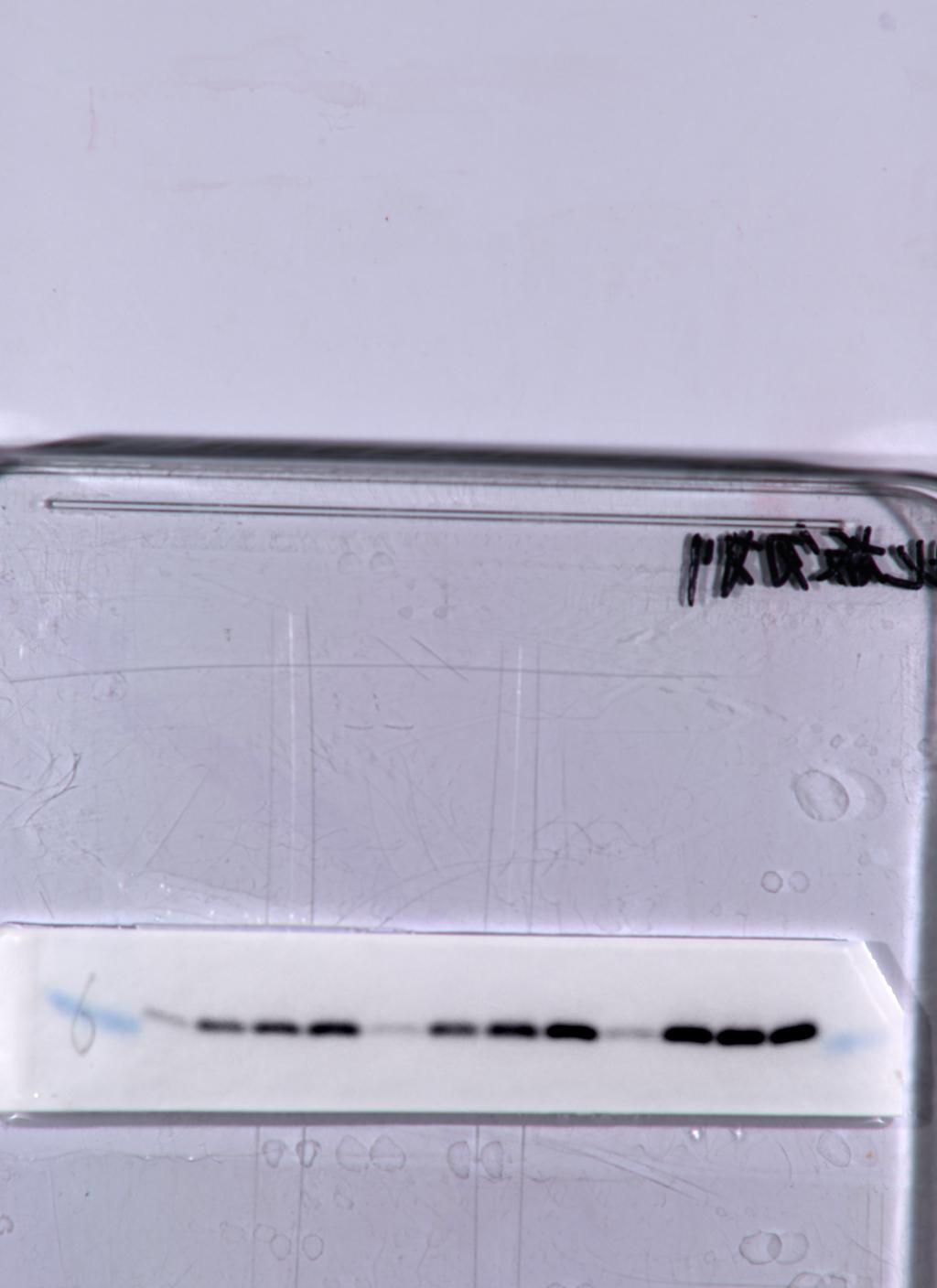

Supplement: Supplementary file 9 — Source data Fig. 4 [file 44321_2024_60_MOESM9_ESM.zip › Figure 4/4C/CN1/Western rH2A 0.4/6 6th rH2A 0.4 _Ch+Marker.jpg]

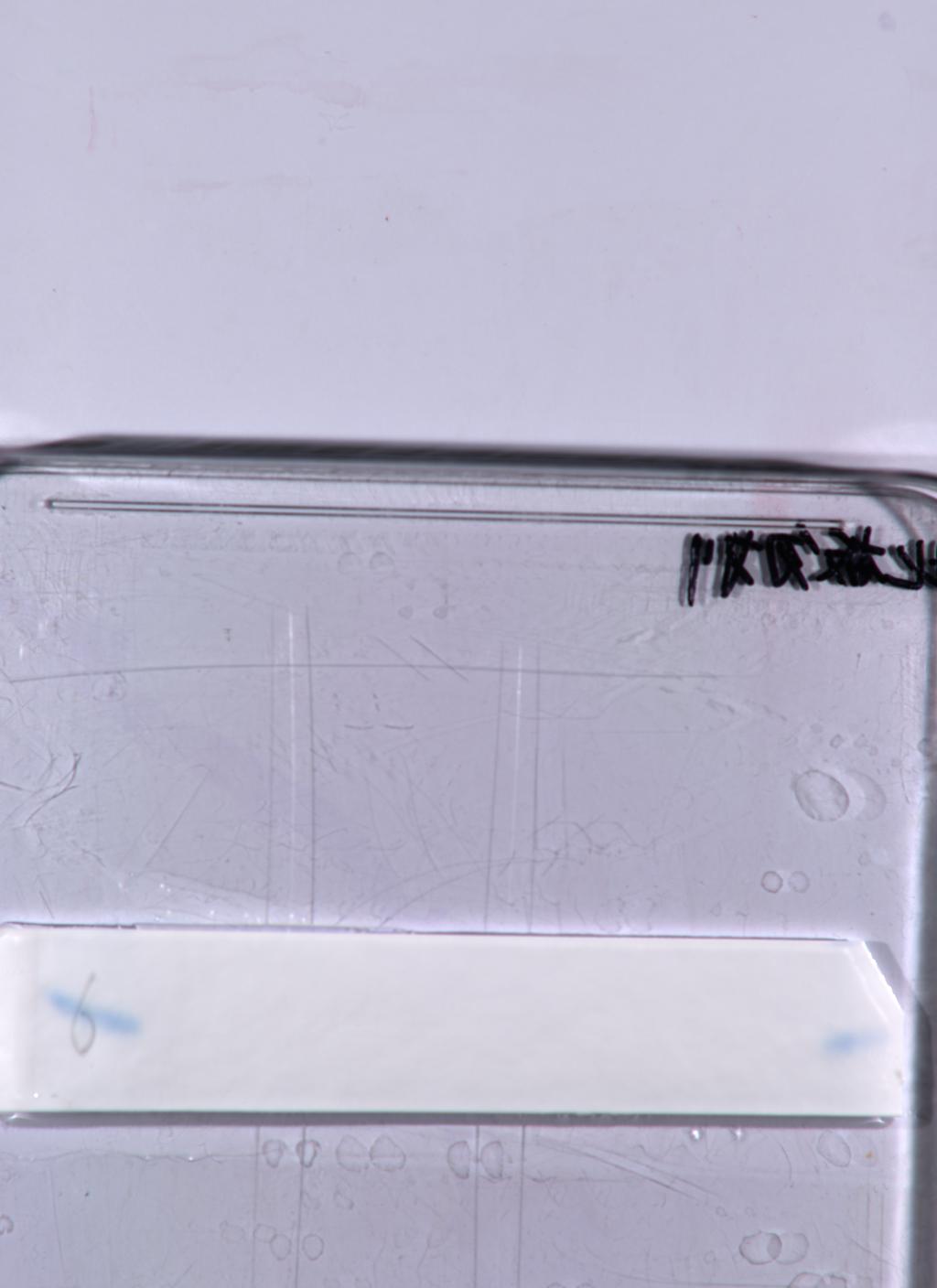

Supplement: Supplementary file 9 — Source data Fig. 4 [file 44321_2024_60_MOESM9_ESM.zip › Figure 4/4C/CN1/Western rH2A 0.4/6 6th rH2A 0.4 _Ch-Marker.jpg]

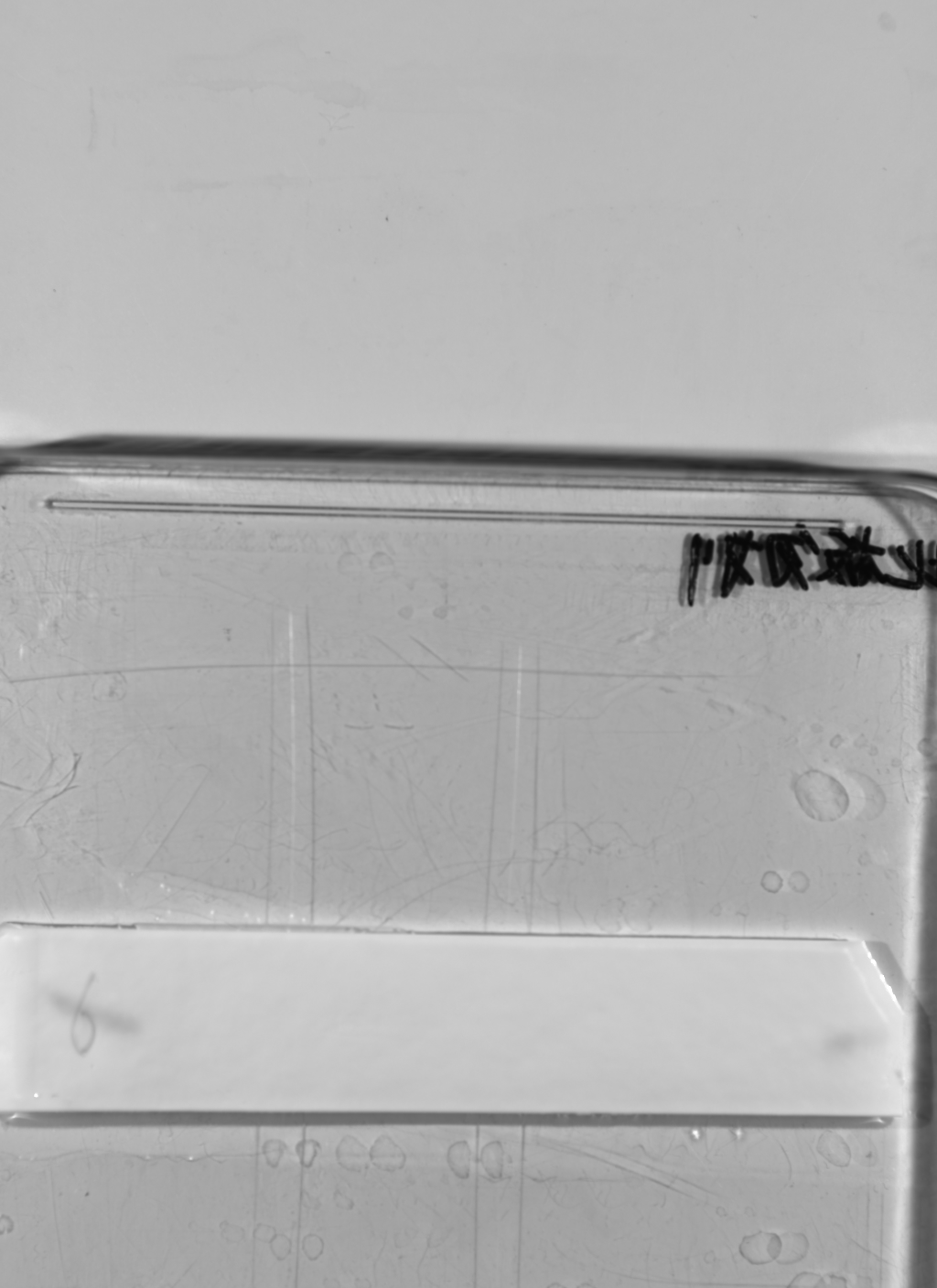

Supplement: Supplementary file 9 — Source data Fig. 4 [file 44321_2024_60_MOESM9_ESM.zip › Figure 4/4C/CN1/Western rH2A 0.4/6 6th rH2A 0.4 _Ch-Marker.tif]

## Slide 1
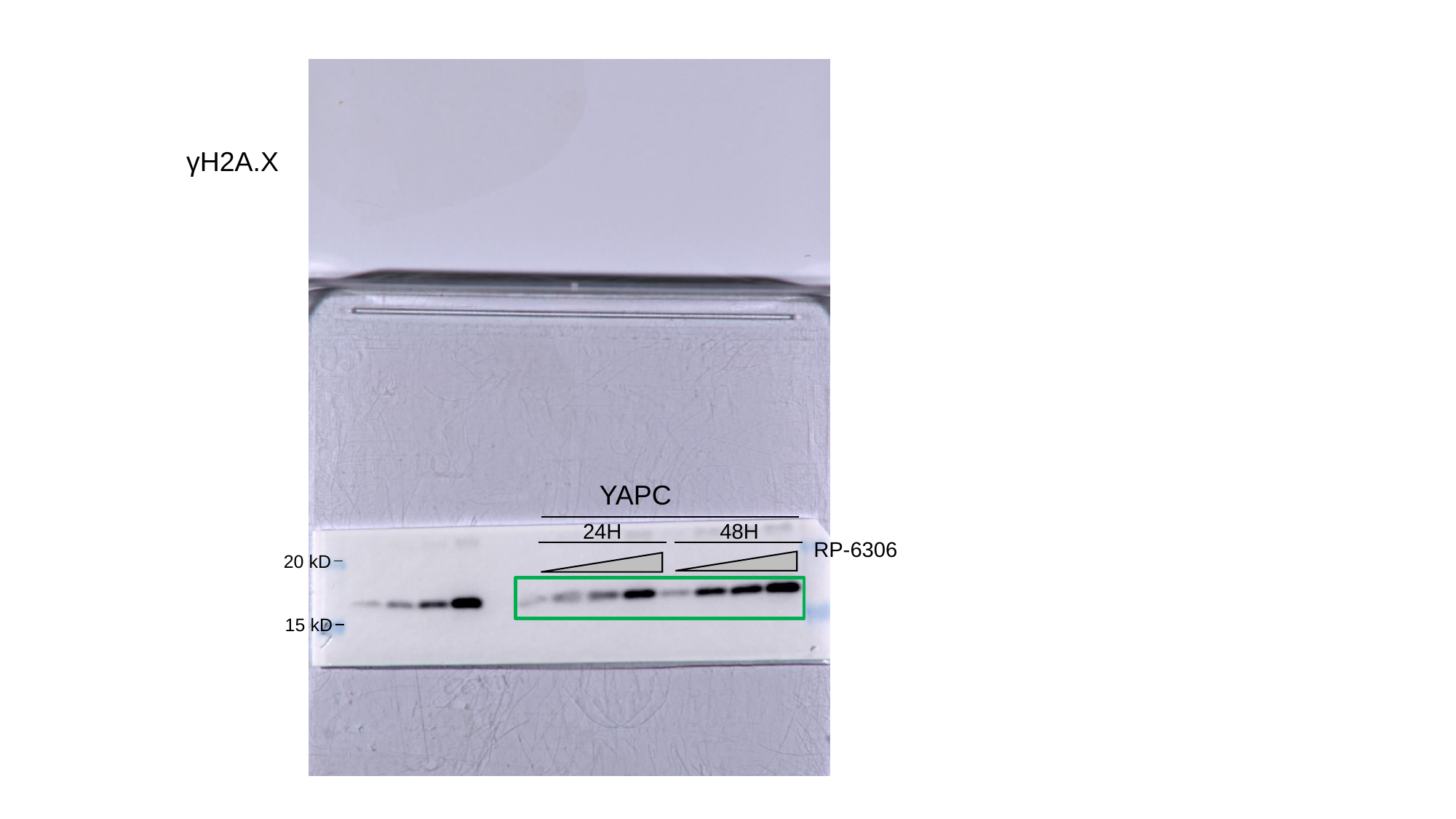

γH2A.X
YAPC
24H
48H
RP-6306
20 kD
15 kD

## Slide 2
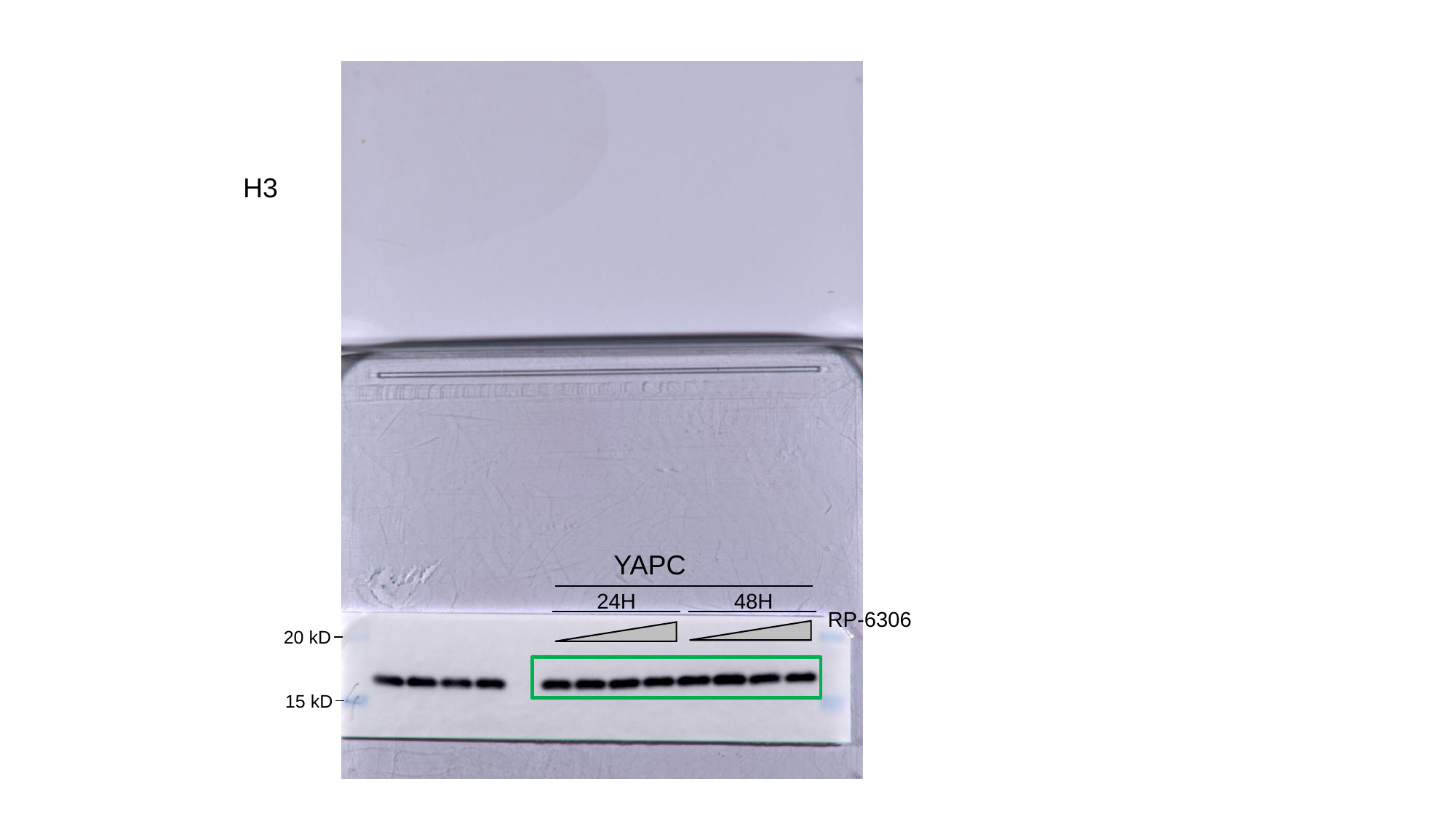

H3
YAPC
24H
48H
RP-6306
20 kD
15 kD

## Slide 3
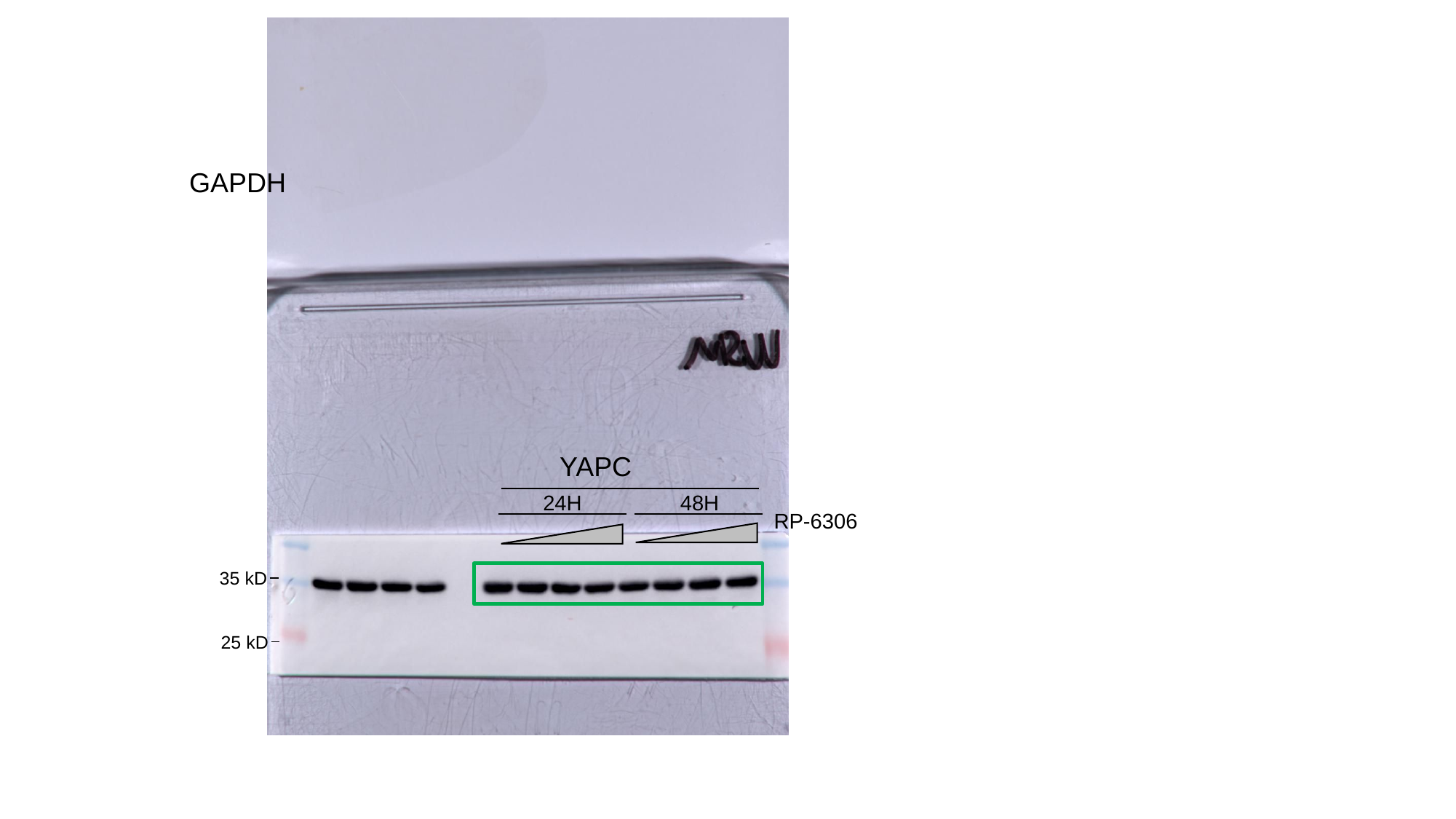

GAPDH
YAPC
24H
48H
RP-6306
35 kD
25 kD

Supplement: Supplementary file 9 — Source data Fig. 4 [file 44321_2024_60_MOESM9_ESM.zip › Figure 4/4C/YAPC/4C YAPC.pptx]

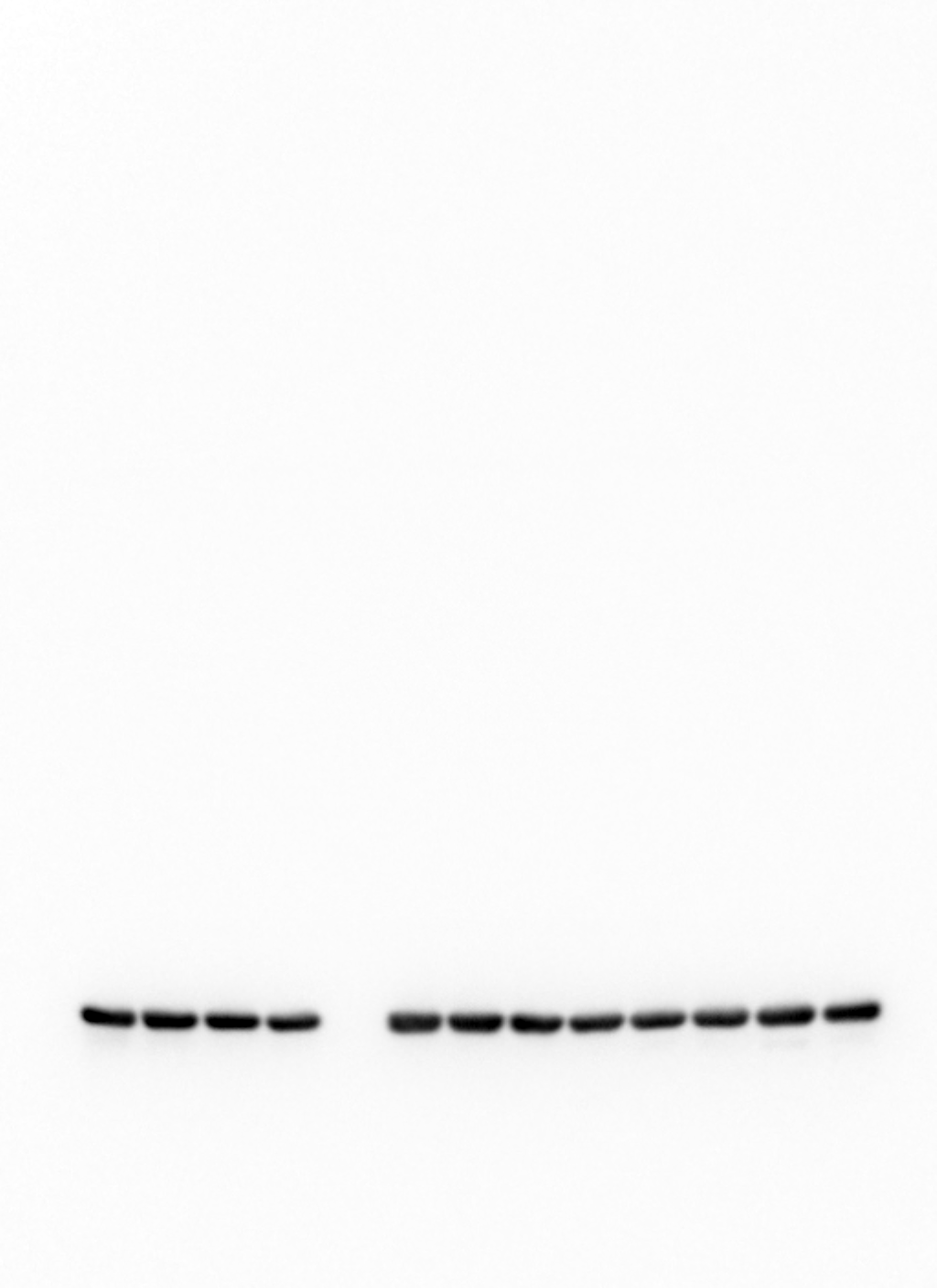

Supplement: Supplementary file 9 — Source data Fig. 4 [file 44321_2024_60_MOESM9_ESM.zip › Figure 4/4C/YAPC/Western GAPDH 0.4/6 GAP 0.4 _Ch.tif]

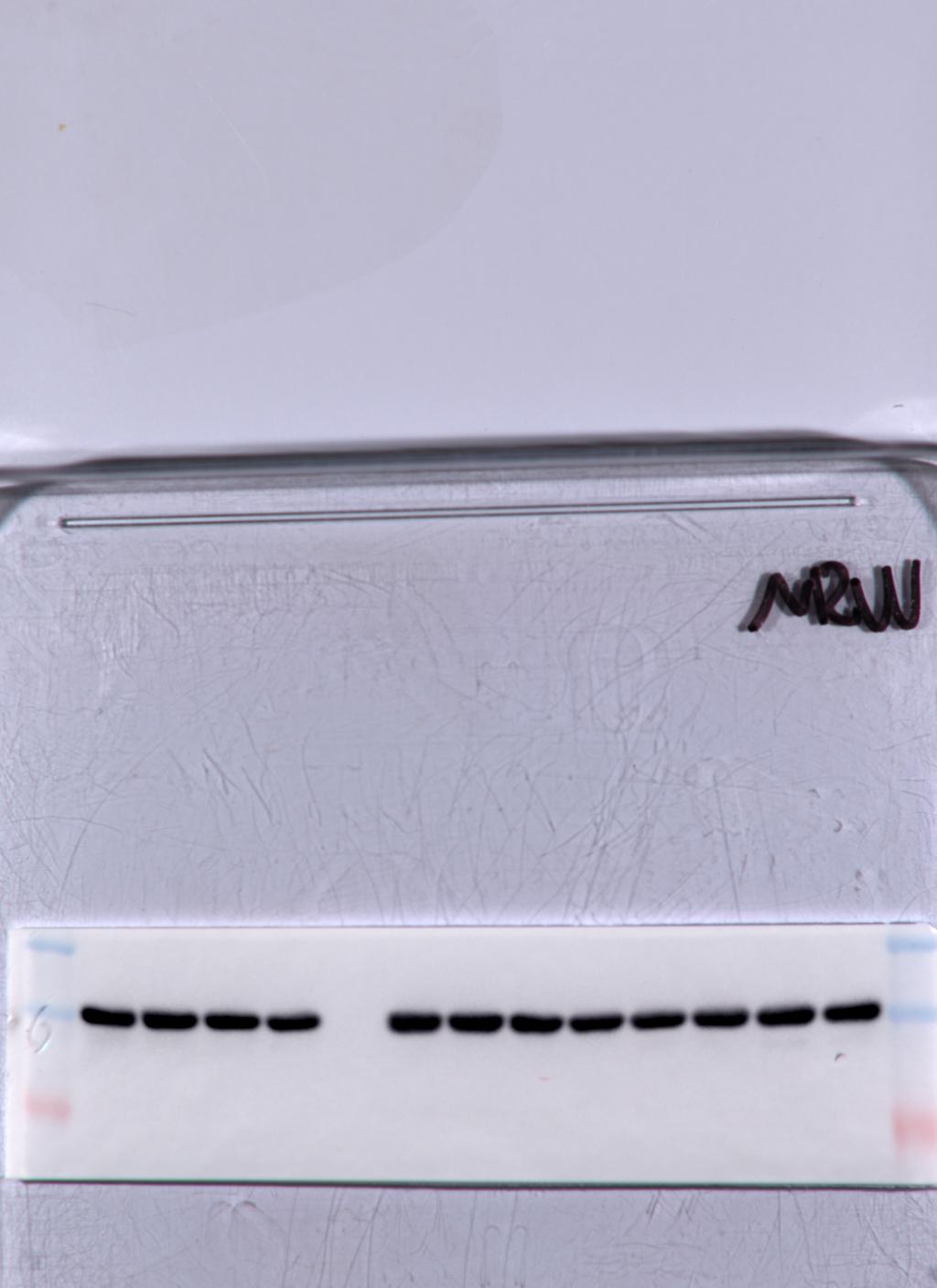

Supplement: Supplementary file 9 — Source data Fig. 4 [file 44321_2024_60_MOESM9_ESM.zip › Figure 4/4C/YAPC/Western GAPDH 0.4/6 GAP 0.4 _Ch+Marker.jpg]

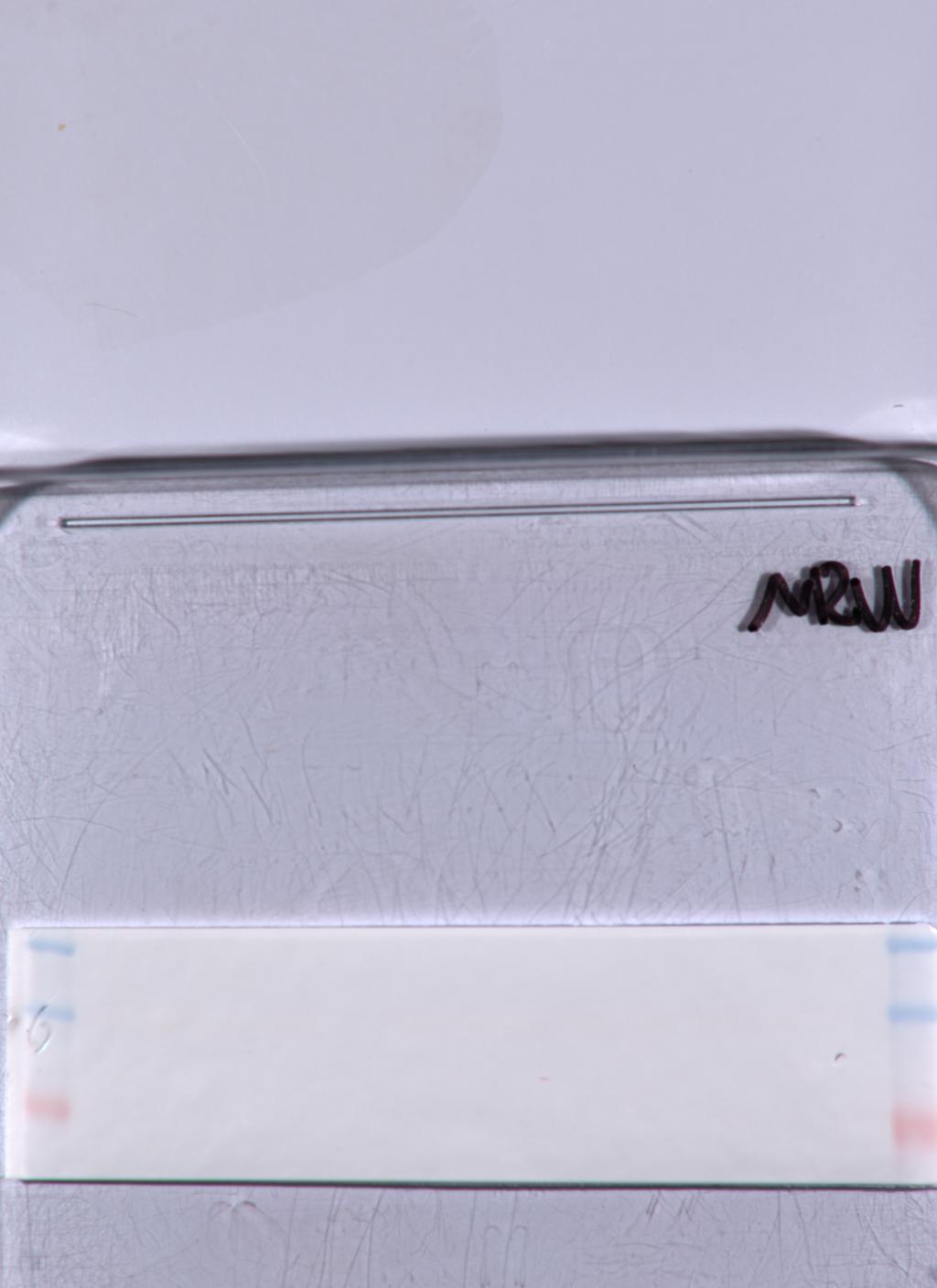

Supplement: Supplementary file 9 — Source data Fig. 4 [file 44321_2024_60_MOESM9_ESM.zip › Figure 4/4C/YAPC/Western GAPDH 0.4/6 GAP 0.4 _Ch-Marker.jpg]

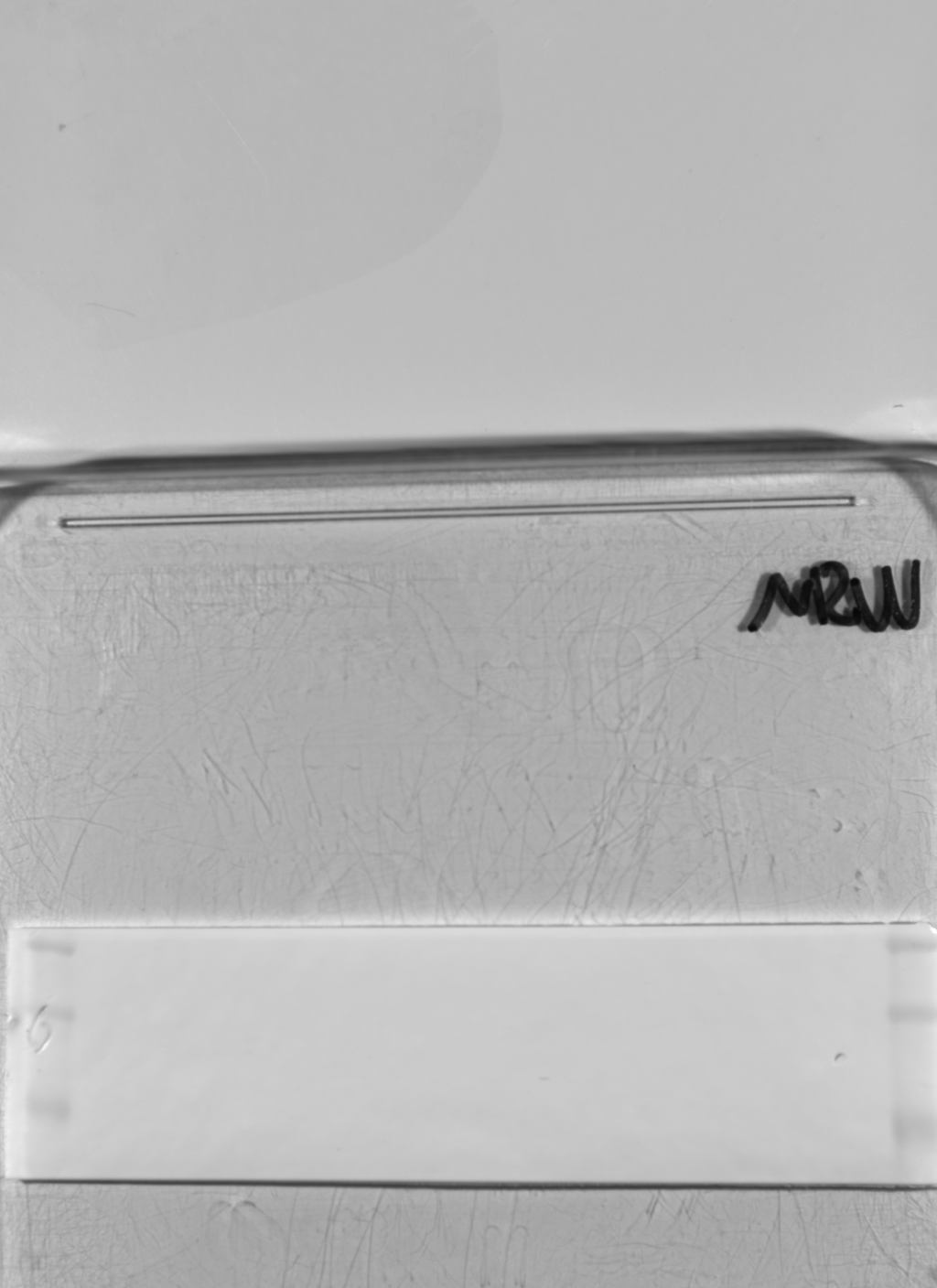

Supplement: Supplementary file 9 — Source data Fig. 4 [file 44321_2024_60_MOESM9_ESM.zip › Figure 4/4C/YAPC/Western GAPDH 0.4/6 GAP 0.4 _Ch-Marker.tif]

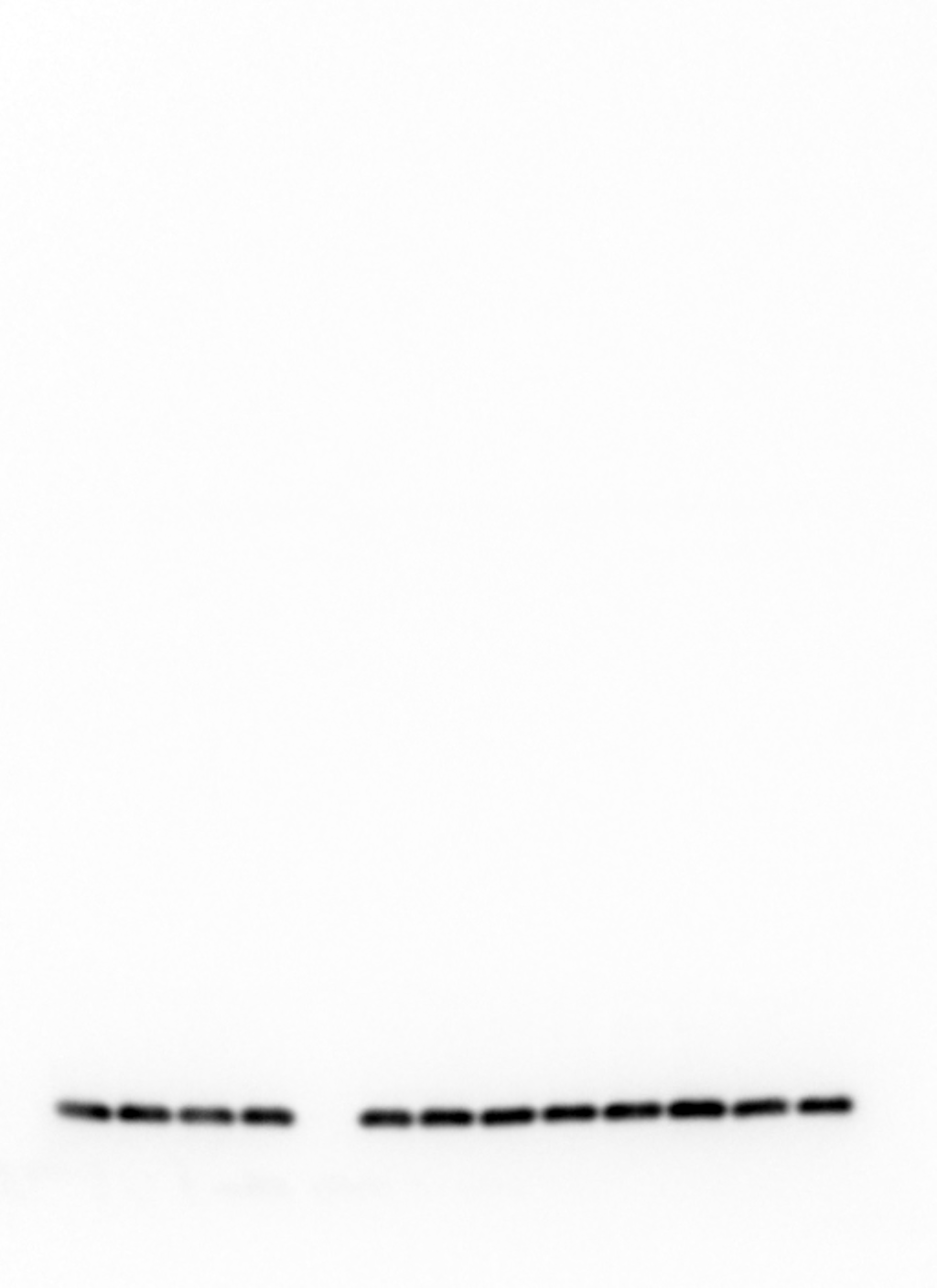

Supplement: Supplementary file 9 — Source data Fig. 4 [file 44321_2024_60_MOESM9_ESM.zip › Figure 4/4C/YAPC/Western H3 0.3/4 3rd H3 0.3 _Ch.tif]

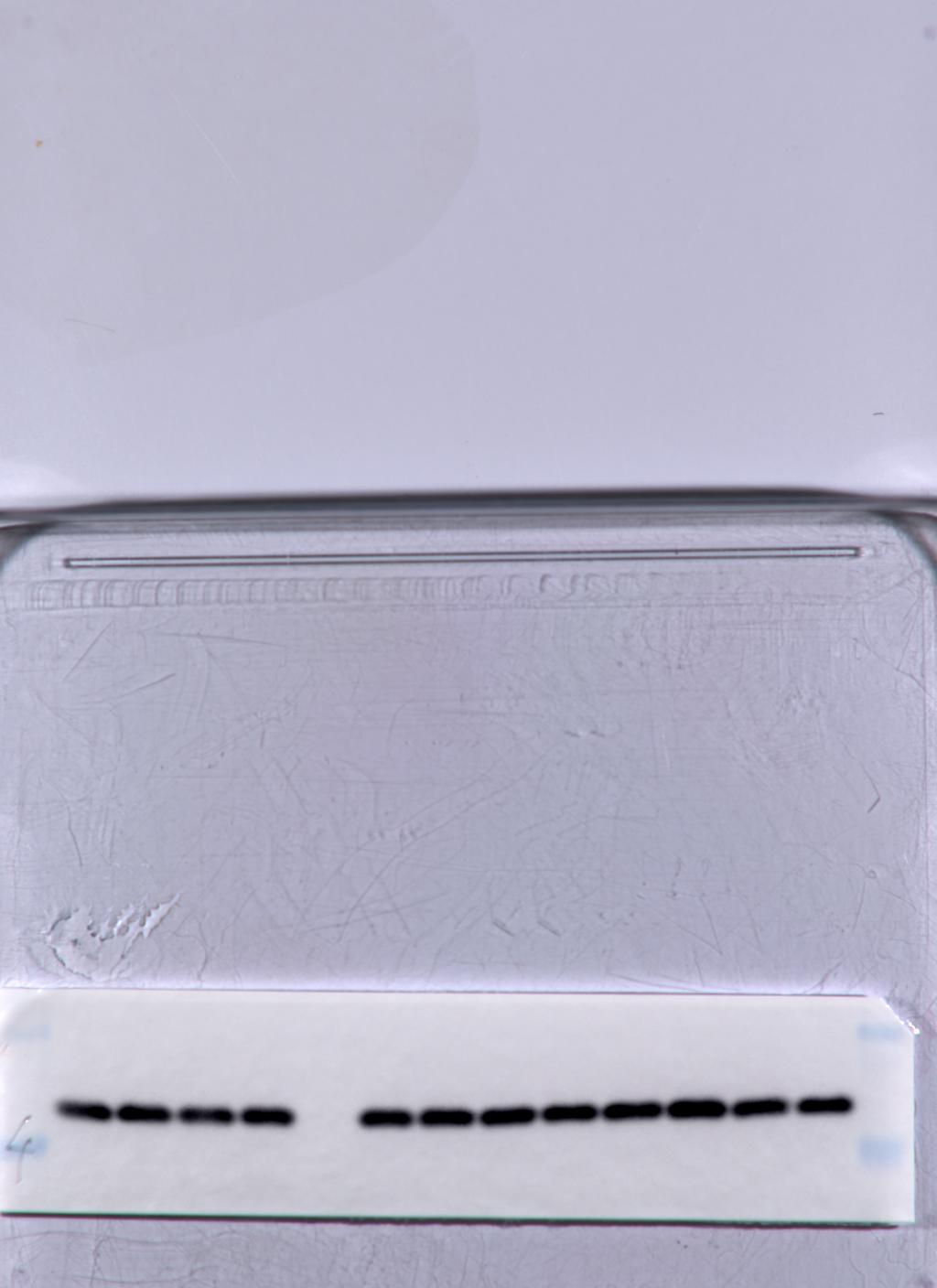

Supplement: Supplementary file 9 — Source data Fig. 4 [file 44321_2024_60_MOESM9_ESM.zip › Figure 4/4C/YAPC/Western H3 0.3/4 3rd H3 0.3 _Ch+Marker.jpg]

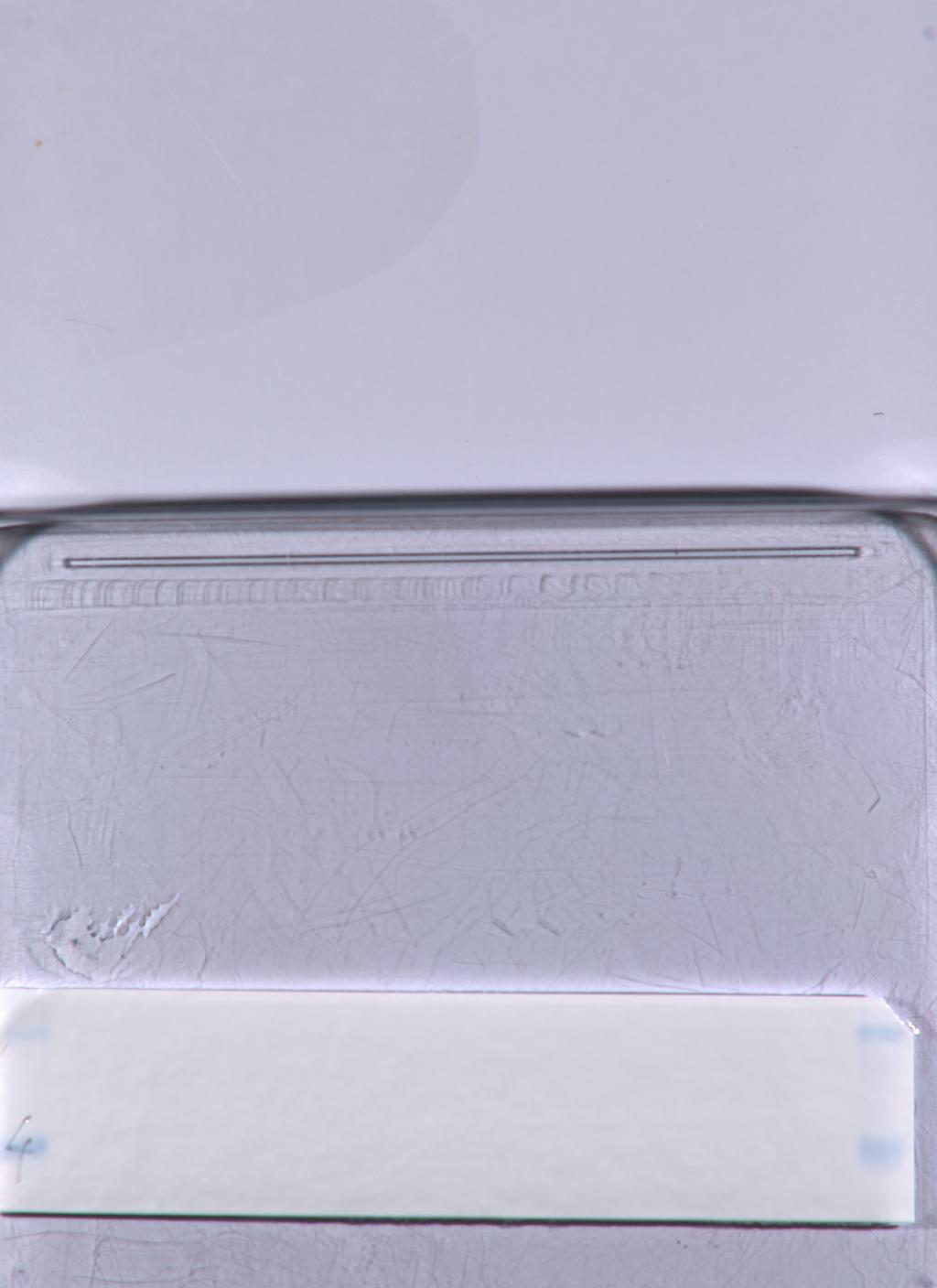

Supplement: Supplementary file 9 — Source data Fig. 4 [file 44321_2024_60_MOESM9_ESM.zip › Figure 4/4C/YAPC/Western H3 0.3/4 3rd H3 0.3 _Ch-Marker.jpg]

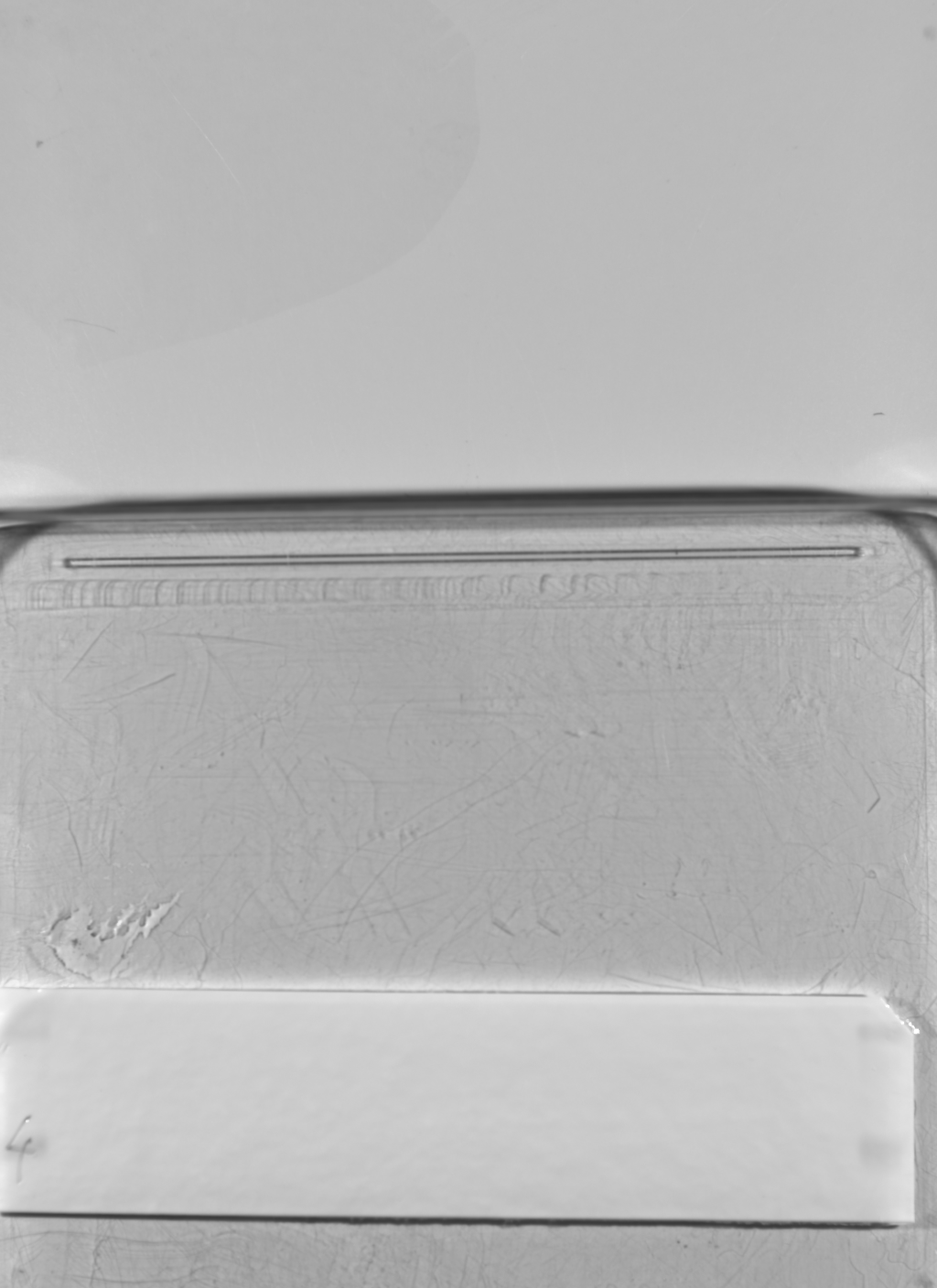

Supplement: Supplementary file 9 — Source data Fig. 4 [file 44321_2024_60_MOESM9_ESM.zip › Figure 4/4C/YAPC/Western H3 0.3/4 3rd H3 0.3 _Ch-Marker.tif]

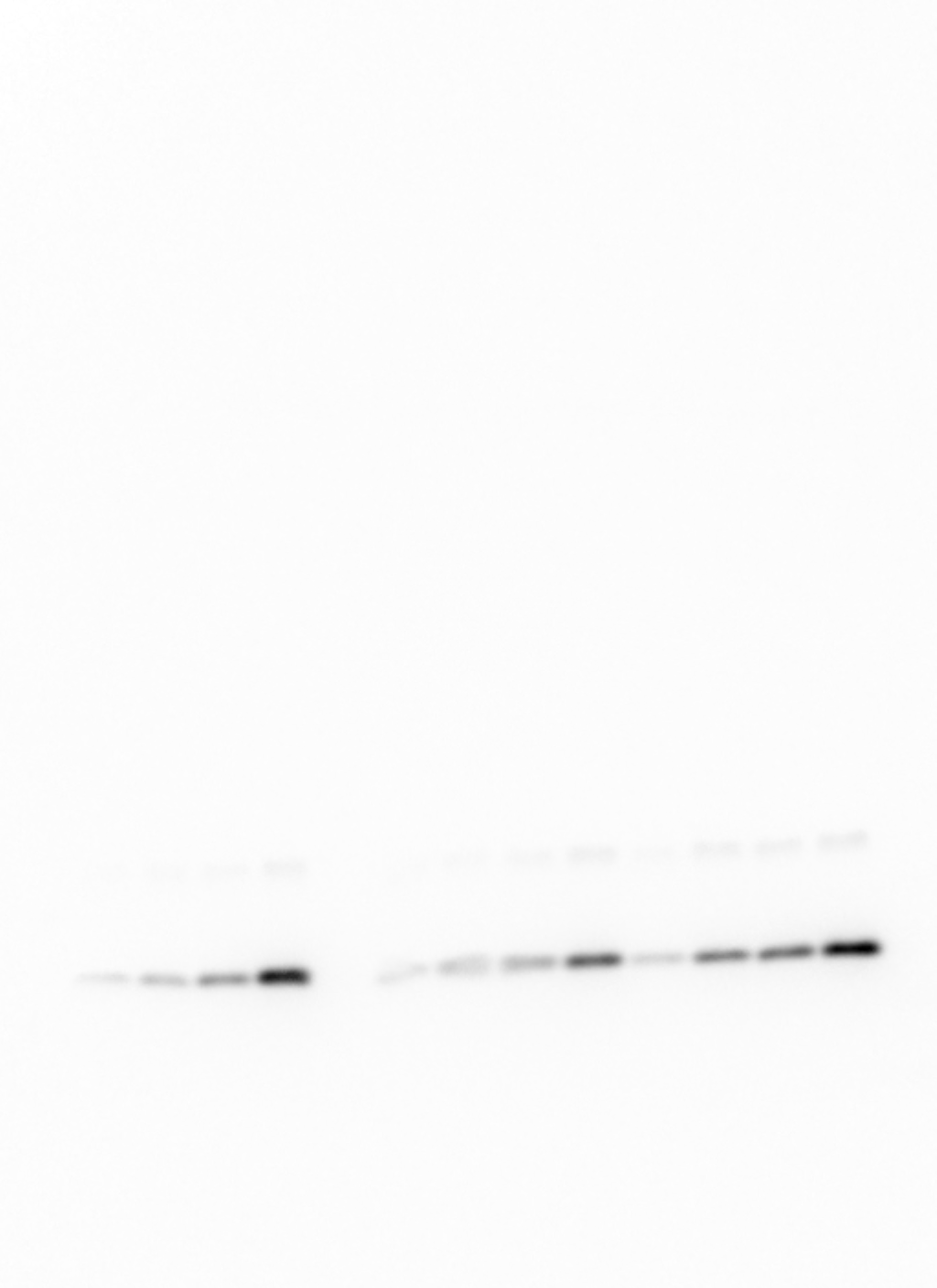

Supplement: Supplementary file 9 — Source data Fig. 4 [file 44321_2024_60_MOESM9_ESM.zip › Figure 4/4C/YAPC/Western rH2A 0.6/5 3rd rH2A 0.6 _Ch.tif]

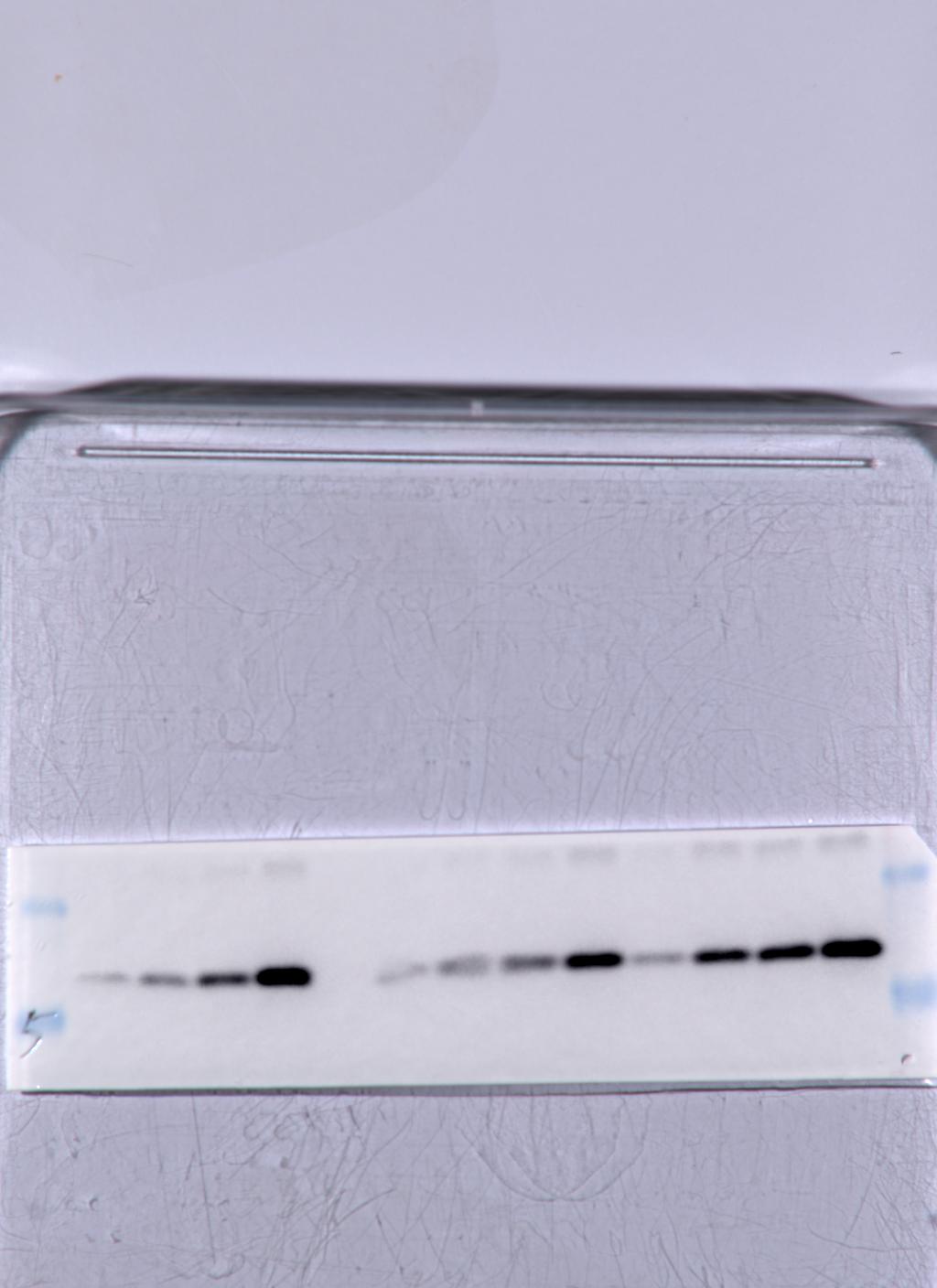

Supplement: Supplementary file 9 — Source data Fig. 4 [file 44321_2024_60_MOESM9_ESM.zip › Figure 4/4C/YAPC/Western rH2A 0.6/5 3rd rH2A 0.6 _Ch+Marker.jpg]

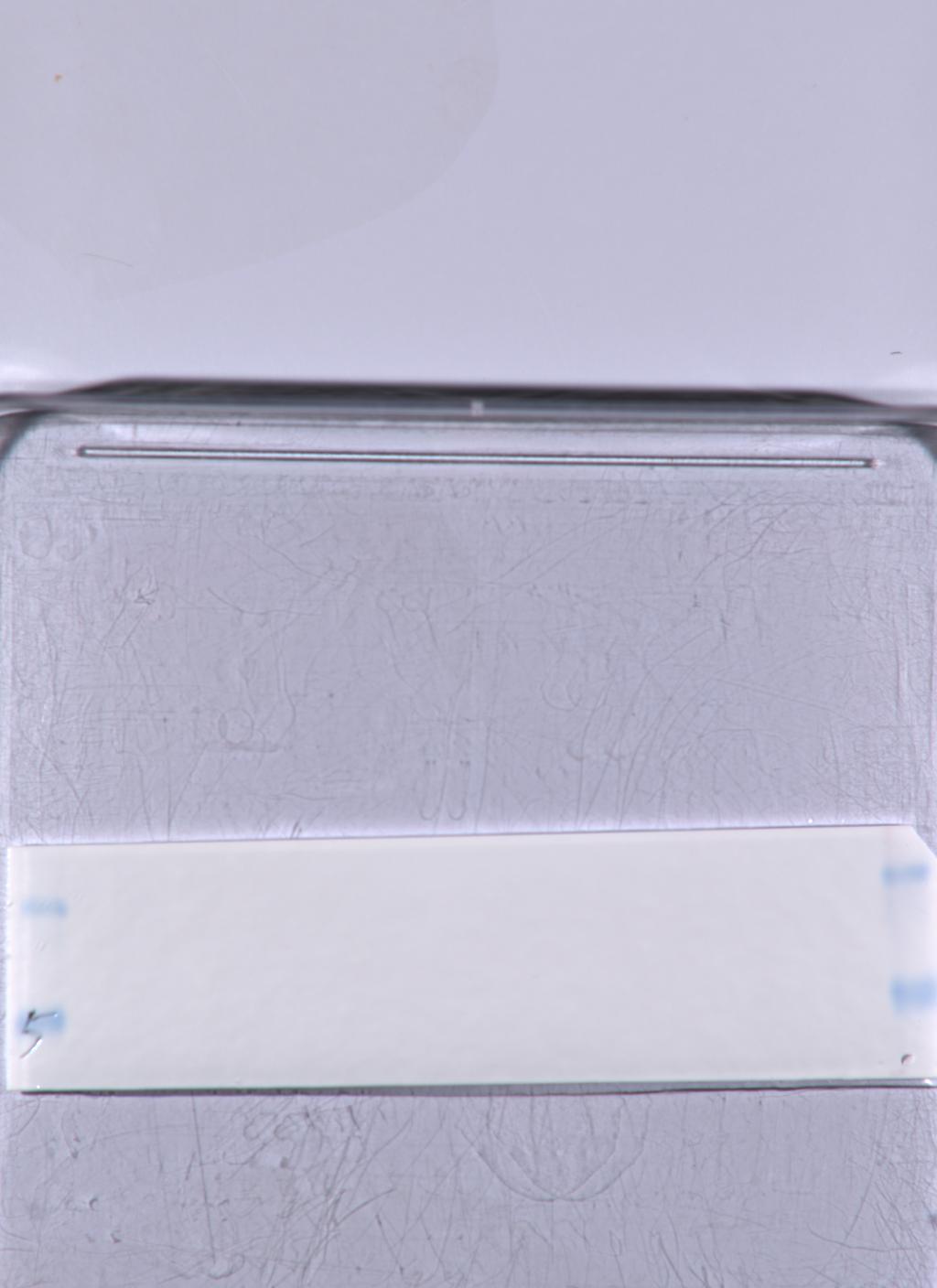

Supplement: Supplementary file 9 — Source data Fig. 4 [file 44321_2024_60_MOESM9_ESM.zip › Figure 4/4C/YAPC/Western rH2A 0.6/5 3rd rH2A 0.6 _Ch-Marker.jpg]

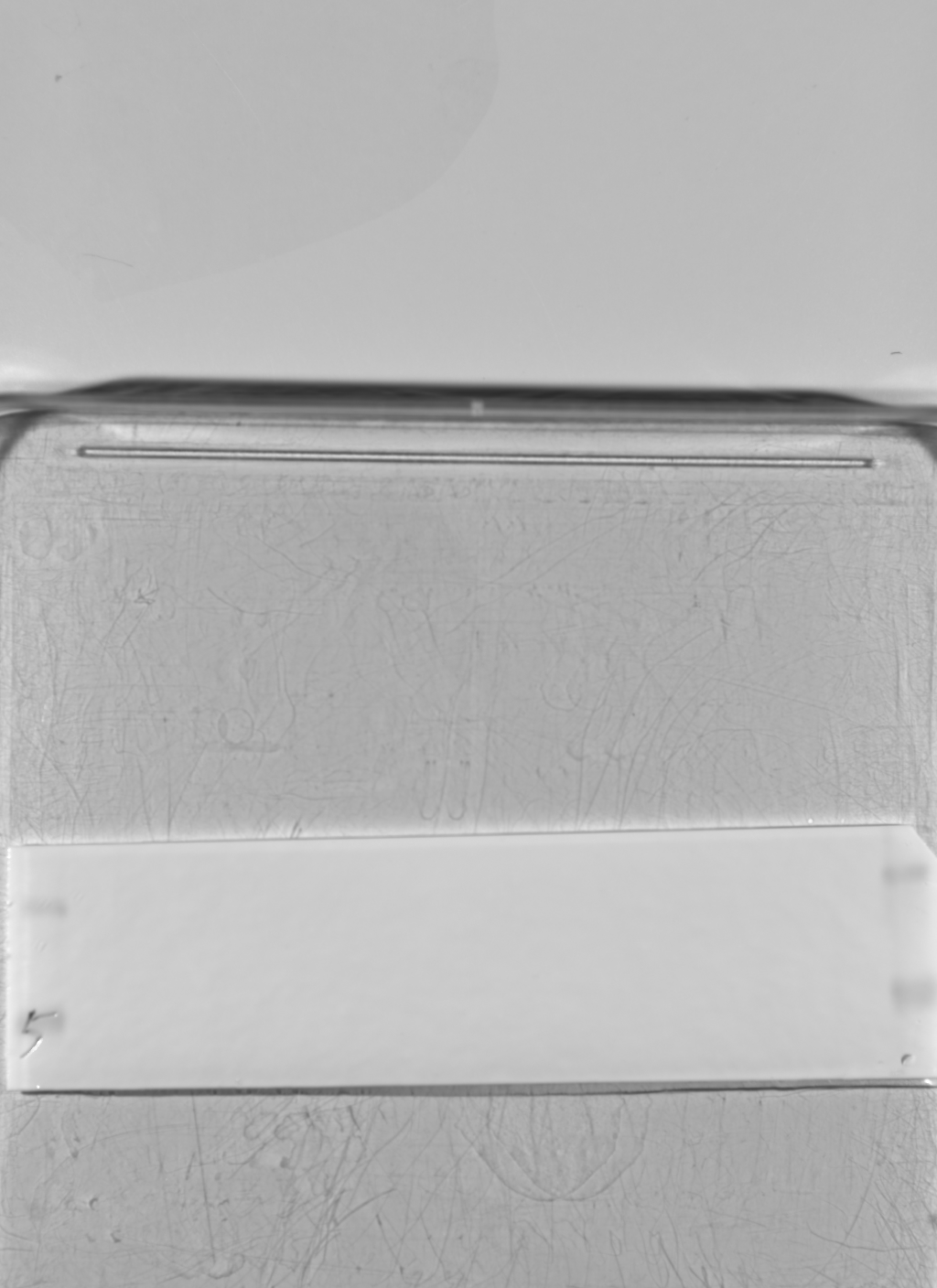

Supplement: Supplementary file 9 — Source data Fig. 4 [file 44321_2024_60_MOESM9_ESM.zip › Figure 4/4C/YAPC/Western rH2A 0.6/5 3rd rH2A 0.6 _Ch-Marker.tif]

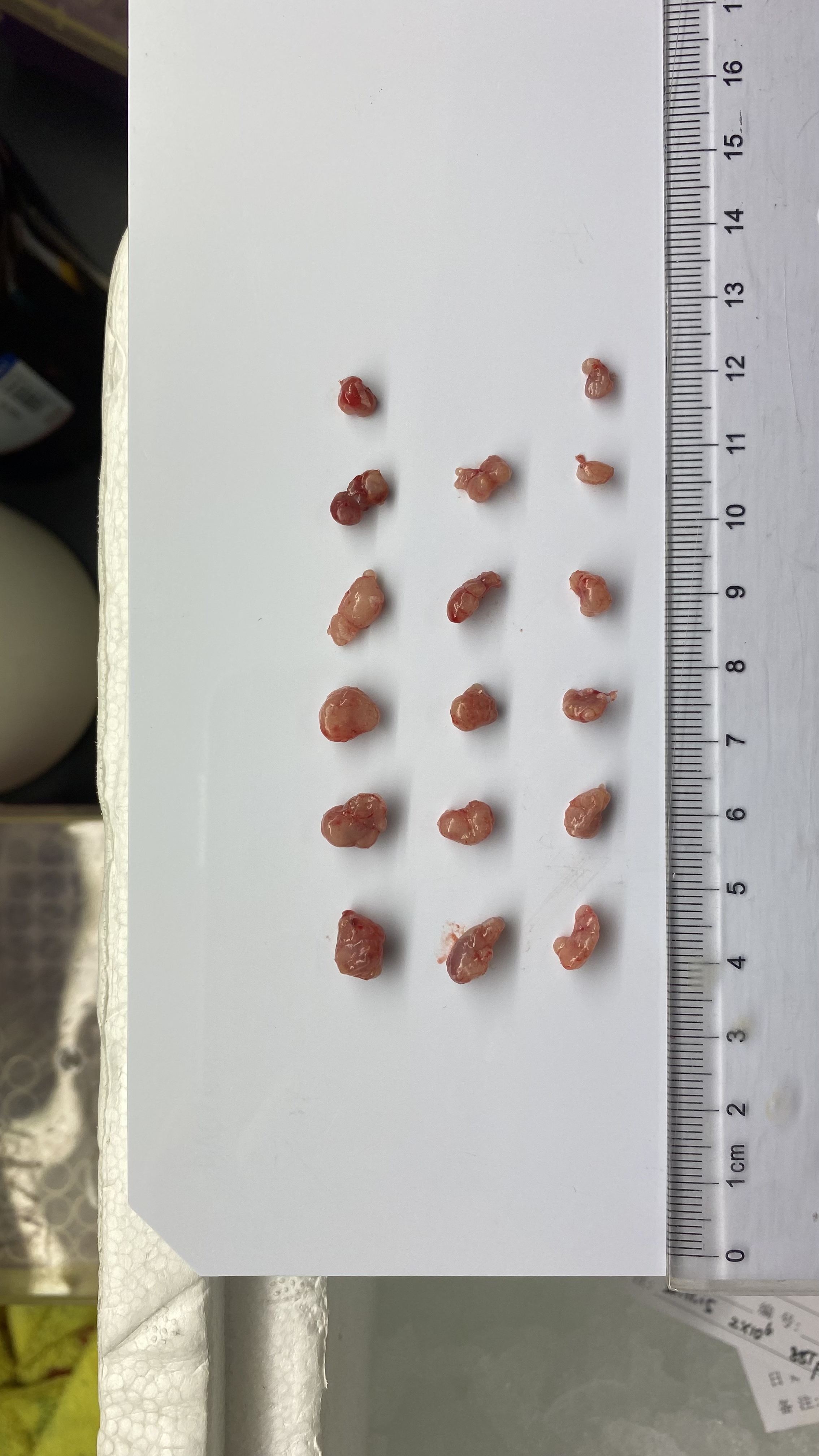

Supplement: Supplementary file 10 — Source data Fig. 5 [file 44321_2024_60_MOESM10_ESM.zip › Figure 5/5B/5B.jpg]

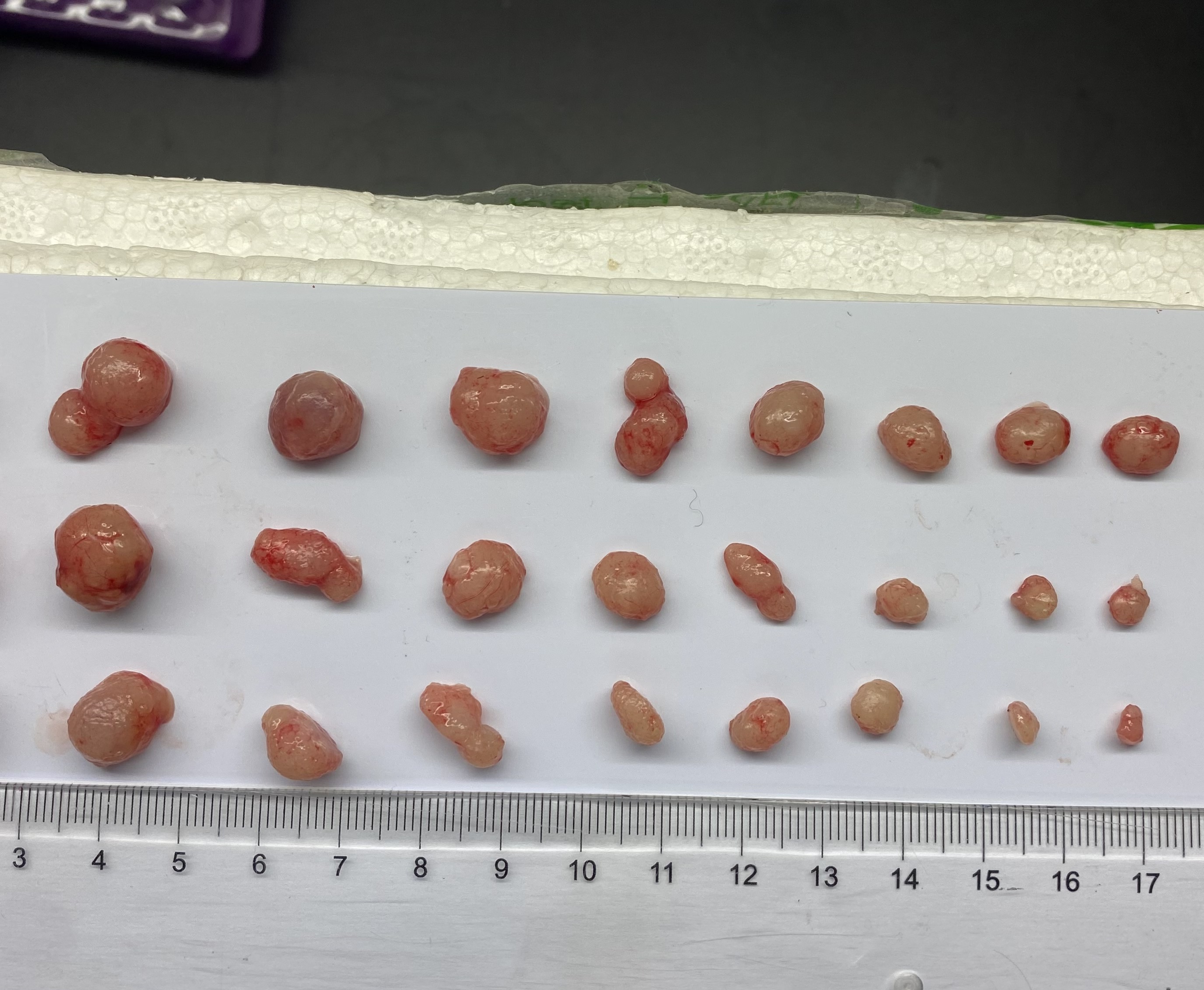

Supplement: Supplementary file 10 — Source data Fig. 5 [file 44321_2024_60_MOESM10_ESM.zip › Figure 5/5E/5E.jpg]

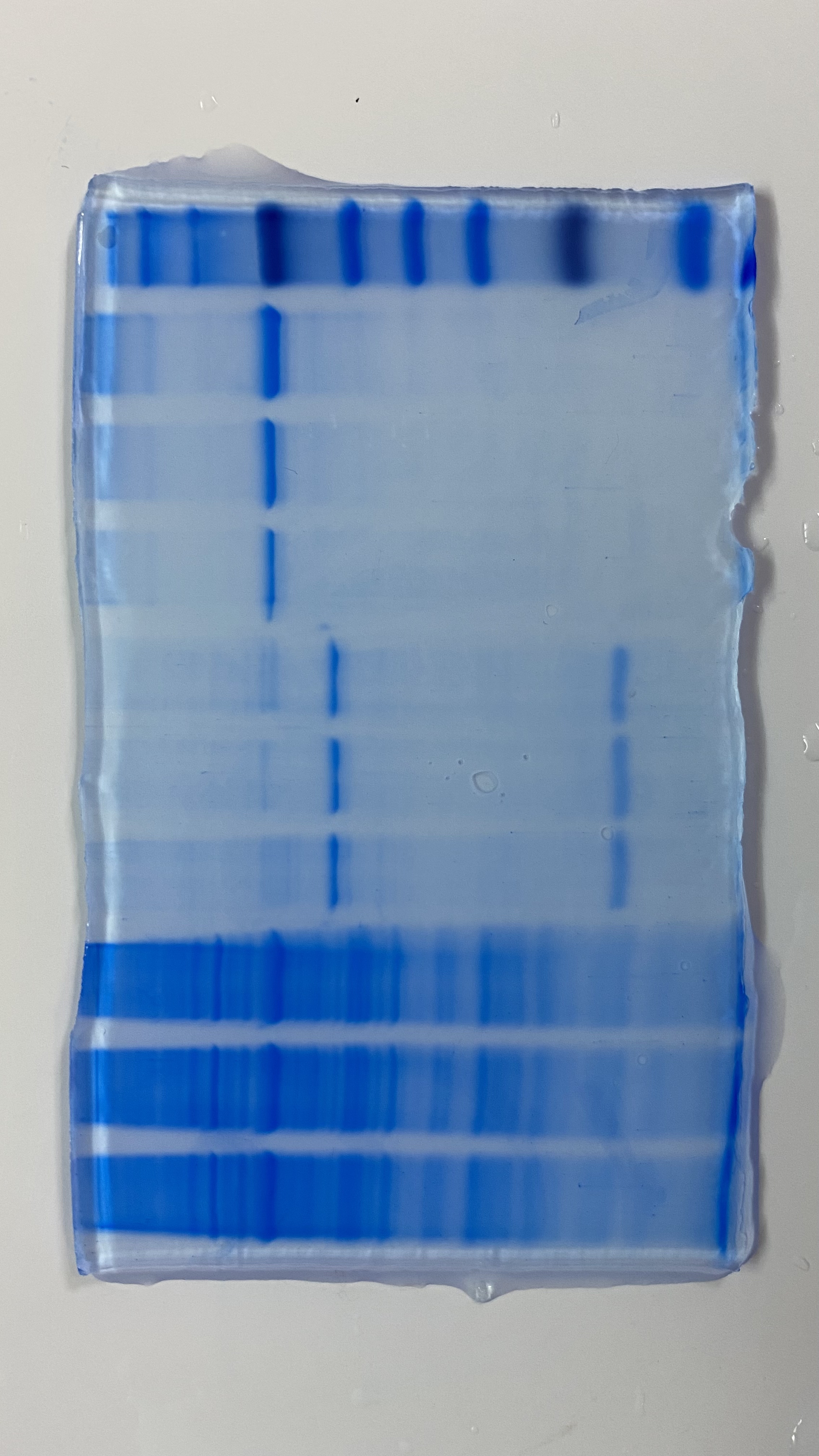

Supplement: Supplementary file 11 — Source data Fig. 6 [file 44321_2024_60_MOESM11_ESM.zip › Figure 6/6A/6A.jpg]

## Slide 1
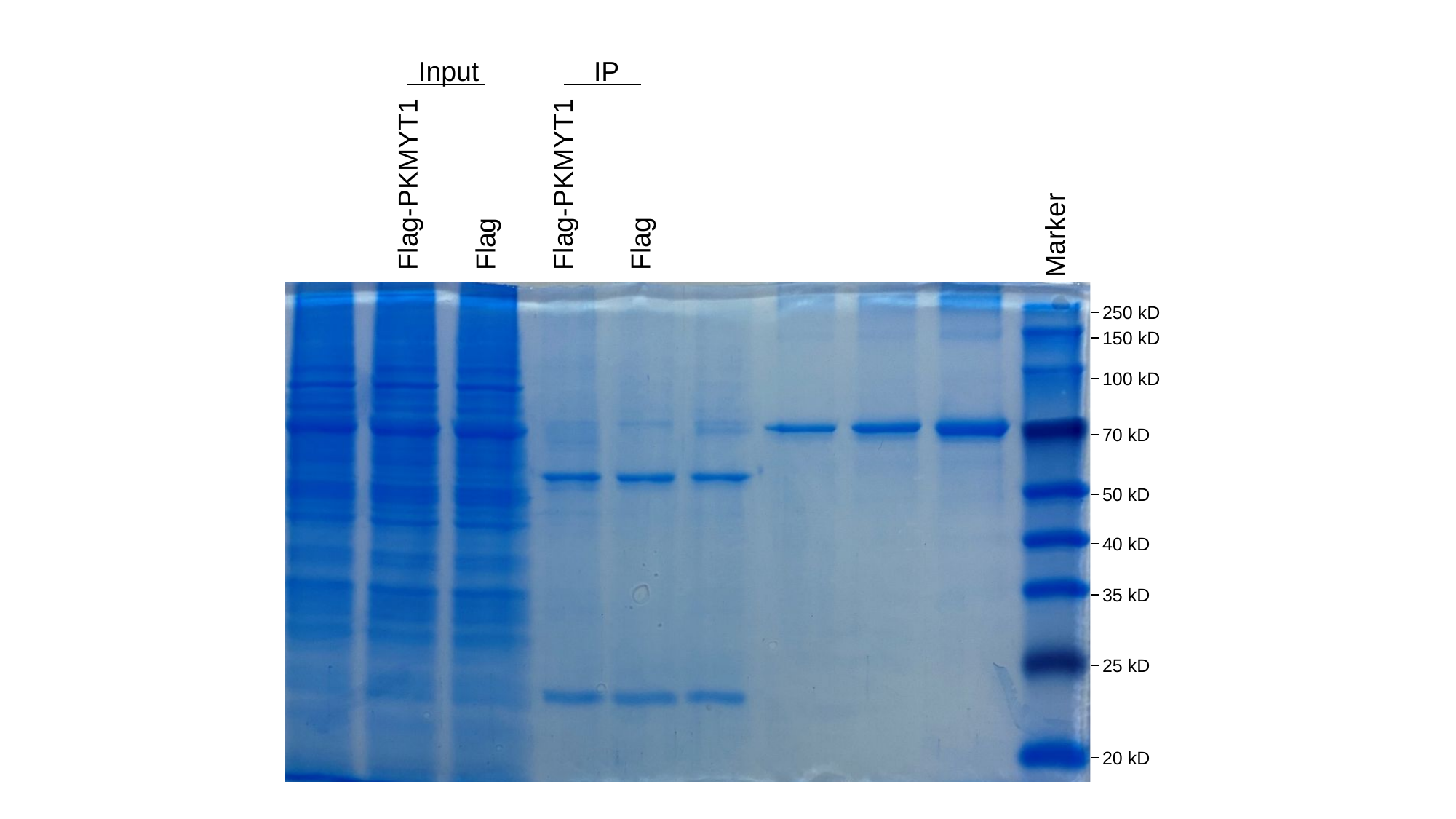

Input
IP
Flag-PKMYT1
Flag-PKMYT1
Marker
Flag
Flag
250 kD
150 kD
100 kD
70 kD
50 kD
40 kD
35 kD
25 kD
20 kD

## Slide 2
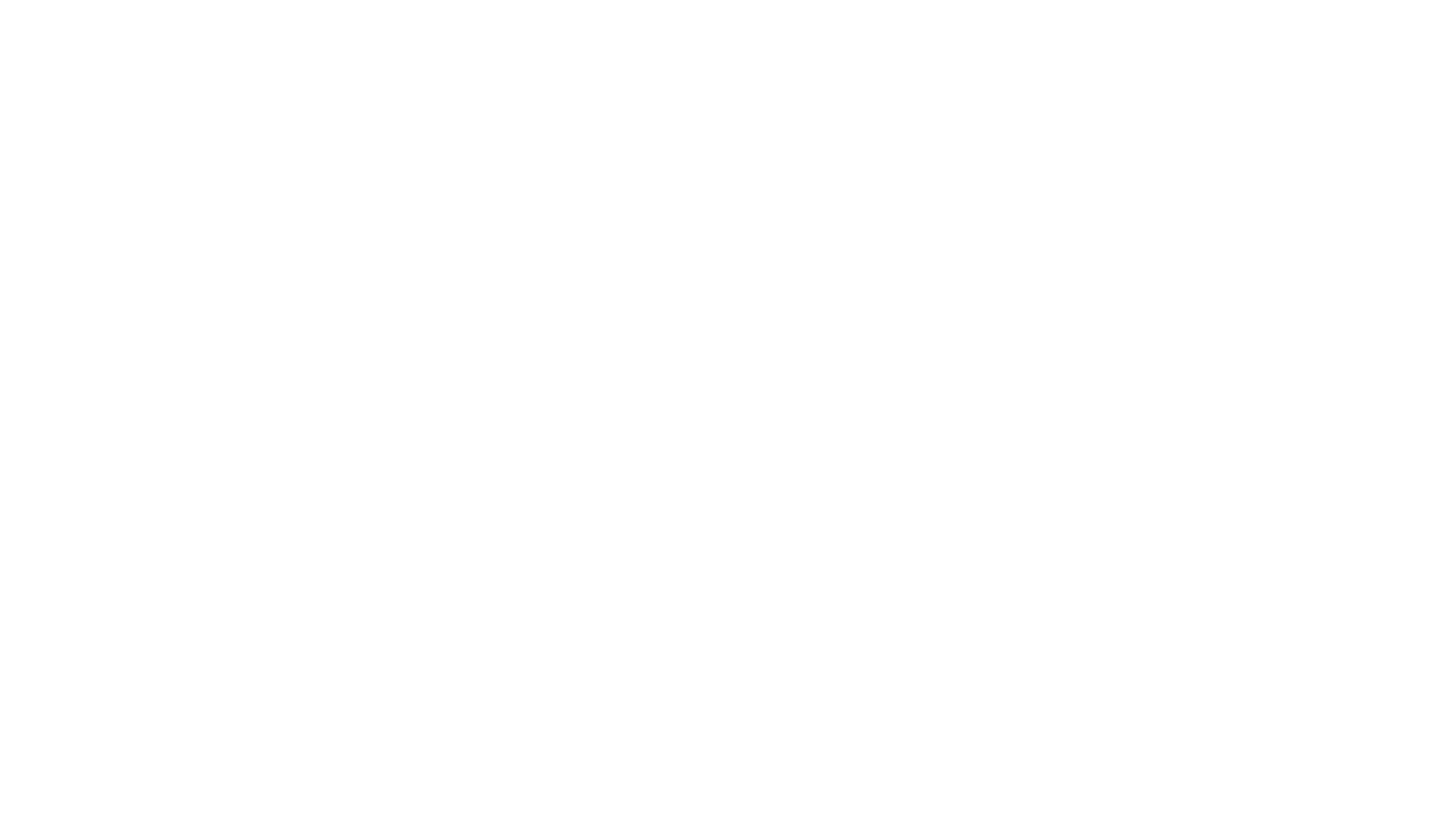

Supplement: Supplementary file 11 — Source data Fig. 6 [file 44321_2024_60_MOESM11_ESM.zip › Figure 6/6A/6A.pptx]

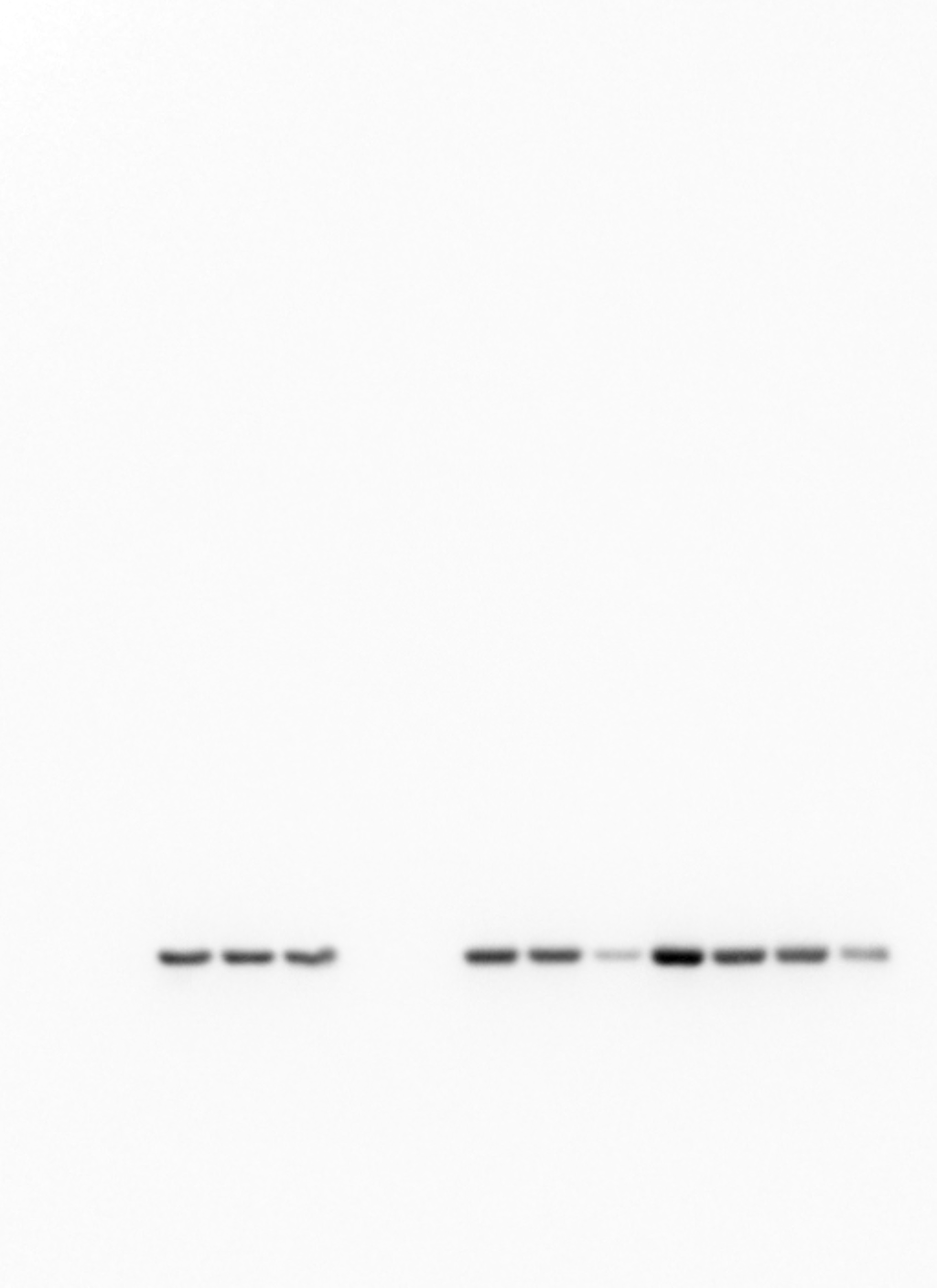

Supplement: Supplementary file 11 — Source data Fig. 6 [file 44321_2024_60_MOESM11_ESM.zip › Figure 6/6C/88T/Western GAPDH 0.8/3 GAP 0.8 _Ch.tif]

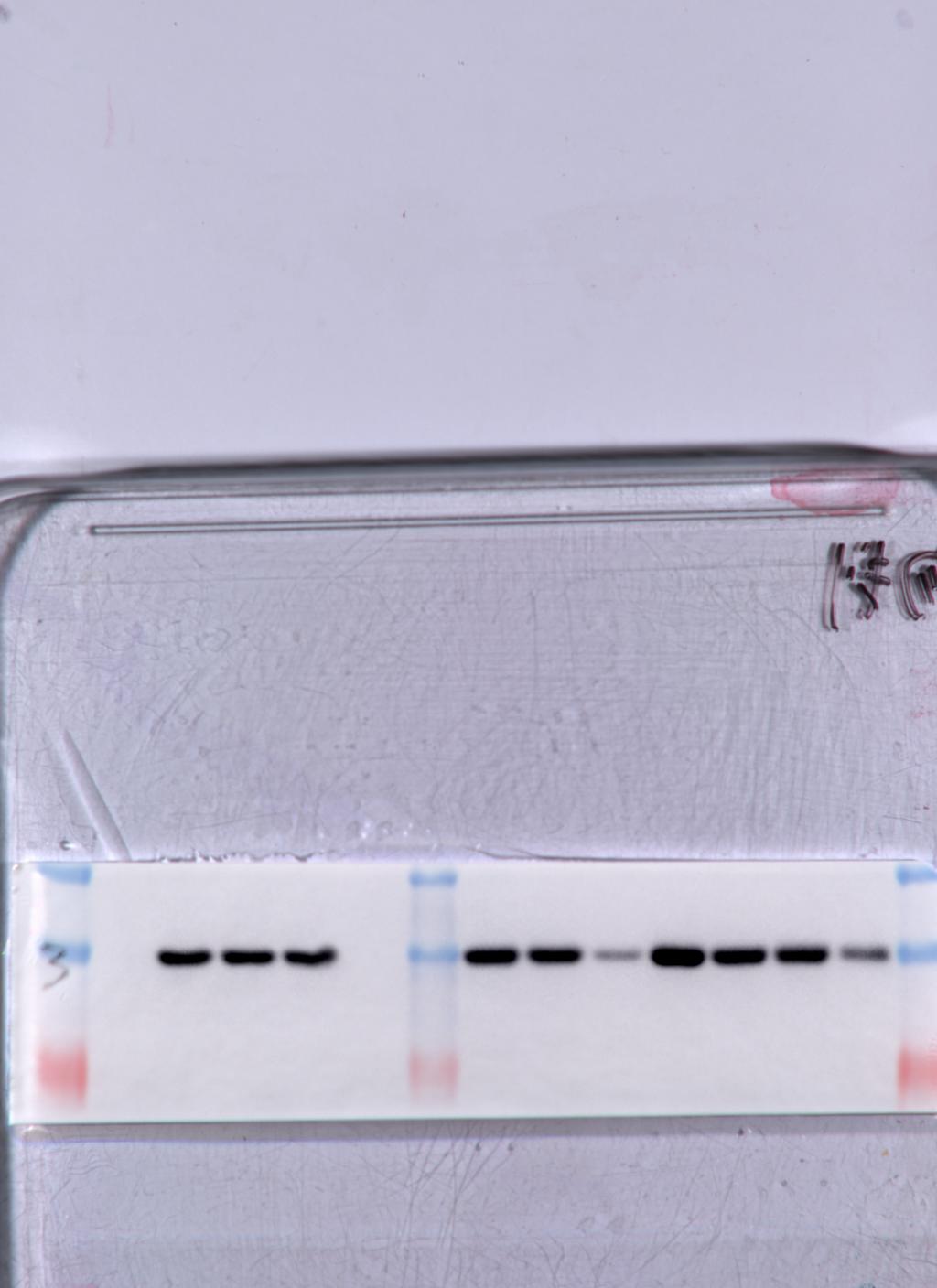

Supplement: Supplementary file 11 — Source data Fig. 6 [file 44321_2024_60_MOESM11_ESM.zip › Figure 6/6C/88T/Western GAPDH 0.8/3 GAP 0.8 _Ch+Marker.jpg]

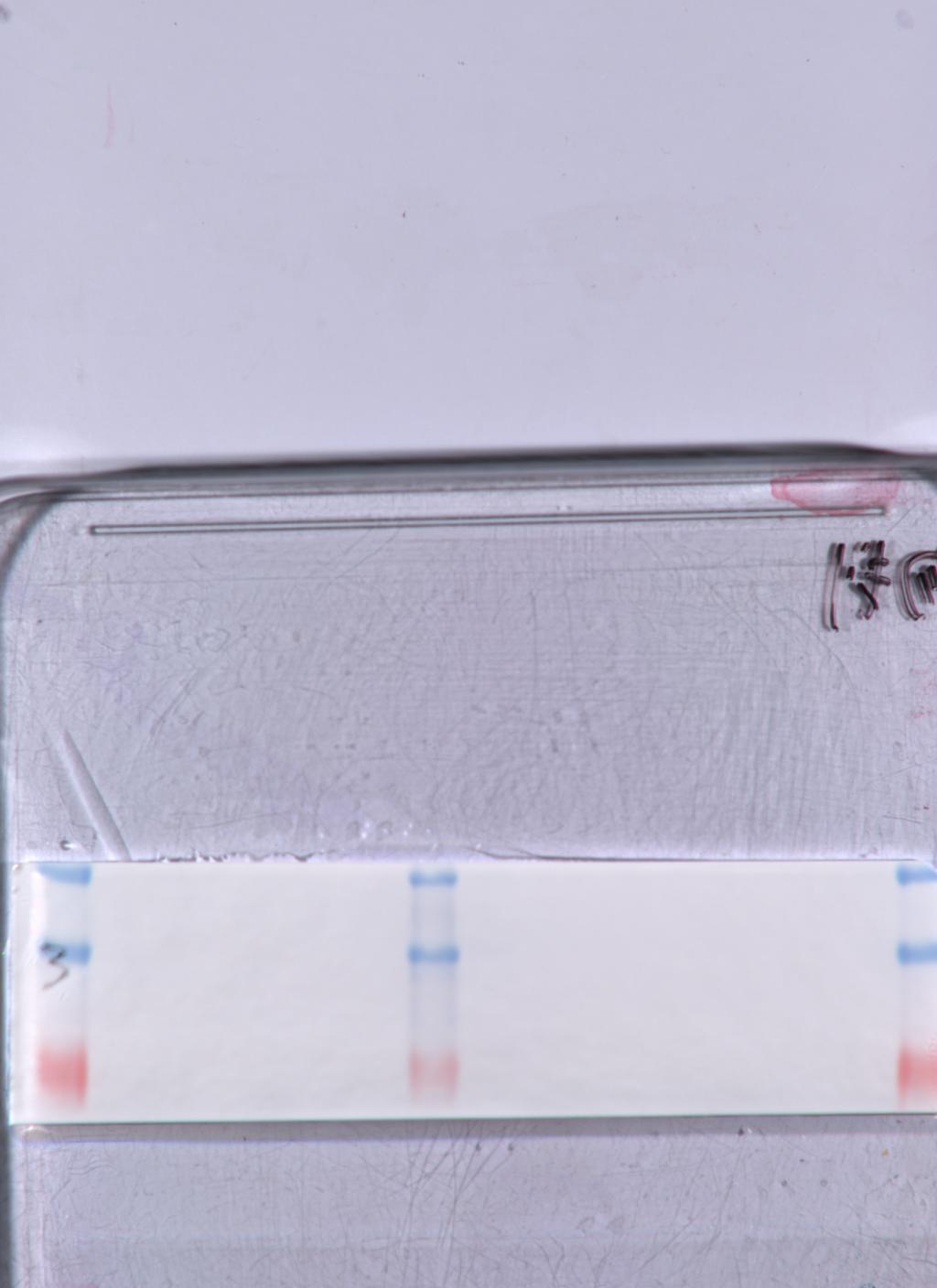

Supplement: Supplementary file 11 — Source data Fig. 6 [file 44321_2024_60_MOESM11_ESM.zip › Figure 6/6C/88T/Western GAPDH 0.8/3 GAP 0.8 _Ch-Marker.jpg]

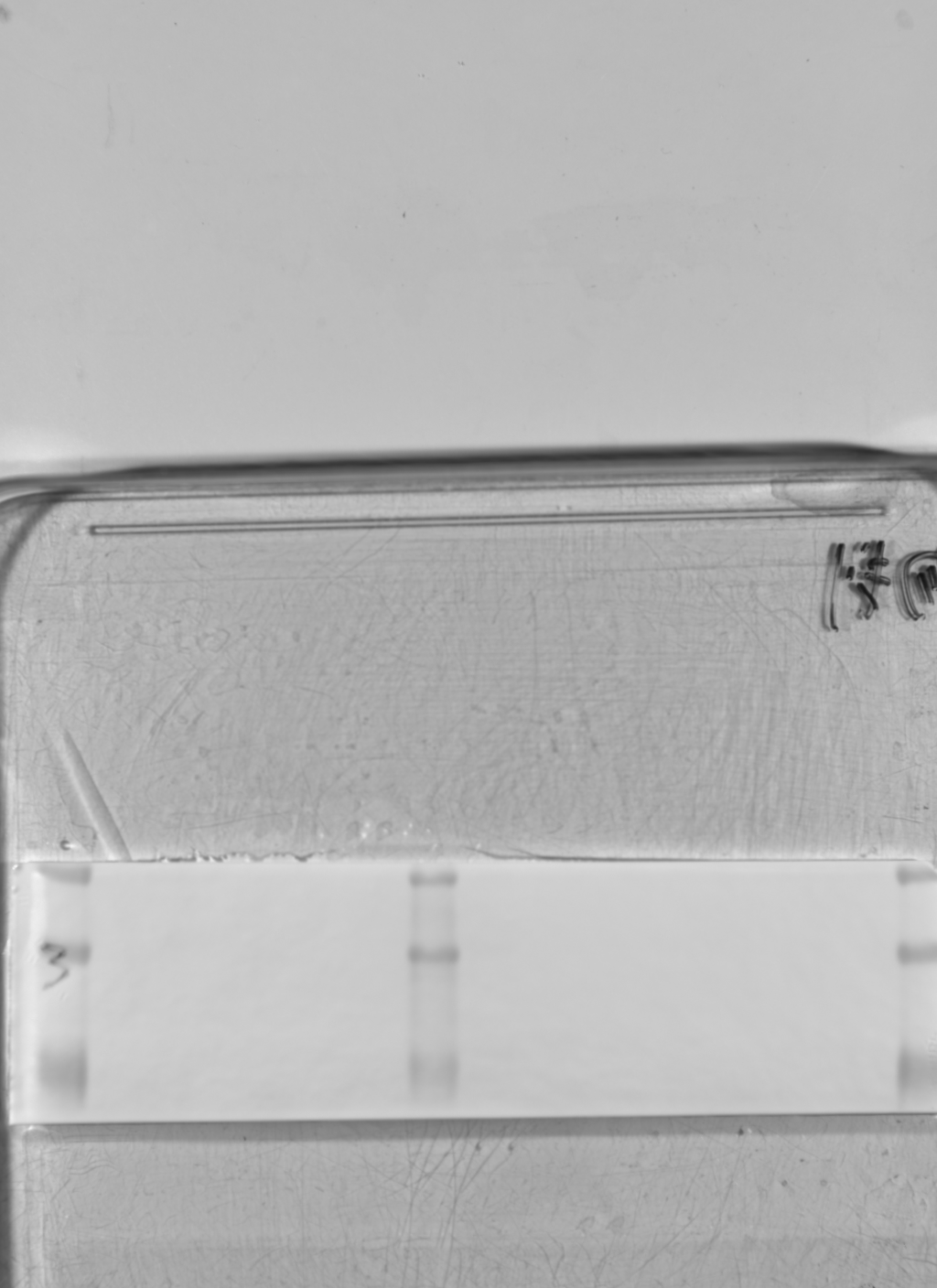

Supplement: Supplementary file 11 — Source data Fig. 6 [file 44321_2024_60_MOESM11_ESM.zip › Figure 6/6C/88T/Western GAPDH 0.8/3 GAP 0.8 _Ch-Marker.tif]

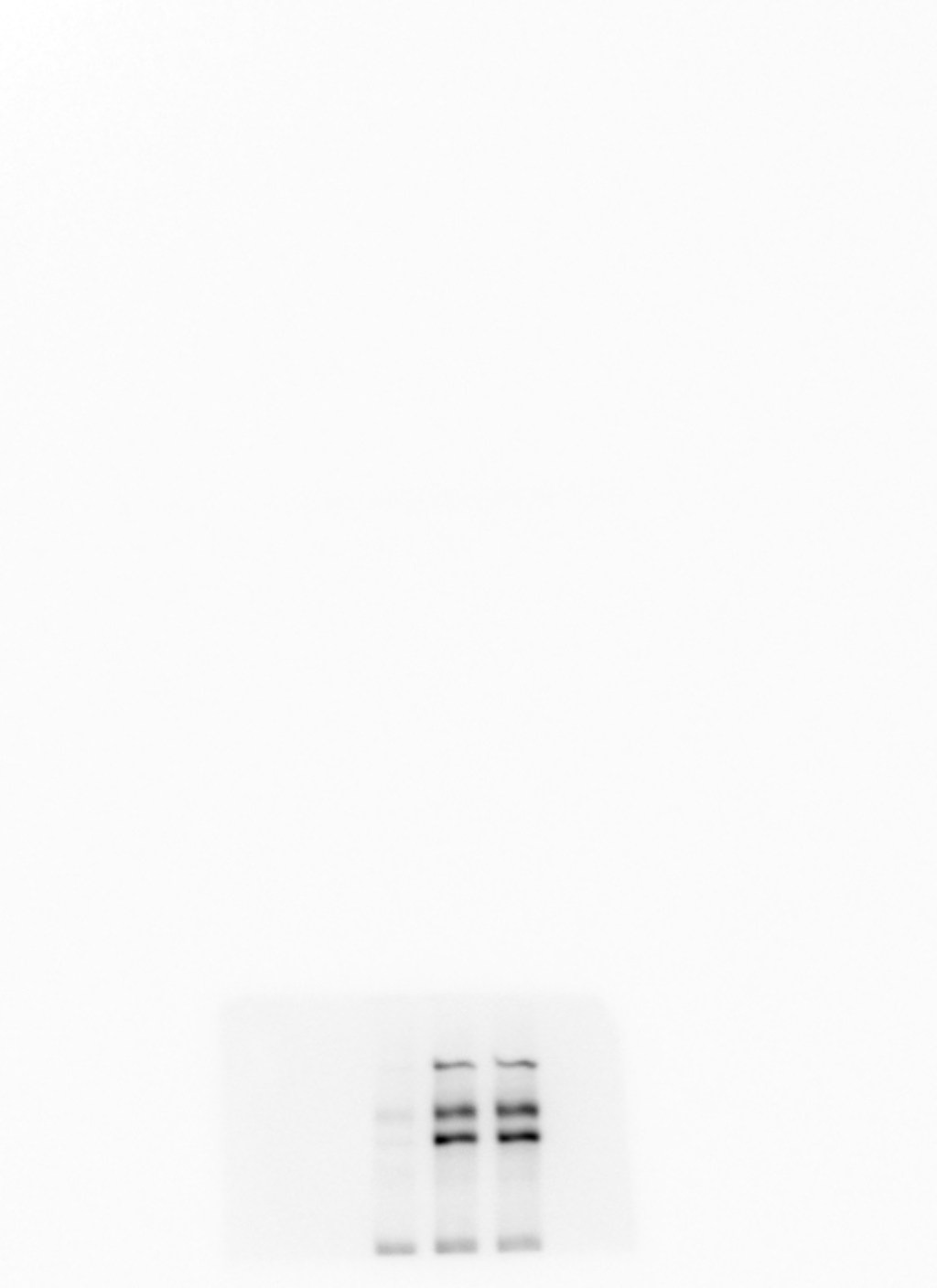

Supplement: Supplementary file 11 — Source data Fig. 6 [file 44321_2024_60_MOESM11_ESM.zip › Figure 6/6C/88T/Western phoPRKDC 1/8-1 1st phoPRK 1Q _Ch.tif]

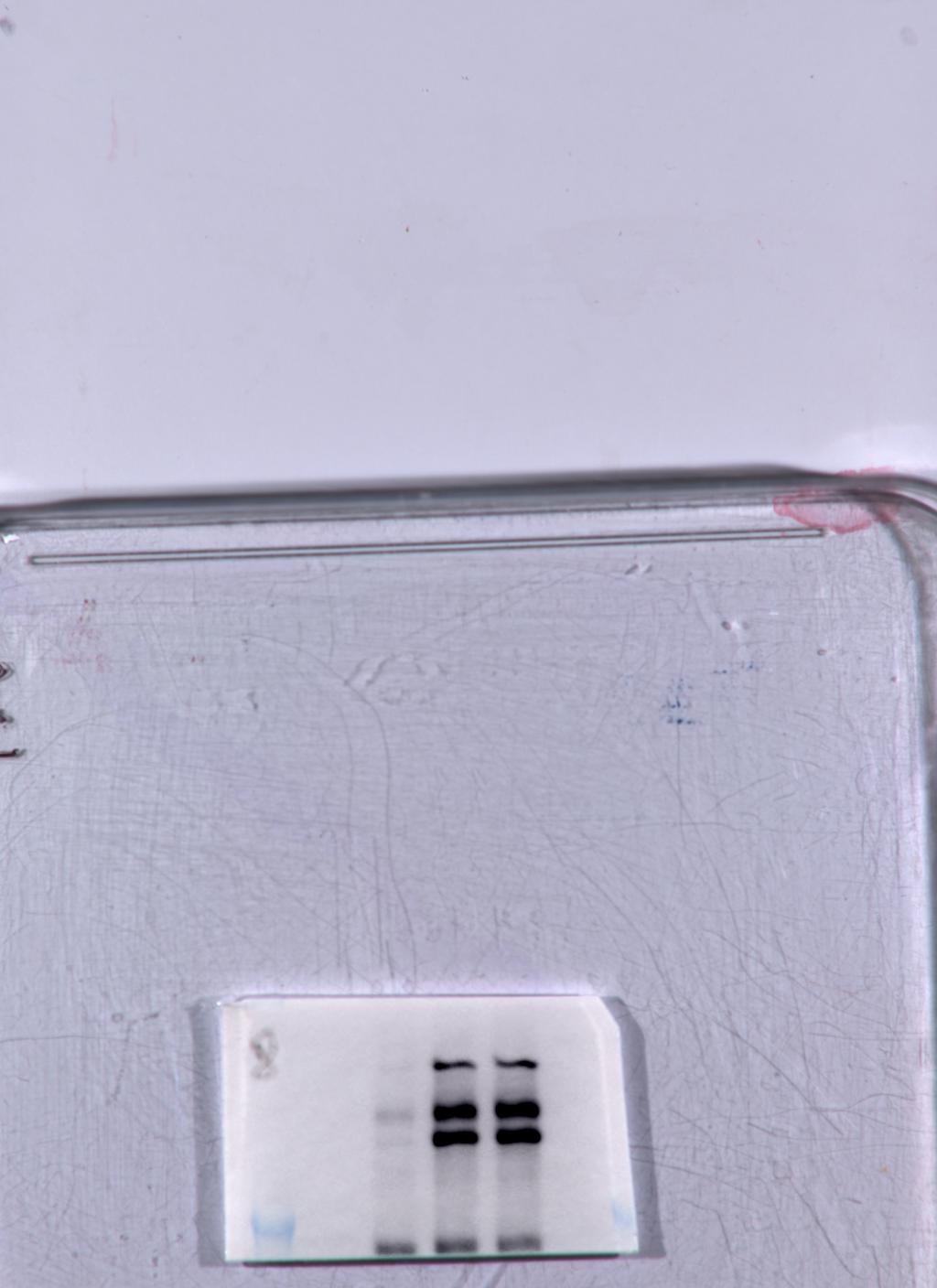

Supplement: Supplementary file 11 — Source data Fig. 6 [file 44321_2024_60_MOESM11_ESM.zip › Figure 6/6C/88T/Western phoPRKDC 1/8-1 1st phoPRK 1Q _Ch+Marker.jpg]

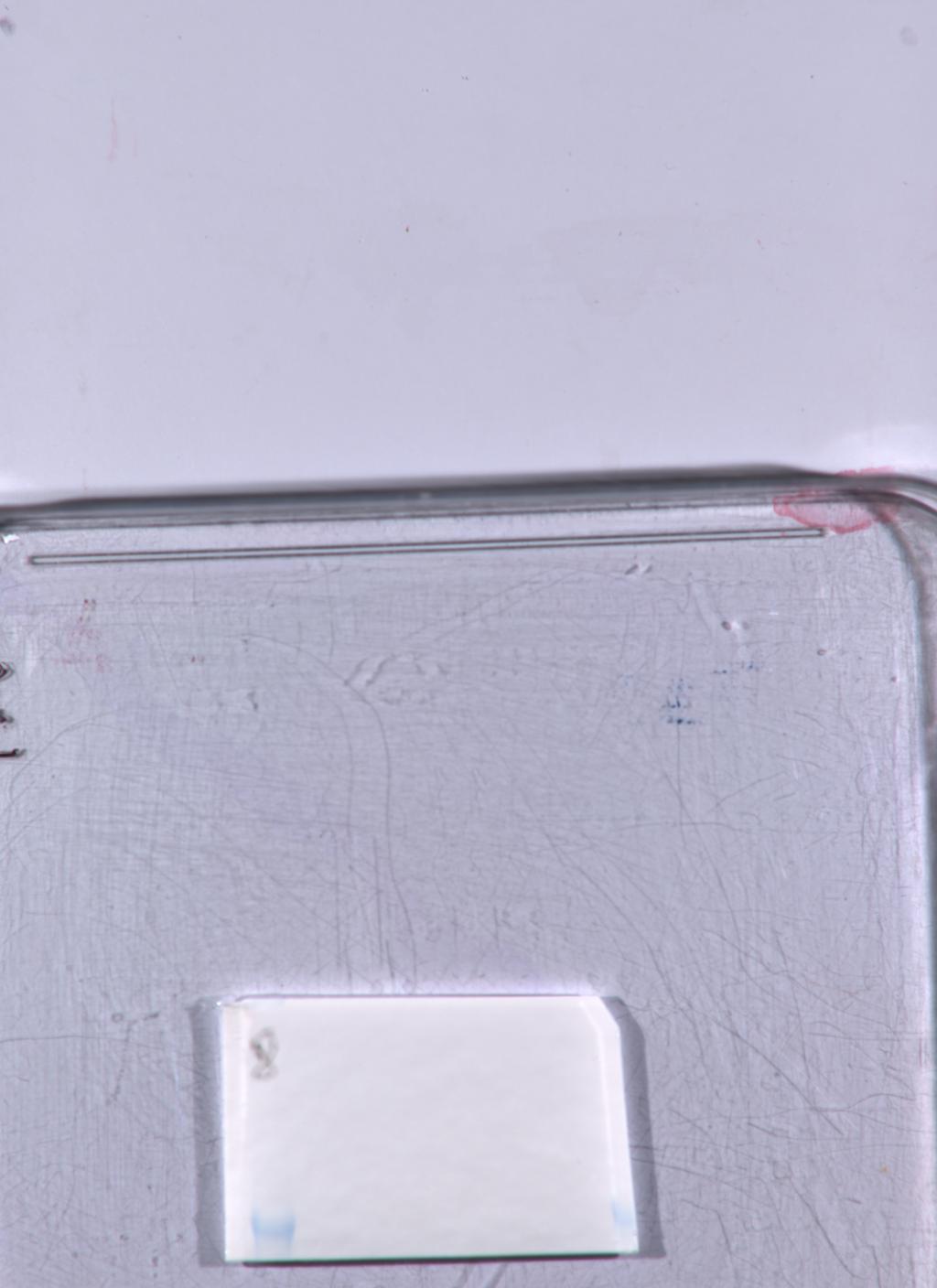

Supplement: Supplementary file 11 — Source data Fig. 6 [file 44321_2024_60_MOESM11_ESM.zip › Figure 6/6C/88T/Western phoPRKDC 1/8-1 1st phoPRK 1Q _Ch-Marker.jpg]
