# Supplementary material for: Genome-wide CRISPR screens identify PKMYT1 as a therapeutic target in pancreatic ductal adenocarcinoma
Source: EMBO Mol Med. 2024 Apr 3;16(5):5. doi: 10.1038/s44321-024-00060-y (PMC11099189; doi:10.1038/s44321-024-00060-y)
Supplement: Supplementary file 11 — Source data Fig. 6 [file 44321_2024_60_MOESM11_ESM.zip › Figure 6/6C/YAPC/6C YAPC.pptx]

## Slide 1
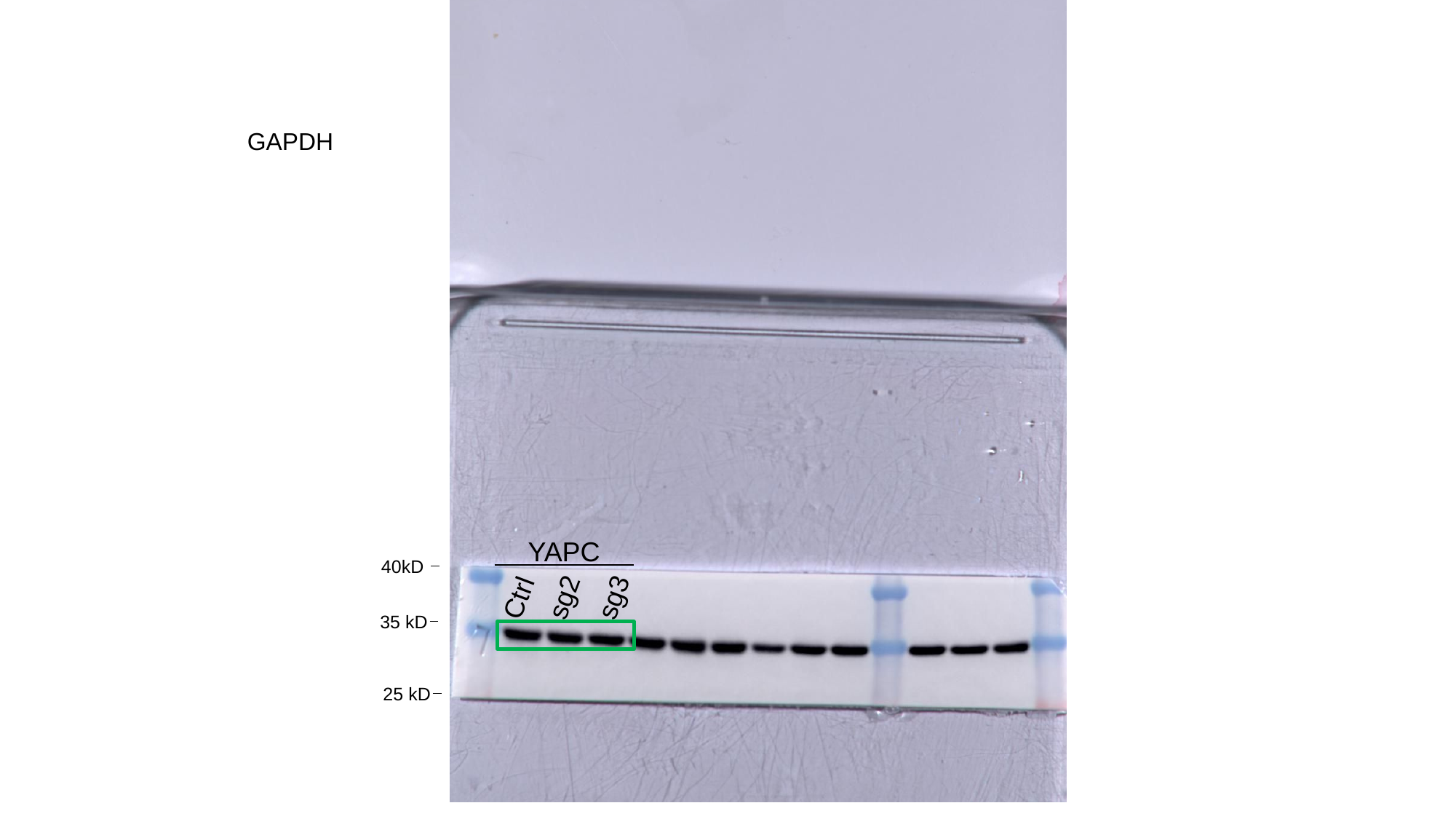

GAPDH
YAPC
Ctrl
sg3
sg2
40kD
35 kD
25 kD

## Slide 2
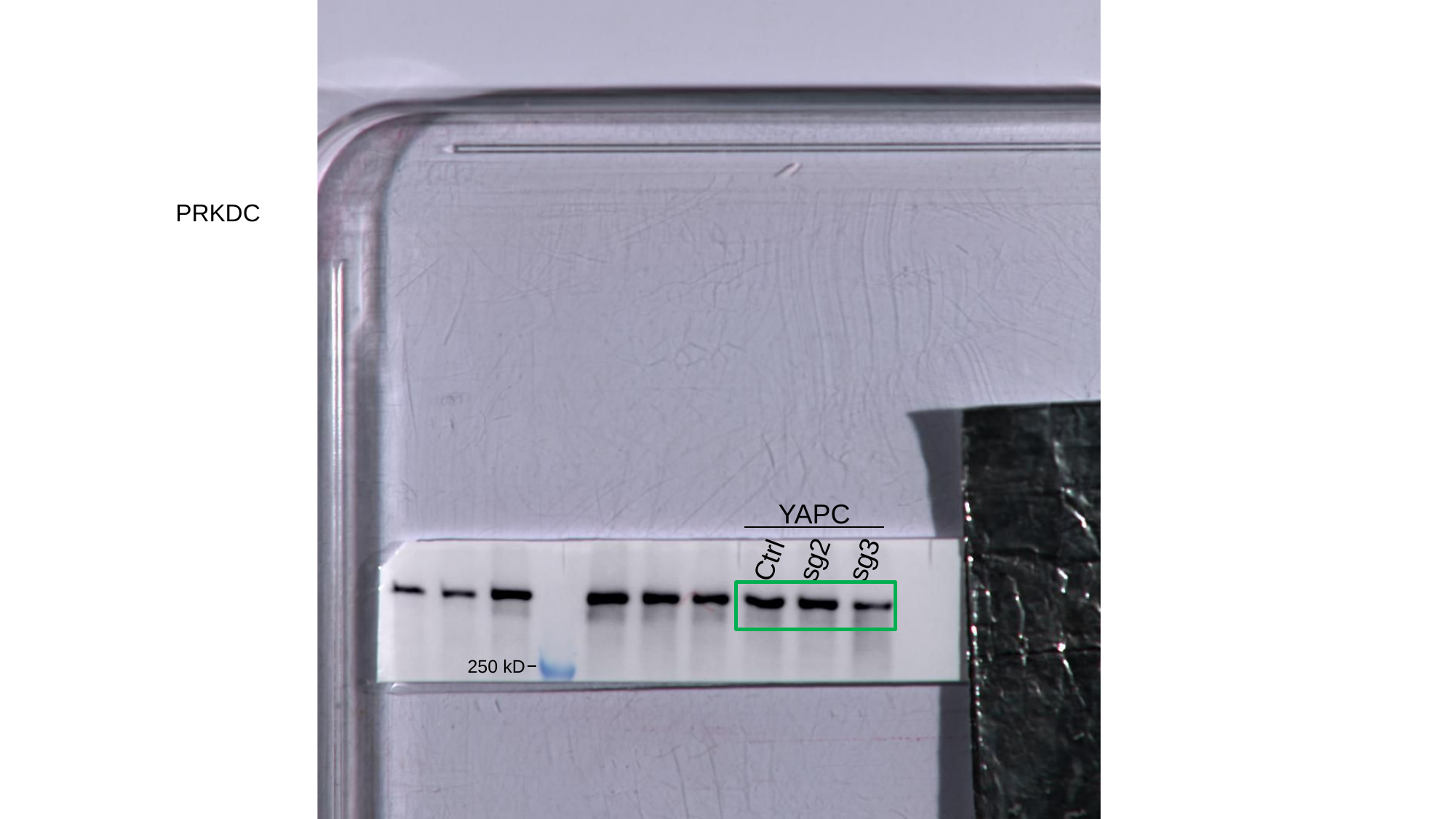

PRKDC
YAPC
Ctrl
sg3
sg2
250 kD

## Slide 3
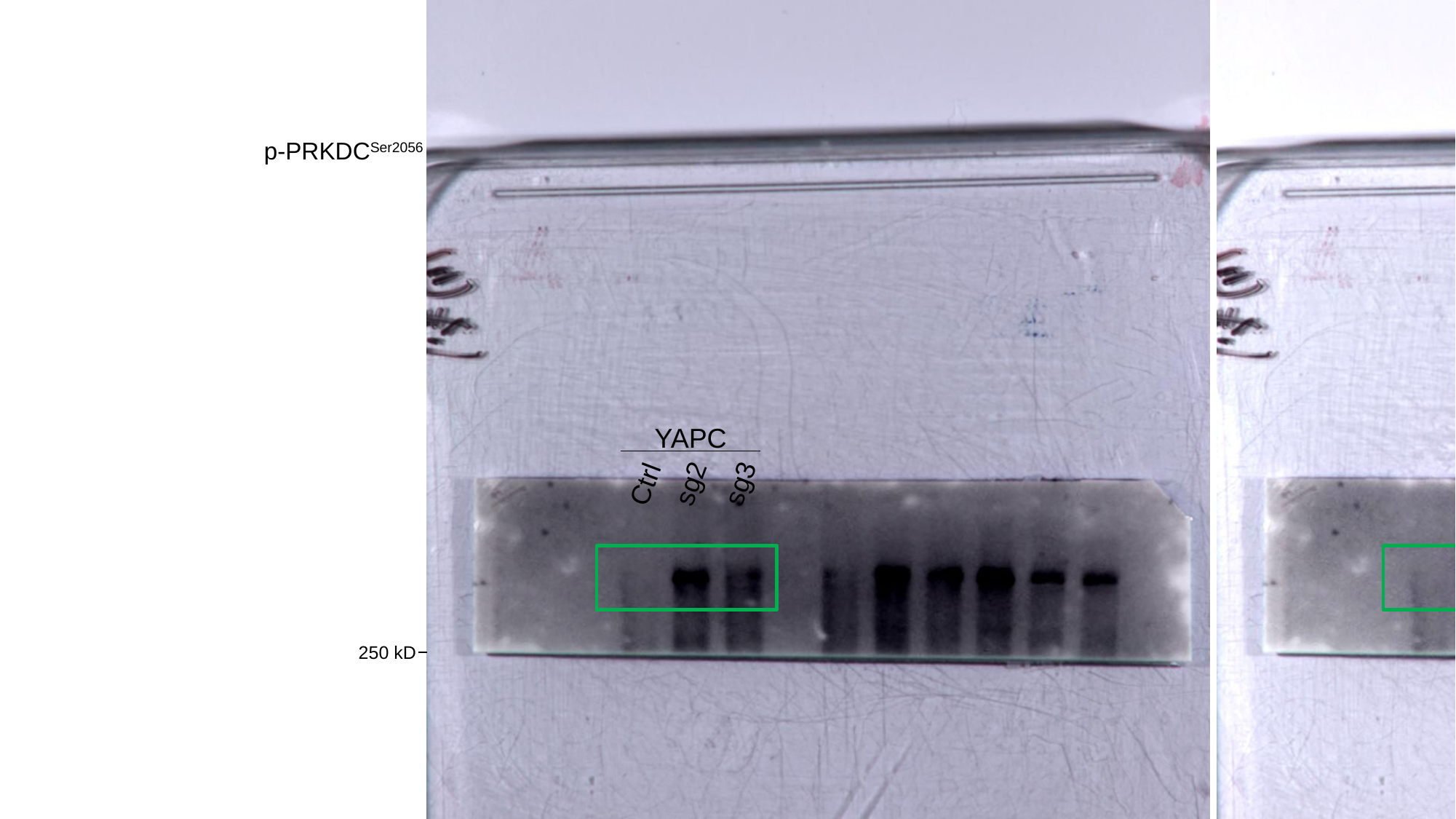

p-PRKDCSer2056
YAPC
Ctrl
sg3
sg2
250 kD

## Slide 4
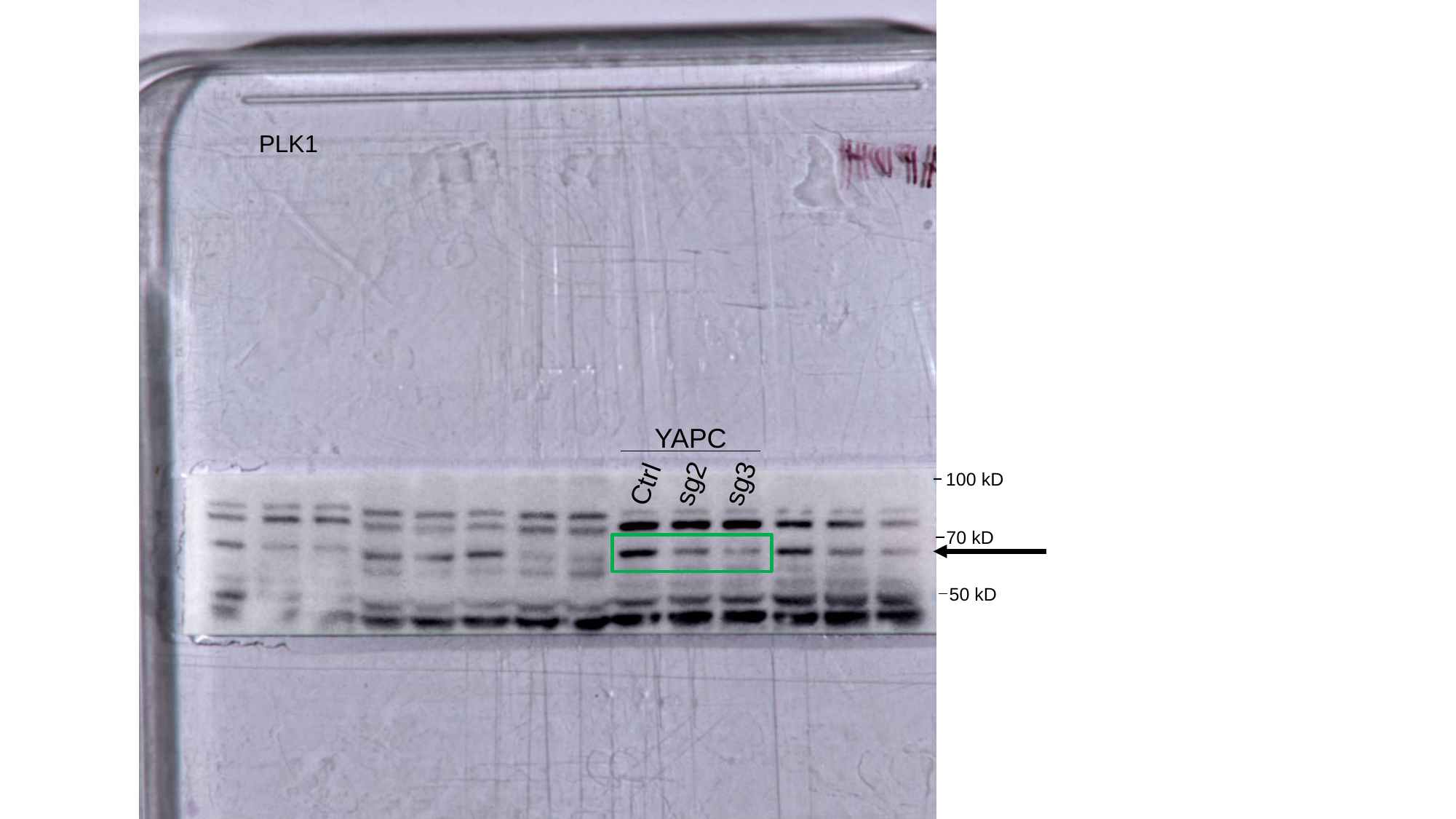

PLK1
YAPC
Ctrl
sg3
sg2
100 kD
70 kD
50 kD
