# Supplementary material for: Genome-wide CRISPR screens identify PKMYT1 as a therapeutic target in pancreatic ductal adenocarcinoma
Source: EMBO Mol Med. 2024 Apr 3;16(5):5. doi: 10.1038/s44321-024-00060-y (PMC11099189; doi:10.1038/s44321-024-00060-y)
Supplement: Supplementary file 11 — Source data Fig. 6 [file 44321_2024_60_MOESM11_ESM.zip › Figure 6/6D/88T YAPC/6D 88T YAPC.pptx]

## Slide 1
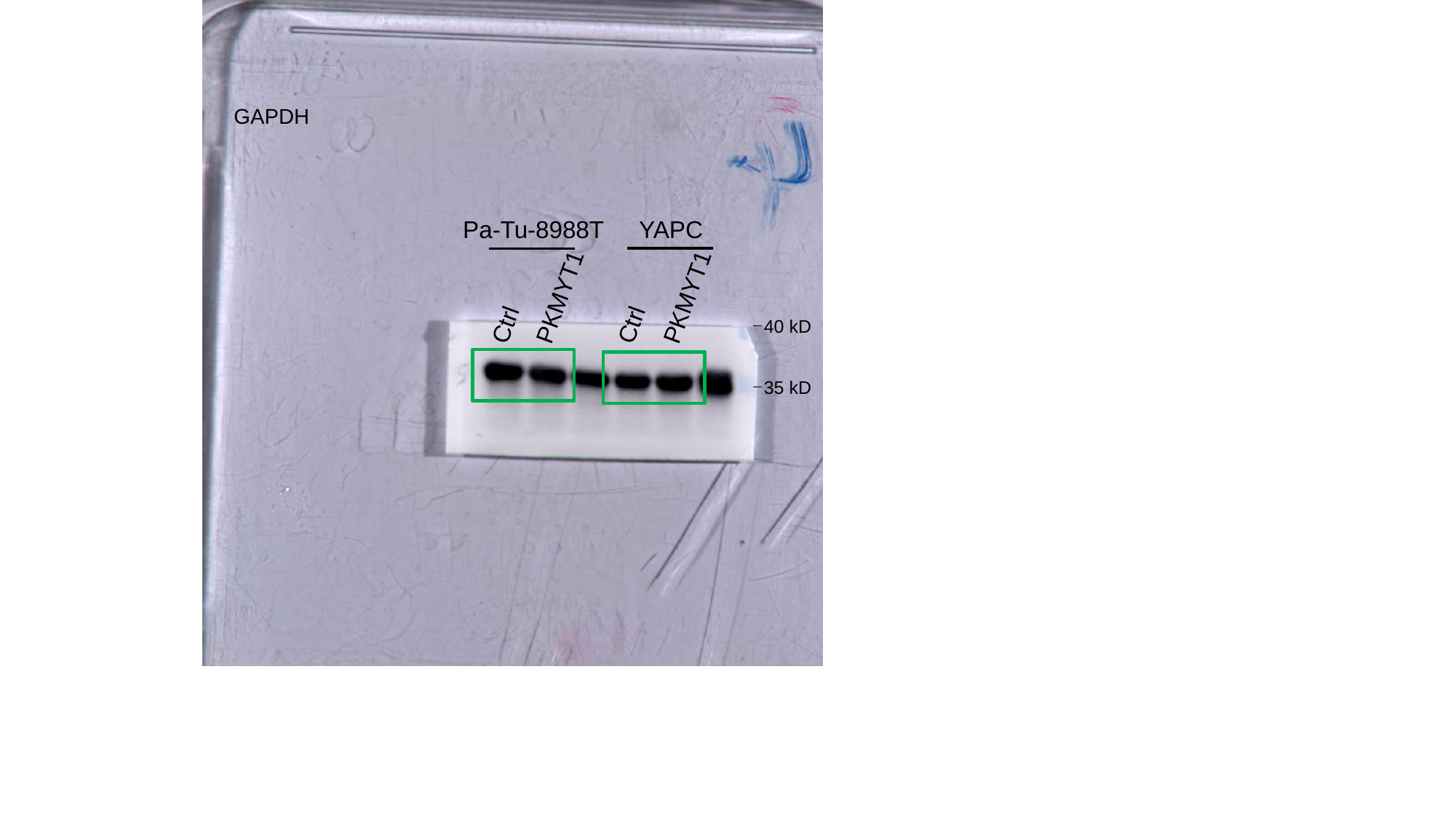

GAPDH
Pa-Tu-8988T
YAPC
PKMYT1
PKMYT1
Ctrl
Ctrl
40 kD
35 kD

## Slide 2
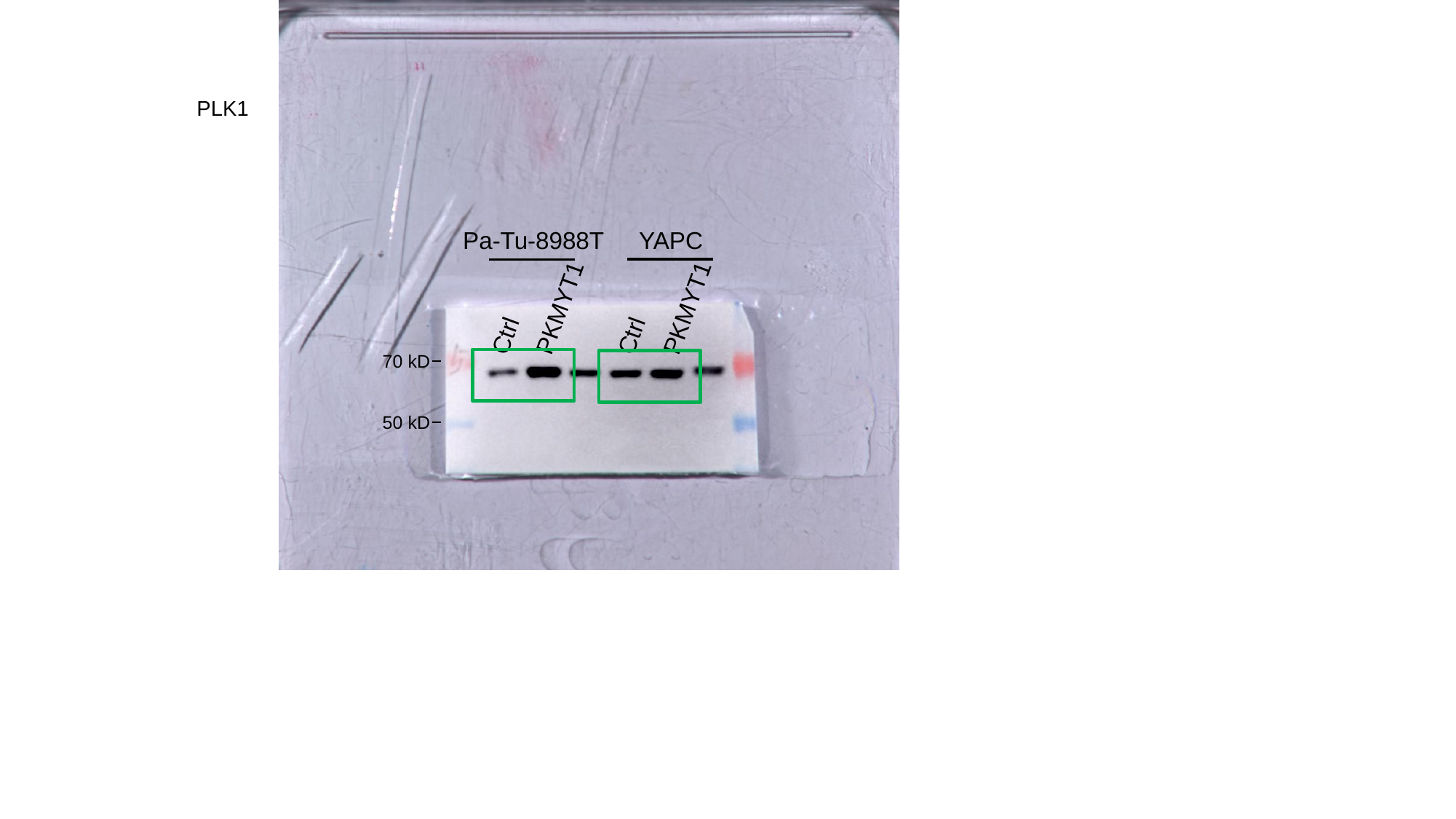

PLK1
Pa-Tu-8988T
YAPC
PKMYT1
PKMYT1
Ctrl
Ctrl
70 kD
50 kD
