# Supplementary material for: Genome-wide CRISPR screens identify PKMYT1 as a therapeutic target in pancreatic ductal adenocarcinoma
Source: EMBO Mol Med. 2024 Apr 3;16(5):5. doi: 10.1038/s44321-024-00060-y (PMC11099189; doi:10.1038/s44321-024-00060-y)
Supplement: Supplementary file 11 — Source data Fig. 6 [file 44321_2024_60_MOESM11_ESM.zip › Figure 6/6E/YAPC/6E YAPC.pptx]

## Slide 1
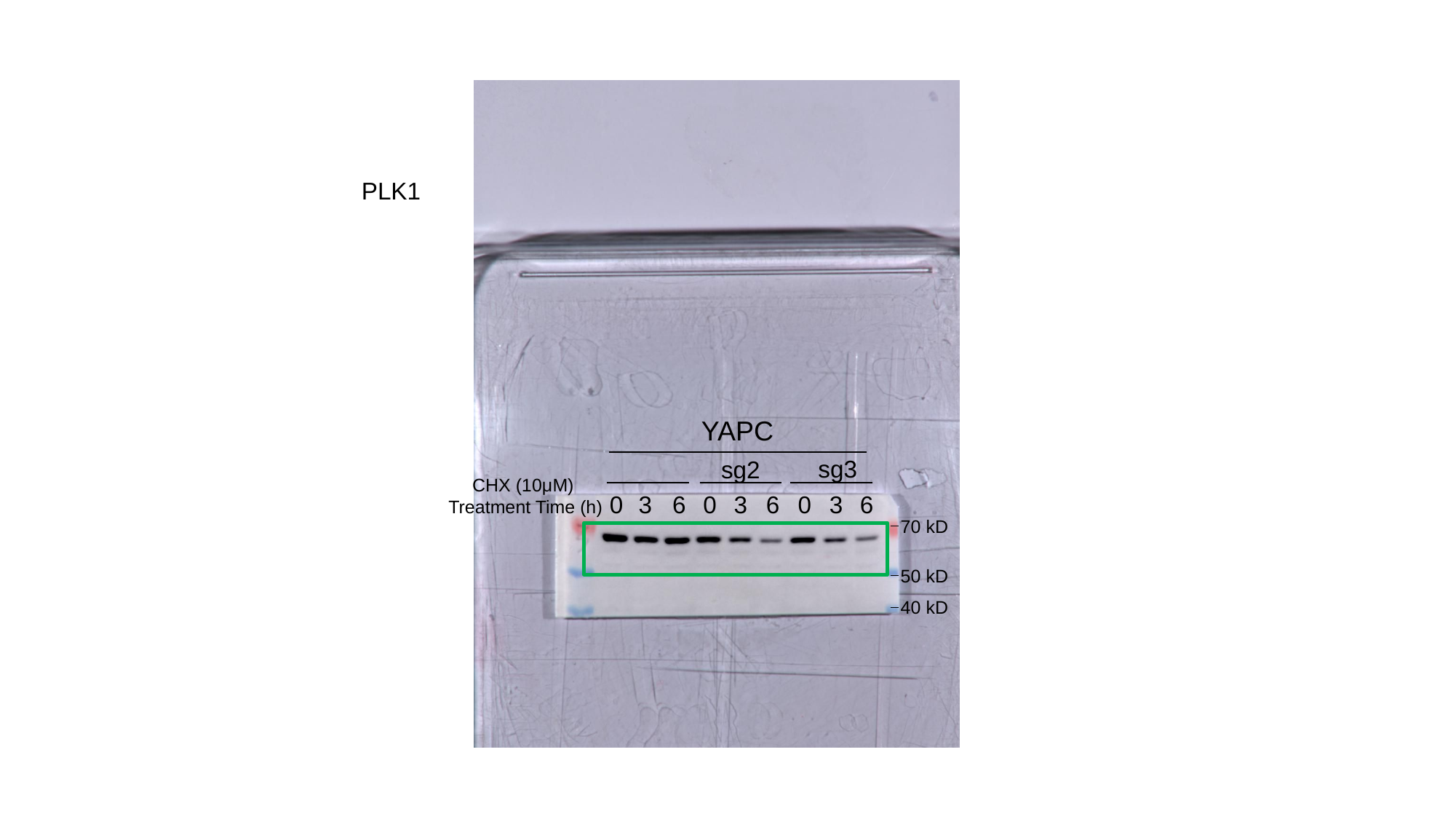

PLK1
YAPC
sg3
sg2
CHX (10μM)
 Treatment Time (h)
0
3
6
0
3
6
0
3
6
70 kD
50 kD
40 kD

## Slide 2
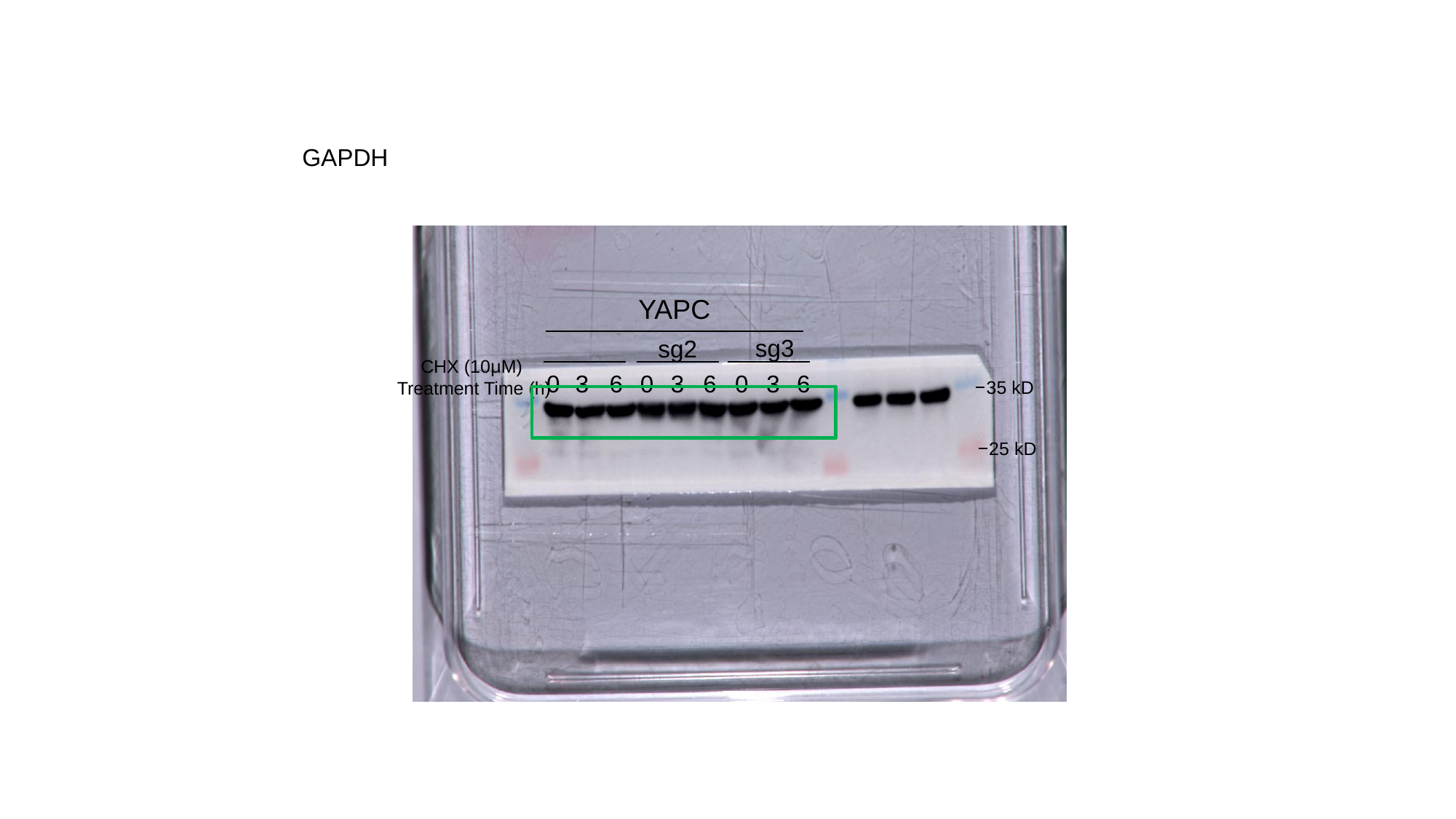

GAPDH
YAPC
sg3
sg2
CHX (10μM)
 Treatment Time (h)
0
3
6
0
3
6
0
3
6
35 kD
25 kD
