# Supplementary figures and images for: Genome-wide CRISPR screens identify PKMYT1 as a therapeutic target in pancreatic ductal adenocarcinoma (part 4 of 4)
Source: EMBO Mol Med. 2024 Apr 3;16(5):5. doi: 10.1038/s44321-024-00060-y (PMC11099189; doi:10.1038/s44321-024-00060-y)

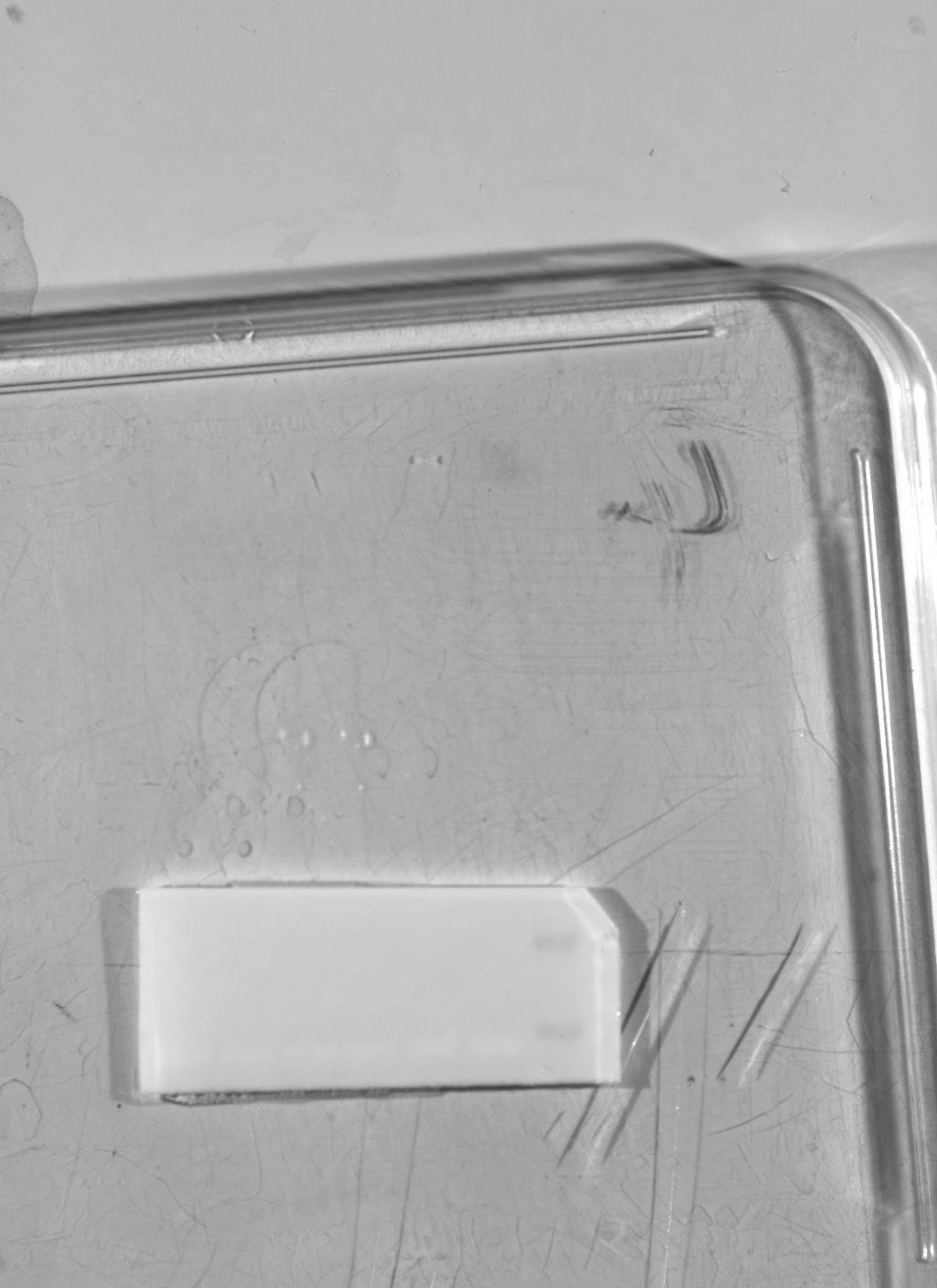

Supplement: Supplementary file 11 — Source data Fig. 6 [file 44321_2024_60_MOESM11_ESM.zip › Figure 6/6F/YAPC/GAPDH 0.1/5-2 GAP 0.1 _Ch-Marker.tif]

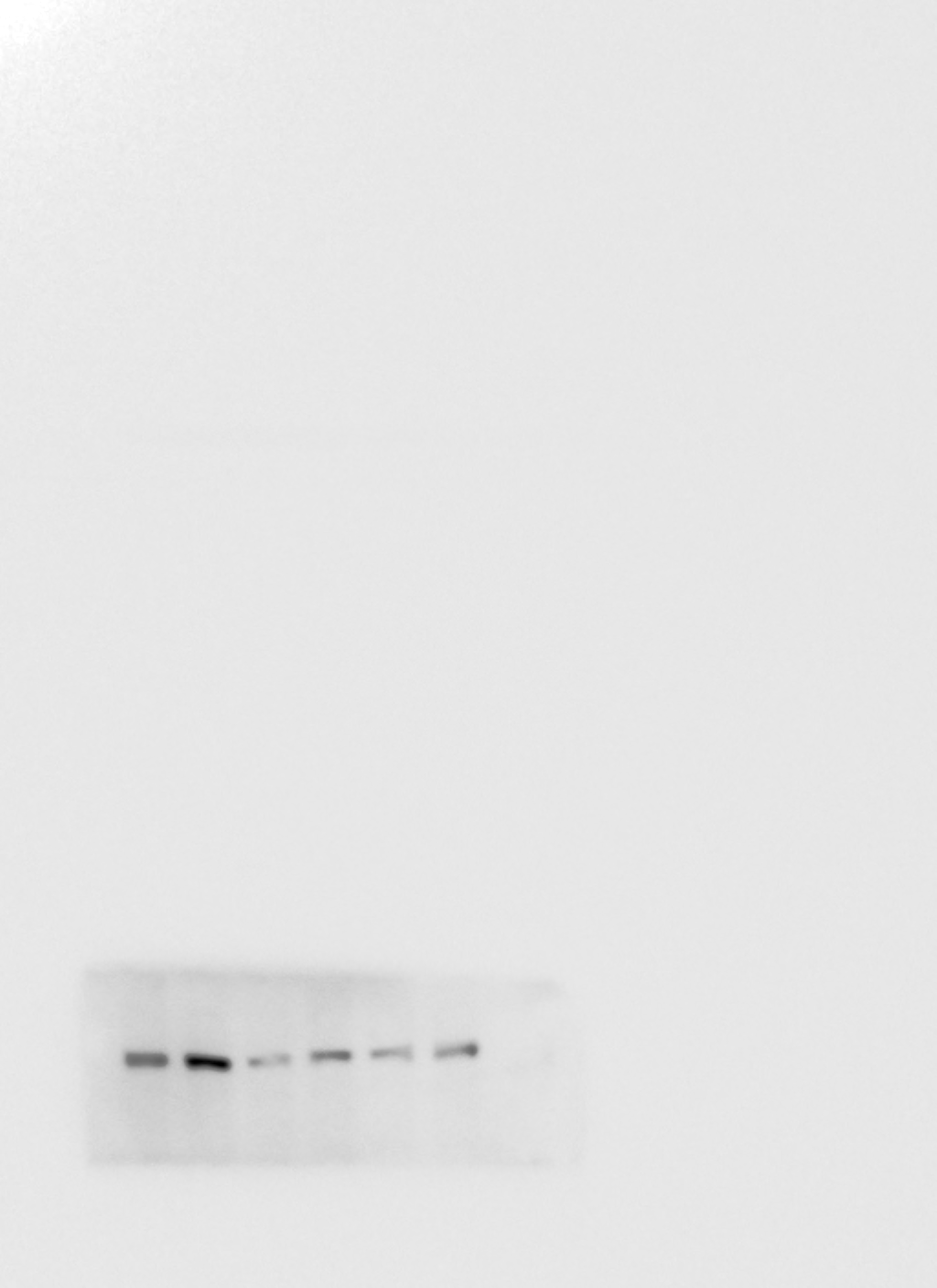

Supplement: Supplementary file 11 — Source data Fig. 6 [file 44321_2024_60_MOESM11_ESM.zip › Figure 6/6F/YAPC/PLK1 3.8/5-2 PLK 2nd 3.8 _Ch.tif]

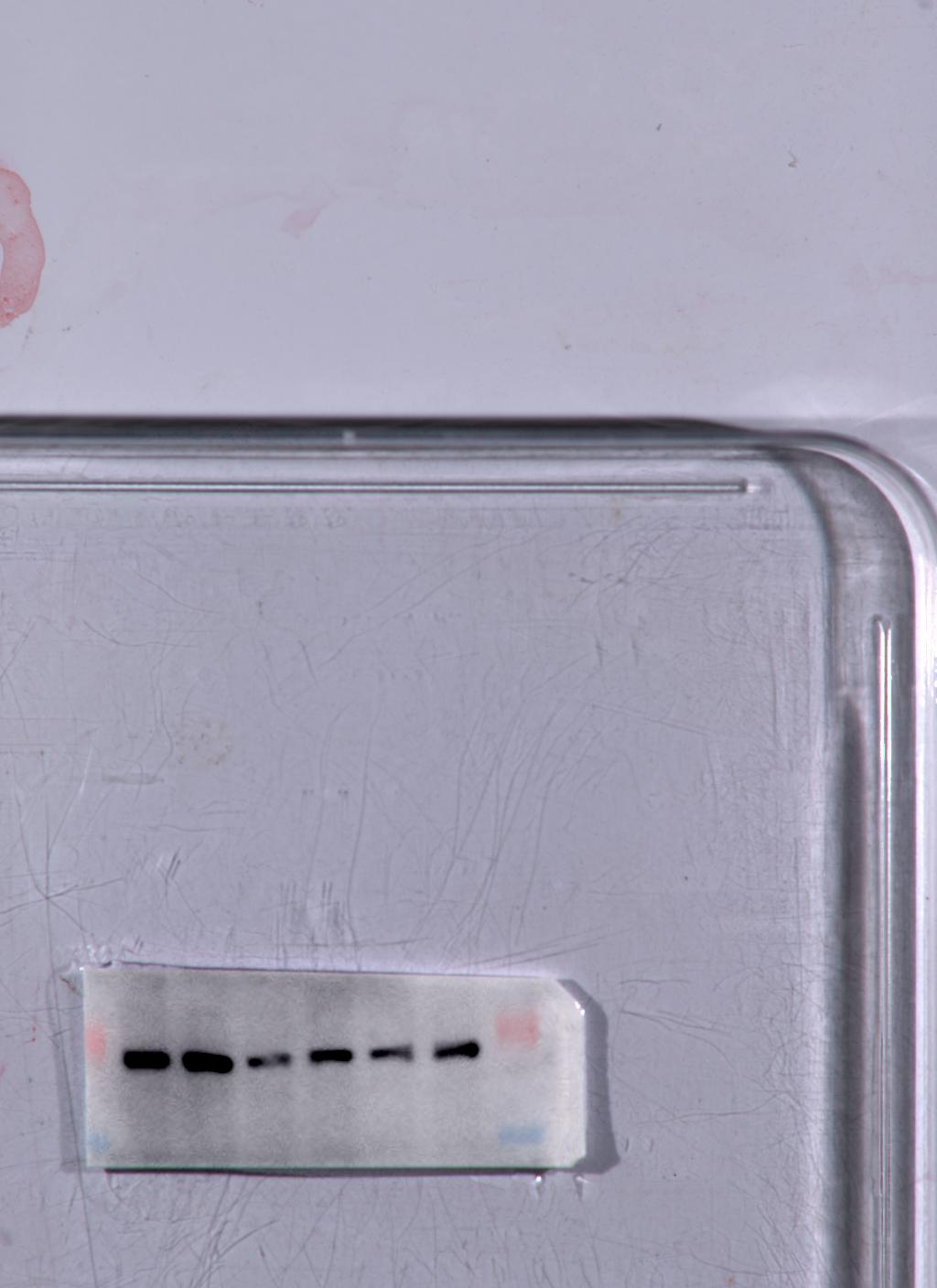

Supplement: Supplementary file 11 — Source data Fig. 6 [file 44321_2024_60_MOESM11_ESM.zip › Figure 6/6F/YAPC/PLK1 3.8/5-2 PLK 2nd 3.8 _Ch+Marker.jpg]

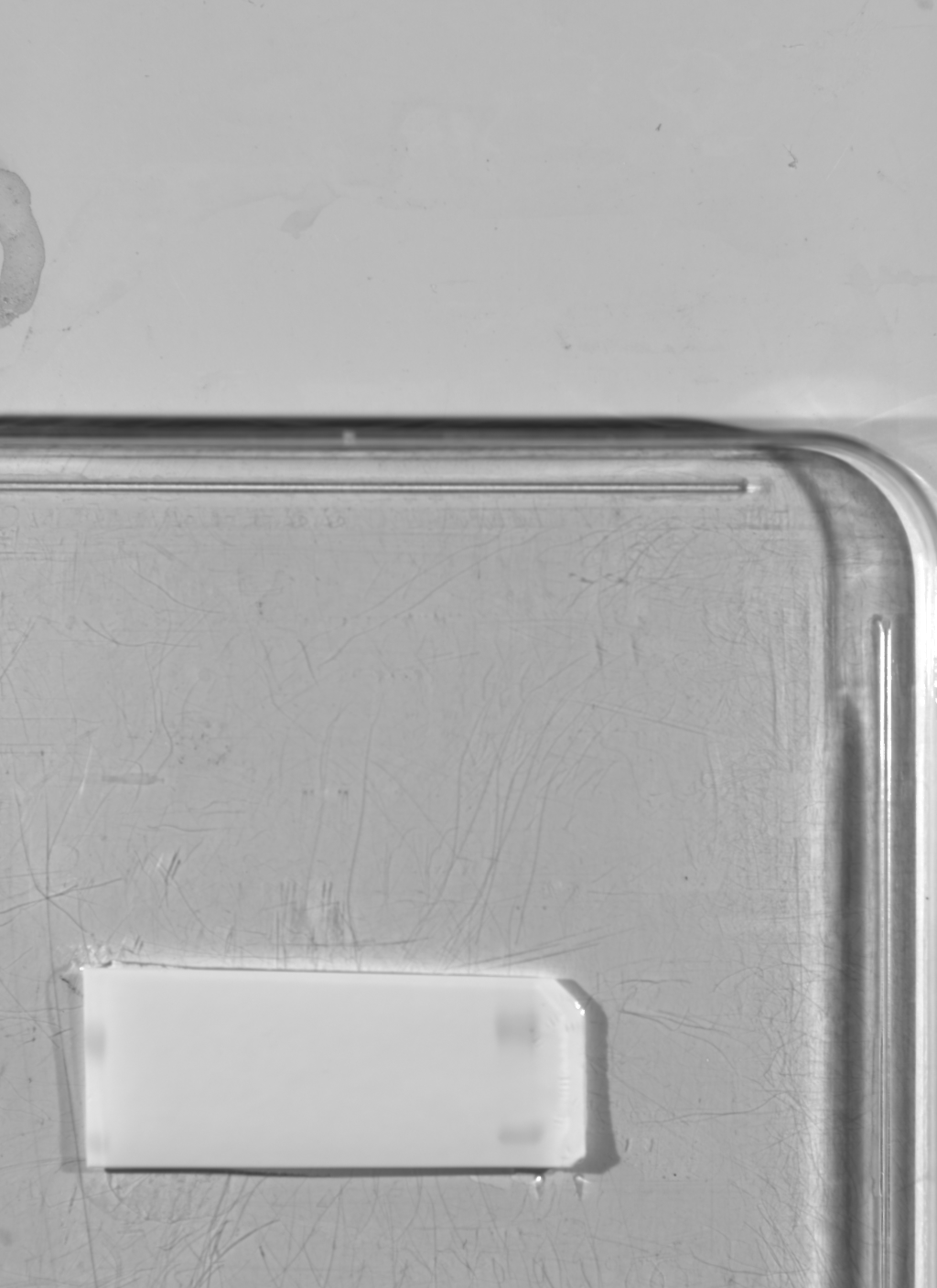

Supplement: Supplementary file 11 — Source data Fig. 6 [file 44321_2024_60_MOESM11_ESM.zip › Figure 6/6F/YAPC/PLK1 3.8/5-2 PLK 2nd 3.8 _Ch-Marker.tif]

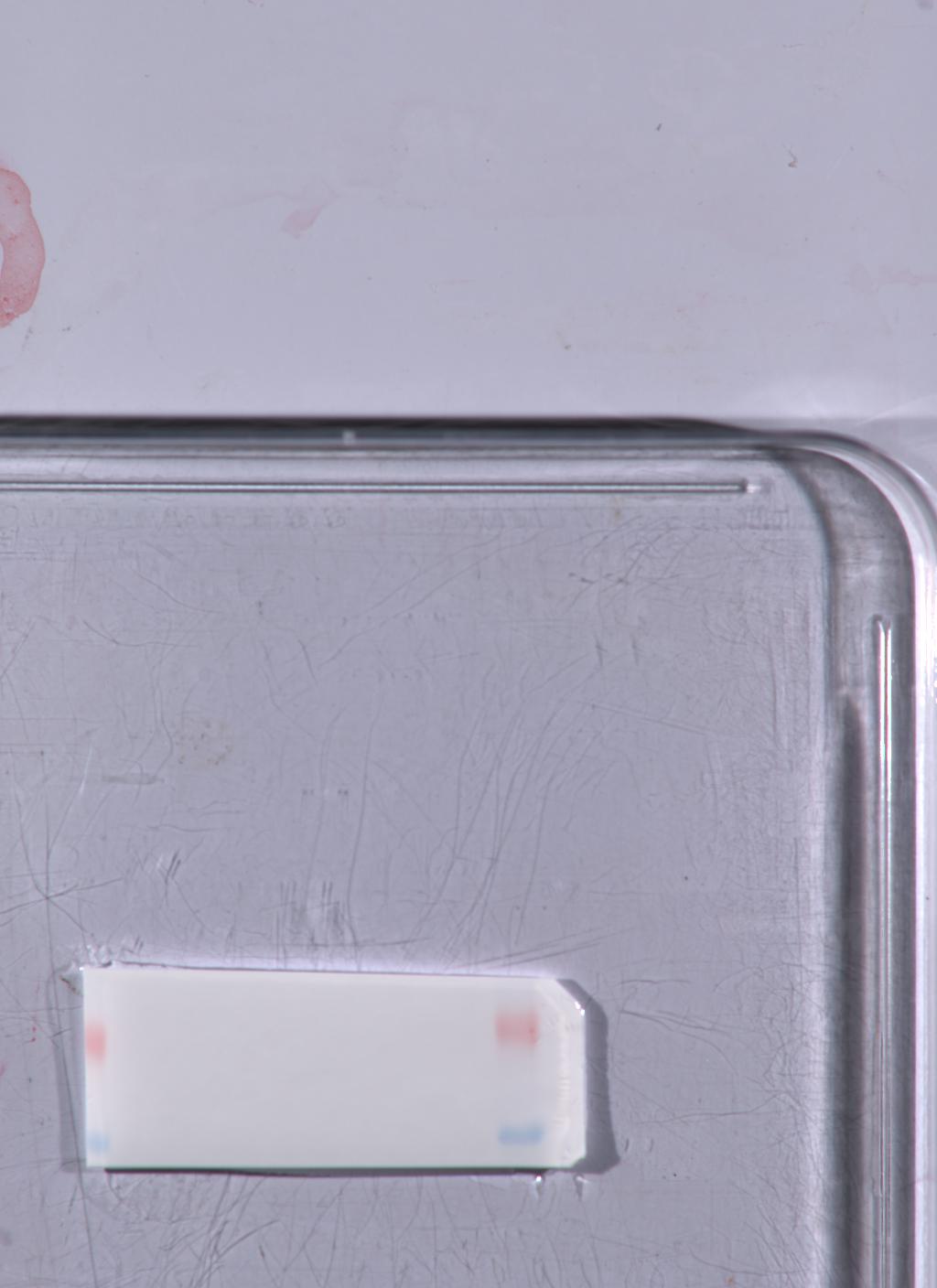

Supplement: Supplementary file 11 — Source data Fig. 6 [file 44321_2024_60_MOESM11_ESM.zip › Figure 6/6F/YAPC/PLK1 3.8/5-2 PLK 2nd 3.8 Ch-Marker.jpg]

## Slide 1
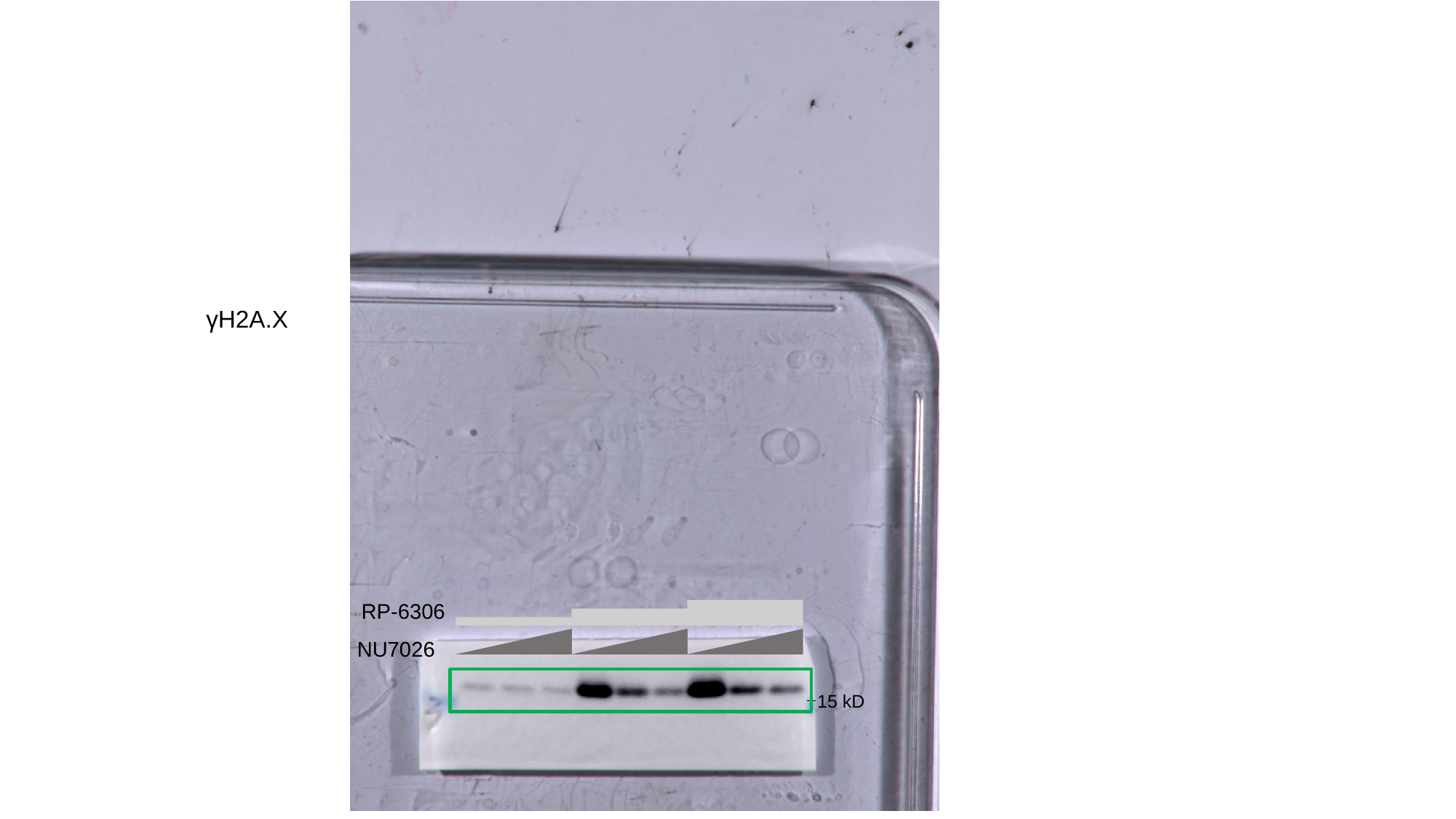

γH2A.X
RP-6306
NU7026
15 kD

## Slide 2
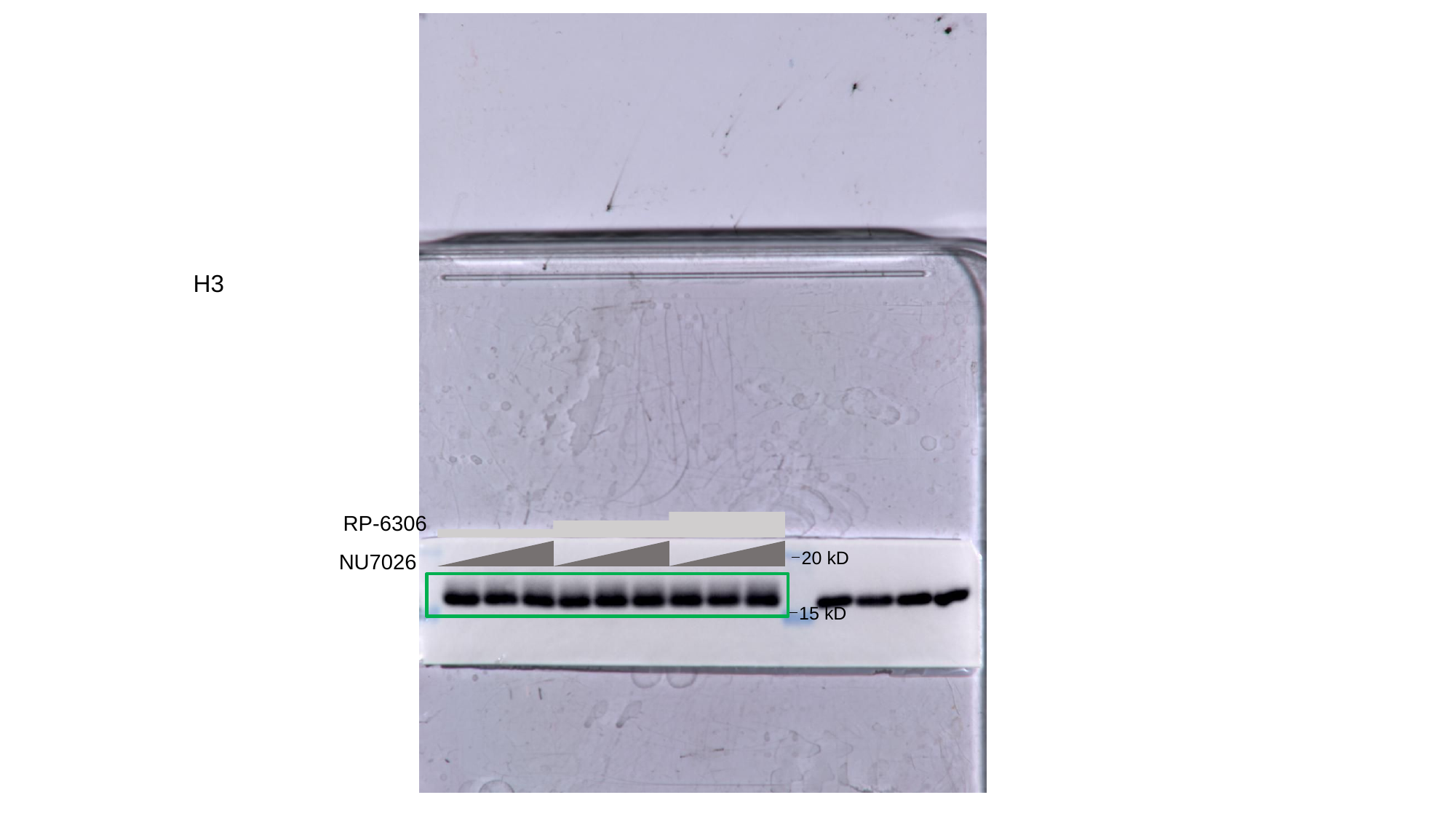

H3
RP-6306
20 kD
NU7026
15 kD

## Slide 3
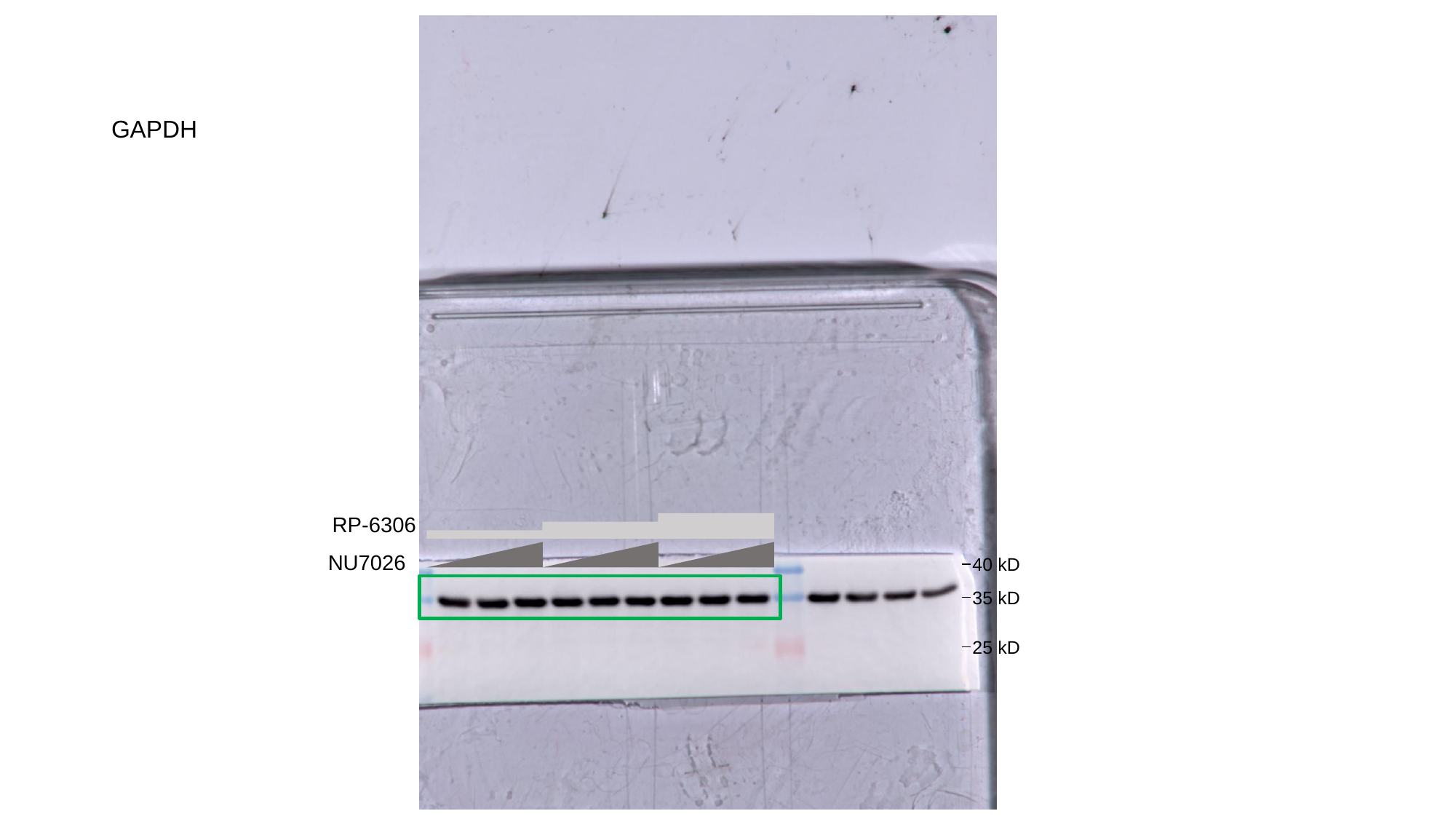

GAPDH
RP-6306
NU7026
40 kD
35 kD
25 kD

Supplement: Supplementary file 12 — Source data Fig. 7 [file 44321_2024_60_MOESM12_ESM.zip › Figure 7/7A/NU7026.pptx]

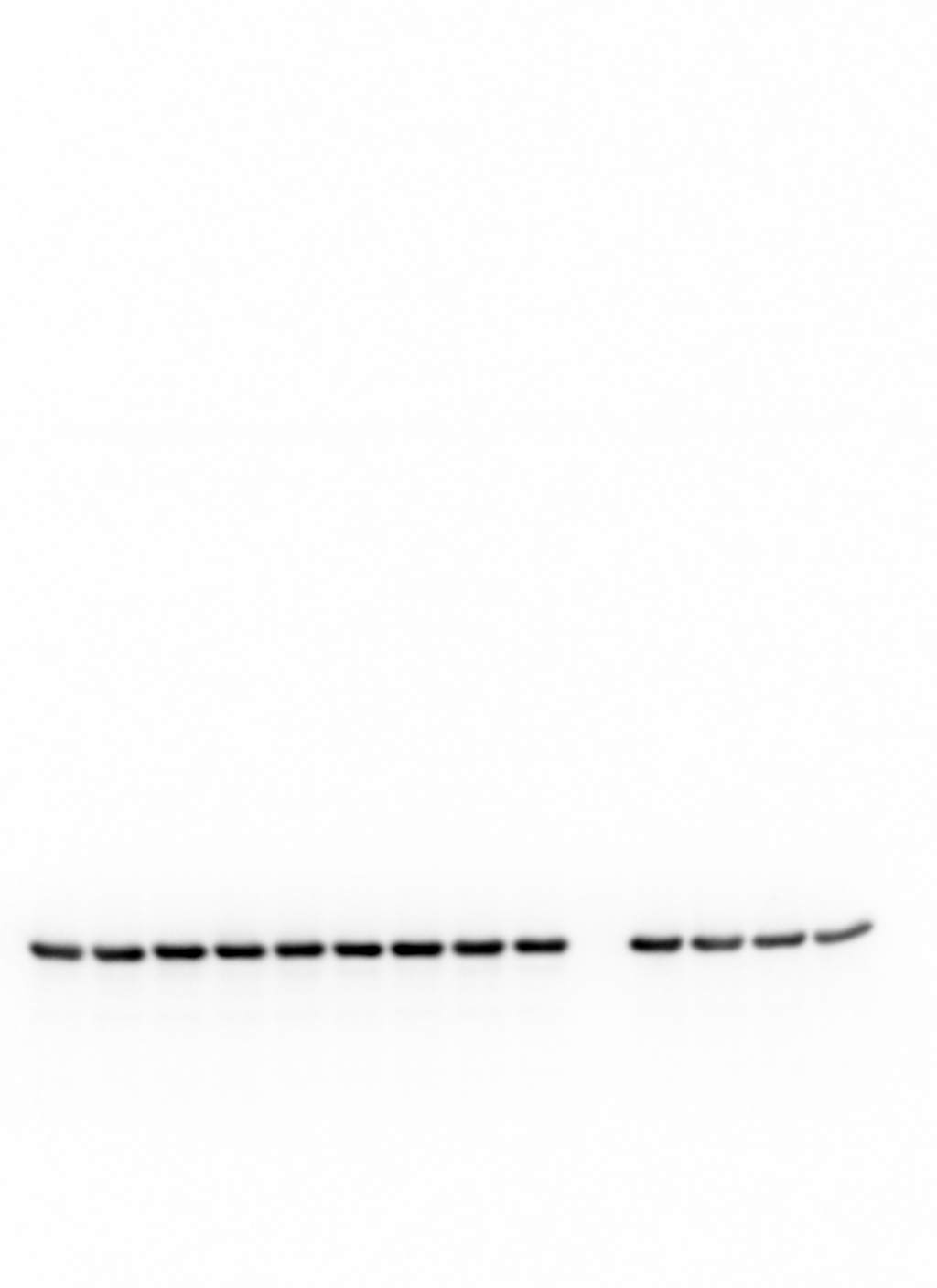

Supplement: Supplementary file 12 — Source data Fig. 7 [file 44321_2024_60_MOESM12_ESM.zip › Figure 7/7A/NU7026/GAPDH 0.1/3 GAP 0.1 _Ch.tif]

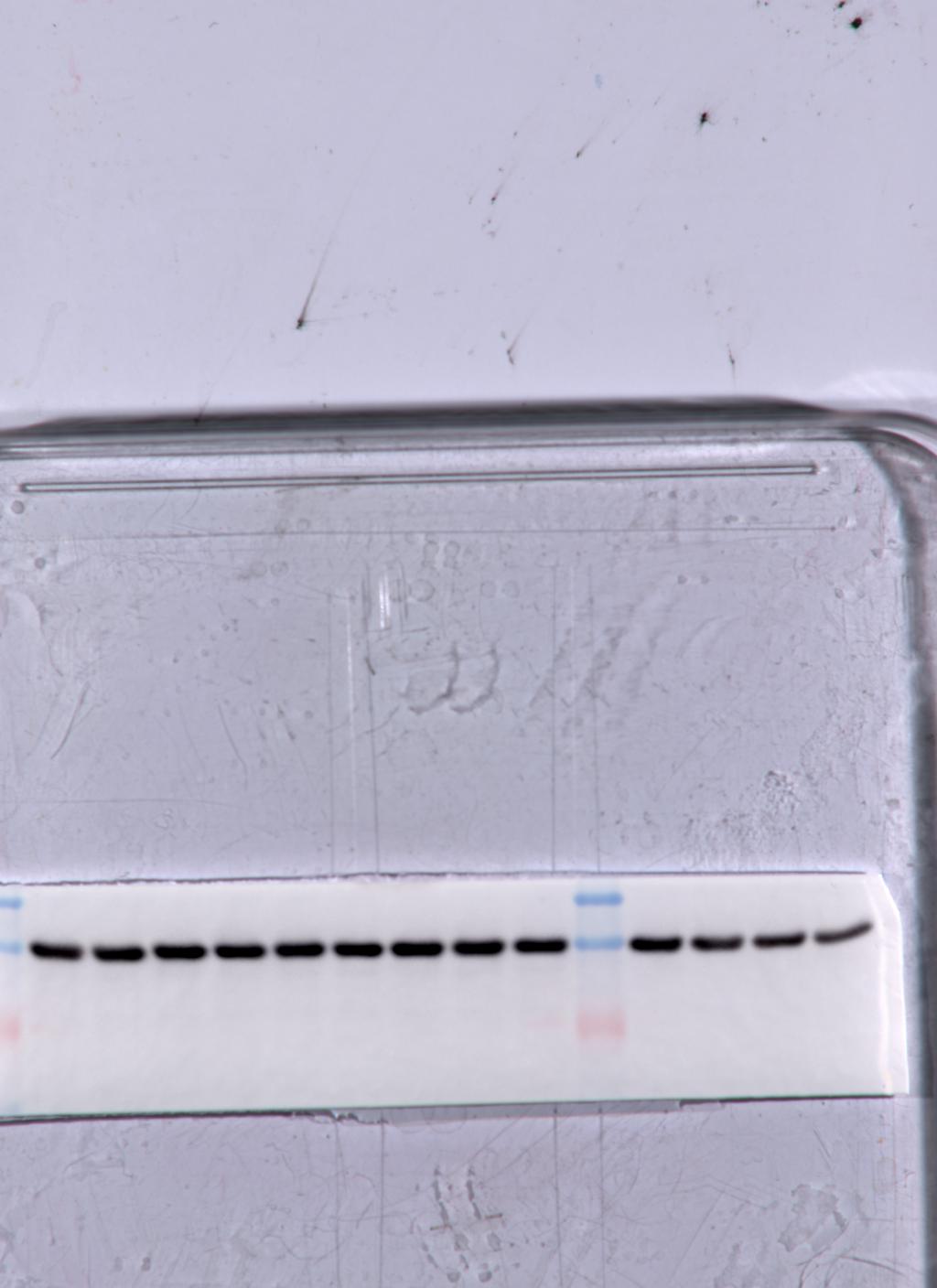

Supplement: Supplementary file 12 — Source data Fig. 7 [file 44321_2024_60_MOESM12_ESM.zip › Figure 7/7A/NU7026/GAPDH 0.1/3 GAP 0.1 _Ch+Marker.jpg]

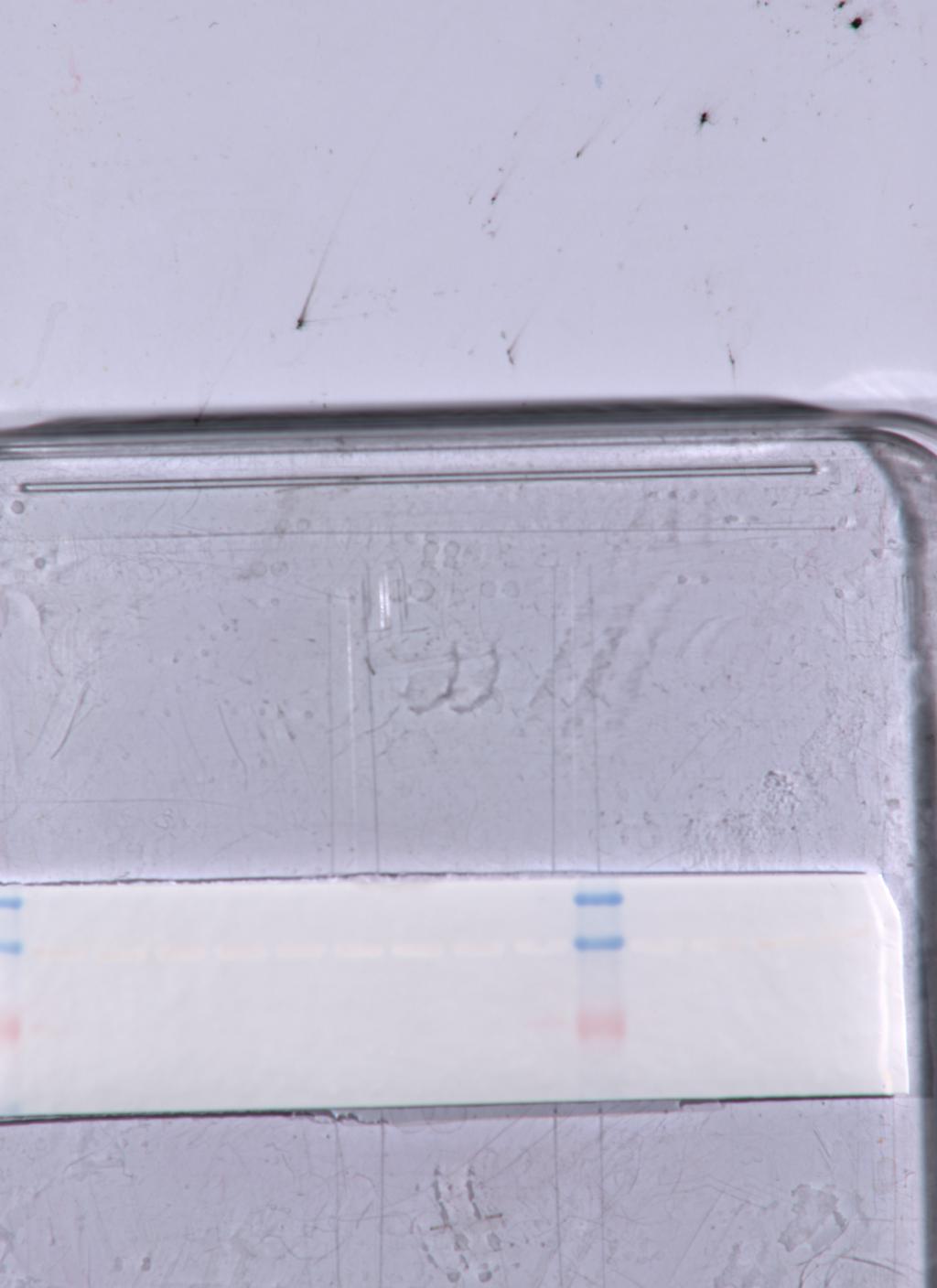

Supplement: Supplementary file 12 — Source data Fig. 7 [file 44321_2024_60_MOESM12_ESM.zip › Figure 7/7A/NU7026/GAPDH 0.1/3 GAP 0.1 _Ch-Marker.jpg]

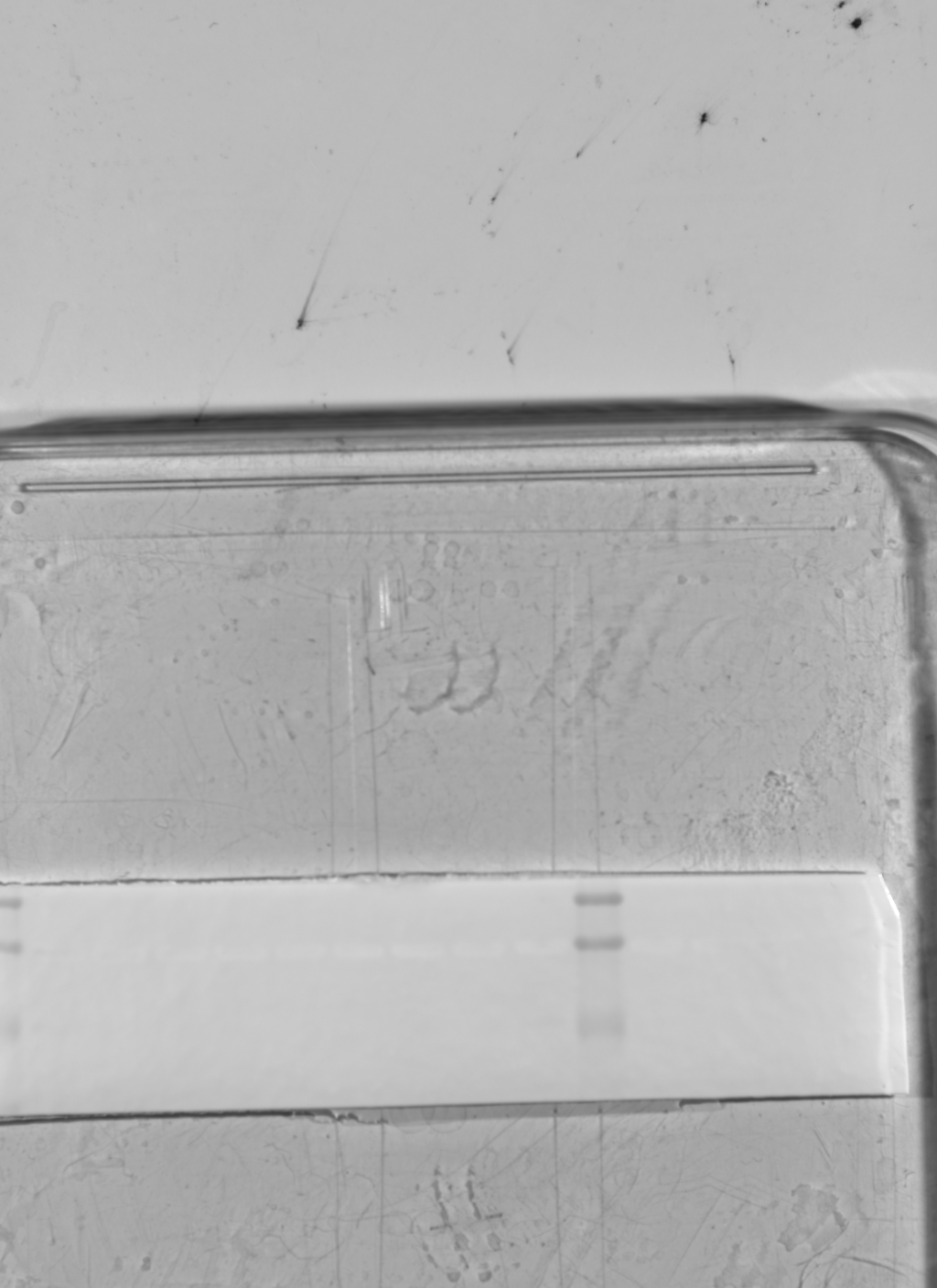

Supplement: Supplementary file 12 — Source data Fig. 7 [file 44321_2024_60_MOESM12_ESM.zip › Figure 7/7A/NU7026/GAPDH 0.1/3 GAP 0.1 _Ch-Marker.tif]

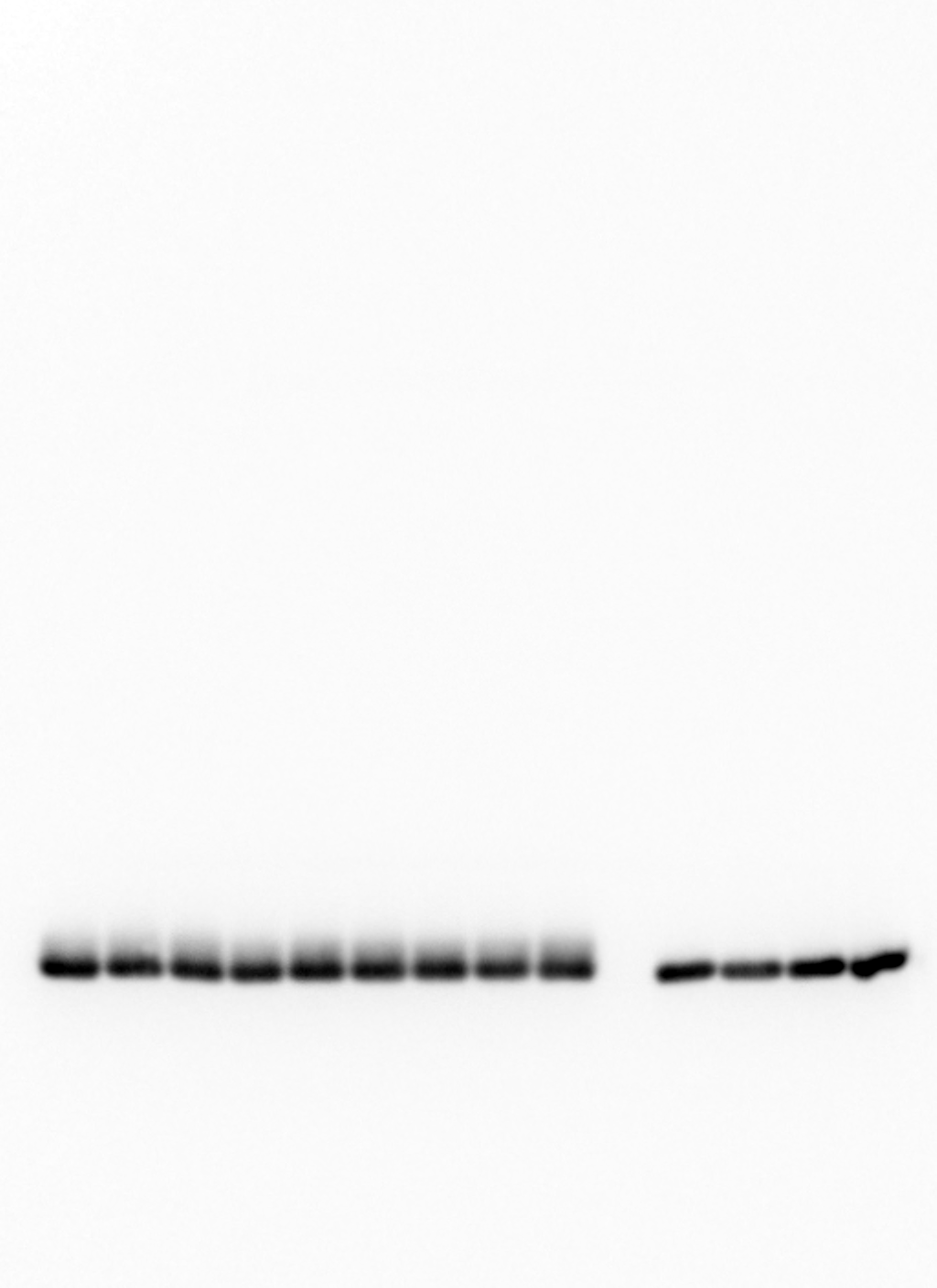

Supplement: Supplementary file 12 — Source data Fig. 7 [file 44321_2024_60_MOESM12_ESM.zip › Figure 7/7A/NU7026/H3 1st/4 H3 1st 0.6 _Ch.tif]

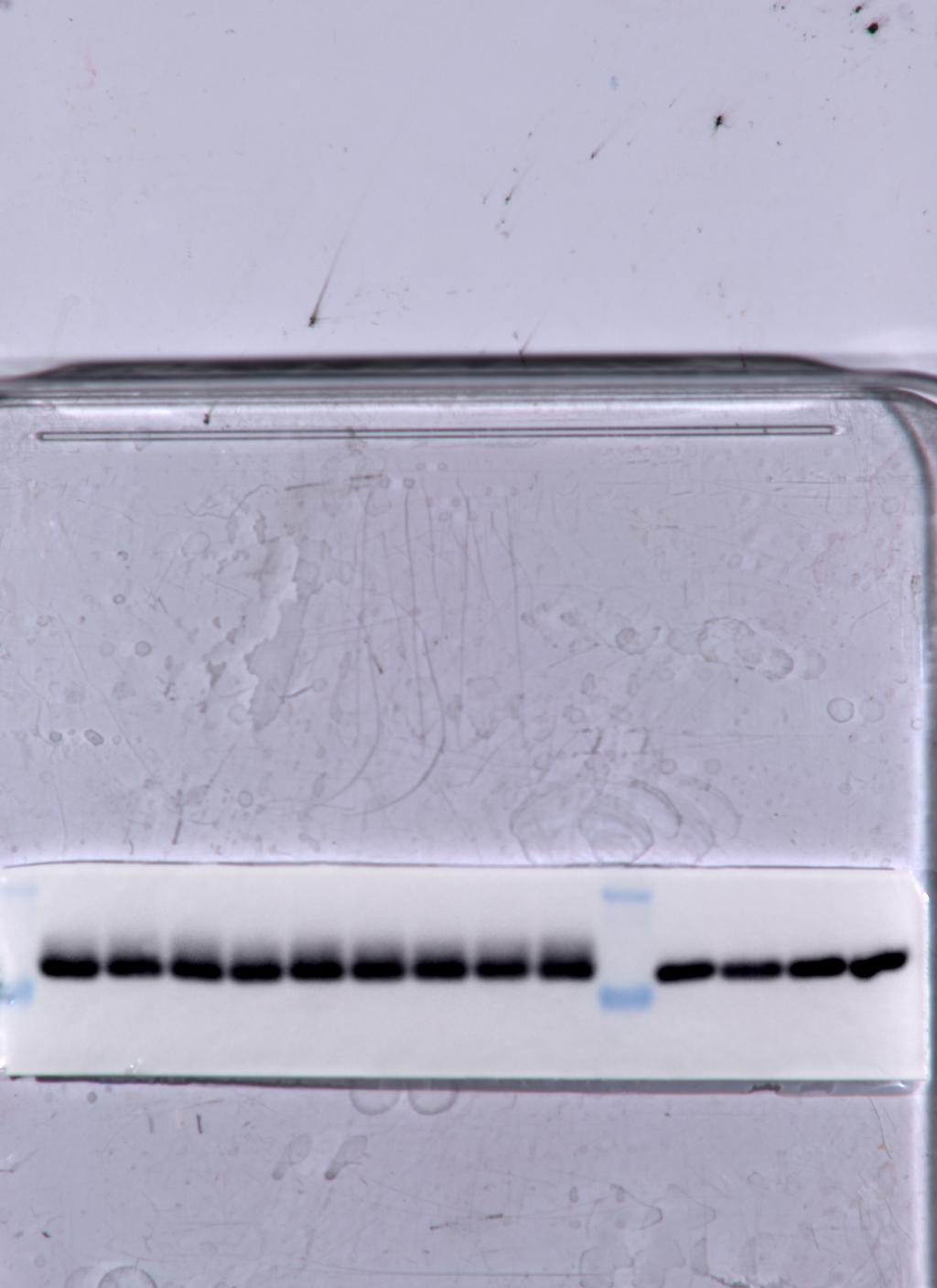

Supplement: Supplementary file 12 — Source data Fig. 7 [file 44321_2024_60_MOESM12_ESM.zip › Figure 7/7A/NU7026/H3 1st/4 H3 1st 0.6 _Ch+Marker.jpg]

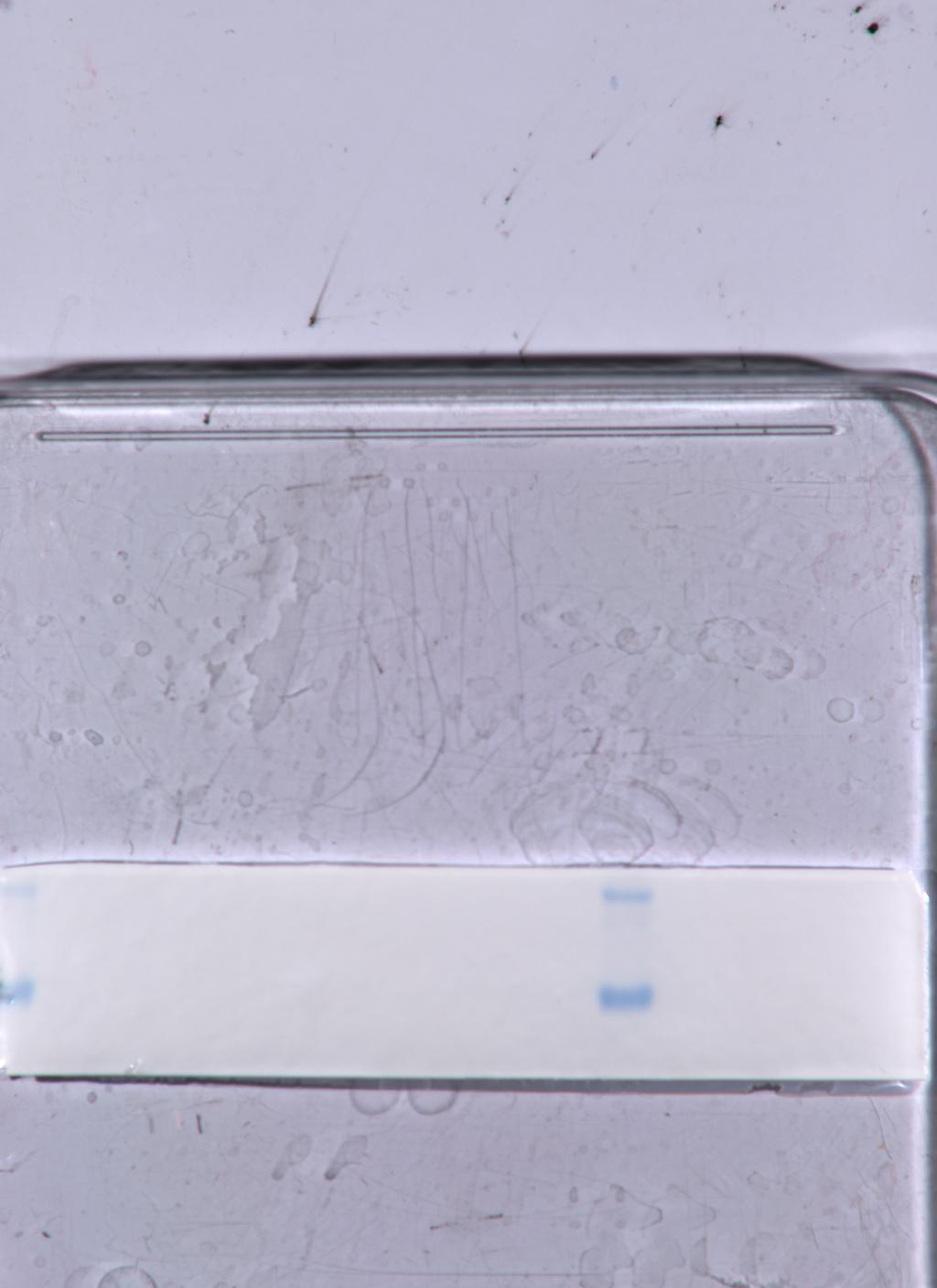

Supplement: Supplementary file 12 — Source data Fig. 7 [file 44321_2024_60_MOESM12_ESM.zip › Figure 7/7A/NU7026/H3 1st/4 H3 1st 0.6 _Ch-Marker.jpg]

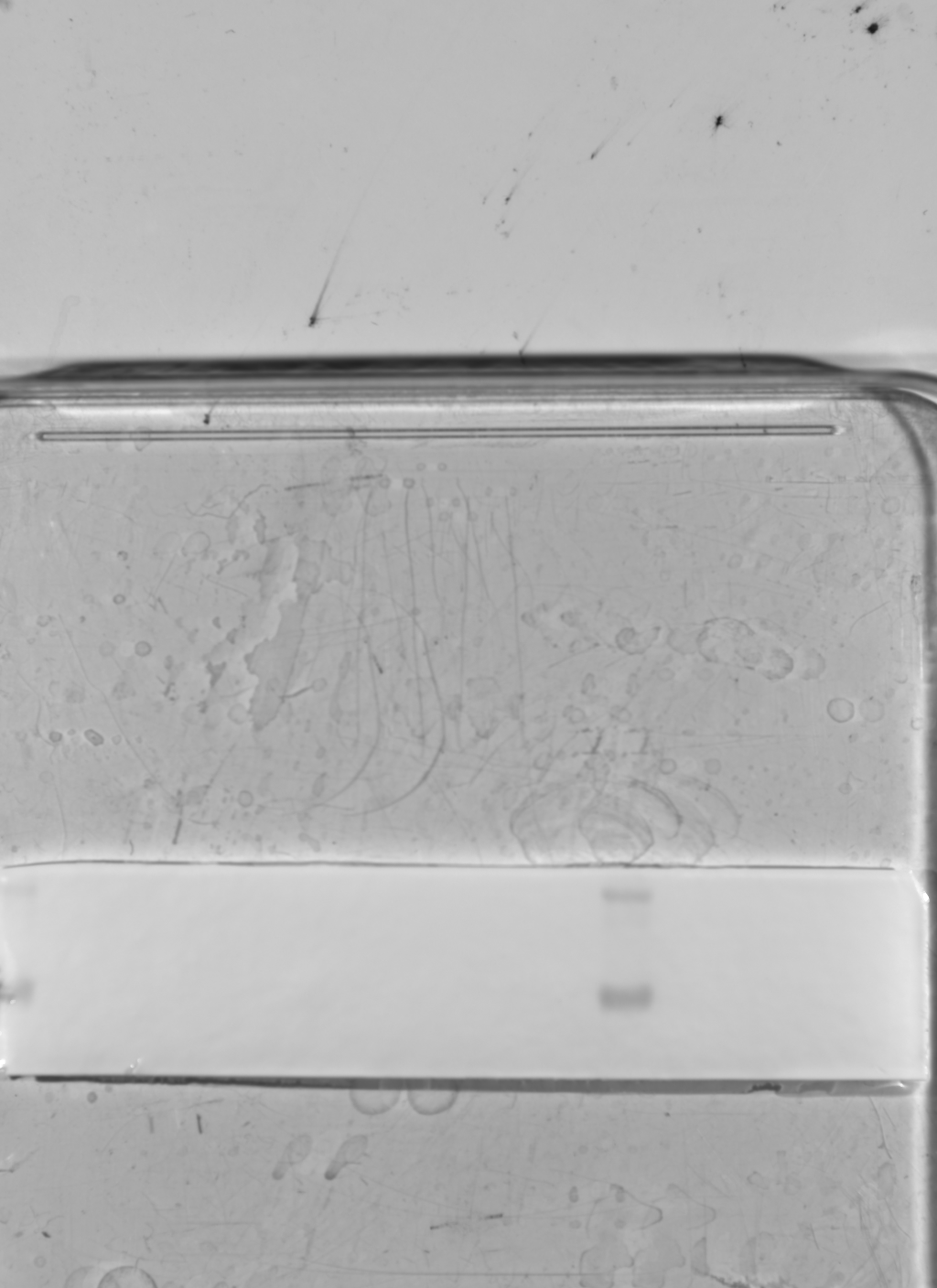

Supplement: Supplementary file 12 — Source data Fig. 7 [file 44321_2024_60_MOESM12_ESM.zip › Figure 7/7A/NU7026/H3 1st/4 H3 1st 0.6 _Ch-Marker.tif]

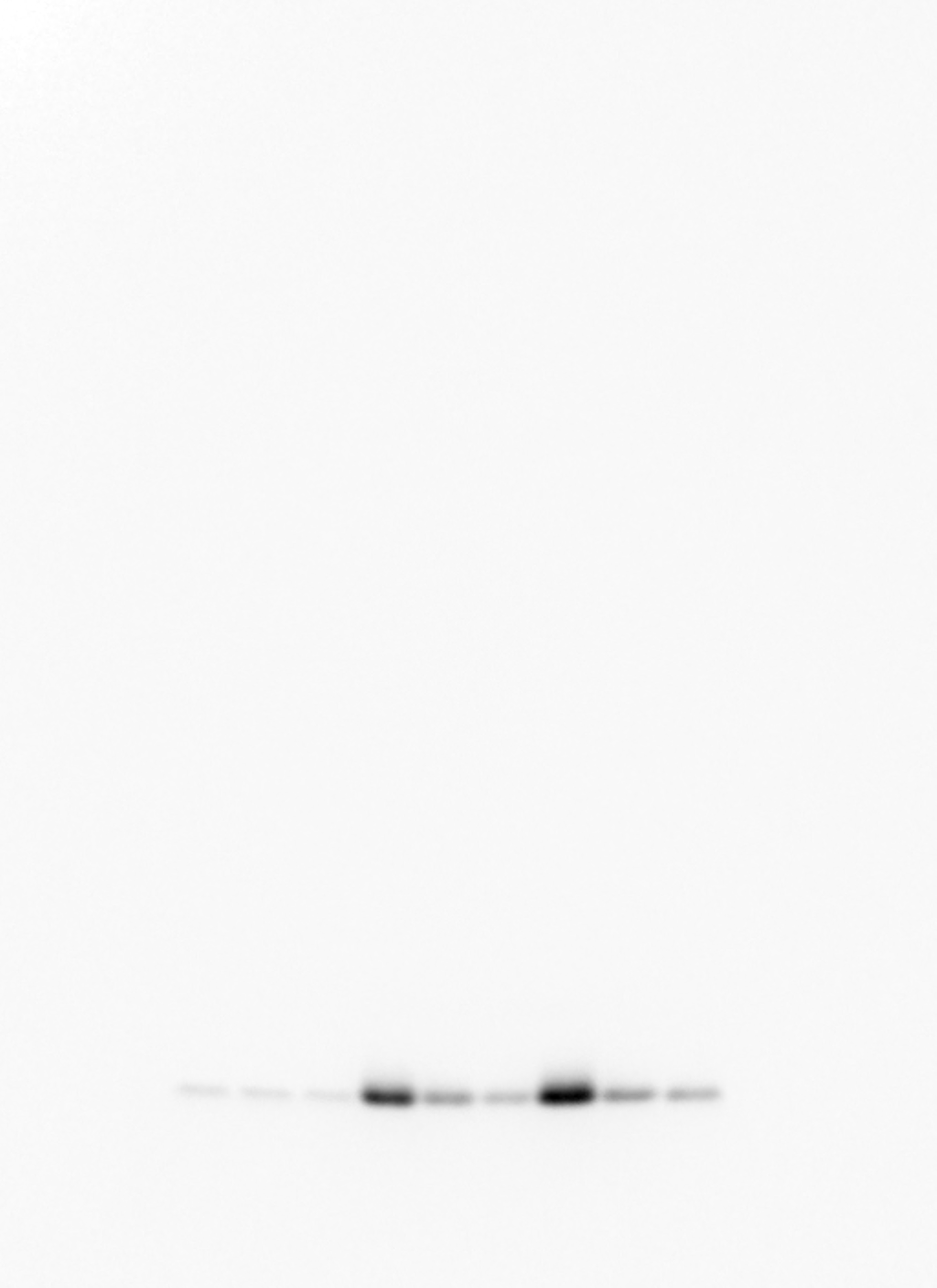

Supplement: Supplementary file 12 — Source data Fig. 7 [file 44321_2024_60_MOESM12_ESM.zip › Figure 7/7A/NU7026/rH2A 1st/3-1 rH2A 1st 1.3 _Ch.tif]

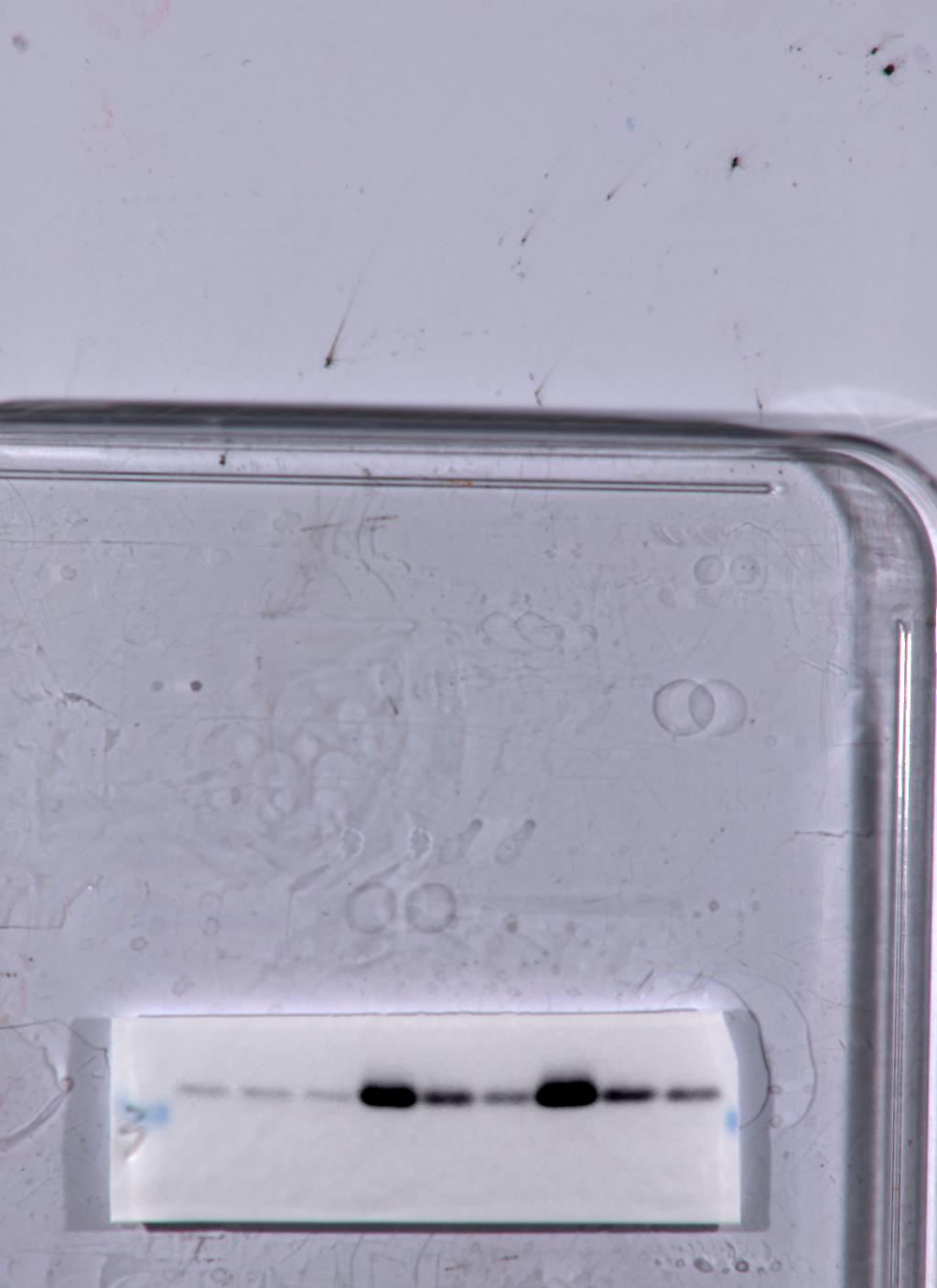

Supplement: Supplementary file 12 — Source data Fig. 7 [file 44321_2024_60_MOESM12_ESM.zip › Figure 7/7A/NU7026/rH2A 1st/3-1 rH2A 1st 1.3 _Ch+Marker.jpg]

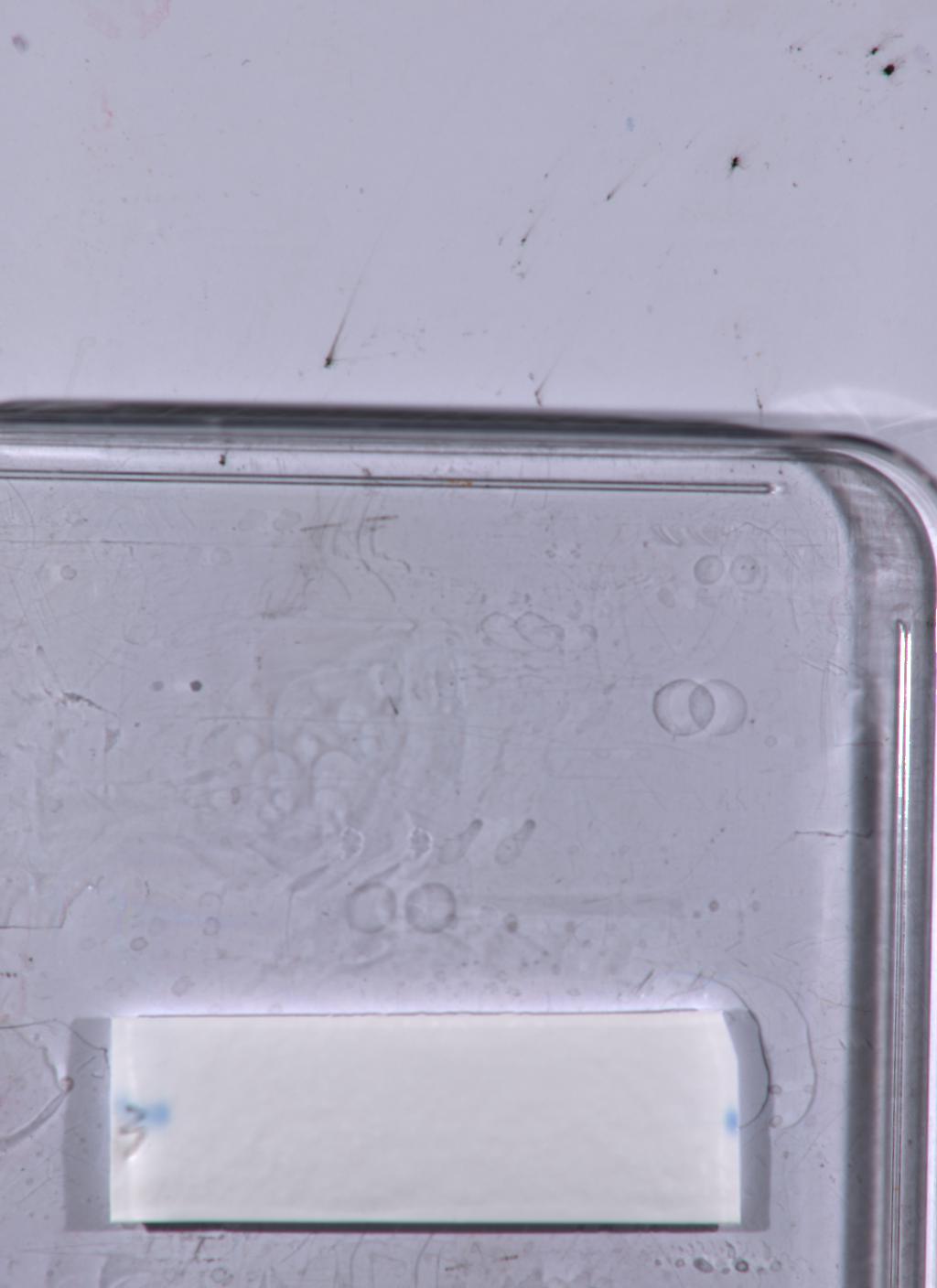

Supplement: Supplementary file 12 — Source data Fig. 7 [file 44321_2024_60_MOESM12_ESM.zip › Figure 7/7A/NU7026/rH2A 1st/3-1 rH2A 1st 1.3 _Ch-Marker.jpg]

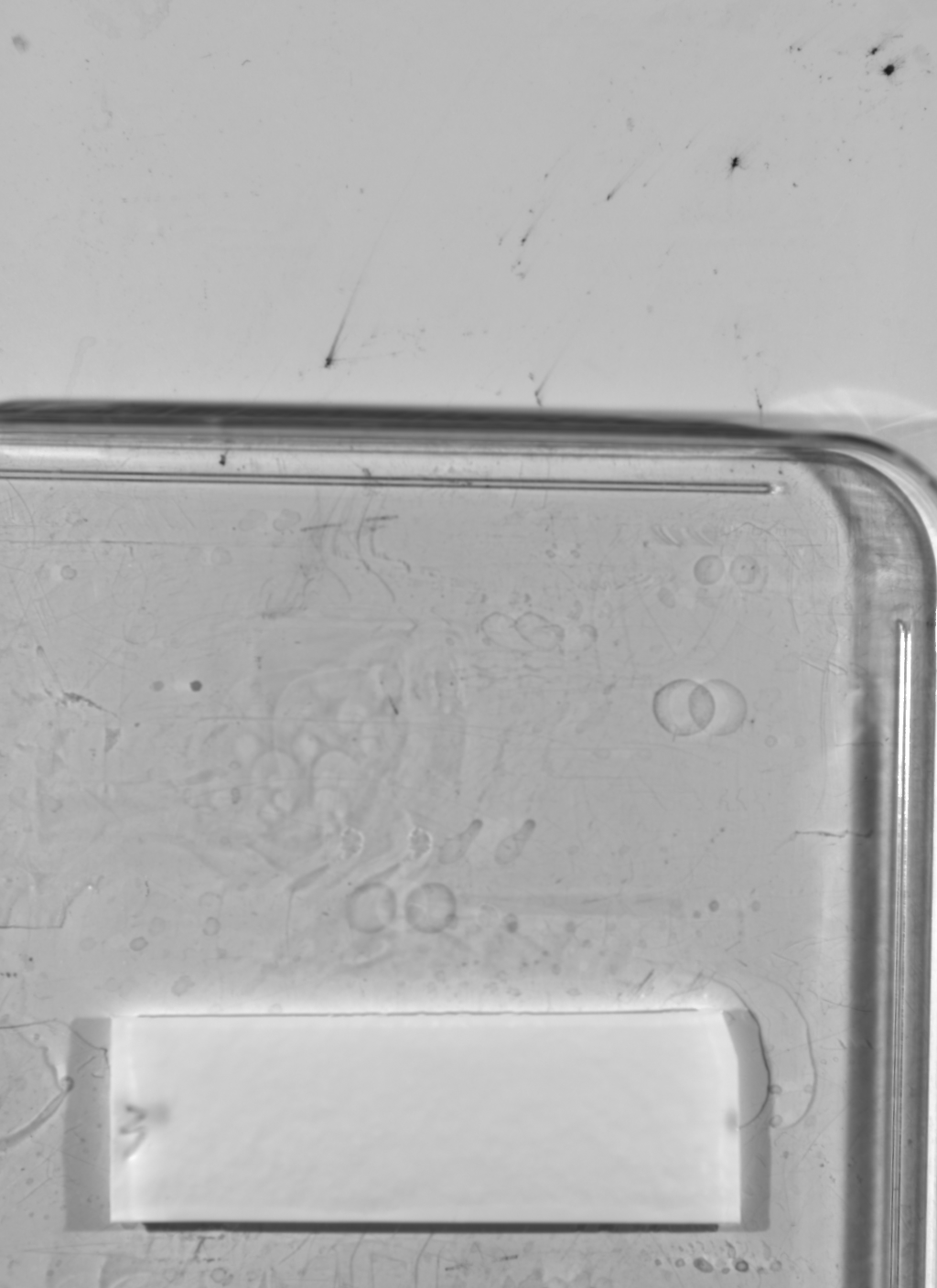

Supplement: Supplementary file 12 — Source data Fig. 7 [file 44321_2024_60_MOESM12_ESM.zip › Figure 7/7A/NU7026/rH2A 1st/3-1 rH2A 1st 1.3 _Ch-Marker.tif]

## Slide 1
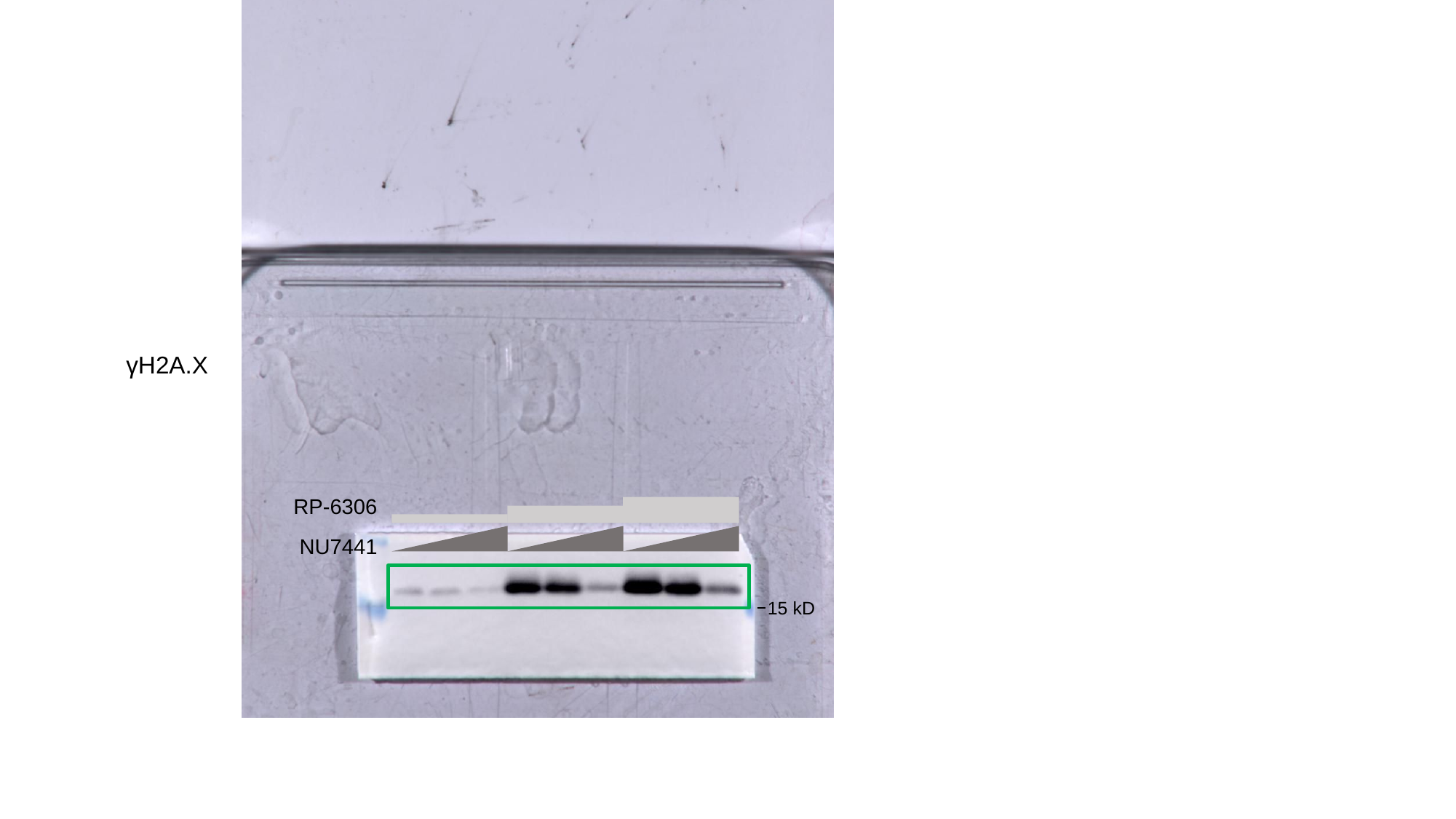

γH2A.X
RP-6306
NU7441
15 kD

## Slide 2
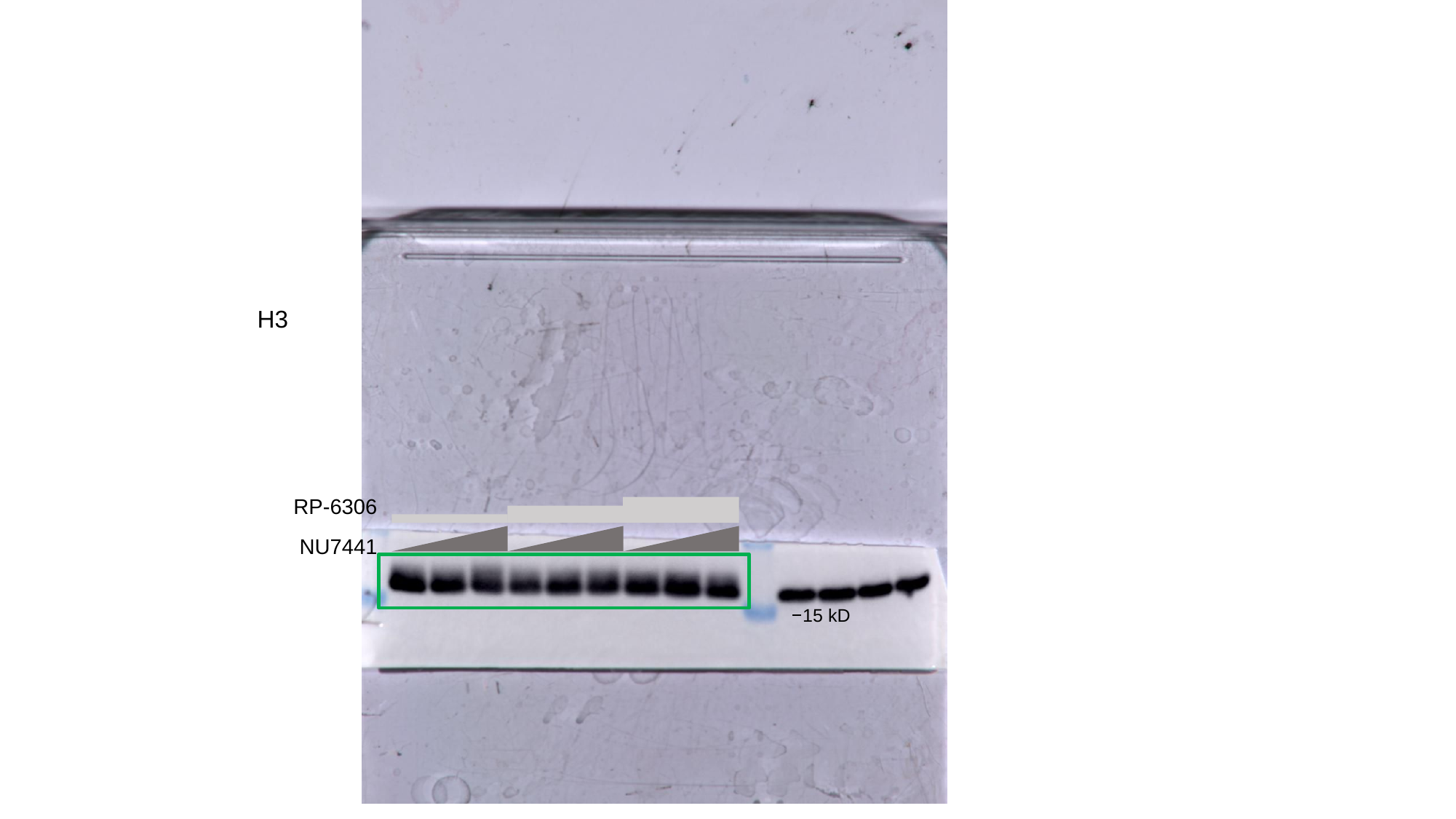

H3
RP-6306
NU7441
15 kD

## Slide 3
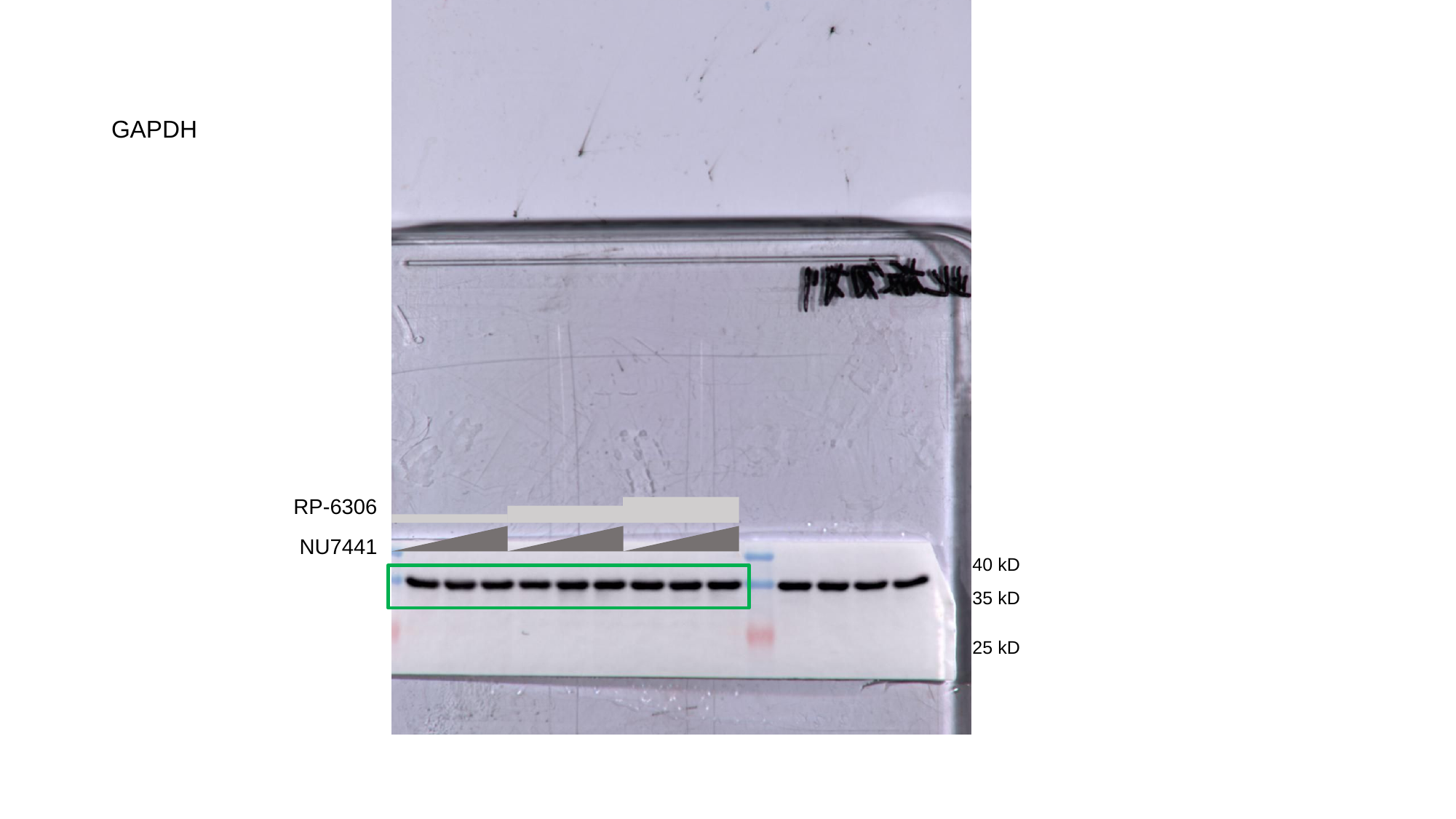

GAPDH
RP-6306
NU7441
40 kD
35 kD
25 kD

Supplement: Supplementary file 12 — Source data Fig. 7 [file 44321_2024_60_MOESM12_ESM.zip › Figure 7/7A/NU7441.pptx]

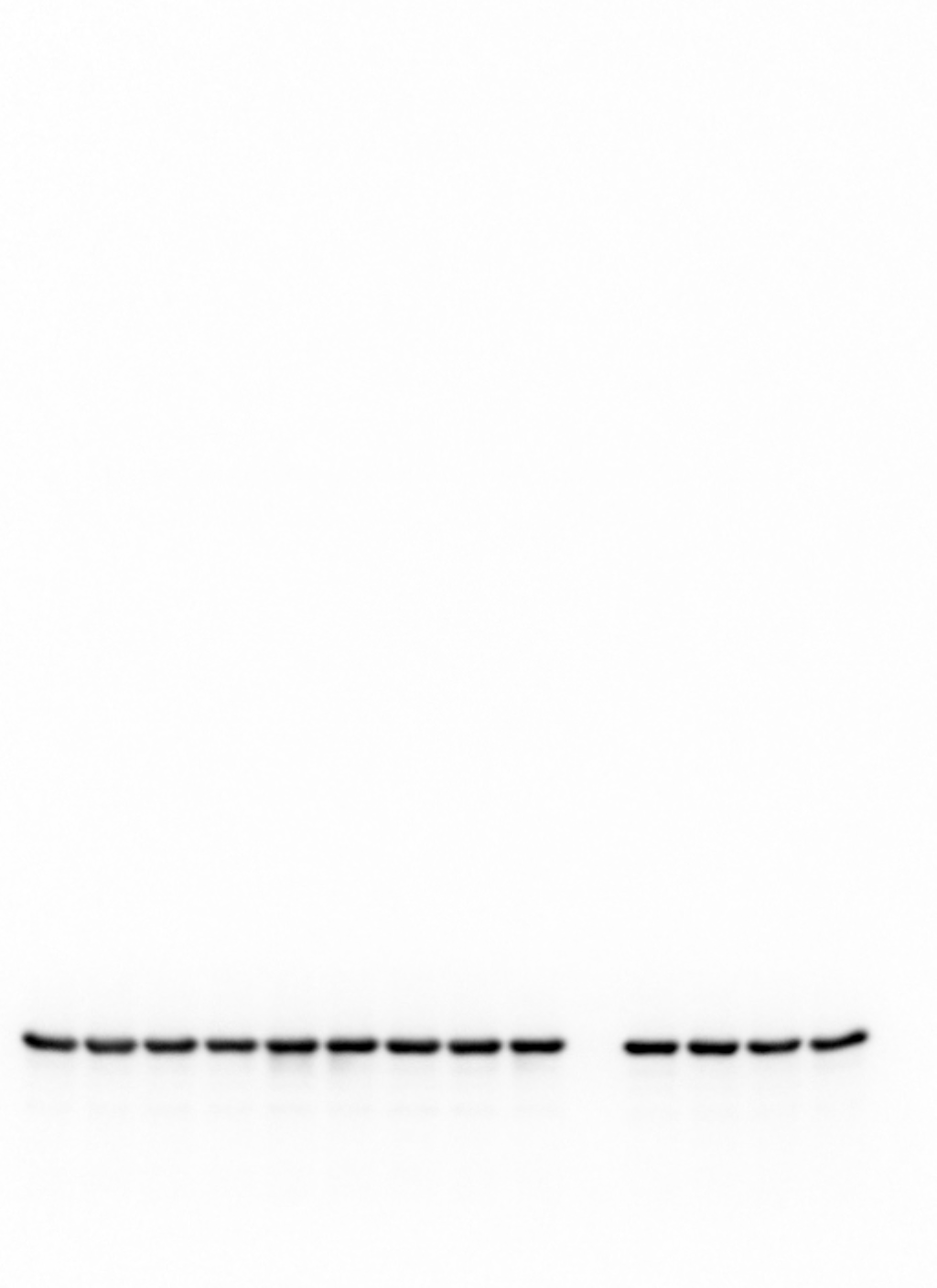

Supplement: Supplementary file 12 — Source data Fig. 7 [file 44321_2024_60_MOESM12_ESM.zip › Figure 7/7A/NU7441/GAPDH 0.1/1 GAP 0.1 _Ch.tif]

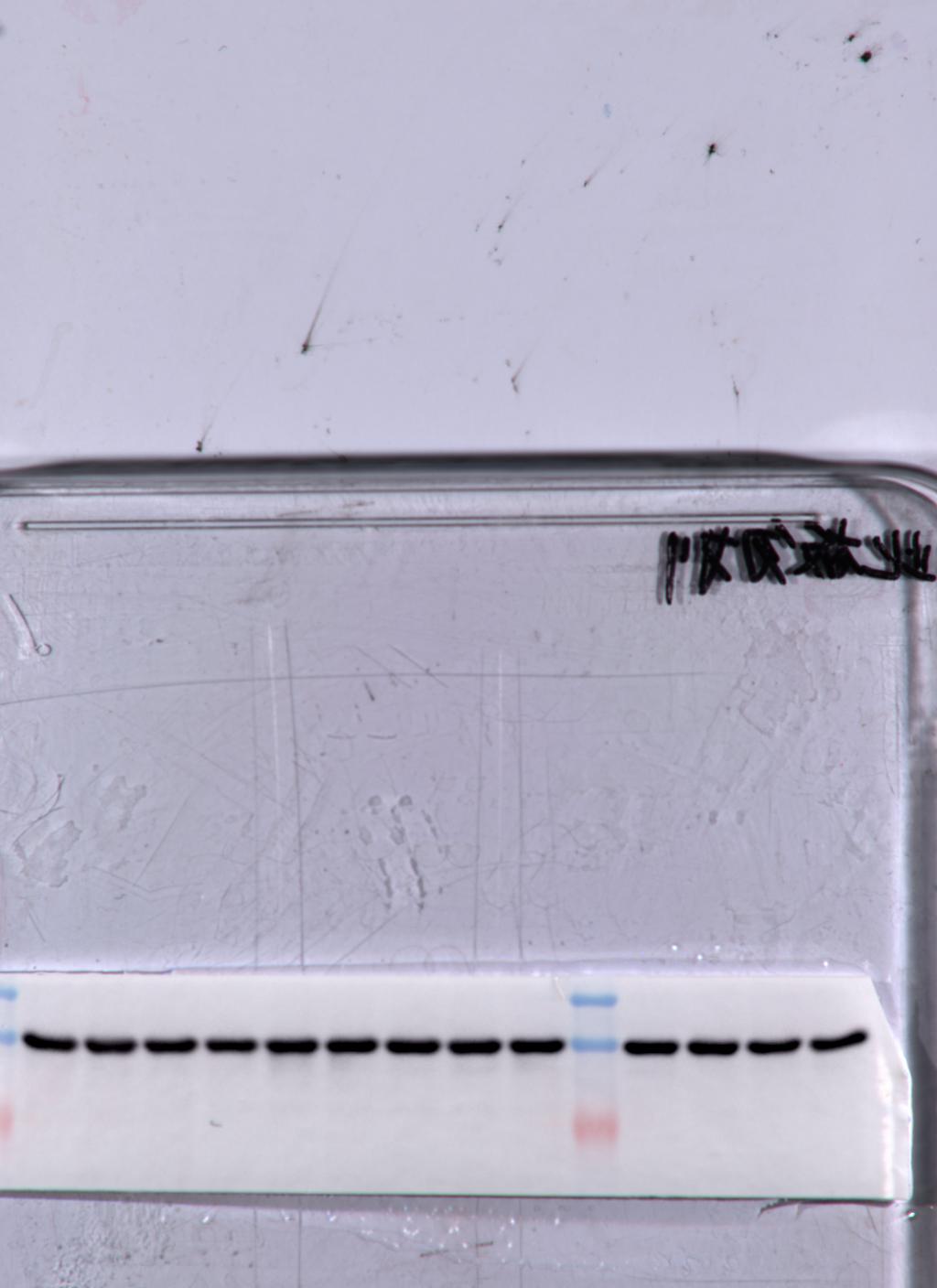

Supplement: Supplementary file 12 — Source data Fig. 7 [file 44321_2024_60_MOESM12_ESM.zip › Figure 7/7A/NU7441/GAPDH 0.1/1 GAP 0.1 _Ch+Marker.jpg]

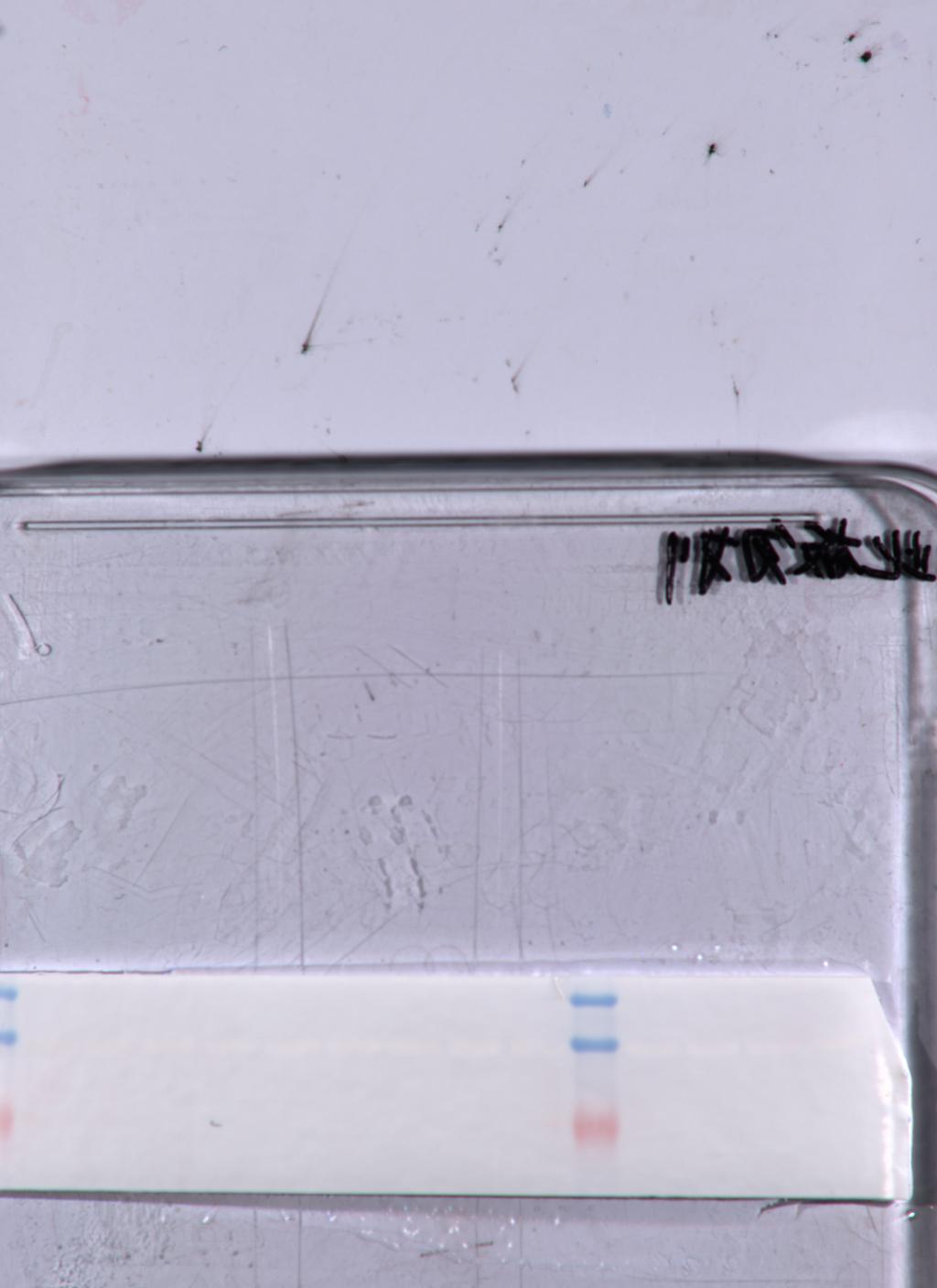

Supplement: Supplementary file 12 — Source data Fig. 7 [file 44321_2024_60_MOESM12_ESM.zip › Figure 7/7A/NU7441/GAPDH 0.1/1 GAP 0.1 _Ch-Marker.jpg]

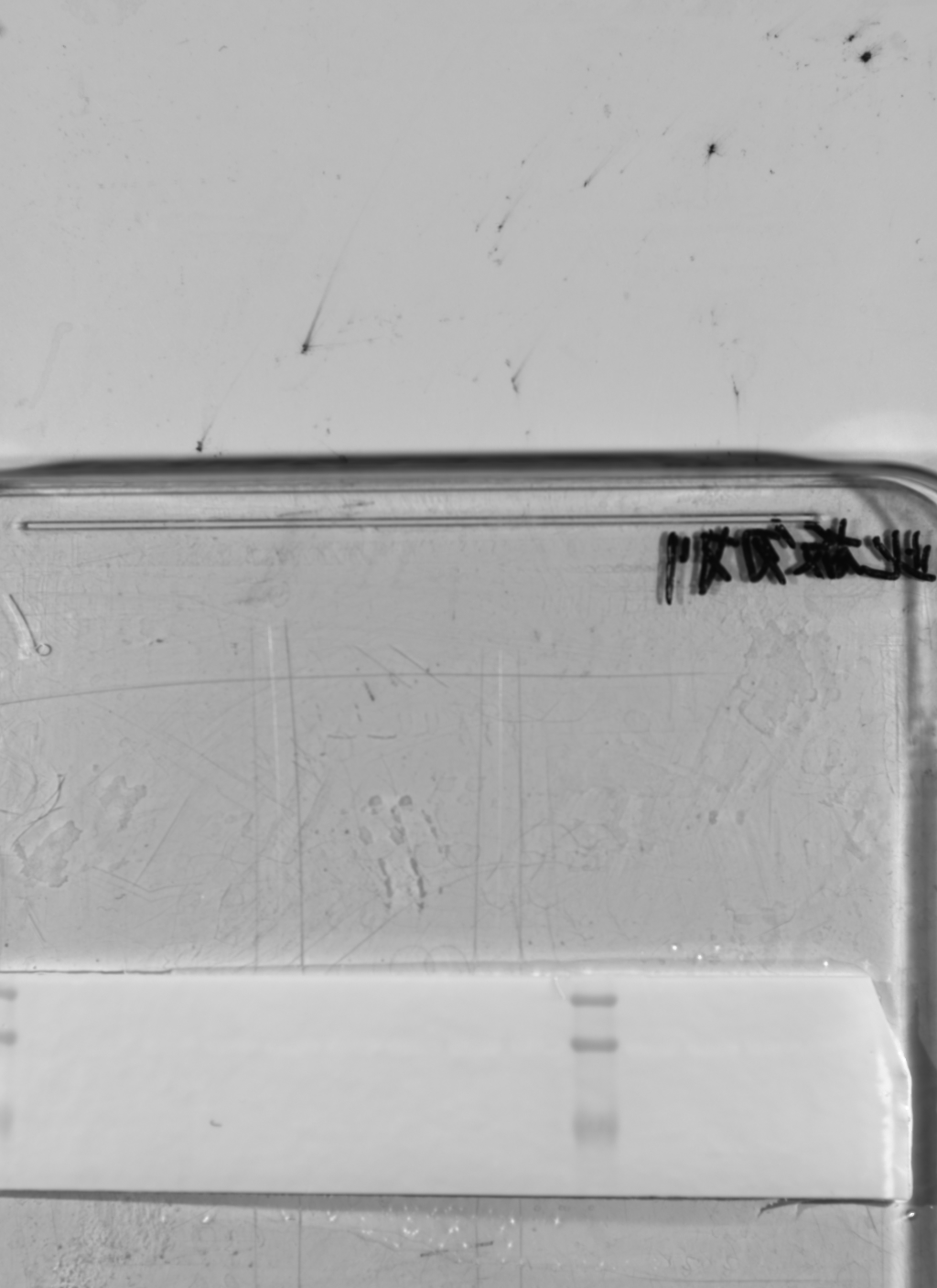

Supplement: Supplementary file 12 — Source data Fig. 7 [file 44321_2024_60_MOESM12_ESM.zip › Figure 7/7A/NU7441/GAPDH 0.1/1 GAP 0.1 _Ch-Marker.tif]

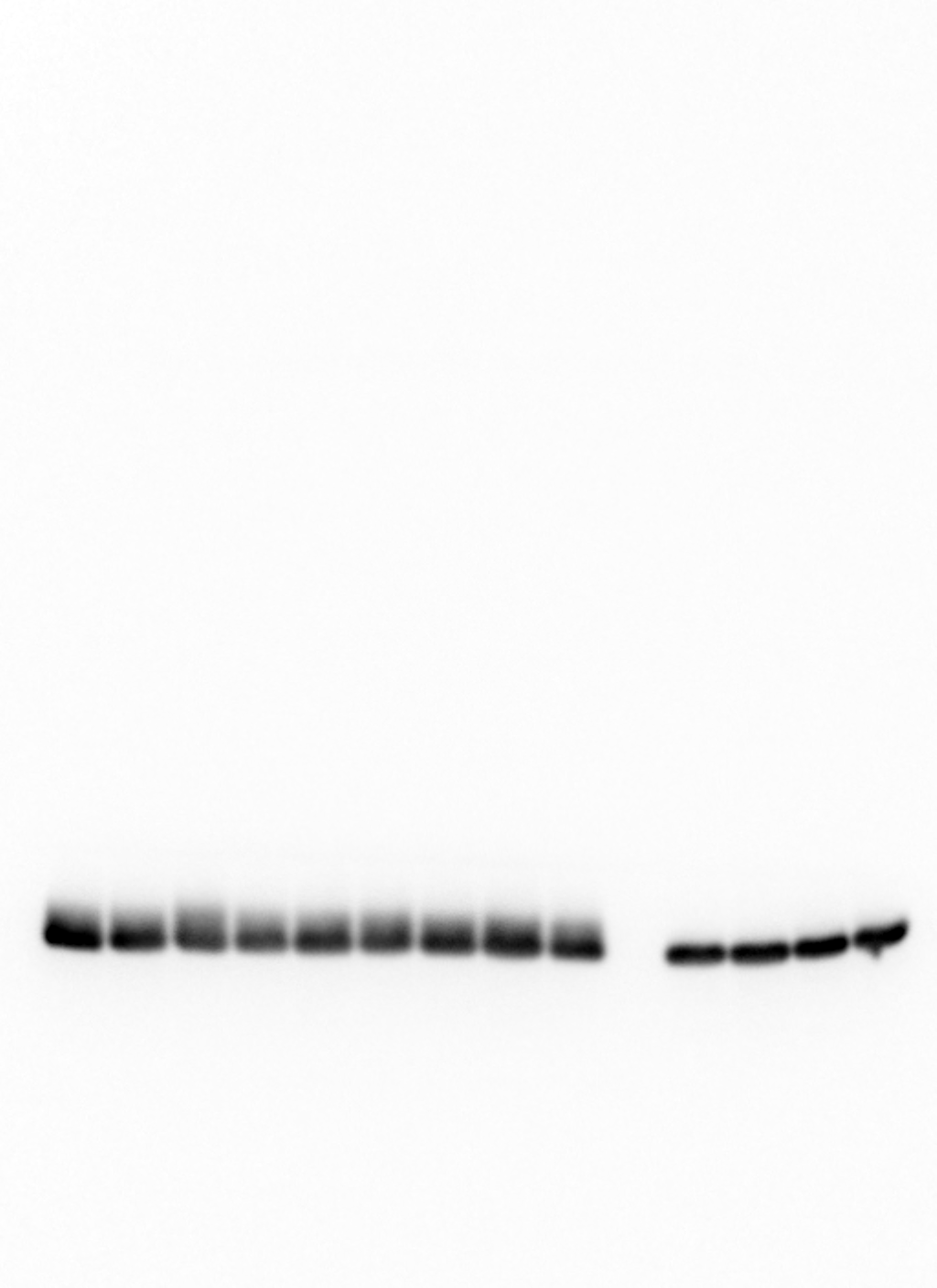

Supplement: Supplementary file 12 — Source data Fig. 7 [file 44321_2024_60_MOESM12_ESM.zip › Figure 7/7A/NU7441/H3 0.3/2 H3 1st 0.3 _Ch.tif]

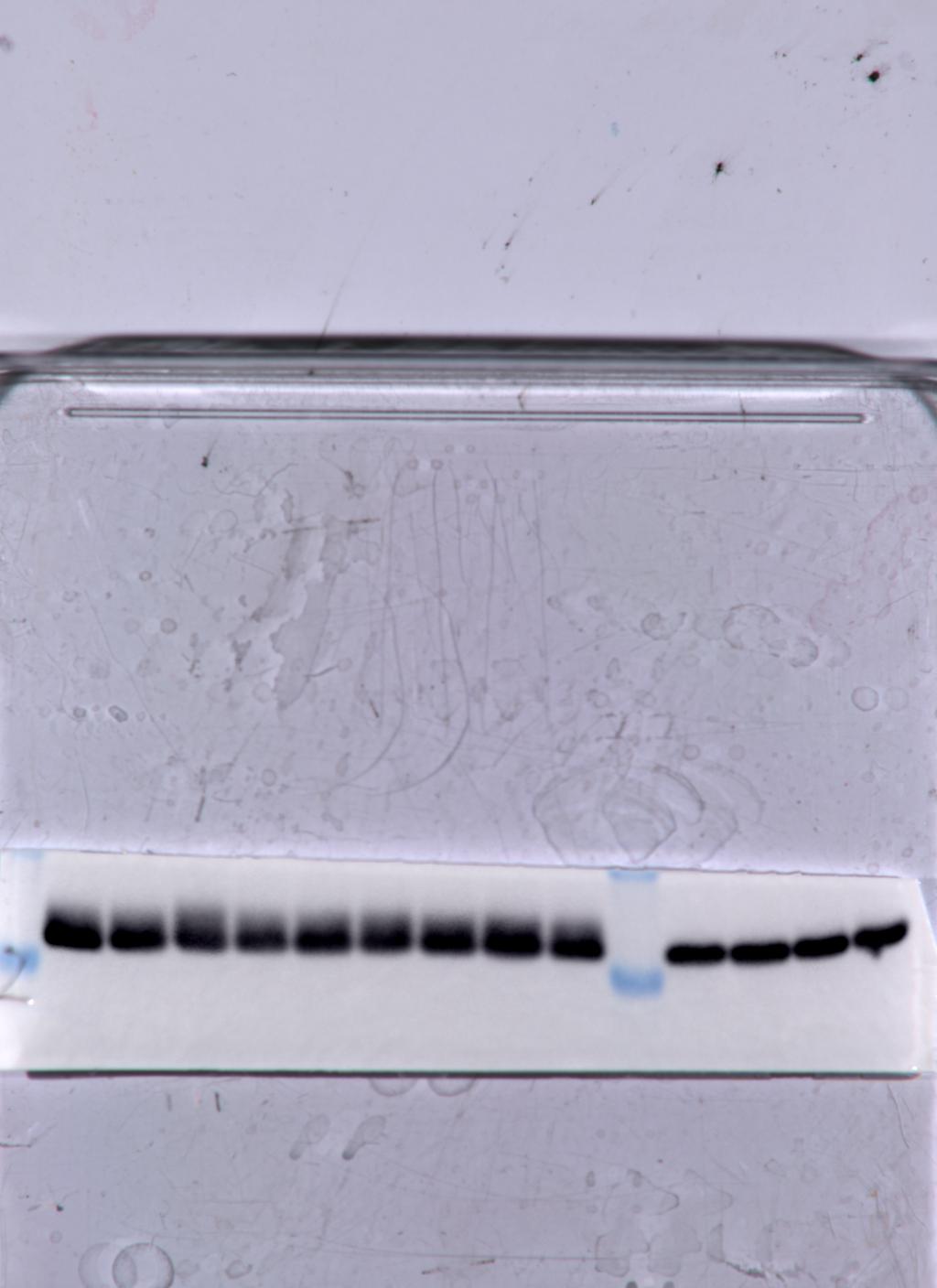

Supplement: Supplementary file 12 — Source data Fig. 7 [file 44321_2024_60_MOESM12_ESM.zip › Figure 7/7A/NU7441/H3 0.3/2 H3 1st 0.3 _Ch+Marker.jpg]

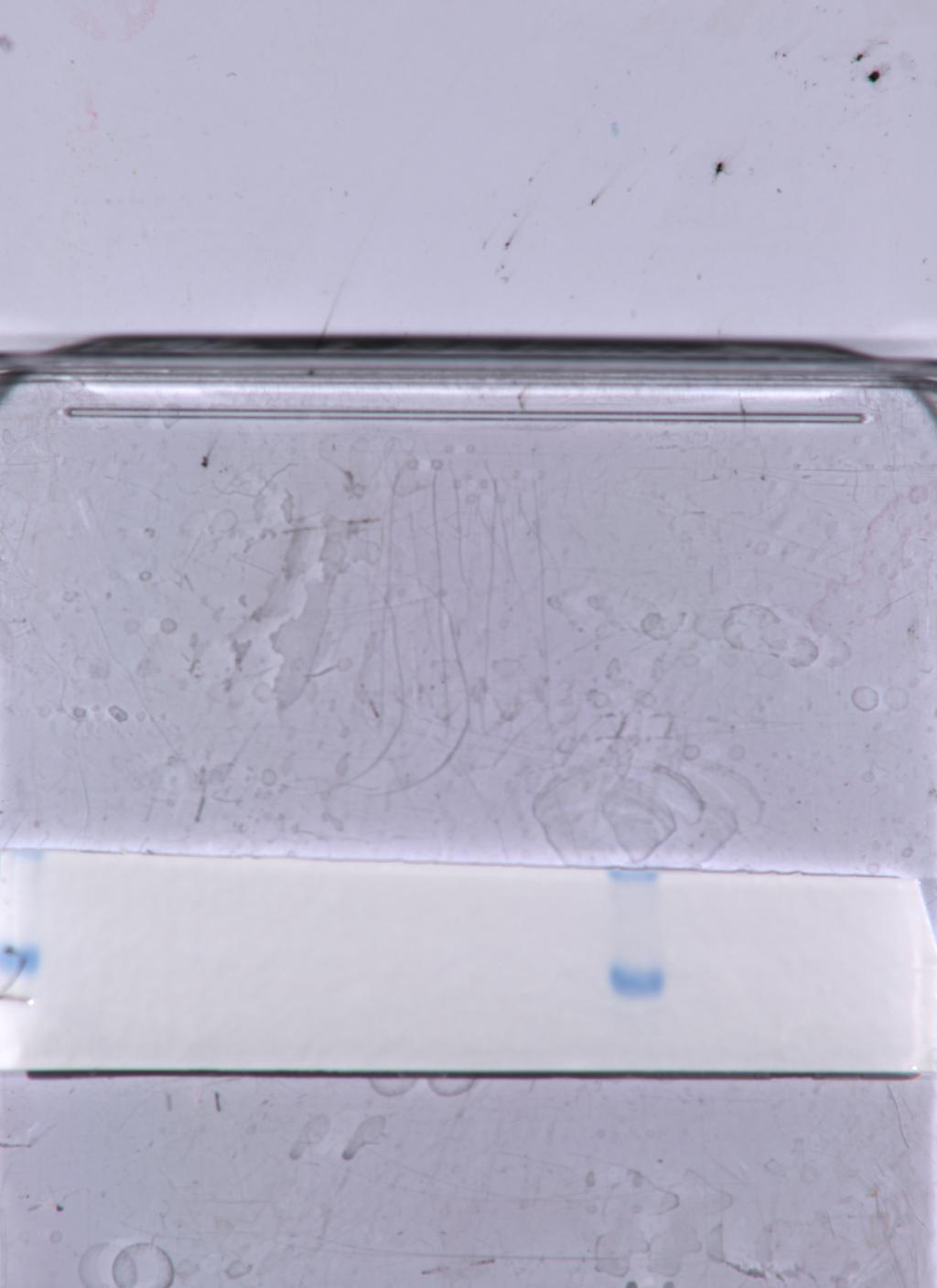

Supplement: Supplementary file 12 — Source data Fig. 7 [file 44321_2024_60_MOESM12_ESM.zip › Figure 7/7A/NU7441/H3 0.3/2 H3 1st 0.3 _Ch-Marker.jpg]

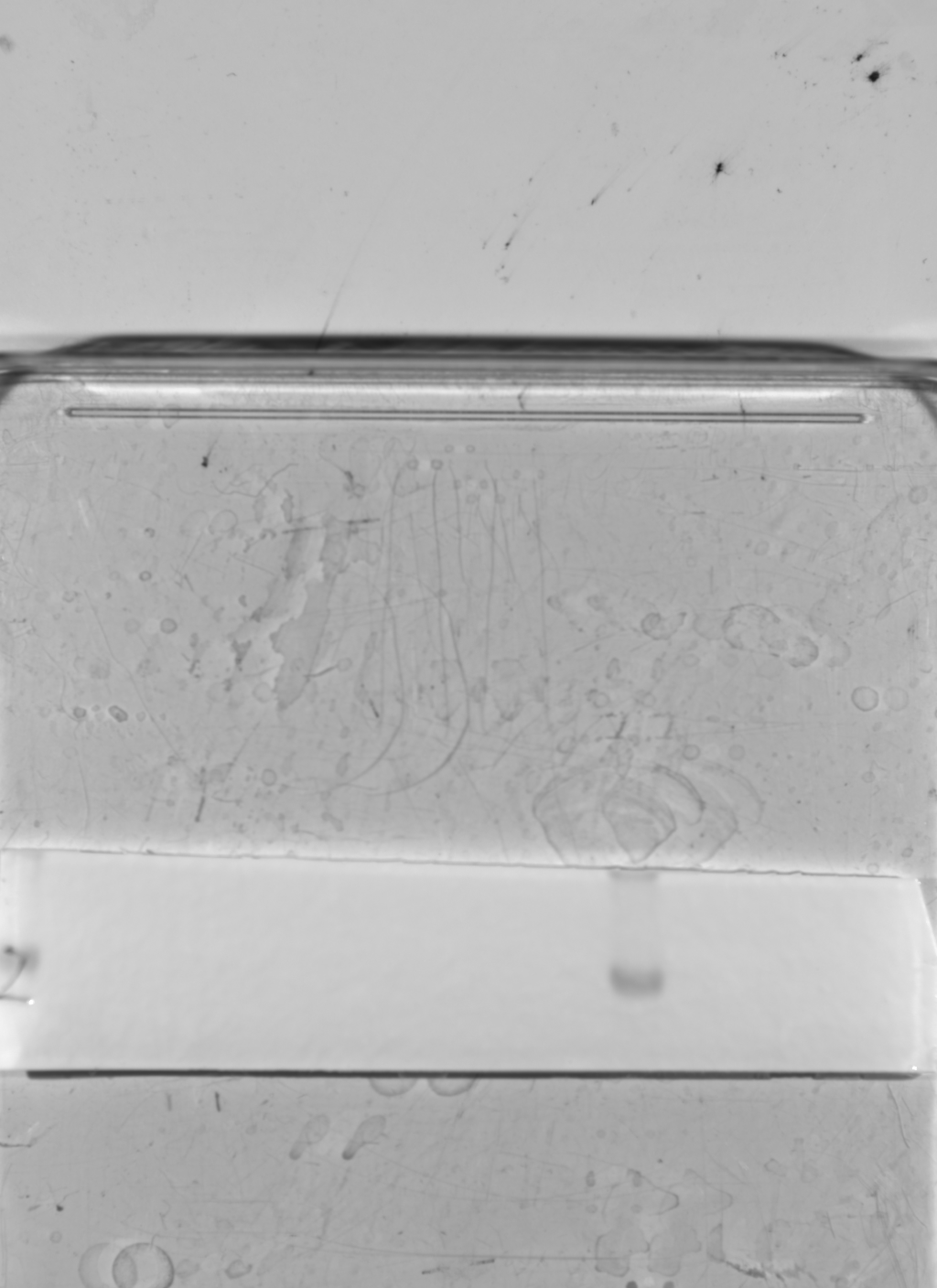

Supplement: Supplementary file 12 — Source data Fig. 7 [file 44321_2024_60_MOESM12_ESM.zip › Figure 7/7A/NU7441/H3 0.3/2 H3 1st 0.3 _Ch-Marker.tif]

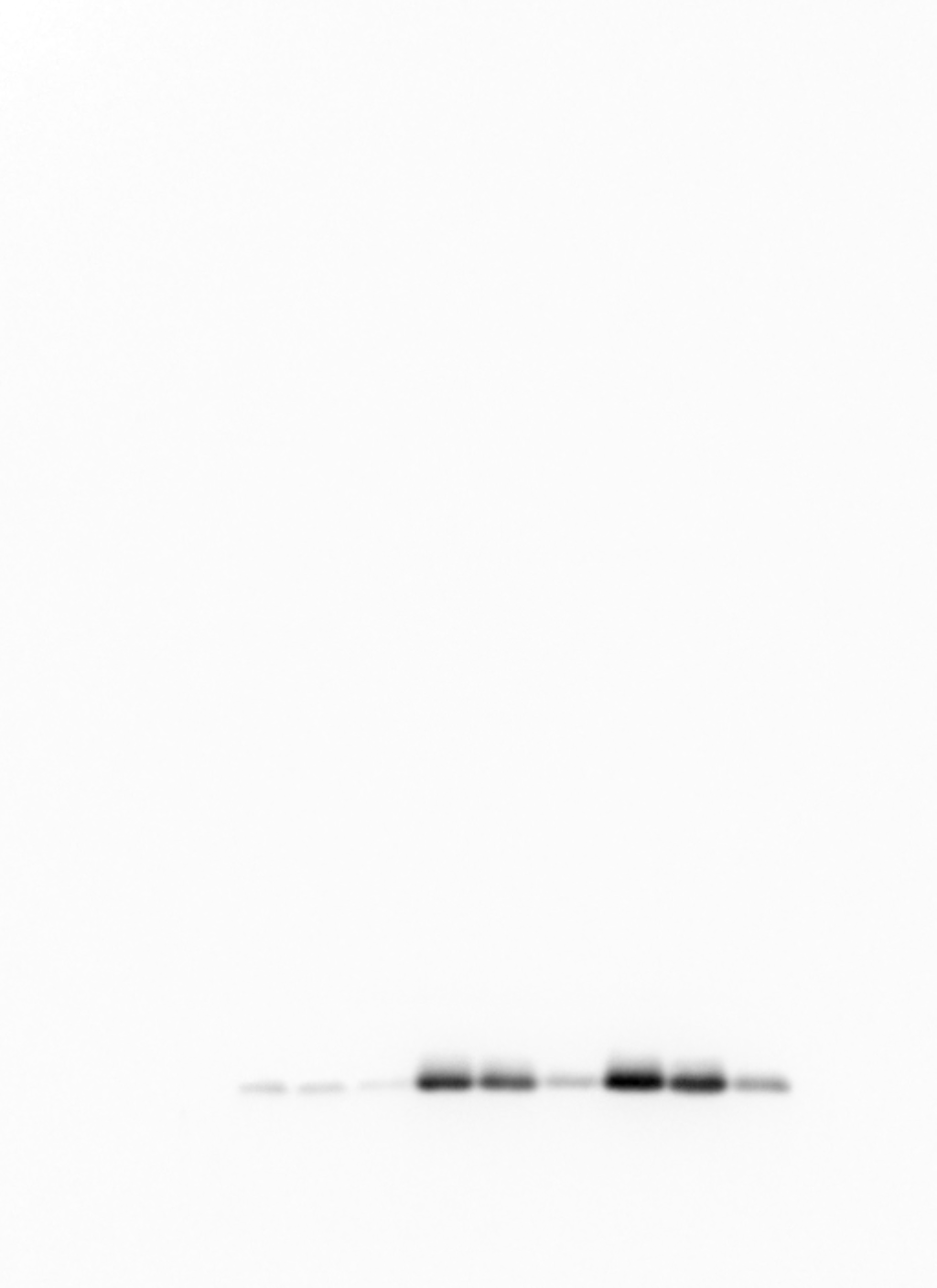

Supplement: Supplementary file 12 — Source data Fig. 7 [file 44321_2024_60_MOESM12_ESM.zip › Figure 7/7A/NU7441/rH2A 0.9/1-1 rH2A 1st 0.9 _Ch.tif]

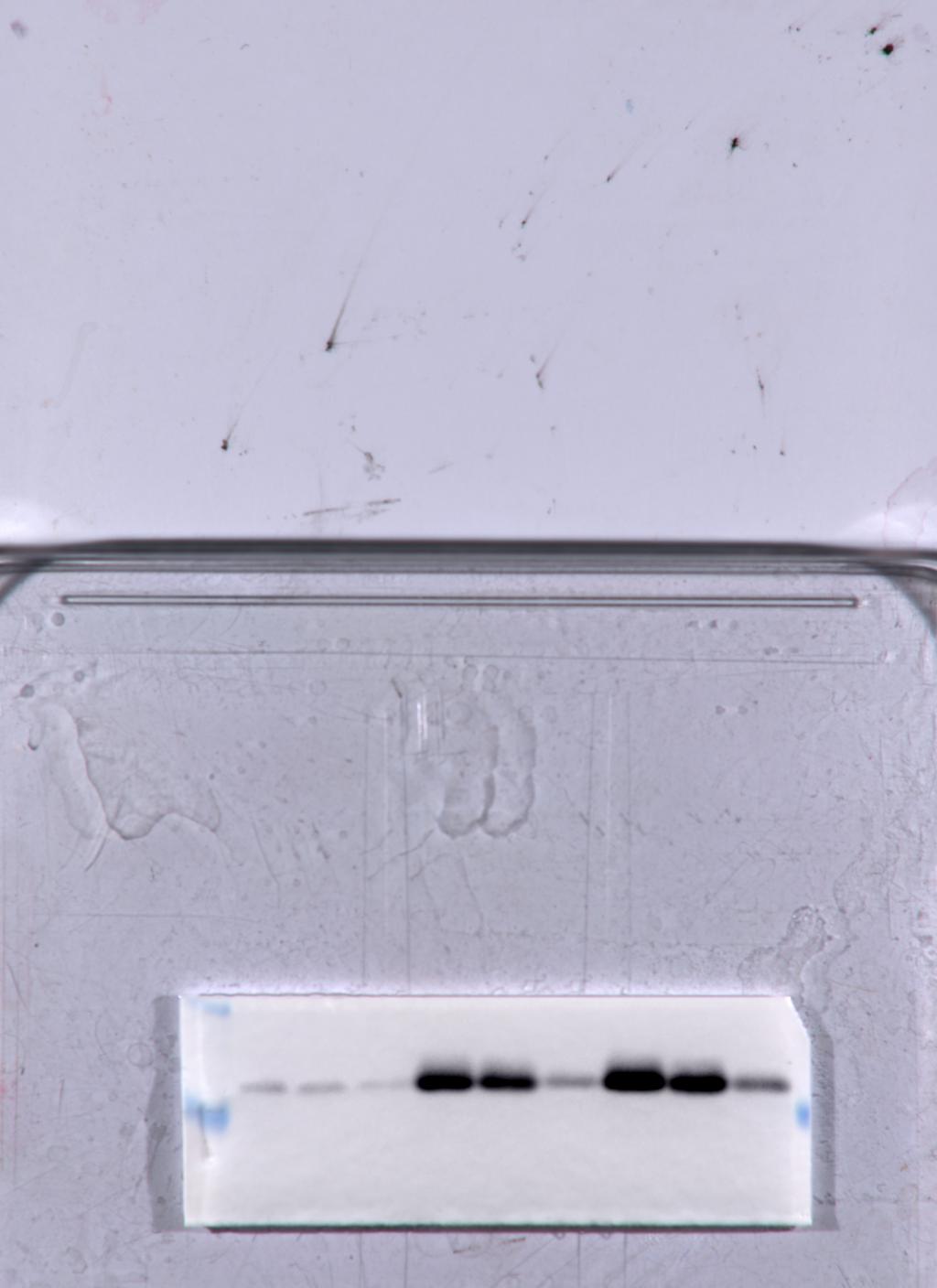

Supplement: Supplementary file 12 — Source data Fig. 7 [file 44321_2024_60_MOESM12_ESM.zip › Figure 7/7A/NU7441/rH2A 0.9/1-1 rH2A 1st 0.9 _Ch+Marker.jpg]

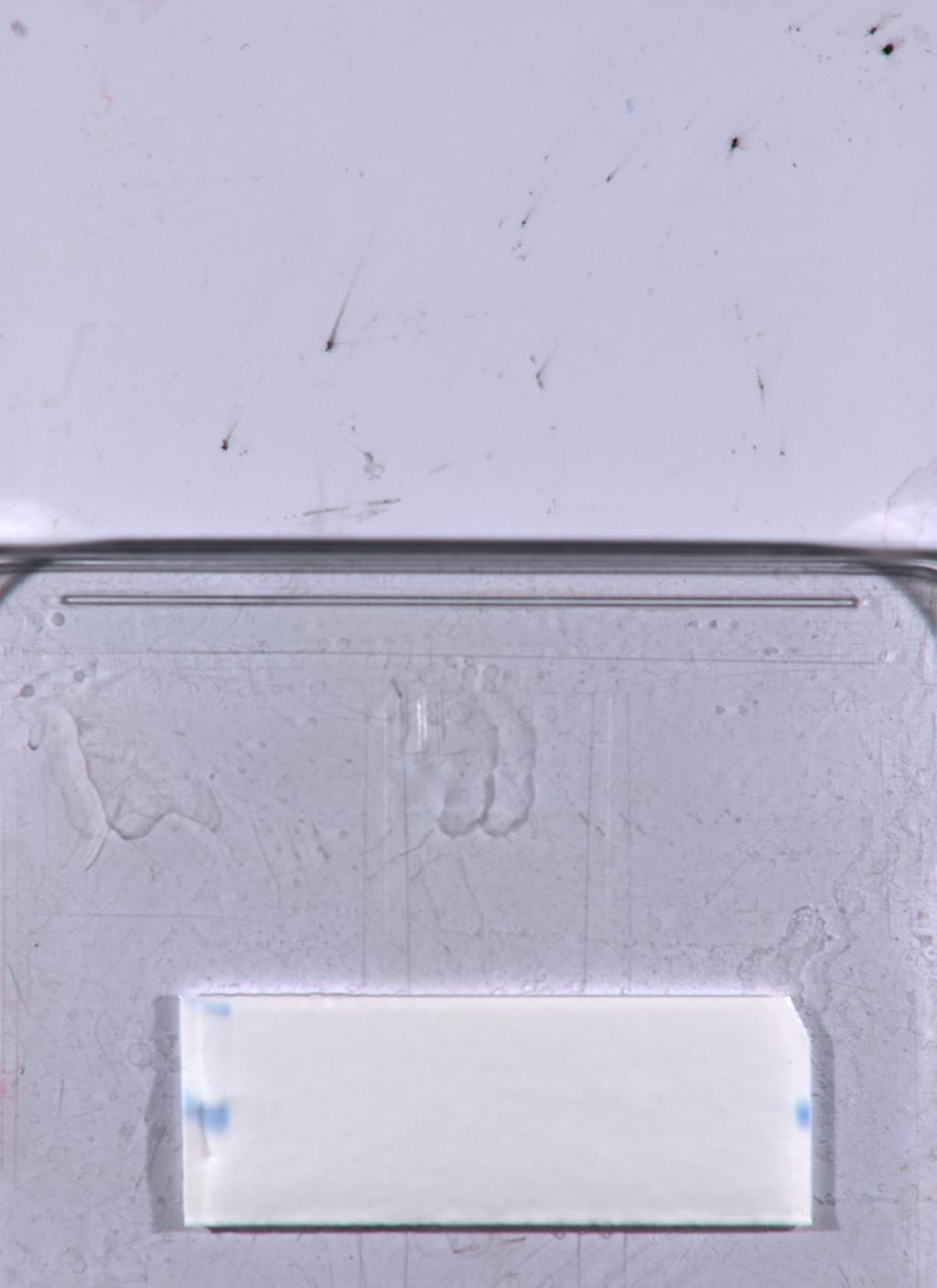

Supplement: Supplementary file 12 — Source data Fig. 7 [file 44321_2024_60_MOESM12_ESM.zip › Figure 7/7A/NU7441/rH2A 0.9/1-1 rH2A 1st 0.9 _Ch-Marker.jpg]

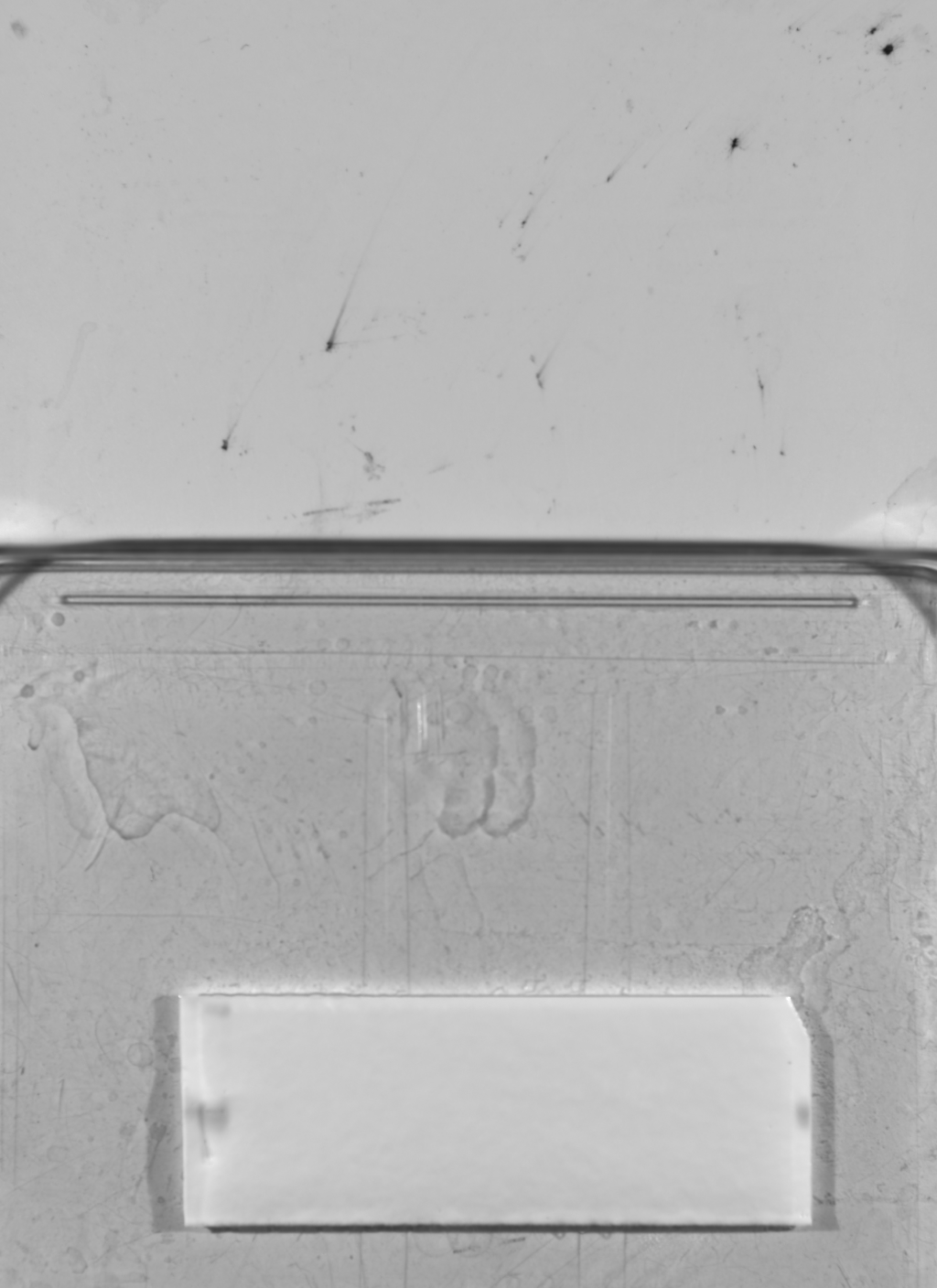

Supplement: Supplementary file 12 — Source data Fig. 7 [file 44321_2024_60_MOESM12_ESM.zip › Figure 7/7A/NU7441/rH2A 0.9/1-1 rH2A 1st 0.9 _Ch-Marker.tif]

## Slide 1
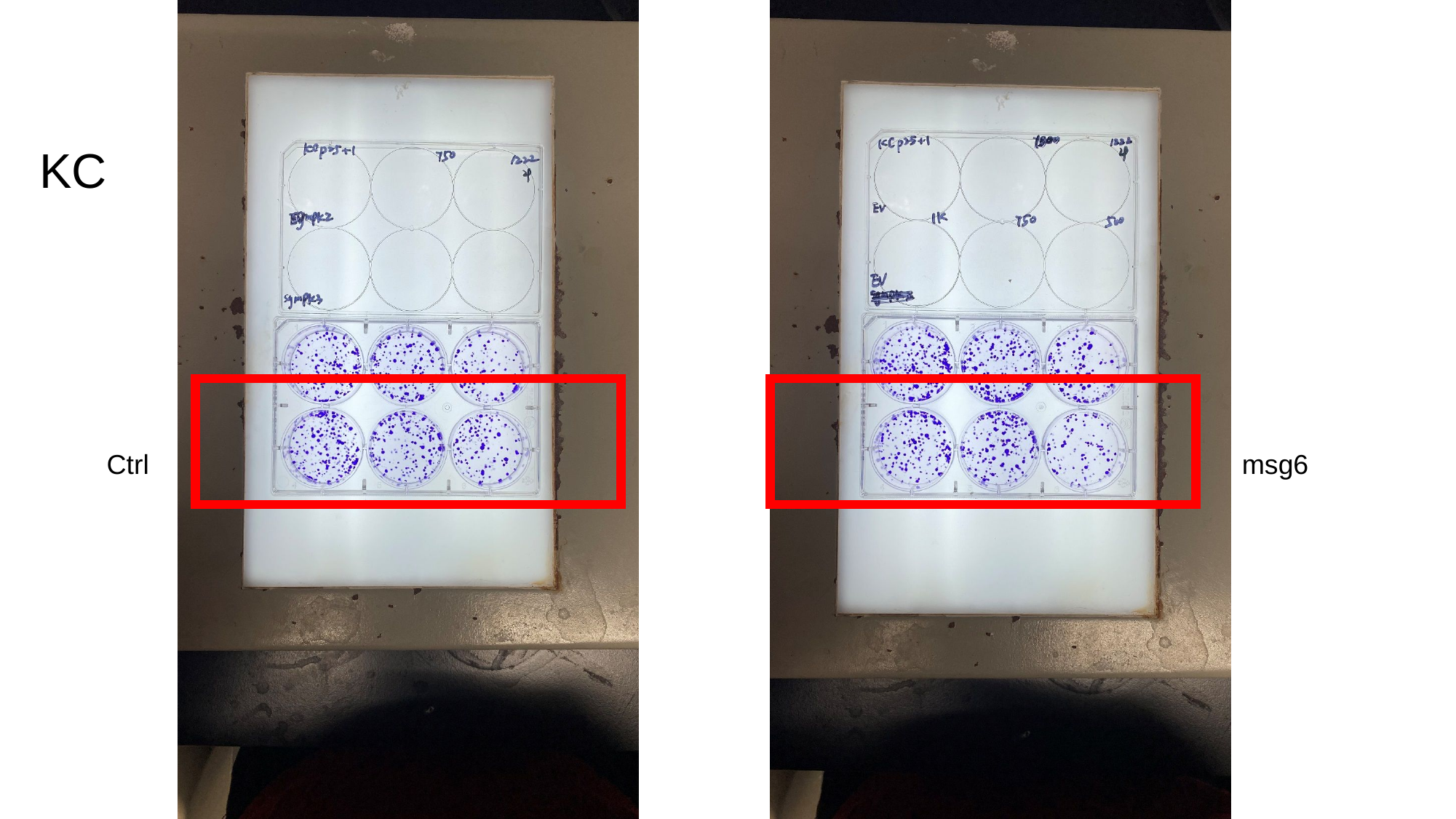

KC
Ctrl
msg6

## Slide 2
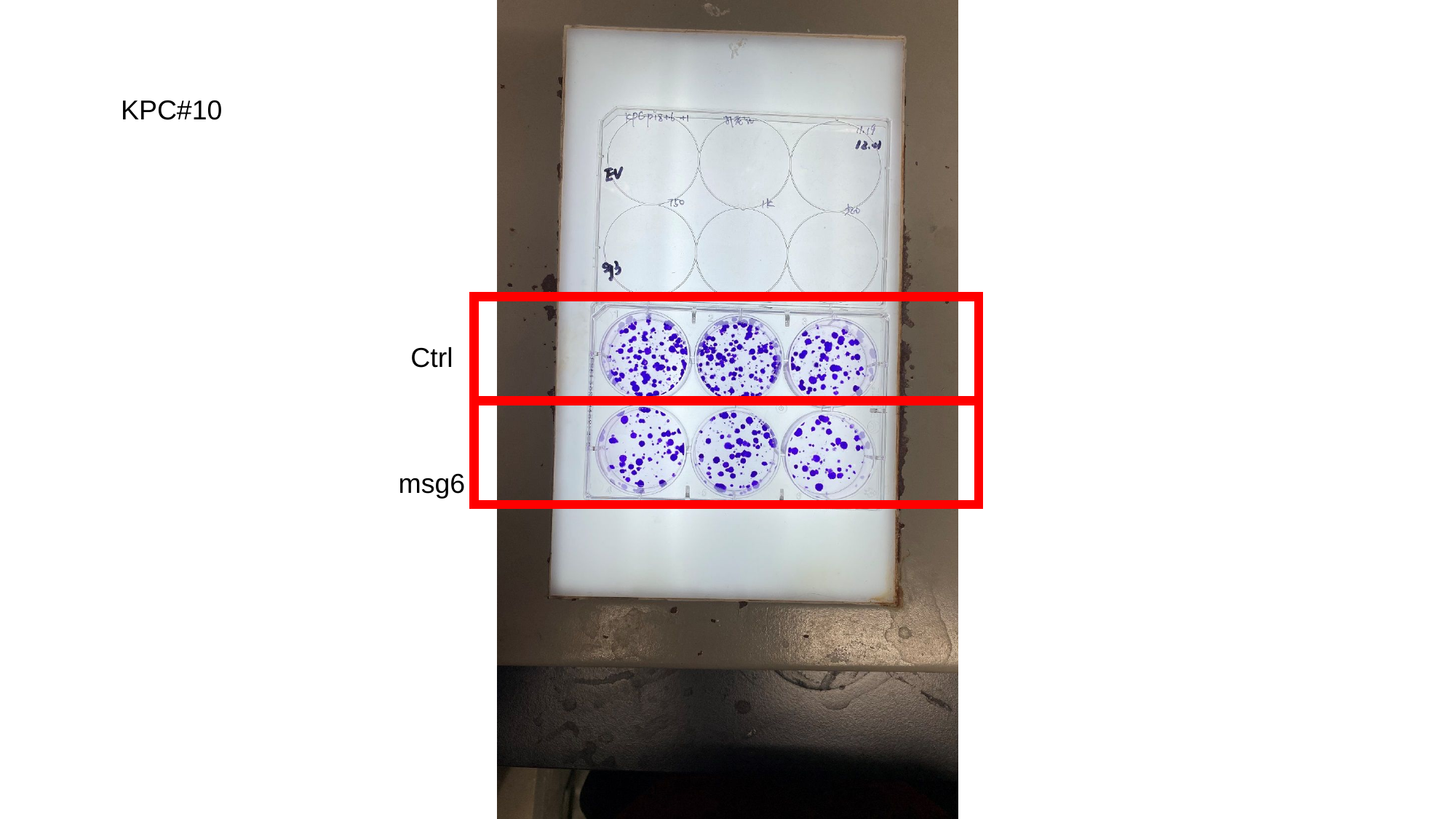

KPC#10
Ctrl
msg6

Supplement: Supplementary file 13 — Source data Fig. 8 [file 44321_2024_60_MOESM13_ESM.zip › Figure 8/8E/8E.pptx]

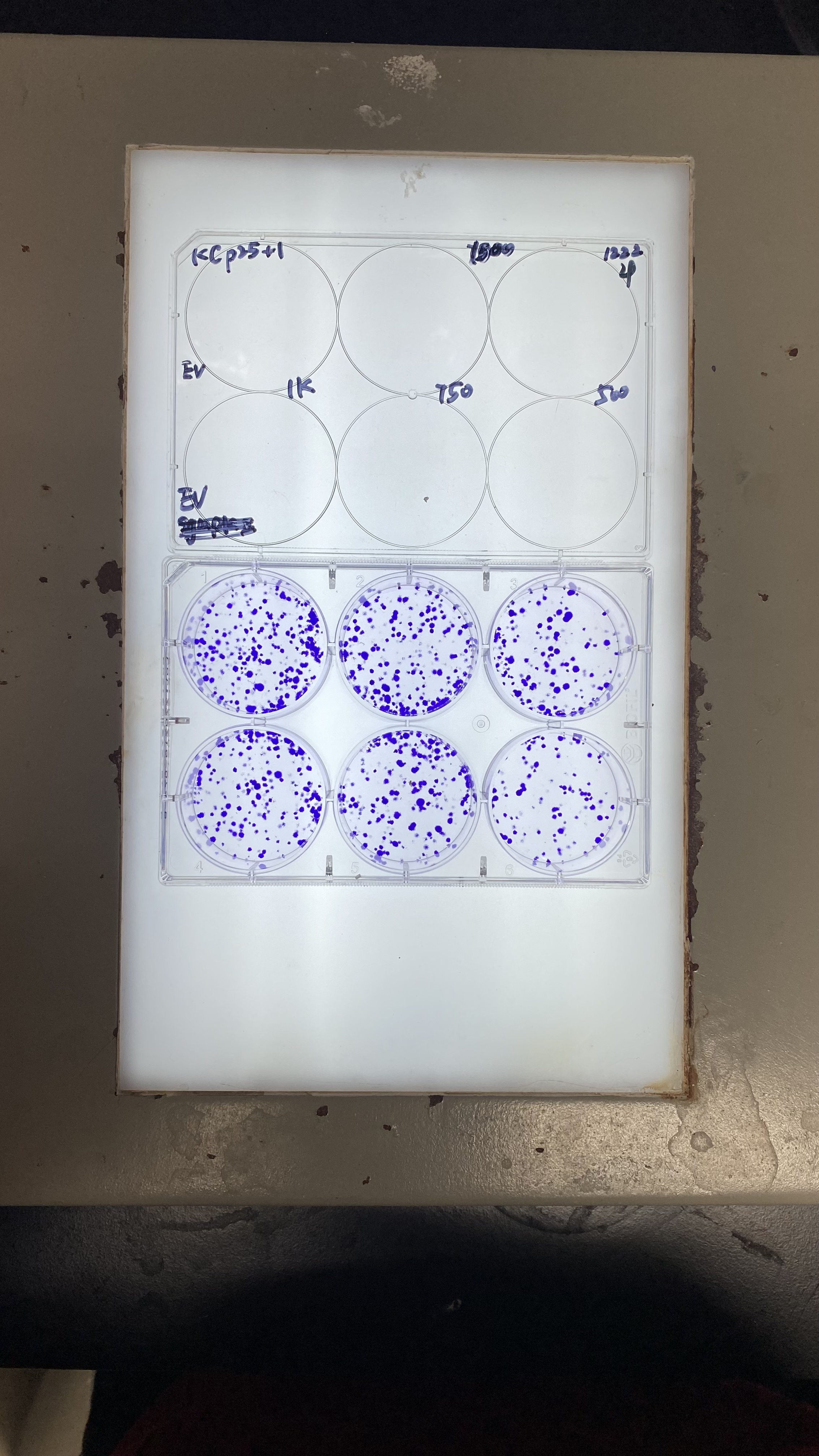

Supplement: Supplementary file 13 — Source data Fig. 8 [file 44321_2024_60_MOESM13_ESM.zip › Figure 8/8E/KC/EV.jpg]

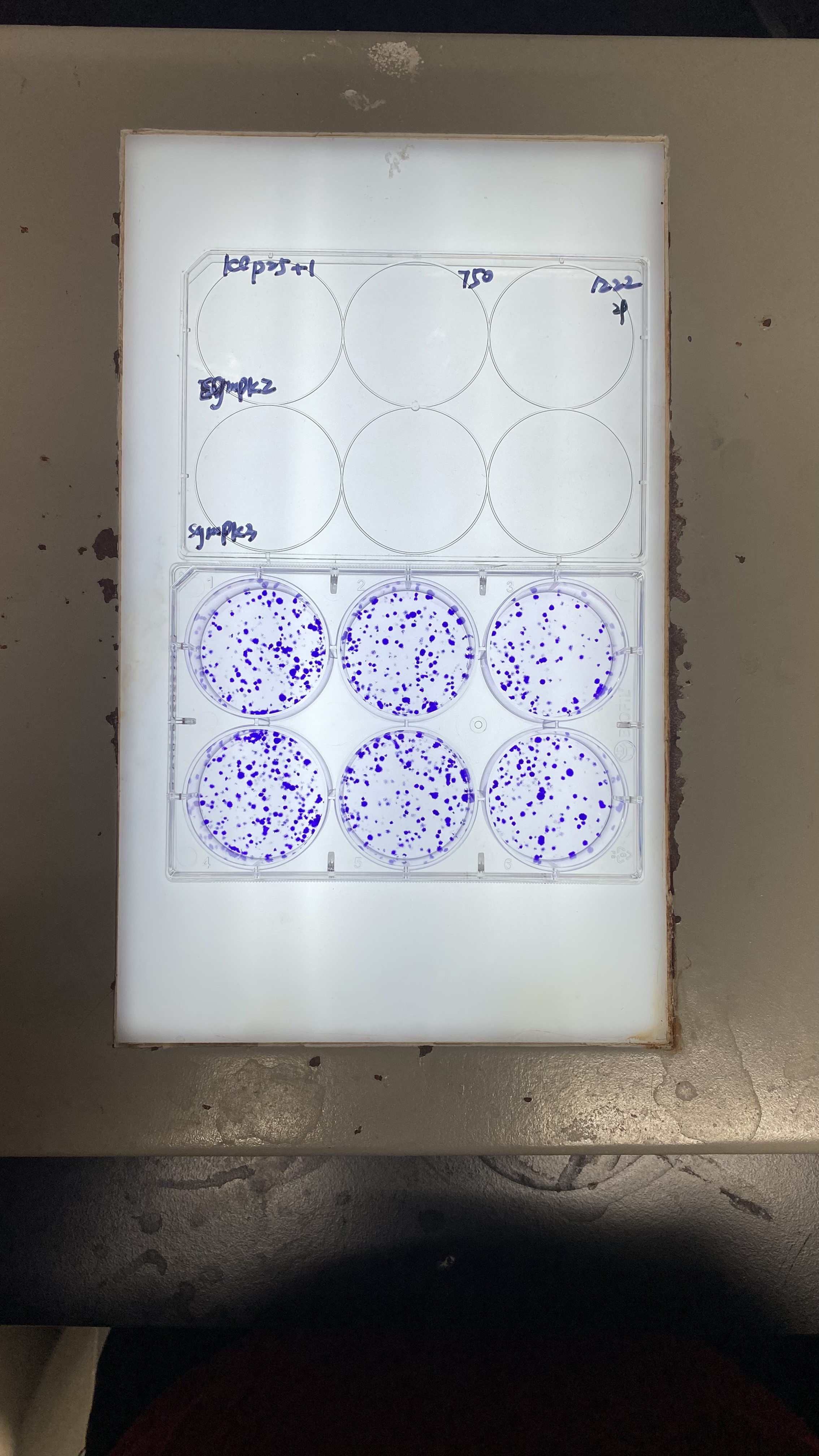

Supplement: Supplementary file 13 — Source data Fig. 8 [file 44321_2024_60_MOESM13_ESM.zip › Figure 8/8E/KC/sgPK 2 sgPK3.jpg]

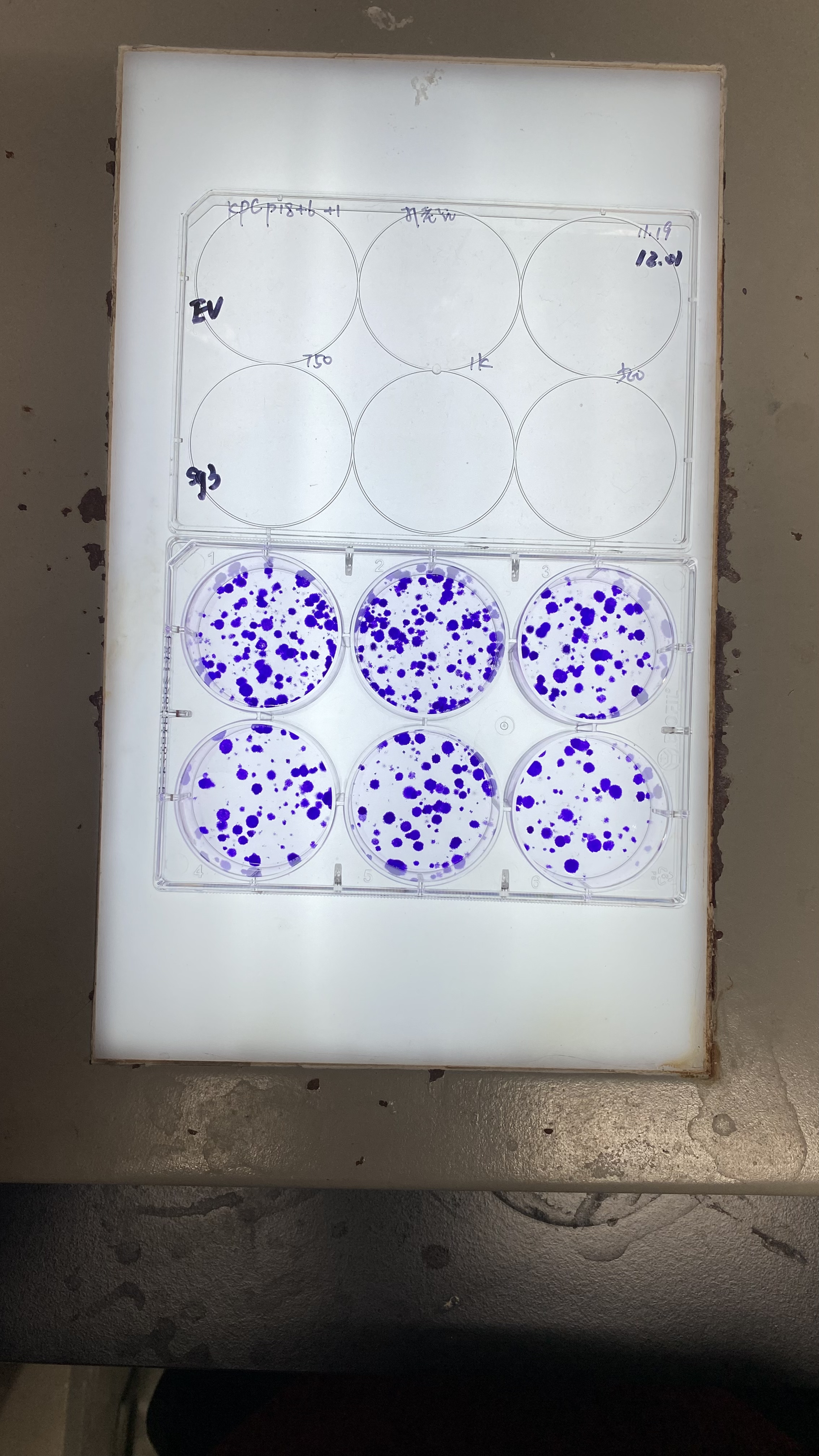

Supplement: Supplementary file 13 — Source data Fig. 8 [file 44321_2024_60_MOESM13_ESM.zip › Figure 8/8E/KPC#10/EV sg3.jpg]

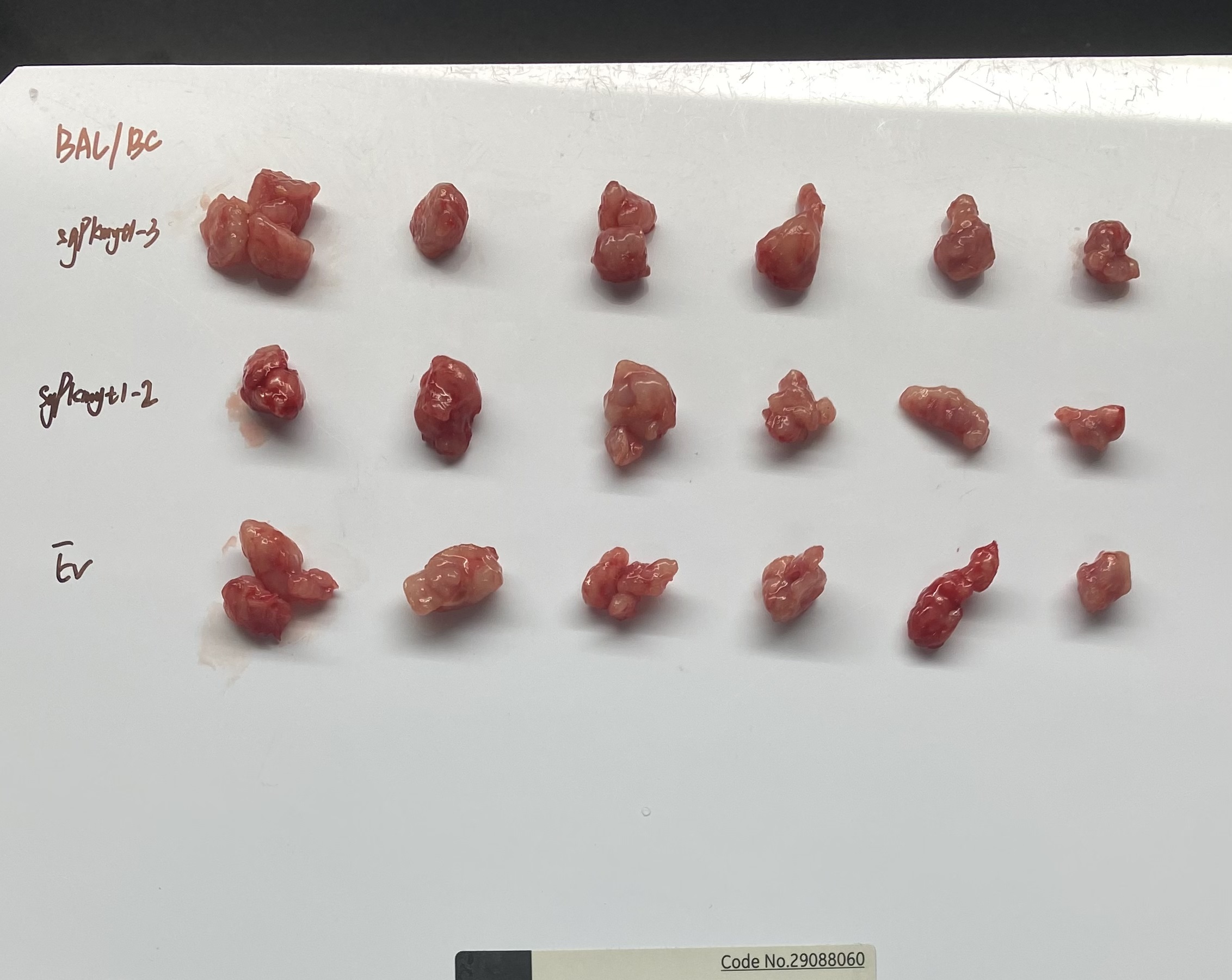

Supplement: Supplementary file 13 — Source data Fig. 8 [file 44321_2024_60_MOESM13_ESM.zip › Figure 8/8I/KC.jpg]

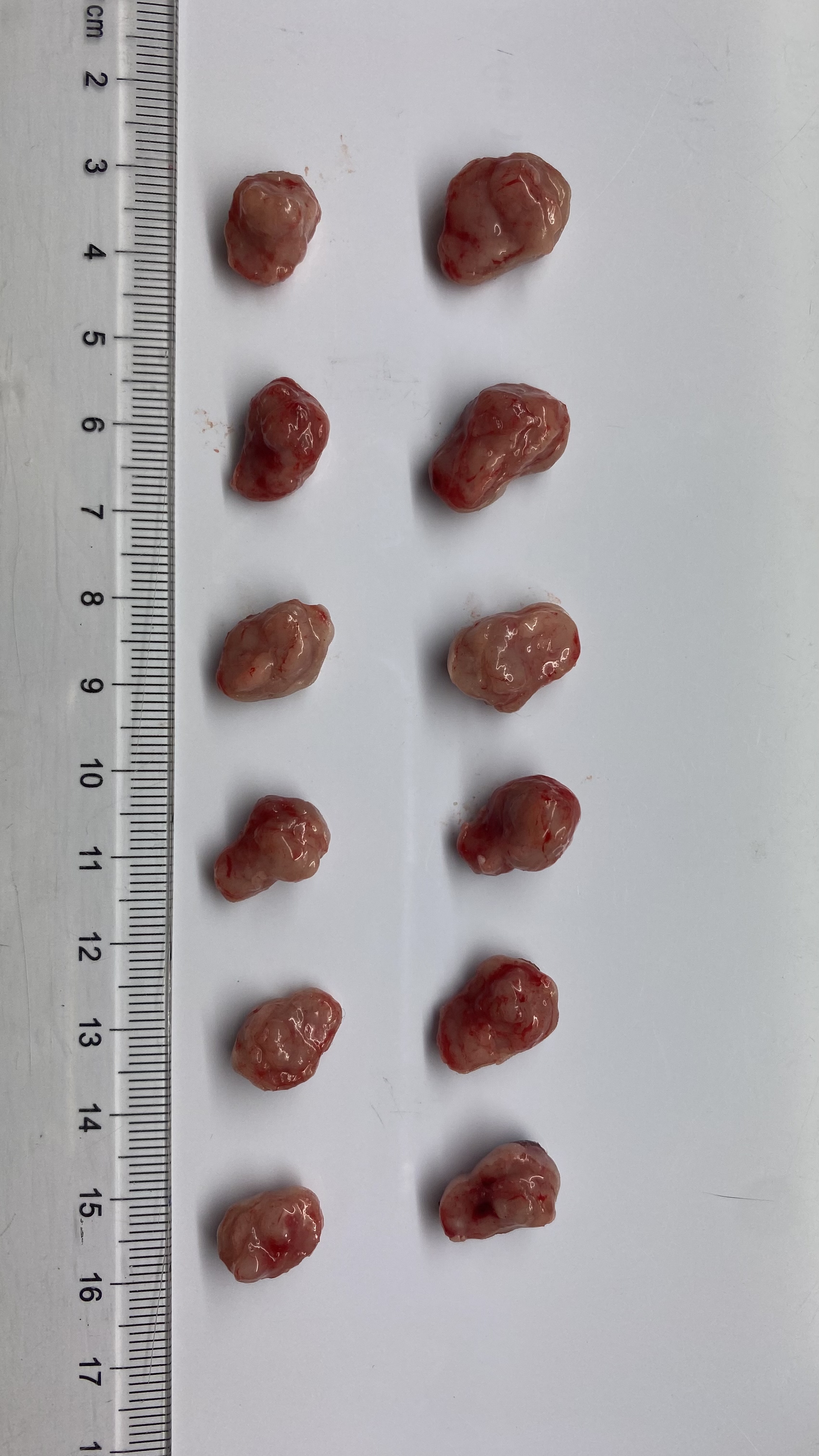

Supplement: Supplementary file 13 — Source data Fig. 8 [file 44321_2024_60_MOESM13_ESM.zip › Figure 8/8I/KPC#10.jpg]

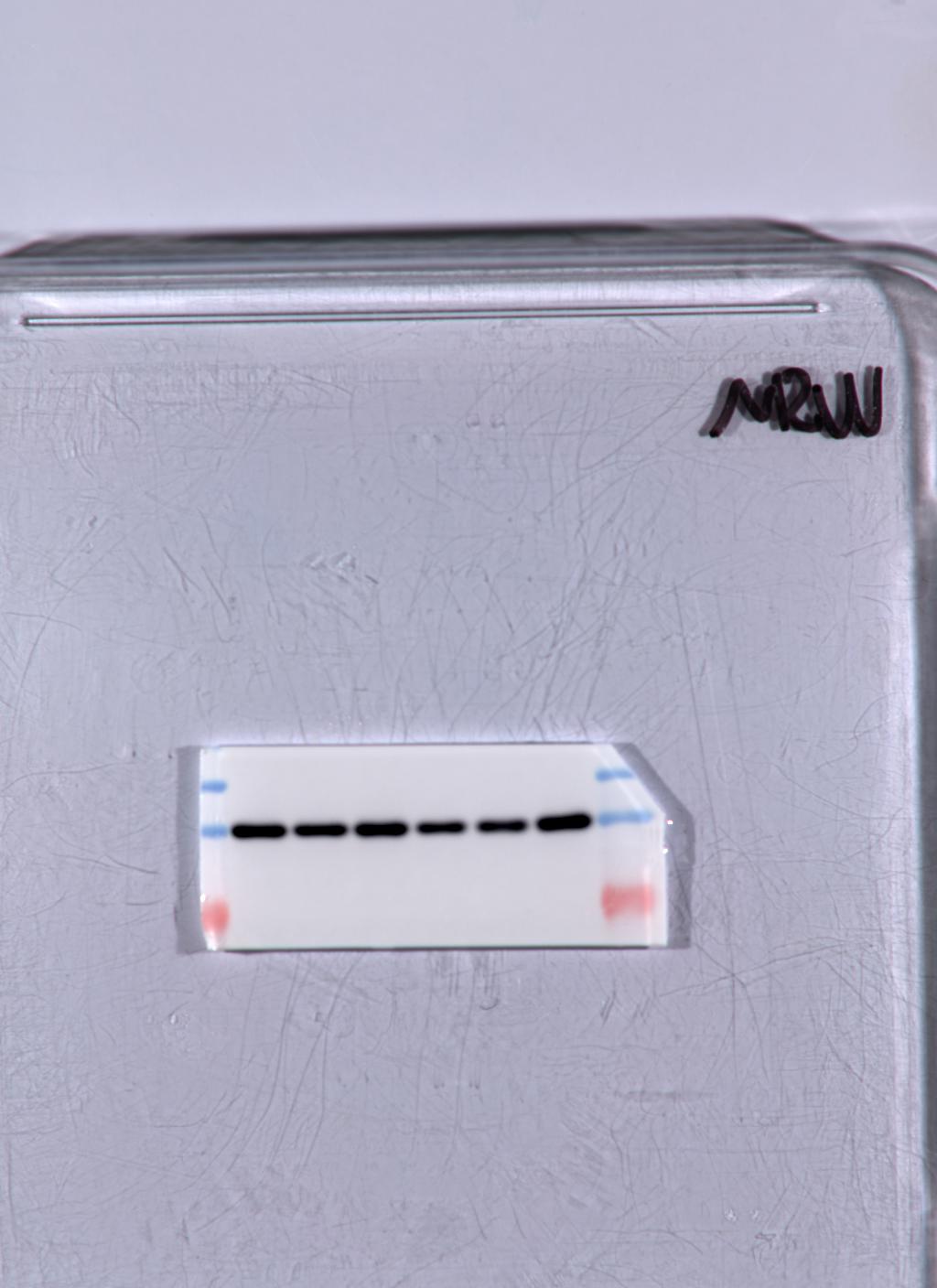

Supplement: Supplementary file 14 — EV Figure Source Data [file 44321_2024_60_MOESM14_ESM.zip › Figure EV2C Source Data/EV2C/88T YAPC/88T-Western GAP 0.1/2 GAP 0.1 _Ch+Marker.jpg]

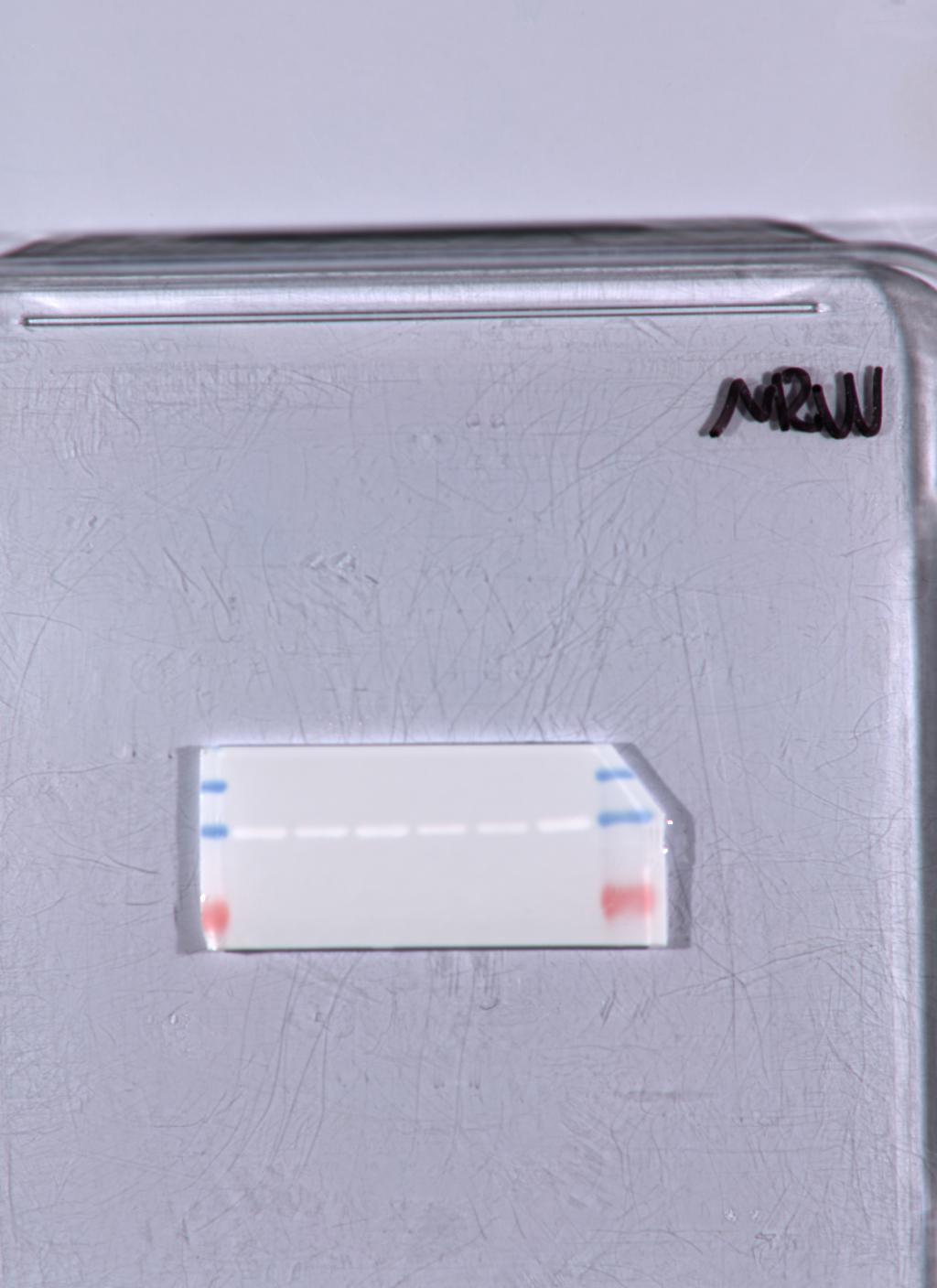

Supplement: Supplementary file 14 — EV Figure Source Data [file 44321_2024_60_MOESM14_ESM.zip › Figure EV2C Source Data/EV2C/88T YAPC/88T-Western GAP 0.1/2 GAP 0.1 _Ch-Marker.jpg]

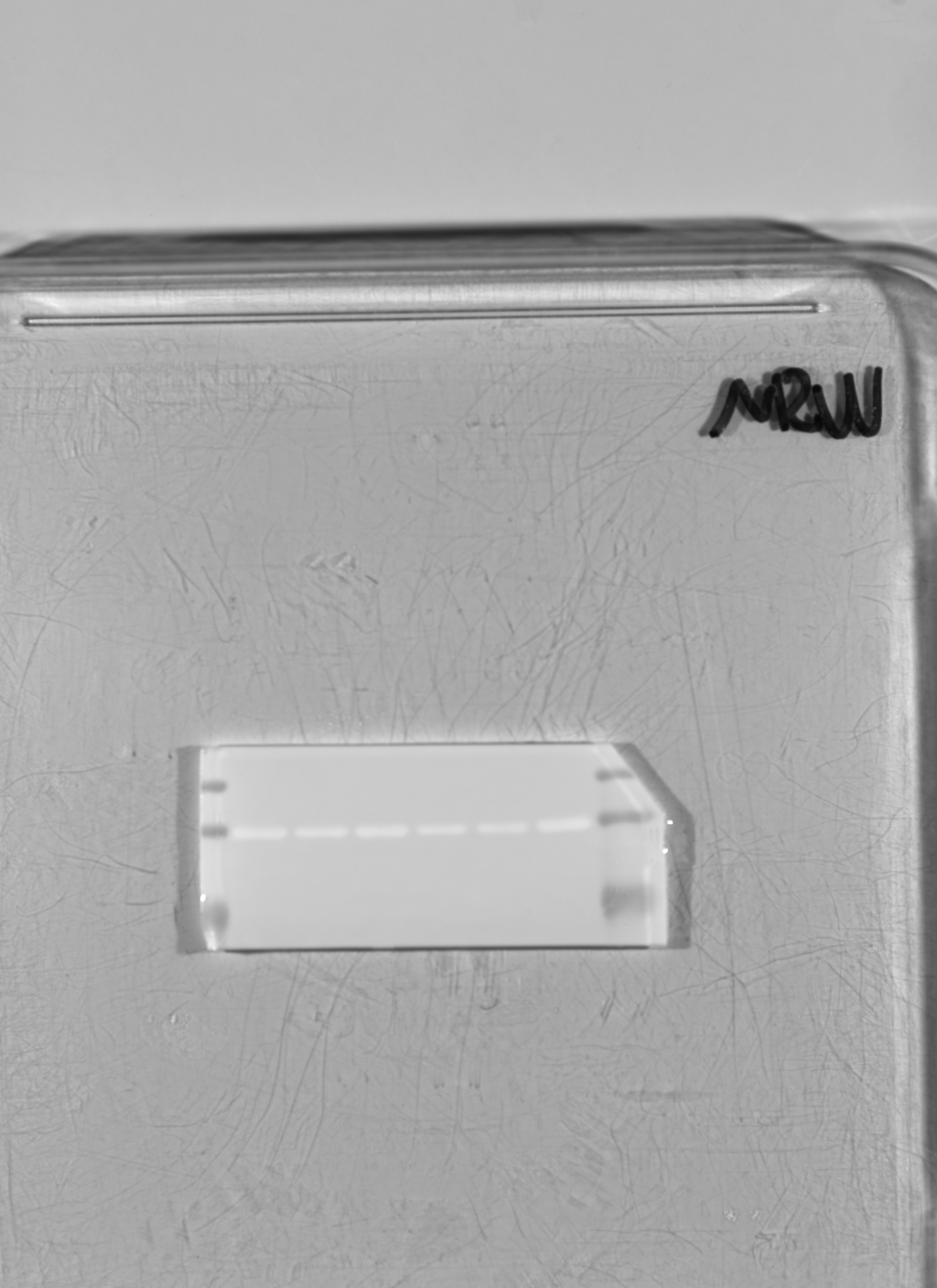

Supplement: Supplementary file 14 — EV Figure Source Data [file 44321_2024_60_MOESM14_ESM.zip › Figure EV2C Source Data/EV2C/88T YAPC/88T-Western GAP 0.1/2 GAP 0.1 _Ch-Marker.tif]

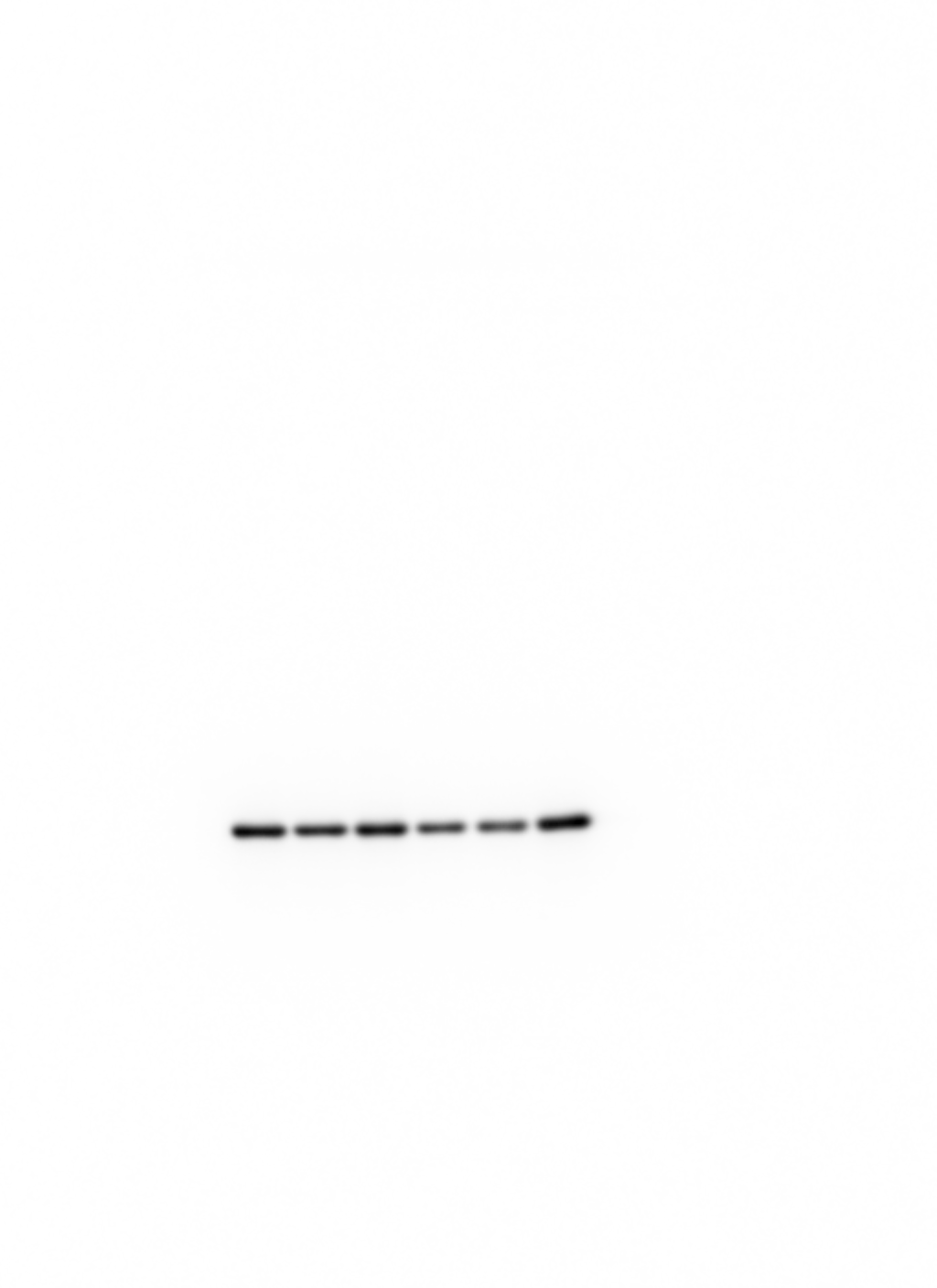

Supplement: Supplementary file 14 — EV Figure Source Data [file 44321_2024_60_MOESM14_ESM.zip › Figure EV2C Source Data/EV2C/88T YAPC/88T-Western GAP 0.1/2 GAP 0.1 _Ch.tif]

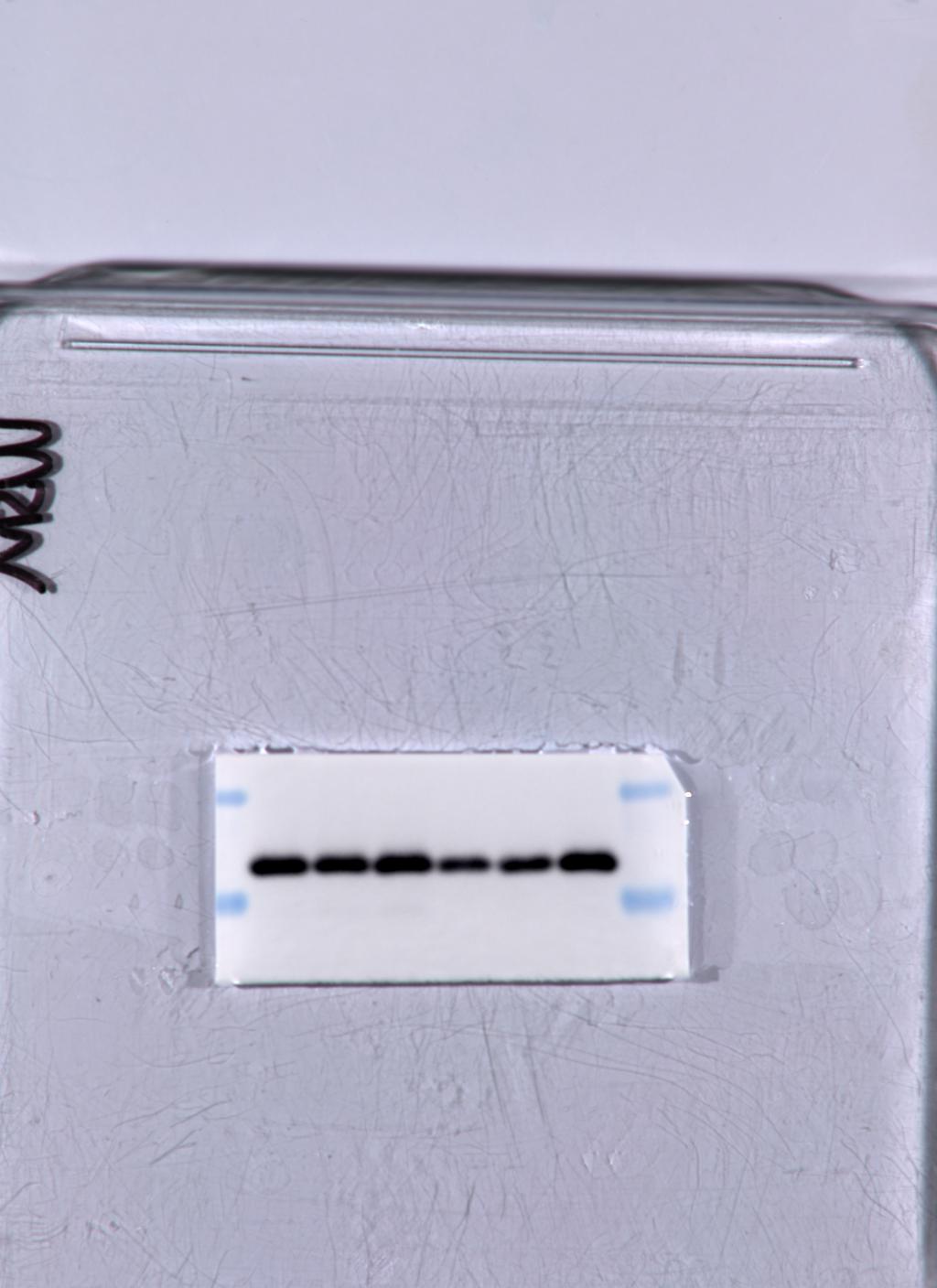

Supplement: Supplementary file 14 — EV Figure Source Data [file 44321_2024_60_MOESM14_ESM.zip › Figure EV2C Source Data/EV2C/88T YAPC/88T-Western H3 0.1/1-2 H3 0.1 _Ch+Marker.jpg]

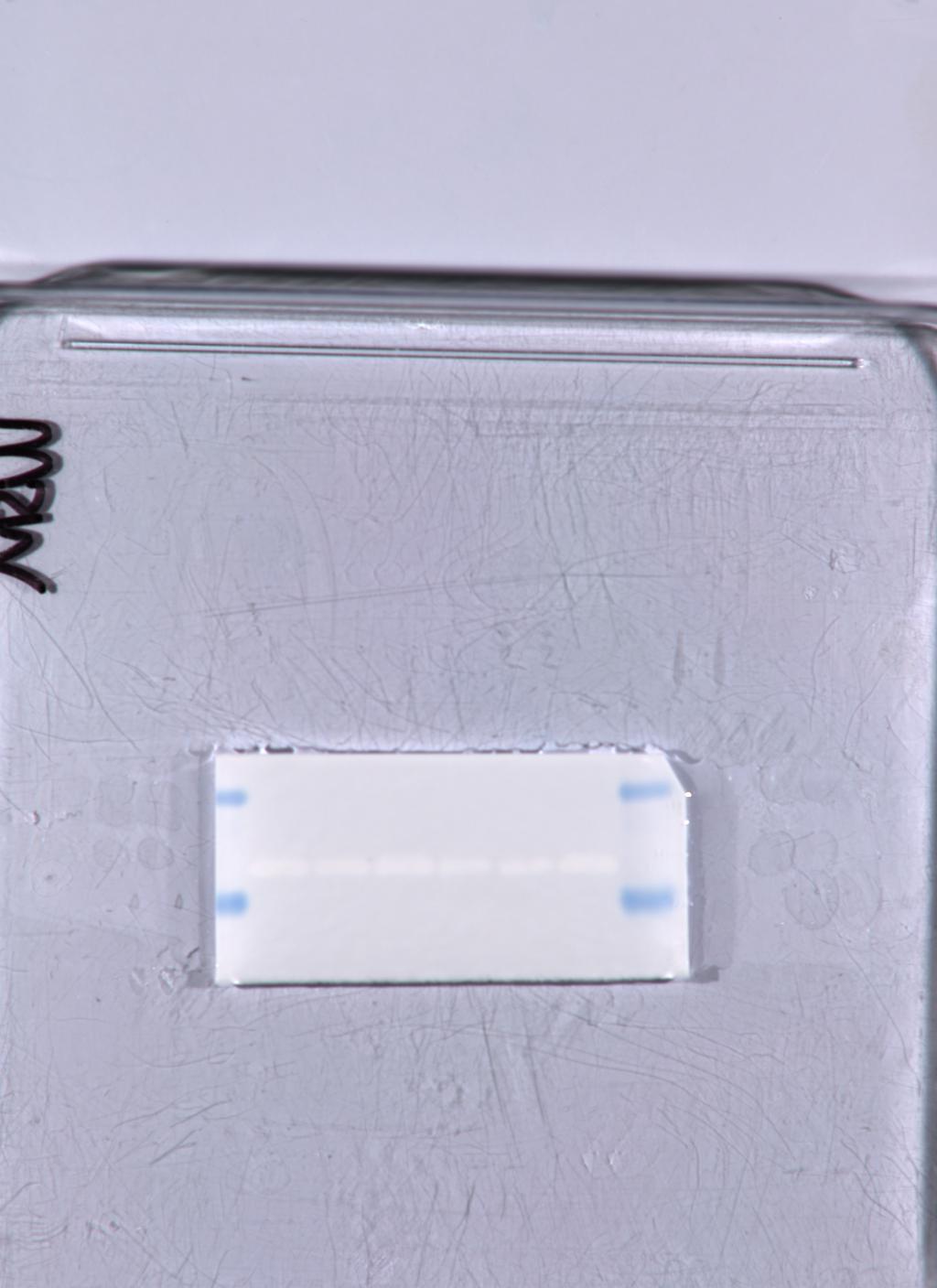

Supplement: Supplementary file 14 — EV Figure Source Data [file 44321_2024_60_MOESM14_ESM.zip › Figure EV2C Source Data/EV2C/88T YAPC/88T-Western H3 0.1/1-2 H3 0.1 _Ch-Marker.jpg]

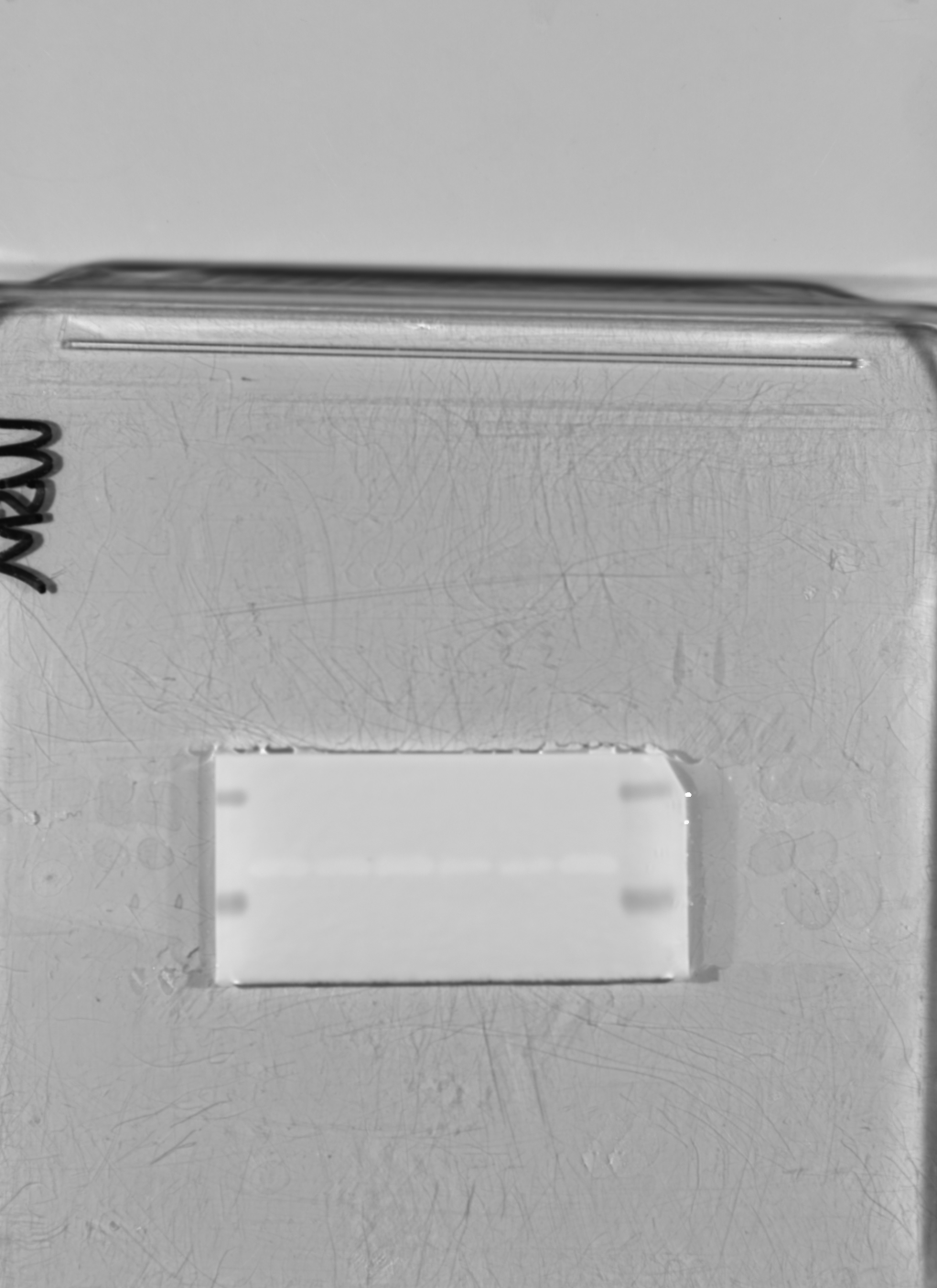

Supplement: Supplementary file 14 — EV Figure Source Data [file 44321_2024_60_MOESM14_ESM.zip › Figure EV2C Source Data/EV2C/88T YAPC/88T-Western H3 0.1/1-2 H3 0.1 _Ch-Marker.tif]

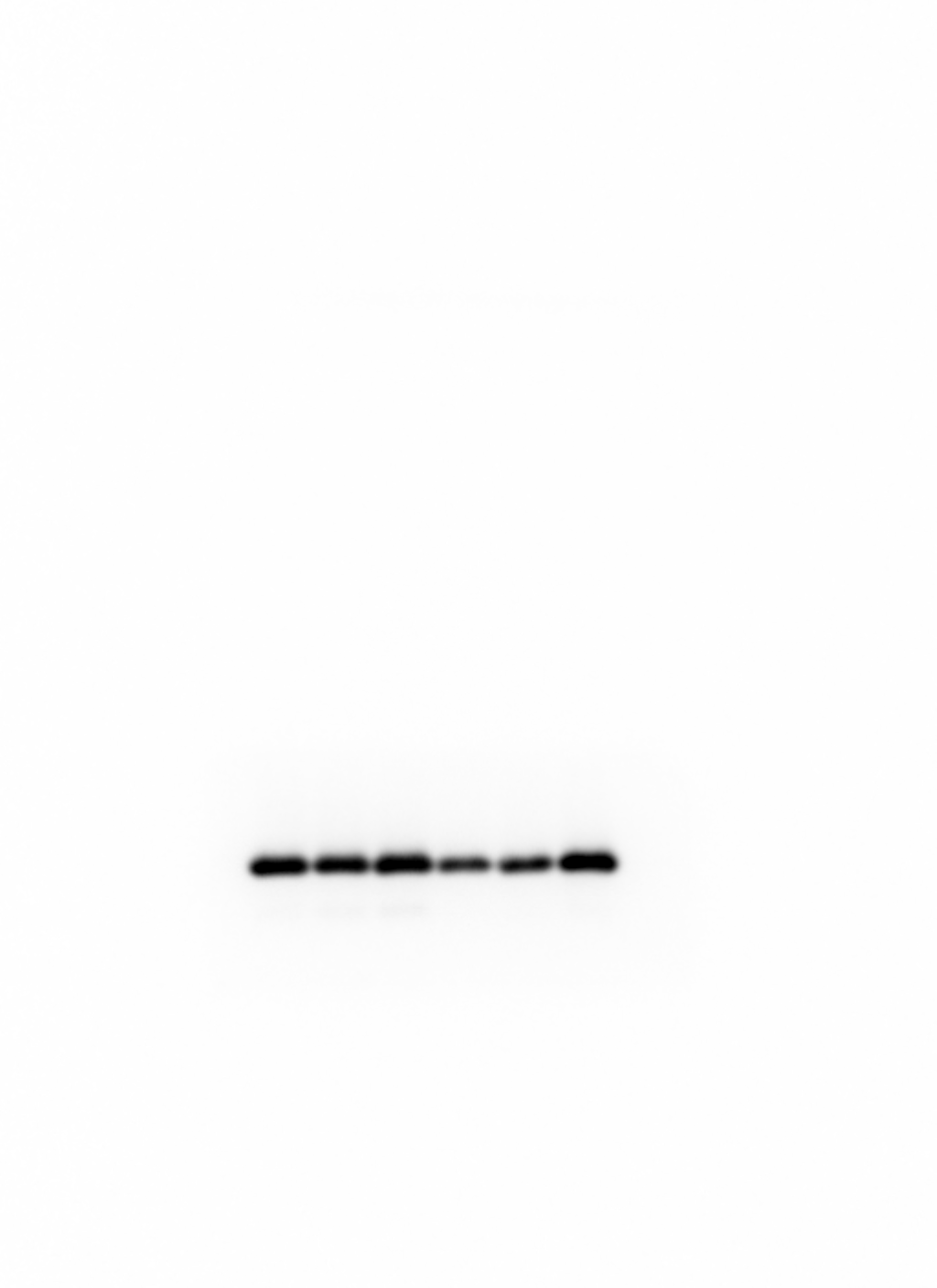

Supplement: Supplementary file 14 — EV Figure Source Data [file 44321_2024_60_MOESM14_ESM.zip › Figure EV2C Source Data/EV2C/88T YAPC/88T-Western H3 0.1/1-2 H3 0.1 _Ch.tif]

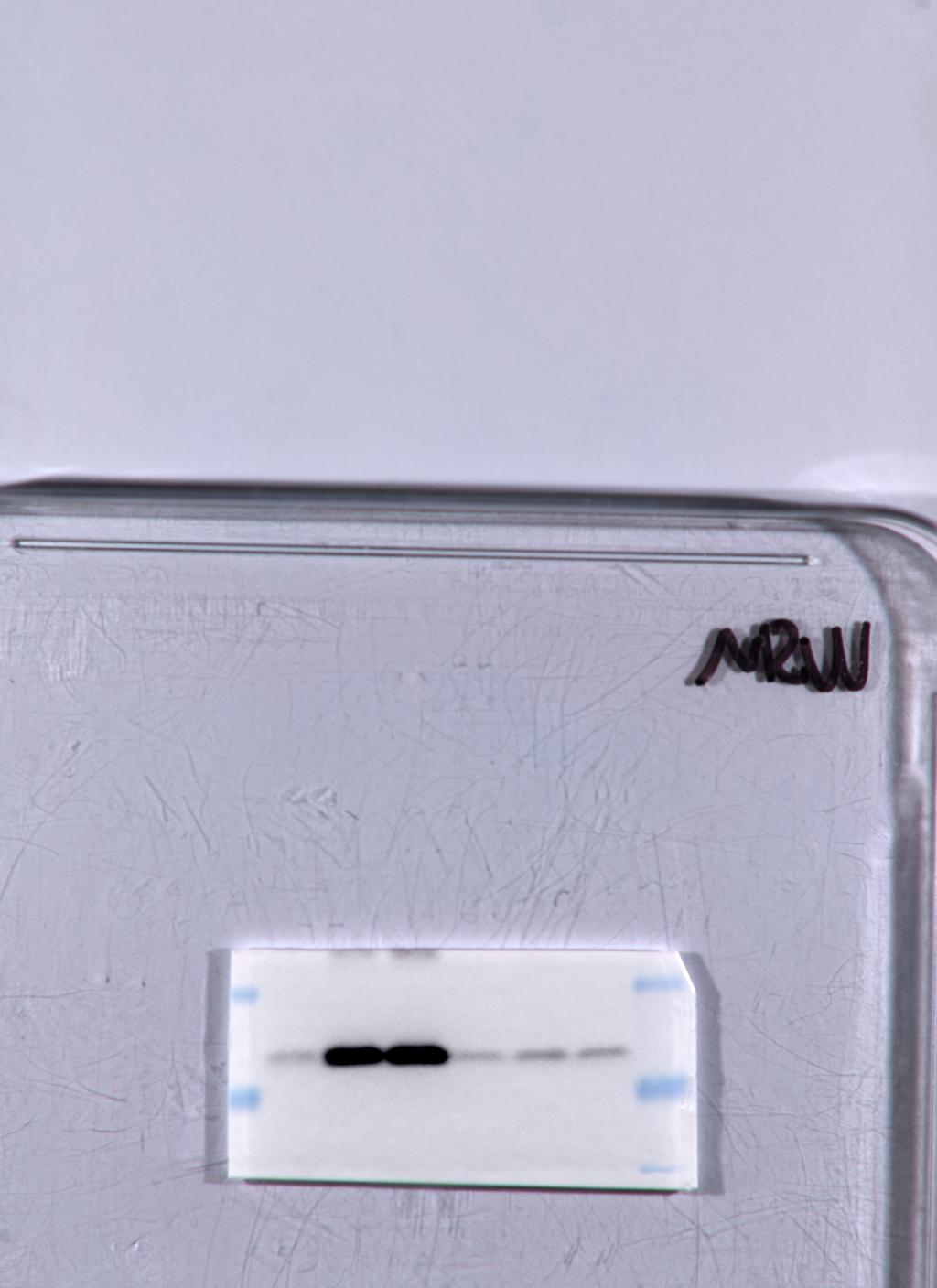

Supplement: Supplementary file 14 — EV Figure Source Data [file 44321_2024_60_MOESM14_ESM.zip › Figure EV2C Source Data/EV2C/88T YAPC/88T-Western γH2A 0.3/2-2 γH2A 0.3 _Ch+Marker.jpg]

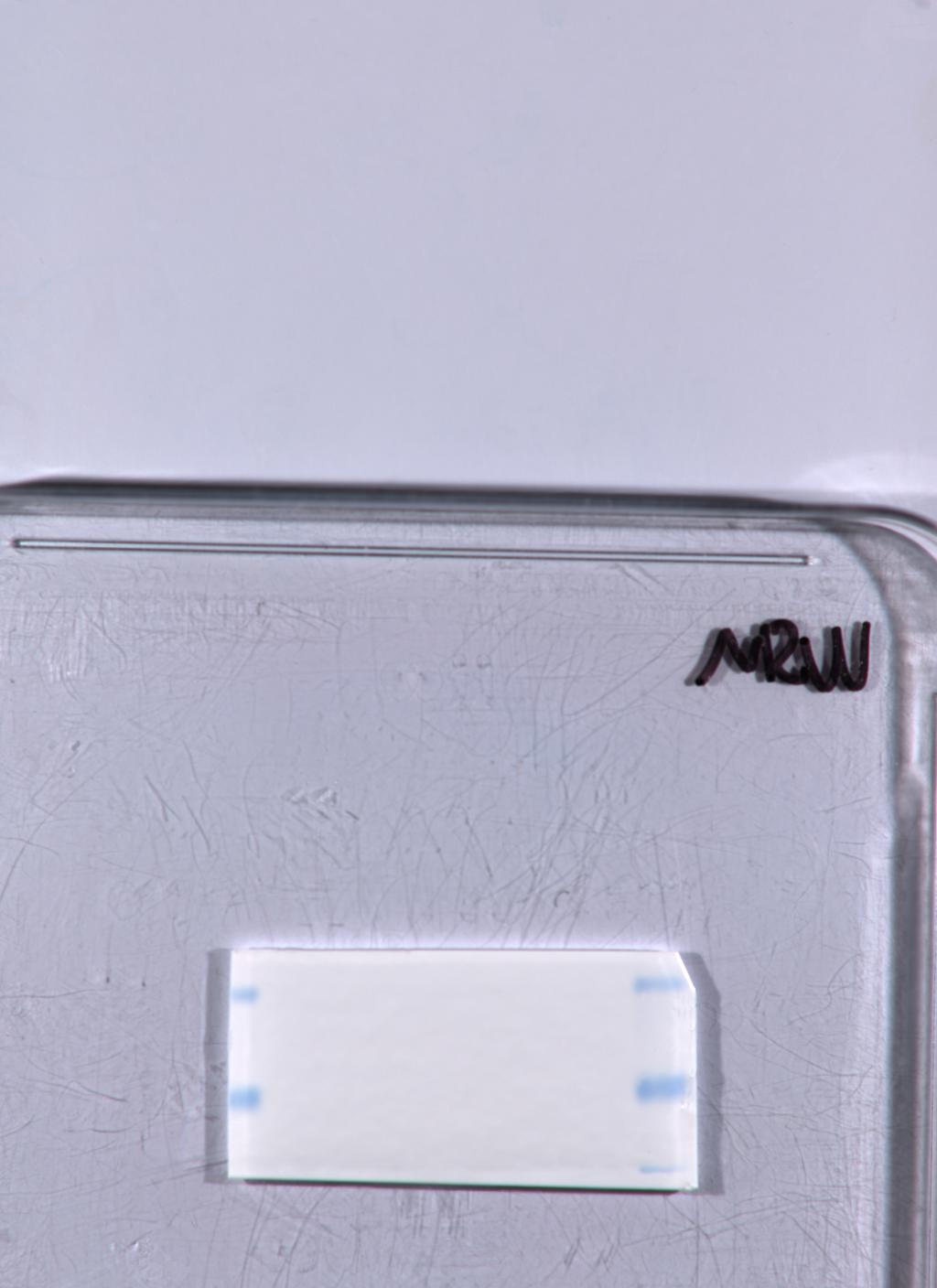

Supplement: Supplementary file 14 — EV Figure Source Data [file 44321_2024_60_MOESM14_ESM.zip › Figure EV2C Source Data/EV2C/88T YAPC/88T-Western γH2A 0.3/2-2 γH2A 0.3 _Ch-Marker.jpg]

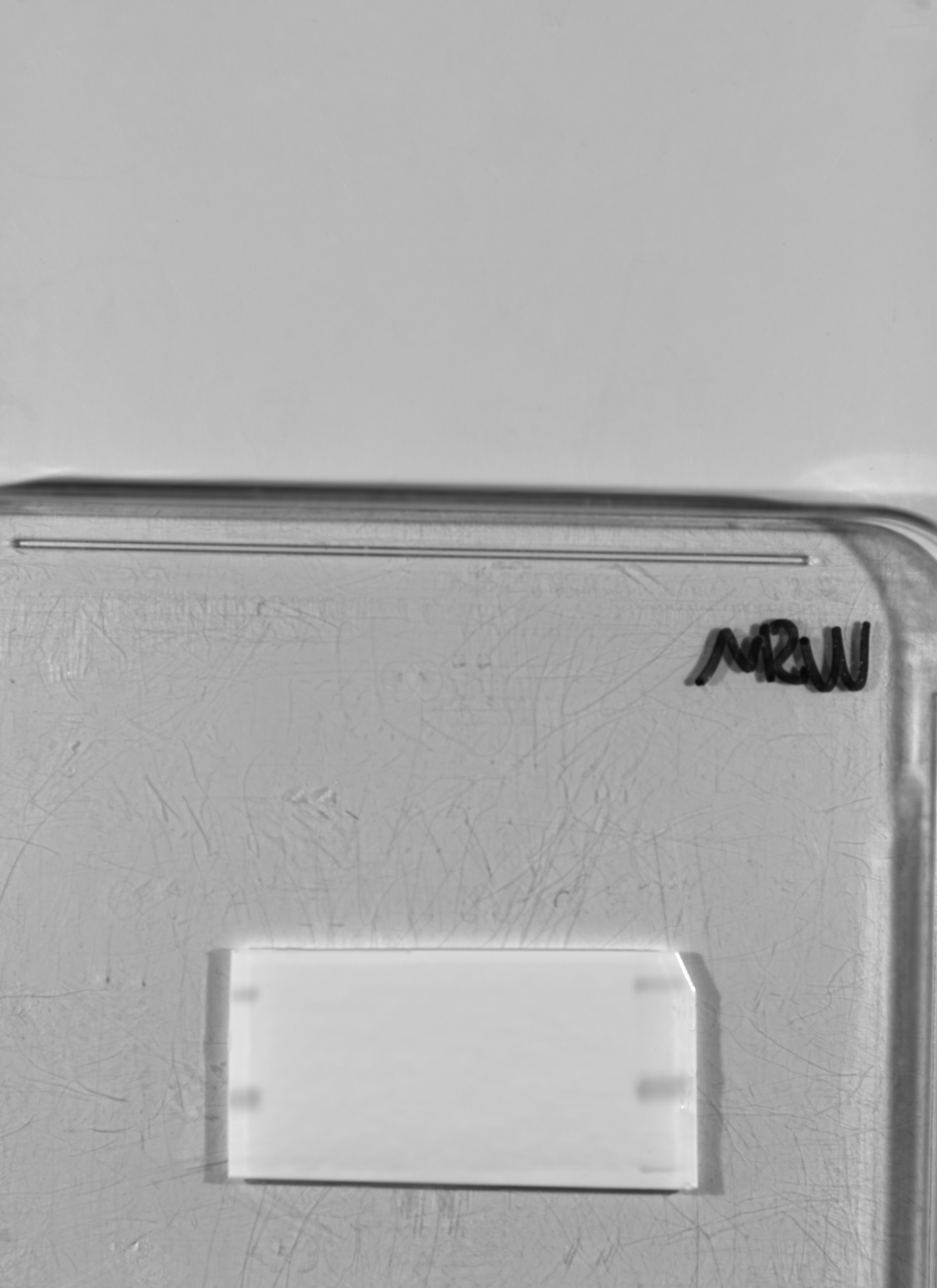

Supplement: Supplementary file 14 — EV Figure Source Data [file 44321_2024_60_MOESM14_ESM.zip › Figure EV2C Source Data/EV2C/88T YAPC/88T-Western γH2A 0.3/2-2 γH2A 0.3 _Ch-Marker.tif]

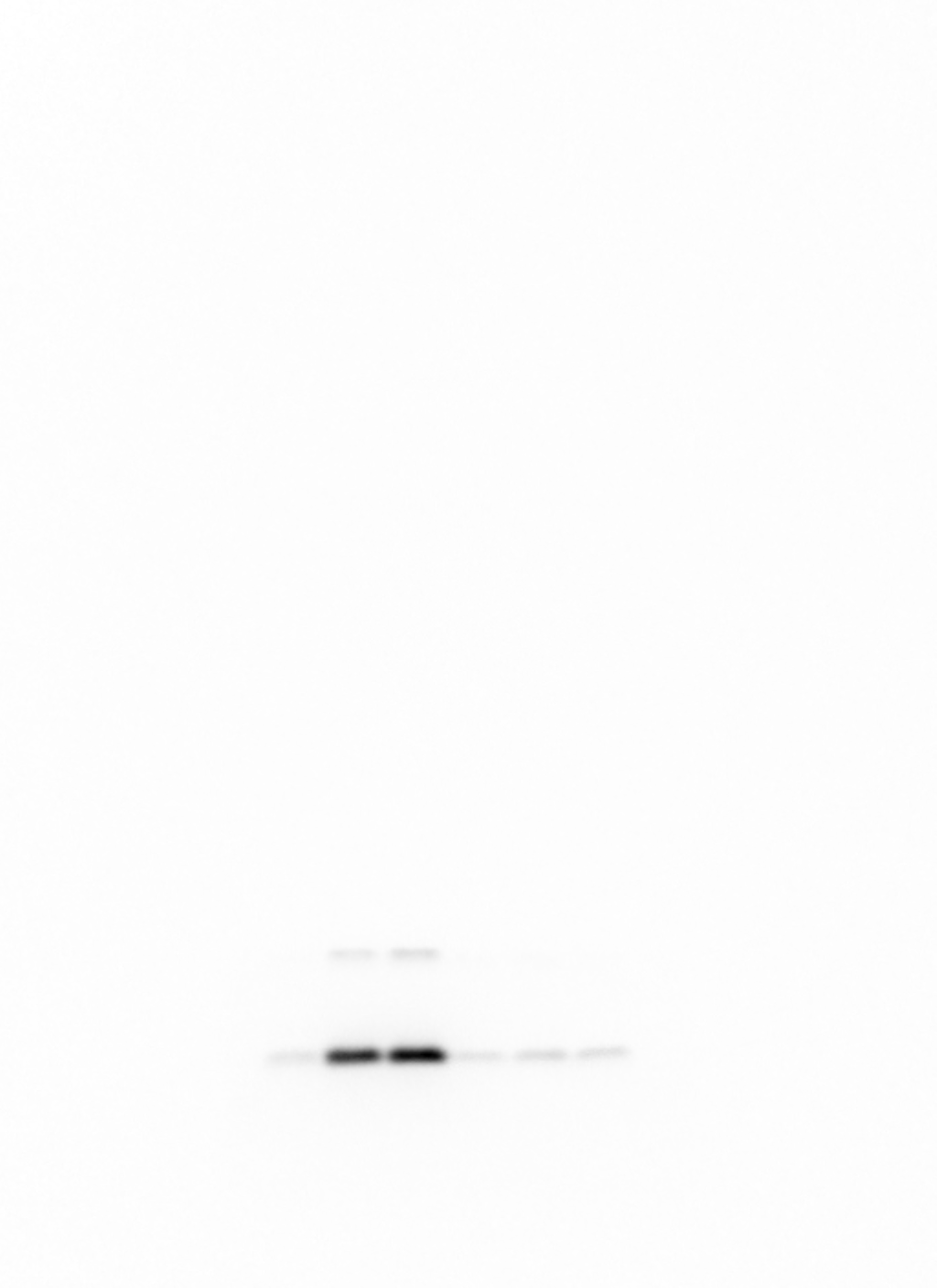

Supplement: Supplementary file 14 — EV Figure Source Data [file 44321_2024_60_MOESM14_ESM.zip › Figure EV2C Source Data/EV2C/88T YAPC/88T-Western γH2A 0.3/2-2 γH2A 0.3 _Ch.tif]

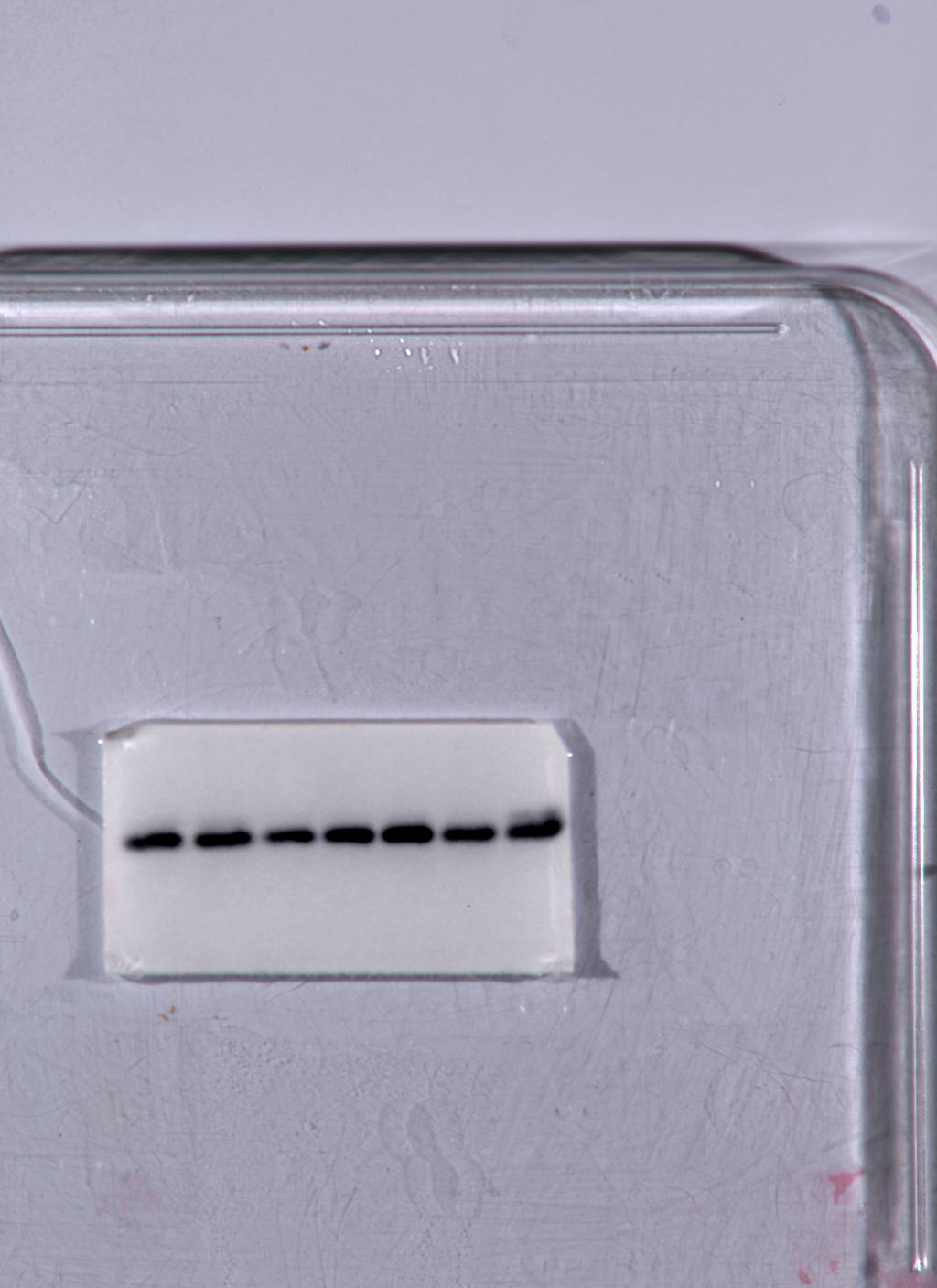

Supplement: Supplementary file 14 — EV Figure Source Data [file 44321_2024_60_MOESM14_ESM.zip › Figure EV2C Source Data/EV2C/88T YAPC/Western CDK1 123.8/wsm 8 CDK1 123.8 _Ch+Marker.jpg]

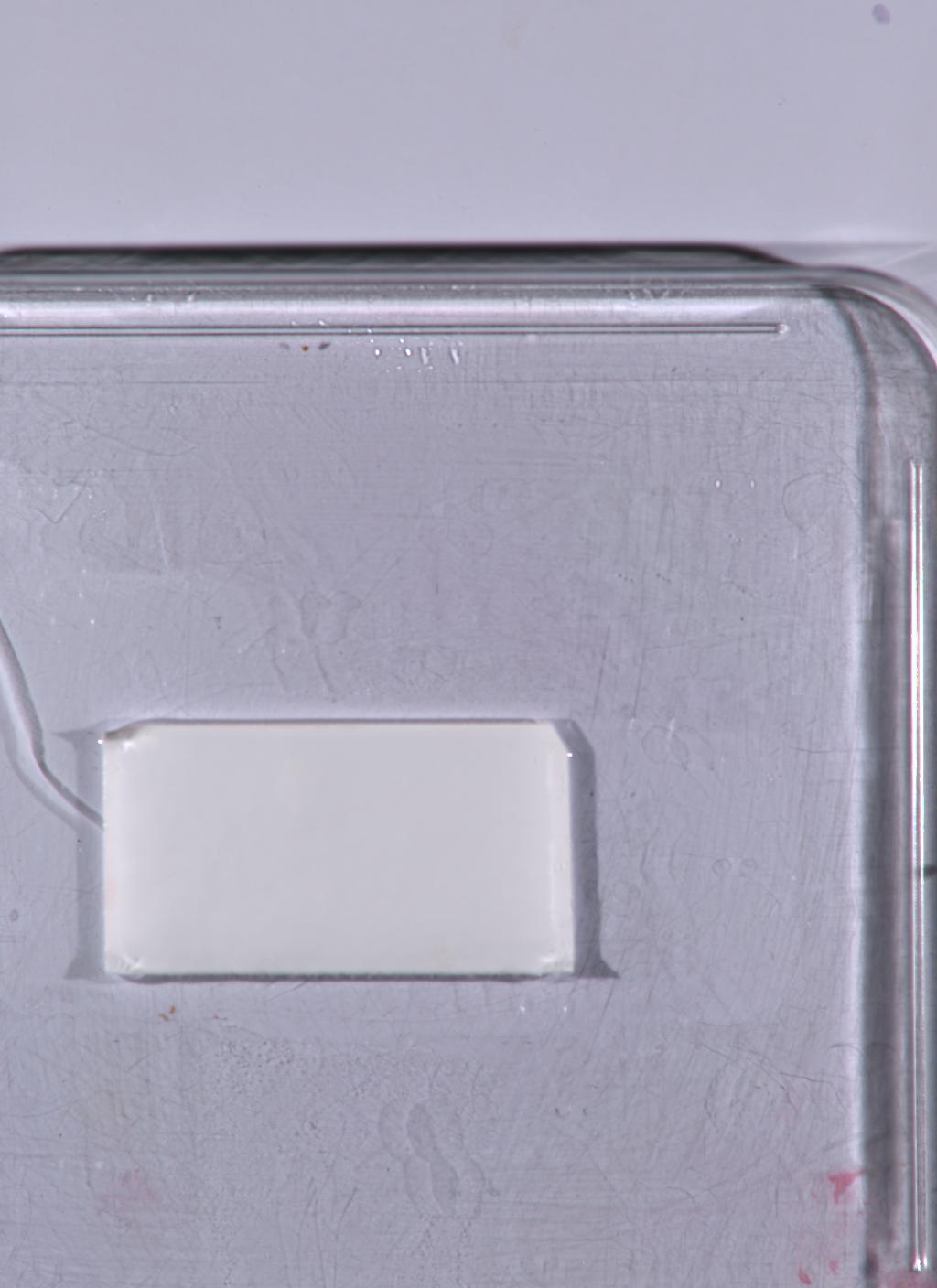

Supplement: Supplementary file 14 — EV Figure Source Data [file 44321_2024_60_MOESM14_ESM.zip › Figure EV2C Source Data/EV2C/88T YAPC/Western CDK1 123.8/wsm 8 CDK1 123.8 _Ch-Marker.jpg]

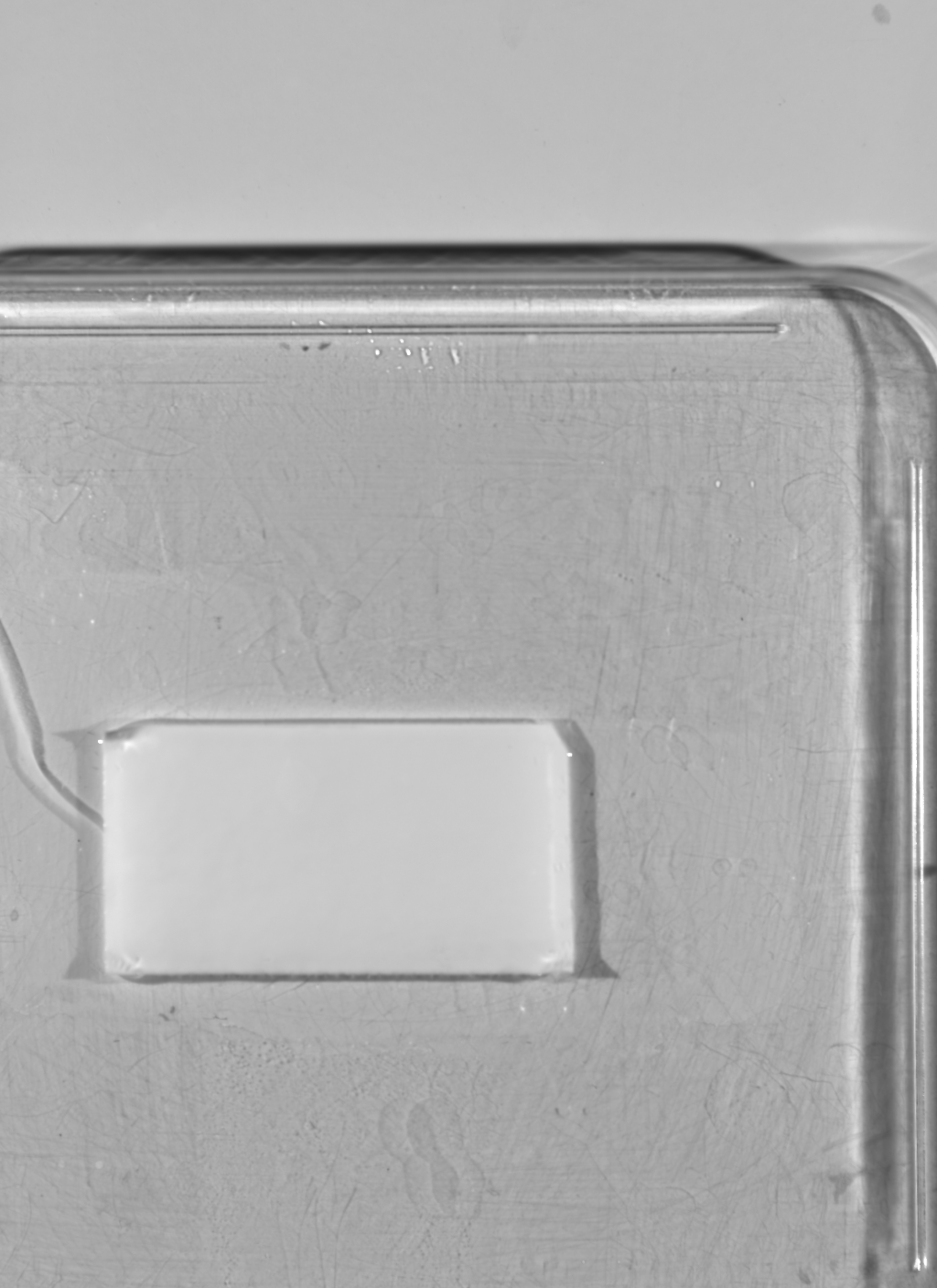

Supplement: Supplementary file 14 — EV Figure Source Data [file 44321_2024_60_MOESM14_ESM.zip › Figure EV2C Source Data/EV2C/88T YAPC/Western CDK1 123.8/wsm 8 CDK1 123.8 _Ch-Marker.tif]

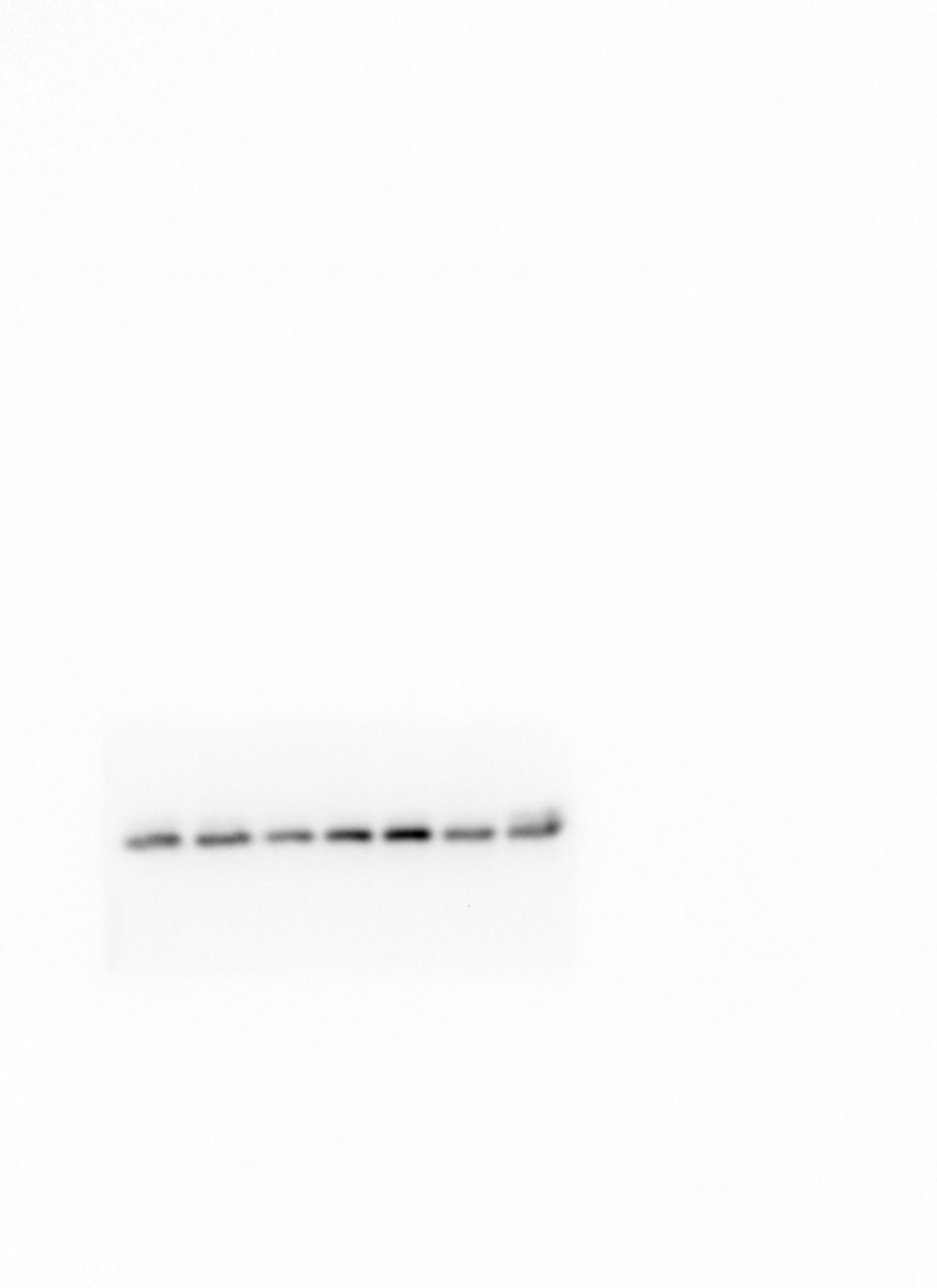

Supplement: Supplementary file 14 — EV Figure Source Data [file 44321_2024_60_MOESM14_ESM.zip › Figure EV2C Source Data/EV2C/88T YAPC/Western CDK1 123.8/wsm 8 CDK1 123.8 _Ch.tif]

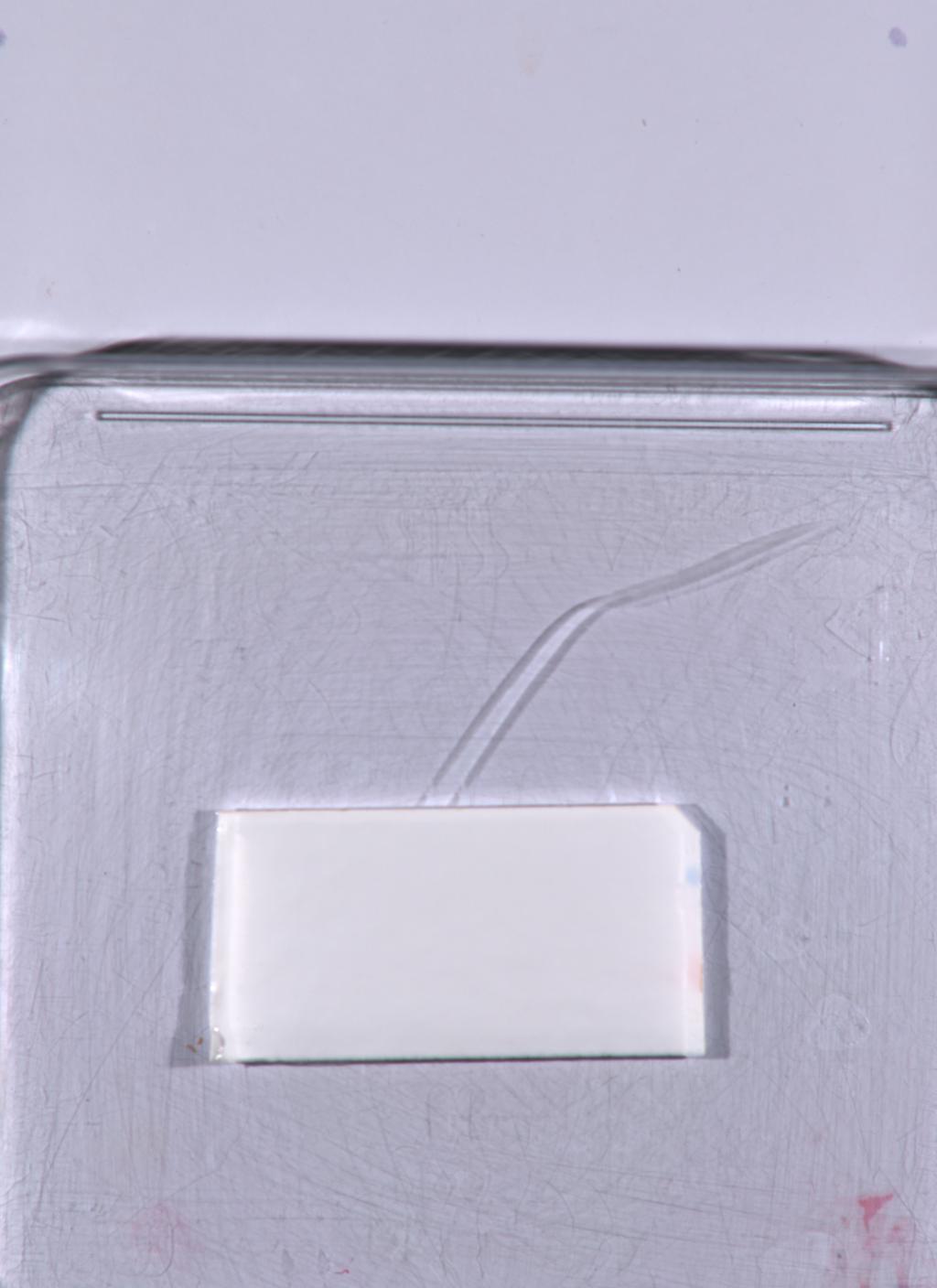

Supplement: Supplementary file 14 — EV Figure Source Data [file 44321_2024_60_MOESM14_ESM.zip › Figure EV2C Source Data/EV2C/88T YAPC/Western phoCDK1-T14 1.7/wsm 7 P-CDK1 1.7 2021.06.30_15.39.52_Ch-Marker.jpg]

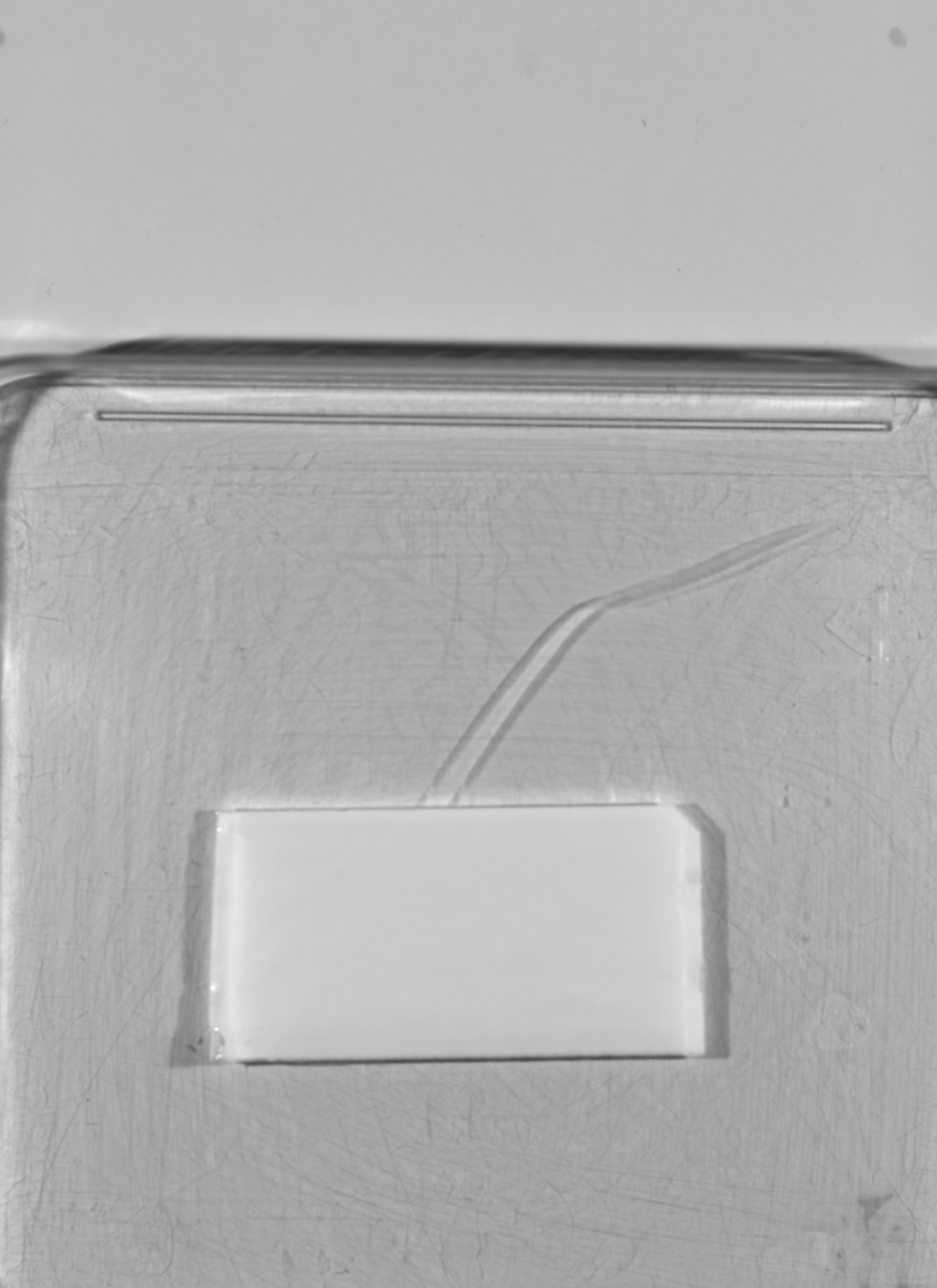

Supplement: Supplementary file 14 — EV Figure Source Data [file 44321_2024_60_MOESM14_ESM.zip › Figure EV2C Source Data/EV2C/88T YAPC/Western phoCDK1-T14 1.7/wsm 7 P-CDK1 1.7 2021.06.30_15.39.52_Ch-Marker.tif]

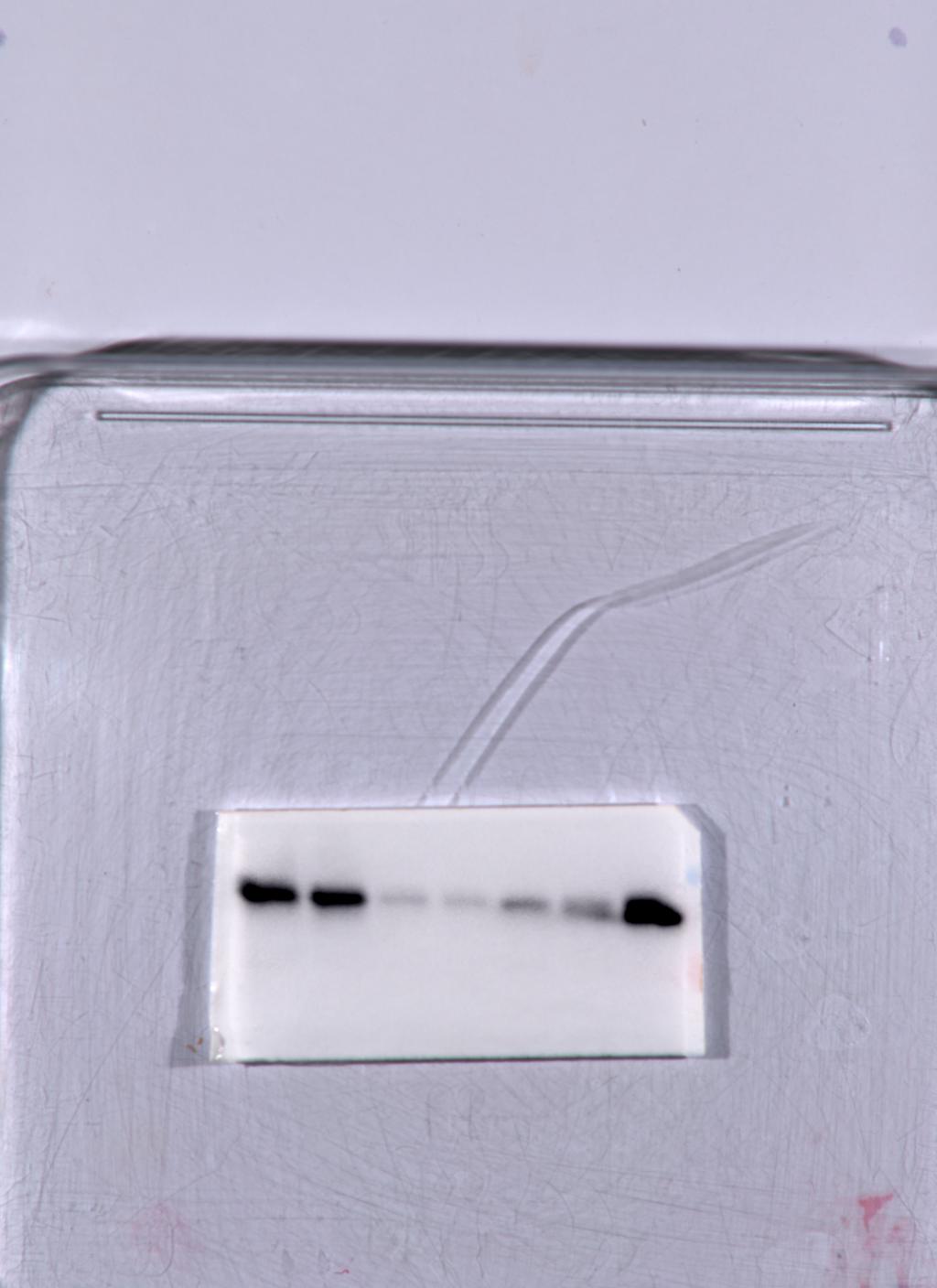

Supplement: Supplementary file 14 — EV Figure Source Data [file 44321_2024_60_MOESM14_ESM.zip › Figure EV2C Source Data/EV2C/88T YAPC/Western phoCDK1-T14 1.7/wsm 7 P-CDK1 1.7 _Ch+Marker.jpg]

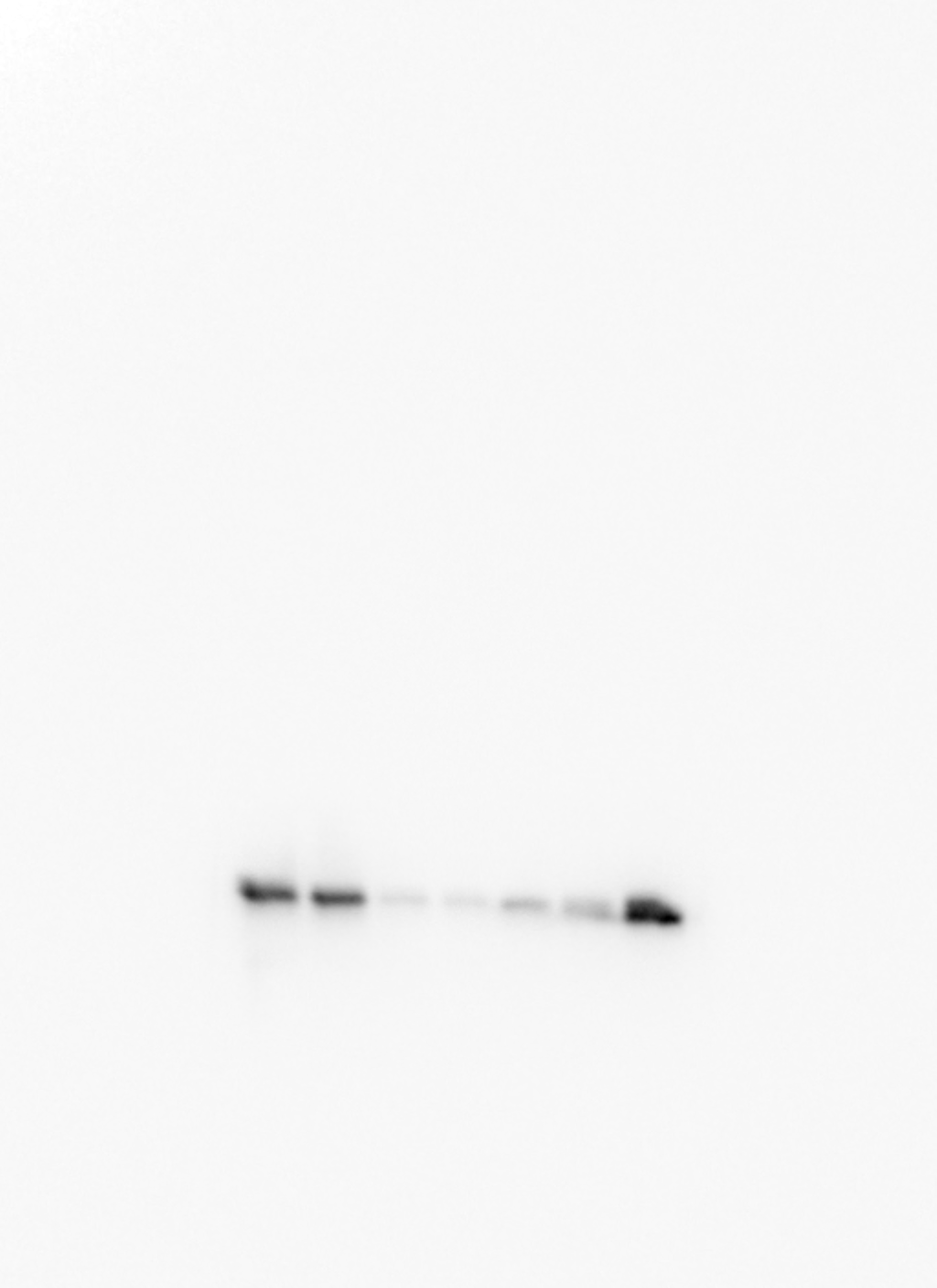

Supplement: Supplementary file 14 — EV Figure Source Data [file 44321_2024_60_MOESM14_ESM.zip › Figure EV2C Source Data/EV2C/88T YAPC/Western phoCDK1-T14 1.7/wsm 7 P-CDK1 1.7 _Ch.tif]

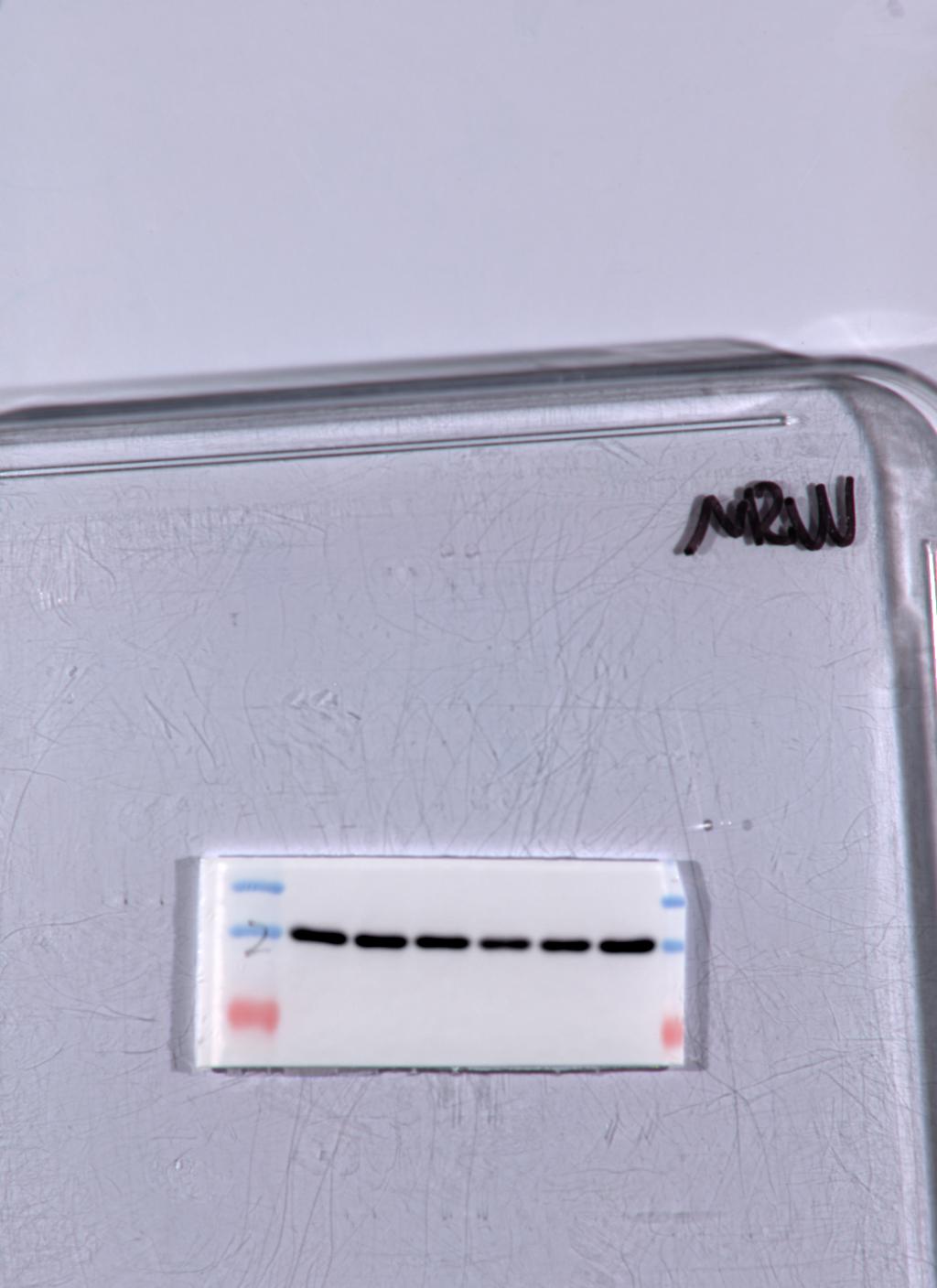

Supplement: Supplementary file 14 — EV Figure Source Data [file 44321_2024_60_MOESM14_ESM.zip › Figure EV2C Source Data/EV2C/88T YAPC/YAPC-Western GAP 0.1/2-1 GAP 0.1 _Ch+Marker.jpg]

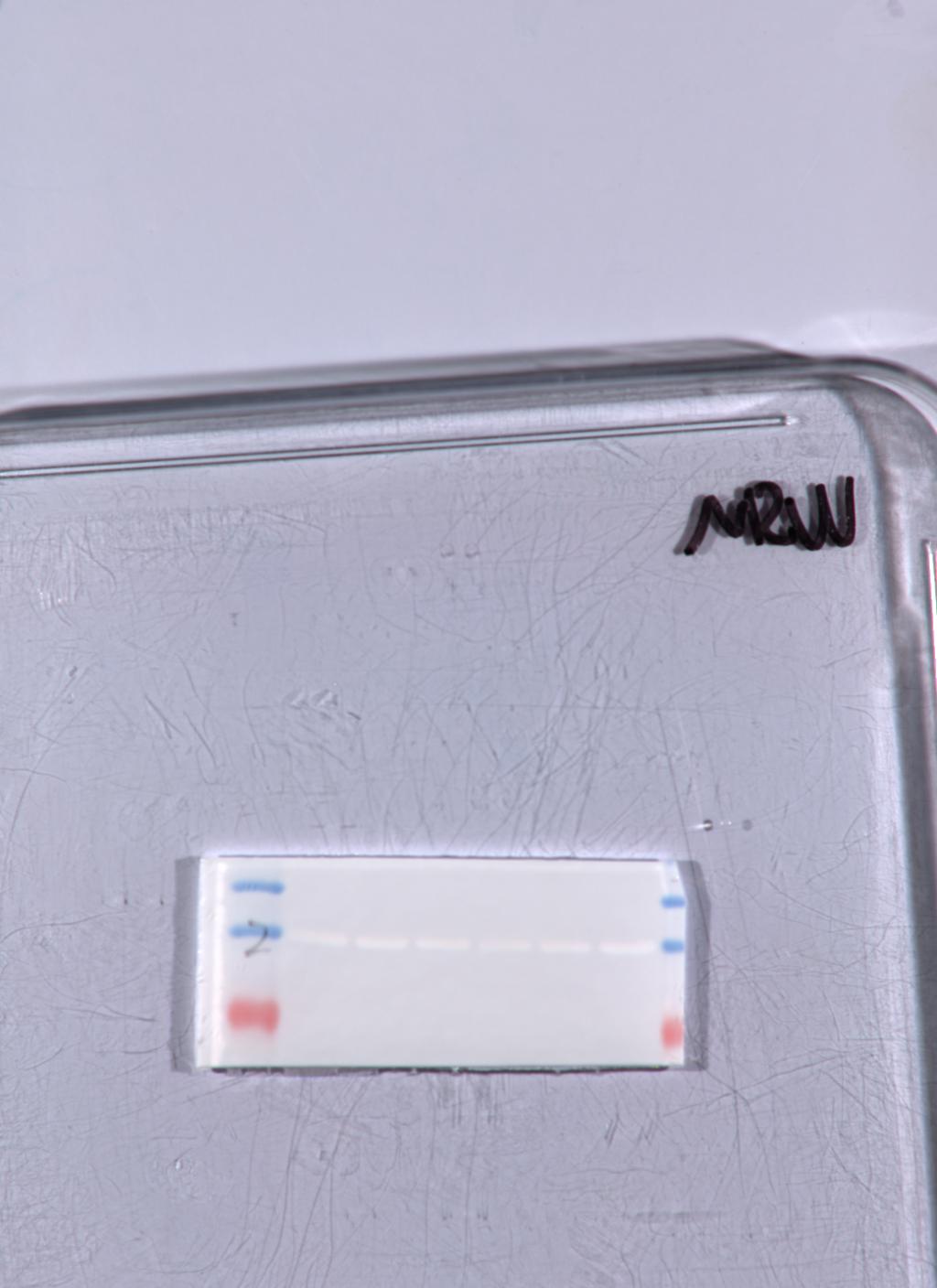

Supplement: Supplementary file 14 — EV Figure Source Data [file 44321_2024_60_MOESM14_ESM.zip › Figure EV2C Source Data/EV2C/88T YAPC/YAPC-Western GAP 0.1/2-1 GAP 0.1 _Ch-Marker.jpg]

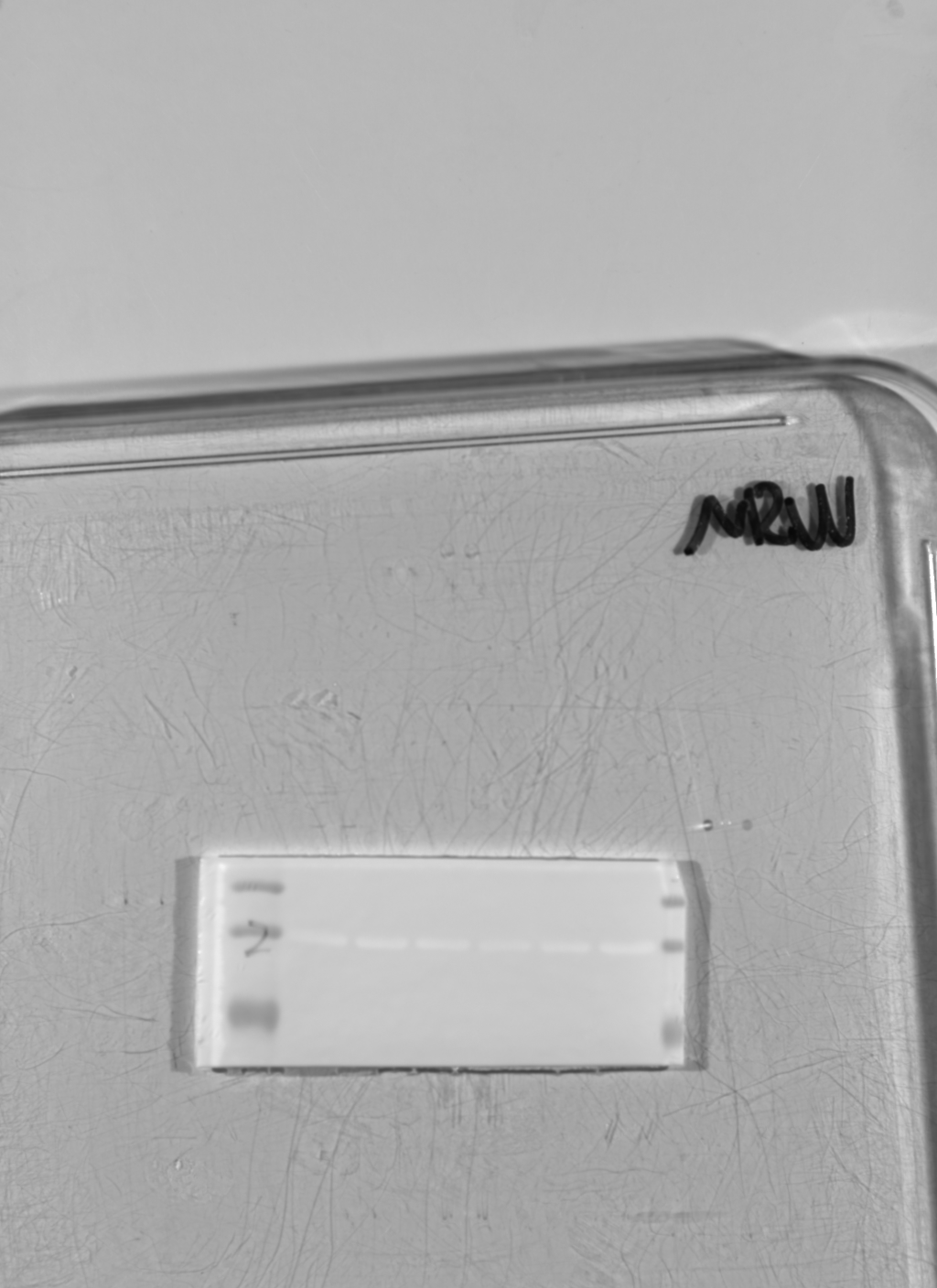

Supplement: Supplementary file 14 — EV Figure Source Data [file 44321_2024_60_MOESM14_ESM.zip › Figure EV2C Source Data/EV2C/88T YAPC/YAPC-Western GAP 0.1/2-1 GAP 0.1 _Ch-Marker.tif]

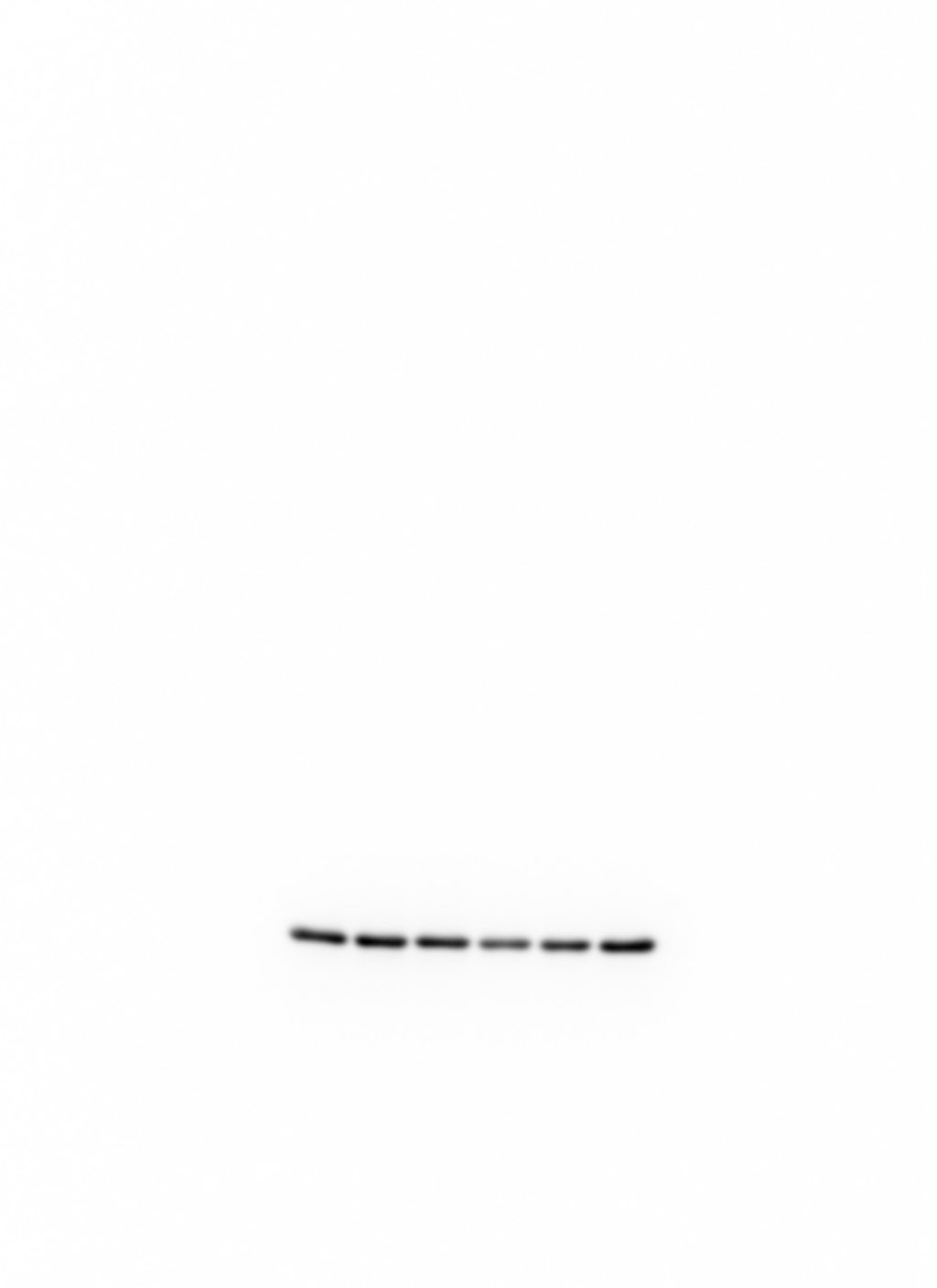

Supplement: Supplementary file 14 — EV Figure Source Data [file 44321_2024_60_MOESM14_ESM.zip › Figure EV2C Source Data/EV2C/88T YAPC/YAPC-Western GAP 0.1/2-1 GAP 0.1 _Ch.tif]

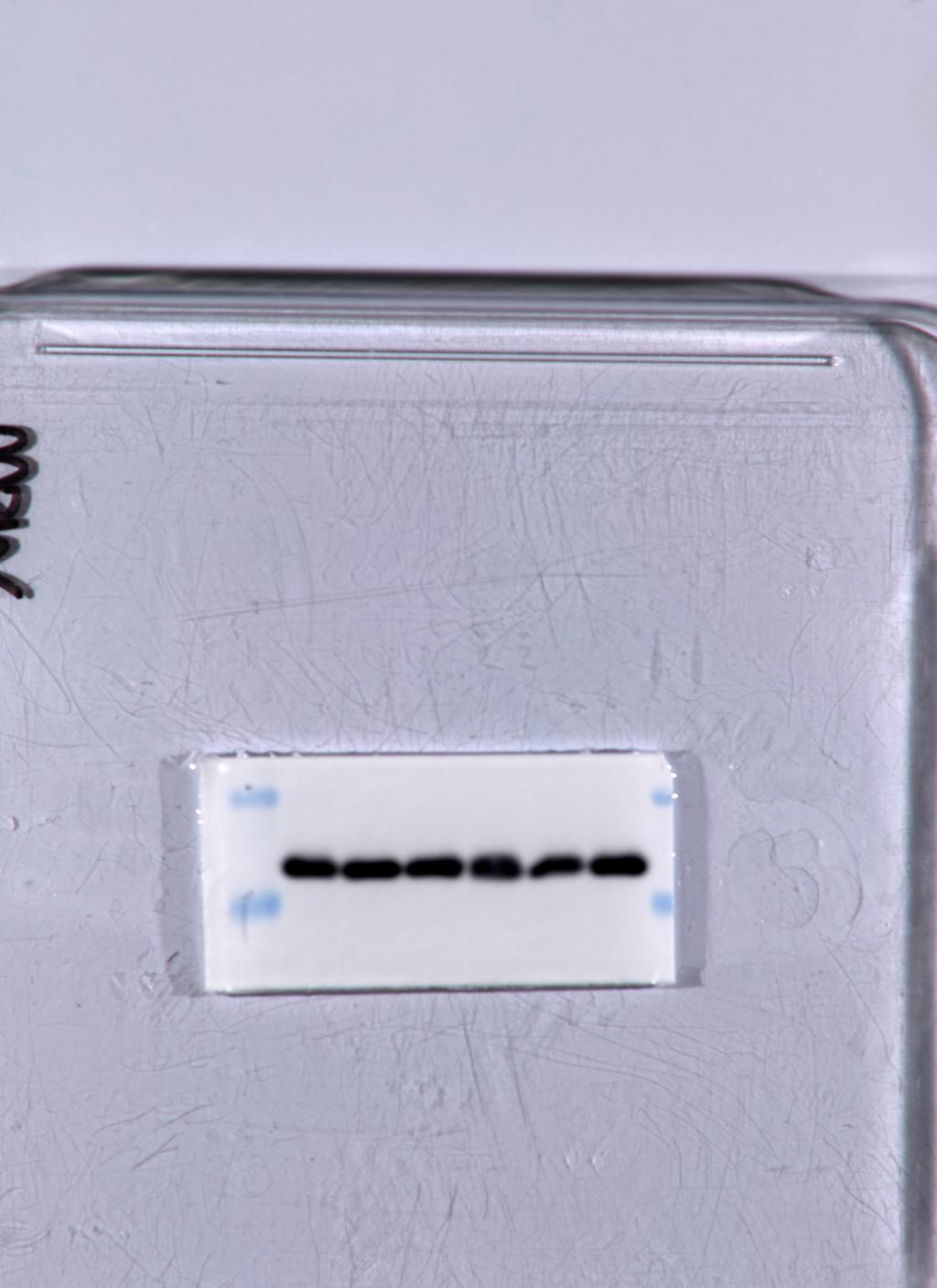

Supplement: Supplementary file 14 — EV Figure Source Data [file 44321_2024_60_MOESM14_ESM.zip › Figure EV2C Source Data/EV2C/88T YAPC/YAPC-Western H3 0.1/1-1 H3 0.1 _Ch+Marker.jpg]

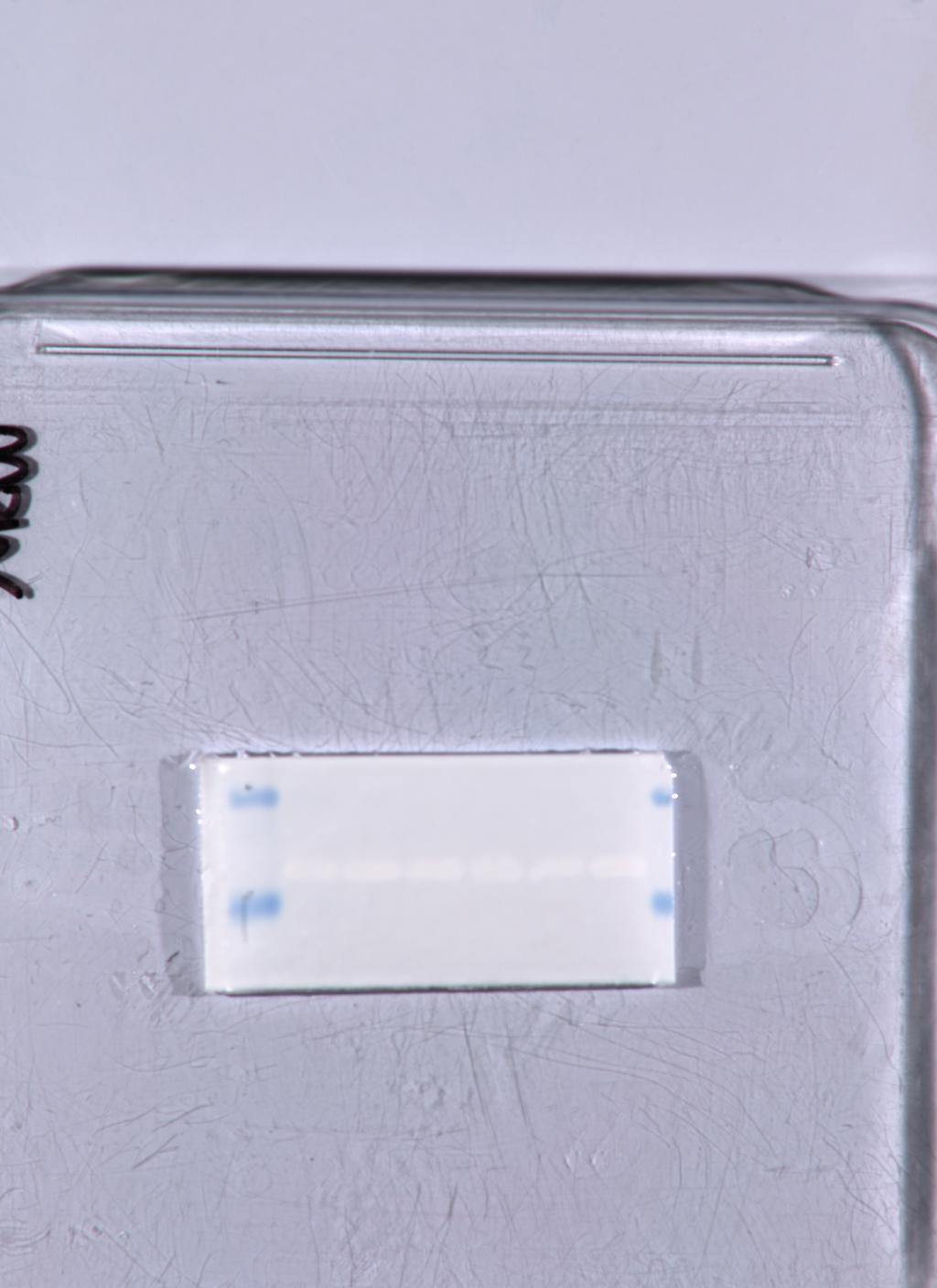

Supplement: Supplementary file 14 — EV Figure Source Data [file 44321_2024_60_MOESM14_ESM.zip › Figure EV2C Source Data/EV2C/88T YAPC/YAPC-Western H3 0.1/1-1 H3 0.1 _Ch-Marker.jpg]

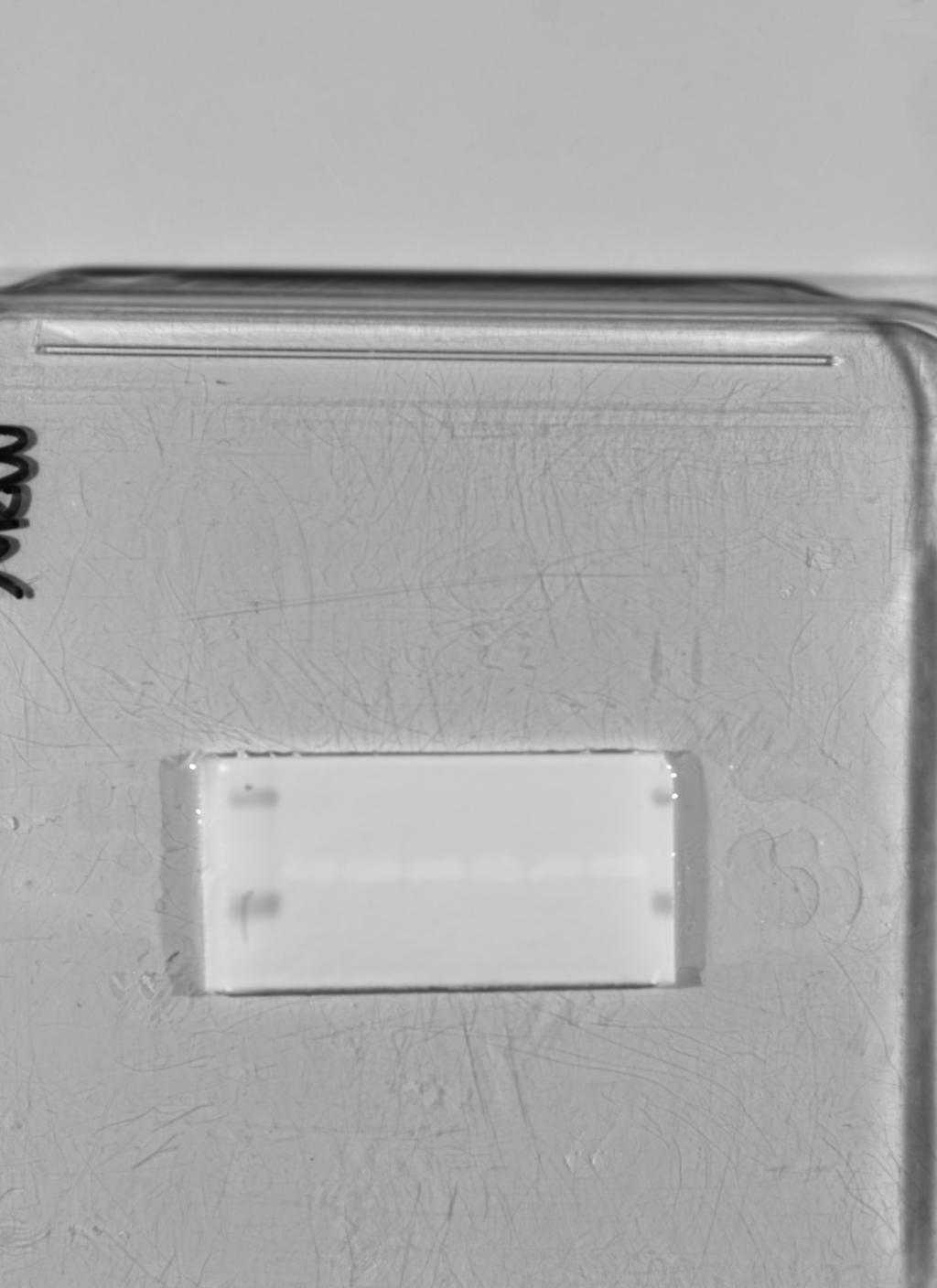

Supplement: Supplementary file 14 — EV Figure Source Data [file 44321_2024_60_MOESM14_ESM.zip › Figure EV2C Source Data/EV2C/88T YAPC/YAPC-Western H3 0.1/1-1 H3 0.1 _Ch-Marker.tif]

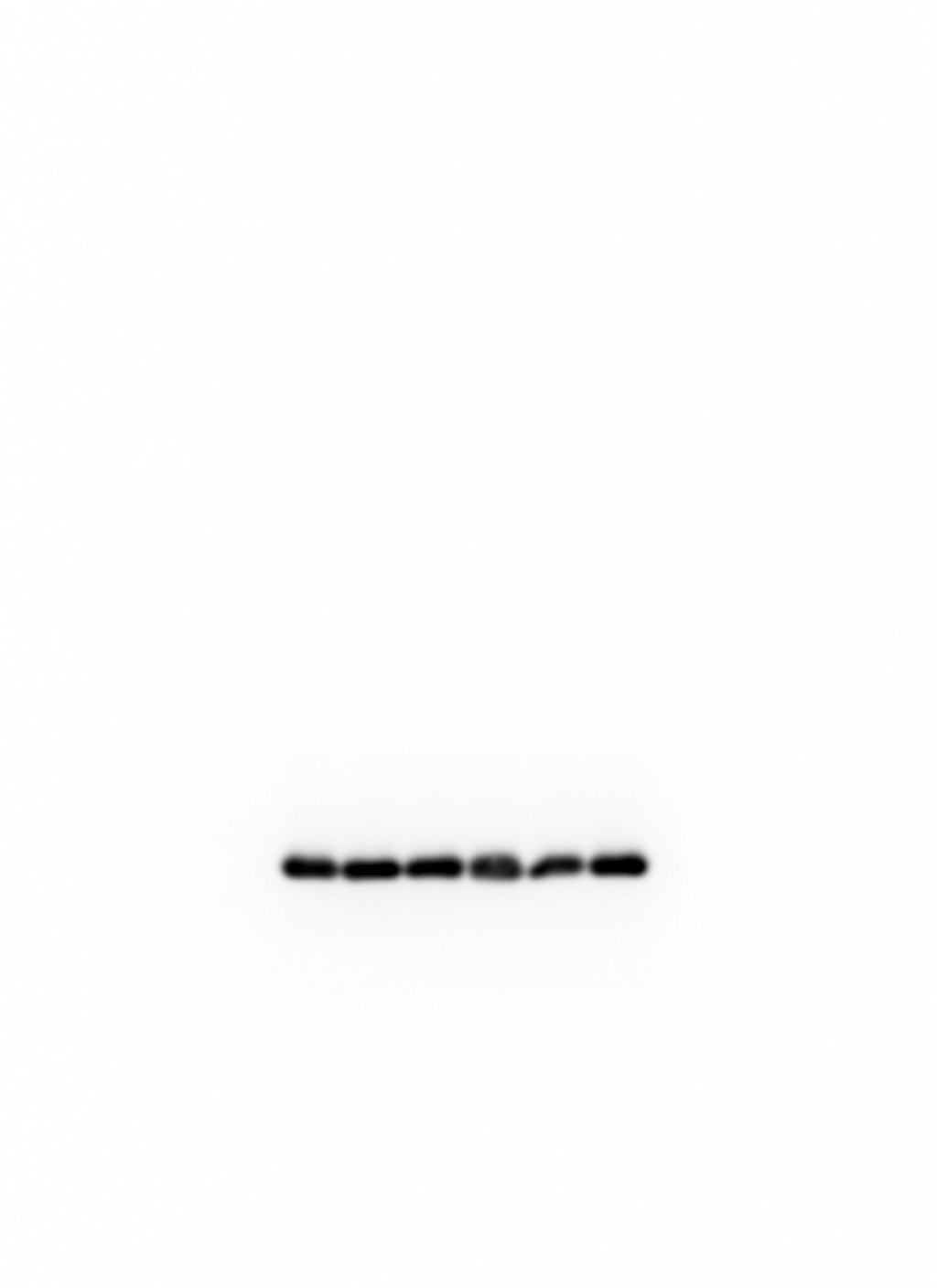

Supplement: Supplementary file 14 — EV Figure Source Data [file 44321_2024_60_MOESM14_ESM.zip › Figure EV2C Source Data/EV2C/88T YAPC/YAPC-Western H3 0.1/1-1 H3 0.1 _Ch.tif]

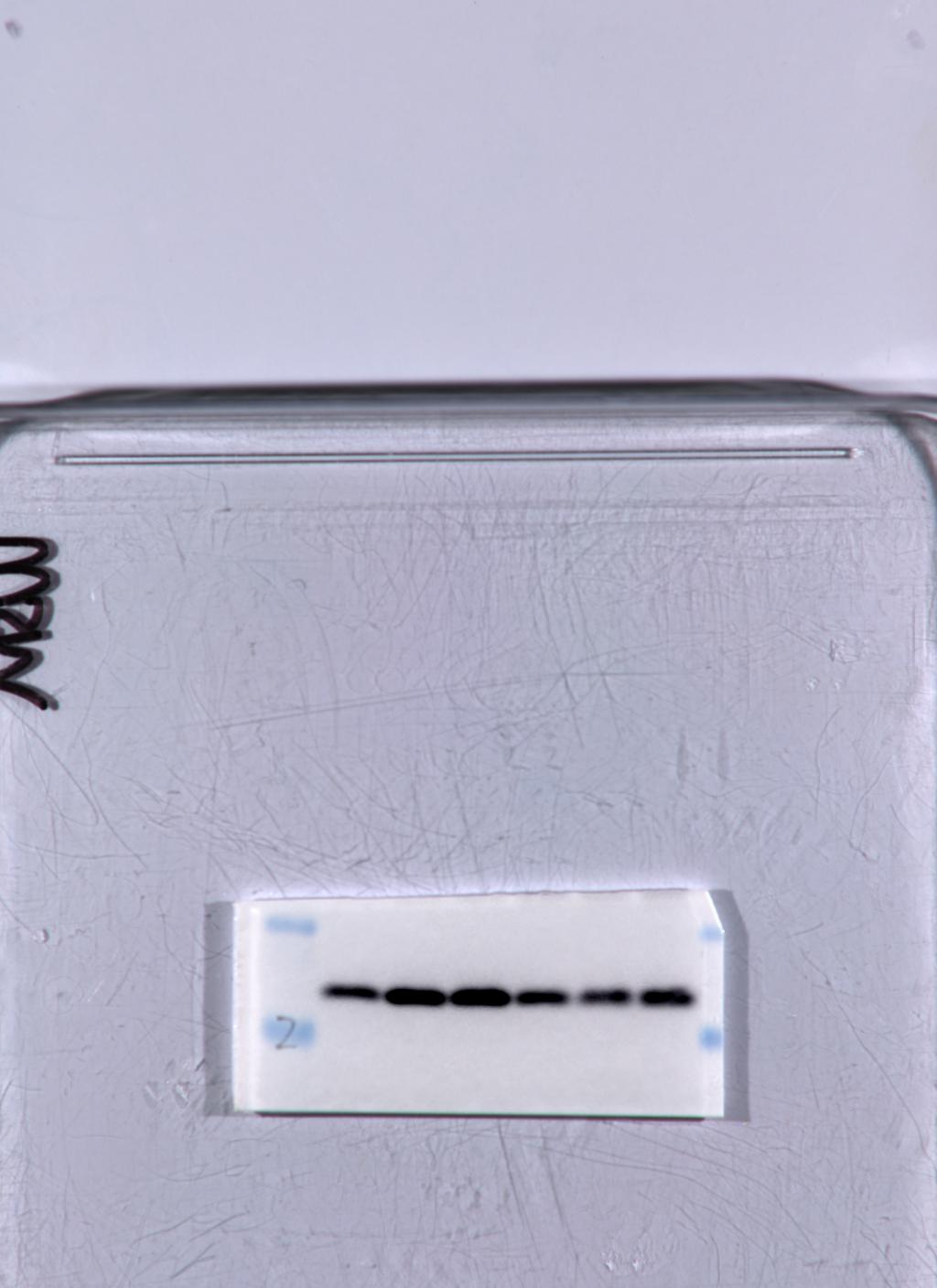

Supplement: Supplementary file 14 — EV Figure Source Data [file 44321_2024_60_MOESM14_ESM.zip › Figure EV2C Source Data/EV2C/88T YAPC/YAPC-Western γH2A 0.5/2-1 γH2A 0.5 _Ch+Marker.jpg]

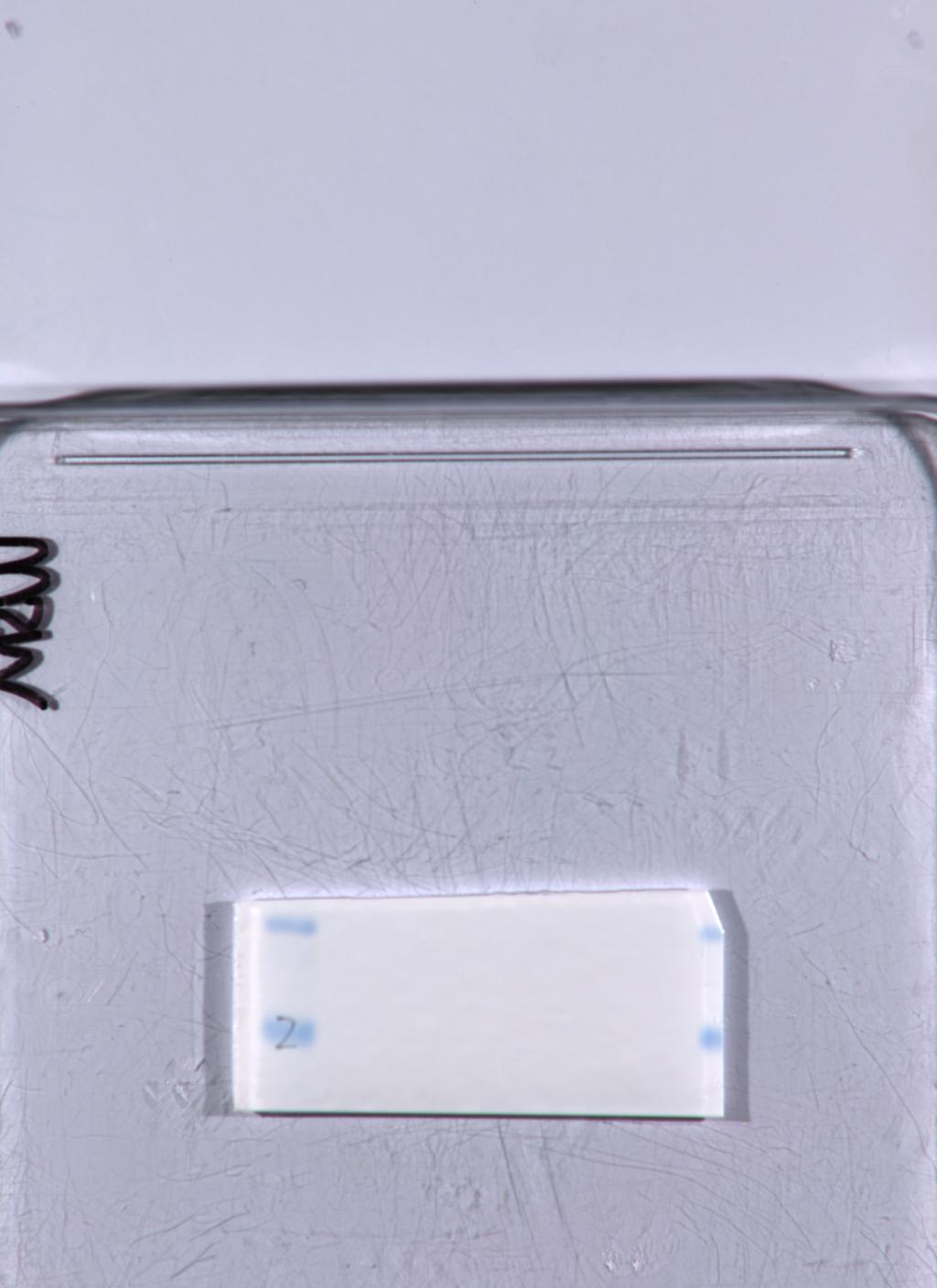

Supplement: Supplementary file 14 — EV Figure Source Data [file 44321_2024_60_MOESM14_ESM.zip › Figure EV2C Source Data/EV2C/88T YAPC/YAPC-Western γH2A 0.5/2-1 γH2A 0.5 _Ch-Marker.jpg]

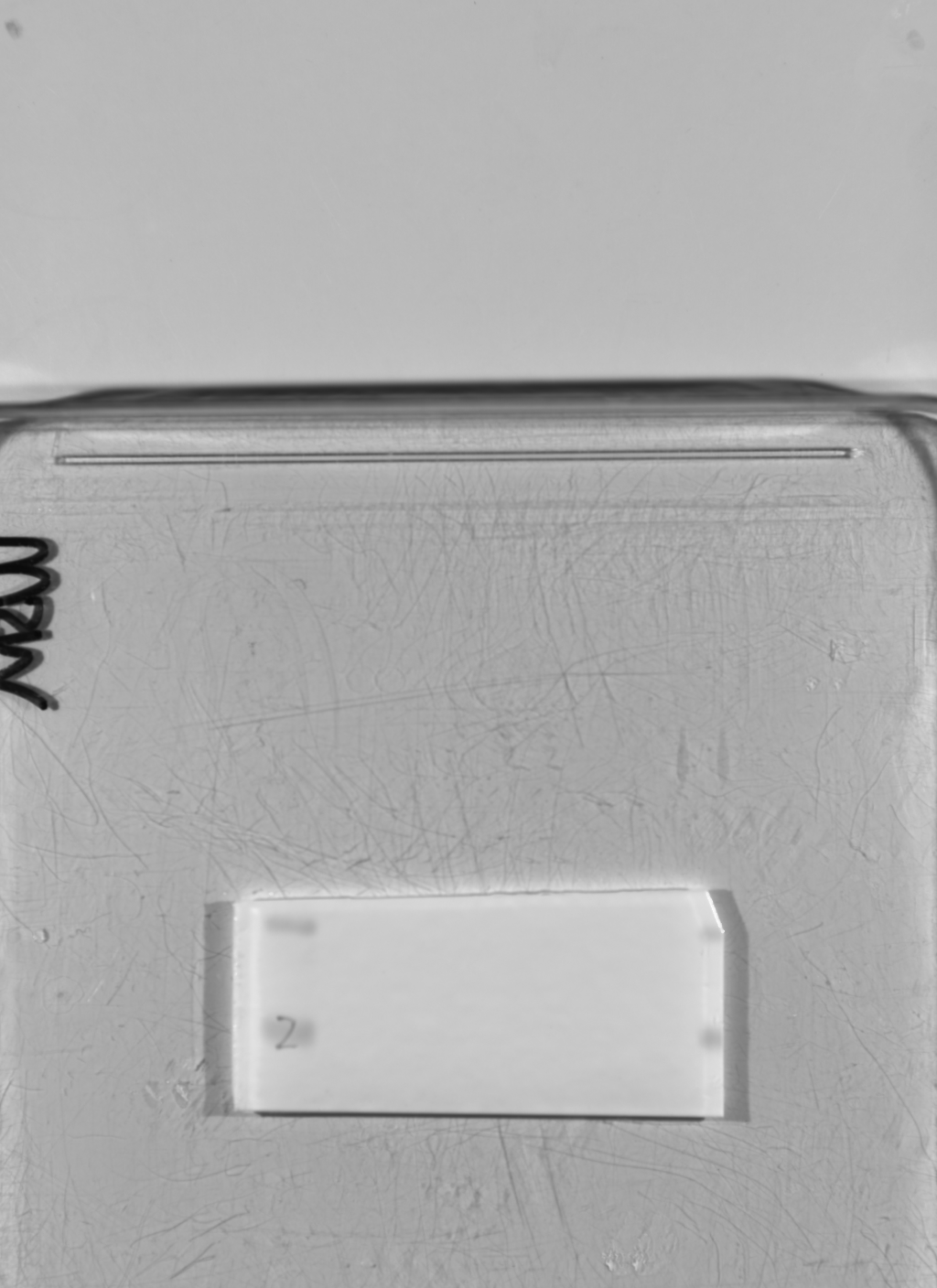

Supplement: Supplementary file 14 — EV Figure Source Data [file 44321_2024_60_MOESM14_ESM.zip › Figure EV2C Source Data/EV2C/88T YAPC/YAPC-Western γH2A 0.5/2-1 γH2A 0.5 _Ch-Marker.tif]

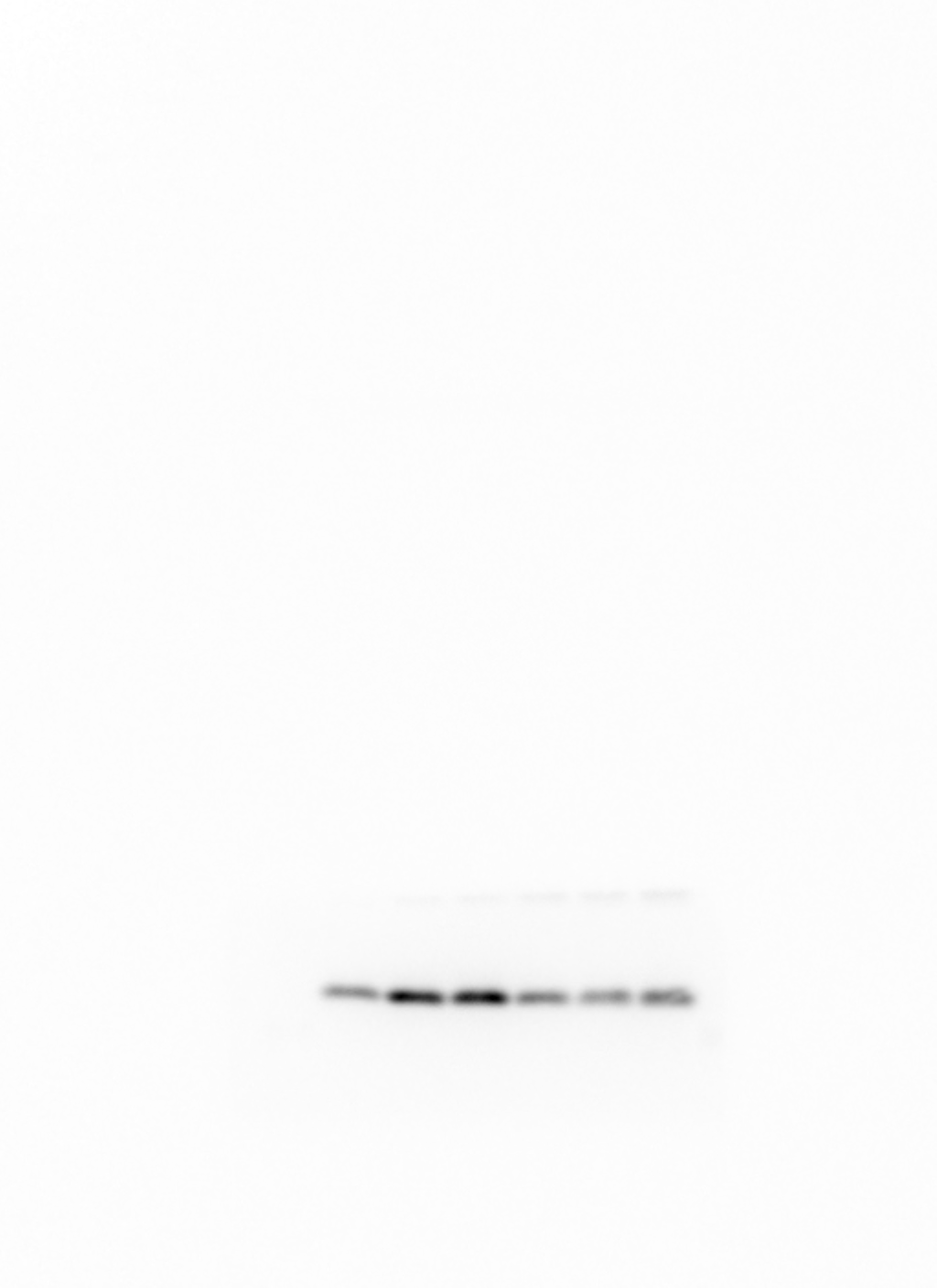

Supplement: Supplementary file 14 — EV Figure Source Data [file 44321_2024_60_MOESM14_ESM.zip › Figure EV2C Source Data/EV2C/88T YAPC/YAPC-Western γH2A 0.5/2-1 γH2A 0.5 _Ch.tif]

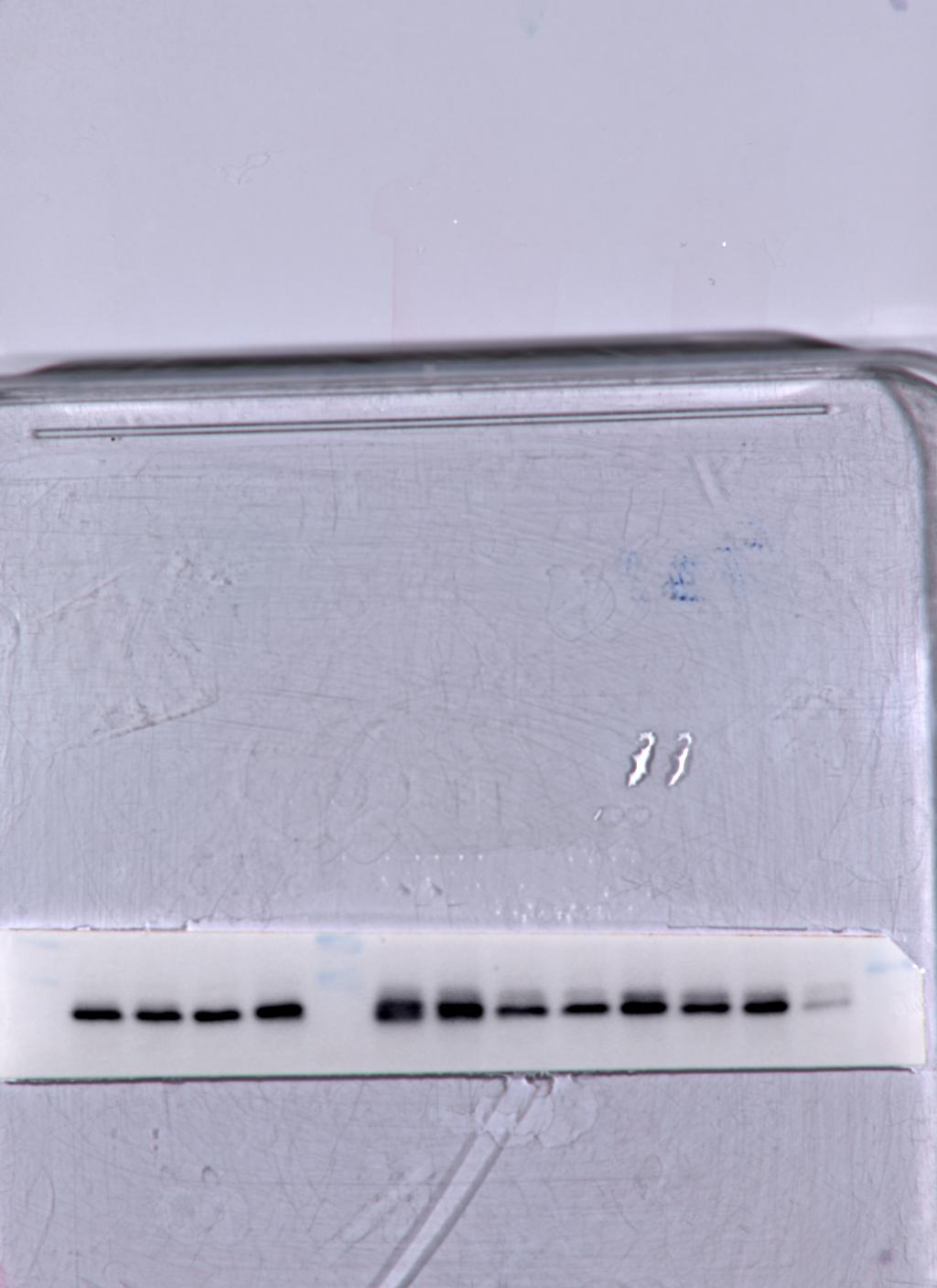

Supplement: Supplementary file 14 — EV Figure Source Data [file 44321_2024_60_MOESM14_ESM.zip › Figure EV2C Source Data/EV2C/CN1/Western CDK1 145.5/wsm 12 cdk1 145.5 _Ch+Marker.jpg]

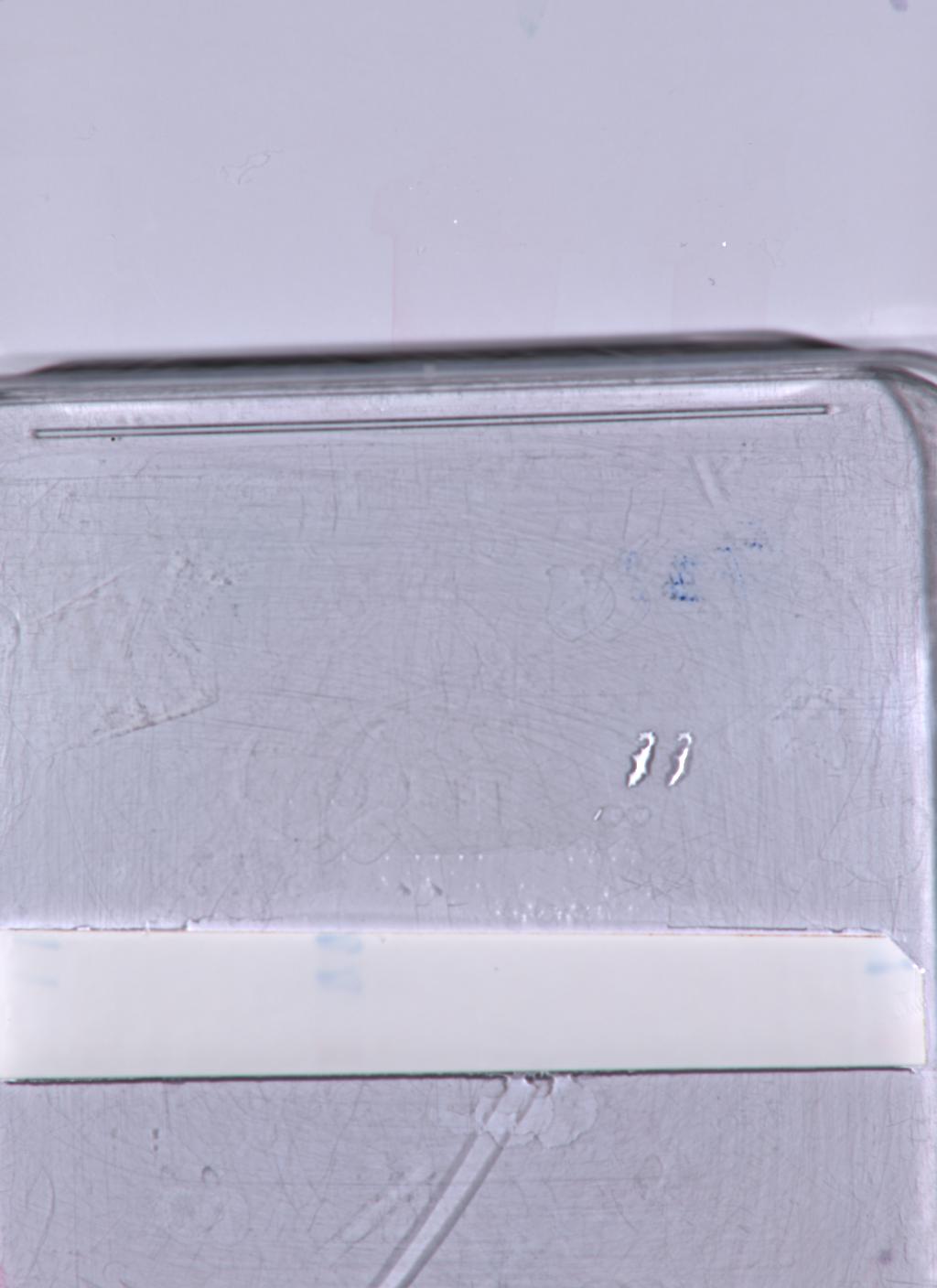

Supplement: Supplementary file 14 — EV Figure Source Data [file 44321_2024_60_MOESM14_ESM.zip › Figure EV2C Source Data/EV2C/CN1/Western CDK1 145.5/wsm 12 cdk1 145.5 _Ch-Marker.jpg]

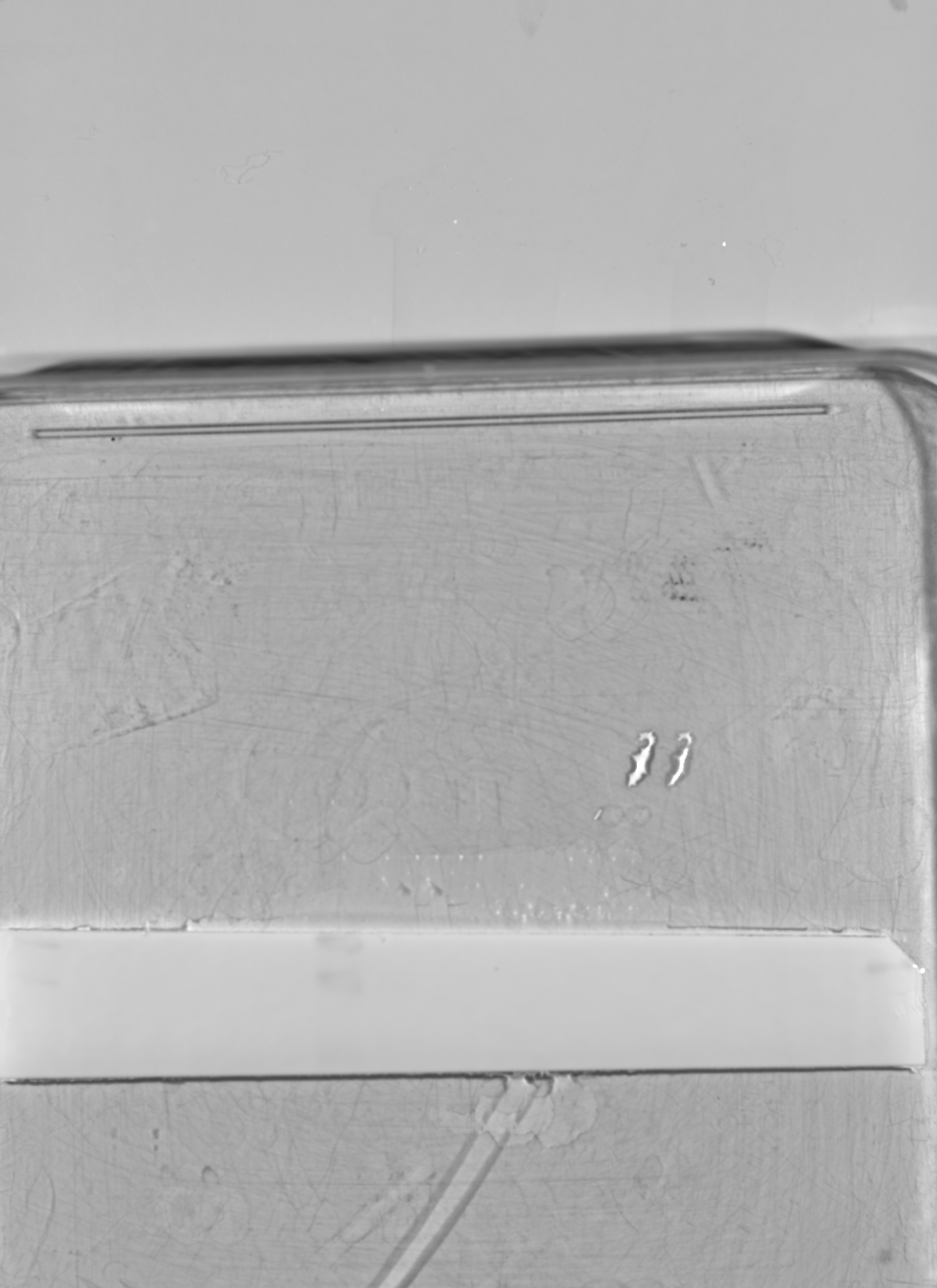

Supplement: Supplementary file 14 — EV Figure Source Data [file 44321_2024_60_MOESM14_ESM.zip › Figure EV2C Source Data/EV2C/CN1/Western CDK1 145.5/wsm 12 cdk1 145.5 _Ch-Marker.tif]

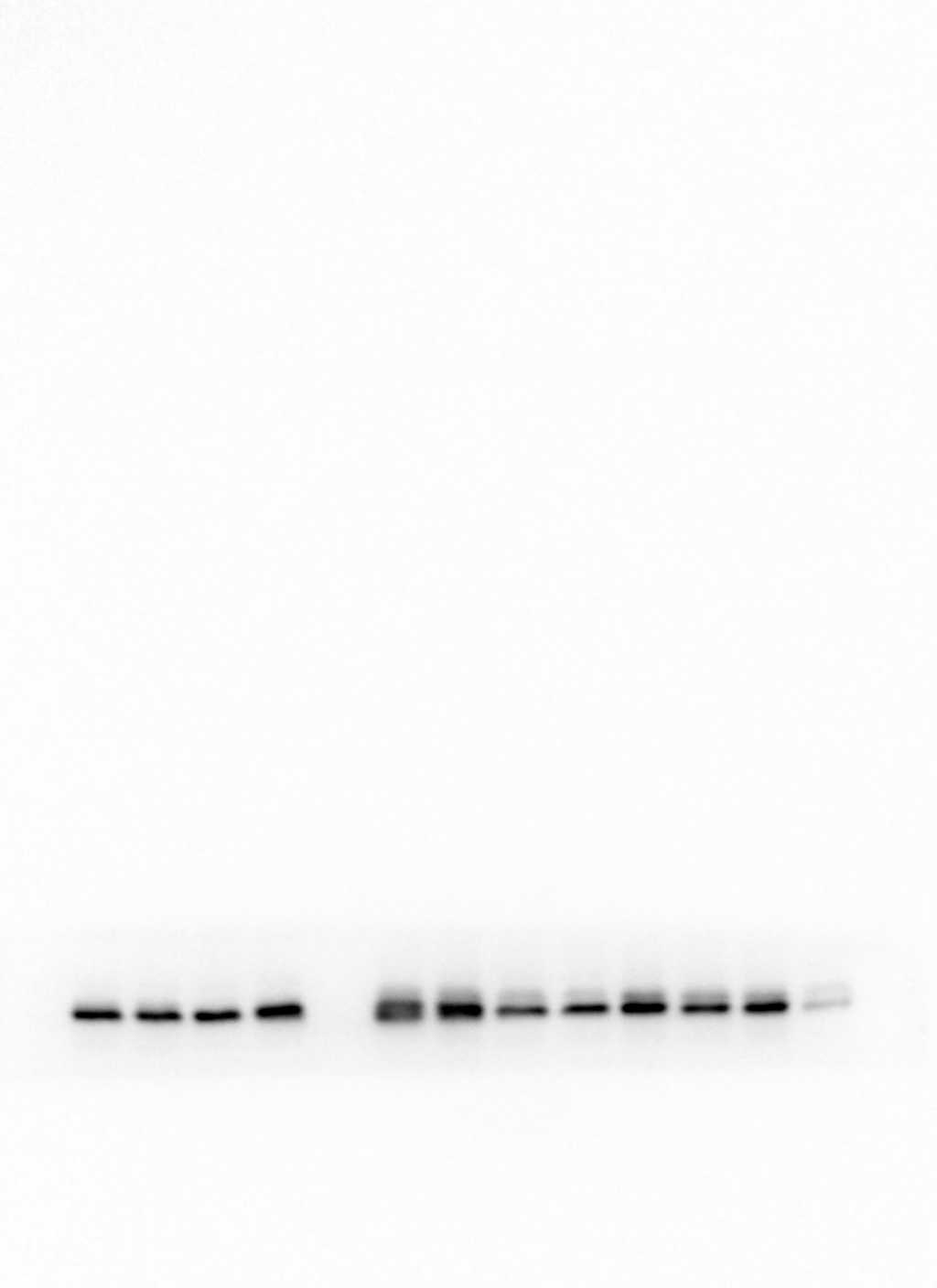

Supplement: Supplementary file 14 — EV Figure Source Data [file 44321_2024_60_MOESM14_ESM.zip › Figure EV2C Source Data/EV2C/CN1/Western CDK1 145.5/wsm 12 cdk1 145.5 _Ch.tif]

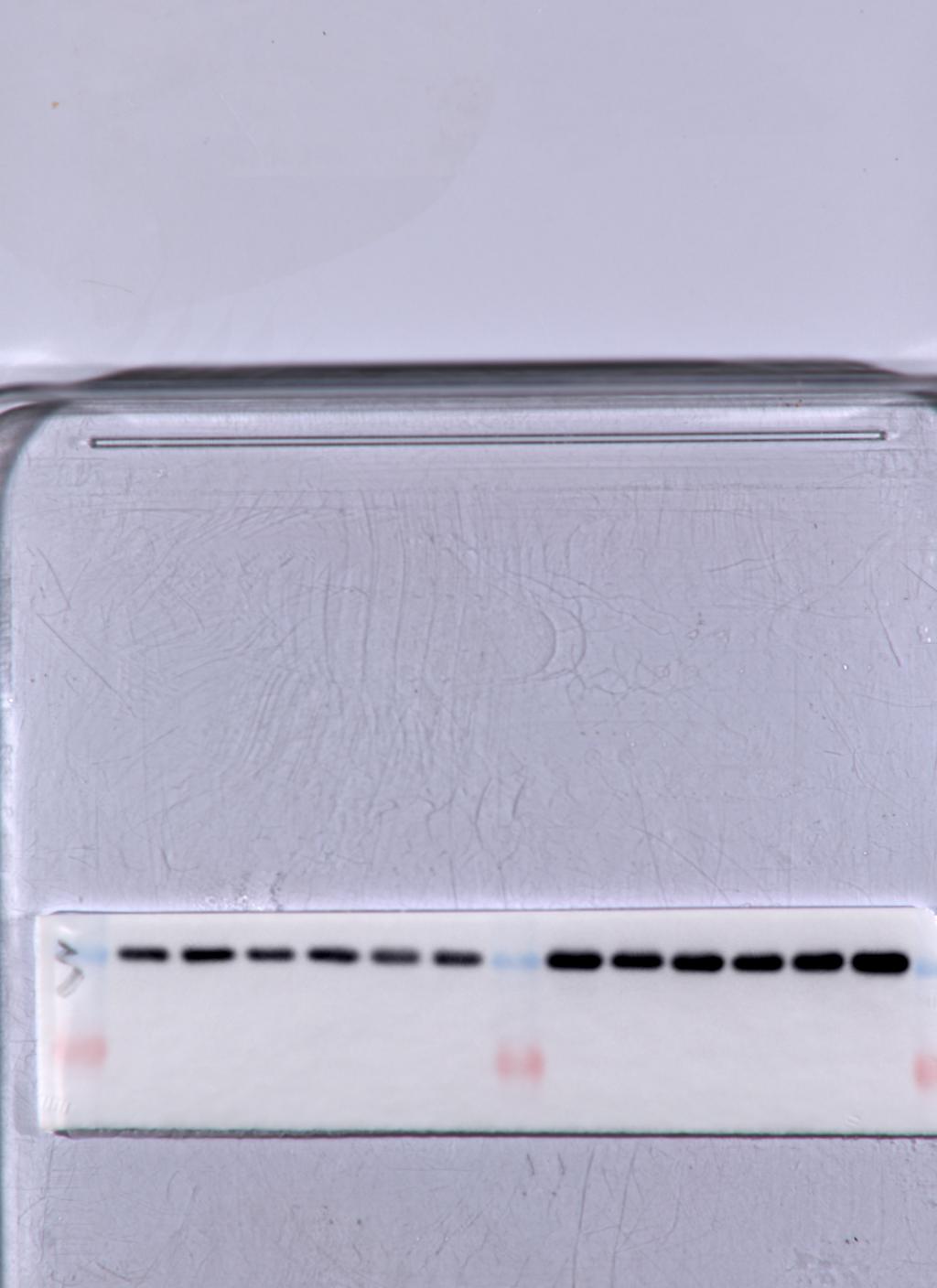

Supplement: Supplementary file 14 — EV Figure Source Data [file 44321_2024_60_MOESM14_ESM.zip › Figure EV2C Source Data/EV2C/CN1/Western GAP 0.3/3 2nd GAP 0.3 _Ch+Marker.jpg]

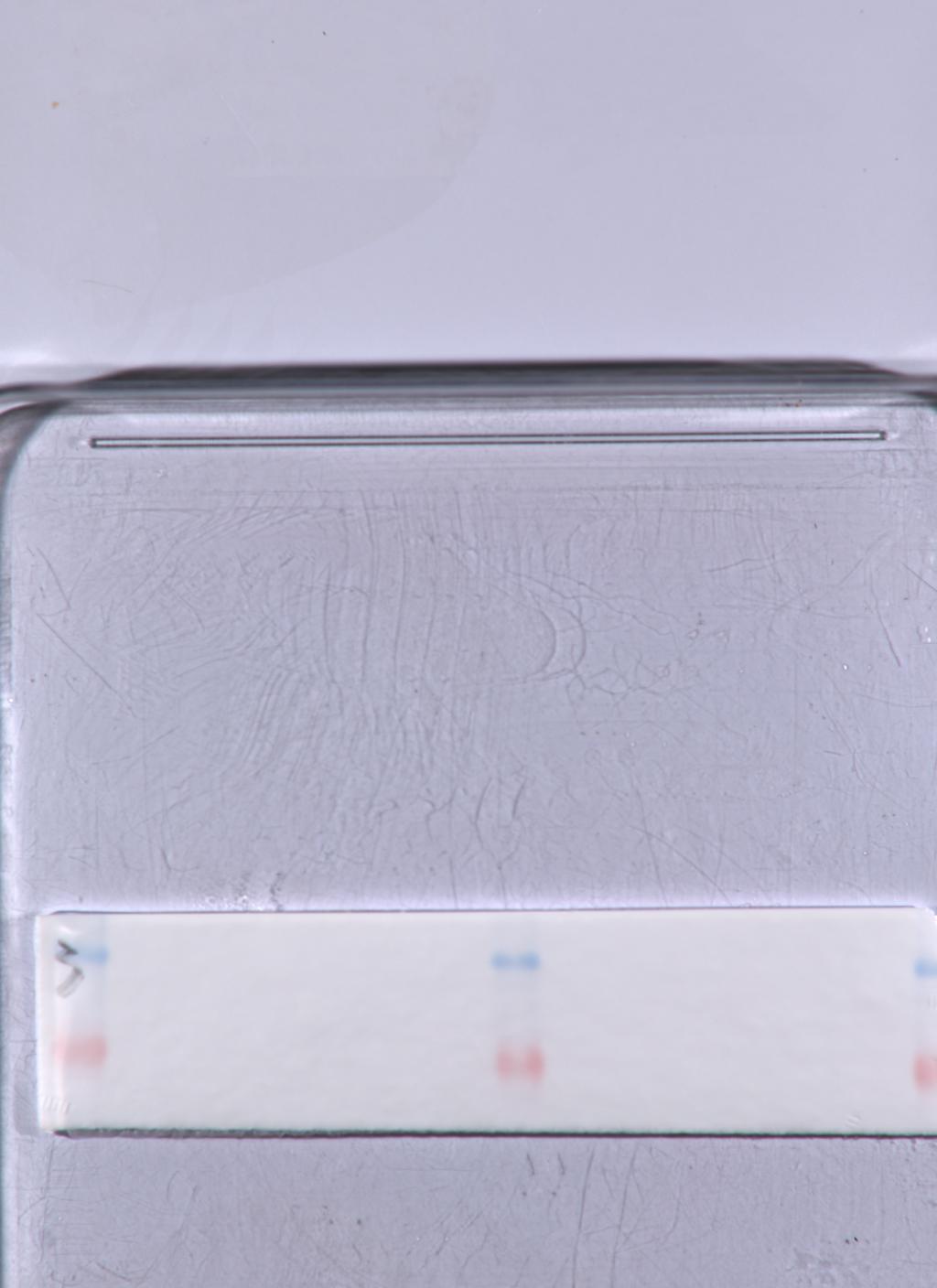

Supplement: Supplementary file 14 — EV Figure Source Data [file 44321_2024_60_MOESM14_ESM.zip › Figure EV2C Source Data/EV2C/CN1/Western GAP 0.3/3 2nd GAP 0.3 _Ch-Marker.jpg]

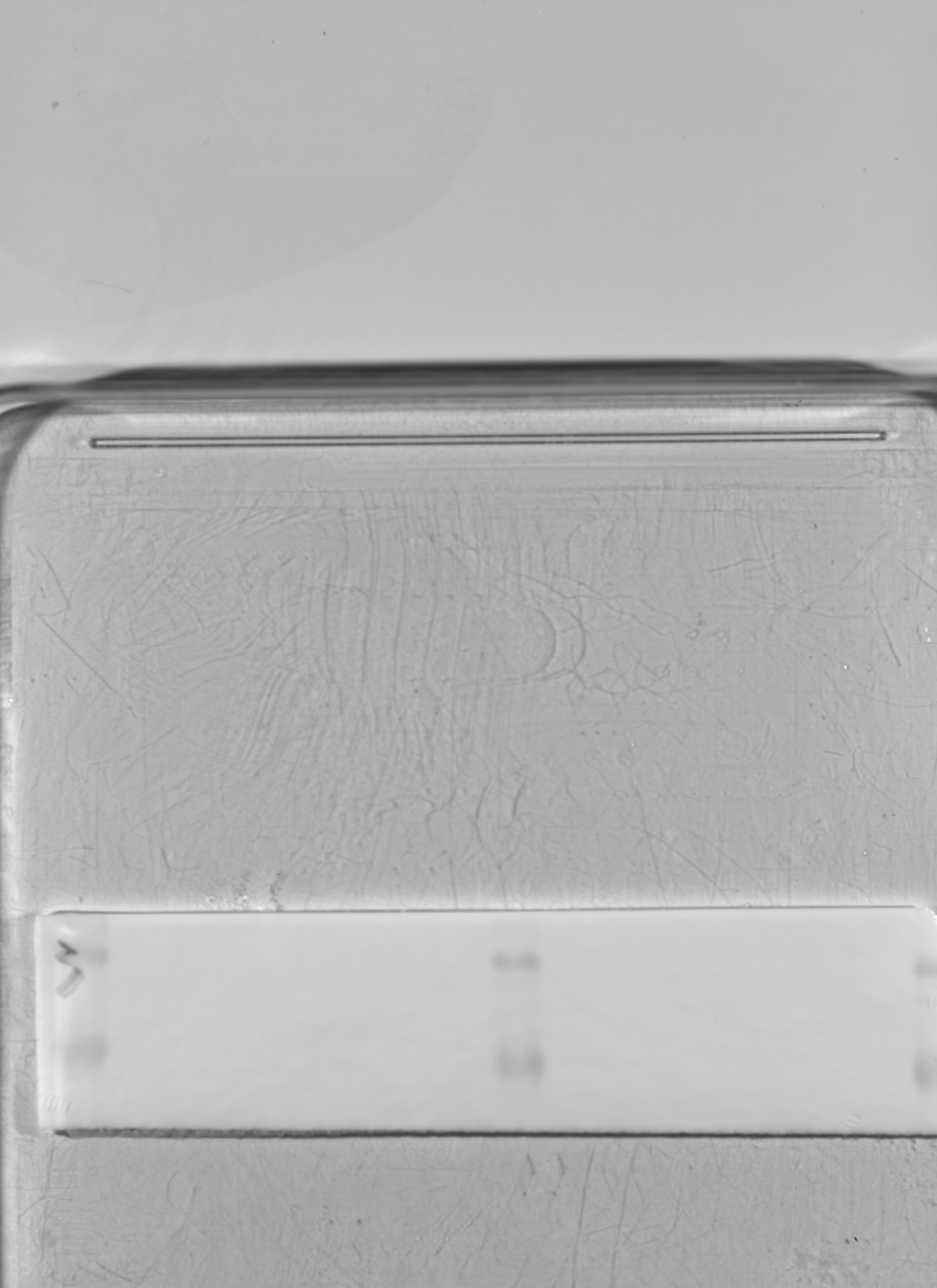

Supplement: Supplementary file 14 — EV Figure Source Data [file 44321_2024_60_MOESM14_ESM.zip › Figure EV2C Source Data/EV2C/CN1/Western GAP 0.3/3 2nd GAP 0.3 _Ch-Marker.tif]

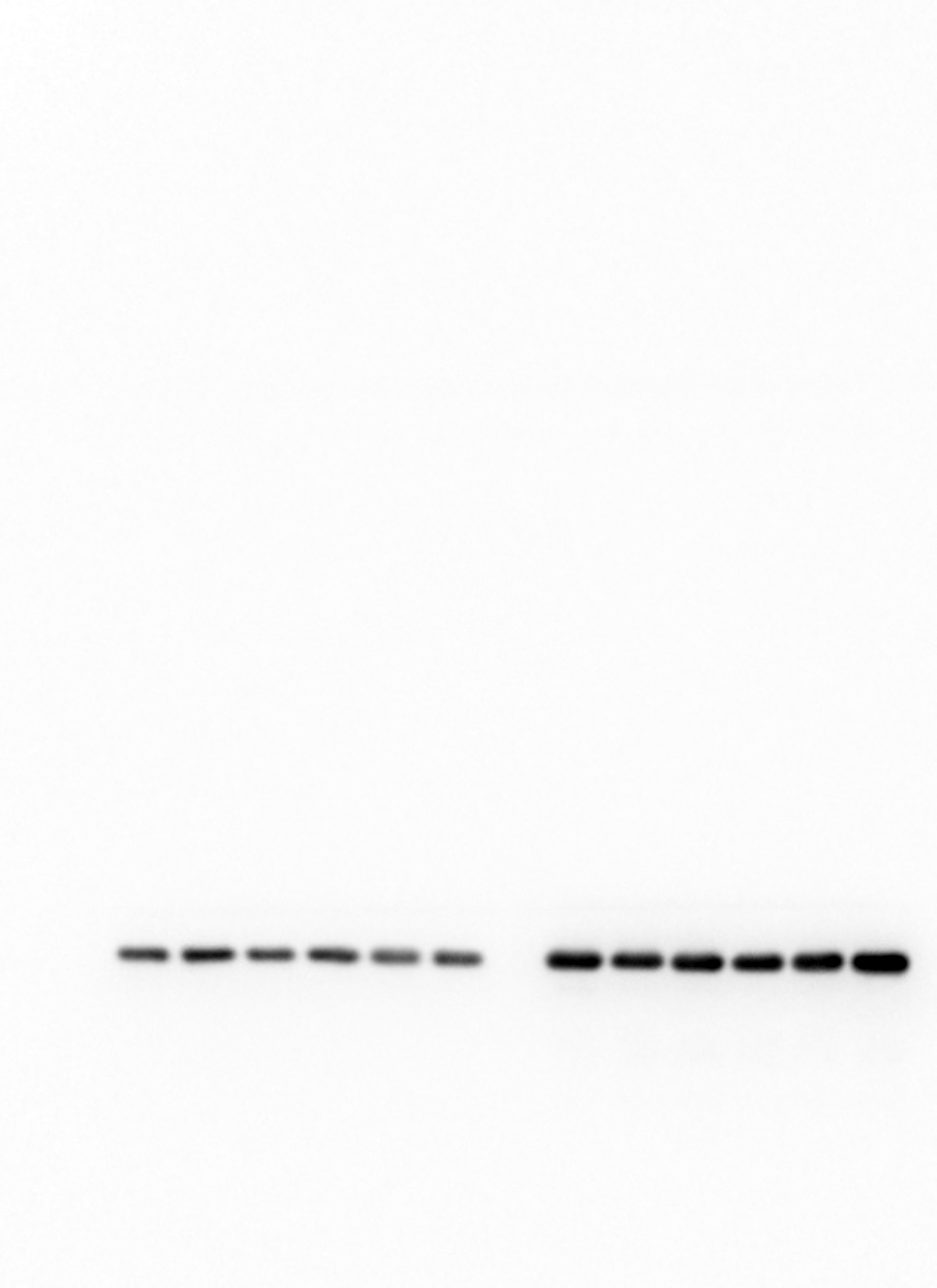

Supplement: Supplementary file 14 — EV Figure Source Data [file 44321_2024_60_MOESM14_ESM.zip › Figure EV2C Source Data/EV2C/CN1/Western GAP 0.3/3 2nd GAP 0.3 _Ch.tif]

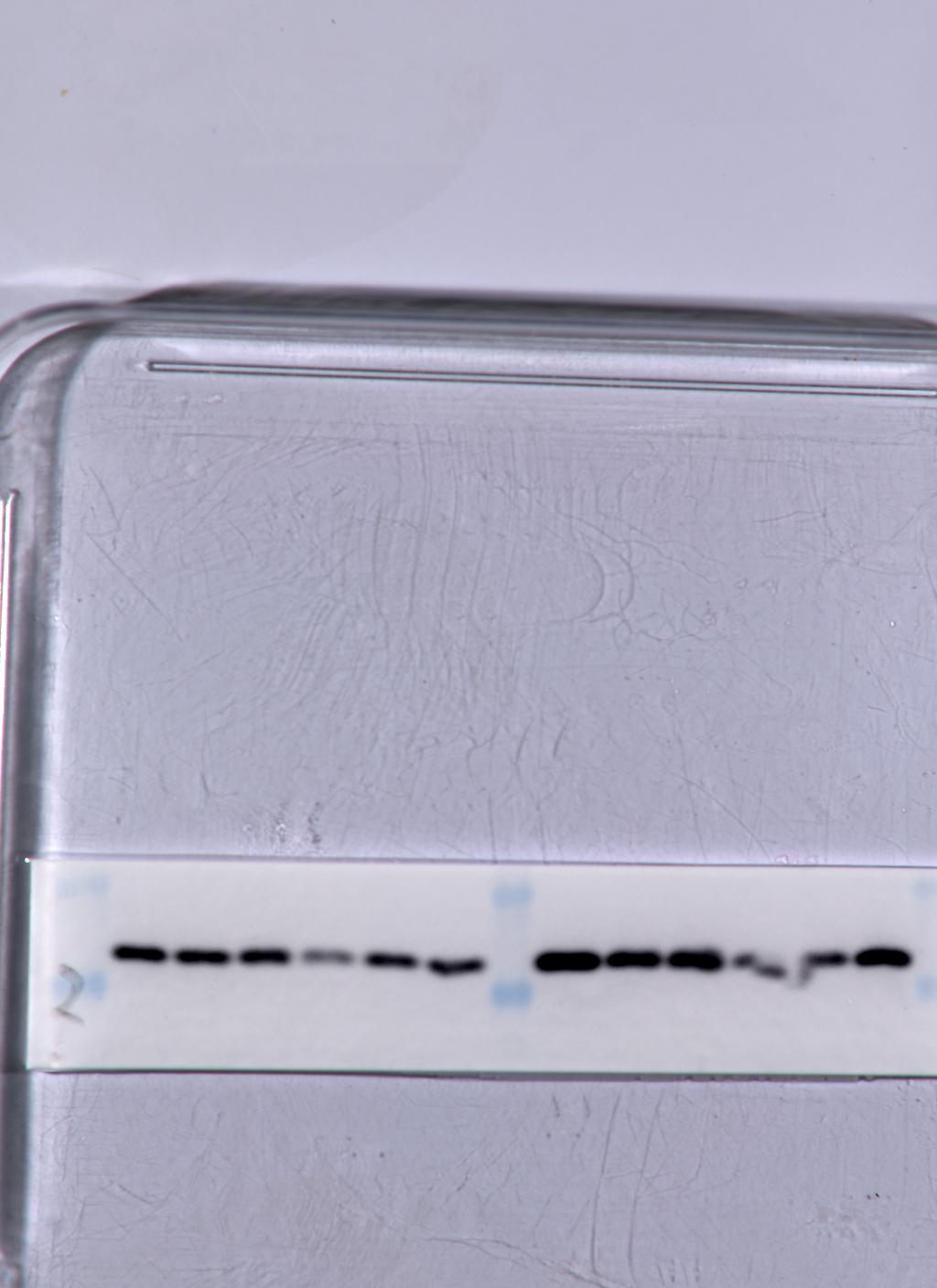

Supplement: Supplementary file 14 — EV Figure Source Data [file 44321_2024_60_MOESM14_ESM.zip › Figure EV2C Source Data/EV2C/CN1/Western H3 0.2/2 H3 0.2 2022.08.19_19.34.59_Ch+Marker.jpg]

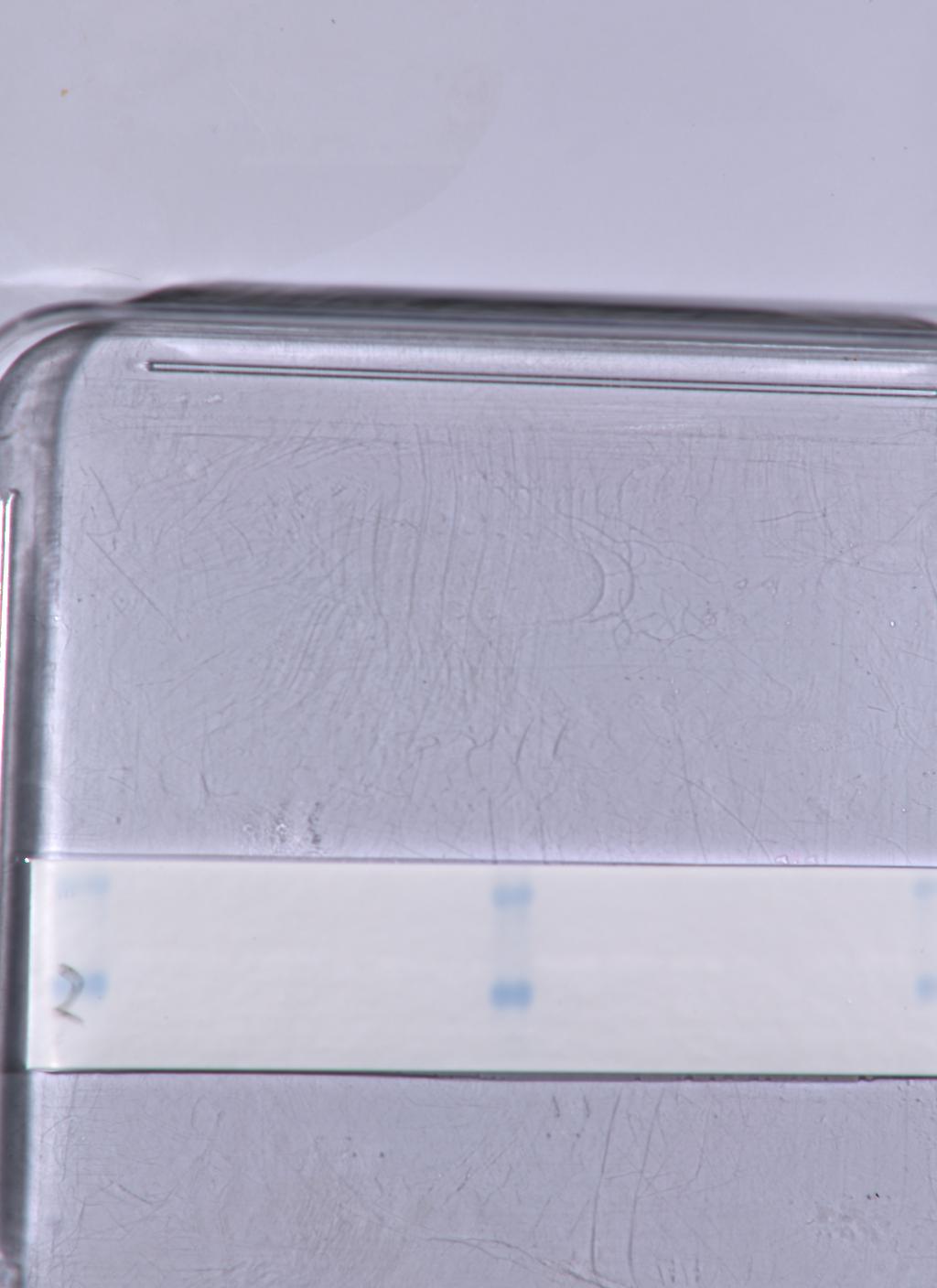

Supplement: Supplementary file 14 — EV Figure Source Data [file 44321_2024_60_MOESM14_ESM.zip › Figure EV2C Source Data/EV2C/CN1/Western H3 0.2/2 H3 0.2 2022.08.19_19.34.59_Ch-Marker.jpg]

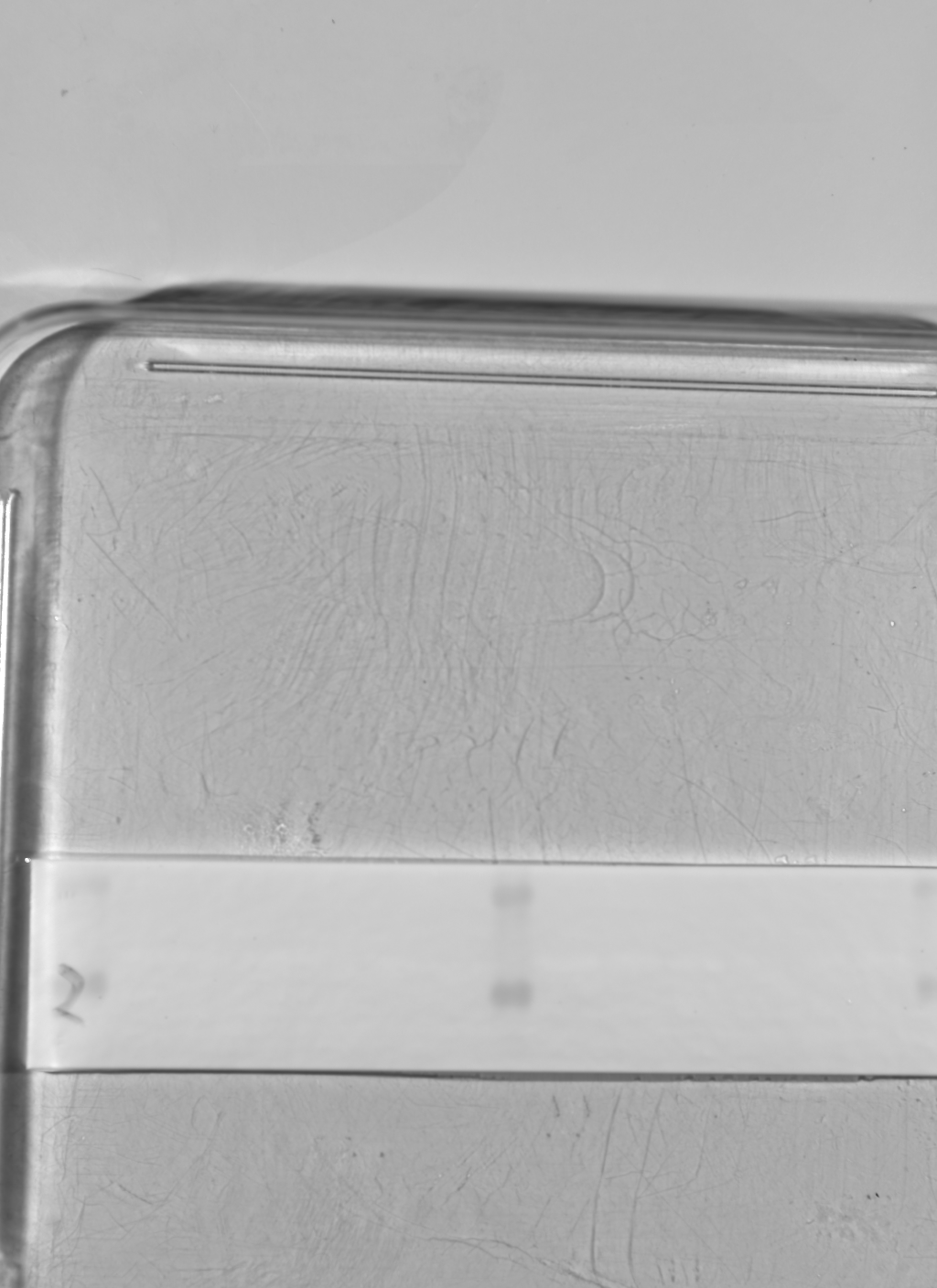

Supplement: Supplementary file 14 — EV Figure Source Data [file 44321_2024_60_MOESM14_ESM.zip › Figure EV2C Source Data/EV2C/CN1/Western H3 0.2/2 H3 0.2 2022.08.19_19.34.59_Ch-Marker.tif]

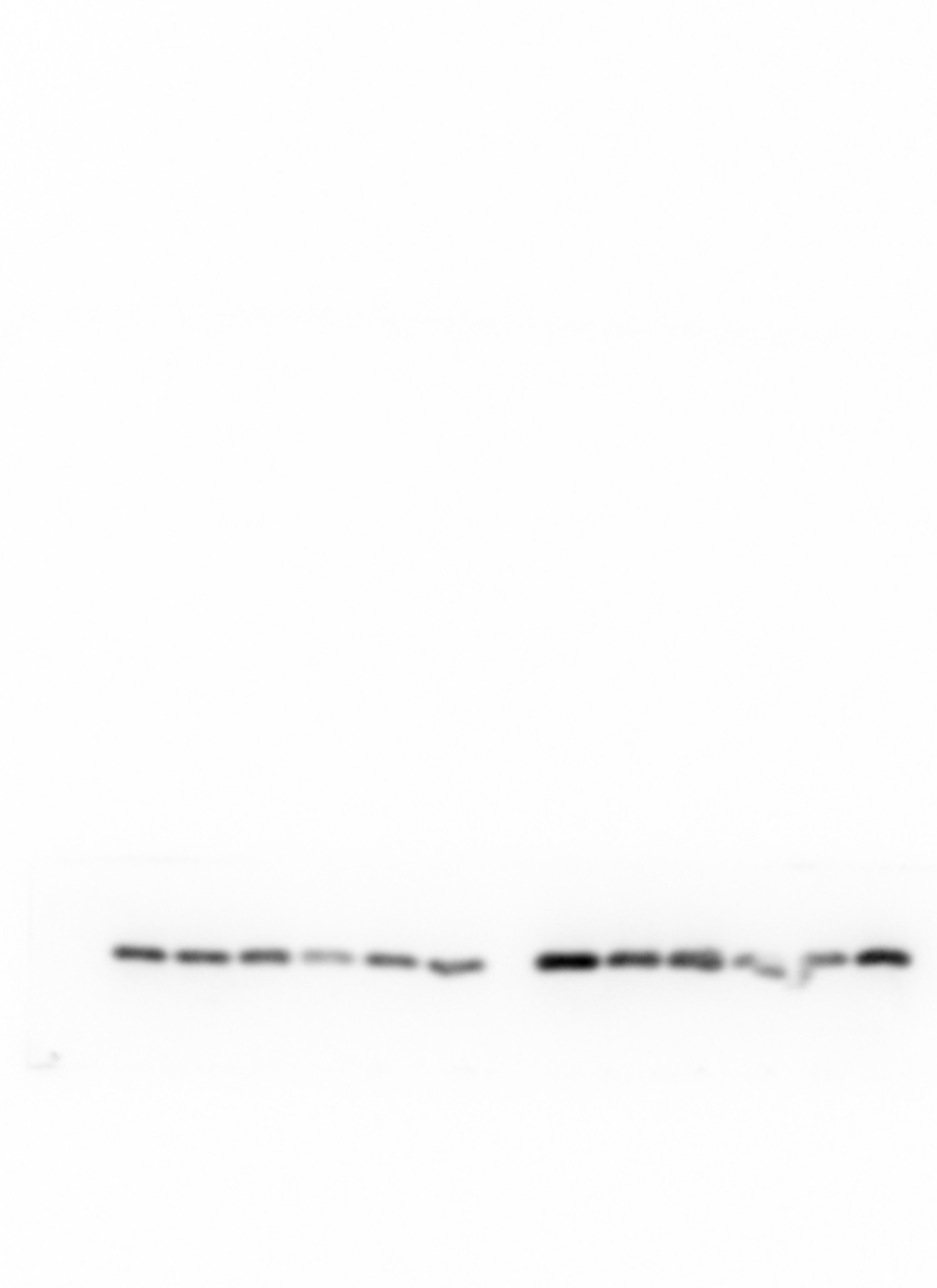

Supplement: Supplementary file 14 — EV Figure Source Data [file 44321_2024_60_MOESM14_ESM.zip › Figure EV2C Source Data/EV2C/CN1/Western H3 0.2/2 H3 0.2 2022.08.19_19.34.59_Ch.tif]

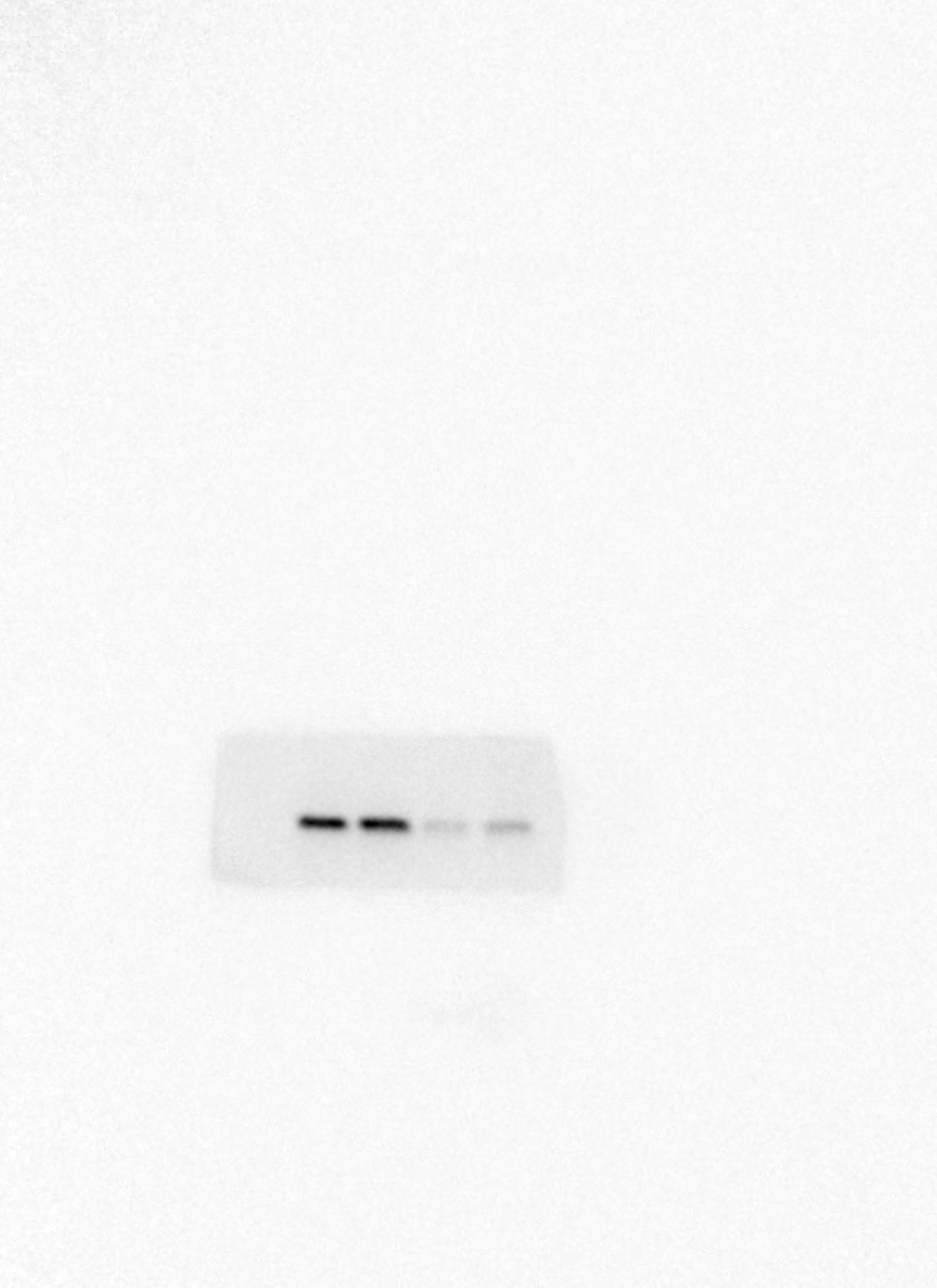

Supplement: Supplementary file 14 — EV Figure Source Data [file 44321_2024_60_MOESM14_ESM.zip › Figure EV2C Source Data/EV2C/CN1/Western phoCDK1-T14 120/wsm 1 phoCDK1-T14 120 _Ch.tif]

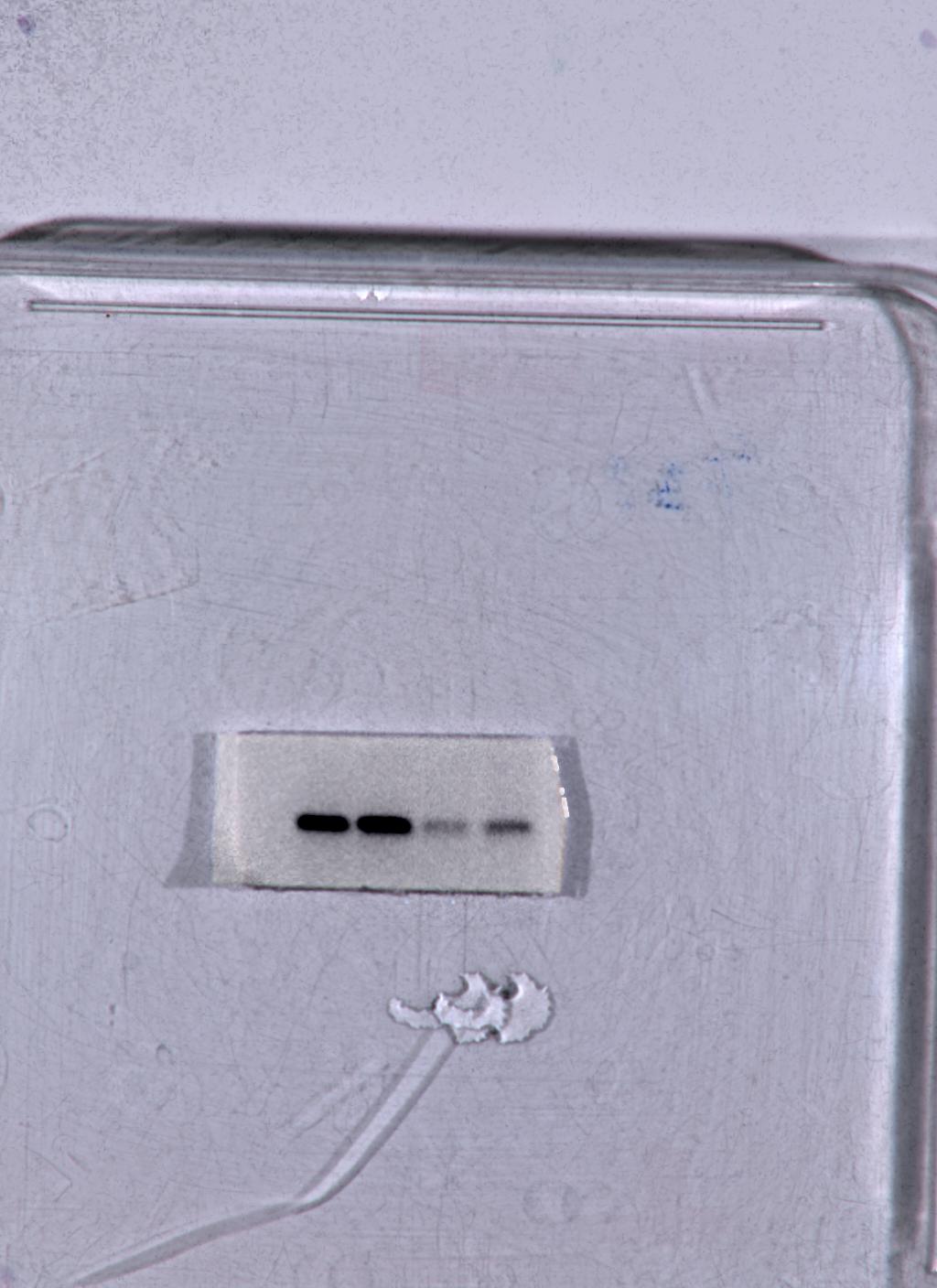

Supplement: Supplementary file 14 — EV Figure Source Data [file 44321_2024_60_MOESM14_ESM.zip › Figure EV2C Source Data/EV2C/CN1/Western phoCDK1-T14 120/wsm 1 phoCDK1-T14 120 _Ch+Marker.jpg]

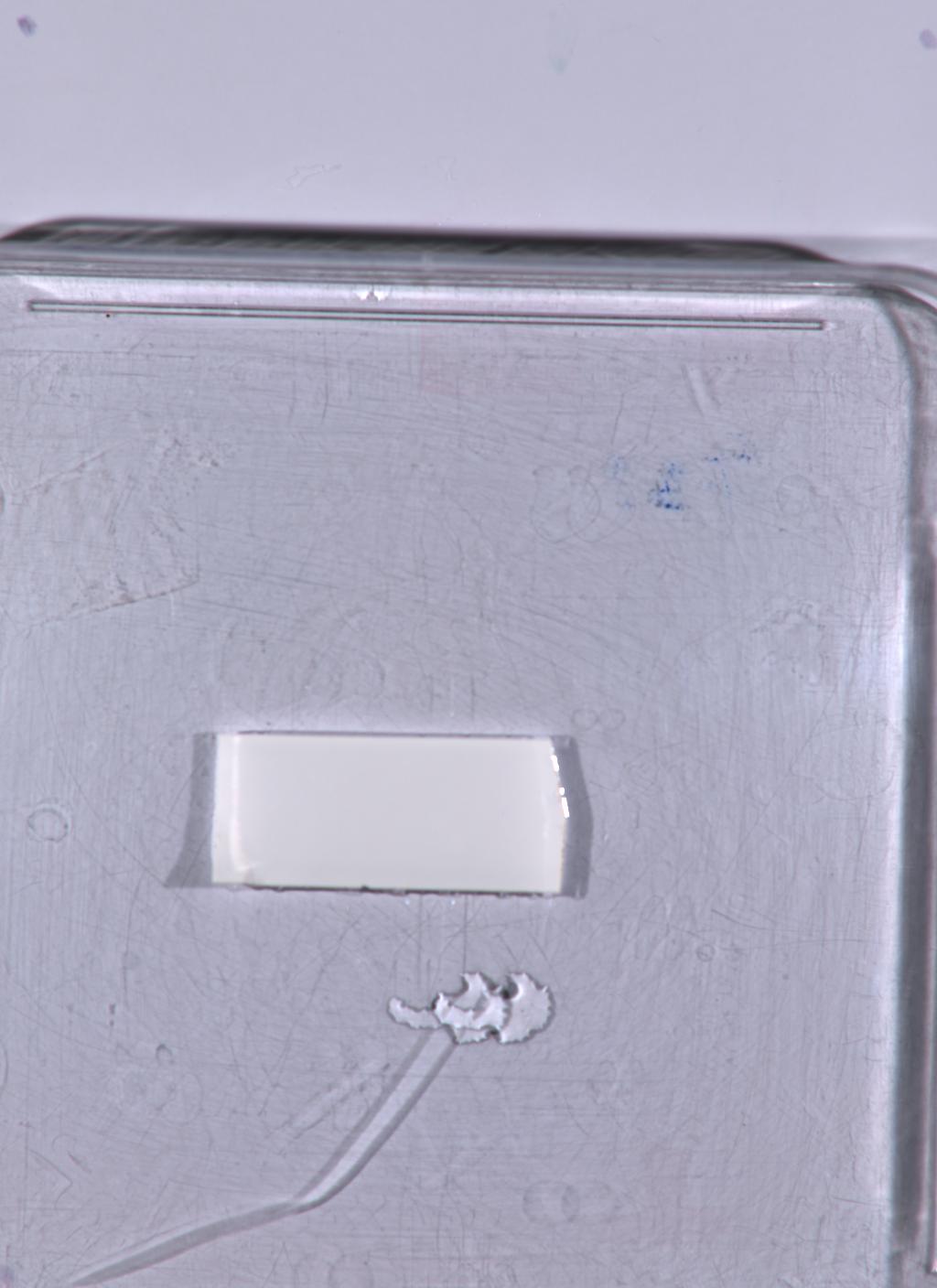

Supplement: Supplementary file 14 — EV Figure Source Data [file 44321_2024_60_MOESM14_ESM.zip › Figure EV2C Source Data/EV2C/CN1/Western phoCDK1-T14 120/wsm 1 phoCDK1-T14 120 _Ch-Marker.jpg]

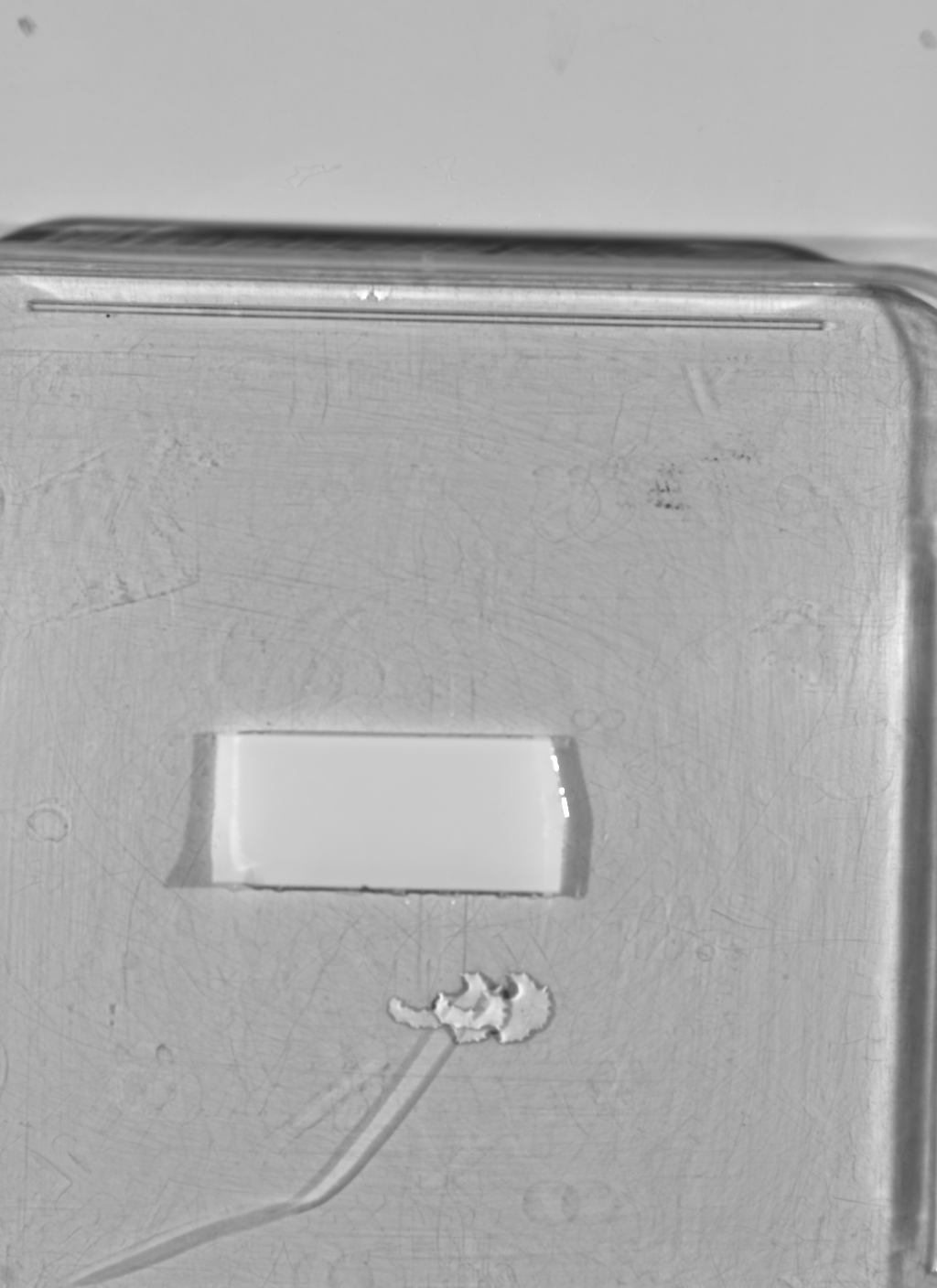

Supplement: Supplementary file 14 — EV Figure Source Data [file 44321_2024_60_MOESM14_ESM.zip › Figure EV2C Source Data/EV2C/CN1/Western phoCDK1-T14 120/wsm 1 phoCDK1-T14 120 _Ch-Marker.tif]

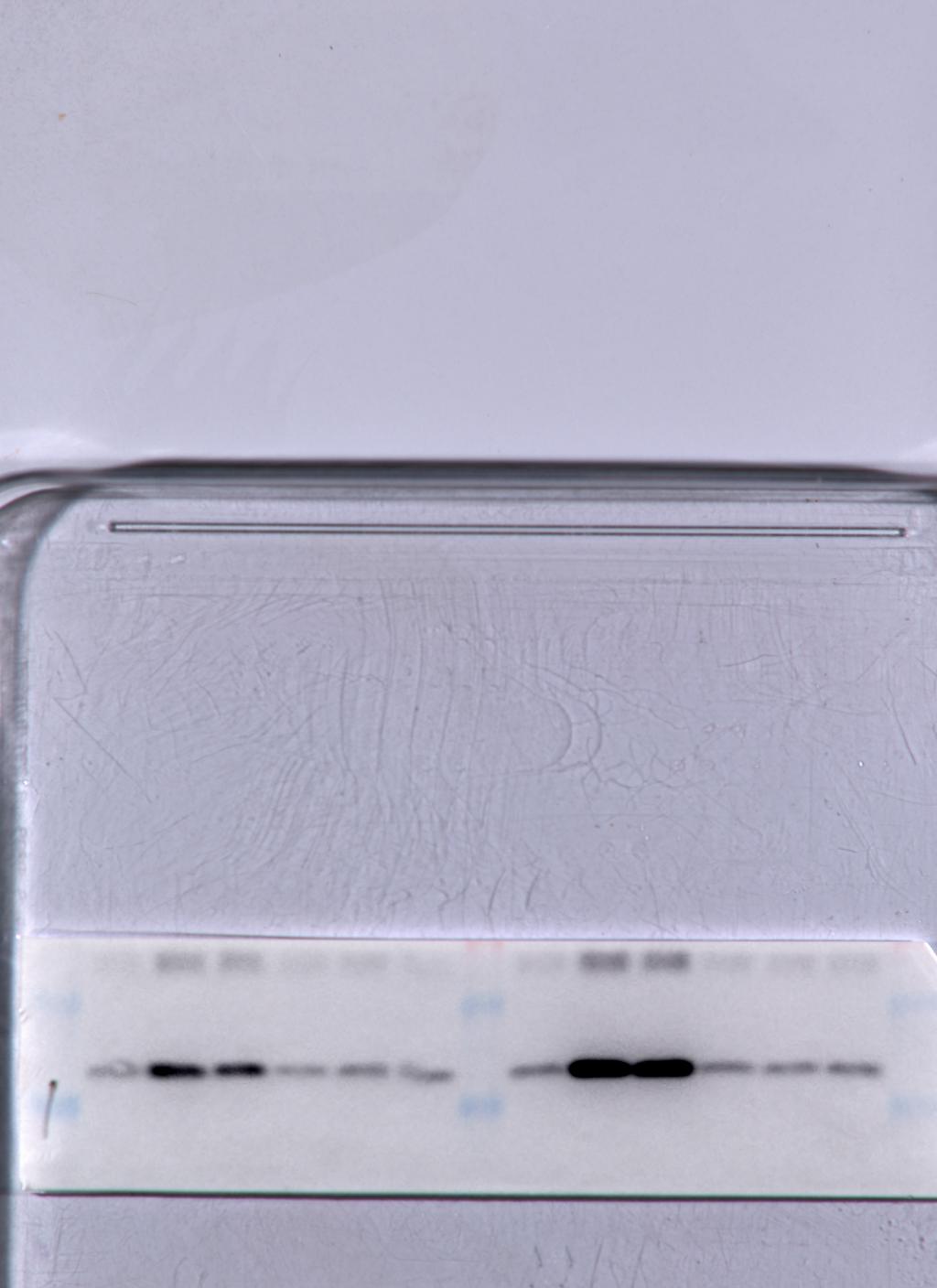

Supplement: Supplementary file 14 — EV Figure Source Data [file 44321_2024_60_MOESM14_ESM.zip › Figure EV2C Source Data/EV2C/CN1/Western γH2A 9/1 γH2A 9 _Ch+Marker.jpg]

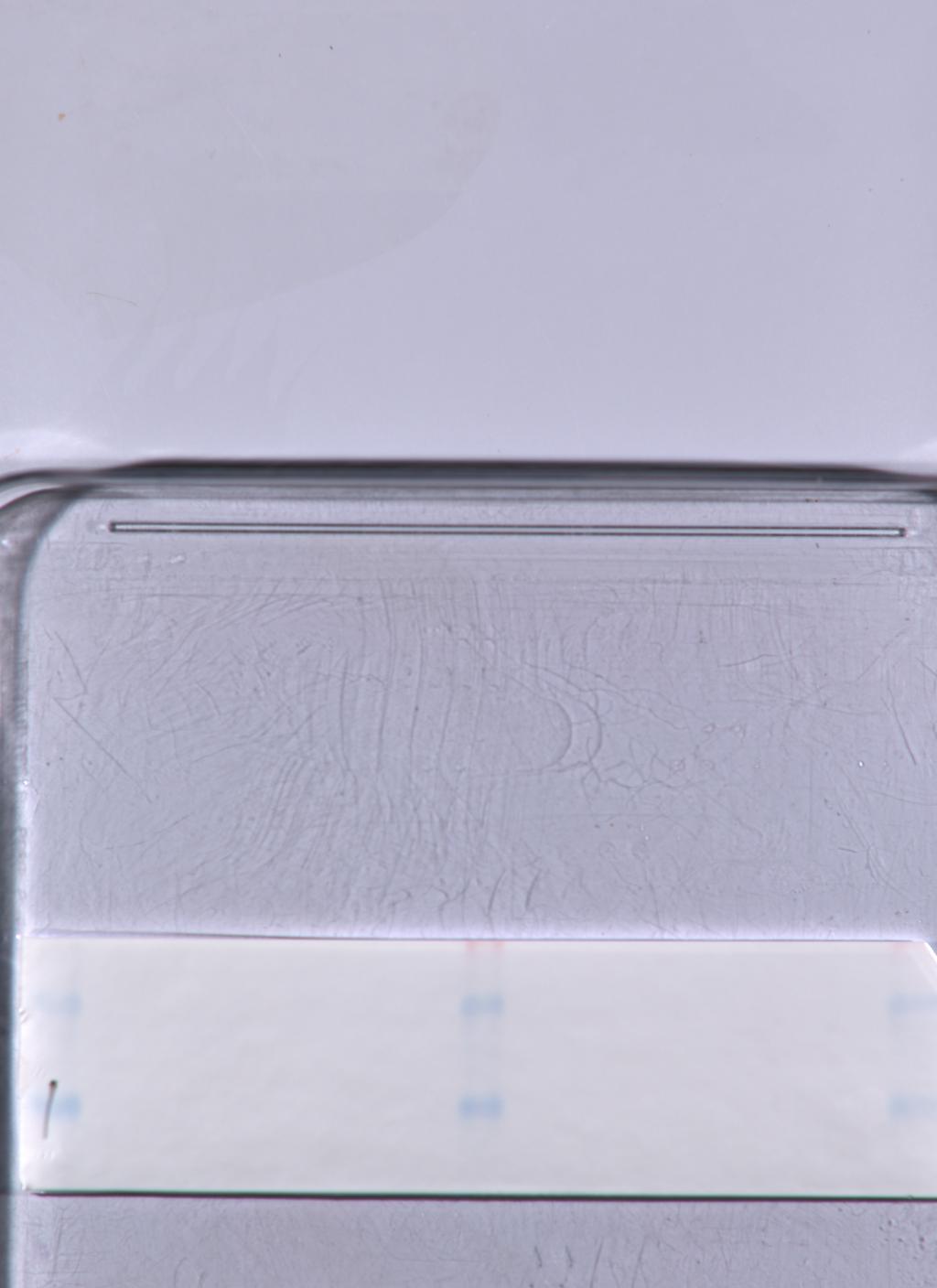

Supplement: Supplementary file 14 — EV Figure Source Data [file 44321_2024_60_MOESM14_ESM.zip › Figure EV2C Source Data/EV2C/CN1/Western γH2A 9/1 γH2A 9 _Ch-Marker.jpg]

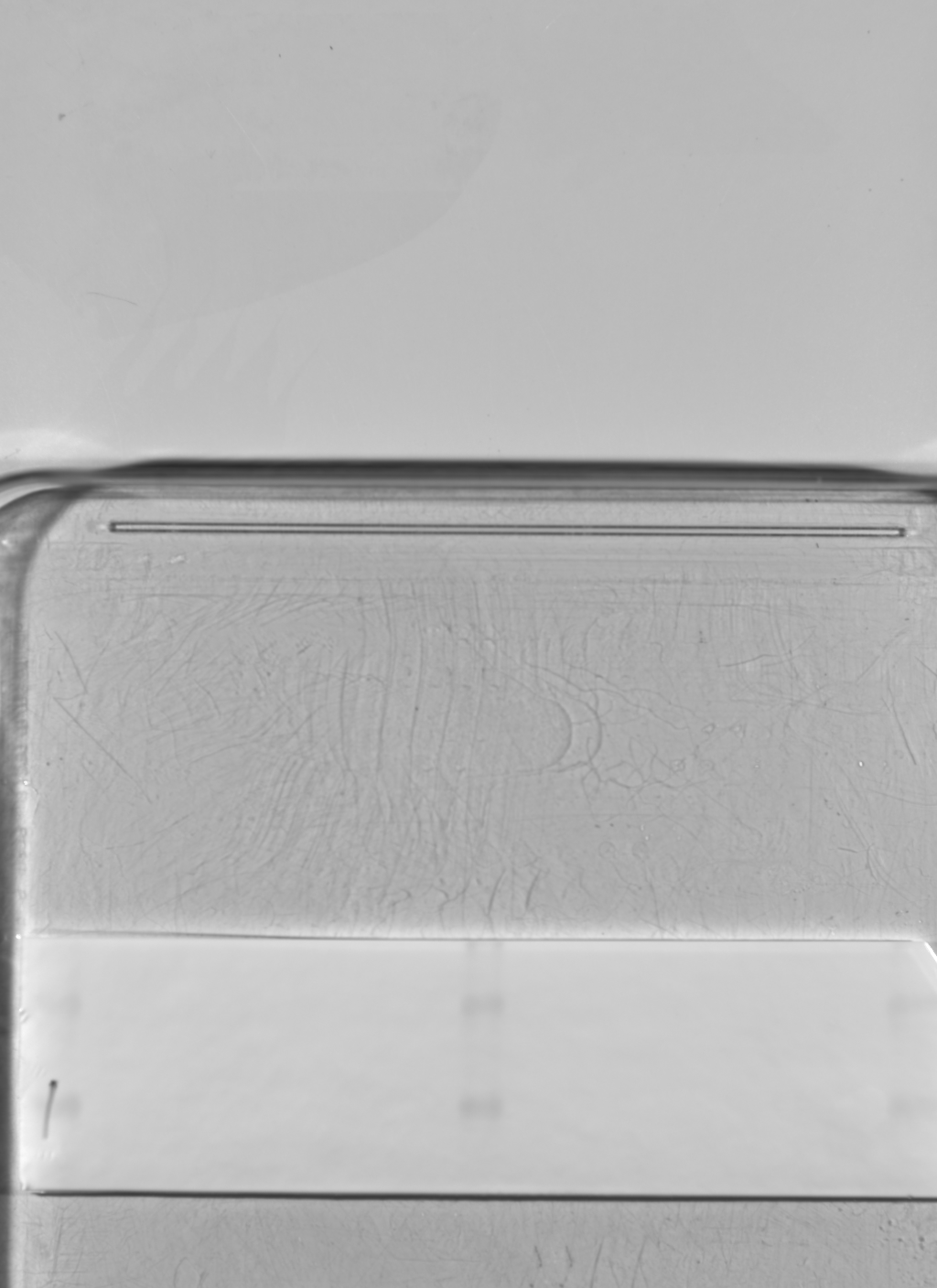

Supplement: Supplementary file 14 — EV Figure Source Data [file 44321_2024_60_MOESM14_ESM.zip › Figure EV2C Source Data/EV2C/CN1/Western γH2A 9/1 γH2A 9 _Ch-Marker.tif]

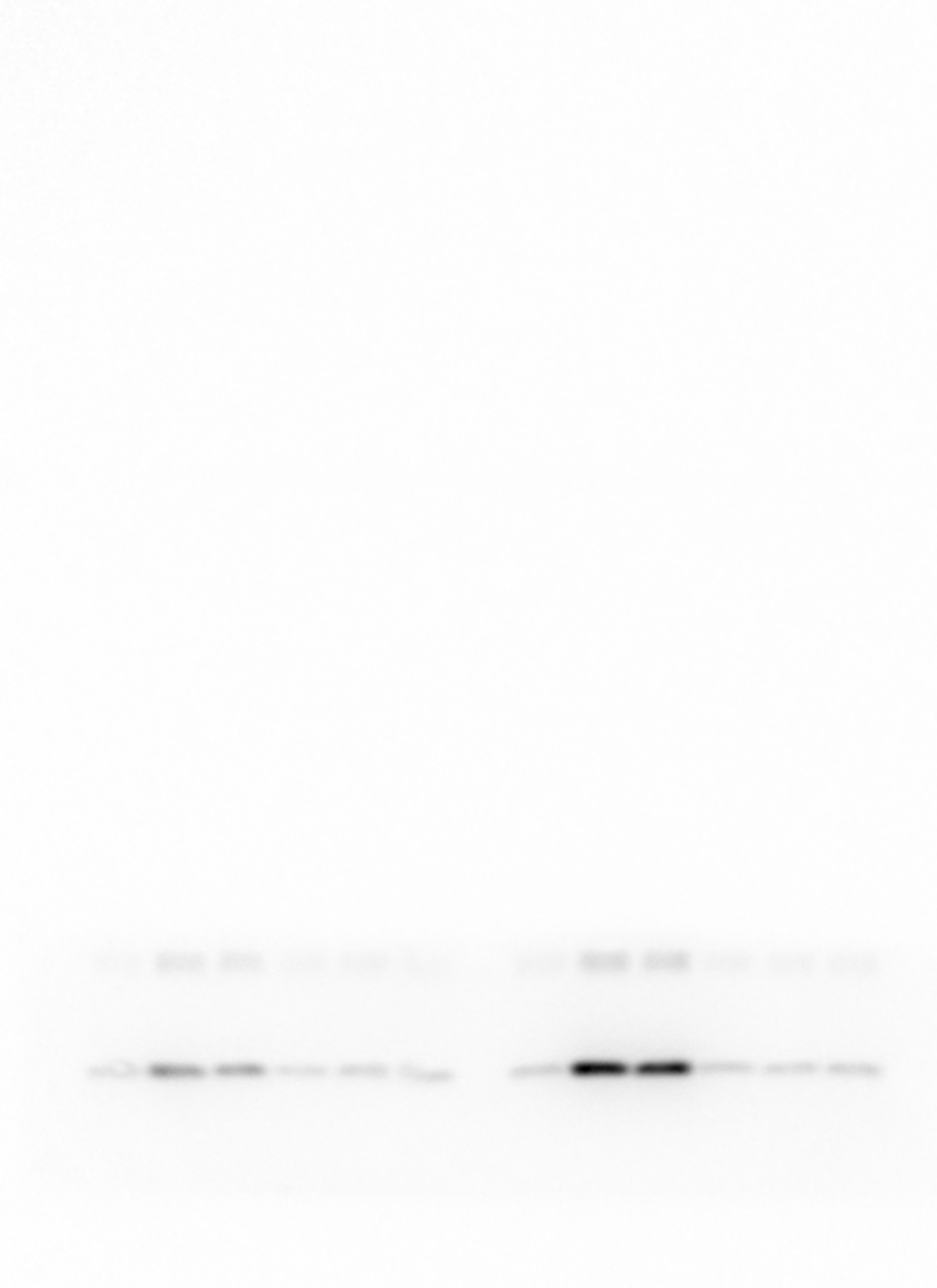

Supplement: Supplementary file 14 — EV Figure Source Data [file 44321_2024_60_MOESM14_ESM.zip › Figure EV2C Source Data/EV2C/CN1/Western γH2A 9/1 γH2A 9 _Ch.tif]
