# Supplementary figures and images for: Genome-wide CRISPR screens identify PKMYT1 as a therapeutic target in pancreatic ductal adenocarcinoma (part 3 of 4)
Source: EMBO Mol Med. 2024 Apr 3;16(5):5. doi: 10.1038/s44321-024-00060-y (PMC11099189; doi:10.1038/s44321-024-00060-y)

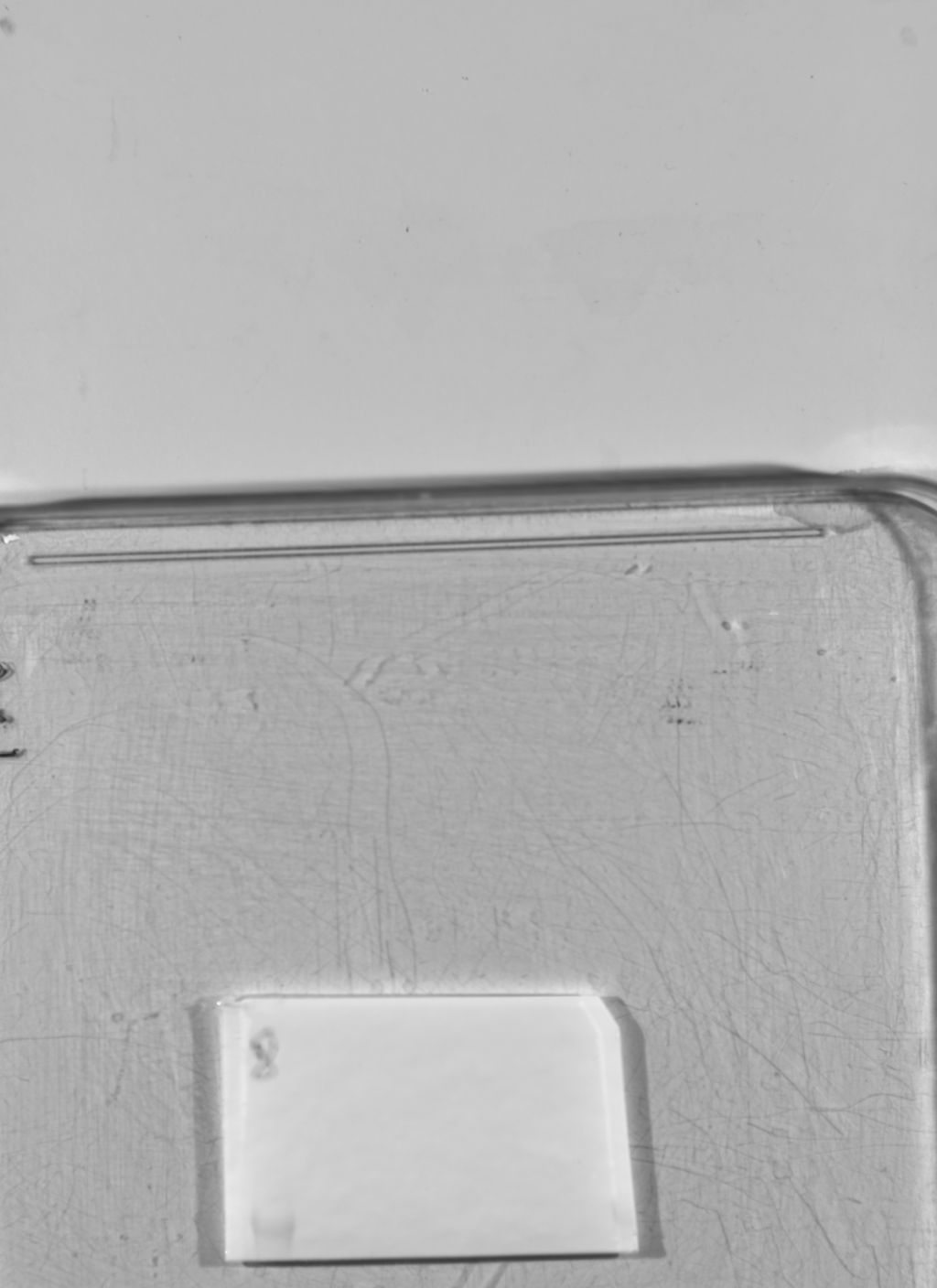

Supplement: Supplementary file 11 — Source data Fig. 6 [file 44321_2024_60_MOESM11_ESM.zip › Figure 6/6C/88T/Western phoPRKDC 1/8-1 1st phoPRK 1Q _Ch-Marker.tif]

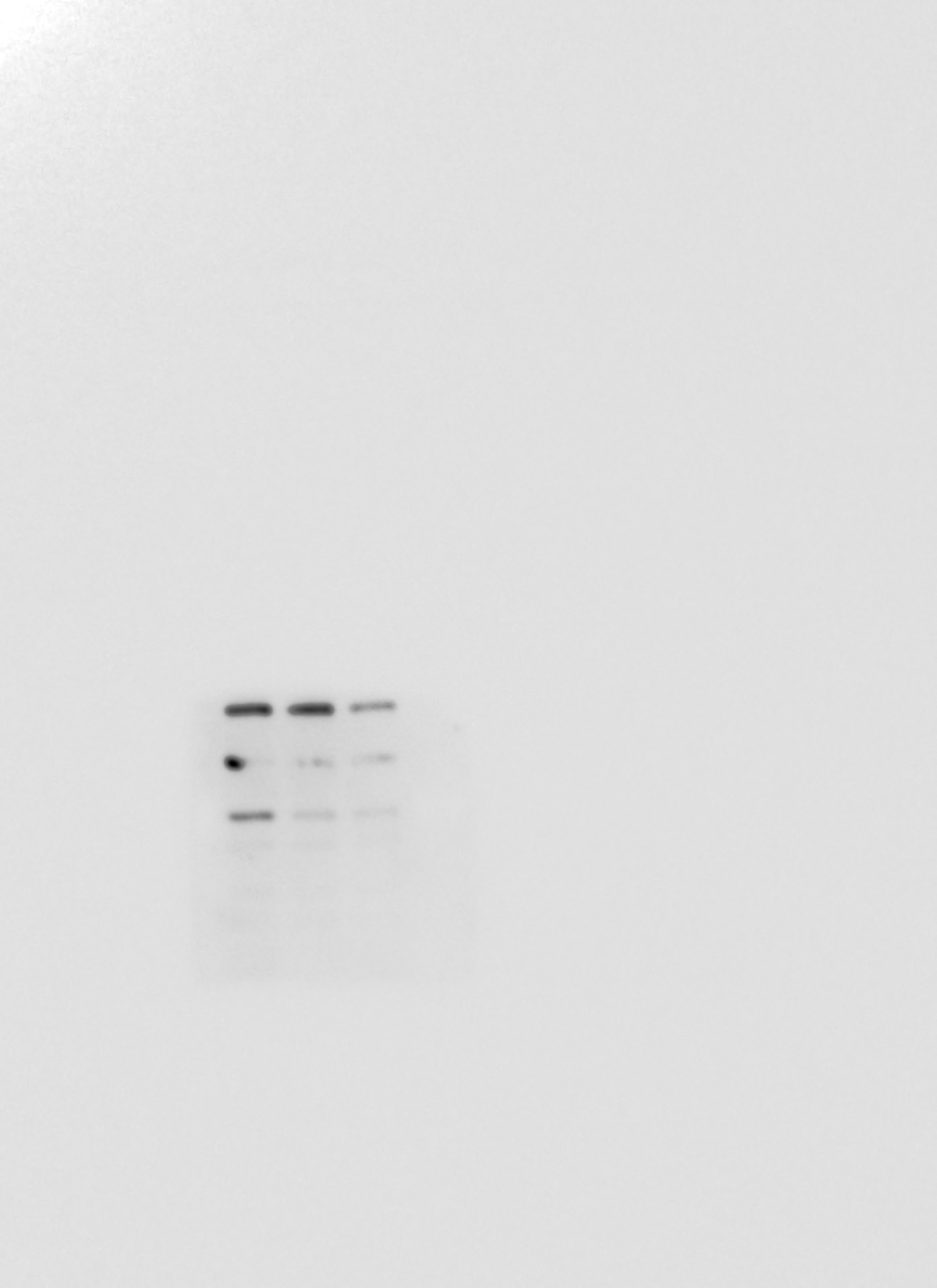

Supplement: Supplementary file 11 — Source data Fig. 6 [file 44321_2024_60_MOESM11_ESM.zip › Figure 6/6C/88T/Western PLK 5.1/4-2 2nd PLK 5.1 _Ch.tif]

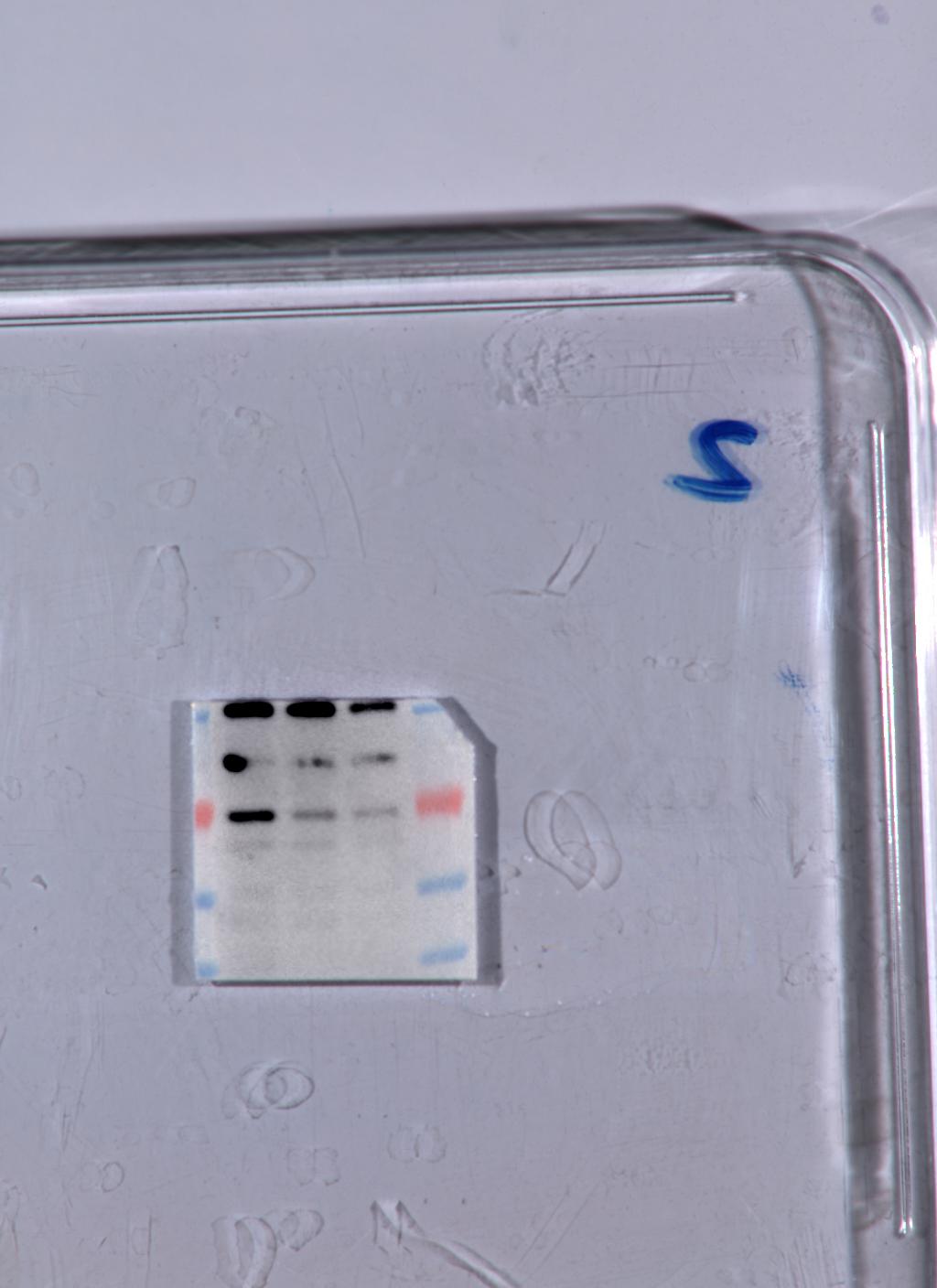

Supplement: Supplementary file 11 — Source data Fig. 6 [file 44321_2024_60_MOESM11_ESM.zip › Figure 6/6C/88T/Western PLK 5.1/4-2 2nd PLK 5.1 _Ch+Marker.jpg]

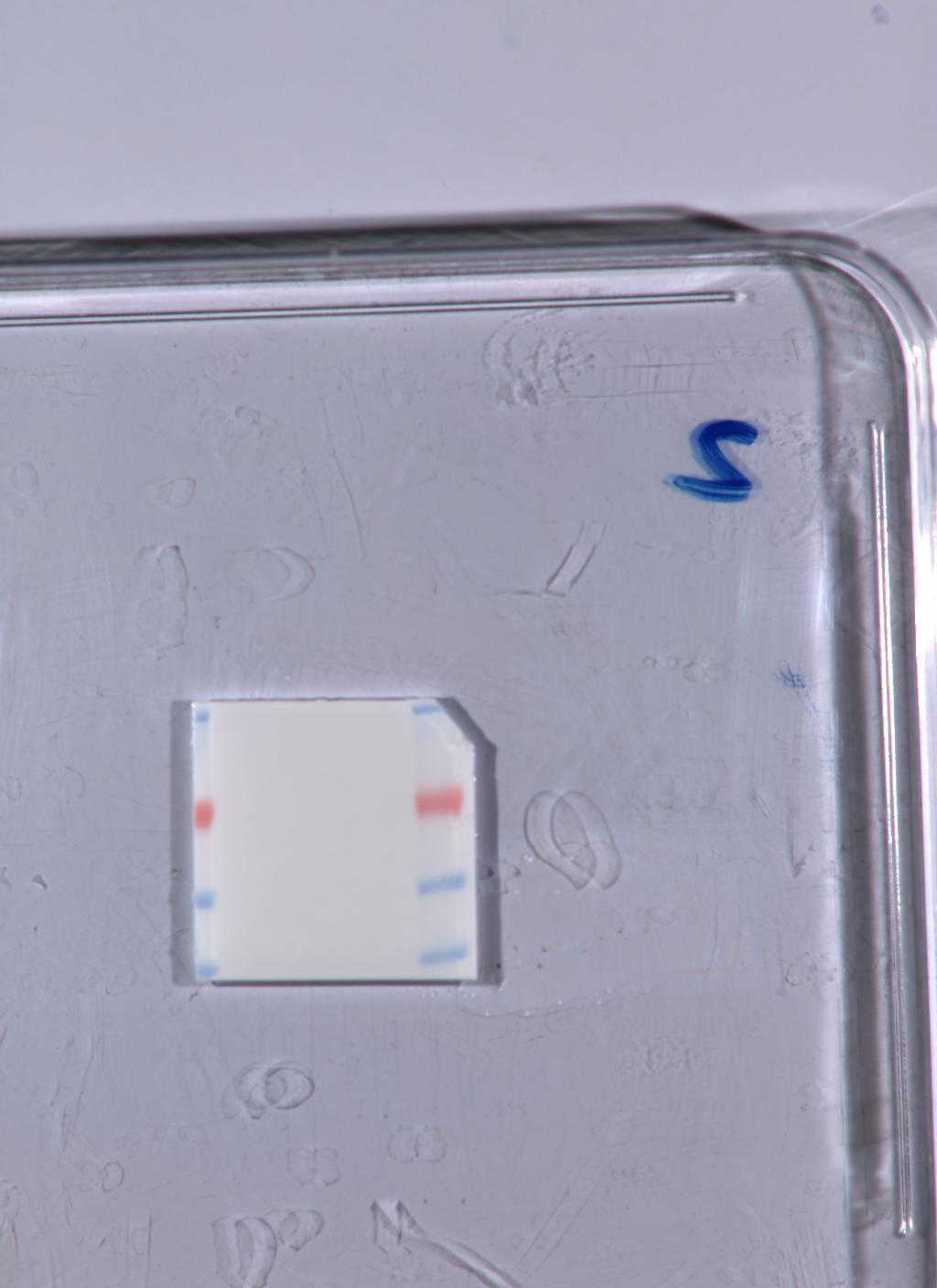

Supplement: Supplementary file 11 — Source data Fig. 6 [file 44321_2024_60_MOESM11_ESM.zip › Figure 6/6C/88T/Western PLK 5.1/4-2 2nd PLK 5.1 _Ch-Marker.jpg]

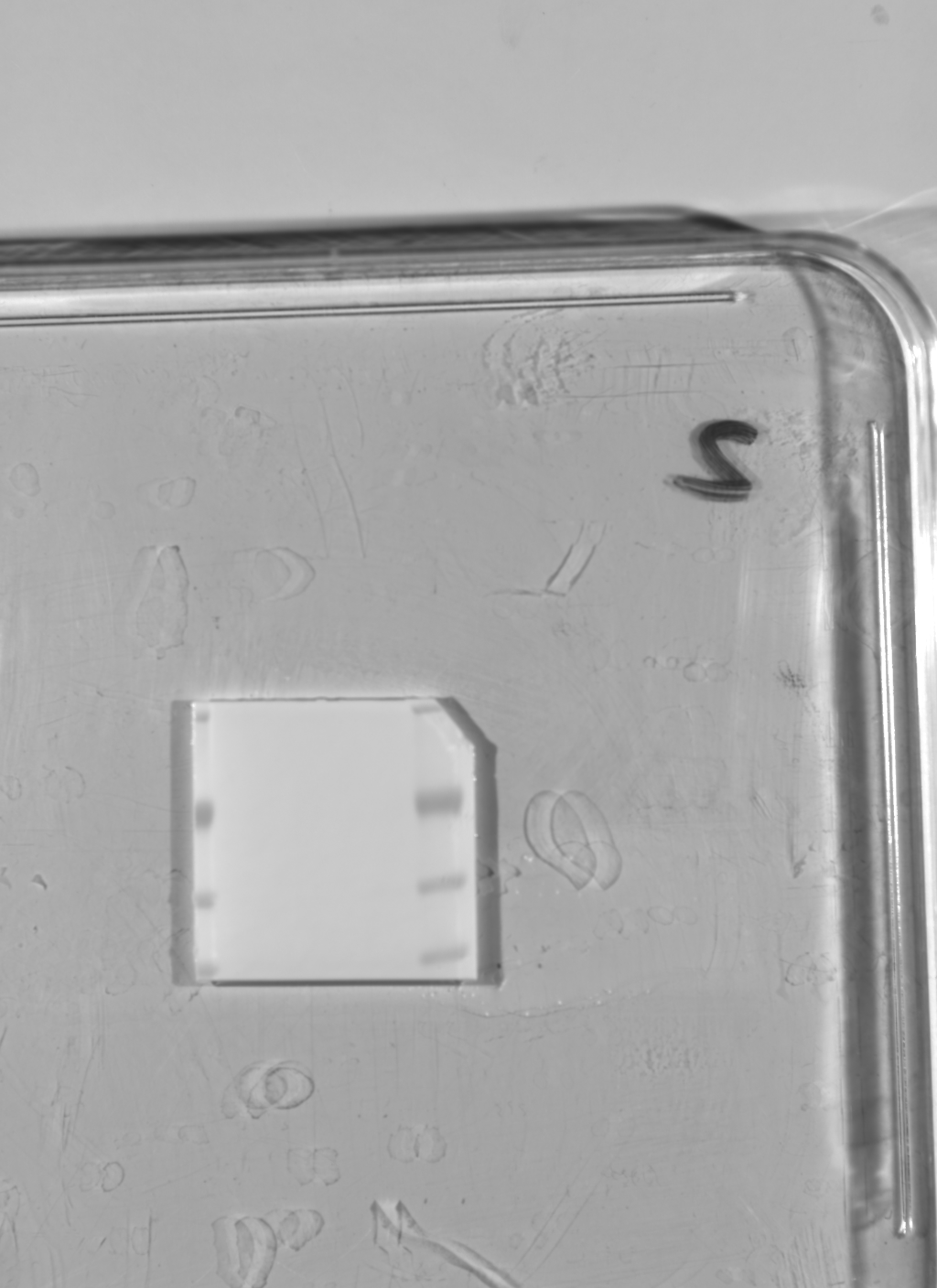

Supplement: Supplementary file 11 — Source data Fig. 6 [file 44321_2024_60_MOESM11_ESM.zip › Figure 6/6C/88T/Western PLK 5.1/4-2 2nd PLK 5.1 _Ch-Marker.tif]

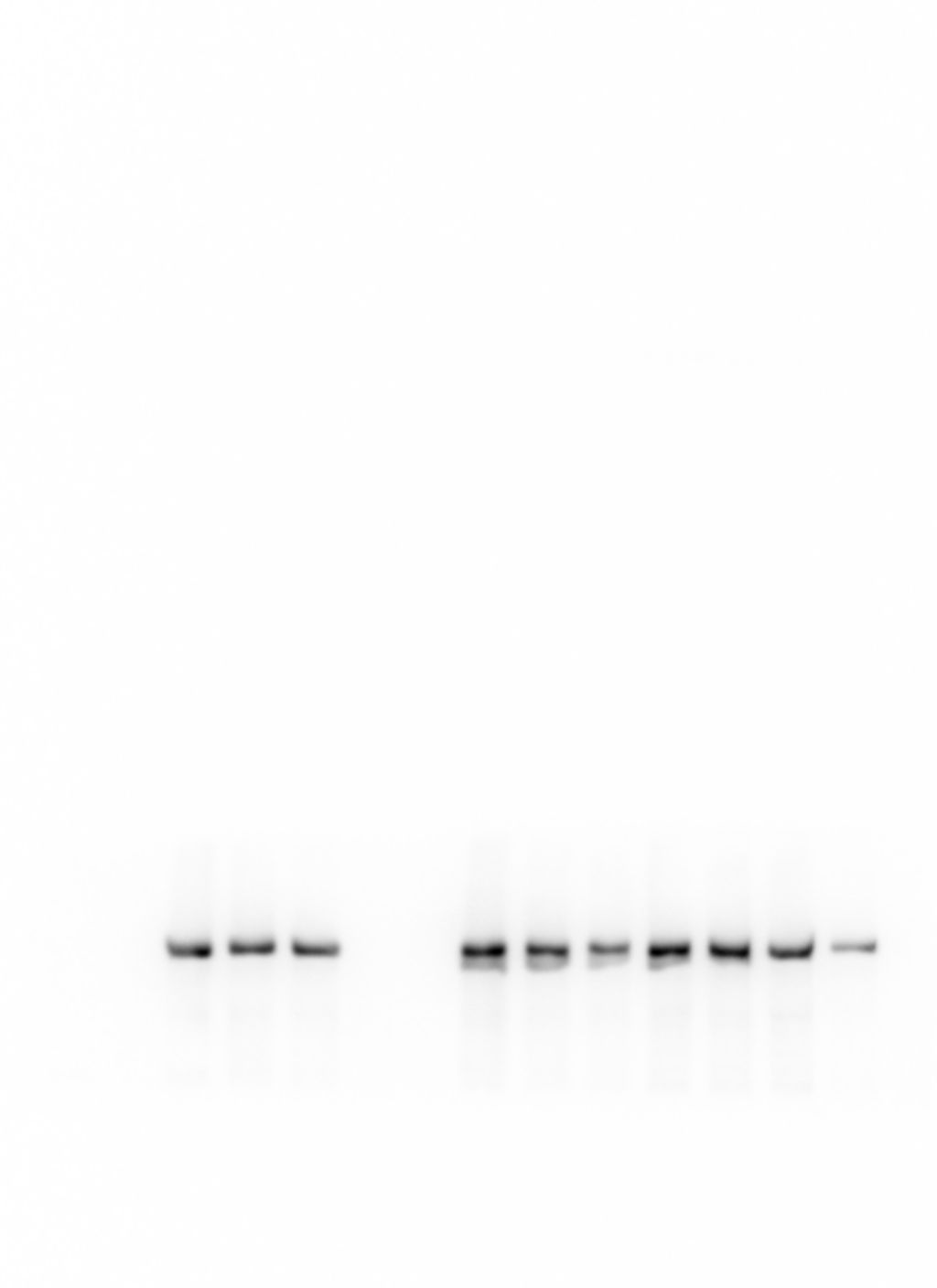

Supplement: Supplementary file 11 — Source data Fig. 6 [file 44321_2024_60_MOESM11_ESM.zip › Figure 6/6C/88T/Western PRKDC 0.1/7 1st PRK 0.1Q _Ch.tif]

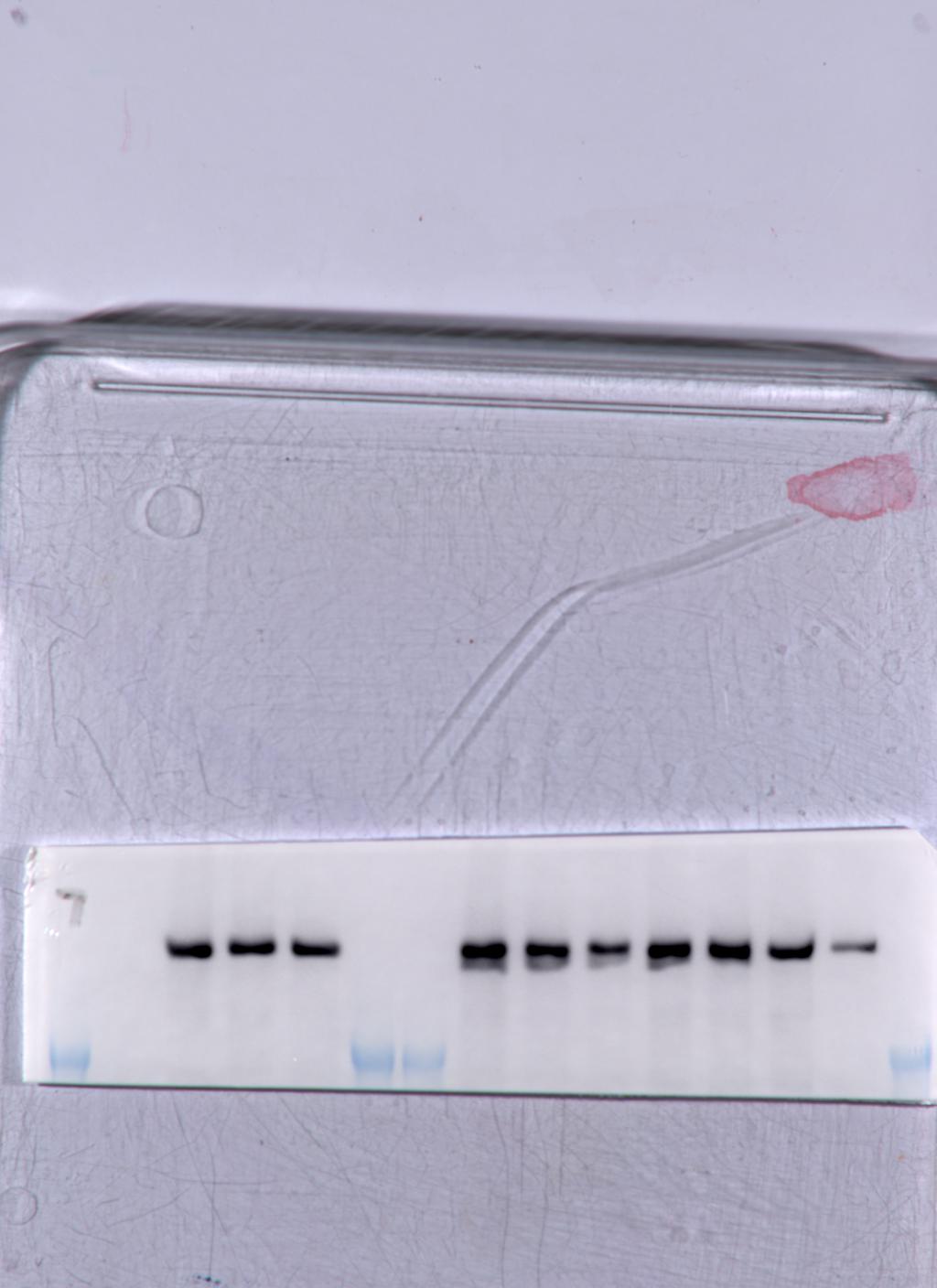

Supplement: Supplementary file 11 — Source data Fig. 6 [file 44321_2024_60_MOESM11_ESM.zip › Figure 6/6C/88T/Western PRKDC 0.1/7 1st PRK 0.1Q _Ch+Marker.jpg]

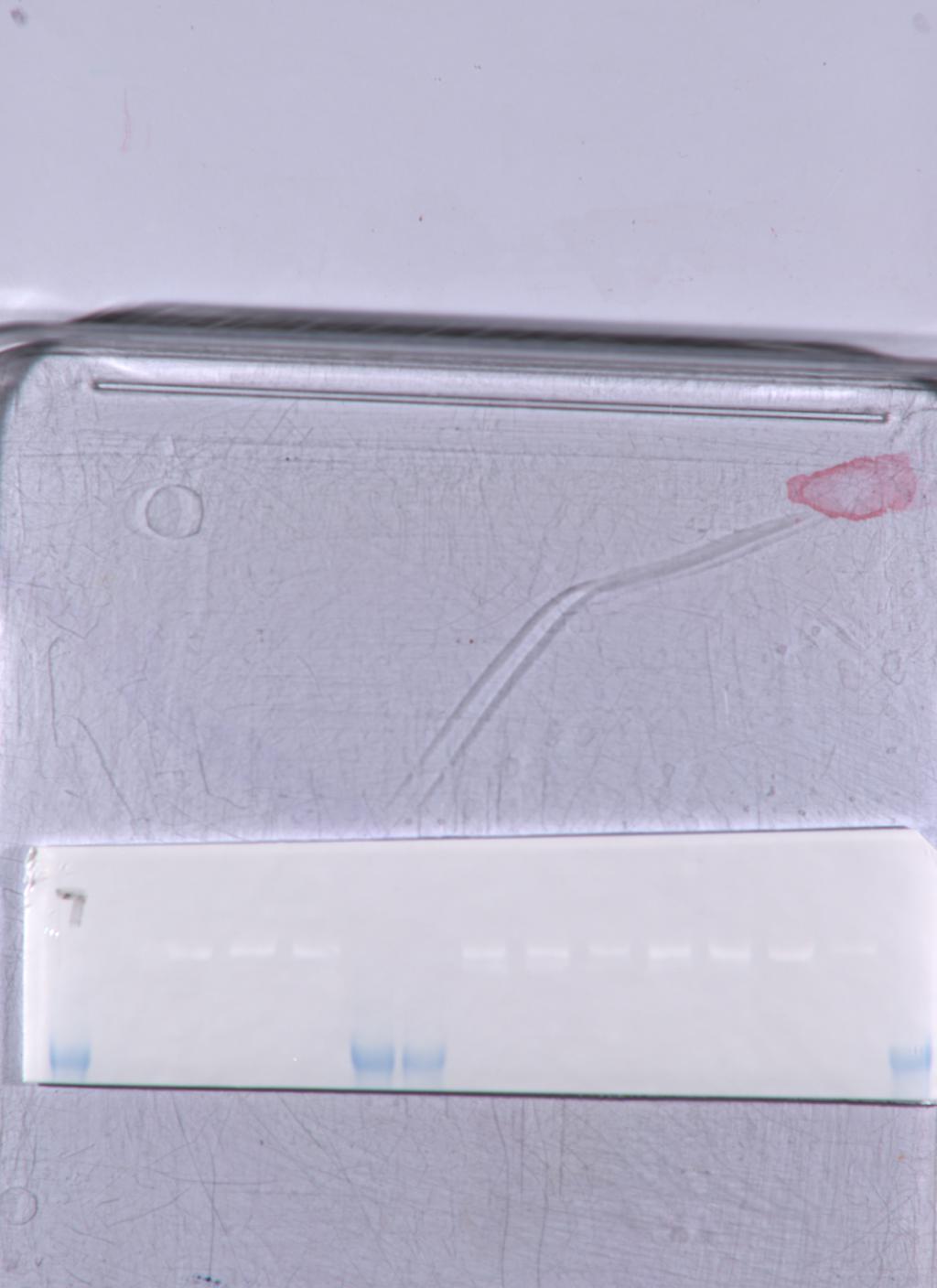

Supplement: Supplementary file 11 — Source data Fig. 6 [file 44321_2024_60_MOESM11_ESM.zip › Figure 6/6C/88T/Western PRKDC 0.1/7 1st PRK 0.1Q _Ch-Marker.jpg]

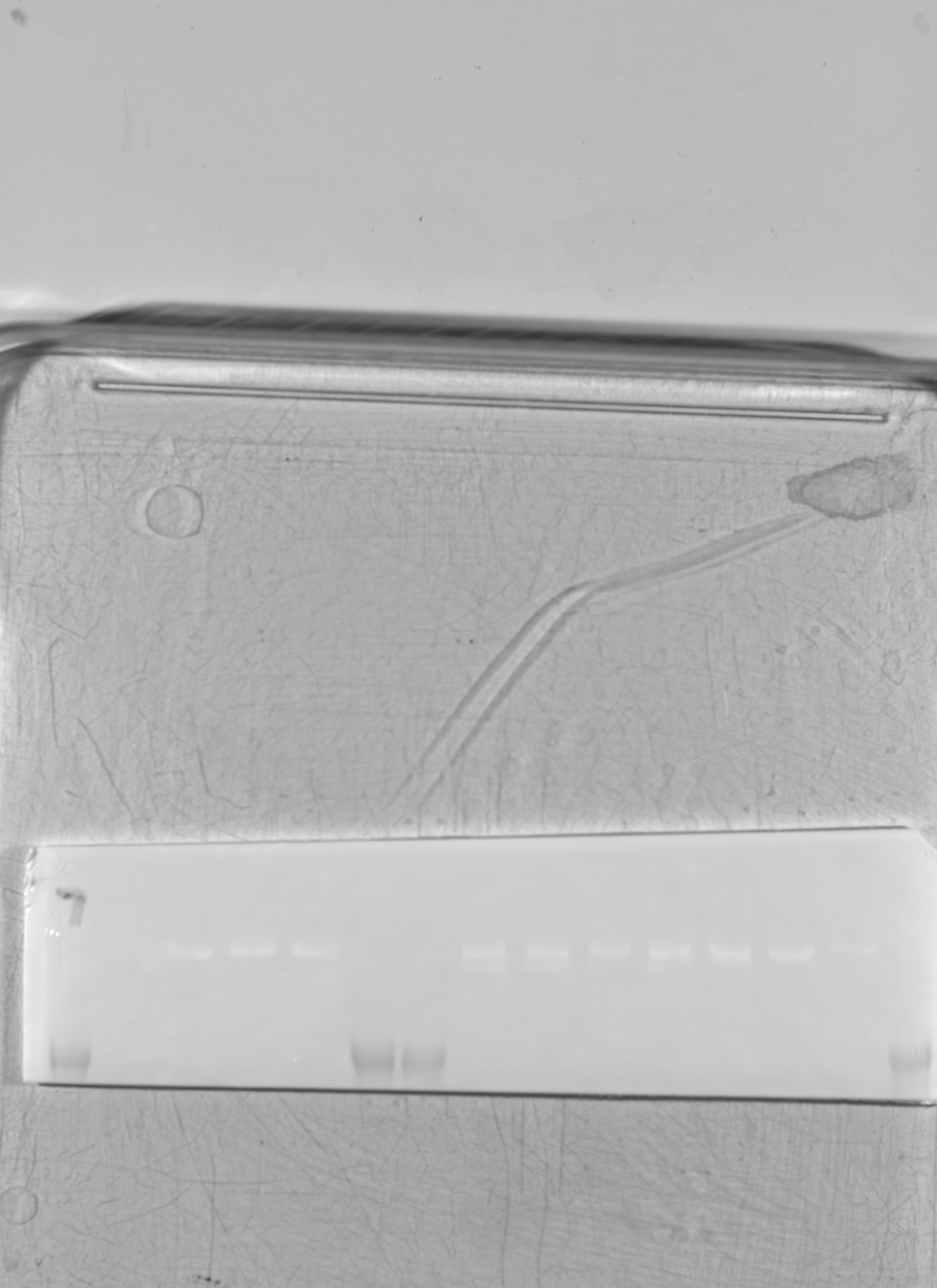

Supplement: Supplementary file 11 — Source data Fig. 6 [file 44321_2024_60_MOESM11_ESM.zip › Figure 6/6C/88T/Western PRKDC 0.1/7 1st PRK 0.1Q _Ch-Marker.tif]

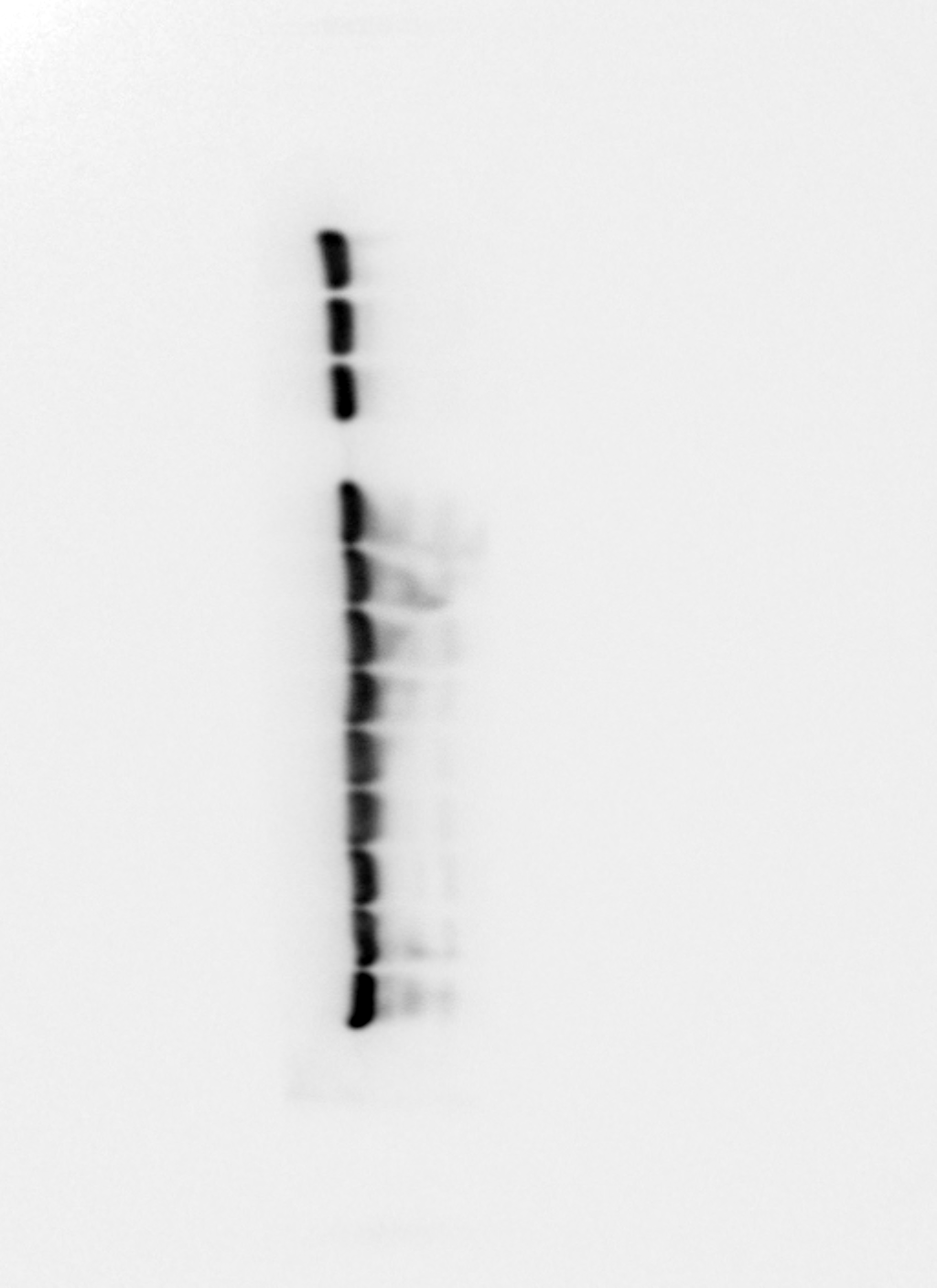

Supplement: Supplementary file 11 — Source data Fig. 6 [file 44321_2024_60_MOESM11_ESM.zip › Figure 6/6C/CN1/Western GAPDH 2.3/3 3rd GAP 2.3 _Ch.tif]

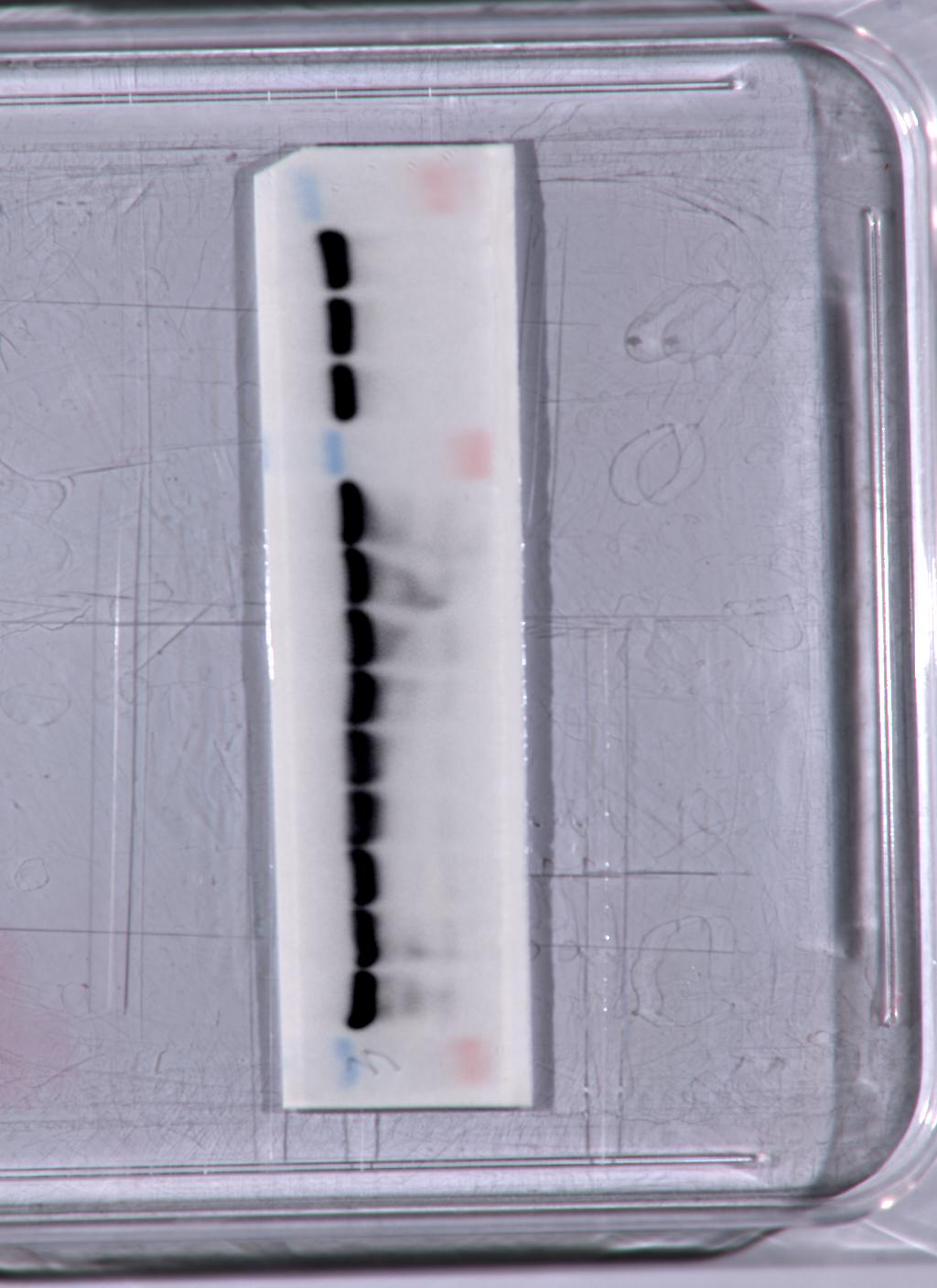

Supplement: Supplementary file 11 — Source data Fig. 6 [file 44321_2024_60_MOESM11_ESM.zip › Figure 6/6C/CN1/Western GAPDH 2.3/3 3rd GAP 2.3 _Ch+Marker.jpg]

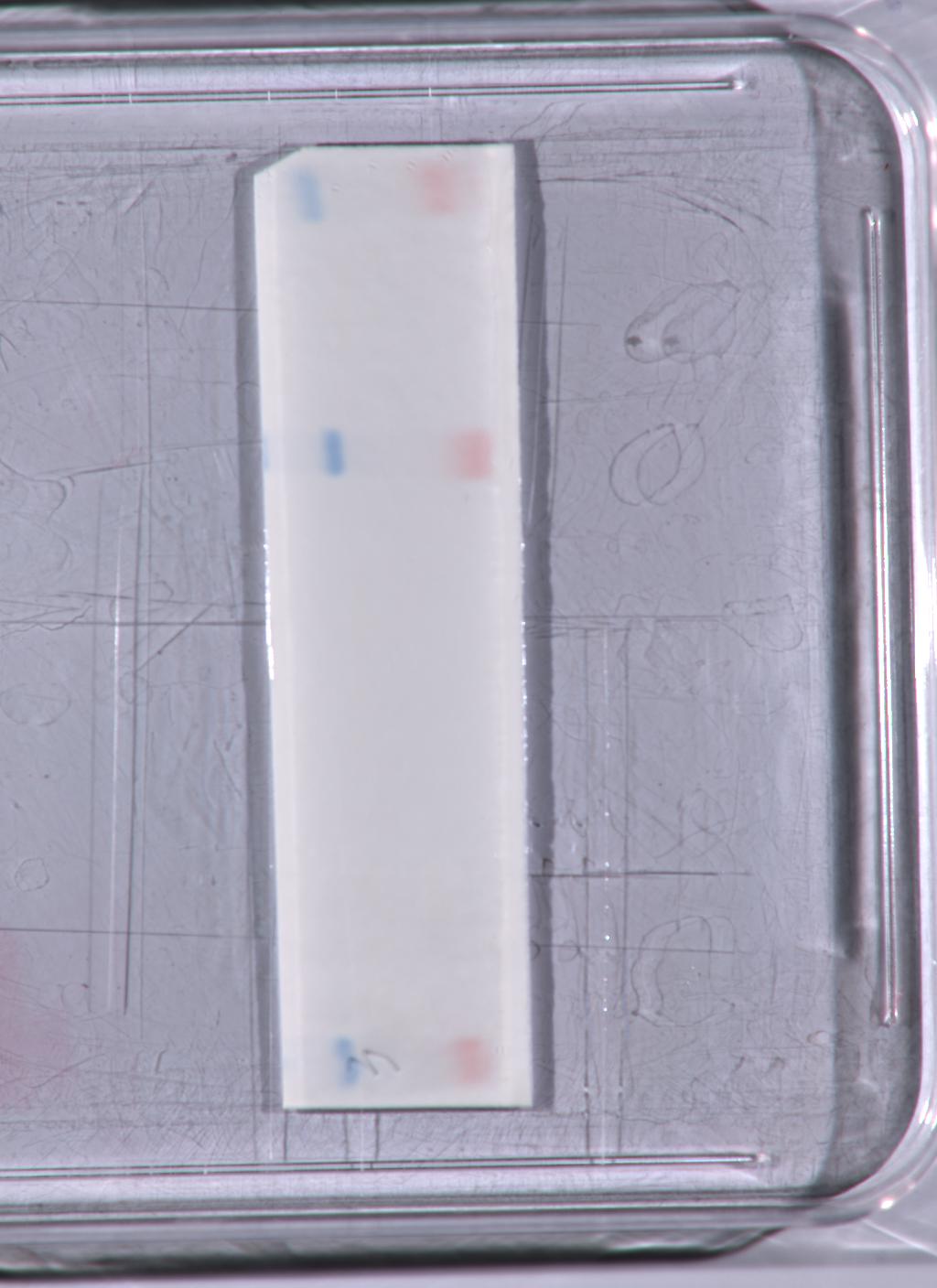

Supplement: Supplementary file 11 — Source data Fig. 6 [file 44321_2024_60_MOESM11_ESM.zip › Figure 6/6C/CN1/Western GAPDH 2.3/3 3rd GAP 2.3 _Ch-Marker.jpg]

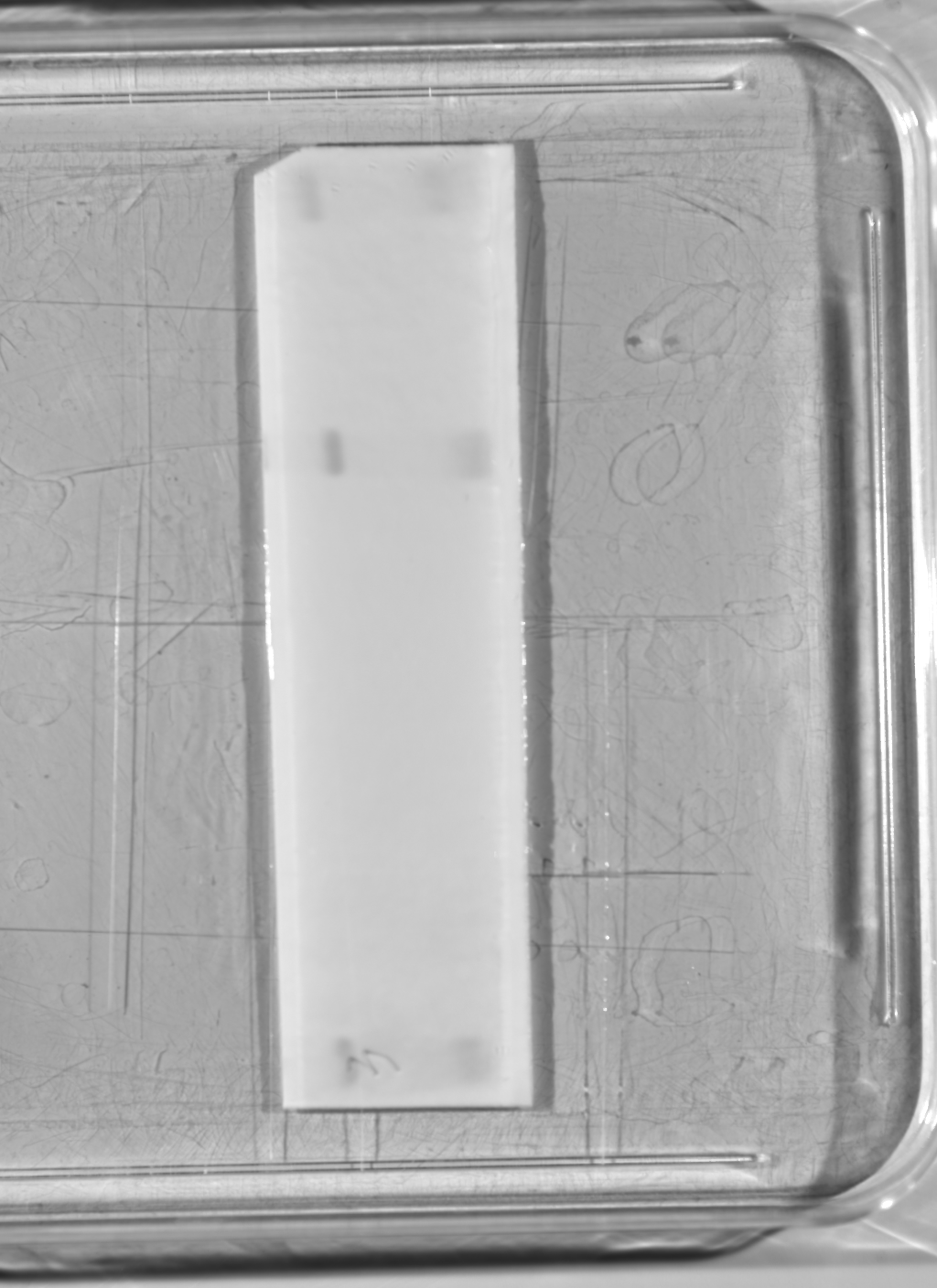

Supplement: Supplementary file 11 — Source data Fig. 6 [file 44321_2024_60_MOESM11_ESM.zip › Figure 6/6C/CN1/Western GAPDH 2.3/3 3rd GAP 2.3 _Ch-Marker.tif]

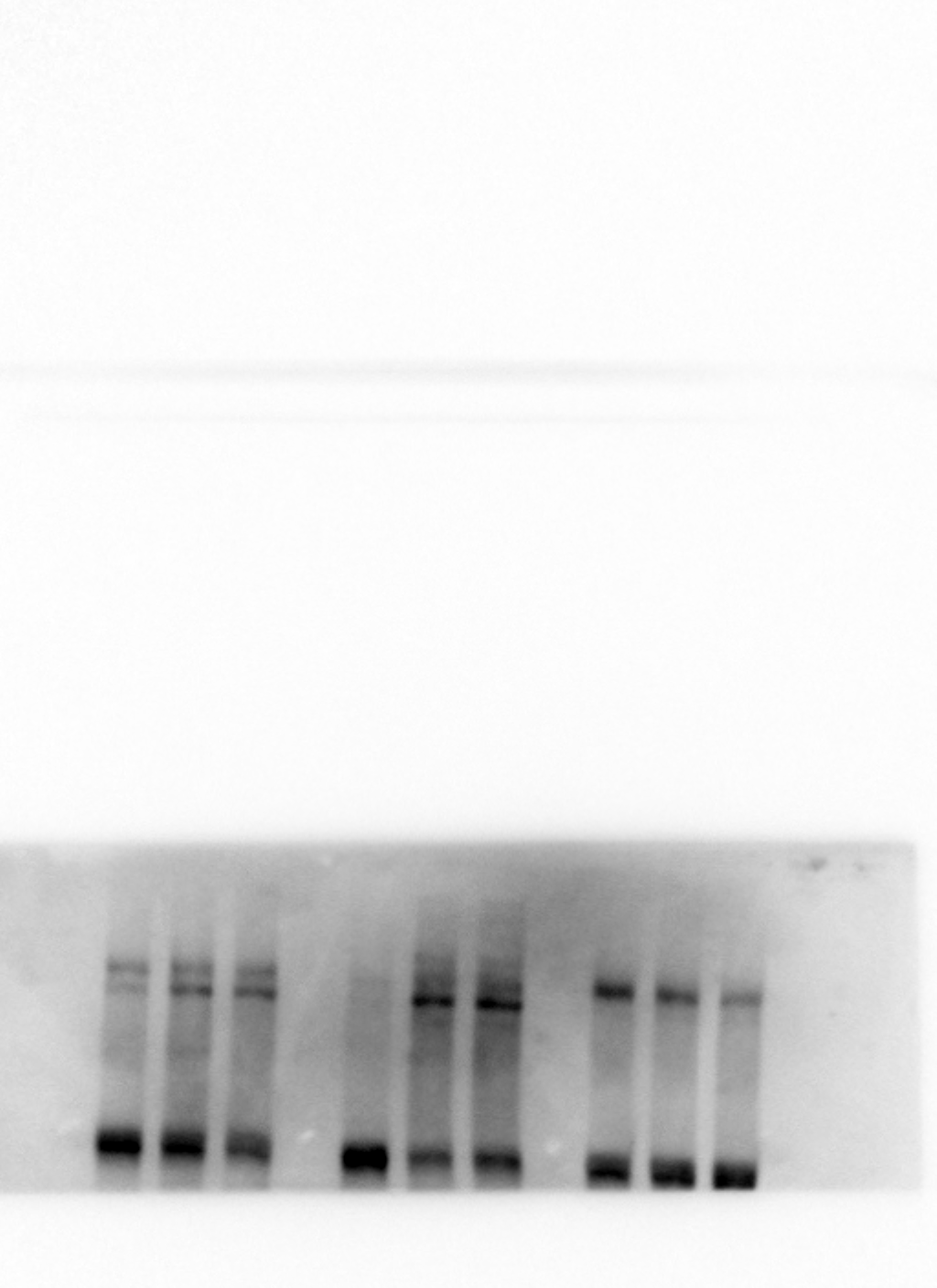

Supplement: Supplementary file 11 — Source data Fig. 6 [file 44321_2024_60_MOESM11_ESM.zip › Figure 6/6C/CN1/Western pho-PRKDC 47.6/1 3rd pho-PRK 47.6 _Ch.tif]

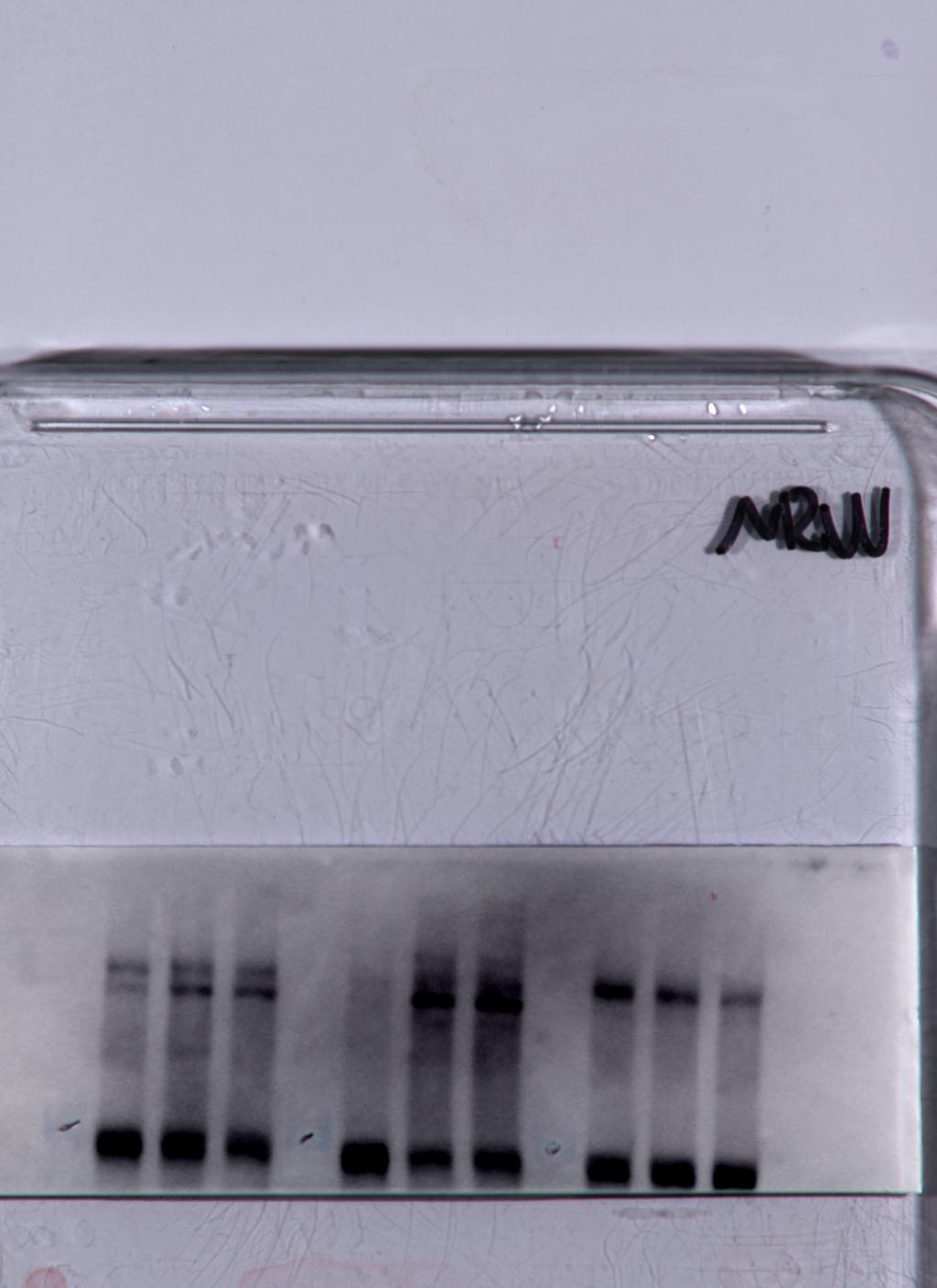

Supplement: Supplementary file 11 — Source data Fig. 6 [file 44321_2024_60_MOESM11_ESM.zip › Figure 6/6C/CN1/Western pho-PRKDC 47.6/1 3rd pho-PRK 47.6 _Ch+Marker.jpg]

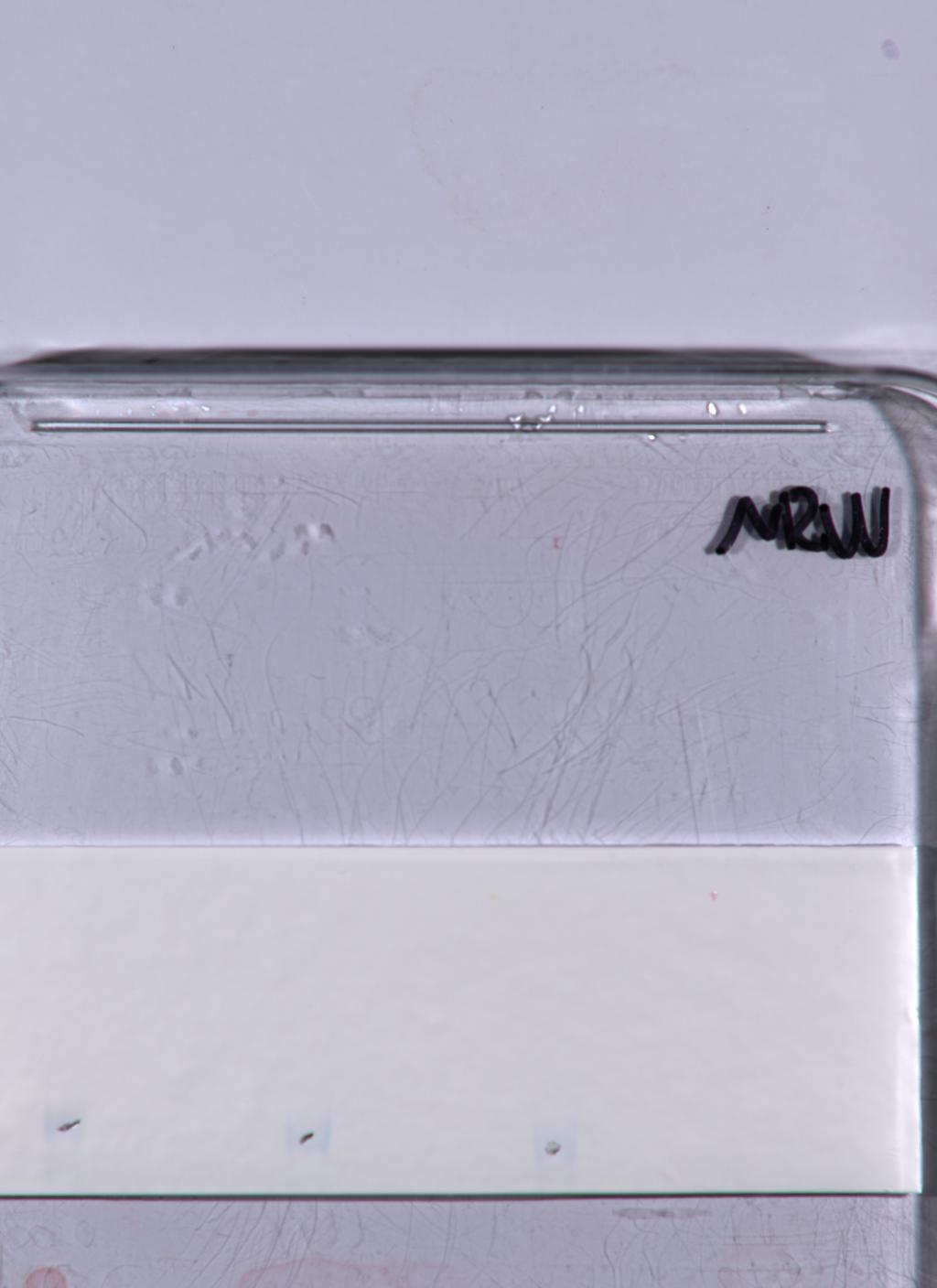

Supplement: Supplementary file 11 — Source data Fig. 6 [file 44321_2024_60_MOESM11_ESM.zip › Figure 6/6C/CN1/Western pho-PRKDC 47.6/1 3rd pho-PRK 47.6 _Ch-Marker.jpg]

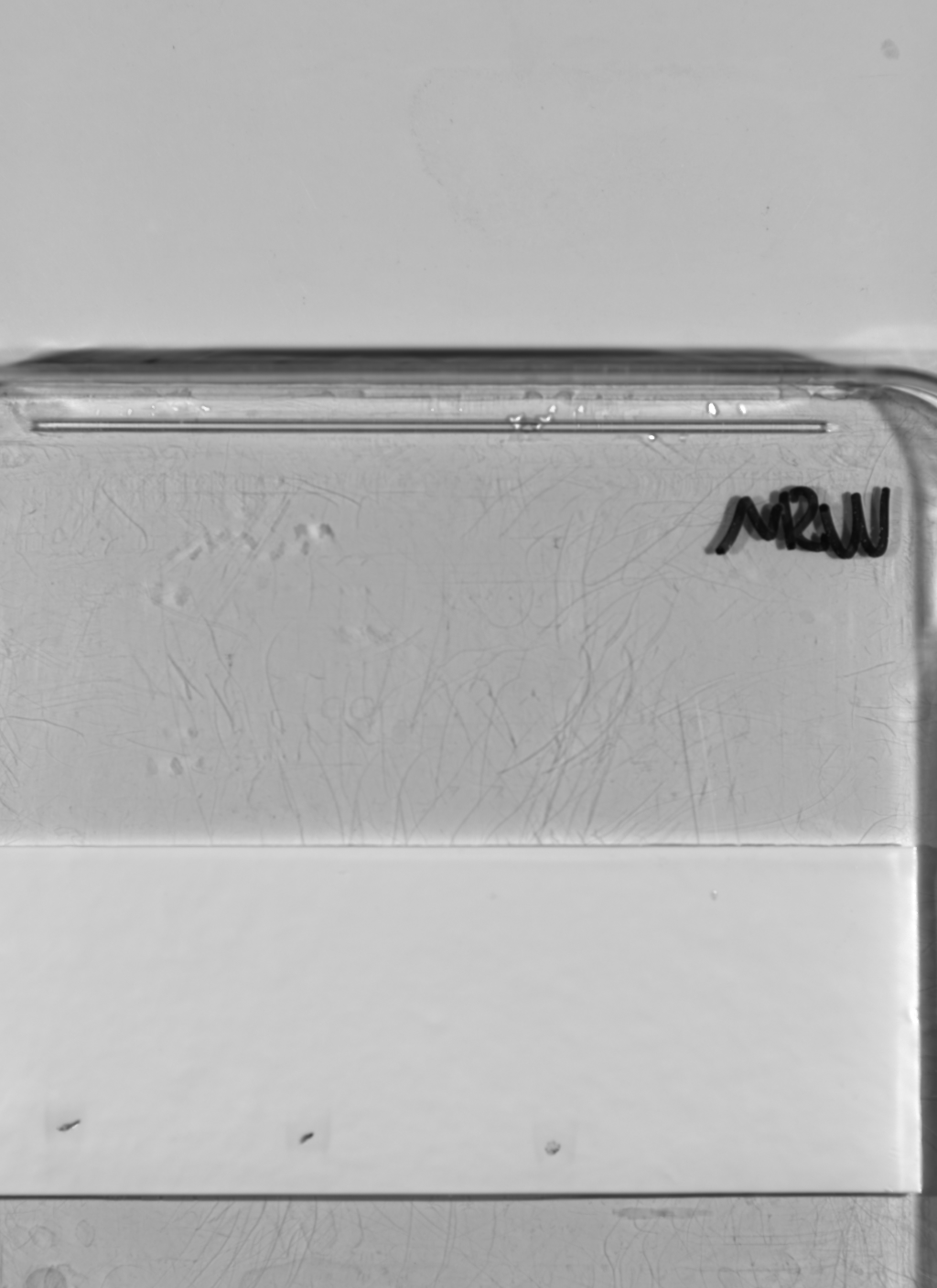

Supplement: Supplementary file 11 — Source data Fig. 6 [file 44321_2024_60_MOESM11_ESM.zip › Figure 6/6C/CN1/Western pho-PRKDC 47.6/1 3rd pho-PRK 47.6 _Ch-Marker.tif]

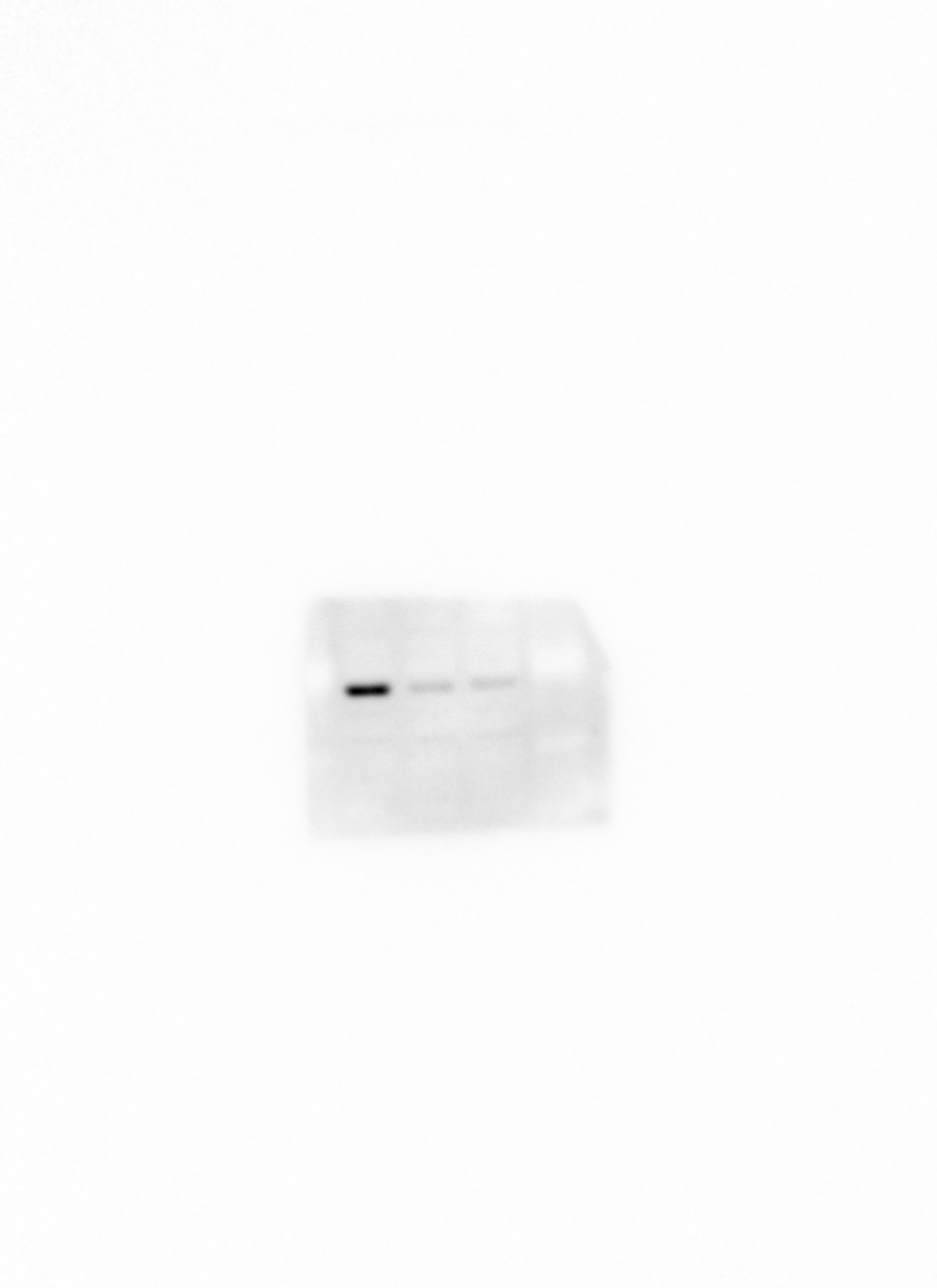

Supplement: Supplementary file 11 — Source data Fig. 6 [file 44321_2024_60_MOESM11_ESM.zip › Figure 6/6C/CN1/Western PLK 38.8/3-2 1st PLK 38.8 _Ch.tif]

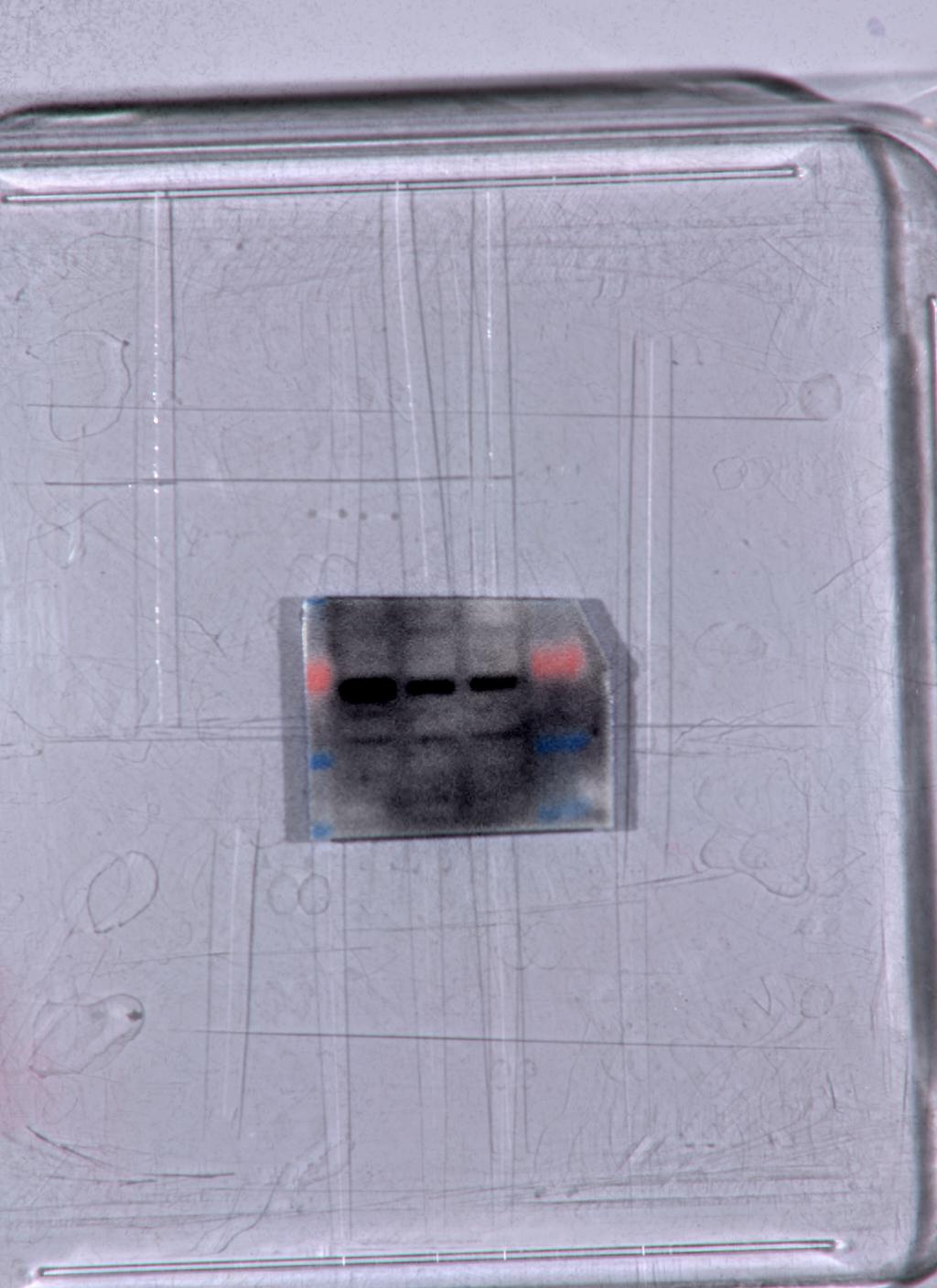

Supplement: Supplementary file 11 — Source data Fig. 6 [file 44321_2024_60_MOESM11_ESM.zip › Figure 6/6C/CN1/Western PLK 38.8/3-2 1st PLK 38.8 _Ch+Marker.jpg]

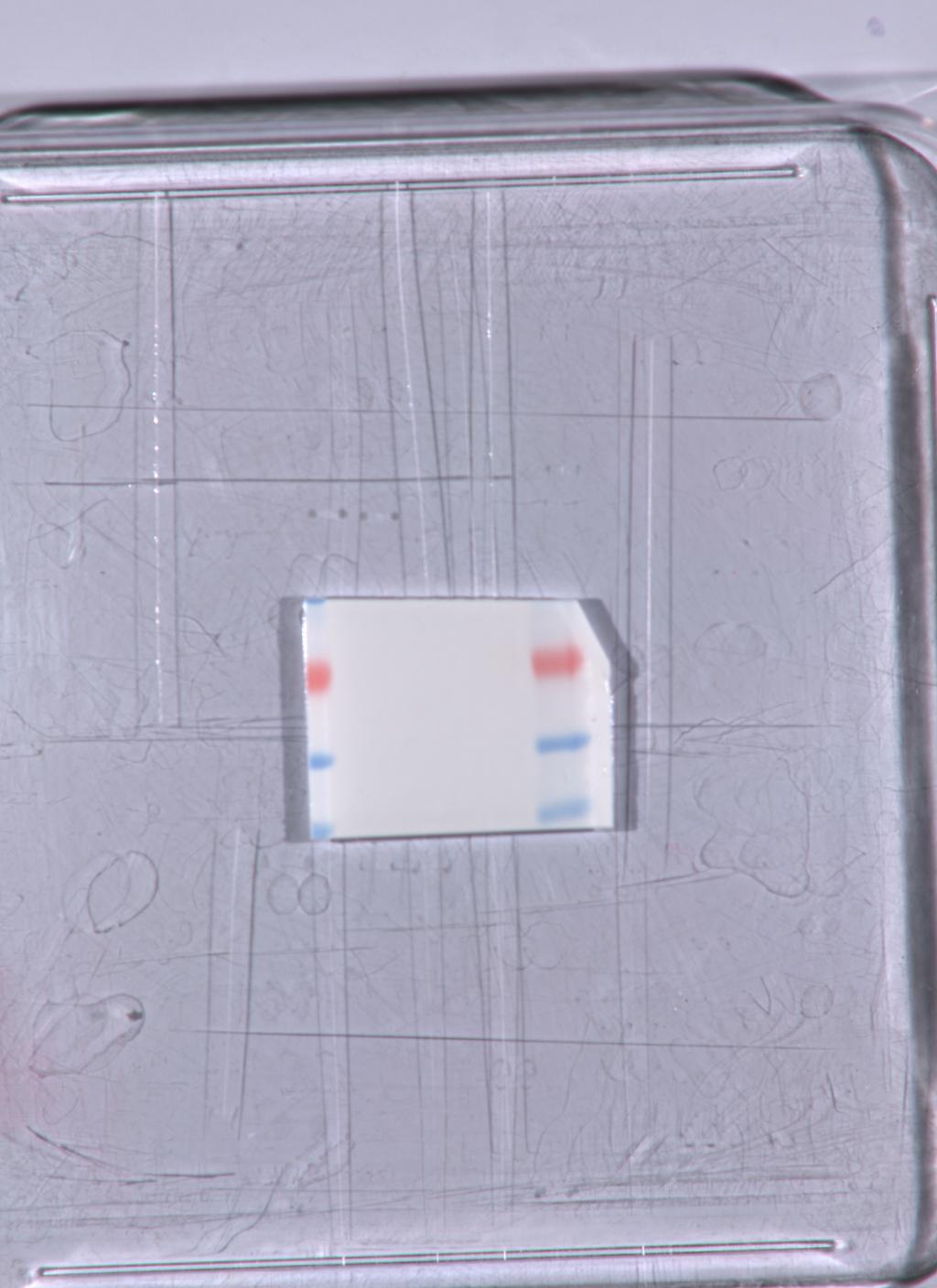

Supplement: Supplementary file 11 — Source data Fig. 6 [file 44321_2024_60_MOESM11_ESM.zip › Figure 6/6C/CN1/Western PLK 38.8/3-2 1st PLK 38.8 _Ch-Marker.jpg]

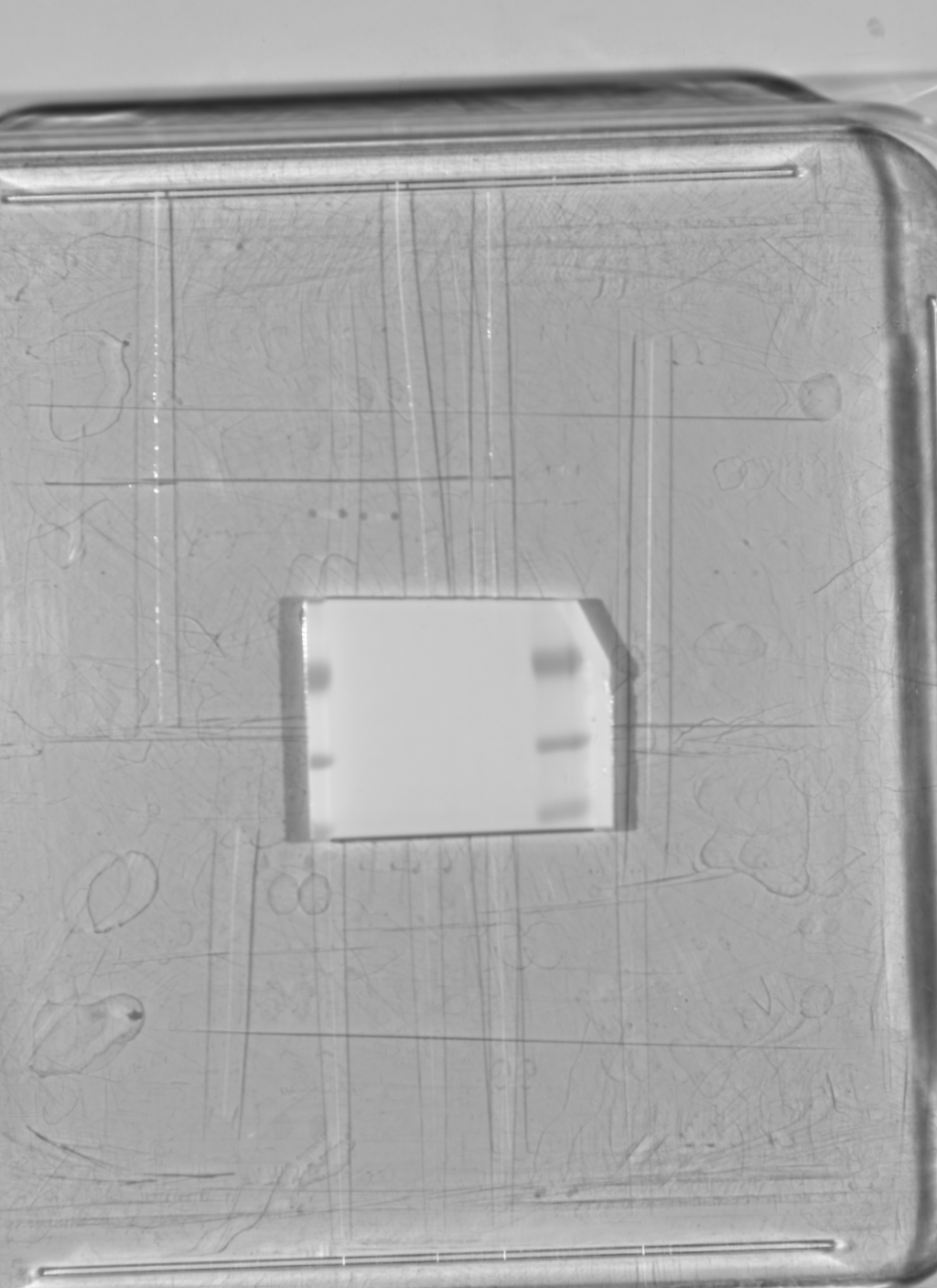

Supplement: Supplementary file 11 — Source data Fig. 6 [file 44321_2024_60_MOESM11_ESM.zip › Figure 6/6C/CN1/Western PLK 38.8/3-2 1st PLK 38.8 _Ch-Marker.tif]

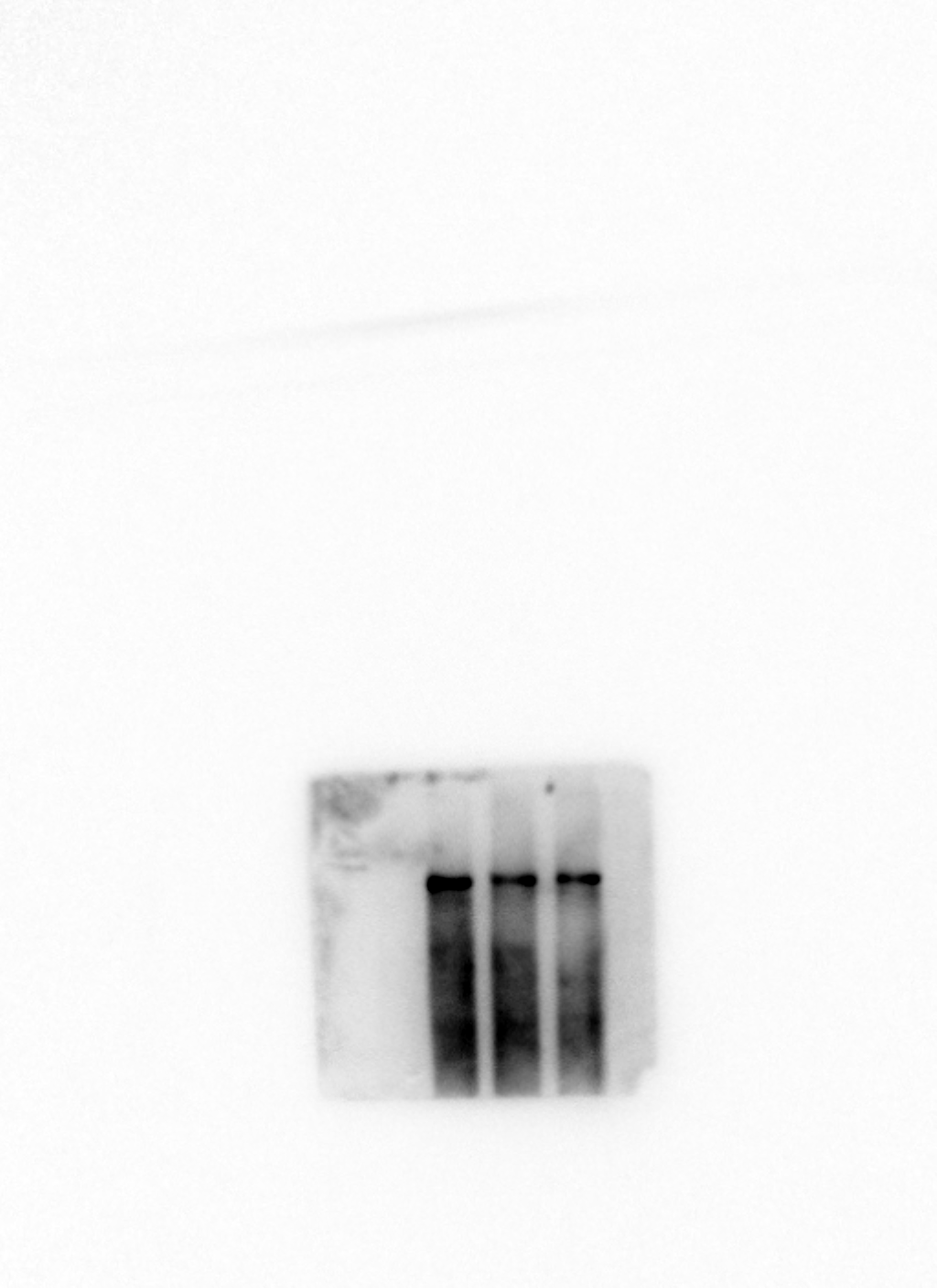

Supplement: Supplementary file 11 — Source data Fig. 6 [file 44321_2024_60_MOESM11_ESM.zip › Figure 6/6C/CN1/Western PRKDC 34/2-1 3rd PRK 34 _Ch.tif]

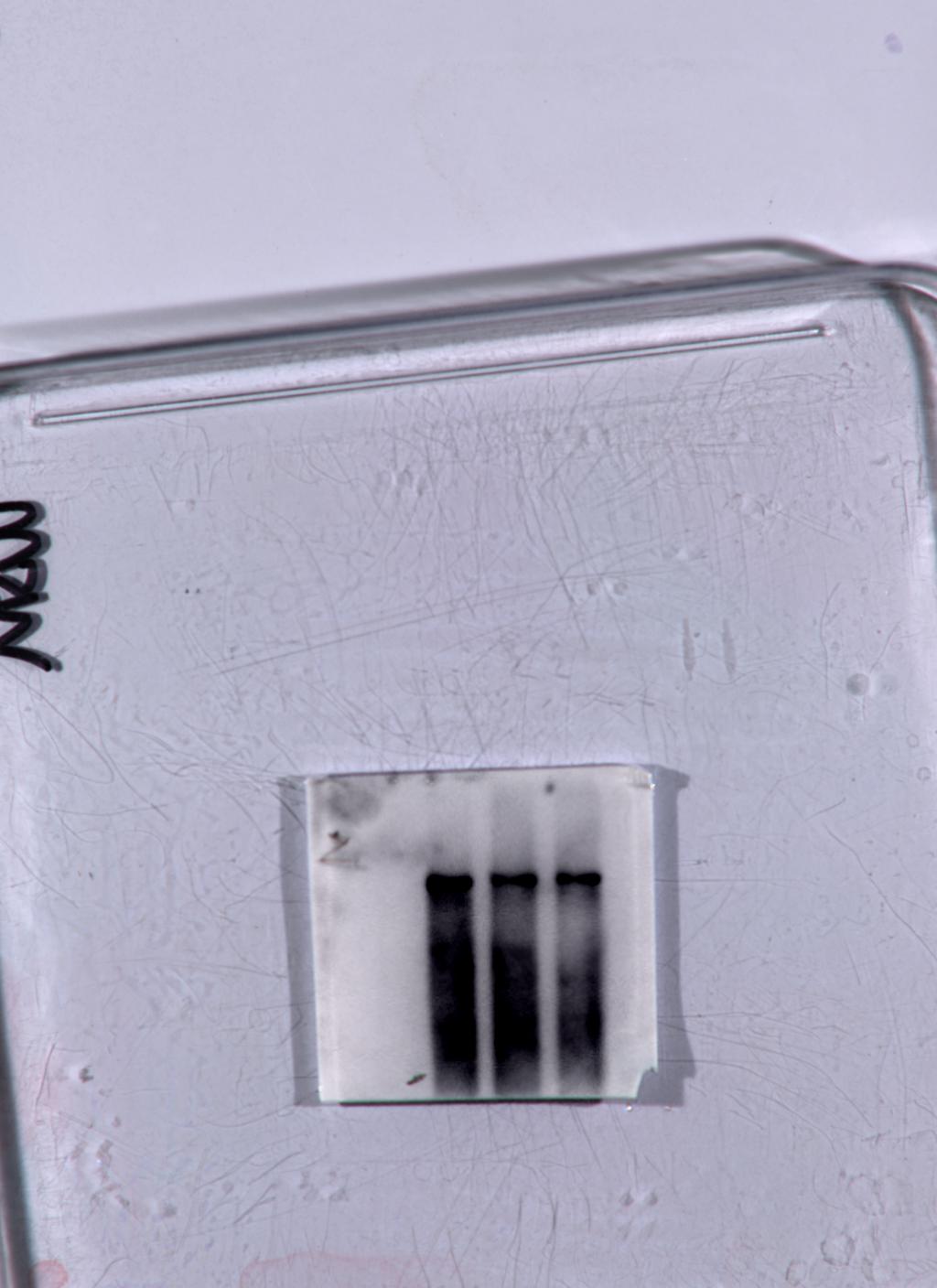

Supplement: Supplementary file 11 — Source data Fig. 6 [file 44321_2024_60_MOESM11_ESM.zip › Figure 6/6C/CN1/Western PRKDC 34/2-1 3rd PRK 34 _Ch+Marker.jpg]

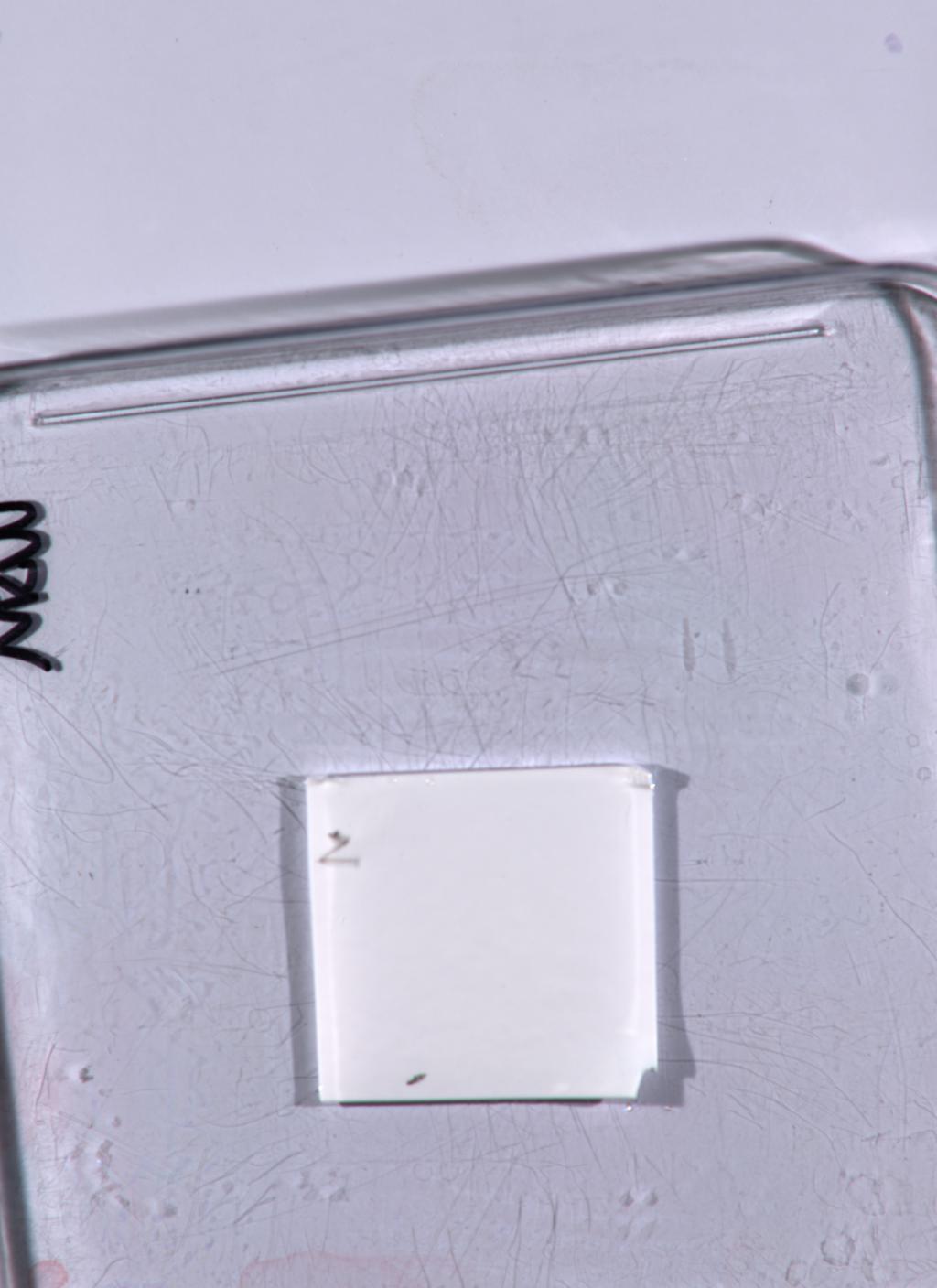

Supplement: Supplementary file 11 — Source data Fig. 6 [file 44321_2024_60_MOESM11_ESM.zip › Figure 6/6C/CN1/Western PRKDC 34/2-1 3rd PRK 34 _Ch-Marker.jpg]

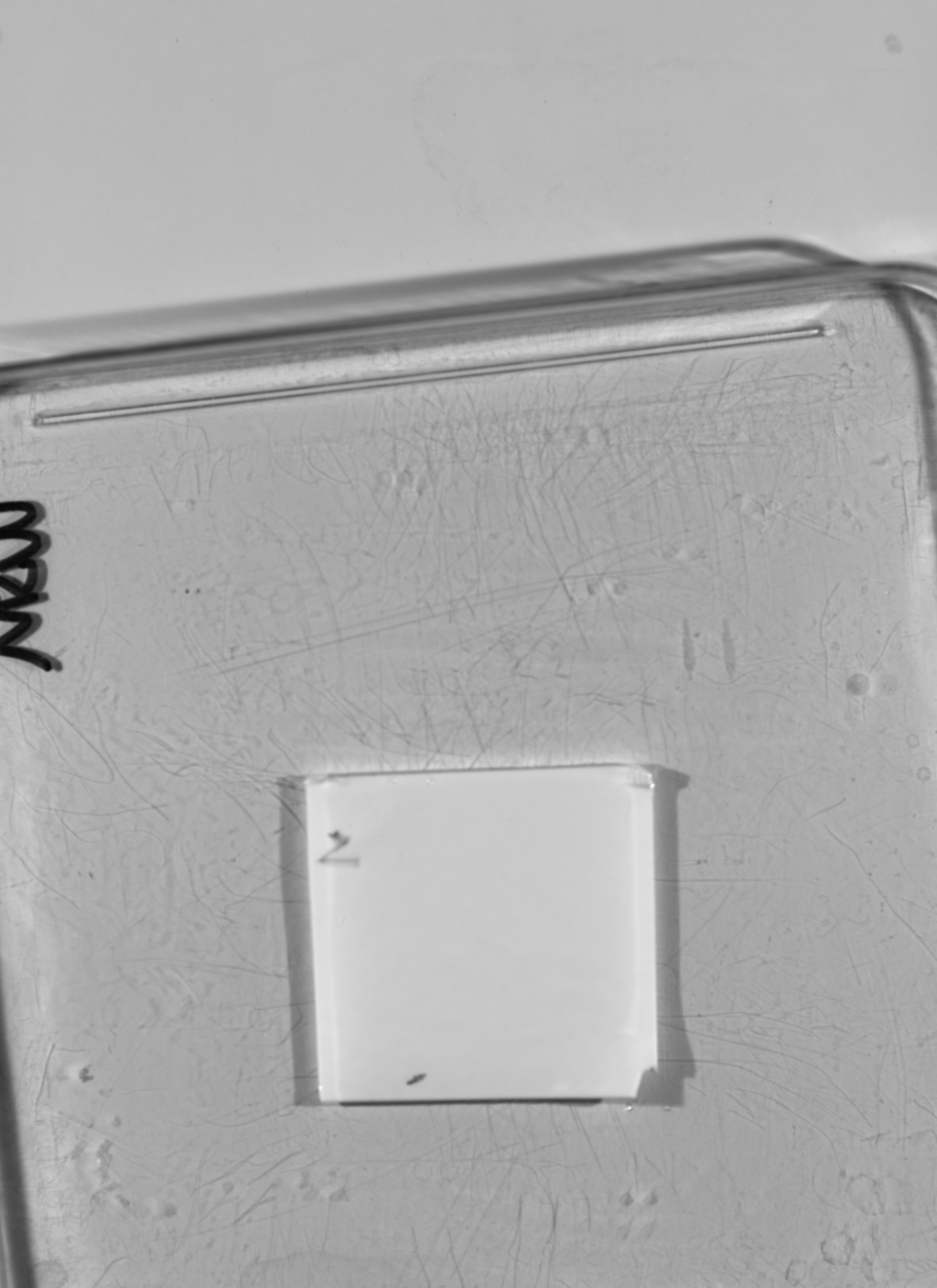

Supplement: Supplementary file 11 — Source data Fig. 6 [file 44321_2024_60_MOESM11_ESM.zip › Figure 6/6C/CN1/Western PRKDC 34/2-1 3rd PRK 34 _Ch-Marker.tif]

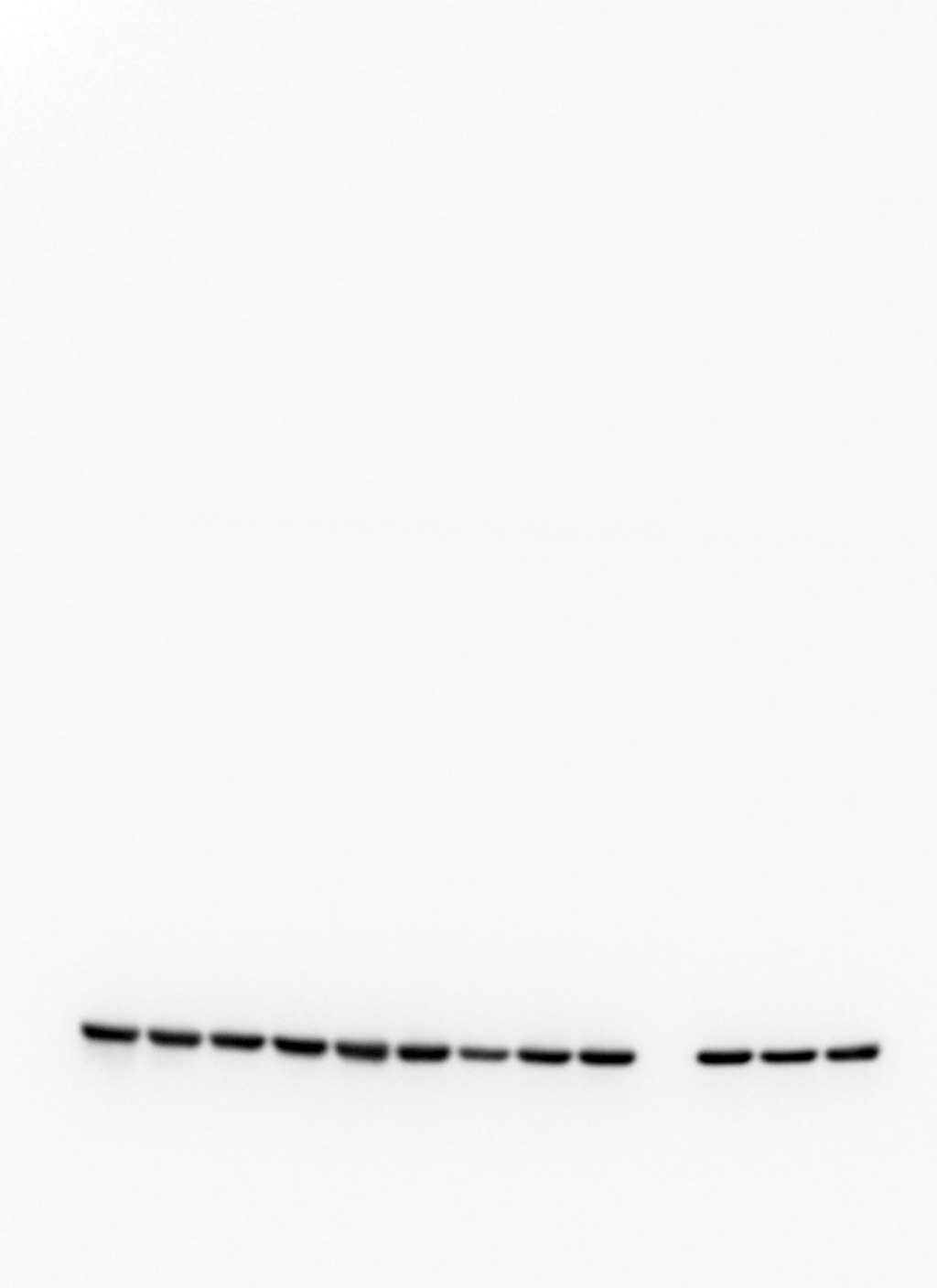

Supplement: Supplementary file 11 — Source data Fig. 6 [file 44321_2024_60_MOESM11_ESM.zip › Figure 6/6C/YAPC/Western GAPDH 1.5/7 GAP 1.5 _Ch.tif]

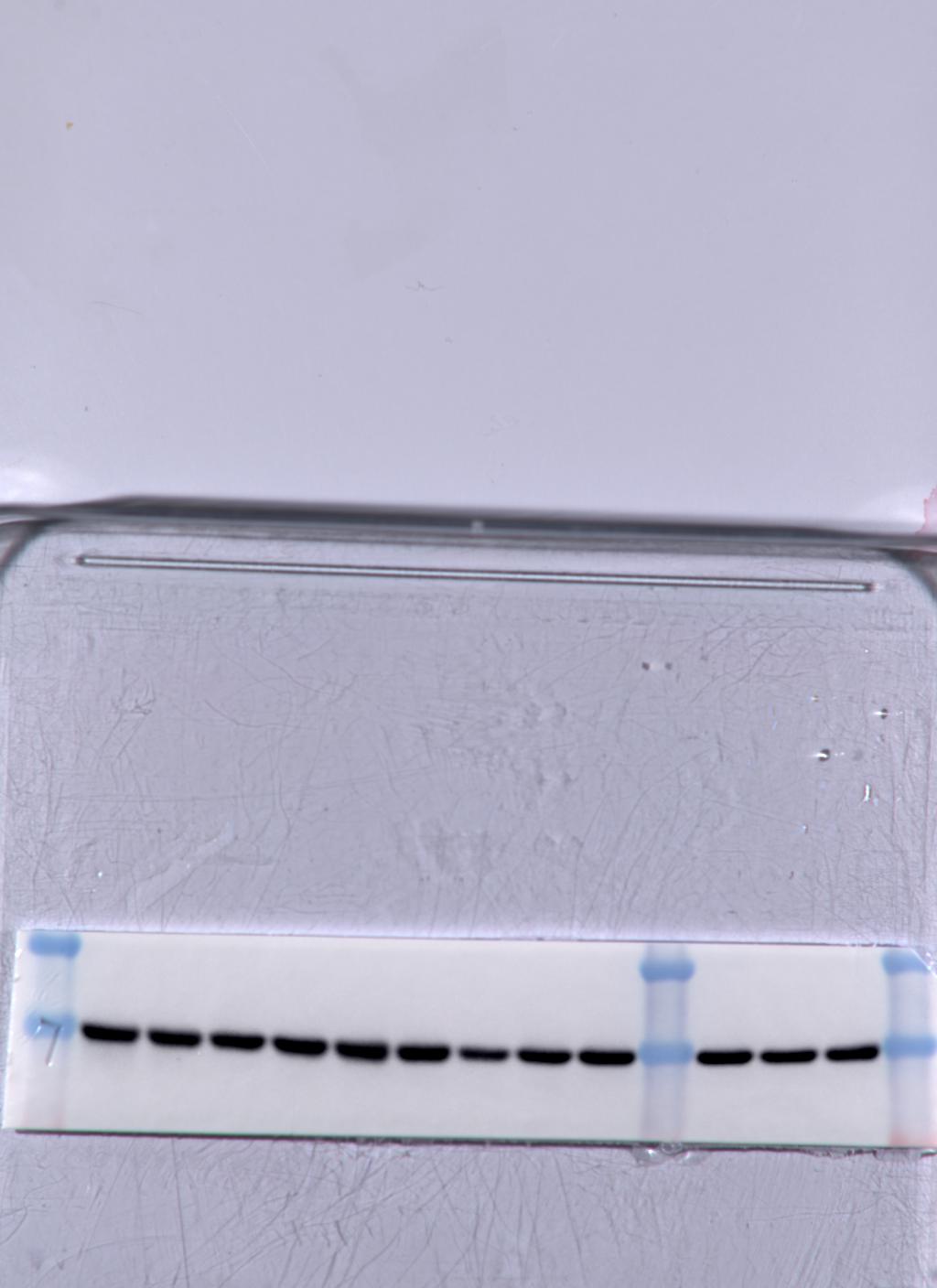

Supplement: Supplementary file 11 — Source data Fig. 6 [file 44321_2024_60_MOESM11_ESM.zip › Figure 6/6C/YAPC/Western GAPDH 1.5/7 GAP 1.5 _Ch+Marker.jpg]

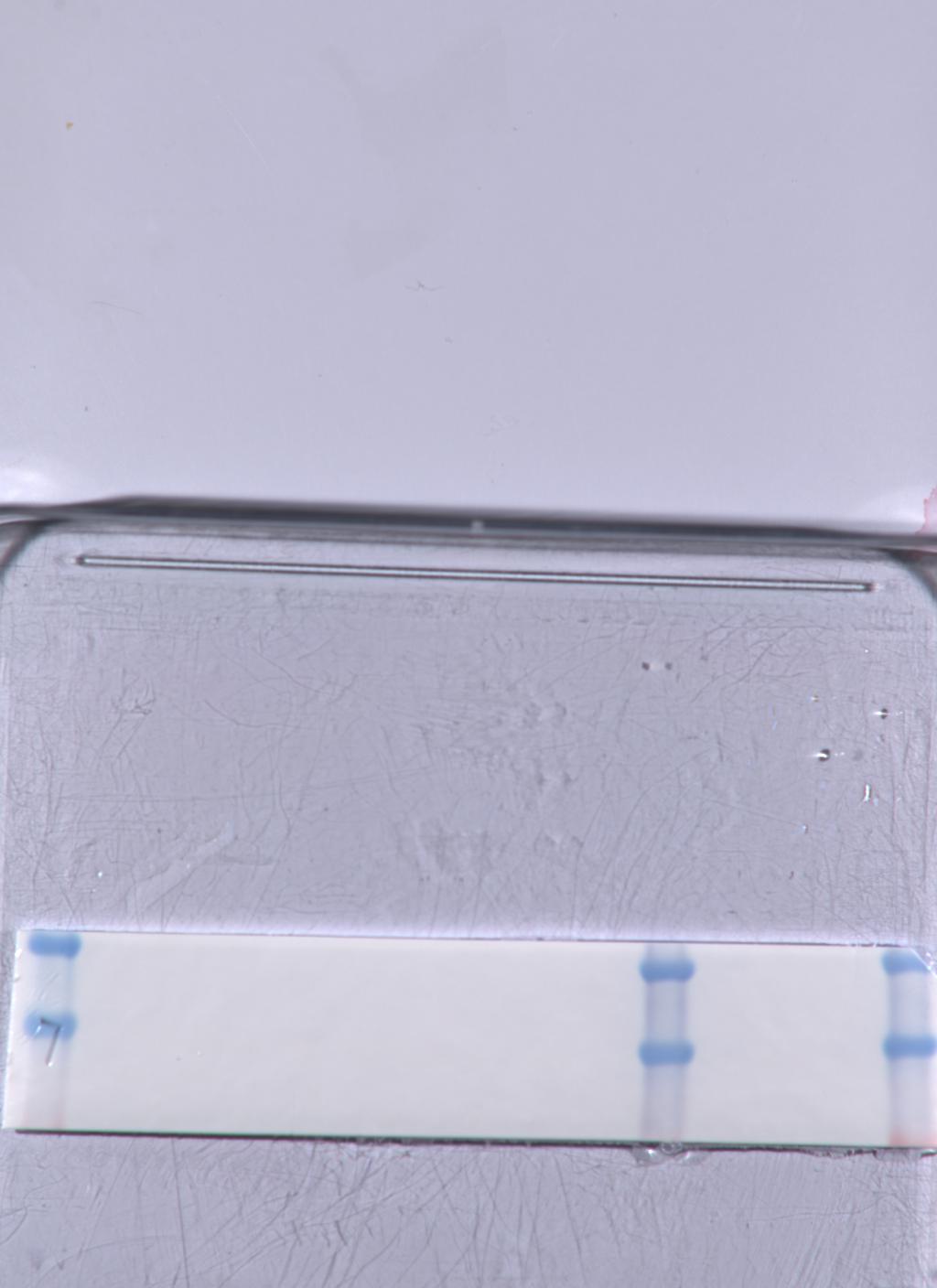

Supplement: Supplementary file 11 — Source data Fig. 6 [file 44321_2024_60_MOESM11_ESM.zip › Figure 6/6C/YAPC/Western GAPDH 1.5/7 GAP 1.5 _Ch-Marker.jpg]

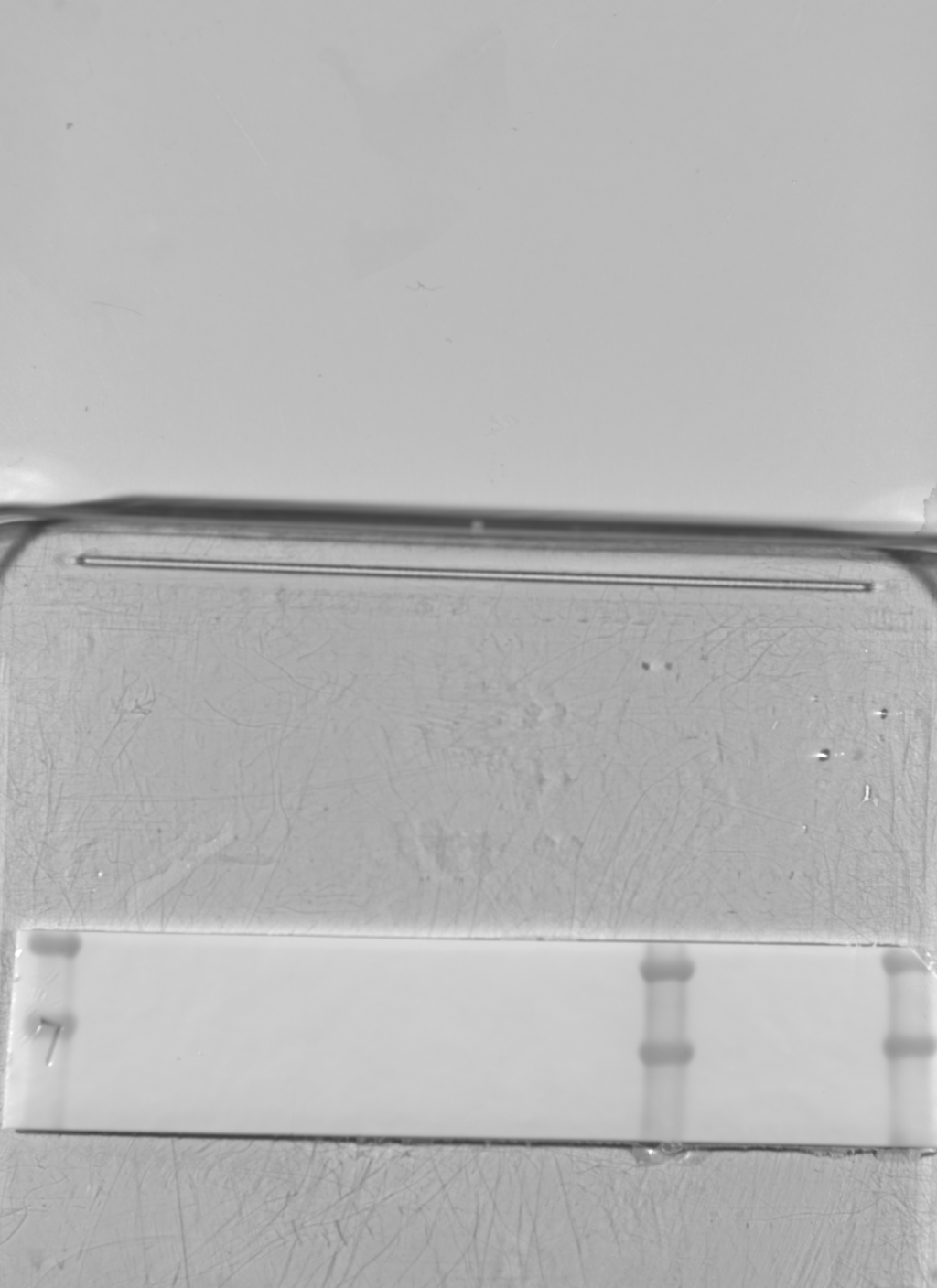

Supplement: Supplementary file 11 — Source data Fig. 6 [file 44321_2024_60_MOESM11_ESM.zip › Figure 6/6C/YAPC/Western GAPDH 1.5/7 GAP 1.5 _Ch-Marker.tif]

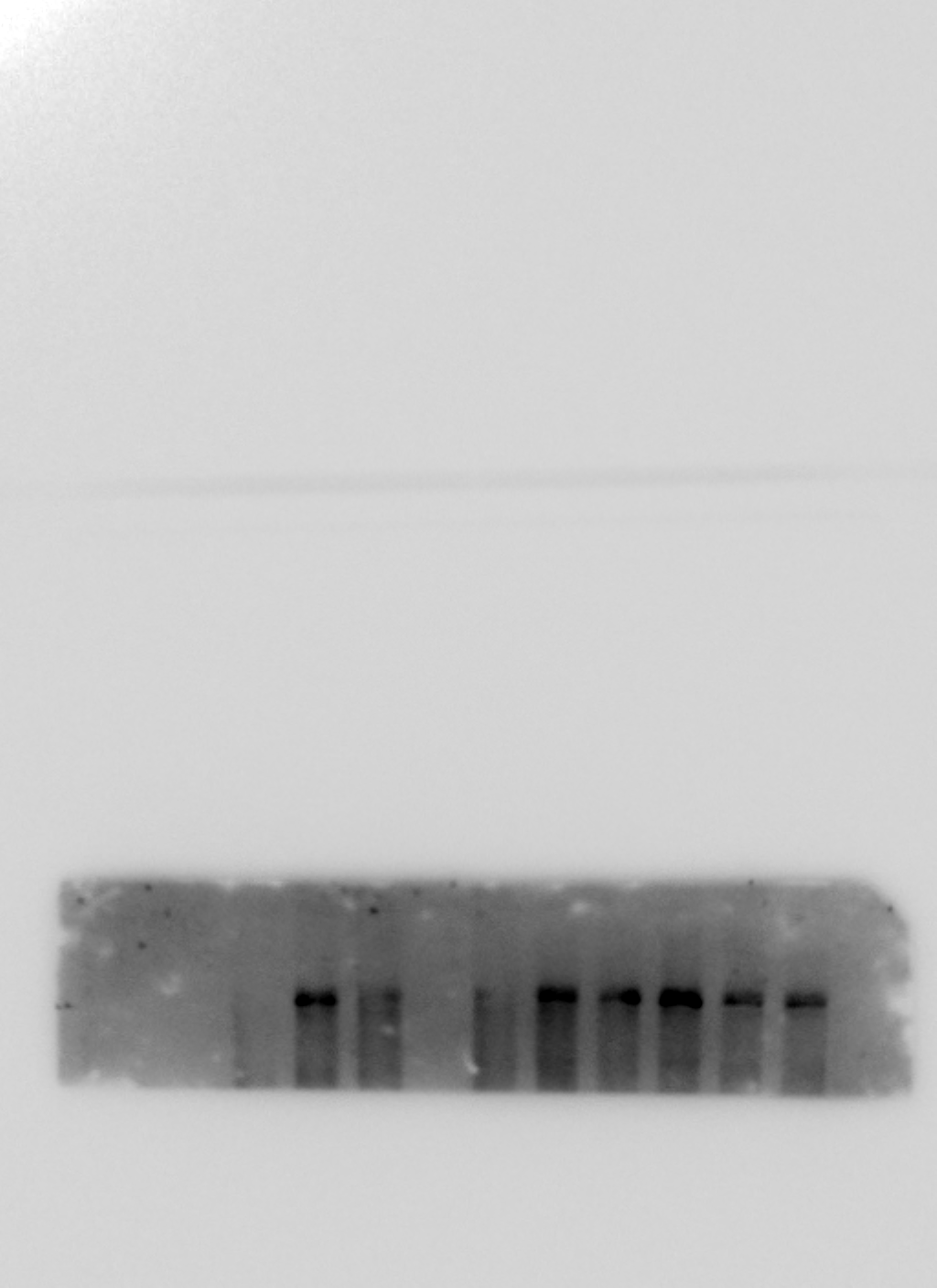

Supplement: Supplementary file 11 — Source data Fig. 6 [file 44321_2024_60_MOESM11_ESM.zip › Figure 6/6C/YAPC/Western phoPRKDC 7.1/4 4th phoPRK 7.1 _Ch.tif]

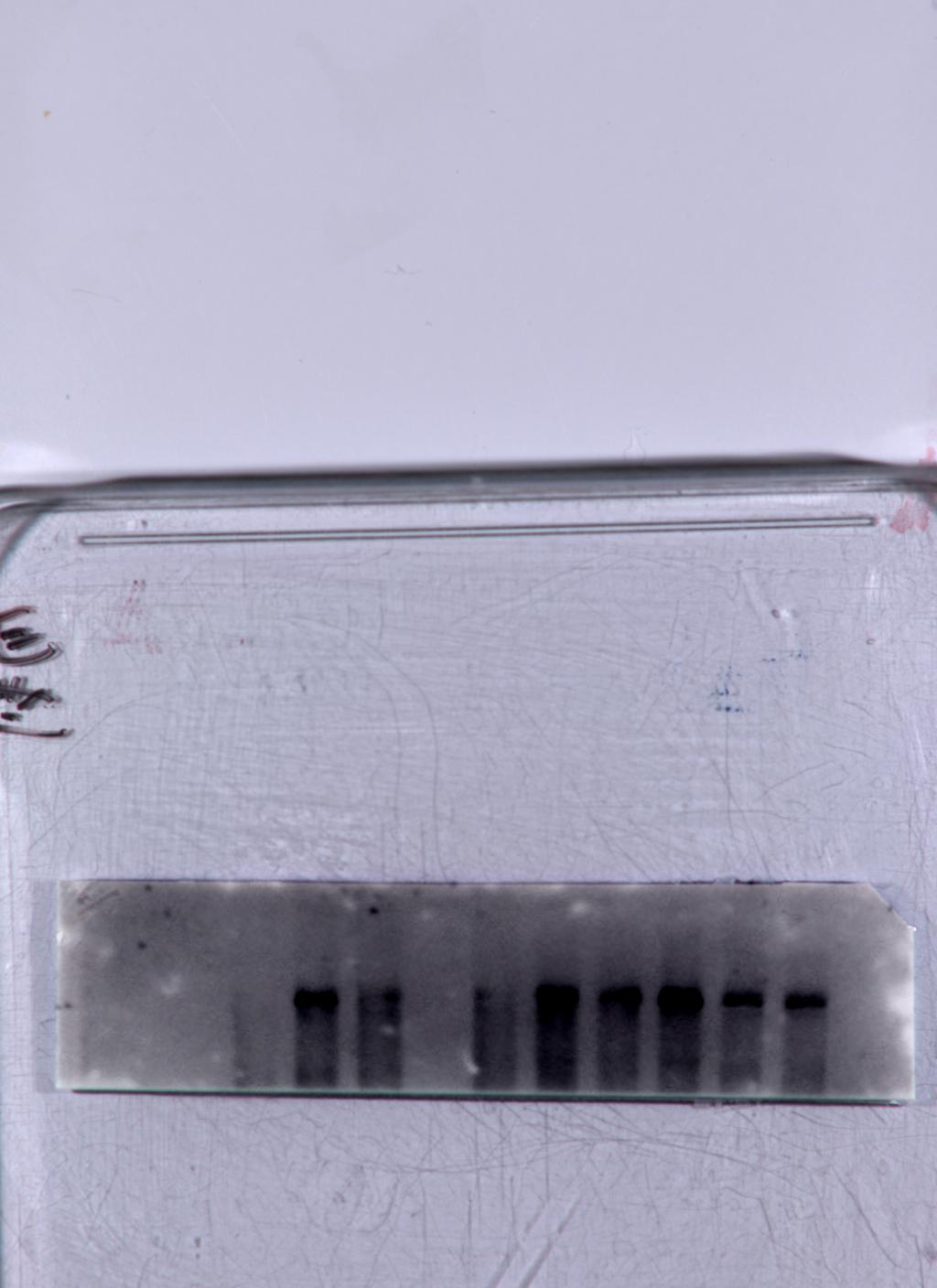

Supplement: Supplementary file 11 — Source data Fig. 6 [file 44321_2024_60_MOESM11_ESM.zip › Figure 6/6C/YAPC/Western phoPRKDC 7.1/4 4th phoPRK 7.1 _Ch+Marker.jpg]

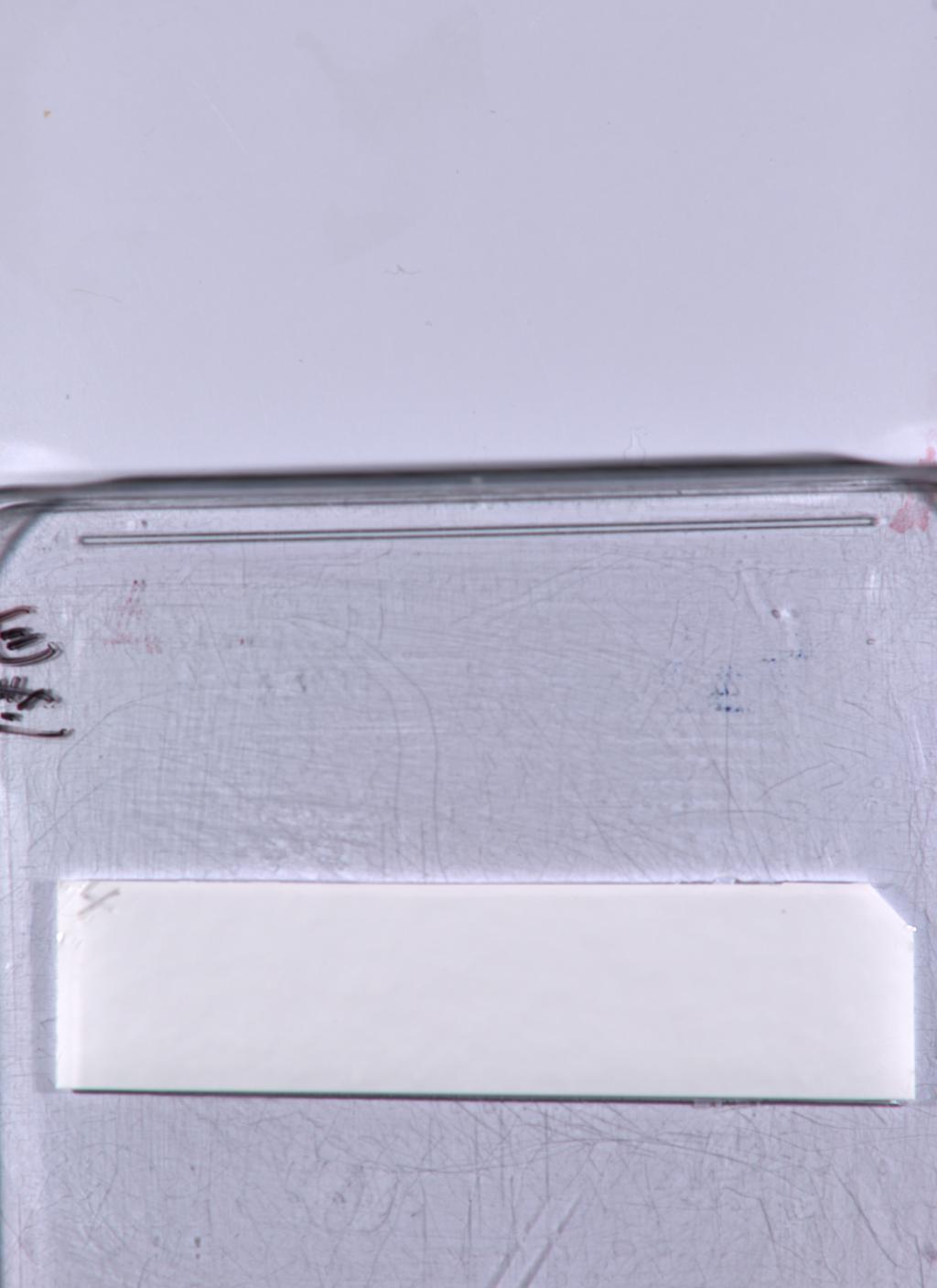

Supplement: Supplementary file 11 — Source data Fig. 6 [file 44321_2024_60_MOESM11_ESM.zip › Figure 6/6C/YAPC/Western phoPRKDC 7.1/4 4th phoPRK 7.1 _Ch-Marker.jpg]

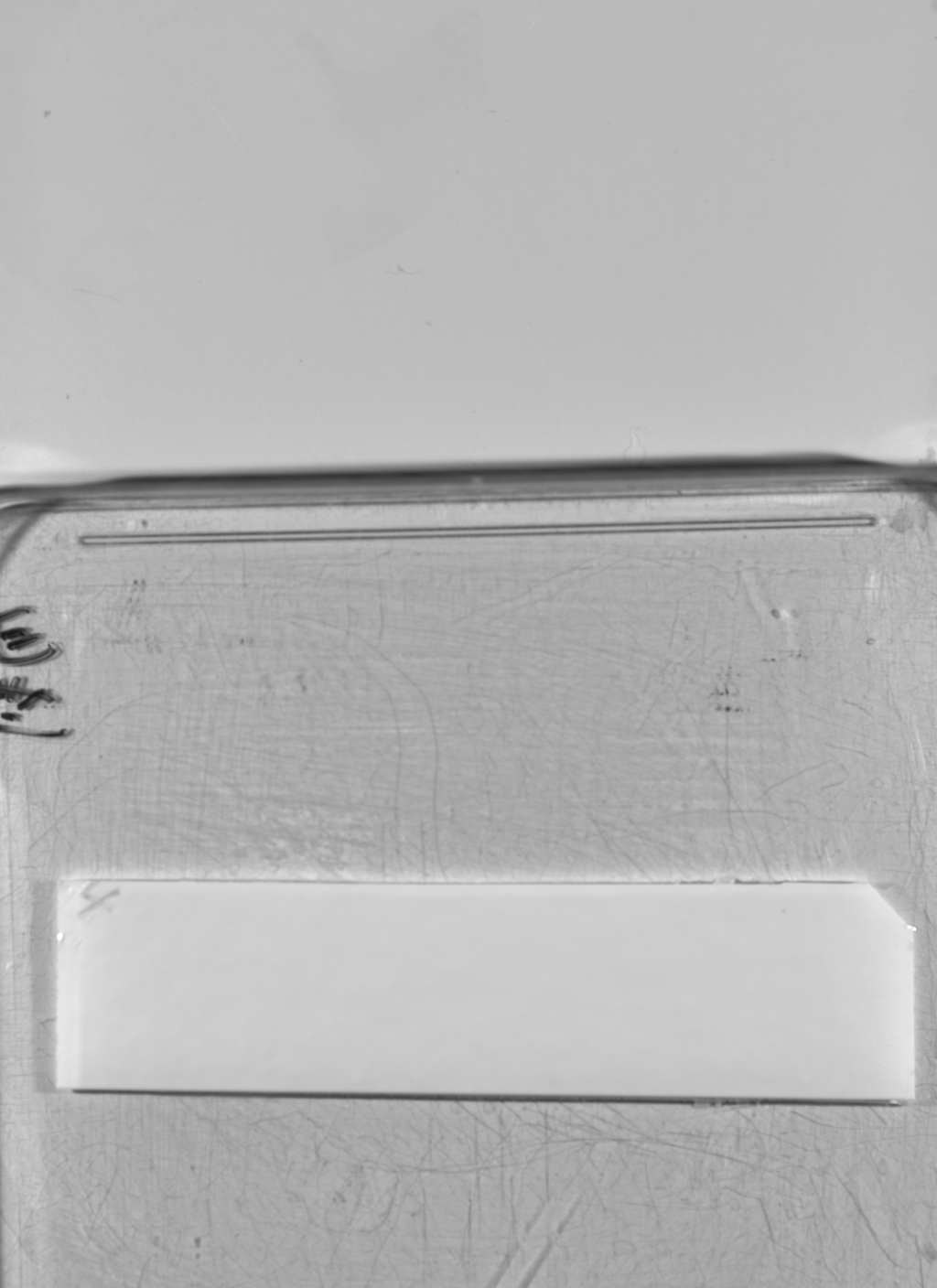

Supplement: Supplementary file 11 — Source data Fig. 6 [file 44321_2024_60_MOESM11_ESM.zip › Figure 6/6C/YAPC/Western phoPRKDC 7.1/4 4th phoPRK 7.1 _Ch-Marker.tif]

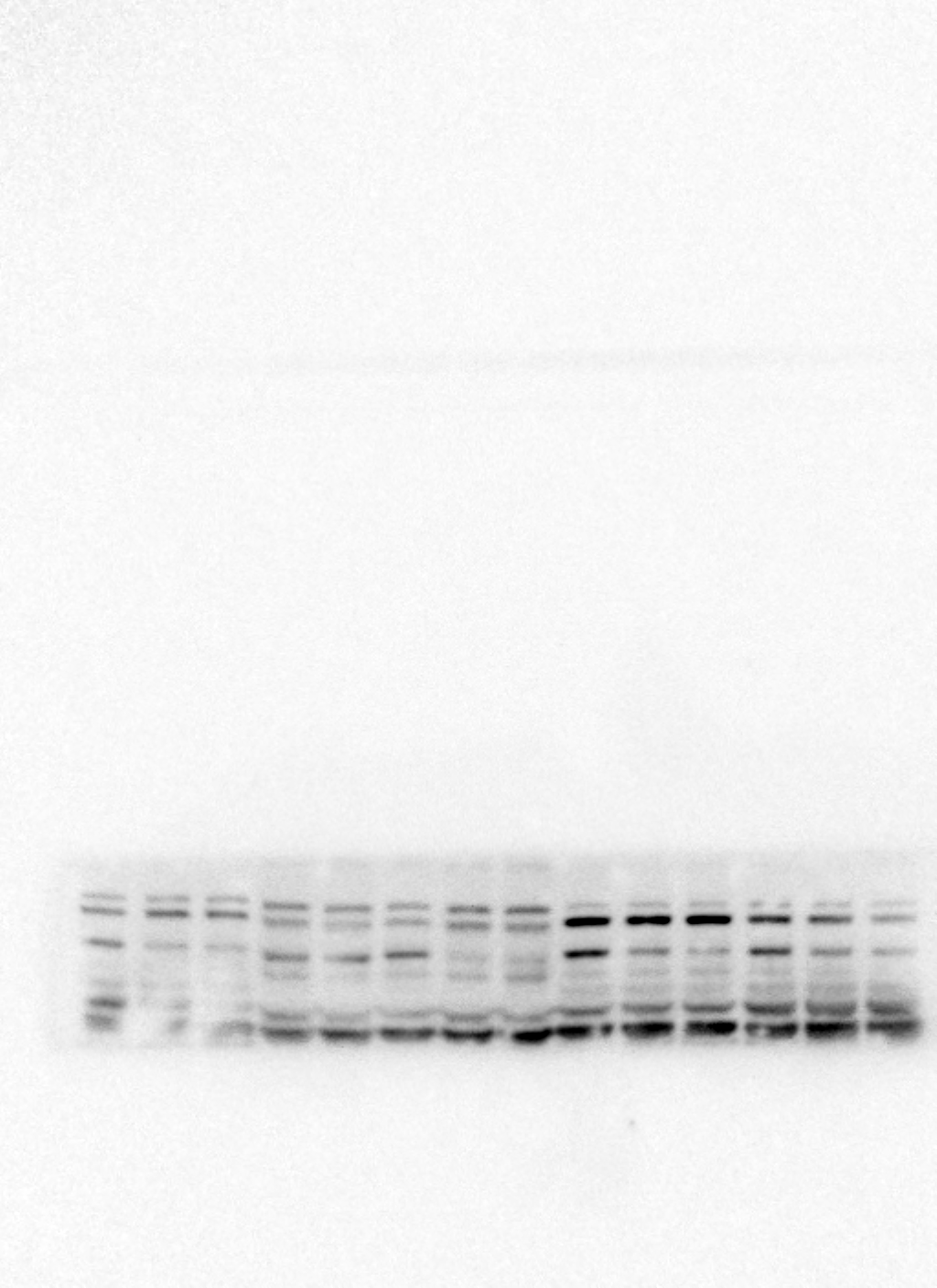

Supplement: Supplementary file 11 — Source data Fig. 6 [file 44321_2024_60_MOESM11_ESM.zip › Figure 6/6C/YAPC/Western PLK1 120/wsm 2 PLK1 120 _Ch.tif]

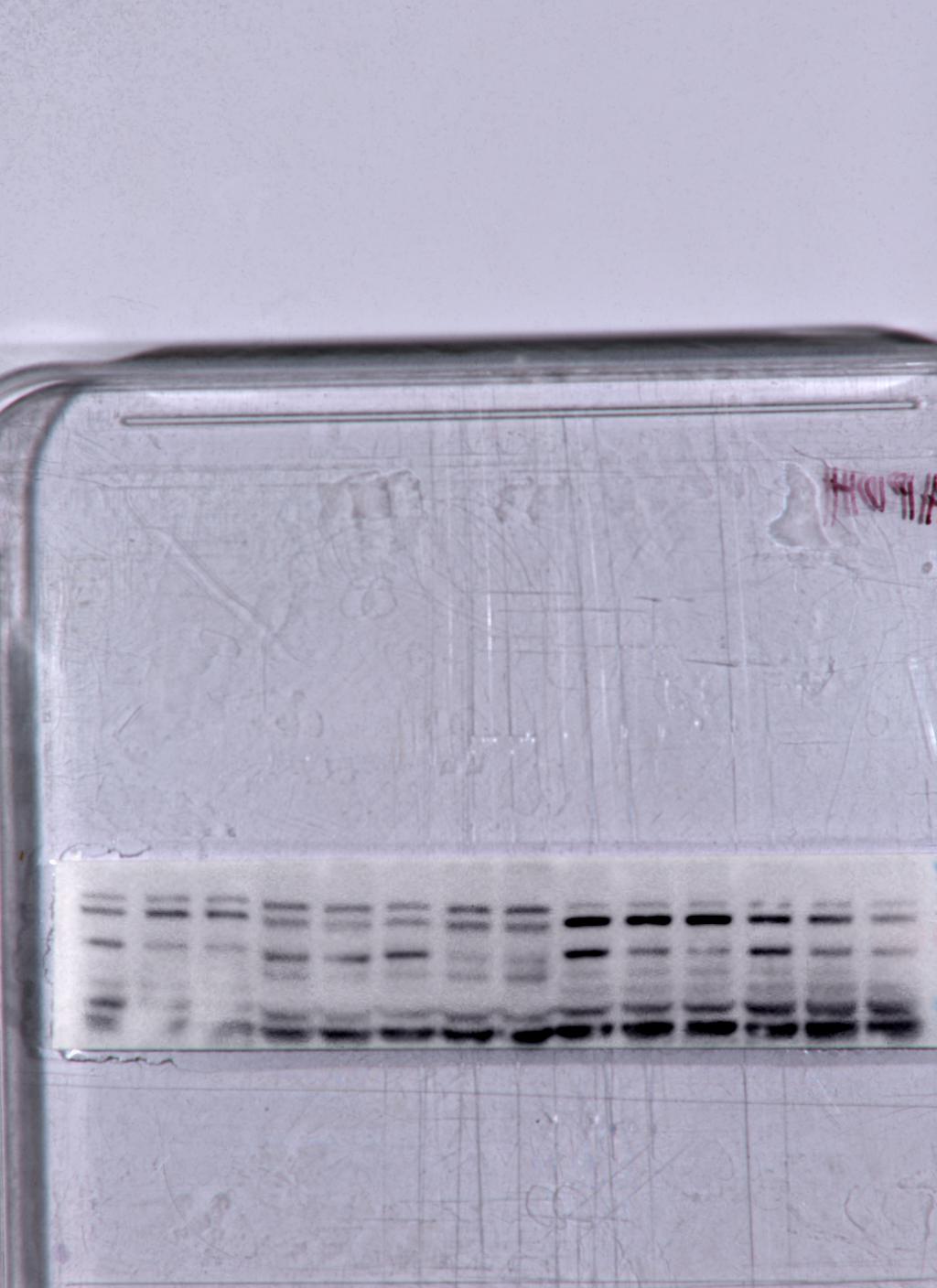

Supplement: Supplementary file 11 — Source data Fig. 6 [file 44321_2024_60_MOESM11_ESM.zip › Figure 6/6C/YAPC/Western PLK1 120/wsm 2 PLK1 120 _Ch+Marker.jpg]

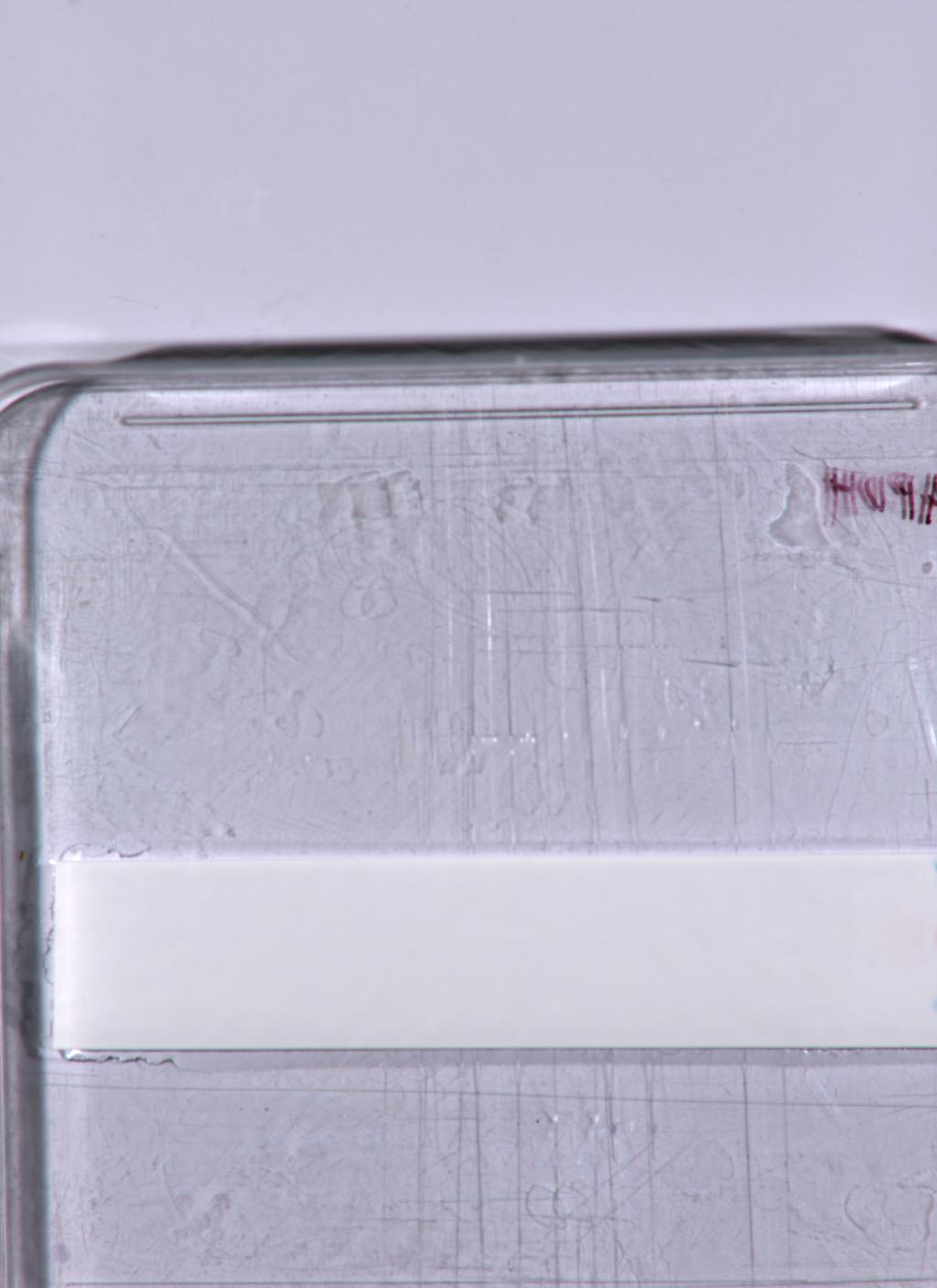

Supplement: Supplementary file 11 — Source data Fig. 6 [file 44321_2024_60_MOESM11_ESM.zip › Figure 6/6C/YAPC/Western PLK1 120/wsm 2 PLK1 120 _Ch-Marker.jpg]

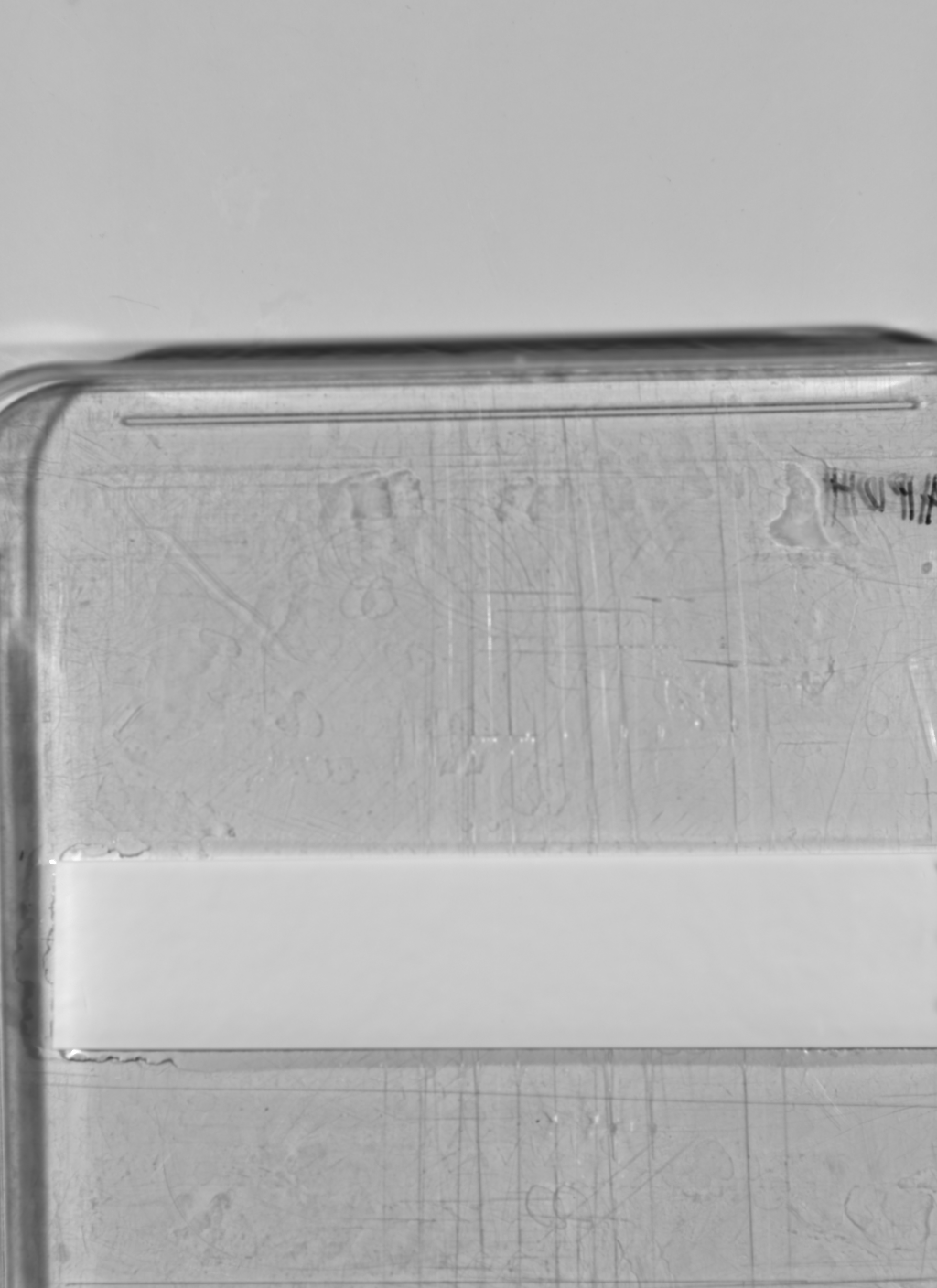

Supplement: Supplementary file 11 — Source data Fig. 6 [file 44321_2024_60_MOESM11_ESM.zip › Figure 6/6C/YAPC/Western PLK1 120/wsm 2 PLK1 120 _Ch-Marker.tif]

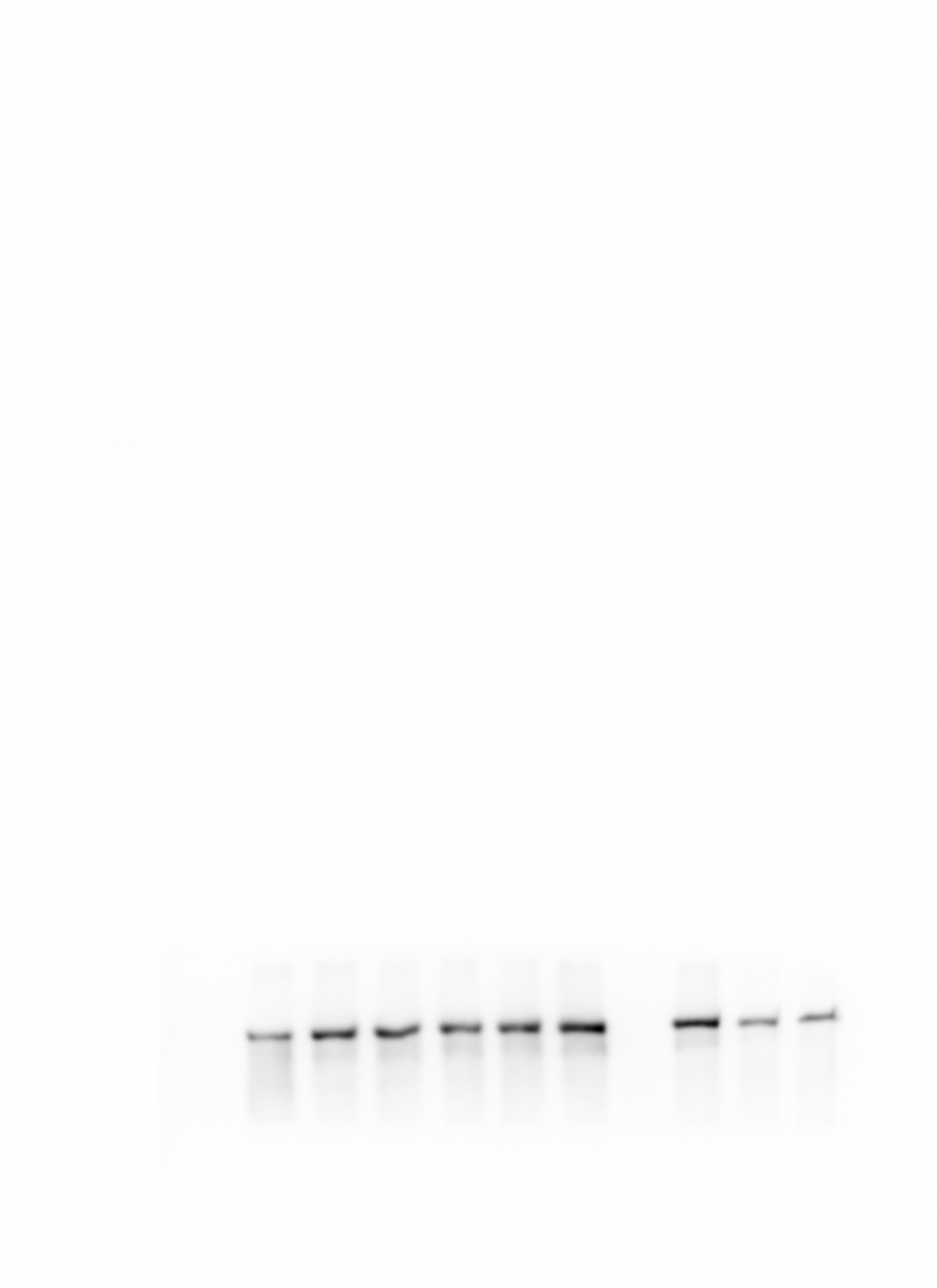

Supplement: Supplementary file 11 — Source data Fig. 6 [file 44321_2024_60_MOESM11_ESM.zip › Figure 6/6C/YAPC/Western PRKDC 0.2/A 2nd PRK 0.2 _Ch.tif]

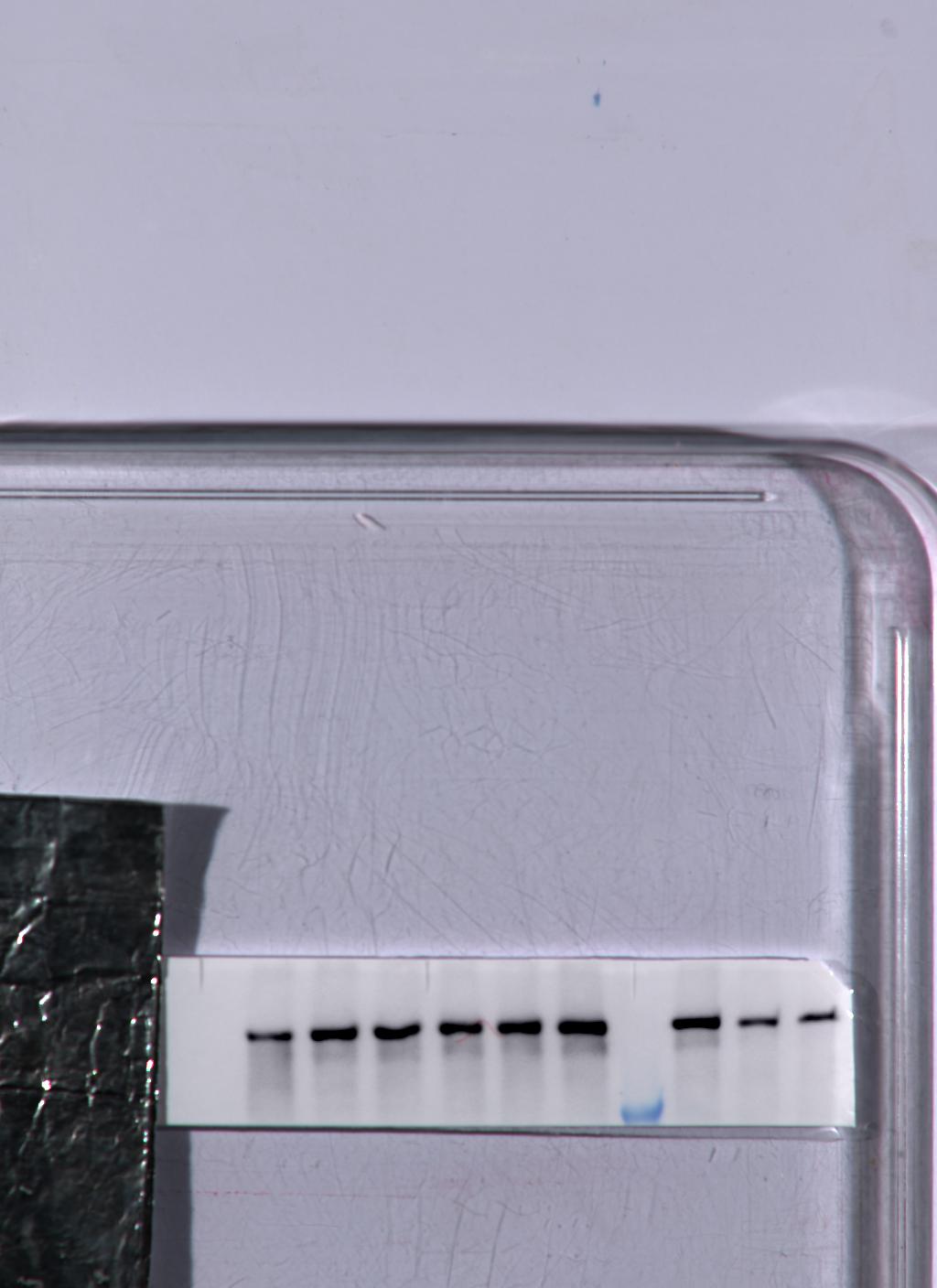

Supplement: Supplementary file 11 — Source data Fig. 6 [file 44321_2024_60_MOESM11_ESM.zip › Figure 6/6C/YAPC/Western PRKDC 0.2/A 2nd PRK 0.2 _Ch+Marker.jpg]

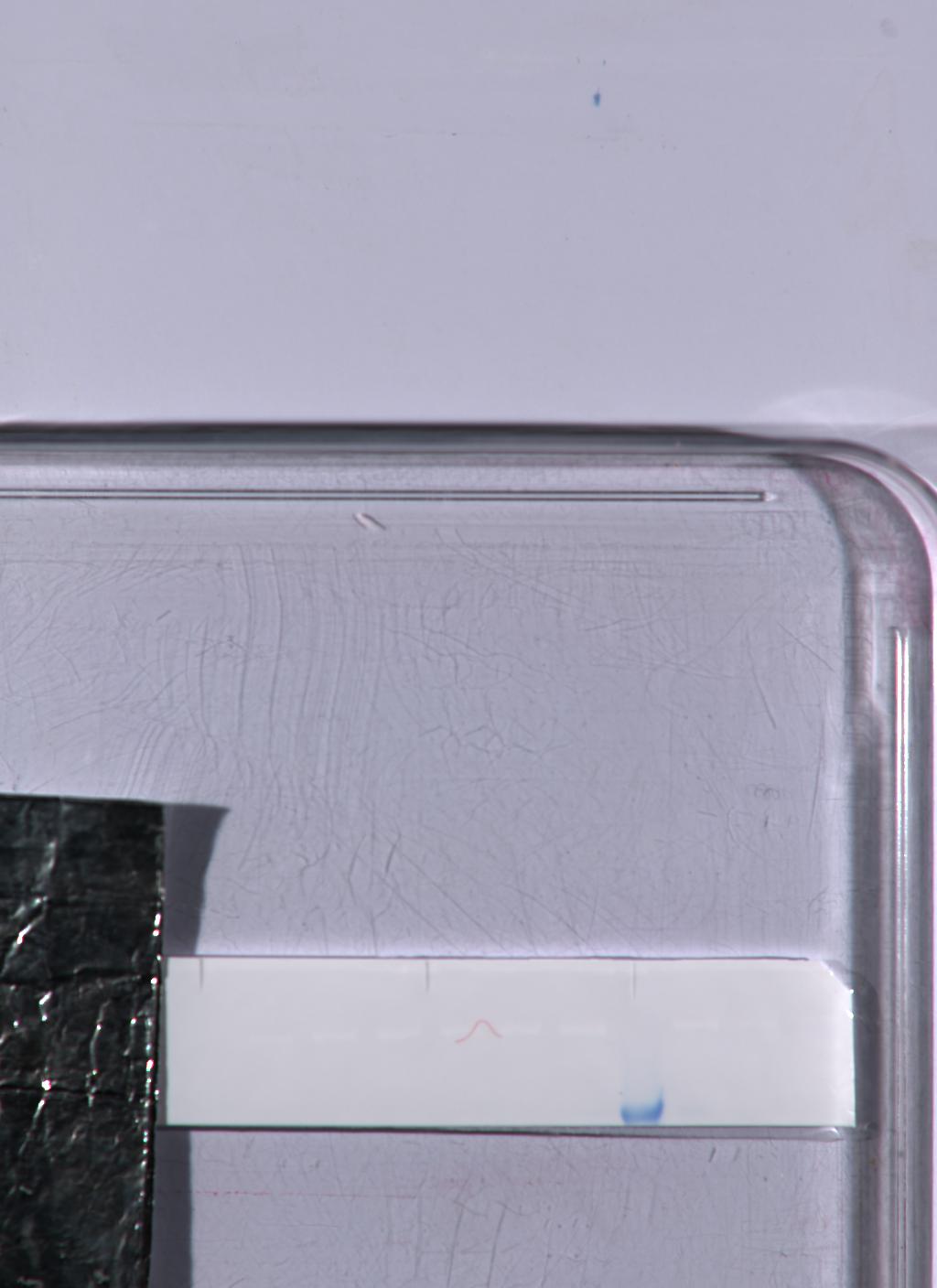

Supplement: Supplementary file 11 — Source data Fig. 6 [file 44321_2024_60_MOESM11_ESM.zip › Figure 6/6C/YAPC/Western PRKDC 0.2/A 2nd PRK 0.2 _Ch-Marker.jpg]

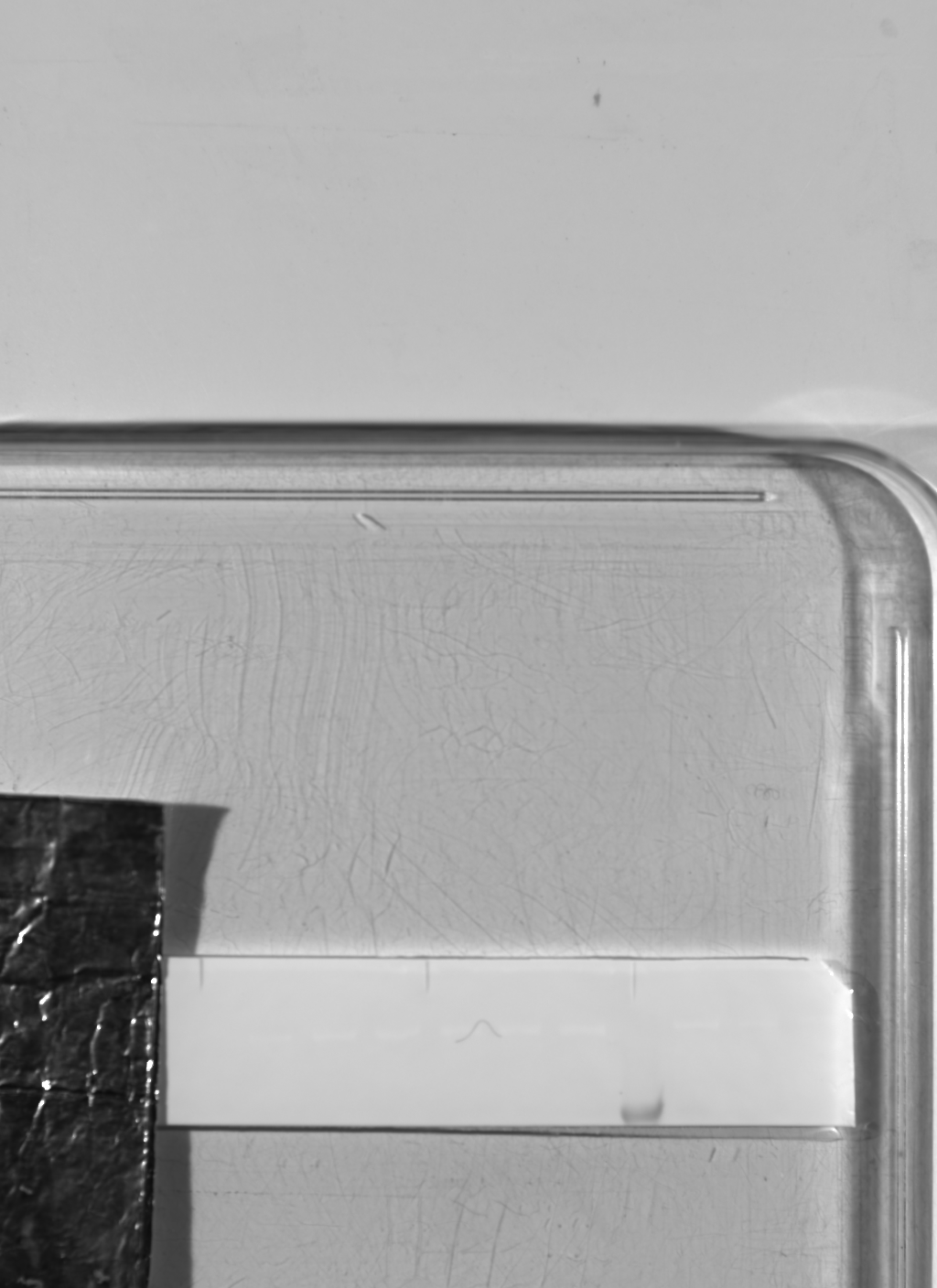

Supplement: Supplementary file 11 — Source data Fig. 6 [file 44321_2024_60_MOESM11_ESM.zip › Figure 6/6C/YAPC/Western PRKDC 0.2/A 2nd PRK 0.2 _Ch-Marker.tif]

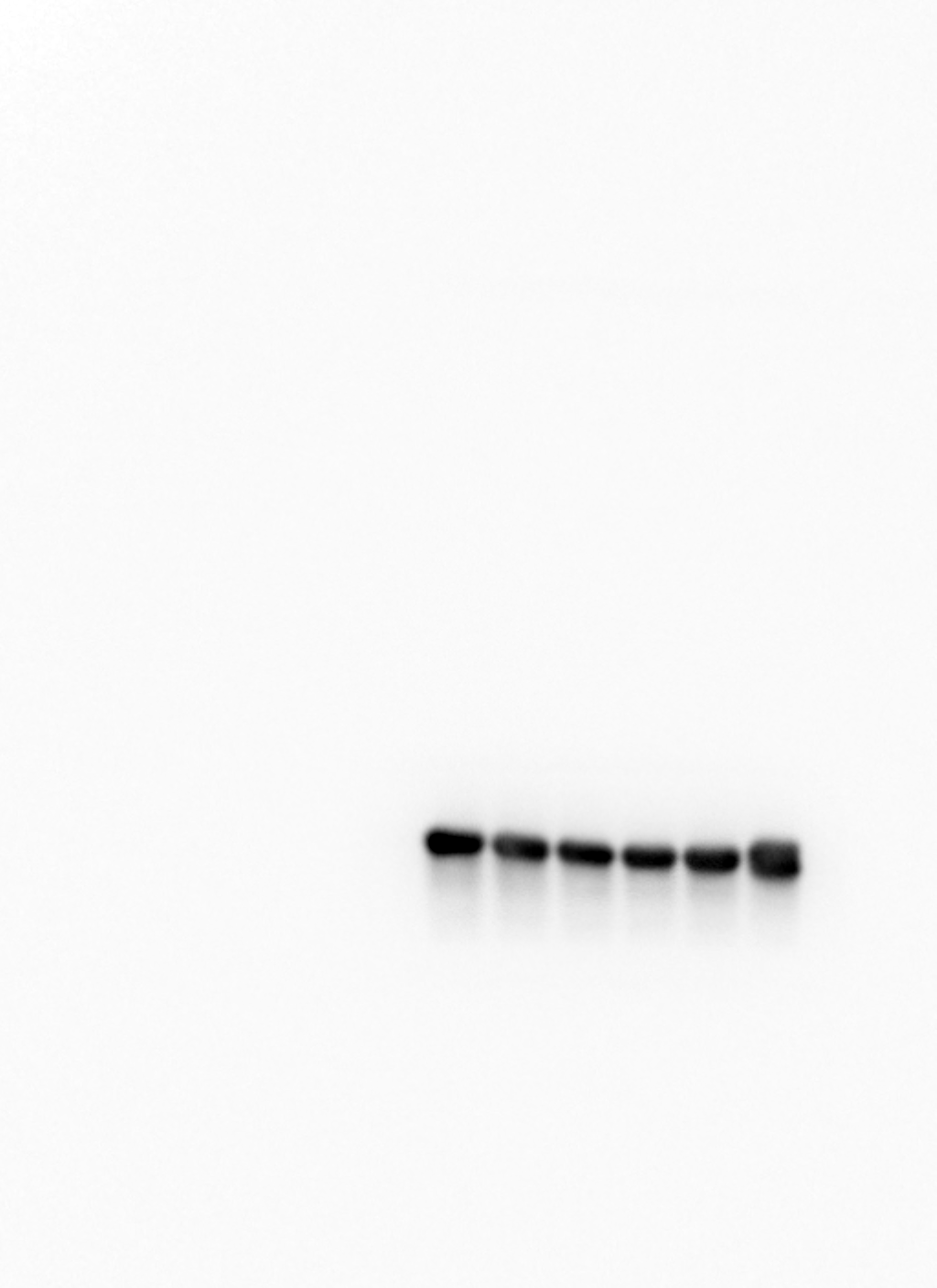

Supplement: Supplementary file 11 — Source data Fig. 6 [file 44321_2024_60_MOESM11_ESM.zip › Figure 6/6D/88T YAPC/Western GAPDH 0.7/5-1 1st GAP 0.7 _Ch.tif]

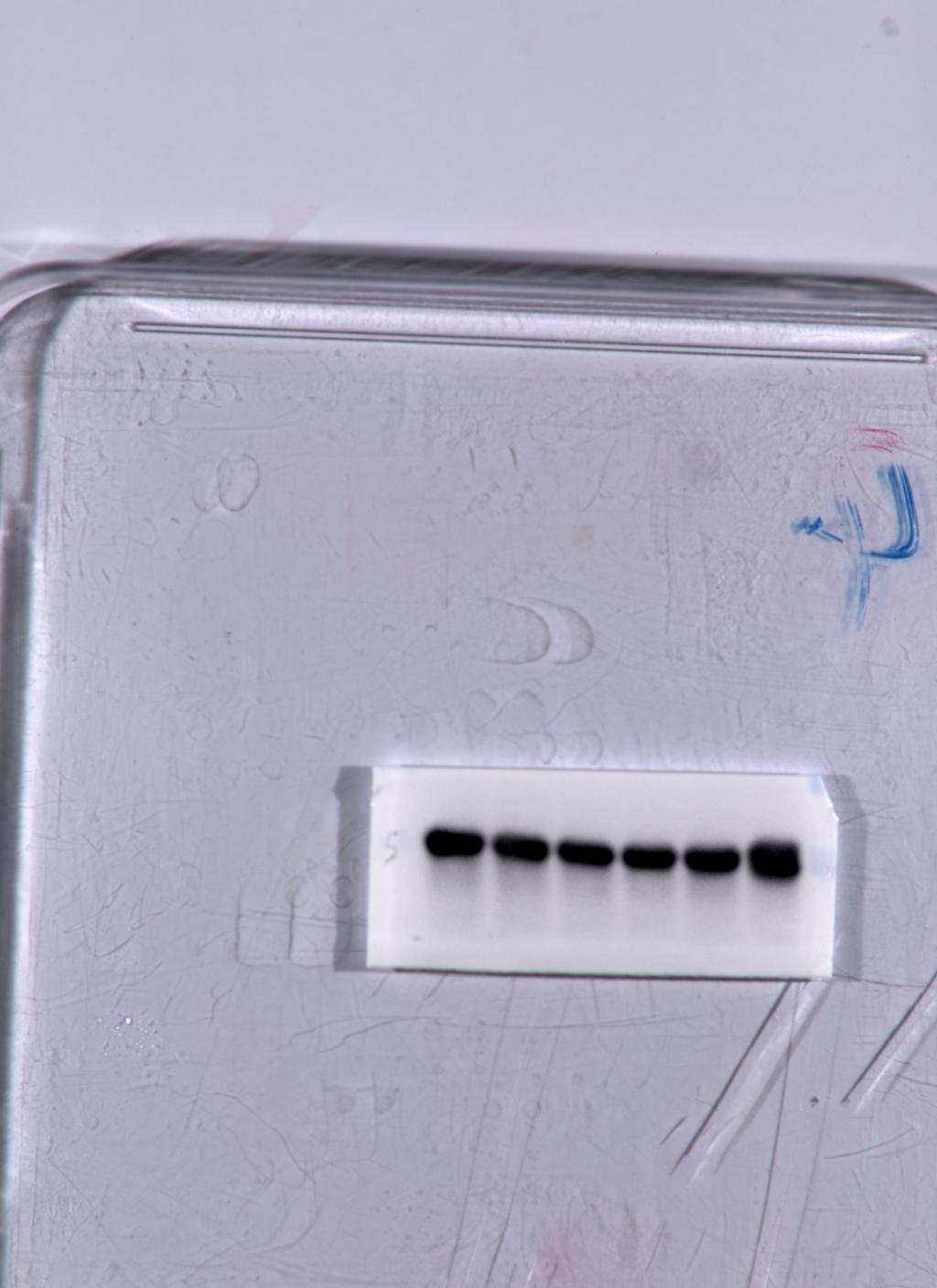

Supplement: Supplementary file 11 — Source data Fig. 6 [file 44321_2024_60_MOESM11_ESM.zip › Figure 6/6D/88T YAPC/Western GAPDH 0.7/5-1 1st GAP 0.7 _Ch+Marker.jpg]

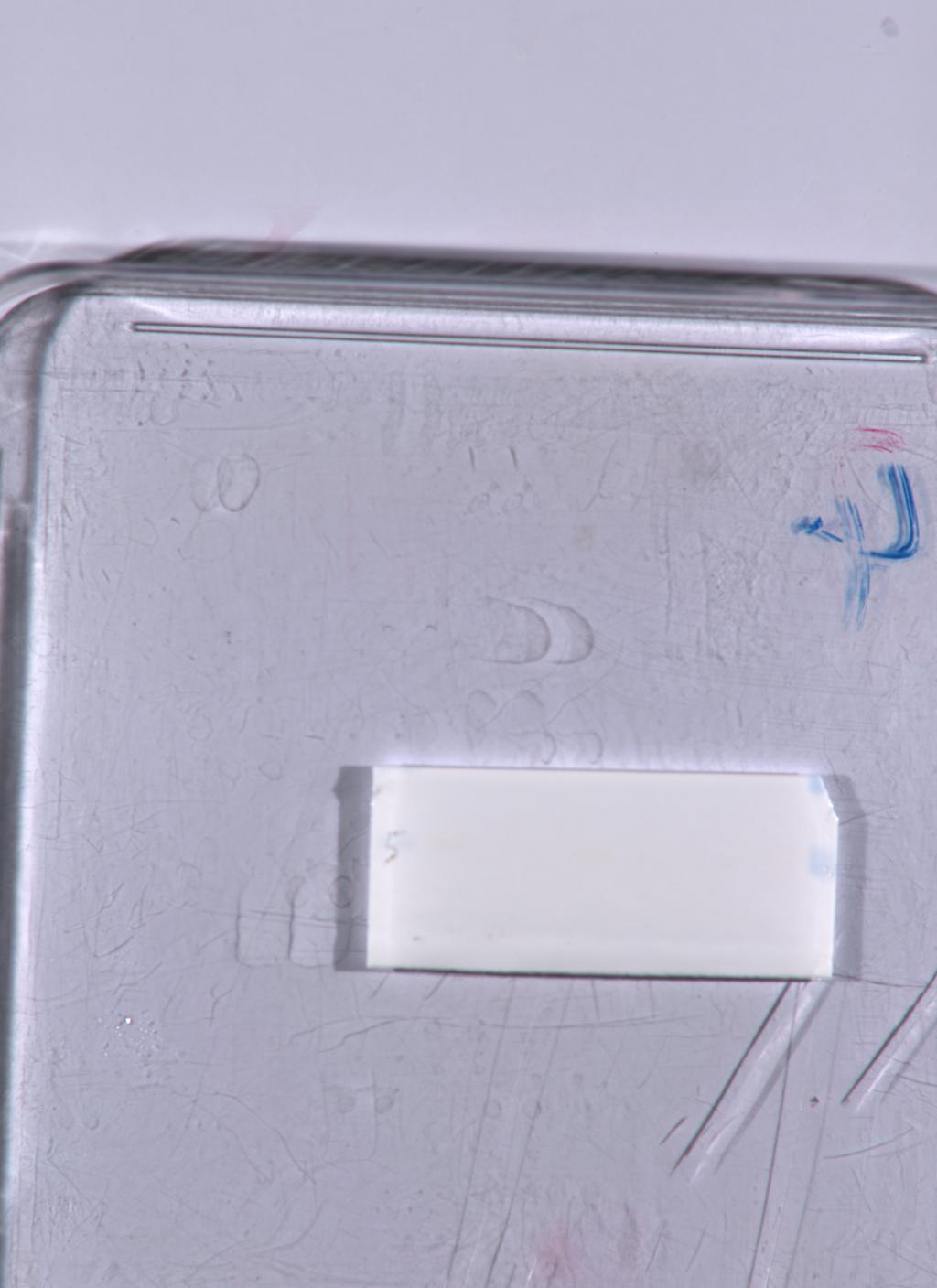

Supplement: Supplementary file 11 — Source data Fig. 6 [file 44321_2024_60_MOESM11_ESM.zip › Figure 6/6D/88T YAPC/Western GAPDH 0.7/5-1 1st GAP 0.7 _Ch-Marker.jpg]

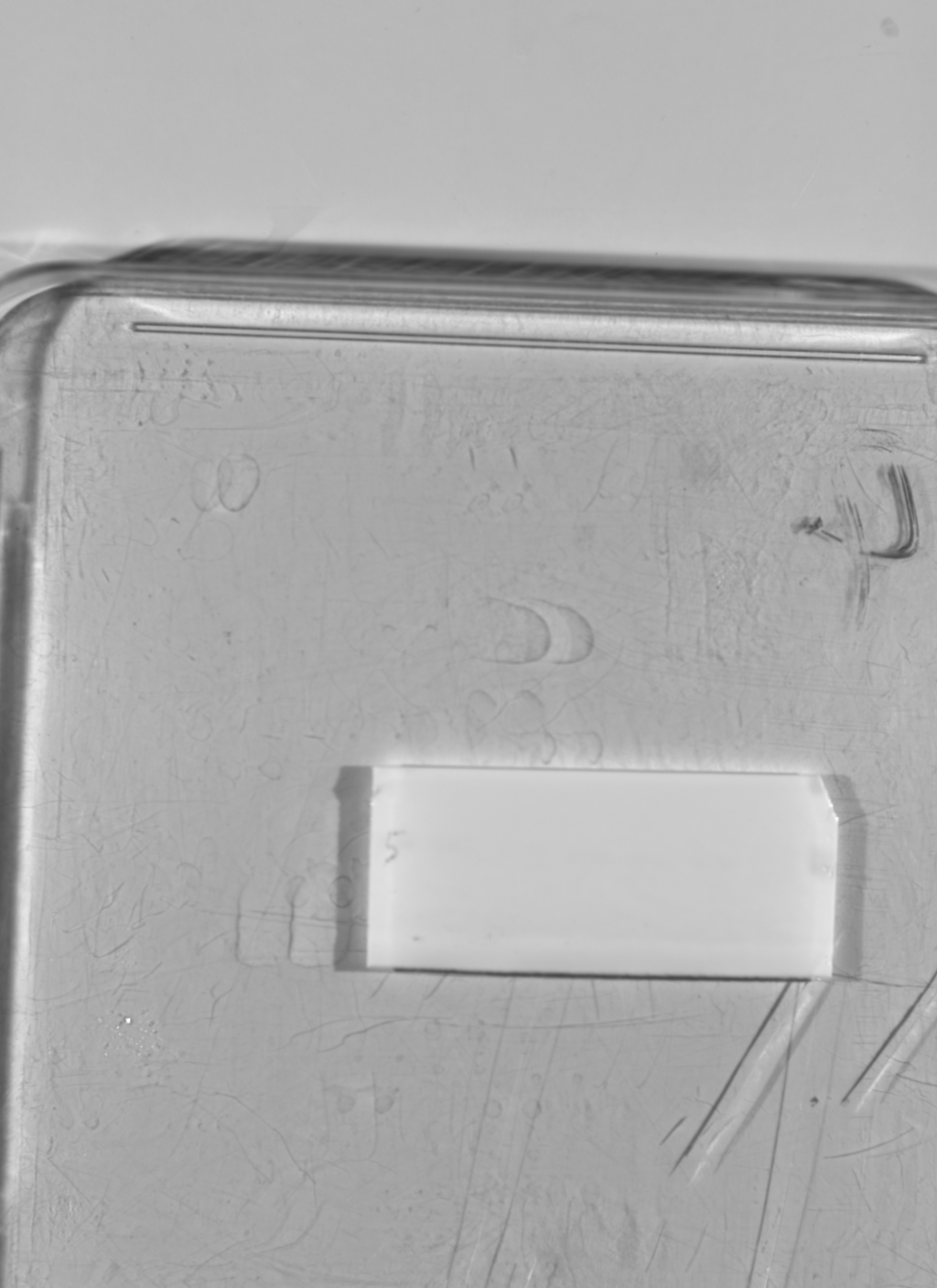

Supplement: Supplementary file 11 — Source data Fig. 6 [file 44321_2024_60_MOESM11_ESM.zip › Figure 6/6D/88T YAPC/Western GAPDH 0.7/5-1 1st GAP 0.7 _Ch-Marker.tif]

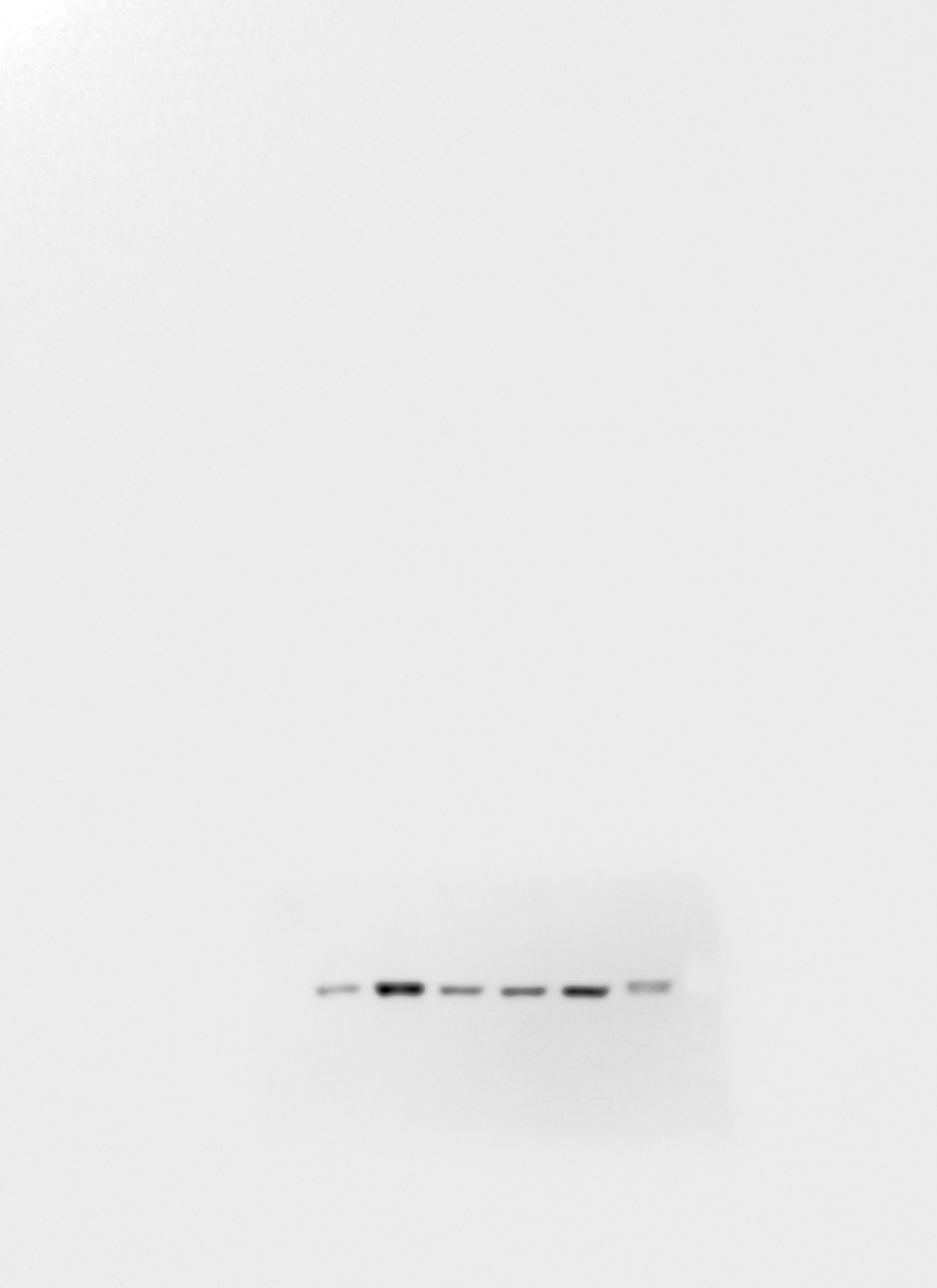

Supplement: Supplementary file 11 — Source data Fig. 6 [file 44321_2024_60_MOESM11_ESM.zip › Figure 6/6D/88T YAPC/Western PLK1 3.9/5-1 3th PLK 3.9 _Ch.tif]

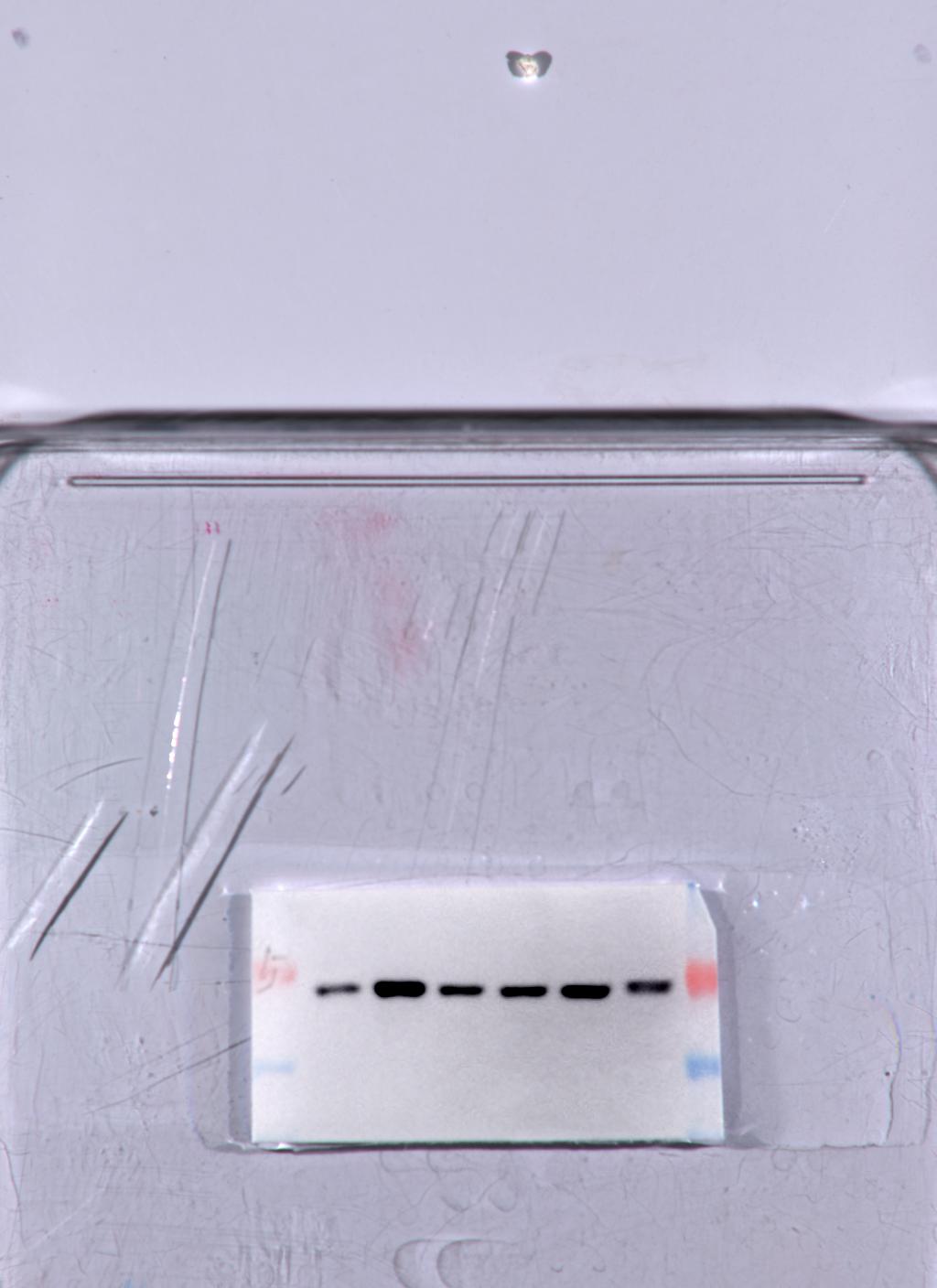

Supplement: Supplementary file 11 — Source data Fig. 6 [file 44321_2024_60_MOESM11_ESM.zip › Figure 6/6D/88T YAPC/Western PLK1 3.9/5-1 3th PLK 3.9 _Ch+Marker.jpg]

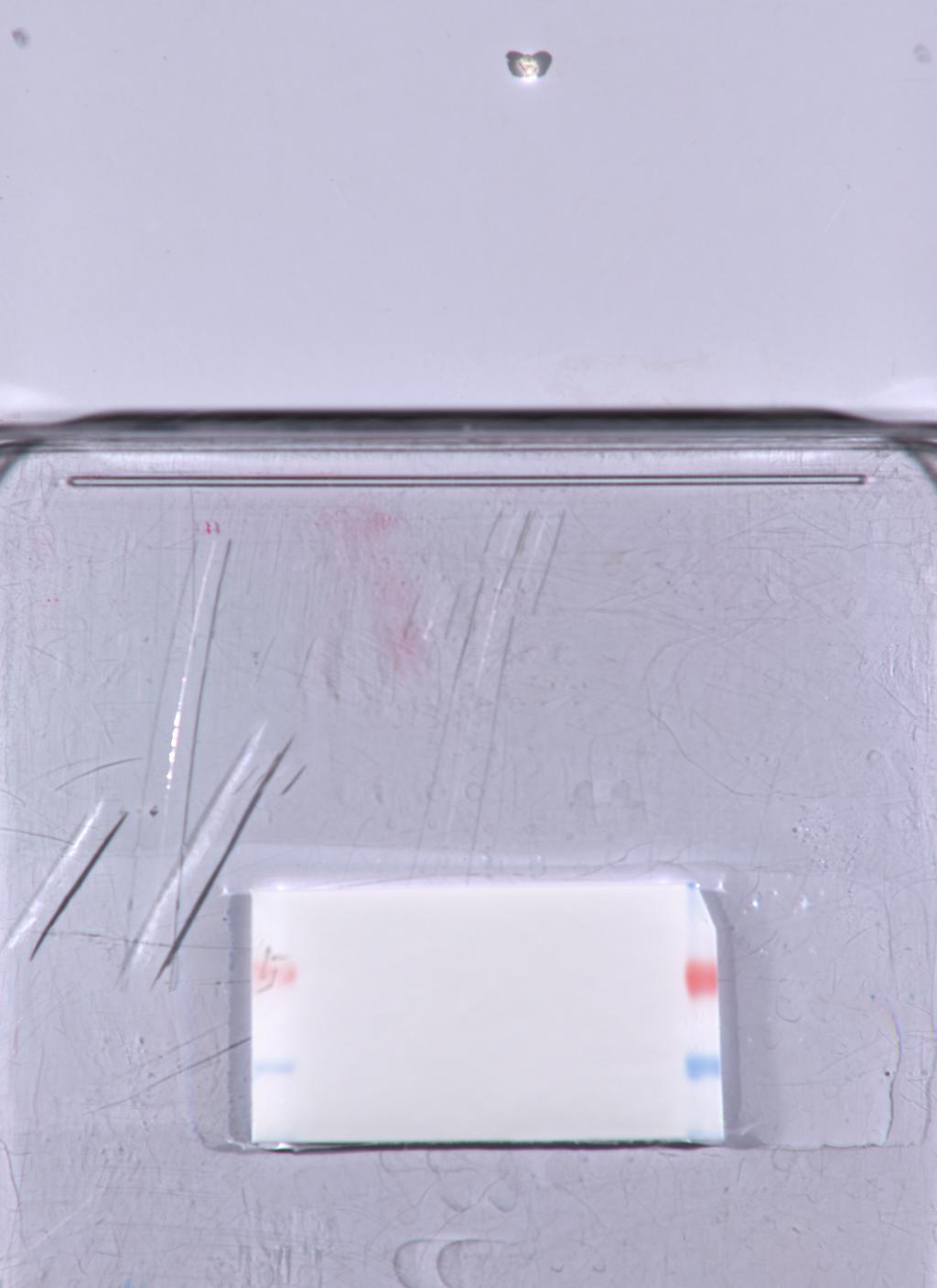

Supplement: Supplementary file 11 — Source data Fig. 6 [file 44321_2024_60_MOESM11_ESM.zip › Figure 6/6D/88T YAPC/Western PLK1 3.9/5-1 3th PLK 3.9 _Ch-Marker.jpg]

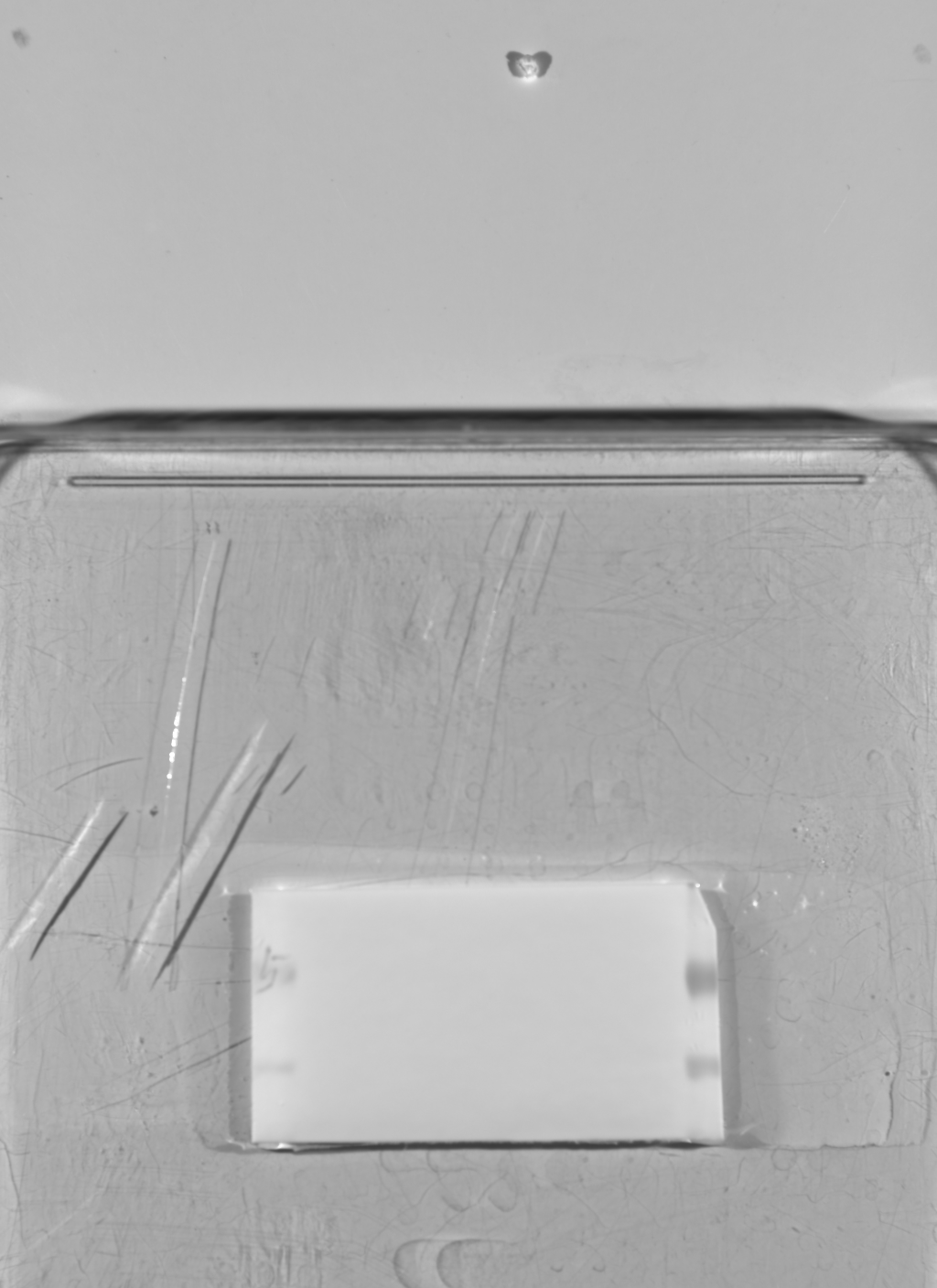

Supplement: Supplementary file 11 — Source data Fig. 6 [file 44321_2024_60_MOESM11_ESM.zip › Figure 6/6D/88T YAPC/Western PLK1 3.9/5-1 3th PLK 3.9 _Ch-Marker.tif]

## Slide 1
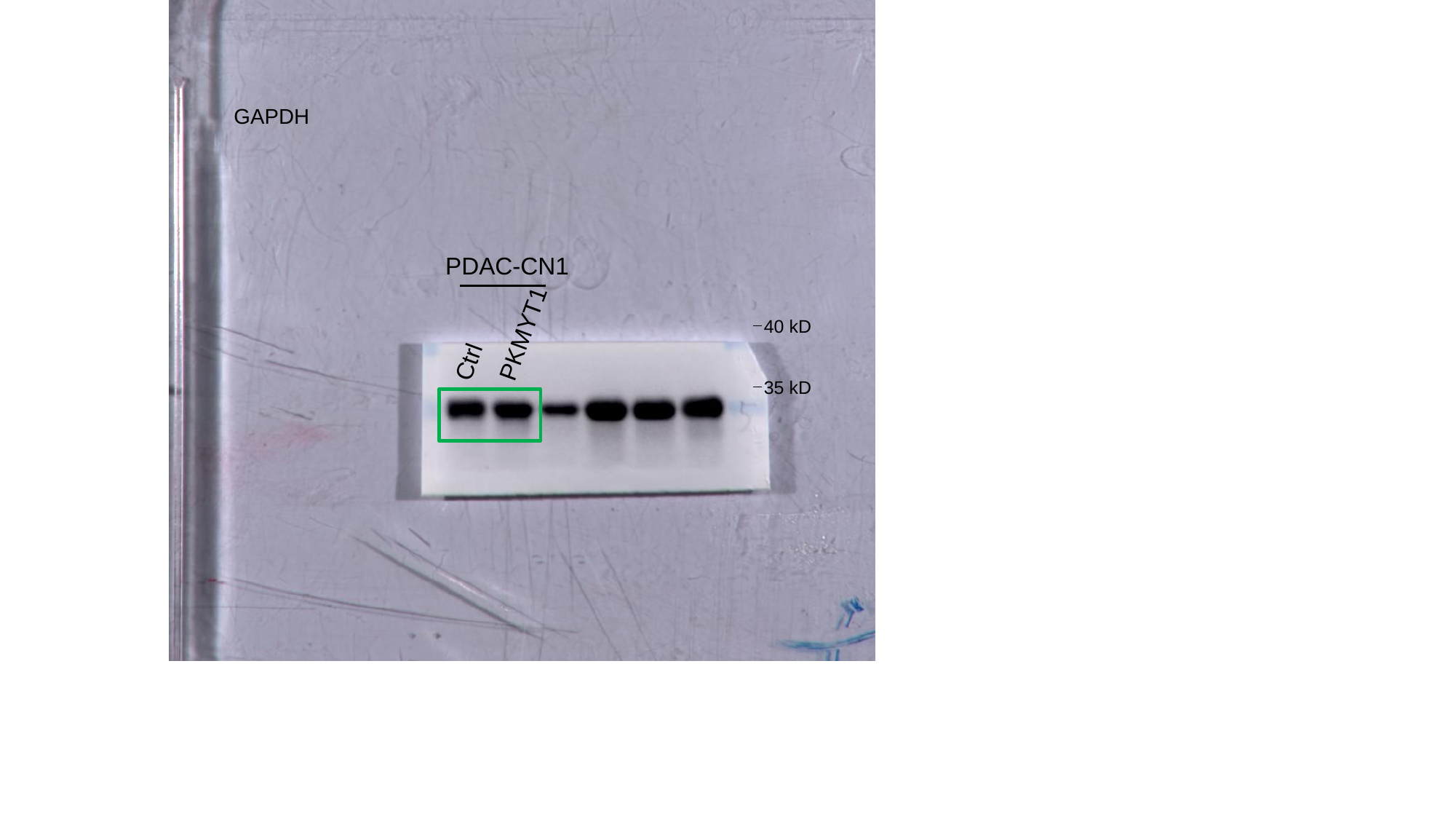

GAPDH
PDAC-CN1
PKMYT1
Ctrl
40 kD
35 kD

## Slide 2
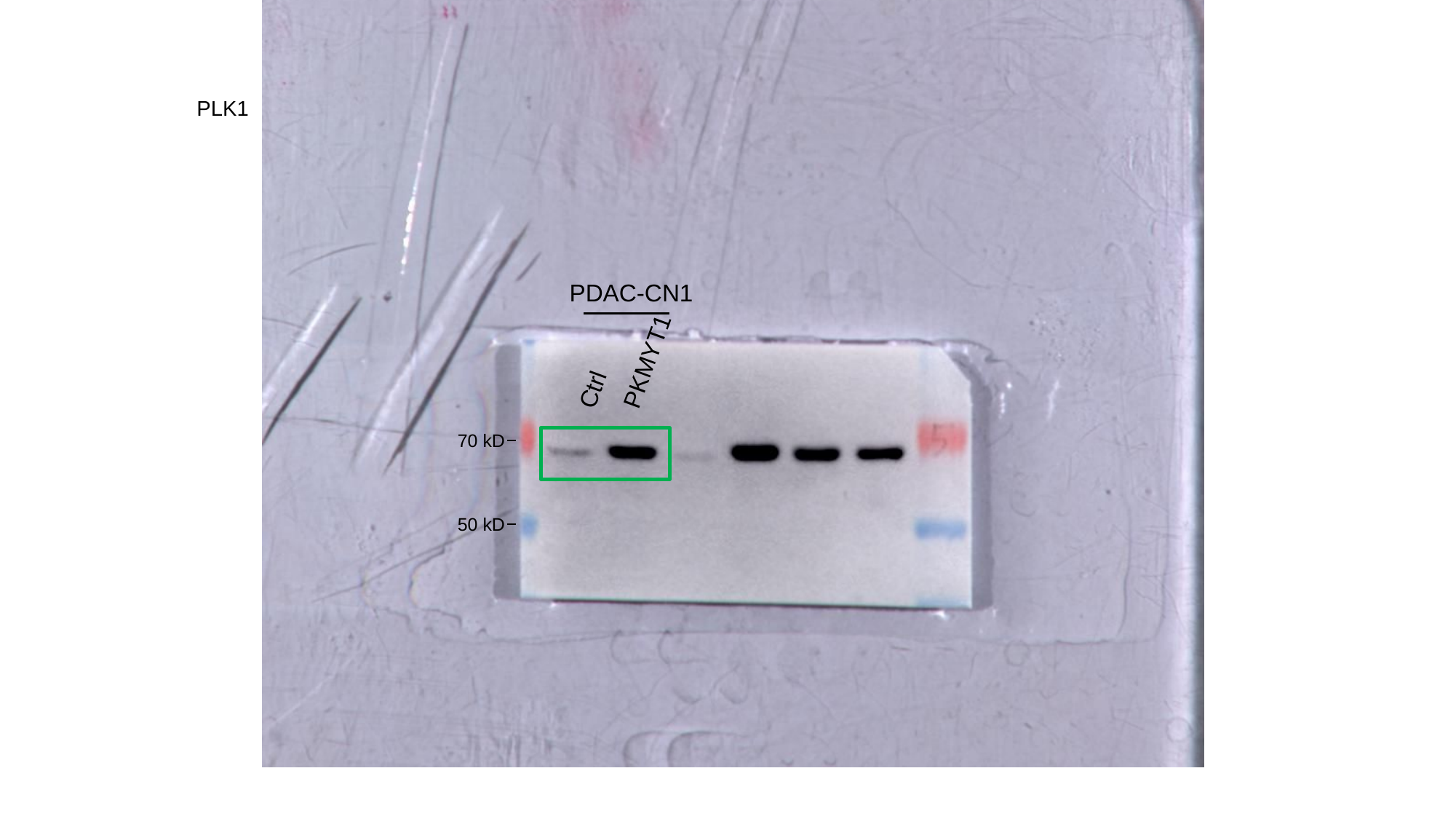

PLK1
PDAC-CN1
PKMYT1
Ctrl
70 kD
50 kD

Supplement: Supplementary file 11 — Source data Fig. 6 [file 44321_2024_60_MOESM11_ESM.zip › Figure 6/6D/CN1/6D CN1.pptx]

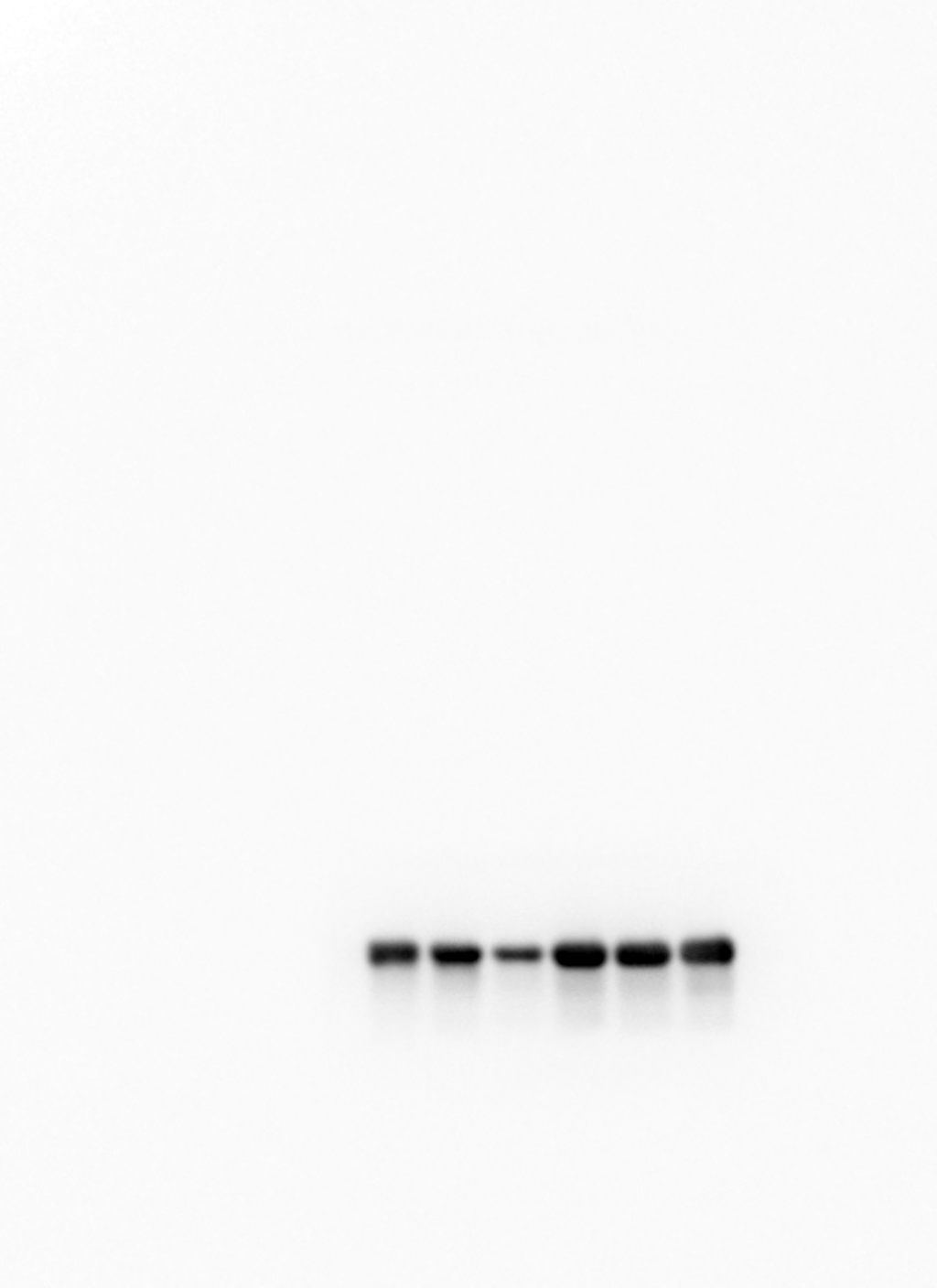

Supplement: Supplementary file 11 — Source data Fig. 6 [file 44321_2024_60_MOESM11_ESM.zip › Figure 6/6D/CN1/Western GAPDH 0.8/5-2 1st GAP 0.8 _Ch.tif]

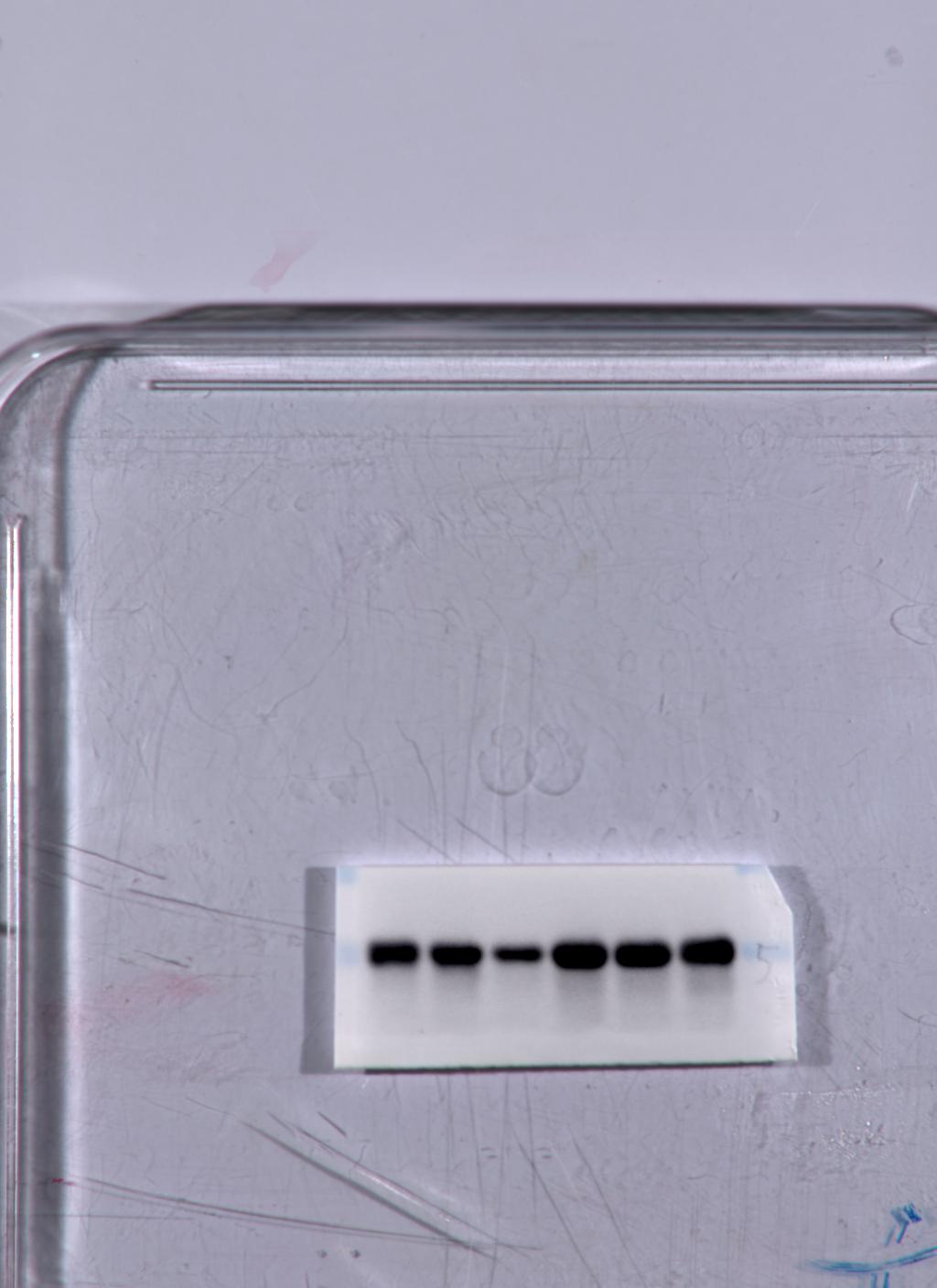

Supplement: Supplementary file 11 — Source data Fig. 6 [file 44321_2024_60_MOESM11_ESM.zip › Figure 6/6D/CN1/Western GAPDH 0.8/5-2 1st GAP 0.8 _Ch+Marker.jpg]

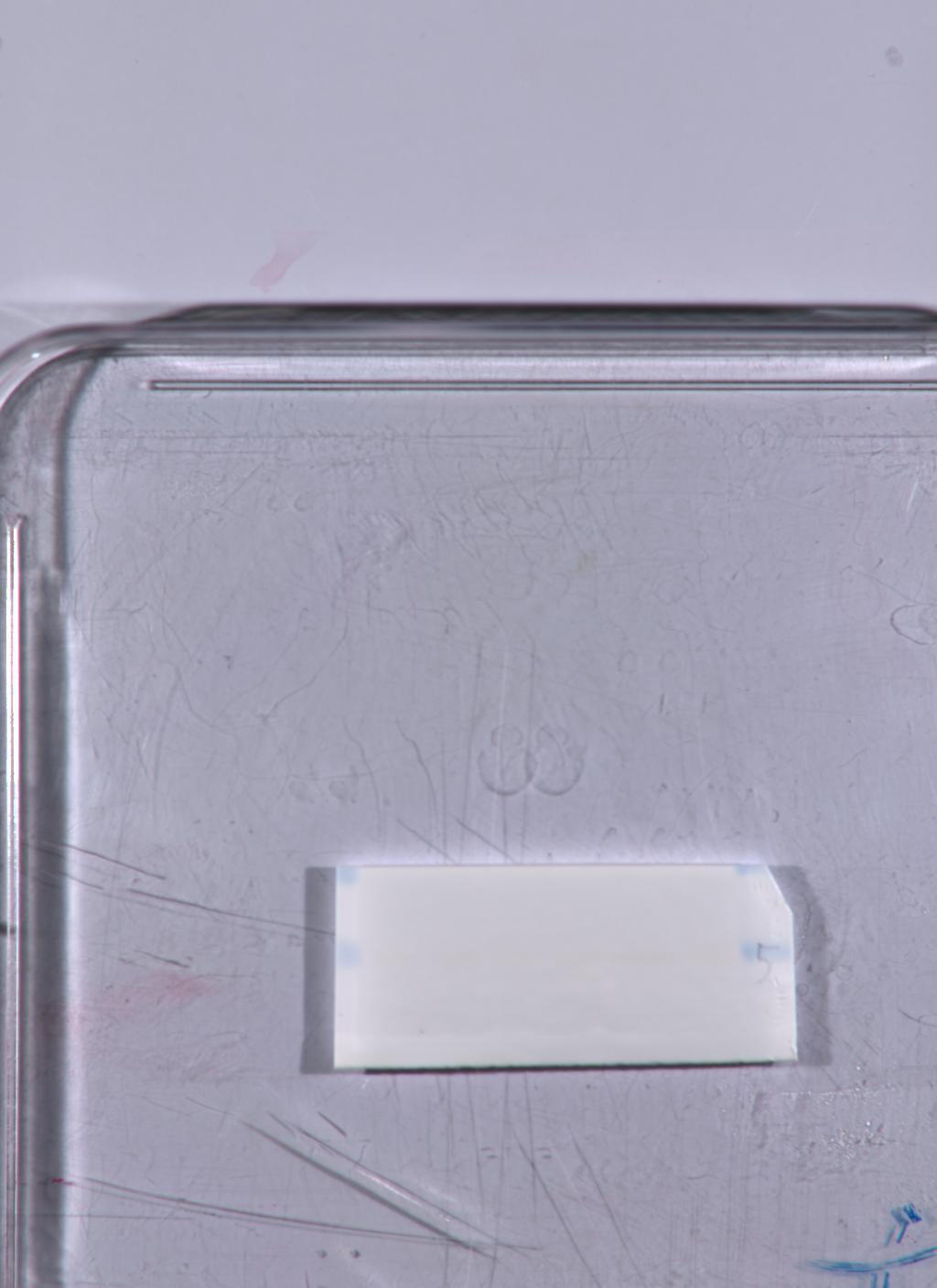

Supplement: Supplementary file 11 — Source data Fig. 6 [file 44321_2024_60_MOESM11_ESM.zip › Figure 6/6D/CN1/Western GAPDH 0.8/5-2 1st GAP 0.8 _Ch-Marker.jpg]

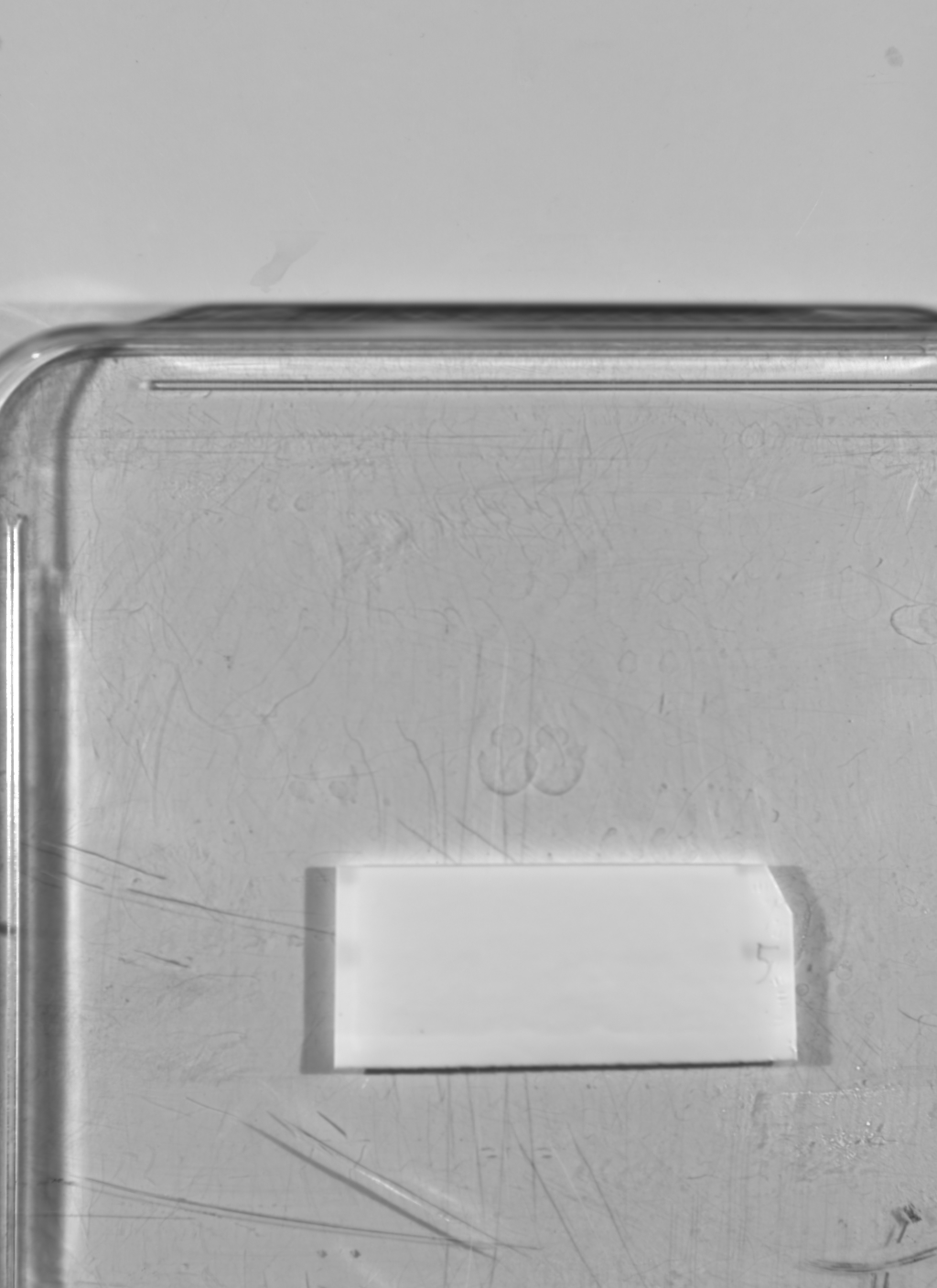

Supplement: Supplementary file 11 — Source data Fig. 6 [file 44321_2024_60_MOESM11_ESM.zip › Figure 6/6D/CN1/Western GAPDH 0.8/5-2 1st GAP 0.8 _Ch-Marker.tif]

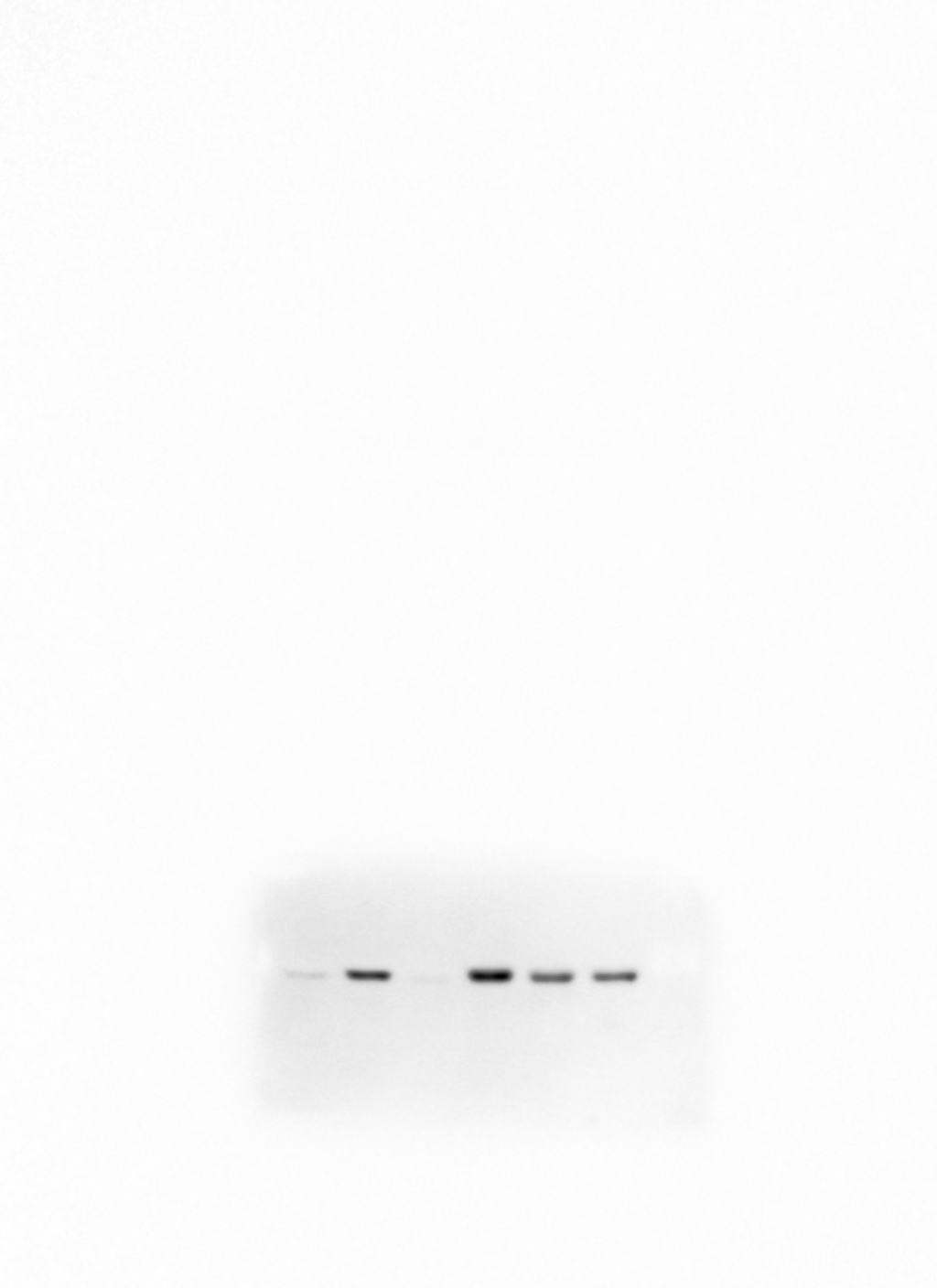

Supplement: Supplementary file 11 — Source data Fig. 6 [file 44321_2024_60_MOESM11_ESM.zip › Figure 6/6D/CN1/Western PLK1 9.9/5-2 3th PLK 9.9 2022.05.12_13.37.43_Ch.tif]

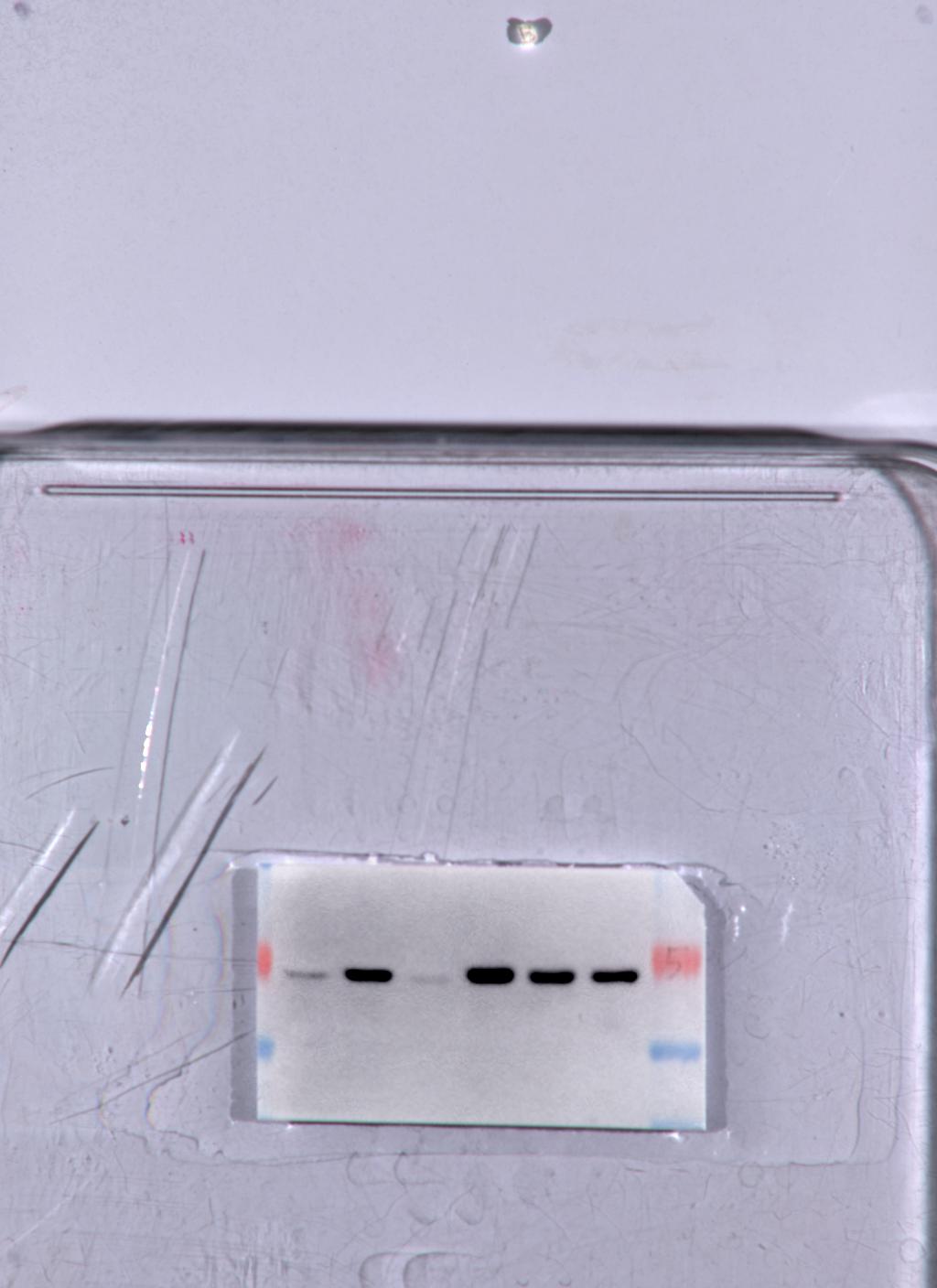

Supplement: Supplementary file 11 — Source data Fig. 6 [file 44321_2024_60_MOESM11_ESM.zip › Figure 6/6D/CN1/Western PLK1 9.9/5-2 3th PLK 9.9 2022.05.12_13.37.43_Ch+Marker.jpg]

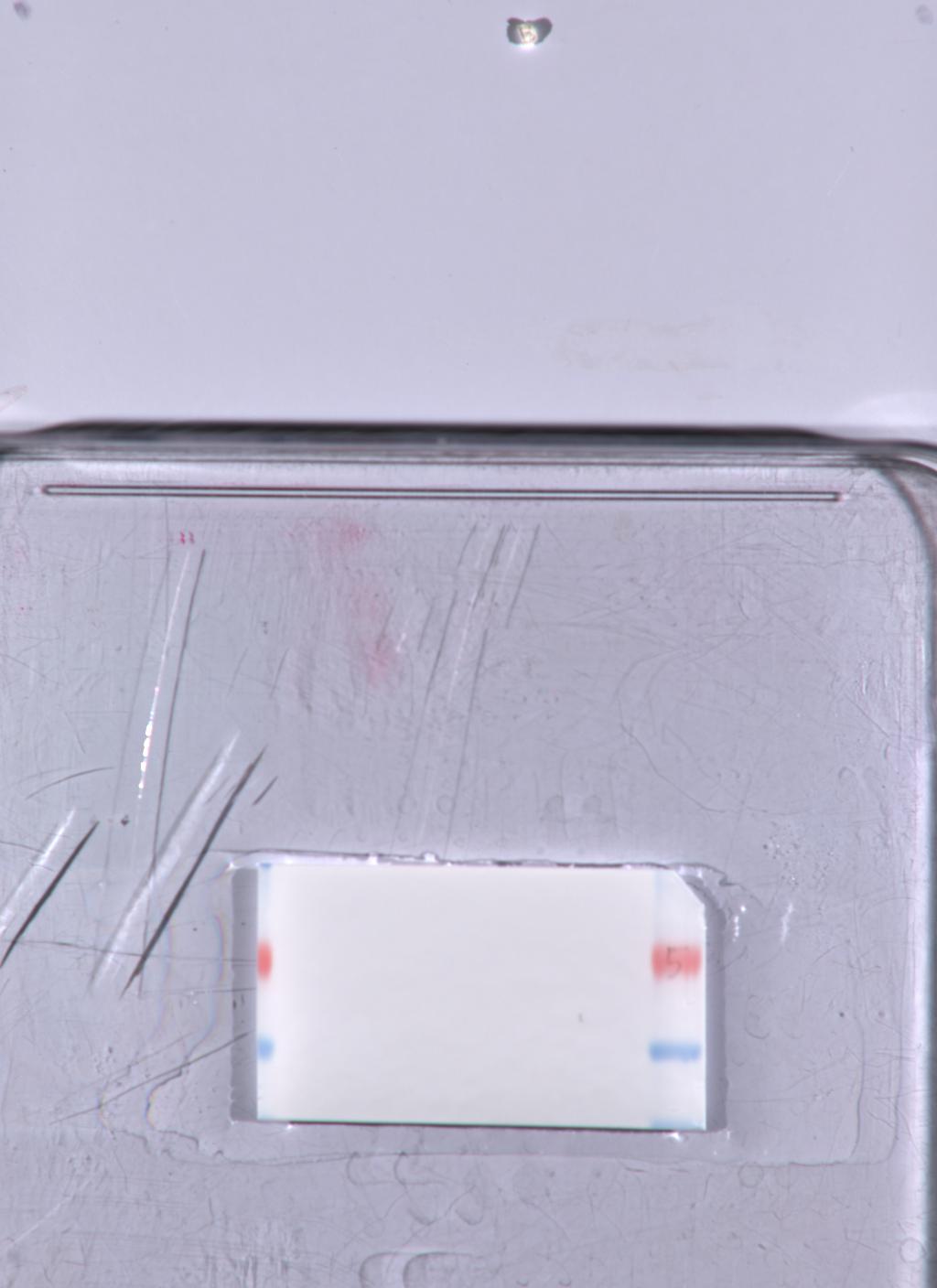

Supplement: Supplementary file 11 — Source data Fig. 6 [file 44321_2024_60_MOESM11_ESM.zip › Figure 6/6D/CN1/Western PLK1 9.9/5-2 3th PLK 9.9 2022.05.12_13.37.43_Ch-Marker.jpg]

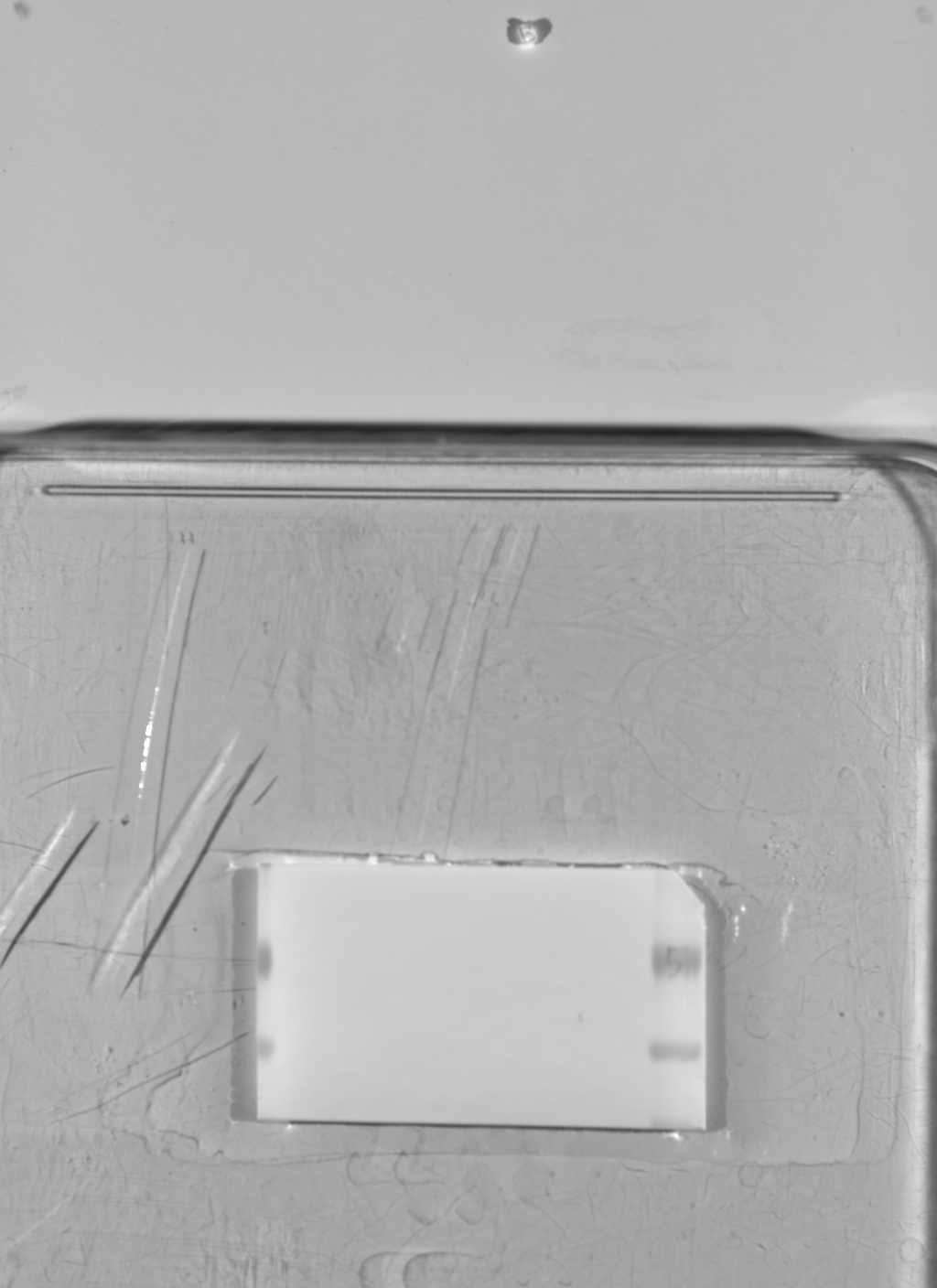

Supplement: Supplementary file 11 — Source data Fig. 6 [file 44321_2024_60_MOESM11_ESM.zip › Figure 6/6D/CN1/Western PLK1 9.9/5-2 3th PLK 9.9 2022.05.12_13.37.43_Ch-Marker.tif]

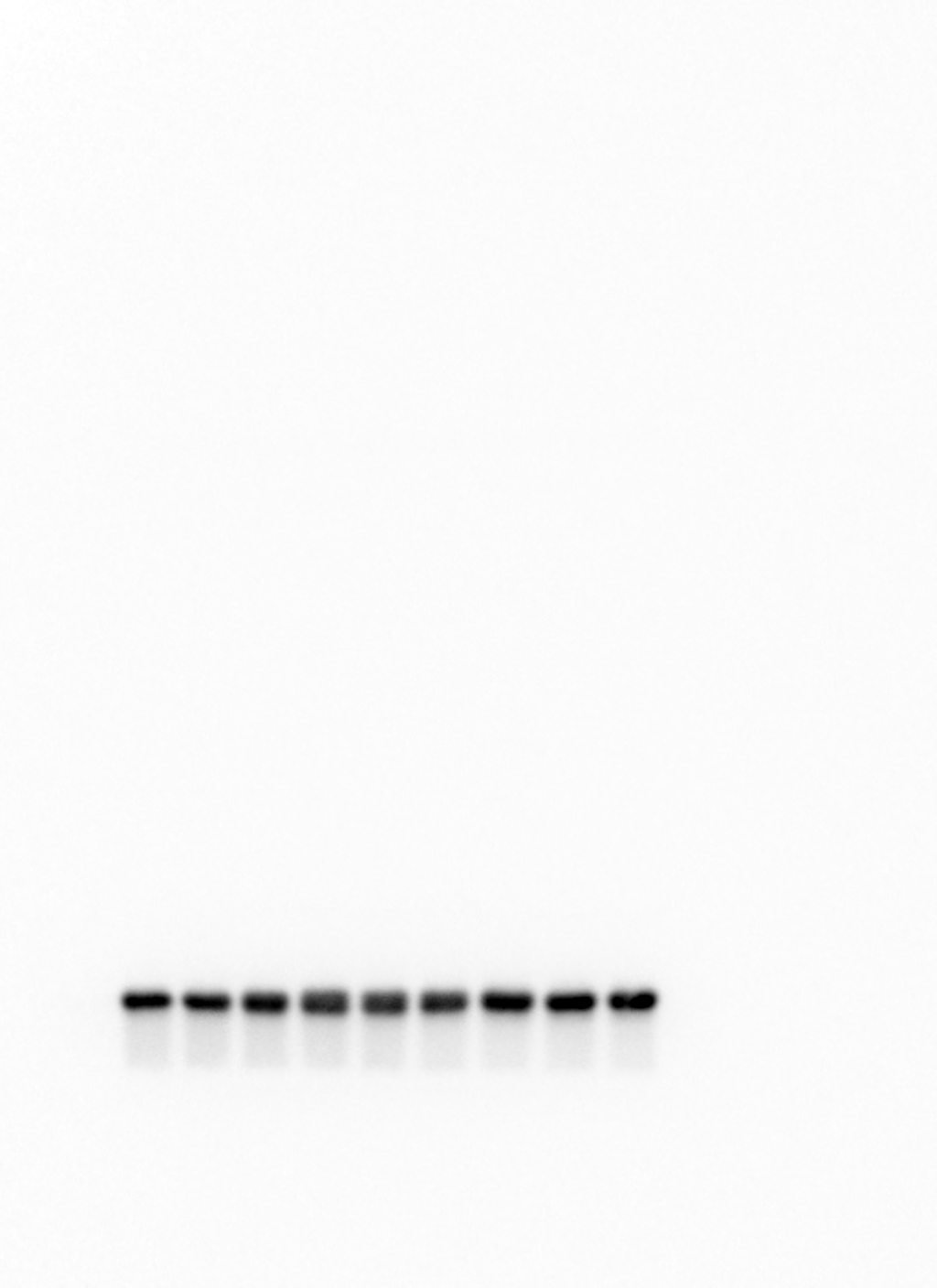

Supplement: Supplementary file 11 — Source data Fig. 6 [file 44321_2024_60_MOESM11_ESM.zip › Figure 6/6E/88T/Western GAPDH 0.4/4 1st GAP 0.4 _Ch.tif]

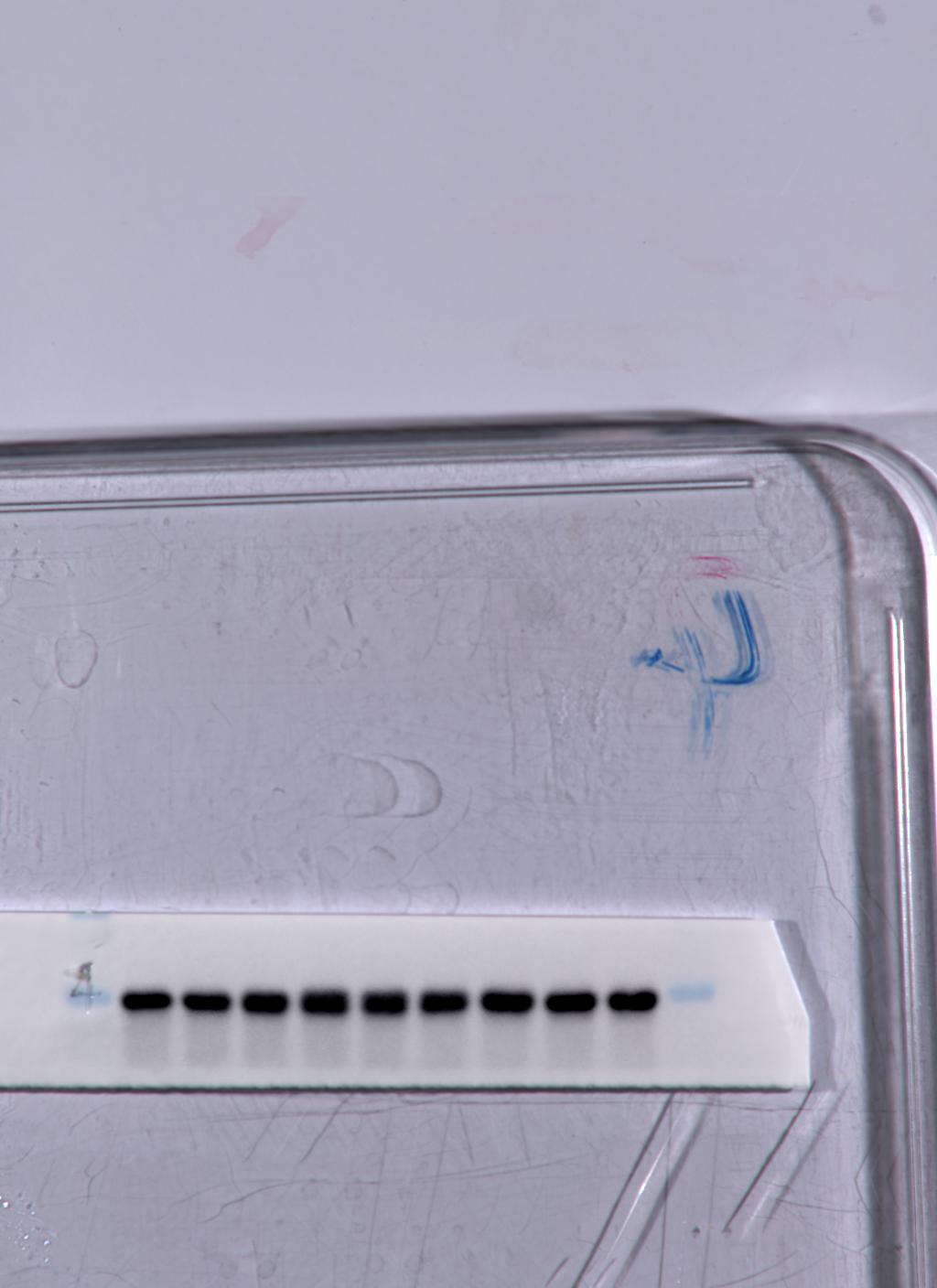

Supplement: Supplementary file 11 — Source data Fig. 6 [file 44321_2024_60_MOESM11_ESM.zip › Figure 6/6E/88T/Western GAPDH 0.4/4 1st GAP 0.4 _Ch+Marker.jpg]

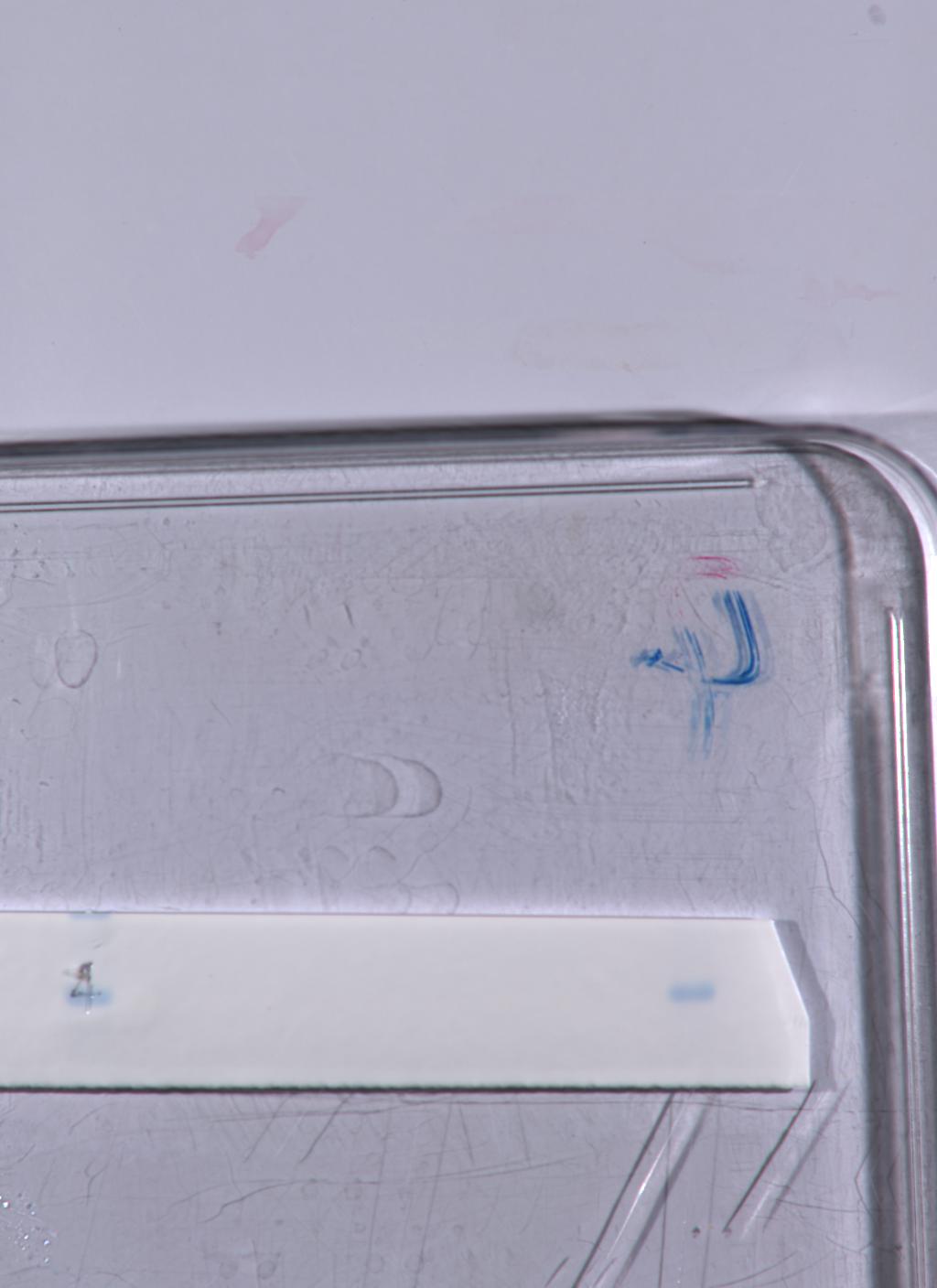

Supplement: Supplementary file 11 — Source data Fig. 6 [file 44321_2024_60_MOESM11_ESM.zip › Figure 6/6E/88T/Western GAPDH 0.4/4 1st GAP 0.4 _Ch-Marker.jpg]

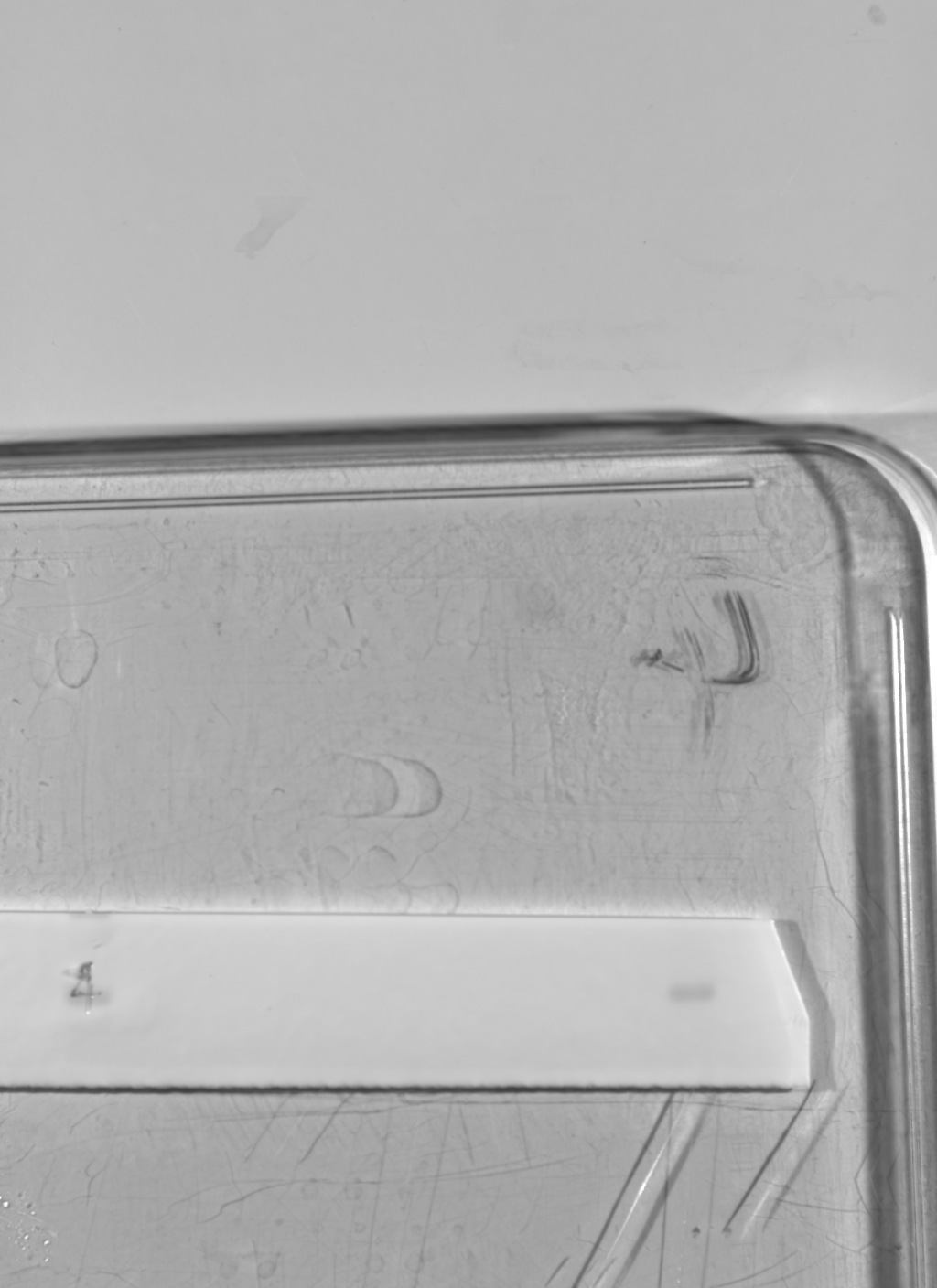

Supplement: Supplementary file 11 — Source data Fig. 6 [file 44321_2024_60_MOESM11_ESM.zip › Figure 6/6E/88T/Western GAPDH 0.4/4 1st GAP 0.4 _Ch-Marker.tif]

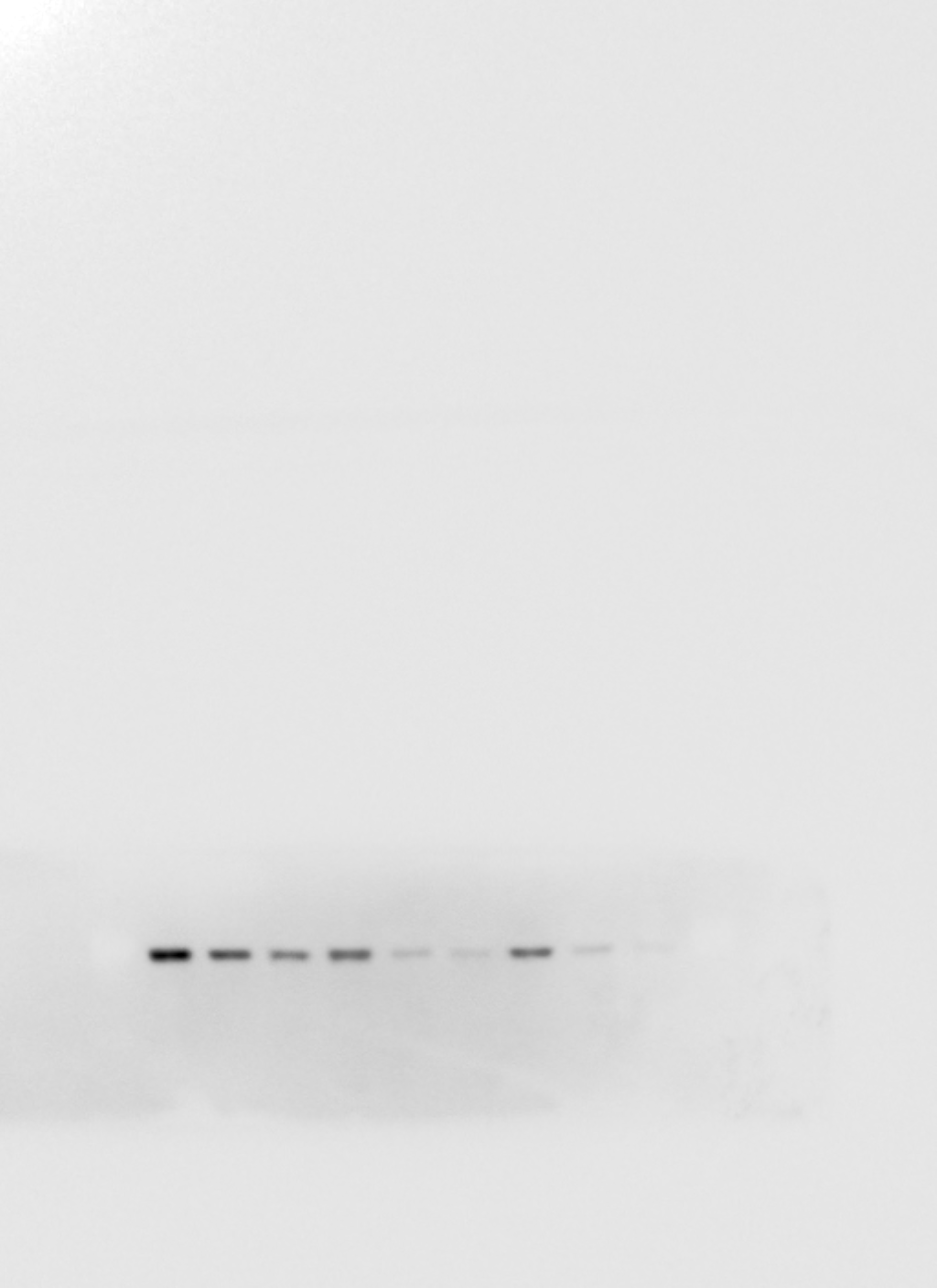

Supplement: Supplementary file 11 — Source data Fig. 6 [file 44321_2024_60_MOESM11_ESM.zip › Figure 6/6E/88T/Western PLK1 5.5/4 4th PLK 5.5 _Ch.tif]

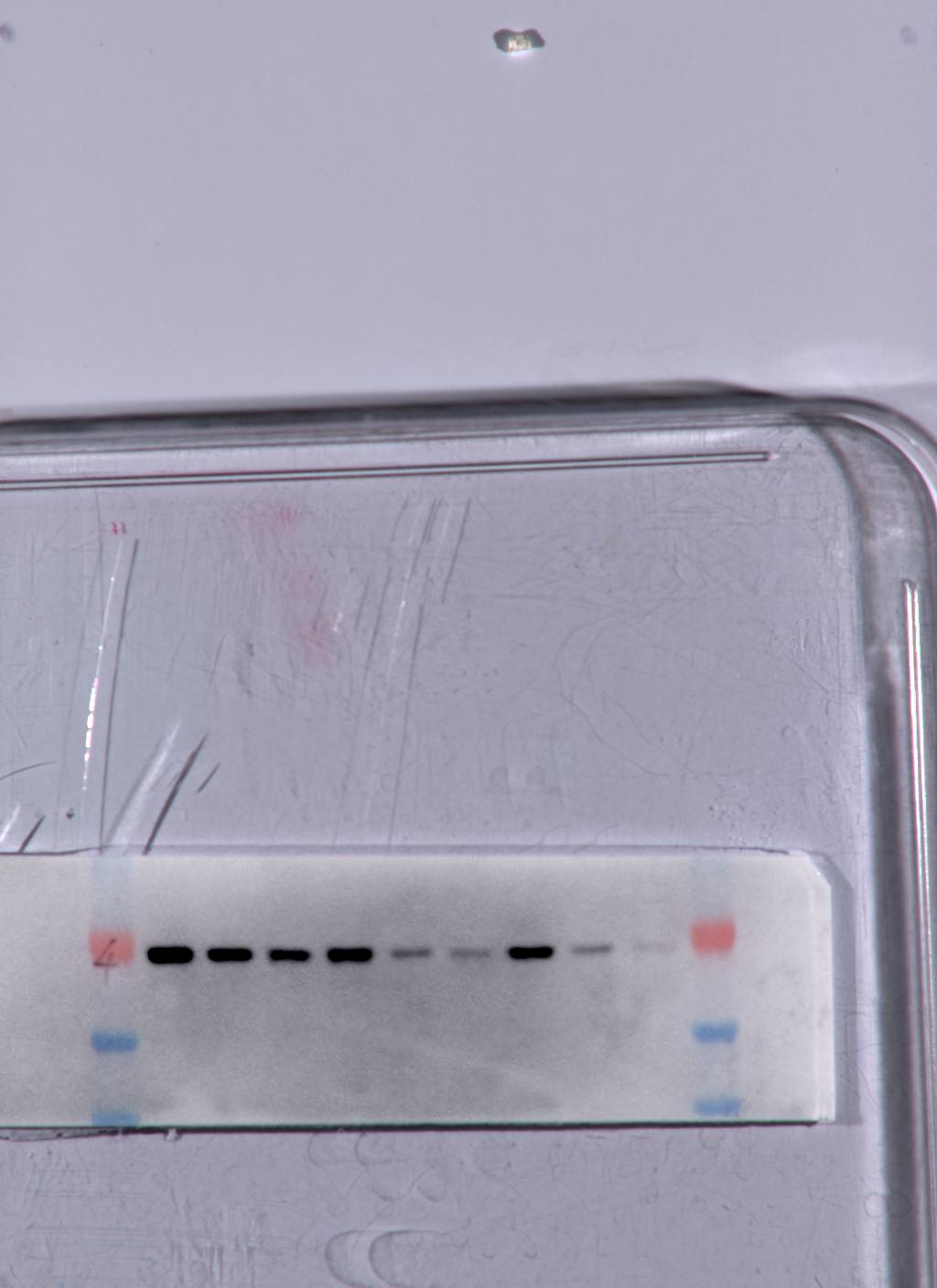

Supplement: Supplementary file 11 — Source data Fig. 6 [file 44321_2024_60_MOESM11_ESM.zip › Figure 6/6E/88T/Western PLK1 5.5/4 4th PLK 5.5 _Ch+Marker.jpg]

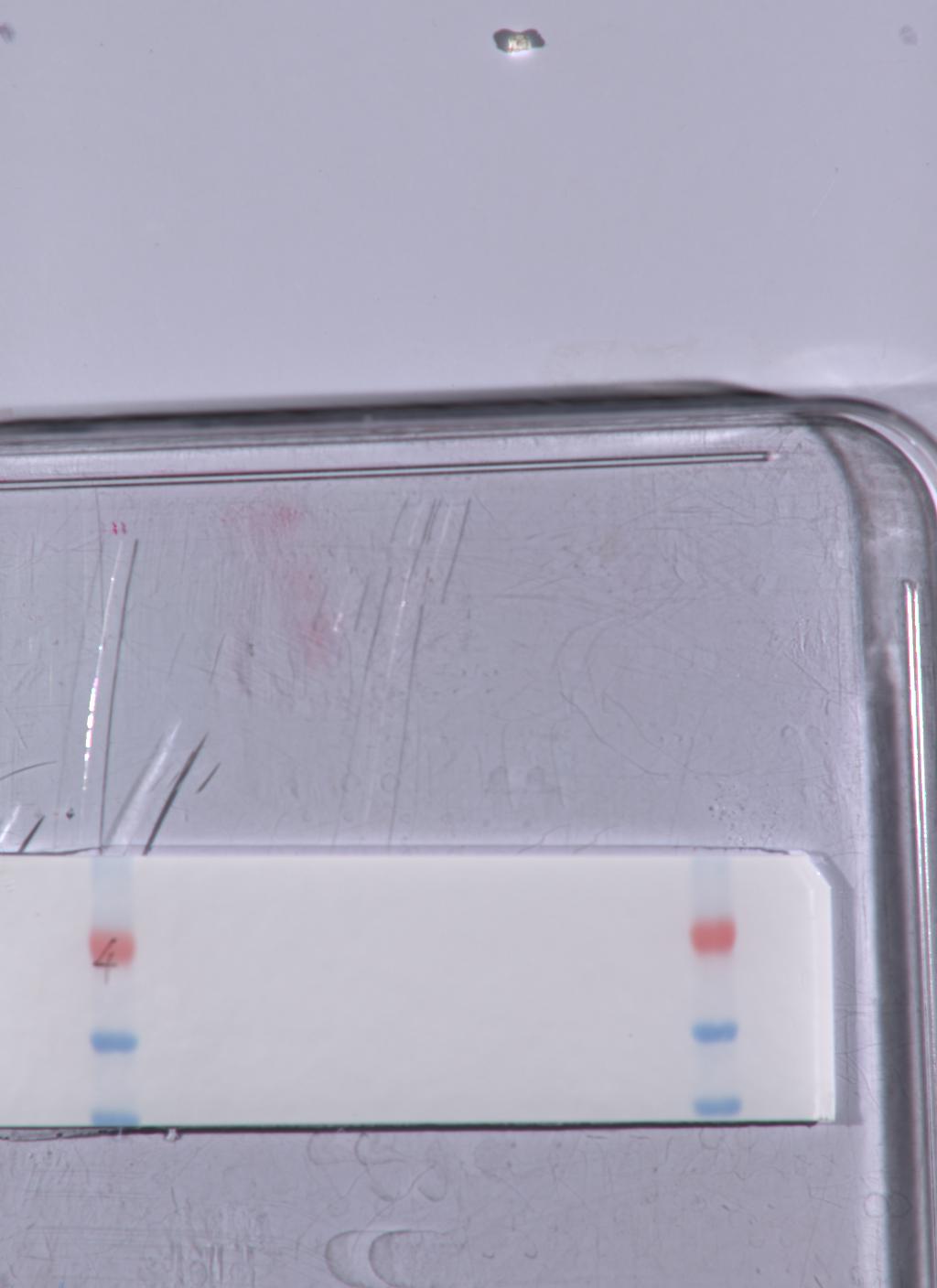

Supplement: Supplementary file 11 — Source data Fig. 6 [file 44321_2024_60_MOESM11_ESM.zip › Figure 6/6E/88T/Western PLK1 5.5/4 4th PLK 5.5 _Ch-Marker.jpg]

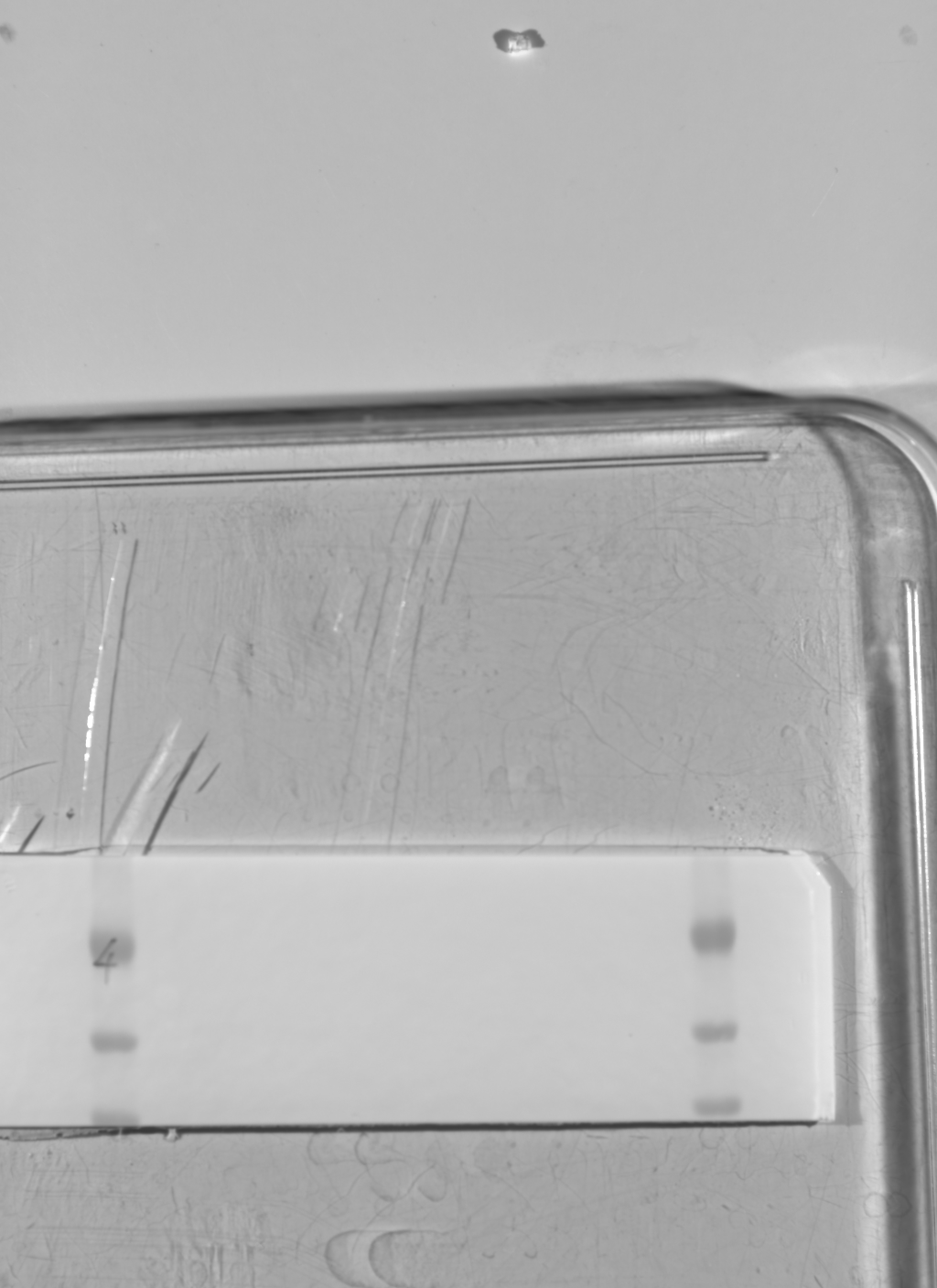

Supplement: Supplementary file 11 — Source data Fig. 6 [file 44321_2024_60_MOESM11_ESM.zip › Figure 6/6E/88T/Western PLK1 5.5/4 4th PLK 5.5 _Ch-Marker.tif]

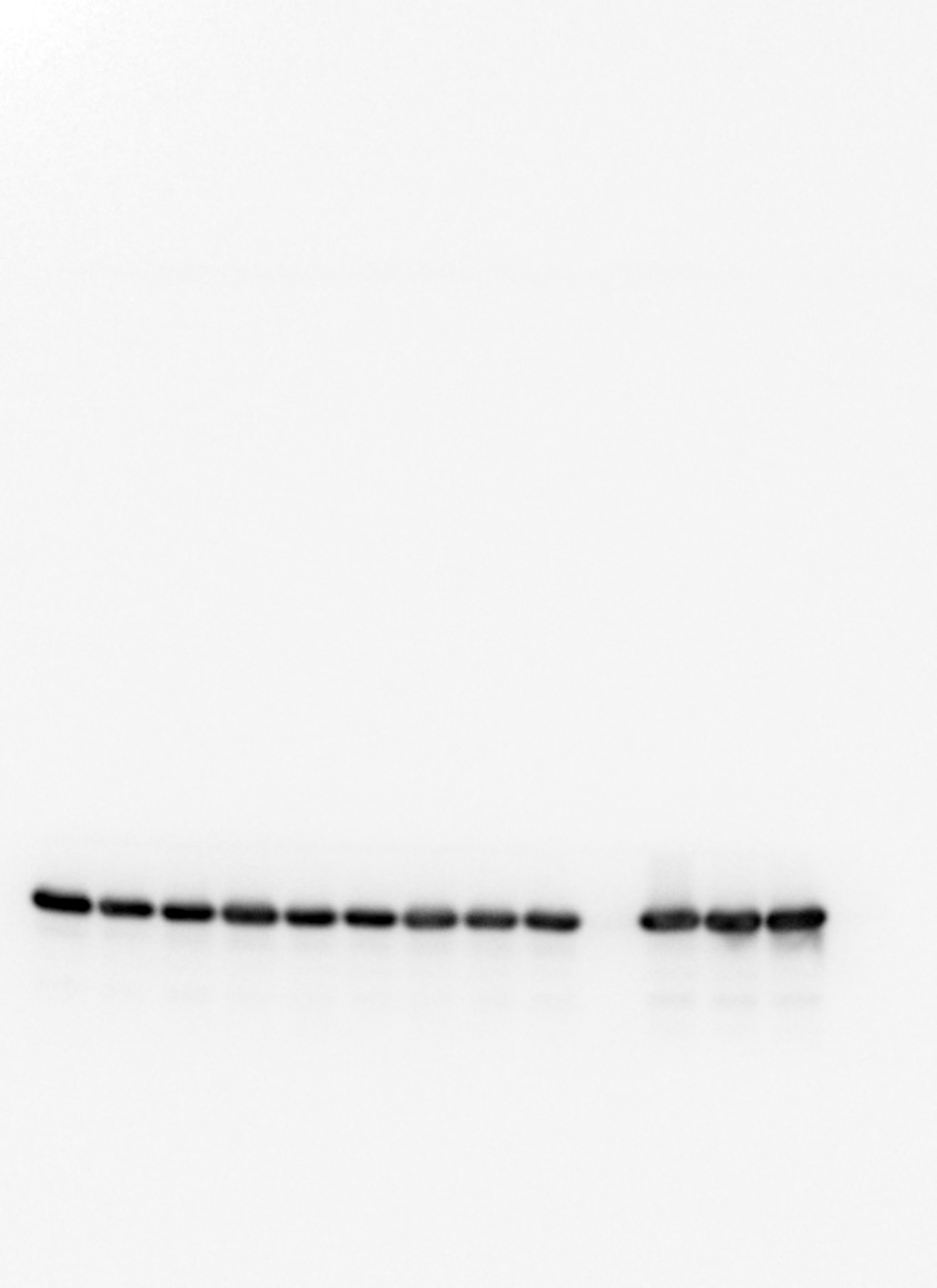

Supplement: Supplementary file 11 — Source data Fig. 6 [file 44321_2024_60_MOESM11_ESM.zip › Figure 6/6E/CN1/Western GAPDH 1.6/4 GAP 1.6 _Ch.tif]

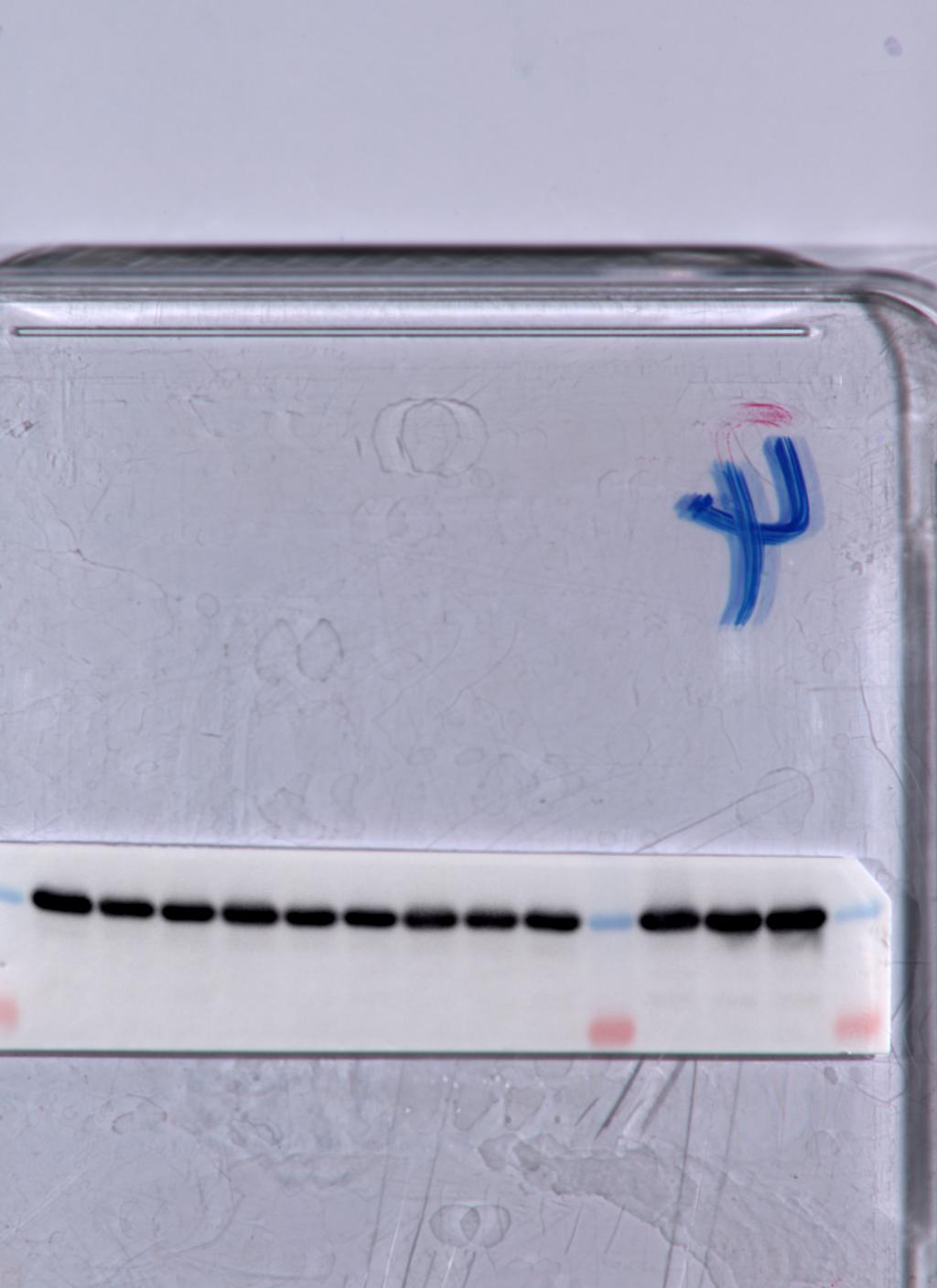

Supplement: Supplementary file 11 — Source data Fig. 6 [file 44321_2024_60_MOESM11_ESM.zip › Figure 6/6E/CN1/Western GAPDH 1.6/4 GAP 1.6 _Ch+Marker.jpg]

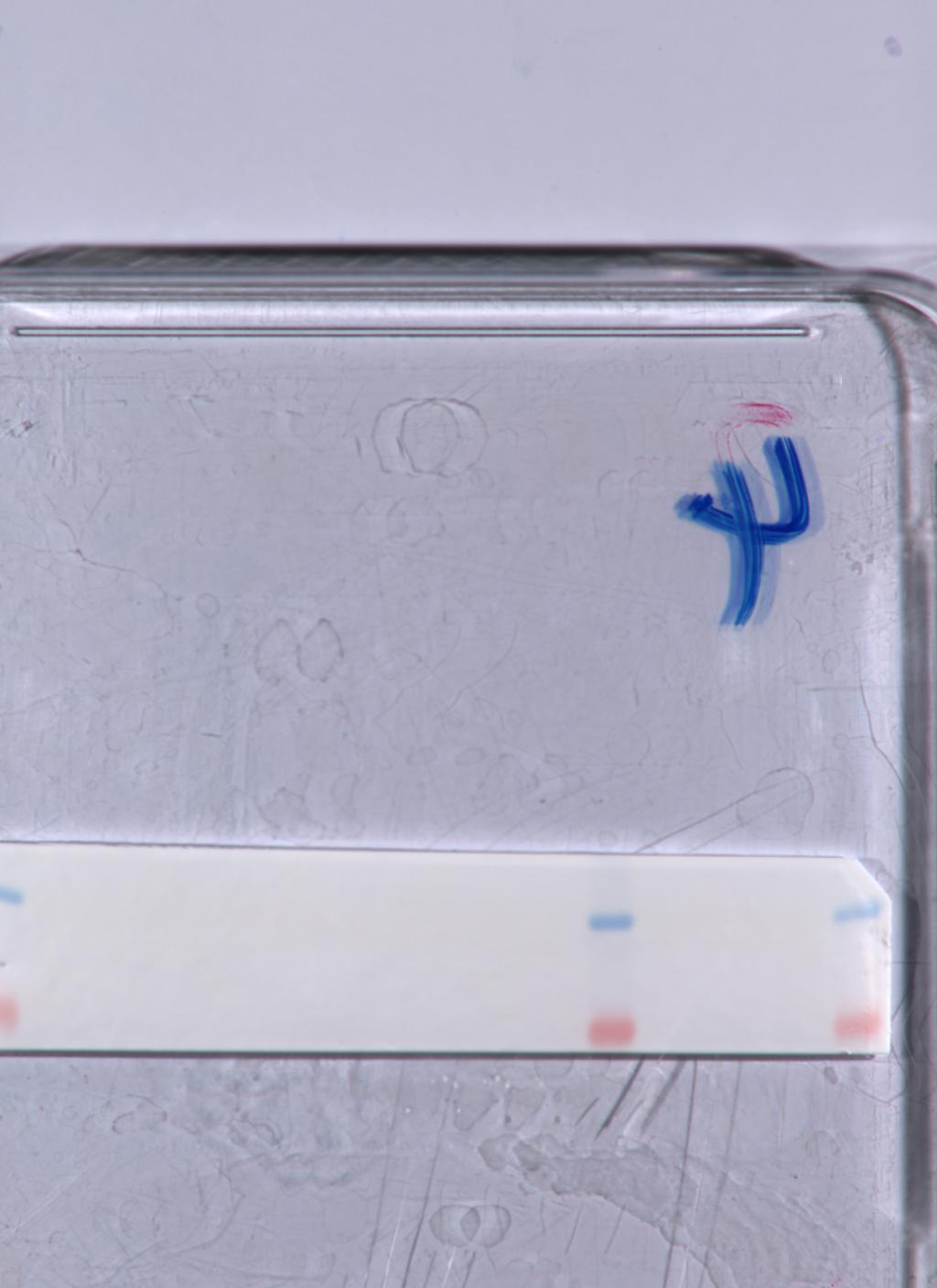

Supplement: Supplementary file 11 — Source data Fig. 6 [file 44321_2024_60_MOESM11_ESM.zip › Figure 6/6E/CN1/Western GAPDH 1.6/4 GAP 1.6 _Ch-Marker.jpg]

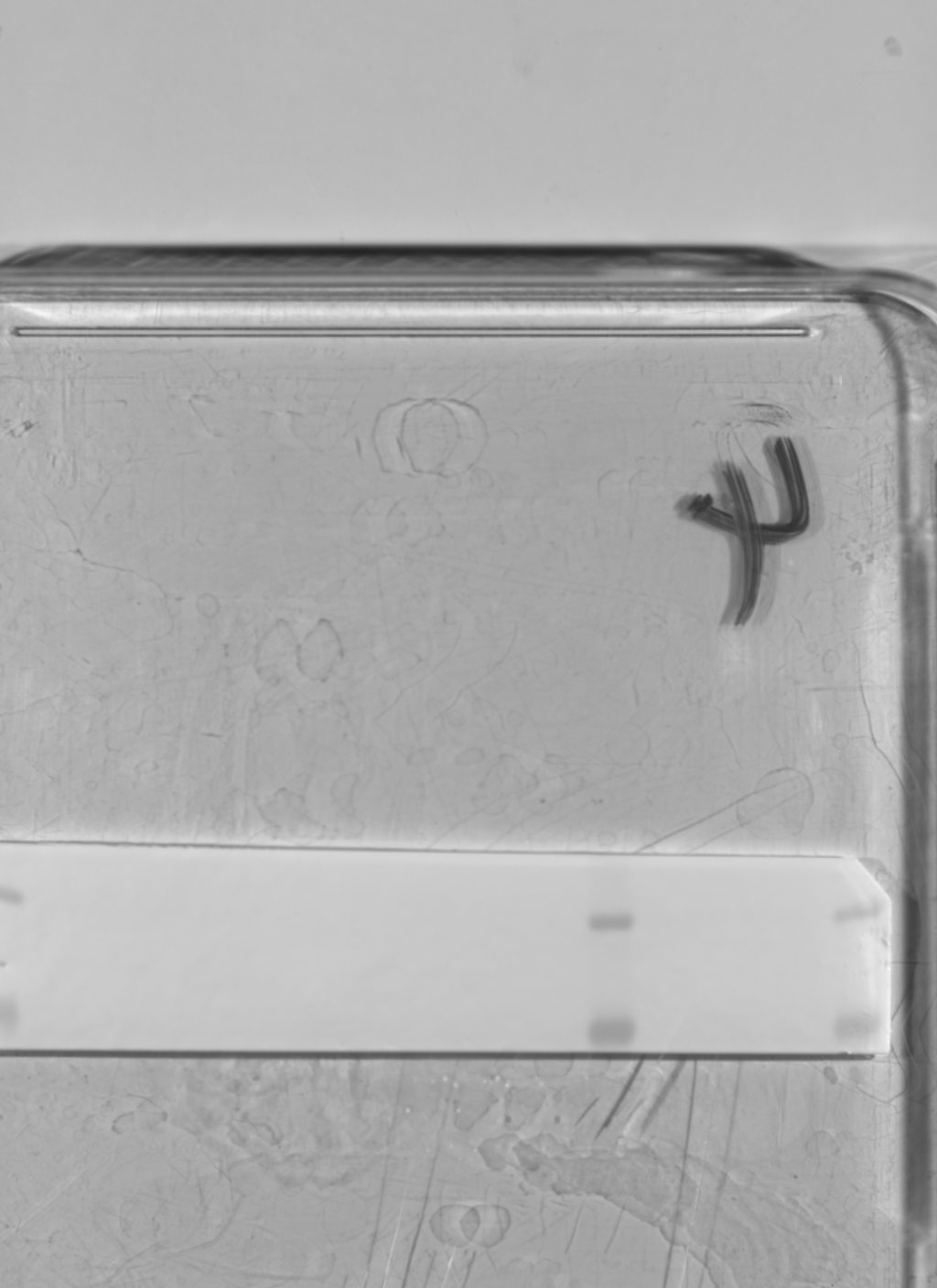

Supplement: Supplementary file 11 — Source data Fig. 6 [file 44321_2024_60_MOESM11_ESM.zip › Figure 6/6E/CN1/Western GAPDH 1.6/4 GAP 1.6 _Ch-Marker.tif]

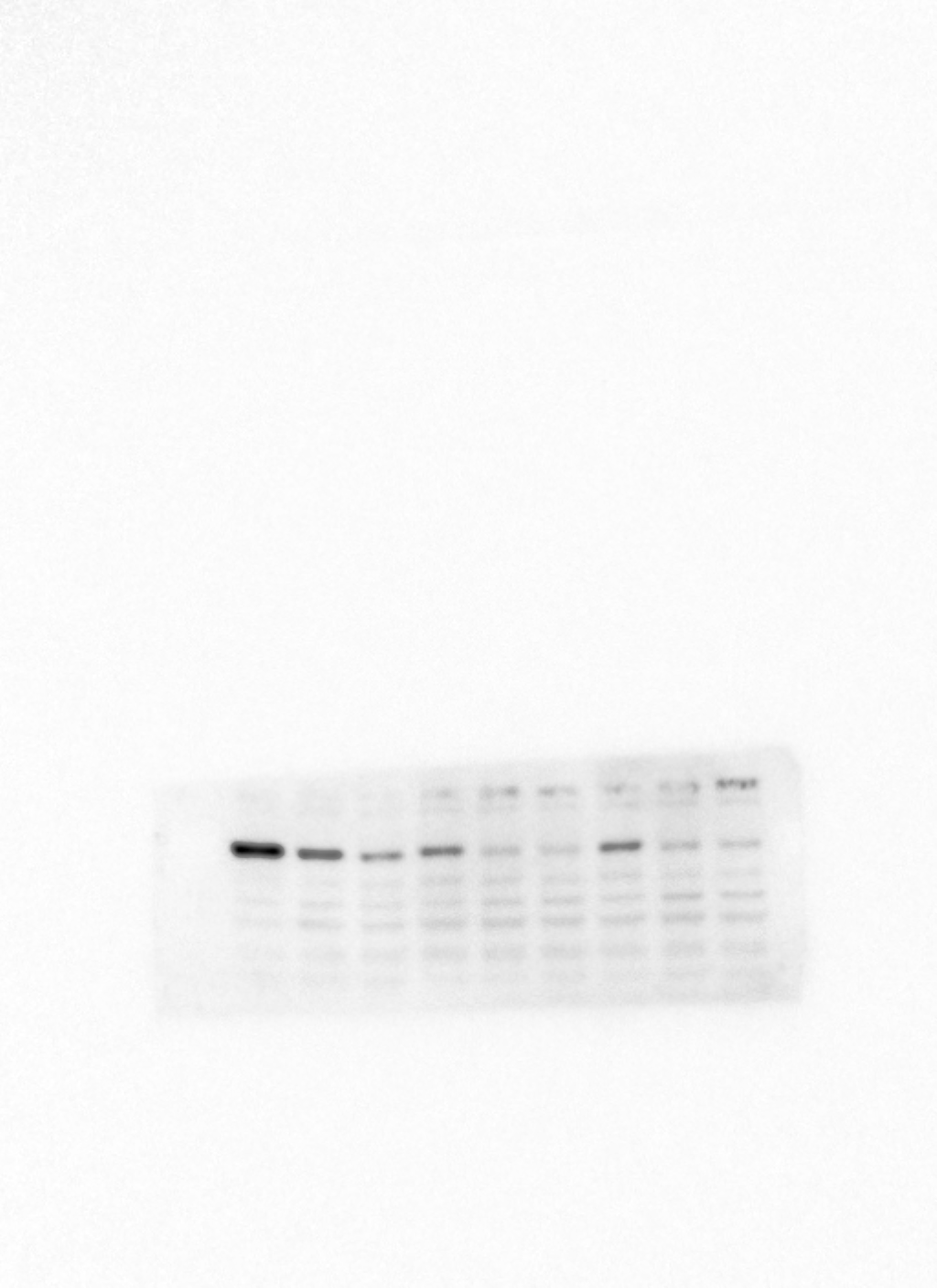

Supplement: Supplementary file 11 — Source data Fig. 6 [file 44321_2024_60_MOESM11_ESM.zip › Figure 6/6E/CN1/Western PLK1 12.3/4 2nd PLK 12.3 _Ch.tif]

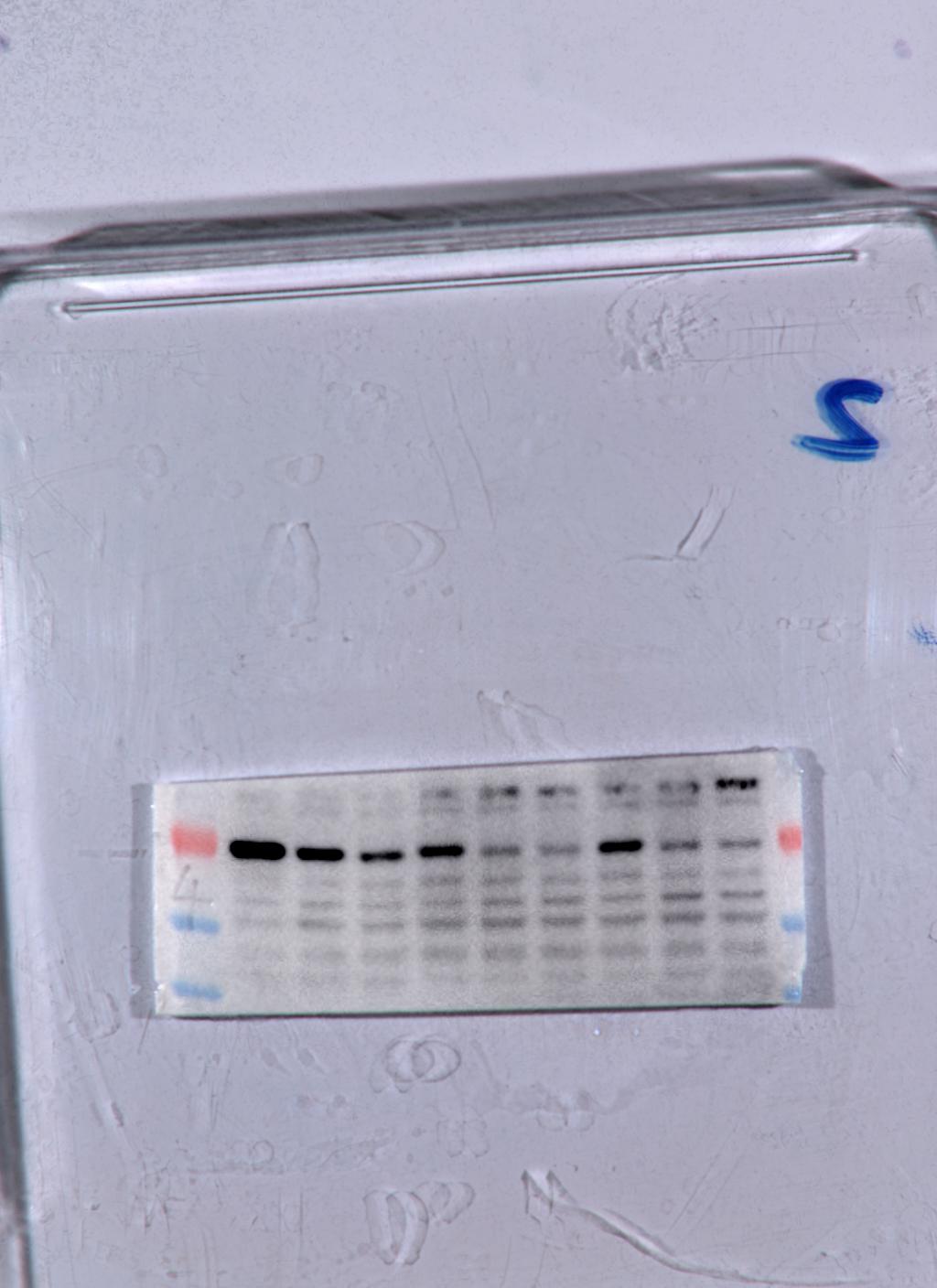

Supplement: Supplementary file 11 — Source data Fig. 6 [file 44321_2024_60_MOESM11_ESM.zip › Figure 6/6E/CN1/Western PLK1 12.3/4 2nd PLK 12.3 _Ch+Marker.jpg]

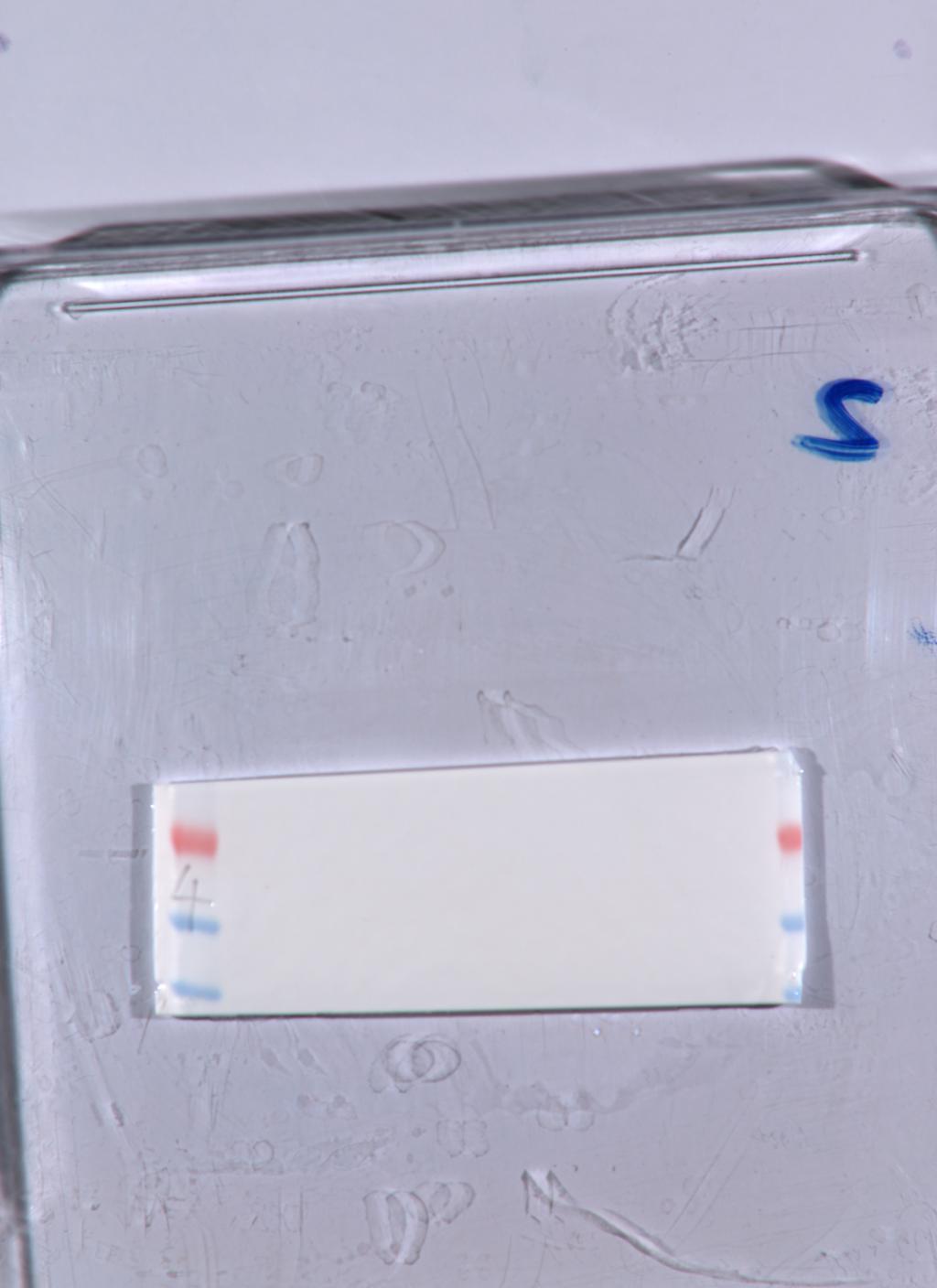

Supplement: Supplementary file 11 — Source data Fig. 6 [file 44321_2024_60_MOESM11_ESM.zip › Figure 6/6E/CN1/Western PLK1 12.3/4 2nd PLK 12.3 _Ch-Marker.jpg]

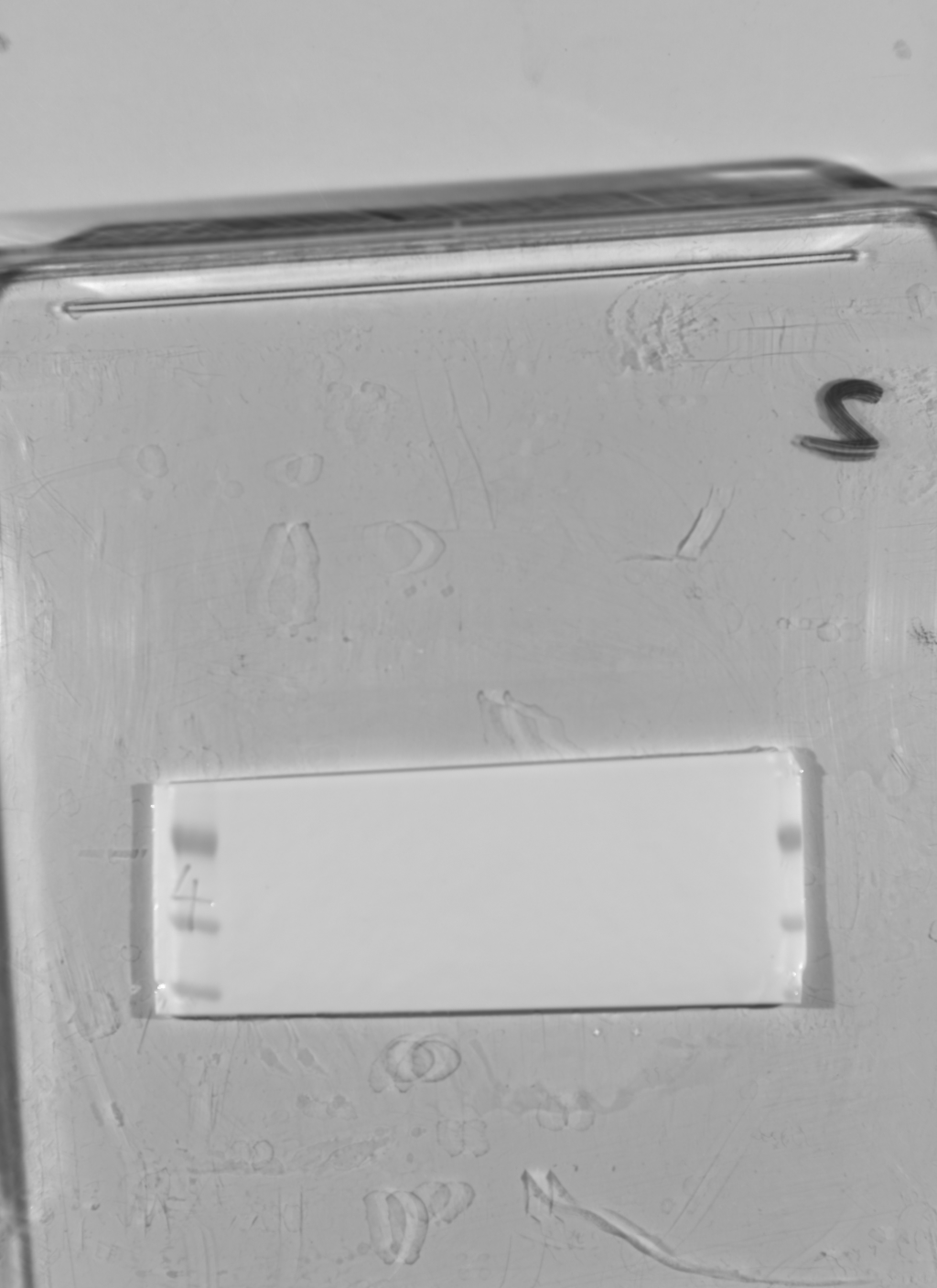

Supplement: Supplementary file 11 — Source data Fig. 6 [file 44321_2024_60_MOESM11_ESM.zip › Figure 6/6E/CN1/Western PLK1 12.3/4 2nd PLK 12.3 _Ch-Marker.tif]

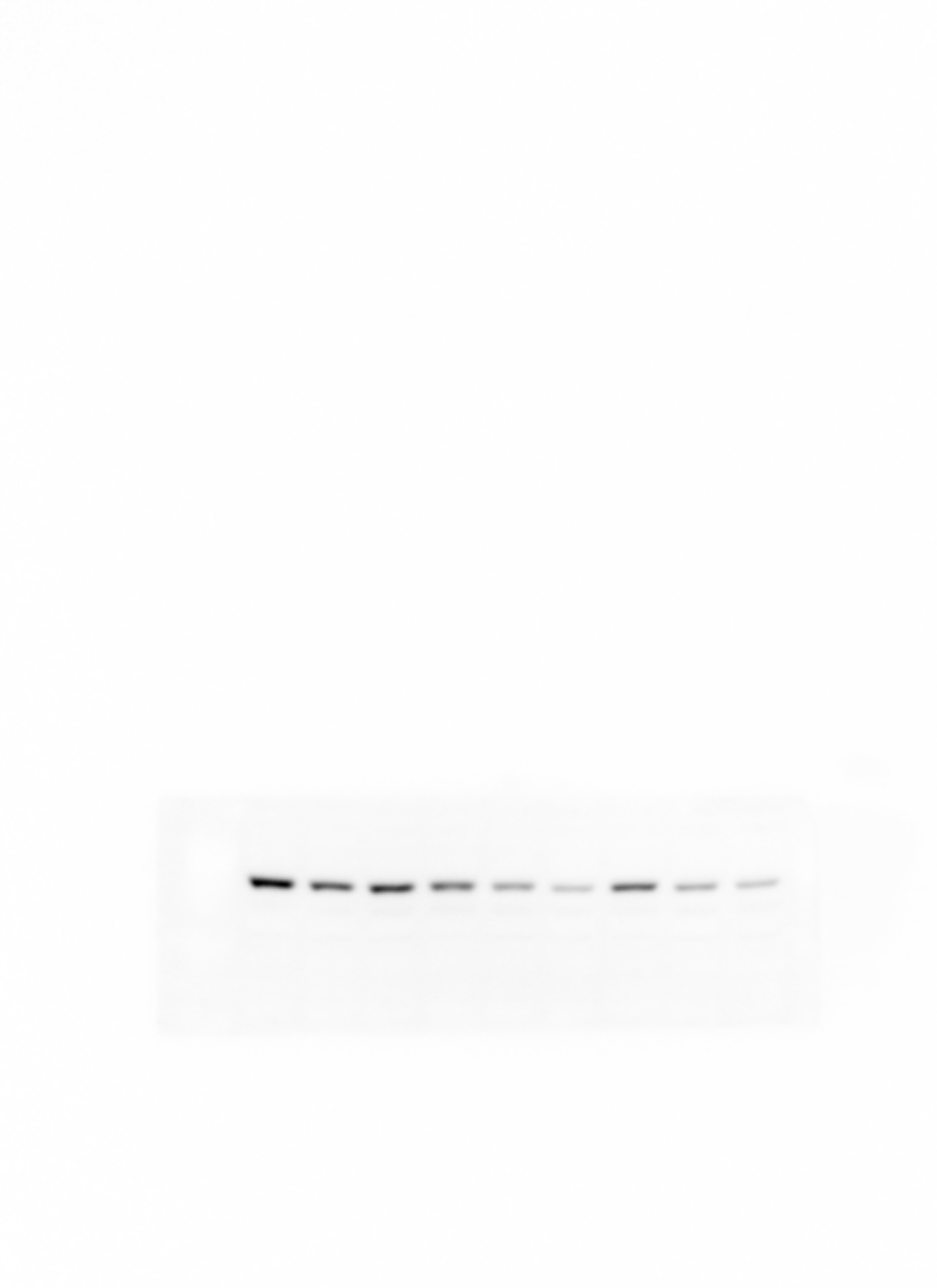

Supplement: Supplementary file 11 — Source data Fig. 6 [file 44321_2024_60_MOESM11_ESM.zip › Figure 6/6E/YAPC/Western PLK1 31.9/3-1 1st PLK 31.9 _Ch.tif]

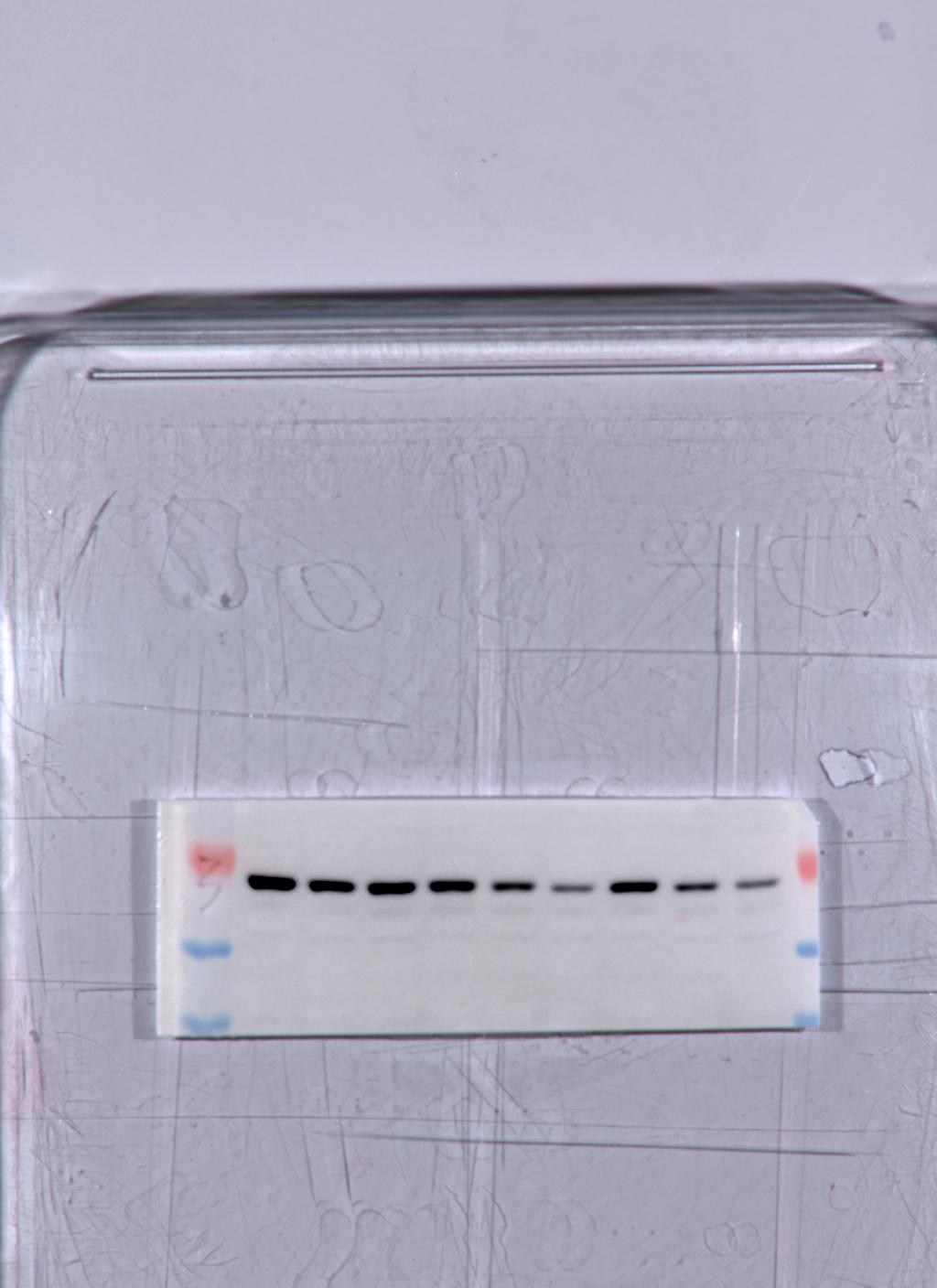

Supplement: Supplementary file 11 — Source data Fig. 6 [file 44321_2024_60_MOESM11_ESM.zip › Figure 6/6E/YAPC/Western PLK1 31.9/3-1 1st PLK 31.9 _Ch+Marker.jpg]

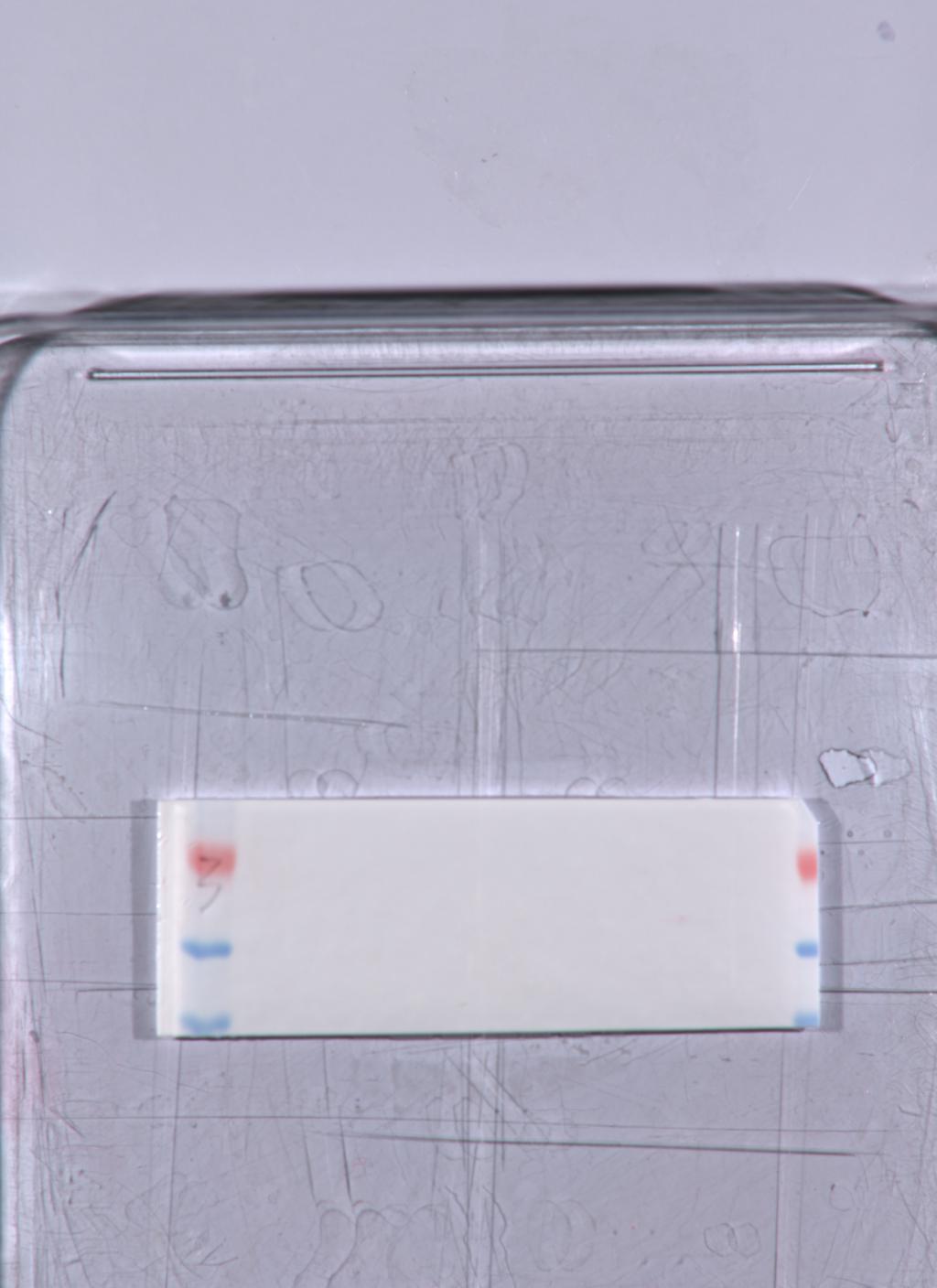

Supplement: Supplementary file 11 — Source data Fig. 6 [file 44321_2024_60_MOESM11_ESM.zip › Figure 6/6E/YAPC/Western PLK1 31.9/3-1 1st PLK 31.9 _Ch-Marker.jpg]

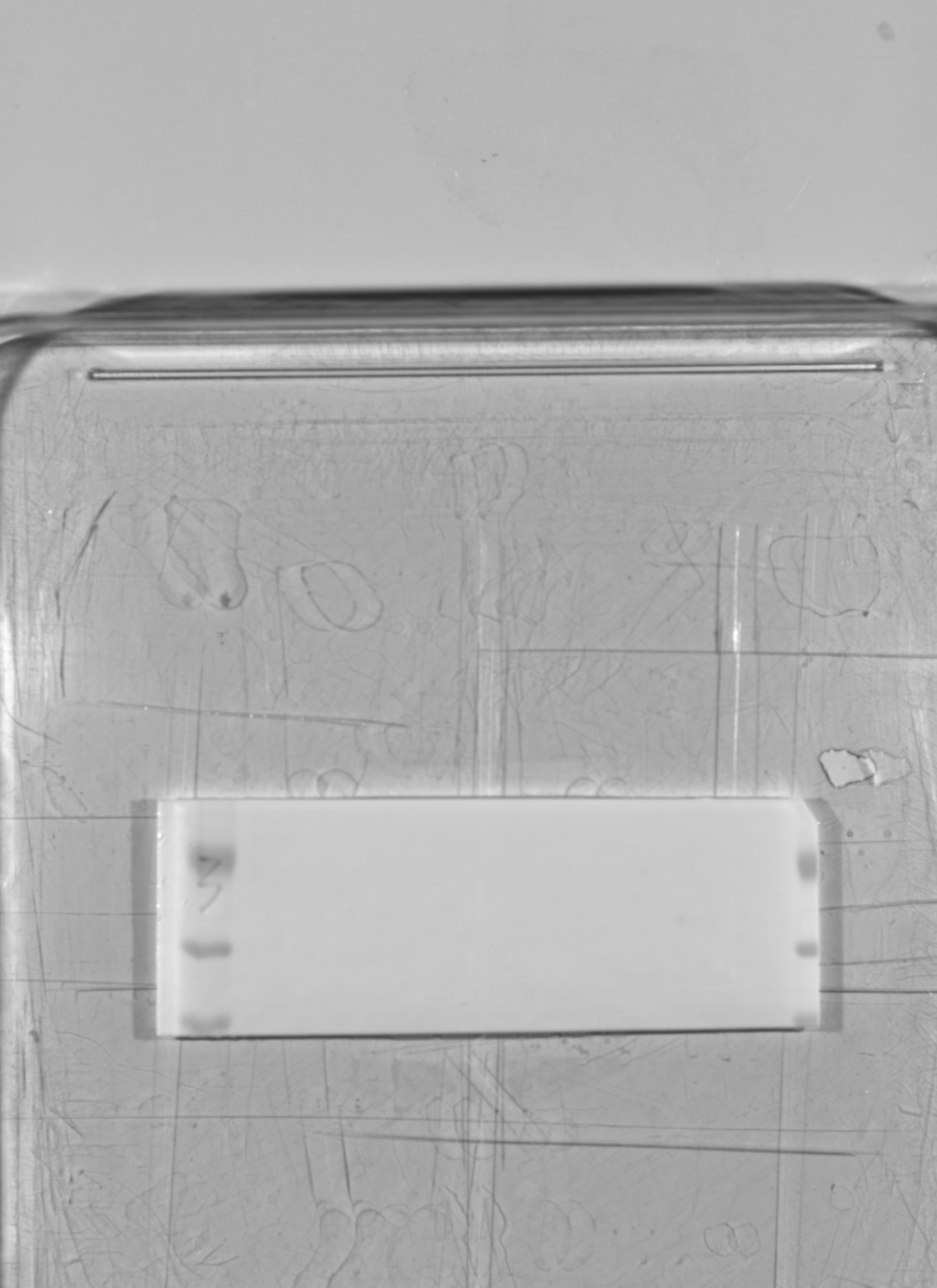

Supplement: Supplementary file 11 — Source data Fig. 6 [file 44321_2024_60_MOESM11_ESM.zip › Figure 6/6E/YAPC/Western PLK1 31.9/3-1 1st PLK 31.9 _Ch-Marker.tif]

## Slide 1
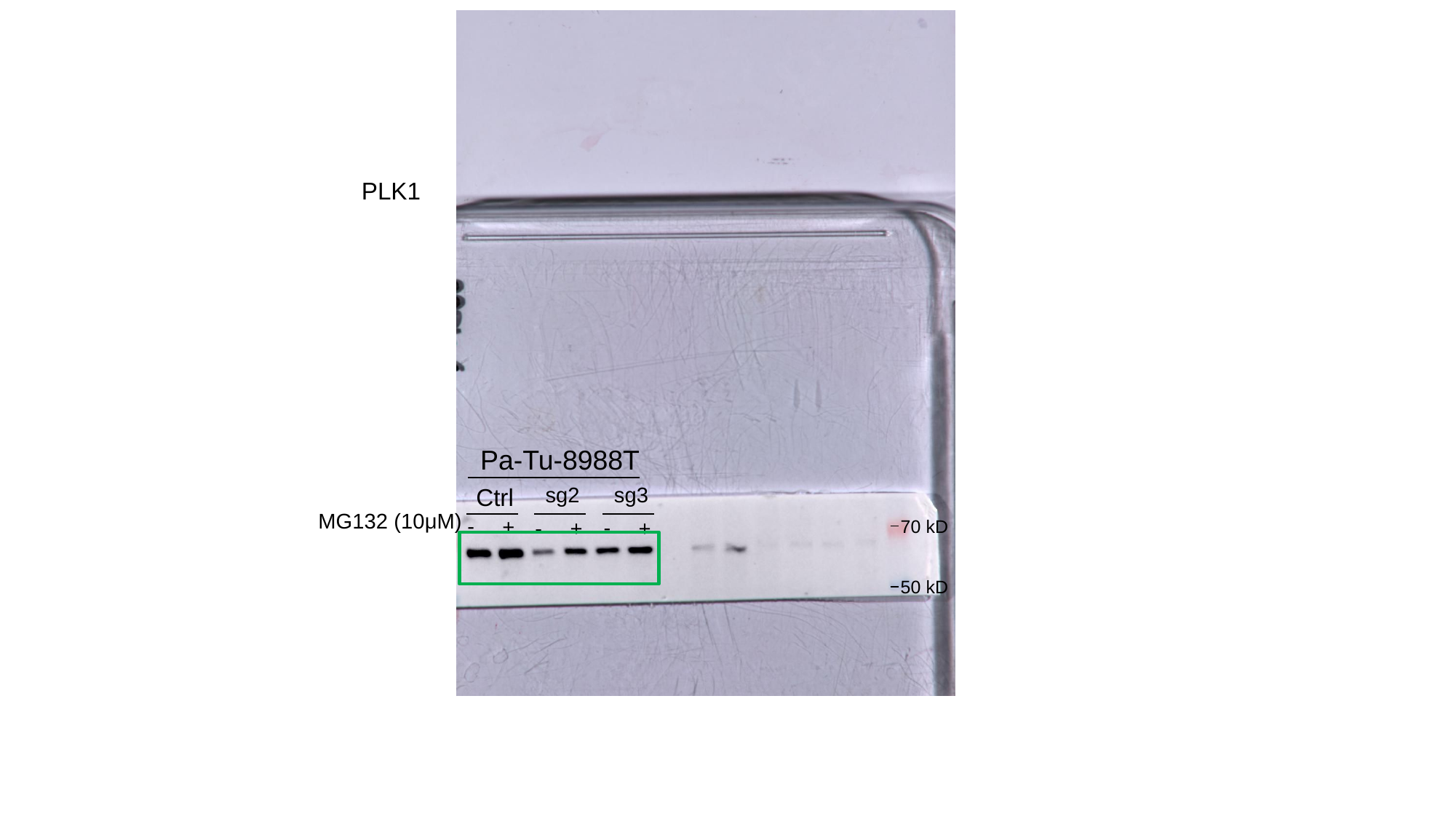

PLK1
Pa-Tu-8988T
Ctrl
sg2
sg3
MG132 (10μM)
-
+
-
-
70 kD
+
+
50 kD

## Slide 2
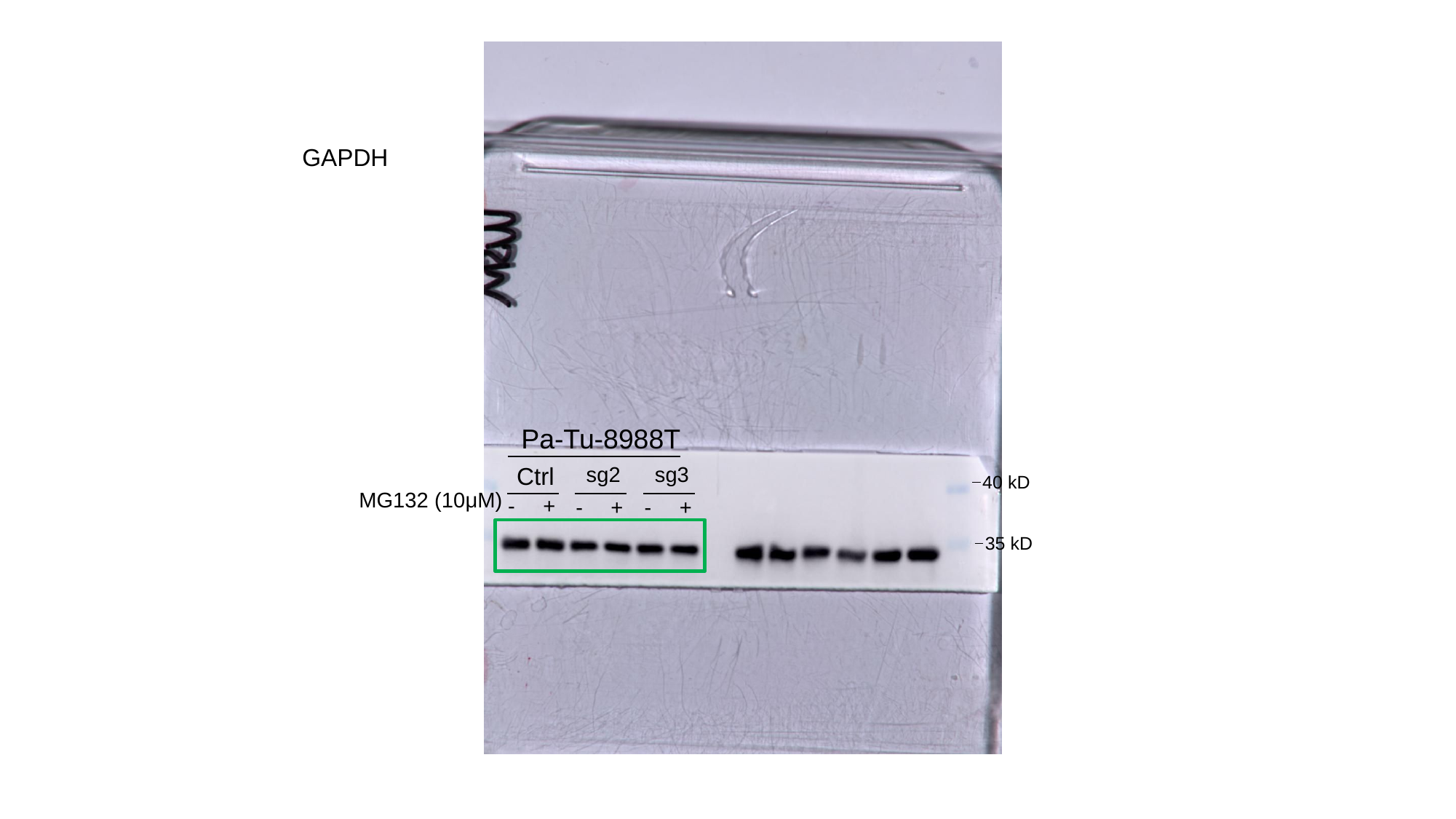

GAPDH
Pa-Tu-8988T
Ctrl
sg2
sg3
40 kD
MG132 (10μM)
-
+
-
-
+
+
35 kD

Supplement: Supplementary file 11 — Source data Fig. 6 [file 44321_2024_60_MOESM11_ESM.zip › Figure 6/6F/88T/6F 88T.pptx]

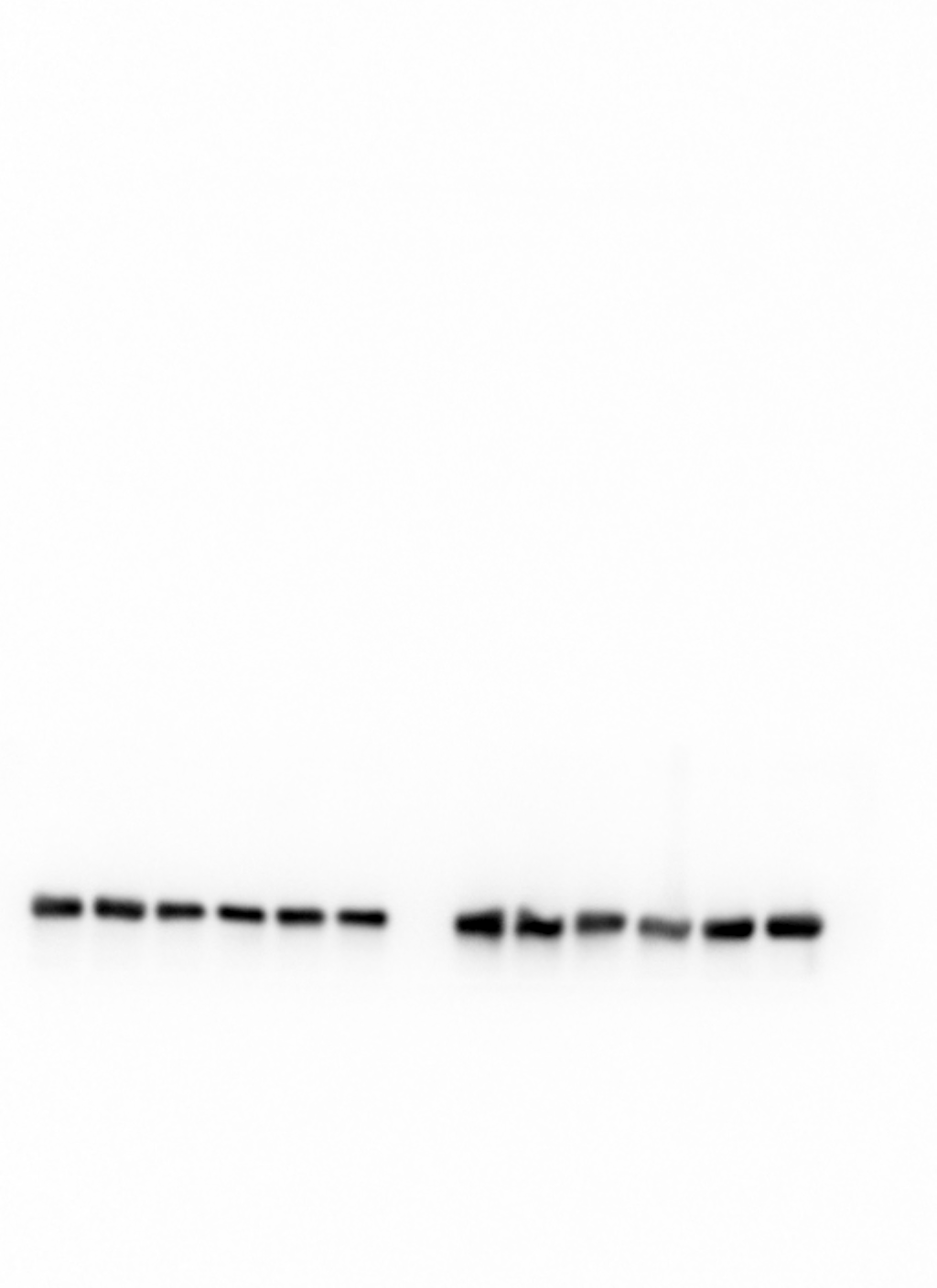

Supplement: Supplementary file 11 — Source data Fig. 6 [file 44321_2024_60_MOESM11_ESM.zip › Figure 6/6F/88T/Western GAPDH 0.1/6 GAP 0.1 _Ch.tif]

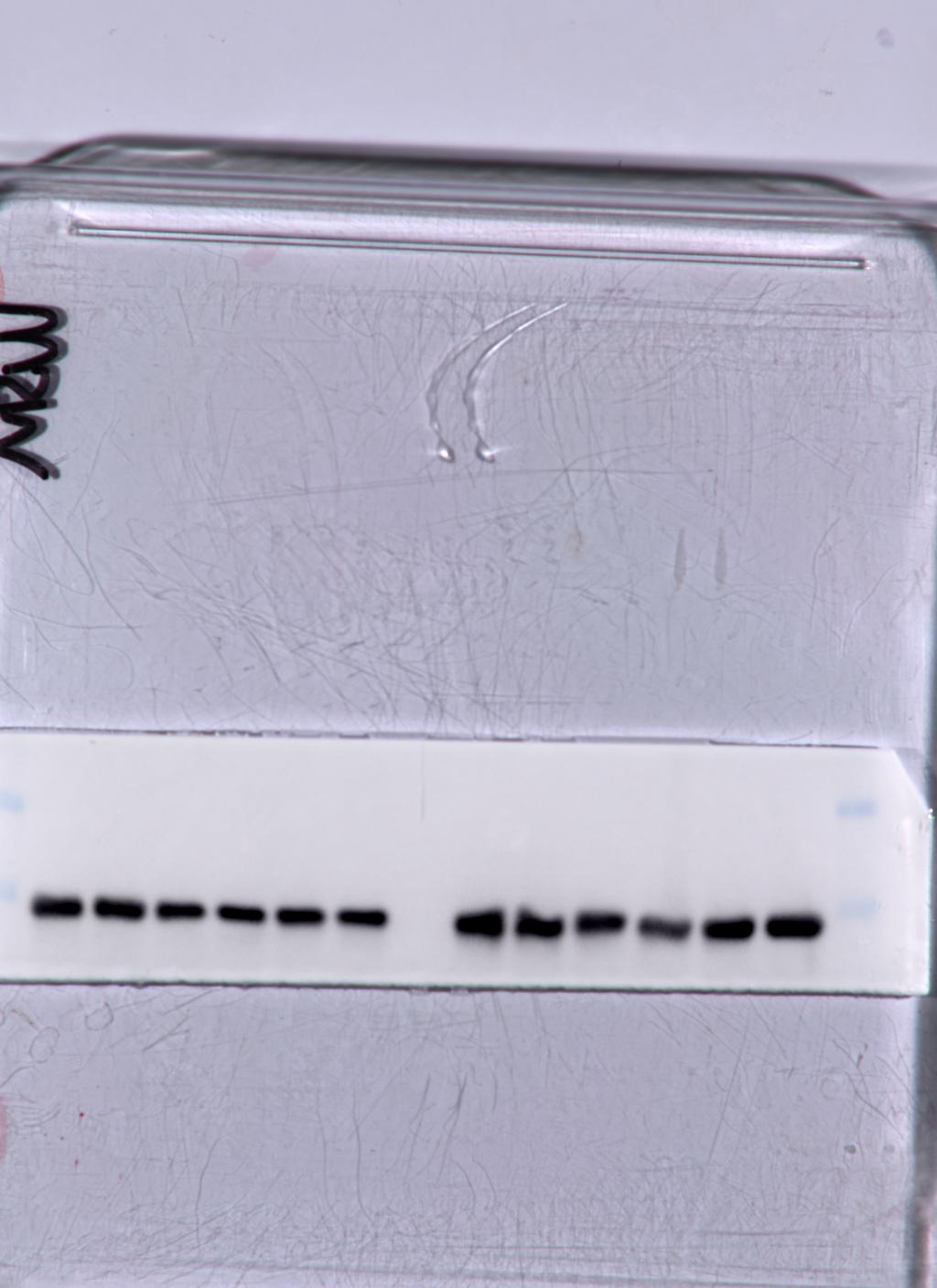

Supplement: Supplementary file 11 — Source data Fig. 6 [file 44321_2024_60_MOESM11_ESM.zip › Figure 6/6F/88T/Western GAPDH 0.1/6 GAP 0.1 _Ch+Marker.jpg]

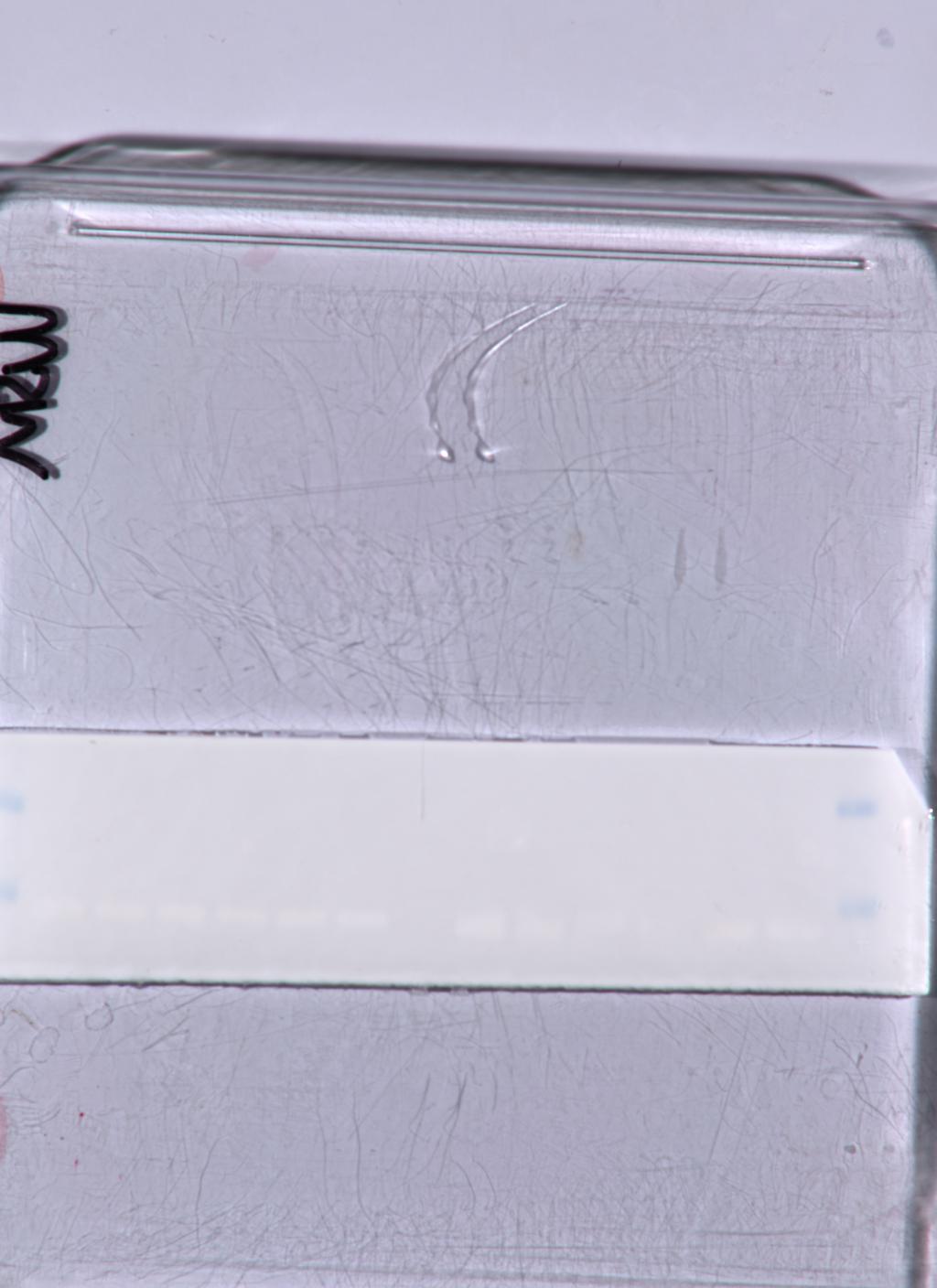

Supplement: Supplementary file 11 — Source data Fig. 6 [file 44321_2024_60_MOESM11_ESM.zip › Figure 6/6F/88T/Western GAPDH 0.1/6 GAP 0.1 _Ch-Marker.jpg]

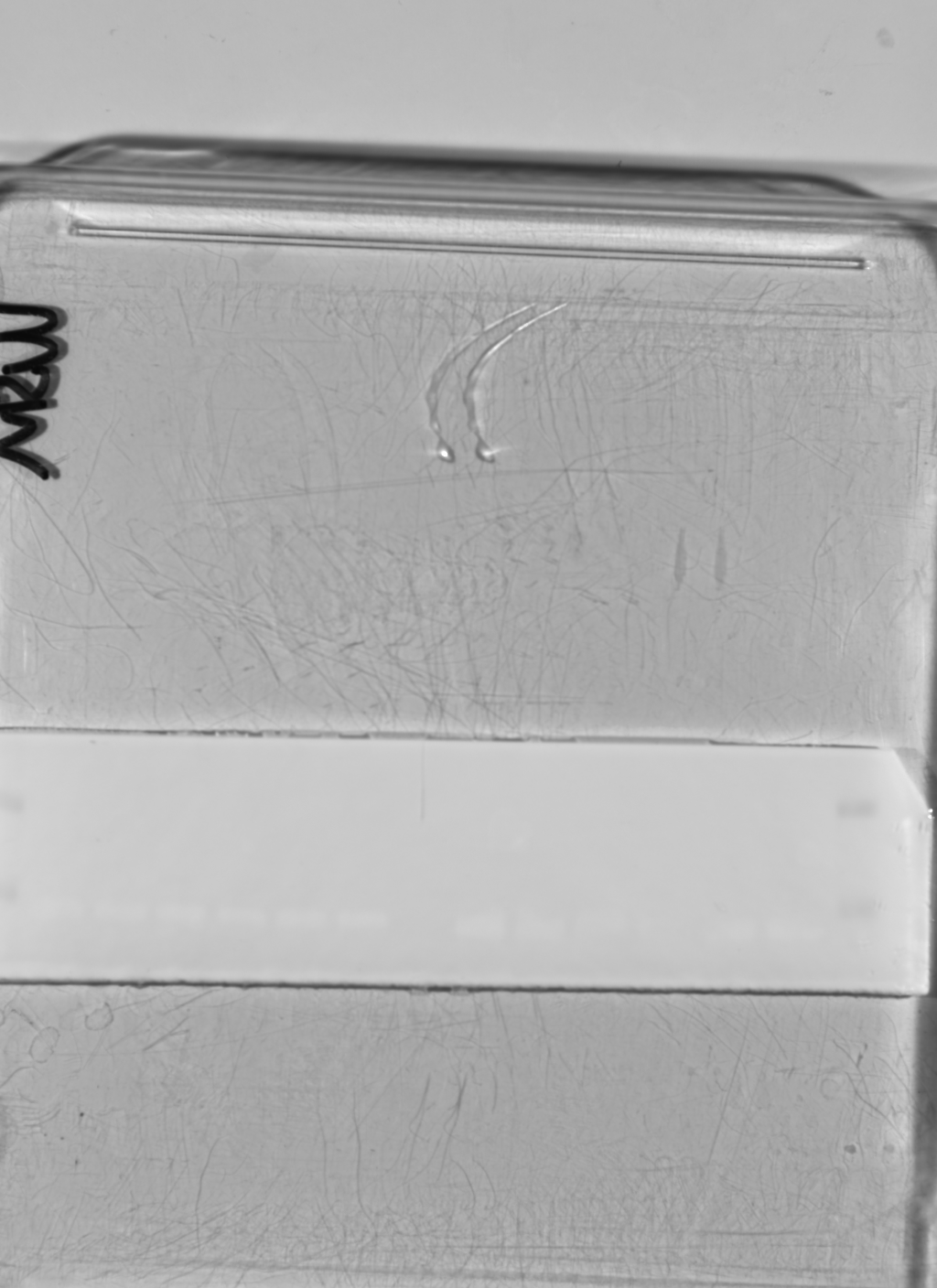

Supplement: Supplementary file 11 — Source data Fig. 6 [file 44321_2024_60_MOESM11_ESM.zip › Figure 6/6F/88T/Western GAPDH 0.1/6 GAP 0.1 _Ch-Marker.tif]

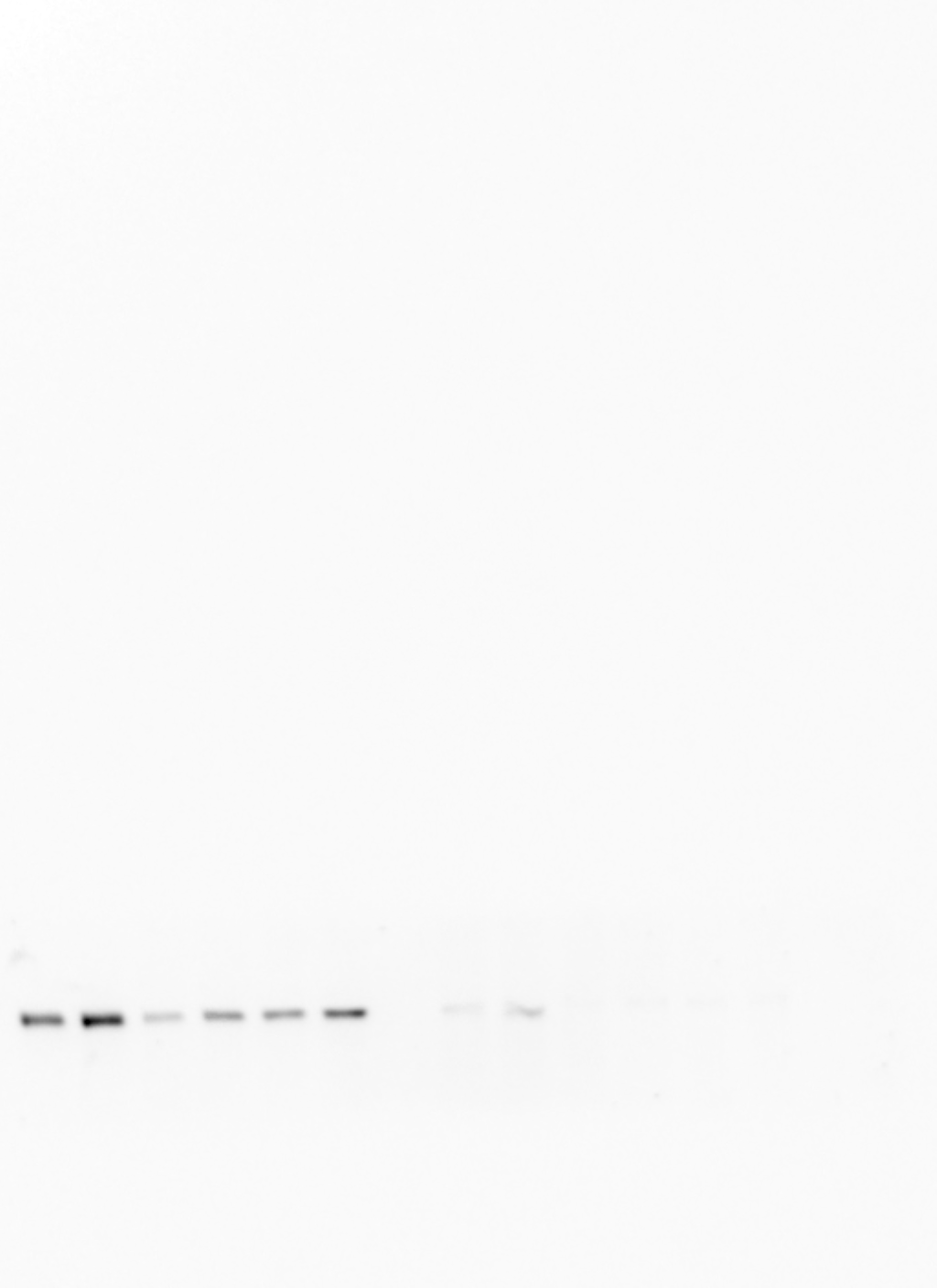

Supplement: Supplementary file 11 — Source data Fig. 6 [file 44321_2024_60_MOESM11_ESM.zip › Figure 6/6F/88T/Western PLK1 1.7/6 3rd PLK 1.7 _Ch.tif]

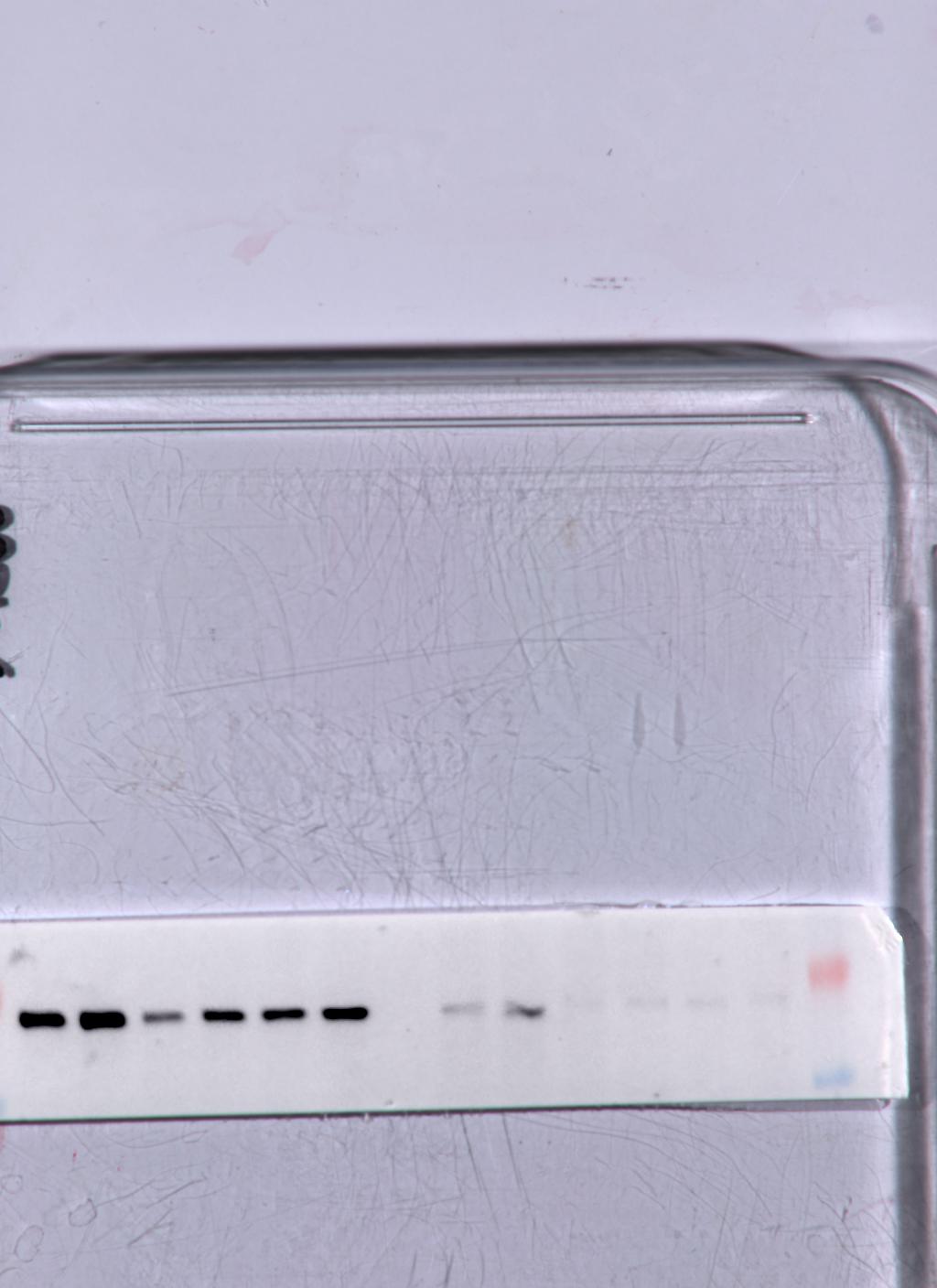

Supplement: Supplementary file 11 — Source data Fig. 6 [file 44321_2024_60_MOESM11_ESM.zip › Figure 6/6F/88T/Western PLK1 1.7/6 3rd PLK 1.7 _Ch+Marker.jpg]

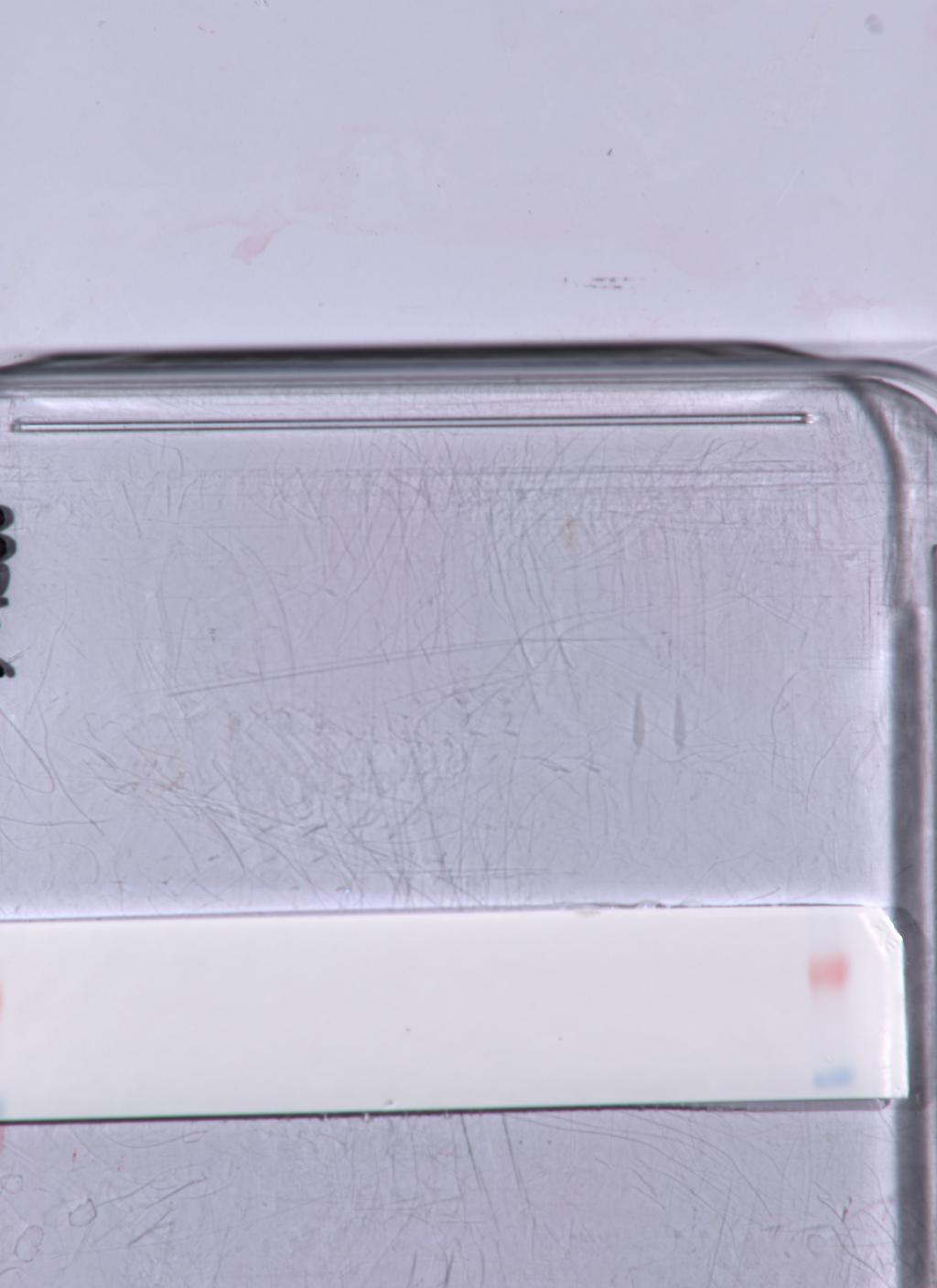

Supplement: Supplementary file 11 — Source data Fig. 6 [file 44321_2024_60_MOESM11_ESM.zip › Figure 6/6F/88T/Western PLK1 1.7/6 3rd PLK 1.7 _Ch-Marker.jpg]

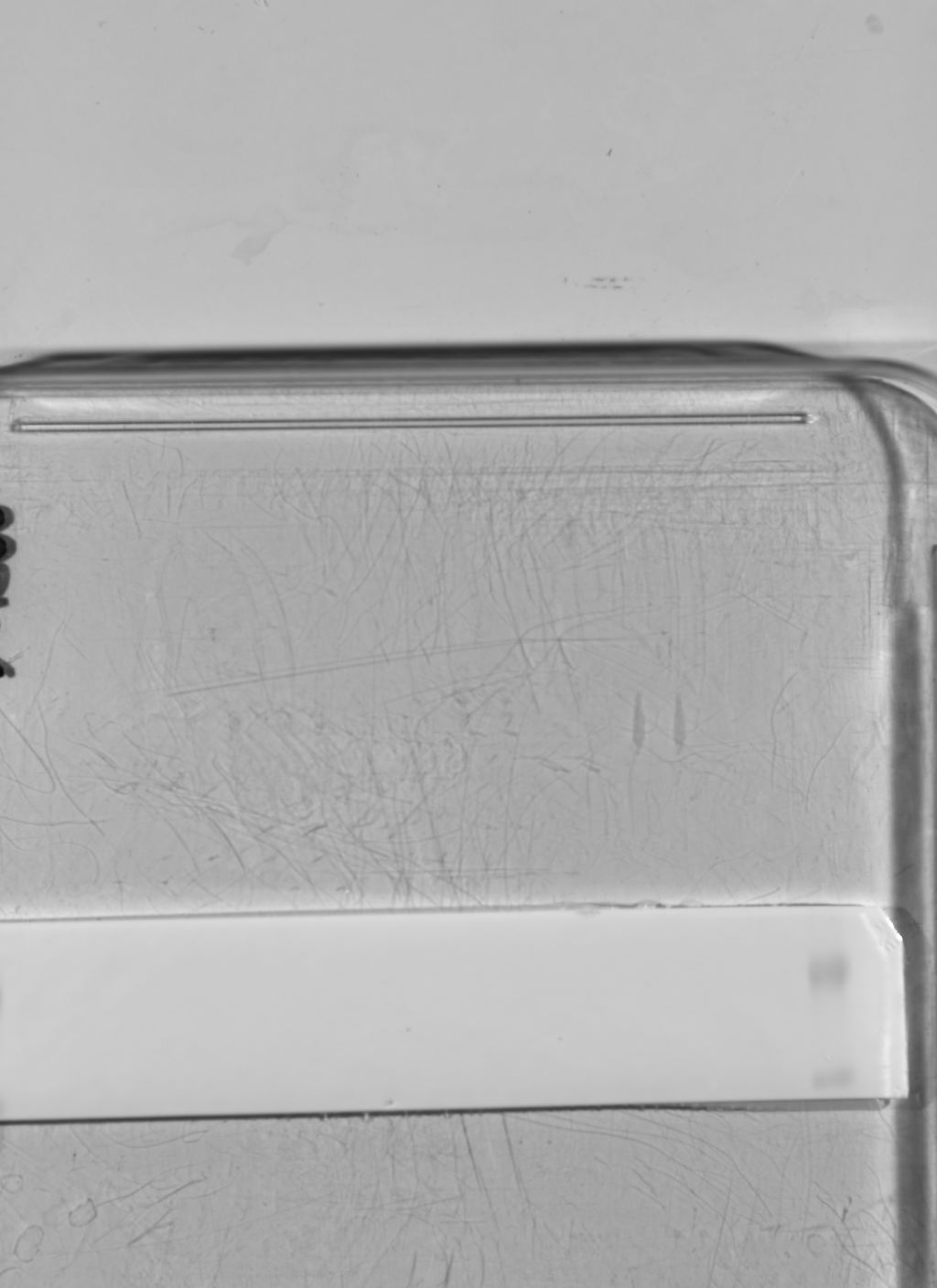

Supplement: Supplementary file 11 — Source data Fig. 6 [file 44321_2024_60_MOESM11_ESM.zip › Figure 6/6F/88T/Western PLK1 1.7/6 3rd PLK 1.7 _Ch-Marker.tif]

## Slide 1
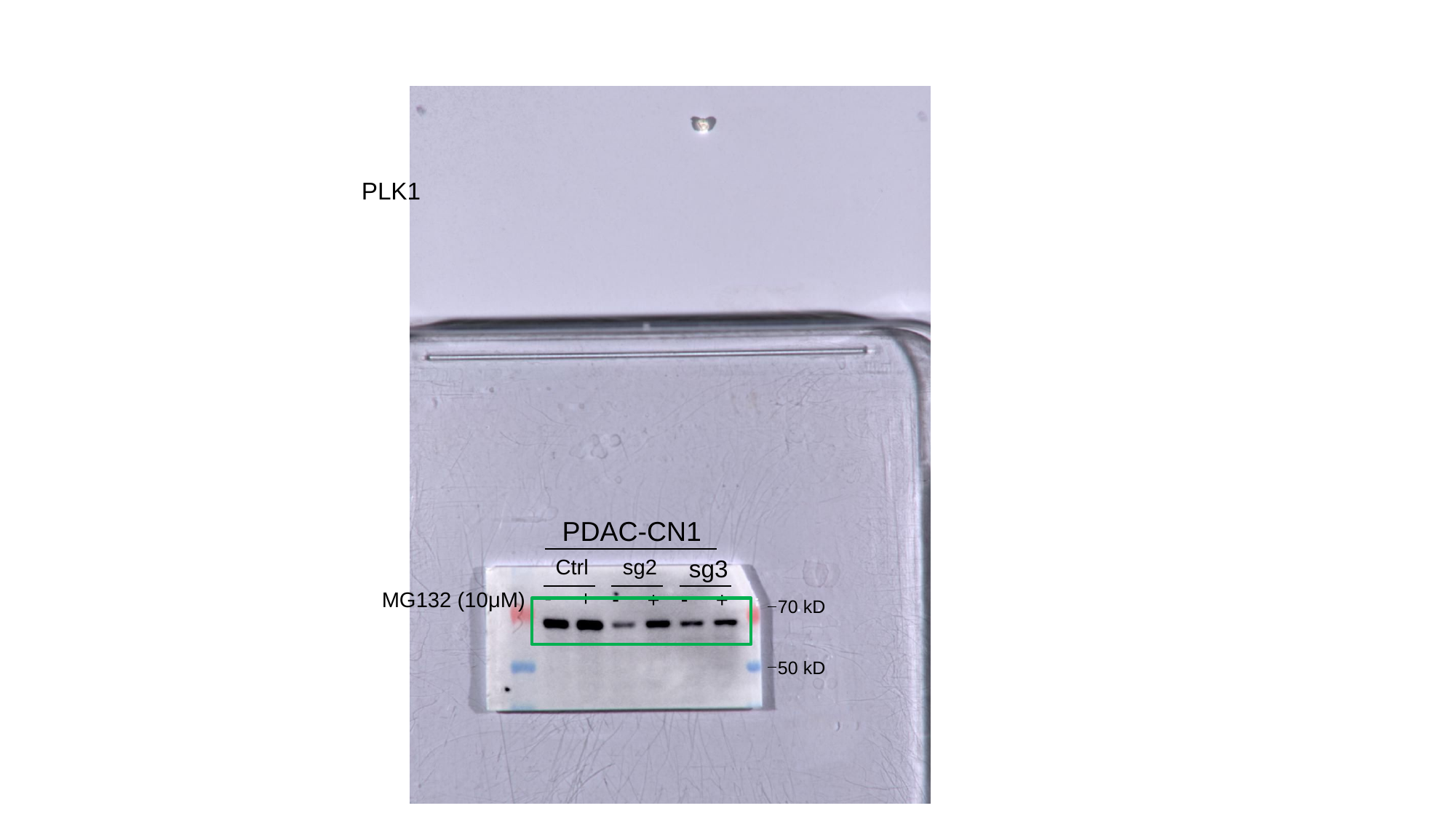

PLK1
PDAC-CN1
Ctrl
sg2
sg3
-
+
-
-
+
+
MG132 (10μM)
70 kD
50 kD

## Slide 2
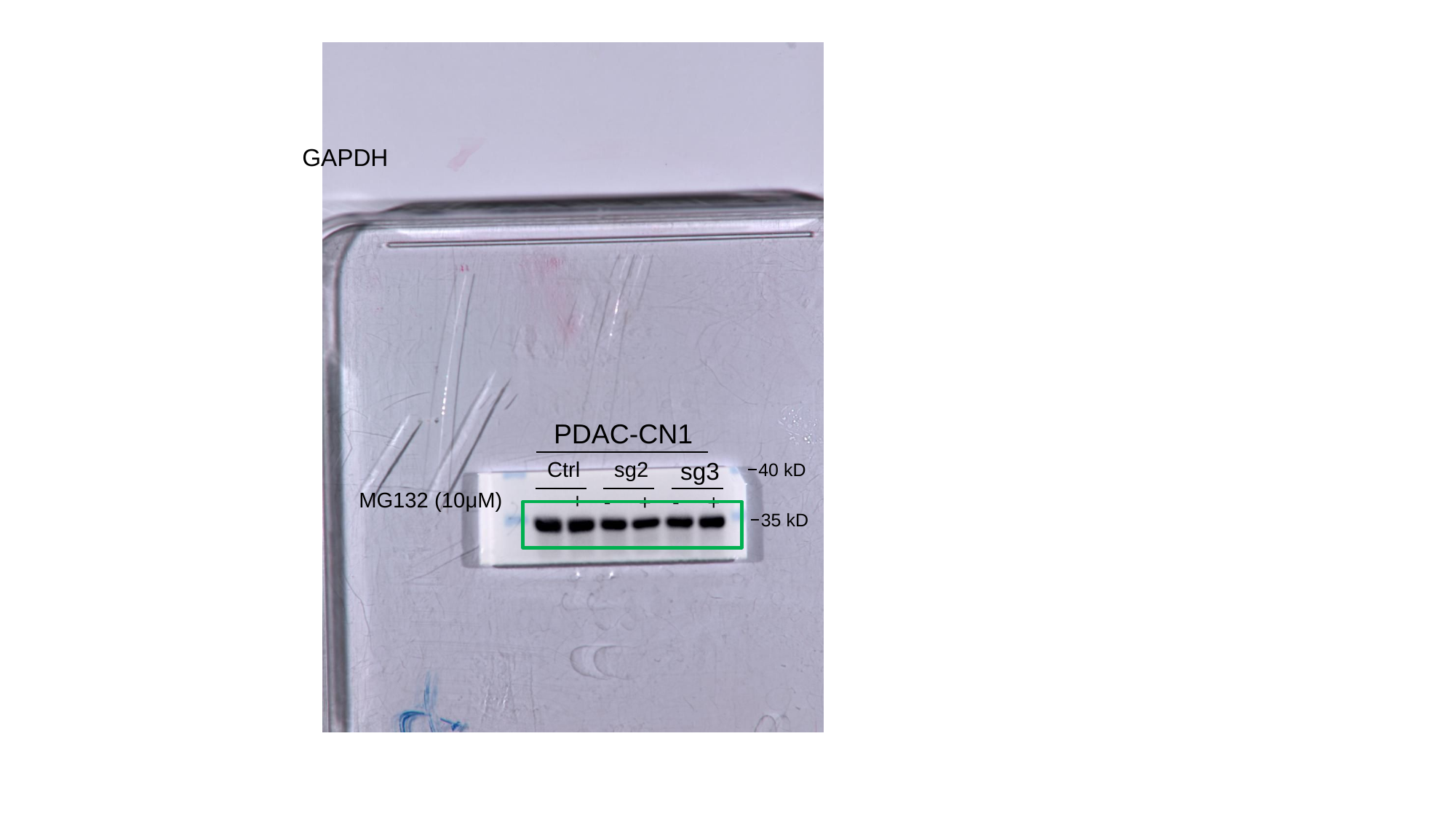

GAPDH
PDAC-CN1
Ctrl
sg2
sg3
-
+
-
-
+
+
40 kD
MG132 (10μM)
35 kD

Supplement: Supplementary file 11 — Source data Fig. 6 [file 44321_2024_60_MOESM11_ESM.zip › Figure 6/6F/CN1/6F CN1.pptx]

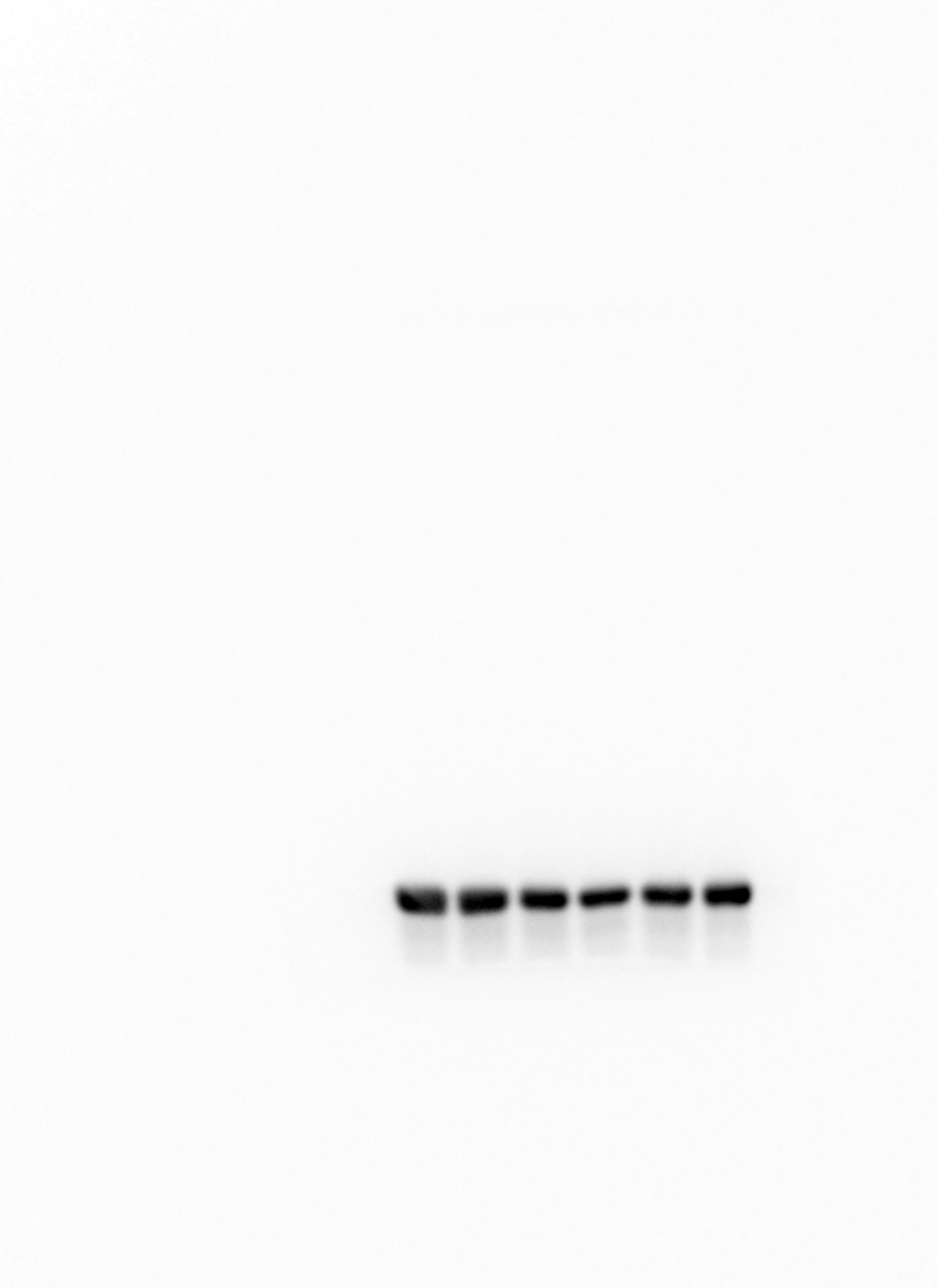

Supplement: Supplementary file 11 — Source data Fig. 6 [file 44321_2024_60_MOESM11_ESM.zip › Figure 6/6F/CN1/GAPDH 0.6/3-1 1st GAP 0.6 _Ch.tif]

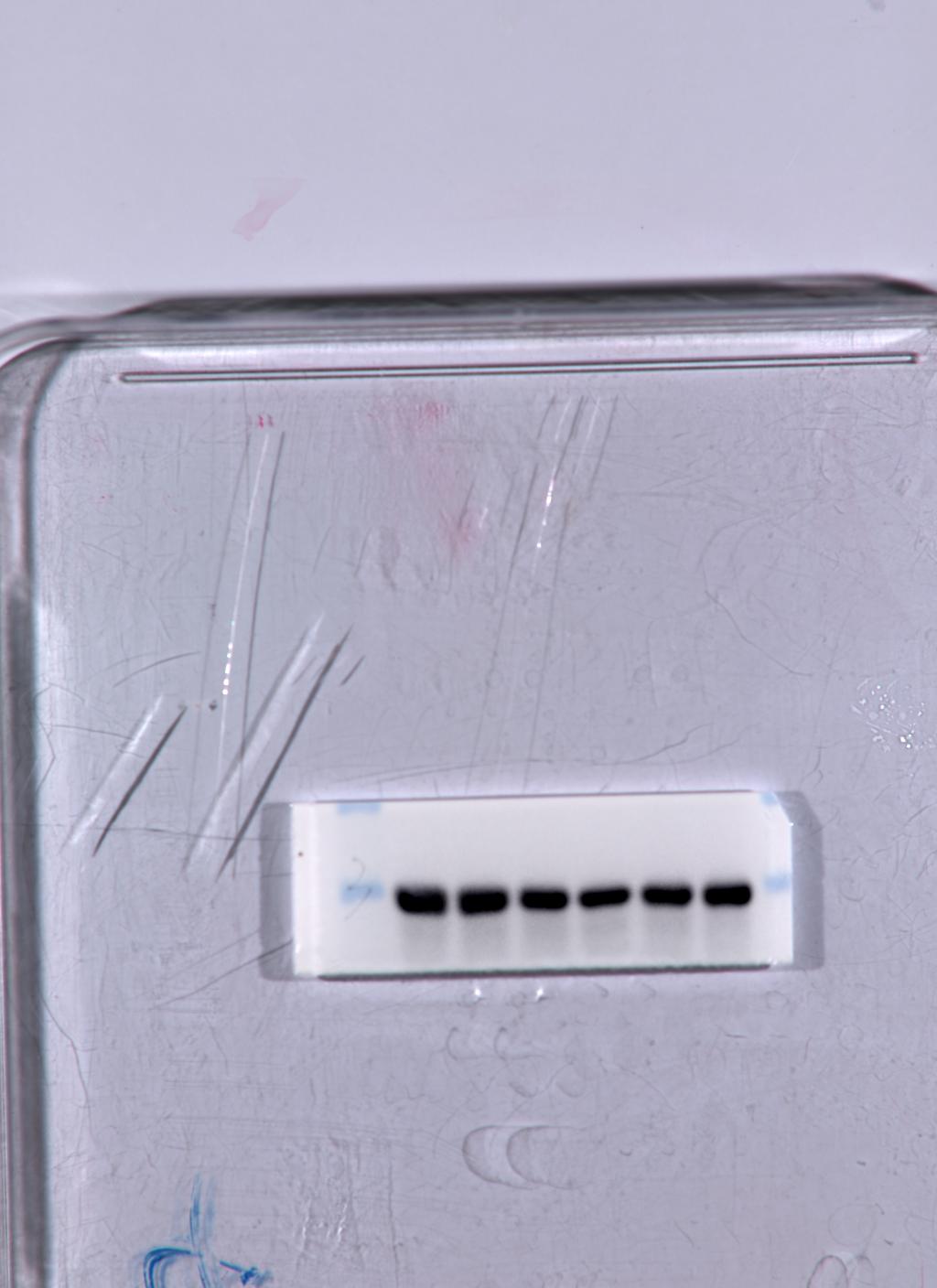

Supplement: Supplementary file 11 — Source data Fig. 6 [file 44321_2024_60_MOESM11_ESM.zip › Figure 6/6F/CN1/GAPDH 0.6/3-1 1st GAP 0.6 _Ch+Marker.jpg]

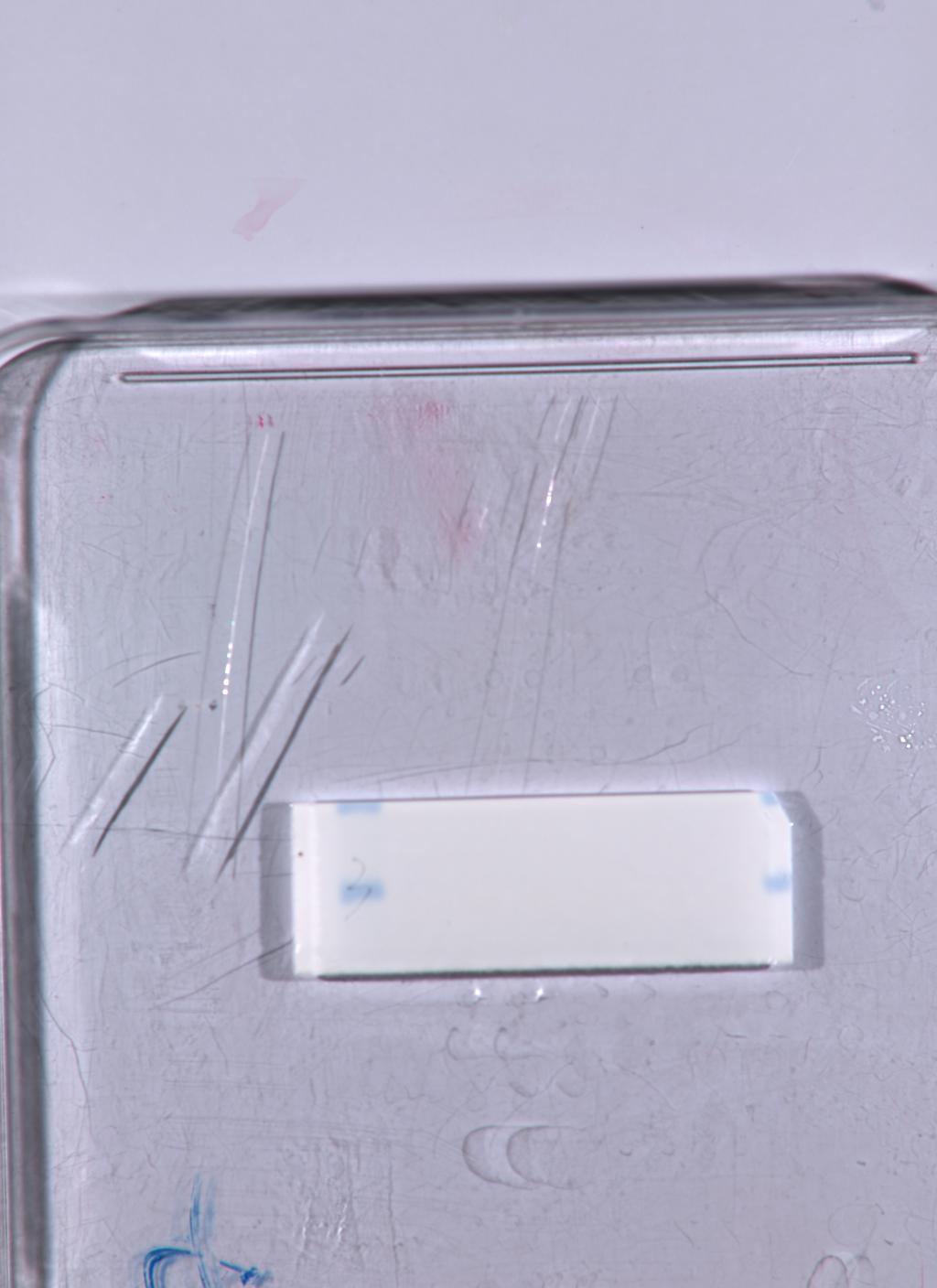

Supplement: Supplementary file 11 — Source data Fig. 6 [file 44321_2024_60_MOESM11_ESM.zip › Figure 6/6F/CN1/GAPDH 0.6/3-1 1st GAP 0.6 _Ch-Marker.jpg]

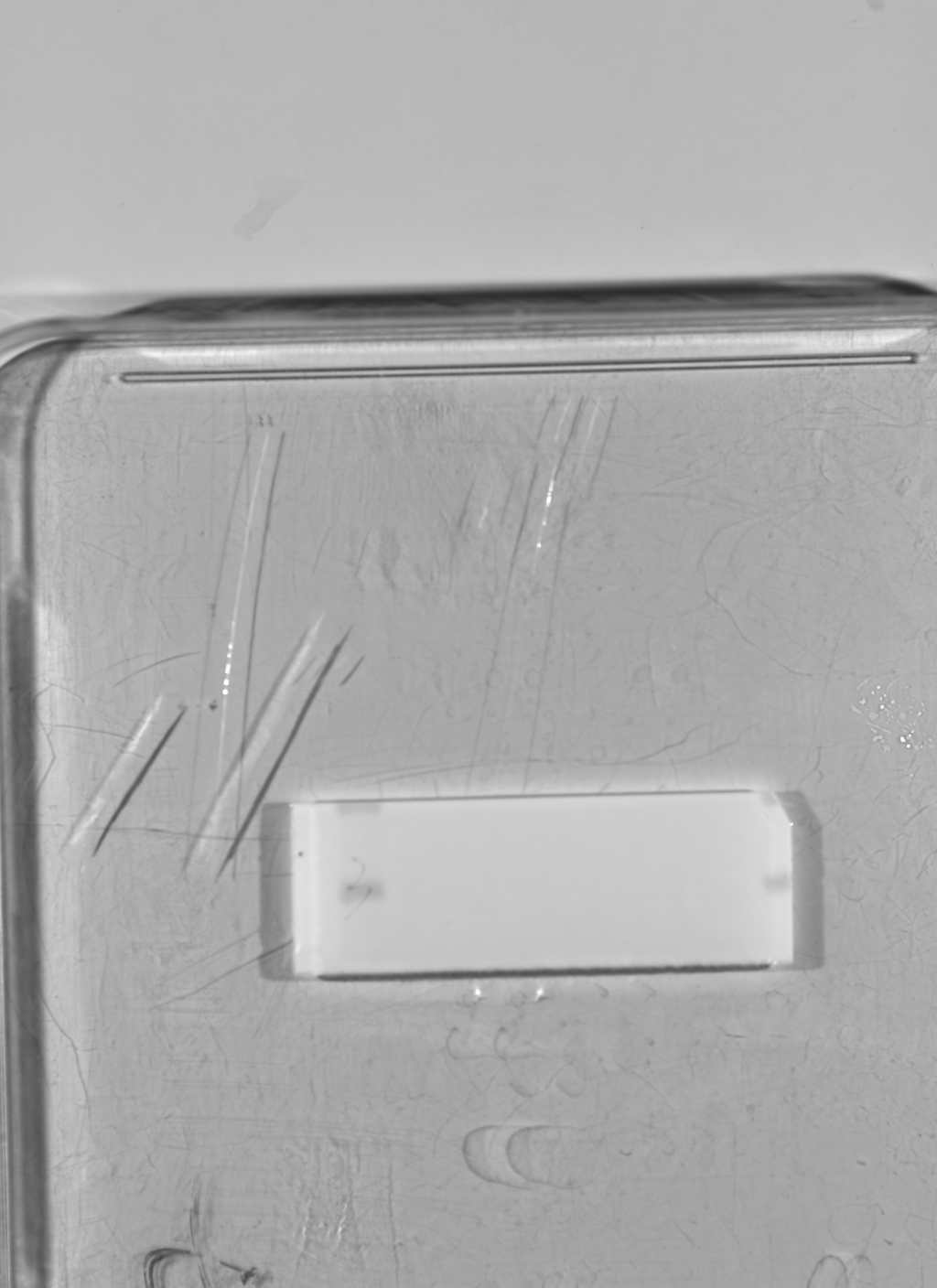

Supplement: Supplementary file 11 — Source data Fig. 6 [file 44321_2024_60_MOESM11_ESM.zip › Figure 6/6F/CN1/GAPDH 0.6/3-1 1st GAP 0.6 _Ch-Marker.tif]

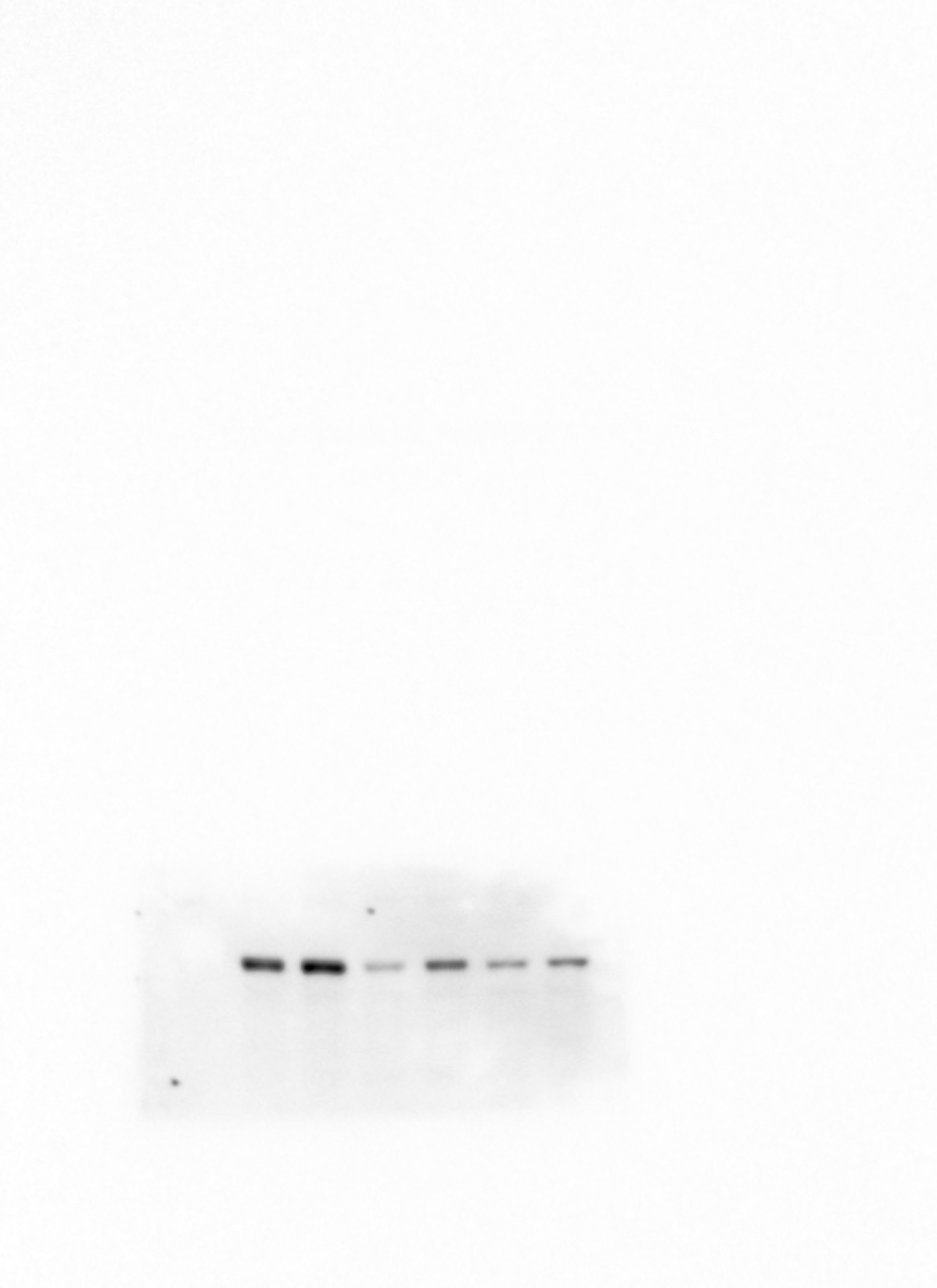

Supplement: Supplementary file 11 — Source data Fig. 6 [file 44321_2024_60_MOESM11_ESM.zip › Figure 6/6F/CN1/PLK1 15.1/_Ch.tif]

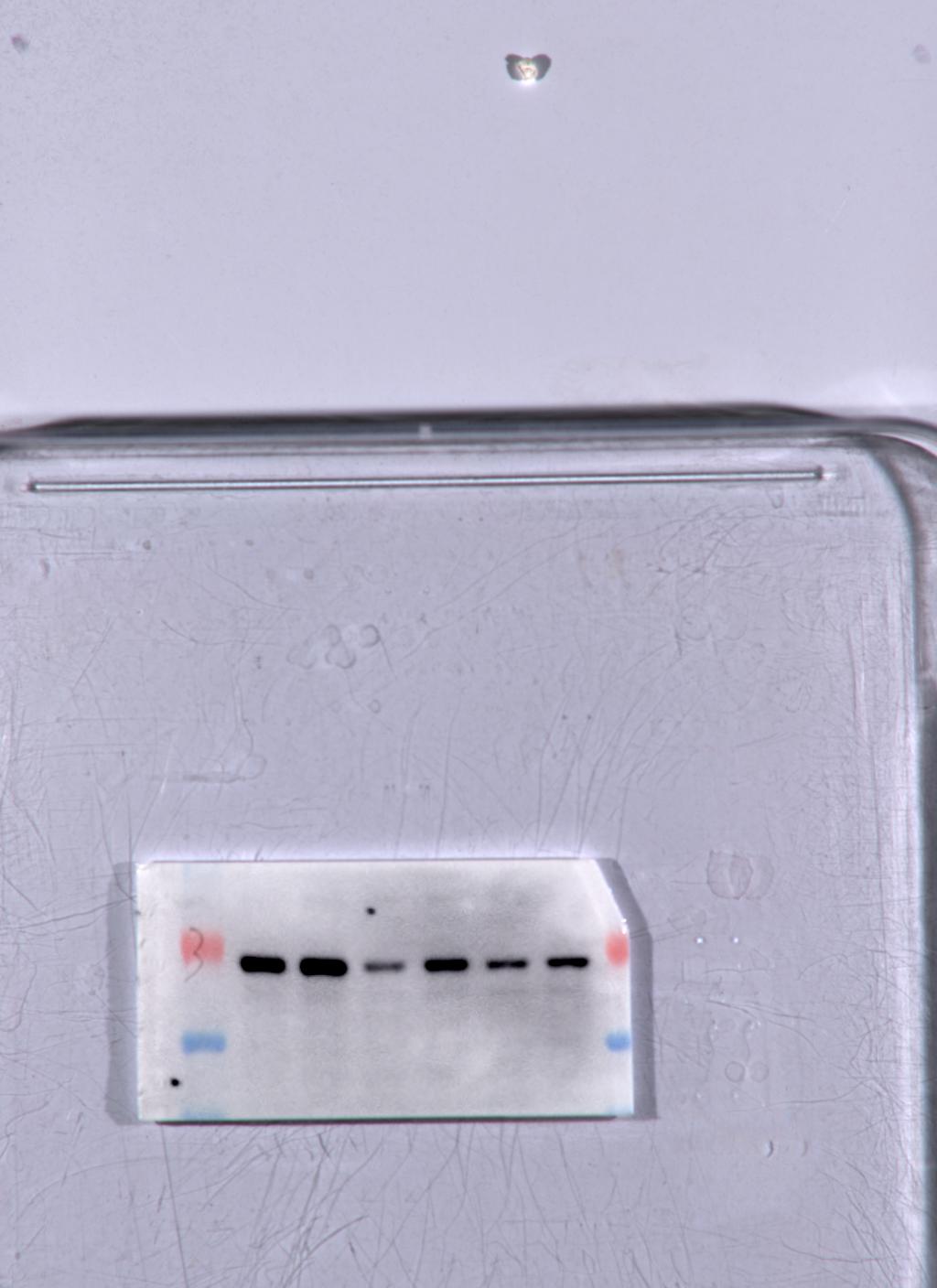

Supplement: Supplementary file 11 — Source data Fig. 6 [file 44321_2024_60_MOESM11_ESM.zip › Figure 6/6F/CN1/PLK1 15.1/3-1 1st PLK 15.1 _Ch+Marker.jpg]

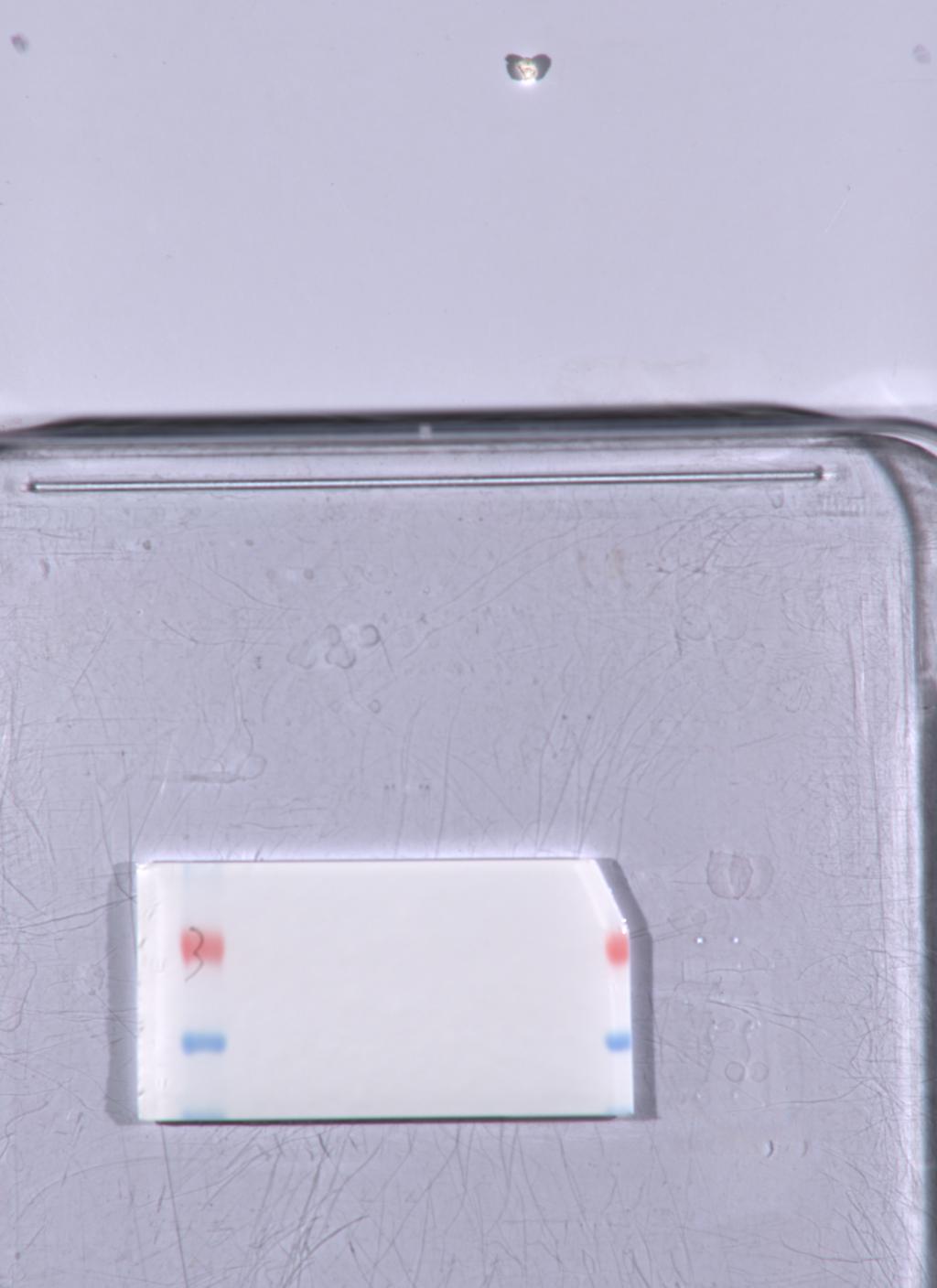

Supplement: Supplementary file 11 — Source data Fig. 6 [file 44321_2024_60_MOESM11_ESM.zip › Figure 6/6F/CN1/PLK1 15.1/3-1 1st PLK 15.1 _Ch-Marker.jpg]

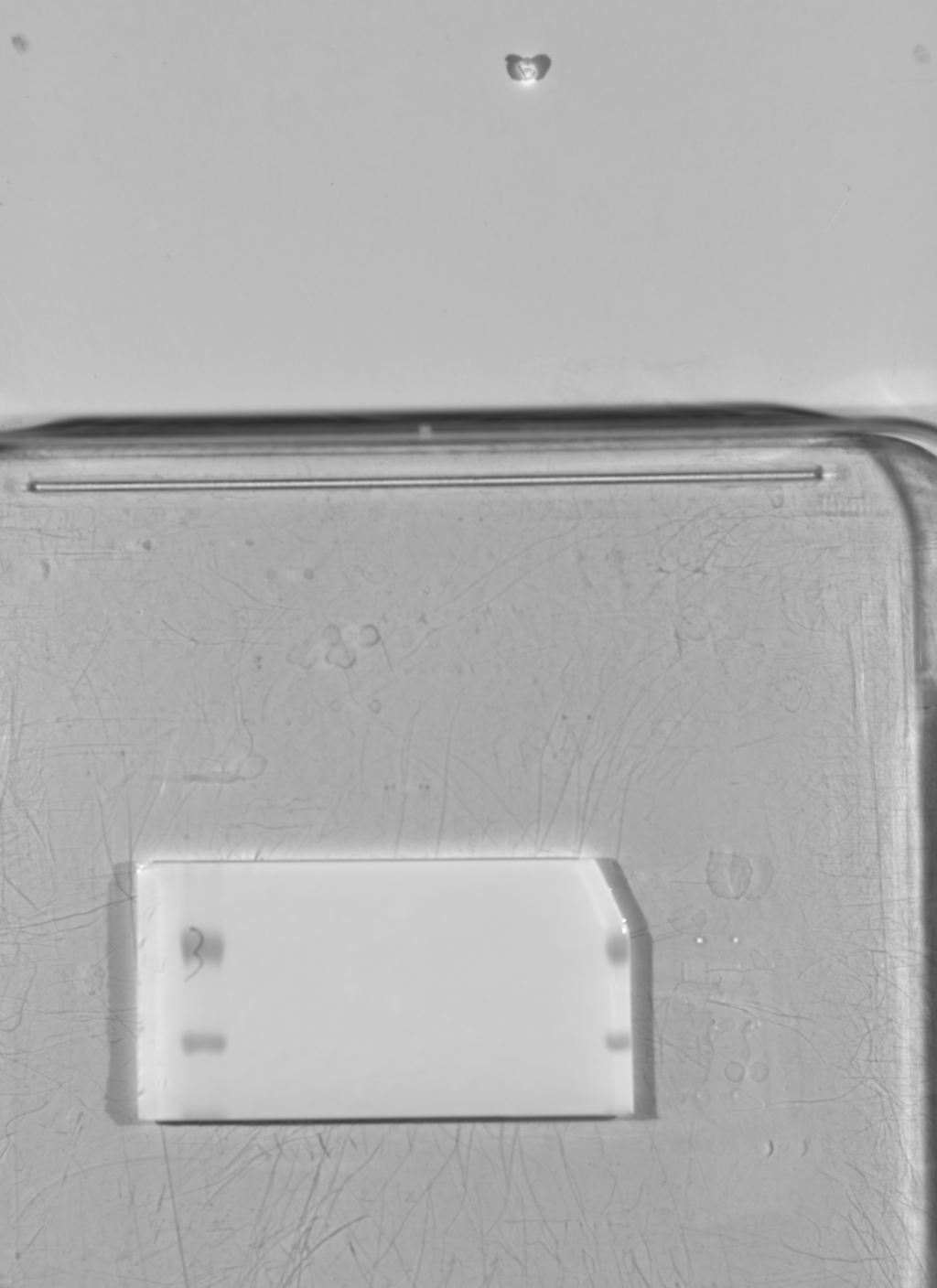

Supplement: Supplementary file 11 — Source data Fig. 6 [file 44321_2024_60_MOESM11_ESM.zip › Figure 6/6F/CN1/PLK1 15.1/3-1 1st PLK 15.1 _Ch-Marker.tif]

## Slide 1
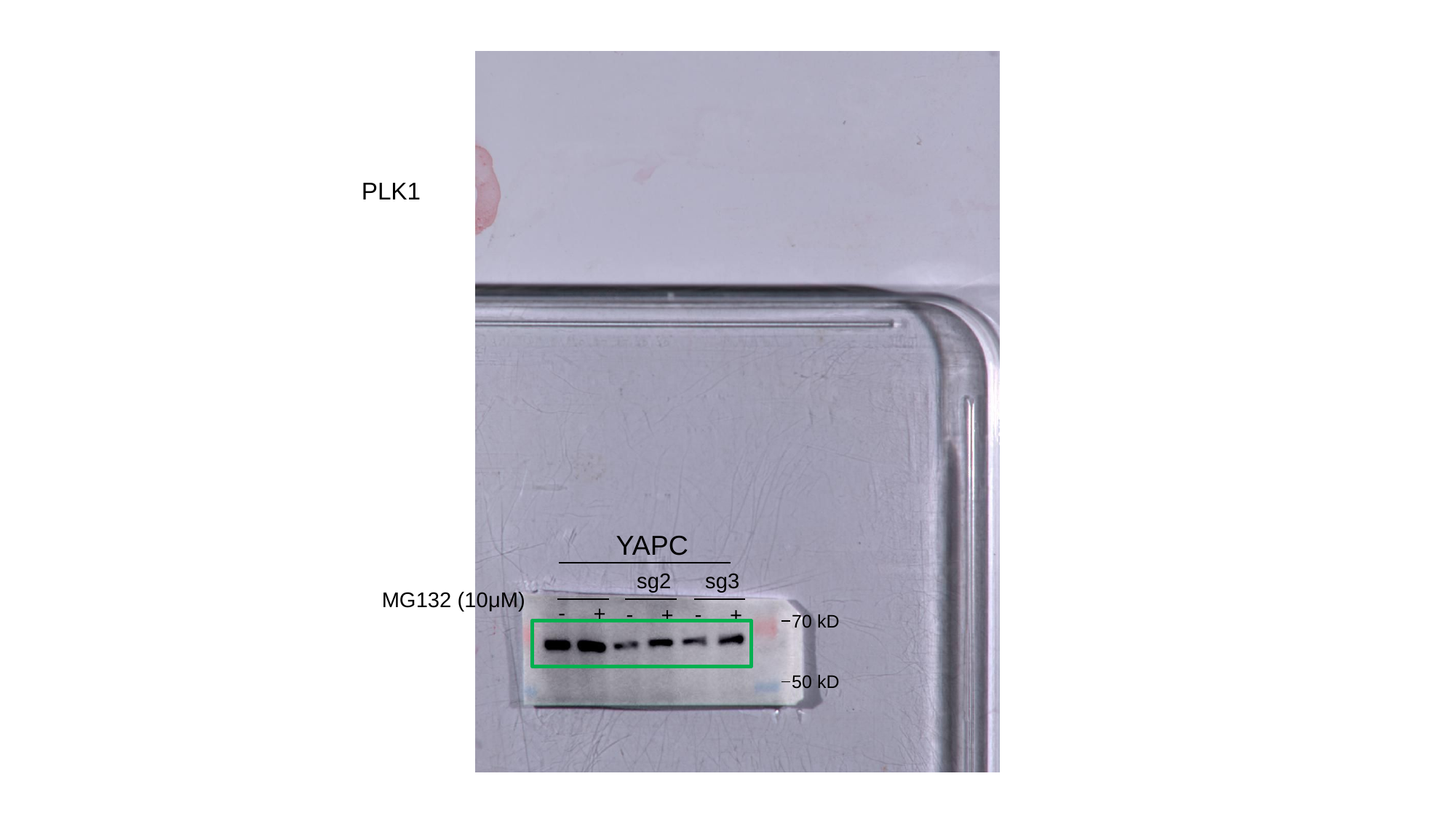

PLK1
YAPC
sg2
sg3
MG132 (10μM)
-
+
-
-
+
+
70 kD
50 kD

## Slide 2
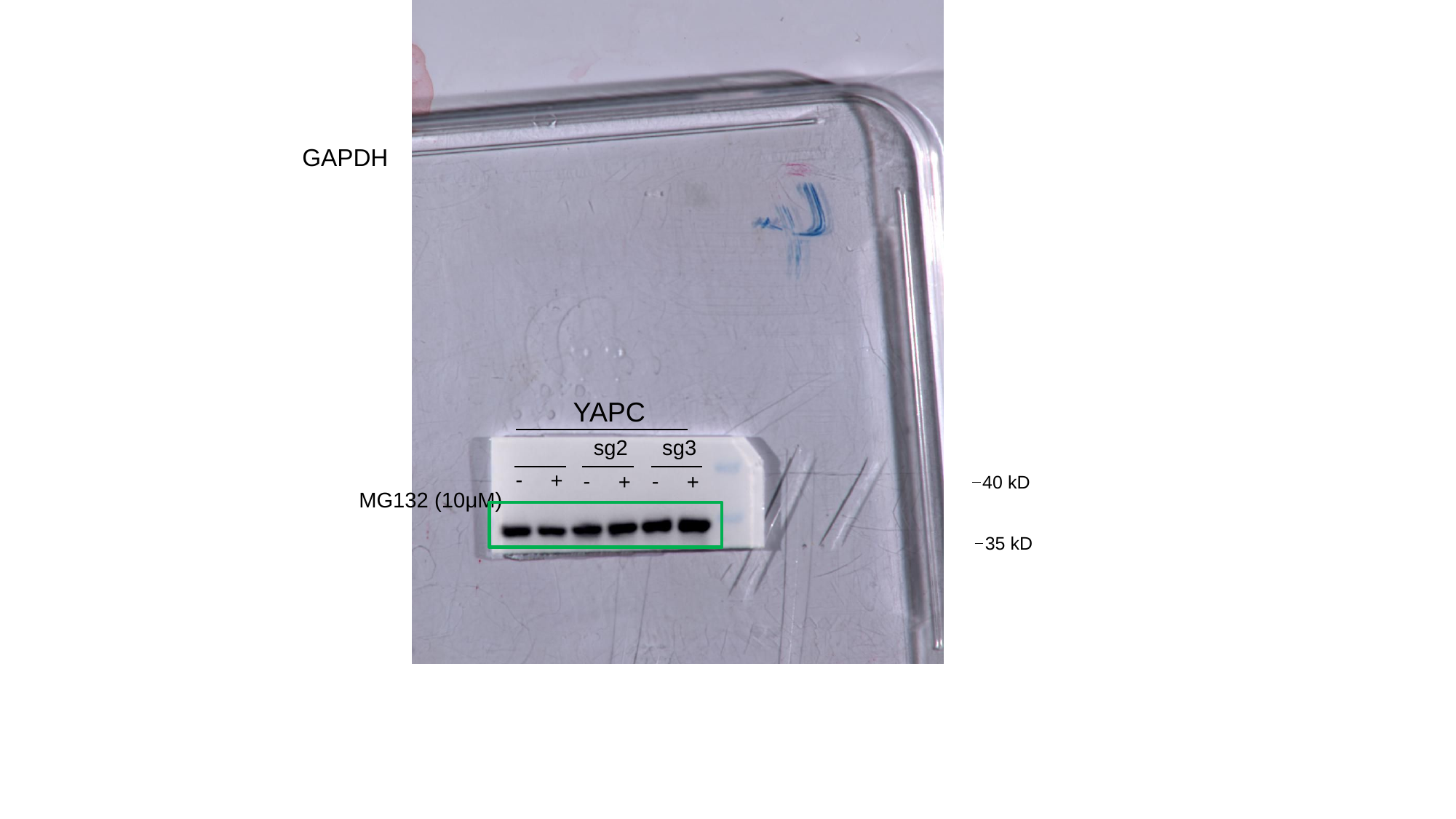

GAPDH
YAPC
sg2
sg3
-
+
-
-
+
+
40 kD
MG132 (10μM)
35 kD

Supplement: Supplementary file 11 — Source data Fig. 6 [file 44321_2024_60_MOESM11_ESM.zip › Figure 6/6F/YAPC/6F YAPC.pptx]

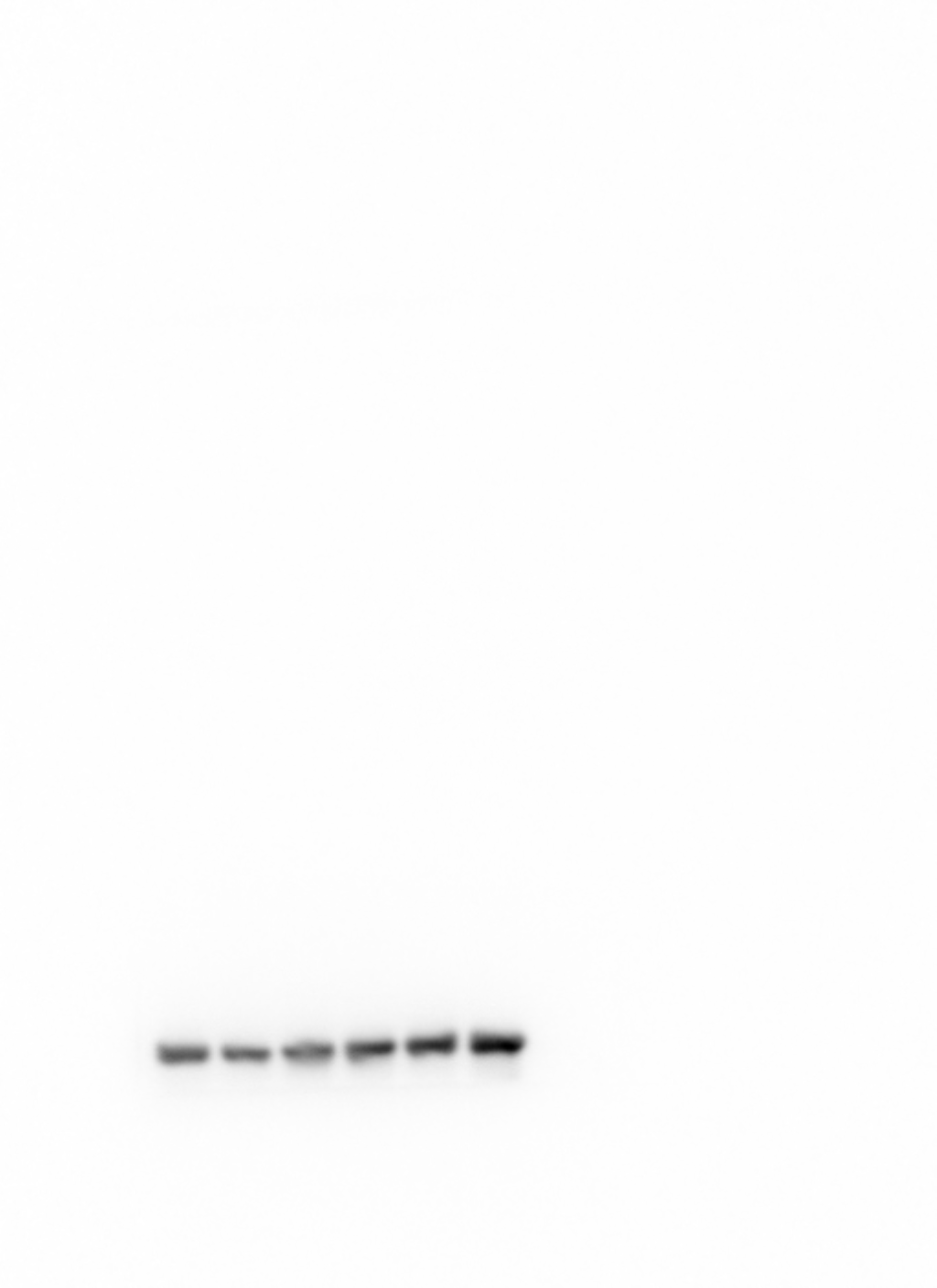

Supplement: Supplementary file 11 — Source data Fig. 6 [file 44321_2024_60_MOESM11_ESM.zip › Figure 6/6F/YAPC/GAPDH 0.1/5-2 GAP 0.1 _Ch.tif]

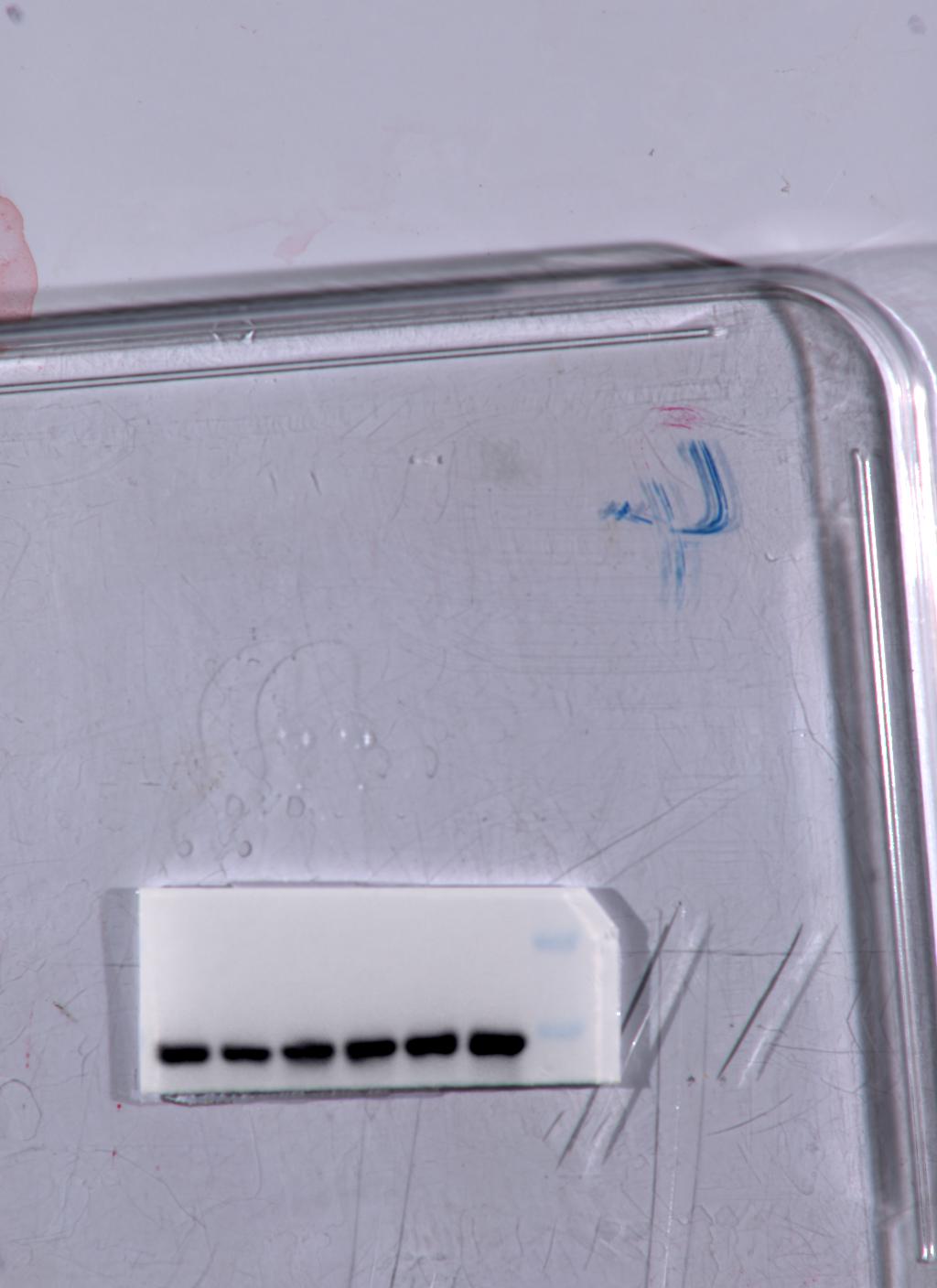

Supplement: Supplementary file 11 — Source data Fig. 6 [file 44321_2024_60_MOESM11_ESM.zip › Figure 6/6F/YAPC/GAPDH 0.1/5-2 GAP 0.1 _Ch+Marker.jpg]

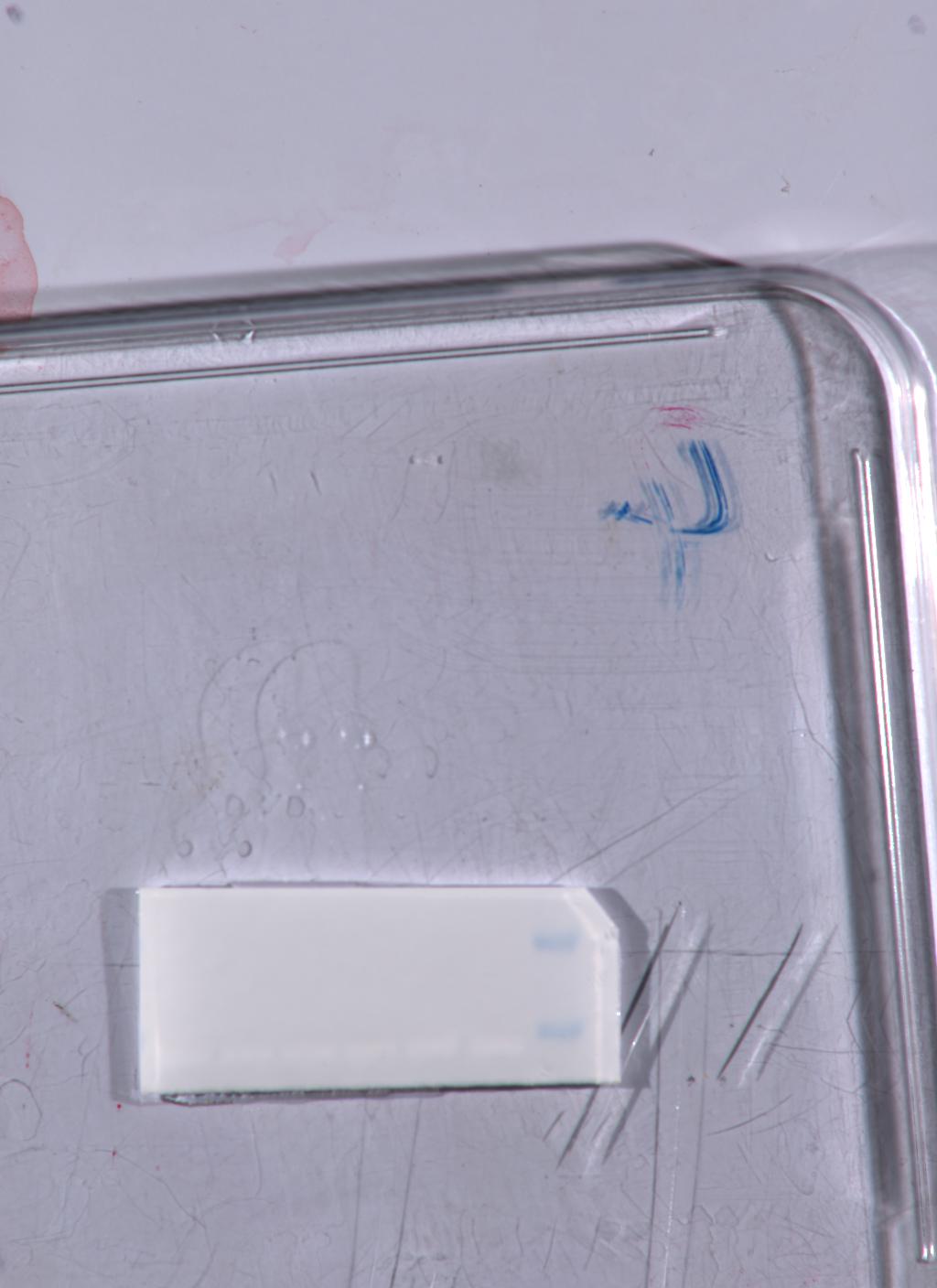

Supplement: Supplementary file 11 — Source data Fig. 6 [file 44321_2024_60_MOESM11_ESM.zip › Figure 6/6F/YAPC/GAPDH 0.1/5-2 GAP 0.1 _Ch-Marker.jpg]
