# Supplementary material for: Genome-wide CRISPR screens identify PKMYT1 as a therapeutic target in pancreatic ductal adenocarcinoma
Source: EMBO Mol Med. 2024 Apr 3;16(5):5. doi: 10.1038/s44321-024-00060-y (PMC11099189; doi:10.1038/s44321-024-00060-y)
Supplement: Supplementary file 14 — EV Figure Source Data [file 44321_2024_60_MOESM14_ESM.zip › Figure EV2C Source Data/EV2C/88T YAPC/EV2C 88T YAPC.pptx]

## Slide 1
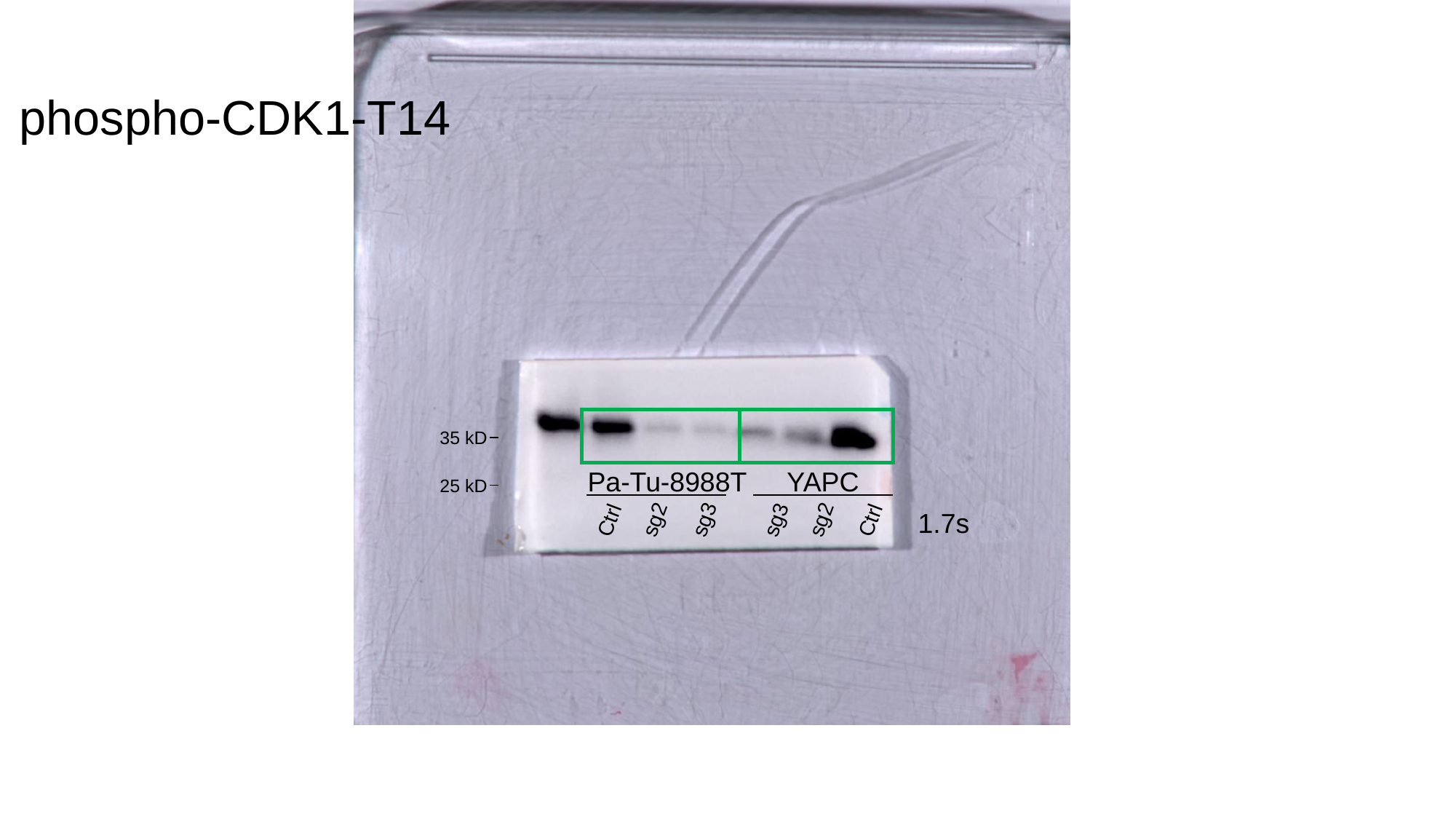

phospho-CDK1-T14
35 kD
Pa-Tu-8988T
YAPC
Ctrl
sg3
25 kD
sg3
sg2
Ctrl
sg2
1.7s

## Slide 2
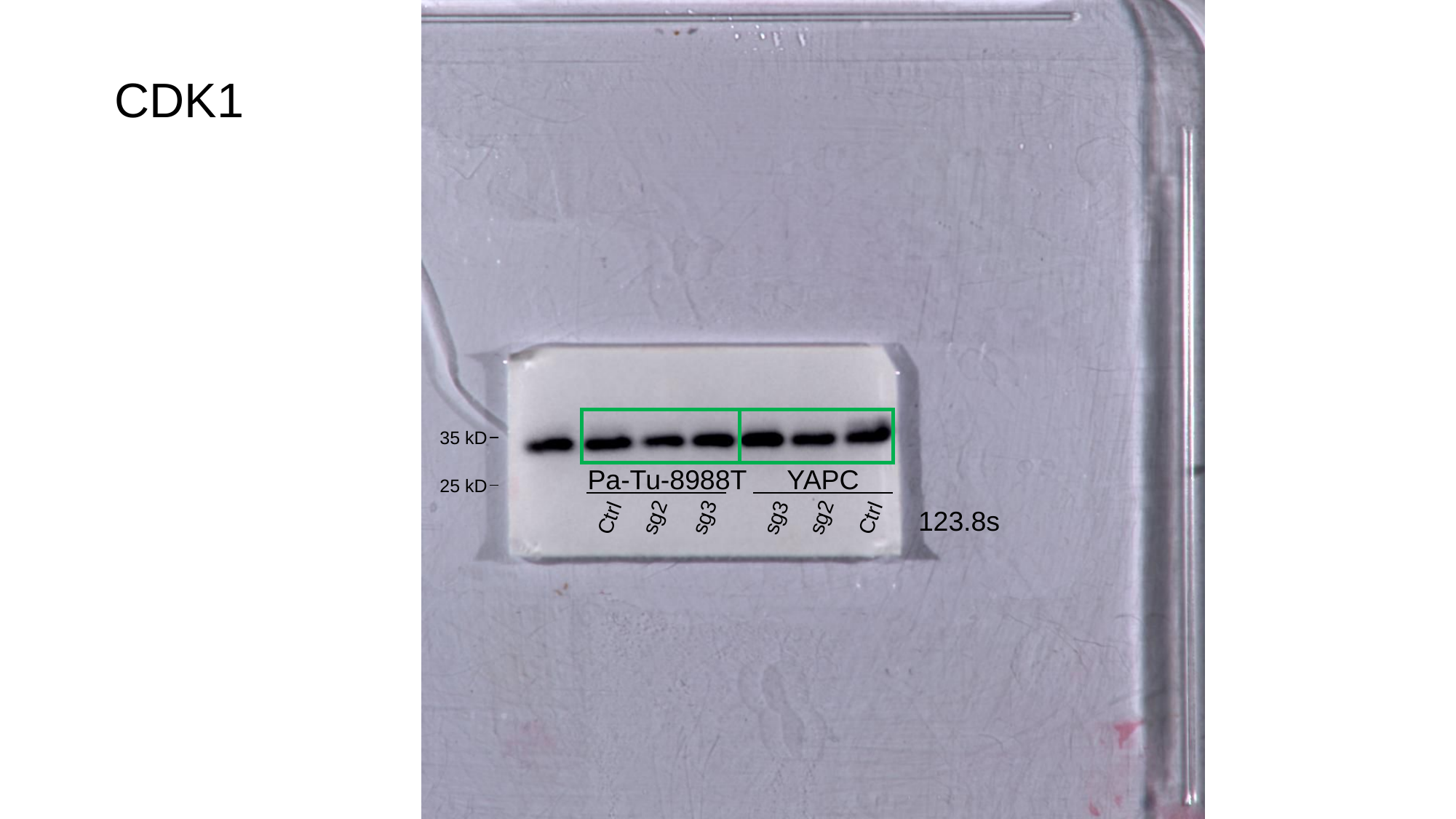

CDK1
35 kD
Pa-Tu-8988T
YAPC
Ctrl
sg3
25 kD
sg3
sg2
Ctrl
sg2
123.8s

## Slide 3
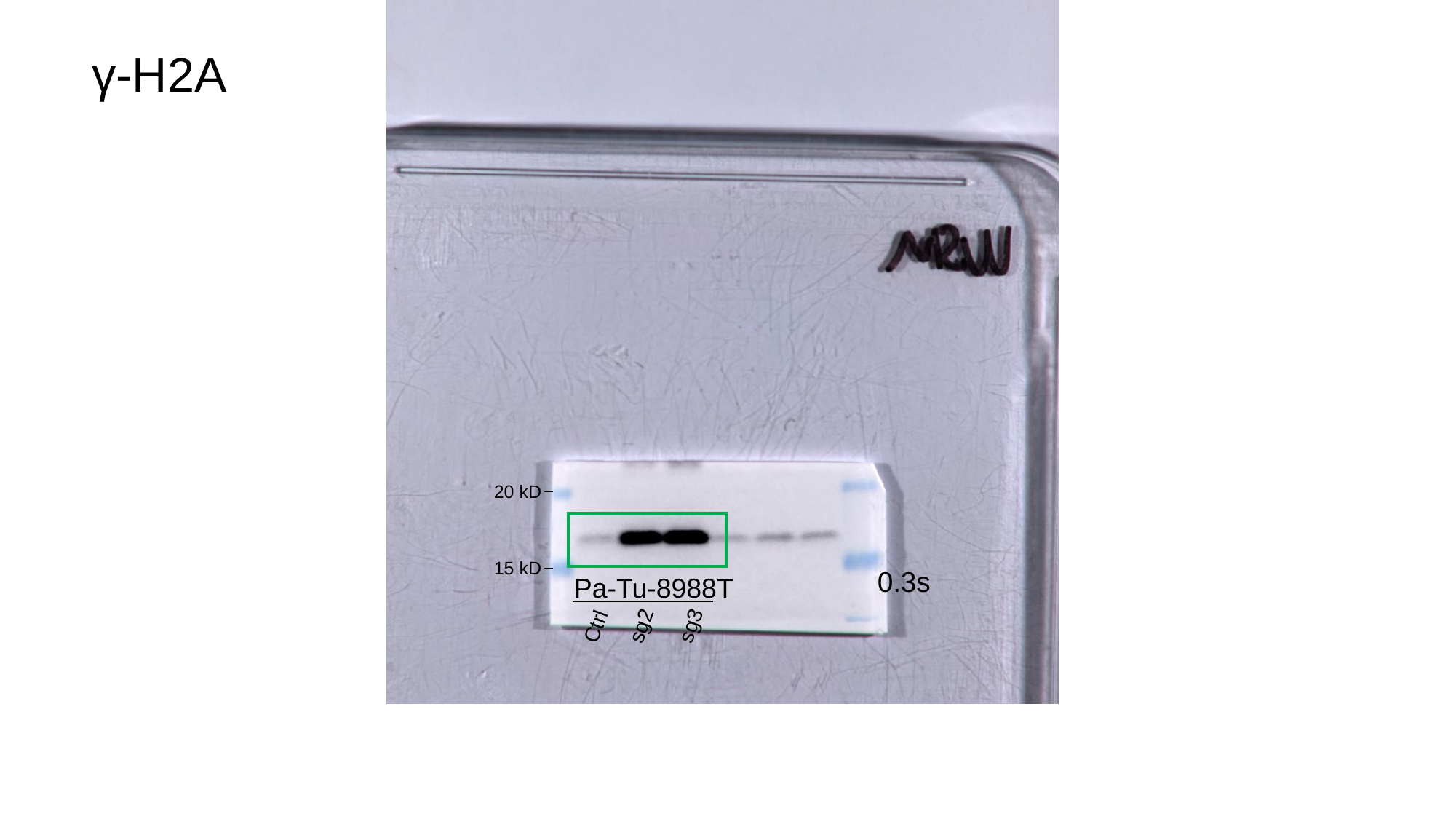

γ-H2A
20 kD
15 kD
0.3s
Pa-Tu-8988T
Ctrl
sg3
sg2

## Slide 4
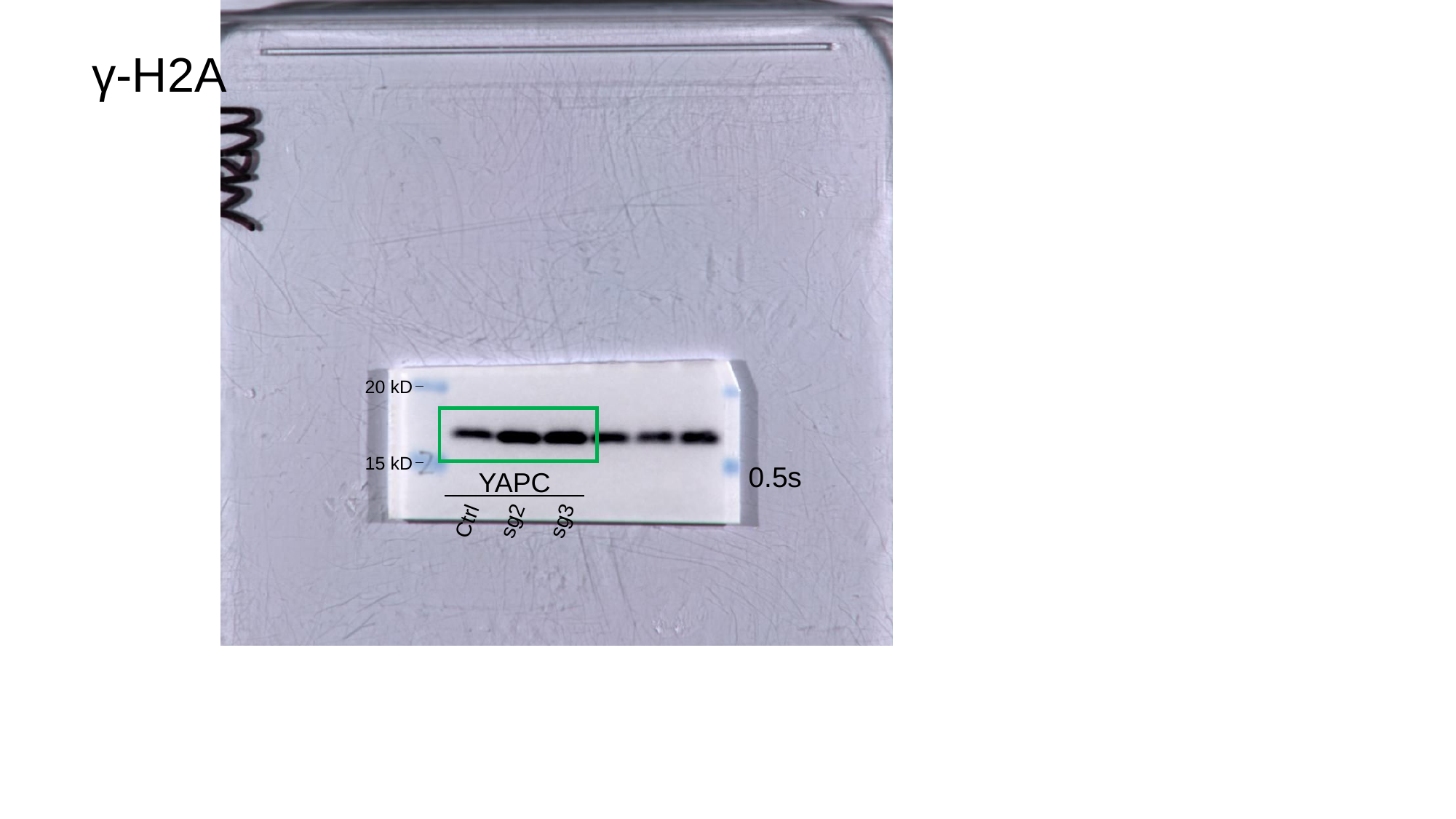

γ-H2A
20 kD
15 kD
0.5s
YAPC
Ctrl
sg3
sg2

## Slide 5
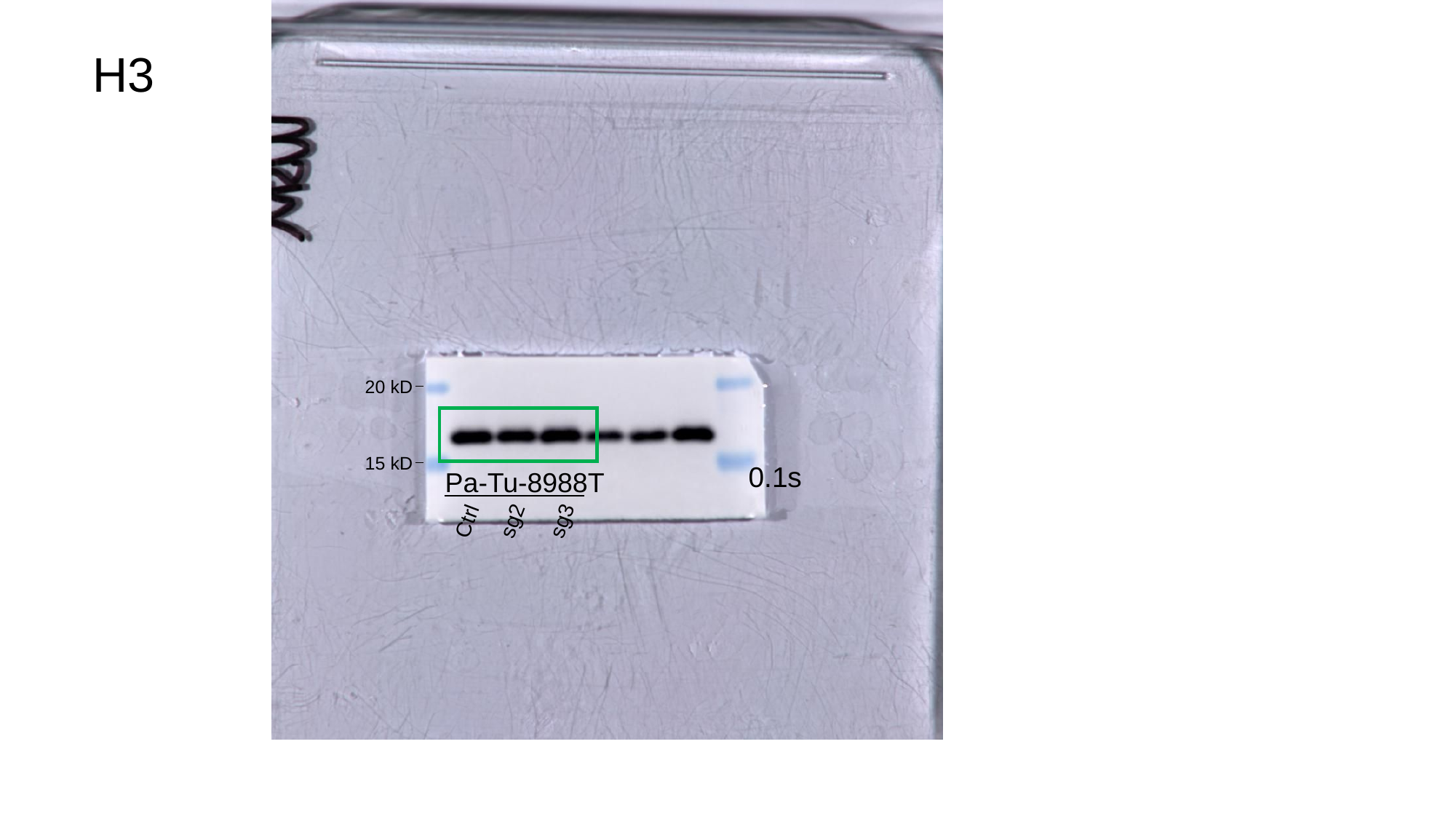

H3
20 kD
15 kD
0.1s
Pa-Tu-8988T
Ctrl
sg3
sg2

## Slide 6
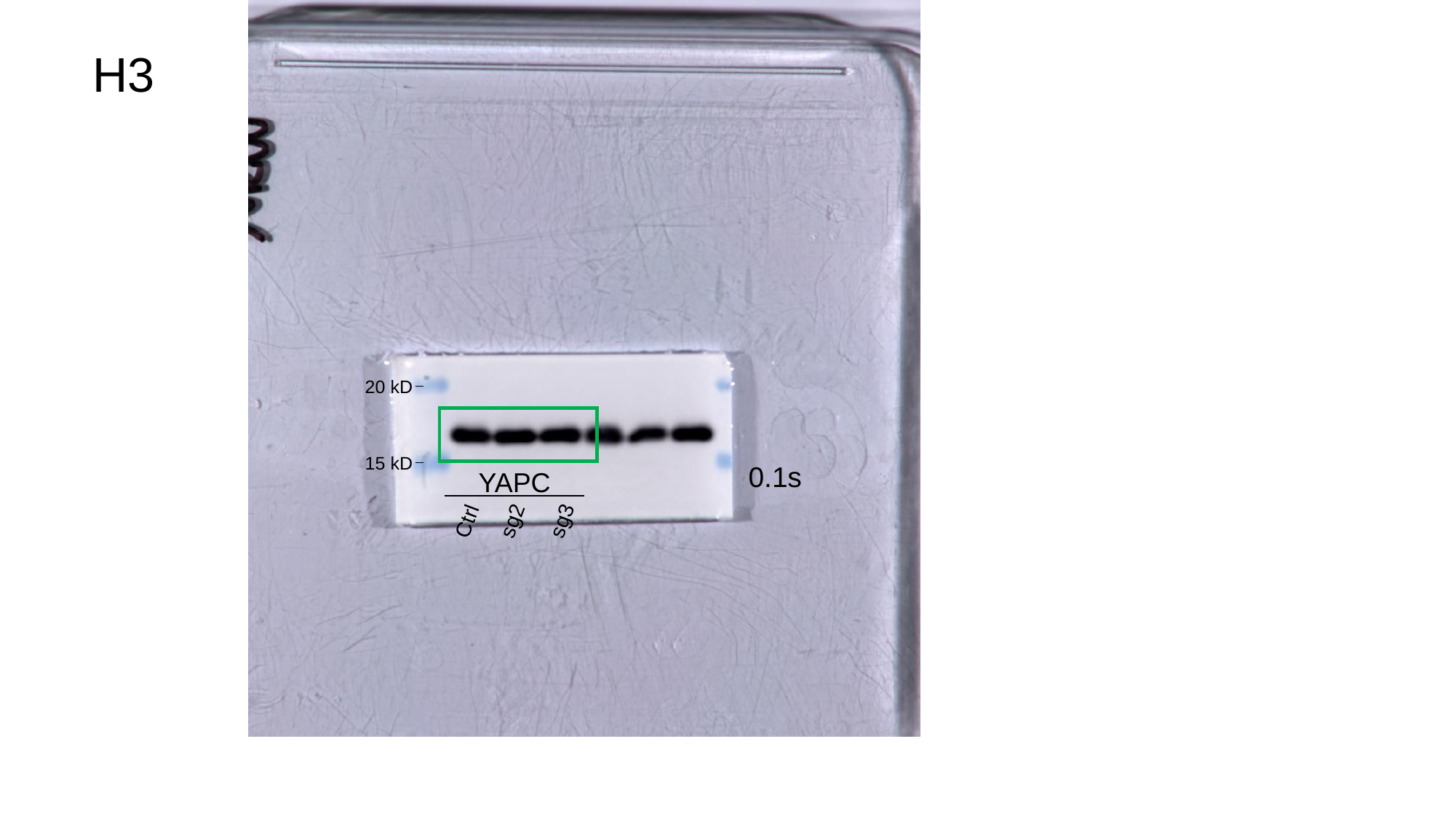

H3
20 kD
15 kD
0.1s
YAPC
Ctrl
sg3
sg2

## Slide 7
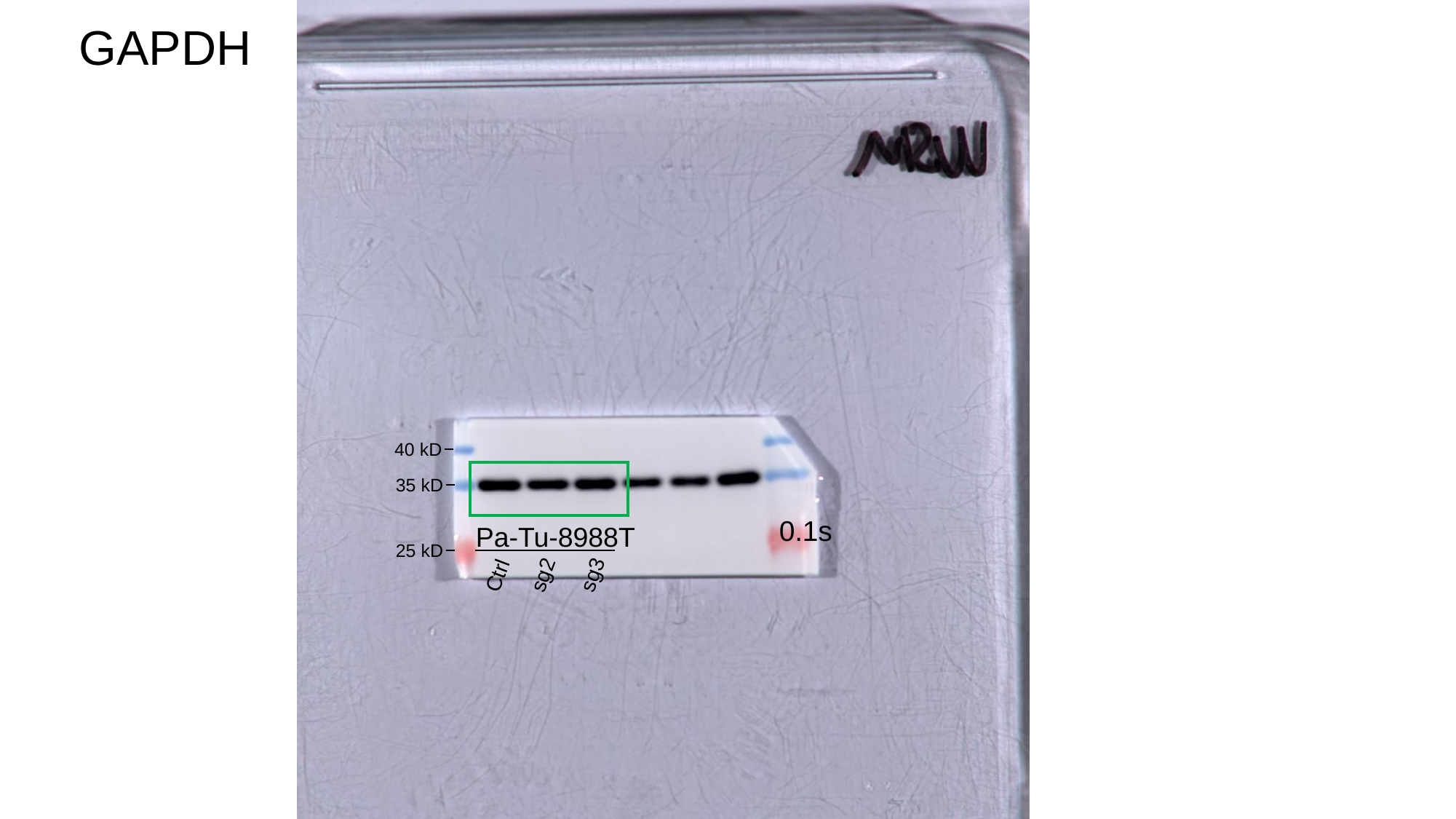

GAPDH
40 kD
35 kD
0.1s
Pa-Tu-8988T
Ctrl
25 kD
sg3
sg2

## Slide 8
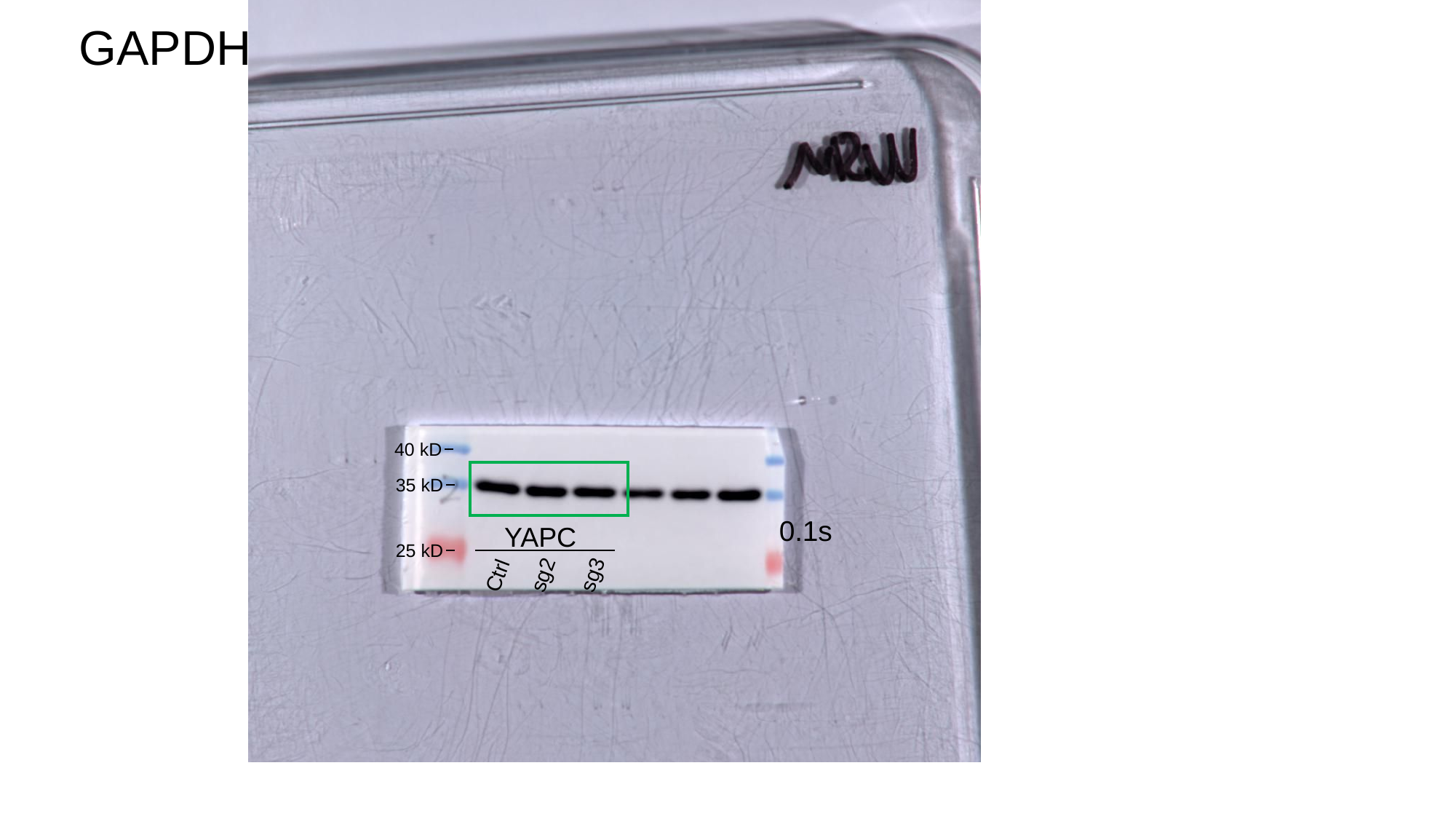

GAPDH
40 kD
35 kD
0.1s
YAPC
Ctrl
25 kD
sg3
sg2
