# Supplementary material for: Genome-wide CRISPR screens identify PKMYT1 as a therapeutic target in pancreatic ductal adenocarcinoma
Source: EMBO Mol Med. 2024 Apr 3;16(5):5. doi: 10.1038/s44321-024-00060-y (PMC11099189; doi:10.1038/s44321-024-00060-y)
Supplement: Supplementary file 14 — EV Figure Source Data [file 44321_2024_60_MOESM14_ESM.zip › Figure EV2C Source Data/EV2C/CN1/EV2C CN1.pptx]

## Slide 1
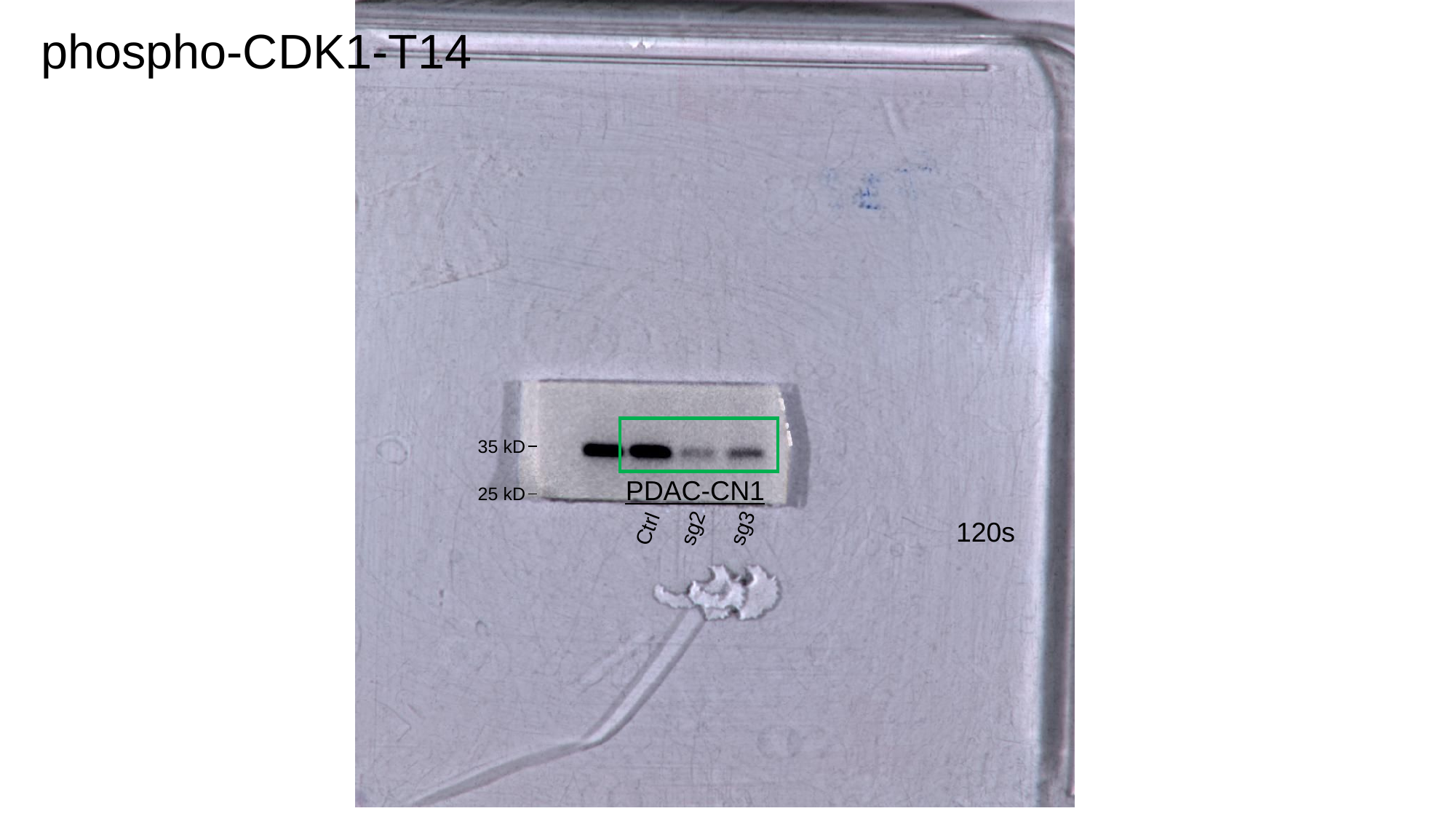

phospho-CDK1-T14
35 kD
PDAC-CN1
Ctrl
25 kD
sg3
sg2
120s

## Slide 2
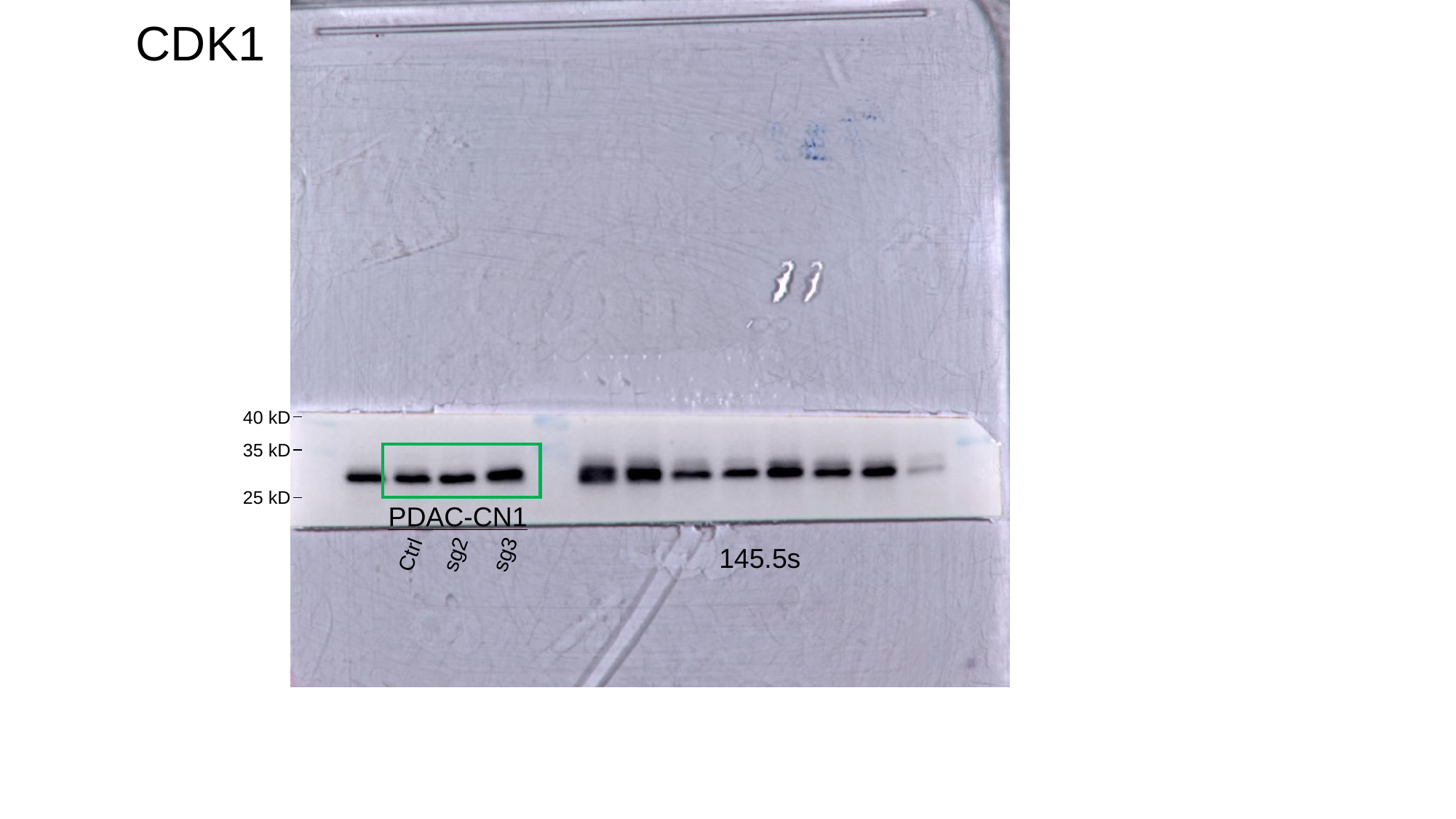

CDK1
40 kD
35 kD
25 kD
PDAC-CN1
Ctrl
sg3
sg2
145.5s

## Slide 3
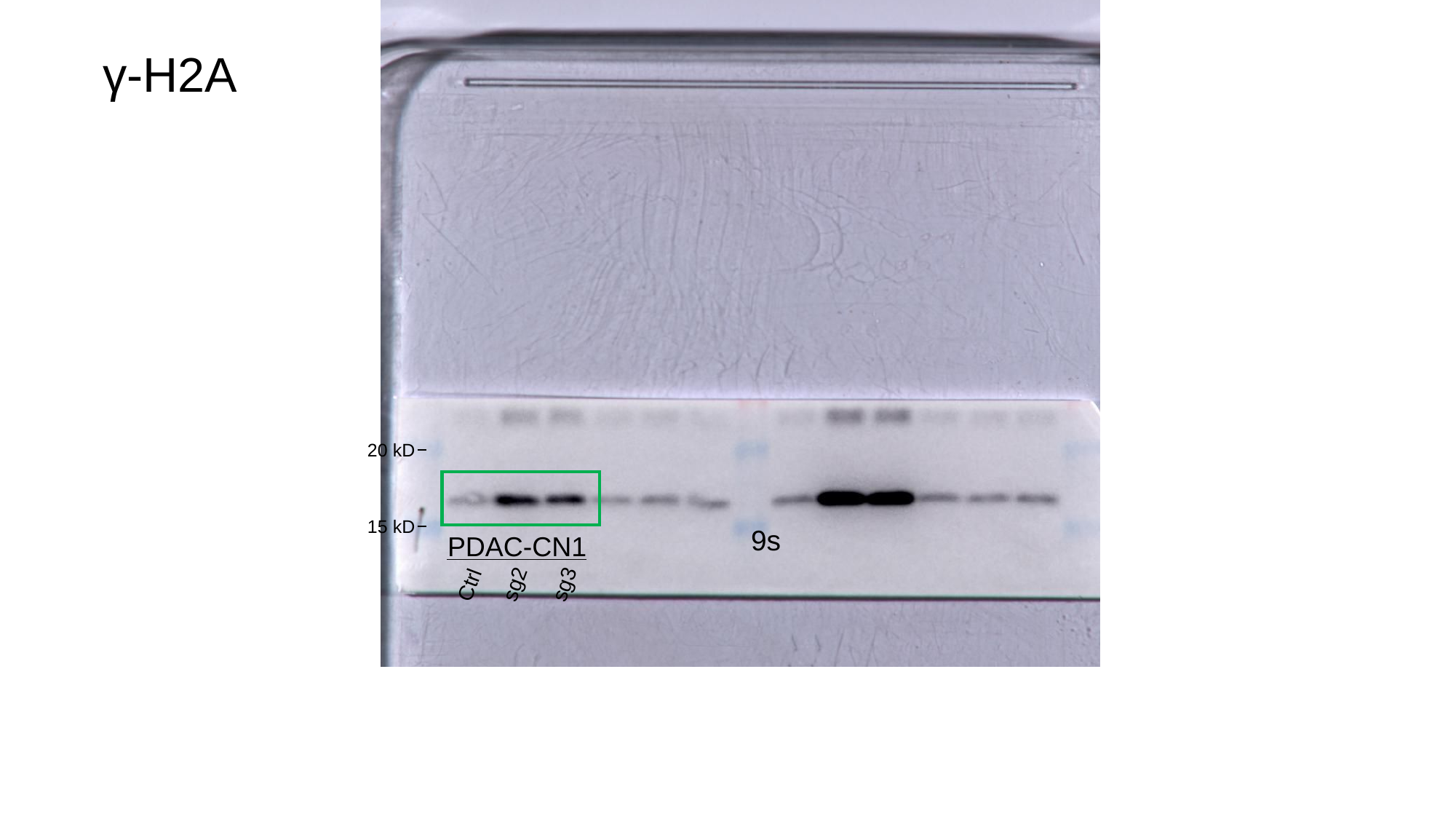

γ-H2A
20 kD
15 kD
9s
PDAC-CN1
Ctrl
sg3
sg2

## Slide 4
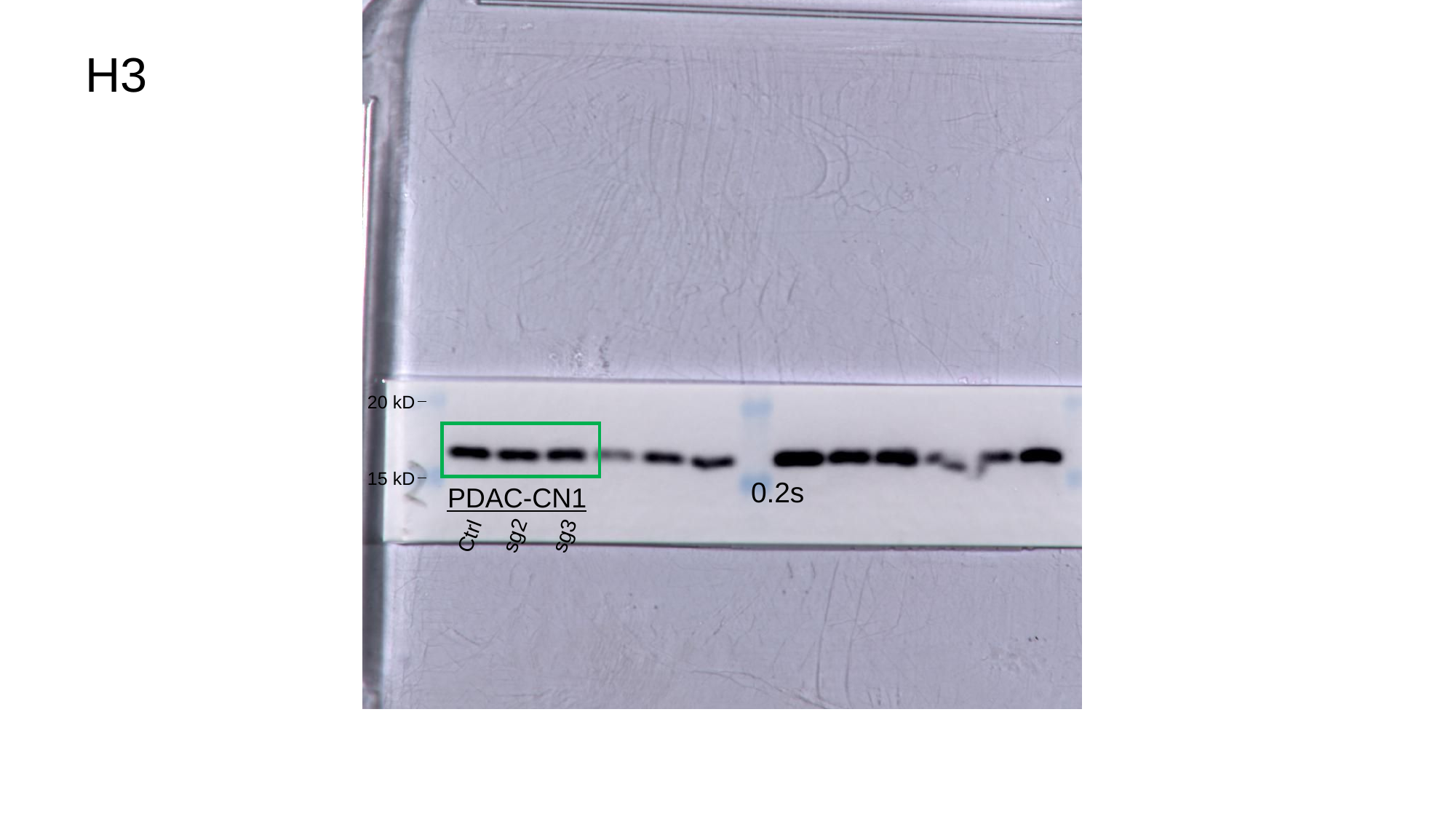

H3
20 kD
15 kD
0.2s
PDAC-CN1
Ctrl
sg3
sg2

## Slide 5
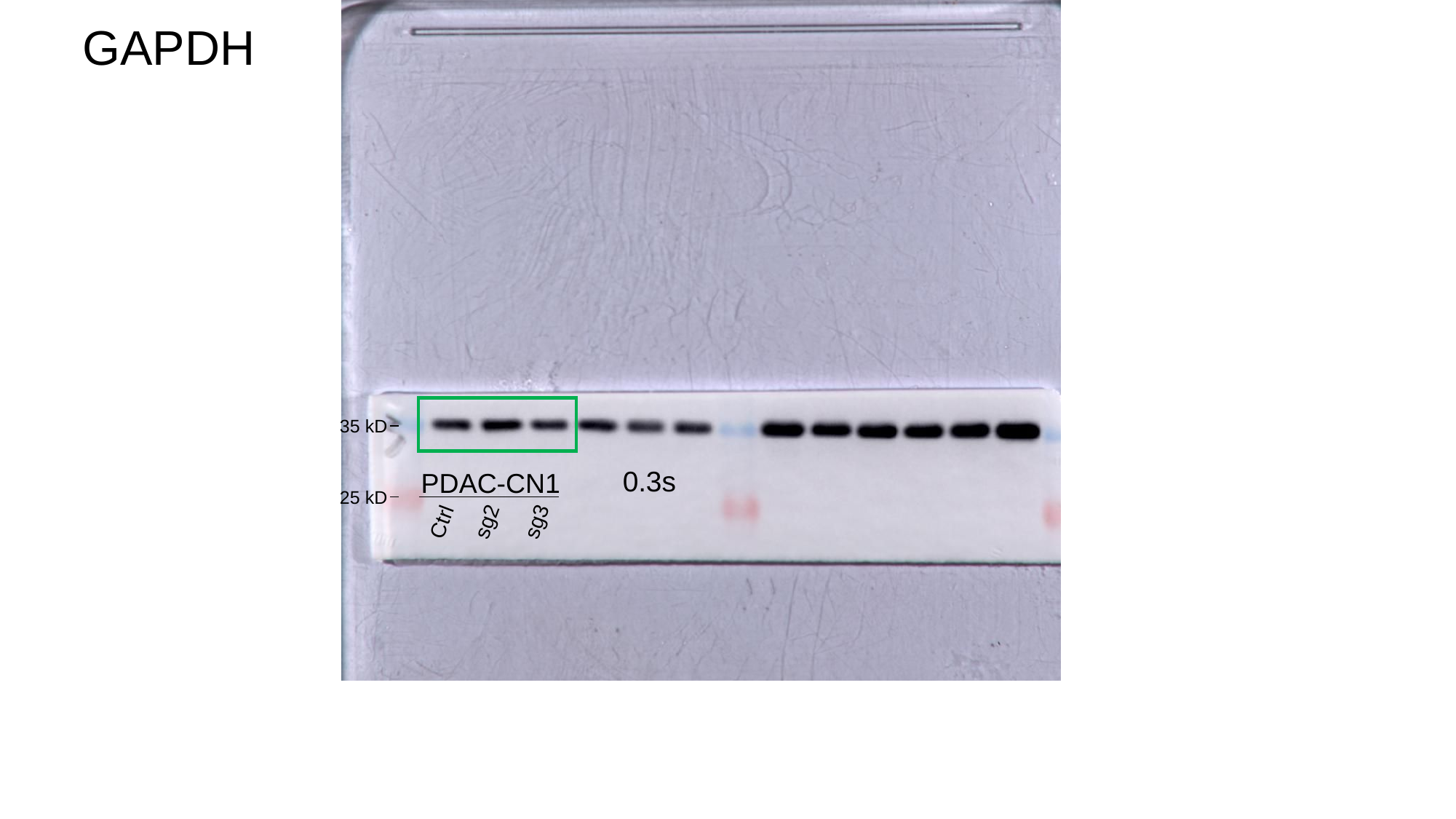

GAPDH
35 kD
0.3s
PDAC-CN1
Ctrl
25 kD
sg3
sg2
