# Supplementary material for: Genome-wide CRISPR screens identify PKMYT1 as a therapeutic target in pancreatic ductal adenocarcinoma
Source: EMBO Mol Med. 2024 Apr 3;16(5):5. doi: 10.1038/s44321-024-00060-y (PMC11099189; doi:10.1038/s44321-024-00060-y)
Supplement: Supplementary file 16 — Expanded View Figures [file 44321_2024_60_MOESM16_ESM.pdf]

# Expanded View Figures

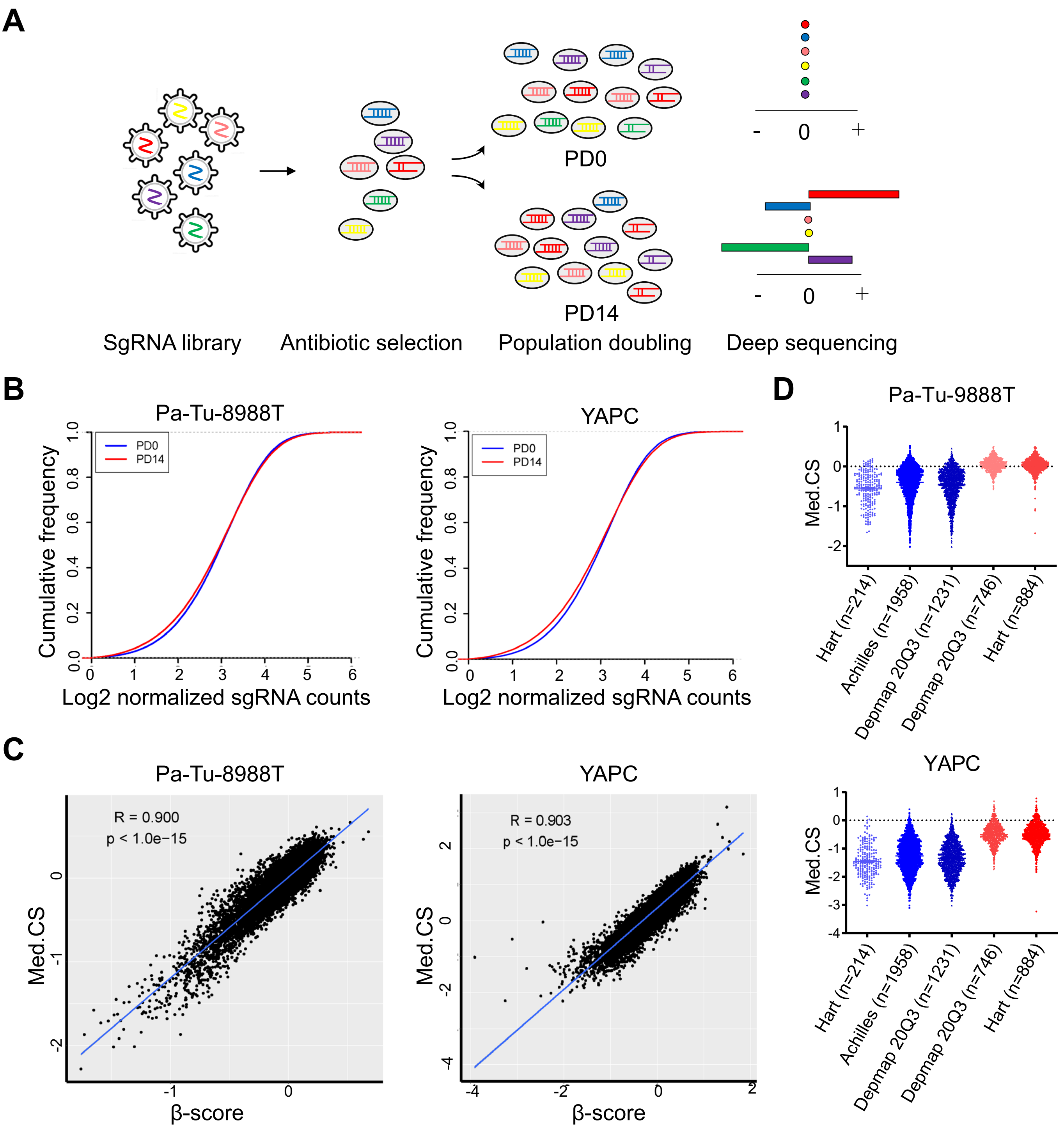


**Figure EV1. Genome-wide CRISPR screen overview.**

(**A**) Schematic outlining the design of the genome-wide CRISPR/Cas9 knockout screens in PDAC cells. (**B**) Cumulative frequency of sgRNAs during the 14 PDs after transduction in Pa-Tu-8988T and YAPC cells. The shift in the PD14 curve indicates the depletion of a subset of sgRNAs. (**C**) Significant correlation between the med.CS and β score in the Pa-Tu-8988T and YAPC cell lines. Unpaired *t* test; pearson correlation coefficient was used. (**D**) Plot comparing the med.CS of known pan-essential (blue points) and non-pan-essential genes (red points) in Pa-Tu-8988T and YAPC cells. *n* indicates the number of genes.

© The Author(s)

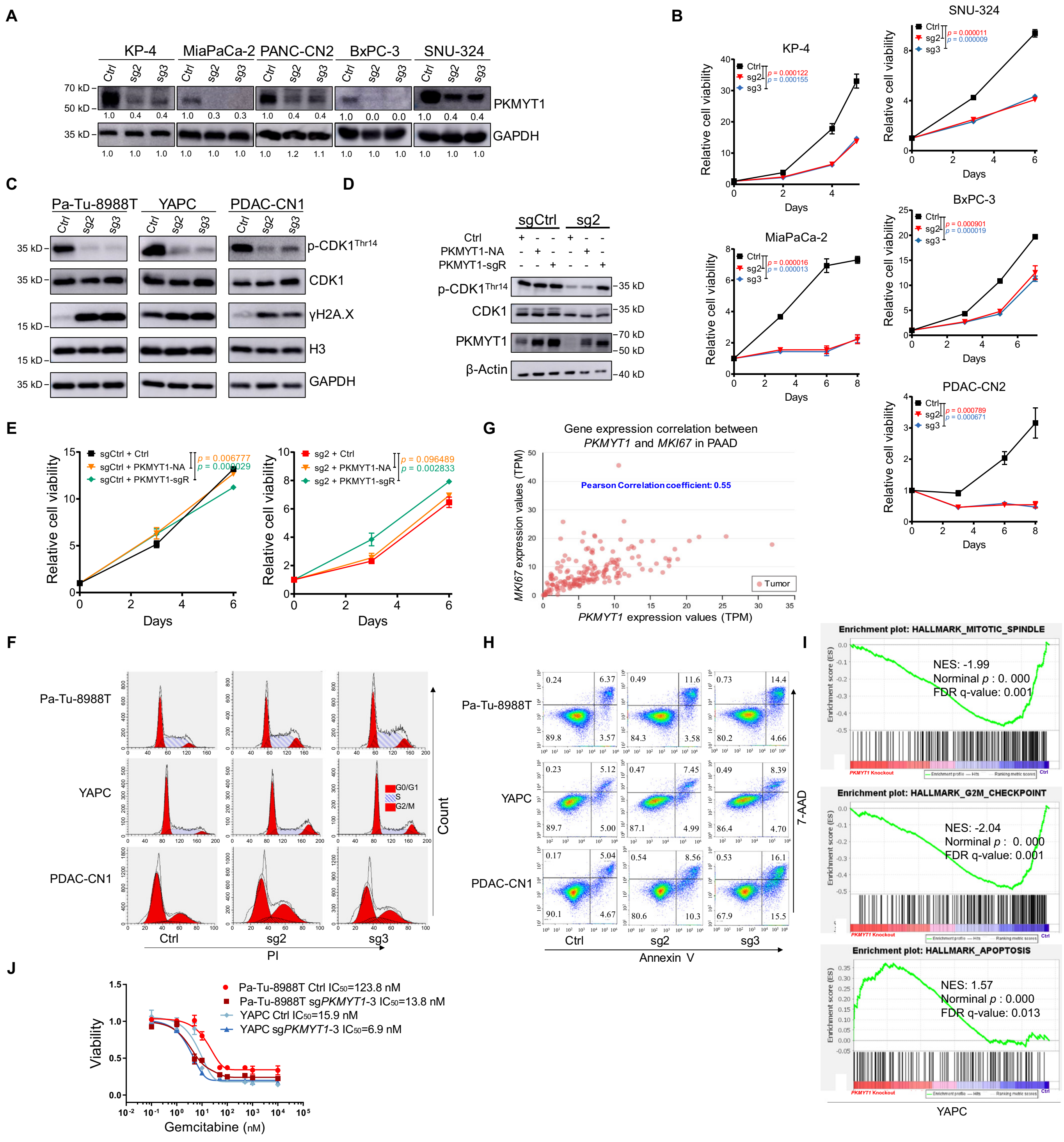
A
KP-4
MiaPaCa-2
PANC-CN2
BxPC-3
SNU-324
PKMYT1
GAPDH
B
KP-4
MiaPaCa-2
SNU-324
BxPC-3
PDAC-CN2
Relative cell viability
Days
C
Pa-Tu-8988T
YAPC
PDAC-CN1
p-CDK1 Thr14
CDK1
γH2A.X
H3
GAPDH
D
sgCtrl
sg2
PKMYT1-NA
PKMYT1-sgR
β-Actin
E
sgCtrl + Ctrl
sgCtrl + PKMYT1-NA
sgCtrl + PKMYT1-sgR
sg2 + Ctrl
sg2 + PKMYT1-NA
sg2 + PKMYT1-sgR
G
Gene expression correlation between PKMYT1 and MKI67 in PAAD
Pearson Correlation coefficient: 0.55
MKI67 expression values (TPM)
PKMYT1 expression values (TPM)
Tumor
F
G0/G1
S
G2/M
Count
PI
H
7-AAD
Annexin V
I
Enrichment plot: HALLMARK_MITOTIC_SPINDLE
NES: -1.99
Nominal p : 0. 000
FDR q-value: 0.001
Enrichment plot: HALLMARK_G2M_CHECKPOINT
NES: -2.04
Nominal p : 0. 000
FDR q-value: 0.001
Enrichment plot: HALLMARK_APOPTOSIS
NES: 1.57
Nominal p : 0.000
FDR q-value: 0.013
YAPC
J
Pa-Tu-8988T Ctrl IC50=123.8 nM
Pa-Tu-8988T sgPKMYT1-3 IC50=13.8 nM
YAPC Ctrl IC50=15.9 nM
YAPC sgPKMYT1-3 IC50=6.9 nM
Viability
Gemcitabine (nM)


© The Author(s)

**Figure EV2. Depletion of *PKMYT1* inhibits tumor growth and proliferation in PDAC models in vitro and in vivo.**

(**A**) CRISPR-mediated *PKMYT1* knockout (sg2 and sg3) decreases PKMYT1 protein expression in four more cell lines (KP, Miapaca2, SNU-324 and Bxpc3) and one more primary cultured cell (PDAC-CN2). (**B**) Lentivirus-mediated *PKMYT1* knockout reduces the viability of cells in Fig. EV2A. The error bars indicate the mean ± s.d. of three replicates; unpaired *t* test. (**C**) Knockout of *PKMYT1* decreases CDK1 Thr14 phosphorylation and increases γH2A.X accumulation. (**D**, **E**) CellTiter-Glo viability assay of the indicated YAPC cells transduced with lentiviral vectors expressing sgRNA targeting *PKMYT1* (sg2) or control sgRNA (sgCtrl) along with sgRNA-resistant PKMYT1 (PKMYT1-sgR) or kinase-dead sgRNA-resistant PKMYT1-N238A (PKMYT1-NA). The protein kinase activity of PKMYT1 is, at least partially, required for the regulation of tumor proliferation. The error bars indicate the mean ± s.d. of three replicates; unpaired *t* test. (**F**) Representative results of cell cycle analysis by flow cytometry for Fig. 3H. (**G**) *PKMYT1* expression correlates with proliferative index indicated by Ki67 ($n = 179$). (**H**) Representative results of apoptosis analysis by flow cytometry for Fig. 3J. (**I**) GSEA of differentially expressed genes in YAPC cells demonstrates that *PKMYT1* knockout regulates the genes involved in the cell cycle (including mitotic spindle, and G2M checkpoint) and apoptosis pathway. NES normalized enrichment score. (**J**) *PKMYT1* depletion sensitizes pancreatic cancer cells to gemcitabine. The error bars indicate the mean ± s.d. of three replicates. Figures EV2C, EV2F, and EV2H represent data from two biological replicates. Source data are available online for this figure.

© The Author(s) 

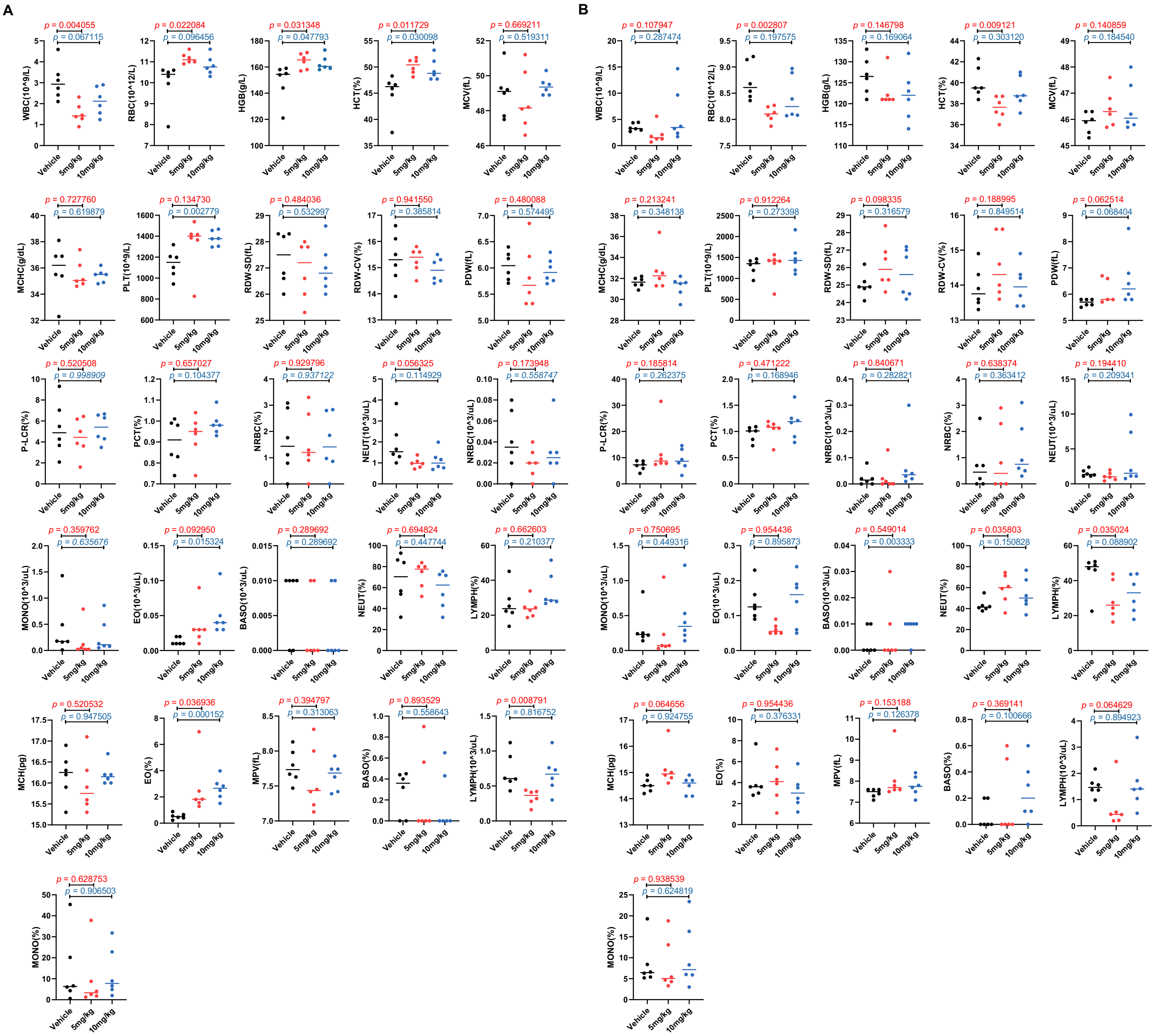


**Figure EV3. RP-6306, a PKMYT1 inhibitor, suppresses tumor growth and proliferation in PDAC CDX and PDX models.**

Routine peripheral blood tests show no significant differences in the RP-6306-treated mice bearing Pa-Tu-8988T xenografts (**A**) ($n = 6$ per group) and PDXs (**B**) ($n = 6$ per group). WBC white blood cell, RBC red blood cell, HGB hemoglobin, HCT hematocrit, MCV mean corpuscular volume, MCH mean corpuscular hemoglobin, MCHC mean corpuscular-hemoglobin concentration, PLT platelet, PDW platelet distribution width, MPV mean platelet volume, P-LCR platelet-large cell ratio, PCT plateletcrit, NRBC nucleated red blood cells, NEUT neutrophil, LYMPH lymphocyte, MONO monocyte, EO eosinophil, BASO basophile. Unpaired *t* test.


© The Author(s)

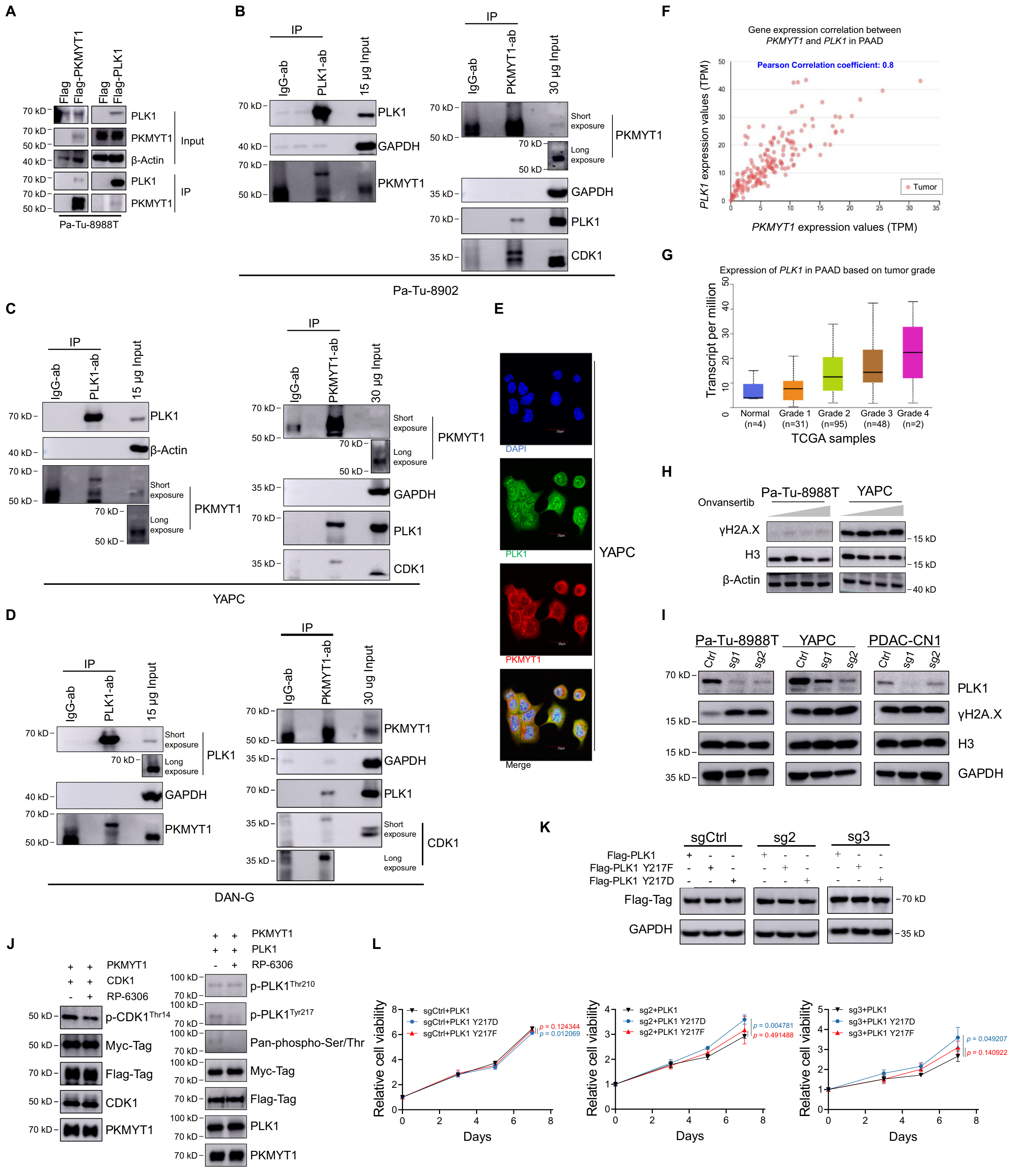
A
Flag
Flag-PKMYT1
Flag
Flag-PLK1
PLK1
PKMYT1
β-Actin
PLK1
PKMYT1
Input
IP
Pa-Tu-8988T
B
IP
IgG-ab
PLK1-ab
15 μg Input
PLK1
GAPDH
PKMYT1
IP
IgG-ab
PKMYT1-ab
30 μg Input
Short exposure
Long exposure
PKMYT1
GAPDH
PLK1
CDK1
Pa-Tu-8902
C
IP
IgG-ab
PLK1-ab
15 μg Input
PLK1
β-Actin
Short exposure
Long exposure
PKMYT1
IP
IgG-ab
PKMYT1-ab
30 μg Input
Short exposure
Long exposure
PKMYT1
GAPDH
PLK1
CDK1
YAPC
D
IP
IgG-ab
PLK1-ab
15 μg Input
Short exposure
Long exposure
PLK1
GAPDH
PKMYT1
IP
IgG-ab
PKMYT1-ab
30 ug Input
PKMYT1
GAPDH
PLK1
Short exposure
Long exposure
CDK1
DAN-G
E
DAPI
PLK1
PKMYT1
Merge
YAPC
F
Gene expression correlation between PKMYT1 and PLK1 in PAAD
Pearson Correlation coefficient: 0.8
PLK1 expression values (TPM)
PKMYT1 expression values (TPM)
Tumor
G
Expression of PLK1 in PAAD based on tumor grade
Transcript per million
Normal (n=4)
Grade 1 (n=31)
Grade 2 (n=95)
Grade 3 (n=48)
Grade 4 (n=2)
TCGA samples
H
Onvansertib
Pa-Tu-8988T
YAPC
γH2A.X
H3
β-Actin
I
Pa-Tu-8988T
YAPC
PDAC-CN1
Ctrl
sg1
sg2
PLK1
γH2A.X
H3
GAPDH
J
PKMYT1
CDK1
RP-6306
p-CDK1Thr14
Myc-Tag
Flag-Tag
CDK1
PKMYT1
PKMYT1
PLK1
RP-6306
p-PLK1Thr210
p-PLK1Tyr217
Pan-phospho-Ser/Thr
Myc-Tag
Flag-Tag
PLK1
PKMYT1
K
sgCtrl
sg2
sg3
Flag-PLK1
Flag-PLK1 Y217F
Flag-PLK1 Y217D
Flag-Tag
GAPDH
L
sgCtrl+PLK1
sgCtrl+PLK1 Y217D
sgCtrl+PLK1 Y217F
p = 0.124344
p = 0.012069
sg2+PLK1
sg2+PLK1 Y217D
sg2+PLK1 Y217F
p = 0.004781
p = 0.491488
sg3+PLK1
sg3+PLK1 Y217D
sg3+PLK1 Y217F
p = 0.049207
p = 0.140922
Relative cell viability
Days

**Figure EV4. PKMYT1 interacts with PLK1 and regulates PLK1 expression and phosphorylation.**

(**A**) PLK1 interacts with PKMYT1 in Pa-Tu-8988T. The cells were transduced with Flag-PKMYT1, Flag-PLK1 or Flag lentivirus, and cell lysates were subjected to a co-IP assay using an anti-Flag antibody, followed by immunoblotting with the indicated antibodies. (**B–D**) The endogenous PLK1-PKMYT1 interaction is confirmed in YAPC, Pa-Tu-8902 and DAN-G cells by a co-IP assay. (**E**) Immunofluorescence assay shows that PKMYT1 protein distributes in the cytoplasm, and PLK1 protein spreads in nucleus and cytoplasm. Cytoplasm PKMYT1 and PLK1 overlap. Scale bars: 20 µm. (**F**) Positive correlation between the expression levels of *PKMYT1* and *PLK1* ($n = 179$). (**G**) *PLK1* expression in human pancreatic cancer tissues with different grades (Normal, $n = 4$; Grade 1, $n = 31$; Grade 2, $n = 95$; Grade 3, $n = 48$; Grade 4, $n = 2$). The low bound, centerline, and upper bound of boxplot represent the first quartile, the median, and the third quartile of data, respectively; the upper and lower whiskers extend to the largest and smallest value. (**H**) Onvansertib (PLK1 inhibitor) treatment has no effect on DNA damage accumulation. Concentrations of compound used are listed in "Methods". (**I**) Knockout of *PLK1* (sg1 and sg2) does not induce DNA damage accumulation in all cell lines. (**J**) Phosphorylation signals were revealed using western blotting (Pan-phospho-Ser/Thr antibody, Phospho-PLK1-Thr210 antibody; Phospho-PLK1–Tyr217 antibody). Myc-CDK1 phosphorylation by PKMYT1 was used as a control. Western blotting analysis indicates PKMYT1 phosphorylates PLK1 Tyr217 and other Ser/Thr sites except Thr210. (**K**, **L**) CellTiter-Glo viability assay of the indicated YAPC cells transduced with lentiviral vectors expressing sgRNA targeting *PKMYT1* (sg2 or sg3) or control sgRNA (sgCtrl) along with phospho-mimicking PLK1 Tyr217Asp (Y217D) mutant, Tyr217Phe mutant (Y217F, which can not be phosphorylated) or wild-type PLK1. Tyr217 phosphorylation is involved in the oncogenic function of PKMYT1. The error bars indicate the mean ± s.d. of three replicates; unpaired *t* test.


© The Author(s)

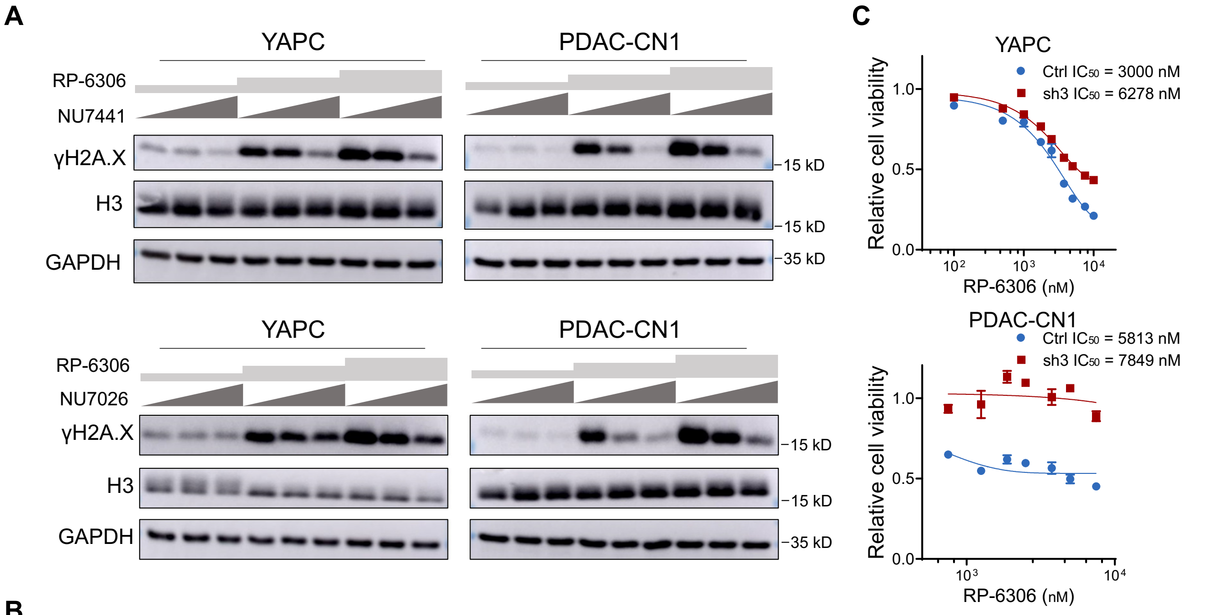


B

PDAC-CN1

CI < 1, synergism;
CI = 1, additive effect;
CI > 1, antagonism

| # | Combinations RP-6306 (μM) | NU7441 (μM) |
|---|---|---|
| 1 | 1 | 0.5 |
| 2 | 2.5 | 0.5 |
| 3 | 5 | 0.5 |
| 4 | 1 | 5 |
| 5 | 2.5 | 5 |
| 6 | 5 | 5 |
| 7 | 1 | 10 |
| 8 | 2.5 | 10 |
| 9 | 5 | 10 |

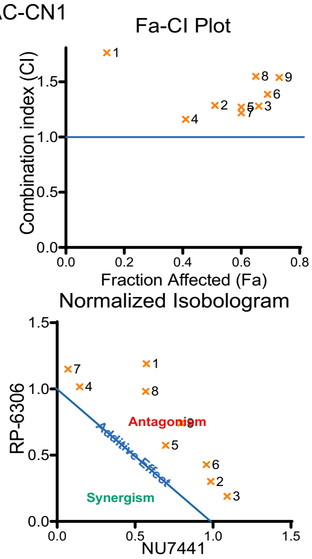


PDAC-CN1

| # | Combinations RP-6306 (μM) | NU7026 (μM) |
|---|---|---|
| 1 | 1 | 5 |
| 2 | 2.5 | 5 |
| 3 | 5 | 5 |
| 4 | 1 | 10 |
| 5 | 2.5 | 10 |
| 6 | 5 | 10 |
| 7 | 1 | 20 |
| 8 | 2.5 | 20 |
| 9 | 5 | 20 |

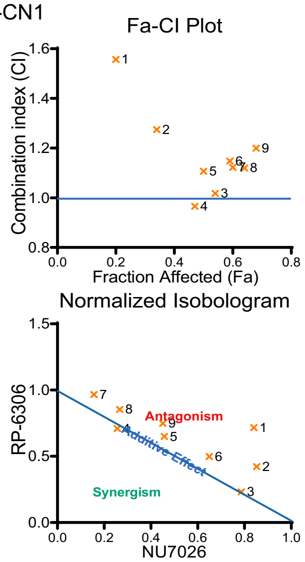


YAPC

| # | Combinations RP-6306 (μM) | NU7441 (μM) |
|---|---|---|
| 1 | 0.5 | 0.5 |
| 2 | 1 | 0.5 |
| 3 | 2.5 | 0.5 |
| 4 | 5 | 0.5 |
| 5 | 0.5 | 1 |
| 6 | 1 | 1 |
| 7 | 2.5 | 1 |
| 8 | 5 | 1 |
| 9 | 0.5 | 5 |
| 10 | 1 | 5 |
| 11 | 2.5 | 5 |
| 12 | 5 | 5 |

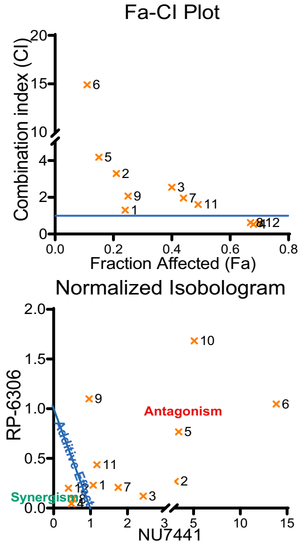


YAPC

| # | Combinations RP-6306 (μM) | NU7026 (μM) |
|---|---|---|
| 1 | 0.5 | 8 |
| 2 | 1 | 8 |
| 3 | 2.5 | 8 |
| 4 | 5 | 8 |
| 5 | 0.5 | 16 |
| 6 | 1 | 16 |
| 7 | 2.5 | 16 |
| 8 | 5 | 16 |
| 9 | 0.5 | 24 |
| 10 | 1 | 24 |
| 11 | 2.5 | 24 |
| 12 | 5 | 24 |

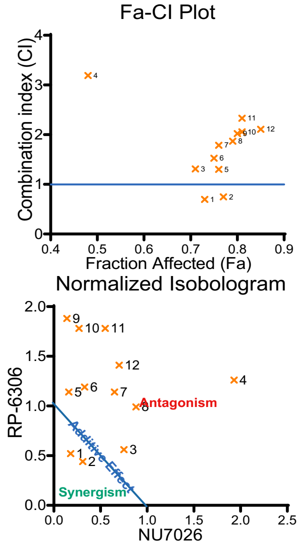


© The Author(s)

**Figure EV5. PKMYT1 ablation activates PRKDC, whose activity modulates the sensitivity of PDAC to PKMYT1 inhibition.**

(**A**) The PRKDC inhibitor NU7441 and NU7026 disturb γH2A.X accumulation induced by the RP-6306 treatment. As the concentration of RP-6306 (lane 1, 4, and 7) increases, γH2AX accumulation increases. For each dosage of RP-6306 (lane 4–6, and lane 7–9), γH2AX accumulation decreases in a NU7441/NU7026 concentration-dependent manner, showing that PRKDC is involved in DNA damage response induced by PKMYT1 inhibition. Concentrations of inhibitors are listed in "Methods". (**B**) The PRKDC inhibitor NU7441 (left) and NU7026 (right) modulate the sensitivity of PDAC to PKMYT1 inhibitor. CI (top) and isobologram (bottom) analyses reveal the antagonistic effect between PKMYT1 inhibitor and PRKDC inhibitors in PDAC (CI > 1). Representative Fa-CI plots (top) and normalized isobolograms (bottom) are shown ($n = 3$ per group). (**C**) *PRKDC* knockdown (sh3) desensitizes cells to RP-6306. $IC_{50}$ values are shown. The error bars indicate the mean ± s.e.m. of three replicates.

 © The Author(s)
